# Patterns of Protein Evolution in Cytochrome *c* Oxidase 1 (COI) from the Class Arachnida

**DOI:** 10.1371/journal.pone.0135053

**Published:** 2015-08-26

**Authors:** Monica R Young, Paul D. N. Hebert

**Affiliations:** Biodiversity Institute of Ontario, University of Guelph, Guelph, Ontario, Canada; Onderstepoort Veterinary Institute, SOUTH AFRICA

## Abstract

Because sequence information is now available for the 648bp barcode region of cytochrome *c* oxidase 1 (COI) from more than 400,000 animal species, this gene segment can be used to probe patterns of mitochondrial evolution. The present study examines levels of amino acid substitution and the frequency of indels in COI from 4177 species of arachnids, including representatives from all 16 orders and 43% of its families (267/625). It examines divergences at three taxonomic levels—among members of each order to an outgroup, among families in each order and among BINs, a species proxy, in each family. Order Distances vary fourfold (0.10–0.39), while the mean of the Family Distances for the ten orders ranges fivefold (0.07–0.35). BIN Distances show great variation, ranging from 0.01 or less in 12 families to more than 0.25 in eight families. Patterns of amino acid substitution in COI are generally congruent with previously reported variation in nucleotide substitution rates in arachnids, but provide some new insights, such as clear rate acceleration in the Opiliones. By revealing a strong association between elevated rates of nucleotide and amino acid substitution, this study builds evidence for the selective importance of the rate variation among arachnid lineages. Moreover, it establishes that groups whose COI genes have elevated levels of amino acid substitution also regularly possess indels, a dramatic form of protein reconfiguration. Overall, this study suggests that the mitochondrial genome of some arachnid groups is dynamic with high rates of amino acid substitution and frequent indels, while it is ‘locked down’ in others. Dynamic genomes are most prevalent in arachnids with short generation times, but the possible impact of breeding system deserves investigation since many of the rapidly evolving lineages reproduce by haplodiploidy, a mode of reproduction absent in ‘locked down’ taxa.

## Introduction

With nearly 100,000 known species and perhaps a million awaiting description [1], the arachnids are the most diverse class of arthropods after insects. These species are assigned to ten well-accepted orders and to a final lineage, the Acari, whose boundaries are less secure. Krantz & Walter [2] partitioned it into two superorders and six orders—the Acariformes (Sarcoptiformes, Trombidiformes), and the Parasitiformes (Holothyrida, Ixodida, Mesostigmata, Opilioacarida), but others regard the Acari as just two orders (Acariformes, Parasitiformes). Taxonomic diversity varies substantially among these lineages (Table 1); the Araneae are the most diverse of the large-bodied taxa with 45,000 described species, but the Acariformes likely includes more than a million [3]. Although arachnid monophyly is well-supported, phylogenetic relationships among its orders are unclear [4]. This uncertainty reflects, at least in part, complexities introduced by accelerated rates of molecular evolution in several orders, particularly the Acariformes, the Parasitiformes and the Pseudoscorpiones [5–7]. This rate variation has often been linked to the 50-fold divergence in generation lengths of arachnids which range from a few weeks to several years [8–10].

**Table 1:** Sequence divergence at COI for three taxonomic levels as measured by Dayhoff distance.

Taxon	BINs	Families (n)	Order Distance	Family Distance	BIN Distance	Overall Distance	Families/ Species with Indels
Holothyrida	1	1	0.148~				0
Opilioacarida	1	1	0.155~				0
Ricinulei	2	1	0.171**				0
Uropygi	3	1	0.177**				0
Palpigradi	1	1	0.247~				0
Schizomida	4	2?	0.277**				0
Solifugae	5	4	0.126**	0.071**	0.023~	0.073	0
Amblypygi	8	4	0.103**	0.084**	0.051**	0.079	0
Scorpiones	127	6	0.154**	0.099**	0.036**	0.096	0
Araneae	1517	66	0.250**	0.158**	0.064**	0.124	0
Ixodida	72	3	0.187*	0.213**	0.097*	0.166	0
Sarcoptiformes	820	74	0.275**	0.250**	0.046**	0.190	1/9
Mesostigmata	414	24	0.260**	0.229**	0.120*	0.203	4/29
Pseudoscorpiones	90	21	0.390**	0.331**	0.190*	0.303	16/65
Trombidiformes	937	38	0.328**	0.341**	0.111**	0.260	6/25
Opiliones	175	20	0.292**	0.354**	0.108	0.251	5/65

Order Distance quantifies amino acid variation among families in 16 orders of arachnids to the outgroup *Limulus polyphemus*. Family Distances quantify amino acid variation among families within ten of these orders, while BIN Distance quantifies the mean amino acid divergence among the BINs within each family in these orders. The Overall Distance is the mean of the other three distance values.

* SE < 0.025

** SE < 0.015

^~^ SE = NA (n = 1)

Broad patterns of mitochondrial genome evolution have seen limited investigation in arachnids, because most prior molecular work has been directed toward resolving phylogenetic affinities. These studies have, however, revealed four-fold variation in rates of nucleotide substitution in mitochondrial genes among different Acari lineages [7]. Prior studies have also revealed gene rearrangements and indels in arachnid mitochondrial genomes [11–12]. The present investigation examines levels of amino acid substitution and patterns of indel distribution in the barcode region of COI among arachnid lineages. While indels are uncommon in COI [13–15], their incidence has been linked to shifts in nucleotide usage and evolutionary rates [16]. Prior work has also shown that single mitochondrial genes can reliably indicate variation in both evolutionary rates [17–19] and the nucleotide composition [20–21] of mitochondrial genomes. This study assesses levels of amino acid substitution and the incidence of indels in COI among major lineages of the Arachnida as a clue to broader patterns of mitochondrial genome evolution.

## Methods

### Sequence Selection and Validation

All publically available and BIN compliant [22] barcode sequences for arachnids on BOLD [23] on January 1, 2015 were assembled into a Dataset (DS-MYBCA) available at the following DOI (dx.doi.org/10.5883/DS-MYBCA). All new sequences were also deposited in GenBank (S1 Table). These 23,185 sequences included representatives from 4177 putative species (BINs) with representatives of 16 orders and 267 families. All sequences were checked to confirm their lack of stop codons or frameshift mutations that would indicate their likely derivation from a NUMT. To avoid pseudoreplication at a species level, the longest sequence with fewest ambiguous base calls (generally 657bp with 0 Ns) from each of the 4177 BINs was extracted to create a BIN dataset (DS-MYBCRED) with a single representative of each BIN and an outgroup sequence (*Limulus polyphemus*). These sequences were aligned in MEGA6 [24] using ClustalW [25] on the amino acid translation. The resulting alignment was manually verified to confirm its accuracy. The BIN Dataset (S1 Alignment, S2 Table) is available on BOLD at the following DOI (dx.doi.org/10.5883/DS-MYBCRED).

Approximately two thirds of the records in the BIN Dataset were linked to a voucher specimen, enabling the validation of familial (and often more precise) taxonomic assignments. However, other BIN records derived from GenBank sequences, most of which cannot be linked to a voucher. In these cases, verification of family assignment relied on checking the consistency of placement of the sequence in a Neighbor-Joining Tree in relation to other species assigned to its presumptive family. In addition, sequences with uncertain placement were blasted on GenBank for further validation. Five records in the BIN dataset lack a family assignment; they were retained because they are the sole sequences available for the orders Schizomida and Palpigradi.

### Analysis of Amino Acid Variation

Patterns of amino acid substitution in COI were evaluated at three taxonomic levels (Order, Family, BIN) employing both uncorrected p-distances and Dayhoff distances calculated in MEGA6 [24]. A total of 219 amino acid sites were compared as gaps were considered as pairwise deletions. Family and BIN distances were visualized using vector plots for orders with more than two families with each vector placed equidistantly around a centroid with its length reflecting average Dayhoff distance.

The distance of each BIN to the outgroup taxon (Xiphosurida: *Limulus polyphemus*) was calculated before averaging the values for all BINs in a particular family to quantify its mean divergence to the outgroup. The family values for each order were then averaged to determine its divergence from the outgroup. This is subsequently referred to as the Order Distance. Divergence values between each BIN in a particular family and each BIN in all other families belonging to the same order were averaged to quantify the level of amino acid substitution for each family in an order. This is subsequently referred to as the Family Distance. Finally, divergence values among the BINs in each family were averaged to quantify the level of amino acid substitution among the species in each family. This is subsequently referred to as the BIN Distance.

### Sensitivity of Results to Taxon Concepts

Although the arachnids have a relatively stable higher taxonomy, groups with a conserved morphology might well be ‘undersplit’ versus those with more dramatic morphological variation. We tested the potential impact of this effect by examining two orders with very different rates of amino acid substitution at COI—the phenotypically diverse Araneae with slow molecular evolution and the morphologically conservative Opiliones with high rates of molecular change. Our analysis asked the following question—how do rates of perceived molecular evolution in the Opiliones shift if each genus is raised to a family and subsequently used to re-calculate Family Distance, BIN Distance, and Overall Distance?

### Analysis of Indels

The sequence alignment for each BIN was manually inspected for insertions and deletions against a consensus sequence for all arachnids. When an indel was detected, the trace files responsible for the sequence record were inspected to confirm the validity of the indel. Because this validation step was not possible for sequences on GenBank, indels based on a single GenBank record were excluded, but when an indel was present in sequence records from several taxa in the same family, it was retained. The location of each indel was plotted on a standard secondary structure model for the barcode region. Phylogenetic information for the arachnid orders [6,26–29] was subsequently used to assess the number of independent origins of indels.

## Results

### Amino Acid Variation Among Arachnid Lineages

**Fig 1:**
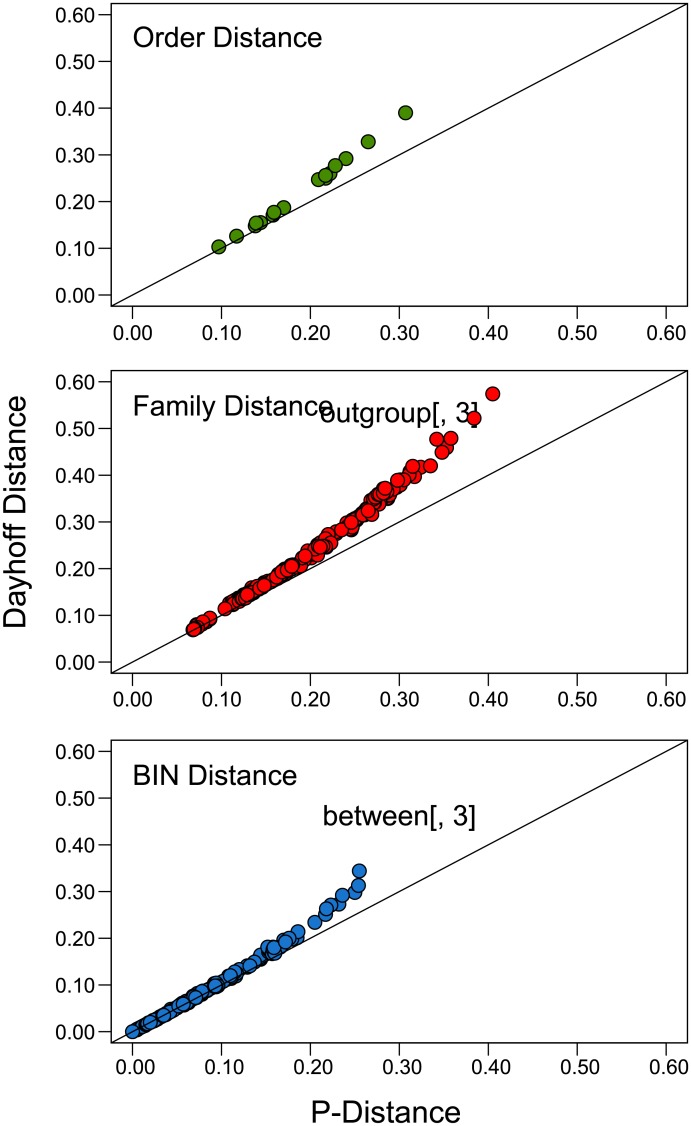
The relationship between p-distance and Dayhoff distance based on amino acid divergences at COI for three taxonomic levels, Order, Family, and BIN. The solid line plots the case where p-distance and Dayhoff distance are identical.

Values for p-distance and Dayhoff distance were closely coincident when < 0.20, but differences between the two metrics were substantial at higher divergences (Fig 1). Because a considerable fraction of the distance values exceeded 0.20, Dayhoff distance was adopted for all subsequent analyses.

**Fig 2:**
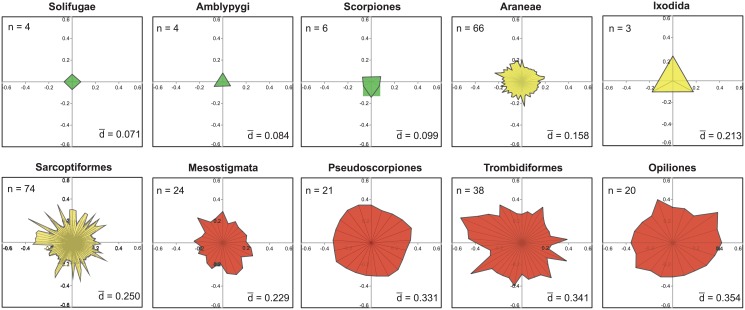
Vector plots showing amino acid divergences (Dayhoff Distance) in the barcode region of COI for the families in ten arachnid orders. n is the number of families in each order and d is the mean family divergence of each order. Different colors highlight the orders in the three levels of divergence.

**Fig 3:**
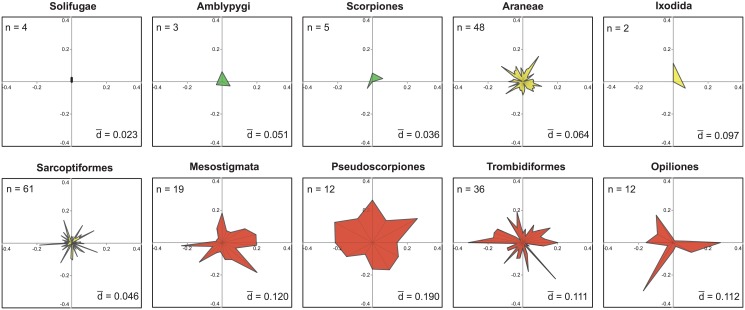
Vector plots showing amino acid divergences (mean Dayhoff Distance) in the barcode region of COI for the BINs within the families in ten arachnid orders. n is the number of families in each order represented by two or more species and d is the mean family divergence of each order. Different colors highlight the orders in the three levels of divergence.

**Fig 4:**
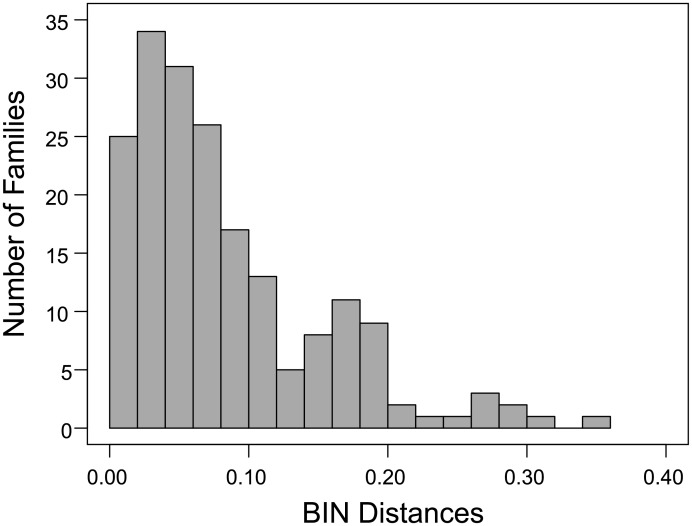
Mean levels of amino acid substitution (Dayhoff distance) in the COI barcode region among the BINs in 199 families of arachnids.

The Order Distances ranged four-fold from a low of 10.3% in the Amblypygi to a high of 39.0% in the Pseudoscorpiones (Table 1). As six orders were represented by a single family, Family Distances could only be determined for ten orders but these results showed four-fold variation with mean values for these orders ranging from 7.1% in Solifugae to 35.4% in Opiliones (Table 1, Fig 2). BIN distances were determined for families in the same ten orders and they showed more than 25-fold variation, ranging from 1% or less in 12 families to more than 25% in eight families (Figs 3 and 4, S3 Table). There was no relationship between BIN Distance and the number of taxa for a family (S1 Fig), but the patterning of BIN Distances varied among orders. All BIN Distances for Amblypygi, Araneae, Ixodida and Scorpiones were low (< 0.12) excepting three of the 66 families of Araneae. By contrast, 11 of 12 BIN Distances for the Pseudoscorpiones were > 0.14. In contrast to the limited variation in BIN Distance in these five orders, three orders (Opiliones, Sarcoptiformes, Trombidiformes) showed 15-fold variation in their BIN Distances (< 0.02 to > 0.30), while the Mesostigmata showed 10-fold variation.

**Fig 5:**
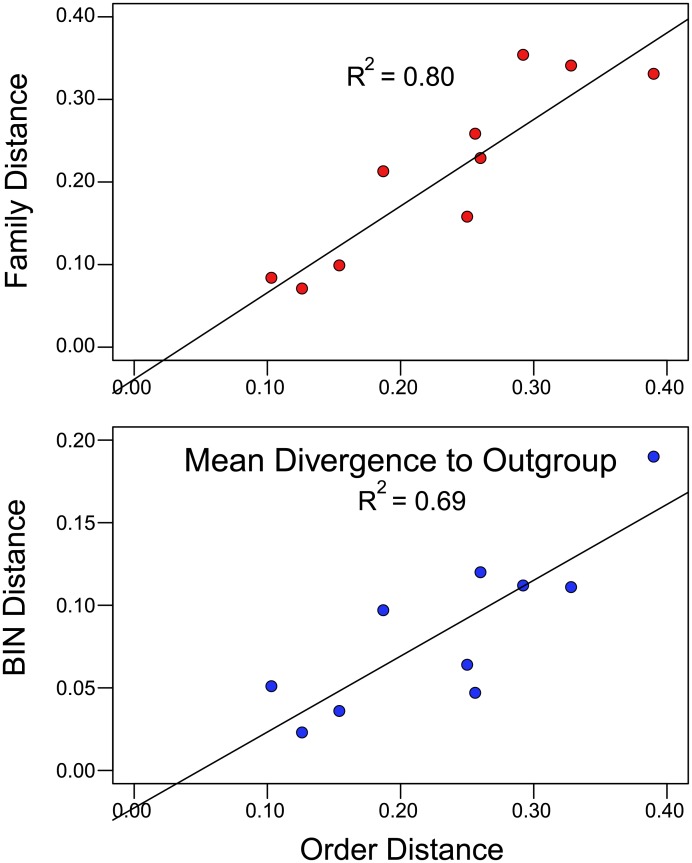
Regression of the Order Distance (Dayhoff distance) based on amino acid divergence in the barcode region of COI to the outgroup against mean Family Distance, and mean BIN Distance.

There was consistency in rates of amino acid substitution across the varied taxonomic levels as taxa with a high Order Distance also showed high Family Distance (R^2^ = 0.80, *p* = 0.0005) and high BIN Distances (R^2^ = 0.69, *p* = 0.003) (Fig 5). As a result, a composite measure of amino acid divergence was calculated by averaging the three distance values for each order. Three orders (Amblypygi, Scorpiones, Solifugae) showed low divergence (distances < 0.10), three orders (Araneae, Ixodida, Sarcoptiformes) had intermediate divergence (distances = 0.10–0.20), while four orders (Mesostigmata, Opiliones, Pseudoscorpiones, Trombidiformes) possessed high divergence (distances > 0.20).

### Sensitivity Test of Taxonomic Concept

Although the elevation of genera to family status raised the number of families of Opiliones from 20 to 43, it had little impact on the key metrics. Family Distance actually increased while BIN Distance and Overall Distance showed only small declines (Table 2). As a consequence, levels of amino acid substitution for the Opiliones remained substantially higher than the Araneae (0.249 vs 0.124 respectively).

**Table 2:** Comparison of sequence divergence at COI for Araneae and Opiliones, including two hierarchical treatments within the Opiliones.

Taxon	Classification Used for Distance Measure	BINs	Families (n)	Order Distance	Family Distance	BIN Distance	Overall Distance
Araneae	Families	1517	66	0.250**	0.158**	0.064**	0.124
Opiliones	Families	175	20	0.292**	0.354**	0.108	0.251
Opiliones	Genera elevated to Families	175	43	0.292**	0.356**	0.098*	0.249

Order Distance quantifies amino acid variation among families in 16 orders of arachnids to the outgroup *Limulus polyphemus*. Family Distances quantify amino acid variation among families within ten of these orders, while BIN Distance quantifies the mean amino acid divergence among the BINs within each family in these orders. The Overall Distance is the mean of the other three distance values.

* SE < 0.025

** SE < 0.015

### Indels for COI in Arachnid Lineages

**Fig 6:**
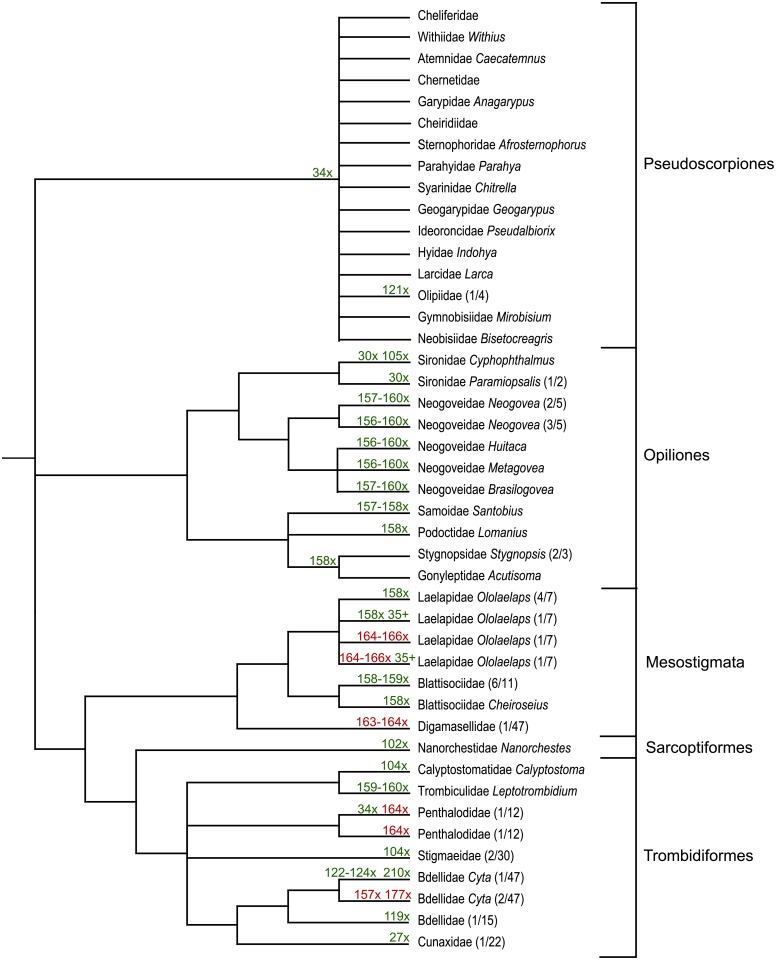
Phylogenetic relationships of taxa with indels in the COI barcode region with ‘X’ designating a deletion and ‘+’ an insertion. The numbers indicate the position of each indel in the alignment with green for those in loop regions, and red for those in others.

Indels were detected in 5 of the 16 orders (Mesostigmata, Opiliones, Pseudoscorpiones, Sarcoptiformes, Trombidiformes) (Table 1, S2 Fig). All involved a deletion, varying in length from one to five amino acids, except for a single amino acid insertion within two species of Mesostigmata (Fig 6). Certain insertions or deletions were conserved in a set of allied taxa. For example, 16 families in the suborder Iocheirata (Pseudoscorpiones) possessed an amino acid deletion at site 34 which was absent in five families in its other suborder (Epiocheirata). The Sarcoptiformes showed a similar pattern with a single amino acid deletion at site 102 in all 9 species of *Nanorchestes* (Sarcoptiformes) which was absent in other members of this order. A single amino acid deletion was present at site 158 in *Cheiroseius* (Mesostigmata: Blattisociidae) and in several other unidentified taxa in this family while several other unidentified blattisociids also had a deletion at the neighbouring site 159.

Certain deletions occurred in unrelated taxa, suggesting their recurrent origin. For instance, species in two families (Laelapidae, Blattisociidae) of Mesostigmata shared a deletion at site 158 with species in five families of Opiliones (Fig 6). The Laelapidae showed numerous indels at diverse locations. One species had a three amino acid deletion spanning sites 164–166, while another had the same deletion as well as an insertion at site 35. Another species in this family had the same insertion at site 35, but only a single amino acid deletion at site 158. Indels were also diverse in the Trombidiformes. Only 3 of 47 species of *Cyta* (Trombidiformes: Bdellidae) had deletions, but they involved the loss of two to four amino acids. Two of these species shared deletions at sites 157 and 177, while the other had a three amino acid deletion spanning sites 122–124, and another at site 210. One species of Penthalodidae (Trombidiformes) had an amino acid deletion at site 164, while another had a second deletion at site 34. Two of the 31 species of Stigmaeidae (Trombidiformes) had a single amino acid deletion at site 104.

The Opiliones showed the most variation in indel length. Nine of 20 species of Neogoveidae possessed the longest indel, a five amino acid deletion spanning sites 156–160 (Fig 6). Two of four species of *Paramiopsalis* (Sironidae) possessed a deletion at site 30 which was also present in all 14 species of the closely allied genus *Cyphopthalmus*, but they also had a second deletion at site 105.

### Association With Generation Length

**Fig 7:**
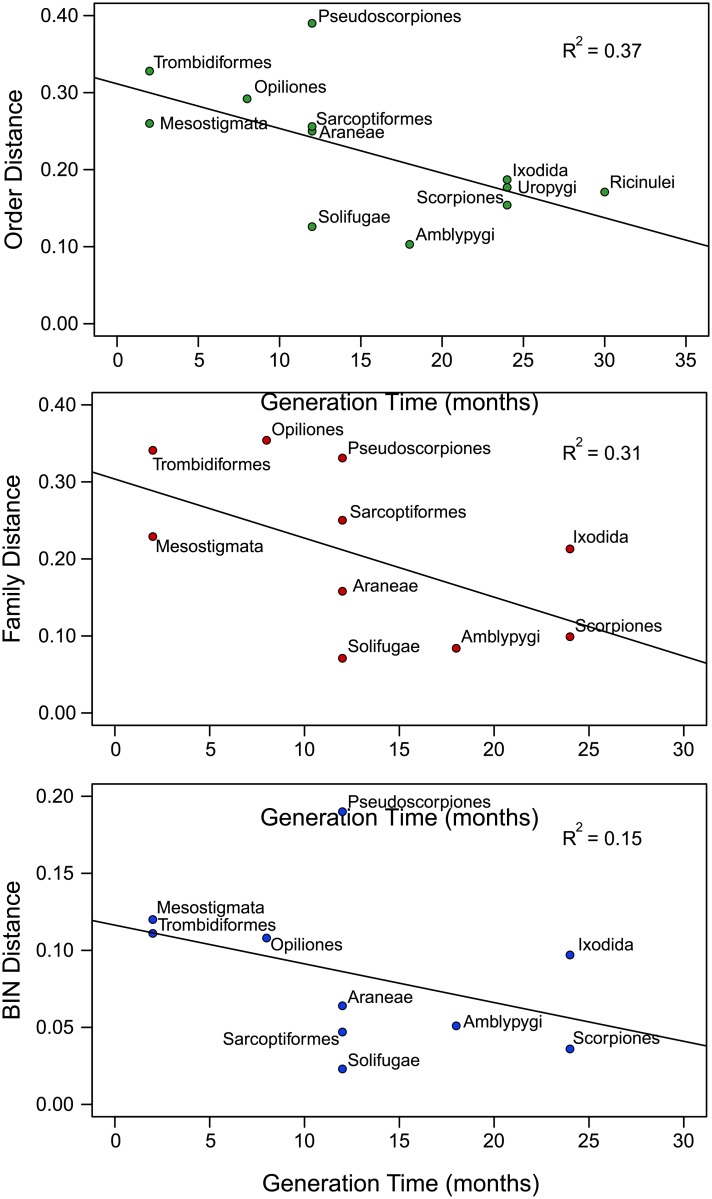
Linear regression of the relationship between generation time and average Dayhoff distances at three taxonomic levels, Order, Family, and BIN.

As generation length data were unavailable for four orders, analysis was restricted to 12 orders (S4 Table) [3,8,30–36]. Examination of the relationship between Order Distance and mean generation time revealed a significant negative relationship (R^2^ = 0.37, *p* = 0.04) and the same association was apparent to the other two distance measures (Family R^2^ = 0.31, *p* = 0.10; BIN R^2^ = 0.15, *p* = 0.27) (Fig 7).

## Discussion

Prior studies have revealed significant variation in nucleotide substitution rates among arachnids in both mitochondrial and nuclear genes, but they examined many fewer taxa than the current investigation. Murrell et al. [5] found substantial rate heterogeneity in three lineages of Acari, while Klompen et al. [6] documented elevated rates of substitution in nuclear genes in both superorders of Acari as well as in the Pseudoscorpiones. They also reported rate variation among the orders of Parasitiformes with lower divergences in the Holothyrida and Ixodida than in the Mesostigmata. Arabi et al. [12] broadened the evidence for rate variation to both nuclear and mitochondrial genes and confirmed accelerated evolution in the same three lineages (Acariformes, Parasitiformes, Pseudoscorpiones).

The present study reveals patterns of rate variation congruent with those detected in earlier investigations, but provides further details on its taxonomic localization. For example, this study confirms that the Pseudoscorpiones are rate accelerated, but documents its occurrence at all levels of the taxonomic hierarchy as members of this order possess the highest BIN and Order Distances and the third-highest Family Distance. The present results also confirm rate acceleration in the superorder Acariformes, but indicate that it is greater in the Trombidiformes than the Sarcoptiformes. Some earlier studies [6,12] combined results from these orders, masking this rate difference. The present results further establish variation among the three suborders of Sarcoptiformes with higher rates in the Astigmata than the Oribatida or Endeostigmata (S3 Table), a pattern detected at a nucleotide level in both nuclear and mitochondrial genes [5,7]. Finally, the COI data confirm that rates of evolution are higher in the Mesostigmata than the Ixodida. Given these many cases of congruence, the detection of high rates of COI evolution in the Opiliones was unexpected as Klompen et al. [6] found the divergence rates at two nuclear genes were similar in Opiliones to those in orders with slow evolution (Scorpiones, Araneae, Solifugae). By contrast, this study reveals strong evidence for rate acceleration in the Opiliones as they possess the highest BIN Distance (0.300 in Sironidae) for any family and the highest average Family Distance (0.354) for any order. The present study also revealed strident differences in BIN Distance among different families. Some of this variation reflected the fact that families in some orders showed either consistently low or high rates of amino acid substitution. However, families in other orders showed 10–15 fold differences in their rates of amino acid substitution. Klompen et al. [6] reported variable rates among mesostigmatan infraorders, with the highest rates detected in Dermanyssina, but the present study provides much more information on the taxonomic localization of rate variation. We carried out one test to evaluate the possibility that the subjectivity of higher taxonomic categories might explain some of the variation apparent among lineages. The analysis showed that the high levels of amino acid variation among families of Opiliones compared to Araneae were not a consequence of undersplitting. In fact, treating different genera of Opiliones as orders generated the same level of amino acid substitution as that observed in ordinal comparisons, indicating that general patterns of rate variation are robust to shifts in high-level taxonomy.

Indels are uncommon in the COI gene in most animal groups [13,15], and the arachnids are no exception. For example, Blagoev et al. [37] did not detect any indels in their study of COI variation in 1018 species of Canadian spiders. However, their absence is not universal. Boyer et al. [11] found several COI deletions in species from the opilionid family Sironidae, and this study detected them within 31 of the 255 arachnid families examined. Although some deletions were conserved within members of a genus or, in one case, a suborder, indicating their long-term stability, most appeared to have a recent origin as evidenced by their taxonomic localization. Moreover, variation in lengths and locations coupled with phylogenetic mapping indicated at least 27 independent origins for these deletions. Interestingly, all of the lineages with indels also possessed high rates of amino acid substitution. For example, members of the Sironidae had the second highest BIN distance (0.300) and third-highest Family Distance (0.492) among the 255 arachnid families examined in this study. The generality of this pattern was indicated by the fact that the average Family Distance was significantly higher for the 33 families with indels than for the 222 lacking them (mean = 0.34 versus mean = 0.21, T_80_ = 11.9, *p* <0.000001). Arabi et al. [12] found that lineages with accelerated rates of molecular evolution showed a higher incidence of indels in nuclear genes and more extensive mitochondrial genomic rearrangements. Other work has supported the association between accelerated rates of sequence evolution and mitogenomic rearrangements in other arthropods [16,38].

The present study indicates that the Class Arachnida is composed of lineages with very different patterns of mitochondrial evolution. Most lineages, including nearly all large-bodied species, show low rates of amino acid substitution and no evidence of indels in COI, while other taxa combine high rates of amino acid substitution with frequent indels. Although the present study only examined a segment of one gene, these patterns likely extend across the entire mitochondrial genomes of these taxa. Why do different lineages of arachnids show such dramatic differences in their levels of amino acid reconfiguration? Past studies have linked rates of nucleotide substitution to variation in body size, DNA replication and repair efficiency [5], metabolic rate, and lifespan [6]. The present study indicated an inverse relationship between each of the three Distance metrics and generation length, supporting the common linkage between short lifespans and accelerated evolution. However, certain families with long generation times, such as Cyphophthalmi [39], possessed some of the highest divergences. Generation length is variable between families within an order, and often even at lower hierarchical levels. An analysis of average generation length for families in comparison with Family and BIN Distance would provide deeper insights, but more work is needed to improve the breadth of life history data across Arachnida. Additionally, this correlation might arise as a result of other factor(s) associated with generation length. Breeding system variation is one candidate as species with long life cycles (and low rates of COI evolution) all reproduce sexually, while many of the small-bodied taxa reproduce asexually (e.g. oribatids) or via haplodiploidy. In fact, many of the families with the highest divergences reproduce by haplodiploidy including four families of Mesostigmata (Ascidae, Phytoseiidae, Laleapidae, Macrochelidae), five families of Trombidiformes (Eriophyidae, Scutacaridae, Stigmaeidae, Tetranychidae, Tydeidae), and many of the fast-evolving families of Sarcoptiformes in the suborder Astigmata [26]. Although haplodiploidy has not been reported in certain orders with accelerated rates, such as Pseudoscorpiones and Opiliones, some forms of haplodiploidy, particularly those involving male paternal chromosome elimination, can only be revealed through breeding studies which examine segregation patterns at polymorphic markers [40], studies which do not seem to have been conducted on any species in these orders.

In summary, this study has provided new details on patterns and rates of mitochondrial genome evolution in arachnid lineages based on the analysis of amino acid diversity in COI. The results indicate substantial differences among lineages in rates of amino acid substitution and structural variation. Arachnid lineages with accelerated rates of amino acid evolution not only exhibit larger divergences to the common ancestor, but also marked divergence between and within their families. Efforts to gain a deeper understanding of the factors responsible for this variation would be advanced through the comparison of whole mitochondrial genomes in taxa spanning the spectrum of divergence rates. Further parameterization of the COI sequence library to obtain representation for all families and more species from each family would also help to clarify patterns of rate variation. However, the congruence between results obtained from the analysis of barcode data and earlier phylogenetic studies reveals the way in which COI sequence data can provide an overview of patterns of amino acid evolution across both other groups of arthropods and animal life at large.

## Supporting Information

S1 Alignment Sequence alignment of the MYBCRED dataset. (FAS)

S1 Fig Linear regression of BIN Distance against the number of A) BINs and B) genera in a family. (PDF)

S2 Fig Secondary structure of the barcode region in A) Mesostigmata B) Sarcoptiformes, C) Trombidiformes D) Opiliones and E) Pseudoscorpiones, showing sites of insertions (+) and deletions (x). (PDF)

S1 Table List of GenBank Accession numbers for each sequence in the full dataset DS-MYBCA (dx.doi.org/10.5883/DS-MYBCA, n = 23185) on BOLD, the Barcode of Life Datasystems. (PDF)

S2 Table List of sequences from each order used for family level analyses and the outgroup (n = 4178), with associated taxonomic assignments, Process IDs, GenBank Accession numbers, from the dataset MYBCRED (dx.doi.org/10.5883/DS-MYBCRED) on BOLD, the Barcode of Life Datasystems. (PDF)

S3 Table Mean Dayhoff distance (+/- SE) based on amino acid divergences in the barcode region of COI for ten orders of arachnids with data for more than one family. Values are reported for the divergences among families in each order (Family Distance) and among the BINs in each family (BIN Distance). All Distances > 0.25 are shown in bold, while families highlighted in green are those which included at least one species with an indel.
(PDF)

S4 Table Mean generation time estimated for 12 of the 16 arachnid orders. (PDF)
