# Correction: Patterns of Protein Evolution in Cytochrome *c* Oxidase 1 (COI) from the Class Arachnida

**DOI:** 10.1371/journal.pone.0138167

**Published:** 2015-09-10

**Authors:** Monica R Young, Paul D. N. Hebert

There are errors in the formatting of Figs 1, 2, 5 and 7. Please view the correct Figs 1, 2, 5 and 7 here.

**Fig 1:**
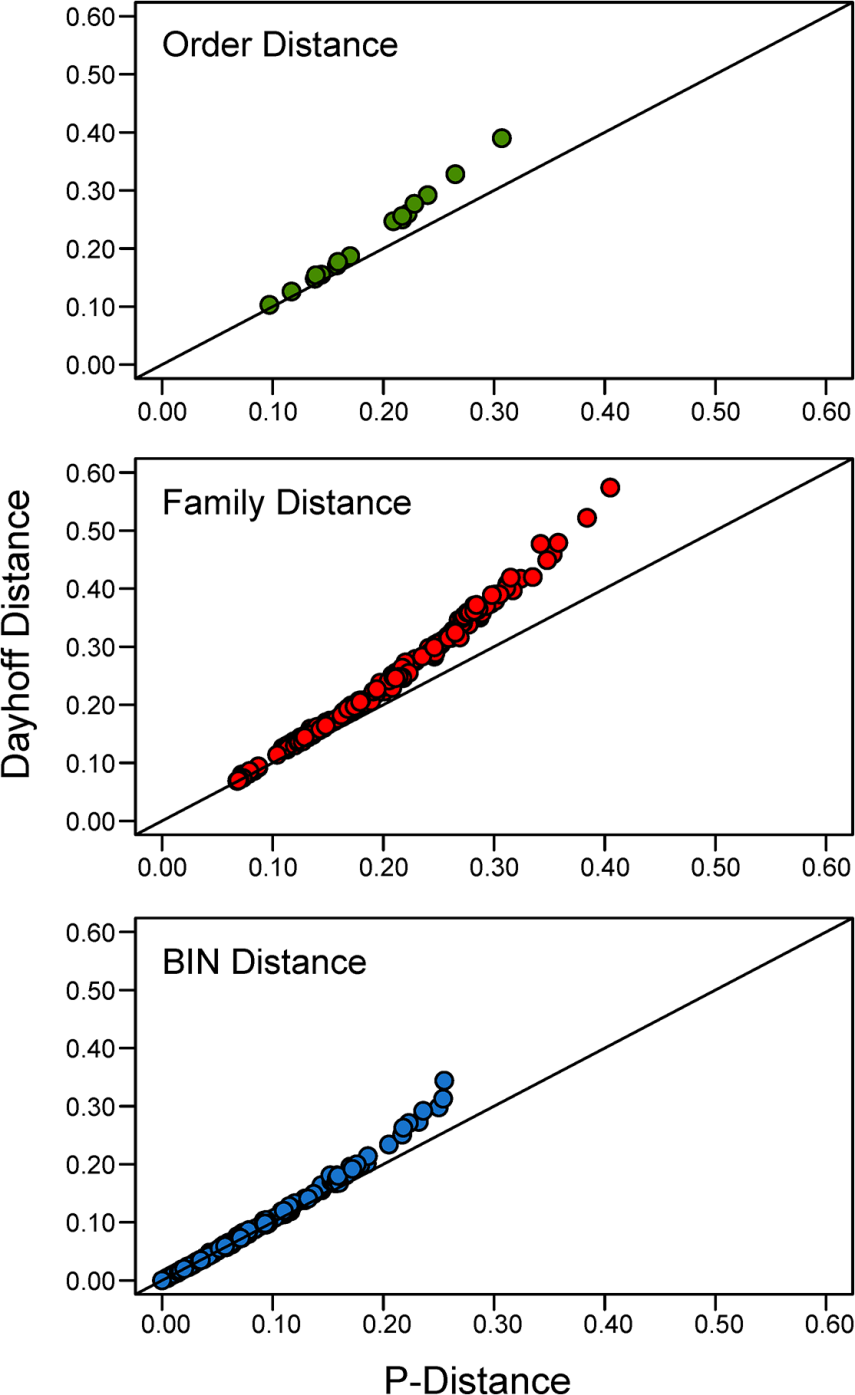
The relationship between p-distance and Dayhoff distance based on amino acid divergences at COI for three taxonomic levels, Order, Family, and BIN. The solid line plots the case where p-distance and Dayhoff distance are identical.

**Fig 2:**
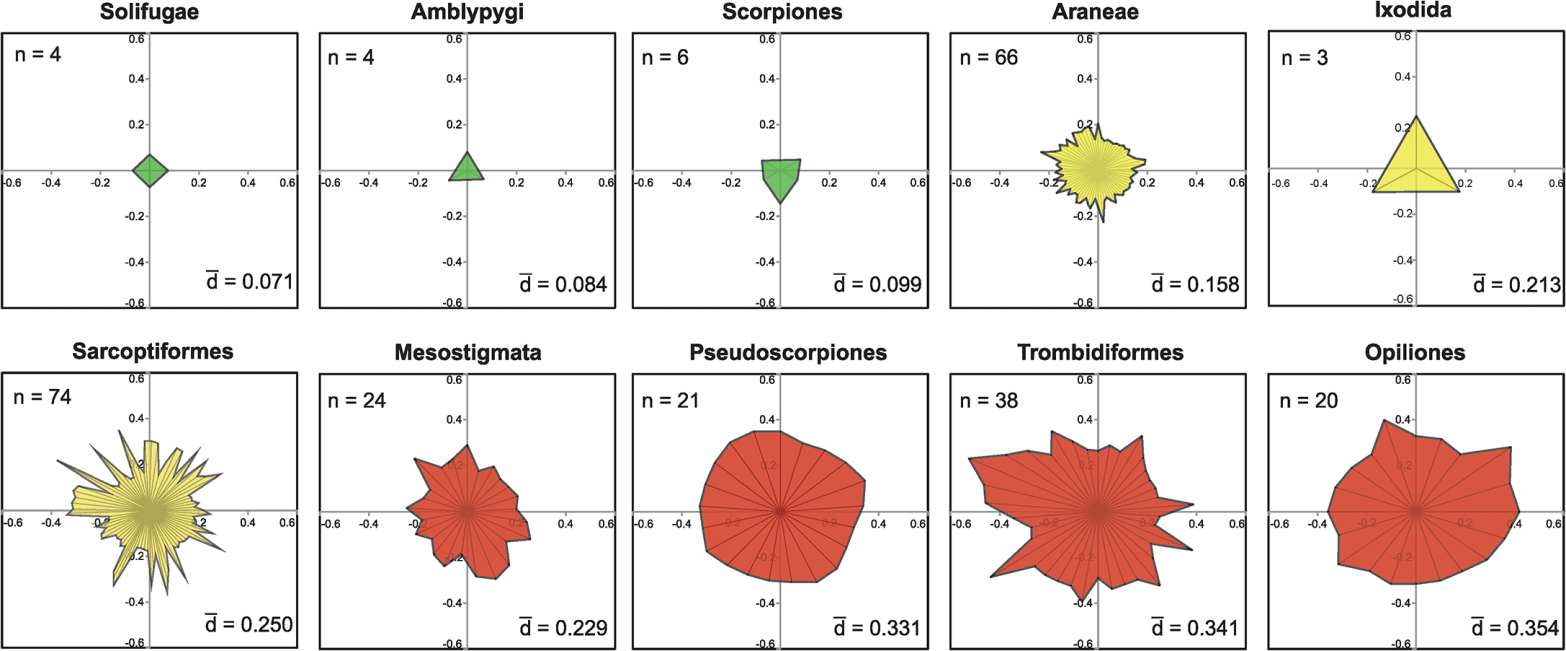
Vector plots showing amino acid divergences (Dayhoff Distance) in the barcode region of COI for the families in ten arachnid orders. n is the number of families in each order and d is the mean family divergence of each order. Different colors highlight the orders in the three levels of divergence.

**Fig 5:**
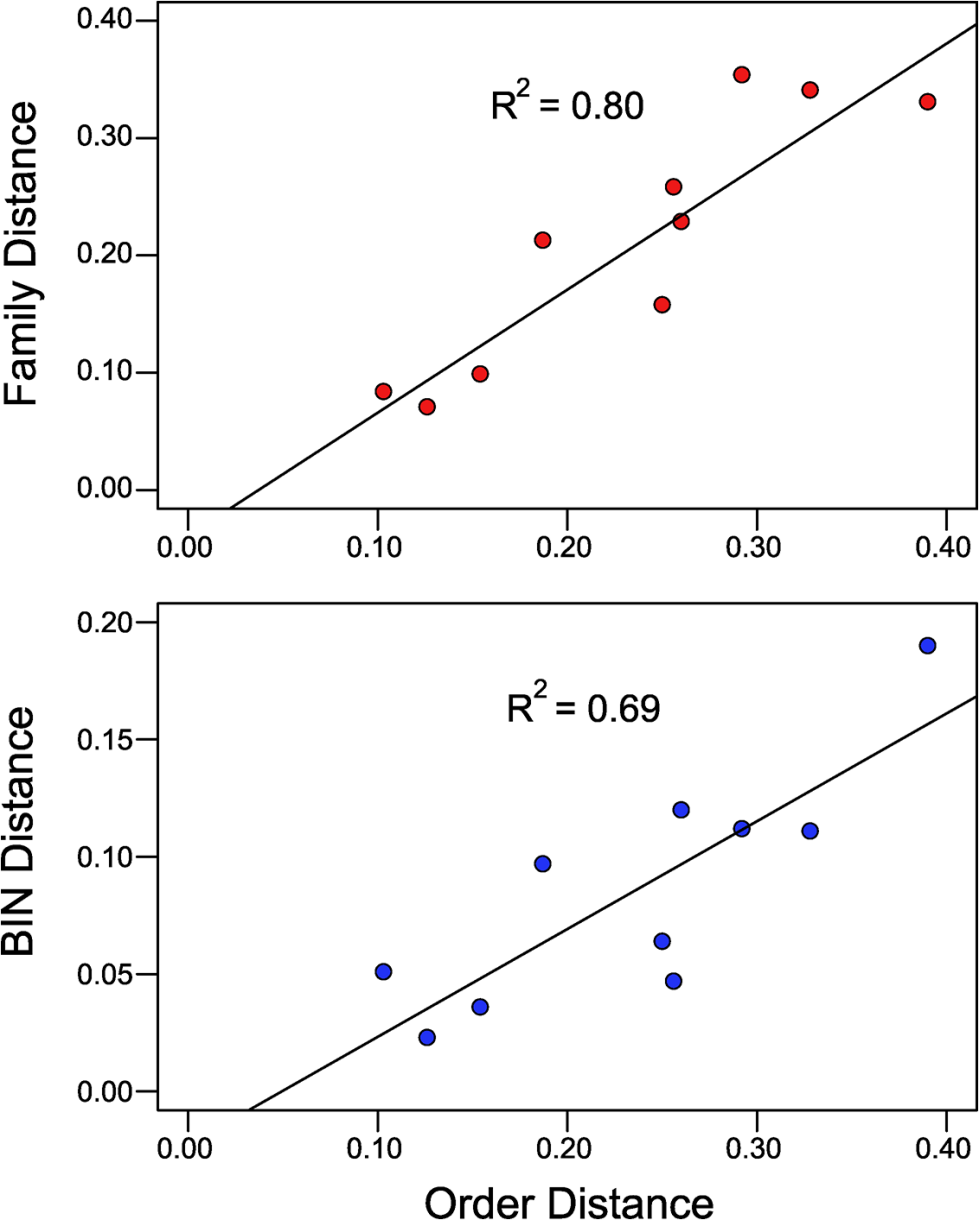
Regression of the Order Distance (Dayhoff distance) based on amino acid divergence in the barcode region of COI to the outgroup against mean Family Distance, and mean BIN Distance.

**Fig 7:**
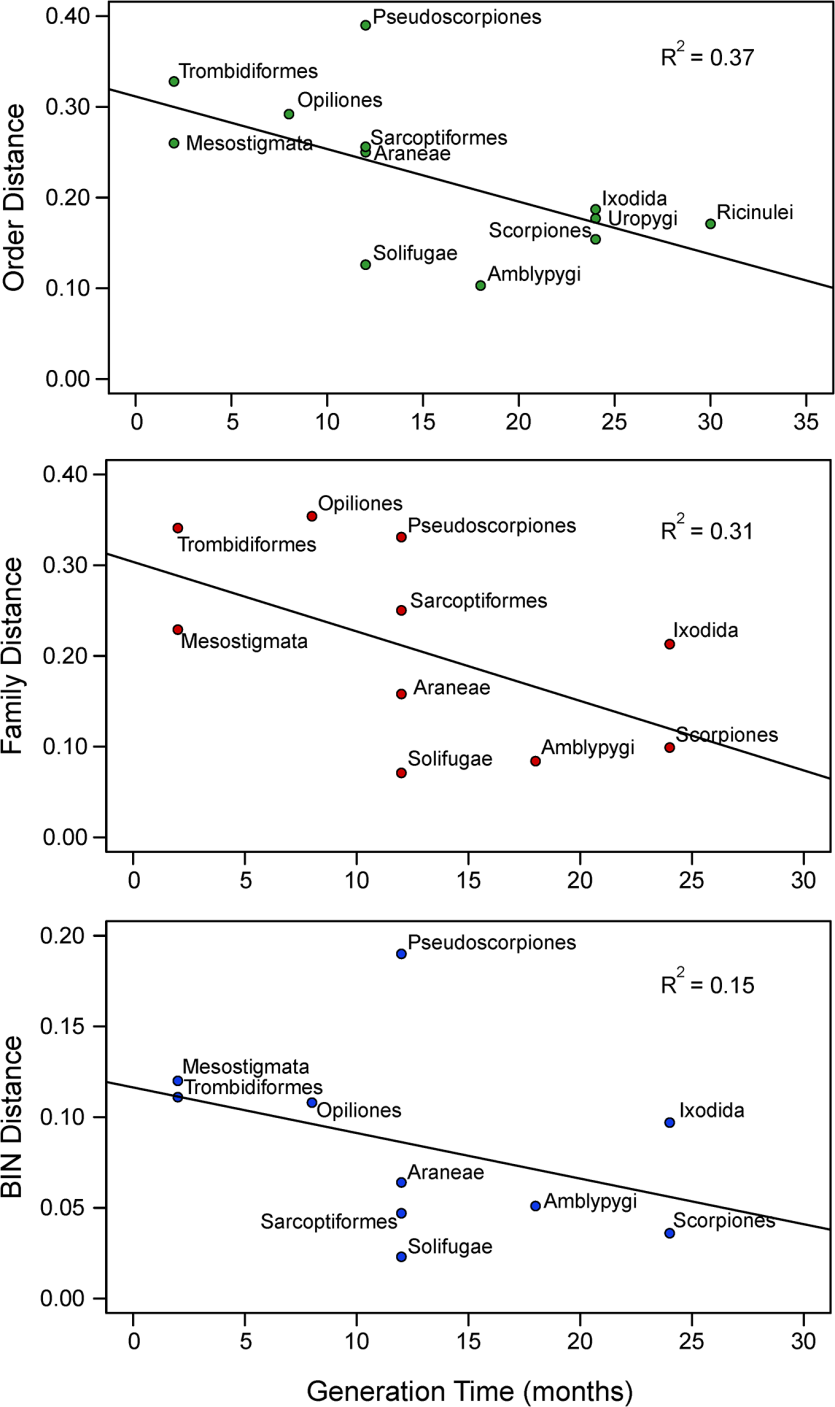
Linear regression of the relationship between generation time and average Dayhoff distances at three taxonomic levels, Order, Family, and BIN.
