# Supplementary figures and images for: Patterns of Protein Evolution in Cytochrome c Oxidase 1 (COI) from the Class Arachnida
Source: PLoS One. 2015 Aug 26;10(8):e0135053. doi: 10.1371/journal.pone.0135053 (PMC4550450; doi:10.1371/journal.pone.0135053)

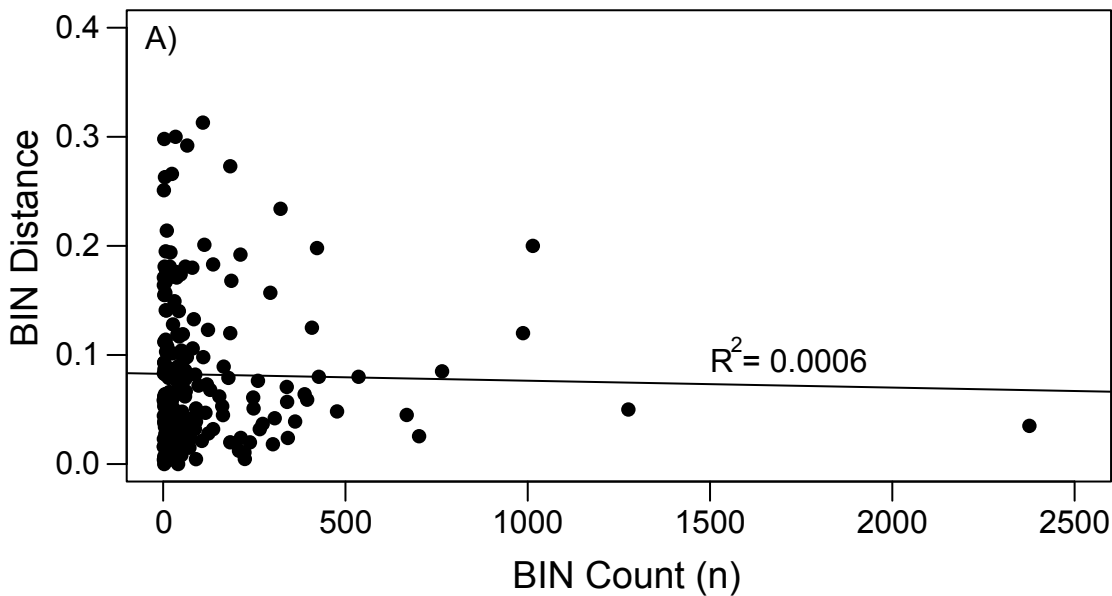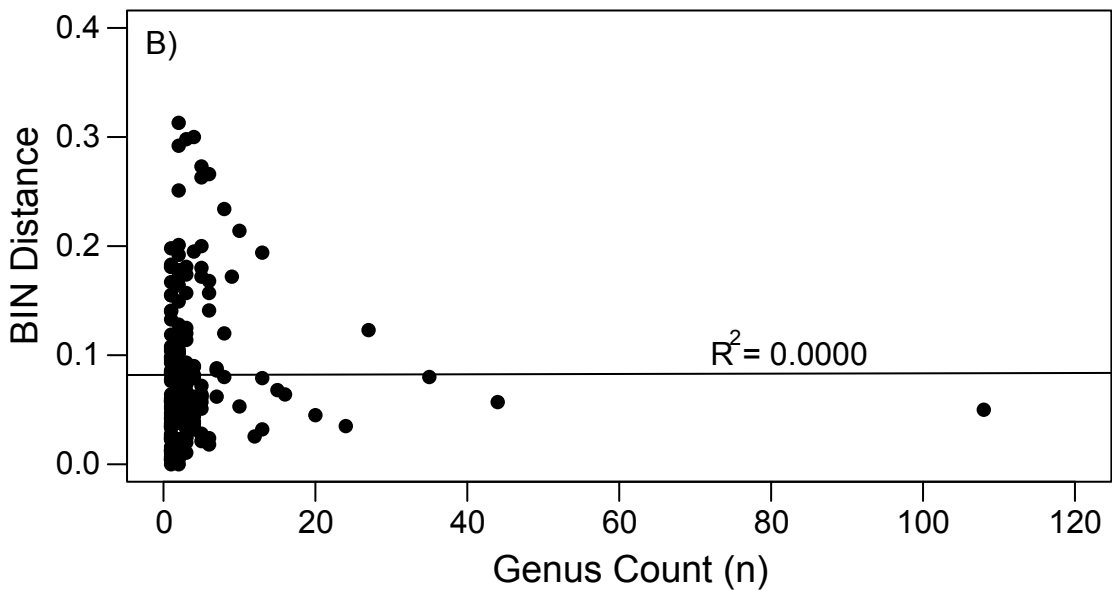

Supplement: S1 Fig — (PDF) [file pone.0135053.s002.pdf]

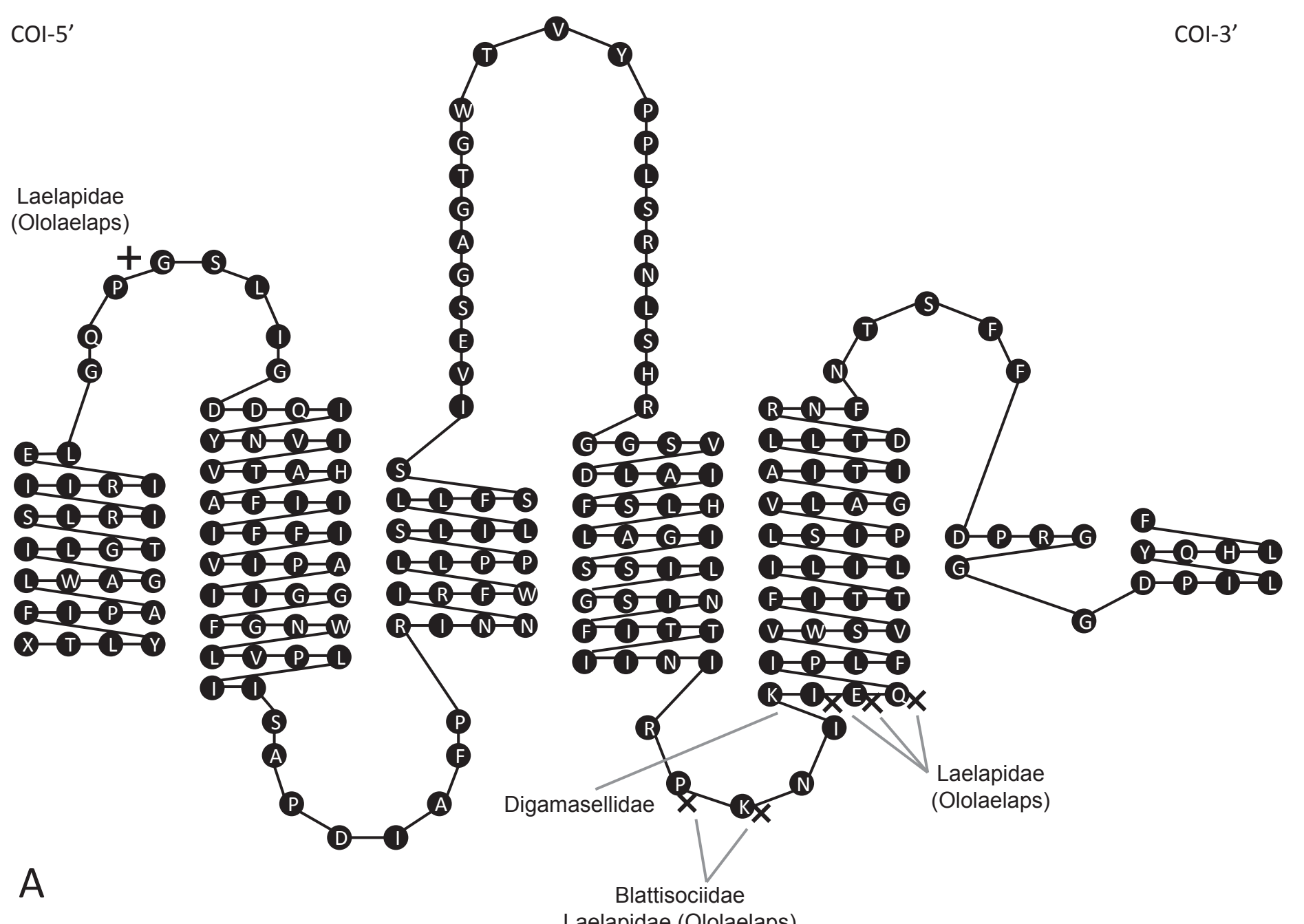

Supplement: S2 Fig — (PDF) [file pone.0135053.s003.pdf]
