# Supplementary material for: Patterns of Protein Evolution in Cytochrome c Oxidase 1 (COI) from the Class Arachnida
Source: PLoS One. 2015 Aug 26;10(8):e0135053. doi: 10.1371/journal.pone.0135053 (PMC4550450; doi:10.1371/journal.pone.0135053)
Supplement: S1 Table — (PDF) [file pone.0135053.s004.pdf]

S1 Table. List of GenBank Accession numbers for each sequence in the full dataset DS-MYBCA (dx.doi.org/10.5883/DS-MYBCA, n = 23185) on BOLD, the Barcode of Life Datasystems.

Order	Family	*Genus/Species*	Process ID	GenBank
Amblypygi	Charinidae	*Sarax*	GACAM014-13	JN018110
Amblypygi	Charontidae	*Stygophrynus*	GACAM011-13	JN018113
Amblypygi	Charontidae	*Stygophrynus*	GACAM012-13	JN018112
Amblypygi	Charontidae	*Stygophrynus*	GACAM013-13	JN018111
Amblypygi	Phrynichidae	*Damon diadema*	GBCH3455-09	NC_011293
Amblypygi	Phrynichidae	*Damon diadema*	GBCH3529-09	FJ204233
Amblypygi	Phrynichidae	*Phrynichus orientalis*	GACAM001-13	JN018195
Amblypygi	Phrynichidae	*Phrynichus orientalis*	GACAM008-13	JN018116
Amblypygi	Phrynidae	*Heterophrynus longicornis*	GACAM005-13	JN018119
Amblypygi	Phrynidae	*Phrynus sp. 1 SEM-2008*	GBCH2392-08	EU520641
Amblypygi	Phrynidae	*Phrynus sp. 1 SEM-2008*	GBCH3208-08	NC_010775
Araneae	Actinopodidae	*Missulena*	GBCH8413-13	KC708095
Araneae	Actinopodidae	*Missulena*	GBCH8415-13	KC708093
Araneae	Actinopodidae	*Missulena*	GBCH8416-13	KC708092
Araneae	Actinopodidae	*Missulena*	GBCH8419-13	KC708089
Araneae	Actinopodidae	*Missulena*	GBCH8420-13	KC708088
Araneae	Actinopodidae	*Missulena*	GBCH8421-13	KC708087
Araneae	Actinopodidae	*Missulena*	GBCH8422-13	KC708086
Araneae	Actinopodidae	*Missulena*	GBCH8423-13	KC708085
Araneae	Actinopodidae	*Missulena*	GBCH8424-13	KC708084
Araneae	Actinopodidae	*Missulena*	GBCH8425-13	KC708083
Araneae	Actinopodidae	*Missulena*	GBCH8426-13	KC708082
Araneae	Actinopodidae	*Missulena*	GBCH8427-13	KC708081
Araneae	Actinopodidae	*Missulena*	GBCH8428-13	KC708080
Araneae	Actinopodidae	*Missulena*	GBCH8429-13	KC708079
Araneae	Actinopodidae	*Missulena*	GBCH8430-13	KC708078
Araneae	Actinopodidae	*Missulena*	GBCH8431-13	KC708077
Araneae	Actinopodidae	*Missulena*	GBCH8432-13	KC708076
Araneae	Actinopodidae	*Missulena*	GBCH8433-13	KC708075
Araneae	Actinopodidae	*Missulena*	GBCH8434-13	KC708074
Araneae	Actinopodidae	*Missulena granulosa*	GBCH8414-13	KC708094
Araneae	Actinopodidae	*Missulena pruinosa*	GBCH8412-13	KC708096
Araneae	Agelenidae	*Agelena koreana*	GBCH11069-13	JN817201
Araneae	Agelenidae	*Agelena labyrinthica*	GBCH11071-13	JN817199
Araneae	Agelenidae	*Agelena limbata*	GBCH11072-13	JN817198
Araneae	Agelenidae	*Agelenopsis aleenae*	GBCH1045-06	AY770786
Araneae	Agelenidae	*Agelenopsis aleenae*	GBCH1046-06	AY770787
Araneae	Agelenidae	*Agelenopsis aleenae*	GBCH1047-06	AY770788
Araneae	Agelenidae	*Agelenopsis aleenae*	GBCH1048-06	AY770789
Araneae	Agelenidae	*Agelenopsis aleenae*	GBCH1049-06	AY770790
Araneae	Agelenidae	*Agelenopsis aleenae*	GBCH1050-06	AY770791
Araneae	Agelenidae	*Agelenopsis aleenae*	GBCH1077-06	AY770818
Araneae	Agelenidae	*Agelenopsis aleenae*	GBCH1078-06	AY770819
Araneae	Agelenidae	*Agelenopsis aleenae*	GBCH1079-06	AY770820
Araneae	Agelenidae	*Agelenopsis aleenae*	GBCH1080-06	AY770821
Araneae	Agelenidae	*Agelenopsis aleenae*	GBCH1081-06	AY770822
Araneae	Agelenidae	*Agelenopsis aleenae*	GBCH1082-06	AY770823
Araneae	Agelenidae	*Agelenopsis aleenae*	GBCH1083-06	AY770824
Araneae	Agelenidae	*Agelenopsis aleenae*	GBCH1084-06	AY770825

Araneae	Agelenidae	*Agelenopsis aleenae*	GBCH1085-06	AY770826
Araneae	Agelenidae	*Agelenopsis aleenae*	GBCH1086-06	AY770827
Araneae	Agelenidae	*Agelenopsis aperta*	GBCH0984-06	AY676066
Araneae	Agelenidae	*Agelenopsis aperta*	GBCH0986-06	AY676068
Araneae	Agelenidae	*Agelenopsis aperta*	GBCH0987-06	AY676069
Araneae	Agelenidae	*Agelenopsis aperta*	GBCH0988-06	AY676070
Araneae	Agelenidae	*Agelenopsis aperta*	GBCH0990-06	AY676072
Araneae	Agelenidae	*Agelenopsis aperta*	GBCH0991-06	AY676073
Araneae	Agelenidae	*Agelenopsis aperta*	GBCH0992-06	AY676074
Araneae	Agelenidae	*Agelenopsis aperta*	GBCH0993-06	AY676075
Araneae	Agelenidae	*Agelenopsis aperta*	GBCH0995-06	AY676077
Araneae	Agelenidae	*Agelenopsis aperta*	GBCH0997-06	AY676079
Araneae	Agelenidae	*Agelenopsis aperta*	GBCH0998-06	AY676080
Araneae	Agelenidae	*Agelenopsis aperta*	GBCH1000-06	AY676082
Araneae	Agelenidae	*Agelenopsis aperta*	GBCH1002-06	AY676084
Araneae	Agelenidae	*Agelenopsis aperta*	GBCH1003-06	AY676085
Araneae	Agelenidae	*Agelenopsis aperta*	GBCH1006-06	AY676088
Araneae	Agelenidae	*Agelenopsis aperta*	GBCH1013-06	AY676095
Araneae	Agelenidae	*Agelenopsis aperta*	GBCH1014-06	AY676096
Araneae	Agelenidae	*Agelenopsis aperta*	GBCH1015-06	AY676097
Araneae	Agelenidae	*Agelenopsis aperta*	GBCH1016-06	AY676098
Araneae	Agelenidae	*Agelenopsis aperta*	GBCH1017-06	AY676099
Araneae	Agelenidae	*Agelenopsis aperta*	GBCH1031-06	AY676113
Araneae	Agelenidae	*Agelenopsis aperta*	GBCH1037-06	AY770778
Araneae	Agelenidae	*Agelenopsis aperta*	GBCH1040-06	AY770781
Araneae	Agelenidae	*Agelenopsis aperta*	GBCH1042-06	AY770783
Araneae	Agelenidae	*Agelenopsis aperta*	GBCH1043-06	AY770784
Araneae	Agelenidae	*Agelenopsis aperta*	GBCH1044-06	AY770785
Araneae	Agelenidae	*Agelenopsis aperta*	GBCH4047-09	FJ607552
Araneae	Agelenidae	*Agelenopsis emertoni*	ARONT059-09	GU682803
Araneae	Agelenidae	*Agelenopsis longistyla*	GBCH1073-06	AY770814
Araneae	Agelenidae	*Agelenopsis longistyla*	GBCH1087-06	AY770828
Araneae	Agelenidae	*Agelenopsis n. sp. NAA-2005*	GBCH1076-06	AY770817
Araneae	Agelenidae	*Agelenopsis naevia*	GBCH1060-06	AY770801
Araneae	Agelenidae	*Agelenopsis naevia*	GBCH1068-06	AY770809
Araneae	Agelenidae	*Agelenopsis oklahoma*	GBCH1062-06	AY770803
Araneae	Agelenidae	*Agelenopsis oklahoma*	GBCH1063-06	AY770804
Araneae	Agelenidae	*Agelenopsis oklahoma*	GBCH1067-06	AY770808
Araneae	Agelenidae	*Agelenopsis spatula*	GBCH1061-06	AY770802
Araneae	Agelenidae	*Agelenopsis utahana*	ARONT086-09	GU682802
Araneae	Agelenidae	*Agelenopsis utahana*	CNBPE681-13	KM828355
Araneae	Agelenidae	*Agelenopsis utahana*	CNBPF441-13	KM828725
Araneae	Agelenidae	*Agelenopsis utahana*	CNBPG506-13	KM834612
Araneae	Agelenidae	*Agelenopsis utahana*	CNBPO527-13	KM826149
Araneae	Agelenidae	*Agelenopsis utahana*	CNBPS425-13	KM830810
Araneae	Agelenidae	*Agelenopsis utahana*	CNEIC3168-12	KM828675
Araneae	Agelenidae	*Agelenopsis utahana*	CNEID3466-12	KM838241
Araneae	Agelenidae	*Agelenopsis utahana*	CNEID3467-12	KM831838
Araneae	Agelenidae	*Agelenopsis utahana*	CNEID3470-13	KM833241
Araneae	Agelenidae	*Agelenopsis utahana*	CNEIE1946-13	KM831224
Araneae	Agelenidae	*Agelenopsis utahana*	CNGLF1916-13	KM825755
Araneae	Agelenidae	*Agelenopsis utahana*	CNJAE764-12	KM837452
Araneae	Agelenidae	*Agelenopsis utahana*	CNPAC646-13	KM827869

Araneae	Agelenidae	*Agelenopsis utahana*	CNPAE541-13	KM827574
Araneae	Agelenidae	*Agelenopsis utahana*	CNPAM002-13	KM837818
Araneae	Agelenidae	*Agelenopsis utahana*	CNRME5223-13	KM840698
Araneae	Agelenidae	*Agelenopsis utahana*	CNRME5225-13	KM838563
Araneae	Agelenidae	*Agelenopsis utahana*	CNRME5226-13	KM840006
Araneae	Agelenidae	*Agelenopsis utahana*	CNSLE488-12	KM830993
Araneae	Agelenidae	*Agelenopsis utahana*	CNSLE489-12	KM831066
Araneae	Agelenidae	*Agelenopsis utahana*	CNSLG307-12	KM834583
Araneae	Agelenidae	*Agelenopsis utahana*	CNSLI535-12	KM838206
Araneae	Agelenidae	*Agelenopsis utahana*	CNSLJ276-12	KM829562
Araneae	Agelenidae	*Agelenopsis utahana*	CNSLJ277-12	KM833757
Araneae	Agelenidae	*Agelenopsis utahana*	CNSLS002-13	KM829000
Araneae	Agelenidae	*Agelenopsis utahana*	CNSLS016-13	KM833859
Araneae	Agelenidae	*Agelenopsis utahana*	CNSLS017-13	KM835780
Araneae	Agelenidae	*Agelenopsis utahana*	CNSLT001-13	KM829688
Araneae	Agelenidae	*Agelenopsis utahana*	CNSLT007-13	KM828680
Araneae	Agelenidae	*Agelenopsis utahana*	CNSLT008-13	KM831448
Araneae	Agelenidae	*Agelenopsis utahana*	CNSLU003-13	KM831635
Araneae	Agelenidae	*Agelenopsis utahana*	CNWLF002-12	KM837341
Araneae	Agelenidae	*Agelenopsis utahana*	GBCH1054-06	AY770795
Araneae	Agelenidae	*Agelenopsis utahana*	GBCH1070-06	AY770811
Araneae	Agelenidae	*Agelenopsis utahana*	GBCH1071-06	AY770812
Araneae	Agelenidae	*Agelenopsis utahana*	GBCH1072-06	AY770813
Araneae	Agelenidae	*Agelenopsis utahana*	GBCH1088-06	AY770829
Araneae	Agelenidae	*Agelenopsis utahana*	JSARA009-11	KP653572
Araneae	Agelenidae	*Agelenopsis utahana*	RBCH014-04	DQ127510
Araneae	Agelenidae	*Agelenopsis utahana*	RBCH037-04	DQ127485
Araneae	Agelenidae	*Agelenopsis utahana*	RBCH192-04	DQ127338
Araneae	Agelenidae	*Agelenopsis utahana*	RBINA3391-13	KP655592
Araneae	Agelenidae	*Agelenopsis utahana*	SPRMA268-10	HQ580869
Araneae	Agelenidae	*Agelenopsis utahana*	SPRMA489-10	JF887025
Araneae	Agelenidae	*Agelenopsis utahana*	SPRMA490-10	JF887026
Araneae	Agelenidae	*Agelenopsis utahana*	SPRMA699-10	JF887132
Araneae	Agelenidae	*Allagelena difficilis*	GBCH11070-13	JN817200
Araneae	Agelenidae	*Barronopsis sp. NAA-2005*	GBCH1069-06	AY770810
Araneae	Agelenidae	*Barronopsis texana*	GBCH1055-06	AY770796
Araneae	Agelenidae	*Barronopsis texana*	GBCH1074-06	AY770815
Araneae	Agelenidae	*Barronopsis texana*	GBCH1075-06	AY770816
Araneae	Agelenidae	*Coras juvenilis*	ARONT465-10	HM880617
Araneae	Agelenidae	*Coras juvenilis*	ARONT517-10	HQ924407
Araneae	Agelenidae	*Coras juvenilis*	ARONT524-10	HQ924413
Araneae	Agelenidae	*Coras juvenilis*	ERSPI008-08	KP654727
Araneae	Agelenidae	*Coras juvenilis*	ERSPI058-08	KP653015
Araneae	Agelenidae	*Coras juvenilis*	ERSPI100-08	KP648297
Araneae	Agelenidae	*Coras juvenilis*	ERSPI190-09	KP645864
Araneae	Agelenidae	*Coras juvenilis*	SPRMA532-10	JF887044
Araneae	Agelenidae	*Coras juvenilis*	SPRMA533-10	JF887045
Araneae	Agelenidae	*Coras lamellosus*	ERSPI247-09	HM376097
Araneae	Agelenidae	*Coras montanus*	ARONT425-09	GU682929
Araneae	Agelenidae	*Coras montanus*	ARONT426-09	GU682926
Araneae	Agelenidae	*Coras montanus*	RBCH012-04	DQ127523
Araneae	Agelenidae	*Draconarius coreanus*	GBCH11061-13	JN817209
Araneae	Agelenidae	*Draconarius kayasanensis*	GBCH11060-13	JN817210

Araneae	Agelenidae	*Eratigena agrestis*	ARONT031-09	GU682805
Araneae	Agelenidae	*Eratigena agrestis*	ARONT090-09	GU682826
Araneae	Agelenidae	*Eratigena agrestis*	ARONT091-09	GU682806
Araneae	Agelenidae	*Eratigena agrestis*	ARONT296-09	GU682862
Araneae	Agelenidae	*Eratigena agrestis*	ARONT297-09	GU682863
Araneae	Agelenidae	*Eratigena agrestis*	ARONT298-09	GU682860
Araneae	Agelenidae	*Eratigena agrestis*	ARONT299-09	GU682861
Araneae	Agelenidae	*Eratigena agrestis*	ARONT316-09	GU682858
Araneae	Agelenidae	*Eratigena agrestis*	ARONT317-09	GU682859
Araneae	Agelenidae	*Eratigena agrestis*	ARONT320-09	GU682857
Araneae	Agelenidae	*Eratigena agrestis*	ARONT348-09	GU682856
Araneae	Agelenidae	*Eratigena agrestis*	ARONT821-10	HQ924666
Araneae	Agelenidae	*Eratigena agrestis*	ARONT822-10	HQ924667
Araneae	Agelenidae	*Eratigena agrestis*	ARONT823-10	HQ924668
Araneae	Agelenidae	*Eratigena agrestis*	ERSPI326-09	GU682516
Araneae	Agelenidae	*Eratigena agrestis*	ERSPI327-09	GU682507
Araneae	Agelenidae	*Eratigena agrestis*	GACAC333-12	FN554804
Araneae	Agelenidae	*Eratigena agrestis*	GACAC345-12	FN554816
Araneae	Agelenidae	*Eratigena agrestis*	GACAC377-12	FR714880
Araneae	Agelenidae	*Eratigena agrestis*	GACAC378-12	FR714881
Araneae	Agelenidae	*Eratigena agrestis*	RBINA3044-13	KP652954
Araneae	Agelenidae	*Eratigena agrestis*	SPRMA267-10	HQ580868
Araneae	Agelenidae	*Eratigena agrestis*	SPRMA291-10	HQ977164
Araneae	Agelenidae	*Eratigena agrestis*	SPRMA353-10	HQ580906
Araneae	Agelenidae	*Eratigena agrestis*	SPRMA354-10	HQ580907
Araneae	Agelenidae	*Eratigena agrestis*	SPRMA496-10	JF887030
Araneae	Agelenidae	*Eratigena agrestis*	SPRMA497-10	JF887031
Araneae	Agelenidae	*Eratigena agrestis*	SPRMA501-10	JF887035
Araneae	Agelenidae	*Eratigena atrica*	SPRMA277-10	KP648567
Araneae	Agelenidae	*Eratigena atrica*	SPRMA491-10	JF887027
Araneae	Agelenidae	*Eratigena atrica*	SPRMA498-10	JF887032
Araneae	Agelenidae	*Eratigena atrica*	SPRMA499-10	JF887033
Araneae	Agelenidae	*Eratigena atrica*	SPRMA500-10	JF887034
Araneae	Agelenidae	*Eratigena atrica*	SPRMA712-10	KP653954
Araneae	Agelenidae	*Iwogumoa songminjae*	GBCH11059-13	JN817211
Araneae	Agelenidae	*Novalena intermedia*	GBCH1528-06	DQ628618
Araneae	Agelenidae	*Novalena intermedia*	GBCH3530-09	EU979472
Araneae	Agelenidae	*Novalena sp. NAA-2005*	GBCH1057-06	AY770798
Araneae	Agelenidae	*Pireneitega spinivulva*	GBCH11057-13	JN817213
Araneae	Agelenidae	*Tegecoelotes secundus*	GBCH11058-13	JN817212
Araneae	Agelenidae	*Tegenaria domestica*	ARONT321-09	GU682893
Araneae	Agelenidae	*Tegenaria domestica*	ARONT322-09	GU682888
Araneae	Agelenidae	*Tegenaria domestica*	GACAC337-12	FN554808
Araneae	Agelenidae	*Tegenaria domestica*	GACAC346-12	FN554817
Araneae	Agelenidae	*Tegenaria domestica*	GACAC383-12	FR714886
Araneae	Agelenidae	*Tegenaria domestica*	GACAC384-12	FR714887
Araneae	Agelenidae	*Tegenaria domestica*	GBCH1495-06	DQ628544
Araneae	Agelenidae	*Tegenaria domestica*	GBCH2235-07	AY138835
Araneae	Agelenidae	*Tegenaria domestica*	SPRMA492-10	JF887028
Araneae	Agelenidae	*Tegenaria domestica*	SPRMA493-10	JF887029
Araneae	Agelenidae	*Tegenaria duellica*	GBCH2238-07	AY138832
Araneae	Agelenidae	*Wadotes*	TDWGB828-10	HQ979253
Araneae	Agelenidae	*Wadotes calcaratus*	ARONT362-09	GU682855

Araneae	Agelenidae	*Wadotes calcaratus*	ARONT363-09	GU682892
Araneae	Agelenidae	*Wadotes hybridus*	RBINA4171-13	KP654006
Araneae	Agelenidae	*Wadotes hybridus*	RBINA4172-13	KP648240
Araneae	Amaurobiidae	*Amaurobius borealis*	ARONT544-10	HQ924431
Araneae	Amaurobiidae	*Amaurobius ferox*	ARONT331-09	GU682867
Araneae	Amaurobiidae	*Amaurobius ferox*	ARONT332-09	GU682864
Araneae	Amaurobiidae	*Amaurobius ferox*	ARONT333-09	GU682865
Araneae	Amaurobiidae	*Amaurobius ferox*	GACAC348-12	FN554819
Araneae	Amaurobiidae	*Amaurobius ferox*	RBCH034-04	DQ127498
Araneae	Amaurobiidae	*Amaurobius sp. 1RB*	SPRMA722-10	JF887147
Araneae	Amaurobiidae	*Amaurobius sp. BB-2004*	GBCH0807-06	AY560797
Araneae	Amaurobiidae	*Callobius bennetti*	ARONT357-09	GU682866
Araneae	Amaurobiidae	*Callobius bennetti*	CNBPB566-13	KM840316
Araneae	Amaurobiidae	*Callobius bennetti*	RBINA3426-13	KP654497
Araneae	Amaurobiidae	*Callobius enus*	SPRMA296-10	HQ977168
Araneae	Amaurobiidae	*Callobius enus*	SPRMA861-12	KP652721
Araneae	Amaurobiidae	*Callobius koreanus*	GBCH11062-13	JN817208
Araneae	Amaurobiidae	*Callobius nomeus*	CNBAA472-12	KM826555
Araneae	Amaurobiidae	*Callobius nomeus*	CNBAD699-12	KM834280
Araneae	Amaurobiidae	*Callobius nomeus*	CNBAE237-12	KM838795
Araneae	Amaurobiidae	*Callobius nomeus*	CNBAI527-13	KM825321
Araneae	Amaurobiidae	*Callobius nomeus*	SPRMA300-10	HQ977171
Araneae	Amaurobiidae	*Callobius nomeus*	SPRMA528-10	JF887043
Araneae	Amaurobiidae	*Callobius nomeus*	SPRMA700-10	KP648617
Araneae	Amaurobiidae	*Callobius pictus*	SPIAL191-10	HQ580653
Araneae	Amaurobiidae	*Callobius pictus*	SPIAL203-10	HQ580661
Araneae	Amaurobiidae	*Callobius pictus*	SPIAL239-10	HQ580687
Araneae	Amaurobiidae	*Callobius pictus*	SPIAL251-10	HQ580697
Araneae	Amaurobiidae	*Callobius pictus*	SPRMA106-10	HQ580746
Araneae	Amaurobiidae	*Callobius pictus*	SPRMA523-10	JF887038
Araneae	Amaurobiidae	*Callobius pictus*	SPRMA524-10	JF887039
Araneae	Amaurobiidae	*Callobius pictus*	SPRMA726-10	JF887149
Araneae	Amaurobiidae	*Callobius pictus*	SPRMA960-12	KP655754
Araneae	Amaurobiidae	*Callobius severus*	SPRMA274-10	HQ580875
Araneae	Amaurobiidae	*Callobius severus*	SPRMA525-10	JF887040
Araneae	Amaurobiidae	*Callobius severus*	SPRMA526-10	JF887041
Araneae	Amaurobiidae	*Callobius severus*	SPRMA527-10	JF887042
Araneae	Amaurobiidae	*Callobius sp. TAB-2009*	GBCH4040-09	FJ607559
Araneae	Amaurobiidae	*Cybaeopsis euopla*	CNPAA471-13	KM837251
Araneae	Amaurobiidae	*Cybaeopsis euopla*	CNPAA472-13	KM837204
Araneae	Amaurobiidae	*Cybaeopsis euopla*	SPICH998-09	GU684503
Araneae	Amaurobiidae	*Cybaeopsis wabritaska*	SPIAL263-10	HQ580706
Araneae	Amaurobiidae	*Cybaeopsis wabritaska*	SPRMA216-10	HQ580829
Araneae	Amaurobiidae	*Zanomys aquilonia*	SPRMA203-10	HQ580817
Araneae	Amaurobiidae	*Zanomys aquilonia*	SPRMA530-10	KP652907
Araneae	Amaurobiidae	*Zanomys aquilonia*	SPRMA987-12	KP646830
Araneae	Anapidae	*Acrobleps*	GBCH8372-13	GU456874
Araneae	Anapidae	*Acrobleps hygrophilus*	GBCH8374-13	GU456872
Araneae	Anapidae	*Anapisona kethleyi*	GBCH8371-13	GU456875
Araneae	Anapidae	*Conculus*	GBCH8373-13	GU456873
Araneae	Antrodiaetidae	*Antrodiaetus pacificus*	SPRMA003-10	HQ977115
Araneae	Antrodiaetidae	*Antrodiaetus pacificus*	SPRMA004-10	HQ977116
Araneae	Antrodiaetidae	*Antrodiaetus pacificus*	SPRMA270-10	HQ580871

Araneae	Antrodiaetidae	*Antrodiaetus pacificus*	SPRMA282-10	HQ580881
Araneae	Antrodiaetidae	*Antrodiaetus pacificus*	SPRMA711-10	JF887140
Araneae	Anyphaenidae	*Anyphaena*	TDWGB739-10	HQ979183
Araneae	Anyphaenidae	*Anyphaena*	TDWGB859-10	HQ979284
Araneae	Anyphaenidae	*Anyphaena*	TDWGB902-10	HQ979325
Araneae	Anyphaenidae	*Anyphaena*	TDWGB903-10	HQ979326
Araneae	Anyphaenidae	*Anyphaena*	TDWGB931-10	HQ979351
Araneae	Anyphaenidae	*Anyphaena aperta*	CNGIA112-12	KM831709
Araneae	Anyphaenidae	*Anyphaena aperta*	CNGIA113-12	KM837853
Araneae	Anyphaenidae	*Anyphaena aperta*	SPRMA586-10	JF887076
Araneae	Anyphaenidae	*Anyphaena aperta*	SPRMA587-10	JF887077
Araneae	Anyphaenidae	*Anyphaena aperta*	SPRMA588-10	KP647534
Araneae	Anyphaenidae	*Anyphaena celer*	ARONT409-09	HM434065
Araneae	Anyphaenidae	*Anyphaena celer*	CNSLF664-12	KM828550
Araneae	Anyphaenidae	*Anyphaena celer*	CNSLQ088-13	KM840768
Araneae	Anyphaenidae	*Anyphaena celer*	RBINA3304-13	KP654325
Araneae	Anyphaenidae	*Anyphaena pacifica*	SPRMA708-10	JF887139
Araneae	Anyphaenidae	*Anyphaena pectorosa*	ARONT683-10	HQ924542
Araneae	Anyphaenidae	*Anyphaena pectorosa*	ARONT684-10	HQ924543
Araneae	Anyphaenidae	*Anyphaena pectorosa*	ARONT685-10	HQ924544
Araneae	Anyphaenidae	*Anyphaena pectorosa*	ARONT686-10	HQ924545
Araneae	Anyphaenidae	*Anyphaena pectorosa*	PHAUG1824-12	KP653193
Araneae	Anyphaenidae	*Anyphaena pectorosa*	PHAUG1826-12	KP652249
Araneae	Anyphaenidae	*Anyphaena pectorosa*	PHJUN4061-12	KP647318
Araneae	Anyphaenidae	*Anyphaena pectorosa*	PHJUN4063-12	KP647185
Araneae	Anyphaenidae	*Anyphaena pectorosa*	PHJUN4064-12	KP650721
Araneae	Anyphaenidae	*Anyphaena pectorosa*	PHMTU047-10	JN307925
Araneae	Anyphaenidae	*Anyphaena pectorosa*	PHOCT953-12	KP653249
Araneae	Anyphaenidae	*Anyphaena pectorosa*	PHSEP1955-11	KP652773
Araneae	Anyphaenidae	*Anyphaena pectorosa*	PHSEP1959-11	KP646212
Araneae	Anyphaenidae	*Anyphaena pectorosa*	PHSEP1963-11	KP648001
Araneae	Anyphaenidae	*Anyphaena pectorosa*	RBCH169-04	DQ127363
Araneae	Anyphaenidae	*Anyphaena pectorosa*	RBINA1217-13	KP651264
Araneae	Anyphaenidae	*Anyphaena pectorosa*	RBINA2686-13	KP656798
Araneae	Anyphaenidae	*Anyphaena pectorosa*	RBINA2688-13	KP656955
Araneae	Anyphaenidae	*Anyphaena pectorosa*	RBINA2734-13	KP653688
Araneae	Anyphaenidae	*Anyphaena pectorosa*	RBINA2805-13	KP650308
Araneae	Anyphaenidae	*Anyphaena pectorosa*	RBINA2806-13	KP653986
Araneae	Anyphaenidae	*Anyphaena pectorosa*	RBINA2845-13	KP654226
Araneae	Anyphaenidae	*Anyphaena pectorosa*	RBINA3228-13	KP651845
Araneae	Anyphaenidae	*Anyphaena pectorosa*	RBINA3312-13	KP650182
Araneae	Anyphaenidae	*Anyphaena pectorosa*	RBINA3330-13	KP647560
Araneae	Anyphaenidae	*Anyphaena pectorosa*	RBINA3435-13	KP647207
Araneae	Anyphaenidae	*Anyphaena pectorosa*	RBINA3464-13	KP646290
Araneae	Anyphaenidae	*Anyphaena pectorosa*	RBINA3976-13	KP646609
Araneae	Anyphaenidae	*Anyphaena pectorosa*	RBINA3983-13	KP646516
Araneae	Anyphaenidae	*Anyphaena pectorosa*	RBINA417-13	KP646660
Araneae	Anyphaenidae	*Anyphaena pectorosa*	SMTPB10031-13	KP650318
Araneae	Anyphaenidae	*Anyphaena pectorosa*	SMTPB10032-13	KP653291
Araneae	Anyphaenidae	*Anyphaena pectorosa*	SMTPB10033-13	KP647288
Araneae	Anyphaenidae	*Anyphaena pectorosa*	SMTPB10034-13	KP646761
Araneae	Anyphaenidae	*Anyphaena pectorosa*	SMTPB10037-13	KP651968
Araneae	Anyphaenidae	*Anyphaena pectorosa*	SMTPB10038-13	KP646950

Araneae	Anyphaenidae	*Anyphaena pectorosa*	SMTPB1486-13	KP656750
Araneae	Anyphaenidae	*Anyphaena pectorosa*	SMTPB20914-13	KP651048
Araneae	Anyphaenidae	*Anyphaena pectorosa*	SMTPB20915-13	KP649744
Araneae	Anyphaenidae	*Anyphaena pectorosa*	SMTPB20916-13	KP655251
Araneae	Anyphaenidae	*Anyphaena pectorosa*	SMTPB20917-13	KP645833
Araneae	Anyphaenidae	*Anyphaena pectorosa*	SMTPB20918-13	KP656912
Araneae	Anyphaenidae	*Anyphaena pectorosa*	SMTPB20919-13	KP650720
Araneae	Anyphaenidae	*Anyphaena pectorosa*	SMTPB20920-13	KP656701
Araneae	Anyphaenidae	*Anyphaena pectorosa*	SMTPB20921-13	KP653140
Araneae	Anyphaenidae	*Anyphaena pectorosa*	SMTPB8354-13	KP649541
Araneae	Anyphaenidae	*Anyphaena pectorosa*	SMTPB8355-13	KP651775
Araneae	Anyphaenidae	*Hibana gracilis*	CNSLB467-12	KM840094
Araneae	Anyphaenidae	*Hibana gracilis*	CNSLJ279-12	KM827464
Araneae	Anyphaenidae	*Hibana gracilis*	PHMTU084-10	KP648590
Araneae	Anyphaenidae	*Hibana gracilis*	PHMTU090-10	KP654031
Araneae	Anyphaenidae	*Hibana gracilis*	PHSEP1945-11	KP653000
Araneae	Anyphaenidae	*Hibana gracilis*	PHSEP1958-11	KP648612
Araneae	Anyphaenidae	*Hibana gracilis*	SMTPB16626-13	KP645965
Araneae	Anyphaenidae	*Hibana gracilis*	SMTPB16627-13	KP645596
Araneae	Anyphaenidae	*Hibana gracilis*	SMTPB21263-13	KP647149
Araneae	Anyphaenidae	*Hibana gracilis*	SMTPB21265-13	KP651371
Araneae	Anyphaenidae	*Hibana gracilis*	SMTPB7473-13	KP652413
Araneae	Anyphaenidae	*Hibana gracilis*	SMTPB7474-13	KP646172
Araneae	Anyphaenidae	*Hibana gracilis*	SMTPB7475-13	KP653765
Araneae	Anyphaenidae	*Wulfila saltabundus*	ARONT024-09	GU682780
Araneae	Anyphaenidae	*Wulfila saltabundus*	ARONT233-09	GU682729
Araneae	Anyphaenidae	*Wulfila saltabundus*	ARONT266-09	GU682662
Araneae	Anyphaenidae	*Wulfila saltabundus*	ARONT267-09	GU682660
Araneae	Anyphaenidae	*Wulfila saltabundus*	ARONT408-09	GU682895
Araneae	Anyphaenidae	*Wulfila saltabundus*	ARONT708-10	HQ924565
Araneae	Anyphaenidae	*Wulfila saltabundus*	ARONT777-10	HQ924625
Araneae	Anyphaenidae	*Wulfila saltabundus*	ARONT779-10	HQ924627
Araneae	Anyphaenidae	*Wulfila saltabundus*	ARONT780-10	HQ924628
Araneae	Anyphaenidae	*Wulfila saltabundus*	CNPPD2694-12	KJ092138
Araneae	Anyphaenidae	*Wulfila saltabundus*	CNPPD2695-12	KJ085985
Araneae	Anyphaenidae	*Wulfila saltabundus*	CNPPD2699-12	KJ164636
Araneae	Anyphaenidae	*Wulfila saltabundus*	CNPPE2328-12	KJ163389
Araneae	Anyphaenidae	*Wulfila saltabundus*	ERSPI292-09	GU682534
Araneae	Anyphaenidae	*Wulfila saltabundus*	JSARA016-11	KP649280
Araneae	Anyphaenidae	*Wulfila saltabundus*	JSARA017-11	KP646638
Araneae	Anyphaenidae	*Wulfila saltabundus*	JSARA035-11	KP645998
Araneae	Anyphaenidae	*Wulfila saltabundus*	JSARA060-11	KP656181
Araneae	Anyphaenidae	*Wulfila saltabundus*	RBCH136-04	DQ127394
Araneae	Anyphaenidae	*Wulfila saltabundus*	RBINA3314-13	KP657480
Araneae	Anyphaenidae	*Wulfila saltabundus*	RBINA3326-13	KP650559
Araneae	Anyphaenidae	*Wulfila saltabundus*	RBINA3327-13	KP649682
Araneae	Anyphaenidae	*Wulfila saltabundus*	RBINA3335-13	KP649611
Araneae	Anyphaenidae	*Wulfila saltabundus*	RBINA3413-13	KP646645
Araneae	Anyphaenidae	*Wulfila saltabundus*	RBINA3419-13	KP654614
Araneae	Anyphaenidae	*Wulfila saltabundus*	RBINA3570-13	KP647806
Araneae	Anyphaenidae	*Wulfila saltabundus*	RBINA3858-13	KP649477
Araneae	Anyphaenidae	*Wulfila saltabundus*	RBINA3984-13	KP650464
Araneae	Anyphaenidae	*Wulfila saltabundus*	RBINA4371-13	KP655846

Araneae	Anyphaenidae	*Wulfila saltabundus*	RBINA4885-13	KP645529
Araneae	Anyphaenidae	*Wulfila saltabundus*	RBINA4886-13	KP655301
Araneae	Anyphaenidae	*Wulfila saltabundus*	RBINA4890-13	KP656104
Araneae	Anyphaenidae	*Wulfila saltabundus*	RBINA4898-13	KP656187
Araneae	Anyphaenidae	*Wulfila saltabundus*	RBINA5794-13	KP645721
Araneae	Anyphaenidae	*Wulfila saltabundus*	RBINA5799-13	KP651440
Araneae	Anyphaenidae	*Wulfila saltabundus*	RBINA5834-13	KP648881
Araneae	Anyphaenidae	*Wulfila saltabundus*	RBINA5846-13	KP654096
Araneae	Anyphaenidae	*Wulfila saltabundus*	RBINA5847-13	KP655775
Araneae	Anyphaenidae	*Wulfila saltabundus*	RBINA5848-13	KP653813
Araneae	Anyphaenidae	*Wulfila saltabundus*	RBINA815-13	KP653789
Araneae	Anyphaenidae	*Wulfila saltabundus*	SMTPB14559-13	KP649204
Araneae	Araneidae	*Acanthepeira stellata*	ARONT386-09	GU682952
Araneae	Araneidae	*Acanthepeira stellata*	ERSPI298-09	GU682530
Araneae	Araneidae	*Acanthepeira stellata*	ERSPI299-09	GU682531
Araneae	Araneidae	*Aculepeira carbonarioides*	GBADC022-10	HQ956685
Araneae	Araneidae	*Aculepeira packardi*	SSBAC4443-13	KM826210
Araneae	Araneidae	*Aculepeira packardi*	SSBAF7746-13	KM832933
Araneae	Araneidae	*Aculepeira packardi*	SSJAE7788-13	KM834431
Araneae	Araneidae	*Araneus acusisetus*	GBCH11126-13	JN817144
Araneae	Araneidae	*Araneus angulatus*	GBCH11132-13	JN817138
Araneae	Araneidae	*Araneus angulatus*	GBCH11133-13	JN817137
Araneae	Araneidae	*Araneus corticarius*	SPIRU1088-11	KF367836
Araneae	Araneidae	*Araneus diadematus*	ARONT080-09	GU682758
Araneae	Araneidae	*Araneus diadematus*	ARONT283-09	GU682733
Araneae	Araneidae	*Araneus diadematus*	ARONT284-09	GU682736
Araneae	Araneidae	*Araneus diadematus*	ARONT325-09	GU682735
Araneae	Araneidae	*Araneus diadematus*	ARONT326-09	GU682734
Araneae	Araneidae	*Araneus diadematus*	ARONT327-09	GU682585
Araneae	Araneidae	*Araneus diadematus*	ARONT330-09	GU682738
Araneae	Araneidae	*Araneus diadematus*	ERSPI409-09	GU682441
Araneae	Araneidae	*Araneus diadematus*	GBCH4046-09	FJ607553
Araneae	Araneidae	*Araneus diadematus*	GBCH7796-13	JN018130
Araneae	Araneidae	*Araneus diadematus*	SPRMA014-10	HQ977123
Araneae	Araneidae	*Araneus ejusmodi*	GBCH11127-13	JN817143
Araneae	Araneidae	*Araneus gemmoides*	GBCH1208-06	DQ146861
Araneae	Araneidae	*Araneus gemmoides*	SPRMA015-10	HQ977124
Araneae	Araneidae	*Araneus groenlandicola*	RBCH107-04	DQ127427
Araneae	Araneidae	*Araneus groenlandicola*	SWSWE077-09	GU681023
Araneae	Araneidae	*Araneus ishisawai*	GBCH11130-13	JN817140
Araneae	Araneidae	*Araneus iviei*	SSEIB8435-13	KM824673
Araneae	Araneidae	*Araneus iviei*	SSPAA6046-13	KM835613
Araneae	Araneidae	*Araneus iviei*	SSPAA6086-13	KM838576
Araneae	Araneidae	*Araneus iviei*	SSPAB5306-13	KM832263
Araneae	Araneidae	*Araneus iviei*	SSPAC12578-13	KM834008
Araneae	Araneidae	*Araneus marmoreus*	ARONT312-09	GU682584
Araneae	Araneidae	*Araneus marmoreus*	CNEID3468-12	KM832790
Araneae	Araneidae	*Araneus marmoreus*	CNJAE766-12	KM829381
Araneae	Araneidae	*Araneus marmoreus*	CNJAE768-12	KM838487
Araneae	Araneidae	*Araneus marmoreus*	CNWLB648-13	KM839201
Araneae	Araneidae	*Araneus marmoreus*	GBCH3915-09	EU003278
Araneae	Araneidae	*Araneus marmoreus*	JSAUG1693-12	KP649861
Araneae	Araneidae	*Araneus marmoreus*	SPRMA017-10	HQ977125

Araneae	Araneidae	*Araneus marmoreus*	SPRMA018-10	HQ977126
Araneae	Araneidae	*Araneus marmoreus*	SPRMA174-10	HQ580794
Araneae	Araneidae	*Araneus marmoreus*	SPRMA176-10	HQ580796
Araneae	Araneidae	*Araneus marmoreus*	SPRMA706-10	JF887137
Araneae	Araneidae	*Araneus nordmanni*	RBCH175-04	DQ127353
Araneae	Araneidae	*Araneus nordmanni*	SPICH1163-09	GU684587
Araneae	Araneidae	*Araneus nordmanni*	SPRMA130-10	KP655022
Araneae	Araneidae	*Araneus pratensis*	TDWGB735-10	HQ979180
Araneae	Araneidae	*Araneus pratensis*	TDWGB737-10	HQ979182
Araneae	Araneidae	*Araneus quadratus*	GACAC390-12	FR775772
Araneae	Araneidae	*Araneus saevus*	ARONT021-09	GU682760
Araneae	Araneidae	*Araneus saevus*	PHMTT617-10	JN307890
Araneae	Araneidae	*Araneus saevus*	RBCH203-04	DQ127333
Araneae	Araneidae	*Araneus sp. 1GAB*	SSBAE3002-13	KM834430
Araneae	Araneidae	*Araneus sp. 1GAB*	SSBAE3995-13	KM833451
Araneae	Araneidae	*Araneus sp. 1GAB*	SSBAE4114-13	KM839929
Araneae	Araneidae	*Araneus sp. 1GAB*	SSBAE6064-13	KM839354
Araneae	Araneidae	*Araneus sp. 1GAB*	SSJAC2361-13	KM831059
Araneae	Araneidae	*Araneus sp. 2GAB*	ARONT313-09	HM434063
Araneae	Araneidae	*Araneus sp. 2GAB*	CNSLH311-12	KM835210
Araneae	Araneidae	*Araneus stella*	GBCH11128-13	JN817142
Araneae	Araneidae	*Araneus thaddeus*	ARONT153-09	HM434053
Araneae	Araneidae	*Araneus thaddeus*	ARONT572-10	HQ924458
Araneae	Araneidae	*Araneus trifolium*	ARONT279-09	GU682891
Araneae	Araneidae	*Araneus trifolium*	ARONT280-09	GU682656
Araneae	Araneidae	*Araneus trifolium*	ARONT309-09	GU682571
Araneae	Araneidae	*Araneus trifolium*	RBINA3257-13	KP655962
Araneae	Araneidae	*Araneus trifolium*	RBINA3273-13	KP653900
Araneae	Araneidae	*Araneus trifolium*	RBINA3276-13	KP645978
Araneae	Araneidae	*Araneus variegatus*	GBCH11131-13	JN817139
Araneae	Araneidae	*Araniella displicata*	ARONT115-09	HQ924377
Araneae	Araneidae	*Araniella displicata*	ARONT116-09	GU682652
Araneae	Araneidae	*Araniella displicata*	ARONT412-09	GU682953
Araneae	Araneidae	*Araniella displicata*	ARONT413-09	GU682954
Araneae	Araneidae	*Araniella displicata*	ARONT667-10	HQ924526
Araneae	Araneidae	*Araniella displicata*	CNFNR3171-14	KP645647
Araneae	Araneidae	*Araniella displicata*	CNPAF958-13	KM835784
Araneae	Araneidae	*Araniella displicata*	CNPAH374-13	KM825074
Araneae	Araneidae	*Araniella displicata*	PHJUN4059-12	KP657042
Araneae	Araneidae	*Araniella displicata*	PHMTU063-10	JN307932
Araneae	Araneidae	*Araniella displicata*	PHMTU064-10	JN307933
Araneae	Araneidae	*Araniella displicata*	RBCH056-04	DQ127472
Araneae	Araneidae	*Araniella displicata*	RBCH067-04	DQ127467
Araneae	Araneidae	*Araniella displicata*	RBCH077-04	DQ127445
Araneae	Araneidae	*Araniella displicata*	RBCH078-04	DQ127446
Araneae	Araneidae	*Araniella displicata*	RBCH173-04	DQ127351
Araneae	Araneidae	*Araniella displicata*	RBINA1072-13	KP652980
Araneae	Araneidae	*Araniella displicata*	SMTPD1630-13	KP646335
Araneae	Araneidae	*Araniella displicata*	SSBAC2918-12	KM834835
Araneae	Araneidae	*Araniella displicata*	SSBAC4444-13	KM838347
Araneae	Araneidae	*Araniella displicata*	SSBAD5975-13	KM832242
Araneae	Araneidae	*Araniella displicata*	SSBAF6060-13	KM839002
Araneae	Araneidae	*Araniella displicata*	SSFDC5330-14	KR070394

Araneae	Araneidae	*Araniella displicata*	SSWLC4118-13	KM836477
Araneae	Araneidae	*Araniella displicata*	TDWGB890-10	HQ979315
Araneae	Araneidae	*Araniella yaginumai*	GBCH11107-13	JN817163
Araneae	Araneidae	*Argiope amoena*	GBCH11113-13	JN817157
Araneae	Araneidae	*Argiope argentata*	GBCH4045-09	FJ607554
Araneae	Araneidae	*Argiope aurantia*	ERSPI407-09	GU682439
Araneae	Araneidae	*Argiope aurantia*	GBCH1209-06	DQ146862
Araneae	Araneidae	*Argiope aurantia*	GBCH3232-09	FJ525332
Araneae	Araneidae	*Argiope aurantia*	GBCH5085-10	GU353211
Araneae	Araneidae	*Argiope aurantia*	RBCH161-04	DQ127371
Araneae	Araneidae	*Argiope aurantia*	RBCH196-04	DQ127342
Araneae	Araneidae	*Argiope boesenbergi*	GBCH11112-13	JN817158
Araneae	Araneidae	*Argiope bruennichi*	GBCH10000-13	KC193804
Araneae	Araneidae	*Argiope bruennichi*	GBCH10001-13	KC193803
Araneae	Araneidae	*Argiope bruennichi*	GBCH10002-13	KC193802
Araneae	Araneidae	*Argiope bruennichi*	GBCH10003-13	KC193801
Araneae	Araneidae	*Argiope bruennichi*	GBCH10004-13	KC193800
Araneae	Araneidae	*Argiope bruennichi*	GBCH10005-13	KC193799
Araneae	Araneidae	*Argiope bruennichi*	GBCH10006-13	KC193798
Araneae	Araneidae	*Argiope bruennichi*	GBCH10007-13	KC193797
Araneae	Araneidae	*Argiope bruennichi*	GBCH10008-13	KC193796
Araneae	Araneidae	*Argiope bruennichi*	GBCH10009-13	KC193795
Araneae	Araneidae	*Argiope bruennichi*	GBCH10010-13	KC193794
Araneae	Araneidae	*Argiope bruennichi*	GBCH10011-13	KC193793
Araneae	Araneidae	*Argiope bruennichi*	GBCH10012-13	KC193792
Araneae	Araneidae	*Argiope bruennichi*	GBCH10013-13	KC193791
Araneae	Araneidae	*Argiope bruennichi*	GBCH10014-13	KC193790
Araneae	Araneidae	*Argiope bruennichi*	GBCH10015-13	KC193789
Araneae	Araneidae	*Argiope bruennichi*	GBCH10016-13	KC193788
Araneae	Araneidae	*Argiope bruennichi*	GBCH10017-13	KC193787
Araneae	Araneidae	*Argiope bruennichi*	GBCH10184-13	KC195016
Araneae	Araneidae	*Argiope bruennichi*	GBCH10185-13	KC195015
Araneae	Araneidae	*Argiope bruennichi*	GBCH10186-13	KC195014
Araneae	Araneidae	*Argiope bruennichi*	GBCH10187-13	KC195013
Araneae	Araneidae	*Argiope bruennichi*	GBCH10188-13	KC195012
Araneae	Araneidae	*Argiope bruennichi*	GBCH10189-13	KC195011
Araneae	Araneidae	*Argiope bruennichi*	GBCH10190-13	KC195010
Araneae	Araneidae	*Argiope bruennichi*	GBCH10191-13	KC195009
Araneae	Araneidae	*Argiope bruennichi*	GBCH10192-13	KC195008
Araneae	Araneidae	*Argiope bruennichi*	GBCH10193-13	KC195007
Araneae	Araneidae	*Argiope bruennichi*	GBCH10194-13	KC195006
Araneae	Araneidae	*Argiope bruennichi*	GBCH10195-13	KC195005
Araneae	Araneidae	*Argiope bruennichi*	GBCH10196-13	KC195004
Araneae	Araneidae	*Argiope bruennichi*	GBCH10197-13	KC195003
Araneae	Araneidae	*Argiope bruennichi*	GBCH10198-13	KC195002
Araneae	Araneidae	*Argiope bruennichi*	GBCH10199-13	KC195001
Araneae	Araneidae	*Argiope bruennichi*	GBCH10200-13	KC195000
Araneae	Araneidae	*Argiope bruennichi*	GBCH10201-13	KC194999
Araneae	Araneidae	*Argiope bruennichi*	GBCH10202-13	KC194998
Araneae	Araneidae	*Argiope bruennichi*	GBCH10203-13	KC194997
Araneae	Araneidae	*Argiope bruennichi*	GBCH10204-13	KC194996
Araneae	Araneidae	*Argiope bruennichi*	GBCH10205-13	KC194995
Araneae	Araneidae	*Argiope bruennichi*	GBCH10206-13	KC194994

Araneae	Araneidae	*Argiope bruennichi*	GBCH10207-13	KC194993
Araneae	Araneidae	*Argiope bruennichi*	GBCH10208-13	KC194992
Araneae	Araneidae	*Argiope bruennichi*	GBCH10209-13	KC194991
Araneae	Araneidae	*Argiope bruennichi*	GBCH10210-13	KC194990
Araneae	Araneidae	*Argiope bruennichi*	GBCH10211-13	KC194989
Araneae	Araneidae	*Argiope bruennichi*	GBCH10212-13	KC194988
Araneae	Araneidae	*Argiope bruennichi*	GBCH10213-13	KC194987
Araneae	Araneidae	*Argiope bruennichi*	GBCH10214-13	KC194986
Araneae	Araneidae	*Argiope bruennichi*	GBCH10215-13	KC194985
Araneae	Araneidae	*Argiope bruennichi*	GBCH10216-13	KC194984
Araneae	Araneidae	*Argiope bruennichi*	GBCH10217-13	KC194983
Araneae	Araneidae	*Argiope bruennichi*	GBCH10218-13	KC194982
Araneae	Araneidae	*Argiope bruennichi*	GBCH10219-13	KC194981
Araneae	Araneidae	*Argiope bruennichi*	GBCH10220-13	KC194980
Araneae	Araneidae	*Argiope bruennichi*	GBCH10221-13	KC194979
Araneae	Araneidae	*Argiope bruennichi*	GBCH10222-13	KC194978
Araneae	Araneidae	*Argiope bruennichi*	GBCH10223-13	KC194977
Araneae	Araneidae	*Argiope bruennichi*	GBCH10224-13	KC194976
Araneae	Araneidae	*Argiope bruennichi*	GBCH10225-13	KC194975
Araneae	Araneidae	*Argiope bruennichi*	GBCH10226-13	KC194974
Araneae	Araneidae	*Argiope bruennichi*	GBCH10227-13	KC194973
Araneae	Araneidae	*Argiope bruennichi*	GBCH10228-13	KC194972
Araneae	Araneidae	*Argiope bruennichi*	GBCH10229-13	KC194971
Araneae	Araneidae	*Argiope bruennichi*	GBCH10230-13	KC194970
Araneae	Araneidae	*Argiope bruennichi*	GBCH10231-13	KC194969
Araneae	Araneidae	*Argiope bruennichi*	GBCH10232-13	KC194968
Araneae	Araneidae	*Argiope bruennichi*	GBCH10233-13	KC194967
Araneae	Araneidae	*Argiope bruennichi*	GBCH10234-13	KC194966
Araneae	Araneidae	*Argiope bruennichi*	GBCH10235-13	KC194965
Araneae	Araneidae	*Argiope bruennichi*	GBCH10236-13	KC194964
Araneae	Araneidae	*Argiope bruennichi*	GBCH10237-13	KC194963
Araneae	Araneidae	*Argiope bruennichi*	GBCH10238-13	KC194962
Araneae	Araneidae	*Argiope bruennichi*	GBCH10239-13	KC194961
Araneae	Araneidae	*Argiope bruennichi*	GBCH10240-13	KC194960
Araneae	Araneidae	*Argiope bruennichi*	GBCH10241-13	KC194959
Araneae	Araneidae	*Argiope bruennichi*	GBCH10242-13	KC194958
Araneae	Araneidae	*Argiope bruennichi*	GBCH10243-13	KC194957
Araneae	Araneidae	*Argiope bruennichi*	GBCH10244-13	KC194956
Araneae	Araneidae	*Argiope bruennichi*	GBCH10245-13	KC194955
Araneae	Araneidae	*Argiope bruennichi*	GBCH10246-13	KC194954
Araneae	Araneidae	*Argiope bruennichi*	GBCH10247-13	KC194953
Araneae	Araneidae	*Argiope bruennichi*	GBCH10248-13	KC194952
Araneae	Araneidae	*Argiope bruennichi*	GBCH10249-13	KC194951
Araneae	Araneidae	*Argiope bruennichi*	GBCH10250-13	KC194950
Araneae	Araneidae	*Argiope bruennichi*	GBCH10251-13	KC194949
Araneae	Araneidae	*Argiope bruennichi*	GBCH10252-13	KC194948
Araneae	Araneidae	*Argiope bruennichi*	GBCH10253-13	KC194947
Araneae	Araneidae	*Argiope bruennichi*	GBCH10254-13	KC194946
Araneae	Araneidae	*Argiope bruennichi*	GBCH10255-13	KC194945
Araneae	Araneidae	*Argiope bruennichi*	GBCH10256-13	KC194944
Araneae	Araneidae	*Argiope bruennichi*	GBCH10257-13	KC194943
Araneae	Araneidae	*Argiope bruennichi*	GBCH10258-13	KC194942
Araneae	Araneidae	*Argiope bruennichi*	GBCH10259-13	KC194941

Araneae	Araneidae	*Argiope bruennichi*	GBCH10260-13	KC194940
Araneae	Araneidae	*Argiope bruennichi*	GBCH10261-13	KC194939
Araneae	Araneidae	*Argiope bruennichi*	GBCH10262-13	KC194938
Araneae	Araneidae	*Argiope bruennichi*	GBCH10263-13	KC194937
Araneae	Araneidae	*Argiope bruennichi*	GBCH10264-13	KC194936
Araneae	Araneidae	*Argiope bruennichi*	GBCH10265-13	KC194935
Araneae	Araneidae	*Argiope bruennichi*	GBCH10266-13	KC194934
Araneae	Araneidae	*Argiope bruennichi*	GBCH10267-13	KC194933
Araneae	Araneidae	*Argiope bruennichi*	GBCH10268-13	KC194932
Araneae	Araneidae	*Argiope bruennichi*	GBCH10269-13	KC194931
Araneae	Araneidae	*Argiope bruennichi*	GBCH10270-13	KC194930
Araneae	Araneidae	*Argiope bruennichi*	GBCH10271-13	KC194929
Araneae	Araneidae	*Argiope bruennichi*	GBCH10272-13	KC194928
Araneae	Araneidae	*Argiope bruennichi*	GBCH10273-13	KC194927
Araneae	Araneidae	*Argiope bruennichi*	GBCH10274-13	KC194926
Araneae	Araneidae	*Argiope bruennichi*	GBCH10275-13	KC194925
Araneae	Araneidae	*Argiope bruennichi*	GBCH10276-13	KC194924
Araneae	Araneidae	*Argiope bruennichi*	GBCH10277-13	KC194923
Araneae	Araneidae	*Argiope bruennichi*	GBCH10278-13	KC194922
Araneae	Araneidae	*Argiope bruennichi*	GBCH10279-13	KC194921
Araneae	Araneidae	*Argiope bruennichi*	GBCH10280-13	KC194920
Araneae	Araneidae	*Argiope bruennichi*	GBCH10281-13	KC194919
Araneae	Araneidae	*Argiope bruennichi*	GBCH10282-13	KC194918
Araneae	Araneidae	*Argiope bruennichi*	GBCH10283-13	KC194917
Araneae	Araneidae	*Argiope bruennichi*	GBCH10284-13	KC194916
Araneae	Araneidae	*Argiope bruennichi*	GBCH10285-13	KC194915
Araneae	Araneidae	*Argiope bruennichi*	GBCH10286-13	KC194914
Araneae	Araneidae	*Argiope bruennichi*	GBCH10287-13	KC194913
Araneae	Araneidae	*Argiope bruennichi*	GBCH10288-13	KC194912
Araneae	Araneidae	*Argiope bruennichi*	GBCH10289-13	KC194911
Araneae	Araneidae	*Argiope bruennichi*	GBCH10290-13	KC194910
Araneae	Araneidae	*Argiope bruennichi*	GBCH10291-13	KC194909
Araneae	Araneidae	*Argiope bruennichi*	GBCH10292-13	KC194908
Araneae	Araneidae	*Argiope bruennichi*	GBCH10293-13	KC194907
Araneae	Araneidae	*Argiope bruennichi*	GBCH10294-13	KC194906
Araneae	Araneidae	*Argiope bruennichi*	GBCH10295-13	KC194905
Araneae	Araneidae	*Argiope bruennichi*	GBCH10296-13	KC194904
Araneae	Araneidae	*Argiope bruennichi*	GBCH10297-13	KC194903
Araneae	Araneidae	*Argiope bruennichi*	GBCH10298-13	KC194902
Araneae	Araneidae	*Argiope bruennichi*	GBCH10299-13	KC194901
Araneae	Araneidae	*Argiope bruennichi*	GBCH10300-13	KC194900
Araneae	Araneidae	*Argiope bruennichi*	GBCH10301-13	KC194899
Araneae	Araneidae	*Argiope bruennichi*	GBCH10302-13	KC194898
Araneae	Araneidae	*Argiope bruennichi*	GBCH10303-13	KC194897
Araneae	Araneidae	*Argiope bruennichi*	GBCH10304-13	KC194896
Araneae	Araneidae	*Argiope bruennichi*	GBCH10305-13	KC194895
Araneae	Araneidae	*Argiope bruennichi*	GBCH10306-13	KC194894
Araneae	Araneidae	*Argiope bruennichi*	GBCH10307-13	KC194893
Araneae	Araneidae	*Argiope bruennichi*	GBCH10308-13	KC194892
Araneae	Araneidae	*Argiope bruennichi*	GBCH10309-13	KC194891
Araneae	Araneidae	*Argiope bruennichi*	GBCH10310-13	KC194890
Araneae	Araneidae	*Argiope bruennichi*	GBCH10311-13	KC194889
Araneae	Araneidae	*Argiope bruennichi*	GBCH10312-13	KC194888

Araneae	Araneidae	*Argiope bruennichi*	GBCH10313-13	KC194887
Araneae	Araneidae	*Argiope bruennichi*	GBCH10314-13	KC194886
Araneae	Araneidae	*Argiope bruennichi*	GBCH10315-13	KC194885
Araneae	Araneidae	*Argiope bruennichi*	GBCH10316-13	KC194884
Araneae	Araneidae	*Argiope bruennichi*	GBCH10317-13	KC194883
Araneae	Araneidae	*Argiope bruennichi*	GBCH10318-13	KC194882
Araneae	Araneidae	*Argiope bruennichi*	GBCH10319-13	KC194881
Araneae	Araneidae	*Argiope bruennichi*	GBCH10320-13	KC194880
Araneae	Araneidae	*Argiope bruennichi*	GBCH10321-13	KC194879
Araneae	Araneidae	*Argiope bruennichi*	GBCH10322-13	KC194878
Araneae	Araneidae	*Argiope bruennichi*	GBCH10323-13	KC194877
Araneae	Araneidae	*Argiope bruennichi*	GBCH10324-13	KC194876
Araneae	Araneidae	*Argiope bruennichi*	GBCH10325-13	KC194875
Araneae	Araneidae	*Argiope bruennichi*	GBCH10326-13	KC194874
Araneae	Araneidae	*Argiope bruennichi*	GBCH10327-13	KC194873
Araneae	Araneidae	*Argiope bruennichi*	GBCH10328-13	KC194872
Araneae	Araneidae	*Argiope bruennichi*	GBCH10329-13	KC194871
Araneae	Araneidae	*Argiope bruennichi*	GBCH10330-13	KC194870
Araneae	Araneidae	*Argiope bruennichi*	GBCH1033-06	AY731171
Araneae	Araneidae	*Argiope bruennichi*	GBCH10331-13	KC194869
Araneae	Araneidae	*Argiope bruennichi*	GBCH10332-13	KC194868
Araneae	Araneidae	*Argiope bruennichi*	GBCH10333-13	KC194867
Araneae	Araneidae	*Argiope bruennichi*	GBCH10334-13	KC194866
Araneae	Araneidae	*Argiope bruennichi*	GBCH10335-13	KC194865
Araneae	Araneidae	*Argiope bruennichi*	GBCH10336-13	KC194864
Araneae	Araneidae	*Argiope bruennichi*	GBCH10337-13	KC194863
Araneae	Araneidae	*Argiope bruennichi*	GBCH10338-13	KC194862
Araneae	Araneidae	*Argiope bruennichi*	GBCH10339-13	KC194861
Araneae	Araneidae	*Argiope bruennichi*	GBCH10340-13	KC194860
Araneae	Araneidae	*Argiope bruennichi*	GBCH10341-13	KC194859
Araneae	Araneidae	*Argiope bruennichi*	GBCH10342-13	KC194858
Araneae	Araneidae	*Argiope bruennichi*	GBCH10343-13	KC194857
Araneae	Araneidae	*Argiope bruennichi*	GBCH10344-13	KC194856
Araneae	Araneidae	*Argiope bruennichi*	GBCH10345-13	KC194855
Araneae	Araneidae	*Argiope bruennichi*	GBCH10346-13	KC194854
Araneae	Araneidae	*Argiope bruennichi*	GBCH10347-13	KC194853
Araneae	Araneidae	*Argiope bruennichi*	GBCH10348-13	KC194852
Araneae	Araneidae	*Argiope bruennichi*	GBCH10349-13	KC194851
Araneae	Araneidae	*Argiope bruennichi*	GBCH10350-13	KC194850
Araneae	Araneidae	*Argiope bruennichi*	GBCH10351-13	KC194849
Araneae	Araneidae	*Argiope bruennichi*	GBCH10352-13	KC194848
Araneae	Araneidae	*Argiope bruennichi*	GBCH10353-13	KC194847
Araneae	Araneidae	*Argiope bruennichi*	GBCH10354-13	KC194846
Araneae	Araneidae	*Argiope bruennichi*	GBCH10355-13	KC194845
Araneae	Araneidae	*Argiope bruennichi*	GBCH10356-13	KC194844
Araneae	Araneidae	*Argiope bruennichi*	GBCH10357-13	KC194843
Araneae	Araneidae	*Argiope bruennichi*	GBCH10358-13	KC194842
Araneae	Araneidae	*Argiope bruennichi*	GBCH10359-13	KC194841
Araneae	Araneidae	*Argiope bruennichi*	GBCH10360-13	KC194840
Araneae	Araneidae	*Argiope bruennichi*	GBCH10361-13	KC194839
Araneae	Araneidae	*Argiope bruennichi*	GBCH10362-13	KC194838
Araneae	Araneidae	*Argiope bruennichi*	GBCH10363-13	KC194837
Araneae	Araneidae	*Argiope bruennichi*	GBCH10364-13	KC194836

Araneae	Araneidae	*Argiope bruennichi*	GBCH10365-13	KC194835
Araneae	Araneidae	*Argiope bruennichi*	GBCH10366-13	KC194834
Araneae	Araneidae	*Argiope bruennichi*	GBCH10367-13	KC194833
Araneae	Araneidae	*Argiope bruennichi*	GBCH10368-13	KC194832
Araneae	Araneidae	*Argiope bruennichi*	GBCH10369-13	KC194831
Araneae	Araneidae	*Argiope bruennichi*	GBCH10370-13	KC194830
Araneae	Araneidae	*Argiope bruennichi*	GBCH10371-13	KC194829
Araneae	Araneidae	*Argiope bruennichi*	GBCH10372-13	KC194828
Araneae	Araneidae	*Argiope bruennichi*	GBCH10373-13	KC194827
Araneae	Araneidae	*Argiope bruennichi*	GBCH10374-13	KC194826
Araneae	Araneidae	*Argiope bruennichi*	GBCH10375-13	KC194825
Araneae	Araneidae	*Argiope bruennichi*	GBCH10376-13	KC194824
Araneae	Araneidae	*Argiope bruennichi*	GBCH10377-13	KC194823
Araneae	Araneidae	*Argiope bruennichi*	GBCH10378-13	KC194822
Araneae	Araneidae	*Argiope bruennichi*	GBCH10379-13	KC194821
Araneae	Araneidae	*Argiope bruennichi*	GBCH10380-13	KC194820
Araneae	Araneidae	*Argiope bruennichi*	GBCH10381-13	KC194819
Araneae	Araneidae	*Argiope bruennichi*	GBCH10382-13	KC194758
Araneae	Araneidae	*Argiope bruennichi*	GBCH10383-13	KC194757
Araneae	Araneidae	*Argiope bruennichi*	GBCH10384-13	KC194756
Araneae	Araneidae	*Argiope bruennichi*	GBCH10385-13	KC194755
Araneae	Araneidae	*Argiope bruennichi*	GBCH10386-13	KC194754
Araneae	Araneidae	*Argiope bruennichi*	GBCH10387-13	KC194753
Araneae	Araneidae	*Argiope bruennichi*	GBCH10388-13	KC194752
Araneae	Araneidae	*Argiope bruennichi*	GBCH10389-13	KC194751
Araneae	Araneidae	*Argiope bruennichi*	GBCH10390-13	KC194750
Araneae	Araneidae	*Argiope bruennichi*	GBCH10391-13	KC194749
Araneae	Araneidae	*Argiope bruennichi*	GBCH10392-13	KC194748
Araneae	Araneidae	*Argiope bruennichi*	GBCH10393-13	KC194747
Araneae	Araneidae	*Argiope bruennichi*	GBCH10394-13	KC194746
Araneae	Araneidae	*Argiope bruennichi*	GBCH10395-13	KC194745
Araneae	Araneidae	*Argiope bruennichi*	GBCH10396-13	KC194744
Araneae	Araneidae	*Argiope bruennichi*	GBCH10397-13	KC194743
Araneae	Araneidae	*Argiope bruennichi*	GBCH10398-13	KC194742
Araneae	Araneidae	*Argiope bruennichi*	GBCH10399-13	KC194741
Araneae	Araneidae	*Argiope bruennichi*	GBCH10400-13	KC194740
Araneae	Araneidae	*Argiope bruennichi*	GBCH10401-13	KC194739
Araneae	Araneidae	*Argiope bruennichi*	GBCH10402-13	KC194738
Araneae	Araneidae	*Argiope bruennichi*	GBCH10403-13	KC194737
Araneae	Araneidae	*Argiope bruennichi*	GBCH10404-13	KC194736
Araneae	Araneidae	*Argiope bruennichi*	GBCH10405-13	KC194735
Araneae	Araneidae	*Argiope bruennichi*	GBCH10406-13	KC194734
Araneae	Araneidae	*Argiope bruennichi*	GBCH10407-13	KC194733
Araneae	Araneidae	*Argiope bruennichi*	GBCH10408-13	KC194732
Araneae	Araneidae	*Argiope bruennichi*	GBCH10409-13	KC194731
Araneae	Araneidae	*Argiope bruennichi*	GBCH10410-13	KC194730
Araneae	Araneidae	*Argiope bruennichi*	GBCH10411-13	KC194729
Araneae	Araneidae	*Argiope bruennichi*	GBCH10412-13	KC194728
Araneae	Araneidae	*Argiope bruennichi*	GBCH10413-13	KC194727
Araneae	Araneidae	*Argiope bruennichi*	GBCH10414-13	KC194726
Araneae	Araneidae	*Argiope bruennichi*	GBCH10415-13	KC194725
Araneae	Araneidae	*Argiope bruennichi*	GBCH10416-13	KC194724
Araneae	Araneidae	*Argiope bruennichi*	GBCH10417-13	KC194723

Araneae	Araneidae	*Argiope bruennichi*	GBCH10418-13	KC194722
Araneae	Araneidae	*Argiope bruennichi*	GBCH10419-13	KC194721
Araneae	Araneidae	*Argiope bruennichi*	GBCH10420-13	KC194720
Araneae	Araneidae	*Argiope bruennichi*	GBCH10421-13	KC194719
Araneae	Araneidae	*Argiope bruennichi*	GBCH10422-13	KC194718
Araneae	Araneidae	*Argiope bruennichi*	GBCH10423-13	KC194717
Araneae	Araneidae	*Argiope bruennichi*	GBCH10424-13	KC194716
Araneae	Araneidae	*Argiope bruennichi*	GBCH10425-13	KC194715
Araneae	Araneidae	*Argiope bruennichi*	GBCH10426-13	KC194714
Araneae	Araneidae	*Argiope bruennichi*	GBCH10427-13	KC194713
Araneae	Araneidae	*Argiope bruennichi*	GBCH10428-13	KC194712
Araneae	Araneidae	*Argiope bruennichi*	GBCH10429-13	KC194711
Araneae	Araneidae	*Argiope bruennichi*	GBCH10430-13	KC194710
Araneae	Araneidae	*Argiope bruennichi*	GBCH10431-13	KC194709
Araneae	Araneidae	*Argiope bruennichi*	GBCH10432-13	KC194708
Araneae	Araneidae	*Argiope bruennichi*	GBCH10433-13	KC194707
Araneae	Araneidae	*Argiope bruennichi*	GBCH10434-13	KC194706
Araneae	Araneidae	*Argiope bruennichi*	GBCH10435-13	KC194705
Araneae	Araneidae	*Argiope bruennichi*	GBCH10436-13	KC194704
Araneae	Araneidae	*Argiope bruennichi*	GBCH10437-13	KC194703
Araneae	Araneidae	*Argiope bruennichi*	GBCH10438-13	KC194702
Araneae	Araneidae	*Argiope bruennichi*	GBCH10439-13	KC194701
Araneae	Araneidae	*Argiope bruennichi*	GBCH10440-13	KC194700
Araneae	Araneidae	*Argiope bruennichi*	GBCH10441-13	KC194699
Araneae	Araneidae	*Argiope bruennichi*	GBCH10442-13	KC194698
Araneae	Araneidae	*Argiope bruennichi*	GBCH10443-13	KC194697
Araneae	Araneidae	*Argiope bruennichi*	GBCH10444-13	KC194696
Araneae	Araneidae	*Argiope bruennichi*	GBCH10445-13	KC194695
Araneae	Araneidae	*Argiope bruennichi*	GBCH10446-13	KC194694
Araneae	Araneidae	*Argiope bruennichi*	GBCH10447-13	KC194693
Araneae	Araneidae	*Argiope bruennichi*	GBCH10448-13	KC194692
Araneae	Araneidae	*Argiope bruennichi*	GBCH10449-13	KC194691
Araneae	Araneidae	*Argiope bruennichi*	GBCH10450-13	KC194690
Araneae	Araneidae	*Argiope bruennichi*	GBCH10451-13	KC194689
Araneae	Araneidae	*Argiope bruennichi*	GBCH10452-13	KC194688
Araneae	Araneidae	*Argiope bruennichi*	GBCH10453-13	KC194687
Araneae	Araneidae	*Argiope bruennichi*	GBCH10454-13	KC194686
Araneae	Araneidae	*Argiope bruennichi*	GBCH10455-13	KC194685
Araneae	Araneidae	*Argiope bruennichi*	GBCH10456-13	KC194684
Araneae	Araneidae	*Argiope bruennichi*	GBCH10457-13	KC194683
Araneae	Araneidae	*Argiope bruennichi*	GBCH10458-13	KC194682
Araneae	Araneidae	*Argiope bruennichi*	GBCH10459-13	KC194681
Araneae	Araneidae	*Argiope bruennichi*	GBCH10460-13	KC194680
Araneae	Araneidae	*Argiope bruennichi*	GBCH10461-13	KC194679
Araneae	Araneidae	*Argiope bruennichi*	GBCH10462-13	KC194678
Araneae	Araneidae	*Argiope bruennichi*	GBCH10463-13	KC194677
Araneae	Araneidae	*Argiope bruennichi*	GBCH10464-13	KC194676
Araneae	Araneidae	*Argiope bruennichi*	GBCH10465-13	KC194675
Araneae	Araneidae	*Argiope bruennichi*	GBCH10466-13	KC194674
Araneae	Araneidae	*Argiope bruennichi*	GBCH10467-13	KC194673
Araneae	Araneidae	*Argiope bruennichi*	GBCH10468-13	KC194672
Araneae	Araneidae	*Argiope bruennichi*	GBCH10469-13	KC194671
Araneae	Araneidae	*Argiope bruennichi*	GBCH10470-13	KC194670

Araneae	Araneidae	*Argiope bruennichi*	GBCH10471-13	KC194669
Araneae	Araneidae	*Argiope bruennichi*	GBCH10472-13	KC194668
Araneae	Araneidae	*Argiope bruennichi*	GBCH10473-13	KC194667
Araneae	Araneidae	*Argiope bruennichi*	GBCH10474-13	KC194666
Araneae	Araneidae	*Argiope bruennichi*	GBCH10475-13	KC194665
Araneae	Araneidae	*Argiope bruennichi*	GBCH10476-13	KC194664
Araneae	Araneidae	*Argiope bruennichi*	GBCH10477-13	KC194663
Araneae	Araneidae	*Argiope bruennichi*	GBCH10478-13	KC194662
Araneae	Araneidae	*Argiope bruennichi*	GBCH10479-13	KC194661
Araneae	Araneidae	*Argiope bruennichi*	GBCH10480-13	KC194660
Araneae	Araneidae	*Argiope bruennichi*	GBCH10481-13	KC194659
Araneae	Araneidae	*Argiope bruennichi*	GBCH10482-13	KC194658
Araneae	Araneidae	*Argiope bruennichi*	GBCH10483-13	KC194657
Araneae	Araneidae	*Argiope bruennichi*	GBCH10484-13	KC194656
Araneae	Araneidae	*Argiope bruennichi*	GBCH10485-13	KC194655
Araneae	Araneidae	*Argiope bruennichi*	GBCH10486-13	KC194654
Araneae	Araneidae	*Argiope bruennichi*	GBCH10487-13	KC194653
Araneae	Araneidae	*Argiope bruennichi*	GBCH10488-13	KC194652
Araneae	Araneidae	*Argiope bruennichi*	GBCH10489-13	KC194651
Araneae	Araneidae	*Argiope bruennichi*	GBCH10490-13	KC194650
Araneae	Araneidae	*Argiope bruennichi*	GBCH10491-13	KC194649
Araneae	Araneidae	*Argiope bruennichi*	GBCH10492-13	KC194648
Araneae	Araneidae	*Argiope bruennichi*	GBCH10493-13	KC194647
Araneae	Araneidae	*Argiope bruennichi*	GBCH10494-13	KC194646
Araneae	Araneidae	*Argiope bruennichi*	GBCH10495-13	KC194645
Araneae	Araneidae	*Argiope bruennichi*	GBCH10496-13	KC194644
Araneae	Araneidae	*Argiope bruennichi*	GBCH10497-13	KC194643
Araneae	Araneidae	*Argiope bruennichi*	GBCH10498-13	KC194642
Araneae	Araneidae	*Argiope bruennichi*	GBCH10499-13	KC194641
Araneae	Araneidae	*Argiope bruennichi*	GBCH10500-13	KC194640
Araneae	Araneidae	*Argiope bruennichi*	GBCH10501-13	KC194639
Araneae	Araneidae	*Argiope bruennichi*	GBCH10502-13	KC194638
Araneae	Araneidae	*Argiope bruennichi*	GBCH10503-13	KC194637
Araneae	Araneidae	*Argiope bruennichi*	GBCH10504-13	KC194636
Araneae	Araneidae	*Argiope bruennichi*	GBCH10505-13	KC194635
Araneae	Araneidae	*Argiope bruennichi*	GBCH10506-13	KC194634
Araneae	Araneidae	*Argiope bruennichi*	GBCH10507-13	KC194633
Araneae	Araneidae	*Argiope bruennichi*	GBCH10508-13	KC194632
Araneae	Araneidae	*Argiope bruennichi*	GBCH10509-13	KC194631
Araneae	Araneidae	*Argiope bruennichi*	GBCH10510-13	KC194630
Araneae	Araneidae	*Argiope bruennichi*	GBCH10511-13	KC194629
Araneae	Araneidae	*Argiope bruennichi*	GBCH10512-13	KC194628
Araneae	Araneidae	*Argiope bruennichi*	GBCH10513-13	KC194627
Araneae	Araneidae	*Argiope bruennichi*	GBCH10514-13	KC194626
Araneae	Araneidae	*Argiope bruennichi*	GBCH10515-13	KC194625
Araneae	Araneidae	*Argiope bruennichi*	GBCH10516-13	KC194624
Araneae	Araneidae	*Argiope bruennichi*	GBCH10517-13	KC194623
Araneae	Araneidae	*Argiope bruennichi*	GBCH10518-13	KC194622
Araneae	Araneidae	*Argiope bruennichi*	GBCH10519-13	KC194621
Araneae	Araneidae	*Argiope bruennichi*	GBCH10520-13	KC194620
Araneae	Araneidae	*Argiope bruennichi*	GBCH10521-13	KC194619
Araneae	Araneidae	*Argiope bruennichi*	GBCH10522-13	KC194618
Araneae	Araneidae	*Argiope bruennichi*	GBCH10523-13	KC194617

Araneae	Araneidae	*Argiope bruennichi*	GBCH10524-13	KC194616
Araneae	Araneidae	*Argiope bruennichi*	GBCH10525-13	KC194615
Araneae	Araneidae	*Argiope bruennichi*	GBCH10526-13	KC194614
Araneae	Araneidae	*Argiope bruennichi*	GBCH10527-13	KC194613
Araneae	Araneidae	*Argiope bruennichi*	GBCH10528-13	KC194612
Araneae	Araneidae	*Argiope bruennichi*	GBCH10529-13	KC194611
Araneae	Araneidae	*Argiope bruennichi*	GBCH10530-13	KC194610
Araneae	Araneidae	*Argiope bruennichi*	GBCH10531-13	KC194609
Araneae	Araneidae	*Argiope bruennichi*	GBCH10532-13	KC194608
Araneae	Araneidae	*Argiope bruennichi*	GBCH10533-13	KC194607
Araneae	Araneidae	*Argiope bruennichi*	GBCH10534-13	KC194606
Araneae	Araneidae	*Argiope bruennichi*	GBCH10535-13	KC194605
Araneae	Araneidae	*Argiope bruennichi*	GBCH10536-13	KC194604
Araneae	Araneidae	*Argiope bruennichi*	GBCH10537-13	KC194603
Araneae	Araneidae	*Argiope bruennichi*	GBCH10538-13	KC194602
Araneae	Araneidae	*Argiope bruennichi*	GBCH10539-13	KC194601
Araneae	Araneidae	*Argiope bruennichi*	GBCH10540-13	KC194600
Araneae	Araneidae	*Argiope bruennichi*	GBCH10541-13	KC194599
Araneae	Araneidae	*Argiope bruennichi*	GBCH10542-13	KC194598
Araneae	Araneidae	*Argiope bruennichi*	GBCH10543-13	KC194597
Araneae	Araneidae	*Argiope bruennichi*	GBCH10544-13	KC194596
Araneae	Araneidae	*Argiope bruennichi*	GBCH10545-13	KC194595
Araneae	Araneidae	*Argiope bruennichi*	GBCH10546-13	KC194594
Araneae	Araneidae	*Argiope bruennichi*	GBCH10547-13	KC194593
Araneae	Araneidae	*Argiope bruennichi*	GBCH10548-13	KC194592
Araneae	Araneidae	*Argiope bruennichi*	GBCH10549-13	KC194591
Araneae	Araneidae	*Argiope bruennichi*	GBCH10550-13	KC194590
Araneae	Araneidae	*Argiope bruennichi*	GBCH10551-13	KC194589
Araneae	Araneidae	*Argiope bruennichi*	GBCH10552-13	KC194588
Araneae	Araneidae	*Argiope bruennichi*	GBCH10553-13	KC194587
Araneae	Araneidae	*Argiope bruennichi*	GBCH10554-13	KC194586
Araneae	Araneidae	*Argiope bruennichi*	GBCH10555-13	KC194585
Araneae	Araneidae	*Argiope bruennichi*	GBCH10556-13	KC194584
Araneae	Araneidae	*Argiope bruennichi*	GBCH10557-13	KC194583
Araneae	Araneidae	*Argiope bruennichi*	GBCH10558-13	KC194582
Araneae	Araneidae	*Argiope bruennichi*	GBCH10559-13	KC194581
Araneae	Araneidae	*Argiope bruennichi*	GBCH10560-13	KC194580
Araneae	Araneidae	*Argiope bruennichi*	GBCH10561-13	KC194579
Araneae	Araneidae	*Argiope bruennichi*	GBCH10562-13	KC194578
Araneae	Araneidae	*Argiope bruennichi*	GBCH10563-13	KC194577
Araneae	Araneidae	*Argiope bruennichi*	GBCH10564-13	KC194576
Araneae	Araneidae	*Argiope bruennichi*	GBCH10565-13	KC194575
Araneae	Araneidae	*Argiope bruennichi*	GBCH10566-13	KC194574
Araneae	Araneidae	*Argiope bruennichi*	GBCH10567-13	KC194573
Araneae	Araneidae	*Argiope bruennichi*	GBCH10568-13	KC194572
Araneae	Araneidae	*Argiope bruennichi*	GBCH10569-13	KC194571
Araneae	Araneidae	*Argiope bruennichi*	GBCH10570-13	KC194570
Araneae	Araneidae	*Argiope bruennichi*	GBCH10571-13	KC194569
Araneae	Araneidae	*Argiope bruennichi*	GBCH10572-13	KC194568
Araneae	Araneidae	*Argiope bruennichi*	GBCH10573-13	KC194567
Araneae	Araneidae	*Argiope bruennichi*	GBCH10574-13	KC194566
Araneae	Araneidae	*Argiope bruennichi*	GBCH10575-13	KC194565
Araneae	Araneidae	*Argiope bruennichi*	GBCH10576-13	KC194564

Araneae	Araneidae	*Argiope bruennichi*	GBCH10577-13	KC194563
Araneae	Araneidae	*Argiope bruennichi*	GBCH10578-13	KC194562
Araneae	Araneidae	*Argiope bruennichi*	GBCH10579-13	KC194561
Araneae	Araneidae	*Argiope bruennichi*	GBCH10580-13	KC194560
Araneae	Araneidae	*Argiope bruennichi*	GBCH10581-13	KC194559
Araneae	Araneidae	*Argiope bruennichi*	GBCH10582-13	KC194558
Araneae	Araneidae	*Argiope bruennichi*	GBCH10583-13	KC194557
Araneae	Araneidae	*Argiope bruennichi*	GBCH10584-13	KC194556
Araneae	Araneidae	*Argiope bruennichi*	GBCH10585-13	KC194555
Araneae	Araneidae	*Argiope bruennichi*	GBCH10586-13	KC194554
Araneae	Araneidae	*Argiope bruennichi*	GBCH10587-13	KC194553
Araneae	Araneidae	*Argiope bruennichi*	GBCH10588-13	KC194552
Araneae	Araneidae	*Argiope bruennichi*	GBCH10589-13	KC194551
Araneae	Araneidae	*Argiope bruennichi*	GBCH10590-13	KC194550
Araneae	Araneidae	*Argiope bruennichi*	GBCH10591-13	KC194549
Araneae	Araneidae	*Argiope bruennichi*	GBCH10592-13	KC194548
Araneae	Araneidae	*Argiope bruennichi*	GBCH10593-13	KC194547
Araneae	Araneidae	*Argiope bruennichi*	GBCH10594-13	KC194546
Araneae	Araneidae	*Argiope bruennichi*	GBCH10595-13	KC194545
Araneae	Araneidae	*Argiope bruennichi*	GBCH10596-13	KC194544
Araneae	Araneidae	*Argiope bruennichi*	GBCH10597-13	KC194543
Araneae	Araneidae	*Argiope bruennichi*	GBCH10598-13	KC194542
Araneae	Araneidae	*Argiope bruennichi*	GBCH10599-13	KC194541
Araneae	Araneidae	*Argiope bruennichi*	GBCH10600-13	KC194540
Araneae	Araneidae	*Argiope bruennichi*	GBCH10601-13	KC194539
Araneae	Araneidae	*Argiope bruennichi*	GBCH10602-13	KC194538
Araneae	Araneidae	*Argiope bruennichi*	GBCH10603-13	KC194537
Araneae	Araneidae	*Argiope bruennichi*	GBCH10604-13	KC194536
Araneae	Araneidae	*Argiope bruennichi*	GBCH10605-13	KC194535
Araneae	Araneidae	*Argiope bruennichi*	GBCH10606-13	KC194534
Araneae	Araneidae	*Argiope bruennichi*	GBCH10607-13	KC194533
Araneae	Araneidae	*Argiope bruennichi*	GBCH10608-13	KC194532
Araneae	Araneidae	*Argiope bruennichi*	GBCH10609-13	KC194531
Araneae	Araneidae	*Argiope bruennichi*	GBCH10610-13	KC194530
Araneae	Araneidae	*Argiope bruennichi*	GBCH10611-13	KC194529
Araneae	Araneidae	*Argiope bruennichi*	GBCH10612-13	KC194528
Araneae	Araneidae	*Argiope bruennichi*	GBCH10613-13	KC194527
Araneae	Araneidae	*Argiope bruennichi*	GBCH10614-13	KC194526
Araneae	Araneidae	*Argiope bruennichi*	GBCH10615-13	KC194525
Araneae	Araneidae	*Argiope bruennichi*	GBCH10616-13	KC194524
Araneae	Araneidae	*Argiope bruennichi*	GBCH10617-13	KC194523
Araneae	Araneidae	*Argiope bruennichi*	GBCH10618-13	KC194522
Araneae	Araneidae	*Argiope bruennichi*	GBCH10619-13	KC194521
Araneae	Araneidae	*Argiope bruennichi*	GBCH10620-13	KC194520
Araneae	Araneidae	*Argiope bruennichi*	GBCH10621-13	KC194519
Araneae	Araneidae	*Argiope bruennichi*	GBCH10622-13	KC194418
Araneae	Araneidae	*Argiope bruennichi*	GBCH10623-13	KC194417
Araneae	Araneidae	*Argiope bruennichi*	GBCH10624-13	KC194416
Araneae	Araneidae	*Argiope bruennichi*	GBCH10625-13	KC194415
Araneae	Araneidae	*Argiope bruennichi*	GBCH10626-13	KC194414
Araneae	Araneidae	*Argiope bruennichi*	GBCH10627-13	KC194413
Araneae	Araneidae	*Argiope bruennichi*	GBCH10628-13	KC194412
Araneae	Araneidae	*Argiope bruennichi*	GBCH10629-13	KC194411

Araneae	Araneidae	*Argiope bruennichi*	GBCH10630-13	KC194410
Araneae	Araneidae	*Argiope bruennichi*	GBCH10631-13	KC194409
Araneae	Araneidae	*Argiope bruennichi*	GBCH10632-13	KC194408
Araneae	Araneidae	*Argiope bruennichi*	GBCH10633-13	KC194407
Araneae	Araneidae	*Argiope bruennichi*	GBCH10634-13	KC194406
Araneae	Araneidae	*Argiope bruennichi*	GBCH10635-13	KC194405
Araneae	Araneidae	*Argiope bruennichi*	GBCH10636-13	KC194404
Araneae	Araneidae	*Argiope bruennichi*	GBCH10637-13	KC194403
Araneae	Araneidae	*Argiope bruennichi*	GBCH10638-13	KC194402
Araneae	Araneidae	*Argiope bruennichi*	GBCH10639-13	KC194401
Araneae	Araneidae	*Argiope bruennichi*	GBCH10640-13	KC194400
Araneae	Araneidae	*Argiope bruennichi*	GBCH10641-13	KC194399
Araneae	Araneidae	*Argiope bruennichi*	GBCH10642-13	KC194398
Araneae	Araneidae	*Argiope bruennichi*	GBCH10643-13	KC194397
Araneae	Araneidae	*Argiope bruennichi*	GBCH10644-13	KC194396
Araneae	Araneidae	*Argiope bruennichi*	GBCH10645-13	KC194395
Araneae	Araneidae	*Argiope bruennichi*	GBCH10646-13	KC194394
Araneae	Araneidae	*Argiope bruennichi*	GBCH10647-13	KC194393
Araneae	Araneidae	*Argiope bruennichi*	GBCH10648-13	KC194392
Araneae	Araneidae	*Argiope bruennichi*	GBCH10649-13	KC194391
Araneae	Araneidae	*Argiope bruennichi*	GBCH10650-13	KC194390
Araneae	Araneidae	*Argiope bruennichi*	GBCH10651-13	KC194389
Araneae	Araneidae	*Argiope bruennichi*	GBCH10652-13	KC194388
Araneae	Araneidae	*Argiope bruennichi*	GBCH10653-13	KC194387
Araneae	Araneidae	*Argiope bruennichi*	GBCH10654-13	KC194386
Araneae	Araneidae	*Argiope bruennichi*	GBCH10655-13	KC194385
Araneae	Araneidae	*Argiope bruennichi*	GBCH10656-13	KC194384
Araneae	Araneidae	*Argiope bruennichi*	GBCH10657-13	KC194383
Araneae	Araneidae	*Argiope bruennichi*	GBCH10658-13	KC194382
Araneae	Araneidae	*Argiope bruennichi*	GBCH10659-13	KC194381
Araneae	Araneidae	*Argiope bruennichi*	GBCH10660-13	KC194380
Araneae	Araneidae	*Argiope bruennichi*	GBCH10661-13	KC194379
Araneae	Araneidae	*Argiope bruennichi*	GBCH10662-13	KC194378
Araneae	Araneidae	*Argiope bruennichi*	GBCH10663-13	KC194377
Araneae	Araneidae	*Argiope bruennichi*	GBCH10664-13	KC194376
Araneae	Araneidae	*Argiope bruennichi*	GBCH10665-13	KC194375
Araneae	Araneidae	*Argiope bruennichi*	GBCH10666-13	KC194374
Araneae	Araneidae	*Argiope bruennichi*	GBCH10667-13	KC194373
Araneae	Araneidae	*Argiope bruennichi*	GBCH10668-13	KC194372
Araneae	Araneidae	*Argiope bruennichi*	GBCH10669-13	KC194371
Araneae	Araneidae	*Argiope bruennichi*	GBCH10670-13	KC194370
Araneae	Araneidae	*Argiope bruennichi*	GBCH10671-13	KC194369
Araneae	Araneidae	*Argiope bruennichi*	GBCH10672-13	KC194368
Araneae	Araneidae	*Argiope bruennichi*	GBCH10673-13	KC194367
Araneae	Araneidae	*Argiope bruennichi*	GBCH10674-13	KC194366
Araneae	Araneidae	*Argiope bruennichi*	GBCH10675-13	KC194365
Araneae	Araneidae	*Argiope bruennichi*	GBCH10676-13	KC194364
Araneae	Araneidae	*Argiope bruennichi*	GBCH10677-13	KC194363
Araneae	Araneidae	*Argiope bruennichi*	GBCH10678-13	KC194362
Araneae	Araneidae	*Argiope bruennichi*	GBCH10679-13	KC194361
Araneae	Araneidae	*Argiope bruennichi*	GBCH10680-13	KC194360
Araneae	Araneidae	*Argiope bruennichi*	GBCH10681-13	KC194359
Araneae	Araneidae	*Argiope bruennichi*	GBCH10682-13	KC194358

Araneae	Araneidae	*Argiope bruennichi*	GBCH10683-13	KC194357
Araneae	Araneidae	*Argiope bruennichi*	GBCH10684-13	KC194356
Araneae	Araneidae	*Argiope bruennichi*	GBCH10685-13	KC194355
Araneae	Araneidae	*Argiope bruennichi*	GBCH10686-13	KC194354
Araneae	Araneidae	*Argiope bruennichi*	GBCH10687-13	KC194353
Araneae	Araneidae	*Argiope bruennichi*	GBCH10688-13	KC194352
Araneae	Araneidae	*Argiope bruennichi*	GBCH10689-13	KC194351
Araneae	Araneidae	*Argiope bruennichi*	GBCH10690-13	KC194350
Araneae	Araneidae	*Argiope bruennichi*	GBCH10691-13	KC194349
Araneae	Araneidae	*Argiope bruennichi*	GBCH10692-13	KC194348
Araneae	Araneidae	*Argiope bruennichi*	GBCH10693-13	KC194347
Araneae	Araneidae	*Argiope bruennichi*	GBCH10694-13	KC194346
Araneae	Araneidae	*Argiope bruennichi*	GBCH10695-13	KC194345
Araneae	Araneidae	*Argiope bruennichi*	GBCH10696-13	KC194344
Araneae	Araneidae	*Argiope bruennichi*	GBCH10697-13	KC194343
Araneae	Araneidae	*Argiope bruennichi*	GBCH10698-13	KC194342
Araneae	Araneidae	*Argiope bruennichi*	GBCH10699-13	KC194341
Araneae	Araneidae	*Argiope bruennichi*	GBCH10700-13	KC194340
Araneae	Araneidae	*Argiope bruennichi*	GBCH10701-13	KC194339
Araneae	Araneidae	*Argiope bruennichi*	GBCH10702-13	KC194338
Araneae	Araneidae	*Argiope bruennichi*	GBCH10703-13	KC194337
Araneae	Araneidae	*Argiope bruennichi*	GBCH10704-13	KC194336
Araneae	Araneidae	*Argiope bruennichi*	GBCH10705-13	KC194335
Araneae	Araneidae	*Argiope bruennichi*	GBCH10706-13	KC194334
Araneae	Araneidae	*Argiope bruennichi*	GBCH10707-13	KC194333
Araneae	Araneidae	*Argiope bruennichi*	GBCH10708-13	KC194332
Araneae	Araneidae	*Argiope bruennichi*	GBCH10709-13	KC194331
Araneae	Araneidae	*Argiope bruennichi*	GBCH10710-13	KC194330
Araneae	Araneidae	*Argiope bruennichi*	GBCH10711-13	KC194329
Araneae	Araneidae	*Argiope bruennichi*	GBCH10712-13	KC194328
Araneae	Araneidae	*Argiope bruennichi*	GBCH10713-13	KC194327
Araneae	Araneidae	*Argiope bruennichi*	GBCH10714-13	KC194326
Araneae	Araneidae	*Argiope bruennichi*	GBCH10715-13	KC194325
Araneae	Araneidae	*Argiope bruennichi*	GBCH10716-13	KC194324
Araneae	Araneidae	*Argiope bruennichi*	GBCH10717-13	KC194323
Araneae	Araneidae	*Argiope bruennichi*	GBCH10718-13	KC194322
Araneae	Araneidae	*Argiope bruennichi*	GBCH10719-13	KC194321
Araneae	Araneidae	*Argiope bruennichi*	GBCH10720-13	KC194320
Araneae	Araneidae	*Argiope bruennichi*	GBCH10721-13	KC194319
Araneae	Araneidae	*Argiope bruennichi*	GBCH11109-13	JN817161
Araneae	Araneidae	*Argiope bruennichi*	GBCH11110-13	JN817160
Araneae	Araneidae	*Argiope bruennichi*	GBCH8590-13	KC195752
Araneae	Araneidae	*Argiope bruennichi*	GBCH8591-13	KC195751
Araneae	Araneidae	*Argiope bruennichi*	GBCH8592-13	KC195750
Araneae	Araneidae	*Argiope bruennichi*	GBCH8593-13	KC195749
Araneae	Araneidae	*Argiope bruennichi*	GBCH8594-13	KC195748
Araneae	Araneidae	*Argiope bruennichi*	GBCH8595-13	KC195747
Araneae	Araneidae	*Argiope bruennichi*	GBCH8596-13	KC195746
Araneae	Araneidae	*Argiope bruennichi*	GBCH8597-13	KC195745
Araneae	Araneidae	*Argiope bruennichi*	GBCH8598-13	KC195744
Araneae	Araneidae	*Argiope bruennichi*	GBCH8599-13	KC195743
Araneae	Araneidae	*Argiope bruennichi*	GBCH8600-13	KC195742
Araneae	Araneidae	*Argiope bruennichi*	GBCH8601-13	KC195741

Araneae	Araneidae	*Argiope bruennichi*	GBCH8602-13	KC195740
Araneae	Araneidae	*Argiope bruennichi*	GBCH8603-13	KC195739
Araneae	Araneidae	*Argiope bruennichi*	GBCH8604-13	KC195738
Araneae	Araneidae	*Argiope bruennichi*	GBCH8605-13	KC195737
Araneae	Araneidae	*Argiope bruennichi*	GBCH8606-13	KC195736
Araneae	Araneidae	*Argiope bruennichi*	GBCH8607-13	KC195735
Araneae	Araneidae	*Argiope bruennichi*	GBCH8608-13	KC195734
Araneae	Araneidae	*Argiope bruennichi*	GBCH8609-13	KC195733
Araneae	Araneidae	*Argiope bruennichi*	GBCH8610-13	KC195732
Araneae	Araneidae	*Argiope bruennichi*	GBCH8611-13	KC195731
Araneae	Araneidae	*Argiope bruennichi*	GBCH8612-13	KC195730
Araneae	Araneidae	*Argiope bruennichi*	GBCH8613-13	KC195729
Araneae	Araneidae	*Argiope bruennichi*	GBCH8614-13	KC195728
Araneae	Araneidae	*Argiope bruennichi*	GBCH8615-13	KC195727
Araneae	Araneidae	*Argiope bruennichi*	GBCH8616-13	KC195726
Araneae	Araneidae	*Argiope bruennichi*	GBCH8617-13	KC195725
Araneae	Araneidae	*Argiope bruennichi*	GBCH8618-13	KC195724
Araneae	Araneidae	*Argiope bruennichi*	GBCH8619-13	KC195723
Araneae	Araneidae	*Argiope bruennichi*	GBCH8620-13	KC195722
Araneae	Araneidae	*Argiope bruennichi*	GBCH8621-13	KC195721
Araneae	Araneidae	*Argiope bruennichi*	GBCH8622-13	KC195720
Araneae	Araneidae	*Argiope bruennichi*	GBCH8623-13	KC195719
Araneae	Araneidae	*Argiope bruennichi*	GBCH8624-13	KC195718
Araneae	Araneidae	*Argiope bruennichi*	GBCH8625-13	KC195717
Araneae	Araneidae	*Argiope bruennichi*	GBCH8626-13	KC195716
Araneae	Araneidae	*Argiope bruennichi*	GBCH8627-13	KC195715
Araneae	Araneidae	*Argiope bruennichi*	GBCH8628-13	KC195714
Araneae	Araneidae	*Argiope bruennichi*	GBCH8629-13	KC195713
Araneae	Araneidae	*Argiope bruennichi*	GBCH8630-13	KC195712
Araneae	Araneidae	*Argiope bruennichi*	GBCH8631-13	KC195711
Araneae	Araneidae	*Argiope bruennichi*	GBCH8632-13	KC195710
Araneae	Araneidae	*Argiope bruennichi*	GBCH8633-13	KC195709
Araneae	Araneidae	*Argiope bruennichi*	GBCH8634-13	KC195708
Araneae	Araneidae	*Argiope bruennichi*	GBCH8635-13	KC195707
Araneae	Araneidae	*Argiope bruennichi*	GBCH8636-13	KC195706
Araneae	Araneidae	*Argiope bruennichi*	GBCH8637-13	KC195705
Araneae	Araneidae	*Argiope bruennichi*	GBCH8638-13	KC195704
Araneae	Araneidae	*Argiope bruennichi*	GBCH8639-13	KC195703
Araneae	Araneidae	*Argiope bruennichi*	GBCH8640-13	KC195702
Araneae	Araneidae	*Argiope bruennichi*	GBCH8641-13	KC195701
Araneae	Araneidae	*Argiope bruennichi*	GBCH8642-13	KC195700
Araneae	Araneidae	*Argiope bruennichi*	GBCH8643-13	KC195699
Araneae	Araneidae	*Argiope bruennichi*	GBCH8644-13	KC195698
Araneae	Araneidae	*Argiope bruennichi*	GBCH8645-13	KC195697
Araneae	Araneidae	*Argiope bruennichi*	GBCH8646-13	KC195696
Araneae	Araneidae	*Argiope bruennichi*	GBCH8647-13	KC195695
Araneae	Araneidae	*Argiope bruennichi*	GBCH8648-13	KC195694
Araneae	Araneidae	*Argiope bruennichi*	GBCH8649-13	KC195693
Araneae	Araneidae	*Argiope bruennichi*	GBCH8650-13	KC195692
Araneae	Araneidae	*Argiope bruennichi*	GBCH8651-13	KC195691
Araneae	Araneidae	*Argiope bruennichi*	GBCH8652-13	KC195690
Araneae	Araneidae	*Argiope bruennichi*	GBCH8653-13	KC195689
Araneae	Araneidae	*Argiope bruennichi*	GBCH8654-13	KC195688

Araneae	Araneidae	*Argiope bruennichi*	GBCH8655-13	KC195687
Araneae	Araneidae	*Argiope bruennichi*	GBCH8656-13	KC195686
Araneae	Araneidae	*Argiope bruennichi*	GBCH8657-13	KC195685
Araneae	Araneidae	*Argiope bruennichi*	GBCH8658-13	KC195684
Araneae	Araneidae	*Argiope bruennichi*	GBCH8659-13	KC195683
Araneae	Araneidae	*Argiope bruennichi*	GBCH8660-13	KC195682
Araneae	Araneidae	*Argiope bruennichi*	GBCH8661-13	KC195681
Araneae	Araneidae	*Argiope bruennichi*	GBCH8662-13	KC195680
Araneae	Araneidae	*Argiope bruennichi*	GBCH8663-13	KC195679
Araneae	Araneidae	*Argiope bruennichi*	GBCH8664-13	KC195678
Araneae	Araneidae	*Argiope bruennichi*	GBCH8665-13	KC195677
Araneae	Araneidae	*Argiope bruennichi*	GBCH8666-13	KC195676
Araneae	Araneidae	*Argiope bruennichi*	GBCH8667-13	KC195675
Araneae	Araneidae	*Argiope bruennichi*	GBCH8668-13	KC195674
Araneae	Araneidae	*Argiope bruennichi*	GBCH8669-13	KC195673
Araneae	Araneidae	*Argiope bruennichi*	GBCH8670-13	KC195672
Araneae	Araneidae	*Argiope bruennichi*	GBCH8671-13	KC195671
Araneae	Araneidae	*Argiope bruennichi*	GBCH8672-13	KC195670
Araneae	Araneidae	*Argiope bruennichi*	GBCH8673-13	KC195669
Araneae	Araneidae	*Argiope bruennichi*	GBCH8674-13	KC195668
Araneae	Araneidae	*Argiope bruennichi*	GBCH8675-13	KC195667
Araneae	Araneidae	*Argiope bruennichi*	GBCH8676-13	KC195666
Araneae	Araneidae	*Argiope bruennichi*	GBCH8677-13	KC195665
Araneae	Araneidae	*Argiope bruennichi*	GBCH8678-13	KC195664
Araneae	Araneidae	*Argiope bruennichi*	GBCH8679-13	KC195663
Araneae	Araneidae	*Argiope bruennichi*	GBCH8680-13	KC195662
Araneae	Araneidae	*Argiope bruennichi*	GBCH8681-13	KC195661
Araneae	Araneidae	*Argiope bruennichi*	GBCH8682-13	KC195660
Araneae	Araneidae	*Argiope bruennichi*	GBCH8683-13	KC195659
Araneae	Araneidae	*Argiope bruennichi*	GBCH8684-13	KC195658
Araneae	Araneidae	*Argiope bruennichi*	GBCH8685-13	KC195657
Araneae	Araneidae	*Argiope bruennichi*	GBCH8686-13	KC195656
Araneae	Araneidae	*Argiope bruennichi*	GBCH8687-13	KC195655
Araneae	Araneidae	*Argiope bruennichi*	GBCH8688-13	KC195654
Araneae	Araneidae	*Argiope bruennichi*	GBCH8689-13	KC195653
Araneae	Araneidae	*Argiope bruennichi*	GBCH8690-13	KC195652
Araneae	Araneidae	*Argiope bruennichi*	GBCH8691-13	KC195651
Araneae	Araneidae	*Argiope bruennichi*	GBCH8692-13	KC195650
Araneae	Araneidae	*Argiope bruennichi*	GBCH8693-13	KC195649
Araneae	Araneidae	*Argiope bruennichi*	GBCH8694-13	KC195648
Araneae	Araneidae	*Argiope bruennichi*	GBCH8695-13	KC195647
Araneae	Araneidae	*Argiope bruennichi*	GBCH8696-13	KC195646
Araneae	Araneidae	*Argiope bruennichi*	GBCH8697-13	KC195645
Araneae	Araneidae	*Argiope bruennichi*	GBCH8698-13	KC195644
Araneae	Araneidae	*Argiope bruennichi*	GBCH8699-13	KC195643
Araneae	Araneidae	*Argiope bruennichi*	GBCH8700-13	KC195642
Araneae	Araneidae	*Argiope bruennichi*	GBCH8701-13	KC195641
Araneae	Araneidae	*Argiope bruennichi*	GBCH8702-13	KC195640
Araneae	Araneidae	*Argiope bruennichi*	GBCH8703-13	KC195639
Araneae	Araneidae	*Argiope bruennichi*	GBCH8704-13	KC195638
Araneae	Araneidae	*Argiope bruennichi*	GBCH8705-13	KC195637
Araneae	Araneidae	*Argiope bruennichi*	GBCH8706-13	KC195636
Araneae	Araneidae	*Argiope bruennichi*	GBCH8707-13	KC195635

Araneae	Araneidae	*Argiope bruennichi*	GBCH8708-13	KC195634
Araneae	Araneidae	*Argiope bruennichi*	GBCH8709-13	KC195633
Araneae	Araneidae	*Argiope bruennichi*	GBCH8710-13	KC195632
Araneae	Araneidae	*Argiope bruennichi*	GBCH8711-13	KC195631
Araneae	Araneidae	*Argiope bruennichi*	GBCH8712-13	KC195630
Araneae	Araneidae	*Argiope bruennichi*	GBCH8713-13	KC195629
Araneae	Araneidae	*Argiope bruennichi*	GBCH8714-13	KC195628
Araneae	Araneidae	*Argiope bruennichi*	GBCH8715-13	KC195627
Araneae	Araneidae	*Argiope bruennichi*	GBCH8716-13	KC195626
Araneae	Araneidae	*Argiope bruennichi*	GBCH8717-13	KC195625
Araneae	Araneidae	*Argiope bruennichi*	GBCH8718-13	KC195624
Araneae	Araneidae	*Argiope bruennichi*	GBCH8719-13	KC195623
Araneae	Araneidae	*Argiope bruennichi*	GBCH8720-13	KC195622
Araneae	Araneidae	*Argiope bruennichi*	GBCH8721-13	KC195621
Araneae	Araneidae	*Argiope bruennichi*	GBCH8722-13	KC195620
Araneae	Araneidae	*Argiope bruennichi*	GBCH8723-13	KC195619
Araneae	Araneidae	*Argiope bruennichi*	GBCH8724-13	KC195618
Araneae	Araneidae	*Argiope bruennichi*	GBCH8725-13	KC195617
Araneae	Araneidae	*Argiope bruennichi*	GBCH8726-13	KC195616
Araneae	Araneidae	*Argiope bruennichi*	GBCH8727-13	KC195615
Araneae	Araneidae	*Argiope bruennichi*	GBCH8728-13	KC195614
Araneae	Araneidae	*Argiope bruennichi*	GBCH8729-13	KC195613
Araneae	Araneidae	*Argiope bruennichi*	GBCH8730-13	KC195612
Araneae	Araneidae	*Argiope bruennichi*	GBCH8731-13	KC195611
Araneae	Araneidae	*Argiope bruennichi*	GBCH8732-13	KC195610
Araneae	Araneidae	*Argiope bruennichi*	GBCH8733-13	KC195609
Araneae	Araneidae	*Argiope bruennichi*	GBCH8734-13	KC195608
Araneae	Araneidae	*Argiope bruennichi*	GBCH8735-13	KC195607
Araneae	Araneidae	*Argiope bruennichi*	GBCH8736-13	KC195606
Araneae	Araneidae	*Argiope bruennichi*	GBCH8737-13	KC195605
Araneae	Araneidae	*Argiope bruennichi*	GBCH8738-13	KC195604
Araneae	Araneidae	*Argiope bruennichi*	GBCH8739-13	KC195603
Araneae	Araneidae	*Argiope bruennichi*	GBCH8740-13	KC195602
Araneae	Araneidae	*Argiope bruennichi*	GBCH8741-13	KC195601
Araneae	Araneidae	*Argiope bruennichi*	GBCH8742-13	KC195600
Araneae	Araneidae	*Argiope bruennichi*	GBCH8743-13	KC195599
Araneae	Araneidae	*Argiope bruennichi*	GBCH8744-13	KC195598
Araneae	Araneidae	*Argiope bruennichi*	GBCH8745-13	KC195597
Araneae	Araneidae	*Argiope bruennichi*	GBCH8746-13	KC195596
Araneae	Araneidae	*Argiope bruennichi*	GBCH8747-13	KC195595
Araneae	Araneidae	*Argiope bruennichi*	GBCH8748-13	KC195594
Araneae	Araneidae	*Argiope bruennichi*	GBCH8749-13	KC195593
Araneae	Araneidae	*Argiope bruennichi*	GBCH8750-13	KC195592
Araneae	Araneidae	*Argiope bruennichi*	GBCH8751-13	KC195591
Araneae	Araneidae	*Argiope bruennichi*	GBCH8752-13	KC195590
Araneae	Araneidae	*Argiope bruennichi*	GBCH8753-13	KC195589
Araneae	Araneidae	*Argiope bruennichi*	GBCH8754-13	KC195588
Araneae	Araneidae	*Argiope bruennichi*	GBCH8755-13	KC195587
Araneae	Araneidae	*Argiope bruennichi*	GBCH8756-13	KC195586
Araneae	Araneidae	*Argiope bruennichi*	GBCH8757-13	KC195585
Araneae	Araneidae	*Argiope bruennichi*	GBCH8758-13	KC195584
Araneae	Araneidae	*Argiope bruennichi*	GBCH8759-13	KC195583
Araneae	Araneidae	*Argiope bruennichi*	GBCH8760-13	KC195582

Araneae	Araneidae	*Argiope bruennichi*	GBCH8761-13	KC195581
Araneae	Araneidae	*Argiope bruennichi*	GBCH8762-13	KC195580
Araneae	Araneidae	*Argiope bruennichi*	GBCH8763-13	KC195579
Araneae	Araneidae	*Argiope bruennichi*	GBCH8764-13	KC195578
Araneae	Araneidae	*Argiope bruennichi*	GBCH8765-13	KC195577
Araneae	Araneidae	*Argiope bruennichi*	GBCH8766-13	KC195576
Araneae	Araneidae	*Argiope bruennichi*	GBCH8767-13	KC195575
Araneae	Araneidae	*Argiope bruennichi*	GBCH8768-13	KC195574
Araneae	Araneidae	*Argiope bruennichi*	GBCH8769-13	KC195573
Araneae	Araneidae	*Argiope bruennichi*	GBCH8770-13	KC195572
Araneae	Araneidae	*Argiope bruennichi*	GBCH8771-13	KC195571
Araneae	Araneidae	*Argiope bruennichi*	GBCH8772-13	KC195570
Araneae	Araneidae	*Argiope bruennichi*	GBCH8773-13	KC195569
Araneae	Araneidae	*Argiope bruennichi*	GBCH8774-13	KC195568
Araneae	Araneidae	*Argiope bruennichi*	GBCH8775-13	KC195567
Araneae	Araneidae	*Argiope bruennichi*	GBCH8776-13	KC195566
Araneae	Araneidae	*Argiope bruennichi*	GBCH8777-13	KC195565
Araneae	Araneidae	*Argiope bruennichi*	GBCH8778-13	KC195564
Araneae	Araneidae	*Argiope bruennichi*	GBCH8779-13	KC195563
Araneae	Araneidae	*Argiope bruennichi*	GBCH8780-13	KC195562
Araneae	Araneidae	*Argiope bruennichi*	GBCH8781-13	KC195561
Araneae	Araneidae	*Argiope bruennichi*	GBCH8782-13	KC195560
Araneae	Araneidae	*Argiope bruennichi*	GBCH8783-13	KC195559
Araneae	Araneidae	*Argiope bruennichi*	GBCH8784-13	KC195558
Araneae	Araneidae	*Argiope bruennichi*	GBCH8785-13	KC195557
Araneae	Araneidae	*Argiope bruennichi*	GBCH8786-13	KC195556
Araneae	Araneidae	*Argiope bruennichi*	GBCH8787-13	KC195555
Araneae	Araneidae	*Argiope bruennichi*	GBCH8788-13	KC195554
Araneae	Araneidae	*Argiope bruennichi*	GBCH8789-13	KC195553
Araneae	Araneidae	*Argiope bruennichi*	GBCH8790-13	KC195552
Araneae	Araneidae	*Argiope bruennichi*	GBCH8791-13	KC195551
Araneae	Araneidae	*Argiope bruennichi*	GBCH8792-13	KC195550
Araneae	Araneidae	*Argiope bruennichi*	GBCH8793-13	KC195549
Araneae	Araneidae	*Argiope bruennichi*	GBCH8794-13	KC195548
Araneae	Araneidae	*Argiope bruennichi*	GBCH8795-13	KC195547
Araneae	Araneidae	*Argiope bruennichi*	GBCH8796-13	KC195546
Araneae	Araneidae	*Argiope bruennichi*	GBCH8797-13	KC195545
Araneae	Araneidae	*Argiope bruennichi*	GBCH8798-13	KC195544
Araneae	Araneidae	*Argiope bruennichi*	GBCH8799-13	KC195543
Araneae	Araneidae	*Argiope bruennichi*	GBCH8800-13	KC195542
Araneae	Araneidae	*Argiope bruennichi*	GBCH8801-13	KC195541
Araneae	Araneidae	*Argiope bruennichi*	GBCH8802-13	KC195540
Araneae	Araneidae	*Argiope bruennichi*	GBCH8803-13	KC195539
Araneae	Araneidae	*Argiope bruennichi*	GBCH8804-13	KC195538
Araneae	Araneidae	*Argiope bruennichi*	GBCH8805-13	KC195537
Araneae	Araneidae	*Argiope bruennichi*	GBCH8806-13	KC195536
Araneae	Araneidae	*Argiope bruennichi*	GBCH8807-13	KC195535
Araneae	Araneidae	*Argiope bruennichi*	GBCH8808-13	KC195534
Araneae	Araneidae	*Argiope bruennichi*	GBCH8809-13	KC195533
Araneae	Araneidae	*Argiope bruennichi*	GBCH8810-13	KC195532
Araneae	Araneidae	*Argiope bruennichi*	GBCH8811-13	KC195531
Araneae	Araneidae	*Argiope bruennichi*	GBCH8812-13	KC195530
Araneae	Araneidae	*Argiope bruennichi*	GBCH8813-13	KC195529

Araneae	Araneidae	*Argiope bruennichi*	GBCH8814-13	KC195528
Araneae	Araneidae	*Argiope bruennichi*	GBCH8815-13	KC195527
Araneae	Araneidae	*Argiope bruennichi*	GBCH8816-13	KC195526
Araneae	Araneidae	*Argiope bruennichi*	GBCH8817-13	KC195525
Araneae	Araneidae	*Argiope bruennichi*	GBCH8818-13	KC195524
Araneae	Araneidae	*Argiope bruennichi*	GBCH8819-13	KC195523
Araneae	Araneidae	*Argiope bruennichi*	GBCH8820-13	KC195522
Araneae	Araneidae	*Argiope bruennichi*	GBCH8821-13	KC195521
Araneae	Araneidae	*Argiope bruennichi*	GBCH8822-13	KC195520
Araneae	Araneidae	*Argiope bruennichi*	GBCH8823-13	KC195519
Araneae	Araneidae	*Argiope bruennichi*	GBCH8824-13	KC195518
Araneae	Araneidae	*Argiope bruennichi*	GBCH8825-13	KC195517
Araneae	Araneidae	*Argiope bruennichi*	GBCH8826-13	KC195516
Araneae	Araneidae	*Argiope bruennichi*	GBCH8827-13	KC195515
Araneae	Araneidae	*Argiope bruennichi*	GBCH8828-13	KC195514
Araneae	Araneidae	*Argiope bruennichi*	GBCH8829-13	KC195513
Araneae	Araneidae	*Argiope bruennichi*	GBCH8830-13	KC195512
Araneae	Araneidae	*Argiope bruennichi*	GBCH8831-13	KC195511
Araneae	Araneidae	*Argiope bruennichi*	GBCH8832-13	KC195510
Araneae	Araneidae	*Argiope bruennichi*	GBCH8833-13	KC195509
Araneae	Araneidae	*Argiope bruennichi*	GBCH8834-13	KC195508
Araneae	Araneidae	*Argiope bruennichi*	GBCH8835-13	KC195507
Araneae	Araneidae	*Argiope bruennichi*	GBCH8836-13	KC195506
Araneae	Araneidae	*Argiope bruennichi*	GBCH8837-13	KC195505
Araneae	Araneidae	*Argiope bruennichi*	GBCH8838-13	KC195504
Araneae	Araneidae	*Argiope bruennichi*	GBCH8839-13	KC195503
Araneae	Araneidae	*Argiope bruennichi*	GBCH8840-13	KC195502
Araneae	Araneidae	*Argiope bruennichi*	GBCH8841-13	KC195501
Araneae	Araneidae	*Argiope bruennichi*	GBCH8842-13	KC195500
Araneae	Araneidae	*Argiope bruennichi*	GBCH8843-13	KC195499
Araneae	Araneidae	*Argiope bruennichi*	GBCH8844-13	KC195498
Araneae	Araneidae	*Argiope bruennichi*	GBCH8845-13	KC195497
Araneae	Araneidae	*Argiope bruennichi*	GBCH8846-13	KC195496
Araneae	Araneidae	*Argiope bruennichi*	GBCH8847-13	KC195495
Araneae	Araneidae	*Argiope bruennichi*	GBCH8848-13	KC195494
Araneae	Araneidae	*Argiope bruennichi*	GBCH8849-13	KC195493
Araneae	Araneidae	*Argiope bruennichi*	GBCH8850-13	KC195492
Araneae	Araneidae	*Argiope bruennichi*	GBCH8851-13	KC195491
Araneae	Araneidae	*Argiope bruennichi*	GBCH8852-13	KC195490
Araneae	Araneidae	*Argiope bruennichi*	GBCH8853-13	KC195489
Araneae	Araneidae	*Argiope bruennichi*	GBCH8854-13	KC195488
Araneae	Araneidae	*Argiope bruennichi*	GBCH8855-13	KC195487
Araneae	Araneidae	*Argiope bruennichi*	GBCH8856-13	KC195486
Araneae	Araneidae	*Argiope bruennichi*	GBCH8857-13	KC195485
Araneae	Araneidae	*Argiope bruennichi*	GBCH8858-13	KC195484
Araneae	Araneidae	*Argiope bruennichi*	GBCH8859-13	KC195483
Araneae	Araneidae	*Argiope bruennichi*	GBCH8860-13	KC195482
Araneae	Araneidae	*Argiope bruennichi*	GBCH8861-13	KC195481
Araneae	Araneidae	*Argiope bruennichi*	GBCH8862-13	KC195480
Araneae	Araneidae	*Argiope bruennichi*	GBCH8863-13	KC195479
Araneae	Araneidae	*Argiope bruennichi*	GBCH8864-13	KC195478
Araneae	Araneidae	*Argiope bruennichi*	GBCH8865-13	KC195477
Araneae	Araneidae	*Argiope bruennichi*	GBCH8866-13	KC195476

Araneae	Araneidae	*Argiope bruennichi*	GBCH8867-13	KC195475
Araneae	Araneidae	*Argiope bruennichi*	GBCH8868-13	KC195474
Araneae	Araneidae	*Argiope bruennichi*	GBCH8869-13	KC195473
Araneae	Araneidae	*Argiope bruennichi*	GBCH8870-13	KC195472
Araneae	Araneidae	*Argiope bruennichi*	GBCH8871-13	KC195471
Araneae	Araneidae	*Argiope bruennichi*	GBCH8872-13	KC195470
Araneae	Araneidae	*Argiope bruennichi*	GBCH8873-13	KC195469
Araneae	Araneidae	*Argiope bruennichi*	GBCH8874-13	KC195468
Araneae	Araneidae	*Argiope bruennichi*	GBCH8875-13	KC195467
Araneae	Araneidae	*Argiope bruennichi*	GBCH8876-13	KC195466
Araneae	Araneidae	*Argiope bruennichi*	GBCH8877-13	KC195465
Araneae	Araneidae	*Argiope bruennichi*	GBCH8878-13	KC195464
Araneae	Araneidae	*Argiope bruennichi*	GBCH8879-13	KC195463
Araneae	Araneidae	*Argiope bruennichi*	GBCH8880-13	KC195462
Araneae	Araneidae	*Argiope bruennichi*	GBCH8881-13	KC195461
Araneae	Araneidae	*Argiope bruennichi*	GBCH8882-13	KC195460
Araneae	Araneidae	*Argiope bruennichi*	GBCH8883-13	KC195459
Araneae	Araneidae	*Argiope bruennichi*	GBCH8884-13	KC195458
Araneae	Araneidae	*Argiope bruennichi*	GBCH8885-13	KC195457
Araneae	Araneidae	*Argiope bruennichi*	GBCH8886-13	KC195456
Araneae	Araneidae	*Argiope bruennichi*	GBCH8887-13	KC195455
Araneae	Araneidae	*Argiope bruennichi*	GBCH8888-13	KC195454
Araneae	Araneidae	*Argiope bruennichi*	GBCH8889-13	KC195453
Araneae	Araneidae	*Argiope bruennichi*	GBCH8890-13	KC195452
Araneae	Araneidae	*Argiope bruennichi*	GBCH8891-13	KC195451
Araneae	Araneidae	*Argiope bruennichi*	GBCH8892-13	KC195450
Araneae	Araneidae	*Argiope bruennichi*	GBCH8893-13	KC195449
Araneae	Araneidae	*Argiope bruennichi*	GBCH8894-13	KC195448
Araneae	Araneidae	*Argiope bruennichi*	GBCH8895-13	KC195447
Araneae	Araneidae	*Argiope bruennichi*	GBCH8896-13	KC195446
Araneae	Araneidae	*Argiope bruennichi*	GBCH8897-13	KC195445
Araneae	Araneidae	*Argiope bruennichi*	GBCH8898-13	KC195444
Araneae	Araneidae	*Argiope bruennichi*	GBCH8899-13	KC195443
Araneae	Araneidae	*Argiope bruennichi*	GBCH8900-13	KC195442
Araneae	Araneidae	*Argiope bruennichi*	GBCH8901-13	KC195441
Araneae	Araneidae	*Argiope bruennichi*	GBCH8902-13	KC195440
Araneae	Araneidae	*Argiope bruennichi*	GBCH8903-13	KC195439
Araneae	Araneidae	*Argiope bruennichi*	GBCH8904-13	KC195438
Araneae	Araneidae	*Argiope bruennichi*	GBCH8905-13	KC195437
Araneae	Araneidae	*Argiope bruennichi*	GBCH8906-13	KC195436
Araneae	Araneidae	*Argiope bruennichi*	GBCH8907-13	KC195435
Araneae	Araneidae	*Argiope bruennichi*	GBCH8908-13	KC195434
Araneae	Araneidae	*Argiope bruennichi*	GBCH8909-13	KC195433
Araneae	Araneidae	*Argiope bruennichi*	GBCH8910-13	KC195432
Araneae	Araneidae	*Argiope bruennichi*	GBCH8911-13	KC195431
Araneae	Araneidae	*Argiope bruennichi*	GBCH8912-13	KC195430
Araneae	Araneidae	*Argiope bruennichi*	GBCH8913-13	KC195429
Araneae	Araneidae	*Argiope bruennichi*	GBCH8914-13	KC195428
Araneae	Araneidae	*Argiope bruennichi*	GBCH8915-13	KC195427
Araneae	Araneidae	*Argiope bruennichi*	GBCH8916-13	KC195426
Araneae	Araneidae	*Argiope bruennichi*	GBCH8917-13	KC195425
Araneae	Araneidae	*Argiope bruennichi*	GBCH8918-13	KC195424
Araneae	Araneidae	*Argiope bruennichi*	GBCH8919-13	KC195423

Araneae	Araneidae	*Argiope bruennichi*	GBCH8920-13	KC195422
Araneae	Araneidae	*Argiope bruennichi*	GBCH8921-13	KC195421
Araneae	Araneidae	*Argiope bruennichi*	GBCH8922-13	KC195420
Araneae	Araneidae	*Argiope bruennichi*	GBCH8923-13	KC195419
Araneae	Araneidae	*Argiope bruennichi*	GBCH8924-13	KC195418
Araneae	Araneidae	*Argiope bruennichi*	GBCH8925-13	KC195417
Araneae	Araneidae	*Argiope bruennichi*	GBCH8926-13	KC195416
Araneae	Araneidae	*Argiope bruennichi*	GBCH8927-13	KC195415
Araneae	Araneidae	*Argiope bruennichi*	GBCH8928-13	KC195414
Araneae	Araneidae	*Argiope bruennichi*	GBCH8929-13	KC195413
Araneae	Araneidae	*Argiope bruennichi*	GBCH8930-13	KC195412
Araneae	Araneidae	*Argiope bruennichi*	GBCH8931-13	KC195411
Araneae	Araneidae	*Argiope bruennichi*	GBCH8932-13	KC195410
Araneae	Araneidae	*Argiope bruennichi*	GBCH8933-13	KC195409
Araneae	Araneidae	*Argiope bruennichi*	GBCH8934-13	KC195408
Araneae	Araneidae	*Argiope bruennichi*	GBCH8935-13	KC195407
Araneae	Araneidae	*Argiope bruennichi*	GBCH8936-13	KC195406
Araneae	Araneidae	*Argiope bruennichi*	GBCH8937-13	KC195405
Araneae	Araneidae	*Argiope bruennichi*	GBCH8938-13	KC195404
Araneae	Araneidae	*Argiope bruennichi*	GBCH8939-13	KC195403
Araneae	Araneidae	*Argiope bruennichi*	GBCH8940-13	KC195402
Araneae	Araneidae	*Argiope bruennichi*	GBCH8941-13	KC195401
Araneae	Araneidae	*Argiope bruennichi*	GBCH8942-13	KC195400
Araneae	Araneidae	*Argiope bruennichi*	GBCH8943-13	KC195399
Araneae	Araneidae	*Argiope bruennichi*	GBCH8944-13	KC195398
Araneae	Araneidae	*Argiope bruennichi*	GBCH8945-13	KC195397
Araneae	Araneidae	*Argiope bruennichi*	GBCH8946-13	KC195396
Araneae	Araneidae	*Argiope bruennichi*	GBCH8947-13	KC195395
Araneae	Araneidae	*Argiope bruennichi*	GBCH8948-13	KC195394
Araneae	Araneidae	*Argiope bruennichi*	GBCH8949-13	KC195393
Araneae	Araneidae	*Argiope bruennichi*	GBCH8950-13	KC195392
Araneae	Araneidae	*Argiope bruennichi*	GBCH8951-13	KC195391
Araneae	Araneidae	*Argiope bruennichi*	GBCH8952-13	KC195390
Araneae	Araneidae	*Argiope bruennichi*	GBCH8953-13	KC195389
Araneae	Araneidae	*Argiope bruennichi*	GBCH8954-13	KC195388
Araneae	Araneidae	*Argiope bruennichi*	GBCH8955-13	KC195387
Araneae	Araneidae	*Argiope bruennichi*	GBCH8956-13	KC195386
Araneae	Araneidae	*Argiope bruennichi*	GBCH8957-13	KC195385
Araneae	Araneidae	*Argiope bruennichi*	GBCH8958-13	KC195384
Araneae	Araneidae	*Argiope bruennichi*	GBCH8959-13	KC195383
Araneae	Araneidae	*Argiope bruennichi*	GBCH8960-13	KC195382
Araneae	Araneidae	*Argiope bruennichi*	GBCH8961-13	KC195381
Araneae	Araneidae	*Argiope bruennichi*	GBCH8962-13	KC195380
Araneae	Araneidae	*Argiope bruennichi*	GBCH8963-13	KC195379
Araneae	Araneidae	*Argiope bruennichi*	GBCH8964-13	KC195378
Araneae	Araneidae	*Argiope bruennichi*	GBCH8965-13	KC195377
Araneae	Araneidae	*Argiope bruennichi*	GBCH8966-13	KC195376
Araneae	Araneidae	*Argiope bruennichi*	GBCH8967-13	KC195375
Araneae	Araneidae	*Argiope bruennichi*	GBCH8968-13	KC195374
Araneae	Araneidae	*Argiope bruennichi*	GBCH8969-13	KC195373
Araneae	Araneidae	*Argiope bruennichi*	GBCH8970-13	KC195372
Araneae	Araneidae	*Argiope bruennichi*	GBCH8971-13	KC195371
Araneae	Araneidae	*Argiope bruennichi*	GBCH8972-13	KC195370

Araneae	Araneidae	*Argiope bruennichi*	GBCH8973-13	KC195369
Araneae	Araneidae	*Argiope bruennichi*	GBCH8974-13	KC195368
Araneae	Araneidae	*Argiope bruennichi*	GBCH8975-13	KC195367
Araneae	Araneidae	*Argiope bruennichi*	GBCH8976-13	KC195366
Araneae	Araneidae	*Argiope bruennichi*	GBCH8977-13	KC195365
Araneae	Araneidae	*Argiope bruennichi*	GBCH8978-13	KC195364
Araneae	Araneidae	*Argiope bruennichi*	GBCH8979-13	KC195363
Araneae	Araneidae	*Argiope bruennichi*	GBCH8980-13	KC195362
Araneae	Araneidae	*Argiope bruennichi*	GBCH8981-13	KC195361
Araneae	Araneidae	*Argiope bruennichi*	GBCH8982-13	KC195360
Araneae	Araneidae	*Argiope bruennichi*	GBCH8983-13	KC195359
Araneae	Araneidae	*Argiope bruennichi*	GBCH8984-13	KC195358
Araneae	Araneidae	*Argiope bruennichi*	GBCH8985-13	KC195357
Araneae	Araneidae	*Argiope bruennichi*	GBCH8986-13	KC195356
Araneae	Araneidae	*Argiope bruennichi*	GBCH8987-13	KC195355
Araneae	Araneidae	*Argiope bruennichi*	GBCH8988-13	KC195354
Araneae	Araneidae	*Argiope bruennichi*	GBCH8989-13	KC195353
Araneae	Araneidae	*Argiope bruennichi*	GBCH8990-13	KC195352
Araneae	Araneidae	*Argiope bruennichi*	GBCH8991-13	KC195351
Araneae	Araneidae	*Argiope bruennichi*	GBCH8992-13	KC195350
Araneae	Araneidae	*Argiope bruennichi*	GBCH8993-13	KC195349
Araneae	Araneidae	*Argiope bruennichi*	GBCH8994-13	KC195348
Araneae	Araneidae	*Argiope bruennichi*	GBCH8995-13	KC195347
Araneae	Araneidae	*Argiope bruennichi*	GBCH8996-13	KC195346
Araneae	Araneidae	*Argiope bruennichi*	GBCH8997-13	KC195345
Araneae	Araneidae	*Argiope bruennichi*	GBCH8998-13	KC195344
Araneae	Araneidae	*Argiope bruennichi*	GBCH8999-13	KC195343
Araneae	Araneidae	*Argiope bruennichi*	GBCH9000-13	KC195342
Araneae	Araneidae	*Argiope bruennichi*	GBCH9001-13	KC195341
Araneae	Araneidae	*Argiope bruennichi*	GBCH9002-13	KC195340
Araneae	Araneidae	*Argiope bruennichi*	GBCH9003-13	KC195339
Araneae	Araneidae	*Argiope bruennichi*	GBCH9004-13	KC195338
Araneae	Araneidae	*Argiope bruennichi*	GBCH9005-13	KC195337
Araneae	Araneidae	*Argiope bruennichi*	GBCH9006-13	KC195336
Araneae	Araneidae	*Argiope bruennichi*	GBCH9007-13	KC195335
Araneae	Araneidae	*Argiope bruennichi*	GBCH9008-13	KC195334
Araneae	Araneidae	*Argiope bruennichi*	GBCH9009-13	KC195333
Araneae	Araneidae	*Argiope bruennichi*	GBCH9010-13	KC195332
Araneae	Araneidae	*Argiope bruennichi*	GBCH9011-13	KC195331
Araneae	Araneidae	*Argiope bruennichi*	GBCH9012-13	KC195330
Araneae	Araneidae	*Argiope bruennichi*	GBCH9013-13	KC195329
Araneae	Araneidae	*Argiope bruennichi*	GBCH9014-13	KC195328
Araneae	Araneidae	*Argiope bruennichi*	GBCH9015-13	KC195327
Araneae	Araneidae	*Argiope bruennichi*	GBCH9016-13	KC195326
Araneae	Araneidae	*Argiope bruennichi*	GBCH9017-13	KC195325
Araneae	Araneidae	*Argiope bruennichi*	GBCH9018-13	KC195324
Araneae	Araneidae	*Argiope bruennichi*	GBCH9019-13	KC195323
Araneae	Araneidae	*Argiope bruennichi*	GBCH9020-13	KC195322
Araneae	Araneidae	*Argiope bruennichi*	GBCH9021-13	KC195321
Araneae	Araneidae	*Argiope bruennichi*	GBCH9022-13	KC195320
Araneae	Araneidae	*Argiope bruennichi*	GBCH9023-13	KC195319
Araneae	Araneidae	*Argiope bruennichi*	GBCH9024-13	KC195318
Araneae	Araneidae	*Argiope bruennichi*	GBCH9025-13	KC195317

Araneae	Araneidae	*Argiope bruennichi*	GBCH9026-13	KC195316
Araneae	Araneidae	*Argiope bruennichi*	GBCH9027-13	KC195315
Araneae	Araneidae	*Argiope bruennichi*	GBCH9028-13	KC195314
Araneae	Araneidae	*Argiope bruennichi*	GBCH9029-13	KC195313
Araneae	Araneidae	*Argiope bruennichi*	GBCH9030-13	KC195312
Araneae	Araneidae	*Argiope bruennichi*	GBCH9031-13	KC195311
Araneae	Araneidae	*Argiope bruennichi*	GBCH9032-13	KC195310
Araneae	Araneidae	*Argiope bruennichi*	GBCH9033-13	KC195309
Araneae	Araneidae	*Argiope bruennichi*	GBCH9034-13	KC195308
Araneae	Araneidae	*Argiope bruennichi*	GBCH9035-13	KC195307
Araneae	Araneidae	*Argiope bruennichi*	GBCH9036-13	KC195306
Araneae	Araneidae	*Argiope bruennichi*	GBCH9037-13	KC195305
Araneae	Araneidae	*Argiope bruennichi*	GBCH9038-13	KC195304
Araneae	Araneidae	*Argiope bruennichi*	GBCH9039-13	KC195303
Araneae	Araneidae	*Argiope bruennichi*	GBCH9040-13	KC195302
Araneae	Araneidae	*Argiope bruennichi*	GBCH9041-13	KC195301
Araneae	Araneidae	*Argiope bruennichi*	GBCH9042-13	KC195300
Araneae	Araneidae	*Argiope bruennichi*	GBCH9043-13	KC195299
Araneae	Araneidae	*Argiope bruennichi*	GBCH9044-13	KC195298
Araneae	Araneidae	*Argiope bruennichi*	GBCH9045-13	KC195297
Araneae	Araneidae	*Argiope bruennichi*	GBCH9046-13	KC195296
Araneae	Araneidae	*Argiope bruennichi*	GBCH9047-13	KC195295
Araneae	Araneidae	*Argiope bruennichi*	GBCH9048-13	KC195294
Araneae	Araneidae	*Argiope bruennichi*	GBCH9049-13	KC195293
Araneae	Araneidae	*Argiope bruennichi*	GBCH9050-13	KC195292
Araneae	Araneidae	*Argiope bruennichi*	GBCH9051-13	KC195291
Araneae	Araneidae	*Argiope bruennichi*	GBCH9052-13	KC195290
Araneae	Araneidae	*Argiope bruennichi*	GBCH9053-13	KC195289
Araneae	Araneidae	*Argiope bruennichi*	GBCH9054-13	KC195288
Araneae	Araneidae	*Argiope bruennichi*	GBCH9055-13	KC195287
Araneae	Araneidae	*Argiope bruennichi*	GBCH9056-13	KC195286
Araneae	Araneidae	*Argiope bruennichi*	GBCH9057-13	KC195285
Araneae	Araneidae	*Argiope bruennichi*	GBCH9058-13	KC195284
Araneae	Araneidae	*Argiope bruennichi*	GBCH9059-13	KC195283
Araneae	Araneidae	*Argiope bruennichi*	GBCH9060-13	KC195282
Araneae	Araneidae	*Argiope bruennichi*	GBCH9061-13	KC195281
Araneae	Araneidae	*Argiope bruennichi*	GBCH9062-13	KC195280
Araneae	Araneidae	*Argiope bruennichi*	GBCH9063-13	KC195279
Araneae	Araneidae	*Argiope bruennichi*	GBCH9064-13	KC195278
Araneae	Araneidae	*Argiope bruennichi*	GBCH9065-13	KC195277
Araneae	Araneidae	*Argiope bruennichi*	GBCH9066-13	KC195276
Araneae	Araneidae	*Argiope bruennichi*	GBCH9067-13	KC195275
Araneae	Araneidae	*Argiope bruennichi*	GBCH9068-13	KC195274
Araneae	Araneidae	*Argiope bruennichi*	GBCH9069-13	KC195273
Araneae	Araneidae	*Argiope bruennichi*	GBCH9070-13	KC195272
Araneae	Araneidae	*Argiope bruennichi*	GBCH9071-13	KC195271
Araneae	Araneidae	*Argiope bruennichi*	GBCH9072-13	KC195270
Araneae	Araneidae	*Argiope bruennichi*	GBCH9073-13	KC195269
Araneae	Araneidae	*Argiope bruennichi*	GBCH9074-13	KC195268
Araneae	Araneidae	*Argiope bruennichi*	GBCH9075-13	KC195267
Araneae	Araneidae	*Argiope bruennichi*	GBCH9076-13	KC195266
Araneae	Araneidae	*Argiope bruennichi*	GBCH9077-13	KC195265
Araneae	Araneidae	*Argiope bruennichi*	GBCH9078-13	KC195264

Araneae	Araneidae	*Argiope bruennichi*	GBCH9079-13	KC195263
Araneae	Araneidae	*Argiope bruennichi*	GBCH9080-13	KC195262
Araneae	Araneidae	*Argiope bruennichi*	GBCH9081-13	KC195261
Araneae	Araneidae	*Argiope bruennichi*	GBCH9082-13	KC195260
Araneae	Araneidae	*Argiope bruennichi*	GBCH9083-13	KC195259
Araneae	Araneidae	*Argiope bruennichi*	GBCH9084-13	KC195258
Araneae	Araneidae	*Argiope bruennichi*	GBCH9085-13	KC195257
Araneae	Araneidae	*Argiope bruennichi*	GBCH9086-13	KC195256
Araneae	Araneidae	*Argiope bruennichi*	GBCH9087-13	KC195255
Araneae	Araneidae	*Argiope bruennichi*	GBCH9088-13	KC195254
Araneae	Araneidae	*Argiope bruennichi*	GBCH9089-13	KC195253
Araneae	Araneidae	*Argiope bruennichi*	GBCH9090-13	KC195252
Araneae	Araneidae	*Argiope bruennichi*	GBCH9091-13	KC195251
Araneae	Araneidae	*Argiope bruennichi*	GBCH9092-13	KC195250
Araneae	Araneidae	*Argiope bruennichi*	GBCH9093-13	KC195249
Araneae	Araneidae	*Argiope bruennichi*	GBCH9094-13	KC195248
Araneae	Araneidae	*Argiope bruennichi*	GBCH9095-13	KC195247
Araneae	Araneidae	*Argiope bruennichi*	GBCH9096-13	KC195246
Araneae	Araneidae	*Argiope bruennichi*	GBCH9097-13	KC195245
Araneae	Araneidae	*Argiope bruennichi*	GBCH9098-13	KC195244
Araneae	Araneidae	*Argiope bruennichi*	GBCH9099-13	KC195243
Araneae	Araneidae	*Argiope bruennichi*	GBCH9100-13	KC195242
Araneae	Araneidae	*Argiope bruennichi*	GBCH9101-13	KC195241
Araneae	Araneidae	*Argiope bruennichi*	GBCH9102-13	KC195240
Araneae	Araneidae	*Argiope bruennichi*	GBCH9103-13	KC195239
Araneae	Araneidae	*Argiope bruennichi*	GBCH9104-13	KC195238
Araneae	Araneidae	*Argiope bruennichi*	GBCH9105-13	KC195237
Araneae	Araneidae	*Argiope bruennichi*	GBCH9106-13	KC195236
Araneae	Araneidae	*Argiope bruennichi*	GBCH9107-13	KC195235
Araneae	Araneidae	*Argiope bruennichi*	GBCH9108-13	KC195234
Araneae	Araneidae	*Argiope bruennichi*	GBCH9109-13	KC195233
Araneae	Araneidae	*Argiope bruennichi*	GBCH9110-13	KC195232
Araneae	Araneidae	*Argiope bruennichi*	GBCH9111-13	KC195231
Araneae	Araneidae	*Argiope bruennichi*	GBCH9112-13	KC195230
Araneae	Araneidae	*Argiope bruennichi*	GBCH9113-13	KC195229
Araneae	Araneidae	*Argiope bruennichi*	GBCH9114-13	KC195228
Araneae	Araneidae	*Argiope bruennichi*	GBCH9115-13	KC195227
Araneae	Araneidae	*Argiope bruennichi*	GBCH9116-13	KC195226
Araneae	Araneidae	*Argiope bruennichi*	GBCH9117-13	KC195225
Araneae	Araneidae	*Argiope bruennichi*	GBCH9118-13	KC195224
Araneae	Araneidae	*Argiope bruennichi*	GBCH9119-13	KC195223
Araneae	Araneidae	*Argiope bruennichi*	GBCH9120-13	KC195222
Araneae	Araneidae	*Argiope bruennichi*	GBCH9121-13	KC195221
Araneae	Araneidae	*Argiope bruennichi*	GBCH9122-13	KC195220
Araneae	Araneidae	*Argiope bruennichi*	GBCH9123-13	KC195219
Araneae	Araneidae	*Argiope bruennichi*	GBCH9124-13	KC195218
Araneae	Araneidae	*Argiope bruennichi*	GBCH9125-13	KC195217
Araneae	Araneidae	*Argiope bruennichi*	GBCH9126-13	KC195216
Araneae	Araneidae	*Argiope bruennichi*	GBCH9127-13	KC195215
Araneae	Araneidae	*Argiope bruennichi*	GBCH9128-13	KC195214
Araneae	Araneidae	*Argiope bruennichi*	GBCH9129-13	KC195213
Araneae	Araneidae	*Argiope bruennichi*	GBCH9130-13	KC195212
Araneae	Araneidae	*Argiope bruennichi*	GBCH9131-13	KC195211

Araneae	Araneidae	*Argiope bruennichi*	GBCH9132-13	KC195210
Araneae	Araneidae	*Argiope bruennichi*	GBCH9133-13	KC195209
Araneae	Araneidae	*Argiope bruennichi*	GBCH9134-13	KC195208
Araneae	Araneidae	*Argiope bruennichi*	GBCH9135-13	KC195207
Araneae	Araneidae	*Argiope bruennichi*	GBCH9136-13	KC195206
Araneae	Araneidae	*Argiope bruennichi*	GBCH9137-13	KC195205
Araneae	Araneidae	*Argiope bruennichi*	GBCH9138-13	KC195204
Araneae	Araneidae	*Argiope bruennichi*	GBCH9139-13	KC195203
Araneae	Araneidae	*Argiope bruennichi*	GBCH9140-13	KC195202
Araneae	Araneidae	*Argiope bruennichi*	GBCH9141-13	KC195201
Araneae	Araneidae	*Argiope bruennichi*	GBCH9142-13	KC195200
Araneae	Araneidae	*Argiope bruennichi*	GBCH9143-13	KC195199
Araneae	Araneidae	*Argiope bruennichi*	GBCH9144-13	KC195198
Araneae	Araneidae	*Argiope bruennichi*	GBCH9145-13	KC195197
Araneae	Araneidae	*Argiope bruennichi*	GBCH9146-13	KC195196
Araneae	Araneidae	*Argiope bruennichi*	GBCH9147-13	KC195195
Araneae	Araneidae	*Argiope bruennichi*	GBCH9148-13	KC195194
Araneae	Araneidae	*Argiope bruennichi*	GBCH9149-13	KC195193
Araneae	Araneidae	*Argiope bruennichi*	GBCH9150-13	KC195192
Araneae	Araneidae	*Argiope bruennichi*	GBCH9151-13	KC195191
Araneae	Araneidae	*Argiope bruennichi*	GBCH9152-13	KC195190
Araneae	Araneidae	*Argiope bruennichi*	GBCH9153-13	KC195189
Araneae	Araneidae	*Argiope bruennichi*	GBCH9154-13	KC195188
Araneae	Araneidae	*Argiope bruennichi*	GBCH9155-13	KC195187
Araneae	Araneidae	*Argiope bruennichi*	GBCH9156-13	KC195186
Araneae	Araneidae	*Argiope bruennichi*	GBCH9157-13	KC195185
Araneae	Araneidae	*Argiope bruennichi*	GBCH9158-13	KC195184
Araneae	Araneidae	*Argiope bruennichi*	GBCH9159-13	KC195183
Araneae	Araneidae	*Argiope bruennichi*	GBCH9160-13	KC195182
Araneae	Araneidae	*Argiope bruennichi*	GBCH9161-13	KC195181
Araneae	Araneidae	*Argiope bruennichi*	GBCH9162-13	KC195180
Araneae	Araneidae	*Argiope bruennichi*	GBCH9163-13	KC195179
Araneae	Araneidae	*Argiope bruennichi*	GBCH9164-13	KC195178
Araneae	Araneidae	*Argiope bruennichi*	GBCH9165-13	KC195177
Araneae	Araneidae	*Argiope bruennichi*	GBCH9166-13	KC195176
Araneae	Araneidae	*Argiope bruennichi*	GBCH9167-13	KC195175
Araneae	Araneidae	*Argiope bruennichi*	GBCH9168-13	KC195174
Araneae	Araneidae	*Argiope bruennichi*	GBCH9169-13	KC195173
Araneae	Araneidae	*Argiope bruennichi*	GBCH9170-13	KC195172
Araneae	Araneidae	*Argiope bruennichi*	GBCH9171-13	KC195171
Araneae	Araneidae	*Argiope bruennichi*	GBCH9172-13	KC195170
Araneae	Araneidae	*Argiope bruennichi*	GBCH9173-13	KC195169
Araneae	Araneidae	*Argiope bruennichi*	GBCH9174-13	KC195168
Araneae	Araneidae	*Argiope bruennichi*	GBCH9175-13	KC195167
Araneae	Araneidae	*Argiope bruennichi*	GBCH9176-13	KC195166
Araneae	Araneidae	*Argiope bruennichi*	GBCH9177-13	KC195165
Araneae	Araneidae	*Argiope bruennichi*	GBCH9178-13	KC195164
Araneae	Araneidae	*Argiope bruennichi*	GBCH9179-13	KC195163
Araneae	Araneidae	*Argiope bruennichi*	GBCH9180-13	KC195162
Araneae	Araneidae	*Argiope bruennichi*	GBCH9181-13	KC195161
Araneae	Araneidae	*Argiope bruennichi*	GBCH9182-13	KC195160
Araneae	Araneidae	*Argiope bruennichi*	GBCH9183-13	KC195159
Araneae	Araneidae	*Argiope bruennichi*	GBCH9184-13	KC195158

Araneae	Araneidae	*Argiope bruennichi*	GBCH9185-13	KC195157
Araneae	Araneidae	*Argiope bruennichi*	GBCH9186-13	KC195156
Araneae	Araneidae	*Argiope bruennichi*	GBCH9187-13	KC195155
Araneae	Araneidae	*Argiope bruennichi*	GBCH9188-13	KC195154
Araneae	Araneidae	*Argiope bruennichi*	GBCH9189-13	KC195153
Araneae	Araneidae	*Argiope bruennichi*	GBCH9190-13	KC195152
Araneae	Araneidae	*Argiope bruennichi*	GBCH9191-13	KC195151
Araneae	Araneidae	*Argiope bruennichi*	GBCH9192-13	KC195150
Araneae	Araneidae	*Argiope bruennichi*	GBCH9193-13	KC195149
Araneae	Araneidae	*Argiope bruennichi*	GBCH9194-13	KC195148
Araneae	Araneidae	*Argiope bruennichi*	GBCH9195-13	KC195147
Araneae	Araneidae	*Argiope bruennichi*	GBCH9196-13	KC195146
Araneae	Araneidae	*Argiope bruennichi*	GBCH9197-13	KC195145
Araneae	Araneidae	*Argiope bruennichi*	GBCH9198-13	KC195144
Araneae	Araneidae	*Argiope bruennichi*	GBCH9199-13	KC195143
Araneae	Araneidae	*Argiope bruennichi*	GBCH9200-13	KC195142
Araneae	Araneidae	*Argiope bruennichi*	GBCH9201-13	KC195141
Araneae	Araneidae	*Argiope bruennichi*	GBCH9202-13	KC195140
Araneae	Araneidae	*Argiope bruennichi*	GBCH9203-13	KC195139
Araneae	Araneidae	*Argiope bruennichi*	GBCH9204-13	KC195138
Araneae	Araneidae	*Argiope bruennichi*	GBCH9205-13	KC195137
Araneae	Araneidae	*Argiope bruennichi*	GBCH9206-13	KC195136
Araneae	Araneidae	*Argiope bruennichi*	GBCH9207-13	KC195135
Araneae	Araneidae	*Argiope bruennichi*	GBCH9208-13	KC195134
Araneae	Araneidae	*Argiope bruennichi*	GBCH9209-13	KC195133
Araneae	Araneidae	*Argiope bruennichi*	GBCH9210-13	KC195132
Araneae	Araneidae	*Argiope bruennichi*	GBCH9211-13	KC195131
Araneae	Araneidae	*Argiope bruennichi*	GBCH9212-13	KC195130
Araneae	Araneidae	*Argiope bruennichi*	GBCH9213-13	KC195129
Araneae	Araneidae	*Argiope bruennichi*	GBCH9214-13	KC195128
Araneae	Araneidae	*Argiope bruennichi*	GBCH9215-13	KC195127
Araneae	Araneidae	*Argiope bruennichi*	GBCH9216-13	KC195126
Araneae	Araneidae	*Argiope bruennichi*	GBCH9217-13	KC195125
Araneae	Araneidae	*Argiope bruennichi*	GBCH9218-13	KC195124
Araneae	Araneidae	*Argiope bruennichi*	GBCH9219-13	KC195123
Araneae	Araneidae	*Argiope bruennichi*	GBCH9220-13	KC195122
Araneae	Araneidae	*Argiope bruennichi*	GBCH9221-13	KC195121
Araneae	Araneidae	*Argiope bruennichi*	GBCH9222-13	KC195120
Araneae	Araneidae	*Argiope bruennichi*	GBCH9223-13	KC195119
Araneae	Araneidae	*Argiope bruennichi*	GBCH9224-13	KC195118
Araneae	Araneidae	*Argiope bruennichi*	GBCH9225-13	KC195117
Araneae	Araneidae	*Argiope bruennichi*	GBCH9226-13	KC195116
Araneae	Araneidae	*Argiope bruennichi*	GBCH9227-13	KC195115
Araneae	Araneidae	*Argiope bruennichi*	GBCH9228-13	KC195114
Araneae	Araneidae	*Argiope bruennichi*	GBCH9229-13	KC195113
Araneae	Araneidae	*Argiope bruennichi*	GBCH9230-13	KC195112
Araneae	Araneidae	*Argiope bruennichi*	GBCH9231-13	KC195111
Araneae	Araneidae	*Argiope bruennichi*	GBCH9232-13	KC195110
Araneae	Araneidae	*Argiope bruennichi*	GBCH9233-13	KC195109
Araneae	Araneidae	*Argiope bruennichi*	GBCH9234-13	KC195108
Araneae	Araneidae	*Argiope bruennichi*	GBCH9235-13	KC195107
Araneae	Araneidae	*Argiope bruennichi*	GBCH9236-13	KC195106
Araneae	Araneidae	*Argiope bruennichi*	GBCH9237-13	KC195105

Araneae	Araneidae	*Argiope bruennichi*	GBCH9238-13	KC195104
Araneae	Araneidae	*Argiope bruennichi*	GBCH9239-13	KC195103
Araneae	Araneidae	*Argiope bruennichi*	GBCH9240-13	KC195102
Araneae	Araneidae	*Argiope bruennichi*	GBCH9241-13	KC195101
Araneae	Araneidae	*Argiope bruennichi*	GBCH9242-13	KC195100
Araneae	Araneidae	*Argiope bruennichi*	GBCH9243-13	KC195099
Araneae	Araneidae	*Argiope bruennichi*	GBCH9244-13	KC195098
Araneae	Araneidae	*Argiope bruennichi*	GBCH9245-13	KC195097
Araneae	Araneidae	*Argiope bruennichi*	GBCH9246-13	KC195096
Araneae	Araneidae	*Argiope bruennichi*	GBCH9247-13	KC195095
Araneae	Araneidae	*Argiope bruennichi*	GBCH9248-13	KC195094
Araneae	Araneidae	*Argiope bruennichi*	GBCH9249-13	KC195093
Araneae	Araneidae	*Argiope bruennichi*	GBCH9250-13	KC195092
Araneae	Araneidae	*Argiope bruennichi*	GBCH9251-13	KC195091
Araneae	Araneidae	*Argiope bruennichi*	GBCH9252-13	KC195090
Araneae	Araneidae	*Argiope bruennichi*	GBCH9253-13	KC195089
Araneae	Araneidae	*Argiope bruennichi*	GBCH9254-13	KC195088
Araneae	Araneidae	*Argiope bruennichi*	GBCH9255-13	KC195087
Araneae	Araneidae	*Argiope bruennichi*	GBCH9256-13	KC195086
Araneae	Araneidae	*Argiope bruennichi*	GBCH9257-13	KC195085
Araneae	Araneidae	*Argiope bruennichi*	GBCH9258-13	KC195084
Araneae	Araneidae	*Argiope bruennichi*	GBCH9259-13	KC195083
Araneae	Araneidae	*Argiope bruennichi*	GBCH9260-13	KC195082
Araneae	Araneidae	*Argiope bruennichi*	GBCH9261-13	KC195081
Araneae	Araneidae	*Argiope bruennichi*	GBCH9262-13	KC195080
Araneae	Araneidae	*Argiope bruennichi*	GBCH9263-13	KC195079
Araneae	Araneidae	*Argiope bruennichi*	GBCH9264-13	KC195078
Araneae	Araneidae	*Argiope bruennichi*	GBCH9265-13	KC195077
Araneae	Araneidae	*Argiope bruennichi*	GBCH9266-13	KC195076
Araneae	Araneidae	*Argiope bruennichi*	GBCH9267-13	KC195075
Araneae	Araneidae	*Argiope bruennichi*	GBCH9268-13	KC195074
Araneae	Araneidae	*Argiope bruennichi*	GBCH9269-13	KC195073
Araneae	Araneidae	*Argiope bruennichi*	GBCH9270-13	KC195072
Araneae	Araneidae	*Argiope bruennichi*	GBCH9271-13	KC195071
Araneae	Araneidae	*Argiope bruennichi*	GBCH9272-13	KC195070
Araneae	Araneidae	*Argiope bruennichi*	GBCH9273-13	KC195069
Araneae	Araneidae	*Argiope bruennichi*	GBCH9274-13	KC195068
Araneae	Araneidae	*Argiope bruennichi*	GBCH9275-13	KC195067
Araneae	Araneidae	*Argiope bruennichi*	GBCH9276-13	KC195066
Araneae	Araneidae	*Argiope bruennichi*	GBCH9277-13	KC195065
Araneae	Araneidae	*Argiope bruennichi*	GBCH9278-13	KC195064
Araneae	Araneidae	*Argiope bruennichi*	GBCH9279-13	KC195063
Araneae	Araneidae	*Argiope bruennichi*	GBCH9280-13	KC195062
Araneae	Araneidae	*Argiope bruennichi*	GBCH9281-13	KC195061
Araneae	Araneidae	*Argiope bruennichi*	GBCH9282-13	KC195060
Araneae	Araneidae	*Argiope bruennichi*	GBCH9283-13	KC195059
Araneae	Araneidae	*Argiope bruennichi*	GBCH9284-13	KC195058
Araneae	Araneidae	*Argiope bruennichi*	GBCH9285-13	KC195057
Araneae	Araneidae	*Argiope bruennichi*	GBCH9286-13	KC195056
Araneae	Araneidae	*Argiope bruennichi*	GBCH9287-13	KC195055
Araneae	Araneidae	*Argiope bruennichi*	GBCH9288-13	KC195054
Araneae	Araneidae	*Argiope bruennichi*	GBCH9289-13	KC195053
Araneae	Araneidae	*Argiope bruennichi*	GBCH9290-13	KC195052

Araneae	Araneidae	*Argiope bruennichi*	GBCH9291-13	KC195051
Araneae	Araneidae	*Argiope bruennichi*	GBCH9292-13	KC195050
Araneae	Araneidae	*Argiope bruennichi*	GBCH9293-13	KC195049
Araneae	Araneidae	*Argiope bruennichi*	GBCH9294-13	KC195048
Araneae	Araneidae	*Argiope bruennichi*	GBCH9295-13	KC195047
Araneae	Araneidae	*Argiope bruennichi*	GBCH9296-13	KC195046
Araneae	Araneidae	*Argiope bruennichi*	GBCH9297-13	KC195045
Araneae	Araneidae	*Argiope bruennichi*	GBCH9298-13	KC195044
Araneae	Araneidae	*Argiope bruennichi*	GBCH9299-13	KC195043
Araneae	Araneidae	*Argiope bruennichi*	GBCH9300-13	KC195042
Araneae	Araneidae	*Argiope bruennichi*	GBCH9301-13	KC195041
Araneae	Araneidae	*Argiope bruennichi*	GBCH9302-13	KC195040
Araneae	Araneidae	*Argiope bruennichi*	GBCH9303-13	KC195039
Araneae	Araneidae	*Argiope bruennichi*	GBCH9304-13	KC195038
Araneae	Araneidae	*Argiope bruennichi*	GBCH9305-13	KC195037
Araneae	Araneidae	*Argiope bruennichi*	GBCH9306-13	KC195036
Araneae	Araneidae	*Argiope bruennichi*	GBCH9307-13	KC195035
Araneae	Araneidae	*Argiope bruennichi*	GBCH9308-13	KC195034
Araneae	Araneidae	*Argiope bruennichi*	GBCH9309-13	KC195033
Araneae	Araneidae	*Argiope bruennichi*	GBCH9310-13	KC195032
Araneae	Araneidae	*Argiope bruennichi*	GBCH9311-13	KC195031
Araneae	Araneidae	*Argiope bruennichi*	GBCH9312-13	KC195030
Araneae	Araneidae	*Argiope bruennichi*	GBCH9313-13	KC195029
Araneae	Araneidae	*Argiope bruennichi*	GBCH9314-13	KC195028
Araneae	Araneidae	*Argiope bruennichi*	GBCH9315-13	KC195027
Araneae	Araneidae	*Argiope bruennichi*	GBCH9316-13	KC195026
Araneae	Araneidae	*Argiope bruennichi*	GBCH9317-13	KC195025
Araneae	Araneidae	*Argiope bruennichi*	GBCH9318-13	KC195024
Araneae	Araneidae	*Argiope bruennichi*	GBCH9319-13	KC195023
Araneae	Araneidae	*Argiope bruennichi*	GBCH9320-13	KC195022
Araneae	Araneidae	*Argiope bruennichi*	GBCH9321-13	KC195021
Araneae	Araneidae	*Argiope bruennichi*	GBCH9322-13	KC195020
Araneae	Araneidae	*Argiope bruennichi*	GBCH9323-13	KC195019
Araneae	Araneidae	*Argiope bruennichi*	GBCH9324-13	KC195018
Araneae	Araneidae	*Argiope bruennichi*	GBCH9325-13	KC195017
Araneae	Araneidae	*Argiope bruennichi*	GBCH9326-13	KC194818
Araneae	Araneidae	*Argiope bruennichi*	GBCH9327-13	KC194817
Araneae	Araneidae	*Argiope bruennichi*	GBCH9328-13	KC194816
Araneae	Araneidae	*Argiope bruennichi*	GBCH9329-13	KC194815
Araneae	Araneidae	*Argiope bruennichi*	GBCH9330-13	KC194814
Araneae	Araneidae	*Argiope bruennichi*	GBCH9331-13	KC194813
Araneae	Araneidae	*Argiope bruennichi*	GBCH9332-13	KC194812
Araneae	Araneidae	*Argiope bruennichi*	GBCH9333-13	KC194811
Araneae	Araneidae	*Argiope bruennichi*	GBCH9334-13	KC194810
Araneae	Araneidae	*Argiope bruennichi*	GBCH9335-13	KC194809
Araneae	Araneidae	*Argiope bruennichi*	GBCH9336-13	KC194808
Araneae	Araneidae	*Argiope bruennichi*	GBCH9337-13	KC194807
Araneae	Araneidae	*Argiope bruennichi*	GBCH9338-13	KC194806
Araneae	Araneidae	*Argiope bruennichi*	GBCH9339-13	KC194805
Araneae	Araneidae	*Argiope bruennichi*	GBCH9340-13	KC194804
Araneae	Araneidae	*Argiope bruennichi*	GBCH9341-13	KC194803
Araneae	Araneidae	*Argiope bruennichi*	GBCH9342-13	KC194802
Araneae	Araneidae	*Argiope bruennichi*	GBCH9343-13	KC194801

Araneae	Araneidae	*Argiope bruennichi*	GBCH9344-13	KC194800
Araneae	Araneidae	*Argiope bruennichi*	GBCH9345-13	KC194799
Araneae	Araneidae	*Argiope bruennichi*	GBCH9346-13	KC194798
Araneae	Araneidae	*Argiope bruennichi*	GBCH9347-13	KC194797
Araneae	Araneidae	*Argiope bruennichi*	GBCH9348-13	KC194796
Araneae	Araneidae	*Argiope bruennichi*	GBCH9349-13	KC194795
Araneae	Araneidae	*Argiope bruennichi*	GBCH9350-13	KC194794
Araneae	Araneidae	*Argiope bruennichi*	GBCH9351-13	KC194793
Araneae	Araneidae	*Argiope bruennichi*	GBCH9352-13	KC194792
Araneae	Araneidae	*Argiope bruennichi*	GBCH9353-13	KC194791
Araneae	Araneidae	*Argiope bruennichi*	GBCH9354-13	KC194790
Araneae	Araneidae	*Argiope bruennichi*	GBCH9355-13	KC194789
Araneae	Araneidae	*Argiope bruennichi*	GBCH9356-13	KC194788
Araneae	Araneidae	*Argiope bruennichi*	GBCH9357-13	KC194787
Araneae	Araneidae	*Argiope bruennichi*	GBCH9358-13	KC194786
Araneae	Araneidae	*Argiope bruennichi*	GBCH9359-13	KC194785
Araneae	Araneidae	*Argiope bruennichi*	GBCH9360-13	KC194784
Araneae	Araneidae	*Argiope bruennichi*	GBCH9361-13	KC194783
Araneae	Araneidae	*Argiope bruennichi*	GBCH9362-13	KC194782
Araneae	Araneidae	*Argiope bruennichi*	GBCH9363-13	KC194781
Araneae	Araneidae	*Argiope bruennichi*	GBCH9364-13	KC194780
Araneae	Araneidae	*Argiope bruennichi*	GBCH9365-13	KC194779
Araneae	Araneidae	*Argiope bruennichi*	GBCH9366-13	KC194778
Araneae	Araneidae	*Argiope bruennichi*	GBCH9367-13	KC194777
Araneae	Araneidae	*Argiope bruennichi*	GBCH9368-13	KC194776
Araneae	Araneidae	*Argiope bruennichi*	GBCH9369-13	KC194775
Araneae	Araneidae	*Argiope bruennichi*	GBCH9370-13	KC194774
Araneae	Araneidae	*Argiope bruennichi*	GBCH9371-13	KC194773
Araneae	Araneidae	*Argiope bruennichi*	GBCH9372-13	KC194772
Araneae	Araneidae	*Argiope bruennichi*	GBCH9373-13	KC194771
Araneae	Araneidae	*Argiope bruennichi*	GBCH9374-13	KC194770
Araneae	Araneidae	*Argiope bruennichi*	GBCH9375-13	KC194769
Araneae	Araneidae	*Argiope bruennichi*	GBCH9376-13	KC194768
Araneae	Araneidae	*Argiope bruennichi*	GBCH9377-13	KC194767
Araneae	Araneidae	*Argiope bruennichi*	GBCH9378-13	KC194766
Araneae	Araneidae	*Argiope bruennichi*	GBCH9379-13	KC194765
Araneae	Araneidae	*Argiope bruennichi*	GBCH9380-13	KC194764
Araneae	Araneidae	*Argiope bruennichi*	GBCH9381-13	KC194763
Araneae	Araneidae	*Argiope bruennichi*	GBCH9382-13	KC194762
Araneae	Araneidae	*Argiope bruennichi*	GBCH9383-13	KC194761
Araneae	Araneidae	*Argiope bruennichi*	GBCH9384-13	KC194760
Araneae	Araneidae	*Argiope bruennichi*	GBCH9385-13	KC194759
Araneae	Araneidae	*Argiope bruennichi*	GBCH9386-13	KC194518
Araneae	Araneidae	*Argiope bruennichi*	GBCH9387-13	KC194517
Araneae	Araneidae	*Argiope bruennichi*	GBCH9388-13	KC194516
Araneae	Araneidae	*Argiope bruennichi*	GBCH9389-13	KC194515
Araneae	Araneidae	*Argiope bruennichi*	GBCH9390-13	KC194514
Araneae	Araneidae	*Argiope bruennichi*	GBCH9391-13	KC194513
Araneae	Araneidae	*Argiope bruennichi*	GBCH9392-13	KC194512
Araneae	Araneidae	*Argiope bruennichi*	GBCH9393-13	KC194511
Araneae	Araneidae	*Argiope bruennichi*	GBCH9394-13	KC194510
Araneae	Araneidae	*Argiope bruennichi*	GBCH9395-13	KC194509
Araneae	Araneidae	*Argiope bruennichi*	GBCH9396-13	KC194508

Araneae	Araneidae	*Argiope bruennichi*	GBCH9397-13	KC194507
Araneae	Araneidae	*Argiope bruennichi*	GBCH9398-13	KC194506
Araneae	Araneidae	*Argiope bruennichi*	GBCH9399-13	KC194505
Araneae	Araneidae	*Argiope bruennichi*	GBCH9400-13	KC194504
Araneae	Araneidae	*Argiope bruennichi*	GBCH9401-13	KC194503
Araneae	Araneidae	*Argiope bruennichi*	GBCH9402-13	KC194502
Araneae	Araneidae	*Argiope bruennichi*	GBCH9403-13	KC194501
Araneae	Araneidae	*Argiope bruennichi*	GBCH9404-13	KC194500
Araneae	Araneidae	*Argiope bruennichi*	GBCH9405-13	KC194499
Araneae	Araneidae	*Argiope bruennichi*	GBCH9406-13	KC194498
Araneae	Araneidae	*Argiope bruennichi*	GBCH9407-13	KC194497
Araneae	Araneidae	*Argiope bruennichi*	GBCH9408-13	KC194496
Araneae	Araneidae	*Argiope bruennichi*	GBCH9409-13	KC194495
Araneae	Araneidae	*Argiope bruennichi*	GBCH9410-13	KC194494
Araneae	Araneidae	*Argiope bruennichi*	GBCH9411-13	KC194493
Araneae	Araneidae	*Argiope bruennichi*	GBCH9412-13	KC194492
Araneae	Araneidae	*Argiope bruennichi*	GBCH9413-13	KC194491
Araneae	Araneidae	*Argiope bruennichi*	GBCH9414-13	KC194490
Araneae	Araneidae	*Argiope bruennichi*	GBCH9415-13	KC194489
Araneae	Araneidae	*Argiope bruennichi*	GBCH9416-13	KC194488
Araneae	Araneidae	*Argiope bruennichi*	GBCH9417-13	KC194487
Araneae	Araneidae	*Argiope bruennichi*	GBCH9418-13	KC194486
Araneae	Araneidae	*Argiope bruennichi*	GBCH9419-13	KC194485
Araneae	Araneidae	*Argiope bruennichi*	GBCH9420-13	KC194484
Araneae	Araneidae	*Argiope bruennichi*	GBCH9421-13	KC194483
Araneae	Araneidae	*Argiope bruennichi*	GBCH9422-13	KC194482
Araneae	Araneidae	*Argiope bruennichi*	GBCH9423-13	KC194481
Araneae	Araneidae	*Argiope bruennichi*	GBCH9424-13	KC194480
Araneae	Araneidae	*Argiope bruennichi*	GBCH9425-13	KC194479
Araneae	Araneidae	*Argiope bruennichi*	GBCH9426-13	KC194478
Araneae	Araneidae	*Argiope bruennichi*	GBCH9427-13	KC194477
Araneae	Araneidae	*Argiope bruennichi*	GBCH9428-13	KC194476
Araneae	Araneidae	*Argiope bruennichi*	GBCH9429-13	KC194475
Araneae	Araneidae	*Argiope bruennichi*	GBCH9430-13	KC194474
Araneae	Araneidae	*Argiope bruennichi*	GBCH9431-13	KC194473
Araneae	Araneidae	*Argiope bruennichi*	GBCH9432-13	KC194472
Araneae	Araneidae	*Argiope bruennichi*	GBCH9433-13	KC194471
Araneae	Araneidae	*Argiope bruennichi*	GBCH9434-13	KC194470
Araneae	Araneidae	*Argiope bruennichi*	GBCH9435-13	KC194469
Araneae	Araneidae	*Argiope bruennichi*	GBCH9436-13	KC194468
Araneae	Araneidae	*Argiope bruennichi*	GBCH9437-13	KC194467
Araneae	Araneidae	*Argiope bruennichi*	GBCH9438-13	KC194466
Araneae	Araneidae	*Argiope bruennichi*	GBCH9439-13	KC194465
Araneae	Araneidae	*Argiope bruennichi*	GBCH9440-13	KC194464
Araneae	Araneidae	*Argiope bruennichi*	GBCH9441-13	KC194463
Araneae	Araneidae	*Argiope bruennichi*	GBCH9442-13	KC194462
Araneae	Araneidae	*Argiope bruennichi*	GBCH9443-13	KC194461
Araneae	Araneidae	*Argiope bruennichi*	GBCH9444-13	KC194460
Araneae	Araneidae	*Argiope bruennichi*	GBCH9445-13	KC194459
Araneae	Araneidae	*Argiope bruennichi*	GBCH9446-13	KC194458
Araneae	Araneidae	*Argiope bruennichi*	GBCH9447-13	KC194457
Araneae	Araneidae	*Argiope bruennichi*	GBCH9448-13	KC194456
Araneae	Araneidae	*Argiope bruennichi*	GBCH9449-13	KC194455

Araneae	Araneidae	*Argiope bruennichi*	GBCH9450-13	KC194454
Araneae	Araneidae	*Argiope bruennichi*	GBCH9451-13	KC194453
Araneae	Araneidae	*Argiope bruennichi*	GBCH9452-13	KC194452
Araneae	Araneidae	*Argiope bruennichi*	GBCH9453-13	KC194451
Araneae	Araneidae	*Argiope bruennichi*	GBCH9454-13	KC194450
Araneae	Araneidae	*Argiope bruennichi*	GBCH9455-13	KC194449
Araneae	Araneidae	*Argiope bruennichi*	GBCH9456-13	KC194448
Araneae	Araneidae	*Argiope bruennichi*	GBCH9457-13	KC194447
Araneae	Araneidae	*Argiope bruennichi*	GBCH9458-13	KC194446
Araneae	Araneidae	*Argiope bruennichi*	GBCH9459-13	KC194445
Araneae	Araneidae	*Argiope bruennichi*	GBCH9460-13	KC194444
Araneae	Araneidae	*Argiope bruennichi*	GBCH9461-13	KC194443
Araneae	Araneidae	*Argiope bruennichi*	GBCH9462-13	KC194442
Araneae	Araneidae	*Argiope bruennichi*	GBCH9463-13	KC194441
Araneae	Araneidae	*Argiope bruennichi*	GBCH9464-13	KC194440
Araneae	Araneidae	*Argiope bruennichi*	GBCH9465-13	KC194439
Araneae	Araneidae	*Argiope bruennichi*	GBCH9466-13	KC194438
Araneae	Araneidae	*Argiope bruennichi*	GBCH9467-13	KC194437
Araneae	Araneidae	*Argiope bruennichi*	GBCH9468-13	KC194436
Araneae	Araneidae	*Argiope bruennichi*	GBCH9469-13	KC194435
Araneae	Araneidae	*Argiope bruennichi*	GBCH9470-13	KC194434
Araneae	Araneidae	*Argiope bruennichi*	GBCH9471-13	KC194433
Araneae	Araneidae	*Argiope bruennichi*	GBCH9472-13	KC194432
Araneae	Araneidae	*Argiope bruennichi*	GBCH9473-13	KC194431
Araneae	Araneidae	*Argiope bruennichi*	GBCH9474-13	KC194430
Araneae	Araneidae	*Argiope bruennichi*	GBCH9475-13	KC194429
Araneae	Araneidae	*Argiope bruennichi*	GBCH9476-13	KC194428
Araneae	Araneidae	*Argiope bruennichi*	GBCH9477-13	KC194427
Araneae	Araneidae	*Argiope bruennichi*	GBCH9478-13	KC194426
Araneae	Araneidae	*Argiope bruennichi*	GBCH9479-13	KC194425
Araneae	Araneidae	*Argiope bruennichi*	GBCH9480-13	KC194424
Araneae	Araneidae	*Argiope bruennichi*	GBCH9481-13	KC194423
Araneae	Araneidae	*Argiope bruennichi*	GBCH9482-13	KC194422
Araneae	Araneidae	*Argiope bruennichi*	GBCH9483-13	KC194421
Araneae	Araneidae	*Argiope bruennichi*	GBCH9484-13	KC194420
Araneae	Araneidae	*Argiope bruennichi*	GBCH9485-13	KC194419
Araneae	Araneidae	*Argiope bruennichi*	GBCH9486-13	KC194318
Araneae	Araneidae	*Argiope bruennichi*	GBCH9487-13	KC194317
Araneae	Araneidae	*Argiope bruennichi*	GBCH9488-13	KC194316
Araneae	Araneidae	*Argiope bruennichi*	GBCH9489-13	KC194315
Araneae	Araneidae	*Argiope bruennichi*	GBCH9490-13	KC194314
Araneae	Araneidae	*Argiope bruennichi*	GBCH9491-13	KC194313
Araneae	Araneidae	*Argiope bruennichi*	GBCH9492-13	KC194312
Araneae	Araneidae	*Argiope bruennichi*	GBCH9493-13	KC194311
Araneae	Araneidae	*Argiope bruennichi*	GBCH9494-13	KC194310
Araneae	Araneidae	*Argiope bruennichi*	GBCH9495-13	KC194309
Araneae	Araneidae	*Argiope bruennichi*	GBCH9496-13	KC194308
Araneae	Araneidae	*Argiope bruennichi*	GBCH9497-13	KC194307
Araneae	Araneidae	*Argiope bruennichi*	GBCH9498-13	KC194306
Araneae	Araneidae	*Argiope bruennichi*	GBCH9499-13	KC194305
Araneae	Araneidae	*Argiope bruennichi*	GBCH9500-13	KC194304
Araneae	Araneidae	*Argiope bruennichi*	GBCH9501-13	KC194303
Araneae	Araneidae	*Argiope bruennichi*	GBCH9502-13	KC194302

Araneae	Araneidae	*Argiope bruennichi*	GBCH9503-13	KC194301
Araneae	Araneidae	*Argiope bruennichi*	GBCH9504-13	KC194300
Araneae	Araneidae	*Argiope bruennichi*	GBCH9505-13	KC194299
Araneae	Araneidae	*Argiope bruennichi*	GBCH9506-13	KC194298
Araneae	Araneidae	*Argiope bruennichi*	GBCH9507-13	KC194297
Araneae	Araneidae	*Argiope bruennichi*	GBCH9508-13	KC194296
Araneae	Araneidae	*Argiope bruennichi*	GBCH9509-13	KC194295
Araneae	Araneidae	*Argiope bruennichi*	GBCH9510-13	KC194294
Araneae	Araneidae	*Argiope bruennichi*	GBCH9511-13	KC194293
Araneae	Araneidae	*Argiope bruennichi*	GBCH9512-13	KC194292
Araneae	Araneidae	*Argiope bruennichi*	GBCH9513-13	KC194291
Araneae	Araneidae	*Argiope bruennichi*	GBCH9514-13	KC194290
Araneae	Araneidae	*Argiope bruennichi*	GBCH9515-13	KC194289
Araneae	Araneidae	*Argiope bruennichi*	GBCH9516-13	KC194288
Araneae	Araneidae	*Argiope bruennichi*	GBCH9517-13	KC194287
Araneae	Araneidae	*Argiope bruennichi*	GBCH9518-13	KC194286
Araneae	Araneidae	*Argiope bruennichi*	GBCH9519-13	KC194285
Araneae	Araneidae	*Argiope bruennichi*	GBCH9520-13	KC194284
Araneae	Araneidae	*Argiope bruennichi*	GBCH9521-13	KC194283
Araneae	Araneidae	*Argiope bruennichi*	GBCH9522-13	KC194282
Araneae	Araneidae	*Argiope bruennichi*	GBCH9523-13	KC194281
Araneae	Araneidae	*Argiope bruennichi*	GBCH9524-13	KC194280
Araneae	Araneidae	*Argiope bruennichi*	GBCH9525-13	KC194279
Araneae	Araneidae	*Argiope bruennichi*	GBCH9526-13	KC194278
Araneae	Araneidae	*Argiope bruennichi*	GBCH9527-13	KC194277
Araneae	Araneidae	*Argiope bruennichi*	GBCH9528-13	KC194276
Araneae	Araneidae	*Argiope bruennichi*	GBCH9529-13	KC194275
Araneae	Araneidae	*Argiope bruennichi*	GBCH9530-13	KC194274
Araneae	Araneidae	*Argiope bruennichi*	GBCH9531-13	KC194273
Araneae	Araneidae	*Argiope bruennichi*	GBCH9532-13	KC194272
Araneae	Araneidae	*Argiope bruennichi*	GBCH9533-13	KC194271
Araneae	Araneidae	*Argiope bruennichi*	GBCH9534-13	KC194270
Araneae	Araneidae	*Argiope bruennichi*	GBCH9535-13	KC194269
Araneae	Araneidae	*Argiope bruennichi*	GBCH9536-13	KC194268
Araneae	Araneidae	*Argiope bruennichi*	GBCH9537-13	KC194267
Araneae	Araneidae	*Argiope bruennichi*	GBCH9538-13	KC194266
Araneae	Araneidae	*Argiope bruennichi*	GBCH9539-13	KC194265
Araneae	Araneidae	*Argiope bruennichi*	GBCH9540-13	KC194264
Araneae	Araneidae	*Argiope bruennichi*	GBCH9541-13	KC194263
Araneae	Araneidae	*Argiope bruennichi*	GBCH9542-13	KC194262
Araneae	Araneidae	*Argiope bruennichi*	GBCH9543-13	KC194261
Araneae	Araneidae	*Argiope bruennichi*	GBCH9544-13	KC194260
Araneae	Araneidae	*Argiope bruennichi*	GBCH9545-13	KC194259
Araneae	Araneidae	*Argiope bruennichi*	GBCH9546-13	KC194258
Araneae	Araneidae	*Argiope bruennichi*	GBCH9547-13	KC194257
Araneae	Araneidae	*Argiope bruennichi*	GBCH9548-13	KC194256
Araneae	Araneidae	*Argiope bruennichi*	GBCH9549-13	KC194255
Araneae	Araneidae	*Argiope bruennichi*	GBCH9550-13	KC194254
Araneae	Araneidae	*Argiope bruennichi*	GBCH9551-13	KC194253
Araneae	Araneidae	*Argiope bruennichi*	GBCH9552-13	KC194252
Araneae	Araneidae	*Argiope bruennichi*	GBCH9553-13	KC194251
Araneae	Araneidae	*Argiope bruennichi*	GBCH9554-13	KC194250
Araneae	Araneidae	*Argiope bruennichi*	GBCH9555-13	KC194249

Araneae	Araneidae	*Argiope bruennichi*	GBCH9556-13	KC194248
Araneae	Araneidae	*Argiope bruennichi*	GBCH9557-13	KC194247
Araneae	Araneidae	*Argiope bruennichi*	GBCH9558-13	KC194246
Araneae	Araneidae	*Argiope bruennichi*	GBCH9559-13	KC194245
Araneae	Araneidae	*Argiope bruennichi*	GBCH9560-13	KC194244
Araneae	Araneidae	*Argiope bruennichi*	GBCH9561-13	KC194243
Araneae	Araneidae	*Argiope bruennichi*	GBCH9562-13	KC194242
Araneae	Araneidae	*Argiope bruennichi*	GBCH9563-13	KC194241
Araneae	Araneidae	*Argiope bruennichi*	GBCH9564-13	KC194240
Araneae	Araneidae	*Argiope bruennichi*	GBCH9565-13	KC194239
Araneae	Araneidae	*Argiope bruennichi*	GBCH9566-13	KC194238
Araneae	Araneidae	*Argiope bruennichi*	GBCH9567-13	KC194237
Araneae	Araneidae	*Argiope bruennichi*	GBCH9568-13	KC194236
Araneae	Araneidae	*Argiope bruennichi*	GBCH9569-13	KC194235
Araneae	Araneidae	*Argiope bruennichi*	GBCH9570-13	KC194234
Araneae	Araneidae	*Argiope bruennichi*	GBCH9571-13	KC194233
Araneae	Araneidae	*Argiope bruennichi*	GBCH9572-13	KC194232
Araneae	Araneidae	*Argiope bruennichi*	GBCH9573-13	KC194231
Araneae	Araneidae	*Argiope bruennichi*	GBCH9574-13	KC194230
Araneae	Araneidae	*Argiope bruennichi*	GBCH9575-13	KC194229
Araneae	Araneidae	*Argiope bruennichi*	GBCH9576-13	KC194228
Araneae	Araneidae	*Argiope bruennichi*	GBCH9577-13	KC194227
Araneae	Araneidae	*Argiope bruennichi*	GBCH9578-13	KC194226
Araneae	Araneidae	*Argiope bruennichi*	GBCH9579-13	KC194225
Araneae	Araneidae	*Argiope bruennichi*	GBCH9580-13	KC194224
Araneae	Araneidae	*Argiope bruennichi*	GBCH9581-13	KC194223
Araneae	Araneidae	*Argiope bruennichi*	GBCH9582-13	KC194222
Araneae	Araneidae	*Argiope bruennichi*	GBCH9583-13	KC194221
Araneae	Araneidae	*Argiope bruennichi*	GBCH9584-13	KC194220
Araneae	Araneidae	*Argiope bruennichi*	GBCH9585-13	KC194219
Araneae	Araneidae	*Argiope bruennichi*	GBCH9586-13	KC194218
Araneae	Araneidae	*Argiope bruennichi*	GBCH9587-13	KC194217
Araneae	Araneidae	*Argiope bruennichi*	GBCH9588-13	KC194216
Araneae	Araneidae	*Argiope bruennichi*	GBCH9589-13	KC194215
Araneae	Araneidae	*Argiope bruennichi*	GBCH9590-13	KC194214
Araneae	Araneidae	*Argiope bruennichi*	GBCH9591-13	KC194213
Araneae	Araneidae	*Argiope bruennichi*	GBCH9592-13	KC194212
Araneae	Araneidae	*Argiope bruennichi*	GBCH9593-13	KC194211
Araneae	Araneidae	*Argiope bruennichi*	GBCH9594-13	KC194210
Araneae	Araneidae	*Argiope bruennichi*	GBCH9595-13	KC194209
Araneae	Araneidae	*Argiope bruennichi*	GBCH9596-13	KC194208
Araneae	Araneidae	*Argiope bruennichi*	GBCH9597-13	KC194207
Araneae	Araneidae	*Argiope bruennichi*	GBCH9598-13	KC194206
Araneae	Araneidae	*Argiope bruennichi*	GBCH9599-13	KC194205
Araneae	Araneidae	*Argiope bruennichi*	GBCH9600-13	KC194204
Araneae	Araneidae	*Argiope bruennichi*	GBCH9601-13	KC194203
Araneae	Araneidae	*Argiope bruennichi*	GBCH9602-13	KC194202
Araneae	Araneidae	*Argiope bruennichi*	GBCH9603-13	KC194201
Araneae	Araneidae	*Argiope bruennichi*	GBCH9604-13	KC194200
Araneae	Araneidae	*Argiope bruennichi*	GBCH9605-13	KC194199
Araneae	Araneidae	*Argiope bruennichi*	GBCH9606-13	KC194198
Araneae	Araneidae	*Argiope bruennichi*	GBCH9607-13	KC194197
Araneae	Araneidae	*Argiope bruennichi*	GBCH9608-13	KC194196

Araneae	Araneidae	*Argiope bruennichi*	GBCH9609-13	KC194195
Araneae	Araneidae	*Argiope bruennichi*	GBCH9610-13	KC194194
Araneae	Araneidae	*Argiope bruennichi*	GBCH9611-13	KC194193
Araneae	Araneidae	*Argiope bruennichi*	GBCH9612-13	KC194192
Araneae	Araneidae	*Argiope bruennichi*	GBCH9613-13	KC194191
Araneae	Araneidae	*Argiope bruennichi*	GBCH9614-13	KC194190
Araneae	Araneidae	*Argiope bruennichi*	GBCH9615-13	KC194189
Araneae	Araneidae	*Argiope bruennichi*	GBCH9616-13	KC194188
Araneae	Araneidae	*Argiope bruennichi*	GBCH9617-13	KC194187
Araneae	Araneidae	*Argiope bruennichi*	GBCH9618-13	KC194186
Araneae	Araneidae	*Argiope bruennichi*	GBCH9619-13	KC194185
Araneae	Araneidae	*Argiope bruennichi*	GBCH9620-13	KC194184
Araneae	Araneidae	*Argiope bruennichi*	GBCH9621-13	KC194183
Araneae	Araneidae	*Argiope bruennichi*	GBCH9622-13	KC194182
Araneae	Araneidae	*Argiope bruennichi*	GBCH9623-13	KC194181
Araneae	Araneidae	*Argiope bruennichi*	GBCH9624-13	KC194180
Araneae	Araneidae	*Argiope bruennichi*	GBCH9625-13	KC194179
Araneae	Araneidae	*Argiope bruennichi*	GBCH9626-13	KC194178
Araneae	Araneidae	*Argiope bruennichi*	GBCH9627-13	KC194177
Araneae	Araneidae	*Argiope bruennichi*	GBCH9628-13	KC194176
Araneae	Araneidae	*Argiope bruennichi*	GBCH9629-13	KC194175
Araneae	Araneidae	*Argiope bruennichi*	GBCH9630-13	KC194174
Araneae	Araneidae	*Argiope bruennichi*	GBCH9631-13	KC194173
Araneae	Araneidae	*Argiope bruennichi*	GBCH9632-13	KC194172
Araneae	Araneidae	*Argiope bruennichi*	GBCH9633-13	KC194171
Araneae	Araneidae	*Argiope bruennichi*	GBCH9634-13	KC194170
Araneae	Araneidae	*Argiope bruennichi*	GBCH9635-13	KC194169
Araneae	Araneidae	*Argiope bruennichi*	GBCH9636-13	KC194168
Araneae	Araneidae	*Argiope bruennichi*	GBCH9637-13	KC194167
Araneae	Araneidae	*Argiope bruennichi*	GBCH9638-13	KC194166
Araneae	Araneidae	*Argiope bruennichi*	GBCH9639-13	KC194165
Araneae	Araneidae	*Argiope bruennichi*	GBCH9640-13	KC194164
Araneae	Araneidae	*Argiope bruennichi*	GBCH9641-13	KC194163
Araneae	Araneidae	*Argiope bruennichi*	GBCH9642-13	KC194162
Araneae	Araneidae	*Argiope bruennichi*	GBCH9643-13	KC194161
Araneae	Araneidae	*Argiope bruennichi*	GBCH9644-13	KC194160
Araneae	Araneidae	*Argiope bruennichi*	GBCH9645-13	KC194159
Araneae	Araneidae	*Argiope bruennichi*	GBCH9646-13	KC194158
Araneae	Araneidae	*Argiope bruennichi*	GBCH9647-13	KC194157
Araneae	Araneidae	*Argiope bruennichi*	GBCH9648-13	KC194156
Araneae	Araneidae	*Argiope bruennichi*	GBCH9649-13	KC194155
Araneae	Araneidae	*Argiope bruennichi*	GBCH9650-13	KC194154
Araneae	Araneidae	*Argiope bruennichi*	GBCH9651-13	KC194153
Araneae	Araneidae	*Argiope bruennichi*	GBCH9652-13	KC194152
Araneae	Araneidae	*Argiope bruennichi*	GBCH9653-13	KC194151
Araneae	Araneidae	*Argiope bruennichi*	GBCH9654-13	KC194150
Araneae	Araneidae	*Argiope bruennichi*	GBCH9655-13	KC194149
Araneae	Araneidae	*Argiope bruennichi*	GBCH9656-13	KC194148
Araneae	Araneidae	*Argiope bruennichi*	GBCH9657-13	KC194147
Araneae	Araneidae	*Argiope bruennichi*	GBCH9658-13	KC194146
Araneae	Araneidae	*Argiope bruennichi*	GBCH9659-13	KC194145
Araneae	Araneidae	*Argiope bruennichi*	GBCH9660-13	KC194144
Araneae	Araneidae	*Argiope bruennichi*	GBCH9661-13	KC194143

Araneae	Araneidae	*Argiope bruennichi*	GBCH9662-13	KC194142
Araneae	Araneidae	*Argiope bruennichi*	GBCH9663-13	KC194141
Araneae	Araneidae	*Argiope bruennichi*	GBCH9664-13	KC194140
Araneae	Araneidae	*Argiope bruennichi*	GBCH9665-13	KC194139
Araneae	Araneidae	*Argiope bruennichi*	GBCH9666-13	KC194138
Araneae	Araneidae	*Argiope bruennichi*	GBCH9667-13	KC194137
Araneae	Araneidae	*Argiope bruennichi*	GBCH9668-13	KC194136
Araneae	Araneidae	*Argiope bruennichi*	GBCH9669-13	KC194135
Araneae	Araneidae	*Argiope bruennichi*	GBCH9670-13	KC194134
Araneae	Araneidae	*Argiope bruennichi*	GBCH9671-13	KC194133
Araneae	Araneidae	*Argiope bruennichi*	GBCH9672-13	KC194132
Araneae	Araneidae	*Argiope bruennichi*	GBCH9673-13	KC194131
Araneae	Araneidae	*Argiope bruennichi*	GBCH9674-13	KC194130
Araneae	Araneidae	*Argiope bruennichi*	GBCH9675-13	KC194129
Araneae	Araneidae	*Argiope bruennichi*	GBCH9676-13	KC194128
Araneae	Araneidae	*Argiope bruennichi*	GBCH9677-13	KC194127
Araneae	Araneidae	*Argiope bruennichi*	GBCH9678-13	KC194126
Araneae	Araneidae	*Argiope bruennichi*	GBCH9679-13	KC194125
Araneae	Araneidae	*Argiope bruennichi*	GBCH9680-13	KC194124
Araneae	Araneidae	*Argiope bruennichi*	GBCH9681-13	KC194123
Araneae	Araneidae	*Argiope bruennichi*	GBCH9682-13	KC194122
Araneae	Araneidae	*Argiope bruennichi*	GBCH9683-13	KC194121
Araneae	Araneidae	*Argiope bruennichi*	GBCH9684-13	KC194120
Araneae	Araneidae	*Argiope bruennichi*	GBCH9685-13	KC194119
Araneae	Araneidae	*Argiope bruennichi*	GBCH9686-13	KC194118
Araneae	Araneidae	*Argiope bruennichi*	GBCH9687-13	KC194117
Araneae	Araneidae	*Argiope bruennichi*	GBCH9688-13	KC194116
Araneae	Araneidae	*Argiope bruennichi*	GBCH9689-13	KC194115
Araneae	Araneidae	*Argiope bruennichi*	GBCH9690-13	KC194114
Araneae	Araneidae	*Argiope bruennichi*	GBCH9691-13	KC194113
Araneae	Araneidae	*Argiope bruennichi*	GBCH9692-13	KC194112
Araneae	Araneidae	*Argiope bruennichi*	GBCH9693-13	KC194111
Araneae	Araneidae	*Argiope bruennichi*	GBCH9694-13	KC194110
Araneae	Araneidae	*Argiope bruennichi*	GBCH9695-13	KC194109
Araneae	Araneidae	*Argiope bruennichi*	GBCH9696-13	KC194108
Araneae	Araneidae	*Argiope bruennichi*	GBCH9697-13	KC194107
Araneae	Araneidae	*Argiope bruennichi*	GBCH9698-13	KC194106
Araneae	Araneidae	*Argiope bruennichi*	GBCH9699-13	KC194105
Araneae	Araneidae	*Argiope bruennichi*	GBCH9700-13	KC194104
Araneae	Araneidae	*Argiope bruennichi*	GBCH9701-13	KC194103
Araneae	Araneidae	*Argiope bruennichi*	GBCH9702-13	KC194102
Araneae	Araneidae	*Argiope bruennichi*	GBCH9703-13	KC194101
Araneae	Araneidae	*Argiope bruennichi*	GBCH9704-13	KC194100
Araneae	Araneidae	*Argiope bruennichi*	GBCH9705-13	KC194099
Araneae	Araneidae	*Argiope bruennichi*	GBCH9706-13	KC194098
Araneae	Araneidae	*Argiope bruennichi*	GBCH9707-13	KC194097
Araneae	Araneidae	*Argiope bruennichi*	GBCH9708-13	KC194096
Araneae	Araneidae	*Argiope bruennichi*	GBCH9709-13	KC194095
Araneae	Araneidae	*Argiope bruennichi*	GBCH9710-13	KC194094
Araneae	Araneidae	*Argiope bruennichi*	GBCH9711-13	KC194093
Araneae	Araneidae	*Argiope bruennichi*	GBCH9712-13	KC194092
Araneae	Araneidae	*Argiope bruennichi*	GBCH9713-13	KC194091
Araneae	Araneidae	*Argiope bruennichi*	GBCH9714-13	KC194090

Araneae	Araneidae	*Argiope bruennichi*	GBCH9715-13	KC194089
Araneae	Araneidae	*Argiope bruennichi*	GBCH9716-13	KC194088
Araneae	Araneidae	*Argiope bruennichi*	GBCH9717-13	KC194087
Araneae	Araneidae	*Argiope bruennichi*	GBCH9718-13	KC194086
Araneae	Araneidae	*Argiope bruennichi*	GBCH9719-13	KC194085
Araneae	Araneidae	*Argiope bruennichi*	GBCH9720-13	KC194084
Araneae	Araneidae	*Argiope bruennichi*	GBCH9721-13	KC194083
Araneae	Araneidae	*Argiope bruennichi*	GBCH9722-13	KC194082
Araneae	Araneidae	*Argiope bruennichi*	GBCH9723-13	KC194081
Araneae	Araneidae	*Argiope bruennichi*	GBCH9724-13	KC194080
Araneae	Araneidae	*Argiope bruennichi*	GBCH9725-13	KC194079
Araneae	Araneidae	*Argiope bruennichi*	GBCH9726-13	KC194078
Araneae	Araneidae	*Argiope bruennichi*	GBCH9727-13	KC194077
Araneae	Araneidae	*Argiope bruennichi*	GBCH9728-13	KC194076
Araneae	Araneidae	*Argiope bruennichi*	GBCH9729-13	KC194075
Araneae	Araneidae	*Argiope bruennichi*	GBCH9730-13	KC194074
Araneae	Araneidae	*Argiope bruennichi*	GBCH9731-13	KC194073
Araneae	Araneidae	*Argiope bruennichi*	GBCH9732-13	KC194072
Araneae	Araneidae	*Argiope bruennichi*	GBCH9733-13	KC194071
Araneae	Araneidae	*Argiope bruennichi*	GBCH9734-13	KC194070
Araneae	Araneidae	*Argiope bruennichi*	GBCH9735-13	KC194069
Araneae	Araneidae	*Argiope bruennichi*	GBCH9736-13	KC194068
Araneae	Araneidae	*Argiope bruennichi*	GBCH9737-13	KC194067
Araneae	Araneidae	*Argiope bruennichi*	GBCH9738-13	KC194066
Araneae	Araneidae	*Argiope bruennichi*	GBCH9739-13	KC194065
Araneae	Araneidae	*Argiope bruennichi*	GBCH9740-13	KC194064
Araneae	Araneidae	*Argiope bruennichi*	GBCH9741-13	KC194063
Araneae	Araneidae	*Argiope bruennichi*	GBCH9742-13	KC194062
Araneae	Araneidae	*Argiope bruennichi*	GBCH9743-13	KC194061
Araneae	Araneidae	*Argiope bruennichi*	GBCH9744-13	KC194060
Araneae	Araneidae	*Argiope bruennichi*	GBCH9745-13	KC194059
Araneae	Araneidae	*Argiope bruennichi*	GBCH9746-13	KC194058
Araneae	Araneidae	*Argiope bruennichi*	GBCH9747-13	KC194057
Araneae	Araneidae	*Argiope bruennichi*	GBCH9748-13	KC194056
Araneae	Araneidae	*Argiope bruennichi*	GBCH9749-13	KC194055
Araneae	Araneidae	*Argiope bruennichi*	GBCH9750-13	KC194054
Araneae	Araneidae	*Argiope bruennichi*	GBCH9751-13	KC194053
Araneae	Araneidae	*Argiope bruennichi*	GBCH9752-13	KC194052
Araneae	Araneidae	*Argiope bruennichi*	GBCH9753-13	KC194051
Araneae	Araneidae	*Argiope bruennichi*	GBCH9754-13	KC194050
Araneae	Araneidae	*Argiope bruennichi*	GBCH9755-13	KC194049
Araneae	Araneidae	*Argiope bruennichi*	GBCH9756-13	KC194048
Araneae	Araneidae	*Argiope bruennichi*	GBCH9757-13	KC194047
Araneae	Araneidae	*Argiope bruennichi*	GBCH9758-13	KC194046
Araneae	Araneidae	*Argiope bruennichi*	GBCH9759-13	KC194045
Araneae	Araneidae	*Argiope bruennichi*	GBCH9760-13	KC194044
Araneae	Araneidae	*Argiope bruennichi*	GBCH9761-13	KC194043
Araneae	Araneidae	*Argiope bruennichi*	GBCH9762-13	KC194042
Araneae	Araneidae	*Argiope bruennichi*	GBCH9763-13	KC194041
Araneae	Araneidae	*Argiope bruennichi*	GBCH9764-13	KC194040
Araneae	Araneidae	*Argiope bruennichi*	GBCH9765-13	KC194039
Araneae	Araneidae	*Argiope bruennichi*	GBCH9766-13	KC194038
Araneae	Araneidae	*Argiope bruennichi*	GBCH9767-13	KC194037

Araneae	Araneidae	*Argiope bruennichi*	GBCH9768-13	KC194036
Araneae	Araneidae	*Argiope bruennichi*	GBCH9769-13	KC194035
Araneae	Araneidae	*Argiope bruennichi*	GBCH9770-13	KC194034
Araneae	Araneidae	*Argiope bruennichi*	GBCH9771-13	KC194033
Araneae	Araneidae	*Argiope bruennichi*	GBCH9772-13	KC194032
Araneae	Araneidae	*Argiope bruennichi*	GBCH9773-13	KC194031
Araneae	Araneidae	*Argiope bruennichi*	GBCH9774-13	KC194030
Araneae	Araneidae	*Argiope bruennichi*	GBCH9775-13	KC194029
Araneae	Araneidae	*Argiope bruennichi*	GBCH9776-13	KC194028
Araneae	Araneidae	*Argiope bruennichi*	GBCH9777-13	KC194027
Araneae	Araneidae	*Argiope bruennichi*	GBCH9778-13	KC194026
Araneae	Araneidae	*Argiope bruennichi*	GBCH9779-13	KC194025
Araneae	Araneidae	*Argiope bruennichi*	GBCH9780-13	KC194024
Araneae	Araneidae	*Argiope bruennichi*	GBCH9781-13	KC194023
Araneae	Araneidae	*Argiope bruennichi*	GBCH9782-13	KC194022
Araneae	Araneidae	*Argiope bruennichi*	GBCH9783-13	KC194021
Araneae	Araneidae	*Argiope bruennichi*	GBCH9784-13	KC194020
Araneae	Araneidae	*Argiope bruennichi*	GBCH9785-13	KC194019
Araneae	Araneidae	*Argiope bruennichi*	GBCH9786-13	KC194018
Araneae	Araneidae	*Argiope bruennichi*	GBCH9787-13	KC194017
Araneae	Araneidae	*Argiope bruennichi*	GBCH9788-13	KC194016
Araneae	Araneidae	*Argiope bruennichi*	GBCH9789-13	KC194015
Araneae	Araneidae	*Argiope bruennichi*	GBCH9790-13	KC194014
Araneae	Araneidae	*Argiope bruennichi*	GBCH9791-13	KC194013
Araneae	Araneidae	*Argiope bruennichi*	GBCH9792-13	KC194012
Araneae	Araneidae	*Argiope bruennichi*	GBCH9793-13	KC194011
Araneae	Araneidae	*Argiope bruennichi*	GBCH9794-13	KC194010
Araneae	Araneidae	*Argiope bruennichi*	GBCH9795-13	KC194009
Araneae	Araneidae	*Argiope bruennichi*	GBCH9796-13	KC194008
Araneae	Araneidae	*Argiope bruennichi*	GBCH9797-13	KC194007
Araneae	Araneidae	*Argiope bruennichi*	GBCH9798-13	KC194006
Araneae	Araneidae	*Argiope bruennichi*	GBCH9799-13	KC194005
Araneae	Araneidae	*Argiope bruennichi*	GBCH9800-13	KC194004
Araneae	Araneidae	*Argiope bruennichi*	GBCH9801-13	KC194003
Araneae	Araneidae	*Argiope bruennichi*	GBCH9802-13	KC194002
Araneae	Araneidae	*Argiope bruennichi*	GBCH9803-13	KC194001
Araneae	Araneidae	*Argiope bruennichi*	GBCH9804-13	KC194000
Araneae	Araneidae	*Argiope bruennichi*	GBCH9805-13	KC193999
Araneae	Araneidae	*Argiope bruennichi*	GBCH9806-13	KC193998
Araneae	Araneidae	*Argiope bruennichi*	GBCH9807-13	KC193997
Araneae	Araneidae	*Argiope bruennichi*	GBCH9808-13	KC193996
Araneae	Araneidae	*Argiope bruennichi*	GBCH9809-13	KC193995
Araneae	Araneidae	*Argiope bruennichi*	GBCH9810-13	KC193994
Araneae	Araneidae	*Argiope bruennichi*	GBCH9811-13	KC193993
Araneae	Araneidae	*Argiope bruennichi*	GBCH9812-13	KC193992
Araneae	Araneidae	*Argiope bruennichi*	GBCH9813-13	KC193991
Araneae	Araneidae	*Argiope bruennichi*	GBCH9814-13	KC193990
Araneae	Araneidae	*Argiope bruennichi*	GBCH9815-13	KC193989
Araneae	Araneidae	*Argiope bruennichi*	GBCH9816-13	KC193988
Araneae	Araneidae	*Argiope bruennichi*	GBCH9817-13	KC193987
Araneae	Araneidae	*Argiope bruennichi*	GBCH9818-13	KC193986
Araneae	Araneidae	*Argiope bruennichi*	GBCH9819-13	KC193985
Araneae	Araneidae	*Argiope bruennichi*	GBCH9820-13	KC193984

Araneae	Araneidae	*Argiope bruennichi*	GBCH9821-13	KC193983
Araneae	Araneidae	*Argiope bruennichi*	GBCH9822-13	KC193982
Araneae	Araneidae	*Argiope bruennichi*	GBCH9823-13	KC193981
Araneae	Araneidae	*Argiope bruennichi*	GBCH9824-13	KC193980
Araneae	Araneidae	*Argiope bruennichi*	GBCH9825-13	KC193979
Araneae	Araneidae	*Argiope bruennichi*	GBCH9826-13	KC193978
Araneae	Araneidae	*Argiope bruennichi*	GBCH9827-13	KC193977
Araneae	Araneidae	*Argiope bruennichi*	GBCH9828-13	KC193976
Araneae	Araneidae	*Argiope bruennichi*	GBCH9829-13	KC193975
Araneae	Araneidae	*Argiope bruennichi*	GBCH9830-13	KC193974
Araneae	Araneidae	*Argiope bruennichi*	GBCH9831-13	KC193973
Araneae	Araneidae	*Argiope bruennichi*	GBCH9832-13	KC193972
Araneae	Araneidae	*Argiope bruennichi*	GBCH9833-13	KC193971
Araneae	Araneidae	*Argiope bruennichi*	GBCH9834-13	KC193970
Araneae	Araneidae	*Argiope bruennichi*	GBCH9835-13	KC193969
Araneae	Araneidae	*Argiope bruennichi*	GBCH9836-13	KC193968
Araneae	Araneidae	*Argiope bruennichi*	GBCH9837-13	KC193967
Araneae	Araneidae	*Argiope bruennichi*	GBCH9838-13	KC193966
Araneae	Araneidae	*Argiope bruennichi*	GBCH9839-13	KC193965
Araneae	Araneidae	*Argiope bruennichi*	GBCH9840-13	KC193964
Araneae	Araneidae	*Argiope bruennichi*	GBCH9841-13	KC193963
Araneae	Araneidae	*Argiope bruennichi*	GBCH9842-13	KC193962
Araneae	Araneidae	*Argiope bruennichi*	GBCH9843-13	KC193961
Araneae	Araneidae	*Argiope bruennichi*	GBCH9844-13	KC193960
Araneae	Araneidae	*Argiope bruennichi*	GBCH9845-13	KC193959
Araneae	Araneidae	*Argiope bruennichi*	GBCH9846-13	KC193958
Araneae	Araneidae	*Argiope bruennichi*	GBCH9847-13	KC193957
Araneae	Araneidae	*Argiope bruennichi*	GBCH9848-13	KC193956
Araneae	Araneidae	*Argiope bruennichi*	GBCH9849-13	KC193955
Araneae	Araneidae	*Argiope bruennichi*	GBCH9850-13	KC193954
Araneae	Araneidae	*Argiope bruennichi*	GBCH9851-13	KC193953
Araneae	Araneidae	*Argiope bruennichi*	GBCH9852-13	KC193952
Araneae	Araneidae	*Argiope bruennichi*	GBCH9853-13	KC193951
Araneae	Araneidae	*Argiope bruennichi*	GBCH9854-13	KC193950
Araneae	Araneidae	*Argiope bruennichi*	GBCH9855-13	KC193949
Araneae	Araneidae	*Argiope bruennichi*	GBCH9856-13	KC193948
Araneae	Araneidae	*Argiope bruennichi*	GBCH9857-13	KC193947
Araneae	Araneidae	*Argiope bruennichi*	GBCH9858-13	KC193946
Araneae	Araneidae	*Argiope bruennichi*	GBCH9859-13	KC193945
Araneae	Araneidae	*Argiope bruennichi*	GBCH9860-13	KC193944
Araneae	Araneidae	*Argiope bruennichi*	GBCH9861-13	KC193943
Araneae	Araneidae	*Argiope bruennichi*	GBCH9862-13	KC193942
Araneae	Araneidae	*Argiope bruennichi*	GBCH9863-13	KC193941
Araneae	Araneidae	*Argiope bruennichi*	GBCH9864-13	KC193940
Araneae	Araneidae	*Argiope bruennichi*	GBCH9865-13	KC193939
Araneae	Araneidae	*Argiope bruennichi*	GBCH9866-13	KC193938
Araneae	Araneidae	*Argiope bruennichi*	GBCH9867-13	KC193937
Araneae	Araneidae	*Argiope bruennichi*	GBCH9868-13	KC193936
Araneae	Araneidae	*Argiope bruennichi*	GBCH9869-13	KC193935
Araneae	Araneidae	*Argiope bruennichi*	GBCH9870-13	KC193934
Araneae	Araneidae	*Argiope bruennichi*	GBCH9871-13	KC193933
Araneae	Araneidae	*Argiope bruennichi*	GBCH9872-13	KC193932
Araneae	Araneidae	*Argiope bruennichi*	GBCH9873-13	KC193931

Araneae	Araneidae	*Argiope bruennichi*	GBCH9874-13	KC193930
Araneae	Araneidae	*Argiope bruennichi*	GBCH9875-13	KC193929
Araneae	Araneidae	*Argiope bruennichi*	GBCH9876-13	KC193928
Araneae	Araneidae	*Argiope bruennichi*	GBCH9877-13	KC193927
Araneae	Araneidae	*Argiope bruennichi*	GBCH9878-13	KC193926
Araneae	Araneidae	*Argiope bruennichi*	GBCH9879-13	KC193925
Araneae	Araneidae	*Argiope bruennichi*	GBCH9880-13	KC193924
Araneae	Araneidae	*Argiope bruennichi*	GBCH9881-13	KC193923
Araneae	Araneidae	*Argiope bruennichi*	GBCH9882-13	KC193922
Araneae	Araneidae	*Argiope bruennichi*	GBCH9883-13	KC193921
Araneae	Araneidae	*Argiope bruennichi*	GBCH9884-13	KC193920
Araneae	Araneidae	*Argiope bruennichi*	GBCH9885-13	KC193919
Araneae	Araneidae	*Argiope bruennichi*	GBCH9886-13	KC193918
Araneae	Araneidae	*Argiope bruennichi*	GBCH9887-13	KC193917
Araneae	Araneidae	*Argiope bruennichi*	GBCH9888-13	KC193916
Araneae	Araneidae	*Argiope bruennichi*	GBCH9889-13	KC193915
Araneae	Araneidae	*Argiope bruennichi*	GBCH9890-13	KC193914
Araneae	Araneidae	*Argiope bruennichi*	GBCH9891-13	KC193913
Araneae	Araneidae	*Argiope bruennichi*	GBCH9892-13	KC193912
Araneae	Araneidae	*Argiope bruennichi*	GBCH9893-13	KC193911
Araneae	Araneidae	*Argiope bruennichi*	GBCH9894-13	KC193910
Araneae	Araneidae	*Argiope bruennichi*	GBCH9895-13	KC193909
Araneae	Araneidae	*Argiope bruennichi*	GBCH9896-13	KC193908
Araneae	Araneidae	*Argiope bruennichi*	GBCH9897-13	KC193907
Araneae	Araneidae	*Argiope bruennichi*	GBCH9898-13	KC193906
Araneae	Araneidae	*Argiope bruennichi*	GBCH9899-13	KC193905
Araneae	Araneidae	*Argiope bruennichi*	GBCH9900-13	KC193904
Araneae	Araneidae	*Argiope bruennichi*	GBCH9901-13	KC193903
Araneae	Araneidae	*Argiope bruennichi*	GBCH9902-13	KC193902
Araneae	Araneidae	*Argiope bruennichi*	GBCH9903-13	KC193901
Araneae	Araneidae	*Argiope bruennichi*	GBCH9904-13	KC193900
Araneae	Araneidae	*Argiope bruennichi*	GBCH9905-13	KC193899
Araneae	Araneidae	*Argiope bruennichi*	GBCH9906-13	KC193898
Araneae	Araneidae	*Argiope bruennichi*	GBCH9907-13	KC193897
Araneae	Araneidae	*Argiope bruennichi*	GBCH9908-13	KC193896
Araneae	Araneidae	*Argiope bruennichi*	GBCH9909-13	KC193895
Araneae	Araneidae	*Argiope bruennichi*	GBCH9910-13	KC193894
Araneae	Araneidae	*Argiope bruennichi*	GBCH9911-13	KC193893
Araneae	Araneidae	*Argiope bruennichi*	GBCH9912-13	KC193892
Araneae	Araneidae	*Argiope bruennichi*	GBCH9913-13	KC193891
Araneae	Araneidae	*Argiope bruennichi*	GBCH9914-13	KC193890
Araneae	Araneidae	*Argiope bruennichi*	GBCH9915-13	KC193889
Araneae	Araneidae	*Argiope bruennichi*	GBCH9916-13	KC193888
Araneae	Araneidae	*Argiope bruennichi*	GBCH9917-13	KC193887
Araneae	Araneidae	*Argiope bruennichi*	GBCH9918-13	KC193886
Araneae	Araneidae	*Argiope bruennichi*	GBCH9919-13	KC193885
Araneae	Araneidae	*Argiope bruennichi*	GBCH9920-13	KC193884
Araneae	Araneidae	*Argiope bruennichi*	GBCH9921-13	KC193883
Araneae	Araneidae	*Argiope bruennichi*	GBCH9922-13	KC193882
Araneae	Araneidae	*Argiope bruennichi*	GBCH9923-13	KC193881
Araneae	Araneidae	*Argiope bruennichi*	GBCH9924-13	KC193880
Araneae	Araneidae	*Argiope bruennichi*	GBCH9925-13	KC193879
Araneae	Araneidae	*Argiope bruennichi*	GBCH9926-13	KC193878

Araneae	Araneidae	*Argiope bruennichi*	GBCH9927-13	KC193877
Araneae	Araneidae	*Argiope bruennichi*	GBCH9928-13	KC193876
Araneae	Araneidae	*Argiope bruennichi*	GBCH9929-13	KC193875
Araneae	Araneidae	*Argiope bruennichi*	GBCH9930-13	KC193874
Araneae	Araneidae	*Argiope bruennichi*	GBCH9931-13	KC193873
Araneae	Araneidae	*Argiope bruennichi*	GBCH9932-13	KC193872
Araneae	Araneidae	*Argiope bruennichi*	GBCH9933-13	KC193871
Araneae	Araneidae	*Argiope bruennichi*	GBCH9934-13	KC193870
Araneae	Araneidae	*Argiope bruennichi*	GBCH9935-13	KC193869
Araneae	Araneidae	*Argiope bruennichi*	GBCH9936-13	KC193868
Araneae	Araneidae	*Argiope bruennichi*	GBCH9937-13	KC193867
Araneae	Araneidae	*Argiope bruennichi*	GBCH9938-13	KC193866
Araneae	Araneidae	*Argiope bruennichi*	GBCH9939-13	KC193865
Araneae	Araneidae	*Argiope bruennichi*	GBCH9940-13	KC193864
Araneae	Araneidae	*Argiope bruennichi*	GBCH9941-13	KC193863
Araneae	Araneidae	*Argiope bruennichi*	GBCH9942-13	KC193862
Araneae	Araneidae	*Argiope bruennichi*	GBCH9943-13	KC193861
Araneae	Araneidae	*Argiope bruennichi*	GBCH9944-13	KC193860
Araneae	Araneidae	*Argiope bruennichi*	GBCH9945-13	KC193859
Araneae	Araneidae	*Argiope bruennichi*	GBCH9946-13	KC193858
Araneae	Araneidae	*Argiope bruennichi*	GBCH9947-13	KC193857
Araneae	Araneidae	*Argiope bruennichi*	GBCH9948-13	KC193856
Araneae	Araneidae	*Argiope bruennichi*	GBCH9949-13	KC193855
Araneae	Araneidae	*Argiope bruennichi*	GBCH9950-13	KC193854
Araneae	Araneidae	*Argiope bruennichi*	GBCH9951-13	KC193853
Araneae	Araneidae	*Argiope bruennichi*	GBCH9952-13	KC193852
Araneae	Araneidae	*Argiope bruennichi*	GBCH9953-13	KC193851
Araneae	Araneidae	*Argiope bruennichi*	GBCH9954-13	KC193850
Araneae	Araneidae	*Argiope bruennichi*	GBCH9955-13	KC193849
Araneae	Araneidae	*Argiope bruennichi*	GBCH9956-13	KC193848
Araneae	Araneidae	*Argiope bruennichi*	GBCH9957-13	KC193847
Araneae	Araneidae	*Argiope bruennichi*	GBCH9958-13	KC193846
Araneae	Araneidae	*Argiope bruennichi*	GBCH9959-13	KC193845
Araneae	Araneidae	*Argiope bruennichi*	GBCH9960-13	KC193844
Araneae	Araneidae	*Argiope bruennichi*	GBCH9961-13	KC193843
Araneae	Araneidae	*Argiope bruennichi*	GBCH9962-13	KC193842
Araneae	Araneidae	*Argiope bruennichi*	GBCH9963-13	KC193841
Araneae	Araneidae	*Argiope bruennichi*	GBCH9964-13	KC193840
Araneae	Araneidae	*Argiope bruennichi*	GBCH9965-13	KC193839
Araneae	Araneidae	*Argiope bruennichi*	GBCH9966-13	KC193838
Araneae	Araneidae	*Argiope bruennichi*	GBCH9967-13	KC193837
Araneae	Araneidae	*Argiope bruennichi*	GBCH9968-13	KC193836
Araneae	Araneidae	*Argiope bruennichi*	GBCH9969-13	KC193835
Araneae	Araneidae	*Argiope bruennichi*	GBCH9970-13	KC193834
Araneae	Araneidae	*Argiope bruennichi*	GBCH9971-13	KC193833
Araneae	Araneidae	*Argiope bruennichi*	GBCH9972-13	KC193832
Araneae	Araneidae	*Argiope bruennichi*	GBCH9973-13	KC193831
Araneae	Araneidae	*Argiope bruennichi*	GBCH9974-13	KC193830
Araneae	Araneidae	*Argiope bruennichi*	GBCH9975-13	KC193829
Araneae	Araneidae	*Argiope bruennichi*	GBCH9976-13	KC193828
Araneae	Araneidae	*Argiope bruennichi*	GBCH9977-13	KC193827
Araneae	Araneidae	*Argiope bruennichi*	GBCH9978-13	KC193826
Araneae	Araneidae	*Argiope bruennichi*	GBCH9979-13	KC193825

Araneae	Araneidae	*Argiope bruennichi*	GBCH9980-13	KC193824
Araneae	Araneidae	*Argiope bruennichi*	GBCH9981-13	KC193823
Araneae	Araneidae	*Argiope bruennichi*	GBCH9982-13	KC193822
Araneae	Araneidae	*Argiope bruennichi*	GBCH9983-13	KC193821
Araneae	Araneidae	*Argiope bruennichi*	GBCH9984-13	KC193820
Araneae	Araneidae	*Argiope bruennichi*	GBCH9985-13	KC193819
Araneae	Araneidae	*Argiope bruennichi*	GBCH9986-13	KC193818
Araneae	Araneidae	*Argiope bruennichi*	GBCH9987-13	KC193817
Araneae	Araneidae	*Argiope bruennichi*	GBCH9988-13	KC193816
Araneae	Araneidae	*Argiope bruennichi*	GBCH9989-13	KC193815
Araneae	Araneidae	*Argiope bruennichi*	GBCH9990-13	KC193814
Araneae	Araneidae	*Argiope bruennichi*	GBCH9991-13	KC193813
Araneae	Araneidae	*Argiope bruennichi*	GBCH9992-13	KC193812
Araneae	Araneidae	*Argiope bruennichi*	GBCH9993-13	KC193811
Araneae	Araneidae	*Argiope bruennichi*	GBCH9994-13	KC193810
Araneae	Araneidae	*Argiope bruennichi*	GBCH9995-13	KC193809
Araneae	Araneidae	*Argiope bruennichi*	GBCH9996-13	KC193808
Araneae	Araneidae	*Argiope bruennichi*	GBCH9997-13	KC193807
Araneae	Araneidae	*Argiope bruennichi*	GBCH9998-13	KC193806
Araneae	Araneidae	*Argiope bruennichi*	GBCH9999-13	KC193805
Araneae	Araneidae	*Argiope dang*	GBCH11500-13	AB753452
Araneae	Araneidae	*Argiope mangal*	GBCH5091-10	GU353205
Araneae	Araneidae	*Argiope minuta*	GBCH11111-13	JN817159
Araneae	Araneidae	*Argiope submaronica*	GBCH3914-09	EU003279
Araneae	Araneidae	*Argiope trifasciata*	ARONT033-09	GU682759
Araneae	Araneidae	*Argiope trifasciata*	ARONT154-09	GU682572
Araneae	Araneidae	*Argiope trifasciata*	ARONT308-09	GU682741
Araneae	Araneidae	*Argiope trifasciata*	ARONT355-09	GU682570
Araneae	Araneidae	*Argiope trifasciata*	ARONT356-09	GU682739
Araneae	Araneidae	*Argiope trifasciata*	ARONT813-10	HQ924658
Araneae	Araneidae	*Argiope trifasciata*	ARONT814-10	HQ924659
Araneae	Araneidae	*Argiope trifasciata*	GBCH3248-09	FJ525316
Araneae	Araneidae	*Argiope trifasciata*	GBCH5179-10	EU271659
Araneae	Araneidae	*Argiope trifasciata*	GBCH5181-10	EU271653
Araneae	Araneidae	*Argiope trifasciata*	RBCH159-04	DQ127369
Araneae	Araneidae	*Argiope trifasciata*	RBCH193-04	DQ127339
Araneae	Araneidae	*Argiope trifasciata*	RBINA3334-13	KP655435
Araneae	Araneidae	*Argiope trifasciata*	RBINA721-13	KP650224
Araneae	Araneidae	*Argiope trifasciata*	SPRMA020-10	HQ977127
Araneae	Araneidae	*Arkys cornutus*	GBCH4043-09	FJ607556
Araneae	Araneidae	*Backobourkia brouni*	GBCH5079-10	FJ873122
Araneae	Araneidae	*Backobourkia brouni*	GBCH5080-10	FJ873121
Araneae	Araneidae	*Backobourkia brouni*	GBCH5081-10	FJ873120
Araneae	Araneidae	*Backobourkia collina*	GBCH5077-10	FJ873124
Araneae	Araneidae	*Backobourkia collina*	GBCH5078-10	FJ873123
Araneae	Araneidae	*Backobourkia heroine*	GBCH5075-10	FJ873126
Araneae	Araneidae	*Chorizopes nipponicus*	GBCH11105-13	JN817165
Araneae	Araneidae	*Cyclosa alba*	GBCH1211-06	DQ146864
Araneae	Araneidae	*Cyclosa alba*	GBCH4108-09	AB453390
Araneae	Araneidae	*Cyclosa alba*	GBCH4109-09	AB453391
Araneae	Araneidae	*Cyclosa alba*	GBCH4110-09	AB453392
Araneae	Araneidae	*Cyclosa alba*	GBCH4111-09	AB453393
Araneae	Araneidae	*Cyclosa alba*	GBCH4112-09	AB453394

Araneae	Araneidae	*Cyclosa alba*	GBCH4113-09	AB453395
Araneae	Araneidae	*Cyclosa argenteoalba*	GBCH11097-13	JN817173
Araneae	Araneidae	*Cyclosa argenteoalba*	GBCH11098-13	JN817172
Araneae	Araneidae	*Cyclosa conica*	ARONT203-09	GU682717
Araneae	Araneidae	*Cyclosa conica*	ARONT204-09	GU682718
Araneae	Araneidae	*Cyclosa conica*	ARONT651-10	HQ924516
Araneae	Araneidae	*Cyclosa conica*	BBCAN977-10	HQ928191
Araneae	Araneidae	*Cyclosa conica*	ERSPI322-09	GU682513
Araneae	Araneidae	*Cyclosa conica*	GBCH1230-06	DQ146883
Araneae	Araneidae	*Cyclosa conica*	GBCH3911-09	EU003282
Araneae	Araneidae	*Cyclosa conica*	RBCH052-04	DQ127484
Araneae	Araneidae	*Cyclosa conica*	RBCH053-04	DQ127469
Araneae	Araneidae	*Cyclosa conica*	RBCH057-04	DQ127473
Araneae	Araneidae	*Cyclosa conica*	RBCH058-04	DQ127474
Araneae	Araneidae	*Cyclosa conica*	RBCH184-04	DQ127346
Araneae	Araneidae	*Cyclosa conica*	SPRMA138-10	HQ580767
Araneae	Araneidae	*Cyclosa conica*	SPRMA365-10	HQ580915
Araneae	Araneidae	*Cyclosa conica*	SPRMA681-10	KP655120
Araneae	Araneidae	*Cyclosa japonica*	GBCH11099-13	JN817171
Araneae	Araneidae	*Cyclosa laticauda*	GBCH11101-13	JN817169
Araneae	Araneidae	*Cyclosa monticola*	GBCH11102-13	JN817168
Araneae	Araneidae	*Cyclosa mulmeinensis*	GBCH1233-06	DQ146886
Araneae	Araneidae	*Cyclosa mulmeinensis*	GBCH4117-09	AB453766
Araneae	Araneidae	*Cyclosa mulmeinensis*	GBCH4118-09	AB453767
Araneae	Araneidae	*Cyclosa mulmeinensis*	GBCH4119-09	AB453768
Araneae	Araneidae	*Cyclosa mulmeinensis*	GBCH4120-09	AB453769
Araneae	Araneidae	*Cyclosa octotuberculata*	GBCH11103-13	JN817167
Araneae	Araneidae	*Cyrtophora moluccensis*	GBCH4039-09	FJ607560
Araneae	Araneidae	*Eriophora astridae*	GBCH11094-13	JN817176
Araneae	Araneidae	*Eriophora pustulosa*	GBCH5074-10	FJ873127
Araneae	Araneidae	*Eriophora sachalinensis*	GBCH11095-13	JN817175
Araneae	Araneidae	*Eustala anastera*	ARONT709-10	HQ924566
Araneae	Araneidae	*Eustala anastera*	ERSPI316-09	GU682521
Araneae	Araneidae	*Eustala anastera*	ERSPI317-09	GU682522
Araneae	Araneidae	*Eustala anastera*	ERSPI319-09	GU682511
Araneae	Araneidae	*Eustala anastera*	ERSPI320-09	GU682512
Araneae	Araneidae	*Eustala anastera*	PHMTV444-10	KP645751
Araneae	Araneidae	*Eustala anastera*	RBCH155-04	DQ127381
Araneae	Araneidae	*Eustala anastera*	RBCH194-04	DQ127340
Araneae	Araneidae	*Eustala anastera*	RBINA1848-13	KP647599
Araneae	Araneidae	*Eustala anastera*	RBINA2675-13	KP646262
Araneae	Araneidae	*Eustala anastera*	RBINA3463-13	KP647058
Araneae	Araneidae	*Eustala anastera*	RBINA3973-13	KP656450
Araneae	Araneidae	*Eustala anastera*	SMTPB17030-13	KP657257
Araneae	Araneidae	*Eustala anastera*	SMTPB20152-13	KP652857
Araneae	Araneidae	*Eustala cepina*	CNPPC1917-12	KJ090437
Araneae	Araneidae	*Eustala cepina*	CNPPC1918-12	KJ163901
Araneae	Araneidae	*Eustala cepina*	CNPPD2595-12	KJ086756
Araneae	Araneidae	*Eustala cepina*	CNPPD2688-12	KJ089496
Araneae	Araneidae	*Eustala cepina*	CNPPD2691-12	KJ090677
Araneae	Araneidae	*Eustala cepina*	CNPPD2693-12	KJ087086
Araneae	Araneidae	*Eustala cepina*	CNPPD2696-12	KJ167052
Araneae	Araneidae	*Eustala cepina*	CNPPD2703-12	KJ089447

Araneae	Araneidae	*Eustala cepina*	CNPPE2324-12	KJ092225
Araneae	Araneidae	*Eustala cepina*	CNPPE2325-12	KJ086613
Araneae	Araneidae	*Eustala cepina*	CNPPF1451-12	KJ167806
Araneae	Araneidae	*Eustala cepina*	CNPPJ1368-12	KJ208382
Araneae	Araneidae	*Eustala cepina*	ERSPI318-09	HM376104
Araneae	Araneidae	*Eustala cepina*	RBINA2667-13	KP655864
Araneae	Araneidae	*Eustala emertoni*	PHMTU094-10	KP648805
Araneae	Araneidae	*Eustala rosae*	CNPEJ231-14	KP646328
Araneae	Araneidae	*Eustala rosae*	CNPEM2402-14	KP646150
Araneae	Araneidae	*Eustala sp. 1GAB*	TDWGB839-10	HQ979264
Araneae	Araneidae	*Eustala sp. TAB-2009*	GBCH3244-09	FJ525320
Araneae	Araneidae	*Gasteracantha cancriformis*	GBCH3243-09	FJ525321
Araneae	Araneidae	*Gasteracantha cancriformis*	GBCH3906-09	EU003287
Araneae	Araneidae	*Gasteracantha kuhli*	GBCH11106-13	JN817164
Araneae	Araneidae	*Gasteracantha sp.*	RBCH176-04	DQ127354
Araneae	Araneidae	*Gea heptagon*	ARONT414-09	GU682951
Araneae	Araneidae	*Gea heptagon*	ERSPI287-09	GU682541
Araneae	Araneidae	*Gea heptagon*	RBINA1868-13	KP645799
Araneae	Araneidae	*Hypsosinga pygmaea*	ARONT501-10	KP650815
Araneae	Araneidae	*Hypsosinga pygmaea*	ARONT502-10	HQ924396
Araneae	Araneidae	*Hypsosinga pygmaea*	ARONT503-10	HQ924397
Araneae	Araneidae	*Hypsosinga pygmaea*	ARONT504-10	HQ924398
Araneae	Araneidae	*Hypsosinga pygmaea*	ARONT505-10	HQ924399
Araneae	Araneidae	*Hypsosinga pygmaea*	GBADC048-10	HQ956709
Araneae	Araneidae	*Hypsosinga pygmaea*	RBCH069-04	DQ127453
Araneae	Araneidae	*Larinioides cornutus*	ARONT290-09	GU682743
Araneae	Araneidae	*Larinioides cornutus*	ARONT366-09	GU682745
Araneae	Araneidae	*Larinioides cornutus*	ARONT367-09	GU682740
Araneae	Araneidae	*Larinioides cornutus*	ARONT368-09	GU682742
Araneae	Araneidae	*Larinioides cornutus*	CNEIC3176-13	KM836774
Araneae	Araneidae	*Larinioides cornutus*	CNEIE1945-13	KM833917
Araneae	Araneidae	*Larinioides cornutus*	CNEIF2254-12	KM839848
Araneae	Araneidae	*Larinioides cornutus*	CNEIF2258-12	KM824994
Araneae	Araneidae	*Larinioides cornutus*	CNEIF2260-12	KM834040
Araneae	Araneidae	*Larinioides cornutus*	CNEIF2265-12	KM825731
Araneae	Araneidae	*Larinioides cornutus*	CNEIF2268-12	KM839081
Araneae	Araneidae	*Larinioides cornutus*	CNEIF2269-12	KM829273
Araneae	Araneidae	*Larinioides cornutus*	CNEIF2376-12	KM830117
Araneae	Araneidae	*Larinioides cornutus*	CNEIG013-12	KM824970
Araneae	Araneidae	*Larinioides cornutus*	CNEIG1482-13	KM827936
Araneae	Araneidae	*Larinioides cornutus*	CNEIH024-13	KM824340
Araneae	Araneidae	*Larinioides cornutus*	CNEIH028-13	KM840332
Araneae	Araneidae	*Larinioides cornutus*	CNPPA4064-12	KJ087001
Araneae	Araneidae	*Larinioides cornutus*	CNPPC1915-12	KJ083907
Araneae	Araneidae	*Larinioides cornutus*	CNPPE2349-12	KJ163844
Araneae	Araneidae	*Larinioides cornutus*	CNPPF1447-12	KJ163279
Araneae	Araneidae	*Larinioides cornutus*	CNPPF1454-12	KJ166394
Araneae	Araneidae	*Larinioides cornutus*	CNPPG910-12	KJ166491
Araneae	Araneidae	*Larinioides cornutus*	GBCH11125-13	JN817145
Araneae	Araneidae	*Larinioides cornutus*	GBCH3242-09	FJ525322
Araneae	Araneidae	*Larinioides cornutus*	GBCH3904-09	EU003289
Araneae	Araneidae	*Larinioides cornutus*	MIONB473-11	KM833579
Araneae	Araneidae	*Larinioides cornutus*	RBCH108-04	DQ127428

Araneae	Araneidae	*Larinioides cornutus*	RBCH130-04	DQ127402
Araneae	Araneidae	*Larinioides cornutus*	RBCH131-04	DQ127403
Araneae	Araneidae	*Larinioides cornutus*	RBINA1235-13	KP646576
Araneae	Araneidae	*Larinioides cornutus*	RBINA5817-13	KP645580
Araneae	Araneidae	*Larinioides patagiatus*	ARONT291-09	GU682746
Araneae	Araneidae	*Larinioides patagiatus*	ARONT292-09	GU682744
Araneae	Araneidae	*Larinioides patagiatus*	ARONT293-09	GU682747
Araneae	Araneidae	*Larinioides patagiatus*	ARONT310-09	GU682748
Araneae	Araneidae	*Larinioides patagiatus*	ARONT369-09	GU682749
Araneae	Araneidae	*Larinioides patagiatus*	CNEIA2652-13	KM836701
Araneae	Araneidae	*Larinioides patagiatus*	CNEID3469-13	KM834019
Araneae	Araneidae	*Larinioides patagiatus*	CNEIF2251-12	KM826767
Araneae	Araneidae	*Larinioides patagiatus*	CNEIF2252-12	KM829445
Araneae	Araneidae	*Larinioides patagiatus*	CNEIF2253-12	KM832967
Araneae	Araneidae	*Larinioides patagiatus*	CNEIF2257-12	KM834914
Araneae	Araneidae	*Larinioides patagiatus*	CNEIF2259-12	KM836107
Araneae	Araneidae	*Larinioides patagiatus*	CNEIG014-12	KM829864
Araneae	Araneidae	*Larinioides patagiatus*	CNPCE072-12	KM839150
Araneae	Araneidae	*Larinioides patagiatus*	CNPPD2597-12	KJ086285
Araneae	Araneidae	*Larinioides patagiatus*	CNPPF1452-12	KJ167035
Araneae	Araneidae	*Larinioides patagiatus*	CNPPF1453-12	KJ164403
Araneae	Araneidae	*Larinioides patagiatus*	CNPPF1462-12	KJ163417
Araneae	Araneidae	*Larinioides patagiatus*	CNWLF010-12	KM827769
Araneae	Araneidae	*Larinioides patagiatus*	CNWLG001-12	KM839000
Araneae	Araneidae	*Larinioides patagiatus*	CNWLH003-12	KM827533
Araneae	Araneidae	*Larinioides patagiatus*	CNWLJ023-12	KM830225
Araneae	Araneidae	*Larinioides patagiatus*	JSARA052-11	KP654002
Araneae	Araneidae	*Larinioides patagiatus*	RBCH001-04	DQ127524
Araneae	Araneidae	*Larinioides patagiatus*	RBCH132-04	DQ127404
Araneae	Araneidae	*Larinioides patagiatus*	SPICH724-09	GU684356
Araneae	Araneidae	*Larinioides patagiatus*	SPRMA021-10	HQ977128
Araneae	Araneidae	*Larinioides sclopetarius*	ARONT074-09	GU682768
Araneae	Araneidae	*Larinioides sclopetarius*	ARONT075-09	GU682765
Araneae	Araneidae	*Larinioides sclopetarius*	ARONT076-09	GU682766
Araneae	Araneidae	*Larinioides sclopetarius*	ARONT077-09	GU682763
Araneae	Araneidae	*Larinioides sclopetarius*	ARONT285-09	GU682839
Araneae	Araneidae	*Larinioides sclopetarius*	ARONT286-09	GU682840
Araneae	Araneidae	*Larinioides sclopetarius*	ARONT287-09	GU682837
Araneae	Araneidae	*Larinioides sclopetarius*	ARONT288-09	GU682838
Araneae	Araneidae	*Larinioides sclopetarius*	ARONT289-09	GU682756
Araneae	Araneidae	*Larinioides sclopetarius*	ARONT323-09	GU682757
Araneae	Araneidae	*Larinioides sclopetarius*	ARONT335-09	GU682750
Araneae	Araneidae	*Larinioides sclopetarius*	ARONT336-09	GU682754
Araneae	Araneidae	*Larinioides sclopetarius*	ARONT337-09	GU682755
Araneae	Araneidae	*Larinioides sclopetarius*	ARONT347-09	GU682752
Araneae	Araneidae	*Larinioides sclopetarius*	ARONT364-09	GU682751
Araneae	Araneidae	*Larinioides sclopetarius*	ARONT365-09	GU682753
Araneae	Araneidae	*Larinioides sclopetarius*	ERSPI412-09	GU682443
Araneae	Araneidae	*Larinioides sclopetarius*	ERSPI414-09	GU682444
Araneae	Araneidae	*Larinioides sclopetarius*	ERSPI415-09	GU682437
Araneae	Araneidae	*Larinioides sclopetarius*	ERSPI416-09	GU682438
Araneae	Araneidae	*Larinioides sclopetarius*	GBCH0809-06	AY560799
Araneae	Araneidae	*Larinioides sclopetarius*	GBCH11124-13	JN817146

Araneae	Araneidae	*Larinioides sclopetarius*	RBCH059-04	DQ127475
Araneae	Araneidae	*Larinioides sclopetarius*	RBCH204-04	DQ127334
Araneae	Araneidae	*Larinioides sclopetarius*	SMTPB5988-13	KP657219
Araneae	Araneidae	*Mangora gibberosa*	ARONT186-09	GU682575
Araneae	Araneidae	*Mangora gibberosa*	ARONT187-09	GU682573
Araneae	Araneidae	*Mangora gibberosa*	ARONT810-10	HQ924655
Araneae	Araneidae	*Mangora gibberosa*	ARONT811-10	HQ924656
Araneae	Araneidae	*Mangora gibberosa*	ARONT812-10	HQ924657
Araneae	Araneidae	*Mangora gibberosa*	RBINA3305-13	KP647086
Araneae	Araneidae	*Mangora gibberosa*	RBINA3402-13	KP651105
Araneae	Araneidae	*Mangora gibberosa*	RBINA3405-13	KP645419
Araneae	Araneidae	*Mangora maculata*	GBCH3900-09	EU003293
Araneae	Araneidae	*Mangora maculata*	RBINA3293-13	KP649640
Araneae	Araneidae	*Mangora placida*	ARONT268-09	GU682728
Araneae	Araneidae	*Mangora placida*	ARONT276-09	HM434062
Araneae	Araneidae	*Mangora placida*	ARONT402-09	GU682956
Araneae	Araneidae	*Mangora placida*	ARONT508-10	HQ924401
Araneae	Araneidae	*Mangora placida*	ARONT716-10	HQ924572
Araneae	Araneidae	*Mangora placida*	RBINA1845-13	KP647542
Araneae	Araneidae	*Mangora placida*	RBINA2753-13	KP654158
Araneae	Araneidae	*Mangora placida*	RBINA2797-13	KP650954
Araneae	Araneidae	*Mangora placida*	RBINA2798-13	KP650408
Araneae	Araneidae	*Mangora placida*	RBINA2800-13	KP651057
Araneae	Araneidae	*Mangora placida*	RBINA2801-13	KP645548
Araneae	Araneidae	*Mangora placida*	RBINA2808-13	KP651362
Araneae	Araneidae	*Mangora placida*	RBINA2810-13	KP654773
Araneae	Araneidae	*Mangora placida*	RBINA2825-13	KP645966
Araneae	Araneidae	*Mangora placida*	RBINA2829-13	KP654783
Araneae	Araneidae	*Mangora placida*	RBINA2830-13	KP654067
Araneae	Araneidae	*Mangora placida*	RBINA3309-13	KP649389
Araneae	Araneidae	*Mangora placida*	RBINA3371-13	KP656226
Araneae	Araneidae	*Mangora placida*	RBINA3373-13	KP646207
Araneae	Araneidae	*Mangora placida*	RBINA3378-13	KP649232
Araneae	Araneidae	*Mangora placida*	RBINA3393-13	KP654151
Araneae	Araneidae	*Mangora placida*	RBINA5293-13	KP652157
Araneae	Araneidae	*Mangora placida*	RBINA5294-13	KP655331
Araneae	Araneidae	*Mangora placida*	RBINA5295-13	KP647945
Araneae	Araneidae	*Mangora placida*	RBINA5296-13	KP647808
Araneae	Araneidae	*Mangora placida*	RBINA5297-13	KP646649
Araneae	Araneidae	*Mangora placida*	RBINA5298-13	KP652353
Araneae	Araneidae	*Mangora placida*	RBINA5299-13	KP656696
Araneae	Araneidae	*Mangora placida*	RBINA5301-13	KP657233
Araneae	Araneidae	*Mangora placida*	RBINA5303-13	KP650678
Araneae	Araneidae	*Mangora placida*	RBINA5310-13	KP650015
Araneae	Araneidae	*Mangora placida*	RBINA5823-13	KP650102
Araneae	Araneidae	*Mangora placida*	RBINA5827-13	KP656316
Araneae	Araneidae	*Mangora placida*	RBINA990-13	KP646094
Araneae	Araneidae	*Mangora placida*	RBINA992-13	KP652540
Araneae	Araneidae	*Mangora placida*	SMTPB7319-13	KP656891
Araneae	Araneidae	*Mecynogea lemniscata*	GBCH3899-09	EU003294
Araneae	Araneidae	*Metepeira palustris*	CNGRC123-12	KM828761
Araneae	Araneidae	*Metepeira sp. 1GAB*	SSJAB3595-13	KM835110
Araneae	Araneidae	*Metepeira sp. TAB-2005*	GBCH1210-06	DQ146863

Araneae	Araneidae	*Metepeira sp. TAB-2009*	GBCH3239-09	FJ525325
Araneae	Araneidae	*Neoscona adianta*	GBCH11118-13	JN817152
Araneae	Araneidae	*Neoscona arabesca*	ARONT339-09	GU682737
Araneae	Araneidae	*Neoscona arabesca*	CNEIA2653-13	KM831232
Araneae	Araneidae	*Neoscona arabesca*	ERSPI403-09	GU682446
Araneae	Araneidae	*Neoscona arabesca*	GBCH3893-09	EU003301
Araneae	Araneidae	*Neoscona arabesca*	JSARA004-11	KP651542
Araneae	Araneidae	*Neoscona arabesca*	JSARA040-11	KP651047
Araneae	Araneidae	*Neoscona arabesca*	JSARA044-11	KP650119
Araneae	Araneidae	*Neoscona arabesca*	JSARA088-11	KP652397
Araneae	Araneidae	*Neoscona arabesca*	RBCH032-04	DQ127496
Araneae	Araneidae	*Neoscona arabesca*	RBCH039-04	DQ127487
Araneae	Araneidae	*Neoscona arabesca*	RBCH080-04	DQ127448
Araneae	Araneidae	*Neoscona arabesca*	RBCH116-04	DQ127420
Araneae	Araneidae	*Neoscona arabesca*	RBINA1112-13	KP649815
Araneae	Araneidae	*Neoscona arabesca*	SMTPB16295-13	KP656659
Araneae	Araneidae	*Neoscona arabesca*	SPRMA363-10	HQ580913
Araneae	Araneidae	*Neoscona crucifera*	GBCH3237-09	FJ525327
Araneae	Araneidae	*Neoscona multiplicans*	GBCH11120-13	JN817150
Araneae	Araneidae	*Neoscona nautica*	GBCH11123-13	JN817147
Araneae	Araneidae	*Neoscona pseudonautica*	GBCH11122-13	JN817148
Araneae	Araneidae	*Neoscona punctigera*	GBCH11119-13	JN817151
Araneae	Araneidae	*Neoscona punctigera*	GBCH5082-10	GU353214
Araneae	Araneidae	*Neoscona scylla*	GBCH11121-13	JN817149
Araneae	Araneidae	*Neoscona scylloides*	GBCH11117-13	JN817153
Araneae	Araneidae	*Neoscona subpullata*	GBCH11114-13	JN817156
Araneae	Araneidae	*Neoscona theisi*	GBCH11115-13	JN817155
Araneae	Araneidae	*Neoscona vigilans*	GBCH7175-13	HQ441945
Araneae	Araneidae	*Paraplectana sakaguchii*	GBCH7256-13	AB627002
Araneae	Araneidae	*Paraplectana tsushimensis*	GBCH7252-13	AB627006
Araneae	Araneidae	*Paraplectana tsushimensis*	GBCH7253-13	AB627005
Araneae	Araneidae	*Paraplectana tsushimensis*	GBCH7254-13	AB627004
Araneae	Araneidae	*Paraplectana tsushimensis*	GBCH7255-13	AB627003
Araneae	Araneidae	*Parazygiella dispar*	SPIAL216-10	HQ580671
Araneae	Araneidae	*Parazygiella dispar*	SPIAL228-10	HQ580678
Araneae	Araneidae	*Parazygiella dispar*	SPIAL240-10	HQ580688
Araneae	Araneidae	*Parazygiella dispar*	SPRMA201-10	HQ580815
Araneae	Araneidae	*Yaginumia sia*	GBCH11096-13	JN817174
Araneae	Araneidae	*Zygiella atrica*	ARONT096-09	GU682582
Araneae	Araneidae	*Zygiella atrica*	ARONT097-09	GU682578
Araneae	Araneidae	*Zygiella atrica*	ARONT098-09	GU682583
Araneae	Araneidae	*Zygiella atrica*	ARONT099-09	GU682657
Araneae	Araneidae	*Zygiella atrica*	ARONT100-09	GU682580
Araneae	Araneidae	*Zygiella atrica*	ARONT181-09	GU682581
Araneae	Araneidae	*Zygiella atrica*	ARONT182-09	GU682579
Araneae	Araneidae	*Zygiella atrica*	GBCH4010-09	FJ607589
Araneae	Araneidae	*Zygiella atrica*	SPRMA022-10	HQ977129
Araneae	Araneidae	*Zygiella atrica*	SPRMA202-10	HQ580816
Araneae	Araneidae	*Zygiella atrica*	SPRMA280-10	HQ580879
Araneae	Araneidae	*Zygiella atrica*	SPRMA744-10	JF887160
Araneae	Araneidae	*Zygiella nearctica*	SPICH1126-09	GU684584
Araneae	Araneidae	*Zygiella x-notata*	GBCH1513-06	DQ628603
Araneae	Araneidae	*Zygiella x-notata*	GBCH3883-09	EU003311

Araneae	Araneidae	*Zygiella x-notata*	SPRMA278-10	HQ580877
Araneae	Araneidae	*Zygiella x-notata*	SPRMA687-10	JF887124
Araneae	Araneidae	*Zygiella x-notata*	SPRMA692-10	JF887126
Araneae	Archaeidae	*Afrarchaea*	GBCH11409-13	JX240248
Araneae	Archaeidae	*Afrarchaea woodae*	GBCH11408-13	JX240249
Araneae	Archaeidae	*Austrarchaea*	GBCH11406-13	JX240251
Araneae	Archaeidae	*Austrarchaea nodosa*	GBCH11407-13	JX240250
Araneae	Archaeidae	*Eriauchenius bourgini*	GBCH11410-13	JX240247
Araneae	Archaeidae	*Eriauchenius bourgini*	GBCH2822-08	DQ914580
Araneae	Archaeidae	*Eriauchenius griswoldi*	GBCH2827-08	DQ914575
Araneae	Archaeidae	*Eriauchenius griswoldi*	GBCH2828-08	DQ914574
Araneae	Archaeidae	*Eriauchenius jeanneli*	GBCH11413-13	JX240244
Araneae	Archaeidae	*Eriauchenius lavatenda*	GBCH11414-13	JX240243
Araneae	Archaeidae	*Eriauchenius legendrei*	GBCH11412-13	JX240245
Araneae	Archaeidae	*Eriauchenius voronakely*	GBCH2837-08	DQ914565
Araneae	Archaeidae	*Eriauchenius voronakely*	GBCH2838-08	DQ914564
Araneae	Archaeidae	*Eriauchenius workmani*	GBCH11411-13	JX240246
Araneae	Archaeidae	*Zephyrarchaea mainae*	GBCH11405-13	JX240252
Araneae	Caponiidae	*Caponia*	GBCH7209-13	JN689215
Araneae	Clubionidae	*Clubiona abboti*	ARONT140-09	GU682613
Araneae	Clubionidae	*Clubiona abboti*	ARONT141-09	GU682614
Araneae	Clubionidae	*Clubiona abboti*	ARONT169-09	GU682612
Araneae	Clubionidae	*Clubiona abboti*	ARONT755-10	HQ924607
Araneae	Clubionidae	*Clubiona abboti*	ARONT801-10	HQ924646
Araneae	Clubionidae	*Clubiona abboti*	JSARA015-11	KP651054
Araneae	Clubionidae	*Clubiona abboti*	JSARA018-11	KP648740
Araneae	Clubionidae	*Clubiona abboti*	JSARA021-11	KP657006
Araneae	Clubionidae	*Clubiona abboti*	JSARA024-11	KP654091
Araneae	Clubionidae	*Clubiona abboti*	JSARA029-11	KP656111
Araneae	Clubionidae	*Clubiona abboti*	JSARA030-11	KP649691
Araneae	Clubionidae	*Clubiona abboti*	JSARA031-11	KP653017
Araneae	Clubionidae	*Clubiona abboti*	JSARA032-11	KP653047
Araneae	Clubionidae	*Clubiona abboti*	JSARA033-11	KP653960
Araneae	Clubionidae	*Clubiona abboti*	JSARA041-11	KP645816
Araneae	Clubionidae	*Clubiona abboti*	JSARA045-11	KP654169
Araneae	Clubionidae	*Clubiona abboti*	JSARA089-11	KP653168
Araneae	Clubionidae	*Clubiona abboti*	JSJUL2418-12	KP656754
Araneae	Clubionidae	*Clubiona abboti*	JSJUN2357-12	KP654312
Araneae	Clubionidae	*Clubiona abboti*	JSMAY1525-12	KP648212
Araneae	Clubionidae	*Clubiona abboti*	JSMAY1526-12	KP654932
Araneae	Clubionidae	*Clubiona abboti*	JSMAY1527-12	KP648027
Araneae	Clubionidae	*Clubiona abboti*	JSMAY1531-12	KP650717
Araneae	Clubionidae	*Clubiona abboti*	JSMAY1533-12	KP650245
Araneae	Clubionidae	*Clubiona abboti*	RBINA108-13	KP653754
Araneae	Clubionidae	*Clubiona abboti*	RBINA1557-13	KP656778
Araneae	Clubionidae	*Clubiona abboti*	RBINA231-13	KP645994
Araneae	Clubionidae	*Clubiona abboti*	RBINA2381-13	KP649799
Araneae	Clubionidae	*Clubiona abboti*	RBINA2668-13	KP648380
Araneae	Clubionidae	*Clubiona abboti*	RBINA3325-13	KP649432
Araneae	Clubionidae	*Clubiona abboti*	RBINA4433-13	KP647316
Araneae	Clubionidae	*Clubiona abboti*	RBINA4695-13	KP648506
Araneae	Clubionidae	*Clubiona abboti*	RBINA5129-13	KP649954
Araneae	Clubionidae	*Clubiona abboti*	RBINA541-13	KP652457

Araneae	Clubionidae	*Clubiona abboti*	RBINA557-13	KP648321
Araneae	Clubionidae	*Clubiona abboti*	SMTPB10030-13	KP653387
Araneae	Clubionidae	*Clubiona abboti*	SMTPB18884-13	KP657110
Araneae	Clubionidae	*Clubiona abboti*	SMTPB18886-13	KP650257
Araneae	Clubionidae	*Clubiona abboti*	SMTPB18890-13	KP657301
Araneae	Clubionidae	*Clubiona abboti*	SMTPB18891-13	KP645407
Araneae	Clubionidae	*Clubiona abboti*	SMTPB21282-13	KP655738
Araneae	Clubionidae	*Clubiona abboti*	SMTPB21283-13	KP657086
Araneae	Clubionidae	*Clubiona abboti*	SMTPB21286-13	KP649597
Araneae	Clubionidae	*Clubiona abboti*	SMTPB21290-13	KP656695
Araneae	Clubionidae	*Clubiona abboti*	SMTPB21292-13	KP648663
Araneae	Clubionidae	*Clubiona abboti*	SMTPB21293-13	KP656418
Araneae	Clubionidae	*Clubiona abboti*	SMTPB21294-13	KP651056
Araneae	Clubionidae	*Clubiona abboti*	SMTPB21297-13	KP650780
Araneae	Clubionidae	*Clubiona abboti*	SMTPB2321-13	KP647555
Araneae	Clubionidae	*Clubiona blesti*	GACAC1051-12	JN377951
Araneae	Clubionidae	*Clubiona blesti*	GACAC1052-12	JN377952
Araneae	Clubionidae	*Clubiona blesti*	GACAC1053-12	JN377953
Araneae	Clubionidae	*Clubiona blesti*	GACAC1054-12	JN377954
Araneae	Clubionidae	*Clubiona blesti*	GACAC1055-12	JN377955
Araneae	Clubionidae	*Clubiona blesti*	GACAC1056-12	JN377956
Araneae	Clubionidae	*Clubiona blesti*	GACAC1057-12	JN377957
Araneae	Clubionidae	*Clubiona blesti*	GACAC1058-12	JN377958
Araneae	Clubionidae	*Clubiona blesti*	GACAC1059-12	JN377959
Araneae	Clubionidae	*Clubiona bryantae*	ARONT417-09	GU682907
Araneae	Clubionidae	*Clubiona cada*	GACAC1060-12	JN377960
Araneae	Clubionidae	*Clubiona cada*	GBCH11789-13	JQ347511
Araneae	Clubionidae	*Clubiona cambridgei*	GACAC1062-12	JN377962
Araneae	Clubionidae	*Clubiona cambridgei*	GACAC1067-12	JN377967
Araneae	Clubionidae	*Clubiona cambridgei*	GACAC1068-12	JN377968
Araneae	Clubionidae	*Clubiona cambridgei*	GACAC1069-12	JN377969
Araneae	Clubionidae	*Clubiona canadensis*	ARONT248-09	GU682710
Araneae	Clubionidae	*Clubiona canadensis*	ARONT359-09	GU682873
Araneae	Clubionidae	*Clubiona canadensis*	ARONT373-09	GU682870
Araneae	Clubionidae	*Clubiona canadensis*	CNBAC742-12	KM829645
Araneae	Clubionidae	*Clubiona canadensis*	CNBAD669-12	KM839580
Araneae	Clubionidae	*Clubiona canadensis*	CNBAD698-12	KM830477
Araneae	Clubionidae	*Clubiona canadensis*	CNBAI521-13	KM837088
Araneae	Clubionidae	*Clubiona canadensis*	CNBAI528-13	KM827023
Araneae	Clubionidae	*Clubiona canadensis*	CNBAI529-13	KM839470
Araneae	Clubionidae	*Clubiona canadensis*	CNBAK454-13	KM837133
Araneae	Clubionidae	*Clubiona canadensis*	CNBPH378-13	KM831114
Araneae	Clubionidae	*Clubiona canadensis*	CNBPI538-13	KM826386
Araneae	Clubionidae	*Clubiona canadensis*	CNBPQ354-13	KM828603
Araneae	Clubionidae	*Clubiona canadensis*	CNBPQ358-13	KM831885
Araneae	Clubionidae	*Clubiona canadensis*	CNEIB1682-12	KM840561
Araneae	Clubionidae	*Clubiona canadensis*	CNGLB057-13	KM833253
Araneae	Clubionidae	*Clubiona canadensis*	CNGLC174-13	KM837879
Araneae	Clubionidae	*Clubiona canadensis*	CNGLF1915-13	KM828065
Araneae	Clubionidae	*Clubiona canadensis*	CNGLF1918-13	KM826391
Araneae	Clubionidae	*Clubiona canadensis*	CNGLF1919-13	KM839896
Araneae	Clubionidae	*Clubiona canadensis*	CNPAA483-13	KM824531
Araneae	Clubionidae	*Clubiona canadensis*	CNPAB299-13	KM835114

Araneae	Clubionidae	*Clubiona canadensis*	CNPAB302-13	KM831829
Araneae	Clubionidae	*Clubiona canadensis*	CNPAB308-13	KM837726
Araneae	Clubionidae	*Clubiona canadensis*	CNPAB309-13	KM838493
Araneae	Clubionidae	*Clubiona canadensis*	CNPAB310-13	KM826426
Araneae	Clubionidae	*Clubiona canadensis*	CNPAB311-13	KM835021
Araneae	Clubionidae	*Clubiona canadensis*	CNPAC675-13	KM834284
Araneae	Clubionidae	*Clubiona canadensis*	CNPAC676-13	KM833299
Araneae	Clubionidae	*Clubiona canadensis*	CNPAC677-13	KM837868
Araneae	Clubionidae	*Clubiona canadensis*	CNPAD926-13	KM829589
Araneae	Clubionidae	*Clubiona canadensis*	CNPAE468-13	KM840186
Araneae	Clubionidae	*Clubiona canadensis*	CNPAF927-13	KM824611
Araneae	Clubionidae	*Clubiona canadensis*	CNPAF929-13	KM830511
Araneae	Clubionidae	*Clubiona canadensis*	CNPAG319-13	KM832762
Araneae	Clubionidae	*Clubiona canadensis*	CNPAH467-13	KM827395
Araneae	Clubionidae	*Clubiona canadensis*	CNPAK002-13	KM831130
Araneae	Clubionidae	*Clubiona canadensis*	CNPAL002-13	KM827165
Araneae	Clubionidae	*Clubiona canadensis*	CNPAN002-13	KM839937
Araneae	Clubionidae	*Clubiona canadensis*	CNPAN003-13	KM832644
Araneae	Clubionidae	*Clubiona canadensis*	CNPAN014-13	KM835518
Araneae	Clubionidae	*Clubiona canadensis*	CNRMD2871-13	KM834527
Araneae	Clubionidae	*Clubiona canadensis*	CNRMF4170-13	KM836896
Araneae	Clubionidae	*Clubiona canadensis*	CNWLE2546-13	KM839630
Araneae	Clubionidae	*Clubiona canadensis*	CNWLE2547-13	KM831921
Araneae	Clubionidae	*Clubiona canadensis*	CNWLE2550-13	KM824646
Araneae	Clubionidae	*Clubiona canadensis*	CNWLE2553-13	KM832447
Araneae	Clubionidae	*Clubiona canadensis*	CNWLE2554-13	KM826439
Araneae	Clubionidae	*Clubiona canadensis*	CNWLF003-12	KM837051
Araneae	Clubionidae	*Clubiona canadensis*	CNWLF007-12	KM829910
Araneae	Clubionidae	*Clubiona canadensis*	CNWLF009-12	KM833693
Araneae	Clubionidae	*Clubiona canadensis*	CNWLH002-12	KM838510
Araneae	Clubionidae	*Clubiona canadensis*	CNWLL014-13	KM826119
Araneae	Clubionidae	*Clubiona canadensis*	PHJUN4058-12	KP646458
Araneae	Clubionidae	*Clubiona canadensis*	RBINA981-13	KP652016
Araneae	Clubionidae	*Clubiona canadensis*	SPRMA303-10	HQ977172
Araneae	Clubionidae	*Clubiona canadensis*	SPRMA584-10	JF887074
Araneae	Clubionidae	*Clubiona canadensis*	SPRMA585-10	JF887075
Araneae	Clubionidae	*Clubiona canadensis*	SPRMA831-12	KP652419
Araneae	Clubionidae	*Clubiona clima*	GACAC1061-12	JN377961
Araneae	Clubionidae	*Clubiona clima*	GACAC1066-12	JN377966
Araneae	Clubionidae	*Clubiona consensa*	GACAC1065-12	JN377965
Araneae	Clubionidae	*Clubiona consensa*	GACAC1070-12	JN377970
Araneae	Clubionidae	*Clubiona convoluta*	GACAC1071-12	JN377971
Araneae	Clubionidae	*Clubiona convoluta*	GACAC1072-12	JN377972
Araneae	Clubionidae	*Clubiona coreana*	GBCH11050-13	JN817220
Araneae	Clubionidae	*Clubiona elaphines*	GBCH11790-13	JQ347510
Araneae	Clubionidae	*Clubiona furcata*	CNEIA2651-13	KM826628
Araneae	Clubionidae	*Clubiona furcata*	CNEIB1687-13	KM824403
Araneae	Clubionidae	*Clubiona furcata*	CNEIB1688-13	KM829942
Araneae	Clubionidae	*Clubiona furcata*	CNEIC3178-13	KM838090
Araneae	Clubionidae	*Clubiona furcata*	CNEID3471-13	KM839748
Araneae	Clubionidae	*Clubiona furcata*	CNEIF2261-12	KM829475
Araneae	Clubionidae	*Clubiona furcata*	CNEIG001-12	KM835166
Araneae	Clubionidae	*Clubiona furcata*	CNEIG010-12	KM832218

Araneae	Clubionidae	*Clubiona furcata*	CNEIG011-12	KM826948
Araneae	Clubionidae	*Clubiona furcata*	CNEIG012-12	KM830750
Araneae	Clubionidae	*Clubiona furcata*	CNEIG1480-13	KM831386
Araneae	Clubionidae	*Clubiona furcata*	CNEIG1481-13	KM835173
Araneae	Clubionidae	*Clubiona furcata*	CNEIH003-13	KM839556
Araneae	Clubionidae	*Clubiona furcata*	CNEIH004-13	KM829287
Araneae	Clubionidae	*Clubiona furcata*	CNEIH009-13	KM825584
Araneae	Clubionidae	*Clubiona furcata*	CNEIH010-13	KM831101
Araneae	Clubionidae	*Clubiona furcata*	CNEIH012-13	KM828829
Araneae	Clubionidae	*Clubiona furcata*	CNEIH021-13	KM836685
Araneae	Clubionidae	*Clubiona furcata*	CNEIH023-13	KM835846
Araneae	Clubionidae	*Clubiona furcata*	CNEIH025-13	KM833624
Araneae	Clubionidae	*Clubiona furcata*	CNEIH026-13	KM832841
Araneae	Clubionidae	*Clubiona furcata*	CNEIH027-13	KM832607
Araneae	Clubionidae	*Clubiona furcata*	CNEIH031-13	KM826782
Araneae	Clubionidae	*Clubiona furcata*	CNEIH034-13	KM831991
Araneae	Clubionidae	*Clubiona furcata*	CNEII005-13	KM837034
Araneae	Clubionidae	*Clubiona furcata*	CNWBE378-13	KM837329
Araneae	Clubionidae	*Clubiona furcata*	GBADC025-10	HQ956688
Araneae	Clubionidae	*Clubiona huttoni*	GACAC1063-12	JN377963
Araneae	Clubionidae	*Clubiona huttoni*	GACAC1064-12	JN377964
Araneae	Clubionidae	*Clubiona japonicola*	GBCH11049-13	JN817221
Araneae	Clubionidae	*Clubiona johnsoni*	ARONT698-10	HQ924556
Araneae	Clubionidae	*Clubiona johnsoni*	RBINA3949-13	KP654934
Araneae	Clubionidae	*Clubiona kastoni*	CNRMB227-12	KM829301
Araneae	Clubionidae	*Clubiona kastoni*	CNRMB231-12	KM837727
Araneae	Clubionidae	*Clubiona kastoni*	CNRMC1575-12	KM829916
Araneae	Clubionidae	*Clubiona kastoni*	CNRMC1576-12	KM825884
Araneae	Clubionidae	*Clubiona kastoni*	CNRMC1577-12	KM832746
Araneae	Clubionidae	*Clubiona kastoni*	CNRMC1691-12	KM839627
Araneae	Clubionidae	*Clubiona kastoni*	CNRMD2820-12	KM825149
Araneae	Clubionidae	*Clubiona kastoni*	CNRMD2821-12	KM824282
Araneae	Clubionidae	*Clubiona kastoni*	CNRMD2824-12	KM834459
Araneae	Clubionidae	*Clubiona kastoni*	CNRMD2864-13	KM833766
Araneae	Clubionidae	*Clubiona kastoni*	CNRMD2865-13	KM839778
Araneae	Clubionidae	*Clubiona kastoni*	CNRMD2872-13	KM833200
Araneae	Clubionidae	*Clubiona kastoni*	CNRMG720-12	KM835569
Araneae	Clubionidae	*Clubiona kastoni*	CNSLB456-12	KM838863
Araneae	Clubionidae	*Clubiona kastoni*	CNSLD632-12	KM834175
Araneae	Clubionidae	*Clubiona kastoni*	RBINA1080-13	KP652433
Araneae	Clubionidae	*Clubiona kastoni*	RBINA1102-13	KP646617
Araneae	Clubionidae	*Clubiona kastoni*	RBINA1187-13	KP649004
Araneae	Clubionidae	*Clubiona kastoni*	RBINA3323-13	KP645555
Araneae	Clubionidae	*Clubiona kulczynskii*	CNBAA471-12	KM836982
Araneae	Clubionidae	*Clubiona kulczynskii*	CNEIH002-13	KM836804
Araneae	Clubionidae	*Clubiona kulczynskii*	CNEIH006-13	KM839321
Araneae	Clubionidae	*Clubiona kulczynskii*	CNJAI008-12	KM827784
Araneae	Clubionidae	*Clubiona kulczynskii*	CNRME5224-13	KM824899
Araneae	Clubionidae	*Clubiona maritima*	RBCH137-04	DQ127395
Araneae	Clubionidae	*Clubiona maritima*	RBCH150-04	DQ127376
Araneae	Clubionidae	*Clubiona maritima*	SMTPB14447-13	KP653578
Araneae	Clubionidae	*Clubiona moesta*	CNEIF2208-12	KM830808
Araneae	Clubionidae	*Clubiona moesta*	CNEIJ003-13	KM838128

Araneae	Clubionidae	*Clubiona moesta*	CNGRF423-12	KM840650
Araneae	Clubionidae	*Clubiona moesta*	CNPAC614-13	KM829426
Araneae	Clubionidae	*Clubiona moesta*	CNPAC615-13	KM837923
Araneae	Clubionidae	*Clubiona moesta*	CNPAJ015-13	KM833098
Araneae	Clubionidae	*Clubiona moesta*	CNPAK024-13	KM827102
Araneae	Clubionidae	*Clubiona moesta*	CNPAO653-13	KM824120
Araneae	Clubionidae	*Clubiona moesta*	CNRMA1105-12	KM837241
Araneae	Clubionidae	*Clubiona moesta*	CNRMB232-12	KM831622
Araneae	Clubionidae	*Clubiona moesta*	CNRMB233-12	KM826432
Araneae	Clubionidae	*Clubiona moesta*	CNRMD2869-13	KM839467
Araneae	Clubionidae	*Clubiona moesta*	CNRMF3847-12	KM833311
Araneae	Clubionidae	*Clubiona moesta*	CNWBD026-13	KM832921
Araneae	Clubionidae	*Clubiona moesta*	CNWBG1012-13	KM829547
Araneae	Clubionidae	*Clubiona moesta*	CNWLA781-13	KM825746
Araneae	Clubionidae	*Clubiona moesta*	CNWLA782-13	KM830124
Araneae	Clubionidae	*Clubiona moesta*	CNWLA783-13	KM829909
Araneae	Clubionidae	*Clubiona moesta*	CNWLB647-13	KM829389
Araneae	Clubionidae	*Clubiona moesta*	CNWLB762-13	KM836083
Araneae	Clubionidae	*Clubiona moesta*	CNWLB764-13	KM827339
Araneae	Clubionidae	*Clubiona moesta*	CNWLD1161-13	KM836183
Araneae	Clubionidae	*Clubiona moesta*	CNWLD1162-13	KM836398
Araneae	Clubionidae	*Clubiona moesta*	CNWLE2559-13	KM840077
Araneae	Clubionidae	*Clubiona moesta*	CNWLF005-12	KM835756
Araneae	Clubionidae	*Clubiona moesta*	CNWLH005-12	KM835384
Araneae	Clubionidae	*Clubiona moesta*	CNWLK139-13	KM834271
Araneae	Clubionidae	*Clubiona moesta*	CNWLL216-13	KM838761
Araneae	Clubionidae	*Clubiona moesta*	PHSEP1964-11	KP655215
Araneae	Clubionidae	*Clubiona mutata*	SSJAE4189-13	KM834132
Araneae	Clubionidae	*Clubiona mutata*	SSJAE4249-13	KM834247
Araneae	Clubionidae	*Clubiona mutata*	SSWLB3939-13	KM828942
Araneae	Clubionidae	*Clubiona mutata*	SSWLB469-13	KM836140
Araneae	Clubionidae	*Clubiona mutata*	SSWLB472-13	KR069891
Araneae	Clubionidae	*Clubiona mutata*	SSWLB5776-13	KM837651
Araneae	Clubionidae	*Clubiona mutata*	SSWLB5779-13	KM834473
Araneae	Clubionidae	*Clubiona mutata*	SSWLB5797-13	KM836094
Araneae	Clubionidae	*Clubiona mutata*	SSWLB5799-13	KM834797
Araneae	Clubionidae	*Clubiona mutata*	SSWLE1781-13	KR069914
Araneae	Clubionidae	*Clubiona mutata*	SSWLE9521-13	KM830518
Araneae	Clubionidae	*Clubiona norvegica*	CNPAJ019-13	KM828737
Araneae	Clubionidae	*Clubiona norvegica*	KKCHE962-09	GU683661
Araneae	Clubionidae	*Clubiona norvegica*	SPRMA198-10	HQ580812
Araneae	Clubionidae	*Clubiona obesa*	ARONT381-09	GU682904
Araneae	Clubionidae	*Clubiona obesa*	ARONT392-09	GU682905
Araneae	Clubionidae	*Clubiona obesa*	ARONT393-09	GU682902
Araneae	Clubionidae	*Clubiona obesa*	ARONT469-10	HM880621
Araneae	Clubionidae	*Clubiona obesa*	CNSLC545-12	KM826304
Araneae	Clubionidae	*Clubiona obesa*	CNSLC546-12	KM826003
Araneae	Clubionidae	*Clubiona obesa*	CNSLD640-12	KM823987
Araneae	Clubionidae	*Clubiona obesa*	CNSLD642-12	KM836424
Araneae	Clubionidae	*Clubiona obesa*	CNSLD643-12	KM827779
Araneae	Clubionidae	*Clubiona obesa*	CNSLI534-12	KM832299
Araneae	Clubionidae	*Clubiona obesa*	CNSLM001-13	KM828877
Araneae	Clubionidae	*Clubiona obesa*	CNSLM003-13	KM830991

Araneae	Clubionidae	*Clubiona obesa*	CNSLM005-13	KM826198
Araneae	Clubionidae	*Clubiona obesa*	CNSLN005-13	KM833316
Araneae	Clubionidae	*Clubiona obesa*	CNSLP002-13	KM839880
Araneae	Clubionidae	*Clubiona obesa*	JSARA007-11	KP646845
Araneae	Clubionidae	*Clubiona obesa*	JSARA022-11	KP645825
Araneae	Clubionidae	*Clubiona obesa*	JSARA027-11	KP645588
Araneae	Clubionidae	*Clubiona obesa*	JSARA034-11	KP645502
Araneae	Clubionidae	*Clubiona obesa*	JSARA038-11	KP656598
Araneae	Clubionidae	*Clubiona obesa*	JSARA043-11	KP656363
Araneae	Clubionidae	*Clubiona obesa*	JSARA047-11	KP648155
Araneae	Clubionidae	*Clubiona obesa*	JSARA051-11	KP652665
Araneae	Clubionidae	*Clubiona obesa*	JSARA055-11	KP652549
Araneae	Clubionidae	*Clubiona obesa*	JSARA064-11	KP654507
Araneae	Clubionidae	*Clubiona obesa*	JSARA069-11	KP655621
Araneae	Clubionidae	*Clubiona obesa*	JSARA072-11	KP650103
Araneae	Clubionidae	*Clubiona obesa*	JSARA077-11	KP656721
Araneae	Clubionidae	*Clubiona obesa*	JSARA081-11	KP657468
Araneae	Clubionidae	*Clubiona obesa*	JSARA092-11	KP655154
Araneae	Clubionidae	*Clubiona obesa*	JSARA101-11	KP655283
Araneae	Clubionidae	*Clubiona obesa*	JSARA102-11	KP656186
Araneae	Clubionidae	*Clubiona obesa*	JSARA103-11	KP652176
Araneae	Clubionidae	*Clubiona obesa*	JSARA104-11	KP646508
Araneae	Clubionidae	*Clubiona obesa*	JSARA105-11	KP646707
Araneae	Clubionidae	*Clubiona obesa*	JSSEP1123-12	KP650229
Araneae	Clubionidae	*Clubiona obesa*	PHOCT935-12	KP652111
Araneae	Clubionidae	*Clubiona obesa*	PHSEP1956-11	KP653601
Araneae	Clubionidae	*Clubiona obesa*	RBINA1206-13	KP645681
Araneae	Clubionidae	*Clubiona obesa*	RBINA1219-13	KP648868
Araneae	Clubionidae	*Clubiona obesa*	RBINA1226-13	KP650860
Araneae	Clubionidae	*Clubiona obesa*	RBINA1231-13	KP655736
Araneae	Clubionidae	*Clubiona obesa*	SMTPB15350-13	KP652966
Araneae	Clubionidae	*Clubiona obesa*	SMTPB15351-13	KP657208
Araneae	Clubionidae	*Clubiona obesa*	SMTPB15381-13	KP648832
Araneae	Clubionidae	*Clubiona obesa*	SMTPB15383-13	KP654612
Araneae	Clubionidae	*Clubiona opeongo*	SSBAC2879-12	KM838707
Araneae	Clubionidae	*Clubiona opeongo*	SSBAC2910-12	KM831032
Araneae	Clubionidae	*Clubiona opeongo*	SSBAC4366-13	KR070287
Araneae	Clubionidae	*Clubiona opeongo*	SSBAC4388-13	KM833655
Araneae	Clubionidae	*Clubiona opeongo*	SSJAE6318-13	KM840039
Araneae	Clubionidae	*Clubiona pallidula*	ARONT247-09	GU682712
Araneae	Clubionidae	*Clubiona pallidula*	ARONT370-09	GU682871
Araneae	Clubionidae	*Clubiona pallidula*	ARONT371-09	GU682868
Araneae	Clubionidae	*Clubiona pallidula*	ARONT372-09	GU682869
Araneae	Clubionidae	*Clubiona pallidula*	ARONT592-10	HQ924467
Araneae	Clubionidae	*Clubiona pallidula*	ARONT613-10	HQ924478
Araneae	Clubionidae	*Clubiona pallidula*	ARONT614-10	HQ924479
Araneae	Clubionidae	*Clubiona pallidula*	ARONT615-10	HQ924480
Araneae	Clubionidae	*Clubiona pallidula*	ARONT616-10	HQ924481
Araneae	Clubionidae	*Clubiona pallidula*	CNGIF088-12	KM824413
Araneae	Clubionidae	*Clubiona pallidula*	CNPCE073-12	KM838885
Araneae	Clubionidae	*Clubiona pallidula*	SMTPB1358-13	KP656607
Araneae	Clubionidae	*Clubiona pallidula*	SMTPB19024-13	KP657386
Araneae	Clubionidae	*Clubiona pallidula*	SMTPB5713-13	KP646939

Araneae	Clubionidae	*Clubiona pallidula*	SMTPB7428-13	KP647135
Araneae	Clubionidae	*Clubiona pallidula*	SMTPB7430-13	KP652123
Araneae	Clubionidae	*Clubiona pallidula*	SPRMA716-10	JF887142
Araneae	Clubionidae	*Clubiona peculiaris*	GBCH11788-13	JQ347512
Araneae	Clubionidae	*Clubiona pikei*	CNPPE2327-12	KJ090471
Araneae	Clubionidae	*Clubiona pikei*	CNPPE2337-12	KJ163421
Araneae	Clubionidae	*Clubiona pikei*	CNPPE2343-12	KJ163828
Araneae	Clubionidae	*Clubiona pikei*	CNPPE2346-12	KJ165803
Araneae	Clubionidae	*Clubiona pikei*	CNPPE2347-12	KJ164433
Araneae	Clubionidae	*Clubiona pikei*	GMNCG466-12	KJ085672
Araneae	Clubionidae	*Clubiona praematura*	KKCHE977-09	GU683674
Araneae	Clubionidae	*Clubiona pygmaea*	CNSLC536-12	KM832835
Araneae	Clubionidae	*Clubiona pygmaea*	CNSLG294-12	KM839234
Araneae	Clubionidae	*Clubiona pygmaea*	CNSLG296-12	KM826253
Araneae	Clubionidae	*Clubiona pygmaea*	CNSLH171-12	KM835789
Araneae	Clubionidae	*Clubiona pygmaea*	CNSLR096-13	KM824731
Araneae	Clubionidae	*Clubiona riparia*	ARONT119-09	HM434051
Araneae	Clubionidae	*Clubiona riparia*	ARONT120-09	GU682610
Araneae	Clubionidae	*Clubiona riparia*	ARONT121-09	GU682611
Araneae	Clubionidae	*Clubiona riparia*	ARONT122-09	GU682608
Araneae	Clubionidae	*Clubiona riparia*	RBINA1327-13	KP648596
Araneae	Clubionidae	*Clubiona riparia*	RBINA1328-13	KP653810
Araneae	Clubionidae	*Clubiona riparia*	RBINA1436-13	KP648028
Araneae	Clubionidae	*Clubiona riparia*	RBINA3310-13	KP646667
Araneae	Clubionidae	*Clubiona saltitans*	SPRMA583-10	JF887073
Araneae	Clubionidae	*Clubiona trivialis*	GBADC017-10	HQ956680
Araneae	Clubionidae	*Elaver excepta*	CNBPC120-12	KM833368
Araneae	Clubionidae	*Elaver excepta*	CNBPD661-13	KM829533
Araneae	Clubionidae	*Elaver excepta*	CNBPD662-13	KM835762
Araneae	Clubionidae	*Elaver excepta*	CNBPD663-13	KM832508
Araneae	Clubionidae	*Elaver excepta*	CNBPL379-13	KM825423
Araneae	Clubionidae	*Elaver excepta*	CNBPL380-13	KM831904
Araneae	Clubionidae	*Elaver excepta*	CNBPM114-13	KM830918
Araneae	Clubionidae	*Elaver excepta*	CNBPQ351-13	KM840755
Araneae	Clubionidae	*Elaver excepta*	CNBPT145-13	KM828768
Araneae	Clubionidae	*Elaver excepta*	CNPPI1855-12	KJ445598
Araneae	Clubionidae	*Elaver excepta*	CNSLB455-12	KM835590
Araneae	Clubionidae	*Elaver excepta*	CNSLC538-12	KM833341
Araneae	Clubionidae	*Elaver excepta*	CNSLC543-12	KM834558
Araneae	Clubionidae	*Elaver excepta*	CNSLE486-12	KM830109
Araneae	Clubionidae	*Elaver excepta*	CNSLG290-12	KM836968
Araneae	Clubionidae	*Elaver excepta*	CNSLG297-12	KM832626
Araneae	Clubionidae	*Elaver excepta*	CNSLL374-13	KM830942
Araneae	Clubionidae	*Elaver excepta*	CNSLM130-13	KM840359
Araneae	Clubionidae	*Elaver excepta*	CNSLN108-13	KM824059
Araneae	Clubionidae	*Elaver excepta*	CNSLN453-13	KM837742
Araneae	Clubionidae	*Elaver excepta*	CNSLO204-13	KM832582
Araneae	Clubionidae	*Elaver excepta*	CNSLO212-13	KM835804
Araneae	Clubionidae	*Elaver excepta*	CNSLQ080-13	KM839901
Araneae	Clubionidae	*Elaver excepta*	CNSLQ084-13	KM835262
Araneae	Clubionidae	*Elaver excepta*	CNSLQ085-13	KM830163
Araneae	Clubionidae	*Elaver excepta*	CNSLQ087-13	KM827488
Araneae	Clubionidae	*Elaver excepta*	CNSLQ090-13	KM836832

Araneae	Clubionidae	*Elaver excepta*	CNSLQ093-13	KM837640
Araneae	Clubionidae	*Elaver excepta*	CNSLQ096-13	KM835533
Araneae	Clubionidae	*Elaver excepta*	CNSLQ107-13	KM831063
Araneae	Clubionidae	*Elaver excepta*	CNSLQ115-13	KM828546
Araneae	Clubionidae	*Elaver excepta*	CNSLU108-13	KM830951
Araneae	Clubionidae	*Elaver excepta*	CNSLU117-13	KM833871
Araneae	Clubionidae		CNBAN017-13	KR070513
Araneae	Clubionidae		CNBAN019-13	KP979165
Araneae	Clubionidae		CNBAN020-13	KP979267
Araneae	Clubionidae		CNBAN622-13	KP979205
Araneae	Corinnidae	*Castianeira sp. 1GAB*	SPRMA703-10	JF887134
Araneae	Corinnidae	*Castianeira variata*	CNPCB045-12	KM839949
Araneae	Corinnidae	*Castianeira walsinghami*	SPRMA140-10	HQ580769
Araneae	Ctenidae	*Anahita fauna*	GBCH11054-13	JN817216
Araneae	Ctenizidae	*Conothele*	GBCH8417-13	KC708091
Araneae	Ctenizidae	*Conothele*	GBCH8418-13	KC708090
Araneae	Cybaeidae	*Allocybaeina littlewalteri*	SPRMA720-10	JF887145
Araneae	Cybaeidae	*Argyroneta aquatica*	GBCH11068-13	JN817202
Araneae	Cybaeidae	*Cybaeota nana*	GBCH5431-10	FJ263787
Araneae	Cybaeidae	*Cybaeota shastae*	SPIAL067-10	HQ580572
Araneae	Cybaeidae	*Cybaeus angustiarum*	GBCH5428-10	FJ263790
Araneae	Cybaeidae	*Cybaeus chauliodous*	GBCH5419-10	FJ263799
Araneae	Cybaeidae	*Cybaeus gidneyi*	GBCH5418-10	FJ263800
Araneae	Cybaeidae	*Cybaeus giganteus*	GBCH5430-10	FJ263788
Araneae	Cybaeidae	*Cybaeus hesper*	GBCH5422-10	FJ263796
Araneae	Cybaeidae	*Cybaeus morosus*	GBCH5426-10	FJ263792
Araneae	Cybaeidae	*Cybaeus morosus*	SPRMA096-10	HQ580737
Araneae	Cybaeidae	*Cybaeus morosus*	SPRMA701-10	KP647334
Araneae	Cybaeidae	*Cybaeus morosus*	SPRMA939-12	KP646799
Araneae	Cybaeidae	*Cybaeus mosanensis*	GBCH11067-13	JN817203
Araneae	Cybaeidae	*Cybaeus patritus*	GBCH5429-10	FJ263789
Araneae	Cybaeidae	*Cybaeus penedentatus*	GBCH5421-10	FJ263797
Araneae	Cybaeidae	*Cybaeus reticulatus*	GBCH5425-10	FJ263793
Araneae	Cybaeidae	*Cybaeus reticulatus*	SPIAL264-10	HQ580707
Araneae	Cybaeidae	*Cybaeus reticulatus*	SPRMA253-10	HQ580858
Araneae	Cybaeidae	*Cybaeus reticulatus*	SPRMA285-10	HQ580884
Araneae	Cybaeidae	*Cybaeus reticulatus*	SPRMA682-10	JF887120
Araneae	Cybaeidae	*Cybaeus sanbruno*	GBCH5423-10	FJ263795
Araneae	Cybaeidae	*Cybaeus signifer*	GBCH5424-10	FJ263794
Araneae	Cybaeidae	*Cybaeus signifer*	SPRMA271-10	HQ580872
Araneae	Cybaeidae	*Cybaeus signifer*	SPRMA520-10	JF887037
Araneae	Cybaeidae	*Cybaeus sinuosus*	SPRMA306-10	HQ977174
Araneae	Cybaeidae	*Cybaeus somesbar*	GBCH5420-10	FJ263798
Araneae	Cybaeidae	*Cybaeus sp. 1GAB*	SPRMA719-10	JF887144
Araneae	Cybaeidae	*Cybaeus sp. 2GAB*	SPRMA718-10	JF887143
Araneae	Cybaeidae	*Cybaeus sp. 2GAB*	SPRMA723-10	JF887148
Araneae	Cybaeidae	*Cybaeus thermydrinos*	SPRMA724-10	KP979257
Araneae	Cybaeidae	*Dolichocybaeus*	GBCH11066-13	JN817204
Araneae	Desidae	*Badumna insignis*	CNBAN015-13	KP979154
Araneae	Desidae	*Badumna longinqua*	GBCH1520-06	DQ628610
Araneae	Desidae	*Badumna longinqua*	GBCH4041-09	FJ607558
Araneae	Desidae	*Paratheuma shirahamaensis*	GBCH11065-13	JN817205
Araneae	Dictynidae	*Arctella lapponica*	KKCHE1044-09	GU683728

Araneae	Dictynidae	*Argenna obesa*	SMTPB21299-13	KP646247
Araneae	Dictynidae	*Brommella monticola*	SPRMA111-10	HQ580749
Araneae	Dictynidae	*Brommella monticola*	SPRMA981-12	KP656238
Araneae	Dictynidae	*Cicurina arcuata*	ERSPI154-09	KP653066
Araneae	Dictynidae	*Cicurina brevis*	ARONT213-09	GU682704
Araneae	Dictynidae	*Cicurina brevis*	ARONT442-09	GU682909
Araneae	Dictynidae	*Cicurina brevis*	ARONT455-09	GU682906
Araneae	Dictynidae	*Cicurina brevis*	ARONT540-10	KR069622
Araneae	Dictynidae	*Cicurina brevis*	CNBPL378-13	KM828706
Araneae	Dictynidae	*Cicurina brevis*	ERSPI285-09	GU682540
Araneae	Dictynidae	*Cicurina brevis*	RBINA3256-13	KP655859
Araneae	Dictynidae	*Cicurina intermedia*	SPRMA147-10	HQ580774
Araneae	Dictynidae	*Cicurina intermedia*	SPRMA153-10	HQ580778
Araneae	Dictynidae	*Cicurina itasca*	ERSPI144-09	KP653448
Araneae	Dictynidae	*Cicurina japonica*	GBCH11063-13	JN817207
Araneae	Dictynidae	*Cicurina pallida*	ERSPI249-09	GU682563
Araneae	Dictynidae	*Cicurina placida*	ARONT423-09	GU682911
Araneae	Dictynidae	*Cicurina placida*	ARONT424-09	GU682908
Araneae	Dictynidae	*Cicurina robusta*	ERSPI045-08	KP648501
Araneae	Dictynidae	*Cicurina robusta*	ERSPI235-09	KP652201
Araneae	Dictynidae	*Cicurina sp. 1GAB*	ARONT699-10	HQ924557
Araneae	Dictynidae	*Cicurina tersa*	SPRMA319-10	HQ580887
Araneae	Dictynidae	*Cicurina tersa*	SPRMA462-10	KR070307
Araneae	Dictynidae	*Cicurina tersa*	SPRMA464-10	JN310302
Araneae	Dictynidae	*Cicurina tersa*	SPRMA973-12	KP649008
Araneae	Dictynidae	*Cicurina toreriaria*	SPRMA760-10	JF887174
Araneae	Dictynidae	*Dictyna bellans*	ARONT482-10	HQ924381
Araneae	Dictynidae	*Dictyna brevitarsa*	GBADC009-10	HQ956673
Araneae	Dictynidae	*Dictyna brevitarsa*	PHMTU053-10	JN307930
Araneae	Dictynidae	*Dictyna brevitarsa*	SPRMA307-10	HQ977175
Araneae	Dictynidae	*Dictyna brevitarsa*	SPRMA470-10	JF887017
Araneae	Dictynidae	*Dictyna foliacea*	ARONT034-09	GU682808
Araneae	Dictynidae	*Dictyna foliacea*	ARONT035-09	GU682815
Araneae	Dictynidae	*Dictyna foliacea*	ERSPI399-09	GU682454
Araneae	Dictynidae	*Dictyna major*	CNWLH325-12	KM827616
Araneae	Dictynidae	*Dictyna major*	ERSPI404-09	GU682448
Araneae	Dictynidae	*Dictyna major*	GBADC040-10	HQ956702
Araneae	Dictynidae	*Dictyna major*	SPICH1048-09	GU684716
Araneae	Dictynidae	*Dictyna major*	SPRMA469-10	JF887016
Araneae	Dictynidae	*Dictyna major*	SPRMA683-10	JF887121
Araneae	Dictynidae	*Dictyna major*	SPRMA906-12	KP649790
Araneae	Dictynidae	*Dictyna personata*	SPRMA895-12	KP656097
Araneae	Dictynidae	*Dictyna sp. 1GAB*	SSWLB3941-13	KM825417
Araneae	Dictynidae	*Dictyna sp. 1GAB*	SSWLB471-13	KM833983
Araneae	Dictynidae	*Dictyna sp. 1GAB*	SSWLB473-13	KM833356
Araneae	Dictynidae	*Dictyna sp. 1GAB*	SSWLB475-13	KM837169
Araneae	Dictynidae	*Dictyna volucripes*	ARONT189-09	GU682607
Araneae	Dictynidae	*Dictyna volucripes*	ARONT274-09	GU682692
Araneae	Dictynidae	*Dictyna volucripes*	ARONT275-09	GU682689
Araneae	Dictynidae	*Dictyna volucripes*	ARONT638-10	HQ924503
Araneae	Dictynidae	*Dictyna volucripes*	ARONT706-10	HQ924563
Araneae	Dictynidae	*Dictyna volucripes*	ERSPI390-09	GU682457
Araneae	Dictynidae	*Dictyna volucripes*	RBINA1865-13	KP650714

Araneae	Dictynidae	*Dictyna volucripes*	RBINA3255-13	KP655448
Araneae	Dictynidae	*Dictyna volucripes*	RBINA3361-13	KP651060
Araneae	Dictynidae	*Dictyna volucripes*	RBINA3379-13	KP647507
Araneae	Dictynidae	*Dictyna volucripes*	RBINA3384-13	KP654161
Araneae	Dictynidae	*Dictyna volucripes*	RBINA3396-13	KP656531
Araneae	Dictynidae	*Dictyna volucripes*	RBINA5800-13	KP651621
Araneae	Dictynidae	*Dictyna volucripes*	RBINA5816-13	KP650603
Araneae	Dictynidae	*Dictyna volucripes*	RBINA5819-13	KP656605
Araneae	Dictynidae	*Dictyna volucripes*	RBINA5821-13	KP651691
Araneae	Dictynidae	*Dictyna volucripes*	RBINA5832-13	KP656904
Araneae	Dictynidae	*Dictyna volucripes*	RBINA5844-13	KP656071
Araneae	Dictynidae	*Dictyna volucripes*	RBINA803-13	KP652459
Araneae	Dictynidae	*Dictyna volucripes*	RBINA829-13	KP653141
Araneae	Dictynidae	*Dictyna volucripes*	RBINA830-13	KP655701
Araneae	Dictynidae	*Dictyna volucripes*	SPRMA471-10	JF887018
Araneae	Dictynidae	*Emblyna annulipes*	ARONT230-09	GU682685
Araneae	Dictynidae	*Emblyna annulipes*	ARONT251-09	GU682683
Araneae	Dictynidae	*Emblyna annulipes*	ARONT252-09	GU682686
Araneae	Dictynidae	*Emblyna annulipes*	CNWLI005-12	KM835931
Araneae	Dictynidae	*Emblyna annulipes*	JSMAY1534-12	KP650031
Araneae	Dictynidae	*Emblyna annulipes*	JSSEP1237-12	KP648017
Araneae	Dictynidae	*Emblyna annulipes*	JSSEP1240-12	KP657009
Araneae	Dictynidae	*Emblyna borealis*	SPRMA875-12	KP655409
Araneae	Dictynidae	*Emblyna borealis*	SSWLB470-13	KM836124
Araneae	Dictynidae	*Emblyna hentzi*	RBINA1864-13	KP651103
Araneae	Dictynidae	*Emblyna hentzi*	RBINA1877-13	KP646205
Araneae	Dictynidae	*Emblyna hentzi*	RBINA5811-13	KP652460
Araneae	Dictynidae	*Emblyna hentzi*	SMTPB3691-13	KP655025
Araneae	Dictynidae	*Emblyna manitoba*	ARONT506-10	HQ924400
Araneae	Dictynidae	*Emblyna maxima*	CNRMD2866-13	KM829494
Araneae	Dictynidae	*Emblyna maxima*	SSFDC5995-14	KP651550
Araneae	Dictynidae	*Emblyna maxima*	SSPAB9957-13	KM834908
Araneae	Dictynidae	*Emblyna peragrata*	CNBAA458-12	KM830638
Araneae	Dictynidae	*Emblyna peragrata*	CNBAB387-12	KM826977
Araneae	Dictynidae	*Emblyna peragrata*	CNBAC743-12	KM840061
Araneae	Dictynidae	*Emblyna peragrata*	SPIAL007-10	HQ580521
Araneae	Dictynidae	*Emblyna peragrata*	SPIAL008-10	HQ580522
Araneae	Dictynidae	*Emblyna peragrata*	SPIAL019-10	KR069493
Araneae	Dictynidae	*Emblyna peragrata*	SPIAL020-10	HQ580532
Araneae	Dictynidae	*Emblyna peragrata*	SPIAL031-10	HQ580543
Araneae	Dictynidae	*Emblyna peragrata*	SPIAL043-10	HQ580553
Araneae	Dictynidae	*Emblyna peragrata*	SPIAL090-10	HQ580591
Araneae	Dictynidae	*Emblyna peragrata*	SPIAL091-10	HQ580592
Araneae	Dictynidae	*Emblyna peragrata*	SPICH1043-09	GU684713
Araneae	Dictynidae	*Emblyna peragrata*	SPRMA200-10	HQ580814
Araneae	Dictynidae	*Emblyna peragrata*	SPRMA866-12	KP649576
Araneae	Dictynidae	*Emblyna phylax*	CNBAF199-12	KM835736
Araneae	Dictynidae	*Emblyna phylax*	CNJAB108-12	KM839035
Araneae	Dictynidae	*Emblyna phylax*	CNPAG320-13	KM834721
Araneae	Dictynidae	*Emblyna phylax*	JSARA046-11	KP656852
Araneae	Dictynidae	*Emblyna sp. 1GAB*	SSKJB4447-14	KR070093
Araneae	Dictynidae	*Emblyna sp. 1GAB*	CNKJK1913-14	KP653034
Araneae	Dictynidae	*Emblyna sp. 1GAB*	CNKJO846-14	KP651564

Araneae	Dictynidae	*Emblyna sp. 1GAB*	SSJAB1356-13	KM836648
Araneae	Dictynidae	*Emblyna sp. 1GAB*	SSJAB4170-13	KM837244
Araneae	Dictynidae	*Emblyna sublata*	ARONT228-09	GU682687
Araneae	Dictynidae	*Emblyna sublata*	ARONT229-09	GU682731
Araneae	Dictynidae	*Emblyna sublata*	ARONT249-09	GU682688
Araneae	Dictynidae	*Emblyna sublata*	ARONT250-09	GU682690
Araneae	Dictynidae	*Emblyna sublata*	ARONT674-10	HQ924533
Araneae	Dictynidae	*Emblyna sublata*	ARONT675-10	HQ924534
Araneae	Dictynidae	*Emblyna sublata*	ARONT676-10	HQ924535
Araneae	Dictynidae	*Emblyna sublata*	ARONT677-10	HQ924536
Araneae	Dictynidae	*Emblyna sublata*	ARONT678-10	HQ924537
Araneae	Dictynidae	*Emblyna sublata*	ARONT679-10	HQ924538
Araneae	Dictynidae	*Emblyna sublata*	ARONT680-10	HQ924539
Araneae	Dictynidae	*Emblyna sublata*	ARONT681-10	HQ924540
Araneae	Dictynidae	*Emblyna sublata*	ARONT682-10	HQ924541
Araneae	Dictynidae	*Emblyna sublata*	CNPPC2006-12	KJ085208
Araneae	Dictynidae	*Emblyna sublata*	CNSLB457-12	KM833635
Araneae	Dictynidae	*Emblyna sublata*	CNSLG289-12	KM839148
Araneae	Dictynidae	*Emblyna sublata*	CNSLG292-12	KM834162
Araneae	Dictynidae	*Emblyna sublata*	CNSLG298-12	KM836173
Araneae	Dictynidae	*Emblyna sublata*	CNSLH169-12	KM824859
Araneae	Dictynidae	*Emblyna sublata*	CNSLH170-12	KM840046
Araneae	Dictynidae	*Emblyna sublata*	CNSLH172-12	KM832774
Araneae	Dictynidae	*Emblyna sublata*	CNSLQ083-13	KM832555
Araneae	Dictynidae	*Emblyna sublata*	CNSLQ089-13	KM824947
Araneae	Dictynidae	*Emblyna sublata*	RBCH079-04	DQ127447
Araneae	Dictynidae	*Emblyna sublata*	RBINA019-13	KP655685
Araneae	Dictynidae	*Emblyna sublata*	RBINA070-13	KP654698
Araneae	Dictynidae	*Emblyna sublata*	RBINA1495-13	KP656056
Araneae	Dictynidae	*Emblyna sublata*	RBINA222-13	KP656934
Araneae	Dictynidae	*Emblyna sublata*	RBINA225-13	KP654623
Araneae	Dictynidae	*Emblyna sublata*	RBINA226-13	KP649510
Araneae	Dictynidae	*Emblyna sublata*	RBINA227-13	KP654555
Araneae	Dictynidae	*Emblyna sublata*	RBINA230-13	KP653079
Araneae	Dictynidae	*Emblyna sublata*	RBINA239-13	KP651617
Araneae	Dictynidae	*Emblyna sublata*	RBINA241-13	KP649519
Araneae	Dictynidae	*Emblyna sublata*	RBINA242-13	KP647021
Araneae	Dictynidae	*Emblyna sublata*	RBINA243-13	KP652055
Araneae	Dictynidae	*Emblyna sublata*	RBINA244-13	KP651879
Araneae	Dictynidae	*Emblyna sublata*	RBINA245-13	KP656633
Araneae	Dictynidae	*Emblyna sublata*	RBINA248-13	KP653136
Araneae	Dictynidae	*Emblyna sublata*	RBINA250-13	KP649878
Araneae	Dictynidae	*Emblyna sublata*	RBINA253-13	KP649545
Araneae	Dictynidae	*Emblyna sublata*	RBINA254-13	KP656656
Araneae	Dictynidae	*Emblyna sublata*	RBINA257-13	KP647189
Araneae	Dictynidae	*Hackmania prominula*	SPICH097-09	HM432624
Araneae	Dictynidae	*Hackmania saphes*	SSBAA5276-12	KM830608
Araneae	Diguetidae	*Diguetia sp. BB-2004*	GBCH0805-06	AY560795
Araneae	Dipluridae	*Euagrus chisoseus*	GBCH4035-09	FJ607564
Araneae	Dipluridae	*Phyxioschema suthepium*	GBCH10811-13	NC_020322
Araneae	Dipluridae	*Phyxioschema suthepium*	GBCH11968-13	JQ407802
Araneae	Dysderidae	*Cryptoparachtes*	GBCH7245-13	JN689136
Araneae	Dysderidae	*Dysdera alegranzaensis*	GBCH3183-08	EU139632

Araneae	Dysderidae	*Dysdera alegranzaensis*	GBCH3184-08	EU139631
Araneae	Dysderidae	*Dysdera alegranzaensis*	GBCH3199-08	EU139614
Araneae	Dysderidae	*Dysdera crocata*	ARONT178-09	HM434054
Araneae	Dysderidae	*Dysdera crocata*	GBCH12418-13	JX017359
Araneae	Dysderidae	*Dysdera crocata*	GBCH7752-13	JN018196
Araneae	Dysderidae	*Dysdera crocata*	RBCH049-04	DQ127481
Araneae	Dysderidae	*Dysdera crocata*	SPRMA008-10	HQ977119
Araneae	Dysderidae	*Dysdera crocata*	SPRMA696-10	JF887130
Araneae	Dysderidae	*Dysdera erythrina*	GBCH4963-10	GQ285643
Araneae	Dysderidae	*Dysdera erythrina*	GBCH4964-10	GQ285642
Araneae	Dysderidae	*Dysdera erythrina*	GBCH4976-10	GQ285630
Araneae	Dysderidae	*Dysdera erythrina lantosquensis*	GBCH4975-10	GQ285631
Araneae	Dysderidae	*Dysdera gibbifera*	GBCH3675-09	EU068034
Araneae	Dysderidae	*Dysdera gibbifera*	GBCH3676-09	EU068033
Araneae	Dysderidae	*Dysdera hernandezi*	GBCH3672-09	EU068037
Araneae	Dysderidae	*Dysdera levipes*	GBCH3668-09	EU068041
Araneae	Dysderidae	*Dysdera lusitanica*	GBCH4977-10	GQ285629
Araneae	Dysderidae	*Dysdera madai*	GBCH3666-09	EU068043
Araneae	Dysderidae	*Dysdera nesiotes*	GBCH0132-06	AF244267
Araneae	Dysderidae	*Dysdera nesiotes*	GBCH3188-08	EU139625
Araneae	Dysderidae	*Dysdera nesiotes*	GBCH3190-08	EU139623
Araneae	Dysderidae	*Dysdera nesiotes*	GBCH3191-08	EU139622
Araneae	Dysderidae	*Dysdera nesiotes*	GBCH3192-08	EU139621
Araneae	Dysderidae	*Dysdera nesiotes*	GBCH3193-08	EU139620
Araneae	Dysderidae	*Dysdera ratonensis*	GBCH3656-09	EU068054
Araneae	Dysderidae	*Dysdera ratonensis*	GBCH3657-09	EU068053
Araneae	Dysderidae	*Dysdera ratonensis*	GBCH3658-09	EU068052
Araneae	Dysderidae	*Dysdera ratonensis*	GBCH3659-09	EU068051
Araneae	Dysderidae	*Dysdera ratonensis*	GBCH3660-09	EU068050
Araneae	Dysderidae	*Dysdera ratonensis*	GBCH3661-09	EU068048
Araneae	Dysderidae	*Dysdera sanborondon*	GBCH3187-08	EU139626
Araneae	Dysderidae	*Dysdera shardana*	GBCH4965-10	GQ285641
Araneae	Dysderidae	*Dysdera shardana*	GBCH4966-10	GQ285640
Araneae	Dysderidae	*Dysdera shardana*	GBCH4967-10	GQ285639
Araneae	Dysderidae	*Dysdera shardana*	GBCH4968-10	GQ285638
Araneae	Dysderidae	*Dysdera shardana*	GBCH4969-10	GQ285637
Araneae	Dysderidae	*Dysdera shardana*	GBCH4970-10	GQ285636
Araneae	Dysderidae	*Dysdera shardana*	GBCH4971-10	GQ285635
Araneae	Dysderidae	*Dysdera shardana*	GBCH4972-10	GQ285634
Araneae	Dysderidae	*Dysdera shardana*	GBCH4973-10	GQ285633
Araneae	Dysderidae	*Dysdera spinidorsa*	GBCH3185-08	EU139628
Araneae	Dysderidae	*Dysdera spinidorsa*	GBCH3186-08	EU139627
Araneae	Dysderidae	*Dysdera valentina*	GBCH4974-10	GQ285632
Araneae	Dysderidae	*Holissus unciger*	GBCH7238-13	JN689149
Araneae	Dysderidae	*Parachtes*	GBCH7210-13	JN689177
Araneae	Dysderidae	*Parachtes*	GBCH7226-13	JN689161
Araneae	Dysderidae	*Parachtes andreinii*	GBCH7212-13	JN689175
Araneae	Dysderidae	*Parachtes deminutus*	GBCH7213-13	JN689174
Araneae	Dysderidae	*Parachtes deminutus*	GBCH7214-13	JN689173
Araneae	Dysderidae	*Parachtes ignavus*	GBCH7236-13	JN689151
Araneae	Dysderidae	*Parachtes limbarae*	GBCH7215-13	JN689172
Araneae	Dysderidae	*Parachtes limbarae*	GBCH7233-13	JN689154
Araneae	Dysderidae	*Parachtes limbarae*	GBCH7234-13	JN689153

Araneae	Dysderidae	*Parachtes siculus*	GBCH7216-13	JN689171
Araneae	Dysderidae	*Parachtes teruelis*	GBCH7217-13	JN689170
Araneae	Dysderidae	*Parachtes teruelis*	GBCH7218-13	JN689169
Araneae	Dysderidae	*Parachtes teruelis*	GBCH7222-13	JN689165
Araneae	Dysderidae	*Parachtes vernae*	GBCH7211-13	JN689176
Araneae	Eresidae	*Stegodyphus*	GBCH11423-13	JX240234
Araneae	Eutichuridae	*Cheiracanthium inclusum*	SPRMA686-10	JF887123
Araneae	Eutichuridae	*Cheiracanthium japonicum*	GBCH11052-13	JN817218
Araneae	Eutichuridae	*Cheiracanthium mildei*	ARONT051-09	GU682807
Araneae	Eutichuridae	*Cheiracanthium mildei*	ARONT129-09	GU682650
Araneae	Eutichuridae	*Cheiracanthium mildei*	ARONT139-09	GU682647
Araneae	Eutichuridae	*Cheiracanthium mildei*	ARONT142-09	GU682651
Araneae	Eutichuridae	*Cheiracanthium mildei*	ARONT143-09	GU682648
Araneae	Eutichuridae	*Cheiracanthium mildei*	ARONT144-09	GU682644
Araneae	Eutichuridae	*Cheiracanthium mildei*	ARONT145-09	GU682645
Araneae	Eutichuridae	*Cheiracanthium mildei*	ARONT146-09	GU682642
Araneae	Eutichuridae	*Cheiracanthium mildei*	ARONT147-09	GU682643
Araneae	Eutichuridae	*Cheiracanthium mildei*	ARONT149-09	GU682640
Araneae	Eutichuridae	*Cheiracanthium mildei*	ARONT150-09	GU682641
Araneae	Eutichuridae	*Cheiracanthium mildei*	ARONT172-09	GU682649
Araneae	Eutichuridae	*Cheiracanthium mildei*	ARONT173-09	GU682646
Araneae	Eutichuridae	*Cheiracanthium mildei*	ARONT174-09	GU682639
Araneae	Eutichuridae	*Cheiracanthium mildei*	ARONT328-09	GU682854
Araneae	Eutichuridae	*Cheiracanthium mildei*	ARONT378-09	GU682917
Araneae	Eutichuridae	*Cheiracanthium mildei*	ARONT731-10	HQ924585
Araneae	Eutichuridae	*Cheiracanthium mildei*	RBCH145-04	DQ127387
Araneae	Eutichuridae	*Cheiracanthium mildei*	SMTPB1033-13	KP647458
Araneae	Eutichuridae	*Cheiracanthium mildei*	SMTPB10362-13	KP652877
Araneae	Eutichuridae	*Cheiracanthium mildei*	SMTPB10363-13	KP648327
Araneae	Eutichuridae	*Cheiracanthium mildei*	SMTPB10423-13	KP650265
Araneae	Eutichuridae	*Cheiracanthium mildei*	SMTPB109-13	KP646760
Araneae	Eutichuridae	*Cheiracanthium mildei*	SMTPB110-13	KP648003
Araneae	Eutichuridae	*Cheiracanthium mildei*	SMTPB13745-13	KP655491
Araneae	Eutichuridae	*Cheiracanthium mildei*	SMTPB1382-13	KP656918
Araneae	Eutichuridae	*Cheiracanthium mildei*	SMTPB1383-13	KP653009
Araneae	Eutichuridae	*Cheiracanthium mildei*	SMTPB1384-13	KP646236
Araneae	Eutichuridae	*Cheiracanthium mildei*	SMTPB15356-13	KP652557
Araneae	Eutichuridae	*Cheiracanthium mildei*	SMTPB16639-13	KP648852
Araneae	Eutichuridae	*Cheiracanthium mildei*	SMTPB16662-13	KP648186
Araneae	Eutichuridae	*Cheiracanthium mildei*	SMTPB18189-13	KP651005
Araneae	Eutichuridae	*Cheiracanthium mildei*	SMTPB18190-13	KP648750
Araneae	Eutichuridae	*Cheiracanthium mildei*	SMTPB18191-13	KP656874
Araneae	Eutichuridae	*Cheiracanthium mildei*	SMTPB18192-13	KP652471
Araneae	Eutichuridae	*Cheiracanthium mildei*	SMTPB18200-13	KP648568
Araneae	Eutichuridae	*Cheiracanthium mildei*	SMTPB18201-13	KP646859
Araneae	Eutichuridae	*Cheiracanthium mildei*	SMTPB18912-13	KP653537
Araneae	Eutichuridae	*Cheiracanthium mildei*	SMTPB18927-13	KP653269
Araneae	Eutichuridae	*Cheiracanthium mildei*	SMTPB21249-13	KP656689
Araneae	Eutichuridae	*Cheiracanthium mildei*	SMTPB2379-13	KP651517
Araneae	Eutichuridae	*Cheiracanthium mildei*	SMTPB2380-13	KP648446
Araneae	Eutichuridae	*Cheiracanthium mildei*	SMTPB2381-13	KP657367
Araneae	Eutichuridae	*Cheiracanthium mildei*	SMTPB5073-13	KP655618
Araneae	Eutichuridae	*Cheiracanthium mildei*	SMTPB5226-13	KP651417

Araneae	Eutichuridae	*Cheiracanthium mildei*	SMTPB5227-13	KP648482
Araneae	Eutichuridae	*Cheiracanthium mildei*	SMTPB5986-13	KP650395
Araneae	Eutichuridae	*Cheiracanthium mildei*	SMTPB5987-13	KP647099
Araneae	Eutichuridae	*Cheiracanthium mildei*	SMTPB6040-13	KP648387
Araneae	Eutichuridae	*Cheiracanthium mildei*	SMTPB7321-13	KP650855
Araneae	Eutichuridae	*Cheiracanthium mildei*	SMTPB7423-13	KP656857
Araneae	Eutichuridae	*Cheiracanthium mildei*	SMTPB7501-13	KP654601
Araneae	Eutichuridae	*Cheiracanthium mildei*	SMTPB9780-13	KP648217
Araneae	Eutichuridae	*Cheiracanthium mildei*	SMTPB9781-13	KP646294
Araneae	Eutichuridae	*Cheiracanthium mildei*	SMTPB9782-13	KP649923
Araneae	Eutichuridae	*Cheiracanthium mildei*	SPRMA534-10	JF887046
Araneae	Eutichuridae	*Cheiracanthium mildei*	SPRMA685-10	JF887122
Araneae	Eutichuridae	*Cheiracanthium uncinatum*	GBCH11051-13	JN817219
Araneae	Eutichuridae	*Strotarchus piscatorius*	CNSLH310-12	KM824524
Araneae	Filistatidae	*Kukulcania hibernalis*	GBCH0806-06	AY560796
Araneae	Filistatidae	*Kukulcania hibernalis*	GBCH11424-13	JX240233
Araneae	Gnaphosidae	*Callilepis pluto*	CNPPE2326-12	KJ167562
Araneae	Gnaphosidae	*Callilepis pluto*	CNPPF1464-12	KJ167190
Araneae	Gnaphosidae	*Callilepis pluto*	CNPPF1465-12	KJ168035
Araneae	Gnaphosidae	*Cesonia bilineata*	SPRMA601-10	JF887082
Araneae	Gnaphosidae	*Drassodes lapidosus*	GBCH0808-06	AY560798
Araneae	Gnaphosidae	*Drassodes mirus*	SPISH001-09	GU683947
Araneae	Gnaphosidae	*Drassodes neglectus*	SPICH1162-09	GU684591
Araneae	Gnaphosidae	*Drassodes neglectus*	SPRMA135-10	HQ580764
Araneae	Gnaphosidae	*Drassodes neglectus*	SPRMA144-10	HQ580772
Araneae	Gnaphosidae	*Drassodes neglectus*	SPRMA172-10	HQ580792
Araneae	Gnaphosidae	*Drassodes neglectus*	SPRMA600-10	JF887081
Araneae	Gnaphosidae	*Drassodes serratidens*	GBCH11045-13	JN817225
Araneae	Gnaphosidae	*Drassyllus*	TDWGB837-10	HQ979262
Araneae	Gnaphosidae	*Drassyllus*	TDWGB933-10	HQ979353
Araneae	Gnaphosidae	*Drassyllus*	TDWGB934-10	HQ979354
Araneae	Gnaphosidae	*Drassyllus biglobus*	GBMIN12198-13	JN817226
Araneae	Gnaphosidae	*Drassyllus depressus*	ARONT165-09	GU682636
Araneae	Gnaphosidae	*Drassyllus depressus*	ARONT168-09	GU682633
Araneae	Gnaphosidae	*Drassyllus depressus*	ARONT743-10	HQ924596
Araneae	Gnaphosidae	*Drassyllus depressus*	ERSPI036-08	KP655670
Araneae	Gnaphosidae	*Drassyllus depressus*	ERSPI279-09	GU682547
Araneae	Gnaphosidae	*Drassyllus depressus*	ERSPI280-09	GU682548
Araneae	Gnaphosidae	*Drassyllus depressus*	ERSPI344-09	GU682495
Araneae	Gnaphosidae	*Drassyllus depressus*	SMTPB21287-13	KP646323
Araneae	Gnaphosidae	*Drassyllus depressus*	SMTPB4785-13	KP652040
Araneae	Gnaphosidae	*Drassyllus depressus*	SPRMA606-10	KP650405
Araneae	Gnaphosidae	*Drassyllus dromeus*	SPRMA605-10	JF887083
Araneae	Gnaphosidae	*Drassyllus insularis*	SPRMA938-12	KP649465
Araneae	Gnaphosidae	*Drassyllus niger*	ERSPI263-09	HM376100
Araneae	Gnaphosidae	*Drassyllus niger*	RBINA3422-13	KP649568
Araneae	Gnaphosidae	*Drassyllus niger*	RBINA3425-13	KP653226
Araneae	Gnaphosidae	*Drassyllus niger*	RBINA3429-13	KP645673
Araneae	Gnaphosidae	*Gnaphosa*	TDWGB940-10	HQ979360
Araneae	Gnaphosidae	*Gnaphosa borea*	JDTGS009-09	GU679903
Araneae	Gnaphosidae	*Gnaphosa brumalis*	SPICH213-09	GU683954
Araneae	Gnaphosidae	*Gnaphosa kompirensis*	GBCH11044-13	JN817227
Araneae	Gnaphosidae	*Gnaphosa microps*	KKCHE755-07	KF368100

Araneae	Gnaphosidae	*Gnaphosa muscorum*	SPICH047-09	GU683906
Araneae	Gnaphosidae	*Gnaphosa muscorum*	SPRMA150-10	HQ580776
Araneae	Gnaphosidae	*Gnaphosa muscorum*	SPRMA159-10	HQ580782
Araneae	Gnaphosidae	*Gnaphosa muscorum*	SPRMA170-10	HQ580790
Araneae	Gnaphosidae	*Gnaphosa muscorum*	SPRMA609-10	JF887084
Araneae	Gnaphosidae	*Gnaphosa muscorum*	SPRMA610-10	JF887085
Araneae	Gnaphosidae	*Gnaphosa muscorum*	SPRMA611-10	JF887086
Araneae	Gnaphosidae	*Gnaphosa orites*	SPIRU1298-11	KF368103
Araneae	Gnaphosidae	*Gnaphosa parvula*	ARONT410-09	GU682914
Araneae	Gnaphosidae	*Gnaphosa parvula*	ERSPI271-09	GU682550
Araneae	Gnaphosidae	*Gnaphosa parvula*	ERSPI339-09	GU682500
Araneae	Gnaphosidae	*Gnaphosa parvula*	RBCH002-04	DQ127525
Araneae	Gnaphosidae	*Gnaphosa parvula*	RBCH022-04	DQ127502
Araneae	Gnaphosidae	*Gnaphosa snohomish*	SPRMA615-10	JF887090
Araneae	Gnaphosidae	*Haplodrassus bicornis*	SPRMA614-10	JF887089
Araneae	Gnaphosidae	*Haplodrassus eunis*	SSBAA5229-12	KM835811
Araneae	Gnaphosidae	*Haplodrassus eunis*	SSBAA5243-12	KM824773
Araneae	Gnaphosidae	*Haplodrassus eunis*	SSBAA5250-12	KM837014
Araneae	Gnaphosidae	*Haplodrassus eunis*	SSBAA5251-12	KM838127
Araneae	Gnaphosidae	*Haplodrassus eunis*	SSBAA5254-12	KM836918
Araneae	Gnaphosidae	*Haplodrassus eunis*	SSBAA5285-12	KM838561
Araneae	Gnaphosidae	*Haplodrassus hiemalis*	SPISH036-09	GU683958
Araneae	Gnaphosidae	*Haplodrassus signifer*	ERSPI254-09	GU682568
Araneae	Gnaphosidae	*Haplodrassus signifer*	SPICH145-09	GU683772
Araneae	Gnaphosidae	*Haplodrassus signifer*	SPRMA347-10	HQ580905
Araneae	Gnaphosidae	*Haplodrassus signifer*	SPRMA612-10	JF887087
Araneae	Gnaphosidae	*Haplodrassus signifer*	SPRMA613-10	JF887088
Araneae	Gnaphosidae	*Herpyllus ecclesiasticus*	ARONT345-09	GU682872
Araneae	Gnaphosidae	*Herpyllus ecclesiasticus*	CNBPI537-13	KM828525
Araneae	Gnaphosidae	*Herpyllus ecclesiasticus*	CNSLG306-12	KM828322
Araneae	Gnaphosidae	*Herpyllus ecclesiasticus*	ERSPI248-09	GU682569
Araneae	Gnaphosidae	*Herpyllus ecclesiasticus*	JSARA054-11	KP647722
Araneae	Gnaphosidae	*Herpyllus ecclesiasticus*	RBCH124-04	DQ127412
Araneae	Gnaphosidae	*Micaria aenea*	SPICH157-09	GU683781
Araneae	Gnaphosidae	*Micaria alpina*	SPICH990-09	GU684496
Araneae	Gnaphosidae	*Micaria constricta*	KKCHE1001-09	GU683690
Araneae	Gnaphosidae	*Micaria constricta*	SPRMA122-10	HQ580756
Araneae	Gnaphosidae	*Micaria constricta*	SPRMA194-10	HQ580808
Araneae	Gnaphosidae	*Micaria dives*	GBCH11043-13	JN817228
Araneae	Gnaphosidae	*Micaria elizabethae*	ERSPI341-09	HM376105
Araneae	Gnaphosidae	*Micaria elizabethae*	RBINA4372-13	KP647474
Araneae	Gnaphosidae	*Micaria foxi*	SPRMA624-10	JF887091
Araneae	Gnaphosidae	*Micaria foxi*	SPRMA625-10	JF887092
Araneae	Gnaphosidae	*Micaria foxi*	SPRMA826-12	KP650704
Araneae	Gnaphosidae	*Micaria pulicaria*	CNGBB534-13	KP647739
Araneae	Gnaphosidae	*Micaria pulicaria*	CNGBI1547-14	KP654086
Araneae	Gnaphosidae	*Micaria pulicaria*	CNGBI1551-14	KP651997
Araneae	Gnaphosidae	*Micaria pulicaria*	CNGBJ1682-14	KP647392
Araneae	Gnaphosidae	*Micaria pulicaria*	CNGBJ1684-14	KP657409
Araneae	Gnaphosidae	*Micaria pulicaria*	CNGBJ1702-14	KP647304
Araneae	Gnaphosidae	*Micaria pulicaria*	SPICH215-09	GU683944
Araneae	Gnaphosidae	*Micaria pulicaria*	SSJAE5583-13	KM825512
Araneae	Gnaphosidae	*Micaria pulicaria*	SSPAC10083-13	KM830329

Araneae	Gnaphosidae	*Micaria pulicaria*	SSPAC10979-13	KM839136
Araneae	Gnaphosidae	*Micaria pulicaria*	SSWLD2595-13	KM827168
Araneae	Gnaphosidae	*Nodocion mateonus*	CNGRF421-12	KM836659
Araneae	Gnaphosidae	*Orodrassus canadensis*	CNGLC175-13	KM825259
Araneae	Gnaphosidae	*Orodrassus canadensis*	CNJAG1007-12	KM828187
Araneae	Gnaphosidae	*Orodrassus coloradensis*	SPRMA124-10	HQ580758
Araneae	Gnaphosidae	*Orodrassus coloradensis*	SPRMA151-10	KP656043
Araneae	Gnaphosidae	*Orodrassus coloradensis*	SPRMA165-10	HQ580786
Araneae	Gnaphosidae	*Scotophaeus blackwalli*	SPRMA695-10	JF887129
Araneae	Gnaphosidae	*Scotophaeus blackwalli*	SPRMA742-10	JF887159
Araneae	Gnaphosidae	*Sergiolus columbianus*	SPRMA626-10	JF887093
Araneae	Gnaphosidae	*Sergiolus columbianus*	SPRMA627-10	KR070210
Araneae	Gnaphosidae	*Sergiolus columbianus*	SPRMA628-10	JF887094
Araneae	Gnaphosidae	*Sergiolus montanus*	CNWLF011-12	KM834495
Araneae	Gnaphosidae	*Sergiolus montanus*	SPRMA809-12	KP656031
Araneae	Gnaphosidae	*Sergiolus montanus*	SSEIB1948-13	KM828092
Araneae	Gnaphosidae	*Sergiolus montanus*	SSJAB3538-13	KM831862
Araneae	Gnaphosidae	*Sosticus insularis*	SPRMA629-10	JF887095
Araneae	Gnaphosidae	*Trachyzelotes jaxartensis*	GBCH11041-13	JN817230
Araneae	Gnaphosidae	*Zelotes fratris*	ARONT802-10	HQ924647
Araneae	Gnaphosidae	*Zelotes fratris*	CNSLI111-12	KM838356
Araneae	Gnaphosidae	*Zelotes fratris*	CNSLI533-12	KM824993
Araneae	Gnaphosidae	*Zelotes fratris*	ERSPI010-08	KP646377
Araneae	Gnaphosidae	*Zelotes fratris*	SPIAL078-10	HQ580582
Araneae	Gnaphosidae	*Zelotes fratris*	SPRMA123-10	HQ580757
Araneae	Gnaphosidae	*Zelotes fratris*	SPRMA179-10	HQ580797
Araneae	Gnaphosidae	*Zelotes fratris*	SPRMA192-10	HQ580806
Araneae	Gnaphosidae	*Zelotes fratris*	SPRMA210-10	HQ580824
Araneae	Gnaphosidae	*Zelotes fratris*	SPRMA292-10	HQ977165
Araneae	Gnaphosidae	*Zelotes fratris*	SPRMA631-10	JF887096
Araneae	Gnaphosidae	*Zelotes fratris*	SPRMA632-10	JF887097
Araneae	Gnaphosidae	*Zelotes fratris*	SPRMA633-10	KP652417
Araneae	Gnaphosidae	*Zelotes fratris*	SPRMA640-10	JF887099
Araneae	Gnaphosidae	*Zelotes puritanus*	SPRMA121-10	HQ580755
Araneae	Gnaphosidae	*Zelotes puritanus*	SPRMA193-10	HQ580807
Araneae	Gnaphosidae	*Zelotes puritanus*	SPRMA638-10	JF887098
Araneae	Gnaphosidae	*Zelotes sula*	KKCHE082-06	KF369090
Araneae	Gnaphosidae	*Zelotes tuobus*	SPRMA160-10	HQ580783
Araneae	Gnaphosidae	*Zelotes tuobus*	SPRMA641-10	JF887100
Araneae	Hahniidae	*Antistea brunnea*	SPIAL104-10	HQ580602
Araneae	Hahniidae	*Antistea brunnea*	SPIAL116-10	HQ580611
Araneae	Hahniidae	*Antistea brunnea*	SPIAL129-10	HQ580619
Araneae	Hahniidae	*Antistea brunnea*	SPIAL141-10	HQ580626
Araneae	Hahniidae	*Antistea brunnea*	SPIAL153-10	HQ580632
Araneae	Hahniidae	*Antistea brunnea*	SPIAL167-10	HQ580641
Araneae	Hahniidae	*Antistea brunnea*	SPIAL232-10	HQ580682
Araneae	Hahniidae	*Calymmaria nana*	SPRMA276-10	HQ580876
Araneae	Hahniidae	*Cryphoeca exlineae*	SPIAL197-10	HQ580657
Araneae	Hahniidae	*Cryphoeca exlineae*	SPIAL209-10	HQ580666
Araneae	Hahniidae	*Cryphoeca exlineae*	SPIAL221-10	HQ580675
Araneae	Hahniidae	*Cryphoeca exlineae*	SPIAL268-10	HQ580711
Araneae	Hahniidae	*Cryphoeca exlineae*	SPIAL280-10	HQ580720
Araneae	Hahniidae	*Cryphoeca exlineae*	SPRMA184-10	HQ580801

Araneae	Hahniidae	*Cryphoeca exlineae*	SPRMA250-10	HQ580856
Araneae	Hahniidae	*Cryphoeca exlineae*	SPRMA289-10	HQ977162
Araneae	Hahniidae	*Cryphoeca exlineae*	SSWLC3110-13	KM838516
Araneae	Hahniidae	*Cryphoeca exlineae*	SSWLF4870-13	KM829101
Araneae	Hahniidae	*Cryphoeca montana*	ARONT546-10	HQ924433
Araneae	Hahniidae	*Cryphoeca sp. 1 JCS-2006*	GBCH1524-06	DQ628614
Araneae	Hahniidae	*Dirksia cinctipes*	CNPCL020-13	KM829266
Araneae	Hahniidae	*Dirksia cinctipes*	SPIAL054-10	HQ580561
Araneae	Hahniidae	*Dirksia cinctipes*	SPIAL066-10	HQ580571
Araneae	Hahniidae	*Dirksia cinctipes*	SPIAL196-10	KR069873
Araneae	Hahniidae	*Dirksia cinctipes*	SPIAL267-10	HQ580710
Araneae	Hahniidae	*Dirksia cinctipes*	SPIAL279-10	HQ580719
Araneae	Hahniidae	*Dirksia cinctipes*	SPRMA235-10	HQ580842
Araneae	Hahniidae	*Dirksia cinctipes*	SPRMA236-10	HQ580843
Araneae	Hahniidae	*Dirksia cinctipes*	SPRMA247-10	HQ580853
Araneae	Hahniidae	*Dirksia cinctipes*	SPRMA676-10	KP646037
Araneae	Hahniidae	*Ethobuella tuonops*	CNPCL024-13	KM830133
Araneae	Hahniidae	*Ethobuella tuonops*	CNPCN056-13	KM829751
Araneae	Hahniidae	*Ethobuella tuonops*	SPIAL105-10	HQ580603
Araneae	Hahniidae	*Ethobuella tuonops*	SPIAL152-10	HQ580631
Araneae	Hahniidae	*Ethobuella tuonops*	SPIAL208-10	HQ580665
Araneae	Hahniidae	*Ethobuella tuonops*	SPIAL220-10	HQ580674
Araneae	Hahniidae	*Ethobuella tuonops*	SPIAL244-10	HQ580692
Araneae	Hahniidae	*Ethobuella tuonops*	SPIAL256-10	HQ580701
Araneae	Hahniidae	*Ethobuella tuonops*	SPRMA225-10	HQ580836
Araneae	Hahniidae	*Ethobuella tuonops*	SPRMA246-10	HQ580852
Araneae	Hahniidae	*Ethobuella tuonops*	SPRMA324-10	HQ580891
Araneae	Hahniidae	*Ethobuella tuonops*	SPRMA484-10	JF887021
Araneae	Hahniidae	*Hahnia cinerea*	SPISH024-09	GU683968
Araneae	Hahniidae	*Hahnia cinerea*	SSJAB2025-13	KM824967
Araneae	Hahniidae	*Hahnia cinerea*	SSJAE11529-13	KM839424
Araneae	Hahniidae	*Hahnia cinerea*	SSJAE11557-13	KM836471
Araneae	Hahniidae	*Hahnia cinerea*	SSJAE4212-13	KM837922
Araneae	Hahniidae	*Hahnia glacialis*	SSJAD2547-13	KM829983
Araneae	Hahniidae	*Hahnia sp. 1GAB*	TDWGB777-10	HQ979220
Araneae	Hahniidae	*Neoantistea agilis*	SPRMA145-10	HQ580773
Araneae	Hahniidae	*Neoantistea gosiuta*	RBINA2376-13	KP651023
Araneae	Hahniidae	*Neoantistea gosiuta*	RBINA5130-13	KP650624
Araneae	Hahniidae	*Neoantistea gosiuta*	SPRMA976-12	KP654212
Araneae	Hahniidae	*Neoantistea magna*	ERSPI067-08	KP648517
Araneae	Hahniidae	*Neoantistea magna*	ERSPI069-08	KP652153
Araneae	Hahniidae	*Neoantistea magna*	ERSPI372-09	GU682475
Araneae	Hahniidae	*Neoantistea magna*	SPRMA204-10	HQ580818
Araneae	Hahniidae	*Neoantistea magna*	SPRMA220-10	HQ580831
Araneae	Hahniidae	*Neoantistea magna*	SPRMA222-10	HQ580833
Araneae	Hahniidae	*Neoantistea quelpartensis*	GBCH11064-13	JN817206
Araneae	Huttoniidae	*Huttonia*	GBCH11420-13	JX240237
Araneae	Hypochilidae	*Hypochilus thorelli*	GBCH0190-06	AF303510
Araneae	Hypochilidae	*Hypochilus thorelli*	GBCH2390-08	EU523753
Araneae	Hypochilidae	*Hypochilus thorelli*	GBCH3210-08	NC_010777
Araneae	Leptonetidae	*Leptoneta coreana*	GBCH11202-13	JN817068
Araneae	Leptonetidae	*Leptoneta hwanseonensis*	GBCH11201-13	JN817069
Araneae	Linyphiidae	*Acanthoneta aggressa*	SSBAE4087-13	KM836086

Araneae	Linyphiidae	*Acanthoneta aggressa*	SSBAE5755-13	KM826711
Araneae	Linyphiidae	*Agyneta allosubtilis*	SPICH004-09	GU683853
Araneae	Linyphiidae	*Agyneta amersaxatilis*	SPICH1095-09	HM416913
Araneae	Linyphiidae	*Agyneta bucklei*	SPRMA1039-12	KP656292
Araneae	Linyphiidae	*Agyneta danielbelangeri*	SPRMA1144-12	KP653192
Araneae	Linyphiidae	*Agyneta danielbelangeri*	SPRMA166-10	HQ580787
Araneae	Linyphiidae	*Agyneta decorata*	SPRMA1051-12	KP652844
Araneae	Linyphiidae	*Agyneta decorata*	SPRMA734-10	JF887155
Araneae	Linyphiidae	*Agyneta fabra*	ARONT655-10	HQ924520
Araneae	Linyphiidae	*Agyneta fabra*	ARONT689-10	HQ924548
Araneae	Linyphiidae	*Agyneta fabra*	CNEIB1685-12	KM833327
Araneae	Linyphiidae	*Agyneta fabra*	JSARA066-11	KP650365
Araneae	Linyphiidae	*Agyneta fabra*	RBINA1095-13	KP652486
Araneae	Linyphiidae	*Agyneta fabra*	RBINA1186-13	KP650829
Araneae	Linyphiidae	*Agyneta fabra*	RBINA1641-13	KP653018
Araneae	Linyphiidae	*Agyneta fabra*	RBINA1642-13	KP652867
Araneae	Linyphiidae	*Agyneta fabra*	RBINA2379-13	KP646365
Araneae	Linyphiidae	*Agyneta fabra*	RBINA2380-13	KP648613
Araneae	Linyphiidae	*Agyneta fabra*	RBINA3353-13	KP647413
Araneae	Linyphiidae	*Agyneta fabra*	RBINA3354-13	KP648144
Araneae	Linyphiidae	*Agyneta fabra*	RBINA5300-13	KP646986
Araneae	Linyphiidae	*Agyneta fillmorana*	CNJAE769-12	KM827074
Araneae	Linyphiidae	*Agyneta fillmorana cf.*	SPRMA916-12	KP655699
Araneae	Linyphiidae	*Agyneta flibuscrocus*	SPRMA1143-12	KP654090
Araneae	Linyphiidae	*Agyneta flibuscrocus*	SPRMA188-10	HQ580805
Araneae	Linyphiidae	*Agyneta flibuscrocus*	SPRMA189-10	KP651807
Araneae	Linyphiidae	*Agyneta jacksoni*	SPICH745-09	GU684372
Araneae	Linyphiidae	*Agyneta lophophor*	SPRMA171-10	HQ580791
Araneae	Linyphiidae	*Agyneta lophophor*	SPRMA915-12	KP648928
Araneae	Linyphiidae	*Agyneta micaria*	RBINA1878-13	KP654499
Araneae	Linyphiidae	*Agyneta nigripes*	SSBAA5273-12	KM827873
Araneae	Linyphiidae	*Agyneta nigripes*	SSWLD3245-13	KM826148
Araneae	Linyphiidae	*Agyneta olivacea*	SPICH002-09	GU683856
Araneae	Linyphiidae	*Agyneta ordinaria*	SPRMA1167-12	KP656369
Araneae	Linyphiidae	*Agyneta perspicua*	SPRMA1025-12	KP655426
Araneae	Linyphiidae	*Agyneta protrudens*	SPRMA1220-12	KP648712
Araneae	Linyphiidae	*Agyneta serrata*	ARONT808-10	HQ924653
Araneae	Linyphiidae	*Agyneta serrata*	RBINA3886-13	KP648052
Araneae	Linyphiidae	*Agyneta serrata*	RBINA3899-13	KP656496
Araneae	Linyphiidae	*Agyneta simplex*	CNEIG009-12	KM827042
Araneae	Linyphiidae	*Agyneta simplex*	SPIAL166-10	HQ580640
Araneae	Linyphiidae	*Agyneta simplex*	SPIAL178-10	HQ580647
Araneae	Linyphiidae	*Agyneta simplex*	SPRMA887-12	KP649979
Araneae	Linyphiidae	*Agyneta simplex*	SSBAD5938-13	KM831639
Araneae	Linyphiidae	*Agyneta simplex*	SSBAD5941-13	KM826339
Araneae	Linyphiidae	*Agyneta simplex*	SSJAB1979-13	KM840508
Araneae	Linyphiidae	*Agyneta simplex*	SSJAB2027-13	KM833337
Araneae	Linyphiidae	*Agyneta simplex*	SSJAE11569-13	KM832285
Araneae	Linyphiidae	*Agyneta simplex*	SSWLE2855-13	KM834746
Araneae	Linyphiidae	*Agyneta sp. 2GAB*	SSBAB2775-12	KM828403
Araneae	Linyphiidae	*Agyneta sp. 3RB*	SPRMA886-12	KP650295
Araneae	Linyphiidae	*Agyneta sp. 4GAB*	SPRMA1040-12	KP646169
Araneae	Linyphiidae	*Agyneta unimaculata*	ARONT032-09	GU682809

Araneae	Linyphiidae	*Agyneta unimaculata*	ARONT160-09	GU682590
Araneae	Linyphiidae	*Agyneta unimaculata*	ARONT161-09	GU682591
Araneae	Linyphiidae	*Agyneta unimaculata*	ERSPI328-09	GU682508
Araneae	Linyphiidae	*Agyneta unimaculata*	ERSPI329-09	GU682509
Araneae	Linyphiidae	*Agyneta unimaculata*	ERSPI330-09	GU682510
Araneae	Linyphiidae	*Agyneta unimaculata*	JSARA006-11	KP655201
Araneae	Linyphiidae	*Agyneta unimaculata*	JSARA067-11	KP646756
Araneae	Linyphiidae	*Agyneta watertoni*	SPRMA1056-12	KP649392
Araneae	Linyphiidae	*Allomengea dentisetis*	ARONT701-10	HQ924559
Araneae	Linyphiidae	*Allomengea dentisetis*	CNEIC3175-12	KM838844
Araneae	Linyphiidae	*Allomengea dentisetis*	KKCHE971-09	GU683668
Araneae	Linyphiidae	*Allomengea scopigera*	KKCHE896-09	KF367812
Araneae	Linyphiidae	*Anguliphantes nasus*	GBCH11155-13	JN817115
Araneae	Linyphiidae	*Arcuphantes pennatus*	GBCH11153-13	JN817117
Araneae	Linyphiidae	*Arcuphantes sylvaticus*	SPRMA1006-12	KP649166
Araneae	Linyphiidae	*Arcuphantes sylvaticus*	SPRMA114-10	HQ580752
Araneae	Linyphiidae	*Arcuphantes sylvaticus*	SPRMA846-12	KP651989
Araneae	Linyphiidae	*Asiceratinops kolymensis*	SPIRU1004-11	KF368808
Araneae	Linyphiidae	*Baryphyma trifrons*	SPIRU1007-11	KF367910
Araneae	Linyphiidae	*Baryphyma trifrons affine*	ARONT773-10	HQ924622
Araneae	Linyphiidae	*Baryphyma trifrons affine*	ARONT774-10	HQ924623
Araneae	Linyphiidae	*Baryphyma trifrons affine*	ARONT775-10	HQ924624
Araneae	Linyphiidae	*Baryphyma trifrons affine*	SPIRU1157-11	KF367918
Araneae	Linyphiidae	*Baryphyma trifrons affine*	SPRMA1048-12	KF408094
Araneae	Linyphiidae	*Bathyphantes alascensis*	SPRMA230-10	HQ580838
Araneae	Linyphiidae	*Bathyphantes alascensis*	SPRMA257-10	HQ580861
Araneae	Linyphiidae	*Bathyphantes alascensis*	SPRMA260-10	HQ580864
Araneae	Linyphiidae	*Bathyphantes alascensis*	SPRMA833-12	KP651297
Araneae	Linyphiidae	*Bathyphantes alboventris*	ARONT477-10	HQ924378
Araneae	Linyphiidae	*Bathyphantes alboventris*	ARONT478-10	HQ924379
Araneae	Linyphiidae	*Bathyphantes alboventris*	ARONT484-10	HQ924383
Araneae	Linyphiidae	*Bathyphantes alboventris*	ARONT625-10	HQ924490
Araneae	Linyphiidae	*Bathyphantes brevipes*	SPIAL198-10	HQ580658
Araneae	Linyphiidae	*Bathyphantes brevipes*	SPIAL210-10	HQ580667
Araneae	Linyphiidae	*Bathyphantes brevipes*	SPIAL222-10	HQ580676
Araneae	Linyphiidae	*Bathyphantes brevipes*	SPIAL281-10	HQ580721
Araneae	Linyphiidae	*Bathyphantes brevipes*	SPRMA072-10	KP647615
Araneae	Linyphiidae	*Bathyphantes brevipes*	SPRMA073-10	HQ977153
Araneae	Linyphiidae	*Bathyphantes brevis*	ARONT606-10	HQ924474
Araneae	Linyphiidae	*Bathyphantes brevis*	JSARA070-11	KP656916
Araneae	Linyphiidae	*Bathyphantes brevis*	SPICH1094-09	GU684638
Araneae	Linyphiidae	*Bathyphantes canadensis*	BBCAN388-09	GU683434
Araneae	Linyphiidae	*Bathyphantes canadensis*	SPIAL224-10	HQ580677
Araneae	Linyphiidae	*Bathyphantes canadensis*	SPICH292-09	GU684338
Araneae	Linyphiidae	*Bathyphantes eumenis*	SPICH872-09	HM416915
Araneae	Linyphiidae	*Bathyphantes eumenis*	SSGBB5821-14	KP655400
Araneae	Linyphiidae	*Bathyphantes eumenis*	SSPAA6011-13	KM839626
Araneae	Linyphiidae	*Bathyphantes eumenis*	SSPAA6088-13	KM834292
Araneae	Linyphiidae	*Bathyphantes eumenis*	SSPAA6492-13	KM835809
Araneae	Linyphiidae	*Bathyphantes eumenis*	SSPAA6494-13	KM829112
Araneae	Linyphiidae	*Bathyphantes eumenis*	SSPAA6496-13	KM837883
Araneae	Linyphiidae	*Bathyphantes eumenis*	SSPAA6497-13	KM830098
Araneae	Linyphiidae	*Bathyphantes eumenis*	SSPAA6498-13	KM833072

Araneae	Linyphiidae	*Bathyphantes eumenis*	SSPAA6499-13	KM839648
Araneae	Linyphiidae	*Bathyphantes eumenis*	SSPAA6500-13	KM827871
Araneae	Linyphiidae	*Bathyphantes eumenis*	SSPAA7518-13	KM840822
Araneae	Linyphiidae	*Bathyphantes eumenis*	SSPAA7522-13	KM838825
Araneae	Linyphiidae	*Bathyphantes eumenis*	SSPAA7535-13	KM830016
Araneae	Linyphiidae	*Bathyphantes eumenis*	SSPAA7560-13	KM829753
Araneae	Linyphiidae	*Bathyphantes eumenis*	SSPAA7578-13	KM838004
Araneae	Linyphiidae	*Bathyphantes eumenis*	SSPAA7580-13	KM838792
Araneae	Linyphiidae	*Bathyphantes eumenis*	SSPAA8758-13	KM828691
Araneae	Linyphiidae	*Bathyphantes eumenis*	SSPAA8760-13	KM828843
Araneae	Linyphiidae	*Bathyphantes eumenis*	SSPAA8762-13	KM838867
Araneae	Linyphiidae	*Bathyphantes eumenis*	SSPAA8765-13	KM840299
Araneae	Linyphiidae	*Bathyphantes eumenis*	SSPAA8767-13	KM840654
Araneae	Linyphiidae	*Bathyphantes eumenis*	SSPAA8769-13	KM835916
Araneae	Linyphiidae	*Bathyphantes gracilis*	CNPAR001-13	KM836935
Araneae	Linyphiidae	*Bathyphantes gracilis*	GBCH5128-10	FJ838650
Araneae	Linyphiidae	*Bathyphantes gracilis*	GBCH5722-13	FJ899798
Araneae	Linyphiidae	*Bathyphantes gracilis*	GBCH5723-13	FJ899797
Araneae	Linyphiidae	*Bathyphantes gracilis*	SPICH556-09	HM432632
Araneae	Linyphiidae	*Bathyphantes keeni*	CNGIB558-12	KM837070
Araneae	Linyphiidae	*Bathyphantes keeni*	CNPCB046-12	KM839945
Araneae	Linyphiidae	*Bathyphantes keeni*	CNPCB051-12	KM834501
Araneae	Linyphiidae	*Bathyphantes keeni*	SPIAL164-10	HQ580638
Araneae	Linyphiidae	*Bathyphantes keeni*	SPRMA081-10	HQ977154
Araneae	Linyphiidae	*Bathyphantes keeni*	SPRMA082-10	HQ977155
Araneae	Linyphiidae	*Bathyphantes keeni*	SPRMA083-10	HQ977156
Araneae	Linyphiidae	*Bathyphantes keeni*	SPRMA231-10	HQ580839
Araneae	Linyphiidae	*Bathyphantes keeni*	SPRMA241-10	HQ580847
Araneae	Linyphiidae	*Bathyphantes keeni*	SPRMA258-10	HQ580862
Araneae	Linyphiidae	*Bathyphantes orica*	SPRMA263-10	HQ580866
Araneae	Linyphiidae	*Bathyphantes pallidus*	BBCAN109-09	GU683269
Araneae	Linyphiidae	*Bathyphantes pallidus*	BBCAN189-09	GU683266
Araneae	Linyphiidae	*Bathyphantes pallidus*	BBCAN245-09	GU683174
Araneae	Linyphiidae	*Bathyphantes pallidus*	BBCAN738-09	GU683478
Araneae	Linyphiidae	*Bathyphantes pallidus*	BBCAN753-09	GU683014
Araneae	Linyphiidae	*Bathyphantes pallidus*	SPISH038-09	GU683961
Araneae	Linyphiidae	*Bathyphantes pallidus*	SSEIA3216-13	KM837259
Araneae	Linyphiidae	*Bathyphantes pallidus*	SSPAA7548-13	KM828908
Araneae	Linyphiidae	*Bathyphantes pallidus*	SSPAA8759-13	KM829385
Araneae	Linyphiidae	*Bathyphantes pallidus*	SSPAA8773-13	KM827718
Araneae	Linyphiidae	*Bathyphantes pallidus*	SSPAB8267-13	KM829606
Araneae	Linyphiidae	*Bathyphantes pallidus*	SSPAB8283-13	KM828830
Araneae	Linyphiidae	*Bathyphantes pallidus*	SSPAB8297-13	KM823986
Araneae	Linyphiidae	*Bathyphantes pallidus*	SSPAB8326-13	KM838204
Araneae	Linyphiidae	*Bathyphantes pallidus*	SSPAC10089-13	KM826476
Araneae	Linyphiidae	*Bathyphantes pallidus*	SSPAC10975-13	KM831075
Araneae	Linyphiidae	*Bathyphantes pallidus*	SSPAC10989-13	KM830536
Araneae	Linyphiidae	*Bathyphantes reprobus*	SPICH1033-09	GU684721
Araneae	Linyphiidae	*Bolyphantes alticeps*	GBCH0411-06	AY078691
Araneae	Linyphiidae	*Carorita limnaea*	SSPAA6460-13	KM840384
Araneae	Linyphiidae	*Carorita limnaea*	SSPAA6461-13	KM829896
Araneae	Linyphiidae	*Carorita limnaea*	SSPAA7508-13	KM831479
Araneae	Linyphiidae	*Carorita limnaea*	SSPAA7533-13	KM826728

Araneae	Linyphiidae	*Carorita limnaea*	SSPAA7561-13	KM838590
Araneae	Linyphiidae	*Carorita limnaea*	SSPAA7575-13	KM833406
Araneae	Linyphiidae	*Carorita limnaea*	SSPAA7577-13	KM833934
Araneae	Linyphiidae	*Centromerita bicolor*	GACAC530-12	GU323030
Araneae	Linyphiidae	*Centromerita bicolor*	GBCH5720-13	FJ899800
Araneae	Linyphiidae	*Centromerita bicolor*	GBCH5721-13	FJ899799
Araneae	Linyphiidae	*Centromerus longibulbus*	SSBAC4379-13	KM832376
Araneae	Linyphiidae	*Centromerus longibulbus*	SSBAC4393-13	KM830846
Araneae	Linyphiidae	*Centromerus longibulbus*	SSBAC4398-13	KM832388
Araneae	Linyphiidae	*Centromerus longibulbus*	SSBAF6181-13	KM827358
Araneae	Linyphiidae	*Centromerus longibulbus*	SSGBB1801-14	KP655721
Araneae	Linyphiidae	*Centromerus longibulbus*	SSJAB2034-13	KM831225
Araneae	Linyphiidae	*Centromerus sp. 2GAB*	SPRMA1094-12	KP650321
Araneae	Linyphiidae	*Centromerus sylvaticus*	ARONT526-10	HQ924414
Araneae	Linyphiidae	*Centromerus sylvaticus*	CNEIH032-13	KM838209
Araneae	Linyphiidae	*Centromerus sylvaticus*	ERSPI014-08	KP648396
Araneae	Linyphiidae	*Centromerus sylvaticus*	ERSPI015-08	KP645919
Araneae	Linyphiidae	*Centromerus sylvaticus*	ERSPI020-08	KP654974
Araneae	Linyphiidae	*Centromerus sylvaticus*	ERSPI021-08	KP655778
Araneae	Linyphiidae	*Centromerus sylvaticus*	ERSPI022-08	KP651653
Araneae	Linyphiidae	*Centromerus sylvaticus*	ERSPI038-08	KP656769
Araneae	Linyphiidae	*Centromerus sylvaticus*	ERSPI039-08	KP645750
Araneae	Linyphiidae	*Centromerus sylvaticus*	ERSPI040-08	KP655144
Araneae	Linyphiidae	*Centromerus sylvaticus*	ERSPI043-08	KP654757
Araneae	Linyphiidae	*Centromerus sylvaticus*	ERSPI044-08	KP653005
Araneae	Linyphiidae	*Centromerus sylvaticus*	ERSPI051-08	KP654842
Araneae	Linyphiidae	*Centromerus sylvaticus*	ERSPI054-08	KP654541
Araneae	Linyphiidae	*Centromerus sylvaticus*	ERSPI068-08	KP651778
Araneae	Linyphiidae	*Centromerus sylvaticus*	ERSPI075-08	KP646071
Araneae	Linyphiidae	*Centromerus sylvaticus*	ERSPI079-08	KP652743
Araneae	Linyphiidae	*Centromerus sylvaticus*	ERSPI085-08	KP648334
Araneae	Linyphiidae	*Centromerus sylvaticus*	ERSPI086-08	KP653719
Araneae	Linyphiidae	*Centromerus sylvaticus*	ERSPI091-08	KP650113
Araneae	Linyphiidae	*Centromerus sylvaticus*	ERSPI095-08	KP646706
Araneae	Linyphiidae	*Centromerus sylvaticus*	ERSPI103-09	KP647605
Araneae	Linyphiidae	*Centromerus sylvaticus*	ERSPI109-09	KP649968
Araneae	Linyphiidae	*Centromerus sylvaticus*	ERSPI110-09	KP652488
Araneae	Linyphiidae	*Centromerus sylvaticus*	ERSPI113-09	KP646012
Araneae	Linyphiidae	*Centromerus sylvaticus*	ERSPI114-09	KP648504
Araneae	Linyphiidae	*Centromerus sylvaticus*	ERSPI115-09	KP654113
Araneae	Linyphiidae	*Centromerus sylvaticus*	ERSPI117-09	KP650835
Araneae	Linyphiidae	*Centromerus sylvaticus*	ERSPI123-09	KP653551
Araneae	Linyphiidae	*Centromerus sylvaticus*	ERSPI127-09	KP650236
Araneae	Linyphiidae	*Centromerus sylvaticus*	ERSPI129-09	KP652909
Araneae	Linyphiidae	*Centromerus sylvaticus*	ERSPI130-09	KP656127
Araneae	Linyphiidae	*Centromerus sylvaticus*	ERSPI131-09	KP648693
Araneae	Linyphiidae	*Centromerus sylvaticus*	ERSPI133-09	KP648289
Araneae	Linyphiidae	*Centromerus sylvaticus*	ERSPI134-09	KP656319
Araneae	Linyphiidae	*Centromerus sylvaticus*	ERSPI135-09	KP648699
Araneae	Linyphiidae	*Centromerus sylvaticus*	ERSPI137-09	KP653672
Araneae	Linyphiidae	*Centromerus sylvaticus*	ERSPI138-09	KP647680
Araneae	Linyphiidae	*Centromerus sylvaticus*	ERSPI139-09	KP648992
Araneae	Linyphiidae	*Centromerus sylvaticus*	ERSPI145-09	KP648827

Araneae	Linyphiidae	*Centromerus sylvaticus*	ERSPI146-09	KP652502
Araneae	Linyphiidae	*Centromerus sylvaticus*	ERSPI147-09	KP649762
Araneae	Linyphiidae	*Centromerus sylvaticus*	ERSPI148-09	KP654457
Araneae	Linyphiidae	*Centromerus sylvaticus*	ERSPI149-09	KP651527
Araneae	Linyphiidae	*Centromerus sylvaticus*	ERSPI150-09	KP656572
Araneae	Linyphiidae	*Centromerus sylvaticus*	ERSPI151-09	KP648708
Araneae	Linyphiidae	*Centromerus sylvaticus*	ERSPI153-09	KP651688
Araneae	Linyphiidae	*Centromerus sylvaticus*	ERSPI155-09	KP648671
Araneae	Linyphiidae	*Centromerus sylvaticus*	ERSPI156-09	KP652876
Araneae	Linyphiidae	*Centromerus sylvaticus*	ERSPI157-09	KP650584
Araneae	Linyphiidae	*Centromerus sylvaticus*	ERSPI158-09	KP654230
Araneae	Linyphiidae	*Centromerus sylvaticus*	ERSPI159-09	KP649767
Araneae	Linyphiidae	*Centromerus sylvaticus*	ERSPI160-09	KP655961
Araneae	Linyphiidae	*Centromerus sylvaticus*	ERSPI161-09	KP646623
Araneae	Linyphiidae	*Centromerus sylvaticus*	ERSPI162-09	KP648781
Araneae	Linyphiidae	*Centromerus sylvaticus*	ERSPI165-09	KP649505
Araneae	Linyphiidae	*Centromerus sylvaticus*	ERSPI167-09	KP655737
Araneae	Linyphiidae	*Centromerus sylvaticus*	ERSPI168-09	KP649474
Araneae	Linyphiidae	*Centromerus sylvaticus*	ERSPI171-09	KP652532
Araneae	Linyphiidae	*Centromerus sylvaticus*	ERSPI172-09	KP653919
Araneae	Linyphiidae	*Centromerus sylvaticus*	ERSPI173-09	KP647775
Araneae	Linyphiidae	*Centromerus sylvaticus*	ERSPI174-09	KP647017
Araneae	Linyphiidae	*Centromerus sylvaticus*	ERSPI175-09	KP654909
Araneae	Linyphiidae	*Centromerus sylvaticus*	ERSPI176-09	KP651086
Araneae	Linyphiidae	*Centromerus sylvaticus*	ERSPI177-09	KP653212
Araneae	Linyphiidae	*Centromerus sylvaticus*	ERSPI178-09	KP650928
Araneae	Linyphiidae	*Centromerus sylvaticus*	ERSPI179-09	KP651956
Araneae	Linyphiidae	*Centromerus sylvaticus*	ERSPI180-09	KP649230
Araneae	Linyphiidae	*Centromerus sylvaticus*	ERSPI181-09	KP652164
Araneae	Linyphiidae	*Centromerus sylvaticus*	ERSPI182-09	KP645737
Araneae	Linyphiidae	*Centromerus sylvaticus*	ERSPI185-09	KP655467
Araneae	Linyphiidae	*Centromerus sylvaticus*	ERSPI186-09	KP651988
Araneae	Linyphiidae	*Centromerus sylvaticus*	ERSPI192-09	KP652707
Araneae	Linyphiidae	*Centromerus sylvaticus*	ERSPI194-09	KP645900
Araneae	Linyphiidae	*Centromerus sylvaticus*	ERSPI198-09	KP645790
Araneae	Linyphiidae	*Centromerus sylvaticus*	ERSPI199-09	KP651580
Araneae	Linyphiidae	*Centromerus sylvaticus*	ERSPI200-09	KP655649
Araneae	Linyphiidae	*Centromerus sylvaticus*	ERSPI202-09	KP646160
Araneae	Linyphiidae	*Centromerus sylvaticus*	ERSPI203-09	KP657102
Araneae	Linyphiidae	*Centromerus sylvaticus*	ERSPI206-09	KP651848
Araneae	Linyphiidae	*Centromerus sylvaticus*	ERSPI207-09	KP652165
Araneae	Linyphiidae	*Centromerus sylvaticus*	ERSPI216-09	KP649106
Araneae	Linyphiidae	*Centromerus sylvaticus*	ERSPI219-09	KP650406
Araneae	Linyphiidae	*Centromerus sylvaticus*	ERSPI220-09	KP647271
Araneae	Linyphiidae	*Centromerus sylvaticus*	ERSPI222-09	KP656019
Araneae	Linyphiidae	*Centromerus sylvaticus*	ERSPI223-09	KP650980
Araneae	Linyphiidae	*Centromerus sylvaticus*	ERSPI224-09	KP650795
Araneae	Linyphiidae	*Centromerus sylvaticus*	ERSPI225-09	KP651618
Araneae	Linyphiidae	*Centromerus sylvaticus*	ERSPI226-09	KP646387
Araneae	Linyphiidae	*Centromerus sylvaticus*	ERSPI231-09	KP649601
Araneae	Linyphiidae	*Centromerus sylvaticus*	ERSPI238-09	KP646551
Araneae	Linyphiidae	*Centromerus sylvaticus*	ERSPI240-09	KP645506
Araneae	Linyphiidae	*Centromerus sylvaticus*	RBINA2377-13	KP648041

Araneae	Linyphiidae	*Centromerus sylvaticus*	RBINA2388-13	KP645838
Araneae	Linyphiidae	*Centromerus sylvaticus*	RBINA542-13	KP650645
Araneae	Linyphiidae	*Ceraticelus atriceps*	CNSLO184-13	KM827536
Araneae	Linyphiidae	*Ceraticelus atriceps*	CNWBH1140-13	KM826588
Araneae	Linyphiidae	*Ceraticelus atriceps*	JSARA026-11	KP651166
Araneae	Linyphiidae	*Ceraticelus atriceps*	JSAUG1869-12	KP652117
Araneae	Linyphiidae	*Ceraticelus atriceps*	JSMAY1523-12	KP647611
Araneae	Linyphiidae	*Ceraticelus atriceps*	RBINA3259-13	KP652640
Araneae	Linyphiidae	*Ceraticelus atriceps*	SMTPB20150-13	KP654810
Araneae	Linyphiidae	*Ceraticelus atriceps*	SMTPB20923-13	KP656548
Araneae	Linyphiidae	*Ceraticelus atriceps*	SPIAL211-10	HQ580668
Araneae	Linyphiidae	*Ceraticelus atriceps*	SPIAL213-10	HQ580670
Araneae	Linyphiidae	*Ceraticelus atriceps*	SPIAL237-10	HQ580686
Araneae	Linyphiidae	*Ceraticelus atriceps*	SPIAL259-10	HQ580704
Araneae	Linyphiidae	*Ceraticelus crassiceps*	SPICH227-09	GU683972
Araneae	Linyphiidae	*Ceraticelus fissiceps*	CNBAA467-12	KM836150
Araneae	Linyphiidae	*Ceraticelus laetabilis*	SSKJB4423-14	KR070047
Araneae	Linyphiidae	*Ceraticelus laetabilis*	SSEIB8404-13	KM824172
Araneae	Linyphiidae	*Ceraticelus rowensis*	SSBAF4392-13	KM833229
Araneae	Linyphiidae	*Ceraticelus rowensis*	SSBAF6185-13	KM830878
Araneae	Linyphiidae	*Ceraticelus similis*	ARONT162-09	GU682655
Araneae	Linyphiidae	*Ceraticelus similis*	ARONT432-09	GU682939
Araneae	Linyphiidae	*Ceraticelus similis*	ARONT437-09	GU682936
Araneae	Linyphiidae	*Ceraticelus similis*	ARONT702-10	HQ924560
Araneae	Linyphiidae	*Ceraticelus similis*	ARONT772-10	HQ924621
Araneae	Linyphiidae	*Ceraticelus similis*	RBINA4698-13	KP650733
Araneae	Linyphiidae	*Ceraticelus similis*	RBINA4700-13	KP656957
Araneae	Linyphiidae	*Ceraticelus similis*	RBINA5238-13	KP652685
Araneae	Linyphiidae	*Ceratinella alaskae*	SPRMA417-10	JF887005
Araneae	Linyphiidae	*Ceratinella alaskae*	SPRMA879-12	KP645550
Araneae	Linyphiidae	*Ceratinella brunnea*	KKCHE594-07	KF367928
Araneae	Linyphiidae	*Ceratinella brunnea*	SMTPB11091-13	KP646781
Araneae	Linyphiidae	*Ceratinella brunnea*	SMTPB1484-13	KP651858
Araneae	Linyphiidae	*Ceratinella brunnea*	SMTPB1485-13	KP657239
Araneae	Linyphiidae	*Ceratinella brunnea*	SMTPB20451-13	KP648153
Araneae	Linyphiidae	*Ceratinella brunnea*	SMTPB5074-13	KP649786
Araneae	Linyphiidae	*Ceratinella brunnea*	SMTPB6319-13	KP653338
Araneae	Linyphiidae	*Ceratinella ornatula*	SPIAL059-10	HQ580564
Araneae	Linyphiidae	*Ceratinella ornatula*	SPICH001-09	GU683858
Araneae	Linyphiidae	*Ceratinella ornatula alaskana*	SPRMA991-12	KP652912
Araneae	Linyphiidae	*Ceratinops annulipes*	SSKJB4418-14	KR070581
Araneae	Linyphiidae	*Ceratinops annulipes*	SSKJB4754-14	KR069633
Araneae	Linyphiidae	*Ceratinops annulipes*	SSPAA6502-13	KM827304
Araneae	Linyphiidae	*Ceratinops crenatus*	SMTPB1066-13	KP654059
Araneae	Linyphiidae	*Ceratinops inflatus*	SPIAL036-10	HQ580547
Araneae	Linyphiidae	*Ceratinops inflatus*	SPIAL048-10	HQ580556
Araneae	Linyphiidae	*Ceratinops inflatus*	SPIAL062-10	HQ580567
Araneae	Linyphiidae	*Ceratinops inflatus*	SPIAL074-10	HQ580578
Araneae	Linyphiidae	*Ceratinops inflatus*	SPIAL086-10	HQ580587
Araneae	Linyphiidae	*Ceratinops inflatus*	SPIAL124-10	HQ580617
Araneae	Linyphiidae	*Ceratinops inflatus*	SPIAL136-10	HQ580624
Araneae	Linyphiidae	*Ceratinops inflatus*	SPIAL233-10	HQ580683
Araneae	Linyphiidae	*Ceratinops inflatus*	SPIAL249-10	HQ580696

Araneae	Linyphiidae	*Ceratinops inflatus*	SPIAL273-10	HQ580715
Araneae	Linyphiidae	*Ceratinops inflatus*	SPIAL285-10	HQ580724
Araneae	Linyphiidae	*Ceratinops inflatus*	SPRMA086-10	HQ977157
Araneae	Linyphiidae	*Ceratinops inflatus*	SPRMA232-10	HQ580840
Araneae	Linyphiidae	*Ceratinops inflatus*	SPRMA238-10	HQ580845
Araneae	Linyphiidae	*Ceratinops inflatus*	SPRMA337-10	HQ580897
Araneae	Linyphiidae	*Ceratinops inflatus*	SPRMA978-12	KP657259
Araneae	Linyphiidae	*Ceratinops latus*	CNGIB559-12	KM836762
Araneae	Linyphiidae	*Ceratinops latus*	CNPPB2377-12	KM839041
Araneae	Linyphiidae	*Ceratinopsis*	TDWGB775-10	HQ979218
Araneae	Linyphiidae	*Ceratinopsis*	TDWGB776-10	HQ979219
Araneae	Linyphiidae	*Ceratinopsis*	TDWGB923-10	HQ979346
Araneae	Linyphiidae	*Ceratinopsis labradorensis*	SSBAC4378-13	KM836539
Araneae	Linyphiidae	*Ceratinopsis labradorensis*	SSBAC4396-13	KM840272
Araneae	Linyphiidae	*Ceratinopsis labradorensis*	SSBAF6158-13	KM825109
Araneae	Linyphiidae	*Ceratinopsis labradorensis*	SSBAF6159-13	KM840047
Araneae	Linyphiidae	*Ceratinopsis labradorensis*	SSBAF6163-13	KM828983
Araneae	Linyphiidae	*Ceratinopsis labradorensis*	SSBAF6166-13	KM834416
Araneae	Linyphiidae	*Ceratinopsis labradorensis*	SSBAF6167-13	KM827585
Araneae	Linyphiidae	*Ceratinopsis labradorensis*	SSBAF6170-13	KM824934
Araneae	Linyphiidae	*Ceratinopsis labradorensis*	SSBAF6173-13	KM827428
Araneae	Linyphiidae	*Ceratinopsis labradorensis*	SSBAF6175-13	KM834645
Araneae	Linyphiidae	*Ceratinopsis nigriceps*	CNBPH357-12	KM828696
Araneae	Linyphiidae	*Ceratinopsis setoensis*	GBCH11149-13	JN817121
Araneae	Linyphiidae	*Cnephalocotes obscurus*	SPICH405-09	GU684235
Araneae	Linyphiidae	*Collinsia ksenia*	SPRMA290-10	HQ977163
Araneae	Linyphiidae	*Collinsia ksenia*	SPRMA341-10	HQ580900
Araneae	Linyphiidae	*Collinsia ksenia*	SPRMA388-10	JF886994
Araneae	Linyphiidae	*Collinsia ksenia*	SPRMA389-10	JF886995
Araneae	Linyphiidae	*Collinsia ksenia*	SPRMA390-10	JF886996
Araneae	Linyphiidae	*Collinsia ksenia*	SPRMA418-10	JF887006
Araneae	Linyphiidae	*Collinsia plumosa*	ARONT514-10	HQ924405
Araneae	Linyphiidae	*Collinsia plumosa*	SMTPB13981-13	KP648019
Araneae	Linyphiidae	*Diplocentria bidentata*	ARONT535-10	HQ924423
Araneae	Linyphiidae	*Diplocentria bidentata*	ARONT543-10	HQ924430
Araneae	Linyphiidae	*Diplocentria bidentata*	ARONT545-10	HQ924432
Araneae	Linyphiidae	*Diplocentria bidentata*	ARONT548-10	HQ924435
Araneae	Linyphiidae	*Diplocentria bidentata*	ARONT552-10	HQ924439
Araneae	Linyphiidae	*Diplocentria bidentata*	ARONT554-10	HQ924441
Araneae	Linyphiidae	*Diplocentria bidentata*	ARONT556-10	HQ924443
Araneae	Linyphiidae	*Diplocentria perplexa*	SPRMA1135-12	KP656885
Araneae	Linyphiidae	*Diplocentria rectangulata*	SPICH407-09	GU684188
Araneae	Linyphiidae	*Diplocentria rectangulata*	SPRMA971-12	KP656567
Araneae	Linyphiidae	*Diplocephalus cristatus*	ARONT219-09	HM434058
Araneae	Linyphiidae	*Diplocephalus cristatus*	ARONT564-10	HQ924450
Araneae	Linyphiidae	*Diplocephalus cristatus*	ARONT565-10	HQ924451
Araneae	Linyphiidae	*Diplocephalus cristatus*	ARONT566-10	HQ924452
Araneae	Linyphiidae	*Diplocephalus subrostratus*	CNRMC1581-12	KM830092
Araneae	Linyphiidae	*Diplostyla concolor*	ARONT176-09	GU682615
Araneae	Linyphiidae	*Diplostyla concolor*	ARONT177-09	GU682616
Araneae	Linyphiidae	*Diplostyla concolor*	ARONT192-09	GU682702
Araneae	Linyphiidae	*Diplostyla concolor*	ARONT214-09	GU682705
Araneae	Linyphiidae	*Diplostyla concolor*	ARONT215-09	GU682703

Araneae	Linyphiidae	*Diplostyla concolor*	ARONT216-09	GU682700
Araneae	Linyphiidae	*Diplostyla concolor*	ARONT217-09	GU682730
Araneae	Linyphiidae	*Diplostyla concolor*	ARONT218-09	GU682701
Araneae	Linyphiidae	*Diplostyla concolor*	ARONT377-09	GU682921
Araneae	Linyphiidae	*Diplostyla concolor*	ARONT481-10	HQ924380
Araneae	Linyphiidae	*Diplostyla concolor*	ARONT567-10	HQ924453
Araneae	Linyphiidae	*Diplostyla concolor*	ARONT607-10	HQ924475
Araneae	Linyphiidae	*Diplostyla concolor*	ARONT630-10	HQ924495
Araneae	Linyphiidae	*Diplostyla concolor*	ARONT818-10	HQ924663
Araneae	Linyphiidae	*Diplostyla concolor*	BBCAN428-09	GU683413
Araneae	Linyphiidae	*Diplostyla concolor*	ERSPI331-09	GU682503
Araneae	Linyphiidae	*Diplostyla concolor*	ERSPI366-09	GU682481
Araneae	Linyphiidae	*Diplostyla concolor*	ERSPI368-09	GU682483
Araneae	Linyphiidae	*Diplostyla concolor*	ERSPI370-09	GU682473
Araneae	Linyphiidae	*Diplostyla concolor*	GBCH5127-10	FJ838651
Araneae	Linyphiidae	*Diplostyla concolor*	RBINA1140-13	KP656078
Araneae	Linyphiidae	*Diplostyla concolor*	RBINA1146-13	KP655497
Araneae	Linyphiidae	*Diplostyla concolor*	RBINA1156-13	KP652636
Araneae	Linyphiidae	*Diplostyla concolor*	RBINA1167-13	KP648105
Araneae	Linyphiidae	*Diplostyla concolor*	RBINA1169-13	KP655570
Araneae	Linyphiidae	*Diplostyla concolor*	RBINA2378-13	KP651934
Araneae	Linyphiidae	*Diplostyla concolor*	RBINA2382-13	KP650407
Araneae	Linyphiidae	*Diplostyla concolor*	RBINA2384-13	KP651021
Araneae	Linyphiidae	*Diplostyla concolor*	RBINA2387-13	KP646009
Araneae	Linyphiidae	*Diplostyla concolor*	RBINA2389-13	KP647265
Araneae	Linyphiidae	*Diplostyla concolor*	RBINA2390-13	KP648229
Araneae	Linyphiidae	*Diplostyla concolor*	RBINA2391-13	KP654637
Araneae	Linyphiidae	*Diplostyla concolor*	RBINA3320-13	KP655140
Araneae	Linyphiidae	*Diplostyla concolor*	RBINA3355-13	KP648338
Araneae	Linyphiidae	*Diplostyla concolor*	RBINA3430-13	KP654118
Araneae	Linyphiidae	*Diplostyla concolor*	RBINA4697-13	KP652942
Araneae	Linyphiidae	*Diplostyla concolor*	RBINA5240-13	KP646691
Araneae	Linyphiidae	*Dismodicus decemoculatus*	CNRMD2823-12	KM836721
Araneae	Linyphiidae	*Dismodicus decemoculatus*	SMTPB14673-13	KP649821
Araneae	Linyphiidae	*Dismodicus decemoculatus*	SPICH051-09	GU683835
Araneae	Linyphiidae	*Doenitzius pruvus*	GBCH11154-13	JN817116
Araneae	Linyphiidae	*Drapetisca alteranda*	CNEIH005-13	KM827804
Araneae	Linyphiidae	*Drapetisca alteranda*	CNWLB763-13	KM831333
Araneae	Linyphiidae	*Entelecara acuminata*	CNGIC289-12	KM840446
Araneae	Linyphiidae	*Entelecara acuminata*	CNGID223-12	KM824936
Araneae	Linyphiidae	*Entelecara sp. 1GAB*	CNWLE2557-13	KM832298
Araneae	Linyphiidae	*Entelecara sp. 1GAB*	SPICH869-09	GU684440
Araneae	Linyphiidae	*Eridantes erigonoides*	ERSPI356-09	HM376106
Araneae	Linyphiidae	*Eridantes erigonoides*	SMTPB21288-13	KP654409
Araneae	Linyphiidae	*Eridantes utibilis*	CNPAA470-13	KM833280
Araneae	Linyphiidae	*Erigone aletris*	CNEIA2648-12	KM827652
Araneae	Linyphiidae	*Erigone aletris*	CNEIE1943-13	KM830288
Araneae	Linyphiidae	*Erigone aletris*	CNPAH372-13	KM833131
Araneae	Linyphiidae	*Erigone aletris*	CNPAH375-13	KM824621
Araneae	Linyphiidae	*Erigone aletris*	SPIAL133-10	HQ580623
Araneae	Linyphiidae	*Erigone aletris*	SPIAL145-10	HQ580628
Araneae	Linyphiidae	*Erigone aletris*	SPIAL157-10	HQ580634
Araneae	Linyphiidae	*Erigone aletris*	SPRMA1029-12	KP651229

Araneae	Linyphiidae	*Erigone aletris*	SPRMA243-10	HQ580849
Araneae	Linyphiidae	*Erigone aletris*	SPRMA381-10	JF886988
Araneae	Linyphiidae	*Erigone aletris*	SPRMA678-10	JF887119
Araneae	Linyphiidae	*Erigone arctica*	ARONT422-09	GU682942
Araneae	Linyphiidae	*Erigone arctica*	CNEID3473-13	KM830111
Araneae	Linyphiidae	*Erigone arctica*	CNEIE1944-13	KM839403
Araneae	Linyphiidae	*Erigone arctica*	JSARA013-11	KP654419
Araneae	Linyphiidae	*Erigone arctica*	SMTPB5072-13	KP653361
Araneae	Linyphiidae	*Erigone arctica*	SPRMA1092-12	KP647890
Araneae	Linyphiidae	*Erigone arctica*	SPRMA1126-12	KP657305
Araneae	Linyphiidae	*Erigone arctophylacis*	SPICH848-09	GU684448
Araneae	Linyphiidae	*Erigone atra*	ARONT654-10	HQ924519
Araneae	Linyphiidae	*Erigone atra*	GBCH0628-06	AY383537
Araneae	Linyphiidae	*Erigone atra*	GBCH2839-08	DQ504375
Araneae	Linyphiidae	*Erigone atra*	GBCH5718-13	FJ899802
Araneae	Linyphiidae	*Erigone atra*	GBCH5719-13	FJ899801
Araneae	Linyphiidae	*Erigone atra*	JSARA062-11	KP654204
Araneae	Linyphiidae	*Erigone atra*	RBINA3359-13	KP647814
Araneae	Linyphiidae	*Erigone atra*	RBINA4899-13	KP655876
Araneae	Linyphiidae	*Erigone atra*	SMTPB6317-13	KP647623
Araneae	Linyphiidae	*Erigone autumnalis*	ARONT171-09	GU682588
Araneae	Linyphiidae	*Erigone autumnalis*	ARONT646-10	HQ924511
Araneae	Linyphiidae	*Erigone autumnalis*	ARONT804-10	HQ924649
Araneae	Linyphiidae	*Erigone autumnalis*	ERSPI342-09	GU682502
Araneae	Linyphiidae	*Erigone autumnalis*	ERSPI343-09	GU682494
Araneae	Linyphiidae	*Erigone autumnalis*	ERSPI345-09	GU682496
Araneae	Linyphiidae	*Erigone autumnalis*	ERSPI349-09	GU682489
Araneae	Linyphiidae	*Erigone autumnalis*	RBINA4650-13	KP655783
Araneae	Linyphiidae	*Erigone autumnalis*	RBINA5162-13	KP651869
Araneae	Linyphiidae	*Erigone autumnalis*	RBINA5275-13	KP653380
Araneae	Linyphiidae	*Erigone autumnalis*	RBINA5276-13	KP652768
Araneae	Linyphiidae	*Erigone blaesa*	ARONT528-10	HQ924416
Araneae	Linyphiidae	*Erigone blaesa*	ARONT639-10	HQ924504
Araneae	Linyphiidae	*Erigone blaesa*	ARONT640-10	HQ924505
Araneae	Linyphiidae	*Erigone blaesa*	ARONT728-10	HQ924582
Araneae	Linyphiidae	*Erigone blaesa*	CNEIB1683-12	KM825866
Araneae	Linyphiidae	*Erigone blaesa*	CNEIC3177-13	KM829586
Araneae	Linyphiidae	*Erigone blaesa*	SMTPB17027-13	KP656758
Araneae	Linyphiidae	*Erigone cristatopalpus*	SPICH1013-09	GU684673
Araneae	Linyphiidae	*Erigone cristatopalpus*	SPRMA1105-12	KP657238
Araneae	Linyphiidae	*Erigone dentipalpis*	GACAC532-12	GU323032
Araneae	Linyphiidae	*Erigone dentipalpis*	GBCH0629-06	AY383538
Araneae	Linyphiidae	*Erigone koshiensis*	GBCH11150-13	JN817120
Araneae	Linyphiidae	*Erigone tirolensis*	KKCHE1005-09	GU683692
Araneae	Linyphiidae	*Erigone tirolensis*	SPIRU1034-11	KF368029
Araneae	Linyphiidae	*Erigonoploides sp. 1GAB*	SPRMA1060-12	KP650724
Araneae	Linyphiidae	*Eskovina clava*	GBCH11148-13	JN817122
Araneae	Linyphiidae	*Estrandia grandaeva*	GBADC011-10	HQ956675
Araneae	Linyphiidae	*Eulaira obscura*	SSWLD3219-13	KM828852
Araneae	Linyphiidae	*Floricomus rostratus*	SPIRU1266-11	KF368088
Araneae	Linyphiidae	*Floronia bucculenta*	GBCH5124-10	FJ838654
Araneae	Linyphiidae	*Frederickus coylei*	SPRMA385-10	JF886991
Araneae	Linyphiidae	*Frederickus coylei*	SPRMA435-10	KP654022

Araneae	Linyphiidae	*Frederickus coylei*	SPRMA974-12	KP652067
Araneae	Linyphiidae	*Frederickus wilburi*	SPRMA980-12	KP652350
Araneae	Linyphiidae	*Frontinella communis*	ARONT406-09	GU682932
Araneae	Linyphiidae	*Frontinella communis*	ARONT407-09	GU682933
Araneae	Linyphiidae	*Frontinella communis*	ARONT687-10	HQ924546
Araneae	Linyphiidae	*Frontinella communis*	ARONT707-10	HQ924564
Araneae	Linyphiidae	*Frontinella communis*	ARONT740-10	HQ924593
Araneae	Linyphiidae	*Frontinella communis*	ARONT741-10	HQ924594
Araneae	Linyphiidae	*Frontinella communis*	ARONT757-10	HQ924609
Araneae	Linyphiidae	*Frontinella communis*	ARONT758-10	HQ924610
Araneae	Linyphiidae	*Frontinella communis*	ARONT759-10	HQ924611
Araneae	Linyphiidae	*Frontinella communis*	GBCH5123-10	FJ838655
Araneae	Linyphiidae	*Frontinella communis*	PHJUL2614-11	KP652489
Araneae	Linyphiidae	*Frontinella communis*	RBCH029-04	DQ127493
Araneae	Linyphiidae	*Frontinella communis*	RBINA994-13	KP646731
Araneae	Linyphiidae	*Frontinella communis*	SPRMA382-10	JF886989
Araneae	Linyphiidae	*Glyphesis scopulifer*	SMTPB17495-13	KP651921
Araneae	Linyphiidae	*Gnathonarium*	GBCH11147-13	JN817123
Araneae	Linyphiidae	*Gnathonarium taczanowskii*	SPRMA1164-12	KP654106
Araneae	Linyphiidae	*Gonatium crassipalpum*	SPIRU1074-11	KF368109
Araneae	Linyphiidae	*Gonatium rubellum*	GBCH5122-10	FJ838656
Araneae	Linyphiidae	*Grammonota angusta*	CNBAD701-12	KM833179
Araneae	Linyphiidae	*Grammonota angusta*	CNBPA221-12	KM832290
Araneae	Linyphiidae	*Grammonota angusta*	CNBPB516-12	KM834406
Araneae	Linyphiidae	*Grammonota angusta*	CNBPB517-12	KM826077
Araneae	Linyphiidae	*Grammonota angusta*	CNBPG393-12	KM835917
Araneae	Linyphiidae	*Grammonota angusta*	CNBPG394-12	KM829182
Araneae	Linyphiidae	*Grammonota angusta*	CNBPG395-12	KM837668
Araneae	Linyphiidae	*Grammonota angusta*	CNBPH359-12	KM839955
Araneae	Linyphiidae	*Grammonota angusta*	CNBPH360-12	KM826897
Araneae	Linyphiidae	*Grammonota angusta*	CNBPH361-12	KM835225
Araneae	Linyphiidae	*Grammonota angusta*	CNBPH362-12	KM832985
Araneae	Linyphiidae	*Grammonota angusta*	CNBPH363-12	KM825123
Araneae	Linyphiidae	*Grammonota angusta*	CNBPH364-12	KM838944
Araneae	Linyphiidae	*Grammonota angusta*	CNBPM117-13	KM828845
Araneae	Linyphiidae	*Grammonota angusta*	CNBPR105-13	KM827057
Araneae	Linyphiidae	*Grammonota angusta*	CNBPS345-13	KM837988
Araneae	Linyphiidae	*Grammonota angusta*	CNBPS346-13	KM824104
Araneae	Linyphiidae	*Grammonota angusta*	CNBPT142-13	KM840025
Araneae	Linyphiidae	*Grammonota angusta*	CNBPT143-13	KM836044
Araneae	Linyphiidae	*Grammonota angusta*	CNBPT144-13	KM824729
Araneae	Linyphiidae	*Grammonota angusta*	CNJAC1503-12	KM839664
Araneae	Linyphiidae	*Grammonota angusta*	CNPAB303-13	KM833083
Araneae	Linyphiidae	*Grammonota angusta*	CNPAF959-13	KM834349
Araneae	Linyphiidae	*Grammonota angusta*	CNPAH373-13	KM837440
Araneae	Linyphiidae	*Grammonota angusta*	CNPAH376-13	KM838055
Araneae	Linyphiidae	*Grammonota angusta*	CNPAH377-13	KM824296
Araneae	Linyphiidae	*Grammonota angusta*	CNPAJ018-13	KM831440
Araneae	Linyphiidae	*Grammonota angusta*	CNPAJ022-13	KM834568
Araneae	Linyphiidae	*Grammonota angusta*	CNPAL031-13	KM828662
Araneae	Linyphiidae	*Grammonota angusta*	CNPPA4069-12	KJ163186
Araneae	Linyphiidae	*Grammonota angusta*	CNRMC1579-12	KM838024
Araneae	Linyphiidae	*Grammonota angusta*	CNRMC1580-12	KM824321

Araneae	Linyphiidae	*Grammonota angusta*	CNWBG1008-13	KM833620
Araneae	Linyphiidae	*Grammonota angusta*	CNWBG1011-13	KM829104
Araneae	Linyphiidae	*Grammonota angusta*	PHAUG1823-12	KP653253
Araneae	Linyphiidae	*Grammonota angusta*	PHAUG1825-12	KP646445
Araneae	Linyphiidae	*Grammonota angusta*	PHJUL2616-11	KP653482
Araneae	Linyphiidae	*Grammonota angusta*	PHJUL2619-11	KP651481
Araneae	Linyphiidae	*Grammonota angusta*	PHMTT620-10	KP656972
Araneae	Linyphiidae	*Grammonota angusta*	PHMTT623-10	JN307894
Araneae	Linyphiidae	*Grammonota angusta*	PHMTT982-10	JN307918
Araneae	Linyphiidae	*Grammonota angusta*	PHMTT985-10	JN307919
Araneae	Linyphiidae	*Grammonota angusta*	PHMTU043-10	JN307922
Araneae	Linyphiidae	*Grammonota angusta*	PHMTU046-10	JN307924
Araneae	Linyphiidae	*Grammonota angusta*	PHMTU048-10	JN307926
Araneae	Linyphiidae	*Grammonota angusta*	PHMTU051-10	JN307928
Araneae	Linyphiidae	*Grammonota angusta*	PHMTU054-10	JN307931
Araneae	Linyphiidae	*Grammonota angusta*	PHMTU073-10	JN307938
Araneae	Linyphiidae	*Grammonota angusta*	PHMTU078-10	JN307941
Araneae	Linyphiidae	*Grammonota angusta*	PHMTU079-10	JN307942
Araneae	Linyphiidae	*Grammonota angusta*	PHMTU080-10	JN307943
Araneae	Linyphiidae	*Grammonota angusta*	PHMTU082-10	JN307944
Araneae	Linyphiidae	*Grammonota angusta*	PHMTU096-10	JN307951
Araneae	Linyphiidae	*Grammonota angusta*	PHMTU098-10	JN307953
Araneae	Linyphiidae	*Grammonota angusta*	PHMTU100-10	JN307954
Araneae	Linyphiidae	*Grammonota angusta*	PHMTU101-10	JN307955
Araneae	Linyphiidae	*Grammonota angusta*	PHMTU102-10	JN307956
Araneae	Linyphiidae	*Grammonota angusta*	SMTPB1068-13	KP653439
Araneae	Linyphiidae	*Grammonota angusta*	SMTPB12363-13	KP646955
Araneae	Linyphiidae	*Grammonota angusta*	SMTPB12370-13	KP648895
Araneae	Linyphiidae	*Grammonota angusta*	SMTPB15245-13	KP649343
Araneae	Linyphiidae	*Grammonota angusta*	SMTPB4250-13	KP654644
Araneae	Linyphiidae	*Grammonota angusta*	SMTPB4784-13	KP650827
Araneae	Linyphiidae	*Grammonota angusta*	SMTPB5900-13	KP647522
Araneae	Linyphiidae	*Grammonota angusta*	SMTPB7660-13	KP656455
Araneae	Linyphiidae	*Grammonota angusta*	SMTPB8962-13	KP649904
Araneae	Linyphiidae	*Grammonota angusta*	SPIRU1087-11	KF368113
Araneae	Linyphiidae	*Grammonota gentilis*	ARONT374-09	GU682938
Araneae	Linyphiidae	*Grammonota gentilis*	CNPAP163-13	KM834262
Araneae	Linyphiidae	*Grammonota gentilis*	RBINA3236-13	KP653239
Araneae	Linyphiidae	*Grammonota gentilis*	RBINA796-13	KP651423
Araneae	Linyphiidae	*Grammonota gigas*	ARONT806-10	HQ924651
Araneae	Linyphiidae	*Grammonota kincaidi*	CNGIC294-12	KM837707
Araneae	Linyphiidae	*Grammonota kincaidi*	CNGIJ928-13	KM831259
Araneae	Linyphiidae	*Grammonota kincaidi*	SPRMA384-10	JF886990
Araneae	Linyphiidae	*Grammonota kincaidi*	SPRMA386-10	JF886992
Araneae	Linyphiidae	*Grammonota maritima*	KKCHE509-07	KF368130
Araneae	Linyphiidae	*Grammonota ornata*	ARONT489-10	HQ924384
Araneae	Linyphiidae	*Grammonota ornata*	ARONT490-10	HQ924385
Araneae	Linyphiidae	*Grammonota ornata*	ARONT491-10	HQ924386
Araneae	Linyphiidae	*Grammonota ornata*	ARONT498-10	HQ924393
Araneae	Linyphiidae	*Helophora insignis*	GBCH5120-10	FJ838658
Araneae	Linyphiidae	*Helophora reducta*	CNBAH021-13	KM833379
Araneae	Linyphiidae	*Helophora reducta*	SPRMA387-10	JF886993
Araneae	Linyphiidae	*Hilaira canaliculata*	SPICH022-09	HM432622

Araneae	Linyphiidae	*Hilaira herniosa*	SSBAB512-12	KM825946
Araneae	Linyphiidae	*Hilaira herniosa*	SSBAE3663-13	KM839408
Araneae	Linyphiidae	*Hilaira herniosa*	SSJAA1873-13	KM832945
Araneae	Linyphiidae	*Hilaira herniosa*	SSJAC1262-13	KM827972
Araneae	Linyphiidae	*Hilaira herniosa*	SSJAD2339-13	KM835037
Araneae	Linyphiidae	*Hilaira herniosa*	SSJAF8501-13	KM837232
Araneae	Linyphiidae	*Horcotes quadricristatus*	SPIRU1347-11	KF368151
Araneae	Linyphiidae	*Hybauchenidium cymbadentatum*	SSPAB8136-13	KM825650
Araneae	Linyphiidae	*Hybauchenidium gibbosum*	KKCHE1002-09	GU683694
Araneae	Linyphiidae	*Hylyphantes graminicola*	GBCH11151-13	JN817119
Araneae	Linyphiidae	*Hypomma marxi*	SMTPB15971-13	KP651689
Araneae	Linyphiidae	*Hypomma marxi*	SMTPB20565-13	KP645615
Araneae	Linyphiidae	*Hypomma marxi*	SPICH1070-09	GU684696
Araneae	Linyphiidae	*Hypomma subarcticum*	SPRMA098-10	HQ580739
Araneae	Linyphiidae	*Idionella rugosa*	RBINA236-13	KP652658
Araneae	Linyphiidae	*Improphantes complicatus*	ARONT017-09	GU682782
Araneae	Linyphiidae	*Improphantes complicatus*	SPICH048-09	GU683846
Araneae	Linyphiidae	*Improphantes complicatus*	SPRMA1218-12	KP645962
Araneae	Linyphiidae	*Improphantes sp. 1GAB*	SPRMA898-12	KP648260
Araneae	Linyphiidae	*Improphantes sp. 1RB*	SPRMA889-12	KP652828
Araneae	Linyphiidae	*Incestophantes lamprus*	SPRMA113-10	HQ580751
Araneae	Linyphiidae	*Incestophantes lamprus*	SPRMA793-12	KP657335
Araneae	Linyphiidae	*Incestophantes mercedes*	SPRMA880-12	KP649775
Araneae	Linyphiidae	*Incestophantes washingtoni*	KKCHE459-07	KF368176
Araneae	Linyphiidae	*Incestophantes washingtoni*	SPRMA190-10	KP648078
Araneae	Linyphiidae	*Incestophantes washingtoni*	SSBAB2754-12	KM837313
Araneae	Linyphiidae	*Incestophantes washingtoni*	SSBAB2888-12	KM824615
Araneae	Linyphiidae	*Incestophantes washingtoni*	SSBAB485-12	KM829197
Araneae	Linyphiidae	*Incestophantes washingtoni*	SSBAB555-12	KM828915
Araneae	Linyphiidae	*Incestophantes washingtoni*	SSBAB575-12	KM832099
Araneae	Linyphiidae	*Incestophantes washingtoni*	SSBAE3615-13	KM836456
Araneae	Linyphiidae	*Incestophantes washingtoni*	SSJAA1104-13	KM828240
Araneae	Linyphiidae	*Islandiana falsifica*	SPICH661-09	GU684536
Araneae	Linyphiidae	*Islandiana flaveola*	ARONT626-10	HQ924491
Araneae	Linyphiidae	*Islandiana flaveola*	ARONT642-10	HQ924507
Araneae	Linyphiidae	*Islandiana flaveola*	ARONT648-10	HQ924513
Araneae	Linyphiidae	*Islandiana flaveola*	ARONT649-10	HQ924514
Araneae	Linyphiidae	*Islandiana flaveola*	ARONT662-10	HQ924523
Araneae	Linyphiidae	*Islandiana flaveola*	ARONT724-10	HQ924579
Araneae	Linyphiidae	*Islandiana flaveola*	ERSPI433-09	HM376111
Araneae	Linyphiidae	*Islandiana flaveola*	SMTPB13982-13	KP652612
Araneae	Linyphiidae	*Islandiana flaveola*	SMTPB15595-13	KP648667
Araneae	Linyphiidae	*Islandiana flaveola*	SPRMA1123-12	KP657121
Araneae	Linyphiidae	*Islandiana holmi*	SPICH003-09	GU683875
Araneae	Linyphiidae	*Kaestneria pullata*	BBCAN782-09	GU682976
Araneae	Linyphiidae	*Kaestneria pullata*	CNEID3472-13	KM835693
Araneae	Linyphiidae	*Kaestneria pullata*	CNPED1417-14	KP654340
Araneae	Linyphiidae	*Kaestneria pullata*	CNRMD2822-12	KM831007
Araneae	Linyphiidae	*Kaestneria pullata*	JSARA020-11	KP650093
Araneae	Linyphiidae	*Kaestneria pullata*	SPICH1084-09	GU684646
Araneae	Linyphiidae	*Kaestneria pullata*	SSEIB8433-13	KM828518
Araneae	Linyphiidae	*Kaestneria pullata*	SSJAE9795-13	KM837893
Araneae	Linyphiidae	*Kaestneria rufula*	SPICH885-09	GU684458

Araneae	Linyphiidae	*Lepthyphantes alpinus*	BBCAN570-09	GU683571
Araneae	Linyphiidae	*Lepthyphantes alpinus*	CNBAE238-12	KM824417
Araneae	Linyphiidae	*Lepthyphantes alpinus*	SPICH1021-09	GU684730
Araneae	Linyphiidae	*Lepthyphantes alpinus*	SPRMA379-10	HQ580924
Araneae	Linyphiidae	*Lepthyphantes furcillifer*	SPRMA139-10	HQ580768
Araneae	Linyphiidae	*Lepthyphantes furcillifer*	SPRMA801-12	KP652815
Araneae	Linyphiidae	*Lepthyphantes furcillifer*	SPRMA903-12	KP646800
Araneae	Linyphiidae	*Lepthyphantes intricatus*	SPRMA953-12	KP649800
Araneae	Linyphiidae	*Lepthyphantes intricatus*	SSPAB8244-13	KM840065
Araneae	Linyphiidae	*Lepthyphantes intricatus*	SSPAB8288-13	KM840255
Araneae	Linyphiidae	*Lepthyphantes intricatus*	SSPAC10971-13	KM831786
Araneae	Linyphiidae	*Lepthyphantes intricatus*	SSPAC10985-13	KM835990
Araneae	Linyphiidae	*Lepthyphantes intricatus*	SSPAC12199-13	KM825007
Araneae	Linyphiidae	*Lepthyphantes leprosus*	ARONT079-09	GU682779
Araneae	Linyphiidae	*Lepthyphantes leprosus*	ARONT130-09	GU682634
Araneae	Linyphiidae	*Lepthyphantes leprosus*	ARONT131-09	GU682631
Araneae	Linyphiidae	*Lepthyphantes leprosus*	ARONT132-09	GU682632
Araneae	Linyphiidae	*Lepthyphantes leprosus*	ARONT135-09	GU682629
Araneae	Linyphiidae	*Lepthyphantes leprosus*	ARONT136-09	GU682630
Araneae	Linyphiidae	*Lepthyphantes leprosus*	ARONT195-09	GU682713
Araneae	Linyphiidae	*Lepthyphantes leprosus*	ARONT196-09	GU682715
Araneae	Linyphiidae	*Lepthyphantes leprosus*	ARONT197-09	GU682716
Araneae	Linyphiidae	*Lepthyphantes leprosus*	ARONT220-09	GU682714
Araneae	Linyphiidae	*Lepthyphantes leprosus*	ARONT443-09	GU682950
Araneae	Linyphiidae	*Lepthyphantes leprosus*	ARONT483-10	HQ924382
Araneae	Linyphiidae	*Lepthyphantes leprosus*	ARONT597-10	HQ924471
Araneae	Linyphiidae	*Lepthyphantes leprosus*	ARONT622-10	HQ924487
Araneae	Linyphiidae	*Lepthyphantes leprosus*	ARONT623-10	HQ924488
Araneae	Linyphiidae	*Lepthyphantes leprosus*	ARONT624-10	HQ924489
Araneae	Linyphiidae	*Lepthyphantes leprosus*	ARONT627-10	HQ924492
Araneae	Linyphiidae	*Lepthyphantes leprosus*	ARONT628-10	HQ924493
Araneae	Linyphiidae	*Lepthyphantes leprosus*	SPRMA1083-12	KP648738
Araneae	Linyphiidae	*Lepthyphantes leprosus*	SPRMA690-10	JF887125
Araneae	Linyphiidae	*Lepthyphantes minutus*	GBCH0409-06	AY078689
Araneae	Linyphiidae	*Lepthyphantes turbatrix*	SPRMA818-12	KP656674
Araneae	Linyphiidae	*Linyphantes aeronauticus*	SPRMA1111-12	KP650251
Araneae	Linyphiidae	*Linyphantes nehalem*	CNGIB545-12	KM827263
Araneae	Linyphiidae	*Linyphantes nehalem*	CNGIJ927-13	KM827495
Araneae	Linyphiidae	*Linyphantes nehalem*	SPRMA1130-12	KP646860
Araneae	Linyphiidae	*Linyphantes nehalem*	SPRMA404-10	KP655060
Araneae	Linyphiidae	*Linyphantes orcinus*	CNPCA033-12	KM839843
Araneae	Linyphiidae	*Linyphantes orcinus*	CNPCB049-12	KM836332
Araneae	Linyphiidae	*Linyphantes orcinus*	CNPCD037-12	KM825499
Araneae	Linyphiidae	*Linyphantes orcinus*	CNPCE074-12	KM825714
Araneae	Linyphiidae	*Linyphantes orcinus*	CNPCE075-12	KM829017
Araneae	Linyphiidae	*Linyphantes orcinus*	CNPCL022-13	KM829581
Araneae	Linyphiidae	*Linyphantes orcinus*	RBINA2711-13	KP648898
Araneae	Linyphiidae	*Linyphantes orcinus*	SPIAL026-10	HQ580538
Araneae	Linyphiidae	*Linyphantes orcinus*	SPIAL147-10	HQ580630
Araneae	Linyphiidae	*Linyphantes orcinus*	SPIAL159-10	HQ580636
Araneae	Linyphiidae	*Linyphantes orcinus*	SPIAL201-10	HQ580660
Araneae	Linyphiidae	*Linyphantes orcinus*	SPIAL206-10	HQ580663
Araneae	Linyphiidae	*Linyphantes orcinus*	SPIAL207-10	HQ580664

Araneae	Linyphiidae	*Linyphantes orcinus*	SPIAL212-10	HQ580669
Araneae	Linyphiidae	*Linyphantes orcinus*	SPIAL218-10	HQ580672
Araneae	Linyphiidae	*Linyphantes orcinus*	SPIAL219-10	HQ580673
Araneae	Linyphiidae	*Linyphantes orcinus*	SPIAL230-10	HQ580680
Araneae	Linyphiidae	*Linyphantes orcinus*	SPIAL260-10	HQ580705
Araneae	Linyphiidae	*Linyphantes orcinus*	SPIAL272-10	HQ580714
Araneae	Linyphiidae	*Linyphantes orcinus*	SPIAL284-10	HQ580723
Araneae	Linyphiidae	*Linyphantes pacificus*	SPRMA237-10	HQ580844
Araneae	Linyphiidae	*Linyphantes pualla*	SPRMA1186-12	KP649670
Araneae	Linyphiidae	*Linyphantes pualla*	SPRMA399-10	KP647690
Araneae	Linyphiidae	*Linyphantes pualla*	SPRMA806-12	KP653111
Araneae	Linyphiidae	*Linyphantes pualla*	SPRMA907-12	KP650109
Araneae	Linyphiidae	*Linyphantes victoria*	CNGIB555-12	KM828337
Araneae	Linyphiidae	*Linyphantes victoria*	CNGIB556-12	KM836337
Araneae	Linyphiidae	*Linyphantes victoria*	SPRMA1037-12	KP651004
Araneae	Linyphiidae	*Linyphantes victoria*	SPRMA407-10	JF887000
Araneae	Linyphiidae	*Linyphantes victoria*	SPRMA409-10	JN310300
Araneae	Linyphiidae	*Linyphia triangularis*	GACAC389-12	FR775771
Araneae	Linyphiidae	*Linyphia triangularis*	GBCH0413-06	AY078693
Araneae	Linyphiidae	*Linyphia triangularis*	GBCH3901-09	EU003292
Araneae	Linyphiidae	*Lophomma depressum*	CNPAA469-13	KM834458
Araneae	Linyphiidae	*Lophomma depressum*	CNPKD956-13	KP650517
Araneae	Linyphiidae	*Lophomma depressum*	SSEIB11406-13	KM833851
Araneae	Linyphiidae	*Macrargus multesimus*	ARONT428-09	HM434066
Araneae	Linyphiidae	*Maso sundevalli*	CNEIB1684-12	KM825357
Araneae	Linyphiidae	*Maso sundevalli*	CNEIC3174-12	KM824909
Araneae	Linyphiidae	*Maso sundevalli*	CNEIF2263-12	KM834666
Araneae	Linyphiidae	*Mecynargus paetulus*	SPICH025-09	GU683878
Araneae	Linyphiidae	*Mecynargus sp. 2GAB*	SPRMA185-10	HQ580802
Araneae	Linyphiidae	*Megalepthyphantes nebulosus*	ARONT238-09	GU682722
Araneae	Linyphiidae	*Megalepthyphantes nebulosus*	ARONT239-09	GU682719
Araneae	Linyphiidae	*Megalepthyphantes nebulosus*	ARONT240-09	GU682720
Araneae	Linyphiidae	*Megalepthyphantes nebulosus*	ARONT376-09	GU682943
Araneae	Linyphiidae	*Megalepthyphantes nebulosus*	ARONT563-10	HQ924449
Araneae	Linyphiidae	*Megalepthyphantes nebulosus*	ARONT598-10	HQ924472
Araneae	Linyphiidae	*Megalepthyphantes nebulosus*	ARONT653-10	HQ924518
Araneae	Linyphiidae	*Mermessus maculatus*	SSBRB2313-14	KR070349
Araneae	Linyphiidae	*Mermessus maculatus*	SSKJA1887-14	KR070183
Araneae	Linyphiidae	*Mermessus maculatus*	SSKJA1890-14	KR070528
Araneae	Linyphiidae	*Mermessus maculatus*	SSKJA1902-14	KR070508
Araneae	Linyphiidae	*Mermessus maculatus*	SSKJB4399-14	KR070745
Araneae	Linyphiidae	*Mermessus maculatus*	SSKJB4440-14	KR070337
Araneae	Linyphiidae	*Mermessus maculatus*	SSBRB4636-14	KR069877
Araneae	Linyphiidae	*Mermessus maculatus*	SSBRB5198-14	KP650580
Araneae	Linyphiidae	*Mermessus maculatus*	SSBRB5199-14	KP657274
Araneae	Linyphiidae	*Mermessus maculatus*	SSBRB5200-14	KP646089
Araneae	Linyphiidae	*Mermessus maculatus*	SSBRB5205-14	KP645521
Araneae	Linyphiidae	*Mermessus maculatus*	SSBRB5206-14	KP648606
Araneae	Linyphiidae	*Mermessus maculatus*	TDWGB767-10	HQ979210
Araneae	Linyphiidae	*Mermessus tridentatus*	ARONT441-09	HM434069
Araneae	Linyphiidae	*Mermessus tridentatus*	ARONT817-10	HQ924662
Araneae	Linyphiidae	*Mermessus tridentatus*	RBINA139-13	KP656713
Araneae	Linyphiidae	*Mermessus tridentatus*	RBINA140-13	KP653865

Araneae	Linyphiidae	*Mermessus tridentatus*	RBINA141-13	KP647785
Araneae	Linyphiidae	*Mermessus tridentatus*	RBINA150-13	KP654695
Araneae	Linyphiidae	*Mermessus tridentatus*	RBINA151-13	KP645879
Araneae	Linyphiidae	*Mermessus tridentatus*	RBINA152-13	KP647140
Araneae	Linyphiidae	*Mermessus trilobatus*	ARONT158-09	GU682589
Araneae	Linyphiidae	*Mermessus trilobatus*	ARONT159-09	GU682586
Araneae	Linyphiidae	*Mermessus trilobatus*	ARONT641-10	HQ924506
Araneae	Linyphiidae	*Mermessus trilobatus*	ARONT643-10	HQ924508
Araneae	Linyphiidae	*Mermessus trilobatus*	ARONT645-10	HQ924510
Araneae	Linyphiidae	*Mermessus trilobatus*	ARONT647-10	HQ924512
Araneae	Linyphiidae	*Mermessus trilobatus*	ARONT650-10	HQ924515
Araneae	Linyphiidae	*Mermessus trilobatus*	ARONT659-10	HQ924521
Araneae	Linyphiidae	*Mermessus trilobatus*	ARONT661-10	HQ924522
Araneae	Linyphiidae	*Mermessus trilobatus*	ARONT726-10	HQ924580
Araneae	Linyphiidae	*Mermessus trilobatus*	ARONT727-10	HQ924581
Araneae	Linyphiidae	*Mermessus trilobatus*	ARONT747-10	KP651606
Araneae	Linyphiidae	*Mermessus trilobatus*	ARONT752-10	HQ924604
Araneae	Linyphiidae	*Mermessus trilobatus*	ARONT805-10	HQ924650
Araneae	Linyphiidae	*Mermessus trilobatus*	ERSPI009-08	KP655257
Araneae	Linyphiidae	*Mermessus trilobatus*	ERSPI041-08	KP651515
Araneae	Linyphiidae	*Mermessus trilobatus*	ERSPI112-09	KP654870
Araneae	Linyphiidae	*Mermessus trilobatus*	ERSPI250-09	GU682564
Araneae	Linyphiidae	*Mermessus trilobatus*	ERSPI282-09	GU682537
Araneae	Linyphiidae	*Mermessus trilobatus*	ERSPI373-09	GU682476
Araneae	Linyphiidae	*Mermessus trilobatus*	RBINA5241-13	KP647863
Araneae	Linyphiidae	*Mermessus trilobatus*	SPIAL123-10	HQ580616
Araneae	Linyphiidae	*Mermessus trilobatus*	SPRMA093-10	HQ977158
Araneae	Linyphiidae	*Mermessus trilobatus*	SPRMA094-10	HQ977159
Araneae	Linyphiidae	*Mermessus trilobatus*	SPRMA1189-12	KP648686
Araneae	Linyphiidae	*Mermessus trilobatus*	SPRMA264-10	KP653521
Araneae	Linyphiidae	*Mermessus undulatus*	CNPEK249-14	KP653743
Araneae	Linyphiidae	*Mermessus undulatus*	SSBAC4408-13	KM827116
Araneae	Linyphiidae	*Mermessus undulatus*	SSEIB7098-13	KM829223
Araneae	Linyphiidae	*Mermessus undulatus*	SSPAB8264-13	KM831431
Araneae	Linyphiidae	*Metopobactrus prominulus*	CNJAC1505-12	KM827412
Araneae	Linyphiidae	*Metopobactrus prominulus*	KKCHE981-09	GU683679
Araneae	Linyphiidae	*Microlinyphia dana*	GBCH0410-06	AY078690
Araneae	Linyphiidae	*Microlinyphia dana*	SPIAL012-10	HQ580525
Araneae	Linyphiidae	*Microlinyphia dana*	SPIAL023-10	HQ580535
Araneae	Linyphiidae	*Microlinyphia dana*	SPIAL024-10	HQ580536
Araneae	Linyphiidae	*Microlinyphia dana*	SPIAL035-10	HQ580546
Araneae	Linyphiidae	*Microlinyphia dana*	SPIAL231-10	HQ580681
Araneae	Linyphiidae	*Microlinyphia dana*	SPIAL257-10	HQ580702
Araneae	Linyphiidae	*Microlinyphia dana*	SPIAL278-10	HQ580718
Araneae	Linyphiidae	*Microlinyphia dana*	SPRMA1076-12	KP649881
Araneae	Linyphiidae	*Microlinyphia dana*	SPRMA108-10	HQ580747
Araneae	Linyphiidae	*Microlinyphia dana*	SPRMA242-10	HQ580848
Araneae	Linyphiidae	*Microlinyphia dana*	SPRMA320-10	HQ580888
Araneae	Linyphiidae	*Microlinyphia dana*	SPRMA410-10	JF887001
Araneae	Linyphiidae	*Microlinyphia mandibulata*	ARONT380-09	GU682937
Araneae	Linyphiidae	*Microlinyphia mandibulata*	ARONT401-09	GU682934
Araneae	Linyphiidae	*Microlinyphia mandibulata*	RBCH003-04	DQ127526
Araneae	Linyphiidae	*Microlinyphia pusilla*	CNBAG034-12	KM828934

Araneae	Linyphiidae	*Microlinyphia pusilla*	CNBAG036-12	KM840638
Araneae	Linyphiidae	*Microlinyphia pusilla*	CNBAG053-12	KM832934
Araneae	Linyphiidae	*Microlinyphia pusilla*	CNBAG054-12	KM839806
Araneae	Linyphiidae	*Microlinyphia pusilla*	SPIRU1035-11	KF368296
Araneae	Linyphiidae	*Microneta viaria*	GBCH11427-13	JQ801599
Araneae	Linyphiidae	*Microneta viaria*	GBCH5118-10	FJ838661
Araneae	Linyphiidae	*Microneta viaria*	SPRMA1064-12	KP654855
Araneae	Linyphiidae	*Mughiphantes sp. 1GAB*	SPICH930-09	GU684464
Araneae	Linyphiidae	*Mughiphantes sp. 1GAB*	SPRMA1087-12	KP647023
Araneae	Linyphiidae	*Mythoplastoides erectus*	SPRMA840-12	KP654913
Araneae	Linyphiidae	*Neriene clathrata*	ARONT134-09	GU682598
Araneae	Linyphiidae	*Neriene clathrata*	ARONT194-09	GU682684
Araneae	Linyphiidae	*Neriene clathrata*	ARONT561-10	HQ924448
Araneae	Linyphiidae	*Neriene clathrata*	ARONT586-10	HQ924461
Araneae	Linyphiidae	*Neriene clathrata*	ARONT587-10	HQ924462
Araneae	Linyphiidae	*Neriene clathrata*	ARONT589-10	HQ924464
Araneae	Linyphiidae	*Neriene clathrata*	ARONT637-10	HQ924502
Araneae	Linyphiidae	*Neriene clathrata*	ARONT644-10	HQ924509
Araneae	Linyphiidae	*Neriene clathrata*	ARONT688-10	HQ924547
Araneae	Linyphiidae	*Neriene clathrata*	ERSPI262-09	HM376099
Araneae	Linyphiidae	*Neriene clathrata*	GBCH5714-13	FJ899809
Araneae	Linyphiidae	*Neriene clathrata*	JSARA073-11	KP654752
Araneae	Linyphiidae	*Neriene clathrata*	RBCH004-04	DQ127527
Araneae	Linyphiidae	*Neriene clathrata*	RBINA3414-13	KP655507
Araneae	Linyphiidae	*Neriene digna*	CNGIE398-12	KM838820
Araneae	Linyphiidae	*Neriene digna*	CNGIJ924-13	KM834654
Araneae	Linyphiidae	*Neriene digna*	CNGLF1923-13	KM832355
Araneae	Linyphiidae	*Neriene digna*	SPIAL014-10	HQ580527
Araneae	Linyphiidae	*Neriene digna*	SPIAL176-10	HQ580645
Araneae	Linyphiidae	*Neriene digna*	SPIAL188-10	HQ580651
Araneae	Linyphiidae	*Neriene digna*	SPRMA362-10	HQ580912
Araneae	Linyphiidae	*Neriene digna*	SPRMA411-10	JF887002
Araneae	Linyphiidae	*Neriene digna*	SPRMA414-10	KP656604
Araneae	Linyphiidae	*Neriene digna*	SPRMA713-10	KP655884
Araneae	Linyphiidae	*Neriene digna*	SPRMA740-10	JF887158
Araneae	Linyphiidae	*Neriene digna*	SPRMA758-10	JF887172
Araneae	Linyphiidae	*Neriene digna*	SPRMA759-10	JF887173
Araneae	Linyphiidae	*Neriene emphana*	GBCH11164-13	JN817106
Araneae	Linyphiidae	*Neriene japonica*	GBCH11156-13	JN817114
Araneae	Linyphiidae	*Neriene limbatinella*	GBCH11161-13	JN817109
Araneae	Linyphiidae	*Neriene montana*	ARONT013-09	GU682813
Araneae	Linyphiidae	*Neriene montana*	ARONT014-09	GU682833
Araneae	Linyphiidae	*Neriene montana*	ARONT015-09	GU682830
Araneae	Linyphiidae	*Neriene montana*	ARONT093-09	GU682811
Araneae	Linyphiidae	*Neriene montana*	ARONT094-09	GU682829
Araneae	Linyphiidae	*Neriene montana*	ARONT101-09	GU682597
Araneae	Linyphiidae	*Neriene montana*	ARONT102-09	GU682596
Araneae	Linyphiidae	*Neriene montana*	ARONT103-09	GU682592
Araneae	Linyphiidae	*Neriene montana*	ARONT104-09	GU682594
Araneae	Linyphiidae	*Neriene montana*	ARONT105-09	GU682599
Araneae	Linyphiidae	*Neriene montana*	ARONT106-09	GU682595
Araneae	Linyphiidae	*Neriene montana*	ARONT107-09	GU682593
Araneae	Linyphiidae	*Neriene montana*	ARONT211-09	GU682679

Araneae	Linyphiidae	*Neriene montana*	ARONT212-09	GU682681
Araneae	Linyphiidae	*Neriene montana*	ARONT237-09	GU682682
Araneae	Linyphiidae	*Neriene montana*	ARONT241-09	GU682680
Araneae	Linyphiidae	*Neriene montana*	ARONT346-09	GU682848
Araneae	Linyphiidae	*Neriene montana*	ARONT584-10	HQ924459
Araneae	Linyphiidae	*Neriene montana*	ARONT585-10	HQ924460
Araneae	Linyphiidae	*Neriene montana*	ARONT588-10	HQ924463
Araneae	Linyphiidae	*Neriene montana*	RBCH005-04	DQ127517
Araneae	Linyphiidae	*Neriene montana*	RBINA099-13	KP647766
Araneae	Linyphiidae	*Neriene montana*	SMTPB18172-13	KP646334
Araneae	Linyphiidae	*Neriene nigripectoris*	GBCH11157-13	JN817113
Araneae	Linyphiidae	*Neriene oidedicata*	GBCH11158-13	JN817112
Araneae	Linyphiidae	*Neriene oidedicata*	GBCH11159-13	JN817111
Araneae	Linyphiidae	*Neriene radiata*	ARONT057-09	GU682767
Araneae	Linyphiidae	*Neriene radiata*	ARONT201-09	GU682691
Araneae	Linyphiidae	*Neriene radiata*	ARONT715-10	HQ924571
Araneae	Linyphiidae	*Neriene radiata*	ARONT742-10	HQ924595
Araneae	Linyphiidae	*Neriene radiata*	ARONT781-10	HQ924629
Araneae	Linyphiidae	*Neriene radiata*	CNGLF1939-13	KM826108
Araneae	Linyphiidae	*Neriene radiata*	CNSLR081-13	KM833346
Araneae	Linyphiidae	*Neriene radiata*	CNWLE2549-13	KM827032
Araneae	Linyphiidae	*Neriene radiata*	CNWLE2552-13	KM834938
Araneae	Linyphiidae	*Neriene radiata*	ERSPI308-09	GU682526
Araneae	Linyphiidae	*Neriene radiata*	ERSPI309-09	GU682527
Araneae	Linyphiidae	*Neriene radiata*	ERSPI310-09	GU682528
Araneae	Linyphiidae	*Neriene radiata*	ERSPI311-09	GU682517
Araneae	Linyphiidae	*Neriene radiata*	ERSPI312-09	GU682518
Araneae	Linyphiidae	*Neriene radiata*	ERSPI397-09	GU682452
Araneae	Linyphiidae	*Neriene radiata*	RBCH060-04	DQ127476
Araneae	Linyphiidae	*Neriene radiata*	RBCH085-04	DQ127437
Araneae	Linyphiidae	*Neriene radiata*	RBCH104-04	DQ127424
Araneae	Linyphiidae	*Neriene radiata*	RBCH182-04	DQ127344
Araneae	Linyphiidae	*Neriene radiata*	RBINA2733-13	KP652678
Araneae	Linyphiidae	*Neriene radiata*	RBINA2796-13	KP651772
Araneae	Linyphiidae	*Neriene radiata*	RBINA2804-13	KP648131
Araneae	Linyphiidae	*Neriene radiata*	RBINA2831-13	KP656987
Araneae	Linyphiidae	*Neriene radiata*	RBINA2832-13	KP656214
Araneae	Linyphiidae	*Neriene radiata*	RBINA2833-13	KP649410
Araneae	Linyphiidae	*Neriene radiata*	RBINA3392-13	KP647303
Araneae	Linyphiidae	*Neriene radiata*	RBINA3394-13	KP648448
Araneae	Linyphiidae	*Neriene radiata*	RBINA3408-13	KP651720
Araneae	Linyphiidae	*Neriene radiata*	RBINA3423-13	KP655360
Araneae	Linyphiidae	*Neriene radiata*	RBINA5798-13	KP654826
Araneae	Linyphiidae	*Neriene radiata*	RBINA5820-13	KP646554
Araneae	Linyphiidae	*Neriene radiata*	RBINA5833-13	KP647950
Araneae	Linyphiidae	*Neriene radiata*	RBINA5843-13	KP656844
Araneae	Linyphiidae	*Neriene sp. TAB-2009*	GBCH4023-09	FJ607576
Araneae	Linyphiidae	*Neriene variabilis*	ERSPI265-09	GU682555
Araneae	Linyphiidae	*Neriene variabilis*	RBCH055-04	DQ127471
Araneae	Linyphiidae	*Notholepthyphantes australis*	GBCH5117-10	FJ838662
Araneae	Linyphiidae	*Oedothorax alascensis*	SPRMA1137-12	KP647294
Araneae	Linyphiidae	*Oedothorax alascensis*	SPRMA1190-12	KP651122
Araneae	Linyphiidae	*Oedothorax alascensis*	SPRMA133-10	HQ580762

Araneae	Linyphiidae	*Oedothorax alascensis*	SPRMA325-10	HQ580892
Araneae	Linyphiidae	*Oedothorax apicatus*	GBCH5115-10	FJ838664
Araneae	Linyphiidae	*Oedothorax apicatus*	GBCH5713-13	FJ899810
Araneae	Linyphiidae	*Oedothorax fuscus*	GBCH5712-13	FJ899811
Araneae	Linyphiidae	*Oedothorax retusus*	GBCH5710-13	FJ899813
Araneae	Linyphiidae	*Oedothorax retusus*	GBCH5711-13	FJ899812
Araneae	Linyphiidae	*Oedothorax trilobatus*	SPICH1103-09	GU684608
Araneae	Linyphiidae	*Oedothorax trilobatus*	SPRMA205-10	HQ580819
Araneae	Linyphiidae	*Oedothorax trilobatus*	SPRMA214-10	HQ580827
Araneae	Linyphiidae	*Oreoneta banffkluane*	SPRMA1229-12	KP653604
Araneae	Linyphiidae	*Oreoneta leviceps*	SPIRU1220-11	KF368304
Araneae	Linyphiidae	*Oreoneta leviceps*	SPRMA1145-12	KP646880
Araneae	Linyphiidae	*Oreonetides rectangulatus*	SSEIB7298-13	KM827167
Araneae	Linyphiidae	*Oreonetides rectangulatus*	SSEIB7317-13	KM827228
Araneae	Linyphiidae	*Oreonetides rectangulatus*	SSEIB7322-13	KM832122
Araneae	Linyphiidae	*Oreonetides rectangulatus*	SSPAB8101-13	KM836160
Araneae	Linyphiidae	*Oreonetides sp. 1GAB*	SPRMA890-12	KP653342
Araneae	Linyphiidae	*Oreonetides vaginatus*	SPICH080-09	GU683850
Araneae	Linyphiidae	*Pelecopsis mengei*	SPICH223-09	GU683971
Araneae	Linyphiidae	*Pelecopsis mengei*	SPRMA1067-12	KP651028
Araneae	Linyphiidae	*Pelecopsis sculpta*	SPIAL107-10	HQ580605
Araneae	Linyphiidae	*Pelecopsis sculpta*	SPRMA1023-12	KP652430
Araneae	Linyphiidae	*Pelecopsis sculpta*	SPRMA223-10	HQ580834
Araneae	Linyphiidae	*Pelecopsis sculpta*	SPRMA321-10	HQ580889
Araneae	Linyphiidae	*Phlattothrata flagellata*	CNRMC1690-12	KM836826
Araneae	Linyphiidae	*Phlattothrata parva*	SPIRU1272-11	KF368661
Araneae	Linyphiidae	*Pityohyphantes alticeps*	CNBAD680-12	KM839829
Araneae	Linyphiidae	*Pityohyphantes alticeps*	CNBAD696-12	KM827316
Araneae	Linyphiidae	*Pityohyphantes alticeps*	CNBAE239-12	KM828410
Araneae	Linyphiidae	*Pityohyphantes alticeps*	CNBAF131-12	KM839012
Araneae	Linyphiidae	*Pityohyphantes alticeps*	CNBAF204-12	KM824061
Araneae	Linyphiidae	*Pityohyphantes alticeps*	CNBAF205-12	KM826390
Araneae	Linyphiidae	*Pityohyphantes alticeps*	SPIAL248-10	HQ580695
Araneae	Linyphiidae	*Pityohyphantes alticeps*	SPRMA1141-12	KP651218
Araneae	Linyphiidae	*Pityohyphantes alticeps*	SPRMA169-10	HQ580789
Araneae	Linyphiidae	*Pityohyphantes alticeps*	SPRMA415-10	JF887003
Araneae	Linyphiidae	*Pityohyphantes alticeps*	SPRMA416-10	JF887004
Araneae	Linyphiidae	*Pityohyphantes costatus*	ARONT117-09	GU682606
Araneae	Linyphiidae	*Pityohyphantes costatus*	ARONT118-09	GU682604
Araneae	Linyphiidae	*Pityohyphantes costatus*	ARONT128-09	GU682605
Araneae	Linyphiidae	*Pityohyphantes costatus*	ARONT209-09	GU682694
Araneae	Linyphiidae	*Pityohyphantes costatus*	ARONT395-09	GU682931
Araneae	Linyphiidae	*Pityohyphantes costatus*	ARONT396-09	GU682928
Araneae	Linyphiidae	*Pityohyphantes costatus*	ARONT593-10	HQ924468
Araneae	Linyphiidae	*Pityohyphantes costatus*	ARONT668-10	HQ924527
Araneae	Linyphiidae	*Pityohyphantes costatus*	ARONT669-10	HQ924528
Araneae	Linyphiidae	*Pityohyphantes costatus*	CNBPE371-12	KM825623
Araneae	Linyphiidae	*Pityohyphantes costatus*	CNEIF2250-12	KM836452
Araneae	Linyphiidae	*Pityohyphantes costatus*	CNEIF2264-12	KM828903
Araneae	Linyphiidae	*Pityohyphantes costatus*	CNEIF2266-12	KM834747
Araneae	Linyphiidae	*Pityohyphantes costatus*	CNEIF2270-12	KM829934
Araneae	Linyphiidae	*Pityohyphantes costatus*	CNEIG008-12	KM830312
Araneae	Linyphiidae	*Pityohyphantes costatus*	CNEIH001-13	KM829250

Araneae	Linyphiidae	*Pityohyphantes costatus*	CNEIH014-13	KM839545
Araneae	Linyphiidae	*Pityohyphantes costatus*	CNEIH033-13	KM832134
Araneae	Linyphiidae	*Pityohyphantes costatus*	CNEII001-13	KM827053
Araneae	Linyphiidae	*Pityohyphantes costatus*	CNEIJ001-13	KM834940
Araneae	Linyphiidae	*Pityohyphantes costatus*	CNEIJ002-13	KM825097
Araneae	Linyphiidae	*Pityohyphantes cristatus*	ARONT126-09	HM434052
Araneae	Linyphiidae	*Pityohyphantes cristatus*	CNBAA474-12	KM827857
Araneae	Linyphiidae	*Pityohyphantes cristatus*	CNBAA475-12	KM830468
Araneae	Linyphiidae	*Pityohyphantes cristatus*	CNBAF133-12	KM825036
Araneae	Linyphiidae	*Pityohyphantes cristatus*	CNBAG038-12	KM837240
Araneae	Linyphiidae	*Pityohyphantes cristatus*	CNBAI523-13	KM833736
Araneae	Linyphiidae	*Pityohyphantes limitaneus*	ERSCH087-07	KF368698
Araneae	Linyphiidae	*Pityohyphantes subarcticus*	ARONT123-09	GU682602
Araneae	Linyphiidae	*Pityohyphantes subarcticus*	ARONT124-09	GU682603
Araneae	Linyphiidae	*Pityohyphantes subarcticus*	ARONT125-09	GU682600
Araneae	Linyphiidae	*Pityohyphantes subarcticus*	ARONT127-09	GU682601
Araneae	Linyphiidae	*Pityohyphantes subarcticus*	ARONT379-09	GU682930
Araneae	Linyphiidae	*Pityohyphantes subarcticus*	CNBAD684-12	KM829030
Araneae	Linyphiidae	*Pityohyphantes subarcticus*	CNBAD700-12	KM831551
Araneae	Linyphiidae	*Pityohyphantes subarcticus*	CNBAE283-12	KM837228
Araneae	Linyphiidae	*Pityohyphantes subarcticus*	CNBAE290-12	KM829798
Araneae	Linyphiidae	*Pityohyphantes subarcticus*	CNBAF132-12	KM828116
Araneae	Linyphiidae	*Pityohyphantes subarcticus*	CNBAG033-12	KM839552
Araneae	Linyphiidae	*Pityohyphantes subarcticus*	CNBAI522-13	KM836582
Araneae	Linyphiidae	*Pityohyphantes subarcticus*	CNBAI526-13	KM825626
Araneae	Linyphiidae	*Pityohyphantes subarcticus*	CNPAD929-13	KM835252
Araneae	Linyphiidae	*Pityohyphantes subarcticus*	CNPAF960-13	KM838954
Araneae	Linyphiidae	*Pityohyphantes subarcticus*	CNPAH371-13	KM838911
Araneae	Linyphiidae	*Pityohyphantes subarcticus*	CNPAM088-13	KM832164
Araneae	Linyphiidae	*Pityohyphantes subarcticus*	PHMTU067-10	JN307934
Araneae	Linyphiidae	*Pityohyphantes subarcticus*	PHMTU085-10	JN307945
Araneae	Linyphiidae	*Pityohyphantes subarcticus*	PHSEP1953-11	KP645507
Araneae	Linyphiidae	*Pityohyphantes subarcticus*	SSBAB2746-12	KM824620
Araneae	Linyphiidae	*Pityohyphantes subarcticus*	SSBAB2756-12	KM826337
Araneae	Linyphiidae	*Pityohyphantes subarcticus*	SSBAB2759-12	KM825425
Araneae	Linyphiidae	*Pityohyphantes subarcticus*	SSBAB2768-12	KM827371
Araneae	Linyphiidae	*Pityohyphantes subarcticus*	SSBAB2785-12	KM838526
Araneae	Linyphiidae	*Pityohyphantes subarcticus*	SSBAB2790-12	KM840261
Araneae	Linyphiidae	*Pityohyphantes subarcticus*	SSBAB2895-12	KM837796
Araneae	Linyphiidae	*Pityohyphantes subarcticus*	SSBAB543-12	KM831309
Araneae	Linyphiidae	*Pityohyphantes subarcticus*	SSBAE4090-13	KM835370
Araneae	Linyphiidae	*Pityohyphantes subarcticus*	SSBAE4092-13	KM829242
Araneae	Linyphiidae	*Pityohyphantes subarcticus*	SSBAE4101-13	KM824902
Araneae	Linyphiidae	*Pityohyphantes subarcticus*	SSBAE5753-13	KM825804
Araneae	Linyphiidae	*Pityohyphantes subarcticus*	SSBAE5764-13	KM835127
Araneae	Linyphiidae	*Pityohyphantes subarcticus*	SSBAE6077-13	KM833352
Araneae	Linyphiidae	*Pityohyphantes subarcticus*	SSJAA2175-13	KM830138
Araneae	Linyphiidae	*Pocadicnemis americana*	CNBPJ001-12	KP654616
Araneae	Linyphiidae	*Pocadicnemis americana*	CNPAE497-13	KM834849
Araneae	Linyphiidae	*Pocadicnemis americana*	CNRMC1689-12	KM829443
Araneae	Linyphiidae	*Pocadicnemis americana*	CNRMD2819-12	KM827849
Araneae	Linyphiidae	*Pocadicnemis americana*	CNWBE382-13	KM831550
Araneae	Linyphiidae	*Pocadicnemis americana*	SMTPB879-13	KP654574

Araneae	Linyphiidae	*Pocadicnemis americana*	SPICH180-09	GU684007
Araneae	Linyphiidae	*Pocadicnemis pumila*	CNPCF134-13	KM831067
Araneae	Linyphiidae	*Pocadicnemis pumila*	SPIAL111-10	HQ580608
Araneae	Linyphiidae	*Pocadicnemis pumila*	SPIAL200-10	HQ580659
Araneae	Linyphiidae	*Pocadicnemis pumila*	SPRMA244-10	HQ580850
Araneae	Linyphiidae	*Pocadicnemis pumila*	SPRMA420-10	JF887007
Araneae	Linyphiidae	*Pocadicnemis pumila*	SPRMA421-10	JN310301
Araneae	Linyphiidae	*Pocadicnemis sp. 2GAB*	ARONT746-10	HQ924599
Araneae	Linyphiidae	*Poeciloneta calcaratus*	ARONT529-10	HQ924417
Araneae	Linyphiidae	*Poeciloneta calcaratus*	CNBAB384-12	KM834236
Araneae	Linyphiidae	*Poeciloneta calcaratus*	CNBAK440-13	KM833275
Araneae	Linyphiidae	*Poeciloneta lyrica*	SPRMA116-10	HQ580753
Araneae	Linyphiidae	*Poeciloneta lyrica*	SPRMA910-12	KP656691
Araneae	Linyphiidae	*Poeciloneta lyrica*	SPRMA957-12	KP655947
Araneae	Linyphiidae	*Poeciloneta variegata*	CNMIE1710-14	KP652054
Araneae	Linyphiidae	*Poeciloneta variegata*	SPICH873-09	HM416916
Araneae	Linyphiidae	*Poeciloneta variegata*	SPRMA856-12	KP651834
Araneae	Linyphiidae	*Poeciloneta variegata*	SSEIA7675-13	KM838470
Araneae	Linyphiidae	*Porrhomma convexum*	SPIEU150-10	HQ975057
Araneae	Linyphiidae	*Porrhomma convexum*	SPIEU151-10	HQ975058
Araneae	Linyphiidae	*Porrhomma convexum*	SPIEU152-10	HQ975059
Araneae	Linyphiidae	*Porrhomma convexum*	SPIEU153-10	HQ975060
Araneae	Linyphiidae	*Porrhomma convexum*	SPIEU154-10	HQ975061
Araneae	Linyphiidae	*Porrhomma convexum*	SPIEU158-10	HQ975064
Araneae	Linyphiidae	*Porrhomma convexum*	SPIEU159-10	HQ975065
Araneae	Linyphiidae	*Porrhomma convexum*	SPIEU160-10	HQ975066
Araneae	Linyphiidae	*Porrhomma convexum*	SSJAA1084-13	KM828801
Araneae	Linyphiidae	*Porrhomma terrestre*	SMTPB1065-13	KP650300
Araneae	Linyphiidae	*Porrhomma terrestre*	SMTPB12083-13	KP656004
Araneae	Linyphiidae	*Porrhomma terrestre*	SPRMA1101-12	KP656791
Araneae	Linyphiidae	*Praestigia kulczynskii*	SPICH241-09	HM432628
Araneae	Linyphiidae	*Satilatlas marxi*	CNRMB229-12	KM827556
Araneae	Linyphiidae	*Satilatlas marxi*	CNRMB230-12	KM840503
Araneae	Linyphiidae	*Satilatlas marxi*	SMTPB17496-13	KP653161
Araneae	Linyphiidae	*Satilatlas marxi*	SMTPB20573-13	KP647948
Araneae	Linyphiidae	*Satilatlas marxi*	SPICH686-09	GU684545
Araneae	Linyphiidae	*Satilatlas monticola*	SPICH083-09	GU683842
Araneae	Linyphiidae	*Sciastes dubius*	KKCHE534-07	KF368772
Araneae	Linyphiidae	*Sciastes hastatus*	SPICH524-09	HM432631
Araneae	Linyphiidae	*Sciastes mentasta*	ARONT559-10	HQ924446
Araneae	Linyphiidae	*Sciastes truncatus*	SPIRU1354-11	KF368775
Araneae	Linyphiidae	*Sciastes truncatus*	SPRMA1155-12	KP646480
Araneae	Linyphiidae	*Scirites finitimus*	SPRMA1119-12	KP651148
Araneae	Linyphiidae	*Scirites sp. 1GAB*	SSPAA7581-13	KM824160
Araneae	Linyphiidae	*Scironis sima*	SPRMA1139-12	KP654741
Araneae	Linyphiidae	*Scironis sima*	SPRMA790-12	KP655938
Araneae	Linyphiidae	*Scironis tarsalis*	ARONT703-10	HQ924561
Araneae	Linyphiidae	*Scironis tarsalis*	ARONT751-10	HQ924603
Araneae	Linyphiidae	*Scotinotylus alpinus*	SPICH828-09	GU684442
Araneae	Linyphiidae	*Scotinotylus alpinus*	SPRMA373-10	HQ580920
Araneae	Linyphiidae	*Scotinotylus bicavatus*	SPRMA187-10	HQ580804
Araneae	Linyphiidae	*Scotinotylus bicavatus*	SPRMA766-12	KP648474
Araneae	Linyphiidae	*Scotinotylus columbia*	SPRMA1103-12	KP651553

Araneae	Linyphiidae	*Scotinotylus columbia*	SPRMA821-12	KP656392
Araneae	Linyphiidae	*Scotinotylus evansi*	SPICH098-09	HM432625
Araneae	Linyphiidae	*Scotinotylus kenus cf.*	SPRMA926-12	KP647756
Araneae	Linyphiidae	*Scotinotylus majesticus*	SPRMA853-12	KP652090
Araneae	Linyphiidae	*Scotinotylus pallidus*	SSPAA7537-13	KM836188
Araneae	Linyphiidae	*Scotinotylus patellatus*	SPIAL038-10	HQ580549
Araneae	Linyphiidae	*Scotinotylus patellatus*	SPIAL050-10	HQ580558
Araneae	Linyphiidae	*Scotinotylus patellatus*	SPIAL058-10	HQ580563
Araneae	Linyphiidae	*Scotinotylus patellatus*	SPIAL060-10	HQ580565
Araneae	Linyphiidae	*Scotinotylus patellatus*	SPIAL072-10	HQ580576
Araneae	Linyphiidae	*Scotinotylus patellatus*	SPIAL084-10	HQ580586
Araneae	Linyphiidae	*Scotinotylus patellatus*	SPIAL183-10	HQ580650
Araneae	Linyphiidae	*Scotinotylus patellatus*	SPIAL234-10	HQ580684
Araneae	Linyphiidae	*Scotinotylus patellatus*	SPRMA248-10	HQ580854
Araneae	Linyphiidae	*Scotinotylus patellatus*	SPRMA761-12	KP656103
Araneae	Linyphiidae	*Scotinotylus sacer*	SPICH033-09	GU683849
Araneae	Linyphiidae	*Scotinotylus sanctus*	SPRMA186-10	HQ580803
Araneae	Linyphiidae	*Scotinotylus sp. 2GAB*	SPRMA429-10	JF887010
Araneae	Linyphiidae	*Scotinotylus sp. 4GAB*	SSBAC4381-13	KM839663
Araneae	Linyphiidae	*Scyletria inflata*	SPICH551-09	GU684147
Araneae	Linyphiidae	*Semljicola lapponicus*	KKCHE781-07	KF368781
Araneae	Linyphiidae	*Semljicola obtusus*	KKCHE950-09	HM377209
Araneae	Linyphiidae	*Semljicola sp. 1GAB*	SPRMA1047-12	KP652202
Araneae	Linyphiidae	*Semljicola sp. 1GAB*	SPRMA212-10	HQ580826
Araneae	Linyphiidae	*Silometopoides pingrensis*	SSBAC4377-13	KM834014
Araneae	Linyphiidae	*Sisicus apertus*	SSBAB2720-12	KM826850
Araneae	Linyphiidae	*Sisicus apertus*	SSBAB2728-12	KM828551
Araneae	Linyphiidae	*Sisicus apertus*	SSBAB2734-12	KM835802
Araneae	Linyphiidae	*Sisis rotundus*	CNRMB228-12	KM831003
Araneae	Linyphiidae	*Sisis rotundus*	SPIRU1348-11	KF368782
Araneae	Linyphiidae	*Soucron arenarium*	SSWLB5807-13	KM832319
Araneae	Linyphiidae	*Souessa spinifera*	JSARA098-11	KP652316
Araneae	Linyphiidae	*Souessa spinifera*	SMTPB20880-13	KP656751
Araneae	Linyphiidae	*Soulgas corticarius*	CNSLQ082-13	KM827773
Araneae	Linyphiidae	*Soulgas corticarius*	JSARA080-11	KP650155
Araneae	Linyphiidae	*Spirembolus mundus*	CNEII006-13	KM831852
Araneae	Linyphiidae	*Spirembolus prominens*	SPRMA436-10	JF887011
Araneae	Linyphiidae	*Styloctetor stativus*	SSEIA2106-13	KM830218
Araneae	Linyphiidae	*Symmigma minimum*	SPIAL106-10	HQ580604
Araneae	Linyphiidae	*Symmigma minimum*	SPIAL169-10	HQ580642
Araneae	Linyphiidae	*Symmigma minimum*	SPIAL246-10	HQ580694
Araneae	Linyphiidae	*Tachygyna ursina*	CNGIH316-13	KM839110
Araneae	Linyphiidae	*Tachygyna ursina*	CNGIJ926-13	KM830305
Araneae	Linyphiidae	*Tachygyna ursina*	SPIAL011-10	HQ580524
Araneae	Linyphiidae	*Tachygyna ursina*	SPIAL082-10	HQ580584
Araneae	Linyphiidae	*Tachygyna ursina*	SPIAL094-10	HQ580594
Araneae	Linyphiidae	*Tachygyna ursina*	SPIAL283-10	HQ580722
Araneae	Linyphiidae	*Tachygyna ursina*	SPRMA224-10	HQ580835
Araneae	Linyphiidae	*Tachygyna ursina*	SPRMA330-10	HQ580895
Araneae	Linyphiidae	*Tachygyna vancouverana*	CNGIB549-12	KM837751
Araneae	Linyphiidae	*Tachygyna vancouverana*	CNGID207-12	KM826515
Araneae	Linyphiidae	*Tachygyna vancouverana*	CNGII555-13	KM830265
Araneae	Linyphiidae	*Tachygyna vancouverana*	CNGII556-13	KM828450

Araneae	Linyphiidae	*Tachygyna vancouverana*	CNGIJ925-13	KM834081
Araneae	Linyphiidae	*Tachygyna vancouverana*	CNGLA083-13	KM839483
Araneae	Linyphiidae	*Tachygyna vancouverana*	SPRMA441-10	JF887012
Araneae	Linyphiidae	*Tachygyna vancouverana*	SPRMA992-12	KP647734
Araneae	Linyphiidae	*Tachygyna vancouverana*	SPRMA995-12	KP653095
Araneae	Linyphiidae	*Tapinocyba affinis cf.*	CNWLB645-12	KM835871
Araneae	Linyphiidae	*Tapinocyba bicarinata*	SPICH031-09	GU683883
Araneae	Linyphiidae	*Tapinocyba cameroni*	SSEIC4639-13	KM828146
Araneae	Linyphiidae	*Tapinocyba cameroni*	SSEIC4641-13	KM824383
Araneae	Linyphiidae	*Tapinocyba cameroni*	SSEIC4653-13	KM829855
Araneae	Linyphiidae	*Tapinocyba cameroni*	SSEIC4659-13	KM836903
Araneae	Linyphiidae	*Tapinocyba cameroni*	SSEIC4677-13	KM824686
Araneae	Linyphiidae	*Tapinocyba cameroni*	SSEIC4878-13	KM838713
Araneae	Linyphiidae	*Tapinocyba cameroni*	SSJAC1464-13	KM837614
Araneae	Linyphiidae	*Tapinocyba cameroni*	SSJAC1495-13	KM824426
Araneae	Linyphiidae	*Tapinocyba cameroni*	SSJAF8176-13	KM826071
Araneae	Linyphiidae	*Tapinocyba cameroni*	SSJAF8224-13	KM825232
Araneae	Linyphiidae	*Tapinocyba cameroni*	SSJAF8631-13	KM833989
Araneae	Linyphiidae	*Tapinocyba cameroni*	SSPAB8856-13	KM828976
Araneae	Linyphiidae	*Tapinocyba cameroni*	SSPAB8857-13	KM824641
Araneae	Linyphiidae	*Tapinocyba cameroni*	SSPAB8860-13	KM831923
Araneae	Linyphiidae	*Tapinocyba cameroni*	SSPAB8864-13	KM826203
Araneae	Linyphiidae	*Tapinocyba cameroni*	SSPAB8865-13	KM827051
Araneae	Linyphiidae	*Tapinocyba cameroni*	SSPAB8866-13	KR069915
Araneae	Linyphiidae	*Tapinocyba cameroni*	SSPAB8870-13	KM832337
Araneae	Linyphiidae	*Tapinocyba cameroni*	SSPAB8879-13	KM826170
Araneae	Linyphiidae	*Tapinocyba cameroni*	SSPAB8880-13	KM838977
Araneae	Linyphiidae	*Tapinocyba dietrichi*	SPRMA338-10	HQ580898
Araneae	Linyphiidae	*Tapinocyba dietrichi*	SPRMA442-10	JF887013
Araneae	Linyphiidae	*Tapinocyba dietrichi*	SPRMA985-12	KP653802
Araneae	Linyphiidae	*Tapinocyba hortensis*	ERSPI278-09	GU682546
Araneae	Linyphiidae	*Tapinocyba hortensis*	ERSPI357-09	GU682485
Araneae	Linyphiidae	*Tapinocyba minuta*	SPICH189-09	HM432626
Araneae	Linyphiidae	*Tapinocyba minuta cf.*	SSWLB3472-13	KR070605
Araneae	Linyphiidae	*Tapinocyba minuta cf.*	SSWLB2441-13	KP653788
Araneae	Linyphiidae	*Tapinocyba minuta cf.*	SSWLB2445-13	KM828363
Araneae	Linyphiidae	*Tapinocyba minuta cf.*	SSWLB2462-13	KM840646
Araneae	Linyphiidae	*Tapinocyba minuta cf.*	SSWLB2489-13	KR070673
Araneae	Linyphiidae	*Tapinocyba minuta cf.*	SSWLB2490-13	KM826807
Araneae	Linyphiidae	*Tapinocyba minuta cf.*	SSWLB3458-13	KM830890
Araneae	Linyphiidae	*Tapinocyba minuta cf.*	SSWLB3467-13	KM837628
Araneae	Linyphiidae	*Tapinocyba minuta cf.*	SSWLB3543-13	KM829639
Araneae	Linyphiidae	*Tapinocyba simplex*	SSKJA4393-14	KR070487
Araneae	Linyphiidae	*Tapinocyba simplex*	SSKJA4395-14	KR070132
Araneae	Linyphiidae	*Tapinocyba simplex*	CNKOA207-14	KP647181
Araneae	Linyphiidae	*Tapinocyba simplex*	CNKOA212-14	KP645760
Araneae	Linyphiidae	*Tapinocyba simplex*	CNKOL120-14	KP652654
Araneae	Linyphiidae	*Tapinocyba simplex*	CNKOL124-14	KP655460
Araneae	Linyphiidae	*Tapinocyba simplex*	CNKOL135-14	KP656827
Araneae	Linyphiidae	*Tapinocyba simplex*	CNKOM676-14	KP656766
Araneae	Linyphiidae	*Tapinocyba simplex*	CNPKN239-14	KP651205
Araneae	Linyphiidae	*Tapinocyba simplex*	SSBRC3198-14	KR070545
Araneae	Linyphiidae	*Tapinocyba simplex*	SSBRC3199-14	KR069928

Araneae	Linyphiidae	*Tapinocyba simplex*	SSEIB7344-13	KM840576
Araneae	Linyphiidae	*Tapinocyba simplex*	SSEIB7686-13	KM830496
Araneae	Linyphiidae	*Tapinocyba simplex*	SSFDC085-14	KR069909
Araneae	Linyphiidae	*Tapinocyba simplex*	SSJAE11360-13	KM838541
Araneae	Linyphiidae	*Tapinocyba simplex*	SSPAA8054-13	KM840434
Araneae	Linyphiidae	*Tapinocyba simplex*	SSPAA8055-13	KM835395
Araneae	Linyphiidae	*Tapinocyba simplex*	SSPAA8056-13	KM840349
Araneae	Linyphiidae	*Tapinocyba simplex*	SSPAB8330-13	KM836165
Araneae	Linyphiidae	*Tapinocyba simplex*	SSPAB8861-13	KM832017
Araneae	Linyphiidae	*Tapinocyba sp. 3GAB*	CNSLC534-12	KM829127
Araneae	Linyphiidae	*Tennesseellum formica*	ERSPI383-09	HM376107
Araneae	Linyphiidae	*Tennesseellum formica*	GBCH11937-13	JN801187
Araneae	Linyphiidae	*Tennesseellum formica*	JSARA063-11	KP655658
Araneae	Linyphiidae	*Tennesseellum formica*	SMTPB10035-13	KP655715
Araneae	Linyphiidae	*Tennesseellum formica*	SMTPB6525-13	KP654887
Araneae	Linyphiidae	*Tennesseellum formica*	SMTPB8961-13	KP655532
Araneae	Linyphiidae	*Tennesseellum formica*	SMTPB9295-13	KP655564
Araneae	Linyphiidae	*Tennesseellum formica*	SPRMA1042-12	KP651887
Araneae	Linyphiidae	*Tenuiphantes tenuis*	CNGIA114-12	KM826933
Araneae	Linyphiidae	*Tenuiphantes tenuis*	CNGIB550-12	KM827855
Araneae	Linyphiidae	*Tenuiphantes tenuis*	GBCH0630-06	AY383539
Araneae	Linyphiidae	*Tenuiphantes tenuis*	GBCH12475-13	KC244266
Araneae	Linyphiidae	*Tenuiphantes tenuis*	GBCH12476-13	KC244265
Araneae	Linyphiidae	*Tenuiphantes tenuis*	GBCH12477-13	KC244264
Araneae	Linyphiidae	*Tenuiphantes tenuis*	GBCH12478-13	KC244263
Araneae	Linyphiidae	*Tenuiphantes tenuis*	GBCH12479-13	KC244262
Araneae	Linyphiidae	*Tenuiphantes tenuis*	GBCH12480-13	KC244261
Araneae	Linyphiidae	*Tenuiphantes tenuis*	GBCH12481-13	KC244260
Araneae	Linyphiidae	*Tenuiphantes tenuis*	GBCH12482-13	KC244259
Araneae	Linyphiidae	*Tenuiphantes tenuis*	GBCH12483-13	KC244258
Araneae	Linyphiidae	*Tenuiphantes tenuis*	GBCH12484-13	KC244257
Araneae	Linyphiidae	*Tenuiphantes tenuis*	GBCH12485-13	KC244256
Araneae	Linyphiidae	*Tenuiphantes tenuis*	GBCH2840-08	DQ504374
Araneae	Linyphiidae	*Tenuiphantes tenuis*	GBCH5700-13	FJ899827
Araneae	Linyphiidae	*Tenuiphantes tenuis*	SPRMA293-10	HQ977166
Araneae	Linyphiidae	*Tenuiphantes tenuis*	SPRMA310-10	HQ977177
Araneae	Linyphiidae	*Tenuiphantes tenuis*	SPRMA394-10	JF886999
Araneae	Linyphiidae	*Tenuiphantes zebra*	ARONT560-10	HQ924447
Araneae	Linyphiidae	*Tenuiphantes zebra*	RBINA1149-13	KP650522
Araneae	Linyphiidae	*Tenuiphantes zebra*	RBINA1164-13	KP652143
Araneae	Linyphiidae	*Tenuiphantes zelatus*	CNGIE396-12	KM840742
Araneae	Linyphiidae	*Tenuiphantes zelatus*	SPRMA393-10	JF886998
Araneae	Linyphiidae	*Tenuiphantes zelatus*	SPRMA395-10	JN310299
Araneae	Linyphiidae	*Tenuiphantes zelatus*	SPRMA966-12	KP645772
Araneae	Linyphiidae	*Tenuiphantes zibus*	SPIAL117-10	HQ580612
Araneae	Linyphiidae	*Tenuiphantes zibus*	SPRMA229-10	HQ580837
Araneae	Linyphiidae	*Tenuiphantes zibus*	SPRMA240-10	HQ580846
Araneae	Linyphiidae	*Tenuiphantes zibus*	SPRMA261-10	HQ580865
Araneae	Linyphiidae	*Tenuiphantes zibus*	SPRMA730-10	JF887152
Araneae	Linyphiidae	*Thyreosthenius parasiticus*	SMTPB10640-13	KP646429
Araneae	Linyphiidae	*Tiso aestivus*	KKCHE1057-09	GU683735
Araneae	Linyphiidae	*Tiso vagans*	SPRMA1120-12	KP655845
Araneae	Linyphiidae	*Tmeticus affinis*	SPICH1091-09	GU684611

Araneae	Linyphiidae	*Tmeticus ornatus*	SPIRU1179-11	KF368924
Araneae	Linyphiidae	*Tunagyna debilis*	SPICH1108-09	GU684637
Araneae	Linyphiidae	*Typhochrestus pygmaeus*	SPRMA1022-12	KP645520
Araneae	Linyphiidae	*Vermontia thoracica*	SSGBB5845-14	KP656512
Araneae	Linyphiidae	*Vermontia thoracica*	SSPAA7584-13	KM826743
Araneae	Linyphiidae	*Wabasso cacuminatus*	SPIRU1335-11	KF368932
Araneae	Linyphiidae	*Wabasso quaestio*	SPICH423-09	GU684195
Araneae	Linyphiidae	*Walckenaeria atrotibialis*	ARONT807-10	HQ924652
Araneae	Linyphiidae	*Walckenaeria atrotibialis*	RBINA5239-13	KP652139
Araneae	Linyphiidae	*Walckenaeria castanea*	ARONT547-10	HQ924434
Araneae	Linyphiidae	*Walckenaeria clavicornis*	SPICH085-09	GU683868
Araneae	Linyphiidae	*Walckenaeria columbia*	SPRMA112-10	HQ580750
Araneae	Linyphiidae	*Walckenaeria columbia*	SPRMA838-12	KP646704
Araneae	Linyphiidae	*Walckenaeria communis*	ARONT525-10	KP648318
Araneae	Linyphiidae	*Walckenaeria communis*	KKCHE535-07	KF368935
Araneae	Linyphiidae	*Walckenaeria communis*	SPRMA1050-12	KP649982
Araneae	Linyphiidae	*Walckenaeria cornuella*	SPRMA791-12	KP650463
Araneae	Linyphiidae	*Walckenaeria directa*	CNEIA2649-12	KM835704
Araneae	Linyphiidae	*Walckenaeria directa*	SPIAL236-10	HQ580685
Araneae	Linyphiidae	*Walckenaeria directa*	SPRMA1138-12	KP647237
Araneae	Linyphiidae	*Walckenaeria exigua*	SMTPB15972-13	KP649283
Araneae	Linyphiidae	*Walckenaeria exigua*	SPICH006-09	GU683863
Araneae	Linyphiidae	*Walckenaeria exigua*	SPRMA1121-12	KP652446
Araneae	Linyphiidae	*Walckenaeria fallax*	ARONT520-10	HQ924409
Araneae	Linyphiidae	*Walckenaeria fallax*	ERSPI270-09	HM376101
Araneae	Linyphiidae	*Walckenaeria fallax*	SMTPB16290-13	KP654109
Araneae	Linyphiidae	*Walckenaeria fallax*	SMTPB16294-13	KP655269
Araneae	Linyphiidae	*Walckenaeria fallax*	SMTPB21284-13	KP655986
Araneae	Linyphiidae	*Walckenaeria fallax*	SMTPB9595-13	KP646581
Araneae	Linyphiidae	*Walckenaeria kochi*	SPICH071-09	GU683871
Araneae	Linyphiidae	*Walckenaeria lepida*	CNEII004-13	KM825249
Araneae	Linyphiidae	*Walckenaeria lepida*	SPIRU975-11	KF368943
Araneae	Linyphiidae	*Walckenaeria obtusa*	GBCH11425-13	JQ801601
Araneae	Linyphiidae	*Walckenaeria obtusa*	GBCH11426-13	JQ801600
Araneae	Linyphiidae	*Walckenaeria palustris*	SSWLB3474-13	KM826678
Araneae	Linyphiidae	*Walckenaeria palustris*	SSWLE3113-13	KM832819
Araneae	Linyphiidae	*Walckenaeria sp. 3GAB*	SSJAC2356-13	KM837865
Araneae	Linyphiidae	*Walckenaeria spiralis*	SPICH038-09	HM432623
Araneae	Linyphiidae	*Walckenaeria tibialis*	ERSPI244-09	HM376095
Araneae	Linyphiidae	*Walckenaeria tibialis*	SMTPB13979-13	KP645697
Araneae	Linyphiidae	*Walckenaeria tricornis*	SPICH007-09	GU683865
Araneae	Linyphiidae	*Walckenaeria tumida*	SMTPB1071-13	KP645628
Araneae	Linyphiidae	*Walckenaeria tumida*	SMTPB8135-13	KP654180
Araneae	Linyphiidae	*Walckenaerianus aimakensis*	SPICH1089-09	GU684629
Araneae	Linyphiidae	*Wubana drassoides*	SPRMA272-10	HQ580873
Araneae	Linyphiidae	*Wubana pacifica*	SPRMA322-10	HQ580890
Araneae	Linyphiidae	*Wubana pacifica*	SPRMA327-10	HQ580893
Araneae	Linyphiidae	*Wubana pacifica*	SPRMA331-10	HQ580896
Araneae	Linyphiidae	*Wubana pacifica*	SPRMA448-10	KP648654
Araneae	Linyphiidae	*Wubana pacifica*	SPRMA449-10	KP650108
Araneae	Linyphiidae	*Wubana pacifica*	SPRMA965-12	KP652893
Araneae	Linyphiidae	*Wubana sp. 1JS*	SPIAL112-10	HQ580609
Araneae	Linyphiidae	*Zornella armata*	SPICH199-09	HM432627

Araneae	Linyphiidae	*Zornella armata*	SPRMA375-10	HQ580922
Araneae	Linyphiidae	*Zornella cryptodon*	SPRMA1085-12	KP646234
Araneae	Linyphiidae	*Zygottus corvallis*	SPRMA1091-12	KP656136
Araneae	Liocranidae	*Agroeca ornata*	ARONT463-10	HM880615
Araneae	Liocranidae	*Agroeca ornata*	ARONT466-10	HM880618
Araneae	Liocranidae	*Agroeca ornata*	ARONT468-10	HM880620
Araneae	Liocranidae	*Agroeca ornata*	ARONT800-10	HQ924645
Araneae	Liocranidae	*Agroeca ornata*	RBINA3146-13	KP647299
Araneae	Liocranidae	*Agroeca ornata*	SPICH122-09	GU683786
Araneae	Liocranidae	*Agroeca pratensis*	ERSPI184-09	KP654336
Araneae	Liocranidae	*Agroeca pratensis*	ERSPI215-09	KP652411
Araneae	Liphistiidae	*Liphistius erawan*	GBCH10810-13	NC_020323
Araneae	Liphistiidae	*Liphistius erawan*	GBCH11967-13	JQ407803
Araneae	Lycosidae	*Alopecosa aculeata*	KKCHE053-06	KF367815
Araneae	Lycosidae	*Alopecosa aculeata*	SPRMA168-10	HQ580788
Araneae	Lycosidae	*Alopecosa aculeata*	SSBAA5900-13	KM833717
Araneae	Lycosidae	*Alopecosa aculeata*	SSBAA5904-13	KM834283
Araneae	Lycosidae	*Alopecosa aculeata*	SSJAB1150-13	KM834031
Araneae	Lycosidae	*Alopecosa aculeata*	SSJAB2268-13	KM833652
Araneae	Lycosidae	*Alopecosa aculeata*	SSJAB3303-13	KM829674
Araneae	Lycosidae	*Alopecosa aculeata*	SSJAE4312-13	KM827820
Araneae	Lycosidae	*Alopecosa kochi*	SPRMA097-10	HQ580738
Araneae	Lycosidae	*Alopecosa kochi*	SPRMA541-10	JF887050
Araneae	Lycosidae	*Alopecosa kochi*	SPRMA967-12	KM254048
Araneae	Lycosidae	*Alopecosa koponeni*	KKCHE1008-09	GU683697
Araneae	Lycosidae	*Alopecosa licenti*	GBCH11093-13	JN817177
Araneae	Lycosidae	*Arctosa alpigena*	SSJAA824-13	KM831132
Araneae	Lycosidae	*Arctosa alpigena*	SSJAA833-13	KM833868
Araneae	Lycosidae	*Arctosa insignita*	SPIAL069-10	HQ580573
Araneae	Lycosidae	*Arctosa perita*	SPRMA543-10	JF887052
Araneae	Lycosidae	*Arctosa perita*	SPRMA545-10	JF887053
Araneae	Lycosidae	*Arctosa raptor*	JDTGS007-09	GU679905
Araneae	Lycosidae	*Arctosa raptor*	SPRMA207-10	HQ580821
Araneae	Lycosidae	*Arctosa raptor*	SPRMA208-10	HQ580822
Araneae	Lycosidae	*Arctosa rubicunda*	RBCH011-04	DQ127522
Araneae	Lycosidae	*Geolycosa escambiensis*	GBCH3030-08	DQ151684
Araneae	Lycosidae	*Geolycosa escambiensis*	GBCH3031-08	DQ151683
Araneae	Lycosidae	*Geolycosa escambiensis*	GBCH3032-08	DQ151682
Araneae	Lycosidae	*Geolycosa escambiensis*	GBCH3033-08	DQ151681
Araneae	Lycosidae	*Geolycosa escambiensis*	GBCH3034-08	DQ151680
Araneae	Lycosidae	*Geolycosa escambiensis*	GBCH3035-08	DQ151679
Araneae	Lycosidae	*Geolycosa escambiensis*	GBCH3036-08	DQ151678
Araneae	Lycosidae	*Geolycosa escambiensis*	GBCH3037-08	DQ151677
Araneae	Lycosidae	*Geolycosa escambiensis*	GBCH3038-08	DQ151676
Araneae	Lycosidae	*Geolycosa escambiensis*	GBCH3039-08	DQ151675
Araneae	Lycosidae	*Geolycosa escambiensis*	GBCH3040-08	DQ151674
Araneae	Lycosidae	*Geolycosa escambiensis*	GBCH3041-08	DQ151673
Araneae	Lycosidae	*Geolycosa escambiensis*	GBCH3042-08	DQ151672
Araneae	Lycosidae	*Geolycosa escambiensis*	GBCH3043-08	DQ151671
Araneae	Lycosidae	*Geolycosa escambiensis*	GBCH3044-08	DQ151670
Araneae	Lycosidae	*Geolycosa escambiensis*	GBCH3045-08	DQ151669
Araneae	Lycosidae	*Geolycosa fatifera*	GBCH3028-08	DQ151686
Araneae	Lycosidae	*Geolycosa fatifera*	GBCH3029-08	DQ151685

Araneae	Lycosidae	*Geolycosa micanopy*	GBCH2983-08	DQ151731
Araneae	Lycosidae	*Geolycosa micanopy*	GBCH2984-08	DQ151730
Araneae	Lycosidae	*Geolycosa micanopy*	GBCH2985-08	DQ151729
Araneae	Lycosidae	*Geolycosa micanopy*	GBCH2986-08	DQ151728
Araneae	Lycosidae	*Geolycosa micanopy*	GBCH2987-08	DQ151727
Araneae	Lycosidae	*Geolycosa micanopy*	GBCH2988-08	DQ151726
Araneae	Lycosidae	*Geolycosa micanopy*	GBCH2989-08	DQ151725
Araneae	Lycosidae	*Geolycosa micanopy*	GBCH2990-08	DQ151724
Araneae	Lycosidae	*Geolycosa micanopy*	GBCH2991-08	DQ151723
Araneae	Lycosidae	*Geolycosa micanopy*	GBCH2992-08	DQ151722
Araneae	Lycosidae	*Geolycosa micanopy*	GBCH2993-08	DQ151721
Araneae	Lycosidae	*Geolycosa micanopy*	GBCH2994-08	DQ151720
Araneae	Lycosidae	*Geolycosa micanopy*	GBCH2995-08	DQ151719
Araneae	Lycosidae	*Geolycosa micanopy*	GBCH2996-08	DQ151718
Araneae	Lycosidae	*Geolycosa micanopy*	GBCH2997-08	DQ151717
Araneae	Lycosidae	*Geolycosa micanopy*	GBCH2998-08	DQ151716
Araneae	Lycosidae	*Geolycosa micanopy*	GBCH2999-08	DQ151715
Araneae	Lycosidae	*Geolycosa micanopy*	GBCH3000-08	DQ151714
Araneae	Lycosidae	*Geolycosa micanopy*	GBCH3001-08	DQ151713
Araneae	Lycosidae	*Geolycosa micanopy*	GBCH3002-08	DQ151712
Araneae	Lycosidae	*Geolycosa micanopy*	GBCH3003-08	DQ151711
Araneae	Lycosidae	*Geolycosa micanopy*	GBCH3004-08	DQ151710
Araneae	Lycosidae	*Geolycosa micanopy*	GBCH3005-08	DQ151709
Araneae	Lycosidae	*Geolycosa micanopy*	GBCH3006-08	DQ151708
Araneae	Lycosidae	*Geolycosa micanopy*	GBCH3007-08	DQ151707
Araneae	Lycosidae	*Geolycosa micanopy*	GBCH3008-08	DQ151706
Araneae	Lycosidae	*Geolycosa micanopy*	GBCH3009-08	DQ151705
Araneae	Lycosidae	*Geolycosa micanopy*	GBCH3010-08	DQ151704
Araneae	Lycosidae	*Geolycosa micanopy*	GBCH3011-08	DQ151703
Araneae	Lycosidae	*Geolycosa micanopy*	GBCH3012-08	DQ151702
Araneae	Lycosidae	*Geolycosa micanopy*	GBCH3013-08	DQ151701
Araneae	Lycosidae	*Geolycosa micanopy*	GBCH3014-08	DQ151700
Araneae	Lycosidae	*Geolycosa micanopy*	GBCH3015-08	DQ151699
Araneae	Lycosidae	*Geolycosa micanopy*	GBCH3016-08	DQ151698
Araneae	Lycosidae	*Geolycosa micanopy*	GBCH3017-08	DQ151697
Araneae	Lycosidae	*Geolycosa micanopy*	GBCH3018-08	DQ151696
Araneae	Lycosidae	*Geolycosa micanopy*	GBCH3019-08	DQ151695
Araneae	Lycosidae	*Geolycosa ornatipes*	GBCH2979-08	DQ151735
Araneae	Lycosidae	*Geolycosa patellonigra*	GBCH2951-08	DQ151763
Araneae	Lycosidae	*Geolycosa patellonigra*	GBCH2952-08	DQ151762
Araneae	Lycosidae	*Geolycosa patellonigra*	GBCH2953-08	DQ151761
Araneae	Lycosidae	*Geolycosa patellonigra*	GBCH2955-08	DQ151759
Araneae	Lycosidae	*Geolycosa patellonigra*	GBCH2956-08	DQ151758
Araneae	Lycosidae	*Geolycosa patellonigra*	GBCH2957-08	DQ151757
Araneae	Lycosidae	*Geolycosa patellonigra*	GBCH2958-08	DQ151756
Araneae	Lycosidae	*Geolycosa patellonigra*	GBCH2959-08	DQ151755
Araneae	Lycosidae	*Geolycosa patellonigra*	GBCH2960-08	DQ151754
Araneae	Lycosidae	*Geolycosa patellonigra*	GBCH2961-08	DQ151753
Araneae	Lycosidae	*Geolycosa patellonigra*	GBCH2962-08	DQ151752
Araneae	Lycosidae	*Geolycosa patellonigra*	GBCH2963-08	DQ151751
Araneae	Lycosidae	*Geolycosa patellonigra*	GBCH2964-08	DQ151750
Araneae	Lycosidae	*Geolycosa patellonigra*	GBCH2965-08	DQ151749
Araneae	Lycosidae	*Geolycosa patellonigra*	GBCH2966-08	DQ151748

Araneae	Lycosidae	*Geolycosa patellonigra*	GBCH2967-08	DQ151747
Araneae	Lycosidae	*Geolycosa patellonigra*	GBCH2968-08	DQ151746
Araneae	Lycosidae	*Geolycosa patellonigra*	GBCH2969-08	DQ151745
Araneae	Lycosidae	*Geolycosa patellonigra*	GBCH2970-08	DQ151744
Araneae	Lycosidae	*Geolycosa patellonigra*	GBCH2971-08	DQ151743
Araneae	Lycosidae	*Geolycosa patellonigra*	GBCH2972-08	DQ151742
Araneae	Lycosidae	*Geolycosa patellonigra*	GBCH2973-08	DQ151741
Araneae	Lycosidae	*Geolycosa patellonigra*	GBCH2974-08	DQ151740
Araneae	Lycosidae	*Geolycosa patellonigra*	GBCH2975-08	DQ151739
Araneae	Lycosidae	*Geolycosa patellonigra*	GBCH2976-08	DQ151738
Araneae	Lycosidae	*Geolycosa patellonigra*	GBCH2977-08	DQ151737
Araneae	Lycosidae	*Geolycosa patellonigra*	GBCH2978-08	DQ151736
Araneae	Lycosidae	*Geolycosa pikei*	GBCH2948-08	DQ151766
Araneae	Lycosidae	*Geolycosa pikei*	GBCH2949-08	DQ151765
Araneae	Lycosidae	*Geolycosa pikei*	GBCH2950-08	DQ151764
Araneae	Lycosidae	*Geolycosa rafaelana*	GBCH2945-08	DQ151769
Araneae	Lycosidae	*Geolycosa rafaelana*	GBCH2946-08	DQ151768
Araneae	Lycosidae	*Geolycosa rafaelana*	GBCH2947-08	DQ151767
Araneae	Lycosidae	*Geolycosa riograndae*	GBCH2943-08	DQ151771
Araneae	Lycosidae	*Geolycosa riograndae*	GBCH2944-08	DQ151770
Araneae	Lycosidae	*Geolycosa turricola*	GBCH2934-08	DQ151780
Araneae	Lycosidae	*Geolycosa turricola*	GBCH2935-08	DQ151779
Araneae	Lycosidae	*Geolycosa turricola*	GBCH2936-08	DQ151778
Araneae	Lycosidae	*Geolycosa turricola*	GBCH2937-08	DQ151777
Araneae	Lycosidae	*Geolycosa turricola*	GBCH2938-08	DQ151776
Araneae	Lycosidae	*Geolycosa turricola*	GBCH2939-08	DQ151775
Araneae	Lycosidae	*Geolycosa turricola*	GBCH2940-08	DQ151774
Araneae	Lycosidae	*Geolycosa turricola*	GBCH2941-08	DQ151773
Araneae	Lycosidae	*Geolycosa vultuosa*	GBCH2896-08	DQ151818
Araneae	Lycosidae	*Geolycosa wrighti*	GBCH2931-08	DQ151783
Araneae	Lycosidae	*Geolycosa wrighti*	GBCH2932-08	DQ151782
Araneae	Lycosidae	*Geolycosa wrighti*	GBCH2933-08	DQ151781
Araneae	Lycosidae	*Geolycosa xera*	GBCH2898-08	DQ151816
Araneae	Lycosidae	*Geolycosa xera*	GBCH2899-08	DQ151815
Araneae	Lycosidae	*Geolycosa xera*	GBCH2900-08	DQ151814
Araneae	Lycosidae	*Geolycosa xera*	GBCH2901-08	DQ151813
Araneae	Lycosidae	*Geolycosa xera*	GBCH2902-08	DQ151812
Araneae	Lycosidae	*Geolycosa xera*	GBCH2903-08	DQ151811
Araneae	Lycosidae	*Geolycosa xera*	GBCH2904-08	DQ151810
Araneae	Lycosidae	*Geolycosa xera*	GBCH2905-08	DQ151809
Araneae	Lycosidae	*Geolycosa xera*	GBCH2906-08	DQ151808
Araneae	Lycosidae	*Geolycosa xera*	GBCH2907-08	DQ151807
Araneae	Lycosidae	*Geolycosa xera*	GBCH2908-08	DQ151806
Araneae	Lycosidae	*Geolycosa xera*	GBCH2909-08	DQ151805
Araneae	Lycosidae	*Geolycosa xera*	GBCH2910-08	DQ151804
Araneae	Lycosidae	*Geolycosa xera*	GBCH2911-08	DQ151803
Araneae	Lycosidae	*Geolycosa xera*	GBCH2912-08	DQ151802
Araneae	Lycosidae	*Geolycosa xera*	GBCH2913-08	DQ151801
Araneae	Lycosidae	*Geolycosa xera*	GBCH2914-08	DQ151800
Araneae	Lycosidae	*Geolycosa xera*	GBCH2915-08	DQ151799
Araneae	Lycosidae	*Geolycosa xera*	GBCH2916-08	DQ151798
Araneae	Lycosidae	*Geolycosa xera*	GBCH2917-08	DQ151797
Araneae	Lycosidae	*Geolycosa xera*	GBCH2918-08	DQ151796

Araneae	Lycosidae	*Geolycosa xera*	GBCH2919-08	DQ151795
Araneae	Lycosidae	*Geolycosa xera*	GBCH2920-08	DQ151794
Araneae	Lycosidae	*Geolycosa xera*	GBCH2921-08	DQ151793
Araneae	Lycosidae	*Geolycosa xera*	GBCH2922-08	DQ151792
Araneae	Lycosidae	*Geolycosa xera*	GBCH2923-08	DQ151791
Araneae	Lycosidae	*Geolycosa xera*	GBCH2924-08	DQ151790
Araneae	Lycosidae	*Geolycosa xera*	GBCH2925-08	DQ151789
Araneae	Lycosidae	*Geolycosa xera*	GBCH2926-08	DQ151788
Araneae	Lycosidae	*Geolycosa xera*	GBCH2927-08	DQ151787
Araneae	Lycosidae	*Geolycosa xera*	GBCH2928-08	DQ151786
Araneae	Lycosidae	*Geolycosa xera*	GBCH2929-08	DQ151785
Araneae	Lycosidae	*Geolycosa xera*	GBCH2930-08	DQ151784
Araneae	Lycosidae	*Gladicosa gulosa*	RBINA3389-13	KP656699
Araneae	Lycosidae	*Gladicosa gulosa*	RBINA3421-13	KP651593
Araneae	Lycosidae	*Gladicosa gulosa*	SPRMA542-10	JF887051
Araneae	Lycosidae	*Hogna carolinensis*	GBCH2888-08	DQ151826
Araneae	Lycosidae	*Hogna carolinensis*	GBCH2889-08	DQ151825
Araneae	Lycosidae	*Hogna carolinensis*	GBCH2890-08	DQ151824
Araneae	Lycosidae	*Hogna carolinensis*	SPRMA367-10	HQ580917
Araneae	Lycosidae	*Hogna espanola*	GBCH5763-13	HM171122
Araneae	Lycosidae	*Hogna frondicola*	SPRMA142-10	KP649116
Araneae	Lycosidae	*Hogna galapagoensis*	GBCH5764-13	HM171121
Araneae	Lycosidae	*Hogna galapagoensis*	GBCH5765-13	HM171120
Araneae	Lycosidae	*Hogna junco*	GBCH5768-13	HM171117
Araneae	Lycosidae	*Hogna junco*	GBCH5769-13	HM171116
Araneae	Lycosidae	*Hogna snodgrassi*	GBCH5766-13	HM171119
Araneae	Lycosidae	*Hogna snodgrassi*	GBCH5767-13	HM171118
Araneae	Lycosidae	*Lycosa godeffroyi*	GBCH2895-08	DQ151819
Araneae	Lycosidae	*Pardosa altamontis*	SPRMA550-10	JF887057
Araneae	Lycosidae	*Pardosa altamontis*	SPRMA963-12	KP653003
Araneae	Lycosidae	*Pardosa amentata*	GBCH5703-13	FJ899820
Araneae	Lycosidae	*Pardosa astrigera*	GBCH11088-13	JN817182
Araneae	Lycosidae	*Pardosa brevivulva*	GBCH11084-13	JN817186
Araneae	Lycosidae	*Pardosa brevivulva*	GBCH11087-13	JN817183
Araneae	Lycosidae	*Pardosa distincta*	ERSPI354-09	GU682493
Araneae	Lycosidae	*Pardosa distincta*	SPRMA143-10	HQ580771
Araneae	Lycosidae	*Pardosa distincta*	SPRMA295-10	HQ977167
Araneae	Lycosidae	*Pardosa distincta*	SPRMA317-10	HQ580886
Araneae	Lycosidae	*Pardosa distincta*	SPRMA551-10	JF887058
Araneae	Lycosidae	*Pardosa distincta*	SPRMA883-12	KP655138
Araneae	Lycosidae	*Pardosa dorsuncata*	CNPCD036-12	KM831227
Araneae	Lycosidae	*Pardosa dorsuncata*	SPRMA105-10	HQ580745
Araneae	Lycosidae	*Pardosa dorsuncata*	SPRMA125-10	HQ580759
Araneae	Lycosidae	*Pardosa dorsuncata*	SPRMA215-10	HQ580828
Araneae	Lycosidae	*Pardosa dorsuncata*	SPRMA259-10	HQ580863
Araneae	Lycosidae	*Pardosa dorsuncata*	SPRMA314-10	HQ977180
Araneae	Lycosidae	*Pardosa dorsuncata*	SPRMA553-10	JF887059
Araneae	Lycosidae	*Pardosa dorsuncata*	SPRMA554-10	JF887060
Araneae	Lycosidae	*Pardosa dorsuncata*	SPRMA555-10	JF887061
Araneae	Lycosidae	*Pardosa fuscula*	KKCHE032-06	KF368417
Araneae	Lycosidae	*Pardosa fuscula*	SPRMA694-10	JF887128
Araneae	Lycosidae	*Pardosa giebeli*	GBCH7617-13	JQ746510
Araneae	Lycosidae	*Pardosa glacialis*	SPUCH001-12	KF368428

Araneae	Lycosidae	*Pardosa hedini*	GBCH11085-13	JN817185
Araneae	Lycosidae	*Pardosa hyperborea*	JDTGS003-09	GU679909
Araneae	Lycosidae	*Pardosa hyperborea*	SPRMA559-10	JF887064
Araneae	Lycosidae	*Pardosa hyperborea*	SSBAA5246-12	KM834335
Araneae	Lycosidae	*Pardosa hyperborea*	SSBAC2870-12	KM824495
Araneae	Lycosidae	*Pardosa hyperborea*	SSBAC2874-12	KM839659
Araneae	Lycosidae	*Pardosa hyperborea*	SSBAC2899-12	KM836521
Araneae	Lycosidae	*Pardosa hyperborea*	SSBAF7670-13	KM826462
Araneae	Lycosidae	*Pardosa hyperborea*	SSBAF7682-13	KM831072
Araneae	Lycosidae	*Pardosa hyperborea*	SSBAF7684-13	KM827521
Araneae	Lycosidae	*Pardosa hyperborea*	SSBAF7689-13	KM829482
Araneae	Lycosidae	*Pardosa lapidicina*	ARONT003-09	GU682784
Araneae	Lycosidae	*Pardosa lapidicina*	ARONT004-09	GU682781
Araneae	Lycosidae	*Pardosa lapidicina*	ARONT611-10	HQ924476
Araneae	Lycosidae	*Pardosa lapidicina*	ARONT612-10	HQ924477
Araneae	Lycosidae	*Pardosa lapidicina*	RBCH122-04	DQ127410
Araneae	Lycosidae	*Pardosa lapidicina*	RBCH186-04	DQ127348
Araneae	Lycosidae	*Pardosa lapponica*	CNJAC1061-12	KM825649
Araneae	Lycosidae	*Pardosa lapponica*	SPRMA376-10	HQ580923
Araneae	Lycosidae	*Pardosa lapponica*	SPRMA556-10	KP651150
Araneae	Lycosidae	*Pardosa laura*	GBCH11086-13	JN817184
Araneae	Lycosidae	*Pardosa lowriei*	SPRMA558-10	JF887063
Araneae	Lycosidae	*Pardosa lowriei*	SPRMA710-10	KP649056
Araneae	Lycosidae	*Pardosa metlakatla*	SPRMA562-10	JF887066
Araneae	Lycosidae	*Pardosa metlakatla*	SPRMA580-10	JF887072
Araneae	Lycosidae	*Pardosa milvina*	ARONT030-09	GU682783
Araneae	Lycosidae	*Pardosa milvina*	ARONT318-09	GU682881
Araneae	Lycosidae	*Pardosa milvina*	ARONT522-10	HQ924411
Araneae	Lycosidae	*Pardosa milvina*	ARONT591-10	HQ924466
Araneae	Lycosidae	*Pardosa milvina*	ARONT631-10	HQ924496
Araneae	Lycosidae	*Pardosa milvina*	ARONT665-10	HQ924525
Araneae	Lycosidae	*Pardosa milvina*	ARONT816-10	HQ924661
Araneae	Lycosidae	*Pardosa milvina*	ERSPI325-09	GU682515
Araneae	Lycosidae	*Pardosa milvina*	ERSPI395-09	GU682447
Araneae	Lycosidae	*Pardosa milvina*	GBCH1197-06	DQ029231
Araneae	Lycosidae	*Pardosa milvina*	GBCH2892-08	DQ151822
Araneae	Lycosidae	*Pardosa milvina*	RBCH102-04	DQ127422
Araneae	Lycosidae	*Pardosa milvina*	RBINA3418-13	KP653237
Araneae	Lycosidae	*Pardosa milvina*	RBINA5813-13	KP653804
Araneae	Lycosidae	*Pardosa milvina*	RBINA5836-13	KP656586
Araneae	Lycosidae	*Pardosa milvina*	SMTPB10387-13	KP647089
Araneae	Lycosidae	*Pardosa milvina*	SMTPB1416-13	KP647406
Araneae	Lycosidae	*Pardosa milvina*	SMTPB2320-13	KP647501
Araneae	Lycosidae	*Pardosa milvina*	SMTPB3439-13	KP650076
Araneae	Lycosidae	*Pardosa milvina*	SMTPB3440-13	KP652991
Araneae	Lycosidae	*Pardosa milvina*	SMTPB3441-13	KP651759
Araneae	Lycosidae	*Pardosa milvina*	SMTPB4012-13	KP645516
Araneae	Lycosidae	*Pardosa milvina*	SMTPB7322-13	KP651315
Araneae	Lycosidae	*Pardosa moesta*	ARONT457-10	HM880611
Araneae	Lycosidae	*Pardosa moesta*	ARONT527-10	HQ924415
Araneae	Lycosidae	*Pardosa moesta*	CNEIH007-13	KM833688
Araneae	Lycosidae	*Pardosa moesta*	CNEIH011-13	KM834391
Araneae	Lycosidae	*Pardosa moesta*	CNJAC1060-12	KM831137

Araneae	Lycosidae	*Pardosa moesta*	ERSPI252-09	GU682566
Araneae	Lycosidae	*Pardosa moesta*	JSARA023-11	KP652835
Araneae	Lycosidae	*Pardosa moesta*	PHMTT974-10	JN307912
Araneae	Lycosidae	*Pardosa moesta*	RBCH006-04	DQ127518
Araneae	Lycosidae	*Pardosa moesta*	RBINA2352-13	KP650001
Araneae	Lycosidae	*Pardosa moesta*	RBINA2386-13	KP652011
Araneae	Lycosidae	*Pardosa moesta*	RBINA2672-13	KP653275
Araneae	Lycosidae	*Pardosa moesta*	RBINA3299-13	KP649068
Araneae	Lycosidae	*Pardosa moesta*	RBINA3362-13	KP647603
Araneae	Lycosidae	*Pardosa moesta*	RBINA3386-13	KP647852
Araneae	Lycosidae	*Pardosa moesta*	RBINA3406-13	KP648067
Araneae	Lycosidae	*Pardosa moesta*	RBINA4694-13	KP650206
Araneae	Lycosidae	*Pardosa moesta*	RBINA556-13	KP656813
Araneae	Lycosidae	*Pardosa moesta*	RBINA5838-13	KP645814
Araneae	Lycosidae	*Pardosa moesta*	SMTPB10393-13	KP650225
Araneae	Lycosidae	*Pardosa moesta*	SMTPB10394-13	KP647449
Araneae	Lycosidae	*Pardosa moesta*	SMTPB10395-13	KP653155
Araneae	Lycosidae	*Pardosa moesta*	SMTPB1070-13	KP654355
Araneae	Lycosidae	*Pardosa moesta*	SMTPB11479-13	KP656678
Araneae	Lycosidae	*Pardosa moesta*	SMTPB11482-13	KP649963
Araneae	Lycosidae	*Pardosa moesta*	SMTPB13465-13	KP646476
Araneae	Lycosidae	*Pardosa moesta*	SMTPB13976-13	KP649123
Araneae	Lycosidae	*Pardosa moesta*	SMTPB14448-13	KP648749
Araneae	Lycosidae	*Pardosa moesta*	SMTPB1487-13	KP648434
Araneae	Lycosidae	*Pardosa moesta*	SMTPB15384-13	KP651378
Araneae	Lycosidae	*Pardosa moesta*	SMTPB17900-13	KP645935
Araneae	Lycosidae	*Pardosa moesta*	SMTPB18173-13	KP652084
Araneae	Lycosidae	*Pardosa moesta*	SMTPB18975-13	KP649648
Araneae	Lycosidae	*Pardosa moesta*	SMTPB20577-13	KP654561
Araneae	Lycosidae	*Pardosa moesta*	SMTPB5259-13	KP648427
Araneae	Lycosidae	*Pardosa moesta*	SMTPB7404-13	KP653175
Araneae	Lycosidae	*Pardosa moesta*	SPIAL120-10	HQ580614
Araneae	Lycosidae	*Pardosa moesta*	SPIAL132-10	HQ580622
Araneae	Lycosidae	*Pardosa moesta*	SPRMA211-10	HQ580825
Araneae	Lycosidae	*Pardosa moesta*	SPRMA218-10	HQ580830
Araneae	Lycosidae	*Pardosa moesta*	SPRMA312-10	HQ977178
Araneae	Lycosidae	*Pardosa moesta*	SPRMA560-10	KP648995
Araneae	Lycosidae	*Pardosa moesta*	SPRMA561-10	JF887065
Araneae	Lycosidae	*Pardosa mulaiki*	SSEIA5762-13	KM840327
Araneae	Lycosidae	*Pardosa nigra*	GBCH7613-13	JQ746514
Araneae	Lycosidae	*Pardosa nigra*	GBCH7614-13	JQ746513
Araneae	Lycosidae	*Pardosa nigra*	GBCH7615-13	JQ746512
Araneae	Lycosidae	*Pardosa ontariensis*	SPRMA547-10	JF887054
Araneae	Lycosidae	*Pardosa palustris*	GBCH5702-13	FJ899821
Araneae	Lycosidae	*Pardosa prativaga*	GBCH5701-13	FJ899822
Araneae	Lycosidae	*Pardosa saturatior*	GBCH7616-13	JQ746511
Araneae	Lycosidae	*Pardosa saxatilis*	ARONT664-10	HQ924524
Araneae	Lycosidae	*Pardosa saxatilis*	ERSPI006-08	KP653324
Araneae	Lycosidae	*Pardosa saxatilis*	ERSPI255-09	GU682558
Araneae	Lycosidae	*Pardosa saxatilis*	ERSPI432-09	GU682435
Araneae	Lycosidae	*Pardosa saxatilis*	SMTPB11481-13	KP656926
Araneae	Lycosidae	*Pardosa saxatilis*	SMTPB21291-13	KP656437
Araneae	Lycosidae	*Pardosa saxatilis*	SMTPB21295-13	KP646613

Araneae	Lycosidae	*Pardosa saxatilis*	SMTPB8346-13	KP652228
Araneae	Lycosidae	*Pardosa sp. 1GAB*	SPRMA369-10	HQ580918
Araneae	Lycosidae	*Pardosa tesquorum*	SPRMA958-12	KP652395
Araneae	Lycosidae	*Pardosa uintana*	JDTGS011-09	GU679901
Araneae	Lycosidae	*Pardosa vancouveri*	SPRMA564-10	JF887067
Araneae	Lycosidae	*Pardosa vancouveri*	SPRMA565-10	JF887068
Araneae	Lycosidae	*Pardosa vancouveri*	SPRMA748-10	KP650569
Araneae	Lycosidae	*Pardosa wagleri*	GBCH0810-06	AY560800
Araneae	Lycosidae	*Pardosa wyuta*	SPRMA102-10	HQ580743
Araneae	Lycosidae	*Pardosa wyuta*	SPRMA316-10	HQ580885
Araneae	Lycosidae	*Pardosa wyuta*	SPRMA566-10	JN310303
Araneae	Lycosidae	*Pardosa wyuta*	SPRMA567-10	JF887069
Araneae	Lycosidae	*Pardosa wyuta*	SPRMA788-12	KP650600
Araneae	Lycosidae	*Pardosa xerampelina*	ARONT456-10	HM880610
Araneae	Lycosidae	*Pardosa xerampelina*	ERSPI019-08	KP645462
Araneae	Lycosidae	*Pardosa xerampelina*	ERSPI064-08	KP649716
Araneae	Lycosidae	*Pardosa xerampelina*	ERSPI066-08	KP645432
Araneae	Lycosidae	*Pirata bryantae*	SPISH002-09	HM432633
Araneae	Lycosidae	*Pirata montanus*	ERSPI116-09	KP656761
Araneae	Lycosidae	*Pirata montanus*	ERSPI277-09	GU682545
Araneae	Lycosidae	*Pirata montanus*	ERSPI350-09	GU682490
Araneae	Lycosidae	*Pirata montanus*	ERSPI363-09	GU682479
Araneae	Lycosidae	*Pirata piraticus*	ARONT207-09	GU682709
Araneae	Lycosidae	*Pirata piraticus*	ARONT590-10	HQ924465
Araneae	Lycosidae	*Pirata piraticus*	JSMAY1528-12	KP654867
Araneae	Lycosidae	*Pirata piraticus*	PHMTU071-10	JN307937
Araneae	Lycosidae	*Pirata piraticus*	PHMTU091-10	JN307948
Araneae	Lycosidae	*Pirata piraticus*	SMTPB14439-13	KP649475
Araneae	Lycosidae	*Pirata piraticus*	SMTPB4011-13	KP651930
Araneae	Lycosidae	*Pirata piraticus*	SPIAL045-10	HQ580554
Araneae	Lycosidae	*Pirata piraticus*	SPIAL096-10	HQ580596
Araneae	Lycosidae	*Pirata piraticus*	SPIAL108-10	HQ580606
Araneae	Lycosidae	*Pirata piraticus*	SPRMA095-10	HQ977160
Araneae	Lycosidae	*Pirata piraticus*	SPRMA221-10	HQ580832
Araneae	Lycosidae	*Pirata piraticus*	SPRMA568-10	JF887070
Araneae	Lycosidae	*Pirata piraticus*	SPRMA570-10	KP655230
Araneae	Lycosidae	*Pirata piraticus*	SPRMA571-10	JF887071
Araneae	Lycosidae	*Pirata piraticus*	SPRMA582-10	KP654923
Araneae	Lycosidae	*Pirata praedo*	ARONT427-09	GU682896
Araneae	Lycosidae	*Pirata praedo*	RBINA3369-13	KP651650
Araneae	Lycosidae	*Pirata procurvus*	GBCH11081-13	JN817189
Araneae	Lycosidae	*Pirata sedentarius*	ARONT109-09	GU682628
Araneae	Lycosidae	*Pirata sedentarius*	ARONT110-09	GU682625
Araneae	Lycosidae	*Pirata sedentarius*	ARONT111-09	GU682626
Araneae	Lycosidae	*Pirata sedentarius*	ARONT112-09	GU682627
Araneae	Lycosidae	*Pirata sedentarius*	ARONT113-09	GU682623
Araneae	Lycosidae	*Pirata sedentarius*	ARONT444-09	GU682897
Araneae	Lycosidae	*Pirata sedentarius*	GBCH10804-13	KC662169
Araneae	Lycosidae	*Pirata sp. 1GAB*	ARONT446-09	HM434070
Araneae	Lycosidae	*Pirata subpiraticus*	GBCH11083-13	JN817187
Araneae	Lycosidae	*Pirata tanakai*	GBCH11080-13	JN817190
Araneae	Lycosidae	*Piratula cantralli*	ARONT208-09	GU682706
Araneae	Lycosidae	*Piratula cantralli*	ARONT513-10	HQ924404

Araneae	Lycosidae	*Piratula cantralli*	ARONT523-10	HQ924412
Araneae	Lycosidae	*Piratula cantralli*	ERSPI260-09	GU682562
Araneae	Lycosidae	*Piratula cantralli*	ERSPI275-09	GU682543
Araneae	Lycosidae	*Piratula cantralli*	ERSPI276-09	GU682544
Araneae	Lycosidae	*Piratula cantralli*	ERSPI431-09	GU682436
Araneae	Lycosidae	*Piratula cantralli*	HEAPR3742-12	KP651252
Araneae	Lycosidae	*Piratula cantralli*	RBINA3268-13	KP651157
Araneae	Lycosidae	*Piratula cantralli*	RBINA5163-13	KP650162
Araneae	Lycosidae	*Piratula cantralli*	SMTPB14668-13	KP646057
Araneae	Lycosidae	*Piratula insularis*	SSBAC2871-12	KM827615
Araneae	Lycosidae	*Piratula insularis*	SSBAC2872-12	KM830983
Araneae	Lycosidae	*Piratula insularis*	SSBAC2876-12	KM826296
Araneae	Lycosidae	*Piratula insularis*	SSBAC2882-12	KM835125
Araneae	Lycosidae	*Piratula insularis*	SSBAC2883-12	KM832564
Araneae	Lycosidae	*Piratula insularis*	SSBAC2884-12	KM826569
Araneae	Lycosidae	*Piratula insularis*	SSBAC2891-12	KM826117
Araneae	Lycosidae	*Piratula insularis*	SSBAC2892-12	KM836693
Araneae	Lycosidae	*Piratula insularis*	SSBAC2901-12	KM833656
Araneae	Lycosidae	*Piratula insularis*	SSBAC2902-12	KM830014
Araneae	Lycosidae	*Piratula insularis*	SSBAC2903-12	KM839805
Araneae	Lycosidae	*Piratula insularis*	SSBAC2904-12	KM834781
Araneae	Lycosidae	*Piratula insularis*	SSBAC2905-12	KM835588
Araneae	Lycosidae	*Piratula insularis*	SSBAC2907-12	KM836207
Araneae	Lycosidae	*Piratula insularis*	SSBAC2909-12	KM828251
Araneae	Lycosidae	*Piratula insularis*	SSBAC2913-12	KM838990
Araneae	Lycosidae	*Piratula insularis*	SSBAC4364-13	KM836297
Araneae	Lycosidae	*Piratula insularis*	SSBAF6164-13	KM830647
Araneae	Lycosidae	*Piratula insularis*	SSBAF7681-13	KM826155
Araneae	Lycosidae	*Piratula insularis*	SSBAF7691-13	KM838660
Araneae	Lycosidae	*Piratula insularis*	SSBAF7692-13	KM833165
Araneae	Lycosidae	*Piratula insularis*	SSBAF7695-13	KM837888
Araneae	Lycosidae	*Piratula insularis*	SSBAF7698-13	KM825547
Araneae	Lycosidae	*Piratula insularis*	SSBAF7703-13	KM836588
Araneae	Lycosidae	*Piratula insularis*	SSBAF7705-13	KM825304
Araneae	Lycosidae	*Piratula insularis*	SSBAF7706-13	KM840709
Araneae	Lycosidae	*Piratula insularis*	SSBAF7708-13	KM824632
Araneae	Lycosidae	*Piratula insularis*	SSPAA6007-13	KM831158
Araneae	Lycosidae	*Piratula insularis*	SSPAA6021-13	KM831503
Araneae	Lycosidae	*Piratula insularis*	SSPAA6025-13	KM835720
Araneae	Lycosidae	*Piratula insularis*	SSPAA6038-13	KM837739
Araneae	Lycosidae	*Piratula insularis*	SSPAA6040-13	KM830669
Araneae	Lycosidae	*Piratula insularis*	SSPAA6044-13	KM829357
Araneae	Lycosidae	*Piratula insularis*	SSPAA6047-13	KM828946
Araneae	Lycosidae	*Piratula insularis*	SSPAA6052-13	KM829981
Araneae	Lycosidae	*Piratula insularis*	SSPAA6060-13	KM839794
Araneae	Lycosidae	*Piratula insularis*	SSPAA6062-13	KM836565
Araneae	Lycosidae	*Piratula insularis*	SSPAA6065-13	KM827060
Araneae	Lycosidae	*Piratula insularis*	SSPAA6070-13	KM832163
Araneae	Lycosidae	*Piratula insularis*	SSPAA6078-13	KM838003
Araneae	Lycosidae	*Piratula insularis*	SSPAA7525-13	KM827899
Araneae	Lycosidae	*Piratula insularis*	SSPAA7576-13	KM832279
Araneae	Lycosidae	*Piratula insularis*	SSPAA7582-13	KM823972
Araneae	Lycosidae	*Piratula insularis*	SSPAA8738-13	KM829516

Araneae	Lycosidae	*Piratula insularis*	SSPAA8744-13	KM828343
Araneae	Lycosidae	*Piratula insularis*	SSPAA8745-13	KM830439
Araneae	Lycosidae	*Piratula insularis*	SSPAA8746-13	KM829481
Araneae	Lycosidae	*Piratula insularis*	SSPAA8766-13	KM826322
Araneae	Lycosidae	*Piratula minuta*	ARONT028-09	GU682788
Araneae	Lycosidae	*Piratula minuta*	ERSPI268-09	GU682557
Araneae	Lycosidae	*Piratula minuta*	ERSPI269-09	GU682549
Araneae	Lycosidae	*Piratula minuta*	ERSPI340-09	GU682501
Araneae	Lycosidae	*Piratula minuta*	ERSPI410-09	GU682442
Araneae	Lycosidae	*Piratula minuta*	RBINA2385-13	KP652836
Araneae	Lycosidae	*Piratula minuta*	RBINA3901-13	KP655364
Araneae	Lycosidae	*Piratula minuta*	RBINA4696-13	KP647106
Araneae	Lycosidae	*Piratula minuta*	RBINA4699-13	KP654078
Araneae	Lycosidae	*Piratula minuta*	RBINA5796-13	KP649412
Araneae	Lycosidae	*Piratula minuta*	SMTPB10638-13	KP648985
Araneae	Lycosidae	*Piratula minuta*	SMTPB11090-13	KP650994
Araneae	Lycosidae	*Piratula minuta*	SMTPB11092-13	KP652140
Araneae	Lycosidae	*Piratula minuta*	SMTPB11127-13	KP652005
Araneae	Lycosidae	*Piratula minuta*	SMTPB11128-13	KP648010
Araneae	Lycosidae	*Piratula minuta*	SMTPB11129-13	KP647074
Araneae	Lycosidae	*Piratula minuta*	SMTPB11131-13	KP652191
Araneae	Lycosidae	*Piratula minuta*	SMTPB11133-13	KP648890
Araneae	Lycosidae	*Piratula minuta*	SMTPB11134-13	KP656051
Araneae	Lycosidae	*Piratula minuta*	SMTPB11135-13	KP655995
Araneae	Lycosidae	*Piratula minuta*	SMTPB12082-13	KP657281
Araneae	Lycosidae	*Piratula minuta*	SMTPB13980-13	KP652519
Araneae	Lycosidae	*Piratula minuta*	SMTPB14440-13	KP657449
Araneae	Lycosidae	*Piratula minuta*	SMTPB14652-13	KP655242
Araneae	Lycosidae	*Piratula minuta*	SMTPB14702-13	KP647778
Araneae	Lycosidae	*Piratula minuta*	SMTPB15242-13	KP650692
Araneae	Lycosidae	*Piratula minuta*	SMTPB15675-13	KP646459
Araneae	Lycosidae	*Piratula minuta*	SMTPB18887-13	KP649712
Araneae	Lycosidae	*Piratula minuta*	SMTPB18888-13	KP645727
Araneae	Lycosidae	*Piratula minuta*	SMTPB1984-13	KP650670
Araneae	Lycosidae	*Piratula minuta*	SMTPB20452-13	KP654910
Araneae	Lycosidae	*Piratula minuta*	SMTPB20453-13	KP654782
Araneae	Lycosidae	*Piratula minuta*	SMTPB20562-13	KP647231
Araneae	Lycosidae	*Piratula minuta*	SMTPB20563-13	KP657452
Araneae	Lycosidae	*Piratula minuta*	SMTPB20564-13	KP654538
Araneae	Lycosidae	*Piratula minuta*	SMTPB20566-13	KP654277
Araneae	Lycosidae	*Piratula minuta*	SMTPB20567-13	KP654273
Araneae	Lycosidae	*Piratula minuta*	SMTPB20568-13	KP649291
Araneae	Lycosidae	*Piratula minuta*	SMTPB20569-13	KP651844
Araneae	Lycosidae	*Piratula minuta*	SMTPB20570-13	KP655562
Araneae	Lycosidae	*Piratula minuta*	SMTPB20571-13	KP652261
Araneae	Lycosidae	*Piratula minuta*	SMTPB20572-13	KP649413
Araneae	Lycosidae	*Piratula minuta*	SMTPB20574-13	KP645759
Araneae	Lycosidae	*Piratula minuta*	SMTPB20575-13	KP655709
Araneae	Lycosidae	*Piratula minuta*	SMTPB20576-13	KP650288
Araneae	Lycosidae	*Piratula minuta*	SMTPB20578-13	KP646611
Araneae	Lycosidae	*Piratula minuta*	SMTPB20579-13	KP648037
Araneae	Lycosidae	*Piratula minuta*	SMTPB20580-13	KP646002
Araneae	Lycosidae	*Piratula minuta*	SMTPB20581-13	KP648761

Araneae	Lycosidae	*Piratula minuta*	SMTPB20924-13	KP653358
Araneae	Lycosidae	*Piratula minuta*	SMTPB21296-13	KP652590
Araneae	Lycosidae	*Piratula minuta*	SMTPB2319-13	KP654137
Araneae	Lycosidae	*Piratula minuta*	SMTPB2322-13	KP652567
Araneae	Lycosidae	*Piratula minuta*	SMTPB2323-13	KP649337
Araneae	Lycosidae	*Piratula minuta*	SMTPB2324-13	KP645890
Araneae	Lycosidae	*Piratula minuta*	SMTPB2325-13	KP646411
Araneae	Lycosidae	*Piratula minuta*	SMTPB2326-13	KP657220
Araneae	Lycosidae	*Piratula minuta*	SMTPB2327-13	KP656293
Araneae	Lycosidae	*Piratula minuta*	SMTPB2330-13	KP656070
Araneae	Lycosidae	*Piratula minuta*	SMTPB2331-13	KP646598
Araneae	Lycosidae	*Piratula minuta*	SMTPB2333-13	KP657012
Araneae	Lycosidae	*Piratula minuta*	SMTPB3382-13	KP654959
Araneae	Lycosidae	*Piratula minuta*	SMTPB3690-13	KP655133
Araneae	Lycosidae	*Piratula minuta*	SMTPB6318-13	KP652162
Araneae	Lycosidae	*Piratula minuta*	SMTPB8347-13	KP653864
Araneae	Lycosidae	*Piratula minuta*	SMTPB8348-13	KP646415
Araneae	Lycosidae	*Piratula minuta*	SMTPB8349-13	KP646909
Araneae	Lycosidae	*Piratula minuta*	SMTPB8350-13	KP654038
Araneae	Lycosidae	*Piratula minuta*	SMTPB8352-13	KP653264
Araneae	Lycosidae	*Piratula minuta*	SMTPB8353-13	KP655407
Araneae	Lycosidae	*Piratula minuta*	SMTPB8356-13	KP655990
Araneae	Lycosidae	*Piratula minuta*	SMTPB8357-13	KP655122
Araneae	Lycosidae	*Piratula minuta*	SMTPB8358-13	KP652949
Araneae	Lycosidae	*Piratula minuta*	SMTPB8359-13	KP653366
Araneae	Lycosidae	*Piratula minuta*	SMTPB880-13	KP654389
Araneae	Lycosidae	*Rabidosa punctulata*	GBCH2897-08	DQ151817
Araneae	Lycosidae	*Rabidosa rabida*	GBCH5180-10	EU271654
Araneae	Lycosidae	*Schizocosa ocreata*	GBCH2729-08	EF584470
Araneae	Lycosidae	*Schizocosa ocreata*	GBCH2730-08	EF584469
Araneae	Lycosidae	*Schizocosa ocreata*	GBCH2731-08	EF584468
Araneae	Lycosidae	*Schizocosa ocreata*	GBCH2732-08	EF584467
Araneae	Lycosidae	*Schizocosa ocreata*	GBCH2733-08	EF584466
Araneae	Lycosidae	*Schizocosa ocreata*	GBCH2734-08	EF584465
Araneae	Lycosidae	*Schizocosa ocreata*	GBCH2735-08	EF584464
Araneae	Lycosidae	*Schizocosa ocreata*	GBCH2740-08	EF112507
Araneae	Lycosidae	*Schizocosa ocreata*	GBCH2741-08	EF112505
Araneae	Lycosidae	*Schizocosa ocreata*	GBCH2742-08	EF112504
Araneae	Lycosidae	*Schizocosa ocreata*	GBCH2743-08	EF112494
Araneae	Lycosidae	*Schizocosa ocreata*	GBCH2744-08	EF112491
Araneae	Lycosidae	*Schizocosa ocreata*	GBCH2745-08	EF112503
Araneae	Lycosidae	*Schizocosa ocreata*	GBCH2746-08	EF112508
Araneae	Lycosidae	*Schizocosa ocreata*	GBCH2751-08	EF112495
Araneae	Lycosidae	*Schizocosa ocreata*	GBCH2752-08	EF112493
Araneae	Lycosidae	*Schizocosa ocreata*	GBCH2753-08	EF112492
Araneae	Lycosidae	*Schizocosa ocreata*	GBCH2761-08	EF112510
Araneae	Lycosidae	*Schizocosa ocreata*	GBCH2762-08	EF112509
Araneae	Lycosidae	*Schizocosa ocreata*	GBCH2763-08	EF112506
Araneae	Lycosidae	*Schizocosa ocreata*	GBCH2764-08	EF112502
Araneae	Lycosidae	*Schizocosa ocreata*	GBCH2765-08	EF112501
Araneae	Lycosidae	*Schizocosa ocreata*	GBCH2766-08	EF112500
Araneae	Lycosidae	*Schizocosa ocreata*	GBCH2767-08	EF112499
Araneae	Lycosidae	*Schizocosa ocreata*	GBCH2768-08	EF112498

Araneae	Lycosidae	*Schizocosa ocreata*	GBCH2769-08	EF112497
Araneae	Lycosidae	*Schizocosa ocreata*	GBCH2770-08	EF112496
Araneae	Lycosidae	*Schizocosa ocreata*	RBCH082-04	DQ127450
Araneae	Lycosidae	*Schizocosa ocreata*	RBCH086-04	DQ127438
Araneae	Lycosidae	*Schizocosa ocreata*	RBCH109-04	DQ127413
Araneae	Lycosidae	*Schizocosa saltatrix*	ARONT461-10	HM880614
Araneae	Lycosidae	*Schizocosa stridulans*	GBCH2747-08	EF112520
Araneae	Lycosidae	*Schizocosa stridulans*	GBCH2748-08	EF112519
Araneae	Lycosidae	*Schizocosa stridulans*	GBCH2749-08	EF112518
Araneae	Lycosidae	*Schizocosa stridulans*	GBCH2750-08	EF112521
Araneae	Lycosidae	*Schizocosa stridulans*	GBCH2755-08	EF112517
Araneae	Lycosidae	*Sosippus placidus*	GBCH2891-08	DQ151823
Araneae	Lycosidae	*sp. lSAWT68*	GBCH2894-08	DQ151820
Araneae	Lycosidae	*sp. LSAWT89*	GBCH2893-08	DQ151821
Araneae	Lycosidae	*Tigrosa helluo*	RBCH118-04	DQ127406
Araneae	Lycosidae	*Tigrosa helluo*	RBCH119-04	DQ127407
Araneae	Lycosidae	*Tigrosa helluo*	RBINA3332-13	KP656122
Araneae	Lycosidae	*Tigrosa helluo*	RBINA3336-13	KP646865
Araneae	Lycosidae	*Tigrosa helluo*	RBINA3399-13	KP649136
Araneae	Lycosidae	*Trebacosa marxi*	ARONT795-10	HQ924641
Araneae	Lycosidae	*Trochosa ruricola*	ARONT027-09	GU682789
Araneae	Lycosidae	*Trochosa ruricola*	ARONT063-09	GU682786
Araneae	Lycosidae	*Trochosa ruricola*	ARONT064-09	GU682787
Araneae	Lycosidae	*Trochosa ruricola*	ARONT089-09	GU682785
Araneae	Lycosidae	*Trochosa ruricola*	ARONT193-09	GU682711
Araneae	Lycosidae	*Trochosa ruricola*	ARONT315-09	GU682883
Araneae	Lycosidae	*Trochosa ruricola*	ARONT319-09	GU682880
Araneae	Lycosidae	*Trochosa ruricola*	ARONT636-10	HQ924501
Araneae	Lycosidae	*Trochosa ruricola*	ARONT652-10	HQ924517
Araneae	Lycosidae	*Trochosa ruricola*	ARONT694-10	HQ924552
Araneae	Lycosidae	*Trochosa ruricola*	ARONT695-10	HQ924553
Araneae	Lycosidae	*Trochosa ruricola*	ARONT696-10	HQ924554
Araneae	Lycosidae	*Trochosa ruricola*	ARONT697-10	HQ924555
Araneae	Lycosidae	*Trochosa ruricola*	ARONT733-10	HQ924587
Araneae	Lycosidae	*Trochosa ruricola*	ARONT748-10	HQ924600
Araneae	Lycosidae	*Trochosa ruricola*	RBINA2979-13	KP649276
Araneae	Lycosidae	*Trochosa ruricola*	RBINA3277-13	KP648587
Araneae	Lycosidae	*Trochosa ruricola*	RBINA3282-13	KP655801
Araneae	Lycosidae	*Trochosa ruricola*	RBINA3283-13	KP653880
Araneae	Lycosidae	*Trochosa ruricola*	SMTPB15357-13	KP654080
Araneae	Lycosidae	*Trochosa ruricola*	SMTPB15358-13	KP645859
Araneae	Lycosidae	*Trochosa ruricola*	SMTPB17691-13	KP646849
Araneae	Lycosidae	*Trochosa terricola*	ARONT464-10	HM880616
Araneae	Lycosidae	*Trochosa terricola*	ARONT471-10	HM880622
Araneae	Lycosidae	*Trochosa terricola*	ARONT472-10	HM880623
Araneae	Lycosidae	*Trochosa terricola*	ARONT473-10	HM880624
Araneae	Lycosidae	*Trochosa terricola*	ARONT474-10	HM880625
Araneae	Lycosidae	*Trochosa terricola*	ARONT475-10	HM880626
Araneae	Lycosidae	*Trochosa terricola*	ARONT476-10	HM880627
Araneae	Lycosidae	*Trochosa terricola*	ARONT756-10	HQ924608
Araneae	Lycosidae	*Trochosa terricola*	ERSPI348-09	GU682499
Araneae	Lycosidae	*Trochosa terricola*	ERSPI355-09	GU682484
Araneae	Lycosidae	*Trochosa terricola*	ERSPI358-09	GU682486

Araneae	Lycosidae	*Trochosa terricola*	ERSPI361-09	GU682488
Araneae	Lycosidae	*Trochosa terricola*	ERSPI369-09	GU682472
Araneae	Lycosidae	*Trochosa terricola*	ERSPI371-09	GU682474
Araneae	Lycosidae	*Trochosa terricola*	RBINA1240-13	KP655833
Araneae	Lycosidae	*Trochosa terricola*	RBINA3388-13	KP650914
Araneae	Lycosidae	*Trochosa terricola*	SPIAL081-10	HQ580583
Araneae	Lycosidae	*Trochosa terricola*	SPRMA100-10	HQ580741
Araneae	Lycosidae	*Trochosa terricola*	SPRMA206-10	HQ580820
Araneae	Lycosidae	*Trochosa terricola*	SPRMA209-10	HQ580823
Araneae	Lycosidae	*Trochosa terricola*	SPRMA304-10	HQ977173
Araneae	Lycosidae	*Trochosa terricola*	SPRMA313-10	HQ977179
Araneae	Lycosidae	*Varacosa avara*	ARONT519-10	HQ924408
Araneae	Lycosidae	*Varacosa avara*	ERSPI001-08	KP656899
Araneae	Lycosidae	*Varacosa avara*	ERSPI002-08	KP646829
Araneae	Lycosidae	*Varacosa avara*	ERSPI003-08	KP657309
Araneae	Lycosidae	*Varacosa avara*	ERSPI004-08	KP646843
Araneae	Lycosidae	*Varacosa avara*	ERSPI005-08	KP655420
Araneae	Lycosidae	*Varacosa avara*	ERSPI011-08	KP655070
Araneae	Lycosidae	*Varacosa avara*	ERSPI012-08	KP656575
Araneae	Lycosidae	*Varacosa avara*	ERSPI018-08	KP646893
Araneae	Lycosidae	*Varacosa avara*	ERSPI023-08	KP654489
Araneae	Lycosidae	*Varacosa avara*	ERSPI026-08	KP657417
Araneae	Lycosidae	*Varacosa avara*	ERSPI028-08	KP654907
Araneae	Lycosidae	*Varacosa avara*	ERSPI029-08	KP649084
Araneae	Lycosidae	*Varacosa avara*	ERSPI030-08	KP645692
Araneae	Lycosidae	*Varacosa avara*	ERSPI031-08	KP647034
Araneae	Lycosidae	*Varacosa avara*	ERSPI032-08	KP652691
Araneae	Lycosidae	*Varacosa avara*	ERSPI033-08	KP652554
Araneae	Lycosidae	*Varacosa avara*	ERSPI034-08	KP645925
Araneae	Lycosidae	*Varacosa avara*	ERSPI035-08	KP654218
Araneae	Lycosidae	*Varacosa avara*	ERSPI042-08	KP654425
Araneae	Lycosidae	*Varacosa avara*	ERSPI046-08	KP648192
Araneae	Lycosidae	*Varacosa avara*	ERSPI047-08	KP656753
Araneae	Lycosidae	*Varacosa avara*	ERSPI053-08	KP654535
Araneae	Lycosidae	*Varacosa avara*	ERSPI056-08	KP652379
Araneae	Lycosidae	*Varacosa avara*	ERSPI057-08	KP646184
Araneae	Lycosidae	*Varacosa avara*	ERSPI060-08	KP652895
Araneae	Lycosidae	*Varacosa avara*	ERSPI063-08	KP653160
Araneae	Lycosidae	*Varacosa avara*	ERSPI065-08	KP654643
Araneae	Lycosidae	*Varacosa avara*	ERSPI070-08	KP647469
Araneae	Lycosidae	*Varacosa avara*	ERSPI071-08	KP651969
Araneae	Lycosidae	*Varacosa avara*	ERSPI074-08	KP649409
Araneae	Lycosidae	*Varacosa avara*	ERSPI077-08	KP648393
Araneae	Lycosidae	*Varacosa avara*	ERSPI082-08	KP657431
Araneae	Lycosidae	*Varacosa avara*	ERSPI092-08	KP650672
Araneae	Lycosidae	*Varacosa avara*	ERSPI093-08	KP656854
Araneae	Lycosidae	*Varacosa avara*	ERSPI094-08	KP645514
Araneae	Lycosidae	*Varacosa avara*	ERSPI097-08	KP650480
Araneae	Lycosidae	*Varacosa avara*	ERSPI101-08	KP646529
Araneae	Lycosidae	*Varacosa avara*	ERSPI104-09	KP647620
Araneae	Lycosidae	*Varacosa avara*	ERSPI105-09	KP652866
Araneae	Lycosidae	*Varacosa avara*	ERSPI106-09	KP646058
Araneae	Lycosidae	*Varacosa avara*	ERSPI107-09	KP652455

Araneae	Lycosidae	*Varacosa avara*	ERSPI108-09	KP656144
Araneae	Lycosidae	*Varacosa avara*	ERSPI111-09	KP649393
Araneae	Lycosidae	*Varacosa avara*	ERSPI118-09	KP652257
Araneae	Lycosidae	*Varacosa avara*	ERSPI119-09	KP656366
Araneae	Lycosidae	*Varacosa avara*	ERSPI120-09	KP649403
Araneae	Lycosidae	*Varacosa avara*	ERSPI121-09	KP653794
Araneae	Lycosidae	*Varacosa avara*	ERSPI122-09	KP649344
Araneae	Lycosidae	*Varacosa avara*	ERSPI125-09	KP656669
Araneae	Lycosidae	*Varacosa avara*	ERSPI132-09	KP645896
Araneae	Lycosidae	*Varacosa avara*	ERSPI136-09	KP651326
Araneae	Lycosidae	*Varacosa avara*	ERSPI140-09	KP646644
Araneae	Lycosidae	*Varacosa avara*	ERSPI141-09	KP649877
Araneae	Lycosidae	*Varacosa avara*	ERSPI142-09	KP652784
Araneae	Lycosidae	*Varacosa avara*	ERSPI143-09	KP653006
Araneae	Lycosidae	*Varacosa avara*	ERSPI152-09	KP646655
Araneae	Lycosidae	*Varacosa avara*	ERSPI163-09	KP649693
Araneae	Lycosidae	*Varacosa avara*	ERSPI187-09	KP651387
Araneae	Lycosidae	*Varacosa avara*	ERSPI188-09	KP649841
Araneae	Lycosidae	*Varacosa avara*	ERSPI189-09	KP651669
Araneae	Lycosidae	*Varacosa avara*	ERSPI195-09	KP645569
Araneae	Lycosidae	*Varacosa avara*	ERSPI196-09	KP649976
Araneae	Lycosidae	*Varacosa avara*	ERSPI197-09	KP651263
Araneae	Lycosidae	*Varacosa avara*	ERSPI201-09	KP651100
Araneae	Lycosidae	*Varacosa avara*	ERSPI204-09	KP653596
Araneae	Lycosidae	*Varacosa avara*	ERSPI205-09	KP651588
Araneae	Lycosidae	*Varacosa avara*	ERSPI208-09	KP649075
Araneae	Lycosidae	*Varacosa avara*	ERSPI209-09	KP654556
Araneae	Lycosidae	*Varacosa avara*	ERSPI210-09	KP647503
Araneae	Lycosidae	*Varacosa avara*	ERSPI211-09	KP646355
Araneae	Lycosidae	*Varacosa avara*	ERSPI212-09	KP654095
Araneae	Lycosidae	*Varacosa avara*	ERSPI217-09	KP653976
Araneae	Lycosidae	*Varacosa avara*	ERSPI218-09	KP650700
Araneae	Lycosidae	*Varacosa avara*	ERSPI227-09	KP646018
Araneae	Lycosidae	*Varacosa avara*	ERSPI228-09	KP656110
Araneae	Lycosidae	*Varacosa avara*	ERSPI229-09	KP652312
Araneae	Lycosidae	*Varacosa avara*	ERSPI230-09	KP649942
Araneae	Lycosidae	*Varacosa avara*	ERSPI234-09	KP646883
Araneae	Lycosidae	*Varacosa avara*	ERSPI236-09	KP653631
Araneae	Lycosidae	*Varacosa avara*	ERSPI237-09	KP651406
Araneae	Lycosidae	*Varacosa avara*	ERSPI239-09	KP646721
Araneae	Lycosidae	*Varacosa avara*	ERSPI241-09	KP653691
Araneae	Lycosidae	*Venator spenceri*	GBCH7637-13	JQ240194
Araneae	Lycosidae	*Venatrix pseudospeciosa*	GBCH1330-06	DQ295867
Araneae	Lycosidae	*Venatrix pseudospeciosa*	GBCH7636-13	JQ240195
Araneae	Lycosidae		CNBAN018-13	KP979291
Araneae	Mecicobothriidae	*Hexura picea*	SPRMA286-10	HQ977161
Araneae	Mecysmaucheniidae	*Aotearoa magna*	GBCH11419-13	JX240238
Araneae	Mecysmaucheniidae	*Chilarchaea quellon*	GBCH11416-13	JX240241
Araneae	Mecysmaucheniidae	*Mecysmauchenius segmentatus*	GBCH11417-13	JX240240
Araneae	Mecysmaucheniidae	*Mesarchaea bellavista*	GBCH11418-13	JX240239
Araneae	Mecysmaucheniidae	*Zearchaea*	GBCH11415-13	JX240242
Araneae	Migidae	*Moggridgea australis*	GBCH5437-11	JF749924
Araneae	Migidae	*Moggridgea intermedia*	GBCH5433-11	JF749928

Araneae	Migidae	*Moggridgea mordax*	GBCH5432-11	JF749929
Araneae	Migidae	*Moggridgea peringueyi*	GBCH5434-11	JF749927
Araneae	Migidae	*Moggridgea rupicoloides*	GBCH5436-11	JF749925
Araneae	Migidae	*Moggridgea terrestris*	GBCH5435-11	JF749926
Araneae	Migidae	*Moggridgea tingle*	GBCH5438-11	JF749923
Araneae	Migidae	*Moggridgea tingle*	GBCH5439-11	JF749922
Araneae	Migidae	*Moggridgea tingle*	GBCH5440-11	JF749921
Araneae	Migidae	*Moggridgea tingle*	GBCH5441-11	JF749920
Araneae	Migidae	*Moggridgea tingle*	GBCH5442-11	JF749919
Araneae	Migidae	*Moggridgea tingle*	GBCH5443-11	JF749918
Araneae	Migidae	*Moggridgea tingle*	GBCH5444-11	JF749917
Araneae	Migidae	*Moggridgea tingle*	GBCH5445-11	JF749916
Araneae	Migidae	*Moggridgea tingle*	GBCH5446-11	JF749915
Araneae	Migidae	*Moggridgea tingle*	GBCH5447-11	JF749914
Araneae	Migidae	*Moggridgea tingle*	GBCH5448-11	JF749913
Araneae	Migidae	*Moggridgea tingle*	GBCH5449-11	JF749912
Araneae	Migidae	*Moggridgea tingle*	GBCH5450-11	JF749911
Araneae	Migidae	*Moggridgea tingle*	GBCH5451-11	JF749910
Araneae	Migidae	*Moggridgea tingle*	GBCH5452-11	JF749909
Araneae	Migidae	*Moggridgea tingle*	GBCH5453-11	JF749908
Araneae	Migidae	*Moggridgea tingle*	GBCH5454-11	JF749907
Araneae	Migidae	*Moggridgea tingle*	GBCH5455-11	JF749906
Araneae	Migidae	*Moggridgea tingle*	GBCH5456-11	JF749905
Araneae	Migidae	*Moggridgea tingle*	GBCH5457-11	JF749904
Araneae	Migidae	*Moggridgea tingle*	GBCH5458-11	JF749903
Araneae	Migidae	*Moggridgea tingle*	GBCH5459-11	JF749902
Araneae	Migidae	*Moggridgea tingle*	GBCH5460-11	JF749901
Araneae	Migidae	*Moggridgea tingle*	GBCH5461-11	JF749900
Araneae	Migidae	*Moggridgea tingle*	GBCH5462-11	JF749899
Araneae	Migidae	*Moggridgea tingle*	GBCH5463-11	JF749898
Araneae	Migidae	*Moggridgea tingle*	GBCH5464-11	JF749897
Araneae	Migidae	*Moggridgea tingle*	GBCH5465-11	JF749896
Araneae	Migidae	*Moggridgea tingle*	GBCH5466-11	JF749895
Araneae	Migidae	*Moggridgea tingle*	GBCH5467-11	JF749894
Araneae	Migidae	*Moggridgea tingle*	GBCH5468-11	JF749893
Araneae	Migidae	*Moggridgea tingle*	GBCH5469-11	JF749892
Araneae	Migidae	*Moggridgea tingle*	GBCH5470-11	JF749891
Araneae	Migidae	*Moggridgea tingle*	GBCH5471-11	JF749890
Araneae	Migidae	*Moggridgea tingle*	GBCH5472-11	JF749889
Araneae	Migidae	*Moggridgea tingle*	GBCH5473-11	JF749888
Araneae	Migidae	*Moggridgea tingle*	GBCH5474-11	JF749887
Araneae	Migidae	*Moggridgea tingle*	GBCH5475-11	JF749886
Araneae	Migidae	*Moggridgea tingle*	GBCH5476-11	JF749885
Araneae	Migidae	*Moggridgea tingle*	GBCH5477-11	JF749884
Araneae	Migidae	*Moggridgea tingle*	GBCH5478-11	JF749883
Araneae	Migidae	*Moggridgea tingle*	GBCH5479-11	JF749882
Araneae	Migidae	*Moggridgea tingle*	GBCH5480-11	JF749881
Araneae	Migidae	*Moggridgea tingle*	GBCH5481-11	JF749880
Araneae	Migidae	*Moggridgea tingle*	GBCH5482-11	JF749879
Araneae	Migidae	*Moggridgea tingle*	GBCH5483-11	JF749878
Araneae	Mimetidae	*Ero japonica*	GBCH11193-13	JN817077
Araneae	Mimetidae	*Mimetus haynesi*	JSARA005-11	KP657148
Araneae	Mimetidae	*Mimetus hesperus*	SPRMA731-10	JF887153

Araneae	Mimetidae	*Mimetus hesperus*	SPRMA732-10	JF887154
Araneae	Mimetidae	*Mimetus hesperus*	SPRMA739-10	JF887157
Araneae	Mimetidae	*Mimetus notius*	ARONT271-09	GU682727
Araneae	Mimetidae	*Mimetus notius*	ARONT272-09	GU682725
Araneae	Mimetidae	*Mimetus notius*	ARONT419-09	GU682941
Araneae	Mimetidae	*Mimetus notius*	CNSLI110-12	KM829988
Araneae	Mimetidae	*Mimetus notius*	CNSLM007-13	KM834375
Araneae	Mimetidae	*Mimetus notius*	CNSLT003-13	KM834807
Araneae	Mimetidae	*Mimetus notius*	JSARA096-11	KP655000
Araneae	Mimetidae	*Mimetus notius*	RBINA3329-13	KP655761
Araneae	Mimetidae	*Mimetus notius*	RBINA3541-13	KP653417
Araneae	Mimetidae	*Mimetus notius*	RBINA416-13	KP645860
Araneae	Mimetidae	*Mimetus notius*	SMTPB21264-13	KP650802
Araneae	Mimetidae	*Mimetus sp. TAB-2009*	GBCH4025-09	FJ607574
Araneae	Miturgidae	*Zora hespera*	SPRMA814-12	KP648913
Araneae	Mysmenidae	*Maymena ambita*	GBCH8370-13	GU456876
Araneae	Mysmenidae	*Microdipoena*	GBCH8352-13	GU456895
Araneae	Mysmenidae	*Microdipoena nyungwe*	GBCH8368-13	GU456878
Araneae	Mysmenidae	*Mysmena*	GBCH8341-13	GU456906
Araneae	Mysmenidae	*Mysmena*	GBCH8342-13	GU456905
Araneae	Mysmenidae	*Mysmena*	GBCH8343-13	GU456904
Araneae	Mysmenidae	*Mysmena*	GBCH8344-13	GU456903
Araneae	Mysmenidae	*Mysmena*	GBCH8345-13	GU456902
Araneae	Mysmenidae	*Mysmena*	GBCH8346-13	GU456901
Araneae	Mysmenidae	*Mysmena*	GBCH8347-13	GU456900
Araneae	Mysmenidae	*Mysmena*	GBCH8348-13	GU456899
Araneae	Mysmenidae	*Mysmena*	GBCH8359-13	GU456887
Araneae	Mysmenidae	*Mysmena*	GBCH8360-13	GU456886
Araneae	Mysmenidae	*Mysmena*	GBCH8361-13	GU456885
Araneae	Mysmenidae	*Trogloneta*	GBCH8356-13	GU456891
Araneae	Mysmenidae		GBCH8349-13	GU456898
Araneae	Mysmenidae		GBCH8350-13	GU456897
Araneae	Mysmenidae		GBCH8351-13	GU456896
Araneae	Mysmenidae		GBCH8353-13	GU456894
Araneae	Mysmenidae		GBCH8355-13	GU456892
Araneae	Mysmenidae		GBCH8364-13	GU456882
Araneae	Nemesiidae	*Aname*	GBCH11836-13	JQ772155
Araneae	Nemesiidae	*Aname*	GBCH11837-13	JQ772154
Araneae	Nemesiidae	*Aname*	GBCH11838-13	JQ772153
Araneae	Nemesiidae	*Aname*	GBCH11839-13	JQ772152
Araneae	Nemesiidae	*Aname*	GBCH11840-13	JQ772151
Araneae	Nemesiidae	*Aname*	GBCH11841-13	JQ772150
Araneae	Nemesiidae	*Aname*	GBCH11842-13	JQ772149
Araneae	Nemesiidae	*Aname*	GBCH11843-13	JQ772148
Araneae	Nemesiidae	*Aname*	GBCH11844-13	JQ772147
Araneae	Nemesiidae	*Aname*	GBCH11845-13	JQ772146
Araneae	Nemesiidae	*Aname*	GBCH11846-13	JQ772145
Araneae	Nemesiidae	*Aname*	GBCH11847-13	JQ772144
Araneae	Nemesiidae	*Aname*	GBCH11848-13	JQ772143
Araneae	Nemesiidae	*Aname*	GBCH11849-13	JQ772142
Araneae	Nemesiidae	*Aname*	GBCH11850-13	JQ772141
Araneae	Nemesiidae	*Aname*	GBCH11851-13	JQ772140
Araneae	Nemesiidae	*Aname*	GBCH11852-13	JQ772139

Araneae	Nemesiidae	*Aname*	GBCH11853-13	JQ772138
Araneae	Nemesiidae	*Aname*	GBCH11854-13	JQ772137
Araneae	Nemesiidae	*Aname*	GBCH11855-13	JQ772136
Araneae	Nemesiidae	*Aname*	GBCH11856-13	JQ772135
Araneae	Nemesiidae	*Aname*	GBCH11857-13	JQ772134
Araneae	Nemesiidae	*Aname*	GBCH11858-13	JQ772133
Araneae	Nemesiidae	*Aname*	GBCH11859-13	JQ772132
Araneae	Nemesiidae	*Aname*	GBCH11860-13	JQ772131
Araneae	Nemesiidae	*Aname*	GBCH11861-13	JQ772130
Araneae	Nemesiidae	*Aname*	GBCH11862-13	JQ772129
Araneae	Nemesiidae	*Aname*	GBCH11863-13	JQ772128
Araneae	Nemesiidae	*Aname*	GBCH11864-13	JQ772127
Araneae	Nemesiidae	*Aname*	GBCH11865-13	JQ772126
Araneae	Nemesiidae	*Aname*	GBCH11866-13	JQ772125
Araneae	Nemesiidae	*Pseudoteyl*	GBCH11867-13	JQ772124
Araneae	Nemesiidae	*Teyl luculentus*	GBCH11868-13	JQ772123
Araneae	Nephilidae	*Clitaetra sp. FAPDNA029*	GBCH3912-09	EU003281
Araneae	Nephilidae	*Herennia multipuncta*	GBCH7179-13	HQ441941
Araneae	Nephilidae	*Herennia sp. FAPDNA031*	GBCH3905-09	EU003288
Araneae	Nephilidae	*Nephila antipodiana*	GBCH7196-13	HQ441924
Araneae	Nephilidae	*Nephila clavata*	GBCH0362-06	AY052586
Araneae	Nephilidae	*Nephila clavata*	GBCH0631-06	AY452691
Araneae	Nephilidae	*Nephila clavata*	GBCH11134-13	JN817136
Araneae	Nephilidae	*Nephila clavata*	GBCH1693-06	NC_008063
Araneae	Nephilidae	*Nephila clavata*	GBCH7191-13	HQ441929
Araneae	Nephilidae	*Nephila clavata*	GBCH7192-13	HQ441928
Araneae	Nephilidae	*Nephila clavata*	GBCH7193-13	HQ441927
Araneae	Nephilidae	*Nephila clavata*	GBCH7740-13	JN032336
Araneae	Nephilidae	*Nephila clavipes*	GBCH3236-09	FJ525328
Araneae	Nephilidae	*Nephila clavipes*	GBCH3892-09	EU003302
Araneae	Nephilidae	*Nephila clavipes*	GBCH7190-13	HQ441930
Araneae	Nephilidae	*Nephila fenestrata*	GBCH7186-13	HQ441934
Araneae	Nephilidae	*Nephila pilipes*	GBCH0364-06	AY052588
Araneae	Nephilidae	*Nephila pilipes*	GBCH0365-06	AY052589
Araneae	Nephilidae	*Nephila pilipes*	GBCH0366-06	AY052590
Araneae	Nephilidae	*Nephila pilipes*	GBCH0367-06	AY052591
Araneae	Nephilidae	*Nephila pilipes*	GBCH0368-06	AY052592
Araneae	Nephilidae	*Nephila pilipes*	GBCH0369-06	AY052593
Araneae	Nephilidae	*Nephila pilipes*	GBCH0370-06	AY052594
Araneae	Nephilidae	*Nephila pilipes*	GBCH0371-06	AY052595
Araneae	Nephilidae	*Nephila pilipes*	GBCH0372-06	AY052596
Araneae	Nephilidae	*Nephila pilipes*	GBCH0373-06	AY052597
Araneae	Nephilidae	*Nephila pilipes*	GBCH0374-06	AY052598
Araneae	Nephilidae	*Nephila pilipes*	GBCH1567-06	DQ779228
Araneae	Nephilidae	*Nephila pilipes*	GBCH1568-06	DQ779229
Araneae	Nephilidae	*Nephila pilipes*	GBCH1569-06	DQ779230
Araneae	Nephilidae	*Nephila pilipes*	GBCH1570-06	DQ779231
Araneae	Nephilidae	*Nephila pilipes*	GBCH1571-06	DQ779232
Araneae	Nephilidae	*Nephila pilipes*	GBCH1572-06	DQ779233
Araneae	Nephilidae	*Nephila pilipes*	GBCH1573-06	DQ779234
Araneae	Nephilidae	*Nephila pilipes*	GBCH1574-06	DQ779235
Araneae	Nephilidae	*Nephila pilipes*	GBCH1575-06	DQ779236
Araneae	Nephilidae	*Nephila pilipes*	GBCH1576-06	DQ779237

Araneae	Nephilidae	*Nephila pilipes*	GBCH1577-06	DQ779238
Araneae	Nephilidae	*Nephila pilipes*	GBCH1578-06	DQ779239
Araneae	Nephilidae	*Nephila pilipes*	GBCH1579-06	DQ779240
Araneae	Nephilidae	*Nephila pilipes*	GBCH1580-06	DQ779241
Araneae	Nephilidae	*Nephila pilipes*	GBCH1581-06	DQ779242
Araneae	Nephilidae	*Nephila pilipes*	GBCH1582-06	DQ779243
Araneae	Nephilidae	*Nephila pilipes*	GBCH1583-06	DQ779244
Araneae	Nephilidae	*Nephila pilipes*	GBCH1584-06	DQ779245
Araneae	Nephilidae	*Nephila pilipes*	GBCH1585-06	DQ779246
Araneae	Nephilidae	*Nephila pilipes*	GBCH1586-06	DQ779247
Araneae	Nephilidae	*Nephila pilipes*	GBCH1587-06	DQ779248
Araneae	Nephilidae	*Nephila pilipes*	GBCH1588-06	DQ779249
Araneae	Nephilidae	*Nephila pilipes*	GBCH1589-06	DQ779250
Araneae	Nephilidae	*Nephila pilipes*	GBCH1590-06	DQ779251
Araneae	Nephilidae	*Nephila pilipes*	GBCH1591-06	DQ779252
Araneae	Nephilidae	*Nephila pilipes*	GBCH1592-06	DQ779253
Araneae	Nephilidae	*Nephila pilipes*	GBCH1593-06	DQ779254
Araneae	Nephilidae	*Nephila pilipes*	GBCH1594-06	DQ779255
Araneae	Nephilidae	*Nephila pilipes*	GBCH1595-06	DQ779256
Araneae	Nephilidae	*Nephila pilipes*	GBCH1596-06	DQ779257
Araneae	Nephilidae	*Nephila pilipes*	GBCH1597-06	DQ779258
Araneae	Nephilidae	*Nephila pilipes*	GBCH1598-06	DQ779259
Araneae	Nephilidae	*Nephila pilipes*	GBCH1599-06	DQ779260
Araneae	Nephilidae	*Nephila pilipes*	GBCH1600-06	DQ779261
Araneae	Nephilidae	*Nephila pilipes*	GBCH1601-06	DQ779262
Araneae	Nephilidae	*Nephila pilipes*	GBCH1602-06	DQ779263
Araneae	Nephilidae	*Nephila pilipes*	GBCH1603-06	DQ779264
Araneae	Nephilidae	*Nephila pilipes*	GBCH1604-06	DQ779265
Araneae	Nephilidae	*Nephila pilipes*	GBCH1605-06	DQ779266
Araneae	Nephilidae	*Nephila pilipes*	GBCH1606-06	DQ779267
Araneae	Nephilidae	*Nephila pilipes*	GBCH1607-06	DQ779268
Araneae	Nephilidae	*Nephila pilipes*	GBCH1608-06	DQ779269
Araneae	Nephilidae	*Nephila pilipes*	GBCH1609-06	DQ779270
Araneae	Nephilidae	*Nephila pilipes*	GBCH1610-06	DQ779271
Araneae	Nephilidae	*Nephila pilipes*	GBCH1611-06	DQ779272
Araneae	Nephilidae	*Nephila pilipes*	GBCH1612-06	DQ779273
Araneae	Nephilidae	*Nephila pilipes*	GBCH1613-06	DQ779274
Araneae	Nephilidae	*Nephila pilipes*	GBCH1614-06	DQ779275
Araneae	Nephilidae	*Nephila pilipes*	GBCH1615-06	DQ779276
Araneae	Nephilidae	*Nephila pilipes*	GBCH1616-06	DQ779277
Araneae	Nephilidae	*Nephila pilipes*	GBCH1617-06	DQ779278
Araneae	Nephilidae	*Nephila pilipes*	GBCH1618-06	DQ779279
Araneae	Nephilidae	*Nephila pilipes*	GBCH1619-06	DQ779280
Araneae	Nephilidae	*Nephila pilipes*	GBCH1620-06	DQ779281
Araneae	Nephilidae	*Nephila pilipes*	GBCH1621-06	DQ779282
Araneae	Nephilidae	*Nephila pilipes*	GBCH1622-06	DQ779283
Araneae	Nephilidae	*Nephila pilipes*	GBCH5872-13	JF835935
Araneae	Nephilidae	*Nephila pilipes*	GBCH7183-13	HQ441937
Araneae	Nephilidae	*Nephila pilipes*	GBCH7739-13	JN032337
Araneae	Nephilidae	*Nephila plumipes*	GBCH7181-13	HQ441939
Araneae	Nephilidae	*Nephila plumipes*	GBCH7182-13	HQ441938
Araneae	Nephilidae	*Nephilengys malabarensis*	GBCH4024-09	FJ607575
Araneae	Nephilidae	*Nephilengys sp. FAPDNA032*	GBCH3891-09	EU003303

Araneae	Nesticidae	*Nesticella brevipes*	GBCH11184-13	JN817086
Araneae	Nesticidae	*Nesticella quelpartensis*	GBCH11185-13	JN817085
Araneae	Nesticidae	*Nesticus barri*	GBCH4979-10	GQ421688
Araneae	Nesticidae	*Nesticus barri*	GBCH4980-10	GQ421687
Araneae	Nesticidae	*Nesticus barri*	GBCH4981-10	GQ421686
Araneae	Nesticidae	*Nesticus barri*	GBCH4982-10	GQ421685
Araneae	Nesticidae	*Nesticus barri*	GBCH4983-10	GQ421684
Araneae	Nesticidae	*Nesticus barri*	GBCH4984-10	GQ421683
Araneae	Nesticidae	*Nesticus barri*	GBCH4985-10	GQ421682
Araneae	Nesticidae	*Nesticus barri*	GBCH4986-10	GQ421681
Araneae	Nesticidae	*Nesticus barri*	GBCH4987-10	GQ421680
Araneae	Nesticidae	*Nesticus barri*	GBCH4988-10	GQ421679
Araneae	Nesticidae	*Nesticus barri*	GBCH4989-10	GQ421678
Araneae	Nesticidae	*Nesticus barri*	GBCH4990-10	GQ421677
Araneae	Nesticidae	*Nesticus barri*	GBCH4991-10	GQ421676
Araneae	Nesticidae	*Nesticus barri*	GBCH4992-10	GQ421675
Araneae	Nesticidae	*Nesticus barri*	GBCH4993-10	GQ421674
Araneae	Nesticidae	*Nesticus barri*	GBCH4994-10	GQ421673
Araneae	Nesticidae	*Nesticus barri*	GBCH4995-10	GQ421672
Araneae	Nesticidae	*Nesticus barri*	GBCH4996-10	GQ421671
Araneae	Nesticidae	*Nesticus barri*	GBCH4997-10	GQ421670
Araneae	Nesticidae	*Nesticus barri*	GBCH4998-10	GQ421669
Araneae	Nesticidae	*Nesticus barri*	GBCH4999-10	GQ421668
Araneae	Nesticidae	*Nesticus barri*	GBCH5000-10	GQ421667
Araneae	Nesticidae	*Nesticus barri*	GBCH5001-10	GQ421666
Araneae	Nesticidae	*Nesticus barri*	GBCH5002-10	GQ421665
Araneae	Nesticidae	*Nesticus barri*	GBCH5003-10	GQ421664
Araneae	Nesticidae	*Nesticus barri*	GBCH5004-10	GQ421663
Araneae	Nesticidae	*Nesticus barri*	GBCH5005-10	GQ421662
Araneae	Nesticidae	*Nesticus barri*	GBCH5006-10	GQ421661
Araneae	Nesticidae	*Nesticus barri*	GBCH5007-10	GQ421660
Araneae	Nesticidae	*Nesticus barri*	GBCH5008-10	GQ421659
Araneae	Nesticidae	*Nesticus barri*	GBCH5009-10	GQ421658
Araneae	Nesticidae	*Nesticus barri*	GBCH5010-10	GQ421657
Araneae	Nesticidae	*Nesticus barri*	GBCH5011-10	GQ421656
Araneae	Nesticidae	*Nesticus barri*	GBCH5012-10	GQ421655
Araneae	Nesticidae	*Nesticus barri*	GBCH5013-10	GQ421654
Araneae	Nesticidae	*Nesticus barri*	GBCH5014-10	GQ421653
Araneae	Nesticidae	*Nesticus barri*	GBCH5015-10	GQ421652
Araneae	Nesticidae	*Nesticus barri*	GBCH5016-10	GQ421651
Araneae	Nesticidae	*Nesticus barri*	GBCH5017-10	GQ421650
Araneae	Nesticidae	*Nesticus barri*	GBCH5018-10	GQ421649
Araneae	Nesticidae	*Nesticus barri*	GBCH5019-10	GQ421648
Araneae	Nesticidae	*Nesticus barri*	GBCH5020-10	GQ421647
Araneae	Nesticidae	*Nesticus barri*	GBCH5021-10	GQ421646
Araneae	Nesticidae	*Nesticus barri*	GBCH5022-10	GQ421645
Araneae	Nesticidae	*Nesticus barri*	GBCH7057-13	HM245858
Araneae	Nesticidae	*Nesticus barri*	GBCH7058-13	HM245857
Araneae	Nesticidae	*Nesticus cellulanus*	ARONT010-09	GU682834
Araneae	Nesticidae	*Nesticus cellulanus*	ARONT012-09	GU682810
Araneae	Nesticidae	*Nesticus coreanus*	GBCH11187-13	JN817083
Araneae	Nesticidae	*Nesticus kyongkeomsanensis*	GBCH11186-13	JN817084
Araneae	Palpimanidae	*Palpimanus*	GBCH11422-13	JX240235

Araneae	Periegopidae	*Periegops*	GBCH10173-13	JX174290
Araneae	Periegopidae	*Periegops*	GBCH10174-13	JX174289
Araneae	Periegopidae	*Periegops*	GBCH10175-13	JX174288
Araneae	Philodromidae	*Apollophanes margareta*	CNGLF1917-13	KM829654
Araneae	Philodromidae	*Ebo bucklei*	SSWLB017-13	KM830711
Araneae	Philodromidae	*Ebo evansae*	SPRMA359-10	HQ580910
Araneae	Philodromidae	*Ebo evansae*	SPRMA942-12	KP651289
Araneae	Philodromidae	*Ebo iviei*	ARONT492-10	HQ924387
Araneae	Philodromidae	*Philodromus alascensis*	JDTGS052-09	GU679872
Araneae	Philodromidae	*Philodromus alascensis*	KKCHE1071-09	GU683746
Araneae	Philodromidae	*Philodromus aureolus*	GACAC535-12	HE575186
Araneae	Philodromidae	*Philodromus aureolus*	GBCH11037-13	JN817234
Araneae	Philodromidae	*Philodromus cespitum*	ARONT224-09	GU682674
Araneae	Philodromidae	*Philodromus cespitum*	ARONT225-09	GU682671
Araneae	Philodromidae	*Philodromus cespitum*	ARONT243-09	GU682669
Araneae	Philodromidae	*Philodromus cespitum*	ARONT244-09	GU682670
Araneae	Philodromidae	*Philodromus cespitum*	ARONT245-09	GU682672
Araneae	Philodromidae	*Philodromus cespitum*	ARONT399-09	GU682900
Araneae	Philodromidae	*Philodromus cespitum*	ARONT400-09	GU682901
Araneae	Philodromidae	*Philodromus cespitum*	ARONT411-09	GU682898
Araneae	Philodromidae	*Philodromus cespitum*	ARONT418-09	GU682899
Araneae	Philodromidae	*Philodromus cespitum*	ARONT619-10	HQ924484
Araneae	Philodromidae	*Philodromus cespitum*	ARONT620-10	HQ924485
Araneae	Philodromidae	*Philodromus cespitum*	ARONT771-10	HQ924620
Araneae	Philodromidae	*Philodromus cespitum*	CNPPD2692-12	KJ088114
Araneae	Philodromidae	*Philodromus cespitum*	CNPPH1196-12	KJ163871
Araneae	Philodromidae	*Philodromus cespitum*	CNPPI1859-12	KJ444870
Araneae	Philodromidae	*Philodromus cespitum*	CNWBE386-13	KM826440
Araneae	Philodromidae	*Philodromus cespitum*	CNWLE2548-13	KM826703
Araneae	Philodromidae	*Philodromus cespitum*	CNWLE2555-13	KM827410
Araneae	Philodromidae	*Philodromus cespitum*	CNWLE2556-13	KM824559
Araneae	Philodromidae	*Philodromus cespitum*	CNWLE2558-13	KM827741
Araneae	Philodromidae	*Philodromus cespitum*	CNWLE2560-13	KM839897
Araneae	Philodromidae	*Philodromus cespitum*	CNWLF001-12	KM836499
Araneae	Philodromidae	*Philodromus cespitum*	CNWLF004-12	KM833086
Araneae	Philodromidae	*Philodromus cespitum*	CNWLF006-12	KM835638
Araneae	Philodromidae	*Philodromus cespitum*	CNWLF008-12	KM829396
Araneae	Philodromidae	*Philodromus cespitum*	CNWLF182-12	KM837901
Araneae	Philodromidae	*Philodromus cespitum*	CNWLG002-12	KM828494
Araneae	Philodromidae	*Philodromus cespitum*	CNWLG865-12	KM832202
Araneae	Philodromidae	*Philodromus cespitum*	CNWLH004-12	KM827054
Araneae	Philodromidae	*Philodromus cespitum*	CNWLH006-12	KM829324
Araneae	Philodromidae	*Philodromus cespitum*	CNWLI002-12	KM837184
Araneae	Philodromidae	*Philodromus cespitum*	CNWLI003-12	KM829167
Araneae	Philodromidae	*Philodromus cespitum*	CNWLI004-12	KM832176
Araneae	Philodromidae	*Philodromus cespitum*	GACAC533-12	HE575184
Araneae	Philodromidae	*Philodromus cespitum*	GACAC534-12	HE575185
Araneae	Philodromidae	*Philodromus cespitum*	GBCH11036-13	JN817235
Araneae	Philodromidae	*Philodromus cespitum*	JSARA012-11	KP645442
Araneae	Philodromidae	*Philodromus cespitum*	JSARA039-11	KP657193
Araneae	Philodromidae	*Philodromus cespitum*	JSJUL2416-12	KP651604
Araneae	Philodromidae	*Philodromus cespitum*	JSJUL2417-12	KP650751
Araneae	Philodromidae	*Philodromus cespitum*	JSJUN2359-12	KP647003

Araneae	Philodromidae	*Philodromus cespitum*	RBCH007-04	DQ127519
Araneae	Philodromidae	*Philodromus cespitum*	RBCH178-04	DQ127356
Araneae	Philodromidae	*Philodromus cespitum*	RBINA223-13	KP646363
Araneae	Philodromidae	*Philodromus cespitum*	RBINA224-13	KP650764
Araneae	Philodromidae	*Philodromus cespitum*	RBINA233-13	KP650481
Araneae	Philodromidae	*Philodromus cespitum*	RBINA255-13	KP651619
Araneae	Philodromidae	*Philodromus cespitum*	RBINA259-13	KP653247
Araneae	Philodromidae	*Philodromus cespitum*	RBINA4168-13	KP652860
Araneae	Philodromidae	*Philodromus cespitum*	SMTPB14547-13	KP647860
Araneae	Philodromidae	*Philodromus cespitum*	SMTPB15008-13	KP657283
Araneae	Philodromidae	*Philodromus cespitum*	SMTPB17028-13	KP648103
Araneae	Philodromidae	*Philodromus cespitum*	SMTPB18889-13	KP652357
Araneae	Philodromidae	*Philodromus cespitum*	SMTPB5071-13	KP655423
Araneae	Philodromidae	*Philodromus cespitum*	SMTPB7424-13	KP650238
Araneae	Philodromidae	*Philodromus cespitum*	SMTPB881-13	KP646361
Araneae	Philodromidae	*Philodromus cespitum*	SMTPB8963-13	KP647784
Araneae	Philodromidae	*Philodromus cespitum*	SMTPB8964-13	KP647910
Araneae	Philodromidae	*Philodromus cespitum*	SMTPB8965-13	KP649946
Araneae	Philodromidae	*Philodromus cespitum*	SMTPB8966-13	KP645413
Araneae	Philodromidae	*Philodromus cespitum*	SMTPB9465-13	KP646990
Araneae	Philodromidae	*Philodromus dispar*	CNGIB543-12	KM839237
Araneae	Philodromidae	*Philodromus dispar*	CNGIB544-12	KM833955
Araneae	Philodromidae	*Philodromus dispar*	CNPCA031-12	KM824206
Araneae	Philodromidae	*Philodromus dispar*	CNPCB043-12	KM840766
Araneae	Philodromidae	*Philodromus dispar*	CNPCD033-12	KM828395
Araneae	Philodromidae	*Philodromus dispar*	SPRMA642-10	JF887101
Araneae	Philodromidae	*Philodromus dispar*	SPRMA643-10	JF887102
Araneae	Philodromidae	*Philodromus dispar*	SPRMA747-10	JF887163
Araneae	Philodromidae	*Philodromus dispar*	SPRMA752-10	JF887166
Araneae	Philodromidae	*Philodromus dispar*	SPRMA757-10	JF887171
Araneae	Philodromidae	*Philodromus histrio*	CNGRF422-12	KM836656
Araneae	Philodromidae	*Philodromus histrio*	KKCHE1036-09	GU683720
Araneae	Philodromidae	*Philodromus mineri*	ERSPI291-09	HM376102
Araneae	Philodromidae	*Philodromus mineri*	SMTPB12362-13	KP655873
Araneae	Philodromidae	*Philodromus mysticus*	CNBAI524-13	KM831778
Araneae	Philodromidae	*Philodromus mysticus*	CNWBA002-13	KM840389
Araneae	Philodromidae	*Philodromus oneida*	CNGLF1528-13	KM836103
Araneae	Philodromidae	*Philodromus oneida*	CNGLF1920-13	KM831025
Araneae	Philodromidae	*Philodromus oneida*	CNGLF1921-13	KM835878
Araneae	Philodromidae	*Philodromus oneida*	CNGLF1924-13	KM835925
Araneae	Philodromidae	*Philodromus oneida*	CNGLF1931-13	KM834228
Araneae	Philodromidae	*Philodromus oneida*	CNGLF1932-13	KM828819
Araneae	Philodromidae	*Philodromus oneida*	CNGLF1945-13	KM826221
Araneae	Philodromidae	*Philodromus peninsulanus*	ARONT754-10	HQ924606
Araneae	Philodromidae	*Philodromus peninsulanus*	CNBPQ352-13	KM829356
Araneae	Philodromidae	*Philodromus peninsulanus*	CNPAC644-13	KM832303
Araneae	Philodromidae	*Philodromus peninsulanus*	CNPPC1920-12	KJ091968
Araneae	Philodromidae	*Philodromus peninsulanus*	CNPPD2698-12	KJ087985
Araneae	Philodromidae	*Philodromus peninsulanus*	CNPPD2702-12	KJ093246
Araneae	Philodromidae	*Philodromus peninsulanus*	CNSLQ081-13	KM828533
Araneae	Philodromidae	*Philodromus peninsulanus*	CNWBE383-13	KM827853
Araneae	Philodromidae	*Philodromus peninsulanus*	JDTGS064-09	GU679865
Araneae	Philodromidae	*Philodromus peninsulanus*	JSARA049-11	KP655463

Araneae	Philodromidae	*Philodromus peninsulanus*	PHMTT976-10	JN307913
Araneae	Philodromidae	*Philodromus peninsulanus*	RBINA5795-13	KP653238
Araneae	Philodromidae	*Philodromus rufus*	GBCH11034-13	JN817237
Araneae	Philodromidae	*Philodromus rufus vibrans*	ARONT226-09	GU682667
Araneae	Philodromidae	*Philodromus rufus vibrans*	ARONT227-09	GU682668
Araneae	Philodromidae	*Philodromus rufus vibrans*	ARONT618-10	HQ924483
Araneae	Philodromidae	*Philodromus rufus vibrans*	ARONT738-10	HQ924591
Araneae	Philodromidae	*Philodromus rufus vibrans*	ARONT782-10	HQ924630
Araneae	Philodromidae	*Philodromus rufus vibrans*	CNBPA222-12	KM830547
Araneae	Philodromidae	*Philodromus rufus vibrans*	CNBPI563-13	KM834266
Araneae	Philodromidae	*Philodromus rufus vibrans*	CNBPM115-13	KM826236
Araneae	Philodromidae	*Philodromus rufus vibrans*	CNBPT146-13	KM831570
Araneae	Philodromidae	*Philodromus rufus vibrans*	CNBPT147-13	KM839161
Araneae	Philodromidae	*Philodromus rufus vibrans*	CNBPT149-13	KM829622
Araneae	Philodromidae	*Philodromus rufus vibrans*	CNGLB056-13	KM836680
Araneae	Philodromidae	*Philodromus rufus vibrans*	CNGLE500-13	KM825640
Araneae	Philodromidae	*Philodromus rufus vibrans*	CNJAA910-12	KM838454
Araneae	Philodromidae	*Philodromus rufus vibrans*	CNJAJ006-12	KM834944
Araneae	Philodromidae	*Philodromus rufus vibrans*	CNPAB312-13	KM829825
Araneae	Philodromidae	*Philodromus rufus vibrans*	CNPAH369-13	KM837323
Araneae	Philodromidae	*Philodromus rufus vibrans*	CNRMA1106-12	KM838542
Araneae	Philodromidae	*Philodromus rufus vibrans*	CNRMG723-12	KM837175
Araneae	Philodromidae	*Philodromus rufus vibrans*	CNSLC533-12	KM824420
Araneae	Philodromidae	*Philodromus rufus vibrans*	CNSLC537-12	KM837610
Araneae	Philodromidae	*Philodromus rufus vibrans*	CNSLO205-13	KM837309
Araneae	Philodromidae	*Philodromus rufus vibrans*	CNSLQ079-13	KM838982
Araneae	Philodromidae	*Philodromus rufus vibrans*	CNSLQ092-13	KM837252
Araneae	Philodromidae	*Philodromus rufus vibrans*	CNSLQ094-13	KM839595
Araneae	Philodromidae	*Philodromus rufus vibrans*	CNSLR097-13	KM827759
Araneae	Philodromidae	*Philodromus rufus vibrans*	CNWBB107-13	KM833453
Araneae	Philodromidae	*Philodromus rufus vibrans*	CNWBC127-13	KM835956
Araneae	Philodromidae	*Philodromus rufus vibrans*	CNWBE380-13	KM833567
Araneae	Philodromidae	*Philodromus rufus vibrans*	CNWBE384-13	KM839605
Araneae	Philodromidae	*Philodromus rufus vibrans*	CNWBG1010-13	KM836159
Araneae	Philodromidae	*Philodromus rufus vibrans*	CNWLB644-12	KM826570
Araneae	Philodromidae	*Philodromus rufus vibrans*	HEOCT995-12	KP648233
Araneae	Philodromidae	*Philodromus rufus vibrans*	JSARA050-11	KP654965
Araneae	Philodromidae	*Philodromus rufus vibrans*	JSARA057-11	KP646947
Araneae	Philodromidae	*Philodromus rufus vibrans*	JSARA058-11	KP651676
Araneae	Philodromidae	*Philodromus rufus vibrans*	JSARA061-11	KP650051
Araneae	Philodromidae	*Philodromus rufus vibrans*	JSARA074-11	KP652314
Araneae	Philodromidae	*Philodromus rufus vibrans*	JSJUL2558-12	KP648137
Araneae	Philodromidae	*Philodromus rufus vibrans*	JSSEP1236-12	KP647957
Araneae	Philodromidae	*Philodromus rufus vibrans*	PHJUL2617-11	KP656175
Araneae	Philodromidae	*Philodromus rufus vibrans*	PHJUN4073-12	KP652607
Araneae	Philodromidae	*Philodromus rufus vibrans*	PHMTT622-10	JN307893
Araneae	Philodromidae	*Philodromus rufus vibrans*	PHMTT624-10	JN307895
Araneae	Philodromidae	*Philodromus rufus vibrans*	PHMTT632-10	JN307900
Araneae	Philodromidae	*Philodromus rufus vibrans*	PHMTT979-10	JN307916
Araneae	Philodromidae	*Philodromus rufus vibrans*	PHMTT980-10	JN307917
Araneae	Philodromidae	*Philodromus rufus vibrans*	PHMTT983-10	KP656402
Araneae	Philodromidae	*Philodromus rufus vibrans*	PHMTU041-10	JN307920
Araneae	Philodromidae	*Philodromus rufus vibrans*	PHMTU045-10	JN307923

Araneae	Philodromidae	*Philodromus rufus vibrans*	PHMTU050-10	JN307927
Araneae	Philodromidae	*Philodromus rufus vibrans*	PHMTU052-10	JN307929
Araneae	Philodromidae	*Philodromus rufus vibrans*	PHMTU069-10	JN307935
Araneae	Philodromidae	*Philodromus rufus vibrans*	PHMTU070-10	JN307936
Araneae	Philodromidae	*Philodromus rufus vibrans*	PHMTU075-10	JN307939
Araneae	Philodromidae	*Philodromus rufus vibrans*	PHMTU077-10	JN307940
Araneae	Philodromidae	*Philodromus rufus vibrans*	PHMTU083-10	KP648782
Araneae	Philodromidae	*Philodromus rufus vibrans*	PHMTU089-10	JN307947
Araneae	Philodromidae	*Philodromus rufus vibrans*	PHMTU097-10	JN307952
Araneae	Philodromidae	*Philodromus rufus vibrans*	PHMTW363-10	KP646794
Araneae	Philodromidae	*Philodromus rufus vibrans*	PHNOV556-12	KP653416
Araneae	Philodromidae	*Philodromus rufus vibrans*	PHOCT949-12	KP652497
Araneae	Philodromidae	*Philodromus rufus vibrans*	PHOCT950-12	KP649034
Araneae	Philodromidae	*Philodromus rufus vibrans*	PHOCT951-12	KP648340
Araneae	Philodromidae	*Philodromus rufus vibrans*	PHOCT954-12	KP645547
Araneae	Philodromidae	*Philodromus rufus vibrans*	PHSEP1947-11	KP656300
Araneae	Philodromidae	*Philodromus rufus vibrans*	RBCH106-04	DQ127426
Araneae	Philodromidae	*Philodromus rufus vibrans*	RBCH172-04	DQ127366
Araneae	Philodromidae	*Philodromus rufus vibrans*	RBCH183-04	DQ127345
Araneae	Philodromidae	*Philodromus rufus vibrans*	RBCH202-04	DQ127332
Araneae	Philodromidae	*Philodromus rufus vibrans*	RBINA1873-13	KP646194
Araneae	Philodromidae	*Philodromus rufus vibrans*	RBINA3358-13	KP648076
Araneae	Philodromidae	*Philodromus rufus vibrans*	RBINA822-13	KP648778
Araneae	Philodromidae	*Philodromus rufus vibrans*	SMTPB15234-13	KP648976
Araneae	Philodromidae	*Philodromus rufus vibrans*	SMTPB15359-13	KP649802
Araneae	Philodromidae	*Philodromus rufus vibrans*	SMTPB17493-13	KP646373
Araneae	Philodromidae	*Philodromus rufus vibrans*	SMTPB17494-13	KP648398
Araneae	Philodromidae	*Philodromus rufus vibrans*	SMTPB17902-13	KP646813
Araneae	Philodromidae	*Philodromus rufus vibrans*	SMTPB17903-13	KP651847
Araneae	Philodromidae	*Philodromus rufus vibrans*	SMTPB20151-13	KP657333
Araneae	Philodromidae	*Philodromus rufus vibrans*	SMTPB3380-13	KP656021
Araneae	Philodromidae	*Philodromus rufus vibrans*	SMTPB4251-13	KP647341
Araneae	Philodromidae	*Philodromus rufus vibrans*	SMTPB7655-13	KP657203
Araneae	Philodromidae	*Philodromus rufus vibrans*	SMTPB7906-13	KP650963
Araneae	Philodromidae	*Philodromus rufus vibrans*	SMTPB9354-13	KP651249
Araneae	Philodromidae	*Philodromus rufus vibrans*	SPRMA199-10	HQ580813
Araneae	Philodromidae	*Philodromus rufus vibrans*	SPRMA849-12	KP647263
Araneae	Philodromidae	*Philodromus sp. SPB-2007*	GBCH3452-09	EU168157
Araneae	Philodromidae	*Philodromus spectabilis*	SPRMA945-12	KP649908
Araneae	Philodromidae	*Philodromus subaureolus*	GBCH11035-13	JN817236
Araneae	Philodromidae	*Philodromus vulgaris*	ARONT352-09	GU682884
Araneae	Philodromidae	*Philodromus vulgaris*	ARONT353-09	GU682885
Araneae	Philodromidae	*Philodromus vulgaris*	ARONT354-09	GU682882
Araneae	Philodromidae	*Philodromus vulgaris*	ARONT394-09	GU682903
Araneae	Philodromidae	*Philodromus vulgaris*	ARONT594-10	HQ924469
Araneae	Philodromidae	*Philodromus vulgaris*	ARONT617-10	HQ924482
Araneae	Philodromidae	*Philodromus vulgaris*	CNBPK415-13	KM830623
Araneae	Philodromidae	*Philodromus vulgaris*	CNRMC1571-12	KM839850
Araneae	Philodromidae	*Philodromus vulgaris*	CNSLK010-12	KM835599
Araneae	Philodromidae	*Philodromus vulgaris*	CNWLI001-12	KM833566
Araneae	Philodromidae	*Philodromus vulgaris*	ERSPI352-09	GU682491
Araneae	Philodromidae	*Philodromus vulgaris*	JSARA075-11	KP651638
Araneae	Philodromidae	*Philodromus vulgaris*	JSARA076-11	KP657407

Araneae	Philodromidae	*Philodromus vulgaris*	PHAUG1822-12	KP650868
Araneae	Philodromidae	*Philodromus vulgaris*	PHMTT621-10	JN307892
Araneae	Philodromidae	*Philodromus vulgaris*	PHMTW371-10	KP649435
Araneae	Philodromidae	*Philodromus vulgaris*	RBCH076-04	DQ127460
Araneae	Philodromidae	*Philodromus vulgaris*	RBCH110-04	DQ127414
Araneae	Philodromidae	*Philodromus vulgaris*	RBCH144-04	DQ127386
Araneae	Philodromidae	*Philodromus vulgaris*	RBCH147-04	DQ127389
Araneae	Philodromidae	*Philodromus vulgaris*	RBCH151-04	DQ127377
Araneae	Philodromidae	*Philodromus vulgaris*	RBINA1849-13	KP653778
Araneae	Philodromidae	*Philodromus vulgaris*	RBINA2843-13	KP656164
Araneae	Philodromidae	*Philodromus vulgaris*	RBINA3328-13	KP654444
Araneae	Philodromidae	*Philodromus vulgaris*	RBINA5311-13	KP652297
Araneae	Philodromidae	*Thanatus bungei*	SPRMA372-10	HQ580919
Araneae	Philodromidae	*Thanatus formicinus*	CNWBE371-13	KM837164
Araneae	Philodromidae	*Thanatus formicinus*	ERSPI242-09	KP647096
Araneae	Philodromidae	*Thanatus formicinus*	KKCHE068-06	KF368896
Araneae	Philodromidae	*Thanatus rubicellus*	JDTGS022-09	GU679896
Araneae	Philodromidae	*Thanatus striatus*	SMTPB18885-13	KP646743
Araneae	Philodromidae	*Thanatus striatus*	SMTPB6523-13	KP645568
Araneae	Philodromidae	*Tibellus asiaticus*	SSWLB3951-13	KM833791
Araneae	Philodromidae	*Tibellus asiaticus*	SSWLE7989-13	KM838675
Araneae	Philodromidae	*Tibellus maritimus*	CNWBA001-13	KM831335
Araneae	Philodromidae	*Tibellus maritimus*	CNWBE372-13	KM826755
Araneae	Philodromidae	*Tibellus maritimus*	GBADC046-10	HQ956707
Araneae	Philodromidae	*Tibellus maritimus*	RBINA3333-13	KP645956
Araneae	Philodromidae	*Tibellus maritimus*	RBINA3381-13	KP653861
Araneae	Philodromidae	*Tibellus maritimus*	RBINA3523-13	KP646839
Araneae	Philodromidae	*Tibellus maritimus*	RBINA3539-13	KP651324
Araneae	Philodromidae	*Tibellus maritimus*	RBINA3545-13	KP655520
Araneae	Philodromidae	*Tibellus maritimus*	RBINA4072-13	KP652583
Araneae	Philodromidae	*Tibellus maritimus*	RBINA4073-13	KP657143
Araneae	Philodromidae	*Tibellus maritimus*	RBINA4077-13	KP650710
Araneae	Philodromidae	*Tibellus maritimus*	RBINA713-13	KP650213
Araneae	Philodromidae	*Tibellus maritimus*	RBINA716-13	KP646202
Araneae	Philodromidae	*Tibellus maritimus*	RBINA719-13	KP648490
Araneae	Philodromidae	*Tibellus maritimus*	SMTPB11088-13	KP654702
Araneae	Philodromidae	*Tibellus maritimus*	SMTPB20454-13	KP656272
Araneae	Philodromidae	*Tibellus oblongus*	GBCH11032-13	JN817239
Araneae	Pholcidae	*Anansus*	GBCH11538-13	JX023572
Araneae	Pholcidae	*Artema atlanta*	GBCH0782-06	AY560771
Araneae	Pholcidae	*Buitinga*	GBCH11558-13	JX023552
Araneae	Pholcidae	*Buitinga cf. nigrescens*	GBCH11537-13	JX023573
Araneae	Pholcidae	*Carapoia*	GBCH11523-13	JX023587
Araneae	Pholcidae	*Carapoia*	GBCH11525-13	JX023585
Araneae	Pholcidae	*Carapoia*	GBCH11526-13	JX023584
Araneae	Pholcidae	*Carapoia*	GBCH11527-13	JX023583
Araneae	Pholcidae	*Carapoia genitalis*	GBCH11528-13	JX023582
Araneae	Pholcidae	*Ciboneya antraia*	GBCH0804-06	AY560794
Araneae	Pholcidae	*Crossopriza lyoni*	GBCH0785-06	AY560774
Araneae	Pholcidae	*Crossopriza lyoni*	GBCH0786-06	AY560775
Araneae	Pholcidae	*Crossopriza lyoni*	GBCH11559-13	JX023551
Araneae	Pholcidae	*Holocnemus hispanicus*	GBCH11510-13	JX023600
Araneae	Pholcidae	*Holocnemus pluchei*	GBCH0787-06	AY560776

Araneae	Pholcidae	*Holocnemus pluchei*	GBCH0788-06	AY560777
Araneae	Pholcidae	*Khorata khammouan*	GBCH11514-13	JX023596
Araneae	Pholcidae	*Leptopholcus*	GBCH11552-13	JX023558
Araneae	Pholcidae	*Leptopholcus dschang*	GBCH11548-13	JX023562
Araneae	Pholcidae	*Leptopholcus gracilis*	GBCH11556-13	JX023554
Araneae	Pholcidae	*Leptopholcus guineensis*	GBCH11562-13	JX023548
Araneae	Pholcidae	*Leptopholcus tipula*	GBCH11508-13	JX023602
Araneae	Pholcidae	*Leptopholcus tipula*	GBCH11550-13	JX023560
Araneae	Pholcidae	*Leptopholcus tipula*	GBCH11565-13	JX023545
Araneae	Pholcidae	*Mesabolivar*	GBCH11518-13	JX023592
Araneae	Pholcidae	*Mesabolivar*	GBCH11520-13	JX023590
Araneae	Pholcidae	*Mesabolivar aurantiacus*	GBCH0789-06	AY560778
Araneae	Pholcidae	*Mesabolivar aurantiacus*	GBCH0790-06	AY560779
Araneae	Pholcidae	*Mesabolivar aurantiacus*	GBCH1550-06	DQ667862
Araneae	Pholcidae	*Mesabolivar brasiliensis*	GBCH0791-06	AY560780
Araneae	Pholcidae	*Mesabolivar brasiliensis*	GBCH1551-06	DQ667863
Araneae	Pholcidae	*Mesabolivar brasiliensis*	GBCH3819-09	DQ667864
Araneae	Pholcidae	*Mesabolivar cyaneotaeniatus*	GBCH0792-06	AY560781
Araneae	Pholcidae	*Mesabolivar cyaneotaeniatus*	GBCH2165-07	DQ667869
Araneae	Pholcidae	*Mesabolivar cyaneotaeniatus*	GBCH2166-07	DQ667868
Araneae	Pholcidae	*Mesabolivar cyaneotaeniatus*	GBCH3816-09	DQ667867
Araneae	Pholcidae	*Mesabolivar cyaneotaeniatus*	GBCH3817-09	DQ667866
Araneae	Pholcidae	*Metagonia cf. beni*	GBCH11534-13	JX023576
Araneae	Pholcidae	*Metagonia cf. bifida*	GBCH11533-13	JX023577
Araneae	Pholcidae	*Metagonia cf. petropolis*	GBCH11505-13	JX023605
Araneae	Pholcidae	*Metagonia cf. petropolis*	GBCH11532-13	JX023578
Araneae	Pholcidae	*Metagonia furcata*	GBCH11519-13	JX023591
Araneae	Pholcidae	*Metagonia sp. BB-2004b*	GBCH0795-06	AY560784
Araneae	Pholcidae	*Micropholcus fauroti*	GBCH11536-13	JX023574
Araneae	Pholcidae	*Modisimus coco*	GBCH11513-13	JX023597
Araneae	Pholcidae	*Nita elsaff*	GBCH11509-13	JX023601
Araneae	Pholcidae	*Nyikoa limbe*	GBCH11546-13	JX023564
Araneae	Pholcidae	*Nyikoa limbe*	GBCH11566-13	JX023544
Araneae	Pholcidae	*Paramicromerys*	GBCH11516-13	JX023594
Araneae	Pholcidae	*Pehrforsskalia conopyga*	GBCH11551-13	JX023559
Araneae	Pholcidae	*Pehrforsskalia conopyga*	GBCH11561-13	JX023549
Araneae	Pholcidae	*Pholcophora americana*	GBCH2140-07	DQ667904
Araneae	Pholcidae	*Pholcophora americana*	SPRMA181-10	HQ580799
Araneae	Pholcidae	*Pholcophora americana*	SPRMA283-10	HQ580882
Araneae	Pholcidae	*Pholcophora americana*	SPRMA356-10	HQ580908
Araneae	Pholcidae	*Pholcophora americana*	SPRMA357-10	HQ580909
Araneae	Pholcidae	*Pholcophora americana*	SPRMA735-10	JF887156
Araneae	Pholcidae	*Pholcophora americana*	SPRMA780-12	KP647977
Araneae	Pholcidae	*Pholcus*	GBCH11512-13	JX023598
Araneae	Pholcidae	*Pholcus*	GBCH11531-13	JX023579
Araneae	Pholcidae	*Pholcus atrigularis*	GBCH11504-13	JX023606
Araneae	Pholcidae	*Pholcus attuleh*	GBCH11547-13	JX023563
Araneae	Pholcidae	*Pholcus baka*	GBCH11535-13	JX023575
Araneae	Pholcidae	*Pholcus bourgini*	GBCH11564-13	JX023546
Araneae	Pholcidae	*Pholcus cf. jaegeri*	GBCH11507-13	JX023603
Araneae	Pholcidae	*Pholcus crypticolens*	GBCH11544-13	JX023566
Araneae	Pholcidae	*Pholcus dade*	GBCH11515-13	JX023595
Araneae	Pholcidae	*Pholcus doucki*	GBCH11563-13	JX023547

Araneae	Pholcidae	*Pholcus fagei*	GBCH11557-13	JX023553
Araneae	Pholcidae	*Pholcus fengcheng*	GBCH11502-13	JX023608
Araneae	Pholcidae	*Pholcus gosuensis*	GBCH11198-13	JN817072
Araneae	Pholcidae	*Pholcus jiuwei*	GBCH11501-13	JX023609
Araneae	Pholcidae	*Pholcus kindia*	GBCH11530-13	JX023580
Araneae	Pholcidae	*Pholcus kribi*	GBCH11549-13	JX023561
Araneae	Pholcidae	*Pholcus leruthi*	GBCH11554-13	JX023556
Araneae	Pholcidae	*Pholcus manueli*	ARONT200-09	GU682732
Araneae	Pholcidae	*Pholcus manueli*	GBCH2135-07	DQ667910
Araneae	Pholcidae	*Pholcus manueli*	GBCH2136-07	DQ667909
Araneae	Pholcidae	*Pholcus manueli*	GBCH2137-07	DQ667908
Araneae	Pholcidae	*Pholcus manueli*	GBCH2138-07	DQ667907
Araneae	Pholcidae	*Pholcus manueli*	GBCH3810-09	DQ667912
Araneae	Pholcidae	*Pholcus manueli*	GBCH3811-09	DQ667911
Araneae	Pholcidae	*Pholcus montanus*	GBCH11194-13	JN817076
Araneae	Pholcidae	*Pholcus ornatus*	GBCH11511-13	JX023599
Araneae	Pholcidae	*Pholcus phalangioides*	GBCH10809-13	NC_020324
Araneae	Pholcidae	*Pholcus phalangioides*	GBCH11199-13	JN817071
Araneae	Pholcidae	*Pholcus phalangioides*	GBCH11966-13	JQ407804
Araneae	Pholcidae	*Pholcus phalangioides*	GBCH1565-06	DQ667919
Araneae	Pholcidae	*Pholcus phalangioides*	GBCH1566-06	DQ667920
Araneae	Pholcidae	*Pholcus phalangioides*	GBCH2124-07	DQ667925
Araneae	Pholcidae	*Pholcus phalangioides*	GBCH2125-07	DQ667924
Araneae	Pholcidae	*Pholcus phalangioides*	GBCH2128-07	DQ667921
Araneae	Pholcidae	*Pholcus sokkrisanensis*	GBCH11196-13	JN817074
Araneae	Pholcidae	*Physocyclus globosus*	GBCH0798-06	AY560788
Araneae	Pholcidae	*Psilochorus*	GBCH11524-13	JX023586
Araneae	Pholcidae	*Psilochorus hesperus*	SPRMA009-10	HQ977120
Araneae	Pholcidae	*Psilochorus hesperus*	SPRMA010-10	HQ977121
Araneae	Pholcidae	*Psilochorus hesperus*	SPRMA011-10	HQ977122
Araneae	Pholcidae	*Psilochorus hesperus*	SPRMA343-10	HQ580901
Araneae	Pholcidae	*Psilochorus hesperus*	SPRMA728-10	JF887151
Araneae	Pholcidae	*Psilochorus hesperus*	SPRMA737-10	KR070587
Araneae	Pholcidae	*Psilochorus itaguyrussu*	GBCH0793-06	AY560782
Araneae	Pholcidae	*Psilochorus itaguyrussu*	GBCH11517-13	JX023593
Araneae	Pholcidae	*Psilochorus simoni*	GBCH0799-06	AY560789
Araneae	Pholcidae	*Psilochorus simoni*	GBCH3804-09	DQ667936
Araneae	Pholcidae	*Quamtana*	GBCH11529-13	JX023581
Araneae	Pholcidae	*Quamtana*	GBCH11543-13	JX023567
Araneae	Pholcidae	*Quamtana filmeri*	GBCH11503-13	JX023607
Araneae	Pholcidae	*Quamtana vidal*	GBCH0802-06	AY560792
Araneae	Pholcidae	*Smeringopina*	GBCH11545-13	JX023565
Araneae	Pholcidae	*Smeringopina guineensis*	GBCH11560-13	JX023550
Araneae	Pholcidae	*Smeringopus*	GBCH11555-13	JX023555
Araneae	Pholcidae	*Spermophora*	GBCH11539-13	JX023571
Araneae	Pholcidae	*Spermophora*	GBCH11540-13	JX023570
Araneae	Pholcidae	*Spermophora cf. berlandi*	GBCH11542-13	JX023568
Araneae	Pholcidae	*Spermophora minotaura*	GBCH11506-13	JX023604
Araneae	Pholcidae	*Spermophora minotaura*	GBCH11541-13	JX023569
Araneae	Pholcidae	*Spermophora minotaura*	GBCH11553-13	JX023557
Araneae	Pholcidae	*Spermophora senoculata*	GBCH0801-06	AY560791
Araneae	Pholcidae	*Spermophora senoculata*	GBCH11195-13	JN817075
Araneae	Pholcidae	*Trichocyclus sp. BB-2004*	GBCH0783-06	AY560772

Araneae	Pholcidae	*Tupigea iguassuensis*	GBCH11522-13	JX023588
Araneae	Pholcidae	*Tupigea nadleri*	GBCH11521-13	JX023589
Araneae	Phrurolithidae	*Phrurotimpus alarius*	ARONT750-10	HQ924602
Araneae	Phrurolithidae	*Phrurotimpus alarius*	RBINA153-13	KP646308
Araneae	Phrurolithidae	*Phrurotimpus alarius*	RBINA3427-13	KP651088
Araneae	Phrurolithidae	*Phrurotimpus alarius*	RBINA5179-13	KP646814
Araneae	Phrurolithidae	*Phrurotimpus alarius*	RBINA5183-13	KP649381
Araneae	Phrurolithidae	*Phrurotimpus alarius*	SPRMA597-10	JF887079
Araneae	Phrurolithidae	*Phrurotimpus borealis*	ARONT438-09	GU682918
Araneae	Phrurolithidae	*Phrurotimpus borealis*	ARONT439-09	GU682919
Araneae	Phrurolithidae	*Phrurotimpus borealis*	CNPCD035-12	KM833886
Araneae	Phrurolithidae	*Phrurotimpus borealis*	CNSLC539-12	KM826366
Araneae	Phrurolithidae	*Phrurotimpus borealis*	ERSPI273-09	GU682552
Araneae	Phrurolithidae	*Phrurotimpus borealis*	ERSPI353-09	GU682492
Araneae	Phrurolithidae	*Phrurotimpus borealis*	ERSPI367-09	GU682482
Araneae	Phrurolithidae	*Phrurotimpus borealis*	RBINA2754-13	KP657464
Araneae	Phrurolithidae	*Phrurotimpus borealis*	RBINA2755-13	KP656449
Araneae	Phrurolithidae	*Phrurotimpus borealis*	RBINA2756-13	KP651408
Araneae	Phrurolithidae	*Phrurotimpus borealis*	RBINA2763-13	KP653904
Araneae	Phrurolithidae	*Phrurotimpus borealis*	RBINA2764-13	KP652806
Araneae	Phrurolithidae	*Phrurotimpus borealis*	RBINA2767-13	KP653165
Araneae	Phrurolithidae	*Phrurotimpus borealis*	RBINA2769-13	KP651796
Araneae	Phrurolithidae	*Phrurotimpus borealis*	RBINA3119-13	KP654933
Araneae	Phrurolithidae	*Phrurotimpus borealis*	RBINA3138-13	KP647824
Araneae	Phrurolithidae	*Phrurotimpus borealis*	RBINA3321-13	KP651492
Araneae	Phrurolithidae	*Phrurotimpus borealis*	RBINA3322-13	KP655398
Araneae	Phrurolithidae	*Phrurotimpus borealis*	RBINA3428-13	KP646559
Araneae	Phrurolithidae	*Phrurotimpus borealis*	RBINA5185-13	KP647774
Araneae	Phrurolithidae	*Phrurotimpus borealis*	RBINA5186-13	KP654673
Araneae	Phrurolithidae	*Phrurotimpus borealis*	RBINA5188-13	KP648359
Araneae	Phrurolithidae	*Phrurotimpus borealis*	RBINA5189-13	KP654890
Araneae	Phrurolithidae	*Phrurotimpus borealis*	SMTPB1067-13	KP649907
Araneae	Phrurolithidae	*Phrurotimpus borealis*	SPRMA598-10	JF887080
Araneae	Phrurolithidae	*Phrurotimpus certus*	SPRMA961-12	KP649354
Araneae	Phrurolithidae	*Scotinella britcheri*	ERSPI272-09	GU682551
Araneae	Phrurolithidae	*Scotinella britcheri*	ERSPI283-09	GU682538
Araneae	Phrurolithidae	*Scotinella madisonia*	SMTPB10316-13	KP647905
Araneae	Phrurolithidae	*Scotinella madisonia*	SMTPB10317-13	KP652518
Araneae	Phrurolithidae	*Scotinella madisonia*	SMTPB13466-13	KP648637
Araneae	Phrurolithidae	*Scotinella madisonia*	SMTPB13467-13	KP653272
Araneae	Phrurolithidae	*Scotinella madisonia*	SMTPB13468-13	KP648307
Araneae	Phrurolithidae	*Scotinella madisonia*	SMTPB13469-13	KP655881
Araneae	Phrurolithidae	*Scotinella madisonia*	SMTPB13470-13	KP656152
Araneae	Phrurolithidae	*Scotinella madisonia*	SMTPB15233-13	KP645618
Araneae	Phrurolithidae	*Scotinella madisonia*	SMTPB15235-13	KP656107
Araneae	Phrurolithidae	*Scotinella madisonia*	SMTPB15236-13	KP647868
Araneae	Phrurolithidae	*Scotinella madisonia*	SMTPB15237-13	KP651031
Araneae	Phrurolithidae	*Scotinella madisonia*	SMTPB15238-13	KP654993
Araneae	Phrurolithidae	*Scotinella madisonia*	SMTPB15239-13	KP652995
Araneae	Phrurolithidae	*Scotinella madisonia*	SMTPB15240-13	KP652392
Araneae	Phrurolithidae	*Scotinella madisonia*	SMTPB15241-13	KP650772
Araneae	Phrurolithidae	*Scotinella madisonia*	SMTPB15243-13	KP656476
Araneae	Phrurolithidae	*Scotinella madisonia*	SMTPB15244-13	KP654254

Araneae	Phrurolithidae	*Scotinella madisonia*	SMTPB15246-13	KP646676
Araneae	Phrurolithidae	*Scotinella madisonia*	SMTPB15247-13	KP646948
Araneae	Phrurolithidae	*Scotinella madisonia*	SMTPB15248-13	KP647567
Araneae	Phrurolithidae	*Scotinella madisonia*	SMTPB15249-13	KP651468
Araneae	Phrurolithidae	*Scotinella madisonia*	SMTPB15250-13	KP652163
Araneae	Phrurolithidae	*Scotinella madisonia*	SMTPB15252-13	KP653692
Araneae	Phrurolithidae	*Scotinella madisonia*	SMTPB15253-13	KP647374
Araneae	Phrurolithidae	*Scotinella madisonia*	SMTPB15254-13	KP653605
Araneae	Phrurolithidae	*Scotinella madisonia*	SMTPB15255-13	KP656420
Araneae	Phrurolithidae	*Scotinella madisonia*	SMTPB15256-13	KP652875
Araneae	Phrurolithidae	*Scotinella madisonia*	SMTPB15257-13	KP655837
Araneae	Phrurolithidae	*Scotinella madisonia*	SMTPB15258-13	KP647179
Araneae	Phrurolithidae	*Scotinella madisonia*	SMTPB15259-13	KP657142
Araneae	Phrurolithidae	*Scotinella madisonia*	SMTPB15260-13	KP648597
Araneae	Phrurolithidae	*Scotinella madisonia*	SMTPB15261-13	KP646522
Araneae	Phrurolithidae	*Scotinella madisonia*	SMTPB15262-13	KP653019
Araneae	Phrurolithidae	*Scotinella madisonia*	SMTPB15669-13	KP650399
Araneae	Phrurolithidae	*Scotinella madisonia*	SMTPB15672-13	KP649356
Araneae	Phrurolithidae	*Scotinella madisonia*	SMTPB15673-13	KP656706
Araneae	Phrurolithidae	*Scotinella madisonia*	SMTPB15674-13	KP650459
Araneae	Phrurolithidae	*Scotinella minnetonka*	ARONT745-10	HQ924598
Araneae	Phrurolithidae	*Scotinella minnetonka*	ARONT799-10	HQ924644
Araneae	Phrurolithidae	*Scotinella pugnata*	ERSPI259-09	HM376098
Araneae	Phyxelididae	*Ambohima andrefana*	GACAC556-12	JF411092
Araneae	Phyxelididae	*Ambohima andrefana*	GACAC557-12	JF411093
Araneae	Phyxelididae	*Ambohima andrefana*	GACAC558-12	JF411094
Araneae	Phyxelididae	*Ambohima andrefana*	GACAC559-12	JF411095
Araneae	Phyxelididae	*Ambohima antisinanana*	GACAC565-12	JF411101
Araneae	Phyxelididae	*Ambohima avaratra*	GACAC574-12	JF411110
Araneae	Phyxelididae	*Ambohima maizina*	GACAC554-12	JF411090
Araneae	Phyxelididae	*Ambohima ranohira*	GACAC560-12	JF411096
Araneae	Phyxelididae	*Ambohima ranohira*	GACAC561-12	JF411097
Araneae	Phyxelididae	*Ambohima ranohira*	GACAC562-12	JF411098
Araneae	Phyxelididae	*Ambohima sublima*	GACAC566-12	JF411102
Araneae	Phyxelididae	*Ambohima sublima*	GACAC567-12	JF411103
Araneae	Phyxelididae	*Ambohima sublima*	GACAC568-12	JF411104
Araneae	Phyxelididae	*Ambohima sublima*	GACAC569-12	JF411105
Araneae	Phyxelididae	*Ambohima sublima*	GACAC570-12	JF411106
Araneae	Phyxelididae	*Ambohima sublima*	GACAC571-12	JF411107
Araneae	Phyxelididae	*Ambohima sublima*	GACAC572-12	JF411108
Araneae	Phyxelididae	*Ambohima sublima*	GACAC573-12	JF411109
Araneae	Phyxelididae	*Ambohima vato*	GACAC563-12	JF411099
Araneae	Phyxelididae	*Ambohima vato*	GACAC564-12	JF411100
Araneae	Phyxelididae	*Ambohima zandry*	GACAC553-12	JF411089
Araneae	Phyxelididae	*Ambohima zoky*	GACAC555-12	JF411091
Araneae	Phyxelididae	*Malaika longipes*	GACAC550-12	JF411086
Araneae	Phyxelididae	*Manampoka atsimo*	GACAC583-12	JF411119
Araneae	Phyxelididae	*Manampoka atsimo*	GACAC584-12	JF411120
Araneae	Phyxelididae	*Phyxelida tanganensis*	GACAC551-12	JF411087
Araneae	Phyxelididae	*Rahavavy fanivelona*	GACAC576-12	JF411112
Araneae	Phyxelididae	*Rahavavy fanivelona*	GACAC577-12	JF411113
Araneae	Phyxelididae	*Rahavavy ida*	GACAC578-12	JF411114
Araneae	Phyxelididae	*Rahavavy ida*	GACAC579-12	JF411115

Araneae	Phyxelididae	*Rahavavy malagasyana*	GACAC580-12	JF411116
Araneae	Phyxelididae	*Rahavavy malagasyana*	GACAC582-12	JF411118
Araneae	Phyxelididae	*Themacrys irrorata*	GACAC575-12	JF411111
Araneae	Phyxelididae	*Xevioso colobata*	GACAC549-12	JF411085
Araneae	Phyxelididae	*Xevioso kulufa*	GACAC548-12	JF411084
Araneae	Pimoidae	*Pimoa altioculata*	CNPCO029-13	KM826392
Araneae	Pimoidae	*Pimoa altioculata*	SPIAL057-10	HQ580562
Araneae	Pimoidae	*Pimoa altioculata*	SPRMA867-12	KP654481
Araneae	Pimoidae	*Pimoa haden*	SPRMA120-10	HQ580754
Araneae	Pimoidae	*Pimoa haden*	SPRMA827-12	KP650096
Araneae	Pimoidae	*Pimoa sp. TAB-2009*	GBCH4015-09	FJ607584
Araneae	Pisauridae	*Dolomedes aquaticus*	GACAC646-12	JF792268
Araneae	Pisauridae	*Dolomedes aquaticus*	GACAC647-12	JF792269
Araneae	Pisauridae	*Dolomedes aquaticus*	GACAC648-12	JF792270
Araneae	Pisauridae	*Dolomedes aquaticus*	GACAC649-12	JF792271
Araneae	Pisauridae	*Dolomedes aquaticus*	GACAC650-12	JF792272
Araneae	Pisauridae	*Dolomedes aquaticus*	GACAC651-12	JF792273
Araneae	Pisauridae	*Dolomedes aquaticus*	GACAC652-12	JF792274
Araneae	Pisauridae	*Dolomedes aquaticus*	GACAC653-12	JF792275
Araneae	Pisauridae	*Dolomedes aquaticus*	GACAC654-12	JF792276
Araneae	Pisauridae	*Dolomedes aquaticus*	GACAC655-12	JF792277
Araneae	Pisauridae	*Dolomedes aquaticus*	GACAC656-12	JF792278
Araneae	Pisauridae	*Dolomedes aquaticus*	GACAC657-12	JF792279
Araneae	Pisauridae	*Dolomedes aquaticus*	GACAC658-12	JF792280
Araneae	Pisauridae	*Dolomedes aquaticus*	GACAC659-12	JF792281
Araneae	Pisauridae	*Dolomedes aquaticus*	GACAC660-12	JF792282
Araneae	Pisauridae	*Dolomedes aquaticus*	GACAC661-12	JF792283
Araneae	Pisauridae	*Dolomedes aquaticus*	GACAC662-12	JF792284
Araneae	Pisauridae	*Dolomedes aquaticus*	GACAC663-12	JF792285
Araneae	Pisauridae	*Dolomedes aquaticus*	GACAC664-12	JF792286
Araneae	Pisauridae	*Dolomedes aquaticus*	GACAC665-12	JF792287
Araneae	Pisauridae	*Dolomedes aquaticus*	GACAC666-12	JF792288
Araneae	Pisauridae	*Dolomedes aquaticus*	GACAC667-12	JF792289
Araneae	Pisauridae	*Dolomedes aquaticus*	GACAC668-12	JF792290
Araneae	Pisauridae	*Dolomedes aquaticus*	GACAC669-12	JF792291
Araneae	Pisauridae	*Dolomedes aquaticus*	GACAC670-12	JF792292
Araneae	Pisauridae	*Dolomedes aquaticus*	GACAC671-12	JF792293
Araneae	Pisauridae	*Dolomedes aquaticus*	GACAC672-12	JF792294
Araneae	Pisauridae	*Dolomedes aquaticus*	GACAC673-12	JF792295
Araneae	Pisauridae	*Dolomedes aquaticus*	GACAC674-12	JF792296
Araneae	Pisauridae	*Dolomedes aquaticus*	GACAC675-12	JF792297
Araneae	Pisauridae	*Dolomedes aquaticus*	GACAC676-12	JF792298
Araneae	Pisauridae	*Dolomedes aquaticus*	GACAC677-12	JF792299
Araneae	Pisauridae	*Dolomedes aquaticus*	GACAC678-12	JF792300
Araneae	Pisauridae	*Dolomedes aquaticus*	GACAC679-12	JF792301
Araneae	Pisauridae	*Dolomedes aquaticus*	GACAC680-12	JF792302
Araneae	Pisauridae	*Dolomedes aquaticus*	GACAC682-12	JF792304
Araneae	Pisauridae	*Dolomedes aquaticus*	GACAC683-12	JF792305
Araneae	Pisauridae	*Dolomedes aquaticus*	GACAC684-12	JF792306
Araneae	Pisauridae	*Dolomedes aquaticus*	GBCH5784-13	GQ337371
Araneae	Pisauridae	*Dolomedes aquaticus*	GBCH5785-13	GQ337370
Araneae	Pisauridae	*Dolomedes aquaticus*	GBCH5786-13	GQ337369
Araneae	Pisauridae	*Dolomedes aquaticus*	GBCH5787-13	GQ337368

Araneae	Pisauridae	*Dolomedes aquaticus*	GBCH5788-13	GQ337367
Araneae	Pisauridae	*Dolomedes aquaticus*	GBCH5789-13	GQ337366
Araneae	Pisauridae	*Dolomedes aquaticus*	GBCH5790-13	GQ337365
Araneae	Pisauridae	*Dolomedes aquaticus*	GBCH5791-13	GQ337364
Araneae	Pisauridae	*Dolomedes aquaticus*	GBCH5792-13	GQ337363
Araneae	Pisauridae	*Dolomedes aquaticus*	GBCH5793-13	GQ337362
Araneae	Pisauridae	*Dolomedes aquaticus*	GBCH5794-13	GQ337361
Araneae	Pisauridae	*Dolomedes aquaticus*	GBCH5795-13	GQ337360
Araneae	Pisauridae	*Dolomedes aquaticus*	GBCH5796-13	GQ337359
Araneae	Pisauridae	*Dolomedes aquaticus*	GBCH5797-13	GQ337358
Araneae	Pisauridae	*Dolomedes aquaticus*	GBCH5798-13	GQ337357
Araneae	Pisauridae	*Dolomedes aquaticus*	GBCH5799-13	GQ337356
Araneae	Pisauridae	*Dolomedes aquaticus*	GBCH5800-13	GQ337355
Araneae	Pisauridae	*Dolomedes dondalei*	GBCH5774-13	GQ337381
Araneae	Pisauridae	*Dolomedes dondalei*	GBCH5775-13	GQ337380
Araneae	Pisauridae	*Dolomedes dondalei*	GBCH5776-13	GQ337379
Araneae	Pisauridae	*Dolomedes dondalei*	GBCH5777-13	GQ337378
Araneae	Pisauridae	*Dolomedes dondalei*	GBCH5778-13	GQ337377
Araneae	Pisauridae	*Dolomedes dondalei*	GBCH5779-13	GQ337376
Araneae	Pisauridae	*Dolomedes dondalei*	GBCH5780-13	GQ337375
Araneae	Pisauridae	*Dolomedes dondalei*	GBCH5781-13	GQ337374
Araneae	Pisauridae	*Dolomedes dondalei*	GBCH5782-13	GQ337373
Araneae	Pisauridae	*Dolomedes dondalei*	GBCH5783-13	GQ337372
Araneae	Pisauridae	*Dolomedes horishanus*	GBCH3537-09	AB374064
Araneae	Pisauridae	*Dolomedes horishanus*	GBCH3538-09	AB374063
Araneae	Pisauridae	*Dolomedes horishanus*	GBCH3539-09	AB374062
Araneae	Pisauridae	*Dolomedes japonicus*	GBCH11078-13	JN817192
Araneae	Pisauridae	*Dolomedes minor*	GBCH5801-13	GQ337354
Araneae	Pisauridae	*Dolomedes minor*	GBCH5802-13	GQ337353
Araneae	Pisauridae	*Dolomedes minor*	GBCH5803-13	GQ337352
Araneae	Pisauridae	*Dolomedes minor*	GBCH5804-13	GQ337351
Araneae	Pisauridae	*Dolomedes minor*	GBCH5805-13	GQ337350
Araneae	Pisauridae	*Dolomedes minor*	GBCH5806-13	GQ337349
Araneae	Pisauridae	*Dolomedes minor*	GBCH5807-13	GQ337348
Araneae	Pisauridae	*Dolomedes minor*	GBCH5808-13	GQ337347
Araneae	Pisauridae	*Dolomedes minor*	GBCH5809-13	GQ337346
Araneae	Pisauridae	*Dolomedes minor*	GBCH5810-13	GQ337345
Araneae	Pisauridae	*Dolomedes minor*	GBCH5811-13	GQ337344
Araneae	Pisauridae	*Dolomedes minor*	GBCH5812-13	GQ337343
Araneae	Pisauridae	*Dolomedes minor*	GBCH5813-13	GQ337342
Araneae	Pisauridae	*Dolomedes minor*	GBCH5814-13	GQ337341
Araneae	Pisauridae	*Dolomedes minor*	GBCH5815-13	GQ337340
Araneae	Pisauridae	*Dolomedes minor*	GBCH5816-13	GQ337339
Araneae	Pisauridae	*Dolomedes minor*	GBCH5817-13	GQ337338
Araneae	Pisauridae	*Dolomedes minor*	GBCH5818-13	GQ337337
Araneae	Pisauridae	*Dolomedes minor*	GBCH5819-13	GQ337336
Araneae	Pisauridae	*Dolomedes minor*	GBCH5820-13	GQ337335
Araneae	Pisauridae	*Dolomedes minor*	GBCH5821-13	GQ337334
Araneae	Pisauridae	*Dolomedes minor*	GBCH5822-13	GQ337333
Araneae	Pisauridae	*Dolomedes minor*	GBCH5823-13	GQ337332
Araneae	Pisauridae	*Dolomedes minor*	GBCH5824-13	GQ337331
Araneae	Pisauridae	*Dolomedes minor*	GBCH5825-13	GQ337330
Araneae	Pisauridae	*Dolomedes minor*	GBCH5826-13	GQ337329

Araneae	Pisauridae	*Dolomedes minor*	GBCH5827-13	GQ337328
Araneae	Pisauridae	*Dolomedes raptor*	GBCH11077-13	JN817193
Araneae	Pisauridae	*Dolomedes raptor*	GBCH3556-09	AB374045
Araneae	Pisauridae	*Dolomedes raptor*	GBCH3557-09	AB374044
Araneae	Pisauridae	*Dolomedes schauinslandi*	GBCH5770-13	GQ337385
Araneae	Pisauridae	*Dolomedes schauinslandi*	GBCH5771-13	GQ337384
Araneae	Pisauridae	*Dolomedes schauinslandi*	GBCH5772-13	GQ337383
Araneae	Pisauridae	*Dolomedes schauinslandi*	GBCH5773-13	GQ337382
Araneae	Pisauridae	*Dolomedes scriptus*	ARONT340-09	GU682879
Araneae	Pisauridae	*Dolomedes scriptus*	ARONT342-09	GU682876
Araneae	Pisauridae	*Dolomedes scriptus*	ARONT343-09	GU682877
Araneae	Pisauridae	*Dolomedes sulfureus*	GBCH11079-13	JN817191
Araneae	Pisauridae	*Dolomedes sulfureus*	GBCH3544-09	AB374057
Araneae	Pisauridae	*Dolomedes sulfureus*	GBCH3545-09	AB374056
Araneae	Pisauridae	*Dolomedes tenebrosus*	ARONT820-10	HQ924665
Araneae	Pisauridae	*Dolomedes tenebrosus*	CNSLI539-12	KM826873
Araneae	Pisauridae	*Dolomedes tenebrosus*	CNSLO001-13	KM828237
Araneae	Pisauridae	*Dolomedes tenebrosus*	GBCH4037-09	FJ607562
Araneae	Pisauridae	*Dolomedes tenebrosus*	RBCH036-04	DQ127500
Araneae	Pisauridae	*Dolomedes tenebrosus*	RBCH072-04	DQ127456
Araneae	Pisauridae	*Dolomedes tenebrosus*	RBCH089-04	DQ127441
Araneae	Pisauridae	*Dolomedes tenebrosus*	RBCH094-04	DQ127430
Araneae	Pisauridae	*Dolomedes tenebrosus*	RBINA2827-13	KP652080
Araneae	Pisauridae	*Dolomedes tenebrosus*	RBINA3285-13	KP656868
Araneae	Pisauridae	*Dolomedes tenebrosus*	RBINA3375-13	KP648655
Araneae	Pisauridae	*Dolomedes tenebrosus*	RBINA3376-13	KP656183
Araneae	Pisauridae	*Dolomedes tenebrosus*	RBINA3390-13	KP648183
Araneae	Pisauridae	*Dolomedes tenebrosus*	RBINA5805-13	KP646634
Araneae	Pisauridae	*Dolomedes triton*	ARONT259-09	GU682708
Araneae	Pisauridae	*Dolomedes triton*	ARONT493-10	HQ924388
Araneae	Pisauridae	*Dolomedes triton*	ARONT494-10	HQ924389
Araneae	Pisauridae	*Dolomedes triton*	ARONT495-10	HQ924390
Araneae	Pisauridae	*Dolomedes triton*	ARONT496-10	HQ924391
Araneae	Pisauridae	*Dolomedes triton*	ARONT497-10	HQ924392
Araneae	Pisauridae	*Dolomedes triton*	SMTPB7311-13	KP650044
Araneae	Pisauridae	*Dolomedes triton*	SPRMA535-10	JF887047
Araneae	Pisauridae	*Dolomedes triton*	SPRMA536-10	JF887048
Araneae	Pisauridae	*Dolomedes triton*	SPRMA537-10	JF887049
Araneae	Pisauridae	*Pisaura ancora*	GBCH11076-13	JN817194
Araneae	Pisauridae	*Pisaura lama*	GBCH11075-13	JN817195
Araneae	Pisauridae	*Pisaurina brevipes*	RBINA3403-13	KP657364
Araneae	Pisauridae		GBCH10172-13	JX137131
Araneae	Psechridae	*Fecenia*	GBCH10095-13	JX137208
Araneae	Psechridae	*Fecenia*	GBCH10096-13	JX137207
Araneae	Psechridae	*Fecenia*	GBCH10133-13	JX137170
Araneae	Psechridae	*Fecenia*	GBCH10148-13	JX137155
Araneae	Psechridae	*Fecenia*	GBCH10149-13	JX137154
Araneae	Psechridae	*Fecenia*	GBCH10150-13	JX137153
Araneae	Psechridae	*Fecenia*	GBCH10151-13	JX137152
Araneae	Psechridae	*Fecenia cylindrata*	GBCH10085-13	JX137218
Araneae	Psechridae	*Fecenia cylindrata*	GBCH10089-13	JX137214
Araneae	Psechridae	*Fecenia cylindrata*	GBCH10090-13	JX137213
Araneae	Psechridae	*Fecenia cylindrata*	GBCH10091-13	JX137212

Araneae	Psechridae	*Fecenia cylindrata*	GBCH10093-13	JX137210
Araneae	Psechridae	*Fecenia cylindrata*	GBCH10094-13	JX137209
Araneae	Psechridae	*Fecenia cylindrata*	GBCH10110-13	JX137193
Araneae	Psechridae	*Fecenia ochracea*	GBCH10134-13	JX137169
Araneae	Psechridae	*Fecenia ochracea*	GBCH10146-13	JX137157
Araneae	Psechridae	*Fecenia ochracea*	GBCH10147-13	JX137156
Araneae	Psechridae	*Fecenia travancoria*	GBCH10165-13	JX137138
Araneae	Psechridae	*Psechrus*	GBCH10102-13	JX137201
Araneae	Psechridae	*Psechrus*	GBCH10121-13	JX137182
Araneae	Psechridae	*Psechrus*	GBCH10122-13	JX137181
Araneae	Psechridae	*Psechrus*	GBCH10124-13	JX137179
Araneae	Psechridae	*Psechrus*	GBCH10136-13	JX137167
Araneae	Psechridae	*Psechrus*	GBCH10153-13	JX137150
Araneae	Psechridae	*Psechrus ancoralis*	GBCH10086-13	JX137217
Araneae	Psechridae	*Psechrus ancoralis*	GBCH10098-13	JX137205
Araneae	Psechridae	*Psechrus ancoralis*	GBCH10107-13	JX137196
Araneae	Psechridae	*Psechrus ancoralis*	GBCH10112-13	JX137191
Araneae	Psechridae	*Psechrus ancoralis*	GBCH10113-13	JX137190
Araneae	Psechridae	*Psechrus ancoralis*	GBCH10114-13	JX137189
Araneae	Psechridae	*Psechrus ancoralis*	GBCH10116-13	JX137187
Araneae	Psechridae	*Psechrus ancoralis*	GBCH10117-13	JX137186
Araneae	Psechridae	*Psechrus ancoralis*	GBCH10120-13	JX137183
Araneae	Psechridae	*Psechrus ancoralis*	GBCH10123-13	JX137180
Araneae	Psechridae	*Psechrus ancoralis*	GBCH10154-13	JX137149
Araneae	Psechridae	*Psechrus antraeus*	GBCH10082-13	JX137221
Araneae	Psechridae	*Psechrus antraeus*	GBCH10118-13	JX137185
Araneae	Psechridae	*Psechrus antraeus*	GBCH10119-13	JX137184
Araneae	Psechridae	*Psechrus ghecuanus*	GBCH10100-13	JX137203
Araneae	Psechridae	*Psechrus ghecuanus*	GBCH10138-13	JX137165
Araneae	Psechridae	*Psechrus ghecuanus*	GBCH10143-13	JX137160
Araneae	Psechridae	*Psechrus himalayanus*	GBCH10135-13	JX137168
Araneae	Psechridae	*Psechrus himalayanus*	GBCH10166-13	JX137137
Araneae	Psechridae	*Psechrus himalayanus*	GBCH10167-13	JX137136
Araneae	Psechridae	*Psechrus khammouan*	GBCH10125-13	JX137178
Araneae	Psechridae	*Psechrus khammouan*	GBCH10126-13	JX137177
Araneae	Psechridae	*Psechrus khammouan*	GBCH10127-13	JX137176
Araneae	Psechridae	*Psechrus luangprabang*	GBCH10092-13	JX137211
Araneae	Psechridae	*Psechrus luangprabang*	GBCH10097-13	JX137206
Araneae	Psechridae	*Psechrus luangprabang*	GBCH10099-13	JX137204
Araneae	Psechridae	*Psechrus luangprabang*	GBCH10101-13	JX137202
Araneae	Psechridae	*Psechrus luangprabang*	GBCH10103-13	JX137200
Araneae	Psechridae	*Psechrus luangprabang*	GBCH10104-13	JX137199
Araneae	Psechridae	*Psechrus luangprabang*	GBCH10106-13	JX137197
Araneae	Psechridae	*Psechrus luangprabang*	GBCH10115-13	JX137188
Araneae	Psechridae	*Psechrus rani*	GBCH10139-13	JX137164
Araneae	Psechridae	*Psechrus rani*	GBCH10140-13	JX137163
Araneae	Psechridae	*Psechrus senoculatus*	GBCH10155-13	JX137148
Araneae	Psechridae	*Psechrus senoculatus*	GBCH10156-13	JX137147
Araneae	Psechridae	*Psechrus sinensis*	GBCH10157-13	JX137146
Araneae	Psechridae	*Psechrus singaporensis*	GBCH10141-13	JX137162
Araneae	Psechridae	*Psechrus torvus*	GBCH10163-13	JX137140
Araneae	Psechridae		GBCH10083-13	JX137220
Araneae	Psechridae		GBCH10084-13	JX137219

Araneae	Psechridae		GBCH10087-13	JX137216
Araneae	Psechridae		GBCH10105-13	JX137198
Araneae	Psechridae		GBCH10108-13	JX137195
Araneae	Psechridae		GBCH10109-13	JX137194
Araneae	Psechridae		GBCH10111-13	JX137192
Araneae	Psechridae		GBCH10128-13	JX137175
Araneae	Psechridae		GBCH10129-13	JX137174
Araneae	Psechridae		GBCH10130-13	JX137173
Araneae	Psechridae		GBCH10131-13	JX137172
Araneae	Psechridae		GBCH10132-13	JX137171
Araneae	Psechridae		GBCH10142-13	JX137161
Araneae	Psechridae		GBCH10144-13	JX137159
Araneae	Psechridae		GBCH10145-13	JX137158
Araneae	Psechridae		GBCH10152-13	JX137151
Araneae	Psechridae		GBCH10158-13	JX137145
Araneae	Psechridae		GBCH10159-13	JX137144
Araneae	Psechridae		GBCH10160-13	JX137143
Araneae	Psechridae		GBCH10161-13	JX137142
Araneae	Psechridae		GBCH10162-13	JX137141
Araneae	Psechridae		GBCH10164-13	JX137139
Araneae	Psechridae		GBCH10168-13	JX137135
Araneae	Psechridae		GBCH10169-13	JX137134
Araneae	Psechridae		GBCH10170-13	JX137133
Araneae	Psechridae		GBCH10171-13	JX137132
Araneae	Salticidae	*Admestina sp. MCH-2001*	GBCH0218-06	AF327996
Araneae	Salticidae	*Admestina tibialis*	CNPPE2333-12	KJ165004
Araneae	Salticidae	*Admestina tibialis*	GMNCG467-12	KJ091626
Araneae	Salticidae	*Asianellus festivus*	GBCH11017-13	JN817254
Araneae	Salticidae	*Carrhotus xanthogramma*	GBCH11016-13	JN817255
Araneae	Salticidae	*Chalcoscirtus alpicola*	SPIRU1282-11	KF367929
Araneae	Salticidae	*Dendryphantes czekanowskii*	SPRMA374-10	HQ580921
Araneae	Salticidae	*Dendryphantes nigromaculatus*	SPRMA134-10	HQ580763
Araneae	Salticidae	*Dendryphantes nigromaculatus*	SPRMA859-12	KP649328
Araneae	Salticidae	*Dendryphantes nigromaculatus*	SPRMA860-12	KP652587
Araneae	Salticidae	*Dendryphantes sp. 1GAB*	RBINA3400-13	KP652400
Araneae	Salticidae	*Eris militaris*	ARONT349-09	GU682850
Araneae	Salticidae	*Eris militaris*	ARONT350-09	GU682851
Araneae	Salticidae	*Eris militaris*	ARONT791-10	HQ924637
Araneae	Salticidae	*Eris militaris*	CNSLQ086-13	KM837823
Araneae	Salticidae	*Eris militaris*	CNSLR086-13	KM835404
Araneae	Salticidae	*Eris militaris*	CNWBE286-13	KM826216
Araneae	Salticidae	*Eris militaris*	CNWBG1009-13	KM832460
Araneae	Salticidae	*Eris militaris*	CNWLG866-12	KM834791
Araneae	Salticidae	*Eris militaris*	JSARA028-11	KP656873
Araneae	Salticidae	*Eris militaris*	JSARA095-11	KP648032
Araneae	Salticidae	*Eris militaris*	JSARA099-11	KP647589
Araneae	Salticidae	*Eris militaris*	JSARA100-11	KP654587
Araneae	Salticidae	*Eris militaris*	RBCH066-04	DQ127466
Araneae	Salticidae	*Eris militaris*	RBCH070-04	DQ127454
Araneae	Salticidae	*Eris militaris*	RBINA1318-13	KP649099
Araneae	Salticidae	*Eris militaris*	RBINA1323-13	KP649522
Araneae	Salticidae	*Eris militaris*	RBINA1326-13	KP646811
Araneae	Salticidae	*Eris militaris*	RBINA1851-13	KP645846

Araneae	Salticidae	*Eris militaris*	RBINA1861-13	KP647880
Araneae	Salticidae	*Eris militaris*	RBINA3342-13	KP648854
Araneae	Salticidae	*Eris militaris*	RBINA3351-13	KP647097
Araneae	Salticidae	*Eris militaris*	SPRMA366-10	HQ580916
Araneae	Salticidae	*Euophrys monadnock*	CNGRD1299-12	KM839479
Araneae	Salticidae	*Euophrys monadnock*	CNGRD1300-12	KM824291
Araneae	Salticidae	*Euophrys monadnock*	SPRMA776-12	KP647526
Araneae	Salticidae	*Evarcha albaria*	GBCH11015-13	JN817256
Araneae	Salticidae	*Evarcha coreana*	GBCH11014-13	JN817257
Araneae	Salticidae	*Evarcha hoyi*	ARONT188-09	HM434057
Araneae	Salticidae	*Evarcha hoyi*	ARONT351-09	GU682853
Araneae	Salticidae	*Evarcha hoyi*	RBINA3380-13	KP649411
Araneae	Salticidae	*Evarcha hoyi*	RBINA3382-13	KP655968
Araneae	Salticidae	*Evarcha hoyi*	RBINA3963-13	KP647635
Araneae	Salticidae	*Evarcha hoyi*	SPRMA101-10	HQ580742
Araneae	Salticidae	*Evarcha hoyi*	SPRMA175-10	HQ580795
Araneae	Salticidae	*Evarcha hoyi*	SPRMA180-10	HQ580798
Araneae	Salticidae	*Evarcha hoyi*	SPRMA281-10	HQ580880
Araneae	Salticidae	*Evarcha hoyi*	SPRMA656-10	JF887110
Araneae	Salticidae	*Evarcha hoyi*	SPRMA657-10	JF887111
Araneae	Salticidae	*Evarcha hoyi*	SPRMA658-10	JF887112
Araneae	Salticidae	*Evarcha hoyi*	SSEIB11399-13	KM836492
Araneae	Salticidae	*Evarcha proszynskii*	SSBAF7690-13	KM828040
Araneae	Salticidae	*Evarcha proszynskii*	SSJAB2258-13	KM825486
Araneae	Salticidae	*Evarcha proszynskii*	SSJAB2259-13	KM824759
Araneae	Salticidae	*Evarcha proszynskii*	SSWLB275-13	KM832621
Araneae	Salticidae	*Evarcha proszynskii*	SSWLF1138-13	KM825898
Araneae	Salticidae	*Evarcha proszynskii*	SSWLF2911-13	KM826949
Araneae	Salticidae	*Evarcha proszynskii*	SSWLF4665-13	KM828573
Araneae	Salticidae	*Habronattus americanus*	SPRMA273-10	HQ580874
Araneae	Salticidae	*Habronattus decorus*	ERSPI313-09	GU682519
Araneae	Salticidae	*Habronattus decorus*	ERSPI346-09	GU682497
Araneae	Salticidae	*Habronattus decorus*	RBCH040-04	DQ127488
Araneae	Salticidae	*Habronattus decorus*	RBCH042-04	DQ127490
Araneae	Salticidae	*Habronattus decorus*	SMTPB2329-13	KP656032
Araneae	Salticidae	*Habronattus oregonensis*	GBCH0812-06	AY571145
Araneae	Salticidae	*Habronattus viridipes*	RBCH143-04	DQ127385
Araneae	Salticidae	*Hakka himeshimensis*	GBCH10993-13	JN817278
Araneae	Salticidae	*Heliophanus flavipes*	GACAC388-12	FR775770
Araneae	Salticidae	*Heliophanus ussuricus*	GBCH10994-13	JN817277
Araneae	Salticidae	*Hentzia mitrata*	CNPPD2701-12	KJ088232
Araneae	Salticidae	*Hentzia mitrata*	CNPPH1195-12	KJ444161
Araneae	Salticidae	*Hentzia mitrata*	CNSLP1064-13	KM831623
Araneae	Salticidae	*Hentzia mitrata*	RBCH189-04	DQ127335
Araneae	Salticidae	*Hentzia mitrata*	RBINA2836-13	KP650722
Araneae	Salticidae	*Hentzia mitrata*	RBINA2839-13	KP655118
Araneae	Salticidae	*Hentzia mitrata*	RBINA2844-13	KP653671
Araneae	Salticidae	*Hentzia mitrata*	RBINA2851-13	KP652204
Araneae	Salticidae	*Hentzia mitrata*	RBINA5304-13	KP654651
Araneae	Salticidae	*Maevia inclemens*	ARONT151-09	GU682638
Araneae	Salticidae	*Maevia inclemens*	SPRMA364-10	HQ580914
Araneae	Salticidae	*Marpissa formosa*	ARONT765-10	HQ924616
Araneae	Salticidae	*Marpissa milleri*	GBCH11008-13	JN817263

Araneae	Salticidae	*Mendoza canestrinii*	GBCH11005-13	JN817266
Araneae	Salticidae	*Mendoza canestrinii*	GBCH11006-13	JN817265
Araneae	Salticidae	*Mendoza canestrinii*	GBCH11007-13	JN817264
Araneae	Salticidae	*Menemerus fulvus*	GBCH11004-13	JN817267
Araneae	Salticidae	*Metaphidippus sp. 1GAB*	SPRMA183-10	HQ580800
Araneae	Salticidae	*Myrmarachne japonica*	GBCH10988-13	JN817283
Araneae	Salticidae	*Naphrys pulex*	ARONT068-09	GU682819
Araneae	Salticidae	*Naphrys pulex*	ARONT069-09	GU682817
Araneae	Salticidae	*Naphrys pulex*	ARONT070-09	GU682816
Araneae	Salticidae	*Naphrys pulex*	ARONT071-09	GU682836
Araneae	Salticidae	*Naphrys pulex*	ARONT072-09	GU682814
Araneae	Salticidae	*Naphrys pulex*	GBCH0541-06	AY297391
Araneae	Salticidae	*Naphrys pulex*	JSARA068-11	KP652349
Araneae	Salticidae	*Naphrys pulex*	RBCH091-04	DQ127443
Araneae	Salticidae	*Naphrys pulex*	RBCH095-04	DQ127431
Araneae	Salticidae	*Neon nelli*	GBCH0210-06	AF327988
Araneae	Salticidae	*Neon nelli*	RBINA1084-13	KP652381
Araneae	Salticidae	*Neon nelli*	RBINA5184-13	KP651232
Araneae	Salticidae	*Neon nelli*	SPRMA665-10	JF887113
Araneae	Salticidae	*Paraphidippus aurantius*	GBCH0237-06	AF328015
Araneae	Salticidae	*Paraphidippus aurantius*	GBCH4018-09	FJ607581
Araneae	Salticidae	*Pelegrina aeneola*	SPIAL021-10	HQ580533
Araneae	Salticidae	*Pelegrina aeneola*	SPIAL033-10	HQ580544
Araneae	Salticidae	*Pelegrina aeneola*	SPRMA977-12	KP651664
Araneae	Salticidae	*Pelegrina flaviceps*	SSJAF9138-13	KM834521
Araneae	Salticidae	*Pelegrina flaviceps*	SSPAA6455-13	KM834379
Araneae	Salticidae	*Pelegrina flaviceps*	SSPAA9248-13	KM827928
Araneae	Salticidae	*Pelegrina flavipes*	ARONT210-09	GU682659
Araneae	Salticidae	*Pelegrina flavipes*	SPRMA197-10	HQ580811
Araneae	Salticidae	*Pelegrina galathea*	ARONT234-09	GU682661
Araneae	Salticidae	*Pelegrina galathea*	ARONT235-09	GU682658
Araneae	Salticidae	*Pelegrina galathea*	ARONT721-10	HQ924576
Araneae	Salticidae	*Pelegrina galathea*	ARONT722-10	HQ924577
Araneae	Salticidae	*Pelegrina galathea*	ARONT783-10	HQ924631
Araneae	Salticidae	*Pelegrina galathea*	ARONT785-10	HQ924633
Araneae	Salticidae	*Pelegrina galathea*	ARONT788-10	HQ924635
Araneae	Salticidae	*Pelegrina galathea*	CNGRB568-12	KM832396
Araneae	Salticidae	*Pelegrina galathea*	ERSPI336-09	GU682504
Araneae	Salticidae	*Pelegrina galathea*	ERSPI401-09	GU682445
Araneae	Salticidae	*Pelegrina galathea*	RBINA1857-13	KP646546
Araneae	Salticidae	*Pelegrina galathea*	RBINA1859-13	KP652730
Araneae	Salticidae	*Pelegrina galathea*	RBINA1863-13	KP648422
Araneae	Salticidae	*Pelegrina galathea*	RBINA1875-13	KP656129
Araneae	Salticidae	*Pelegrina galathea*	RBINA3365-13	KP653926
Araneae	Salticidae	*Pelegrina galathea*	RBINA3383-13	KP649599
Araneae	Salticidae	*Pelegrina galathea*	RBINA3387-13	KP653621
Araneae	Salticidae	*Pelegrina galathea*	RBINA3420-13	KP654368
Araneae	Salticidae	*Pelegrina galathea*	RBINA3433-13	KP650810
Araneae	Salticidae	*Pelegrina galathea*	RBINA4880-13	KP653847
Araneae	Salticidae	*Pelegrina galathea*	RBINA4882-13	KP645675
Araneae	Salticidae	*Pelegrina galathea*	RBINA4883-13	KP649281
Araneae	Salticidae	*Pelegrina galathea*	RBINA4884-13	KP647520
Araneae	Salticidae	*Pelegrina galathea*	RBINA4892-13	KP650638

Araneae	Salticidae	*Pelegrina galathea*	RBINA4895-13	KP645779
Araneae	Salticidae	*Pelegrina galathea*	RBINA4896-13	KP657341
Araneae	Salticidae	*Pelegrina galathea*	RBINA4897-13	KP653343
Araneae	Salticidae	*Pelegrina galathea*	RBINA4904-13	KP646346
Araneae	Salticidae	*Pelegrina galathea*	RBINA5818-13	KP647180
Araneae	Salticidae	*Pelegrina galathea*	RBINA5824-13	KP653694
Araneae	Salticidae	*Pelegrina galathea*	RBINA5837-13	KR069963
Araneae	Salticidae	*Pelegrina galathea*	RBINA5839-13	KP653679
Araneae	Salticidae	*Pelegrina galathea*	RBINA777-13	KP656457
Araneae	Salticidae	*Pelegrina galathea*	RBINA779-13	KP653151
Araneae	Salticidae	*Pelegrina galathea*	RBINA781-13	KP649424
Araneae	Salticidae	*Pelegrina galathea*	RBINA787-13	KP655906
Araneae	Salticidae	*Pelegrina galathea*	RBINA788-13	KP651686
Araneae	Salticidae	*Pelegrina galathea*	RBINA790-13	KP645914
Araneae	Salticidae	*Pelegrina galathea*	RBINA792-13	KP653106
Araneae	Salticidae	*Pelegrina galathea*	RBINA793-13	KP648809
Araneae	Salticidae	*Pelegrina galathea*	RBINA797-13	KP654417
Araneae	Salticidae	*Pelegrina galathea*	RBINA798-13	KP653674
Araneae	Salticidae	*Pelegrina galathea*	RBINA800-13	KP653060
Araneae	Salticidae	*Pelegrina galathea*	RBINA801-13	KP650080
Araneae	Salticidae	*Pelegrina galathea*	RBINA802-13	KP650111
Araneae	Salticidae	*Pelegrina galathea*	RBINA806-13	KP646573
Araneae	Salticidae	*Pelegrina galathea*	RBINA807-13	KP646483
Araneae	Salticidae	*Pelegrina galathea*	RBINA811-13	KP646471
Araneae	Salticidae	*Pelegrina galathea*	RBINA814-13	KP652512
Araneae	Salticidae	*Pelegrina galathea*	RBINA816-13	KP656368
Araneae	Salticidae	*Pelegrina galathea*	RBINA818-13	KP652916
Araneae	Salticidae	*Pelegrina galathea*	RBINA820-13	KP646509
Araneae	Salticidae	*Pelegrina galathea*	RBINA823-13	KP648659
Araneae	Salticidae	*Pelegrina galathea*	RBINA825-13	KP649596
Araneae	Salticidae	*Pelegrina galathea*	RBINA826-13	KP651254
Araneae	Salticidae	*Pelegrina galathea*	RBINA827-13	KP647055
Araneae	Salticidae	*Pelegrina galathea*	RBINA828-13	KP655893
Araneae	Salticidae	*Pelegrina galathea*	RBINA831-13	KP648591
Araneae	Salticidae	*Pelegrina insignis*	ARONT720-10	HQ924575
Araneae	Salticidae	*Pelegrina insignis*	ARONT760-10	HQ924612
Araneae	Salticidae	*Pelegrina insignis*	ARONT764-10	HQ924615
Araneae	Salticidae	*Pelegrina insignis*	ARONT784-10	HQ924632
Araneae	Salticidae	*Pelegrina insignis*	ERSPI301-09	KP650871
Araneae	Salticidae	*Pelegrina insignis*	ERSPI302-09	HQ947607
Araneae	Salticidae	*Pelegrina insignis*	ERSPI303-09	GU682523
Araneae	Salticidae	*Pelegrina insignis*	ERSPI304-09	GU682524
Araneae	Salticidae	*Pelegrina insignis*	ERSPI305-09	GU682525
Araneae	Salticidae	*Pelegrina insignis*	ERSPI382-09	KP653940
Araneae	Salticidae	*Pelegrina insignis*	ERSPI400-09	GU682455
Araneae	Salticidae	*Pelegrina montana*	CNPAB300-13	KM839306
Araneae	Salticidae	*Pelegrina montana*	CNPAB301-13	KM831417
Araneae	Salticidae	*Pelegrina montana*	CNRMC1574-12	KM836993
Araneae	Salticidae	*Pelegrina proterva*	ARONT397-09	GU682894
Araneae	Salticidae	*Pelegrina proterva*	CNSLG291-12	KM827158
Araneae	Salticidae	*Pelegrina proterva*	CNSLI109-12	KM826033
Araneae	Salticidae	*Pelegrina proterva*	CNSLK009-12	KM833528
Araneae	Salticidae	*Pelegrina proterva*	PHSEP1957-11	KP650985

Araneae	Salticidae	*Pelegrina proterva*	RBCH133-04	DQ127391
Araneae	Salticidae	*Pelegrina proterva*	RBINA1029-13	KP649848
Araneae	Salticidae	*Pelegrina proterva*	RBINA1222-13	KP645984
Araneae	Salticidae	*Pelegrina proterva*	RBINA1223-13	KP657437
Araneae	Salticidae	*Pelegrina proterva*	RBINA2664-13	KP650902
Araneae	Salticidae	*Pelegrina proterva*	RBINA2802-13	KP649076
Araneae	Salticidae	*Pelegrina proterva*	RBINA2803-13	KP655722
Araneae	Salticidae	*Pelegrina proterva*	RBINA2809-13	KP651479
Araneae	Salticidae	*Pelegrina proterva*	RBINA2811-13	KP655665
Araneae	Salticidae	*Pelegrina proterva*	RBINA2814-13	KP652036
Araneae	Salticidae	*Pelegrina proterva*	RBINA2840-13	KP655043
Araneae	Salticidae	*Pelegrina proterva*	RBINA3307-13	KP647516
Araneae	Salticidae	*Pelegrina proterva*	RBINA3340-13	KP651189
Araneae	Salticidae	*Pelegrina proterva*	RBINA3372-13	KP656010
Araneae	Salticidae	*Pelegrina proterva*	RBINA3395-13	KP653876
Araneae	Salticidae	*Pellenes ignifrons*	SPRMA782-12	KP645944
Araneae	Salticidae	*Pellenes lapponicus*	RBCH016-04	DQ127512
Araneae	Salticidae	*Pellenes lapponicus*	SPISH009-09	GU683925
Araneae	Salticidae	*Phanias albeolus*	SPRMA315-10	HQ977181
Araneae	Salticidae	*Phanias albeolus*	SPRMA674-10	JF887117
Araneae	Salticidae	*Phidippus audax*	ARONT002-09	GU682795
Araneae	Salticidae	*Phidippus audax*	ARONT023-09	GU682792
Araneae	Salticidae	*Phidippus audax*	ARONT025-09	GU682793
Araneae	Salticidae	*Phidippus audax*	ARONT026-09	GU682791
Araneae	Salticidae	*Phidippus audax*	ARONT092-09	GU682790
Araneae	Salticidae	*Phidippus audax*	ARONT137-09	GU682637
Araneae	Salticidae	*Phidippus audax*	ARONT300-09	GU682852
Araneae	Salticidae	*Phidippus audax*	ARONT621-10	HQ924486
Araneae	Salticidae	*Phidippus audax*	ARONT732-10	HQ924586
Araneae	Salticidae	*Phidippus audax*	ERSPI375-09	GU682466
Araneae	Salticidae	*Phidippus audax*	RBCH038-04	DQ127486
Araneae	Salticidae	*Phidippus audax*	RBCH160-04	DQ127370
Araneae	Salticidae	*Phidippus audax*	RBCH179-04	DQ127357
Araneae	Salticidae	*Phidippus audax*	RBCH195-04	DQ127341
Araneae	Salticidae	*Phidippus audax*	RBCH199-04	DQ127329
Araneae	Salticidae	*Phidippus audax*	RBINA3279-13	KP652057
Araneae	Salticidae	*Phidippus audax*	RBINA3281-13	KP650814
Araneae	Salticidae	*Phidippus audax*	RBINA3286-13	KP648263
Araneae	Salticidae	*Phidippus audax*	RBINA3287-13	KP645439
Araneae	Salticidae	*Phidippus audax*	RBINA3364-13	KP645633
Araneae	Salticidae	*Phidippus audax*	RBINA3407-13	KP654525
Araneae	Salticidae	*Phidippus audax*	RBINA3409-13	KP649102
Araneae	Salticidae	*Phidippus audax*	RBINA3416-13	KP652871
Araneae	Salticidae	*Phidippus audax*	RBINA5808-13	KP656024
Araneae	Salticidae	*Phidippus audax*	RBINA720-13	KP650333
Araneae	Salticidae	*Phidippus audax*	RBINA821-13	KP654213
Araneae	Salticidae	*Phidippus borealis*	CNRMC1572-12	KM840286
Araneae	Salticidae	*Phidippus borealis*	CNRMD2873-13	KM825265
Araneae	Salticidae	*Phidippus borealis*	CNWBE385-13	KM832876
Araneae	Salticidae	*Phidippus borealis*	SPRMA666-10	KP656440
Araneae	Salticidae	*Phidippus borealis*	SPRMA667-10	JF887114
Araneae	Salticidae	*Phidippus clarus*	ARONT704-10	HQ924562
Araneae	Salticidae	*Phidippus clarus*	ARONT705-10	KP655218

Araneae	Salticidae	*Phidippus clarus*	ARONT723-10	HQ924578
Araneae	Salticidae	*Phidippus clarus*	ARONT736-10	HQ924590
Araneae	Salticidae	*Phidippus clarus*	ARONT739-10	HQ924592
Araneae	Salticidae	*Phidippus clarus*	ARONT766-10	HQ924617
Araneae	Salticidae	*Phidippus clarus*	ARONT767-10	HQ924618
Araneae	Salticidae	*Phidippus clarus*	ARONT790-10	HQ924636
Araneae	Salticidae	*Phidippus clarus*	ARONT792-10	HQ924638
Araneae	Salticidae	*Phidippus clarus*	ARONT793-10	HQ924639
Araneae	Salticidae	*Phidippus clarus*	ARONT794-10	HQ924640
Araneae	Salticidae	*Phidippus clarus*	ARONT815-10	HQ924660
Araneae	Salticidae	*Phidippus clarus*	ERSPI385-09	GU682463
Araneae	Salticidae	*Phidippus clarus*	ERSPI386-09	GU682464
Araneae	Salticidae	*Phidippus clarus*	ERSPI388-09	GU682465
Araneae	Salticidae	*Phidippus clarus*	ERSPI389-09	GU682456
Araneae	Salticidae	*Phidippus clarus*	ERSPI408-09	GU682440
Araneae	Salticidae	*Phidippus clarus*	JSARA078-11	KP654962
Araneae	Salticidae	*Phidippus clarus*	RBCH105-04	DQ127425
Araneae	Salticidae	*Phidippus clarus*	RBCH111-04	DQ127415
Araneae	Salticidae	*Phidippus clarus*	RBINA1844-13	KP650575
Araneae	Salticidae	*Phidippus clarus*	RBINA1846-13	KP657385
Araneae	Salticidae	*Phidippus clarus*	RBINA1856-13	KP653426
Araneae	Salticidae	*Phidippus johnsoni*	SPRMA668-10	KP654103
Araneae	Salticidae	*Phidippus johnsoni*	SPRMA669-10	KP653906
Araneae	Salticidae	*Phidippus johnsoni*	SPRMA670-10	JF887115
Araneae	Salticidae	*Phidippus princeps*	ARONT383-09	HM434064
Araneae	Salticidae	*Phidippus princeps*	JSARA071-11	KP655376
Araneae	Salticidae	*Phidippus purpuratus*	ARONT022-09	GU682794
Araneae	Salticidae	*Phidippus purpuratus*	RBCH120-04	DQ127408
Araneae	Salticidae	*Phintella abnormis*	GBCH10987-13	JN817284
Araneae	Salticidae	*Phintella arenicolor*	GBCH10986-13	JN817285
Araneae	Salticidae	*Platycryptus californicus*	SPRMA671-10	JF887116
Araneae	Salticidae	*Platycryptus undatus*	ARONT039-09	GU682796
Araneae	Salticidae	*Platycryptus undatus*	ARONT058-09	GU682797
Araneae	Salticidae	*Platycryptus undatus*	ARONT152-09	GU682635
Araneae	Salticidae	*Platycryptus undatus*	ARONT358-09	GU682890
Araneae	Salticidae	*Platycryptus undatus*	RBCH146-04	DQ127388
Araneae	Salticidae	*Platycryptus undatus*	RBCH200-04	DQ127330
Araneae	Salticidae	*Plexippoides annulipedis*	GBCH11012-13	JN817259
Araneae	Salticidae	*Plexippoides regius*	GBCH11011-13	JN817260
Araneae	Salticidae	*Plexippus paykulli*	GBCH11010-13	JN817261
Araneae	Salticidae	*Pseudeuophrys iwatensis*	GBCH11000-13	JN817271
Araneae	Salticidae	*Pseudicius vulpes*	GBCH10992-13	JN817279
Araneae	Salticidae	*Rhene atrata*	GBCH11003-13	JN817268
Araneae	Salticidae	*Salticus scenicus*	ARONT007-09	GU682812
Araneae	Salticidae	*Salticus scenicus*	ARONT008-09	HM880608
Araneae	Salticidae	*Salticus scenicus*	ARONT009-09	GU682804
Araneae	Salticidae	*Salticus scenicus*	JSARA065-11	KP651589
Araneae	Salticidae	*Salticus scenicus*	RBCH009-04	DQ127521
Araneae	Salticidae	*Salticus scenicus*	SMTPB2332-13	KP657103
Araneae	Salticidae	*Salticus scenicus*	SPRMA673-10	KP647174
Araneae	Salticidae	*Salticus scenicus*	SPRMA754-10	JF887168
Araneae	Salticidae	*Sibianor pullus*	GBCH11002-13	JN817269
Araneae	Salticidae	*Siler cupreus*	GBCH11001-13	JN817270

Araneae	Salticidae	*Sitticus albolineatus*	GBCH10999-13	JN817272
Araneae	Salticidae	*Sitticus ammophilus*	ARONT179-09	HM434055
Araneae	Salticidae	*Sitticus concolor*	CNPPE2332-12	KJ166443
Araneae	Salticidae	*Sitticus finschi*	SPIRU1078-11	KF368783
Araneae	Salticidae	*Sitticus floricola palustris*	ARONT735-10	HQ924589
Araneae	Salticidae	*Sitticus floricola palustris*	ARONT762-10	HQ924614
Araneae	Salticidae	*Sitticus floricola palustris*	ERSPI394-09	GU682461
Araneae	Salticidae	*Sitticus floricola palustris*	SPICH824-09	GU684425
Araneae	Salticidae	*Sitticus floricola palustris*	SPIRU1012-11	KF368788
Araneae	Salticidae	*Sitticus floricola palustris*	SPRMA196-10	HQ580810
Araneae	Salticidae	*Sitticus floricola palustris*	SPRMA693-10	JF887127
Araneae	Salticidae	*Sitticus penicillatus*	GBCH10996-13	JN817275
Araneae	Salticidae	*Sitticus ranieri*	KKCHE945-09	GU683651
Araneae	Salticidae	*Sitticus striatus*	SPIRU1159-11	KF368804
Araneae	Salticidae	*Synageles occidentalis*	CNWBE379-13	KM839132
Araneae	Salticidae	*Synagelides agoriformis*	GBCH10989-13	JN817282
Araneae	Salticidae	*Synemosyna formica*	SPRMA675-10	JF887118
Araneae	Salticidae	*Talavera minuta*	ARONT803-10	HQ924648
Araneae	Salticidae	*Telamonia vlijmi*	GBCH10990-13	JN817281
Araneae	Salticidae	*Terralonus sp. 1GAB*	CNPPF1456-12	KJ166721
Araneae	Salticidae	*Trite planiceps*	GBCH7197-13	JF836863
Araneae	Salticidae	*Trite planiceps*	GBCH7198-13	JF836862
Araneae	Salticidae	*Trite planiceps*	GBCH7199-13	JF836861
Araneae	Salticidae	*Trite planiceps*	GBCH7200-13	JF836860
Araneae	Salticidae	*Trite planiceps*	GBCH7201-13	JF836859
Araneae	Salticidae	*Trite planiceps*	GBCH7202-13	JF836858
Araneae	Salticidae	*Trite planiceps*	GBCH7203-13	JF836857
Araneae	Salticidae	*Trite planiceps*	GBCH7204-13	JF836856
Araneae	Salticidae	*Trite planiceps*	GBCH7205-13	JF836855
Araneae	Salticidae	*Trite planiceps*	GBCH7206-13	JF836854
Araneae	Salticidae	*Trite planiceps*	GBCH7207-13	JF836853
Araneae	Salticidae	*Trite planiceps*	GBCH7208-13	JF836852
Araneae	Salticidae	*Tutelina harti*	GBCH0235-06	AF328013
Araneae	Salticidae	*Tutelina harti*	JSARA019-11	KP652795
Araneae	Salticidae	*Tutelina similis*	ARONT761-10	HQ924613
Araneae	Salticidae	*Tutelina similis*	ARONT787-10	HQ924634
Araneae	Salticidae	*Tutelina similis*	CNPPD2700-12	KJ089887
Araneae	Salticidae	*Tutelina similis*	CNPPJ1372-12	KJ208205
Araneae	Salticidae	*Tutelina similis*	JSARA056-11	KP646538
Araneae	Salticidae	*Tutelina similis*	JSARA079-11	KP646788
Araneae	Salticidae	*Tutelina similis*	JSARA087-11	KP649888
Araneae	Salticidae	*Tutelina similis*	JSARA093-11	KP656863
Araneae	Salticidae	*Tutelina similis*	JSARA094-11	KP648615
Araneae	Salticidae	*Tutelina similis*	RBINA3780-13	KP650819
Araneae	Salticidae	*Tutelina similis*	RBINA5803-13	KP649233
Araneae	Salticidae	*Tutelina sp. 1GAB*	RBINA5809-13	KP647738
Araneae	Scytodidae	*Scytodes thoracica*	ARONT183-09	HM434056
Araneae	Segestriidae	*Segestria bavarica*	GBCH0811-06	AY560802
Araneae	Segestriidae	*Segestria pacifica*	CNGID206-12	KM827799
Araneae	Segestriidae	*Segestria pacifica*	SPRMA006-10	HQ977117
Araneae	Segestriidae	*Segestria pacifica*	SPRMA346-10	HQ580904
Araneae	Sicariidae	*Loxosceles sp. RPD-2010*	GACAC391-12	GQ279111
Araneae	Sicariidae	*Loxosceles sp. RPD-2010*	GACAC398-12	GQ279217

Araneae	Sicariidae	*Loxosceles sp. RPD-2010*	GACAC402-12	GQ279221
Araneae	Sicariidae	*Loxosceles sp. RPD-2010*	GACAC414-12	GQ279233
Araneae	Sicariidae	*Loxosceles sp. RPD-2010*	GACAC416-12	GQ279235
Araneae	Sicariidae	*Loxosceles sp. RPD-2010*	GACAC417-12	GQ279236
Araneae	Sicariidae	*Loxosceles sp. RPD-2010*	GACAC418-12	GQ279237
Araneae	Sparassidae	*Damastes sp. SB-2009*	GBCH5137-10	GQ855822
Araneae	Sparassidae	*Heteropoda aemulans*	GBCH5134-10	GQ855825
Araneae	Sparassidae	*Heteropoda aemulans*	GBCH5144-10	GQ855815
Araneae	Sparassidae	*Heteropoda aemulans*	GBCH5145-10	GQ855814
Araneae	Sparassidae	*Heteropoda dagmarae*	GBCH5146-10	GQ855813
Araneae	Sparassidae	*Heteropoda maxima*	GBCH5147-10	GQ855812
Araneae	Sparassidae	*Heteropoda maxima*	GBCH5149-10	GQ855810
Araneae	Sparassidae	*Heteropoda maxima*	GBCH5150-10	GQ855809
Araneae	Sparassidae	*Heteropoda maxima*	GBCH5152-10	GQ855807
Araneae	Sparassidae	*Heteropoda simplex*	GBCH5131-10	GQ855828
Araneae	Sparassidae	*Heteropoda simplex*	GBCH5133-10	GQ855826
Araneae	Sparassidae	*Heteropoda simplex*	GBCH5135-10	GQ855824
Araneae	Sparassidae	*Heteropoda simplex*	GBCH5136-10	GQ855823
Araneae	Sparassidae	*Heteropoda simplex*	GBCH5140-10	GQ855819
Araneae	Sparassidae	*Heteropoda simplex*	GBCH5143-10	GQ855816
Araneae	Sparassidae	*Heteropoda steineri*	GBCH5132-10	GQ855827
Araneae	Sparassidae	*Heteropoda tetrica*	GBCH5148-10	GQ855811
Araneae	Sparassidae	*Heteropoda venatoria*	GBCH5141-10	GQ855818
Araneae	Sparassidae	*Heteropoda venatoria*	GBCH5142-10	GQ855817
Araneae	Sparassidae	*Pseudopoda namkhan*	GBCH5138-10	GQ855821
Araneae	Sparassidae	*Sinopoda koreana*	GBCH11039-13	JN817232
Araneae	Sparassidae	*Sinopoda sp. SB-2009*	GBCH5151-10	GQ855808
Araneae	Sparassidae	*Sinopoda stellatops*	GBCH11038-13	JN817233
Araneae	Stenochilidae	*Colopea*	GBCH11421-13	JX240236
Araneae	Stiphidiidae	*Stiphidion facetum*	GBCH10137-13	JX137166
Araneae	Symphytognathidae		GBCH8335-13	GU456913
Araneae	Symphytognathidae		GBCH8336-13	GU456912
Araneae	Symphytognathidae		GBCH8337-13	GU456911
Araneae	Symphytognathidae		GBCH8338-13	GU456910
Araneae	Symphytognathidae		GBCH8339-13	GU456909
Araneae	Tetragnathidae	*Azilia guatemalensis*	GBCH3913-09	EU003280
Araneae	Tetragnathidae	*Cyrtognatha espanola*	GBCH3910-09	EU003283
Araneae	Tetragnathidae	*Dolichognatha sp. FAPDNA061*	GBCH3908-09	EU003285
Araneae	Tetragnathidae	*Glenognatha foxi*	ARONT263-09	HM434061
Araneae	Tetragnathidae	*Glenognatha foxi*	RBINA530-13	KP645603
Araneae	Tetragnathidae	*Leucauge*	GBCH11140-13	JN817130
Araneae	Tetragnathidae	*Leucauge*	GBCH7174-13	HQ441946
Araneae	Tetragnathidae	*Leucauge argyra*	GBCH3903-09	EU003290
Araneae	Tetragnathidae	*Leucauge blanda*	GBCH11141-13	JN817129
Araneae	Tetragnathidae	*Leucauge celebesiana*	GBCH11139-13	JN817131
Araneae	Tetragnathidae	*Leucauge venusta*	ARONT056-09	GU682835
Araneae	Tetragnathidae	*Leucauge venusta*	ARONT081-09	GU682832
Araneae	Tetragnathidae	*Leucauge venusta*	ARONT205-09	GU682695
Araneae	Tetragnathidae	*Leucauge venusta*	GBCH3241-09	FJ525323
Araneae	Tetragnathidae	*Leucauge venusta*	GBCH3902-09	EU003291
Araneae	Tetragnathidae	*Leucauge venusta*	GBCH4031-09	FJ607568
Araneae	Tetragnathidae	*Leucauge venusta*	RBCH071-04	DQ127455
Araneae	Tetragnathidae	*Leucauge venusta*	RBCH165-04	DQ127359

Araneae	Tetragnathidae	*Leucauge venusta*	RBCH180-04	DQ127358
Araneae	Tetragnathidae	*Menosira ornata*	GBCH11138-13	JN817132
Araneae	Tetragnathidae	*Meta manchurica*	GBCH11137-13	JN817133
Araneae	Tetragnathidae	*Meta menardi*	GBCH3898-09	EU003295
Araneae	Tetragnathidae	*Meta ovalis*	ARONT454-09	GU682955
Araneae	Tetragnathidae	*Meta ovalis*	GBCH4028-09	FJ607571
Araneae	Tetragnathidae	*Metabus ebanoverde*	GBCH3897-09	EU003296
Araneae	Tetragnathidae	*Metellina curtisi*	CNPCA032-12	KM830955
Araneae	Tetragnathidae	*Metellina curtisi*	CNPCS004-13	KM830247
Araneae	Tetragnathidae	*Metellina curtisi*	SPIAL005-10	HQ580519
Araneae	Tetragnathidae	*Metellina curtisi*	SPIAL076-10	HQ580580
Araneae	Tetragnathidae	*Metellina curtisi*	SPIAL087-10	HQ580588
Araneae	Tetragnathidae	*Metellina curtisi*	SPIAL088-10	HQ580589
Araneae	Tetragnathidae	*Metellina curtisi*	SPRMA027-10	HQ977130
Araneae	Tetragnathidae	*Metellina mengei*	GBCH5715-13	FJ899807
Araneae	Tetragnathidae	*Metellina segmentata*	CNGIG017-12	KM824342
Araneae	Tetragnathidae	*Metellina segmentata*	GBCH4027-09	FJ607572
Araneae	Tetragnathidae	*Metellina segmentata*	SPRMA688-10	KP651521
Araneae	Tetragnathidae	*Metleucauge yunohamensis*	GBCH11136-13	JN817134
Araneae	Tetragnathidae	*Opadometa sp. FAPDNA047*	GBCH3890-09	EU003304
Araneae	Tetragnathidae	*Orsinome sp. FAPDNA052*	GBCH3889-09	EU003305
Araneae	Tetragnathidae	*Pachygnatha autumnalis*	ARONT499-10	HQ924394
Araneae	Tetragnathidae	*Pachygnatha autumnalis*	ARONT796-10	HQ924642
Araneae	Tetragnathidae	*Pachygnatha autumnalis*	ARONT797-10	HQ924643
Araneae	Tetragnathidae	*Pachygnatha autumnalis*	ERSPI246-09	HM376096
Araneae	Tetragnathidae	*Pachygnatha autumnalis*	RBINA3291-13	KP653268
Araneae	Tetragnathidae	*Pachygnatha autumnalis*	RBINA3404-13	KP646770
Araneae	Tetragnathidae	*Pachygnatha autumnalis*	RBINA3417-13	KP653745
Araneae	Tetragnathidae	*Pachygnatha autumnalis*	RBINA3504-13	KP655350
Araneae	Tetragnathidae	*Pachygnatha autumnalis*	RBINA3574-13	KP650594
Araneae	Tetragnathidae	*Pachygnatha autumnalis*	RBINA3981-13	KP654356
Araneae	Tetragnathidae	*Pachygnatha clercki*	GBCH11135-13	JN817135
Araneae	Tetragnathidae	*Pachygnatha clercki*	GBCH5707-13	FJ899816
Araneae	Tetragnathidae	*Pachygnatha clercki*	GBCH5708-13	FJ899815
Araneae	Tetragnathidae	*Pachygnatha clercki*	GBCH5709-13	FJ899814
Araneae	Tetragnathidae	*Pachygnatha clercki*	SPICH1078-09	GU684581
Araneae	Tetragnathidae	*Pachygnatha clercki*	SSPAB8079-13	KM831461
Araneae	Tetragnathidae	*Pachygnatha degeeri*	GBCH3888-09	EU003306
Araneae	Tetragnathidae	*Pachygnatha degeeri*	GBCH5704-13	FJ899819
Araneae	Tetragnathidae	*Pachygnatha degeeri*	GBCH5705-13	FJ899818
Araneae	Tetragnathidae	*Pachygnatha degeeri*	GBCH5706-13	FJ899817
Araneae	Tetragnathidae	*Pachygnatha dorothea*	ARONT236-09	GU682723
Araneae	Tetragnathidae	*Pachygnatha dorothea*	ARONT277-09	GU682724
Araneae	Tetragnathidae	*Pachygnatha dorothea*	ARONT278-09	GU682721
Araneae	Tetragnathidae	*Pachygnatha dorothea*	ARONT500-10	HQ924395
Araneae	Tetragnathidae	*Pachygnatha dorothea*	ARONT568-10	HQ924454
Araneae	Tetragnathidae	*Pachygnatha dorothea*	ARONT569-10	HQ924455
Araneae	Tetragnathidae	*Pachygnatha dorothea*	ARONT570-10	HQ924456
Araneae	Tetragnathidae	*Pachygnatha dorothea*	ARONT571-10	HQ924457
Araneae	Tetragnathidae	*Pachygnatha dorothea*	RBINA112-13	KP654065
Araneae	Tetragnathidae	*Pachygnatha dorothea*	SPRMA677-10	KP657054
Araneae	Tetragnathidae	*Pachygnatha tristriata*	ARONT166-09	GU682653
Araneae	Tetragnathidae	*Pachygnatha tristriata*	ARONT167-09	GU682654

Araneae	Tetragnathidae	*Pachygnatha tristriata*	ARONT429-09	GU682940
Araneae	Tetragnathidae	*Pachygnatha tristriata*	ARONT521-10	HQ924410
Araneae	Tetragnathidae	*Pachygnatha tristriata*	ARONT700-10	HQ924558
Araneae	Tetragnathidae	*Pachygnatha xanthostoma*	RBINA2246-13	KP653681
Araneae	Tetragnathidae	*sp. FAPDNA067*	GBCH4335-09	EU003299
Araneae	Tetragnathidae	*Tetragnatha*	GBCH5699-13	FJ899828
Araneae	Tetragnathidae	*Tetragnatha anuenue*	GBCH0752-06	AY530502
Araneae	Tetragnathidae	*Tetragnatha anuenue*	GBCH0753-06	AY530503
Araneae	Tetragnathidae	*Tetragnatha anuenue*	GBCH0754-06	AY530504
Araneae	Tetragnathidae	*Tetragnatha anuenue*	GBCH0755-06	AY530505
Araneae	Tetragnathidae	*Tetragnatha anuenue*	GBCH0756-06	AY530506
Araneae	Tetragnathidae	*Tetragnatha anuenue*	GBCH0757-06	AY530507
Araneae	Tetragnathidae	*Tetragnatha anuenue*	GBCH0758-06	AY530508
Araneae	Tetragnathidae	*Tetragnatha anuenue*	GBCH0759-06	AY530509
Araneae	Tetragnathidae	*Tetragnatha anuenue*	GBCH0760-06	AY530510
Araneae	Tetragnathidae	*Tetragnatha anuenue*	GBCH0761-06	AY530511
Araneae	Tetragnathidae	*Tetragnatha anuenue*	GBCH0762-06	AY530512
Araneae	Tetragnathidae	*Tetragnatha anuenue*	GBCH0763-06	AY530513
Araneae	Tetragnathidae	*Tetragnatha anuenue*	GBCH0764-06	AY530514
Araneae	Tetragnathidae	*Tetragnatha anuenue*	GBCH0765-06	AY530515
Araneae	Tetragnathidae	*Tetragnatha anuenue*	GBCH0766-06	AY530516
Araneae	Tetragnathidae	*Tetragnatha anuenue*	GBCH0767-06	AY530517
Araneae	Tetragnathidae	*Tetragnatha anuenue*	GBCH0768-06	AY530518
Araneae	Tetragnathidae	*Tetragnatha anuenue*	GBCH0769-06	AY530519
Araneae	Tetragnathidae	*Tetragnatha anuenue*	GBCH0770-06	AY530520
Araneae	Tetragnathidae	*Tetragnatha anuenue*	GBCH0771-06	AY530521
Araneae	Tetragnathidae	*Tetragnatha anuenue*	GBCH0772-06	AY530522
Araneae	Tetragnathidae	*Tetragnatha anuenue*	GBCH0773-06	AY530523
Araneae	Tetragnathidae	*Tetragnatha anuenue*	GBCH0774-06	AY530524
Araneae	Tetragnathidae	*Tetragnatha anuenue*	GBCH0775-06	AY530525
Araneae	Tetragnathidae	*Tetragnatha anuenue*	GBCH0776-06	AY530526
Araneae	Tetragnathidae	*Tetragnatha anuenue*	GBCH0777-06	AY530527
Araneae	Tetragnathidae	*Tetragnatha anuenue*	GBCH0778-06	AY530528
Araneae	Tetragnathidae	*Tetragnatha anuenue*	GBCH0779-06	AY530529
Araneae	Tetragnathidae	*Tetragnatha anuenue*	GBCH0780-06	AY530530
Araneae	Tetragnathidae	*Tetragnatha anuenue*	GBCH0781-06	AY530531
Araneae	Tetragnathidae	*Tetragnatha brevignatha*	GBCH0725-06	AY530475
Araneae	Tetragnathidae	*Tetragnatha brevignatha*	GBCH0726-06	AY530476
Araneae	Tetragnathidae	*Tetragnatha brevignatha*	GBCH0727-06	AY530477
Araneae	Tetragnathidae	*Tetragnatha brevignatha*	GBCH0728-06	AY530478
Araneae	Tetragnathidae	*Tetragnatha brevignatha*	GBCH0729-06	AY530479
Araneae	Tetragnathidae	*Tetragnatha brevignatha*	GBCH0730-06	AY530480
Araneae	Tetragnathidae	*Tetragnatha brevignatha*	GBCH0731-06	AY530481
Araneae	Tetragnathidae	*Tetragnatha brevignatha*	GBCH0732-06	AY530482
Araneae	Tetragnathidae	*Tetragnatha brevignatha*	GBCH0733-06	AY530483
Araneae	Tetragnathidae	*Tetragnatha brevignatha*	GBCH0734-06	AY530484
Araneae	Tetragnathidae	*Tetragnatha brevignatha*	GBCH0735-06	AY530485
Araneae	Tetragnathidae	*Tetragnatha brevignatha*	GBCH0736-06	AY530486
Araneae	Tetragnathidae	*Tetragnatha brevignatha*	GBCH0737-06	AY530487
Araneae	Tetragnathidae	*Tetragnatha brevignatha*	GBCH0738-06	AY530488
Araneae	Tetragnathidae	*Tetragnatha brevignatha*	GBCH0739-06	AY530489
Araneae	Tetragnathidae	*Tetragnatha brevignatha*	GBCH0740-06	AY530490
Araneae	Tetragnathidae	*Tetragnatha brevignatha*	GBCH0741-06	AY530491

Araneae	Tetragnathidae	*Tetragnatha brevignatha*	GBCH0742-06	AY530492
Araneae	Tetragnathidae	*Tetragnatha brevignatha*	GBCH0743-06	AY530493
Araneae	Tetragnathidae	*Tetragnatha brevignatha*	GBCH0744-06	AY530494
Araneae	Tetragnathidae	*Tetragnatha brevignatha*	GBCH0745-06	AY530495
Araneae	Tetragnathidae	*Tetragnatha brevignatha*	GBCH0746-06	AY530496
Araneae	Tetragnathidae	*Tetragnatha brevignatha*	GBCH0747-06	AY530497
Araneae	Tetragnathidae	*Tetragnatha brevignatha*	GBCH0748-06	AY530498
Araneae	Tetragnathidae	*Tetragnatha brevignatha*	GBCH0749-06	AY530499
Araneae	Tetragnathidae	*Tetragnatha brevignatha*	GBCH0750-06	AY530500
Araneae	Tetragnathidae	*Tetragnatha brevignatha*	GBCH0751-06	AY530501
Araneae	Tetragnathidae	*Tetragnatha brevignatha*	GBCH1305-06	DQ178959
Araneae	Tetragnathidae	*Tetragnatha brevignatha*	GBCH1306-06	DQ178961
Araneae	Tetragnathidae	*Tetragnatha brevignatha*	GBCH1314-06	DQ182756
Araneae	Tetragnathidae	*Tetragnatha brevignatha*	GBCH1315-06	DQ182757
Araneae	Tetragnathidae	*Tetragnatha caudata*	ARSO229-08	KP654982
Araneae	Tetragnathidae	*Tetragnatha caudata*	RBINA116-13	KP654977
Araneae	Tetragnathidae	*Tetragnatha caudata*	RBINA238-13	KP657466
Araneae	Tetragnathidae	*Tetragnatha caudata*	SSPAB9958-13	KM825786
Araneae	Tetragnathidae	*Tetragnatha caudata*	TDWGB921-10	HQ979344
Araneae	Tetragnathidae	*Tetragnatha dearmata*	RBINA114-13	KP649057
Araneae	Tetragnathidae	*Tetragnatha dearmata*	RBINA115-13	KP657453
Araneae	Tetragnathidae	*Tetragnatha dearmata*	RBINA118-13	KP657161
Araneae	Tetragnathidae	*Tetragnatha elongata*	ARONT073-09	GU682831
Araneae	Tetragnathidae	*Tetragnatha elongata*	ARONT295-09	GU682889
Araneae	Tetragnathidae	*Tetragnatha elongata*	ARONT338-09	GU682886
Araneae	Tetragnathidae	*Tetragnatha elongata*	ERSPI314-09	GU682520
Araneae	Tetragnathidae	*Tetragnatha elongata*	RBCH048-04	DQ127480
Araneae	Tetragnathidae	*Tetragnatha elongata*	RBCH142-04	DQ127384
Araneae	Tetragnathidae	*Tetragnatha elongata*	RBCH167-04	DQ127361
Araneae	Tetragnathidae	*Tetragnatha elongata*	RBINA071-13	KP654318
Araneae	Tetragnathidae	*Tetragnatha elongata*	RBINA2247-13	KP649935
Araneae	Tetragnathidae	*Tetragnatha elongata*	RBINA228-13	KP655086
Araneae	Tetragnathidae	*Tetragnatha elongata*	RBINA232-13	KP647173
Araneae	Tetragnathidae	*Tetragnatha elongata*	RBINA234-13	KP647454
Araneae	Tetragnathidae	*Tetragnatha elongata*	RBINA235-13	KP653700
Araneae	Tetragnathidae	*Tetragnatha elongata*	RBINA237-13	KP657276
Araneae	Tetragnathidae	*Tetragnatha elongata*	RBINA247-13	KP654908
Araneae	Tetragnathidae	*Tetragnatha elongata*	RBINA249-13	KP647230
Araneae	Tetragnathidae	*Tetragnatha elongata*	RBINA251-13	KP656217
Araneae	Tetragnathidae	*Tetragnatha elongata*	RBINA256-13	KP646624
Araneae	Tetragnathidae	*Tetragnatha elongata*	RBINA260-13	KP647241
Araneae	Tetragnathidae	*Tetragnatha elongata*	RBINA3274-13	KP653220
Araneae	Tetragnathidae	*Tetragnatha elongata*	RBINA3298-13	KP653587
Araneae	Tetragnathidae	*Tetragnatha elongata*	RBINA3449-13	KP653890
Araneae	Tetragnathidae	*Tetragnatha elongata*	RBINA3452-13	KP651984
Araneae	Tetragnathidae	*Tetragnatha elongata*	RBINA3466-13	KP652371
Araneae	Tetragnathidae	*Tetragnatha elongata*	RBINA3506-13	KP648227
Araneae	Tetragnathidae	*Tetragnatha elongata*	RBINA3517-13	KP650637
Araneae	Tetragnathidae	*Tetragnatha elongata*	RBINA3519-13	KP652085
Araneae	Tetragnathidae	*Tetragnatha elongata*	RBINA3526-13	KP656481
Araneae	Tetragnathidae	*Tetragnatha elongata*	RBINA3527-13	KP648280
Araneae	Tetragnathidae	*Tetragnatha elongata*	RBINA3530-13	KP655850
Araneae	Tetragnathidae	*Tetragnatha elongata*	RBINA3532-13	KP652313

Araneae	Tetragnathidae	*Tetragnatha elongata*	RBINA3547-13	KP654146
Araneae	Tetragnathidae	*Tetragnatha elongata*	RBINA5815-13	KP656115
Araneae	Tetragnathidae	*Tetragnatha elongata*	RBINA5822-13	KP649417
Araneae	Tetragnathidae	*Tetragnatha elongata*	RBINA5840-13	KP653575
Araneae	Tetragnathidae	*Tetragnatha elongata*	RBINA982-13	KP650266
Araneae	Tetragnathidae	*Tetragnatha extensa*	CNBAG037-12	KM830625
Araneae	Tetragnathidae	*Tetragnatha extensa*	ERSCH034-07	KF368815
Araneae	Tetragnathidae	*Tetragnatha extensa*	GBCH5698-13	FJ899830
Araneae	Tetragnathidae	*Tetragnatha extensa*	SPIAL034-10	HQ580545
Araneae	Tetragnathidae	*Tetragnatha extensa*	SPRMA704-10	JF887135
Araneae	Tetragnathidae	*Tetragnatha extensa*	SSBAF7744-13	KM834537
Araneae	Tetragnathidae	*Tetragnatha laboriosa*	ARONT809-10	HQ924654
Araneae	Tetragnathidae	*Tetragnatha laboriosa*	CNEIA2650-13	KM829694
Araneae	Tetragnathidae	*Tetragnatha laboriosa*	CNEIC3172-12	KM832448
Araneae	Tetragnathidae	*Tetragnatha laboriosa*	ERSPI398-09	GU682453
Araneae	Tetragnathidae	*Tetragnatha laboriosa*	RBCH075-04	DQ127459
Araneae	Tetragnathidae	*Tetragnatha laboriosa*	SMTPB3479-13	KP646544
Araneae	Tetragnathidae	*Tetragnatha laboriosa*	SPRMA030-10	HQ977132
Araneae	Tetragnathidae	*Tetragnatha montana*	GBCH5696-13	FJ899832
Araneae	Tetragnathidae	*Tetragnatha montana*	GBCH5697-13	FJ899831
Araneae	Tetragnathidae	*Tetragnatha moua*	GBCH3583-09	EU796908
Araneae	Tetragnathidae	*Tetragnatha moua*	GBCH3584-09	EU796907
Araneae	Tetragnathidae	*Tetragnatha moua*	GBCH3585-09	EU796906
Araneae	Tetragnathidae	*Tetragnatha nigrita*	GBCH11146-13	JN817124
Araneae	Tetragnathidae	*Tetragnatha pallescens*	RBCH185-04	DQ127347
Araneae	Tetragnathidae	*Tetragnatha rava*	GBCH3589-09	EU796902
Araneae	Tetragnathidae	*Tetragnatha rava*	GBCH3590-09	EU796901
Araneae	Tetragnathidae	*Tetragnatha straminea*	ARONT082-09	GU682828
Araneae	Tetragnathidae	*Tetragnatha straminea*	ARONT670-10	HQ924529
Araneae	Tetragnathidae	*Tetragnatha straminea*	ARONT671-10	HQ924530
Araneae	Tetragnathidae	*Tetragnatha straminea*	ARONT672-10	HQ924531
Araneae	Tetragnathidae	*Tetragnatha straminea*	ARONT819-10	HQ924664
Araneae	Tetragnathidae	*Tetragnatha straminea*	CNPAD928-13	KM838578
Araneae	Tetragnathidae	*Tetragnatha straminea*	CNSLO002-13	KM840669
Araneae	Tetragnathidae	*Tetragnatha straminea*	RBCH140-04	DQ127398
Araneae	Tetragnathidae	*Tetragnatha straminea*	RBCH171-04	DQ127365
Araneae	Tetragnathidae	*Tetragnatha straminea*	RBCH181-04	DQ127343
Araneae	Tetragnathidae	*Tetragnatha straminea*	RBINA117-13	KP656952
Araneae	Tetragnathidae	*Tetragnatha straminea*	RBINA119-13	KP654787
Araneae	Tetragnathidae	*Tetragnatha straminea*	RBINA1199-13	KP648127
Araneae	Tetragnathidae	*Tetragnatha straminea*	RBINA1209-13	KP655063
Araneae	Tetragnathidae	*Tetragnatha straminea*	RBINA121-13	KP654675
Araneae	Tetragnathidae	*Tetragnatha straminea*	RBINA1214-13	KP648200
Araneae	Tetragnathidae	*Tetragnatha straminea*	RBINA1215-13	KP651364
Araneae	Tetragnathidae	*Tetragnatha straminea*	RBINA1225-13	KP650995
Araneae	Tetragnathidae	*Tetragnatha straminea*	RBINA1228-13	KP655028
Araneae	Tetragnathidae	*Tetragnatha straminea*	RBINA1232-13	KP656762
Araneae	Tetragnathidae	*Tetragnatha straminea*	RBINA1234-13	KP647213
Araneae	Tetragnathidae	*Tetragnatha straminea*	RBINA1320-13	KP656559
Araneae	Tetragnathidae	*Tetragnatha straminea*	RBINA1321-13	KP653556
Araneae	Tetragnathidae	*Tetragnatha straminea*	RBINA1325-13	KP648002
Araneae	Tetragnathidae	*Tetragnatha straminea*	RBINA2249-13	KP655456
Araneae	Tetragnathidae	*Tetragnatha straminea*	RBINA2462-13	KP655083

Araneae	Tetragnathidae	*Tetragnatha straminea*	RBINA2463-13	KP649459
Araneae	Tetragnathidae	*Tetragnatha straminea*	RBINA2464-13	KP648983
Araneae	Tetragnathidae	*Tetragnatha straminea*	RBINA2677-13	KP653377
Araneae	Tetragnathidae	*Tetragnatha straminea*	RBINA2980-13	KP655590
Araneae	Tetragnathidae	*Tetragnatha straminea*	RBINA3290-13	KP645608
Araneae	Tetragnathidae	*Tetragnatha straminea*	RBINA3296-13	KP653990
Araneae	Tetragnathidae	*Tetragnatha straminea*	RBINA3297-13	KP656225
Araneae	Tetragnathidae	*Tetragnatha straminea*	RBINA3341-13	KP656087
Araneae	Tetragnathidae	*Tetragnatha straminea*	RBINA3348-13	KP652321
Araneae	Tetragnathidae	*Tetragnatha straminea*	RBINA3349-13	KP650696
Araneae	Tetragnathidae	*Tetragnatha straminea*	RBINA3363-13	KP645813
Araneae	Tetragnathidae	*Tetragnatha straminea*	RBINA3366-13	KP654668
Araneae	Tetragnathidae	*Tetragnatha straminea*	RBINA3367-13	KP652647
Araneae	Tetragnathidae	*Tetragnatha straminea*	RBINA3497-13	KP653647
Araneae	Tetragnathidae	*Tetragnatha straminea*	RBINA3499-13	KP655958
Araneae	Tetragnathidae	*Tetragnatha straminea*	RBINA3501-13	KP649856
Araneae	Tetragnathidae	*Tetragnatha straminea*	RBINA3510-13	KP653032
Araneae	Tetragnathidae	*Tetragnatha straminea*	RBINA3513-13	KP648042
Araneae	Tetragnathidae	*Tetragnatha straminea*	RBINA3522-13	KP648444
Araneae	Tetragnathidae	*Tetragnatha straminea*	RBINA3528-13	KP655573
Araneae	Tetragnathidae	*Tetragnatha straminea*	RBINA3531-13	KP645602
Araneae	Tetragnathidae	*Tetragnatha straminea*	RBINA3535-13	KP650991
Araneae	Tetragnathidae	*Tetragnatha straminea*	RBINA3537-13	KP646412
Araneae	Tetragnathidae	*Tetragnatha straminea*	RBINA3538-13	KP651864
Araneae	Tetragnathidae	*Tetragnatha straminea*	RBINA3542-13	KP653705
Araneae	Tetragnathidae	*Tetragnatha straminea*	RBINA3546-13	KP655374
Araneae	Tetragnathidae	*Tetragnatha straminea*	RBINA3569-13	KP656169
Araneae	Tetragnathidae	*Tetragnatha straminea*	RBINA4070-13	KP652487
Araneae	Tetragnathidae	*Tetragnatha straminea*	RBINA4071-13	KP650822
Araneae	Tetragnathidae	*Tetragnatha straminea*	RBINA4074-13	KP655020
Araneae	Tetragnathidae	*Tetragnatha straminea*	RBINA4075-13	KP648610
Araneae	Tetragnathidae	*Tetragnatha straminea*	RBINA4078-13	KP653365
Araneae	Tetragnathidae	*Tetragnatha straminea*	RBINA4081-13	KP652574
Araneae	Tetragnathidae	*Tetragnatha straminea*	RBINA4082-13	KP654544
Araneae	Tetragnathidae	*Tetragnatha straminea*	RBINA4888-13	KP655664
Araneae	Tetragnathidae	*Tetragnatha straminea*	RBINA5802-13	KP646403
Araneae	Tetragnathidae	*Tetragnatha straminea*	RBINA5849-13	KP656763
Araneae	Tetragnathidae	*Tetragnatha straminea*	RBINA989-13	KP653207
Araneae	Tetragnathidae	*Tetragnatha straminea*	SSPAA8818-13	KM836346
Araneae	Tetragnathidae	*Tetragnatha straminea*	SSPAA8830-13	KM834854
Araneae	Tetragnathidae	*Tetragnatha straminea*	SSPAB9105-13	KM835294
Araneae	Tetragnathidae	*Tetragnatha straminea*	SSPAB9129-13	KM836544
Araneae	Tetragnathidae	*Tetragnatha straminea*	SSPAG185-13	KM835776
Araneae	Tetragnathidae	*Tetragnatha versicolor*	ARONT604-10	HQ924473
Araneae	Tetragnathidae	*Tetragnatha versicolor*	CNBPD664-13	KM840452
Araneae	Tetragnathidae	*Tetragnatha versicolor*	CNGLB058-13	KM827086
Araneae	Tetragnathidae	*Tetragnatha versicolor*	CNGLF1925-13	KM839067
Araneae	Tetragnathidae	*Tetragnatha versicolor*	CNMIC1627-14	KP647488
Araneae	Tetragnathidae	*Tetragnatha versicolor*	CNPAD927-13	KM836047
Araneae	Tetragnathidae	*Tetragnatha versicolor*	CNPAF957-13	KM837728
Araneae	Tetragnathidae	*Tetragnatha versicolor*	CNPAH370-13	KM824717
Araneae	Tetragnathidae	*Tetragnatha versicolor*	CNPEC082-14	KP651342
Araneae	Tetragnathidae	*Tetragnatha versicolor*	CNPEC1992-14	KP653997

Araneae	Tetragnathidae	*Tetragnatha versicolor*	CNPED066-14	KP651236
Araneae	Tetragnathidae	*Tetragnatha versicolor*	CNPEK253-14	KP650002
Araneae	Tetragnathidae	*Tetragnatha versicolor*	CNPEO1070-14	KP645440
Araneae	Tetragnathidae	*Tetragnatha versicolor*	CNPES332-14	KP656236
Araneae	Tetragnathidae	*Tetragnatha versicolor*	CNPET416-14	KP654914
Araneae	Tetragnathidae	*Tetragnatha versicolor*	CNPPE2322-12	KJ165802
Araneae	Tetragnathidae	*Tetragnatha versicolor*	CNPPE2350-12	KJ164267
Araneae	Tetragnathidae	*Tetragnatha versicolor*	CNPPF1467-12	KJ164006
Araneae	Tetragnathidae	*Tetragnatha versicolor*	CNPPF1468-12	KJ164041
Araneae	Tetragnathidae	*Tetragnatha versicolor*	CNPPG916-12	KJ166389
Araneae	Tetragnathidae	*Tetragnatha versicolor*	CNPPH1173-12	KJ444313
Araneae	Tetragnathidae	*Tetragnatha versicolor*	CNPPH1197-12	KJ443896
Araneae	Tetragnathidae	*Tetragnatha versicolor*	CNSLA293-12	KM824390
Araneae	Tetragnathidae	*Tetragnatha versicolor*	CNSLD641-12	KM835896
Araneae	Tetragnathidae	*Tetragnatha versicolor*	CNSLN003-13	KM836629
Araneae	Tetragnathidae	*Tetragnatha versicolor*	CNSLN004-13	KM828753
Araneae	Tetragnathidae	*Tetragnatha versicolor*	CNSLN563-13	KM838995
Araneae	Tetragnathidae	*Tetragnatha versicolor*	CNSLP003-13	KM825339
Araneae	Tetragnathidae	*Tetragnatha versicolor*	ERSCH029-07	KP656014
Araneae	Tetragnathidae	*Tetragnatha versicolor*	GBCH3247-09	FJ525317
Araneae	Tetragnathidae	*Tetragnatha versicolor*	JSARA003-11	KP645695
Araneae	Tetragnathidae	*Tetragnatha versicolor*	JSAUG1871-12	KP646259
Araneae	Tetragnathidae	*Tetragnatha versicolor*	JSJUL2555-12	KP653231
Araneae	Tetragnathidae	*Tetragnatha versicolor*	JSJUL2557-12	KP654117
Araneae	Tetragnathidae	*Tetragnatha versicolor*	JSSEP1122-12	KP651631
Araneae	Tetragnathidae	*Tetragnatha versicolor*	KKCHE190-06	KF368892
Araneae	Tetragnathidae	*Tetragnatha versicolor*	PHJUN4057-12	KP652981
Araneae	Tetragnathidae	*Tetragnatha versicolor*	PHMTT625-10	JN307896
Araneae	Tetragnathidae	*Tetragnatha versicolor*	PHMTT628-10	JN307899
Araneae	Tetragnathidae	*Tetragnatha versicolor*	PHMTU038-10	KP651828
Araneae	Tetragnathidae	*Tetragnatha versicolor*	PHMTU039-10	KP648940
Araneae	Tetragnathidae	*Tetragnatha versicolor*	PHMTU040-10	KP647962
Araneae	Tetragnathidae	*Tetragnatha versicolor*	PHMTU049-10	KP650989
Araneae	Tetragnathidae	*Tetragnatha versicolor*	PHMTU065-10	KP647914
Araneae	Tetragnathidae	*Tetragnatha versicolor*	PHMTU066-10	KP654202
Araneae	Tetragnathidae	*Tetragnatha versicolor*	PHMTU074-10	KP648521
Araneae	Tetragnathidae	*Tetragnatha versicolor*	PHMTU081-10	KP650744
Araneae	Tetragnathidae	*Tetragnatha versicolor*	PHMTU086-10	JN307946
Araneae	Tetragnathidae	*Tetragnatha versicolor*	PHMTU087-10	KP653368
Araneae	Tetragnathidae	*Tetragnatha versicolor*	PHMTU088-10	KP646091
Araneae	Tetragnathidae	*Tetragnatha versicolor*	PHMTU099-10	KP654884
Araneae	Tetragnathidae	*Tetragnatha versicolor*	PHMTW368-10	KP656658
Araneae	Tetragnathidae	*Tetragnatha versicolor*	PHOCT947-12	KP654299
Araneae	Tetragnathidae	*Tetragnatha versicolor*	PHSEP1950-11	KP651258
Araneae	Tetragnathidae	*Tetragnatha versicolor*	PHSEP1954-11	KP655273
Araneae	Tetragnathidae	*Tetragnatha versicolor*	RBINA1026-13	KP653611
Araneae	Tetragnathidae	*Tetragnatha versicolor*	RBINA1218-13	KP653137
Araneae	Tetragnathidae	*Tetragnatha versicolor*	RBINA252-13	KP647886
Araneae	Tetragnathidae	*Tetragnatha versicolor*	RBINA3444-13	KP656324
Araneae	Tetragnathidae	*Tetragnatha versicolor*	RBINA3498-13	KP653410
Araneae	Tetragnathidae	*Tetragnatha versicolor*	RBINA3502-13	KP657371
Araneae	Tetragnathidae	*Tetragnatha versicolor*	RBINA3675-13	KP656472
Araneae	Tetragnathidae	*Tetragnatha versicolor*	RBINA4881-13	KP651967

Araneae	Tetragnathidae	*Tetragnatha versicolor*	RBINA784-13	KP646085
Araneae	Tetragnathidae	*Tetragnatha versicolor*	SMTPB1069-13	KP655544
Araneae	Tetragnathidae	*Tetragnatha versicolor*	SMTPB11480-13	KP655303
Araneae	Tetragnathidae	*Tetragnatha versicolor*	SMTPB12317-13	KP657310
Araneae	Tetragnathidae	*Tetragnatha versicolor*	SMTPB12318-13	KP650197
Araneae	Tetragnathidae	*Tetragnatha versicolor*	SMTPB3481-13	KP650293
Araneae	Tetragnathidae	*Tetragnatha versicolor*	SMTPB5901-13	KP652456
Araneae	Tetragnathidae	*Tetragnatha versicolor*	SMTPB7656-13	KP648936
Araneae	Tetragnathidae	*Tetragnatha versicolor*	SPRMA029-10	HQ977131
Araneae	Tetragnathidae	*Tetragnatha versicolor*	SPRMA032-10	HQ977134
Araneae	Tetragnathidae	*Tetragnatha versicolor*	SSEIC4906-13	KM828445
Araneae	Tetragnathidae	*Tetragnatha versicolor*	SSPAA5568-13	KM828384
Araneae	Tetragnathidae	*Tetragnatha versicolor*	SSPAB4591-13	KM838299
Araneae	Tetragnathidae	*Tetragnatha versicolor*	SSPAB5307-13	KM833818
Araneae	Tetragnathidae	*Tetragnatha versicolor*	SSPAB9133-13	KM834894
Araneae	Tetragnathidae	*Tetragnatha versicolor*	SSPAB992-13	KM833904
Araneae	Tetragnathidae	*Tetragnatha viridis*	ARONT714-10	HQ924570
Araneae	Tetragnathidae	*Tetragnatha viridis*	CNBPM438-13	KM823998
Araneae	Tetragnathidae	*Tetragnatha viridis*	CNSLH309-12	KM830781
Araneae	Tetragnathidae	*Tetragnatha viridis*	CNSLP145-13	KM824174
Araneae	Tetragnathidae	*Tetragnatha viridis*	RBINA006-13	KP649894
Araneae	Tetragnathidae	*Tetragnatha viridis*	RBINA1027-13	KP656743
Araneae	Tetragnathidae	*Tetragnatha viridis*	RBINA1040-13	KP646166
Araneae	Tetragnathidae	*Tetragnatha viridis*	RBINA120-13	KP647202
Araneae	Tetragnathidae	*Tetragnatha viridis*	RBINA1202-13	KP652069
Araneae	Tetragnathidae	*Tetragnatha viridis*	RBINA1205-13	KP648603
Araneae	Tetragnathidae	*Tetragnatha viridis*	RBINA1207-13	KP654514
Araneae	Tetragnathidae	*Tetragnatha viridis*	RBINA1208-13	KP654252
Araneae	Tetragnathidae	*Tetragnatha viridis*	RBINA1210-13	KP651461
Araneae	Tetragnathidae	*Tetragnatha viridis*	RBINA1211-13	KP649476
Araneae	Tetragnathidae	*Tetragnatha viridis*	RBINA1224-13	KP650993
Araneae	Tetragnathidae	*Tetragnatha viridis*	RBINA1227-13	KP652618
Araneae	Tetragnathidae	*Tetragnatha viridis*	RBINA1229-13	KP655619
Araneae	Tetragnathidae	*Tetragnatha viridis*	RBINA1230-13	KP649310
Araneae	Tetragnathidae	*Tetragnatha viridis*	RBINA1233-13	KP648752
Araneae	Tetragnathidae	*Tetragnatha viridis*	RBINA3045-13	KP647745
Araneae	Tetragnathidae	*Tetragnatha viridis*	RBINA3046-13	KP655639
Araneae	Tetragnathidae	*Tetragnatha viridis*	RBINA3047-13	KP652324
Araneae	Tetragnathidae	*Tetragnatha viridis*	RBINA3292-13	KP653112
Araneae	Tetragnathidae	*Tetragnatha viridis*	RBINA3331-13	KP657475
Araneae	Tetragnathidae	*Tetragnatha viridis*	RBINA3345-13	KP646591
Araneae	Tetragnathidae	*Tetragnatha viridis*	RBINA3347-13	KP645971
Araneae	Tetragnathidae	*Tetragnatha viridis*	RBINA3350-13	KP656655
Araneae	Tetragnathidae	*Tetragnatha viridis*	RBINA3434-13	KP645898
Araneae	Tetragnathidae	*Tetragnatha viridis*	RBINA3484-13	KP646749
Araneae	Tetragnathidae	*Tetragnatha viridis*	RBINA3505-13	KP645412
Araneae	Tetragnathidae	*Tetragnatha viridis*	RBINA3511-13	KP650058
Araneae	Tetragnathidae	*Tetragnatha viridis*	RBINA3516-13	KP649370
Araneae	Tetragnathidae	*Tetragnatha viridis*	RBINA3520-13	KP647432
Araneae	Tetragnathidae	*Tetragnatha viridis*	RBINA3534-13	KP654834
Araneae	Tetragnathidae	*Tetragnatha viridis*	RBINA4889-13	KP655703
Araneae	Tetragnathidae	*Tylorida striata*	GBCH3885-09	EU003309
Araneae	Theraphosidae	*Aphonopelma sp. 1 SEM-2008*	GBCH2389-08	EU523754

Araneae	Theraphosidae	*Aphonopelma sp. 1 SEM-2008*	GBCH3207-08	NC_010780
Araneae	Theraphosidae	*Coremiocnemis cunicularia*	GBCH7750-13	JN018198
Araneae	Theraphosidae	*Cyriopagopus schioedtei*	GBCH7749-13	JN018199
Araneae	Theraphosidae	*Cyriopagopus schioedtei*	GBCH7800-13	JN018126
Araneae	Theraphosidae	*Haplopelma schmidti*	GBCH7799-13	JN018127
Araneae	Theridiidae	*Anelosimus crassipes*	GBCH11174-13	JN817096
Araneae	Theridiidae	*Ariamnes cylindrogaster*	GBCH11165-13	JN817105
Araneae	Theridiidae	*Asagena americana*	SPRMA055-10	HQ977144
Araneae	Theridiidae	*Canalidion montanum*	CNBAB386-12	KM836142
Araneae	Theridiidae	*Chikunia albipes*	GBCH11172-13	JN817098
Araneae	Theridiidae	*Chrysso octomaculata*	GBCH11171-13	JN817099
Araneae	Theridiidae	*Crustulina sticta*	ARONT253-09	HM434059
Araneae	Theridiidae	*Crustulina sticta*	SPRMA127-10	HQ580761
Araneae	Theridiidae	*Crustulina sticta*	SPRMA882-12	KP653582
Araneae	Theridiidae	*Cryptachaea blattea*	GBCH11630-13	JN859123
Araneae	Theridiidae	*Cryptachaea blattea*	GBCH11631-13	JN859122
Araneae	Theridiidae	*Cryptachaea blattea*	GBCH11632-13	JN859121
Araneae	Theridiidae	*Cryptachaea blattea*	GBCH11633-13	JN859120
Araneae	Theridiidae	*Cryptachaea blattea*	GBCH11634-13	JN859119
Araneae	Theridiidae	*Cryptachaea blattea*	GBCH11635-13	JN859118
Araneae	Theridiidae	*Cryptachaea blattea*	GBCH11636-13	JN859117
Araneae	Theridiidae	*Cryptachaea blattea*	GBCH11637-13	JN859116
Araneae	Theridiidae	*Cryptachaea blattea*	GBCH11638-13	JN859115
Araneae	Theridiidae	*Cryptachaea blattea*	GBCH11639-13	JN859114
Araneae	Theridiidae	*Cryptachaea blattea*	GBCH11640-13	JN859113
Araneae	Theridiidae	*Cryptachaea blattea*	GBCH11641-13	JN859112
Araneae	Theridiidae	*Cryptachaea blattea*	GBCH11642-13	JN859111
Araneae	Theridiidae	*Cryptachaea blattea*	GBCH11643-13	JN859110
Araneae	Theridiidae	*Cryptachaea blattea*	GBCH11644-13	JN859109
Araneae	Theridiidae	*Cryptachaea blattea*	GBCH11645-13	JN859108
Araneae	Theridiidae	*Cryptachaea blattea*	GBCH11646-13	JN859107
Araneae	Theridiidae	*Cryptachaea blattea*	GBCH11647-13	JN859106
Araneae	Theridiidae	*Cryptachaea blattea*	GBCH4214-09	FJ917387
Araneae	Theridiidae	*Cryptachaea blattea*	GBCH4215-09	FJ555054
Araneae	Theridiidae	*Cryptachaea blattea*	GBCH4216-09	FJ555053
Araneae	Theridiidae	*Cryptachaea blattea*	GBCH4217-09	FJ555052
Araneae	Theridiidae	*Cryptachaea blattea*	GBCH4218-09	FJ555051
Araneae	Theridiidae	*Cryptachaea blattea*	GBCH4219-09	FJ555050
Araneae	Theridiidae	*Cryptachaea blattea*	GBCH4220-09	FJ555049
Araneae	Theridiidae	*Cryptachaea blattea*	GBCH4236-09	EU935471
Araneae	Theridiidae	*Cryptachaea blattea*	GBCH4237-09	EU935470
Araneae	Theridiidae	*Cryptachaea blattea*	GBCH4238-09	EU935469
Araneae	Theridiidae	*Cryptachaea canionis*	SPRMA038-10	HQ977136
Araneae	Theridiidae	*Cryptachaea veruculata*	GBCH11648-13	JN859105
Araneae	Theridiidae	*Cryptachaea veruculata*	GBCH11649-13	JN859104
Araneae	Theridiidae	*Cryptachaea veruculata*	GBCH11650-13	JN859103
Araneae	Theridiidae	*Cryptachaea veruculata*	GBCH11651-13	JN859102
Araneae	Theridiidae	*Dipoena nigra*	ARONT390-09	GU682916
Araneae	Theridiidae	*Dipoena nigra*	SPRMA035-10	HQ977135
Araneae	Theridiidae	*Dipoena sp. 1GAB*	CNNHB3036-14	KR069950
Araneae	Theridiidae	*Dipoena sp. 1GAB*	CNNHC3140-14	KR069583
Araneae	Theridiidae	*Dipoena sp. 1GAB*	CNPKC916-13	KP649820
Araneae	Theridiidae	*Dipoena sp. 1GAB*	SSPAA6458-13	KM831562

Araneae	Theridiidae	*Dipoena washougalia*	SPRMA975-12	KP651070
Araneae	Theridiidae	*Enoplognatha intrepida*	SPICH837-09	GU684461
Araneae	Theridiidae	*Enoplognatha intrepida*	SPRMA920-12	KP653158
Araneae	Theridiidae	*Enoplognatha ovata*	ARONT053-09	GU682764
Araneae	Theridiidae	*Enoplognatha ovata*	ARONT062-09	GU682761
Araneae	Theridiidae	*Enoplognatha ovata*	ARONT095-09	GU682762
Araneae	Theridiidae	*Enoplognatha ovata*	ARONT148-09	GU682576
Araneae	Theridiidae	*Enoplognatha ovata*	ARONT175-09	GU682577
Araneae	Theridiidae	*Enoplognatha ovata*	ARONT184-09	GU682574
Araneae	Theridiidae	*Enoplognatha ovata*	ARONT264-09	GU682676
Araneae	Theridiidae	*Enoplognatha ovata*	ARONT269-09	GU682675
Araneae	Theridiidae	*Enoplognatha ovata*	ARONT273-09	GU682673
Araneae	Theridiidae	*Enoplognatha ovata*	ARONT734-10	HQ924588
Araneae	Theridiidae	*Enoplognatha ovata*	CNEIA2646-12	KM833375
Araneae	Theridiidae	*Enoplognatha ovata*	CNPCB044-12	KM826228
Araneae	Theridiidae	*Enoplognatha ovata*	CNPCC035-12	KM827645
Araneae	Theridiidae	*Enoplognatha ovata*	CNPCD034-12	KM832903
Araneae	Theridiidae	*Enoplognatha ovata*	CNRMA1104-12	KM825072
Araneae	Theridiidae	*Enoplognatha ovata*	CNSLD634-12	KM838615
Araneae	Theridiidae	*Enoplognatha ovata*	CNSLD635-12	KM825317
Araneae	Theridiidae	*Enoplognatha ovata*	CNSLD636-12	KM836169
Araneae	Theridiidae	*Enoplognatha ovata*	CNSLD638-12	KM825539
Araneae	Theridiidae	*Enoplognatha ovata*	CNSLN456-13	KM833908
Araneae	Theridiidae	*Enoplognatha ovata*	CNSLO183-13	KM839256
Araneae	Theridiidae	*Enoplognatha ovata*	CNSLP004-13	KM835363
Araneae	Theridiidae	*Enoplognatha ovata*	CNSLS001-13	KM828398
Araneae	Theridiidae	*Enoplognatha ovata*	ERSPI324-09	GU682514
Araneae	Theridiidae	*Enoplognatha ovata*	ERSPI405-09	GU682449
Araneae	Theridiidae	*Enoplognatha ovata*	ERSPI406-09	GU682450
Araneae	Theridiidae	*Enoplognatha ovata*	PHJUL2615-11	KP647848
Araneae	Theridiidae	*Enoplognatha ovata*	PHMTT626-10	JN307897
Araneae	Theridiidae	*Enoplognatha ovata*	PHMTT977-10	JN307914
Araneae	Theridiidae	*Enoplognatha ovata*	PHMTT978-10	JN307915
Araneae	Theridiidae	*Enoplognatha ovata*	PHMTV448-10	KP656547
Araneae	Theridiidae	*Enoplognatha ovata*	PHMTW364-10	KP650438
Araneae	Theridiidae	*Enoplognatha ovata*	PHMTW365-10	KP649521
Araneae	Theridiidae	*Enoplognatha ovata*	PHMTW366-10	KP649782
Araneae	Theridiidae	*Enoplognatha ovata*	PHMTW367-10	KP647020
Araneae	Theridiidae	*Enoplognatha ovata*	PHMTW370-10	KP656621
Araneae	Theridiidae	*Enoplognatha ovata*	PHMTW372-10	KP646105
Araneae	Theridiidae	*Enoplognatha ovata*	PHMTW373-10	KP645430
Araneae	Theridiidae	*Enoplognatha ovata*	PHMTW374-10	KP651722
Araneae	Theridiidae	*Enoplognatha ovata*	RBCH088-04	DQ127440
Araneae	Theridiidae	*Enoplognatha ovata*	RBCH090-04	DQ127442
Araneae	Theridiidae	*Enoplognatha ovata*	RBCH103-04	DQ127423
Araneae	Theridiidae	*Enoplognatha ovata*	RBCH174-04	DQ127352
Araneae	Theridiidae	*Enoplognatha ovata*	RBINA018-13	KP647031
Araneae	Theridiidae	*Enoplognatha ovata*	RBINA1086-13	KP646382
Araneae	Theridiidae	*Enoplognatha ovata*	RBINA1094-13	KP650695
Araneae	Theridiidae	*Enoplognatha ovata*	RBINA113-13	KP657332
Araneae	Theridiidae	*Enoplognatha ovata*	RBINA1496-13	KP647035
Araneae	Theridiidae	*Enoplognatha ovata*	RBINA1497-13	KP647800
Araneae	Theridiidae	*Enoplognatha ovata*	RBINA1558-13	KP650148

Araneae	Theridiidae	*Enoplognatha ovata*	RBINA1862-13	KP657387
Araneae	Theridiidae	*Enoplognatha ovata*	RBINA2718-13	KP653885
Araneae	Theridiidae	*Enoplognatha ovata*	RBINA2719-13	KP650447
Araneae	Theridiidae	*Enoplognatha ovata*	RBINA2812-13	KP654520
Araneae	Theridiidae	*Enoplognatha ovata*	RBINA2856-13	KP650262
Araneae	Theridiidae	*Enoplognatha ovata*	RBINA2857-13	KP656192
Araneae	Theridiidae	*Enoplognatha ovata*	RBINA2891-13	KP655753
Araneae	Theridiidae	*Enoplognatha ovata*	RBINA3229-13	KP648074
Araneae	Theridiidae	*Enoplognatha ovata*	RBINA3232-13	KP648364
Araneae	Theridiidae	*Enoplognatha ovata*	RBINA3234-13	KP647431
Araneae	Theridiidae	*Enoplognatha ovata*	RBINA3235-13	KP650762
Araneae	Theridiidae	*Enoplognatha ovata*	RBINA3238-13	KP656905
Araneae	Theridiidae	*Enoplognatha ovata*	RBINA3344-13	KP654317
Araneae	Theridiidae	*Enoplognatha ovata*	RBINA3368-13	KP648237
Araneae	Theridiidae	*Enoplognatha ovata*	RBINA3398-13	KP648730
Araneae	Theridiidae	*Enoplognatha ovata*	RBINA3932-13	KP652145
Araneae	Theridiidae	*Enoplognatha ovata*	RBINA3957-13	KP647798
Araneae	Theridiidae	*Enoplognatha ovata*	RBINA5187-13	KP650434
Araneae	Theridiidae	*Enoplognatha ovata*	RBINA980-13	KP654442
Araneae	Theridiidae	*Enoplognatha ovata*	RBINA997-13	KP647872
Araneae	Theridiidae	*Enoplognatha ovata*	SMTPB11130-13	KP651230
Araneae	Theridiidae	*Enoplognatha ovata*	SPRMA745-10	JF887161
Araneae	Theridiidae	*Enoplognatha ovata*	SPRMA746-10	JF887162
Araneae	Theridiidae	*Enoplognatha thoracica*	SPRMA040-10	HQ977138
Araneae	Theridiidae	*Enoplognatha thoracica*	SPRMA042-10	HQ977139
Araneae	Theridiidae	*Enoplognatha thoracica*	SPRMA345-10	HQ580903
Araneae	Theridiidae	*Episinus nubilus*	GBCH11169-13	JN817101
Araneae	Theridiidae	*Euryopis argentea*	CNWLL215-13	KM826661
Araneae	Theridiidae	*Euryopis funebris*	ARONT711-10	HQ924567
Araneae	Theridiidae	*Euryopis funebris*	CNPPE2345-12	KJ163935
Araneae	Theridiidae	*Euryopis funebris*	CNPPG911-12	KJ167279
Araneae	Theridiidae	*Euryopis funebris*	CNPPG912-12	KJ165928
Araneae	Theridiidae	*Euryopis funebris*	CNPPH1194-12	KJ163686
Araneae	Theridiidae	*Euryopis funebris*	CNPPJ1373-12	KJ208083
Araneae	Theridiidae	*Euryopis funebris*	GBCH0487-06	AY231042
Araneae	Theridiidae	*Euryopis funebris*	RBCH123-04	DQ127411
Araneae	Theridiidae	*Euryopis pepini*	SSWLB2434-13	KM839178
Araneae	Theridiidae	*Euryopis pepini*	SSWLB2436-13	KM830543
Araneae	Theridiidae	*Euryopis pepini*	SSWLB2448-13	KM828069
Araneae	Theridiidae	*Euryopis pepini*	SSWLB2484-13	KM827884
Araneae	Theridiidae	*Euryopis pepini*	SSWLB274-13	KM825047
Araneae	Theridiidae	*Euryopis pepini*	SSWLB3460-13	KM831910
Araneae	Theridiidae	*Euryopis pepini*	SSWLB5134-13	KM825216
Araneae	Theridiidae	*Euryopis scriptipes*	SSJAB1317-13	KM828724
Araneae	Theridiidae	*Euryopis scriptipes*	SSJAB3385-13	KM839048
Araneae	Theridiidae	*Lasaeola prona*	SPRMA919-12	KP648424
Araneae	Theridiidae	*Latrodectus*	GBCH11217-13	KC414075
Araneae	Theridiidae	*Latrodectus geometricus*	GBCH0608-06	AY383065
Araneae	Theridiidae	*Latrodectus geometricus*	GBCH11216-13	KC414076
Araneae	Theridiidae	*Latrodectus geometricus*	GBCH2218-07	AY383066
Araneae	Theridiidae	*Latrodectus geometricus*	GBCH2222-07	AY383050
Araneae	Theridiidae	*Latrodectus geometricus*	GBCH4032-09	FJ607567
Araneae	Theridiidae	*Latrodectus hasselti*	GBCH0597-06	AY383051

Araneae	Theridiidae	*Latrodectus hasselti*	GBCH0611-06	AY383069
Araneae	Theridiidae	*Latrodectus hasselti*	GBCH11213-13	KC414079
Araneae	Theridiidae	*Latrodectus hasselti*	GBCH11214-13	KC414078
Araneae	Theridiidae	*Latrodectus hasselti*	GBCH11215-13	KC414077
Araneae	Theridiidae	*Latrodectus hasselti*	GBCH2690-08	EF121037
Araneae	Theridiidae	*Latrodectus hasselti*	GBCH2691-08	EF121036
Araneae	Theridiidae	*Latrodectus hasselti*	GBCH2692-08	EF121035
Araneae	Theridiidae	*Latrodectus hasselti*	GBCH2693-08	EF121034
Araneae	Theridiidae	*Latrodectus hasselti*	GBCH2694-08	EF121033
Araneae	Theridiidae	*Latrodectus hasselti*	GBCH2695-08	EF121032
Araneae	Theridiidae	*Latrodectus hasselti*	GBCH2696-08	EF121031
Araneae	Theridiidae	*Latrodectus hesperus*	GBCH0612-06	AY383070
Araneae	Theridiidae	*Latrodectus hesperus*	GBCH11211-13	KC414081
Araneae	Theridiidae	*Latrodectus hesperus*	GBCH11212-13	KC414080
Araneae	Theridiidae	*Latrodectus hesperus*	RBCH206-04	DQ127320
Araneae	Theridiidae	*Latrodectus hesperus*	RBCH209-04	DQ127323
Araneae	Theridiidae	*Latrodectus hesperus*	RBCH210-04	DQ127324
Araneae	Theridiidae	*Latrodectus hesperus*	RBCH213-04	DQ127314
Araneae	Theridiidae	*Latrodectus hesperus*	RBCH214-04	DQ127315
Araneae	Theridiidae	*Latrodectus hesperus*	RBCH215-04	DQ127316
Araneae	Theridiidae	*Latrodectus hesperus*	RBCH216-04	DQ127317
Araneae	Theridiidae	*Latrodectus hesperus*	SPRMA046-10	HQ977140
Araneae	Theridiidae	*Latrodectus hesperus*	SPRMA047-10	HQ977141
Araneae	Theridiidae	*Latrodectus katipo*	GBCH0598-06	AY383052
Araneae	Theridiidae	*Latrodectus katipo*	GBCH0599-06	AY383053
Araneae	Theridiidae	*Latrodectus katipo*	GBCH2697-08	EF121030
Araneae	Theridiidae	*Latrodectus katipo*	GBCH2698-08	EF121029
Araneae	Theridiidae	*Latrodectus katipo*	GBCH2699-08	EF121028
Araneae	Theridiidae	*Latrodectus katipo*	GBCH2700-08	EF121027
Araneae	Theridiidae	*Latrodectus katipo*	GBCH2701-08	EF121026
Araneae	Theridiidae	*Latrodectus katipo*	GBCH2702-08	EF121025
Araneae	Theridiidae	*Latrodectus katipo*	GBCH2703-08	EF121024
Araneae	Theridiidae	*Latrodectus katipo*	GBCH2704-08	EF121023
Araneae	Theridiidae	*Latrodectus katipo*	GBCH2705-08	EF121022
Araneae	Theridiidae	*Latrodectus katipo*	GBCH2706-08	EF121021
Araneae	Theridiidae	*Latrodectus katipo*	GBCH2707-08	EF121020
Araneae	Theridiidae	*Latrodectus katipo*	GBCH2708-08	EF121019
Araneae	Theridiidae	*Latrodectus katipo*	GBCH2709-08	EF121018
Araneae	Theridiidae	*Latrodectus katipo*	GBCH2710-08	EF121017
Araneae	Theridiidae	*Latrodectus katipo*	GBCH2711-08	EF121016
Araneae	Theridiidae	*Latrodectus katipo*	GBCH2712-08	EF121015
Araneae	Theridiidae	*Latrodectus katipo*	GBCH2713-08	EF121014
Araneae	Theridiidae	*Latrodectus katipo*	GBCH2714-08	EF121013
Araneae	Theridiidae	*Latrodectus katipo*	GBCH2715-08	EF121012
Araneae	Theridiidae	*Latrodectus katipo*	GBCH2716-08	EF121011
Araneae	Theridiidae	*Latrodectus katipo*	GBCH2717-08	EF121010
Araneae	Theridiidae	*Latrodectus katipo*	GBCH2718-08	EF121009
Araneae	Theridiidae	*Latrodectus katipo*	GBCH2719-08	EF121008
Araneae	Theridiidae	*Latrodectus katipo*	GBCH2720-08	EF121007
Araneae	Theridiidae	*Latrodectus katipo*	GBCH2721-08	EF121006
Araneae	Theridiidae	*Latrodectus katipo*	GBCH3289-09	EU305455
Araneae	Theridiidae	*Latrodectus katipo*	GBCH3290-09	EU305454
Araneae	Theridiidae	*Latrodectus katipo*	GBCH3291-09	EU305453

Araneae	Theridiidae	*Latrodectus katipo*	GBCH3292-09	EU305452
Araneae	Theridiidae	*Latrodectus katipo*	GBCH3293-09	EU305451
Araneae	Theridiidae	*Latrodectus katipo*	GBCH3294-09	EU305450
Araneae	Theridiidae	*Latrodectus katipo*	GBCH3295-09	EU305449
Araneae	Theridiidae	*Latrodectus katipo*	GBCH3296-09	EU305448
Araneae	Theridiidae	*Latrodectus katipo*	GBCH3297-09	EU309678
Araneae	Theridiidae	*Latrodectus mactans*	RBCH207-04	DQ127321
Araneae	Theridiidae	*Latrodectus mactans*	RBCH217-04	DQ127318
Araneae	Theridiidae	*Latrodectus mirabilis*	GBCH0618-06	AY383076
Araneae	Theridiidae	*Latrodectus pallidus*	GBCH0601-06	AY383055
Araneae	Theridiidae	*Latrodectus pallidus*	GBCH0602-06	AY383056
Araneae	Theridiidae	*Latrodectus pallidus*	GBCH11209-13	KC414083
Araneae	Theridiidae	*Latrodectus pallidus*	GBCH11210-13	KC414082
Araneae	Theridiidae	*Latrodectus sp. 010-Lat*	GBCH0616-06	AY383074
Araneae	Theridiidae	*Latrodectus thoracicus*	GBCH8325-13	GU112105
Araneae	Theridiidae	*Latrodectus thoracicus*	GBCH8326-13	GU112104
Araneae	Theridiidae	*Latrodectus thoracicus*	GBCH8327-13	GU112103
Araneae	Theridiidae	*Latrodectus thoracicus*	GBCH8328-13	GU112102
Araneae	Theridiidae	*Latrodectus thoracicus*	GBCH8329-13	GU112101
Araneae	Theridiidae	*Latrodectus thoracicus*	GBCH8330-13	GU112100
Araneae	Theridiidae	*Latrodectus thoracicus*	GBCH8331-13	GU112099
Araneae	Theridiidae	*Latrodectus thoracicus*	GBCH8332-13	GU112098
Araneae	Theridiidae	*Latrodectus tredecimguttatus*	GBCH0623-06	AY383081
Araneae	Theridiidae	*Latrodectus tredecimguttatus*	GBCH11207-13	KC414085
Araneae	Theridiidae	*Latrodectus tredecimguttatus*	GBCH11208-13	KC414084
Araneae	Theridiidae	*Latrodectus variegatus*	GBCH0625-06	AY383083
Araneae	Theridiidae	*Latrodectus variegatus*	GBCH0626-06	AY383084
Araneae	Theridiidae	*Neospintharus trigonum*	ARONT261-09	GU682677
Araneae	Theridiidae	*Neospintharus trigonum*	ARONT262-09	GU682678
Araneae	Theridiidae	*Neospintharus trigonum*	ARONT712-10	HQ924568
Araneae	Theridiidae	*Neospintharus trigonum*	ARONT713-10	HQ924569
Araneae	Theridiidae	*Neospintharus trigonum*	CNSLI112-12	KM837555
Araneae	Theridiidae	*Neospintharus trigonum*	CNSLS511-13	KM828311
Araneae	Theridiidae	*Neospintharus trigonum*	RBINA3233-13	KP652681
Araneae	Theridiidae	*Neospintharus trigonum*	RBINA3455-13	KP647364
Araneae	Theridiidae	*Neospintharus trigonum*	RBINA5807-13	KP648872
Araneae	Theridiidae	*Neospintharus trigonum*	RBINA5814-13	KP655954
Araneae	Theridiidae	*Neospintharus trigonum*	RBINA5825-13	KP652247
Araneae	Theridiidae	*Neottiura bimaculata*	ERSPI295-09	HM376103
Araneae	Theridiidae	*Neottiura bimaculata*	RBINA1546-13	KP649681
Araneae	Theridiidae	*Neottiura bimaculata*	SMTPB5415-13	KP648948
Araneae	Theridiidae	*Neottiura bimaculata*	SMTPB877-13	KP651419
Araneae	Theridiidae	*Neottiura bimaculata*	SPRMA755-10	JF887169
Araneae	Theridiidae	*Nesticodes rufipes*	GBCH0494-06	AY231049
Araneae	Theridiidae	*Nesticodes rufipes*	GBCH11629-13	JN859124
Araneae	Theridiidae	*Ohlertidion ohlerti*	CNJAB109-12	KM828033
Araneae	Theridiidae	*Ohlertidion ohlerti*	GBADC002-10	HQ956666
Araneae	Theridiidae	*Parasteatoda culicivora*	GBCH11182-13	JN817088
Araneae	Theridiidae	*Parasteatoda japonica*	GBCH11180-13	JN817090
Araneae	Theridiidae	*Parasteatoda kompirensis*	GBCH11179-13	JN817091
Araneae	Theridiidae	*Parasteatoda tabulata*	ARONT043-09	GU682769
Araneae	Theridiidae	*Parasteatoda tabulata*	ARONT047-09	GU682770
Araneae	Theridiidae	*Parasteatoda tabulata*	ARONT048-09	GU682818

Araneae	Theridiidae	*Parasteatoda tabulata*	ARONT242-09	GU682666
Araneae	Theridiidae	*Parasteatoda tabulata*	ARONT629-10	HQ924494
Araneae	Theridiidae	*Parasteatoda tabulata*	ERSPI365-09	GU682480
Araneae	Theridiidae	*Parasteatoda tabulata*	ERSPI377-09	GU682468
Araneae	Theridiidae	*Parasteatoda tabulata*	ERSPI378-09	GU682469
Araneae	Theridiidae	*Parasteatoda tabulata*	ERSPI379-09	GU682470
Araneae	Theridiidae	*Parasteatoda tabulata*	ERSPI380-09	GU682471
Araneae	Theridiidae	*Parasteatoda tabulata*	ERSPI381-09	GU682462
Araneae	Theridiidae	*Parasteatoda tabulata*	GBCH11181-13	JN817089
Araneae	Theridiidae	*Parasteatoda tabulata*	RBINA072-13	KP649646
Araneae	Theridiidae	*Parasteatoda tabulata*	RBINA3306-13	KP657057
Araneae	Theridiidae	*Parasteatoda tabulata*	RBINA3357-13	KP649210
Araneae	Theridiidae	*Parasteatoda tabulata*	RBINA3415-13	KP645982
Araneae	Theridiidae	*Parasteatoda tabulata*	RBINA5826-13	KP649265
Araneae	Theridiidae	*Parasteatoda tepidariorum*	ARONT040-09	GU682777
Araneae	Theridiidae	*Parasteatoda tepidariorum*	ARONT041-09	GU682773
Araneae	Theridiidae	*Parasteatoda tepidariorum*	ARONT042-09	GU682774
Araneae	Theridiidae	*Parasteatoda tepidariorum*	ARONT044-09	GU682772
Araneae	Theridiidae	*Parasteatoda tepidariorum*	ARONT045-09	GU682778
Araneae	Theridiidae	*Parasteatoda tepidariorum*	ARONT046-09	GU682821
Araneae	Theridiidae	*Parasteatoda tepidariorum*	ARONT052-09	GU682771
Araneae	Theridiidae	*Parasteatoda tepidariorum*	ARONT054-09	GU682775
Araneae	Theridiidae	*Parasteatoda tepidariorum*	ARONT055-09	GU682776
Araneae	Theridiidae	*Parasteatoda tepidariorum*	ARONT198-09	GU682665
Araneae	Theridiidae	*Parasteatoda tepidariorum*	CNSLJ035-12	KM834218
Araneae	Theridiidae	*Parasteatoda tepidariorum*	ERSPI396-09	GU682451
Araneae	Theridiidae	*Parasteatoda tepidariorum*	GBCH0477-06	AY231029
Araneae	Theridiidae	*Parasteatoda tepidariorum*	GBCH11183-13	JN817087
Araneae	Theridiidae	*Parasteatoda tepidariorum*	GBCH11203-13	KC414089
Araneae	Theridiidae	*Parasteatoda tepidariorum*	GBCH11627-13	JN859126
Araneae	Theridiidae	*Parasteatoda tepidariorum*	GBCH11628-13	JN859125
Araneae	Theridiidae	*Parasteatoda tepidariorum*	GBCH3916-09	EU003277
Araneae	Theridiidae	*Parasteatoda tepidariorum*	HENOV006-12	KR069440
Araneae	Theridiidae	*Parasteatoda tepidariorum*	RBCH035-04	DQ127499
Araneae	Theridiidae	*Parasteatoda tepidariorum*	RBINA1545-13	KP655530
Araneae	Theridiidae	*Parasteatoda tepidariorum*	RBINA1559-13	KP652933
Araneae	Theridiidae	*Parasteatoda tepidariorum*	RBINA1560-13	KP651817
Araneae	Theridiidae	*Parasteatoda tepidariorum*	RBINA3294-13	KP647105
Araneae	Theridiidae	*Parasteatoda tepidariorum*	RBINA3301-13	KP651250
Araneae	Theridiidae	*Parasteatoda tepidariorum*	RBINA3303-13	KP654788
Araneae	Theridiidae	*Parasteatoda tepidariorum*	RBINA3315-13	KP653912
Araneae	Theridiidae	*Parasteatoda tepidariorum*	RBINA991-13	KP657181
Araneae	Theridiidae	*Parasteatoda tepidariorum*	SPRMA750-10	JF887164
Araneae	Theridiidae	*Phoroncidia americana*	GBCH4016-09	FJ607583
Araneae	Theridiidae	*Phycosoma mustelinum*	GBCH11173-13	JN817097
Araneae	Theridiidae	*Phylloneta impressa*	SPIRU977-11	KF368663
Araneae	Theridiidae	*Platnickina mneon*	GBCH11626-13	JN859127
Araneae	Theridiidae	*Platnickina tincta*	ARONT108-09	HM434050
Araneae	Theridiidae	*Platnickina tincta*	CNGIB557-12	KM836351
Araneae	Theridiidae	*Platnickina tincta*	CNGIB565-12	KM837511
Araneae	Theridiidae	*Platnickina tincta*	CNGIH315-13	KM827323
Araneae	Theridiidae	*Platnickina tincta*	CNGLE507-13	KM830243
Araneae	Theridiidae	*Platnickina tincta*	SPRMA065-10	HQ977149

Araneae	Theridiidae	*Platnickina tincta*	SPRMA749-10	KP650294
Araneae	Theridiidae	*Robertus borealis*	SSBAC2895-12	KM825401
Araneae	Theridiidae	*Robertus borealis*	SSBAC4384-13	KM839188
Araneae	Theridiidae	*Robertus borealis*	SSBAC4400-13	KM837880
Araneae	Theridiidae	*Robertus borealis*	SSBAF7701-13	KM829272
Araneae	Theridiidae	*Robertus crosbyi*	SPIRU1039-11	KF368756
Araneae	Theridiidae	*Robertus fuscus*	SPICH626-09	GU684519
Araneae	Theridiidae	*Robertus lyrifer*	KKCHE1087-09	HM377208
Araneae	Theridiidae	*Robertus vigerens*	SPIAL004-10	HQ580518
Araneae	Theridiidae	*Robertus vigerens*	SPIAL016-10	HQ580529
Araneae	Theridiidae	*Robertus vigerens*	SPIAL028-10	HQ580540
Araneae	Theridiidae	*Robertus vigerens*	SPIAL040-10	HQ580550
Araneae	Theridiidae	*Robertus vigerens*	SPIAL052-10	HQ580559
Araneae	Theridiidae	*Robertus vigerens*	SPIAL064-10	HQ580569
Araneae	Theridiidae	*Robertus vigerens*	SPRMA049-10	HQ977142
Araneae	Theridiidae	*Robertus vigerens*	SPRMA052-10	HQ977143
Araneae	Theridiidae	*Robertus vigerens*	SPRMA126-10	HQ580760
Araneae	Theridiidae	*Robertus vigerens*	SPRMA251-10	HQ580857
Araneae	Theridiidae	*Robertus vigerens*	SPRMA254-10	HQ580859
Araneae	Theridiidae	*Robertus vigerens*	SPRMA298-10	HQ977169
Araneae	Theridiidae	*Robertus vigerens*	SPRMA702-10	JF887133
Araneae	Theridiidae	*Rugathodes aurantius*	GBCH1860-07	EF449600
Araneae	Theridiidae	*Rugathodes sexpunctatus*	CNPCA034-12	KM825760
Araneae	Theridiidae	*Rugathodes sexpunctatus*	CNPCA035-12	KM836090
Araneae	Theridiidae	*Rugathodes sexpunctatus*	CNPCA036-12	KM824809
Araneae	Theridiidae	*Rugathodes sexpunctatus*	CNPCA039-13	KM825845
Araneae	Theridiidae	*Rugathodes sexpunctatus*	CNPCB047-12	KM826303
Araneae	Theridiidae	*Rugathodes sexpunctatus*	CNPCB048-12	KM834402
Araneae	Theridiidae	*Rugathodes sexpunctatus*	CNPCC036-12	KM836171
Araneae	Theridiidae	*Rugathodes sexpunctatus*	CNPCC037-12	KM826094
Araneae	Theridiidae	*Rugathodes sexpunctatus*	CNPCC038-12	KM838817
Araneae	Theridiidae	*Rugathodes sexpunctatus*	CNPCC062-13	KM839472
Araneae	Theridiidae	*Rugathodes sexpunctatus*	CNPCE147-13	KM826459
Araneae	Theridiidae	*Rugathodes sexpunctatus*	CNPCL021-13	KM831819
Araneae	Theridiidae	*Rugathodes sexpunctatus*	CNPCQ030-13	KM834115
Araneae	Theridiidae	*Rugathodes sexpunctatus*	SPIAL006-10	HQ580520
Araneae	Theridiidae	*Rugathodes sexpunctatus*	SPIAL018-10	HQ580531
Araneae	Theridiidae	*Rugathodes sexpunctatus*	SPIAL030-10	HQ580542
Araneae	Theridiidae	*Rugathodes sexpunctatus*	SPIAL042-10	HQ580552
Araneae	Theridiidae	*Rugathodes sexpunctatus*	SPIAL053-10	HQ580560
Araneae	Theridiidae	*Rugathodes sexpunctatus*	SPIAL077-10	HQ580581
Araneae	Theridiidae	*Rugathodes sexpunctatus*	SPIAL089-10	HQ580590
Araneae	Theridiidae	*Rugathodes sexpunctatus*	SPRMA329-10	HQ580894
Araneae	Theridiidae	*Steatoda albomaculata*	SPRMA156-10	HQ580780
Araneae	Theridiidae	*Steatoda albomaculata*	SPRMA952-12	KP654227
Araneae	Theridiidae	*Steatoda bipunctata*	ERSPI376-09	GU682467
Araneae	Theridiidae	*Steatoda borealis*	ARONT065-09	GU682827
Araneae	Theridiidae	*Steatoda borealis*	GBCH3887-09	EU003307
Araneae	Theridiidae	*Steatoda borealis*	RBCH112-04	DQ127416
Araneae	Theridiidae	*Steatoda borealis*	SPRMA137-10	HQ580766
Araneae	Theridiidae	*Steatoda borealis*	SPRMA308-10	HQ977176
Araneae	Theridiidae	*Steatoda capensis*	GBCH11206-13	KC414086
Araneae	Theridiidae	*Steatoda cingulata*	GBCH11167-13	JN817103

Araneae	Theridiidae	*Steatoda grossa*	GBCH11204-13	KC414088
Araneae	Theridiidae	*Steatoda grossa*	GBCH11205-13	KC414087
Araneae	Theridiidae	*Steatoda grossa*	GBCH3853-09	AY383086
Araneae	Theridiidae	*Steatoda grossa*	SPRMA058-10	HQ977147
Araneae	Theridiidae	*Steatoda grossa*	SPRMA067-10	HQ977151
Araneae	Theridiidae	*Steatoda grossa*	SPRMA068-10	HQ977152
Araneae	Theridiidae	*Steatoda grossa*	SPRMA266-10	HQ580867
Araneae	Theridiidae	*Steatoda grossa*	SPRMA279-10	HQ580878
Araneae	Theridiidae	*Steatoda hespera*	SPRMA152-10	HQ580777
Araneae	Theridiidae	*Steatoda triangulosa*	ARONT016-09	GU682824
Araneae	Theridiidae	*Steatoda triangulosa*	ARONT133-09	GU682609
Araneae	Theridiidae	*Steatoda triangulosa*	ARONT191-09	GU682693
Araneae	Theridiidae	*Steatoda triangulosa*	ARONT440-09	GU682935
Araneae	Theridiidae	*Steatoda triangulosa*	ERSPI293-09	GU682535
Araneae	Theridiidae	*Steatoda triangulosa*	ERSPI337-09	GU682505
Araneae	Theridiidae	*Steatoda triangulosa*	ERSPI338-09	GU682506
Araneae	Theridiidae	*Steatoda triangulosa*	GBCH11166-13	JN817104
Araneae	Theridiidae	*Stemmops nipponicus*	GBCH11168-13	JN817102
Araneae	Theridiidae	*Takayus latifolius*	GBCH11178-13	JN817092
Araneae	Theridiidae	*Takayus quadrimaculatus*	GBCH11175-13	JN817095
Araneae	Theridiidae	*Takayus takayensis*	GBCH11177-13	JN817093
Araneae	Theridiidae	*Theonoe stridula*	SPIAL041-10	HQ580551
Araneae	Theridiidae	*Theridion albidum*	CNPPH1198-12	KJ443993
Araneae	Theridiidae	*Theridion albidum*	RBINA1132-13	KP653829
Araneae	Theridiidae	*Theridion albidum*	RBINA4434-13	KP650718
Araneae	Theridiidae	*Theridion californicum*	GACAC779-12	JF979383
Araneae	Theridiidae	*Theridion californicum*	GACAC780-12	JF979384
Araneae	Theridiidae	*Theridion californicum*	GACAC781-12	JF979385
Araneae	Theridiidae	*Theridion californicum*	GACAC782-12	JF979386
Araneae	Theridiidae	*Theridion californicum*	GACAC783-12	JF979387
Araneae	Theridiidae	*Theridion californicum*	GACAC784-12	JF979388
Araneae	Theridiidae	*Theridion californicum*	GACAC785-12	JF979389
Araneae	Theridiidae	*Theridion californicum*	GACAC786-12	JF979390
Araneae	Theridiidae	*Theridion californicum*	GACAC787-12	JF979391
Araneae	Theridiidae	*Theridion californicum*	GACAC788-12	JF979392
Araneae	Theridiidae	*Theridion californicum*	GACAC789-12	JF979393
Araneae	Theridiidae	*Theridion californicum*	GACAC790-12	JF979394
Araneae	Theridiidae	*Theridion californicum*	GACAC791-12	JF979395
Araneae	Theridiidae	*Theridion californicum*	GACAC792-12	JF979396
Araneae	Theridiidae	*Theridion californicum*	GACAC793-12	JF979397
Araneae	Theridiidae	*Theridion californicum*	GACAC794-12	JF979398
Araneae	Theridiidae	*Theridion californicum*	GACAC795-12	JF979399
Araneae	Theridiidae	*Theridion californicum*	GACAC796-12	JF979400
Araneae	Theridiidae	*Theridion californicum*	GACAC797-12	JF979401
Araneae	Theridiidae	*Theridion differens*	ARONT403-09	GU682913
Araneae	Theridiidae	*Theridion differens*	ARONT404-09	GU682910
Araneae	Theridiidae	*Theridion differens*	ARONT405-09	GU682912
Araneae	Theridiidae	*Theridion differens*	CNPPE2334-12	KJ088954
Araneae	Theridiidae	*Theridion differens*	CNPPF1463-12	KJ165880
Araneae	Theridiidae	*Theridion differens*	CNPPI1856-12	KJ208306
Araneae	Theridiidae	*Theridion differens*	CNPPI1860-12	KJ207970
Araneae	Theridiidae	*Theridion differens*	GBCH1856-07	EF449604
Araneae	Theridiidae	*Theridion differens*	RBINA5804-13	KP655915

Araneae	Theridiidae	*Theridion differens*	RBINA5806-13	KP649299
Araneae	Theridiidae	*Theridion differens*	RBINA5812-13	KP655578
Araneae	Theridiidae	*Theridion differens*	RBINA5829-13	KP652516
Araneae	Theridiidae	*Theridion differens*	RBINA5835-13	KP652950
Araneae	Theridiidae	*Theridion frondeum*	CNEIB1686-13	KM829332
Araneae	Theridiidae	*Theridion frondeum*	CNEIC3170-12	KM824750
Araneae	Theridiidae	*Theridion frondeum*	CNEIC3171-12	KM829292
Araneae	Theridiidae	*Theridion frondeum*	CNRMD2868-13	KM838163
Araneae	Theridiidae	*Theridion frondeum*	RBCH096-04	DQ127432
Araneae	Theridiidae	*Theridion frondeum*	RBCH097-04	DQ127433
Araneae	Theridiidae	*Theridion frondeum*	RBCH098-04	DQ127434
Araneae	Theridiidae	*Theridion gigantipes*	GBCH11652-13	JN859101
Araneae	Theridiidae	*Theridion gigantipes*	GBCH11653-13	JN859100
Araneae	Theridiidae	*Theridion gigantipes*	GBCH11654-13	JN859099
Araneae	Theridiidae	*Theridion gigantipes*	GBCH11655-13	JN859098
Araneae	Theridiidae	*Theridion gigantipes*	GBCH11656-13	JN859097
Araneae	Theridiidae	*Theridion gigantipes*	GBCH11657-13	JN859096
Araneae	Theridiidae	*Theridion gigantipes*	GBCH11658-13	JN859095
Araneae	Theridiidae	*Theridion gigantipes*	GBCH11659-13	JN859094
Araneae	Theridiidae	*Theridion gigantipes*	GBCH11660-13	JN859093
Araneae	Theridiidae	*Theridion gigantipes*	GBCH11661-13	JN859092
Araneae	Theridiidae	*Theridion gigantipes*	GBCH11662-13	JN859091
Araneae	Theridiidae	*Theridion gigantipes*	GBCH11663-13	JN859090
Araneae	Theridiidae	*Theridion glaucescens*	CNPPE2335-12	KJ085199
Araneae	Theridiidae	*Theridion glaucescens*	CNPPG914-12	KJ166258
Araneae	Theridiidae	*Theridion glaucescens*	RBINA3432-13	KP656988
Araneae	Theridiidae	*Theridion grallator*	GBCH11969-13	JN863390
Araneae	Theridiidae	*Theridion grallator*	GBCH11970-13	JN863389
Araneae	Theridiidae	*Theridion grallator*	GBCH11971-13	JN863388
Araneae	Theridiidae	*Theridion grallator*	GBCH11972-13	JN863387
Araneae	Theridiidae	*Theridion grallator*	GBCH11973-13	JN863386
Araneae	Theridiidae	*Theridion grallator*	GBCH11974-13	JN863385
Araneae	Theridiidae	*Theridion grallator*	GBCH11975-13	JN863384
Araneae	Theridiidae	*Theridion grallator*	GBCH11976-13	JN863383
Araneae	Theridiidae	*Theridion grallator*	GBCH11977-13	JN863382
Araneae	Theridiidae	*Theridion grallator*	GBCH11978-13	JN863381
Araneae	Theridiidae	*Theridion grallator*	GBCH11979-13	JN863380
Araneae	Theridiidae	*Theridion grallator*	GBCH11980-13	JN863379
Araneae	Theridiidae	*Theridion grallator*	GBCH11981-13	JN863378
Araneae	Theridiidae	*Theridion grallator*	GBCH11982-13	JN863377
Araneae	Theridiidae	*Theridion grallator*	GBCH11983-13	JN863376
Araneae	Theridiidae	*Theridion grallator*	GBCH11984-13	JN863375
Araneae	Theridiidae	*Theridion grallator*	GBCH11985-13	JN863374
Araneae	Theridiidae	*Theridion grallator*	GBCH11986-13	JN863373
Araneae	Theridiidae	*Theridion grallator*	GBCH11987-13	JN863372
Araneae	Theridiidae	*Theridion grallator*	GBCH11988-13	JN863371
Araneae	Theridiidae	*Theridion grallator*	GBCH11989-13	JN863370
Araneae	Theridiidae	*Theridion grallator*	GBCH11990-13	JN863369
Araneae	Theridiidae	*Theridion grallator*	GBCH11991-13	JN863368
Araneae	Theridiidae	*Theridion grallator*	GBCH11992-13	JN863367
Araneae	Theridiidae	*Theridion grallator*	GBCH11993-13	JN863366
Araneae	Theridiidae	*Theridion grallator*	GBCH11994-13	JN863365
Araneae	Theridiidae	*Theridion grallator*	GBCH11995-13	JN863364

Araneae	Theridiidae	*Theridion grallator*	GBCH11996-13	JN863363
Araneae	Theridiidae	*Theridion grallator*	GBCH11997-13	JN863362
Araneae	Theridiidae	*Theridion grallator*	GBCH11998-13	JN863361
Araneae	Theridiidae	*Theridion grallator*	GBCH11999-13	JN863360
Araneae	Theridiidae	*Theridion grallator*	GBCH12000-13	JN863359
Araneae	Theridiidae	*Theridion grallator*	GBCH12001-13	JN863358
Araneae	Theridiidae	*Theridion grallator*	GBCH12002-13	JN863357
Araneae	Theridiidae	*Theridion grallator*	GBCH12003-13	JN863356
Araneae	Theridiidae	*Theridion grallator*	GBCH12004-13	JN863355
Araneae	Theridiidae	*Theridion grallator*	GBCH12005-13	JN863354
Araneae	Theridiidae	*Theridion grallator*	GBCH12006-13	JN863353
Araneae	Theridiidae	*Theridion grallator*	GBCH12007-13	JN863352
Araneae	Theridiidae	*Theridion grallator*	GBCH12008-13	JN863351
Araneae	Theridiidae	*Theridion grallator*	GBCH12009-13	JN863350
Araneae	Theridiidae	*Theridion grallator*	GBCH12010-13	JN863349
Araneae	Theridiidae	*Theridion murarium*	CNPPC1919-12	KJ084186
Araneae	Theridiidae	*Theridion murarium*	CNPPC2004-12	KJ084621
Araneae	Theridiidae	*Theridion murarium*	CNPPD2697-12	KJ091364
Araneae	Theridiidae	*Theridion murarium*	ERSPI374-09	GU682477
Araneae	Theridiidae	*Theridion murarium*	GBCH3737-09	EF449611
Araneae	Theridiidae	*Theridion murarium*	JSARA082-11	KP650298
Araneae	Theridiidae	*Theridion murarium*	PHMTT627-10	JN307898
Araneae	Theridiidae	*Theridion murarium*	PHMTU042-10	JN307921
Araneae	Theridiidae	*Theridion murarium*	RBCH062-04	DQ127462
Araneae	Theridiidae	*Theridion murarium*	RBCH063-04	DQ127463
Araneae	Theridiidae	*Theridion murarium*	RBCH064-04	DQ127464
Araneae	Theridiidae	*Theridion murarium*	RBINA824-13	KP652094
Araneae	Theridiidae	*Theridion mystaceum*	SPRMA756-10	JF887170
Araneae	Theridiidae	*Theridion petraeum*	SPRMA918-12	KP648658
Araneae	Theridiidae	*Theridion pictum*	GBADC026-10	HQ956689
Araneae	Theridiidae	*Theridion sp. 2GAB*	SPRMA063-10	HQ977148
Araneae	Theridiidae	*Theridion varians*	ARONT231-09	GU682663
Araneae	Theridiidae	*Theridion varians*	ARONT232-09	GU682664
Araneae	Theridiidae	*Theridion varians*	CNGIB547-12	KM826164
Araneae	Theridiidae	*Theridion varians*	CNGIB548-12	KM826505
Araneae	Theridiidae	*Theridion varians*	CNGIB581-12	KM828741
Araneae	Theridiidae	*Theridion varians*	CNGIC295-12	KM831172
Araneae	Theridiidae	*Theridion varians*	CNGIE397-12	KM826494
Araneae	Theridiidae	*Theridion varians*	SPRMA753-10	JF887167
Araneae	Theridiidae	*Theridula emertoni*	CNPPI1857-12	KJ444474
Araneae	Theridiidae	*Theridula emertoni*	CNPPI1858-12	KJ445088
Araneae	Theridiidae	*Theridula emertoni*	CNPPI1861-12	KJ444966
Araneae	Theridiidae	*Theridula emertoni*	RBINA1842-13	KP648158
Araneae	Theridiidae	*Theridula emertoni*	RBINA2673-13	KP649174
Araneae	Theridiidae	*Theridula emertoni*	RBINA3465-13	KP648491
Araneae	Theridiidae	*Thymoites camano*	SPRMA066-10	HQ977150
Araneae	Theridiidae	*Thymoites camano*	SPRMA768-12	KP646651
Araneae	Theridiidae	*Thymoites oleatus*	SPICH338-09	GU684347
Araneae	Theridiidae	*Thymoites unimaculatus*	ARONT415-09	GU682915
Araneae	Theridiidae	*Thymoites unimaculatus*	ARONT516-10	HQ924406
Araneae	Theridiidae	*Thymoites unimaculatus*	RBINA2248-13	KP645402
Araneae	Theridiidae	*Thymoites unimaculatus*	RBINA2351-13	KP655207
Araneae	Theridiidae	*Thymoites unimaculatus*	RBINA3239-13	KP646689

Araneae	Theridiidae	*Thymoites unimaculatus*	RBINA5810-13	KP652403
Araneae	Theridiidae	*Thymoites unimaculatus*	RBINA5841-13	KP655606
Araneae	Theridiidae	*Thymoites unimaculatus*	RBINA819-13	KP651107
Araneae	Theridiidae	*Wamba crispulus*	CNBPD154-12	KM840816
Araneae	Theridiidae	*Wamba crispulus*	CNSLS534-13	KM826790
Araneae	Theridiidae	*Wamba crispulus*	GBCH1853-07	EF449607
Araneae	Theridiidae	*Wamba crispulus*	PHJUN4070-12	KP651256
Araneae	Theridiidae	*Yunohamella lyrica*	ARONT260-09	HM434060
Araneae	Theridiidae	*Yunohamella lyrica*	PHMTU044-10	KP647779
Araneae	Theridiidae	*Yunohamella lyrica*	PHMTU093-10	KP654329
Araneae	Theridiidae		GBCH7797-13	JN018129
Araneae	Theridiosomatidae	*Theridiosoma gemmosum*	ARONT447-09	GU682946
Araneae	Theridiosomatidae	*Theridiosoma gemmosum*	ARONT448-09	GU682949
Araneae	Theridiosomatidae	*Theridiosoma gemmosum*	ARONT449-09	GU682948
Araneae	Theridiosomatidae	*Theridiosoma gemmosum*	ARONT450-09	GU682947
Araneae	Theridiosomatidae	*Theridiosoma gemmosum*	ARONT451-09	GU682944
Araneae	Theridiosomatidae	*Theridiosoma gemmosum*	ARONT452-09	GU682945
Araneae	Theridiosomatidae	*Theridiosoma gemmosum*	RBINA3231-13	KP648433
Araneae	Theridiosomatidae		GBCH8334-13	GU456914
Araneae	Thomisidae	*Bassaniana utahensis*	ARONT329-09	GU682878
Araneae	Thomisidae	*Bassaniana utahensis*	CNGLE511-13	KM840340
Araneae	Thomisidae	*Bassaniana utahensis*	CNPPJ1370-12	KJ208926
Araneae	Thomisidae	*Bassaniana utahensis*	CNRMH452-13	KM827791
Araneae	Thomisidae	*Bassaniana utahensis*	CNSLR083-13	KM831673
Araneae	Thomisidae	*Bassaniana utahensis*	PHMTU092-10	JN307949
Araneae	Thomisidae	*Bassaniana utahensis*	RBCH045-04	DQ127477
Araneae	Thomisidae	*Bassaniana utahensis*	SPRMA705-10	JF887136
Araneae	Thomisidae	*Bassaniana utahensis*	SPRMA751-10	JF887165
Araneae	Thomisidae	*Coriarachne brunneipes*	SPIRU1095-11	KF367975
Araneae	Thomisidae	*Coriarachne brunneipes*	SPRMA361-10	HQ580911
Araneae	Thomisidae	*Diaea subdola*	GBCH11029-13	JN817242
Araneae	Thomisidae	*Ebelingia kumadai*	GBCH11030-13	JN817241
Araneae	Thomisidae	*Ebrechtella tricuspidata*	GBCH11031-13	JN817240
Araneae	Thomisidae	*Lysiteles coronatus*	GBCH11026-13	JN817245
Araneae	Thomisidae	*Mecaphesa asperata*	ARONT155-09	GU682624
Araneae	Thomisidae	*Mecaphesa asperata*	ARONT156-09	GU682622
Araneae	Thomisidae	*Mecaphesa asperata*	ARONT157-09	GU682621
Araneae	Thomisidae	*Mecaphesa asperata*	ERSPI300-09	GU682532
Araneae	Thomisidae	*Mecaphesa asperata*	RBCH065-04	DQ127465
Araneae	Thomisidae	*Mecaphesa asperata*	RBCH115-04	DQ127419
Araneae	Thomisidae	*Mecaphesa asperata*	RBCH148-04	DQ127390
Araneae	Thomisidae	*Mecaphesa asperata*	RBINA1216-13	KP646127
Araneae	Thomisidae	*Mecaphesa asperata*	RBINA1236-13	KP648068
Araneae	Thomisidae	*Mecaphesa asperata*	RBINA2669-13	KP656726
Araneae	Thomisidae	*Mecaphesa asperata*	RBINA3284-13	KP650128
Araneae	Thomisidae	*Mecaphesa asperata*	RBINA3295-13	KP646023
Araneae	Thomisidae	*Mecaphesa asperata*	RBINA3317-13	KP651978
Araneae	Thomisidae	*Mecaphesa asperata*	RBINA3324-13	KP648047
Araneae	Thomisidae	*Mecaphesa asperata*	RBINA3385-13	KP651095
Araneae	Thomisidae	*Mecaphesa asperata*	RBINA3401-13	KP647843
Araneae	Thomisidae	*Mecaphesa asperata*	RBINA3467-13	KP653444
Araneae	Thomisidae	*Mecaphesa asperata*	RBINA3512-13	KP653233
Araneae	Thomisidae	*Mecaphesa asperata*	RBINA3518-13	KP656261

Araneae	Thomisidae	*Mecaphesa asperata*	RBINA3544-13	KP649256
Araneae	Thomisidae	*Mecaphesa asperata*	RBINA4076-13	KP656994
Araneae	Thomisidae	*Mecaphesa asperata*	RBINA4169-13	KP648585
Araneae	Thomisidae	*Mecaphesa asperata*	RBINA4170-13	KP649260
Araneae	Thomisidae	*Mecaphesa asperata*	RBINA4887-13	KP648822
Araneae	Thomisidae	*Mecaphesa asperata*	RBINA4900-13	KP646226
Araneae	Thomisidae	*Mecaphesa asperata*	RBINA4902-13	KP652841
Araneae	Thomisidae	*Mecaphesa asperata*	RBINA712-13	KP645693
Araneae	Thomisidae	*Mecaphesa asperata*	RBINA714-13	KP647416
Araneae	Thomisidae	*Mecaphesa asperata*	RBINA715-13	KP646489
Araneae	Thomisidae	*Misumena vatia*	JSARA011-11	KP650268
Araneae	Thomisidae	*Misumena vatia*	RBCH153-04	DQ127379
Araneae	Thomisidae	*Misumena vatia*	RBCH156-04	DQ127382
Araneae	Thomisidae	*Misumena vatia*	RBCH157-04	DQ127367
Araneae	Thomisidae	*Misumena vatia*	RBINA1317-13	KP652203
Araneae	Thomisidae	*Misumena vatia*	RBINA1850-13	KP653530
Araneae	Thomisidae	*Misumena vatia*	RBINA1854-13	KP648731
Araneae	Thomisidae	*Misumena vatia*	RBINA1855-13	KP652560
Araneae	Thomisidae	*Misumena vatia*	RBINA1870-13	KP646866
Araneae	Thomisidae	*Misumena vatia*	RBINA1874-13	KP652169
Araneae	Thomisidae	*Misumena vatia*	RBINA2670-13	KP645562
Araneae	Thomisidae	*Misumena vatia*	RBINA993-13	KP653279
Araneae	Thomisidae	*Misumessus oblongus*	ERSPI402-09	HM376108
Araneae	Thomisidae	*Misumessus oblongus*	RBINA1000-13	KP646917
Araneae	Thomisidae	*Misumessus oblongus*	RBINA1843-13	KP653144
Araneae	Thomisidae	*Misumessus oblongus*	RBINA1867-13	KP655550
Araneae	Thomisidae	*Misumessus oblongus*	RBINA1872-13	KP647614
Araneae	Thomisidae	*Misumessus oblongus*	RBINA1876-13	KP648556
Araneae	Thomisidae	*Misumessus oblongus*	RBINA3343-13	KP645868
Araneae	Thomisidae	*Misumessus oblongus*	RBINA3970-13	KP651240
Araneae	Thomisidae	*Misumessus oblongus*	RBINA4901-13	KP650361
Araneae	Thomisidae	*Misumessus oblongus*	RBINA4903-13	KP648221
Araneae	Thomisidae	*Misumessus oblongus*	RBINA4905-13	KP650309
Araneae	Thomisidae	*Misumessus oblongus*	RBINA786-13	KP657373
Araneae	Thomisidae	*Misumessus oblongus*	RBINA791-13	KP652996
Araneae	Thomisidae	*Misumessus oblongus*	RBINA799-13	KP652032
Araneae	Thomisidae	*Misumessus oblongus*	RBINA810-13	KP647580
Araneae	Thomisidae	*Misumessus oblongus*	RBINA817-13	KP652993
Araneae	Thomisidae	*Misumessus oblongus*	SMTPB6526-13	KP653664
Araneae	Thomisidae	*Ozyptila arctica*	JDTGS010-09	GU679904
Araneae	Thomisidae	*Ozyptila conspurcata*	SPRMA161-10	HQ580784
Araneae	Thomisidae	*Ozyptila conspurcata*	SPRMA970-12	KP648683
Araneae	Thomisidae	*Ozyptila gertschi*	ERSPI359-09	GU682487
Araneae	Thomisidae	*Ozyptila gertschi*	SPICH1083-09	GU684593
Araneae	Thomisidae	*Ozyptila pacifica*	SPRMA644-10	JF887103
Araneae	Thomisidae	*Ozyptila praticola*	ARONT138-09	GU682617
Araneae	Thomisidae	*Ozyptila praticola*	ARONT421-09	GU682923
Araneae	Thomisidae	*Ozyptila praticola*	ARONT433-09	GU682920
Araneae	Thomisidae	*Ozyptila praticola*	ARONT453-09	GU682922
Araneae	Thomisidae	*Ozyptila praticola*	ARONT596-10	HQ924470
Araneae	Thomisidae	*Ozyptila praticola*	ERSPI264-09	GU682554
Araneae	Thomisidae	*Ozyptila praticola*	ERSPI266-09	GU682556
Araneae	Thomisidae	*Ozyptila praticola*	ERSPI274-09	GU682553

Araneae	Thomisidae	*Ozyptila praticola*	RBCH041-04	DQ127489
Araneae	Thomisidae	*Ozyptila praticola*	RBCH166-04	DQ127360
Araneae	Thomisidae	*Ozyptila praticola*	RBINA3147-13	KP648481
Araneae	Thomisidae	*Ozyptila praticola*	SMTPB10036-13	KP651947
Araneae	Thomisidae	*Ozyptila praticola*	SMTPB10039-13	KP647335
Araneae	Thomisidae	*Ozyptila sincera canadensis*	CNWBE381-13	KM825075
Araneae	Thomisidae	*Ozyptila sincera canadensis*	SPRMA771-12	KP650846
Araneae	Thomisidae	*Synema globosum*	GBCH11025-13	JN817246
Araneae	Thomisidae	*Tmarus angulatus*	ARONT114-09	GU682619
Araneae	Thomisidae	*Tmarus angulatus*	ARONT185-09	GU682620
Araneae	Thomisidae	*Tmarus angulatus*	ARONT265-09	GU682707
Araneae	Thomisidae	*Tmarus angulatus*	ARONT420-09	GU682925
Araneae	Thomisidae	*Tmarus angulatus*	ARONT717-10	HQ924573
Araneae	Thomisidae	*Tmarus angulatus*	JSARA083-11	KP651599
Araneae	Thomisidae	*Tmarus angulatus*	RBCH093-04	DQ127429
Araneae	Thomisidae	*Tmarus angulatus*	RBINA1322-13	KP652840
Araneae	Thomisidae	*Tmarus piger*	GBCH11023-13	JN817248
Araneae	Thomisidae	*Tmarus stellio*	GBCH11022-13	JN817249
Araneae	Thomisidae	*Xysticus*	GBCH5694-13	FJ899834
Araneae	Thomisidae	*Xysticus benefactor*	SPRMA173-10	HQ580793
Araneae	Thomisidae	*Xysticus benefactor*	SSBAA5230-12	KM824372
Araneae	Thomisidae	*Xysticus benefactor*	SSBAA5231-12	KM828160
Araneae	Thomisidae	*Xysticus benefactor*	SSBAA5232-12	KM828023
Araneae	Thomisidae	*Xysticus benefactor*	SSBAA5234-12	KM827620
Araneae	Thomisidae	*Xysticus benefactor*	SSBAA5236-12	KM833066
Araneae	Thomisidae	*Xysticus benefactor*	SSBAA5241-12	KM825122
Araneae	Thomisidae	*Xysticus benefactor*	SSBAA5264-12	KM834770
Araneae	Thomisidae	*Xysticus benefactor*	SSBAA5265-12	KM827405
Araneae	Thomisidae	*Xysticus benefactor*	SSBAA5266-12	KM840513
Araneae	Thomisidae	*Xysticus benefactor*	SSBAA5267-12	KM826542
Araneae	Thomisidae	*Xysticus benefactor*	SSBAA5287-12	KM833729
Araneae	Thomisidae	*Xysticus benefactor*	SSBAA5289-12	KM833647
Araneae	Thomisidae	*Xysticus benefactor*	SSBAA5290-12	KM827515
Araneae	Thomisidae	*Xysticus benefactor*	SSBAB2771-12	KM836617
Araneae	Thomisidae	*Xysticus benefactor*	SSBAC4422-13	KM828223
Araneae	Thomisidae	*Xysticus benefactor*	SSBAD5977-13	KM837151
Araneae	Thomisidae	*Xysticus benefactor*	SSBAD5978-13	KM829762
Araneae	Thomisidae	*Xysticus benefactor*	SSBAD5981-13	KM835156
Araneae	Thomisidae	*Xysticus benefactor*	SSBAD5982-13	KM838080
Araneae	Thomisidae	*Xysticus benefactor*	SSBAD5983-13	KM837293
Araneae	Thomisidae	*Xysticus benefactor*	SSBAD5984-13	KM827526
Araneae	Thomisidae	*Xysticus benefactor*	SSBAD5986-13	KM828522
Araneae	Thomisidae	*Xysticus benefactor*	SSBAD5987-13	KM827138
Araneae	Thomisidae	*Xysticus benefactor*	SSBAD5989-13	KM824389
Araneae	Thomisidae	*Xysticus benefactor*	SSBAD5990-13	KM830793
Araneae	Thomisidae	*Xysticus benefactor*	SSBAD5992-13	KM834564
Araneae	Thomisidae	*Xysticus benefactor*	SSBAD5993-13	KM828253
Araneae	Thomisidae	*Xysticus benefactor*	SSBAD5994-13	KM828930
Araneae	Thomisidae	*Xysticus benefactor*	SSBAD5996-13	KM838778
Araneae	Thomisidae	*Xysticus benefactor*	SSBAD5998-13	KM830493
Araneae	Thomisidae	*Xysticus benefactor*	SSBAD6005-13	KM839939
Araneae	Thomisidae	*Xysticus benefactor*	SSWLB005-13	KM831518
Araneae	Thomisidae	*Xysticus benefactor*	SSWLB007-13	KM826309

Araneae	Thomisidae	*Xysticus benefactor*	SSWLB018-13	KM827864
Araneae	Thomisidae	*Xysticus benefactor*	SSWLB029-13	KM832992
Araneae	Thomisidae	*Xysticus benefactor*	SSWLB032-13	KM831643
Araneae	Thomisidae	*Xysticus benefactor*	SSWLB271-13	KM831030
Araneae	Thomisidae	*Xysticus benefactor*	SSWLB5726-13	KM826944
Araneae	Thomisidae	*Xysticus benefactor*	SSWLB5813-13	KM831349
Araneae	Thomisidae	*Xysticus benefactor*	SSWLE5659-13	KM836149
Araneae	Thomisidae	*Xysticus benefactor*	SSWLE5662-13	KM837299
Araneae	Thomisidae	*Xysticus benefactor*	SSWLE5672-13	KM832479
Araneae	Thomisidae	*Xysticus benefactor*	SSWLE5696-13	KM836336
Araneae	Thomisidae	*Xysticus benefactor*	SSWLE6341-13	KM839049
Araneae	Thomisidae	*Xysticus benefactor*	SSWLE6373-13	KM836586
Araneae	Thomisidae	*Xysticus benefactor*	SSWLE7982-13	KM836070
Araneae	Thomisidae	*Xysticus benefactor*	SSWLE7988-13	KM831346
Araneae	Thomisidae	*Xysticus benefactor*	SSWLE8005-13	KM833931
Araneae	Thomisidae	*Xysticus benefactor*	SSWLE8879-13	KM827434
Araneae	Thomisidae	*Xysticus benefactor*	SSWLE8891-13	KM839476
Araneae	Thomisidae	*Xysticus benefactor*	SSWLE8895-13	KM835702
Araneae	Thomisidae	*Xysticus benefactor*	SSWLE8897-13	KM836146
Araneae	Thomisidae	*Xysticus benefactor*	SSWLE8898-13	KM829974
Araneae	Thomisidae	*Xysticus bicuspis*	ARONT632-10	HQ924497
Araneae	Thomisidae	*Xysticus bicuspis*	ARONT634-10	HQ924499
Araneae	Thomisidae	*Xysticus bicuspis*	ARONT635-10	HQ924500
Araneae	Thomisidae	*Xysticus bicuspis*	ARONT692-10	HQ924550
Araneae	Thomisidae	*Xysticus bicuspis*	ARONT729-10	HQ924583
Araneae	Thomisidae	*Xysticus bicuspis*	CNGRA006-12	KM831815
Araneae	Thomisidae	*Xysticus bicuspis*	ERSPI290-09	GU682533
Araneae	Thomisidae	*Xysticus britcheri*	JDTGS014-09	GU679900
Araneae	Thomisidae	*Xysticus californicus*	GBCH3428-09	EU168181
Araneae	Thomisidae	*Xysticus californicus*	SPRMA648-10	JF887105
Araneae	Thomisidae	*Xysticus canadensis*	CNBAA473-12	KM839720
Araneae	Thomisidae	*Xysticus canadensis*	CNBAB385-12	KM837624
Araneae	Thomisidae	*Xysticus canadensis*	CNBAI525-13	KM833366
Araneae	Thomisidae	*Xysticus canadensis*	CNBAI530-13	KM838111
Araneae	Thomisidae	*Xysticus canadensis*	CNPAC645-13	KM833331
Araneae	Thomisidae	*Xysticus canadensis*	CNPAK025-13	KM829508
Araneae	Thomisidae	*Xysticus canadensis*	CNPAL032-13	KM840209
Araneae	Thomisidae	*Xysticus canadensis*	RBCH020-04	DQ127516
Araneae	Thomisidae	*Xysticus chippewa*	SSBAA2020-12	KM837148
Araneae	Thomisidae	*Xysticus cristatus*	SPRMA645-10	JN310304
Araneae	Thomisidae	*Xysticus cristatus*	SPRMA646-10	KR070356
Araneae	Thomisidae	*Xysticus cristatus*	SPRMA647-10	JF887104
Araneae	Thomisidae	*Xysticus cunctator*	SPRMA862-12	KP647331
Araneae	Thomisidae	*Xysticus deichmanni*	KKCHE1078-09	GU683755
Araneae	Thomisidae	*Xysticus discursans*	ARONT511-10	HQ924403
Araneae	Thomisidae	*Xysticus discursans*	ARONT633-10	HQ924498
Araneae	Thomisidae	*Xysticus discursans*	ARONT691-10	HQ924549
Araneae	Thomisidae	*Xysticus discursans*	ARONT730-10	HQ924584
Araneae	Thomisidae	*Xysticus discursans*	ARONT744-10	HQ924597
Araneae	Thomisidae	*Xysticus discursans*	ERSPI080-08	KP656720
Araneae	Thomisidae	*Xysticus durus*	KKCHE1079-09	GU683594
Araneae	Thomisidae	*Xysticus elegans*	ARONT382-09	GU682927
Araneae	Thomisidae	*Xysticus elegans*	ARONT391-09	GU682924

Araneae	Thomisidae	*Xysticus elegans*	BBCAN423-09	GU683407
Araneae	Thomisidae	*Xysticus elegans*	CNBPK414-13	KM830687
Araneae	Thomisidae	*Xysticus elegans*	CNBPN454-13	KM835199
Araneae	Thomisidae	*Xysticus elegans*	ERSPI284-09	GU682539
Araneae	Thomisidae	*Xysticus elegans*	PHMTT973-10	JN307911
Araneae	Thomisidae	*Xysticus elegans*	RBCH074-04	DQ127458
Araneae	Thomisidae	*Xysticus elegans*	RBCH081-04	DQ127449
Araneae	Thomisidae	*Xysticus elegans*	RBINA2834-13	KP647386
Araneae	Thomisidae	*Xysticus elegans*	RBINA3237-13	KP656561
Araneae	Thomisidae	*Xysticus elegans*	RBINA414-13	KP650947
Araneae	Thomisidae	*Xysticus elegans*	RBINA415-13	KP652736
Araneae	Thomisidae	*Xysticus ellipticus*	RBCH158-04	DQ127368
Araneae	Thomisidae	*Xysticus ellipticus*	SPICH116-09	GU683801
Araneae	Thomisidae	*Xysticus emertoni*	ARONT314-09	GU682874
Araneae	Thomisidae	*Xysticus emertoni*	CNWBC126-13	KM835759
Araneae	Thomisidae	*Xysticus emertoni*	RBCH030-04	DQ127494
Araneae	Thomisidae	*Xysticus emertoni*	SMTPB15380-13	KP646378
Araneae	Thomisidae	*Xysticus ephippiatus*	GBCH11019-13	JN817252
Araneae	Thomisidae	*Xysticus ephippiatus*	GBCH11021-13	JN817250
Araneae	Thomisidae	*Xysticus ferox*	ARONT467-10	HM880619
Araneae	Thomisidae	*Xysticus ferox*	SSJAB1364-13	KM832710
Araneae	Thomisidae	*Xysticus ferox*	SSJAE11373-13	KM840372
Araneae	Thomisidae	*Xysticus ferox*	SSJAE11504-13	KM835595
Araneae	Thomisidae	*Xysticus funestus*	ARONT334-09	GU682875
Araneae	Thomisidae	*Xysticus funestus*	RBCH163-04	DQ127373
Araneae	Thomisidae	*Xysticus funestus*	RBCH164-04	DQ127374
Araneae	Thomisidae	*Xysticus gulosus*	SPRMA649-10	JN310305
Araneae	Thomisidae	*Xysticus insulicola*	GBCH11020-13	JN817251
Araneae	Thomisidae	*Xysticus labradorensis*	KKCHE1075-09	GU683754
Araneae	Thomisidae	*Xysticus locuples*	SPRMA344-10	HQ580902
Araneae	Thomisidae	*Xysticus locuples*	SPRMA947-12	KP647328
Araneae	Thomisidae	*Xysticus luctans*	ERSPI256-09	GU682559
Araneae	Thomisidae	*Xysticus luctans*	ERSPI257-09	GU682560
Araneae	Thomisidae	*Xysticus luctans*	RBCH162-04	DQ127372
Araneae	Thomisidae	*Xysticus luctuosus*	ARSO275-08	KM840130
Araneae	Thomisidae	*Xysticus luctuosus*	RBCH024-04	DQ127504
Araneae	Thomisidae	*Xysticus luctuosus*	SPICH118-09	GU683803
Araneae	Thomisidae	*Xysticus luctuosus*	SSWLA685-13	KM827969
Araneae	Thomisidae	*Xysticus luctuosus*	SSWLA792-13	KM830324
Araneae	Thomisidae	*Xysticus luctuosus*	SSWLD5775-13	KM836026
Araneae	Thomisidae	*Xysticus luctuosus*	SSWLE5666-13	KM829280
Araneae	Thomisidae	*Xysticus montanensis*	SPRMA104-10	HQ580744
Araneae	Thomisidae	*Xysticus montanensis*	SPRMA650-10	KP645629
Araneae	Thomisidae	*Xysticus montanensis*	SPRMA651-10	JF887106
Araneae	Thomisidae	*Xysticus montanensis*	SPRMA652-10	JF887107
Araneae	Thomisidae	*Xysticus nigromaculatus*	TWSC036-07	KF369066
Araneae	Thomisidae	*Xysticus obscurus*	CNPAF928-13	KM826571
Araneae	Thomisidae	*Xysticus obscurus*	SPICH1009-09	GU684734
Araneae	Thomisidae	*Xysticus pellax*	ARONT170-09	GU682618
Araneae	Thomisidae	*Xysticus pellax*	CNPPE2323-12	KJ167178
Araneae	Thomisidae	*Xysticus pellax*	CNPPJ1366-12	KJ207934
Araneae	Thomisidae	*Xysticus pellax*	RBINA3266-13	KP653270
Araneae	Thomisidae	*Xysticus pretiosus*	SPIAL065-10	HQ580570

Araneae	Thomisidae	*Xysticus pretiosus*	SPRMA099-10	HQ580740
Araneae	Thomisidae	*Xysticus pretiosus*	SPRMA284-10	HQ580883
Araneae	Thomisidae	*Xysticus pretiosus*	SPRMA653-10	KP645713
Araneae	Thomisidae	*Xysticus pretiosus*	SPRMA654-10	JF887108
Araneae	Thomisidae	*Xysticus pretiosus*	SPRMA655-10	JF887109
Araneae	Thomisidae	*Xysticus pretiosus*	SPRMA835-12	KP656759
Araneae	Thomisidae	*Xysticus punctatus*	CNWBG1007-13	KM833505
Araneae	Thomisidae	*Xysticus punctatus*	CNWBH1126-13	KM839919
Araneae	Thomisidae	*Xysticus punctatus*	ERSPI297-09	GU682529
Araneae	Thomisidae	*Xysticus punctatus*	JSARA008-11	KP649516
Araneae	Thomisidae	*Xysticus punctatus*	PHJUL2618-11	KP654573
Araneae	Thomisidae	*Xysticus punctatus*	PHMTT618-10	JN307891
Araneae	Thomisidae	*Xysticus punctatus*	RBCH127-04	DQ127400
Araneae	Thomisidae	*Xysticus sp. MCH-2003*	GBCH0567-06	AY297423
Araneae	Thomisidae	*Xysticus triangulosus*	KKCHE1086-09	GU683600
Araneae	Thomisidae	*Xysticus triangulosus*	SPICH1007-09	GU684731
Araneae	Thomisidae	*Xysticus triguttatus*	SPICH128-09	GU683802
Araneae	Thomisidae	*Xysticus winnipegensis*	ARONT509-10	HQ924402
Araneae	Thomisidae	*Xysticus winnipegensis*	RBINA3313-13	KP655555
Araneae	Trachelidae	*Meriola californica*	CNGIF085-12	KM834636
Araneae	Trachelidae	*Meriola californica*	CNGIF086-12	KM835023
Araneae	Trachelidae	*Trachelas japonicus*	GBCH11046-13	JN817224
Araneae	Trachelidae	*Trachelas tranquillus*	CNPPH1170-12	KJ444177
Araneae	Trachelidae	*Trachelas tranquillus*	CNPPH1175-12	KJ444490
Araneae	Trachelidae	*Trachelas tranquillus*	CNPPH1176-12	KJ443855
Araneae	Trachelidae	*Trachelas tranquillus*	CNPPH1177-12	KJ164681
Araneae	Trachelidae	*Trachelas tranquillus*	CNPPH1178-12	KM837992
Araneae	Trachelidae	*Trachelas tranquillus*	CNPPH1179-12	KJ167816
Araneae	Trachelidae	*Trachelas tranquillus*	CNPPH1181-12	KJ166429
Araneae	Trachelidae	*Trachelas tranquillus*	CNPPH1183-12	KJ444374
Araneae	Trachelidae	*Trachelas tranquillus*	CNPPH1184-12	KJ167839
Araneae	Trachelidae	*Trachelas tranquillus*	CNPPH1185-12	KJ637506
Araneae	Trachelidae	*Trachelas tranquillus*	CNPPH1187-12	KJ444343
Araneae	Trachelidae	*Trachelas tranquillus*	CNPPH1188-12	KJ444378
Araneae	Trachelidae	*Trachelas tranquillus*	CNPPH1190-12	KJ443875
Araneae	Trachelidae	*Trachelas tranquillus*	CNPPH1191-12	KJ444052
Araneae	Trachelidae	*Trachelas tranquillus*	CNPPH1192-12	KJ444528
Araneae	Trachelidae	*Trachelas tranquillus*	CNPPH1193-12	KJ637449
Araneae	Trachelidae	*Trachelas tranquillus*	CNPPI1852-12	KJ209270
Araneae	Trachelidae	*Trachelas tranquillus*	JSARA090-11	KP646050
Araneae	Trachelidae	*Trachelas tranquillus*	RBINA3280-13	KP651275
Araneae	Uloboridae	*Hyptiotes gertschi*	GBCH4033-09	FJ607566
Araneae	Uloboridae	*Octonoba sinensis*	GBCH11190-13	JN817080
Araneae	Uloboridae	*Octonoba yesoensis*	GBCH11189-13	JN817081
Araneae	Uloboridae	*Uloborus diversus*	GBCH3235-09	FJ525329
Araneae	Uloboridae	*Uloborus glomosus*	ARONT719-10	HQ924574
Araneae	Uloboridae	*Uloborus glomosus*	ARONT749-10	HQ924601
Araneae	Uloboridae	*Uloborus glomosus*	CNSLR085-13	KM831234
Araneae	Uloboridae	*Uloborus glomosus*	GBCH3884-09	EU003310
Araneae	Uloboridae	*Uloborus glomosus*	RBINA1021-13	KP656207
Araneae	Uloboridae	*Uloborus glomosus*	RBINA1221-13	KP650968
Araneae	Uloboridae	*Uloborus glomosus*	RBINA2665-13	KP649561
Araneae	Uloboridae	*Uloborus glomosus*	RBINA3316-13	KP648608

Araneae	Uloboridae	*Uloborus glomosus*	RBINA3370-13	KP647896
Araneae	Uloboridae	*Uloborus glomosus*	RBINA3377-13	KP653600
Araneae	Uloboridae	*Uloborus glomosus*	RBINA5302-13	KP649307
Araneae	Uloboridae	*Uloborus glomosus*	RBINA5801-13	KP654210
Araneae	Uloboridae	*Uloborus glomosus*	RBINA5828-13	KP650570
Araneae	Zoropsidae	*Takeoa nishimurai*	GBCH11055-13	JN817215
Holothyrida	Allothyridae	*Allothyrus sp. LamingtonNP-QMS95173*	CYTC5876-15	KC769586
Ixodida	Argasidae	*Argas africolumbae*	GBCH11404-13	NC_019642
Ixodida	Argasidae	*Argas africolumbae*	GBCH11499-13	JQ665720
Ixodida	Argasidae	*Carios capensis*	GBCH0011-06	AB075953
Ixodida	Argasidae	*Carios capensis*	GBCH1679-06	NC_005291
Ixodida	Argasidae	*Ornithodoros moubata*	GBCH0009-06	AB073679
Ixodida	Argasidae	*Ornithodoros moubata*	GBCH1676-06	NC_004357
Ixodida	Argasidae	*Ornithodoros porcinus*	GBCH0032-06	AB105451
Ixodida	Ixodidae	*Amblyomma americanum*	GBCH1239-06	DQ168131
Ixodida	Ixodidae	*Amblyomma cajennense*	GBCH10808-13	NC_020333
Ixodida	Ixodidae	*Amblyomma cajennense*	GBCH11573-13	JX573118
Ixodida	Ixodidae	*Amblyomma maculatum*	MIONB499-11	KM839245
Ixodida	Ixodidae	*Amblyomma sphenodonti*	GBCH1453-06	DQ507239
Ixodida	Ixodidae	*Amblyomma sphenodonti*	GBCH1454-06	DQ507240
Ixodida	Ixodidae	*Amblyomma sphenodonti*	GBCH1455-06	DQ507241
Ixodida	Ixodidae	*Amblyomma sphenodonti*	GBCH1456-06	DQ507242
Ixodida	Ixodidae	*Amblyomma sphenodonti*	GBCH1457-06	DQ507243
Ixodida	Ixodidae	*Amblyomma sphenodonti*	GBCH1458-06	DQ507244
Ixodida	Ixodidae	*Amblyomma triguttatum*	GBCH0033-06	AB113317
Ixodida	Ixodidae	*Amblyomma variegatum*	GBCH5183-10	GU062743
Ixodida	Ixodidae	*Bothriocroton hydrosauri*	GBCH4086-09	FJ584427
Ixodida	Ixodidae	*Bothriocroton hydrosauri*	GBCH4087-09	FJ584426
Ixodida	Ixodidae	*Bothriocroton hydrosauri*	GBCH4088-09	FJ584425
Ixodida	Ixodidae	*Bothriocroton hydrosauri*	GBCH4089-09	FJ584424
Ixodida	Ixodidae	*Bothriocroton hydrosauri*	GBCH4090-09	FJ584423
Ixodida	Ixodidae	*Bothriocroton hydrosauri*	GBCH4091-09	FJ584422
Ixodida	Ixodidae	*Dermacentor marginatus*	GACAC1323-12	JQ625696
Ixodida	Ixodidae	*Dermacentor marginatus*	GACAC1325-12	JQ625698
Ixodida	Ixodidae	*Dermacentor marginatus*	GACAC144-12	FN394327
Ixodida	Ixodidae	*Dermacentor marginatus*	GBCH0078-06	AF132828
Ixodida	Ixodidae	*Dermacentor nitens*	GBCH0351-06	AY008679
Ixodida	Ixodidae	*Dermacentor reticulatus*	GBCH0079-06	AF132829
Ixodida	Ixodidae	*Dermacentor rhinocerinus*	GBCH0080-06	AF132830
Ixodida	Ixodidae	*Dermacentor variabilis*	GBCH0081-06	AF132831
Ixodida	Ixodidae	*Haemaphysalis concinna*	GBCH11614-13	JX394183
Ixodida	Ixodidae	*Haemaphysalis concinna*	GBCH11615-13	JX394182
Ixodida	Ixodidae	*Haemaphysalis concinna*	GBCH11616-13	JX394181
Ixodida	Ixodidae	*Haemaphysalis concinna*	GBCH11617-13	JX394180
Ixodida	Ixodidae	*Haemaphysalis concinna*	GBCH11618-13	JX394179
Ixodida	Ixodidae	*Haemaphysalis flava*	GACAC1315-12	JQ625688
Ixodida	Ixodidae	*Haemaphysalis flava*	GACAC1316-12	JQ625689
Ixodida	Ixodidae	*Haemaphysalis flava*	GACAC589-12	JF758632
Ixodida	Ixodidae	*Haemaphysalis flava*	GBCH0012-06	AB075954
Ixodida	Ixodidae	*Haemaphysalis flava*	GBCH1680-06	NC_005292
Ixodida	Ixodidae	*Haemaphysalis formosensis*	GBCH10807-13	NC_020334
Ixodida	Ixodidae	*Haemaphysalis formosensis*	GBCH11572-13	JX573135
Ixodida	Ixodidae	*Haemaphysalis humerosa*	GBCH0069-06	AF132819

Ixodida	Ixodidae	*Haemaphysalis humerosa*	GBCH11569-13	JX573138
Ixodida	Ixodidae	*Haemaphysalis hystricis*	GBCH11570-13	JX573137
Ixodida	Ixodidae	*Haemaphysalis longicornis*	GACAC1318-12	JQ625691
Ixodida	Ixodidae	*Haemaphysalis longicornis*	GACAC1319-12	JQ625692
Ixodida	Ixodidae	*Haemaphysalis longicornis*	GACAC1320-12	JQ625693
Ixodida	Ixodidae	*Haemaphysalis longicornis*	GACAC1321-12	JQ625694
Ixodida	Ixodidae	*Haemaphysalis longicornis*	GACAC1322-12	JQ625695
Ixodida	Ixodidae	*Haemaphysalis longicornis*	GBCH0070-06	AF132820
Ixodida	Ixodidae	*Haemaphysalis longicornis*	GBCH12037-13	JF758635
Ixodida	Ixodidae	*Haemaphysalis longicornis*	GBCH12039-13	JF758631
Ixodida	Ixodidae	*Haemaphysalis longicornis*	GBCH7607-13	JQ346687
Ixodida	Ixodidae	*Haemaphysalis longicornis*	GBCH7608-13	JQ346686
Ixodida	Ixodidae	*Haemaphysalis parva*	GBCH10806-13	NC_020335
Ixodida	Ixodidae	*Haemaphysalis parva*	GBCH11571-13	JX573136
Ixodida	Ixodidae	*Hyalomma aegyptium*	GBCH0071-06	AF132821
Ixodida	Ixodidae	*Hyalomma detritum*	GBCH4209-09	EU827696
Ixodida	Ixodidae	*Hyalomma detritum*	GBCH4210-09	EU827695
Ixodida	Ixodidae	*Hyalomma detritum*	GBCH4211-09	EU827694
Ixodida	Ixodidae	*Hyalomma dromedarii*	GBCH0072-06	AF132822
Ixodida	Ixodidae	*Hyalomma dromedarii*	GBCH0251-06	AJ437061
Ixodida	Ixodidae	*Hyalomma dromedarii*	GBCH0252-06	AJ437062
Ixodida	Ixodidae	*Hyalomma dromedarii*	GBCH0253-06	AJ437063
Ixodida	Ixodidae	*Hyalomma dromedarii*	GBCH0254-06	AJ437064
Ixodida	Ixodidae	*Hyalomma dromedarii*	GBCH0255-06	AJ437065
Ixodida	Ixodidae	*Hyalomma dromedarii*	GBCH0256-06	AJ437066
Ixodida	Ixodidae	*Hyalomma dromedarii*	GBCH0257-06	AJ437067
Ixodida	Ixodidae	*Hyalomma dromedarii*	GBCH0258-06	AJ437068
Ixodida	Ixodidae	*Hyalomma dromedarii*	GBCH0259-06	AJ437069
Ixodida	Ixodidae	*Hyalomma dromedarii*	GBCH0260-06	AJ437070
Ixodida	Ixodidae	*Hyalomma dromedarii*	GBCH0261-06	AJ437071
Ixodida	Ixodidae	*Hyalomma dromedarii*	GBCH0262-06	AJ437072
Ixodida	Ixodidae	*Hyalomma dromedarii*	GBCH0263-06	AJ437073
Ixodida	Ixodidae	*Hyalomma dromedarii*	GBCH0264-06	AJ437074
Ixodida	Ixodidae	*Hyalomma dromedarii*	GBCH0265-06	AJ437075
Ixodida	Ixodidae	*Hyalomma dromedarii*	GBCH0266-06	AJ437076
Ixodida	Ixodidae	*Hyalomma dromedarii*	GBCH0267-06	AJ437077
Ixodida	Ixodidae	*Hyalomma dromedarii*	GBCH0268-06	AJ437078
Ixodida	Ixodidae	*Hyalomma dromedarii*	GBCH0269-06	AJ437079
Ixodida	Ixodidae	*Hyalomma dromedarii*	GBCH0270-06	AJ437080
Ixodida	Ixodidae	*Hyalomma dromedarii*	GBCH0271-06	AJ437081
Ixodida	Ixodidae	*Hyalomma dromedarii*	GBCH0272-06	AJ437082
Ixodida	Ixodidae	*Hyalomma dromedarii*	GBCH0273-06	AJ437083
Ixodida	Ixodidae	*Hyalomma lusitanicum*	GBCH4162-09	EU827743
Ixodida	Ixodidae	*Hyalomma lusitanicum*	GBCH4163-09	EU827742
Ixodida	Ixodidae	*Hyalomma lusitanicum*	GBCH4164-09	EU827741
Ixodida	Ixodidae	*Hyalomma lusitanicum*	GBCH4165-09	EU827740
Ixodida	Ixodidae	*Hyalomma lusitanicum*	GBCH4166-09	EU827739
Ixodida	Ixodidae	*Hyalomma lusitanicum*	GBCH4167-09	EU827738
Ixodida	Ixodidae	*Hyalomma lusitanicum*	GBCH4168-09	EU827737
Ixodida	Ixodidae	*Hyalomma lusitanicum*	GBCH4169-09	EU827736
Ixodida	Ixodidae	*Hyalomma lusitanicum*	GBCH4170-09	EU827735
Ixodida	Ixodidae	*Hyalomma lusitanicum*	GBCH4171-09	EU827734
Ixodida	Ixodidae	*Hyalomma lusitanicum*	GBCH4172-09	EU827733

Ixodida	Ixodidae	*Hyalomma lusitanicum*	GBCH4173-09	EU827732
Ixodida	Ixodidae	*Hyalomma lusitanicum*	GBCH4174-09	EU827731
Ixodida	Ixodidae	*Hyalomma lusitanicum*	GBCH4175-09	EU827730
Ixodida	Ixodidae	*Hyalomma lusitanicum*	GBCH4176-09	EU827729
Ixodida	Ixodidae	*Hyalomma lusitanicum*	GBCH4177-09	EU827728
Ixodida	Ixodidae	*Hyalomma lusitanicum*	GBCH4178-09	EU827727
Ixodida	Ixodidae	*Hyalomma lusitanicum*	GBCH4179-09	EU827726
Ixodida	Ixodidae	*Hyalomma lusitanicum*	GBCH4180-09	EU827725
Ixodida	Ixodidae	*Hyalomma lusitanicum*	GBCH4181-09	EU827724
Ixodida	Ixodidae	*Hyalomma lusitanicum*	GBCH4182-09	EU827723
Ixodida	Ixodidae	*Hyalomma lusitanicum*	GBCH4183-09	EU827722
Ixodida	Ixodidae	*Hyalomma lusitanicum*	GBCH4184-09	EU827721
Ixodida	Ixodidae	*Hyalomma lusitanicum*	GBCH4185-09	EU827720
Ixodida	Ixodidae	*Hyalomma lusitanicum*	GBCH4186-09	EU827719
Ixodida	Ixodidae	*Hyalomma lusitanicum*	GBCH4187-09	EU827718
Ixodida	Ixodidae	*Hyalomma lusitanicum*	GBCH4188-09	EU827717
Ixodida	Ixodidae	*Hyalomma lusitanicum*	GBCH4189-09	EU827716
Ixodida	Ixodidae	*Hyalomma lusitanicum*	GBCH4190-09	EU827715
Ixodida	Ixodidae	*Hyalomma lusitanicum*	GBCH4191-09	EU827714
Ixodida	Ixodidae	*Hyalomma lusitanicum*	GBCH4192-09	EU827713
Ixodida	Ixodidae	*Hyalomma lusitanicum*	GBCH4193-09	EU827712
Ixodida	Ixodidae	*Hyalomma lusitanicum*	GBCH4194-09	EU827711
Ixodida	Ixodidae	*Hyalomma lusitanicum*	GBCH4195-09	EU827710
Ixodida	Ixodidae	*Hyalomma lusitanicum*	GBCH4196-09	EU827709
Ixodida	Ixodidae	*Hyalomma lusitanicum*	GBCH4197-09	EU827708
Ixodida	Ixodidae	*Hyalomma lusitanicum*	GBCH4198-09	EU827707
Ixodida	Ixodidae	*Hyalomma lusitanicum*	GBCH4199-09	EU827706
Ixodida	Ixodidae	*Hyalomma lusitanicum*	GBCH4200-09	EU827705
Ixodida	Ixodidae	*Hyalomma lusitanicum*	GBCH4201-09	EU827704
Ixodida	Ixodidae	*Hyalomma lusitanicum*	GBCH4202-09	EU827703
Ixodida	Ixodidae	*Hyalomma lusitanicum*	GBCH4203-09	EU827702
Ixodida	Ixodidae	*Hyalomma lusitanicum*	GBCH4204-09	EU827701
Ixodida	Ixodidae	*Hyalomma lusitanicum*	GBCH4205-09	EU827700
Ixodida	Ixodidae	*Hyalomma lusitanicum*	GBCH4206-09	EU827699
Ixodida	Ixodidae	*Hyalomma lusitanicum*	GBCH4207-09	EU827698
Ixodida	Ixodidae	*Hyalomma lusitanicum*	GBCH4208-09	EU827697
Ixodida	Ixodidae	*Hyalomma marginatum*	GBCH0073-06	AF132823
Ixodida	Ixodidae	*Hyalomma marginatum*	GBCH0281-06	AJ437091
Ixodida	Ixodidae	*Hyalomma marginatum*	GBCH0282-06	AJ437092
Ixodida	Ixodidae	*Hyalomma marginatum*	GBCH0283-06	AJ437093
Ixodida	Ixodidae	*Hyalomma marginatum*	GBCH0284-06	AJ437094
Ixodida	Ixodidae	*Hyalomma marginatum*	GBCH0285-06	AJ437095
Ixodida	Ixodidae	*Hyalomma marginatum*	GBCH0286-06	AJ437096
Ixodida	Ixodidae	*Hyalomma marginatum*	GBCH0287-06	AJ437097
Ixodida	Ixodidae	*Hyalomma marginatum*	GBCH0288-06	AJ437098
Ixodida	Ixodidae	*Hyalomma marginatum*	GBCH0289-06	AJ437099
Ixodida	Ixodidae	*Hyalomma marginatum*	GBCH0290-06	AJ437100
Ixodida	Ixodidae	*Hyalomma marginatum*	GBCH0291-06	AJ437101
Ixodida	Ixodidae	*Hyalomma marginatum*	GBCH4212-09	EU827693
Ixodida	Ixodidae	*Hyalomma marginatum*	GBCH4213-09	EU827692
Ixodida	Ixodidae	*Hyalomma truncatum*	GBCH0074-06	AF132824
Ixodida	Ixodidae	*Hyalomma truncatum*	GBCH0274-06	AJ437084
Ixodida	Ixodidae	*Hyalomma truncatum*	GBCH0275-06	AJ437085

Ixodida	Ixodidae	*Hyalomma truncatum*	GBCH0276-06	AJ437086
Ixodida	Ixodidae	*Hyalomma truncatum*	GBCH0277-06	AJ437087
Ixodida	Ixodidae	*Hyalomma truncatum*	GBCH0278-06	AJ437088
Ixodida	Ixodidae	*Hyalomma truncatum*	GBCH0279-06	AJ437089
Ixodida	Ixodidae	*Hyalomma truncatum*	GBCH0280-06	AJ437090
Ixodida	Ixodidae	*Ixodes acutitarsus*	GBCH0031-06	AB105166
Ixodida	Ixodidae	*Ixodes arboricola*	GBCH11602-13	JX394195
Ixodida	Ixodidae	*Ixodes arboricola*	GBCH11603-13	JX394194
Ixodida	Ixodidae	*Ixodes asanumai*	GBCH0057-06	AB231674
Ixodida	Ixodidae	*Ixodes cornuatus*	GBCH3215-09	FJ571511
Ixodida	Ixodidae	*Ixodes cornuatus*	GBCH6958-13	HM545846
Ixodida	Ixodidae	*Ixodes cornuatus*	GBCH6959-13	HM545845
Ixodida	Ixodidae	*Ixodes cornuatus*	GBCH6960-13	HM545844
Ixodida	Ixodidae	*Ixodes cornuatus*	GBCH6961-13	HM545843
Ixodida	Ixodidae	*Ixodes cornuatus*	GBCH6962-13	HM545842
Ixodida	Ixodidae	*Ixodes cornuatus*	GBCH6977-13	HM545827
Ixodida	Ixodidae	*Ixodes cornuatus*	GBCH6978-13	HM545826
Ixodida	Ixodidae	*Ixodes cornuatus*	GBCH6979-13	HM545825
Ixodida	Ixodidae	*Ixodes cornuatus*	GBCH6980-13	HM545824
Ixodida	Ixodidae	*Ixodes cornuatus*	GBCH6981-13	HM545823
Ixodida	Ixodidae	*Ixodes granulatus*	GACAC1313-12	JQ625686
Ixodida	Ixodidae	*Ixodes granulatus*	GACAC1317-12	JQ625690
Ixodida	Ixodidae	*Ixodes granulatus*	GBCH0056-06	AB231673
Ixodida	Ixodidae	*Ixodes granulatus*	GBCH12038-13	JF758633
Ixodida	Ixodidae	*Ixodes hexagonus*	CYTC4101-12	AF081828
Ixodida	Ixodidae	*Ixodes holocyclus*	GBCH0013-06	AB075955
Ixodida	Ixodidae	*Ixodes holocyclus*	GBCH6963-13	HM545841
Ixodida	Ixodidae	*Ixodes holocyclus*	GBCH6964-13	HM545840
Ixodida	Ixodidae	*Ixodes holocyclus*	GBCH6965-13	HM545839
Ixodida	Ixodidae	*Ixodes holocyclus*	GBCH6966-13	HM545838
Ixodida	Ixodidae	*Ixodes holocyclus*	GBCH6967-13	HM545837
Ixodida	Ixodidae	*Ixodes holocyclus*	GBCH6968-13	HM545836
Ixodida	Ixodidae	*Ixodes holocyclus*	GBCH6969-13	HM545835
Ixodida	Ixodidae	*Ixodes holocyclus*	GBCH6970-13	HM545834
Ixodida	Ixodidae	*Ixodes holocyclus*	GBCH6971-13	HM545833
Ixodida	Ixodidae	*Ixodes holocyclus*	GBCH6972-13	HM545832
Ixodida	Ixodidae	*Ixodes holocyclus*	GBCH6973-13	HM545831
Ixodida	Ixodidae	*Ixodes holocyclus*	GBCH6974-13	HM545830
Ixodida	Ixodidae	*Ixodes holocyclus*	GBCH6975-13	HM545829
Ixodida	Ixodidae	*Ixodes holocyclus*	GBCH6976-13	HM545828
Ixodida	Ixodidae	*Ixodes holocyclus*	GBCH6982-13	HM545822
Ixodida	Ixodidae	*Ixodes holocyclus*	GBCH6983-13	HM545821
Ixodida	Ixodidae	*Ixodes holocyclus*	GBCH6984-13	HM545820
Ixodida	Ixodidae	*Ixodes holocyclus*	GBCH6985-13	HM545819
Ixodida	Ixodidae	*Ixodes holocyclus*	GBCH6986-13	HM545818
Ixodida	Ixodidae	*Ixodes holocyclus*	GBCH6987-13	HM545817
Ixodida	Ixodidae	*Ixodes holocyclus*	GBCH6988-13	HM545816
Ixodida	Ixodidae	*Ixodes holocyclus*	GBCH6989-13	HM545815
Ixodida	Ixodidae	*Ixodes holocyclus*	GBCH6990-13	HM545814
Ixodida	Ixodidae	*Ixodes holocyclus*	GBCH6991-13	HM545813
Ixodida	Ixodidae	*Ixodes holocyclus*	GBCH6992-13	HM545812
Ixodida	Ixodidae	*Ixodes holocyclus*	GBCH6993-13	HM545811
Ixodida	Ixodidae	*Ixodes holocyclus*	GBCH6994-13	HM545810

Ixodida	Ixodidae	*Ixodes holocyclus*	GBCH6995-13	HM545809
Ixodida	Ixodidae	*Ixodes holocyclus*	GBCH6996-13	HM545808
Ixodida	Ixodidae	*Ixodes holocyclus*	GBCH6997-13	HM545807
Ixodida	Ixodidae	*Ixodes holocyclus*	GBCH6998-13	HM545806
Ixodida	Ixodidae	*Ixodes holocyclus*	GBCH6999-13	HM545805
Ixodida	Ixodidae	*Ixodes holocyclus*	GBCH7000-13	HM545804
Ixodida	Ixodidae	*Ixodes holocyclus*	GBCH7001-13	HM545803
Ixodida	Ixodidae	*Ixodes holocyclus*	GBCH7002-13	HM545802
Ixodida	Ixodidae	*Ixodes holocyclus*	GBCH7003-13	HM545801
Ixodida	Ixodidae	*Ixodes holocyclus*	GBCH7004-13	HM545800
Ixodida	Ixodidae	*Ixodes holocyclus*	GBCH7005-13	HM545799
Ixodida	Ixodidae	*Ixodes holocyclus*	GBCH7006-13	HM545798
Ixodida	Ixodidae	*Ixodes holocyclus*	GBCH7007-13	HM545797
Ixodida	Ixodidae	*Ixodes holocyclus*	GBCH7008-13	HM545796
Ixodida	Ixodidae	*Ixodes holocyclus*	GBCH7009-13	HM545795
Ixodida	Ixodidae	*Ixodes holocyclus*	GBCH7010-13	HM545794
Ixodida	Ixodidae	*Ixodes holocyclus*	GBCH7011-13	HM545793
Ixodida	Ixodidae	*Ixodes holocyclus*	GBCH7012-13	HM545792
Ixodida	Ixodidae	*Ixodes holocyclus*	GBCH7013-13	HM545791
Ixodida	Ixodidae	*Ixodes holocyclus*	GBCH7014-13	HM545790
Ixodida	Ixodidae	*Ixodes holocyclus*	GBCH7015-13	HM545789
Ixodida	Ixodidae	*Ixodes holocyclus*	GBCH7016-13	HM545788
Ixodida	Ixodidae	*Ixodes holocyclus*	GBCH7017-13	HM545787
Ixodida	Ixodidae	*Ixodes holocyclus*	GBCH7018-13	HM545786
Ixodida	Ixodidae	*Ixodes holocyclus*	GBCH7019-13	HM545785
Ixodida	Ixodidae	*Ixodes holocyclus*	GBCH7020-13	HM545784
Ixodida	Ixodidae	*Ixodes holocyclus*	GBCH7021-13	HM545783
Ixodida	Ixodidae	*Ixodes holocyclus*	GBCH7022-13	HM545782
Ixodida	Ixodidae	*Ixodes holocyclus*	GBCH7023-13	HM545781
Ixodida	Ixodidae	*Ixodes holocyclus*	GBCH7024-13	HM545780
Ixodida	Ixodidae	*Ixodes holocyclus*	GBCH7025-13	HM545779
Ixodida	Ixodidae	*Ixodes holocyclus*	GBCH7026-13	HM545778
Ixodida	Ixodidae	*Ixodes holocyclus*	GBCH7027-13	HM545777
Ixodida	Ixodidae	*Ixodes holocyclus*	GBCH7028-13	HM545776
Ixodida	Ixodidae	*Ixodes holocyclus*	GBCH7029-13	HM545775
Ixodida	Ixodidae	*Ixodes holocyclus*	GBCH7030-13	HM545774
Ixodida	Ixodidae	*Ixodes holocyclus*	GBCH7031-13	HM545773
Ixodida	Ixodidae	*Ixodes holocyclus*	GBCH7032-13	HM545772
Ixodida	Ixodidae	*Ixodes holocyclus*	GBCH7033-13	HM545771
Ixodida	Ixodidae	*Ixodes holocyclus*	GBCH7034-13	HM545770
Ixodida	Ixodidae	*Ixodes holocyclus*	GBCH7035-13	HM545769
Ixodida	Ixodidae	*Ixodes holocyclus*	GBCH7036-13	HM545768
Ixodida	Ixodidae	*Ixodes holocyclus*	GBCH7037-13	HM545767
Ixodida	Ixodidae	*Ixodes holocyclus*	GBCH7038-13	HM545766
Ixodida	Ixodidae	*Ixodes holocyclus*	GBCH7039-13	HM545765
Ixodida	Ixodidae	*Ixodes holocyclus*	GBCH7040-13	HM545764
Ixodida	Ixodidae	*Ixodes holocyclus*	GBCH7041-13	HM545763
Ixodida	Ixodidae	*Ixodes holocyclus*	GBCH7042-13	HM545762
Ixodida	Ixodidae	*Ixodes holocyclus*	GBCH7043-13	HM545761
Ixodida	Ixodidae	*Ixodes holocyclus*	GBCH7044-13	HM545760
Ixodida	Ixodidae	*Ixodes holocyclus*	GBCH7045-13	HM545759
Ixodida	Ixodidae	*Ixodes holocyclus*	GBCH7046-13	HM545758
Ixodida	Ixodidae	*Ixodes holocyclus*	GBCH7047-13	HM545757

Ixodida	Ixodidae	*Ixodes holocyclus*	GBCH7048-13	HM545756
Ixodida	Ixodidae	*Ixodes holocyclus*	GBCH7049-13	HM545755
Ixodida	Ixodidae	*Ixodes holocyclus*	GBCH7050-13	HM545754
Ixodida	Ixodidae	*Ixodes holocyclus*	GBCH7051-13	HM545753
Ixodida	Ixodidae	*Ixodes holocyclus*	GBCH7052-13	HM545752
Ixodida	Ixodidae	*Ixodes holocyclus*	GBCH7053-13	HM545751
Ixodida	Ixodidae	*Ixodes holocyclus*	GBCH7054-13	HM545750
Ixodida	Ixodidae	*Ixodes holocyclus*	GBCH7055-13	HM545749
Ixodida	Ixodidae	*Ixodes holocyclus*	GBCH7056-13	HM545748
Ixodida	Ixodidae	*Ixodes lividus*	GACAC525-12	GU124743
Ixodida	Ixodidae	*Ixodes monospinosus*	GBCH0055-06	AB231672
Ixodida	Ixodidae	*Ixodes nipponensis*	GBCH0054-06	AB231671
Ixodida	Ixodidae	*Ixodes ovatus*	GBCH0053-06	AB231670
Ixodida	Ixodidae	*Ixodes pavlovskyi*	GBCH0052-06	AB231669
Ixodida	Ixodidae	*Ixodes pavlovskyi*	GBCH11665-13	JX288763
Ixodida	Ixodidae	*Ixodes pavlovskyi*	GBCH12108-13	KC688417
Ixodida	Ixodidae	*Ixodes pavlovskyi*	GBCH12109-13	KC688416
Ixodida	Ixodidae	*Ixodes pavlovskyi*	GBCH12110-13	KC688415
Ixodida	Ixodidae	*Ixodes pavlovskyi*	GBCH12111-13	KC688414
Ixodida	Ixodidae	*Ixodes pavlovskyi*	GBCH12112-13	KC688413
Ixodida	Ixodidae	*Ixodes pavlovskyi*	GBCH12113-13	KC688412
Ixodida	Ixodidae	*Ixodes pavlovskyi*	GBCH12114-13	KC688411
Ixodida	Ixodidae	*Ixodes pavlovskyi*	GBCH12115-13	KC688410
Ixodida	Ixodidae	*Ixodes pavlovskyi*	GBCH12116-13	KC688409
Ixodida	Ixodidae	*Ixodes pavlovskyi*	GBCH12117-13	KC688408
Ixodida	Ixodidae	*Ixodes pavlovskyi*	GBCH12118-13	KC688407
Ixodida	Ixodidae	*Ixodes pavlovskyi*	GBCH12119-13	KC688406
Ixodida	Ixodidae	*Ixodes pavlovskyi*	GBCH7603-13	JQ823025
Ixodida	Ixodidae	*Ixodes pavlovskyi*	GBCH7604-13	JQ823024
Ixodida	Ixodidae	*Ixodes persulcatus*	GACAC1314-12	JQ625687
Ixodida	Ixodidae	*Ixodes persulcatus*	GACAC1324-12	JQ625697
Ixodida	Ixodidae	*Ixodes persulcatus*	GBCH0010-06	AB073725
Ixodida	Ixodidae	*Ixodes persulcatus*	GBCH11664-13	JX288764
Ixodida	Ixodidae	*Ixodes persulcatus*	GBCH12040-13	JF758629
Ixodida	Ixodidae	*Ixodes persulcatus*	GBCH12120-13	KC688405
Ixodida	Ixodidae	*Ixodes persulcatus*	GBCH12121-13	KC688404
Ixodida	Ixodidae	*Ixodes persulcatus*	GBCH12122-13	KC688403
Ixodida	Ixodidae	*Ixodes persulcatus*	GBCH12123-13	KC688402
Ixodida	Ixodidae	*Ixodes persulcatus*	GBCH12124-13	KC688401
Ixodida	Ixodidae	*Ixodes persulcatus*	GBCH12125-13	KC688400
Ixodida	Ixodidae	*Ixodes persulcatus*	GBCH12126-13	KC688399
Ixodida	Ixodidae	*Ixodes persulcatus*	GBCH12127-13	KC688398
Ixodida	Ixodidae	*Ixodes persulcatus*	GBCH12128-13	KC688397
Ixodida	Ixodidae	*Ixodes persulcatus*	GBCH12129-13	KC688396
Ixodida	Ixodidae	*Ixodes persulcatus*	GBCH12130-13	KC688395
Ixodida	Ixodidae	*Ixodes persulcatus*	GBCH12131-13	KC688394
Ixodida	Ixodidae	*Ixodes persulcatus*	GBCH12132-13	KC688393
Ixodida	Ixodidae	*Ixodes persulcatus*	GBCH12133-13	KC688392
Ixodida	Ixodidae	*Ixodes persulcatus*	GBCH12134-13	KC688391
Ixodida	Ixodidae	*Ixodes persulcatus*	GBCH12135-13	KC688390
Ixodida	Ixodidae	*Ixodes persulcatus*	GBCH12136-13	KC688389
Ixodida	Ixodidae	*Ixodes persulcatus*	GBCH12137-13	KC688388
Ixodida	Ixodidae	*Ixodes persulcatus*	GBCH12138-13	KC688387

Ixodida	Ixodidae	*Ixodes persulcatus*	GBCH12139-13	KC688386
Ixodida	Ixodidae	*Ixodes persulcatus*	GBCH12140-13	KC688385
Ixodida	Ixodidae	*Ixodes persulcatus*	GBCH12141-13	KC688384
Ixodida	Ixodidae	*Ixodes persulcatus*	GBCH12142-13	KC688383
Ixodida	Ixodidae	*Ixodes persulcatus*	GBCH12143-13	KC688382
Ixodida	Ixodidae	*Ixodes persulcatus*	GBCH12144-13	KC688381
Ixodida	Ixodidae	*Ixodes persulcatus*	GBCH12145-13	KC688380
Ixodida	Ixodidae	*Ixodes persulcatus*	GBCH12146-13	KC688379
Ixodida	Ixodidae	*Ixodes persulcatus*	GBCH12147-13	KC688378
Ixodida	Ixodidae	*Ixodes persulcatus*	GBCH12148-13	KC688377
Ixodida	Ixodidae	*Ixodes persulcatus*	GBCH12149-13	KC688376
Ixodida	Ixodidae	*Ixodes persulcatus*	GBCH12150-13	KC688375
Ixodida	Ixodidae	*Ixodes persulcatus*	GBCH12151-13	KC688374
Ixodida	Ixodidae	*Ixodes persulcatus*	GBCH12152-13	KC688373
Ixodida	Ixodidae	*Ixodes persulcatus*	GBCH12153-13	KC688372
Ixodida	Ixodidae	*Ixodes persulcatus*	GBCH12154-13	KC688371
Ixodida	Ixodidae	*Ixodes persulcatus*	GBCH12155-13	KC688370
Ixodida	Ixodidae	*Ixodes persulcatus*	GBCH12156-13	KC688369
Ixodida	Ixodidae	*Ixodes persulcatus*	GBCH12157-13	KC688368
Ixodida	Ixodidae	*Ixodes persulcatus*	GBCH12158-13	KC688367
Ixodida	Ixodidae	*Ixodes persulcatus*	GBCH12159-13	KC688366
Ixodida	Ixodidae	*Ixodes persulcatus*	GBCH12160-13	KC688365
Ixodida	Ixodidae	*Ixodes persulcatus*	GBCH12161-13	KC688364
Ixodida	Ixodidae	*Ixodes philipi*	GBCH0046-06	AB231663
Ixodida	Ixodidae	*Ixodes philipi*	GBCH0047-06	AB231664
Ixodida	Ixodidae	*Ixodes philipi*	GBCH0048-06	AB231665
Ixodida	Ixodidae	*Ixodes philipi*	GBCH0049-06	AB231666
Ixodida	Ixodidae	*Ixodes redikorzevi*	GBCH11593-13	JX394204
Ixodida	Ixodidae	*Ixodes redikorzevi*	GBCH11594-13	JX394203
Ixodida	Ixodidae	*Ixodes redikorzevi*	GBCH11595-13	JX394202
Ixodida	Ixodidae	*Ixodes redikorzevi*	GBCH11596-13	JX394201
Ixodida	Ixodidae	*Ixodes redikorzevi*	GBCH11597-13	JX394200
Ixodida	Ixodidae	*Ixodes ricinus*	GBCH11333-13	NC_018369
Ixodida	Ixodidae	*Ixodes ricinus*	GBCH11429-13	JX983208
Ixodida	Ixodidae	*Ixodes scapularis*	ETBM003-11	JQ350479
Ixodida	Ixodidae	*Ixodes scapularis*	GBCH12421-13	KC488314
Ixodida	Ixodidae	*Ixodes scapularis*	GBCH12422-13	KC488313
Ixodida	Ixodidae	*Ixodes scapularis*	GBCH12423-13	KC488312
Ixodida	Ixodidae	*Ixodes scapularis*	GBCH12424-13	KC488311
Ixodida	Ixodidae	*Ixodes scapularis*	GBCH12425-13	KC488310
Ixodida	Ixodidae	*Ixodes scapularis*	GBCH12426-13	KC488309
Ixodida	Ixodidae	*Ixodes scapularis*	GBCH12427-13	KC488308
Ixodida	Ixodidae	*Ixodes scapularis*	GBCH12428-13	KC488307
Ixodida	Ixodidae	*Ixodes scapularis*	GBCH12429-13	KC488306
Ixodida	Ixodidae	*Ixodes scapularis*	GBCH12430-13	KC488305
Ixodida	Ixodidae	*Ixodes scapularis*	GBCH12431-13	KC488304
Ixodida	Ixodidae	*Ixodes scapularis*	GBCH12432-13	KC488303
Ixodida	Ixodidae	*Ixodes scapularis*	GBCH12433-13	KC488302
Ixodida	Ixodidae	*Ixodes scapularis*	GBCH12434-13	KC488301
Ixodida	Ixodidae	*Ixodes scapularis*	GBCH12435-13	KC488300
Ixodida	Ixodidae	*Ixodes scapularis*	GBCH12436-13	KC488299
Ixodida	Ixodidae	*Ixodes scapularis*	GBCH12437-13	KC488298
Ixodida	Ixodidae	*Ixodes scapularis*	GBCH12438-13	KC488297

Ixodida	Ixodidae	*Ixodes scapularis*	GBCH12439-13	KC488296
Ixodida	Ixodidae	*Ixodes scapularis*	GBCH12440-13	KC488295
Ixodida	Ixodidae	*Ixodes scapularis*	GBCH12441-13	KC488294
Ixodida	Ixodidae	*Ixodes scapularis*	GBCH12442-13	KC488293
Ixodida	Ixodidae	*Ixodes scapularis*	GBCH12443-13	KC488292
Ixodida	Ixodidae	*Ixodes scapularis*	GBCH12444-13	KC488291
Ixodida	Ixodidae	*Ixodes scapularis*	GBCH12445-13	KC488290
Ixodida	Ixodidae	*Ixodes scapularis*	GBCH12446-13	KC488289
Ixodida	Ixodidae	*Ixodes scapularis*	GBCH12447-13	KC488288
Ixodida	Ixodidae	*Ixodes scapularis*	GBCH12448-13	KC488287
Ixodida	Ixodidae	*Ixodes scapularis*	GBCH12449-13	KC488286
Ixodida	Ixodidae	*Ixodes scapularis*	GBCH12450-13	KC488285
Ixodida	Ixodidae	*Ixodes scapularis*	GBCH12451-13	KC488284
Ixodida	Ixodidae	*Ixodes scapularis*	GBCH12452-13	KC488283
Ixodida	Ixodidae	*Ixodes scapularis*	GBCH12453-13	KC488282
Ixodida	Ixodidae	*Ixodes scapularis*	GBCH12454-13	KC488281
Ixodida	Ixodidae	*Ixodes scapularis*	GBCH12455-13	KC488280
Ixodida	Ixodidae	*Ixodes scapularis*	GBCH12456-13	KC488279
Ixodida	Ixodidae	*Ixodes scapularis*	GBCH12457-13	KC488278
Ixodida	Ixodidae	*Ixodes scapularis*	GBCH12458-13	KC488277
Ixodida	Ixodidae	*Ixodes scapularis*	GBCH12459-13	KC488276
Ixodida	Ixodidae	*Ixodes scapularis*	GBCH12460-13	KC488275
Ixodida	Ixodidae	*Ixodes scapularis*	GBCH12461-13	KC488274
Ixodida	Ixodidae	*Ixodes scapularis*	GBCH12462-13	KC488273
Ixodida	Ixodidae	*Ixodes scapularis*	GBCH12463-13	KC488272
Ixodida	Ixodidae	*Ixodes scapularis*	GBCH12464-13	KC488271
Ixodida	Ixodidae	*Ixodes scapularis*	GBCH12465-13	KC488270
Ixodida	Ixodidae	*Ixodes scapularis*	GBCH12466-13	KC488269
Ixodida	Ixodidae	*Ixodes scapularis*	GBCH12467-13	KC488268
Ixodida	Ixodidae	*Ixodes scapularis*	GBCH12468-13	KC488267
Ixodida	Ixodidae	*Ixodes scapularis*	GBCH12469-13	KC488266
Ixodida	Ixodidae	*Ixodes scapularis*	GBCH12470-13	KC488265
Ixodida	Ixodidae	*Ixodes scapularis*	GBCH12471-13	KC488264
Ixodida	Ixodidae	*Ixodes scapularis*	GBCH12472-13	KC488263
Ixodida	Ixodidae	*Ixodes scapularis*	GBCH12473-13	KC488262
Ixodida	Ixodidae	*Ixodes scapularis*	GBCH12474-13	KC488261
Ixodida	Ixodidae	*Ixodes turdus*	GBCH0051-06	AB231668
Ixodida	Ixodidae	*Ixodes uriae*	GBCH0030-06	AB087746
Ixodida	Ixodidae	*Ixodes vespertilionis*	GBCH0050-06	AB231667
Ixodida	Ixodidae	*Nosomma monstrosum*	GBCH0082-06	AF132832
Ixodida	Ixodidae	*Rhipicephalus annulatus*	GBCH0075-06	AF132825
Ixodida	Ixodidae	*Rhipicephalus annulatus*	GBCH11619-13	JX422019
Ixodida	Ixodidae	*Rhipicephalus appendiculatus*	GBCH0083-06	AF132833
Ixodida	Ixodidae	*Rhipicephalus appendiculatus*	GBCH1812-07	DQ901357
Ixodida	Ixodidae	*Rhipicephalus compositus*	GBCH0084-06	AF132834
Ixodida	Ixodidae	*Rhipicephalus decoloratus*	GBCH0076-06	AF132826
Ixodida	Ixodidae	*Rhipicephalus evertsi*	GBCH0085-06	AF132835
Ixodida	Ixodidae	*Rhipicephalus evertsi*	GBCH0086-06	AF132836
Ixodida	Ixodidae	*Rhipicephalus geigyi*	GBCH0352-06	AY008680
Ixodida	Ixodidae	*Rhipicephalus maculatus*	GBCH0353-06	AY008681
Ixodida	Ixodidae	*Rhipicephalus microplus*	GBCH0077-06	AF132827
Ixodida	Ixodidae	*Rhipicephalus pravus*	GBCH0087-06	AF132837
Ixodida	Ixodidae	*Rhipicephalus pulchellus*	GBCH0354-06	AY008682

Ixodida	Ixodidae	*Rhipicephalus pumilio*	GBCH0356-06	AY008684
Ixodida	Ixodidae	*Rhipicephalus punctatus*	GBCH0088-06	AF132838
Ixodida	Ixodidae	*Rhipicephalus sanguineus*	CYTC4102-12	AF081829
Ixodida	Ixodidae	*Rhipicephalus sanguineus*	GACAC1308-12	JQ625681
Ixodida	Ixodidae	*Rhipicephalus sanguineus*	GACAC1309-12	JQ625682
Ixodida	Ixodidae	*Rhipicephalus sanguineus*	GACAC590-12	JF758634
Ixodida	Ixodidae	*Rhipicephalus sanguineus*	GBCH0089-06	AF132839
Ixodida	Ixodidae	*Rhipicephalus sanguineus*	GBCH11581-13	JX394216
Ixodida	Ixodidae	*Rhipicephalus sanguineus*	GBCH11582-13	JX394215
Ixodida	Ixodidae	*Rhipicephalus sanguineus*	GBCH11583-13	JX394214
Ixodida	Ixodidae	*Rhipicephalus sanguineus*	GBCH11584-13	JX394213
Ixodida	Ixodidae	*Rhipicephalus sanguineus*	GBCH11623-13	JX416325
Ixodida	Ixodidae	*Rhipicephalus simus*	GBCH0090-06	AF132840
Ixodida	Ixodidae	*Rhipicephalus turanicus*	GBCH0091-06	AF132841
Ixodida	Ixodidae	*Rhipicephalus turanicus*	GBCH12083-13	KF251021
Ixodida	Ixodidae	*Rhipicephalus turanicus*	GBCH12084-13	KF251020
Ixodida	Ixodidae	*Rhipicephalus turanicus*	GBCH12085-13	KF251019
Ixodida	Nuttalliellidae	*Nuttalliella namaqua*	GBCH11403-13	NC_019663
Ixodida	Nuttalliellidae	*Nuttalliella namaqua*	GBCH11428-13	JQ665719
Mesostigmata	Ameroseiidae		CHACA968-10	HM405834
Mesostigmata	Ameroseiidae		CNKOA291-14	KR070639
Mesostigmata	Ameroseiidae		CNPAF899-13	KM830526
Mesostigmata	Ameroseiidae		CNPAF900-13	KM826311
Mesostigmata	Ameroseiidae		CNPAF902-13	KM830755
Mesostigmata	Ameroseiidae		CNPAF903-13	KM840036
Mesostigmata	Ameroseiidae		CNPAF917-13	KM828503
Mesostigmata	Ameroseiidae		CNPAF919-13	KM834002
Mesostigmata	Ameroseiidae		JSAUG1784-12	KR070054
Mesostigmata	Ameroseiidae		JSAUG1799-12	KR069313
Mesostigmata	Ameroseiidae		JSAUG1821-12	KR069769
Mesostigmata	Ameroseiidae		JSAUG1852-12	KR069339
Mesostigmata	Ameroseiidae		JSAUG1857-12	KP979322
Mesostigmata	Ameroseiidae		JSOIE113-12	KR070123
Mesostigmata	Ameroseiidae		MYMCB190-11	JX837784
Mesostigmata	Ameroseiidae		MYMCG205-12	JX834760
Mesostigmata	Ameroseiidae		PHAUG1655-11	KR070336
Mesostigmata	Ameroseiidae		PHAUG1672-11	KP979162
Mesostigmata	Ameroseiidae		SSEIA3052-13	KM835331
Mesostigmata	Ameroseiidae		SSPAC12223-13	KM827070
Mesostigmata	Ameroseiidae		SSPAC12226-13	KM824656
Mesostigmata	Ameroseiidae		SSPAC12230-13	KM839612
Mesostigmata	Ameroseiidae		SSPAC12248-13	KM834241
Mesostigmata	Ameroseiidae		SSPAC2158-13	KM839086
Mesostigmata	Ameroseiidae		SSPAC7829-13	KM826442
Mesostigmata	Ameroseiidae		SSPAC7846-13	KM832061
Mesostigmata	Ameroseiidae		SSPAC7849-13	KM829726
Mesostigmata	Ameroseiidae		SSPAC7854-13	KM839209
Mesostigmata	Ameroseiidae		SSPAC7901-13	KM833944
Mesostigmata	Ameroseiidae		SSWEE129-13	KM836520
Mesostigmata	Ameroseiidae		SSWEE155-13	KM835864
Mesostigmata	Ameroseiidae		SSWEE170-13	KM827694
Mesostigmata	Ameroseiidae		SSWLE2881-13	KM833090
Mesostigmata	Arctacaridae	*Arctacarus*	CHACA097-08	JX837937

Mesostigmata	Arctacaridae	*Arctacarus*		CHACB126-10	HQ558413
Mesostigmata	Arctacaridae	*Arctacarus*		CHACB127-10	HQ558414
Mesostigmata	Arctacaridae	*Arctacarus*		CHACB475-10	JX838755
Mesostigmata	Arctacaridae	*Arctacarus*		CHACB476-10	HQ558614
Mesostigmata	Arctacaridae	*Arctacarus*		CHACB477-10	HQ558615
Mesostigmata	Arctacaridae	*Arctacarus*		CHACB478-10	HQ558616
Mesostigmata	Arctacaridae	*Arctacarus*		CHACC107-10	JX838254
Mesostigmata	Arctacaridae	*Arctacarus*		CHACC108-10	JX837740
Mesostigmata	Arctacaridae	*Arctacarus*		CHACC109-10	JX838470
Mesostigmata	Arctacaridae	*Arctacarus*		CHACC110-10	JX834946
Mesostigmata	Arctacaridae	*Arctacarus*		CHACC111-10	JX834620
Mesostigmata	Arctacaridae	*Arctacarus*		MYMCA1270-11	JX834375
Mesostigmata	Arctacaridae	*Arctacarus*		MYMCA1350-11	JX834850
Mesostigmata	Arctacaridae	*Arctacarus*		MYMCA1351-11	JX834583
Mesostigmata	Arctacaridae	*Arctacarus*		MYMCA1409-11	JX836197
Mesostigmata	Arctacaridae	*Arctacarus*		MYMCA1427-11	JX834303
Mesostigmata	Arctacaridae	*Arctacarus*		MYMCA1428-11	JX835778
Mesostigmata	Arctacaridae	*Arctacarus*		MYMCA1429-11	JX838153
Mesostigmata	Arctacaridae	*Arctacarus*		MYMCA1499-11	JX833706
Mesostigmata	Arctacaridae	*Arctacarus*		MYMCA1501-11	JX835155
Mesostigmata	Arctacaridae	*Arctacarus*		MYMCA563-11	JX837214
Mesostigmata	Arctacaridae	*Arctacarus*		MYMCA564-11	JX838167
Mesostigmata	Arctacaridae	*Arctacarus*		MYMCA565-11	JX837962
Mesostigmata	Arctacaridae	*Arctacarus*		MYMCA697-11	JX838116
Mesostigmata	Arctacaridae	*Arctacarus*		MYMCA722-11	JX833763
Mesostigmata	Arctacaridae	*Arctacarus*		MYMCA723-11	JX835257
Mesostigmata	Arctacaridae	*Arctacarus*		MYMCA724-11	JX836068
Mesostigmata	Arctacaridae	*Arctacarus*		MYMCA726-11	JX838077
Mesostigmata	Arctacaridae	*Arctacarus*		MYMCA750-11	JX835358
Mesostigmata	Arctacaridae	*Arctacarus*		MYMCA751-11	JX836381
Mesostigmata	Arctacaridae	*Arctacarus*		MYMCA752-11	JX836224
Mesostigmata	Arctacaridae	*Arctacarus*		MYMCA981-11	JX835769
Mesostigmata	Arctacaridae	*Arctacarus*		MYMCA993-11	JX834745
Mesostigmata	Arctacaridae	*Arctacarus*		MYMCA994-11	JX834888
Mesostigmata	Arctacaridae	*Arctacarus*		MYMCA999-11	JX834567
Mesostigmata	Arctacaridae			SSBAC3372-12	KM835537
Mesostigmata	Arctacaridae			SSJAA2021-13	KM827181
Mesostigmata	Arctacaridae			SSJAC1585-13	KM834919
Mesostigmata	Arctacaridae			SSJAC976-13	KM825875
Mesostigmata	Ascidae	*Antennoseius*		CHACA302-08	JX837595
Mesostigmata	Ascidae	*Antennoseius*		CHACA303-08	JX837802
Mesostigmata	Ascidae	*Antennoseius*		CHACA307-08	JX834150
Mesostigmata	Ascidae	*Antennoseius*		CHACA967-10	HM405833
Mesostigmata	Ascidae	*Antennoseius*		CHACB1041-10	JX835894
Mesostigmata	Ascidae	*Antennoseius*		CHACB412-10	HQ558572
Mesostigmata	Ascidae	*Antennoseius*		MYMCA447-11	JX834536
Mesostigmata	Ascidae	*Antennoseius*		MYMCA448-11	JX835280
Mesostigmata	Ascidae	*Antennoseius*		MYMCA449-11	JX833648
Mesostigmata	Ascidae	*Antennoseius*		MYMCA452-11	JX838669
Mesostigmata	Ascidae	*Antennoseius*		MYMCA456-11	JX837492
Mesostigmata	Ascidae	*Antennoseius*		MYMCB610-11	JX836809
Mesostigmata	Ascidae	*Antennoseius*		MYMCE232-12	JX835806
Mesostigmata	Ascidae	*Antennoseius*		MYMCE233-12	JX834998

Mesostigmata	Ascidae	*Antennoseius*	MYMCE277-12	JX834540
Mesostigmata	Ascidae	*Antennoseius*	MYMCF263-12	JX836931
Mesostigmata	Ascidae	*Antennoseius*	MYMCG220-12	JX833890
Mesostigmata	Ascidae	*Antennoseius*	MYMCG407-12	JX833925
Mesostigmata	Ascidae	*Arctoseius*	CHACC161-10	JX837921
Mesostigmata	Ascidae	*Arctoseius*	MIONB410-10	KP979120
Mesostigmata	Ascidae	*Arctoseius*	MYMCG540-12	JX835450
Mesostigmata	Ascidae	*Arctoseius*	SMTPB10324-13	KR069697
Mesostigmata	Ascidae	*Arctoseius*	SMTPB10325-13	KR069190
Mesostigmata	Ascidae	*Arctoseius*	SMTPB10327-13	KR069895
Mesostigmata	Ascidae	*Arctoseius*	SMTPB10811-13	KR069778
Mesostigmata	Ascidae	*Arctoseius*	SMTPB10812-13	KR069322
Mesostigmata	Ascidae	*Arctoseius*	SMTPB12307-13	KR070449
Mesostigmata	Ascidae	*Arctoseius*	SMTPB12308-13	KR069341
Mesostigmata	Ascidae	*Arctoseius*	SMTPB12361-13	KR069353
Mesostigmata	Ascidae	*Arctoseius*	SMTPB12368-13	KR070486
Mesostigmata	Ascidae	*Arctoseius*	SMTPB12529-13	KR070078
Mesostigmata	Ascidae	*Arctoseius*	SMTPB12840-13	KR070499
Mesostigmata	Ascidae	*Arctoseius*	SMTPB12842-13	KR069303
Mesostigmata	Ascidae	*Arctoseius*	SMTPB12843-13	KR069555
Mesostigmata	Ascidae	*Arctoseius*	SMTPB13436-13	KR070521
Mesostigmata	Ascidae	*Arctoseius*	SMTPB13437-13	KR070504
Mesostigmata	Ascidae	*Arctoseius*	SMTPB13674-13	KR070171
Mesostigmata	Ascidae	*Arctoseius*	SMTPB13677-13	KR069679
Mesostigmata	Ascidae	*Arctoseius*	SMTPB14433-13	KR069280
Mesostigmata	Ascidae	*Arctoseius*	SMTPB1472-13	KR070091
Mesostigmata	Ascidae	*Arctoseius*	SMTPB21362-13	KR070244
Mesostigmata	Ascidae	*Arctoseius*	SMTPB2351-13	KR070526
Mesostigmata	Ascidae	*Arctoseius*	SMTPB3079-13	KR069867
Mesostigmata	Ascidae	*Arctoseius*	SMTPB3080-13	KR070470
Mesostigmata	Ascidae	*Arctoseius*	SMTPB3082-13	KR069929
Mesostigmata	Ascidae	*Arctoseius*	SMTPB3083-13	KR070366
Mesostigmata	Ascidae	*Arctoseius*	SMTPB3084-13	KR070579
Mesostigmata	Ascidae	*Arctoseius*	SMTPB3086-13	KR070388
Mesostigmata	Ascidae	*Arctoseius*	SMTPB3090-13	KR070540
Mesostigmata	Ascidae	*Arctoseius*	SMTPB3091-13	KR069629
Mesostigmata	Ascidae	*Arctoseius*	SMTPB3365-13	KR070481
Mesostigmata	Ascidae	*Arctoseius*	SMTPB3366-13	KR070548
Mesostigmata	Ascidae	*Arctoseius*	SMTPB3367-13	KR069903
Mesostigmata	Ascidae	*Arctoseius*	SMTPB3369-13	KR069222
Mesostigmata	Ascidae	*Arctoseius*	SMTPB3370-13	KR070113
Mesostigmata	Ascidae	*Arctoseius*	SMTPB3378-13	KR070137
Mesostigmata	Ascidae	*Arctoseius*	SMTPB3379-13	KR069277
Mesostigmata	Ascidae	*Arctoseius*	SMTPB3383-13	KR070196
Mesostigmata	Ascidae	*Arctoseius*	SMTPB4007-13	KR070019
Mesostigmata	Ascidae	*Arctoseius*	SMTPB5822-13	KR069372
Mesostigmata	Ascidae	*Arctoseius*	SMTPB5967-13	KR070542
Mesostigmata	Ascidae	*Arctoseius*	SMTPB6225-13	KR070215
Mesostigmata	Ascidae	*Arctoseius*	SMTPB6237-13	KR070462
Mesostigmata	Ascidae	*Arctoseius*	SMTPB6240-13	KR069703
Mesostigmata	Ascidae	*Arctoseius*	SMTPB6242-13	KR069177
Mesostigmata	Ascidae	*Arctoseius*	SMTPB6253-13	KR070665
Mesostigmata	Ascidae	*Arctoseius*	SMTPB6256-13	KR070555

Mesostigmata	Ascidae	*Arctoseius*	SMTPB6262-13	KR070654
Mesostigmata	Ascidae	*Arctoseius*	SMTPB672-13	KR070522
Mesostigmata	Ascidae	*Arctoseius*	SMTPB7095-13	KR070596
Mesostigmata	Ascidae	*Arctoseius*	SMTPB7664-13	KR069983
Mesostigmata	Ascidae	*Arctoseius*	SMTPB882-13	KR069800
Mesostigmata	Ascidae	*Arctoseius*	SMTPB883-13	KR069305
Mesostigmata	Ascidae	*Arctoseius*	SMTPB884-13	KR070643
Mesostigmata	Ascidae	*Arctoseius*	SMTPB8984-13	KR069233
Mesostigmata	Ascidae	*Arctoseius*	SMTPB9303-13	KR070087
Mesostigmata	Ascidae	*Arctoseius*	SMTPB9977-13	KR069232
Mesostigmata	Ascidae	*Arctoseius*	SMTPB9980-13	KR070514
Mesostigmata	Ascidae	*Arctoseius*	SMTPB9982-13	KR070015
Mesostigmata	Ascidae	*Arctoseius*	SMTPB9984-13	KR070006
Mesostigmata	Ascidae	*Arctoseius*	SMTPB9997-13	KR069310
Mesostigmata	Ascidae		CHACA163-08	JX835847
Mesostigmata	Ascidae		CHACA230-08	JX834324
Mesostigmata	Ascidae		CHACA231-08	JX835116
Mesostigmata	Ascidae		CHACA233-08	JX834364
Mesostigmata	Ascidae		CHACA234-08	JX835532
Mesostigmata	Ascidae		CHACA438-09	JX835137
Mesostigmata	Ascidae		CHACA498-09	JX833842
Mesostigmata	Ascidae		CHACA520-09	JX834431
Mesostigmata	Ascidae		CHACA627-09	KR069599
Mesostigmata	Ascidae		CHACB069-10	HQ558377
Mesostigmata	Ascidae		CHACB070-10	HQ558378
Mesostigmata	Ascidae		CHACB071-10	HQ558379
Mesostigmata	Ascidae		CHACB1103-10	HM907201
Mesostigmata	Ascidae		CHACB357-10	HQ558537
Mesostigmata	Ascidae		CHACB480-10	JX835897
Mesostigmata	Ascidae		CHACB481-10	JX838730
Mesostigmata	Ascidae		CHACB533-10	HQ558650
Mesostigmata	Ascidae		CHACB786-10	HQ941484
Mesostigmata	Ascidae		CHACB787-10	HQ941485
Mesostigmata	Ascidae		CHACB837-10	JX837574
Mesostigmata	Ascidae		CHACB926-10	HM907354
Mesostigmata	Ascidae		CHACC114-10	KR070488
Mesostigmata	Ascidae		CHACC116-10	KR069728
Mesostigmata	Ascidae		CHACC117-10	KP979114
Mesostigmata	Ascidae		CHACC118-10	JX835256
Mesostigmata	Ascidae		CHACC119-10	JX836976
Mesostigmata	Ascidae		CHACC163-10	KR070550
Mesostigmata	Ascidae		CHACC164-10	KR069602
Mesostigmata	Ascidae		CHACC165-10	KR069686
Mesostigmata	Ascidae		CNBAA463-12	KM827471
Mesostigmata	Ascidae		CNEIA2612-12	KM834069
Mesostigmata	Ascidae		CNEIH065-13	KM839713
Mesostigmata	Ascidae		CNJAB1038-12	KM836920
Mesostigmata	Ascidae		CNJAB1044-12	KM833920
Mesostigmata	Ascidae		CNJAC1516-12	KM827992
Mesostigmata	Ascidae		CNJAF1923-12	KM838566
Mesostigmata	Ascidae		CNJAF1934-12	KM839247
Mesostigmata	Ascidae		CNJAF1935-12	KM830062
Mesostigmata	Ascidae		CNJAF1941-12	KM837449

Mesostigmata	Ascidae	CNJAF1943-12	KM835128
Mesostigmata	Ascidae	CNJAF1946-12	KM829902
Mesostigmata	Ascidae	CNJAF1949-12	KM833815
Mesostigmata	Ascidae	CNJAF1953-12	KM832018
Mesostigmata	Ascidae	CNJAF1954-12	KM838609
Mesostigmata	Ascidae	CNJAF1955-12	KM829140
Mesostigmata	Ascidae	CNJAF1960-12	KM830469
Mesostigmata	Ascidae	CNJAF1968-12	KM836937
Mesostigmata	Ascidae	CNJAF1976-12	KM831844
Mesostigmata	Ascidae	CNJAF1993-12	KM826937
Mesostigmata	Ascidae	CNJAF2000-12	KM824749
Mesostigmata	Ascidae	CNJAF2005-12	KM839849
Mesostigmata	Ascidae	CNJAF2012-12	KM825694
Mesostigmata	Ascidae	CNJAF2014-12	KM826618
Mesostigmata	Ascidae	CNJAF2019-12	KM830509
Mesostigmata	Ascidae	CNJAF2020-12	KM832410
Mesostigmata	Ascidae	CNJAG1809-12	KM829913
Mesostigmata	Ascidae	CNJAG1820-12	KM838859
Mesostigmata	Ascidae	CNJAG1840-12	KM837421
Mesostigmata	Ascidae	CNJAG1852-12	KM827552
Mesostigmata	Ascidae	CNJAH759-12	KM828136
Mesostigmata	Ascidae	CNJAH770-12	KM826531
Mesostigmata	Ascidae	CNJAH781-12	KM836338
Mesostigmata	Ascidae	CNJAH789-12	KM830949
Mesostigmata	Ascidae	CNJAI854-12	KM840842
Mesostigmata	Ascidae	CNKJB697-14	KR069767
Mesostigmata	Ascidae	CNKJB721-14	KR070412
Mesostigmata	Ascidae	CNKJB740-14	KR069970
Mesostigmata	Ascidae	CNKJB741-14	KR070130
Mesostigmata	Ascidae	CNKJJ153-14	KR069158
Mesostigmata	Ascidae	CNPAC507-13	KM832420
Mesostigmata	Ascidae	CNPAE417-13	KM837223
Mesostigmata	Ascidae	CNPAE426-13	KM827407
Mesostigmata	Ascidae	CNPAE427-13	KM833518
Mesostigmata	Ascidae	CNPAE428-13	KM839162
Mesostigmata	Ascidae	CNPAE429-13	KM838251
Mesostigmata	Ascidae	CNPAE433-13	KM834225
Mesostigmata	Ascidae	CNPAE435-13	KM837797
Mesostigmata	Ascidae	CNPAE436-13	KM825959
Mesostigmata	Ascidae	CNPAE437-13	KM829831
Mesostigmata	Ascidae	CNPAE438-13	KM828166
Mesostigmata	Ascidae	CNPAE439-13	KM837005
Mesostigmata	Ascidae	CNPAE441-13	KM829632
Mesostigmata	Ascidae	CNPAE442-13	KM840754
Mesostigmata	Ascidae	CNPAF623-13	KM825024
Mesostigmata	Ascidae	CNPAF866-13	KM837153
Mesostigmata	Ascidae	CNPAF875-13	KM827081
Mesostigmata	Ascidae	CNRMC1516-12	KM831744
Mesostigmata	Ascidae	CNRMD2671-12	KM829729
Mesostigmata	Ascidae	JSJUN2325-12	KP979197
Mesostigmata	Ascidae	JSOIE075-12	KP979240
Mesostigmata	Ascidae	JSOIE116-12	KR069704
Mesostigmata	Ascidae	MYMCA1025-11	JX834701

Mesostigmata	Ascidae	MYMCA1026-11	JX834040
Mesostigmata	Ascidae	MYMCA194-11	JX836930
Mesostigmata	Ascidae	MYMCA195-11	JX836878
Mesostigmata	Ascidae	MYMCA873-11	JX836365
Mesostigmata	Ascidae	MYMCB163-11	JX838065
Mesostigmata	Ascidae	MYMCB812-11	JX834172
Mesostigmata	Ascidae	MYMCB813-11	JX835659
Mesostigmata	Ascidae	MYMCB814-11	JX833770
Mesostigmata	Ascidae	MYMCB874-11	JX834096
Mesostigmata	Ascidae	MYMCB875-11	JX837637
Mesostigmata	Ascidae	MYMCB876-11	JX835588
Mesostigmata	Ascidae	MYMCC623-11	JX838142
Mesostigmata	Ascidae	MYMCC797-11	JX836285
Mesostigmata	Ascidae	MYMCE036-12	JX834286
Mesostigmata	Ascidae	MYMCE037-12	JX833749
Mesostigmata	Ascidae	MYMCE278-12	JX835480
Mesostigmata	Ascidae	MYMCE309-12	JX838328
Mesostigmata	Ascidae	MYMCE310-12	JX834138
Mesostigmata	Ascidae	MYMCE312-12	JX835209
Mesostigmata	Ascidae	MYMCE699-12	JX834216
Mesostigmata	Ascidae	MYMCE747-12	JX835250
Mesostigmata	Ascidae	MYMCE800-12	JX838517
Mesostigmata	Ascidae	MYMCF063-12	JX835165
Mesostigmata	Ascidae	MYMCF106-12	JX834712
Mesostigmata	Ascidae	MYMCF235-12	JX834750
Mesostigmata	Ascidae	MYMCF339-12	JX835063
Mesostigmata	Ascidae	MYMCF340-12	JX835131
Mesostigmata	Ascidae	MYMCF441-12	JX837289
Mesostigmata	Ascidae	MYMCF720-12	JX838455
Mesostigmata	Ascidae	MYMCF756-12	JX835717
Mesostigmata	Ascidae	MYMCF861-12	JX836769
Mesostigmata	Ascidae	MYMCF912-12	JX838211
Mesostigmata	Ascidae	MYMCG053-12	JX834777
Mesostigmata	Ascidae	MYMCG054-12	JX834651
Mesostigmata	Ascidae	MYMCG122-12	JX837239
Mesostigmata	Ascidae	MYMCG346-12	JX836628
Mesostigmata	Ascidae	MYMCG367-12	JX836530
Mesostigmata	Ascidae	MYMCG369-12	JX838676
Mesostigmata	Ascidae	MYMCG370-12	JX836695
Mesostigmata	Ascidae	MYMCG522-12	JX834553
Mesostigmata	Ascidae	MYMCG524-12	JX836820
Mesostigmata	Ascidae	MYMCG600-12	JX838568
Mesostigmata	Ascidae	PHOCT906-11	KR070598
Mesostigmata	Ascidae	SMTPB10637-13	KR069215
Mesostigmata	Ascidae	SMTPB12302-13	KR069433
Mesostigmata	Ascidae	SMTPB13442-13	KR069741
Mesostigmata	Ascidae	SMTPB13443-13	KR069508
Mesostigmata	Ascidae	SMTPB13898-13	KR070191
Mesostigmata	Ascidae	SMTPB14437-13	KR070642
Mesostigmata	Ascidae	SMTPB14685-13	KR070365
Mesostigmata	Ascidae	SMTPB14686-13	KR070396
Mesostigmata	Ascidae	SMTPB14723-13	KP979118
Mesostigmata	Ascidae	SMTPB1850-13	KR070648

Mesostigmata	Ascidae	SMTPB2347-13	KR069267
Mesostigmata	Ascidae	SMTPB2350-13	KR069900
Mesostigmata	Ascidae	SMTPB2352-13	KR069241
Mesostigmata	Ascidae	SMTPB5033-13	KR069630
Mesostigmata	Ascidae	SMTPB5035-13	KR069972
Mesostigmata	Ascidae	SMTPB5050-13	KR070724
Mesostigmata	Ascidae	SMTPB5051-13	KR070362
Mesostigmata	Ascidae	SMTPB5052-13	KR069264
Mesostigmata	Ascidae	SMTPB5053-13	KR070691
Mesostigmata	Ascidae	SMTPB5054-13	KP979247
Mesostigmata	Ascidae	SMTPB5055-13	KR070592
Mesostigmata	Ascidae	SMTPB5188-13	KP979121
Mesostigmata	Ascidae	SMTPB5413-13	KR070308
Mesostigmata	Ascidae	SMTPB5414-13	KR069254
Mesostigmata	Ascidae	SMTPB5834-13	KR070348
Mesostigmata	Ascidae	SMTPB6228-13	KR069723
Mesostigmata	Ascidae	SMTPB6234-13	KR069543
Mesostigmata	Ascidae	SMTPB6241-13	KR070076
Mesostigmata	Ascidae	SMTPB671-13	KP979129
Mesostigmata	Ascidae	SMTPB6797-13	KR070285
Mesostigmata	Ascidae	SMTPB7907-13	KP979183
Mesostigmata	Ascidae	SMTPB9297-13	KR070243
Mesostigmata	Ascidae	SMTPB9300-13	KR069288
Mesostigmata	Ascidae	SMTPB9985-13	KR069975
Mesostigmata	Ascidae	SMTPD206-13	KR069252
Mesostigmata	Ascidae	SSBAA2000-12	KM840336
Mesostigmata	Ascidae	SSBAA2002-12	KM829195
Mesostigmata	Ascidae	SSBAD3070-12	KM833994
Mesostigmata	Ascidae	SSBAD3971-12	KM839710
Mesostigmata	Ascidae	SSBAD3976-12	KM827604
Mesostigmata	Ascidae	SSBAD3983-12	KM840015
Mesostigmata	Ascidae	SSBAD3998-12	KM832124
Mesostigmata	Ascidae	SSBAD4010-12	KM836425
Mesostigmata	Ascidae	SSBAD4023-12	KM835665
Mesostigmata	Ascidae	SSBAD4037-12	KM828469
Mesostigmata	Ascidae	SSBAD4045-12	KM839052
Mesostigmata	Ascidae	SSBAF2274-13	KM827087
Mesostigmata	Ascidae	SSPAA2214-13	KM830273
Mesostigmata	Ascidae	SSPAA2232-13	KM828638
Mesostigmata	Ascidae	SSPAA2270-13	KM838026
Mesostigmata	Ascidae	SSPAA7996-13	KM837816
Mesostigmata	Ascidae	SSPAA8234-13	KM832051
Mesostigmata	Ascidae	SSPAA8280-13	KM830602
Mesostigmata	Ascidae	SSPAA8291-13	KM831301
Mesostigmata	Ascidae	SSPAA8295-13	KM832366
Mesostigmata	Ascidae	SSPAA8370-13	KM829883
Mesostigmata	Ascidae	SSPAA8371-13	KM828672
Mesostigmata	Ascidae	SSPAA8406-13	KM838159
Mesostigmata	Ascidae	SSPAA8425-13	KM828447
Mesostigmata	Ascidae	SSPAA8426-13	KM830901
Mesostigmata	Ascidae	SSPAA8454-13	KM827136
Mesostigmata	Ascidae	SSPAA8458-13	KM836800
Mesostigmata	Ascidae	SSPAA8459-13	KM832524

Mesostigmata	Ascidae		SSPAA9102-13	KM825220
Mesostigmata	Ascidae		SSPAA9197-13	KM832808
Mesostigmata	Ascidae		SSPAB3993-13	KM839824
Mesostigmata	Ascidae		SSPAC2147-13	KM832625
Mesostigmata	Ascidae		SSPAC2148-13	KM839040
Mesostigmata	Ascidae		SSPAC2153-13	KM838722
Mesostigmata	Ascidae		SSPAC2154-13	KM831943
Mesostigmata	Ascidae		SSPAC2367-13	KM835857
Mesostigmata	Ascidae		SSPAC2369-13	KM826989
Mesostigmata	Ascidae		SSPAC2379-13	KM826122
Mesostigmata	Ascidae		SSPAC2388-13	KM826085
Mesostigmata	Ascidae		SSPAC2389-13	KM825759
Mesostigmata	Ascidae		SSPAC2391-13	KM839037
Mesostigmata	Ascidae		SSPAC2392-13	KM831399
Mesostigmata	Ascidae		SSWEE030-13	KM839378
Mesostigmata	Blattisociidae	*Cheiroseius*	CHACA064-08	JX837689
Mesostigmata	Blattisociidae	*Cheiroseius*	CHACA066-08	JX838276
Mesostigmata	Blattisociidae	*Cheiroseius*	CHACA067-08	JX835706
Mesostigmata	Blattisociidae	*Cheiroseius*	CHACA080-08	JX837095
Mesostigmata	Blattisociidae	*Cheiroseius*	CHACA087-08	JX834416
Mesostigmata	Blattisociidae	*Cheiroseius*	CHACA213-08	JX837403
Mesostigmata	Blattisociidae	*Cheiroseius*	CHACA239-08	JX836306
Mesostigmata	Blattisociidae	*Cheiroseius*	CHACA485-09	JX836234
Mesostigmata	Blattisociidae	*Cheiroseius*	CHACA493-09	JX834382
Mesostigmata	Blattisociidae	*Cheiroseius*	CHACB364-10	HQ558544
Mesostigmata	Blattisociidae	*Cheiroseius*	CHACB365-10	HQ558545
Mesostigmata	Blattisociidae	*Cheiroseius*	CHACB366-10	HQ558546
Mesostigmata	Blattisociidae	*Cheiroseius*	CHACB367-10	HQ558547
Mesostigmata	Blattisociidae	*Cheiroseius*	CHACB755-10	JX838083
Mesostigmata	Blattisociidae	*Cheiroseius*	CHACB781-10	JX835730
Mesostigmata	Blattisociidae	*Cheiroseius*	CHACB785-10	JX838630
Mesostigmata	Blattisociidae	*Cheiroseius*	CNBAI477-13	KM832411
Mesostigmata	Blattisociidae	*Cheiroseius*	CNBAI504-13	KM828102
Mesostigmata	Blattisociidae	*Cheiroseius*	CNBPB536-12	KM837639
Mesostigmata	Blattisociidae	*Cheiroseius*	CNWBD027-13	KM832658
Mesostigmata	Blattisociidae	*Cheiroseius*	CNWLJ024-12	KM839235
Mesostigmata	Blattisociidae	*Cheiroseius*	JSOIE085-12	KP979194
Mesostigmata	Blattisociidae	*Cheiroseius*	JSOIE123-12	KP979200
Mesostigmata	Blattisociidae	*Cheiroseius*	MIAUS023-12	KR070539
Mesostigmata	Blattisociidae	*Cheiroseius*	MIAUS024-12	KP979324
Mesostigmata	Blattisociidae	*Cheiroseius*	MIAUS025-12	KR069839
Mesostigmata	Blattisociidae	*Cheiroseius*	MIONB156-10	KR069409
Mesostigmata	Blattisociidae	*Cheiroseius*	MIONB158-10	KP979144
Mesostigmata	Blattisociidae	*Cheiroseius*	MIONB225-10	KP979143
Mesostigmata	Blattisociidae	*Cheiroseius*	MYMCA324-11	JX833716
Mesostigmata	Blattisociidae	*Cheiroseius*	MYMCA325-11	JX837419
Mesostigmata	Blattisociidae	*Cheiroseius*	MYMCA326-11	JX836462
Mesostigmata	Blattisociidae	*Cheiroseius*	MYMCB428-11	JX834957
Mesostigmata	Blattisociidae	*Cheiroseius*	MYMCB429-11	JX834618
Mesostigmata	Blattisociidae	*Cheiroseius*	MYMCB949-11	JX834177
Mesostigmata	Blattisociidae	*Cheiroseius*	MYMCC793-11	JX838448
Mesostigmata	Blattisociidae	*Cheiroseius*	MYMCC794-11	JX835311
Mesostigmata	Blattisociidae	*Cheiroseius*	MYMCC795-11	JX836740

Mesostigmata	Blattisociidae	*Cheiroseius*		MYMCC796-11	JX835411
Mesostigmata	Blattisociidae	*Cheiroseius*		MYMCC815-11	JX833655
Mesostigmata	Blattisociidae	*Cheiroseius*		MYMCE205-12	JX836863
Mesostigmata	Blattisociidae	*Cheiroseius*		MYMCE206-12	JX837350
Mesostigmata	Blattisociidae	*Cheiroseius*		MYMCE207-12	JX834756
Mesostigmata	Blattisociidae	*Cheiroseius*		MYMCE262-12	JX837127
Mesostigmata	Blattisociidae	*Cheiroseius*		MYMCE264-12	JX837966
Mesostigmata	Blattisociidae	*Cheiroseius*		MYMCE265-12	JX835889
Mesostigmata	Blattisociidae	*Cheiroseius*		MYMCE268-12	JX836324
Mesostigmata	Blattisociidae	*Cheiroseius*		MYMCF323-12	JX835904
Mesostigmata	Blattisociidae	*Cheiroseius*		MYMCF422-12	JX834089
Mesostigmata	Blattisociidae	*Cheiroseius*		MYMCF514-12	JX834188
Mesostigmata	Blattisociidae	*Cheiroseius*		MYMCF884-12	JX834004
Mesostigmata	Blattisociidae	*Cheiroseius*		MYMCG061-12	JX837977
Mesostigmata	Blattisociidae	*Cheiroseius*		MYMCG062-12	JX837975
Mesostigmata	Blattisociidae	*Cheiroseius*		MYMCG115-12	JX834679
Mesostigmata	Blattisociidae	*Cheiroseius*		SMTPB10319-13	KR069717
Mesostigmata	Blattisociidae	*Cheiroseius*		SMTPB10320-13	KR070489
Mesostigmata	Blattisociidae	*Cheiroseius*		SMTPB10321-13	KR069571
Mesostigmata	Blattisociidae	*Cheiroseius*		SMTPB10322-13	KR069396
Mesostigmata	Blattisociidae	*Cheiroseius*		SMTPB10323-13	KR069399
Mesostigmata	Blattisociidae	*Cheiroseius*		SMTPB10331-13	KR069393
Mesostigmata	Blattisociidae	*Cheiroseius*		SMTPB10332-13	KR070145
Mesostigmata	Blattisociidae	*Cheiroseius*		SMTPB10642-13	KR070182
Mesostigmata	Blattisociidae	*Cheiroseius*		SMTPB11476-13	KR069681
Mesostigmata	Blattisociidae	*Cheiroseius*		SMTPB11743-13	KR069996
Mesostigmata	Blattisociidae	*Cheiroseius*		SMTPB15016-13	KR070726
Mesostigmata	Blattisociidae	*Cheiroseius*		SMTPB15017-13	KR069438
Mesostigmata	Blattisociidae	*Cheiroseius*		SMTPB15022-13	KR069296
Mesostigmata	Blattisociidae	*Cheiroseius*		SMTPB16291-13	KR069358
Mesostigmata	Blattisociidae	*Cheiroseius*		SMTPB16292-13	KR069475
Mesostigmata	Blattisociidae	*Cheiroseius*		SMTPB16293-13	KR069327
Mesostigmata	Blattisociidae	*Cheiroseius*		SMTPB3081-13	KR069727
Mesostigmata	Blattisociidae	*Cheiroseius*		SSJAE1613-13	KM827232
Mesostigmata	Blattisociidae	*Cheiroseius*		SSWLA2274-13	KM828169
Mesostigmata	Blattisociidae			CNEIG1583-13	KM824760
Mesostigmata	Blattisociidae			CNEIG1588-13	KM827237
Mesostigmata	Blattisociidae			CNEIG1589-13	KM831960
Mesostigmata	Blattisociidae			CNPAC540-13	KM835338
Mesostigmata	Blattisociidae			CNPAC546-13	KM828205
Mesostigmata	Blattisociidae			CNPAC547-13	KM828693
Mesostigmata	Blattisociidae			CNPPE2363-12	KJ084941
Mesostigmata	Blattisociidae			CNPPI1837-12	KJ445002
Mesostigmata	Blattisociidae			CNSLJ021-12	KM832217
Mesostigmata	Blattisociidae			CNSLJ022-12	KM837447
Mesostigmata	Blattisociidae			CNSLJ023-12	KM828282
Mesostigmata	Blattisociidae			CNSLJ414-12	KM832400
Mesostigmata	Blattisociidae			CNSLJ430-12	KM824328
Mesostigmata	Blattisociidae			CNWLG812-12	KM834519
Mesostigmata	Blattisociidae			CNWLH212-12	KM828779
Mesostigmata	Blattisociidae			CNWLI097-12	KM838642
Mesostigmata	Blattisociidae			JSOIE087-12	KR069659
Mesostigmata	Blattisociidae			JSOIE121-12	KR069149

Mesostigmata	Blattisociidae		MIONB009-10	KP979273
Mesostigmata	Blattisociidae		MYMCA1177-11	JX838726
Mesostigmata	Blattisociidae		MYMCA899-11	JX836054
Mesostigmata	Blattisociidae		MYMCA900-11	JX835643
Mesostigmata	Blattisociidae		MYMCC319-11	JX834801
Mesostigmata	Blattisociidae		MYMCE068-12	JX836250
Mesostigmata	Blattisociidae		MYMCE817-12	JX838509
Mesostigmata	Blattisociidae		MYMCE818-12	JX838310
Mesostigmata	Blattisociidae		MYMCE838-12	JX837944
Mesostigmata	Blattisociidae		MYMCE839-12	JX837174
Mesostigmata	Blattisociidae		MYMCE888-12	JX837128
Mesostigmata	Blattisociidae		MYMCF559-12	JX836860
Mesostigmata	Blattisociidae		MYMCF735-12	JX834988
Mesostigmata	Blattisociidae		MYMCF738-12	JX837588
Mesostigmata	Blattisociidae		MYMCF757-12	JX834423
Mesostigmata	Blattisociidae		MYMCF818-12	JX838305
Mesostigmata	Blattisociidae		MYMCF819-12	JX836366
Mesostigmata	Blattisociidae		MYMCG067-12	JX835187
Mesostigmata	Blattisociidae		MYMCG498-12	JX835543
Mesostigmata	Blattisociidae		MYMCG542-12	JX836210
Mesostigmata	Blattisociidae		MYMCG651-12	JX833775
Mesostigmata	Blattisociidae		SSBAC2380-12	KM836029
Mesostigmata	Dermanyssidae	*Dermanyssus longipes*	MIONB534-12	KP979215
Mesostigmata	Digamasellidae		CHACB1142-10	HM907231
Mesostigmata	Digamasellidae		CHACB1196-10	HM907271
Mesostigmata	Digamasellidae		CHACB1197-10	HM907272
Mesostigmata	Digamasellidae		CHACB359-10	HQ558539
Mesostigmata	Digamasellidae		CHACB360-10	HQ558540
Mesostigmata	Digamasellidae		CHACB361-10	HQ558541
Mesostigmata	Digamasellidae		CHACB990-10	JX836283
Mesostigmata	Digamasellidae		CNBPC214-12	KM831342
Mesostigmata	Digamasellidae		CNBPC218-12	KM827633
Mesostigmata	Digamasellidae		CNBPD580-12	KM839623
Mesostigmata	Digamasellidae		CNEIA2575-12	KM824117
Mesostigmata	Digamasellidae		CNEIA2578-12	KM830696
Mesostigmata	Digamasellidae		CNEIA2579-12	KM826137
Mesostigmata	Digamasellidae		CNFDM541-14	KR070249
Mesostigmata	Digamasellidae		CNFDM555-14	KR069962
Mesostigmata	Digamasellidae		CNGBD132-13	KR069730
Mesostigmata	Digamasellidae		CNGBE881-14	KR070700
Mesostigmata	Digamasellidae		CNGBK1835-14	KR070385
Mesostigmata	Digamasellidae		CNGLA015-13	KM827176
Mesostigmata	Digamasellidae		CNGLA016-13	KM832498
Mesostigmata	Digamasellidae		CNGLA017-13	KM834445
Mesostigmata	Digamasellidae		CNGLA018-13	KM830812
Mesostigmata	Digamasellidae		CNGLC073-13	KM826931
Mesostigmata	Digamasellidae		CNGLC074-13	KM836486
Mesostigmata	Digamasellidae		CNGLC075-13	KM827970
Mesostigmata	Digamasellidae		CNGLC076-13	KM836678
Mesostigmata	Digamasellidae		CNGLC077-13	KM835752
Mesostigmata	Digamasellidae		CNGLC078-13	KM834559
Mesostigmata	Digamasellidae		CNGLC079-13	KM835707
Mesostigmata	Digamasellidae		CNGLC080-13	KM826603

Mesostigmata	Digamasellidae	CNGLC084-13	KM830167
Mesostigmata	Digamasellidae	CNGLD018-13	KM834293
Mesostigmata	Digamasellidae	CNGLD025-13	KM824001
Mesostigmata	Digamasellidae	CNGLD026-13	KM837635
Mesostigmata	Digamasellidae	CNGLD032-13	KM835356
Mesostigmata	Digamasellidae	CNGLD035-13	KM825320
Mesostigmata	Digamasellidae	CNGLD036-13	KM836254
Mesostigmata	Digamasellidae	CNGLD037-13	KM838490
Mesostigmata	Digamasellidae	CNGLD039-13	KM837746
Mesostigmata	Digamasellidae	CNGLD040-13	KM838617
Mesostigmata	Digamasellidae	CNGLD043-13	KM833053
Mesostigmata	Digamasellidae	CNGLD044-13	KM823980
Mesostigmata	Digamasellidae	CNGLD049-13	KM836697
Mesostigmata	Digamasellidae	CNGLD053-13	KM839349
Mesostigmata	Digamasellidae	CNGLD054-13	KM839822
Mesostigmata	Digamasellidae	CNGLD055-13	KM836412
Mesostigmata	Digamasellidae	CNGLD056-13	KM834989
Mesostigmata	Digamasellidae	CNGLD058-13	KM827882
Mesostigmata	Digamasellidae	CNGLD060-13	KM828309
Mesostigmata	Digamasellidae	CNGLD068-13	KM825591
Mesostigmata	Digamasellidae	CNGLF134-13	KM825605
Mesostigmata	Digamasellidae	CNGLF2449-13	KM836229
Mesostigmata	Digamasellidae	CNGLF2452-13	KM833394
Mesostigmata	Digamasellidae	CNGLF2631-13	KM839272
Mesostigmata	Digamasellidae	CNGLF2640-13	KM837330
Mesostigmata	Digamasellidae	CNGLF2648-13	KM833018
Mesostigmata	Digamasellidae	CNGLF2653-13	KM826780
Mesostigmata	Digamasellidae	CNGLF2656-13	KM839454
Mesostigmata	Digamasellidae	CNGLF2657-13	KM837568
Mesostigmata	Digamasellidae	CNGLF2661-13	KM838367
Mesostigmata	Digamasellidae	CNGLF2664-13	KM830360
Mesostigmata	Digamasellidae	CNGLF2668-13	KM840412
Mesostigmata	Digamasellidae	CNGLF2675-13	KM827888
Mesostigmata	Digamasellidae	CNGLF2676-13	KM836686
Mesostigmata	Digamasellidae	CNGLF2682-13	KM825830
Mesostigmata	Digamasellidae	CNGLF2689-13	KM829016
Mesostigmata	Digamasellidae	CNGLF2691-13	KM840414
Mesostigmata	Digamasellidae	CNGLF2699-13	KM828407
Mesostigmata	Digamasellidae	CNGLF2704-13	KM839402
Mesostigmata	Digamasellidae	CNGLF2722-13	KM830854
Mesostigmata	Digamasellidae	CNGLF2730-13	KM836501
Mesostigmata	Digamasellidae	CNJAA875-12	KM826750
Mesostigmata	Digamasellidae	CNJAC1487-12	KM835913
Mesostigmata	Digamasellidae	CNJAC1488-12	KM840449
Mesostigmata	Digamasellidae	CNJAC1493-12	KM839484
Mesostigmata	Digamasellidae	CNJAC1507-12	KM827880
Mesostigmata	Digamasellidae	CNJAC1513-12	KM824108
Mesostigmata	Digamasellidae	CNJAC1543-12	KM835154
Mesostigmata	Digamasellidae	CNJAC1572-12	KM834272
Mesostigmata	Digamasellidae	CNJAC1600-12	KM826163
Mesostigmata	Digamasellidae	CNJAD2341-12	KM838509
Mesostigmata	Digamasellidae	CNJAG1813-12	KM837869
Mesostigmata	Digamasellidae	CNJAG1819-12	KM840478

Mesostigmata	Digamasellidae	CNJAH776-12	KM831191
Mesostigmata	Digamasellidae	CNJAH778-12	KM840608
Mesostigmata	Digamasellidae	CNJAH783-12	KM835813
Mesostigmata	Digamasellidae	CNJAH785-12	KM838073
Mesostigmata	Digamasellidae	CNJAH786-12	KM837566
Mesostigmata	Digamasellidae	CNJAH790-12	KM830467
Mesostigmata	Digamasellidae	CNKJA404-14	KR069714
Mesostigmata	Digamasellidae	CNKJA408-14	KR069721
Mesostigmata	Digamasellidae	CNKJA413-14	KR070668
Mesostigmata	Digamasellidae	CNKJA419-14	KR069193
Mesostigmata	Digamasellidae	CNKJA422-14	KR069818
Mesostigmata	Digamasellidae	CNKJA447-14	KR069676
Mesostigmata	Digamasellidae	CNKJA459-14	KR069429
Mesostigmata	Digamasellidae	CNKJA478-14	KR069750
Mesostigmata	Digamasellidae	CNKJA491-14	KR070166
Mesostigmata	Digamasellidae	CNKJA500-14	KR069391
Mesostigmata	Digamasellidae	CNKJA503-14	KR070580
Mesostigmata	Digamasellidae	CNKJD984-14	KR070002
Mesostigmata	Digamasellidae	CNKJD994-14	KR069389
Mesostigmata	Digamasellidae	CNKJD995-14	KR070525
Mesostigmata	Digamasellidae	CNKJL1286-14	KR070460
Mesostigmata	Digamasellidae	CNKJM2530-14	KR069589
Mesostigmata	Digamasellidae	CNKJM2571-14	KR070099
Mesostigmata	Digamasellidae	CNKJM2576-14	KR070372
Mesostigmata	Digamasellidae	CNLMN1949-14	KR070120
Mesostigmata	Digamasellidae	CNLMN1959-14	KR069247
Mesostigmata	Digamasellidae	CNLMO2397-14	KR070257
Mesostigmata	Digamasellidae	CNPAC312-13	KM837424
Mesostigmata	Digamasellidae	CNPAC313-13	KM829235
Mesostigmata	Digamasellidae	CNPAC314-13	KM839122
Mesostigmata	Digamasellidae	CNPAC315-13	KM832196
Mesostigmata	Digamasellidae	CNPAC361-13	KM831389
Mesostigmata	Digamasellidae	CNPAC362-13	KM831729
Mesostigmata	Digamasellidae	CNPAC363-13	KM835405
Mesostigmata	Digamasellidae	CNPAC364-13	KM831243
Mesostigmata	Digamasellidae	CNPAC365-13	KM840642
Mesostigmata	Digamasellidae	CNPAC478-13	KM826009
Mesostigmata	Digamasellidae	CNPAC493-13	KM832584
Mesostigmata	Digamasellidae	CNPAC494-13	KM836310
Mesostigmata	Digamasellidae	CNPAC495-13	KM832214
Mesostigmata	Digamasellidae	CNPAC496-13	KM839978
Mesostigmata	Digamasellidae	CNPAC497-13	KM839535
Mesostigmata	Digamasellidae	CNPAC498-13	KM839202
Mesostigmata	Digamasellidae	CNPAC499-13	KM829504
Mesostigmata	Digamasellidae	CNPAC501-13	KM826647
Mesostigmata	Digamasellidae	CNPAC508-13	KM838785
Mesostigmata	Digamasellidae	CNPAC511-13	KM834207
Mesostigmata	Digamasellidae	CNPAC516-13	KM830332
Mesostigmata	Digamasellidae	CNPAC517-13	KM828350
Mesostigmata	Digamasellidae	CNPAC521-13	KM831183
Mesostigmata	Digamasellidae	CNPAC529-13	KM832159
Mesostigmata	Digamasellidae	CNPAC530-13	KM831443
Mesostigmata	Digamasellidae	CNPAC531-13	KM833141

Mesostigmata	Digamasellidae	CNPAC533-13	KM838912
Mesostigmata	Digamasellidae	CNPAC534-13	KM833257
Mesostigmata	Digamasellidae	CNPAC535-13	KM828252
Mesostigmata	Digamasellidae	CNPAC536-13	KM833898
Mesostigmata	Digamasellidae	CNPAC541-13	KM838250
Mesostigmata	Digamasellidae	CNPAC542-13	KM832501
Mesostigmata	Digamasellidae	CNPAC544-13	KM832197
Mesostigmata	Digamasellidae	CNPAC553-13	KM831811
Mesostigmata	Digamasellidae	CNPAC554-13	KM824374
Mesostigmata	Digamasellidae	CNPAE440-13	KM837113
Mesostigmata	Digamasellidae	CNPAE456-13	KM835814
Mesostigmata	Digamasellidae	CNPAL453-13	KM829149
Mesostigmata	Digamasellidae	CNPKG1179-14	KR069997
Mesostigmata	Digamasellidae	CNPKG1184-14	KR070441
Mesostigmata	Digamasellidae	CNPKG1209-14	KR070404
Mesostigmata	Digamasellidae	CNPKG1222-14	KR069654
Mesostigmata	Digamasellidae	CNPKG1223-14	KR069223
Mesostigmata	Digamasellidae	CNPKG1224-14	KR070294
Mesostigmata	Digamasellidae	CNPKG1226-14	KR069211
Mesostigmata	Digamasellidae	CNPKG1228-14	KR069911
Mesostigmata	Digamasellidae	CNPKG1242-14	KR070467
Mesostigmata	Digamasellidae	CNPKG1252-14	KR069990
Mesostigmata	Digamasellidae	CNPKG1278-14	KR069374
Mesostigmata	Digamasellidae	CNPKG1281-14	KR070671
Mesostigmata	Digamasellidae	CNPKG1301-14	KR070295
Mesostigmata	Digamasellidae	CNPKG1302-14	KR069592
Mesostigmata	Digamasellidae	CNPKG1315-14	KR069926
Mesostigmata	Digamasellidae	CNPKG1316-14	KR069919
Mesostigmata	Digamasellidae	CNPKG1327-14	KR069918
Mesostigmata	Digamasellidae	CNPKG1328-14	KR069510
Mesostigmata	Digamasellidae	CNPKG1337-14	KR069268
Mesostigmata	Digamasellidae	CNPKG1350-14	KR069309
Mesostigmata	Digamasellidae	CNPKG1354-14	KR070738
Mesostigmata	Digamasellidae	CNPKG1362-14	KR069479
Mesostigmata	Digamasellidae	CNPKG1363-14	KR070302
Mesostigmata	Digamasellidae	CNPKG1364-14	KR069558
Mesostigmata	Digamasellidae	CNPKG1381-14	KR069505
Mesostigmata	Digamasellidae	CNPKG1383-14	KR069678
Mesostigmata	Digamasellidae	CNPKG1396-14	KR069698
Mesostigmata	Digamasellidae	CNPKG1399-14	KR070104
Mesostigmata	Digamasellidae	CNPKG1401-14	KR070290
Mesostigmata	Digamasellidae	CNPKG1404-14	KR070222
Mesostigmata	Digamasellidae	CNPKG1407-14	KR069783
Mesostigmata	Digamasellidae	CNPKG1409-14	KR069998
Mesostigmata	Digamasellidae	CNPPF1036-12	KJ167283
Mesostigmata	Digamasellidae	CNSLC560-12	KM825073
Mesostigmata	Digamasellidae	CNSLO1045-13	KM836379
Mesostigmata	Digamasellidae	CNWLB769-13	KM833328
Mesostigmata	Digamasellidae	CNWLB770-13	KM835662
Mesostigmata	Digamasellidae	CNWLB773-13	KM834098
Mesostigmata	Digamasellidae	CNWLB775-13	KM837102
Mesostigmata	Digamasellidae	CNWLB776-13	KM827567
Mesostigmata	Digamasellidae	MYMCA1215-11	JX837989

Mesostigmata	Digamasellidae	MYMCA1216-11	JX837488
Mesostigmata	Digamasellidae	MYMCA280-11	JX837505
Mesostigmata	Digamasellidae	MYMCA345-11	JX837870
Mesostigmata	Digamasellidae	MYMCA898-11	JX837841
Mesostigmata	Digamasellidae	MYMCB051-11	JX835085
Mesostigmata	Digamasellidae	MYMCB141-11	JX836287
Mesostigmata	Digamasellidae	MYMCB142-11	JX837695
Mesostigmata	Digamasellidae	MYMCB533-11	JX837317
Mesostigmata	Digamasellidae	MYMCC413-11	JX835572
Mesostigmata	Digamasellidae	MYMCF183-12	JX835337
Mesostigmata	Digamasellidae	MYMCF758-12	JX837808
Mesostigmata	Digamasellidae	MYMCF759-12	JX835149
Mesostigmata	Digamasellidae	MYMCG576-12	JX838650
Mesostigmata	Digamasellidae	MYMCG577-12	JX836770
Mesostigmata	Digamasellidae	NCCE071-11	KR069535
Mesostigmata	Digamasellidae	NCCE072-11	KP979228
Mesostigmata	Digamasellidae	NCCE073-11	KR070271
Mesostigmata	Digamasellidae	NCCE074-11	KR069457
Mesostigmata	Digamasellidae	NCCE075-11	KR070740
Mesostigmata	Digamasellidae	NCCE076-11	KR069549
Mesostigmata	Digamasellidae	NCCE077-11	KR070116
Mesostigmata	Digamasellidae	NCCE078-11	KR070190
Mesostigmata	Digamasellidae	NCCE079-11	KR070621
Mesostigmata	Digamasellidae	NCCE080-11	KR070206
Mesostigmata	Digamasellidae	NCCE081-11	KR070625
Mesostigmata	Digamasellidae	NCCE082-11	KR069500
Mesostigmata	Digamasellidae	NCCE083-11	KR070014
Mesostigmata	Digamasellidae	NCCE085-11	KR069260
Mesostigmata	Digamasellidae	NCCE086-11	KR070199
Mesostigmata	Digamasellidae	NCCE087-11	KR069461
Mesostigmata	Digamasellidae	NCCE088-11	KR069331
Mesostigmata	Digamasellidae	NCCE089-11	KR069388
Mesostigmata	Digamasellidae	NCCE090-11	KR070538
Mesostigmata	Digamasellidae	NCCE091-11	KR069959
Mesostigmata	Digamasellidae	NCCE092-11	KR069656
Mesostigmata	Digamasellidae	NCCE093-11	KR070342
Mesostigmata	Digamasellidae	NCCE094-11	KR069317
Mesostigmata	Digamasellidae	NCCE095-11	KR069735
Mesostigmata	Digamasellidae	NCCE096-11	KR069151
Mesostigmata	Digamasellidae	NCCE097-11	KR069300
Mesostigmata	Digamasellidae	NCCE098-11	KR070117
Mesostigmata	Digamasellidae	NCCE099-11	KR069872
Mesostigmata	Digamasellidae	NCCE100-11	KR070705
Mesostigmata	Digamasellidae	NCCE101-11	KR069541
Mesostigmata	Digamasellidae	NCCE102-11	KR070569
Mesostigmata	Digamasellidae	NCCE103-11	KR069521
Mesostigmata	Digamasellidae	NCCE104-11	KR070253
Mesostigmata	Digamasellidae	NCCE105-11	KR070281
Mesostigmata	Digamasellidae	NCCE106-11	KR069536
Mesostigmata	Digamasellidae	NCCE107-11	KR069444
Mesostigmata	Digamasellidae	NCCE108-11	KR070583
Mesostigmata	Digamasellidae	NCCE109-11	KR070585
Mesostigmata	Digamasellidae	NCCE110-11	KR069312

Mesostigmata	Digamasellidae	NCCE111-11	KR069936
Mesostigmata	Digamasellidae	NCCE112-11	KR069147
Mesostigmata	Digamasellidae	NCCE113-11	KR070702
Mesostigmata	Digamasellidae	NCCE114-11	KR069889
Mesostigmata	Digamasellidae	NCCE115-11	KR069718
Mesostigmata	Digamasellidae	NCCE116-11	KR069385
Mesostigmata	Digamasellidae	NCCE117-11	KR070213
Mesostigmata	Digamasellidae	NCCE118-11	KR069784
Mesostigmata	Digamasellidae	NCCE119-11	KR069978
Mesostigmata	Digamasellidae	NCCE120-11	KR069204
Mesostigmata	Digamasellidae	NCCE121-11	KR070112
Mesostigmata	Digamasellidae	NCCE122-11	KR070309
Mesostigmata	Digamasellidae	NCCE123-11	KR070174
Mesostigmata	Digamasellidae	NCCE124-11	KR070035
Mesostigmata	Digamasellidae	NCCE125-11	KR070321
Mesostigmata	Digamasellidae	NCCE126-11	KR070149
Mesostigmata	Digamasellidae	NCCE127-11	KR070557
Mesostigmata	Digamasellidae	NCCE128-11	KR070031
Mesostigmata	Digamasellidae	NCCE129-11	KR069257
Mesostigmata	Digamasellidae	NCCE130-11	KR069452
Mesostigmata	Digamasellidae	NCCE131-11	KR070582
Mesostigmata	Digamasellidae	NCCE132-11	KR070490
Mesostigmata	Digamasellidae	NCCE133-11	KR069684
Mesostigmata	Digamasellidae	NCCE134-11	KR070729
Mesostigmata	Digamasellidae	NCCE135-11	KR069923
Mesostigmata	Digamasellidae	NCCE136-11	KR070359
Mesostigmata	Digamasellidae	NCCE137-11	KR070184
Mesostigmata	Digamasellidae	NCCE138-11	KR069377
Mesostigmata	Digamasellidae	NCCE139-11	KR069379
Mesostigmata	Digamasellidae	NCCE140-11	KR069191
Mesostigmata	Digamasellidae	NCCE141-11	KR070610
Mesostigmata	Digamasellidae	NCCE142-11	KR069235
Mesostigmata	Digamasellidae	NCCE143-11	KR070003
Mesostigmata	Digamasellidae	NCCE144-11	KR070543
Mesostigmata	Digamasellidae	NCCE145-11	KR069960
Mesostigmata	Digamasellidae	NCCE146-11	KR070254
Mesostigmata	Digamasellidae	NCCE147-11	KR069702
Mesostigmata	Digamasellidae	NCCE148-11	KR069982
Mesostigmata	Digamasellidae	NCCE149-11	KR070497
Mesostigmata	Digamasellidae	NCCE150-11	KR069709
Mesostigmata	Digamasellidae	NCCE151-11	KR069804
Mesostigmata	Digamasellidae	NCCE152-11	KR069182
Mesostigmata	Digamasellidae	NCCE153-11	KR070731
Mesostigmata	Digamasellidae	NCCE154-11	KR070279
Mesostigmata	Digamasellidae	NCCE155-11	KR069263
Mesostigmata	Digamasellidae	NCCE156-11	KR070236
Mesostigmata	Digamasellidae	NCCE157-11	KR069297
Mesostigmata	Digamasellidae	NCCE158-11	KR070461
Mesostigmata	Digamasellidae	NCCE159-11	KR070325
Mesostigmata	Digamasellidae	NCCE160-11	KR069977
Mesostigmata	Digamasellidae	NCCE161-11	KR069407
Mesostigmata	Digamasellidae	NCCE162-11	KR069635
Mesostigmata	Digamasellidae	NCCE163-11	KR069801

Mesostigmata	Digamasellidae	NCCE164-11	KR069275
Mesostigmata	Digamasellidae	NCCE165-11	KR069724
Mesostigmata	Digamasellidae	NCCE166-11	KR070403
Mesostigmata	Digamasellidae	NCCE167-11	KR069472
Mesostigmata	Digamasellidae	NCCE168-11	KR070313
Mesostigmata	Digamasellidae	NCCE169-11	KR070074
Mesostigmata	Digamasellidae	NCCE170-11	KR069246
Mesostigmata	Digamasellidae	NCCE171-11	KR069375
Mesostigmata	Digamasellidae	NCCE172-11	KR069937
Mesostigmata	Digamasellidae	NCCE173-11	KR070038
Mesostigmata	Digamasellidae	NCCE174-11	KR070292
Mesostigmata	Digamasellidae	NCCE175-11	KR069441
Mesostigmata	Digamasellidae	NCCE176-11	KR070482
Mesostigmata	Digamasellidae	PHAPR1304-11	KP979218
Mesostigmata	Digamasellidae	PHAPR1305-11	KP979342
Mesostigmata	Digamasellidae	PHJUN3841-11	KP979125
Mesostigmata	Digamasellidae	PHJUN3846-11	KR070044
Mesostigmata	Digamasellidae	SMTPB10326-13	KR069258
Mesostigmata	Digamasellidae	SMTPB10328-13	KR069156
Mesostigmata	Digamasellidae	SMTPB10330-13	KR069976
Mesostigmata	Digamasellidae	SMTPB10816-13	KR070090
Mesostigmata	Digamasellidae	SMTPB11477-13	KR070682
Mesostigmata	Digamasellidae	SMTPB12303-13	KR069964
Mesostigmata	Digamasellidae	SMTPB12304-13	KR069293
Mesostigmata	Digamasellidae	SMTPB12312-13	KR070288
Mesostigmata	Digamasellidae	SMTPB12841-13	KR069815
Mesostigmata	Digamasellidae	SMTPB13434-13	KR070382
Mesostigmata	Digamasellidae	SMTPB13438-13	KR070221
Mesostigmata	Digamasellidae	SMTPB13441-13	KR069689
Mesostigmata	Digamasellidae	SMTPB13669-13	KR070075
Mesostigmata	Digamasellidae	SMTPB13675-13	KR070409
Mesostigmata	Digamasellidae	SMTPB14428-13	KR069882
Mesostigmata	Digamasellidae	SMTPB14432-13	KR069513
Mesostigmata	Digamasellidae	SMTPB14438-13	KR069749
Mesostigmata	Digamasellidae	SMTPB14653-13	KR069577
Mesostigmata	Digamasellidae	SMTPB14654-13	KR070511
Mesostigmata	Digamasellidae	SMTPB14679-13	KR069984
Mesostigmata	Digamasellidae	SMTPB14680-13	KR070224
Mesostigmata	Digamasellidae	SMTPB14681-13	KR069526
Mesostigmata	Digamasellidae	SMTPB14682-13	KP979233
Mesostigmata	Digamasellidae	SMTPB14683-13	KR069816
Mesostigmata	Digamasellidae	SMTPB14724-13	KP979308
Mesostigmata	Digamasellidae	SMTPB14844-13	KR069777
Mesostigmata	Digamasellidae	SMTPB14928-13	KR069478
Mesostigmata	Digamasellidae	SMTPB15015-13	KR069490
Mesostigmata	Digamasellidae	SMTPB15058-13	KR069613
Mesostigmata	Digamasellidae	SMTPB15229-13	KR069947
Mesostigmata	Digamasellidae	SMTPB17302-13	KR070375
Mesostigmata	Digamasellidae	SMTPB21360-13	KR070140
Mesostigmata	Digamasellidae	SMTPB21364-13	KR069207
Mesostigmata	Digamasellidae	SMTPB3085-13	KR069731
Mesostigmata	Digamasellidae	SMTPB4010-13	KR070296
Mesostigmata	Digamasellidae	SMTPB412-13	KR070276

Mesostigmata	Digamasellidae	SMTPB413-13	KR070417
Mesostigmata	Digamasellidae	SMTPB414-13	KR070168
Mesostigmata	Digamasellidae	SMTPB415-13	KR069561
Mesostigmata	Digamasellidae	SMTPB5032-13	KR069708
Mesostigmata	Digamasellidae	SMTPB5034-13	KR070001
Mesostigmata	Digamasellidae	SMTPB5665-13	KR069354
Mesostigmata	Digamasellidae	SMTPB5827-13	KR069623
Mesostigmata	Digamasellidae	SMTPB5828-13	KR070711
Mesostigmata	Digamasellidae	SMTPB5831-13	KR070008
Mesostigmata	Digamasellidae	SMTPB5832-13	KR070496
Mesostigmata	Digamasellidae	SMTPB5899-13	KR069671
Mesostigmata	Digamasellidae	SMTPB6221-13	KR069913
Mesostigmata	Digamasellidae	SMTPB6222-13	KR069576
Mesostigmata	Digamasellidae	SMTPB6224-13	KR070153
Mesostigmata	Digamasellidae	SMTPB6229-13	KR070165
Mesostigmata	Digamasellidae	SMTPB6243-13	KR069352
Mesostigmata	Digamasellidae	SMTPB6252-13	KR070666
Mesostigmata	Digamasellidae	SMTPB6267-13	KR069550
Mesostigmata	Digamasellidae	SMTPB6461-13	KR070335
Mesostigmata	Digamasellidae	SMTPB6483-13	KR069985
Mesostigmata	Digamasellidae	SMTPB6783-13	KR069468
Mesostigmata	Digamasellidae	SMTPB6784-13	KR070122
Mesostigmata	Digamasellidae	SMTPB6971-13	KR069298
Mesostigmata	Digamasellidae	SMTPB7096-13	KR069315
Mesostigmata	Digamasellidae	SMTPB8985-13	KR069981
Mesostigmata	Digamasellidae	SMTPB9596-13	KR070518
Mesostigmata	Digamasellidae	SMTPB9597-13	KR069600
Mesostigmata	Digamasellidae	SMTPB9598-13	KR070398
Mesostigmata	Digamasellidae	SMTPB9599-13	KR069946
Mesostigmata	Digamasellidae	SMTPB9600-13	KR069842
Mesostigmata	Digamasellidae	SMTPB9986-13	KR069387
Mesostigmata	Digamasellidae	SSBAD3081-12	KM826167
Mesostigmata	Digamasellidae	SSBAD3086-12	KM825070
Mesostigmata	Digamasellidae	SSBAD3088-12	KM832776
Mesostigmata	Digamasellidae	SSBAD3089-12	KM832357
Mesostigmata	Digamasellidae	SSBAD3094-12	KM827436
Mesostigmata	Digamasellidae	SSBAD3099-12	KM830947
Mesostigmata	Digamasellidae	SSBAD3100-12	KM829056
Mesostigmata	Digamasellidae	SSBAD3103-12	KM829281
Mesostigmata	Digamasellidae	SSBAD3104-12	KM833246
Mesostigmata	Digamasellidae	SSBAD3106-12	KM825064
Mesostigmata	Digamasellidae	SSBAD3110-12	KM834158
Mesostigmata	Digamasellidae	SSBAD3120-12	KM833869
Mesostigmata	Digamasellidae	SSBAD3121-12	KM824180
Mesostigmata	Digamasellidae	SSBAD3129-12	KM834667
Mesostigmata	Digamasellidae	SSEIB4234-13	KM828180
Mesostigmata	Digamasellidae	SSJAC178-13	KM836154
Mesostigmata	Digamasellidae	SSJAC185-13	KM827163
Mesostigmata	Digamasellidae	SSJAC198-13	KM829496
Mesostigmata	Digamasellidae	SSJAC199-13	KM837692
Mesostigmata	Digamasellidae	SSJAC200-13	KM834897
Mesostigmata	Digamasellidae	SSJAC201-13	KM832592
Mesostigmata	Digamasellidae	SSJAC202-13	KM836033

Mesostigmata	Digamasellidae	SSJAF3157-13	KM835907
Mesostigmata	Digamasellidae	SSPAA9725-13	KM837919
Mesostigmata	Digamasellidae	SSPAB3130-13	KM826271
Mesostigmata	Digamasellidae	SSPAC2155-13	KM829665
Mesostigmata	Digamasellidae	SSPAC2165-13	KM834581
Mesostigmata	Digamasellidae	SSPAC2171-13	KM840165
Mesostigmata	Digamasellidae	SSPAC2173-13	KM827298
Mesostigmata	Digamasellidae	SSPAC2190-13	KM836261
Mesostigmata	Digamasellidae	SSPAC2195-13	KM829885
Mesostigmata	Digamasellidae	SSPAC6726-13	KM825197
Mesostigmata	Digamasellidae	SSPAC6738-13	KM830011
Mesostigmata	Digamasellidae	SSPAC6748-13	KM829785
Mesostigmata	Digamasellidae	SSWLA028-13	KM834933
Mesostigmata	Digamasellidae	SSWLA2257-13	KM829637
Mesostigmata	Digamasellidae	SSWLA2266-13	KM826630
Mesostigmata	Digamasellidae	SSWLA2270-13	KM826973
Mesostigmata	Digamasellidae	SSWLA2287-13	KM830363
Mesostigmata	Digamasellidae	SSWLD4478-13	KM840617
Mesostigmata	Digamasellidae	SSWLD4479-13	KM826796
Mesostigmata	Digamasellidae	SSWLD4483-13	KM833494
Mesostigmata	Digamasellidae	SSWLD4494-13	KM824173
Mesostigmata	Dinychidae	CHACA043-08	JX835957
Mesostigmata	Dinychidae	CHACA044-08	JX834361
Mesostigmata	Dinychidae	CHACA047-08	JX835451
Mesostigmata	Dinychidae	CHACA243-08	JX836003
Mesostigmata	Dinychidae	CHACA244-08	JX838606
Mesostigmata	Dinychidae	CHACA245-08	JX834472
Mesostigmata	Dinychidae	CHACA247-08	JX834121
Mesostigmata	Dinychidae	CHACB527-10	JX834383
Mesostigmata	Dinychidae	CHACB740-10	HQ558780
Mesostigmata	Dinychidae	CHACB795-10	JX837699
Mesostigmata	Dinychidae	CHACB796-10	JX836194
Mesostigmata	Dinychidae	CHACB798-10	HQ941492
Mesostigmata	Dinychidae	CHACB799-10	HQ941493
Mesostigmata	Dinychidae	CHACB834-10	HQ941506
Mesostigmata	Dinychidae	CHACB835-10	HQ941507
Mesostigmata	Dinychidae	CHACB839-10	JX834104
Mesostigmata	Dinychidae	CHACB874-10	HM907319
Mesostigmata	Dinychidae	CHACC183-10	JX833663
Mesostigmata	Dinychidae	CNPAC513-13	KM825588
Mesostigmata	Dinychidae	CNPAC514-13	KM828530
Mesostigmata	Dinychidae	CNPAC515-13	KM827355
Mesostigmata	Dinychidae	CNPAC518-13	KM824885
Mesostigmata	Dinychidae	CNPAC520-13	KM828862
Mesostigmata	Dinychidae	CNPAC522-13	KM825042
Mesostigmata	Dinychidae	CNPAC524-13	KM831261
Mesostigmata	Dinychidae	CNPAC525-13	KM832869
Mesostigmata	Dinychidae	CNPAC527-13	KM827385
Mesostigmata	Dinychidae	CNPAC545-13	KM830122
Mesostigmata	Dinychidae	CNPAC556-13	KM837702
Mesostigmata	Dinychidae	CNPAL771-13	KM835568
Mesostigmata	Dinychidae	CNPAL906-13	KM827245
Mesostigmata	Dinychidae	MIONB413-10	KR070707

Mesostigmata	Dinychidae		MITMH022-07	KR069554
Mesostigmata	Dinychidae		MITMH055-07	KR069314
Mesostigmata	Dinychidae		MYMCE088-12	JX836169
Mesostigmata	Dinychidae		MYMCE089-12	JX836005
Mesostigmata	Dinychidae		MYMCE240-12	JX834845
Mesostigmata	Dinychidae		MYMCE584-12	JX834565
Mesostigmata	Dinychidae		MYMCE695-12	JX836573
Mesostigmata	Dinychidae		MYMCE816-12	JX837272
Mesostigmata	Dinychidae		MYMCE819-12	JX838543
Mesostigmata	Dinychidae		MYMCE841-12	JX837583
Mesostigmata	Dinychidae		MYMCE842-12	JX838400
Mesostigmata	Dinychidae		MYMCE883-12	JX834063
Mesostigmata	Dinychidae		MYMCE884-12	JX835086
Mesostigmata	Dinychidae		MYMCE886-12	JX834227
Mesostigmata	Dinychidae		MYMCE887-12	JX836261
Mesostigmata	Dinychidae		MYMCF191-12	JX834003
Mesostigmata	Dinychidae		MYMCF318-12	JX838773
Mesostigmata	Dinychidae		MYMCF320-12	JX838078
Mesostigmata	Dinychidae		MYMCF557-12	JX836667
Mesostigmata	Dinychidae		MYMCF558-12	JX838379
Mesostigmata	Dinychidae		MYMCF561-12	JX836567
Mesostigmata	Dinychidae		MYMCF589-12	JX838647
Mesostigmata	Dinychidae		MYMCG312-12	JX834246
Mesostigmata	Dinychidae		MYMCG314-12	JX836176
Mesostigmata	Dinychidae		MYMCG506-12	JX833743
Mesostigmata	Dinychidae		MYTMC091-09	GU680456
Mesostigmata	Dinychidae		MYTMC102-09	GU680489
Mesostigmata	Dinychidae		SSEIA7687-13	KM830015
Mesostigmata	Dinychidae		SSEIA7691-13	KM829074
Mesostigmata	Dinychidae		SSEIB8279-13	KM827230
Mesostigmata	Dinychidae		SSEIB8318-13	KM836120
Mesostigmata	Laelapidae	*Cosmolaelaps*	CHACA996-10	HM405855
Mesostigmata	Laelapidae	*Cosmolaelaps*	CHACA998-10	HM405856
Mesostigmata	Laelapidae	*Cosmolaelaps*	CHACB038-10	HQ558351
Mesostigmata	Laelapidae	*Cosmolaelaps*	CHACB039-10	HQ558352
Mesostigmata	Laelapidae	*Cosmolaelaps*	CHACB1097-10	HM907198
Mesostigmata	Laelapidae	*Cosmolaelaps*	CHACB1098-10	HM907199
Mesostigmata	Laelapidae	*Cosmolaelaps*	CHACB1105-10	HM907202
Mesostigmata	Laelapidae	*Cosmolaelaps*	CHACB956-10	HM907376
Mesostigmata	Laelapidae	*Cosmolaelaps*	CHACB957-10	HM907377
Mesostigmata	Laelapidae	*Cosmolaelaps*	CHACB958-10	HM907378
Mesostigmata	Laelapidae	*Cosmolaelaps*	CHACB959-10	HM907379
Mesostigmata	Laelapidae	*Cosmolaelaps*	MYMCC050-11	JX835561
Mesostigmata	Laelapidae	*Cosmolaelaps*	MYMCC051-11	JX836237
Mesostigmata	Laelapidae	*Cosmolaelaps*	MYMCC081-11	JX836240
Mesostigmata	Laelapidae	*Cosmolaelaps*	MYMCE155-12	JX837736
Mesostigmata	Laelapidae	*Cosmolaelaps*	MYMCE156-12	JX834547
Mesostigmata	Laelapidae	*Cosmolaelaps*	MYMCE157-12	JX835201
Mesostigmata	Laelapidae	*Cosmolaelaps*	MYMCE158-12	JX836940
Mesostigmata	Laelapidae	*Cosmolaelaps*	MYMCF699-12	JX836693
Mesostigmata	Laelapidae	*Haemogamasus ambulans*	MYMCC547-11	JX835676
Mesostigmata	Laelapidae	*Hypoaspis*	CHACA063-08	KR069459
Mesostigmata	Laelapidae	*Hypoaspis*	CHACA435-09	JX835922

Mesostigmata	Laelapidae	*Hypoaspis*	CHACA445-09	JX837104
Mesostigmata	Laelapidae	*Hypoaspis*	CHACA495-09	JX836272
Mesostigmata	Laelapidae	*Hypoaspis*	CHACA510-09	JX834045
Mesostigmata	Laelapidae	*Hypoaspis*	CHACA583-09	JX835348
Mesostigmata	Laelapidae	*Hypoaspis*	CHACA972-10	HM405837
Mesostigmata	Laelapidae	*Hypoaspis*	CHACA973-10	HM405838
Mesostigmata	Laelapidae	*Hypoaspis*	CHACB142-10	HQ558425
Mesostigmata	Laelapidae	*Hypoaspis*	CHACB143-10	HQ558426
Mesostigmata	Laelapidae	*Hypoaspis*	CHACB144-10	HQ558427
Mesostigmata	Laelapidae	*Hypoaspis*	CHACB145-10	HQ558428
Mesostigmata	Laelapidae	*Hypoaspis*	CHACB146-10	HQ558429
Mesostigmata	Laelapidae	*Hypoaspis*	CHACB164-10	HQ558446
Mesostigmata	Laelapidae	*Hypoaspis*	CHACB165-10	HQ558447
Mesostigmata	Laelapidae	*Hypoaspis*	CHACB409-10	JX838140
Mesostigmata	Laelapidae	*Hypoaspis*	CHACB429-10	HQ558585
Mesostigmata	Laelapidae	*Hypoaspis*	CHACB704-10	HQ558759
Mesostigmata	Laelapidae	*Hypoaspis*	CHACB756-10	JX833633
Mesostigmata	Laelapidae	*Hypoaspis*	CHACB757-10	JX838505
Mesostigmata	Laelapidae	*Hypoaspis*	CHACB758-10	JX834301
Mesostigmata	Laelapidae	*Hypoaspis*	CHACB759-10	JX837735
Mesostigmata	Laelapidae	*Hypoaspis*	CHACB780-10	HQ941483
Mesostigmata	Laelapidae	*Hypoaspis*	CHACB784-10	JX835307
Mesostigmata	Laelapidae	*Hypoaspis*	CHACC073-10	HM907467
Mesostigmata	Laelapidae	*Hypoaspis*	CHACC075-10	HM907469
Mesostigmata	Laelapidae	*Hypoaspis*	CHACC076-10	HM907470
Mesostigmata	Laelapidae	*Hypoaspis*	CHACC077-10	HM907471
Mesostigmata	Laelapidae	*Hypoaspis*	CHACC079-10	HM907472
Mesostigmata	Laelapidae	*Hypoaspis*	CHACC160-10	JX836374
Mesostigmata	Laelapidae	*Hypoaspis*	MIONB288-10	KR069988
Mesostigmata	Laelapidae	*Hypoaspis*	MIONB289-10	KP979169
Mesostigmata	Laelapidae	*Hypoaspis*	MYMCA323-11	JX837421
Mesostigmata	Laelapidae	*Hypoaspis*	MYMCA362-11	JX834359
Mesostigmata	Laelapidae	*Hypoaspis*	MYMCB427-11	JX835037
Mesostigmata	Laelapidae	*Hypoaspis*	MYMCC268-11	JX838702
Mesostigmata	Laelapidae	*Hypoaspis*	MYMCE033-12	JX837334
Mesostigmata	Laelapidae	*Hypoaspis*	MYMCE034-12	JX838625
Mesostigmata	Laelapidae	*Hypoaspis*	MYMCE159-12	JX836352
Mesostigmata	Laelapidae	*Hypoaspis*	MYMCE160-12	JX833980
Mesostigmata	Laelapidae	*Hypoaspis*	MYMCE797-12	JX838285
Mesostigmata	Laelapidae	*Hypoaspis*	MYMCE798-12	JX833943
Mesostigmata	Laelapidae	*Hypoaspis*	MYMCE948-12	JX838191
Mesostigmata	Laelapidae	*Hypoaspis*	MYMCF060-12	JX835276
Mesostigmata	Laelapidae	*Hypoaspis*	MYMCF126-12	JX835809
Mesostigmata	Laelapidae	*Hypoaspis*	MYMCF127-12	JX835750
Mesostigmata	Laelapidae	*Hypoaspis*	MYMCF129-12	JX837738
Mesostigmata	Laelapidae	*Hypoaspis*	MYMCF130-12	JX834392
Mesostigmata	Laelapidae	*Hypoaspis*	MYMCF193-12	JX835205
Mesostigmata	Laelapidae	*Hypoaspis*	MYMCF194-12	JX835573
Mesostigmata	Laelapidae	*Hypoaspis*	MYMCF195-12	JX835388
Mesostigmata	Laelapidae	*Hypoaspis*	MYMCF236-12	JX837610
Mesostigmata	Laelapidae	*Hypoaspis*	MYMCF261-12	JX834330
Mesostigmata	Laelapidae	*Hypoaspis*	MYMCF262-12	JX835735
Mesostigmata	Laelapidae	*Hypoaspis*	MYMCF293-12	JX834024

Mesostigmata	Laelapidae	*Hypoaspis*	MYMCF337-12	JX836042
Mesostigmata	Laelapidae	*Hypoaspis*	MYMCF338-12	JX836127
Mesostigmata	Laelapidae	*Hypoaspis*	MYMCF439-12	JX836504
Mesostigmata	Laelapidae	*Hypoaspis*	MYMCF440-12	JX836626
Mesostigmata	Laelapidae	*Hypoaspis*	MYMCF696-12	JX837434
Mesostigmata	Laelapidae	*Hypoaspis*	MYMCF719-12	JX838607
Mesostigmata	Laelapidae	*Hypoaspis*	MYMCF856-12	JX837435
Mesostigmata	Laelapidae	*Hypoaspis*	MYMCF857-12	JX834628
Mesostigmata	Laelapidae	*Hypoaspis*	MYMCF858-12	JX835299
Mesostigmata	Laelapidae	*Hypoaspis*	MYMCF879-12	JX836354
Mesostigmata	Laelapidae	*Hypoaspis*	MYMCF880-12	JX834826
Mesostigmata	Laelapidae	*Hypoaspis*	MYMCF881-12	JX835134
Mesostigmata	Laelapidae	*Hypoaspis*	MYMCF911-12	JX836941
Mesostigmata	Laelapidae	*Hypoaspis*	MYMCF931-12	JX836954
Mesostigmata	Laelapidae	*Hypoaspis*	MYMCF932-12	JX837248
Mesostigmata	Laelapidae	*Hypoaspis*	MYMCG001-12	JX836750
Mesostigmata	Laelapidae	*Hypoaspis*	MYMCG002-12	JX834524
Mesostigmata	Laelapidae	*Hypoaspis*	MYMCG059-12	JX836348
Mesostigmata	Laelapidae	*Hypoaspis*	MYMCG060-12	JX835064
Mesostigmata	Laelapidae	*Hypoaspis*	MYMCG135-12	JX836422
Mesostigmata	Laelapidae	*Hypoaspis*	MYMCG218-12	JX836789
Mesostigmata	Laelapidae	*Hypoaspis*	MYMCG219-12	JX835394
Mesostigmata	Laelapidae	*Hypoaspis*	MYMCG233-12	JX834671
Mesostigmata	Laelapidae	*Hypoaspis*	MYMCG347-12	JX837847
Mesostigmata	Laelapidae	*Hypoaspis*	MYMCG348-12	JX837355
Mesostigmata	Laelapidae	*Hypoaspis*	MYMCG455-12	JX837102
Mesostigmata	Laelapidae	*Hypoaspis*	MYMCG456-12	JX834708
Mesostigmata	Laelapidae	*Hypoaspis*	MYMCG457-12	JX836143
Mesostigmata	Laelapidae	*Hypoaspis*	MYMCG459-12	JX834722
Mesostigmata	Laelapidae	*Hypoaspis*	MYMCG597-12	JX837494
Mesostigmata	Laelapidae	*Hypoaspis*	MYMCG653-12	JX835151
Mesostigmata	Laelapidae	*Hypoaspis*	MYMCG654-12	JX835124
Mesostigmata	Laelapidae	*Hypoaspis*	SSEIB8185-13	KM829518
Mesostigmata	Laelapidae	*Hypoaspis aculeifer*	GACAC136-12	FM210170
Mesostigmata	Laelapidae	*Ololaelaps*	MYMCA1057-11	JX837161
Mesostigmata	Laelapidae	*Ololaelaps*	MYMCB078-11	JX837120
Mesostigmata	Laelapidae	*Ololaelaps*	MYMCC103-11	JX838224
Mesostigmata	Laelapidae	*Ololaelaps*	MYMCC178-11	JX836104
Mesostigmata	Laelapidae	*Ololaelaps*	MYMCC179-11	JX838511
Mesostigmata	Laelapidae	*Ololaelaps*	MYMCC180-11	JX834945
Mesostigmata	Laelapidae	*Ololaelaps*	MYMCC431-11	JX838634
Mesostigmata	Laelapidae	*Ololaelaps*	MYMCE150-12	JX834980
Mesostigmata	Laelapidae	*Ololaelaps*	MYMCE421-12	JX838591
Mesostigmata	Laelapidae	*Ololaelaps*	MYMCE476-12	JX836333
Mesostigmata	Laelapidae	*Ololaelaps*	MYMCE552-12	JX837773
Mesostigmata	Laelapidae	*Ololaelaps*	MYMCE570-12	JX836779
Mesostigmata	Laelapidae	*Ololaelaps*	MYMCF259-12	JX833971
Mesostigmata	Laelapidae	*Ololaelaps*	MYMCF260-12	JX833993
Mesostigmata	Laelapidae	*Ololaelaps*	MYMCF319-12	JX836612
Mesostigmata	Laelapidae	*Ololaelaps*	MYMCF349-12	JX835694
Mesostigmata	Laelapidae	*Ololaelaps*	MYMCF722-12	JX837911
Mesostigmata	Laelapidae	*Ololaelaps*	MYMCF734-12	JX834372
Mesostigmata	Laelapidae	*Ololaelaps*	MYMCF836-12	JX837347

Mesostigmata	Laelapidae	*Ololaelaps*	MYMCF903-12	JX835434
Mesostigmata	Laelapidae	*Ololaelaps*	MYMCG058-12	JX837470
Mesostigmata	Laelapidae	*Ololaelaps*	MYMCG118-12	JX833942
Mesostigmata	Laelapidae	*Ololaelaps*	MYMCG119-12	JX837005
Mesostigmata	Laelapidae	*Ololaelaps*	MYMCG134-12	JX837196
Mesostigmata	Laelapidae	*Ololaelaps*	MYMCG207-12	JX834408
Mesostigmata	Laelapidae	*Ololaelaps*	MYMCG453-12	JX836985
Mesostigmata	Laelapidae	*Ololaelaps*	MYMCG454-12	JX837319
Mesostigmata	Laelapidae	*Ololaelaps*	MYMCG507-12	JX837121
Mesostigmata	Laelapidae	*Ololaelaps*	MYMCG508-12	JX838037
Mesostigmata	Laelapidae	*Ololaelaps*	MYMCG509-12	JX835505
Mesostigmata	Laelapidae	*Ololaelaps*	SSPAA7962-13	KM834536
Mesostigmata	Laelapidae		CHACA960-10	HM405829
Mesostigmata	Laelapidae		CHACA971-10	HM405836
Mesostigmata	Laelapidae		CHACB955-10	HM907375
Mesostigmata	Laelapidae		CNFDA418-14	KR070714
Mesostigmata	Laelapidae		CNGLD016-13	KM837895
Mesostigmata	Laelapidae		CNGLD052-13	KM827193
Mesostigmata	Laelapidae		CNGMD1784-14	KR069477
Mesostigmata	Laelapidae		CNJAF1919-12	KM833265
Mesostigmata	Laelapidae		CNJAF1930-12	KM824057
Mesostigmata	Laelapidae		CNJAF1995-12	KM838677
Mesostigmata	Laelapidae		CNLMD1870-14	KR070148
Mesostigmata	Laelapidae		CNPAO101-13	KM825306
Mesostigmata	Laelapidae		CNPAO116-13	KM830606
Mesostigmata	Laelapidae		CNRME4767-12	KM833768
Mesostigmata	Laelapidae		CNRME4772-12	KM829158
Mesostigmata	Laelapidae		CNWLE2589-13	KM838753
Mesostigmata	Laelapidae		CNWLG852-12	KM828327
Mesostigmata	Laelapidae		MYMCC269-11	JX837125
Mesostigmata	Laelapidae		MYMCC270-11	JX838480
Mesostigmata	Laelapidae		MYMCE175-12	JX835078
Mesostigmata	Laelapidae		MYMCE451-12	JX838735
Mesostigmata	Laelapidae		MYMCE878-12	JX838442
Mesostigmata	Laelapidae		MYMCF043-12	JX837931
Mesostigmata	Laelapidae		MYMCF279-12	JX834055
Mesostigmata	Laelapidae		MYMCF280-12	JX838039
Mesostigmata	Laelapidae		MYMCF288-12	JX834262
Mesostigmata	Laelapidae		MYMCF292-12	JX834438
Mesostigmata	Laelapidae		MYMCF294-12	JX835261
Mesostigmata	Laelapidae		MYMCF698-12	JX836363
Mesostigmata	Laelapidae		MYMCF827-12	JX837757
Mesostigmata	Laelapidae		MYMCG221-12	JX837752
Mesostigmata	Laelapidae		MYMCG428-12	JX834401
Mesostigmata	Laelapidae		MYMCG429-12	JX837739
Mesostigmata	Laelapidae		MYMCG433-12	JX836419
Mesostigmata	Laelapidae		MYMCG434-12	JX837260
Mesostigmata	Laelapidae		SMTPB20630-13	KR069242
Mesostigmata	Laelapidae		SMTPB20656-13	KR069167
Mesostigmata	Laelapidae		SMTPB5830-13	KP979317
Mesostigmata	Laelapidae		SMTPB5833-13	KR070680
Mesostigmata	Laelapidae		SSEIB7673-13	KM832468
Mesostigmata	Laelapidae		SSPAA6684-13	KM837690

Mesostigmata	Laelapidae		SSPAA7810-13	KM831175
Mesostigmata	Laelapidae		SSPAA7819-13	KM826237
Mesostigmata	Laelapidae		SSWLA2262-13	KM833110
Mesostigmata	Macrochelidae	*Macrocheles*	CNPPA4153-12	KJ086359
Mesostigmata	Macrochelidae	*Macrocheles*	JSOIE070-12	KP979269
Mesostigmata	Macrochelidae	*Macrocheles*	MIONB103-10	KR070615
Mesostigmata	Macrochelidae	*Macrocheles*	MIONB107-10	KP979340
Mesostigmata	Macrochelidae	*Macrocheles*	MIONB108-10	KP979203
Mesostigmata	Macrochelidae	*Macrocheles*	MIONB117-10	KR069766
Mesostigmata	Macrochelidae	*Macrocheles*	MIONB119-10	KR070420
Mesostigmata	Macrochelidae	*Macrocheles*	MIONB120-10	KR070517
Mesostigmata	Macrochelidae	*Macrocheles*	MIONB121-10	KR069713
Mesostigmata	Macrochelidae	*Macrocheles*	MIONB122-10	KP979285
Mesostigmata	Macrochelidae	*Macrocheles*	MIONB123-10	KP979187
Mesostigmata	Macrochelidae	*Macrocheles*	MIONB336-10	KP979156
Mesostigmata	Macrochelidae	*Macrocheles*	MIONB375-10	KR069248
Mesostigmata	Macrochelidae	*Macrocheles*	MIONB408-10	KR070114
Mesostigmata	Macrochelidae	*Macrocheles*	MIONB409-10	KR069416
Mesostigmata	Macrochelidae	*Macrocheles*	MIONB419-10	KP979180
Mesostigmata	Macrochelidae	*Macrocheles*	MIONB424-10	KR069196
Mesostigmata	Macrochelidae	*Macrocheles*	MIONB441-10	KR070246
Mesostigmata	Macrochelidae	*Macrocheles*	MIONB478-11	KM828634
Mesostigmata	Macrochelidae	*Macrocheles*	MIONB479-11	KM831279
Mesostigmata	Macrochelidae	*Macrocheles*	SMTPB17141-13	KR069726
Mesostigmata	Macrochelidae	*Macrocheles*	SMTPB17142-13	KR069894
Mesostigmata	Macrochelidae	*Macrocheles*	SMTPB6235-13	KR070367
Mesostigmata	Macrochelidae	*Macrocheles*	SMTPB6459-13	KR069797
Mesostigmata	Macrochelidae	*Macrocheles*	SMTPB6460-13	KR070017
Mesostigmata	Macrochelidae	*Macrocheles*	SMTPB8976-13	KR069319
Mesostigmata	Macrochelidae	*Macrocheles*	SMTPB8977-13	KR070360
Mesostigmata	Macrochelidae	*Macrocheles*	SMTPB8978-13	KR070293
Mesostigmata	Macrochelidae	*Macrocheles*	SMTPB8980-13	KR070077
Mesostigmata	Macrochelidae	*Macrocheles*	SMTPB8981-13	KR070330
Mesostigmata	Macrochelidae	*Macrocheles*	SMTPB8982-13	KR070678
Mesostigmata	Macrochelidae	*Macrocheles*	SMTPB8983-13	KR069575
Mesostigmata	Macrochelidae	*Macrocheles*	SMTPB8986-13	KR069861
Mesostigmata	Macrochelidae	*Macrocheles*	SMTPB8987-13	KR070062
Mesostigmata	Macrochelidae	*Macrocheles*	SMTPB8988-13	KR070507
Mesostigmata	Macrochelidae	*Macrocheles*	SMTPB8989-13	KR070374
Mesostigmata	Macrochelidae	*Macrocheles*	SMTPB8990-13	KR069662
Mesostigmata	Macrochelidae	*Macrocheles*	SMTPB9466-13	KR069402
Mesostigmata	Macrochelidae	*Macrocheles*	SMTPB9467-13	KR070024
Mesostigmata	Macrochelidae	*Macrocheles*	SSEIA3049-13	KM828709
Mesostigmata	Macrochelidae		ASAMT013-12	KP979316
Mesostigmata	Macrochelidae		CHACB779-10	HQ941482
Mesostigmata	Macrochelidae		MYMCE204-12	JX834758
Mesostigmata	Macrochelidae		MYMCE266-12	JX838319
Mesostigmata	Macrochelidae		MYMCE267-12	JX837325
Mesostigmata	Macrochelidae		MYMCE269-12	JX836303
Mesostigmata	Macrochelidae		SSEIB4203-13	KM839445
Mesostigmata	Macrochelidae		SSEIB4206-13	KM831957
Mesostigmata	Macrochelidae		SSEIB4218-13	KM831907
Mesostigmata	Macrochelidae		SSEIB4228-13	KM834316

Mesostigmata	Macrochelidae		SSEIB4242-13	KM824053
Mesostigmata	Macrochelidae		SSEIB5795-13	KM836965
Mesostigmata	Macrochelidae		SSWLA1882-13	KM839835
Mesostigmata	Macrochelidae		SSWLA4813-13	KM837265
Mesostigmata	Melicharidae	*Proctolaelaps*	CHACB072-10	JX833916
Mesostigmata	Melicharidae	*Proctolaelaps*	CNEIG1584-13	KM827861
Mesostigmata	Melicharidae	*Proctolaelaps*	CNEIG1585-13	KM824226
Mesostigmata	Melicharidae	*Proctolaelaps*	CNEIH068-13	KM835401
Mesostigmata	Melicharidae	*Proctolaelaps*	CNJAF1991-12	KM828629
Mesostigmata	Melicharidae	*Proctolaelaps*	CNPAD550-13	KM826495
Mesostigmata	Melicharidae	*Proctolaelaps*	CNPAD842-13	KM829480
Mesostigmata	Melicharidae	*Proctolaelaps*	CNPAE460-13	KM835542
Mesostigmata	Melicharidae	*Proctolaelaps*	CNPAI466-13	KM829970
Mesostigmata	Melicharidae	*Proctolaelaps*	CNPCE166-13	KM830734
Mesostigmata	Melicharidae	*Proctolaelaps*	CNPPA4072-12	KJ089837
Mesostigmata	Melicharidae	*Proctolaelaps*	CNPPA4073-12	KJ163201
Mesostigmata	Melicharidae	*Proctolaelaps*	CNPPA4076-12	KJ090099
Mesostigmata	Melicharidae	*Proctolaelaps*	CNPPA4082-12	KJ092971
Mesostigmata	Melicharidae	*Proctolaelaps*	CNPPA4083-12	KJ087032
Mesostigmata	Melicharidae	*Proctolaelaps*	CNPPA4085-12	KJ084915
Mesostigmata	Melicharidae	*Proctolaelaps*	CNPPA4086-12	KJ086408
Mesostigmata	Melicharidae	*Proctolaelaps*	CNPPA4087-12	KJ083789
Mesostigmata	Melicharidae	*Proctolaelaps*	CNPPA4088-12	KJ087302
Mesostigmata	Melicharidae	*Proctolaelaps*	CNPPA4089-12	KJ089547
Mesostigmata	Melicharidae	*Proctolaelaps*	CNPPA4091-12	KJ084786
Mesostigmata	Melicharidae	*Proctolaelaps*	CNPPA4092-12	KJ089275
Mesostigmata	Melicharidae	*Proctolaelaps*	CNPPA4093-12	KJ088178
Mesostigmata	Melicharidae	*Proctolaelaps*	CNPPA4096-12	KJ090006
Mesostigmata	Melicharidae	*Proctolaelaps*	CNPPA4097-12	KJ091187
Mesostigmata	Melicharidae	*Proctolaelaps*	CNPPA4099-12	KJ163884
Mesostigmata	Melicharidae	*Proctolaelaps*	CNPPA4100-12	KJ086023
Mesostigmata	Melicharidae	*Proctolaelaps*	CNPPA4104-12	KJ090215
Mesostigmata	Melicharidae	*Proctolaelaps*	CNPPA4105-12	KJ089261
Mesostigmata	Melicharidae	*Proctolaelaps*	CNPPA4106-12	KJ084087
Mesostigmata	Melicharidae	*Proctolaelaps*	CNPPA4107-12	KJ084005
Mesostigmata	Melicharidae	*Proctolaelaps*	CNPPA4108-12	KJ091856
Mesostigmata	Melicharidae	*Proctolaelaps*	CNPPA4111-12	KJ089817
Mesostigmata	Melicharidae	*Proctolaelaps*	CNPPA4113-12	KJ086334
Mesostigmata	Melicharidae	*Proctolaelaps*	CNPPA4115-12	KJ084405
Mesostigmata	Melicharidae	*Proctolaelaps*	CNPPA4116-12	KJ090579
Mesostigmata	Melicharidae	*Proctolaelaps*	CNPPA4117-12	KJ089594
Mesostigmata	Melicharidae	*Proctolaelaps*	CNPPA4127-12	KJ084105
Mesostigmata	Melicharidae	*Proctolaelaps*	CNPPA4129-12	KJ165242
Mesostigmata	Melicharidae	*Proctolaelaps*	CNPPA4135-12	KJ088172
Mesostigmata	Melicharidae	*Proctolaelaps*	CNPPA4136-12	KJ164646
Mesostigmata	Melicharidae	*Proctolaelaps*	CNPPA4137-12	KJ163227
Mesostigmata	Melicharidae	*Proctolaelaps*	CNPPA4139-12	KJ088291
Mesostigmata	Melicharidae	*Proctolaelaps*	CNPPE1431-12	KJ085139
Mesostigmata	Melicharidae	*Proctolaelaps*	CNRMC1658-12	KM839466
Mesostigmata	Melicharidae	*Proctolaelaps*	CNRME4793-12	KM837505
Mesostigmata	Melicharidae	*Proctolaelaps*	CNSLA297-12	KM837414
Mesostigmata	Melicharidae	*Proctolaelaps*	CNSLF648-12	KM838289
Mesostigmata	Melicharidae	*Proctolaelaps*	CNSLF655-12	KM834466

Mesostigmata	Melicharidae	*Proctolaelaps*		CNSLF660-12	KM831109
Mesostigmata	Melicharidae	*Proctolaelaps*		CNSLF662-12	KM834804
Mesostigmata	Melicharidae	*Proctolaelaps*		CNSLG123-12	KM830814
Mesostigmata	Melicharidae	*Proctolaelaps*		MYMCA896-11	JX838674
Mesostigmata	Melicharidae	*Proctolaelaps*		MYMCA897-11	JX836803
Mesostigmata	Melicharidae	*Proctolaelaps*		MYMCB526-11	JX835045
Mesostigmata	Melicharidae	*Proctolaelaps*		MYMCC893-11	JX834791
Mesostigmata	Melicharidae	*Proctolaelaps*		MYMCE311-12	JX835529
Mesostigmata	Melicharidae	*Proctolaelaps*		MYMCF676-12	JX837268
Mesostigmata	Melicharidae	*Proctolaelaps*		MYMCG368-12	JX835803
Mesostigmata	Melicharidae	*Proctolaelaps*		PHJUL985-11	KR070505
Mesostigmata	Melicharidae	*Proctolaelaps*		PHJUL987-11	KR069282
Mesostigmata	Melicharidae	*Proctolaelaps*		PHJUL991-11	KR069729
Mesostigmata	Melicharidae	*Proctolaelaps*		SSBAD4744-13	KM826037
Mesostigmata	Melicharidae	*Proctolaelaps*		SSBAD5543-13	KM839767
Mesostigmata	Melicharidae	*Proctolaelaps*		SSBAD5569-13	KM831788
Mesostigmata	Melicharidae	*Proctolaelaps*		SSBAE3916-13	KM834744
Mesostigmata	Melicharidae	*Proctolaelaps*		SSJAE1663-13	KM828190
Mesostigmata	Melicharidae	*Proctolaelaps*		SSJAE3151-13	KM824732
Mesostigmata	Melicharidae	*Proctolaelaps*		SSJAF8175-13	KM825028
Mesostigmata	Melicharidae	*Proctolaelaps*		SSJAF8341-13	KM826447
Mesostigmata	Melicharidae	*Proctolaelaps*		SSPAB7978-13	KM825580
Mesostigmata	Melicharidae	*Proctolaelaps*		SSPAC11043-13	KM832309
Mesostigmata	Melicharidae	*Proctolaelaps*		SSPAC7842-13	KM830032
Mesostigmata	Melicharidae	*Proctolaelaps*		SSWLD4499-13	KM837847
Mesostigmata	Melicharidae	*Proctolaelaps*		SSWLF3583-13	KM838195
Mesostigmata	Melicharidae	*Proctolaelaps*		SSWLF3584-13	KM833805
Mesostigmata	Melicharidae	*Proctolaelaps*		SSWLF3597-13	KM829461
Mesostigmata	Melicharidae	*Proctolaelaps*		SSWLF3599-13	KM836036
Mesostigmata	Melicharidae	*Proctolaelaps*		SSWLF3653-13	KM838552
Mesostigmata	Melicharidae	*Proctolaelaps*		SSWLF3656-13	KM831449
Mesostigmata	Melicharidae	*Proctolaelaps*		SSWLF3657-13	KM833634
Mesostigmata	Microgyniidae			CHACB067-10	HQ558375
Mesostigmata	Microgyniidae			CNGLE039-13	KM830249
Mesostigmata	Microgyniidae			CNGLF128-13	KM827917
Mesostigmata	Microgyniidae			CNGLF129-13	KM839854
Mesostigmata	Microgyniidae			CNGLF130-13	KM836793
Mesostigmata	Microgyniidae			CNGLF131-13	KM836500
Mesostigmata	Microgyniidae			CNGLF133-13	KM838947
Mesostigmata	Microgyniidae			CNGLF136-13	KM829621
Mesostigmata	Microgyniidae			CNGLF140-13	KM832497
Mesostigmata	Microgyniidae			CNGLF141-13	KM834323
Mesostigmata	Microgyniidae			CNGLF142-13	KM839459
Mesostigmata	Microgyniidae			MYMCA197-11	JX836313
Mesostigmata	Microgyniidae			SSBAE927-13	KM832713
Mesostigmata	Microgyniidae			SSBAE928-13	KM835167
Mesostigmata	Microgyniidae			SSBAE929-13	KM835462
Mesostigmata	Microgyniidae			SSBAE930-13	KM835070
Mesostigmata	Microgyniidae			SSBAE932-13	KM839820
Mesostigmata	Ologamasidae	*Gamasellus*		CHACA804-09	JX834214
Mesostigmata	Ologamasidae	*Gamasellus*		CHACB1195-10	HM907270
Mesostigmata	Ologamasidae	*Gamasellus*		MYMCE613-12	JX834971
Mesostigmata	Ologamasidae	*Gamasellus*		MYMCE614-12	JX836038

Mesostigmata	Ologamasidae	*Gamasellus*	MYMCG541-12	JX836436
Mesostigmata	Ologamasidae	*Stylochyrus rarior*	GBCH5106-10	GQ927176
Mesostigmata	Ologamasidae	*Stylochyrus rarior*	GBCH7977-13	HM159031
Mesostigmata	Ologamasidae	*Stylochyrus rarior*	GBCH7978-13	HM159030
Mesostigmata	Ologamasidae	*Stylochyrus rarior*	GBCH7979-13	HM159029
Mesostigmata	Ologamasidae	*Stylochyrus rarior*	GBCH7980-13	HM159028
Mesostigmata	Ologamasidae	*Stylochyrus rarior*	GBCH7981-13	HM159027
Mesostigmata	Ologamasidae	*Stylochyrus rarior*	GBCH7983-13	HM159025
Mesostigmata	Ologamasidae	*Stylochyrus rarior*	GBCH7984-13	HM159024
Mesostigmata	Ologamasidae	*Stylochyrus rarior*	GBCH7985-13	HM159023
Mesostigmata	Ologamasidae	*Stylochyrus rarior*	GBCH7986-13	HM159022
Mesostigmata	Ologamasidae	*Stylochyrus rarior*	GBCH7988-13	HM159020
Mesostigmata	Ologamasidae	*Stylochyrus rarior*	GBCH7990-13	HM159018
Mesostigmata	Ologamasidae	*Stylochyrus rarior*	GBCH7991-13	HM159017
Mesostigmata	Ologamasidae	*Stylochyrus rarior*	GBCH7996-13	HM159012
Mesostigmata	Ologamasidae	*Stylochyrus rarior*	GBCH8002-13	HM159006
Mesostigmata	Ologamasidae	*Stylochyrus rarior*	GBCH8003-13	HM159005
Mesostigmata	Ologamasidae	*Stylochyrus rarior*	GBCH8004-13	HM159004
Mesostigmata	Ologamasidae	*Stylochyrus rarior*	GBCH8007-13	HM159001
Mesostigmata	Ologamasidae	*Stylochyrus rarior*	GBCH8011-13	HM158997
Mesostigmata	Ologamasidae		CHACA027-08	JX834168
Mesostigmata	Ologamasidae		CHACA028-08	JX835092
Mesostigmata	Ologamasidae		CHACA096-08	JX834808
Mesostigmata	Ologamasidae		CHACA1000-10	HM405807
Mesostigmata	Ologamasidae		CHACA548-09	KP979349
Mesostigmata	Ologamasidae		CHACA629-09	JX838538
Mesostigmata	Ologamasidae		CHACA633-09	JX834141
Mesostigmata	Ologamasidae		CHACA634-09	JX836326
Mesostigmata	Ologamasidae		CHACA635-09	JX833682
Mesostigmata	Ologamasidae		CHACB041-10	HQ558354
Mesostigmata	Ologamasidae		CHACB1100-10	JX835235
Mesostigmata	Ologamasidae		CHACB1101-10	JX835912
Mesostigmata	Ologamasidae		CHACB1102-10	HM907200
Mesostigmata	Ologamasidae		CHACB1137-10	HM907227
Mesostigmata	Ologamasidae		CHACB1138-10	HM907228
Mesostigmata	Ologamasidae		CHACB199-10	HQ558469
Mesostigmata	Ologamasidae		CHACB426-10	HQ558583
Mesostigmata	Ologamasidae		CHACB427-10	JX835756
Mesostigmata	Ologamasidae		CHACB428-10	HQ558584
Mesostigmata	Ologamasidae		CHACB446-10	JX835683
Mesostigmata	Ologamasidae		CHACB479-10	HQ558617
Mesostigmata	Ologamasidae		CHACB558-10	HQ558673
Mesostigmata	Ologamasidae		CHACB723-10	HQ558773
Mesostigmata	Ologamasidae		CHACB836-10	HQ941508
Mesostigmata	Ologamasidae		CHACC069-10	HM907464
Mesostigmata	Ologamasidae		CHACC070-10	HM907465
Mesostigmata	Ologamasidae		CHACC071-10	HM907466
Mesostigmata	Ologamasidae		CHACC155-10	JX837455
Mesostigmata	Ologamasidae		CHACC156-10	JX836874
Mesostigmata	Ologamasidae		CHACC157-10	JX837744
Mesostigmata	Ologamasidae		CHACC158-10	JX833973
Mesostigmata	Ologamasidae		CHACC225-10	HQ941539
Mesostigmata	Ologamasidae		CHACC226-10	HQ941540

Mesostigmata	Ologamasidae	MIONB018-10	KR069701
Mesostigmata	Ologamasidae	MIONB019-10	KP979136
Mesostigmata	Ologamasidae	MITMH019-07	KR069373
Mesostigmata	Ologamasidae	MYMCA363-11	JX836679
Mesostigmata	Ologamasidae	MYMCA364-11	JX838289
Mesostigmata	Ologamasidae	MYMCA365-11	JX836196
Mesostigmata	Ologamasidae	MYMCA398-11	JX837564
Mesostigmata	Ologamasidae	MYMCA514-11	JX836175
Mesostigmata	Ologamasidae	MYMCA515-11	JX834044
Mesostigmata	Ologamasidae	MYMCA516-11	JX835771
Mesostigmata	Ologamasidae	MYMCB103-11	JX835669
Mesostigmata	Ologamasidae	MYMCB238-11	JX838363
Mesostigmata	Ologamasidae	MYMCB268-11	JX835701
Mesostigmata	Ologamasidae	MYMCB511-11	JX837781
Mesostigmata	Ologamasidae	MYMCB557-11	JX833956
Mesostigmata	Ologamasidae	MYMCB571-11	JX834272
Mesostigmata	Ologamasidae	MYMCC047-11	JX834459
Mesostigmata	Ologamasidae	MYMCC048-11	JX834914
Mesostigmata	Ologamasidae	MYMCC049-11	JX836528
Mesostigmata	Ologamasidae	MYMCC080-11	JX837675
Mesostigmata	Ologamasidae	MYMCC181-11	JX835900
Mesostigmata	Ologamasidae	MYMCC182-11	JX834962
Mesostigmata	Ologamasidae	MYMCC212-11	JX835740
Mesostigmata	Ologamasidae	MYMCC267-11	JX835875
Mesostigmata	Ologamasidae	MYMCC318-11	JX837399
Mesostigmata	Ologamasidae	MYMCC432-11	JX834549
Mesostigmata	Ologamasidae	MYMCC622-11	JX837430
Mesostigmata	Ologamasidae	MYMCC630-11	JX835362
Mesostigmata	Ologamasidae	MYMCC663-11	JX833724
Mesostigmata	Ologamasidae	MYMCE161-12	JX837086
Mesostigmata	Ologamasidae	MYMCE241-12	JX836858
Mesostigmata	Ologamasidae	MYMCE242-12	JX835059
Mesostigmata	Ologamasidae	MYMCE243-12	JX834500
Mesostigmata	Ologamasidae	MYMCE585-12	JX834021
Mesostigmata	Ologamasidae	MYMCE696-12	JX837697
Mesostigmata	Ologamasidae	MYMCF080-12	JX836011
Mesostigmata	Ologamasidae	MYMCF104-12	JX834539
Mesostigmata	Ologamasidae	MYMCF321-12	JX836514
Mesostigmata	Ologamasidae	MYMCF322-12	JX834593
Mesostigmata	Ologamasidae	MYMCF351-12	JX835703
Mesostigmata	Ologamasidae	MYMCF409-12	JX836121
Mesostigmata	Ologamasidae	MYMCF412-12	JX837686
Mesostigmata	Ologamasidae	MYMCF482-12	JX837136
Mesostigmata	Ologamasidae	MYMCF487-12	JX835185
Mesostigmata	Ologamasidae	MYMCF513-12	JX833846
Mesostigmata	Ologamasidae	MYMCF562-12	JX836429
Mesostigmata	Ologamasidae	MYMCF674-12	JX835682
Mesostigmata	Ologamasidae	MYMCG231-12	JX835914
Mesostigmata	Ologamasidae	MYMCG232-12	JX835597
Mesostigmata	Ologamasidae	MYMCG594-12	JX838189
Mesostigmata	Ologamasidae	MYMCG595-12	JX835764
Mesostigmata	Ologamasidae	MYMCG596-12	JX835056
Mesostigmata	Ologamasidae	MYMCG598-12	JX837465

Mesostigmata	Ologamasidae		MYMCG599-12	JX838239
Mesostigmata	Ologamasidae		MYTMC060-09	GU680466
Mesostigmata	Ologamasidae		SSWEE018-13	KM826049
Mesostigmata	Ologamasidae		SSWEE027-13	KM836535
Mesostigmata	Ologamasidae		SSWEE112-13	KM831023
Mesostigmata	Ologamasidae		SSWEE124-13	KM835309
Mesostigmata	Ologamasidae		SSWEE138-13	KM837980
Mesostigmata	Ologamasidae		SSWLA676-13	KM836814
Mesostigmata	Ologamasidae		SSWLC1159-13	KM838616
Mesostigmata	Ologamasidae		SSWLC4139-13	KM824733
Mesostigmata	Oplitidae		MIONB326-10	KR070118
Mesostigmata	Oplitidae		MIONB327-10	KR070265
Mesostigmata	Oplitidae		MIONB328-10	KP979276
Mesostigmata	Pachylaelapidae	*Pachylaelaps*	CHACB528-10	HQ558646
Mesostigmata	Pachylaelapidae	*Pachylaelaps*	CHACB529-10	HQ558647
Mesostigmata	Pachylaelapidae	*Pachylaelaps*	CHACB530-10	HQ558648
Mesostigmata	Pachylaelapidae	*Pachylaelaps*	CHACB531-10	HQ558649
Mesostigmata	Pachylaelapidae	*Pachylaelaps*	CHACB532-10	JX836252
Mesostigmata	Pachylaelapidae		BBLZI079-14	KR069573
Mesostigmata	Pachylaelapidae		CHACA517-09	JX837563
Mesostigmata	Pachylaelapidae		RBINA5685-13	KP979258
Mesostigmata	Parasitidae		CNVUA949-14	KR069605
Mesostigmata	Parasitidae		CNVUA950-14	KR070523
Mesostigmata	Parasitidae		CNVUA952-14	KR070432
Mesostigmata	Parasitidae		SSBRB5263-14	KR070414
Mesostigmata	Parasitidae		SSGBA5098-14	KR070229
Mesostigmata	Parasitidae		SSGBA5099-14	KR070346
Mesostigmata	Parasitidae	*Aclerogamasus similis*	GBCH7726-13	FJ577985
Mesostigmata	Parasitidae	*Aclerogamasus similis*	GBCH7727-13	FJ577984
Mesostigmata	Parasitidae	*Aclerogamasus similis*	GBCH7728-13	FJ577983
Mesostigmata	Parasitidae	*Aclerogamasus similis*	GBCH7729-13	FJ577982
Mesostigmata	Parasitidae	*Aclerogamasus similis*	GBCH7730-13	FJ577981
Mesostigmata	Parasitidae	*Aclerogamasus similis*	GBCH7731-13	FJ577980
Mesostigmata	Parasitidae	*Aclerogamasus similis*	GBCH7732-13	FJ577979
Mesostigmata	Parasitidae	*Aclerogamasus similis*	GBCH7733-13	FJ577978
Mesostigmata	Parasitidae	*Aclerogamasus similis*	GBCH7734-13	FJ577977
Mesostigmata	Parasitidae	*Aclerogamasus similis*	GBCH7735-13	FJ577976
Mesostigmata	Parasitidae	*Aclerogamasus similis*	GBCH7736-13	FJ577975
Mesostigmata	Parasitidae		ARCN084-10	HQ924305
Mesostigmata	Parasitidae		ARCN085-10	HQ924306
Mesostigmata	Parasitidae		ARCN086-10	HQ924307
Mesostigmata	Parasitidae		ARCN142-10	KM831407
Mesostigmata	Parasitidae		ARCN143-10	HQ924350
Mesostigmata	Parasitidae		ARCN157-10	HQ924362
Mesostigmata	Parasitidae		ARCN158-10	HQ924363
Mesostigmata	Parasitidae		ARCN159-10	HQ924364
Mesostigmata	Parasitidae		ARCN162-10	KM825962
Mesostigmata	Parasitidae		ARCN163-10	HQ924365
Mesostigmata	Parasitidae		ARCN164-10	HQ924366
Mesostigmata	Parasitidae		CHACA092-08	JX834035
Mesostigmata	Parasitidae		CHACA1146-10	JX837364
Mesostigmata	Parasitidae		CHACA1147-10	JX834513
Mesostigmata	Parasitidae		CHACA1148-10	JX838501

Mesostigmata	Parasitidae	CHACA412-09	JX833970
Mesostigmata	Parasitidae	CHACA413-09	JX833944
Mesostigmata	Parasitidae	CHACA549-09	JX836239
Mesostigmata	Parasitidae	CHACB1140-10	HM907229
Mesostigmata	Parasitidae	CHACB1141-10	HM907230
Mesostigmata	Parasitidae	CHACC154-10	JX834529
Mesostigmata	Parasitidae	CNEIA2552-12	KM831468
Mesostigmata	Parasitidae	CNEIA2553-12	KM827019
Mesostigmata	Parasitidae	CNEIA2554-12	KM836035
Mesostigmata	Parasitidae	CNEIA2559-12	KM838783
Mesostigmata	Parasitidae	CNEIA2561-12	KM831587
Mesostigmata	Parasitidae	CNEIA2562-12	KM836438
Mesostigmata	Parasitidae	CNEIA2572-12	KM833181
Mesostigmata	Parasitidae	CNEIA2586-12	KM827922
Mesostigmata	Parasitidae	CNEIA2593-12	KM832815
Mesostigmata	Parasitidae	CNEIA2595-12	KM824442
Mesostigmata	Parasitidae	CNEIA2596-12	KM839569
Mesostigmata	Parasitidae	CNEIA2599-12	KM831671
Mesostigmata	Parasitidae	CNEIA2607-12	KM829041
Mesostigmata	Parasitidae	CNEIA2609-12	KM834257
Mesostigmata	Parasitidae	CNEIA2613-12	KM828679
Mesostigmata	Parasitidae	CNEIA2619-12	KM838773
Mesostigmata	Parasitidae	CNEIA2625-12	KM830257
Mesostigmata	Parasitidae	CNEIA2633-12	KM835345
Mesostigmata	Parasitidae	CNEIF2391-12	KM837161
Mesostigmata	Parasitidae	CNEIF2395-12	KM827003
Mesostigmata	Parasitidae	CNEIH061-13	KM834386
Mesostigmata	Parasitidae	CNEIH064-13	KM835965
Mesostigmata	Parasitidae	CNJAA868-12	KM833285
Mesostigmata	Parasitidae	CNJAA869-12	KM829901
Mesostigmata	Parasitidae	CNJAC1112-12	KM836618
Mesostigmata	Parasitidae	CNJAC1121-12	KM837576
Mesostigmata	Parasitidae	CNJAC1437-12	KM829710
Mesostigmata	Parasitidae	CNJAC1441-12	KM825910
Mesostigmata	Parasitidae	CNJAC1491-12	KM836076
Mesostigmata	Parasitidae	CNJAD1817-12	KM837589
Mesostigmata	Parasitidae	CNJAF1920-12	KM827850
Mesostigmata	Parasitidae	CNJAF1929-12	KM833057
Mesostigmata	Parasitidae	CNJAF1951-12	KM836286
Mesostigmata	Parasitidae	CNJAF1987-12	KM831281
Mesostigmata	Parasitidae	CNJAF1989-12	KM832670
Mesostigmata	Parasitidae	CNJAF1990-12	KM840720
Mesostigmata	Parasitidae	CNJAF2001-12	KM838380
Mesostigmata	Parasitidae	CNJAG1818-12	KM839204
Mesostigmata	Parasitidae	CNKOG1173-14	KR070562
Mesostigmata	Parasitidae	CNPAA458-13	KM831633
Mesostigmata	Parasitidae	CNPAA459-13	KM826184
Mesostigmata	Parasitidae	CNPAA460-13	KM838476
Mesostigmata	Parasitidae	CNPAB269-13	KM830196
Mesostigmata	Parasitidae	CNPAB270-13	KM839341
Mesostigmata	Parasitidae	CNPAB271-13	KM824647
Mesostigmata	Parasitidae	CNPAB278-13	KM825543
Mesostigmata	Parasitidae	CNPAB279-13	KM827480

Mesostigmata	Parasitidae	CNPAB281-13	KM824493
Mesostigmata	Parasitidae	CNPAB282-13	KM837358
Mesostigmata	Parasitidae	CNPAB283-13	KM834660
Mesostigmata	Parasitidae	CNPAB284-13	KM839679
Mesostigmata	Parasitidae	CNPAB285-13	KM824826
Mesostigmata	Parasitidae	CNPAB286-13	KM829946
Mesostigmata	Parasitidae	CNPAB287-13	KM828661
Mesostigmata	Parasitidae	CNPAB288-13	KM830766
Mesostigmata	Parasitidae	CNPAB290-13	KM839352
Mesostigmata	Parasitidae	CNPAC548-13	KM833289
Mesostigmata	Parasitidae	CNPAC549-13	KM824839
Mesostigmata	Parasitidae	CNPAC550-13	KM824692
Mesostigmata	Parasitidae	CNPAD843-13	KM838190
Mesostigmata	Parasitidae	CNPAD844-13	KM827303
Mesostigmata	Parasitidae	CNPAD845-13	KM829209
Mesostigmata	Parasitidae	CNPAD846-13	KM840357
Mesostigmata	Parasitidae	CNPAD847-13	KM828479
Mesostigmata	Parasitidae	CNPAD848-13	KM837300
Mesostigmata	Parasitidae	CNPAD849-13	KM837787
Mesostigmata	Parasitidae	CNPAD850-13	KM825595
Mesostigmata	Parasitidae	CNPAD851-13	KM833441
Mesostigmata	Parasitidae	CNPAD852-13	KM826702
Mesostigmata	Parasitidae	CNPAD853-13	KM840151
Mesostigmata	Parasitidae	CNPAD854-13	KM827119
Mesostigmata	Parasitidae	CNPAD855-13	KM834739
Mesostigmata	Parasitidae	CNPAD856-13	KM831875
Mesostigmata	Parasitidae	CNPAD857-13	KM835013
Mesostigmata	Parasitidae	CNPAD858-13	KM833580
Mesostigmata	Parasitidae	CNPAF920-13	KM831147
Mesostigmata	Parasitidae	CNPPC1941-12	KJ084253
Mesostigmata	Parasitidae	CNPPC1942-12	KJ090830
Mesostigmata	Parasitidae	CNPPC1943-12	KJ089709
Mesostigmata	Parasitidae	CNPPC1944-12	KJ091913
Mesostigmata	Parasitidae	CNPPC1945-12	KJ089933
Mesostigmata	Parasitidae	CNPPC1948-12	KJ164224
Mesostigmata	Parasitidae	CNPPC1949-12	KJ092668
Mesostigmata	Parasitidae	CNPPC1950-12	KJ089125
Mesostigmata	Parasitidae	CNPPC1951-12	KJ084886
Mesostigmata	Parasitidae	CNPPC1952-12	KJ087040
Mesostigmata	Parasitidae	CNPPC1953-12	KJ089484
Mesostigmata	Parasitidae	CNPPC1954-12	KJ089510
Mesostigmata	Parasitidae	CNPPC1955-12	KJ164259
Mesostigmata	Parasitidae	CNPPD2631-12	KJ090073
Mesostigmata	Parasitidae	CNPPD2652-12	KJ085730
Mesostigmata	Parasitidae	CNPPD2657-12	KJ086348
Mesostigmata	Parasitidae	CNPPE1435-12	KJ085476
Mesostigmata	Parasitidae	CNPPE1436-12	KJ085482
Mesostigmata	Parasitidae	CNPPE1437-12	KJ166277
Mesostigmata	Parasitidae	CNPPE1447-12	KJ167289
Mesostigmata	Parasitidae	CNPPE1450-12	KJ089988
Mesostigmata	Parasitidae	CNPPE2115-12	KJ088847
Mesostigmata	Parasitidae	CNPPE2132-12	KJ087190
Mesostigmata	Parasitidae	CNPPF1042-12	KJ167902

Mesostigmata	Parasitidae	CNRMC1510-12	KM826777
Mesostigmata	Parasitidae	CNRMC1678-12	KM839826
Mesostigmata	Parasitidae	CNRMD2667-12	KM836364
Mesostigmata	Parasitidae	CNRMD2679-12	KM836458
Mesostigmata	Parasitidae	CNRMD2687-12	KM827024
Mesostigmata	Parasitidae	CNRMD2691-12	KM839179
Mesostigmata	Parasitidae	CNRMD2694-12	KM832775
Mesostigmata	Parasitidae	CNRMD2699-12	KM829018
Mesostigmata	Parasitidae	CNRMD2700-12	KM833845
Mesostigmata	Parasitidae	CNRMD2702-12	KM833933
Mesostigmata	Parasitidae	CNRME4703-12	KM824838
Mesostigmata	Parasitidae	CNRME4705-12	KM838701
Mesostigmata	Parasitidae	CNRME4707-12	KM839526
Mesostigmata	Parasitidae	CNRME4715-12	KM825337
Mesostigmata	Parasitidae	CNRME4717-12	KM825476
Mesostigmata	Parasitidae	CNRME4718-12	KM827787
Mesostigmata	Parasitidae	CNRME4722-12	KM839266
Mesostigmata	Parasitidae	CNRME4732-12	KM840120
Mesostigmata	Parasitidae	CNRME4733-12	KM831638
Mesostigmata	Parasitidae	CNRME4741-12	KM826316
Mesostigmata	Parasitidae	CNRME4762-12	KM825132
Mesostigmata	Parasitidae	CNRME4765-12	KM833617
Mesostigmata	Parasitidae	CNRME4785-12	KM840305
Mesostigmata	Parasitidae	CNRMF4054-12	KM829707
Mesostigmata	Parasitidae	CNSLF644-12	KM828477
Mesostigmata	Parasitidae	CNSLF647-12	KM830372
Mesostigmata	Parasitidae	CNSLF653-12	KM832387
Mesostigmata	Parasitidae	CNSLF654-12	KM836230
Mesostigmata	Parasitidae	CNSLF659-12	KM824718
Mesostigmata	Parasitidae	CNSLF661-12	KM839980
Mesostigmata	Parasitidae	CNSLJ030-12	KM831122
Mesostigmata	Parasitidae	CNSLJ436-12	KM832999
Mesostigmata	Parasitidae	CNWBE661-13	KM834437
Mesostigmata	Parasitidae	CNWBE664-13	KM829042
Mesostigmata	Parasitidae	CNWBE689-13	KM826384
Mesostigmata	Parasitidae	CNWBE709-13	KM825696
Mesostigmata	Parasitidae	CNWBE713-13	KM830021
Mesostigmata	Parasitidae	CNWBE715-13	KM832525
Mesostigmata	Parasitidae	CNWBE727-13	KM824937
Mesostigmata	Parasitidae	CNWLD1147-13	KM830817
Mesostigmata	Parasitidae	CNWLD1149-13	KM829328
Mesostigmata	Parasitidae	CNWLD1150-13	KM828308
Mesostigmata	Parasitidae	CNWLD1151-13	KM824112
Mesostigmata	Parasitidae	CNWLD1153-13	KM837871
Mesostigmata	Parasitidae	CNWLD1159-13	KM828427
Mesostigmata	Parasitidae	CNWLE2562-13	KM830731
Mesostigmata	Parasitidae	CNWLE2565-13	KM826314
Mesostigmata	Parasitidae	CNWLE2566-13	KM834931
Mesostigmata	Parasitidae	CNWLE2578-13	KM832826
Mesostigmata	Parasitidae	CNWLF136-12	KM840126
Mesostigmata	Parasitidae	CNWLF153-12	KM828540
Mesostigmata	Parasitidae	CNWLF168-12	KM833712
Mesostigmata	Parasitidae	JSJUL2404-12	KR069551

Mesostigmata	Parasitidae	JSJUL2526-12	KR070012
Mesostigmata	Parasitidae	JSJUL2537-12	KR069334
Mesostigmata	Parasitidae	JSOIE052-12	KR070599
Mesostigmata	Parasitidae	JSOIE060-12	KR070088
Mesostigmata	Parasitidae	JSOIE069-12	KR070138
Mesostigmata	Parasitidae	JSOIE082-12	KR070677
Mesostigmata	Parasitidae	JSOIE086-12	KR069832
Mesostigmata	Parasitidae	JSOIE092-12	KR069734
Mesostigmata	Parasitidae	JSOIE100-12	KP979353
Mesostigmata	Parasitidae	JSOIE104-12	KR070435
Mesostigmata	Parasitidae	JSOIE112-12	KR070231
Mesostigmata	Parasitidae	MIONB011-10	HM887562
Mesostigmata	Parasitidae	MIONB012-10	HM887563
Mesostigmata	Parasitidae	MIONB014-10	HM887564
Mesostigmata	Parasitidae	MIONB037-10	HM887574
Mesostigmata	Parasitidae	MIONB038-10	HM887575
Mesostigmata	Parasitidae	MIONB106-10	KR069580
Mesostigmata	Parasitidae	MIONB212-10	KP979222
Mesostigmata	Parasitidae	MIONB213-10	KP979297
Mesostigmata	Parasitidae	MIONB243-10	KR069398
Mesostigmata	Parasitidae	MIONB244-10	KR070739
Mesostigmata	Parasitidae	MIONB245-10	KR069294
Mesostigmata	Parasitidae	MIONB246-10	KR069486
Mesostigmata	Parasitidae	MIONB247-10	KR070339
Mesostigmata	Parasitidae	MIONB248-10	KR070029
Mesostigmata	Parasitidae	MIONB340-10	KR070127
Mesostigmata	Parasitidae	MIONB341-10	KR070151
Mesostigmata	Parasitidae	MIONB356-10	KR069202
Mesostigmata	Parasitidae	MIONB358-10	KR070143
Mesostigmata	Parasitidae	MIONB359-10	KR069485
Mesostigmata	Parasitidae	MIONB361-10	KR070478
Mesostigmata	Parasitidae	MIONB362-10	KR070020
Mesostigmata	Parasitidae	MIONB363-10	KR070063
Mesostigmata	Parasitidae	MIONB366-10	KR069538
Mesostigmata	Parasitidae	MIONB382-10	KR070025
Mesostigmata	Parasitidae	MIONB420-10	KP979275
Mesostigmata	Parasitidae	MIONB421-10	KR070708
Mesostigmata	Parasitidae	MIONB431-10	KP979330
Mesostigmata	Parasitidae	MIONB444-10	KR070384
Mesostigmata	Parasitidae	MIONB454-11	KM834295
Mesostigmata	Parasitidae	MIONB455-11	KM837107
Mesostigmata	Parasitidae	MIONB456-11	KM826794
Mesostigmata	Parasitidae	MIONB457-11	KM824139
Mesostigmata	Parasitidae	MIONB458-11	KM829379
Mesostigmata	Parasitidae	MIONB459-11	KM824565
Mesostigmata	Parasitidae	MIONB474-11	KM833471
Mesostigmata	Parasitidae	MIONB475-11	KM837494
Mesostigmata	Parasitidae	MIONB476-11	KM834579
Mesostigmata	Parasitidae	MIONB477-11	KM824494
Mesostigmata	Parasitidae	MIONB481-11	KM827008
Mesostigmata	Parasitidae	MIONB482-11	KM827352
Mesostigmata	Parasitidae	MIONB507-11	KM837877
Mesostigmata	Parasitidae	MIONB508-11	KM834831

Mesostigmata	Parasitidae	MITEP020-10	KM837446
Mesostigmata	Parasitidae	MITEP021-10	KM830506
Mesostigmata	Parasitidae	MITEP022-10	KM835124
Mesostigmata	Parasitidae	MITEP072-10	KM830967
Mesostigmata	Parasitidae	MITEP075-10	KM829193
Mesostigmata	Parasitidae	MYMCB222-11	JX834740
Mesostigmata	Parasitidae	MYMCB223-11	JX834859
Mesostigmata	Parasitidae	MYMCB426-11	JX837039
Mesostigmata	Parasitidae	MYMCD072-11	JX837723
Mesostigmata	Parasitidae	MYMCE208-12	JX836480
Mesostigmata	Parasitidae	MYMCE263-12	JX834329
Mesostigmata	Parasitidae	MYMCE270-12	JX837358
Mesostigmata	Parasitidae	MYMCE271-12	JX837706
Mesostigmata	Parasitidae	MYMCE272-12	JX833767
Mesostigmata	Parasitidae	MYMCE280-12	JX837200
Mesostigmata	Parasitidae	MYMCE359-12	JX838577
Mesostigmata	Parasitidae	MYMCE360-12	JX838772
Mesostigmata	Parasitidae	MYMCE361-12	JX835899
Mesostigmata	Parasitidae	MYMCE362-12	JX836408
Mesostigmata	Parasitidae	MYMCF882-12	JX837827
Mesostigmata	Parasitidae	MYMCF883-12	JX835060
Mesostigmata	Parasitidae	MYMCG525-12	JX836100
Mesostigmata	Parasitidae	MYMCG573-12	JX833753
Mesostigmata	Parasitidae	PHJUL986-11	KR070318
Mesostigmata	Parasitidae	PHJUL988-11	KR069993
Mesostigmata	Parasitidae	PHJUL989-11	KR069581
Mesostigmata	Parasitidae	PHJUL990-11	KR069958
Mesostigmata	Parasitidae	PHMTT635-10	KP979168
Mesostigmata	Parasitidae	PHOCT904-11	KR069707
Mesostigmata	Parasitidae	RBINA5623-13	KP979167
Mesostigmata	Parasitidae	SMTPB20119-13	KR069885
Mesostigmata	Parasitidae	SMTPB3363-13	KR070735
Mesostigmata	Parasitidae	SMTPB5534-13	KR069672
Mesostigmata	Parasitidae	SSBAA2016-12	KM834769
Mesostigmata	Parasitidae	SSBAA2044-12	KM830768
Mesostigmata	Parasitidae	SSBAA2062-12	KM830369
Mesostigmata	Parasitidae	SSBAA2064-12	KM826174
Mesostigmata	Parasitidae	SSBAA2067-12	KM833197
Mesostigmata	Parasitidae	SSBAA2068-12	KM840343
Mesostigmata	Parasitidae	SSBAA2070-12	KM838350
Mesostigmata	Parasitidae	SSBAA2071-12	KM830293
Mesostigmata	Parasitidae	SSBAA2089-12	KM838534
Mesostigmata	Parasitidae	SSBAA3611-12	KM829744
Mesostigmata	Parasitidae	SSBAA3623-12	KM839617
Mesostigmata	Parasitidae	SSBAA3657-12	KM832228
Mesostigmata	Parasitidae	SSBAA5064-12	KM826789
Mesostigmata	Parasitidae	SSBAB1146-12	KM826934
Mesostigmata	Parasitidae	SSBAB118-12	KM838704
Mesostigmata	Parasitidae	SSBAB1203-12	KM832606
Mesostigmata	Parasitidae	SSBAB1210-12	KM835305
Mesostigmata	Parasitidae	SSBAB1211-12	KM831083
Mesostigmata	Parasitidae	SSBAB121-12	KM829603
Mesostigmata	Parasitidae	SSBAB1212-12	KM825191

Mesostigmata	Parasitidae	SSBAB1213-12	KM830738
Mesostigmata	Parasitidae	SSBAB1214-12	KM824917
Mesostigmata	Parasitidae	SSBAB1217-12	KM825839
Mesostigmata	Parasitidae	SSBAB125-12	KM831397
Mesostigmata	Parasitidae	SSBAB2864-12	KM839154
Mesostigmata	Parasitidae	SSBAB2865-12	KM826131
Mesostigmata	Parasitidae	SSBAC2381-12	KM837680
Mesostigmata	Parasitidae	SSBAC3303-12	KM837033
Mesostigmata	Parasitidae	SSBAD3063-12	KM839548
Mesostigmata	Parasitidae	SSBAD3092-12	KM832213
Mesostigmata	Parasitidae	SSBAD3111-12	KM839940
Mesostigmata	Parasitidae	SSBAD3113-12	KM835150
Mesostigmata	Parasitidae	SSBAD3118-12	KM837467
Mesostigmata	Parasitidae	SSBAD3122-12	KM835126
Mesostigmata	Parasitidae	SSBAD3126-12	KM840270
Mesostigmata	Parasitidae	SSBAD3133-12	KM832709
Mesostigmata	Parasitidae	SSBAD3969-12	KM839319
Mesostigmata	Parasitidae	SSBAD3981-12	KM834742
Mesostigmata	Parasitidae	SSBAD4703-13	KM838908
Mesostigmata	Parasitidae	SSBAD4704-13	KM832630
Mesostigmata	Parasitidae	SSBAD4719-13	KM828580
Mesostigmata	Parasitidae	SSBAD4721-13	KM840382
Mesostigmata	Parasitidae	SSBAD4772-13	KM827965
Mesostigmata	Parasitidae	SSBAD5590-13	KM838797
Mesostigmata	Parasitidae	SSBAE3007-13	KM824726
Mesostigmata	Parasitidae	SSBAE3008-13	KM826961
Mesostigmata	Parasitidae	SSBAE3324-13	KM829827
Mesostigmata	Parasitidae	SSBAE3632-13	KM825093
Mesostigmata	Parasitidae	SSBAF4452-13	KM826051
Mesostigmata	Parasitidae	SSBAF4461-13	KM830260
Mesostigmata	Parasitidae	SSBAF4464-13	KM833342
Mesostigmata	Parasitidae	SSBAF4465-13	KM832395
Mesostigmata	Parasitidae	SSBAF4470-13	KM826127
Mesostigmata	Parasitidae	SSBAF4480-13	KM840295
Mesostigmata	Parasitidae	SSBAF4483-13	KM839276
Mesostigmata	Parasitidae	SSEIA038-13	KM837154
Mesostigmata	Parasitidae	SSEIA041-13	KM832536
Mesostigmata	Parasitidae	SSEIA042-13	KM834530
Mesostigmata	Parasitidae	SSEIA047-13	KM829157
Mesostigmata	Parasitidae	SSEIA048-13	KM828607
Mesostigmata	Parasitidae	SSEIA051-13	KM824844
Mesostigmata	Parasitidae	SSEIA053-13	KM839096
Mesostigmata	Parasitidae	SSEIA054-13	KM832581
Mesostigmata	Parasitidae	SSEIA058-13	KM830561
Mesostigmata	Parasitidae	SSEIA2085-13	KM825437
Mesostigmata	Parasitidae	SSEIA2094-13	KM838543
Mesostigmata	Parasitidae	SSEIA3001-13	KM835275
Mesostigmata	Parasitidae	SSEIA3027-13	KM829492
Mesostigmata	Parasitidae	SSEIA3032-13	KM831884
Mesostigmata	Parasitidae	SSEIA3037-13	KM838559
Mesostigmata	Parasitidae	SSEIA7686-13	KM838058
Mesostigmata	Parasitidae	SSEIA7692-13	KM831051
Mesostigmata	Parasitidae	SSEIA7696-13	KM831398

Mesostigmata	Parasitidae		SSEIB4239-13	KM840376
Mesostigmata	Parasitidae		SSEIB4544-13	KM835505
Mesostigmata	Parasitidae		SSEIB4565-13	KM838121
Mesostigmata	Parasitidae		SSEIB4578-13	KM837821
Mesostigmata	Parasitidae		SSEIB5406-13	KM830771
Mesostigmata	Parasitidae		SSEIB5413-13	KM826269
Mesostigmata	Parasitidae		SSEIB5424-13	KM830756
Mesostigmata	Parasitidae		SSEIB5798-13	KM839716
Mesostigmata	Parasitidae		SSEIB5799-13	KM833648
Mesostigmata	Parasitidae		SSEIB7630-13	KM830663
Mesostigmata	Parasitidae		SSEIB7639-13	KM836008
Mesostigmata	Parasitidae		SSEIB7660-13	KM840479
Mesostigmata	Parasitidae		SSJAA1978-13	KM832236
Mesostigmata	Parasitidae		SSJAB1002-13	KM838651
Mesostigmata	Parasitidae		SSJAB1841-13	KM839478
Mesostigmata	Parasitidae		SSJAB1870-13	KM836247
Mesostigmata	Parasitidae		SSJAB3019-13	KM838296
Mesostigmata	Parasitidae		SSJAC177-13	KM826430
Mesostigmata	Parasitidae		SSJAC188-13	KM831478
Mesostigmata	Parasitidae		SSJAF2624-13	KM826347
Mesostigmata	Parasitidae		SSJAF2633-13	KM832439
Mesostigmata	Parasitidae		SSJAF3123-13	KM830381
Mesostigmata	Parasitidae		SSJAF5522-13	KM827015
Mesostigmata	Parasitidae		SSJAF5527-13	KM829907
Mesostigmata	Parasitidae		SSJAF5635-13	KM826485
Mesostigmata	Parasitidae		SSPAA2217-13	KM828635
Mesostigmata	Parasitidae		SSPAA6708-13	KM824915
Mesostigmata	Parasitidae		SSPAA7830-13	KM828118
Mesostigmata	Parasitidae		SSPAB7516-13	KM835018
Mesostigmata	Parasitidae		SSPAB7517-13	KM837076
Mesostigmata	Parasitidae		SSPAB8000-13	KM835256
Mesostigmata	Parasitidae		SSWLA043-13	KM839366
Mesostigmata	Parasitidae		SSWLA1102-13	KM824177
Mesostigmata	Parasitidae		SSWLA5216-13	KM839984
Mesostigmata	Parasitidae		SSWLA5269-13	KM824418
Mesostigmata	Parasitidae		SSWLB1517-13	KM836516
Mesostigmata	Parasitidae		SSWLB1518-13	KM830662
Mesostigmata	Parasitidae		SSWLB2433-13	KM835615
Mesostigmata	Parasitidae		SSWLB2450-13	KM824101
Mesostigmata	Parasitidae		SSWLC058-13	KM827826
Mesostigmata	Parasitidae		SSWLC060-13	KM840218
Mesostigmata	Parasitidae		TDWGB353-10	KR070181
Mesostigmata	Parasitidae		TDWGB354-10	KP979244
Mesostigmata	Parasitidae		TDWGB355-10	KR070299
Mesostigmata	Parasitidae		TDWGB356-10	KR069806
Mesostigmata	Paratennulidae		MIONB347-10	KR069522
Mesostigmata	Paratennulidae		MIONB348-10	KP979211
Mesostigmata	Phytoseiidae		CNNHF1195-14	KR070209
Mesostigmata	Phytoseiidae		SSBRA2331-14	KR069838
Mesostigmata	Phytoseiidae	*Amblyseius degenerans*	GACAC133-12	FM210119
Mesostigmata	Phytoseiidae	*Amblyseius degenerans*	GACAC134-12	FM210120
Mesostigmata	Phytoseiidae	*Amblyseius degenerans*	GACAC135-12	FM210121
Mesostigmata	Phytoseiidae	*Amblyseius eharai*	GBCH11745-13	JX080345

Mesostigmata	Phytoseiidae	*Amblyseius eharai*	GBCH11746-13	JX080344
Mesostigmata	Phytoseiidae	*Amblyseius eharai*	GBCH11747-13	JX080343
Mesostigmata	Phytoseiidae	*Amblyseius eharai*	GBCH11748-13	JX080342
Mesostigmata	Phytoseiidae	*Amblyseius eharai*	GBCH11749-13	JX080341
Mesostigmata	Phytoseiidae	*Amblyseius eharai*	GBCH11750-13	JX080340
Mesostigmata	Phytoseiidae	*Amblyseius eharai*	GBCH11751-13	JX080339
Mesostigmata	Phytoseiidae	*Amblyseius eharai*	GBCH11752-13	JX080338
Mesostigmata	Phytoseiidae	*Amblyseius eharai*	GBCH11753-13	JX080337
Mesostigmata	Phytoseiidae	*Amblyseius eharai*	GBCH11754-13	JX080336
Mesostigmata	Phytoseiidae	*Amblyseius eharai*	GBCH11755-13	JX080335
Mesostigmata	Phytoseiidae	*Amblyseius eharai*	GBCH11756-13	JX080334
Mesostigmata	Phytoseiidae	*Amblyseius eharai*	GBCH11757-13	JX080333
Mesostigmata	Phytoseiidae	*Amblyseius eharai*	GBCH11758-13	JX080332
Mesostigmata	Phytoseiidae	*Amblyseius eharai*	GBCH11759-13	JX080331
Mesostigmata	Phytoseiidae	*Amblyseius herbicolus*	GBCH11760-13	JX080330
Mesostigmata	Phytoseiidae	*Amblyseius herbicolus*	GBCH11761-13	JX080329
Mesostigmata	Phytoseiidae	*Amblyseius herbicolus*	GBCH11762-13	JX080328
Mesostigmata	Phytoseiidae	*Amblyseius herbicolus*	GBCH11763-13	JX080327
Mesostigmata	Phytoseiidae	*Amblyseius herbicolus*	GBCH11764-13	JX080326
Mesostigmata	Phytoseiidae	*Amblyseius largoensis*	GBCH11741-13	JX080349
Mesostigmata	Phytoseiidae	*Amblyseius largoensis*	GBCH11742-13	JX080348
Mesostigmata	Phytoseiidae	*Amblyseius largoensis*	GBCH11743-13	JX080347
Mesostigmata	Phytoseiidae	*Amblyseius largoensis*	GBCH11744-13	JX080346
Mesostigmata	Phytoseiidae	*Euseius alangii*	GBCH11776-13	JX080314
Mesostigmata	Phytoseiidae	*Euseius alangii*	GBCH11777-13	JX080313
Mesostigmata	Phytoseiidae	*Euseius nicholsi*	GBCH7570-13	JQ864562
Mesostigmata	Phytoseiidae	*Euseius nicholsi*	GBCH7571-13	JQ864561
Mesostigmata	Phytoseiidae	*Euseius nicholsi*	GBCH7572-13	JQ864560
Mesostigmata	Phytoseiidae	*Euseius nicholsi*	GBCH7573-13	JQ864559
Mesostigmata	Phytoseiidae	*Euseius nicholsi*	GBCH7574-13	JQ864558
Mesostigmata	Phytoseiidae	*Euseius nicholsi*	GBCH7575-13	JQ864557
Mesostigmata	Phytoseiidae	*Euseius nicholsi*	GBCH7576-13	JQ864556
Mesostigmata	Phytoseiidae	*Euseius nicholsi*	GBCH7577-13	JQ864555
Mesostigmata	Phytoseiidae	*Euseius nicholsi*	GBCH7578-13	JQ864554
Mesostigmata	Phytoseiidae	*Euseius nicholsi*	GBCH7579-13	JQ864553
Mesostigmata	Phytoseiidae	*Euseius nicholsi*	GBCH7580-13	JQ864552
Mesostigmata	Phytoseiidae	*Euseius nicholsi*	GBCH7581-13	JQ864551
Mesostigmata	Phytoseiidae	*Euseius nicholsi*	GBCH7582-13	JQ864550
Mesostigmata	Phytoseiidae	*Euseius nicholsi*	GBCH7583-13	JQ864549
Mesostigmata	Phytoseiidae	*Euseius nicholsi*	GBCH7584-13	JQ864548
Mesostigmata	Phytoseiidae	*Euseius nicholsi*	GBCH7585-13	JQ864547
Mesostigmata	Phytoseiidae	*Euseius nicholsi*	GBCH7586-13	JQ864546
Mesostigmata	Phytoseiidae	*Euseius nicholsi*	GBCH7587-13	JQ864545
Mesostigmata	Phytoseiidae	*Euseius nicholsi*	GBCH7588-13	JQ864544
Mesostigmata	Phytoseiidae	*Euseius nicholsi*	GBCH7589-13	JQ864543
Mesostigmata	Phytoseiidae	*Euseius nicholsi*	GBCH7590-13	JQ864542
Mesostigmata	Phytoseiidae	*Euseius nicholsi*	GBCH7591-13	JQ864541
Mesostigmata	Phytoseiidae	*Euseius nicholsi*	GBCH7592-13	JQ864540
Mesostigmata	Phytoseiidae	*Euseius nicholsi*	GBCH7593-13	JQ864539
Mesostigmata	Phytoseiidae	*Euseius nicholsi*	GBCH7594-13	JQ864538
Mesostigmata	Phytoseiidae	*Euseius nicholsi*	GBCH7595-13	JQ864537
Mesostigmata	Phytoseiidae	*Euseius nicholsi*	GBCH7596-13	JQ864536
Mesostigmata	Phytoseiidae	*Euseius nicholsi*	GBCH7597-13	JQ864535

Mesostigmata	Phytoseiidae	*Euseius nicholsi*	GBCH7598-13	JQ864534
Mesostigmata	Phytoseiidae	*Euseius nicholsi*	GBCH7599-13	JQ864533
Mesostigmata	Phytoseiidae	*Euseius nicholsi*	GBCH7600-13	JQ864532
Mesostigmata	Phytoseiidae	*Euseius nicholsi*	GBCH7601-13	JQ864531
Mesostigmata	Phytoseiidae	*Euseius nicholsi*	GBCH7602-13	JQ864530
Mesostigmata	Phytoseiidae	*Metaseiulus occidentalis*	GBCH1879-07	NC_009093
Mesostigmata	Phytoseiidae	*Metaseiulus occidentalis*	GBCH3756-09	EF221760
Mesostigmata	Phytoseiidae	*Phytoseiulus persimilis*	GACAC141-12	FM210191
Mesostigmata	Phytoseiidae	*Phytoseiulus persimilis*	GACAC142-12	FM210192
Mesostigmata	Phytoseiidae	*Phytoseiulus persimilis*	GACAC143-12	FM210193
Mesostigmata	Phytoseiidae	*Phytoseiulus persimilis*	GBCH4472-10	NC_014049
Mesostigmata	Phytoseiidae	*Phytoseiulus persimilis*	GBCH5032-10	GQ222414
Mesostigmata	Phytoseiidae	*Phytoseius aleuritius*	GBCH11783-13	JX080307
Mesostigmata	Phytoseiidae	*Phytoseius aleuritius*	GBCH11784-13	JX080306
Mesostigmata	Phytoseiidae	*Phytoseius minutus*	GBCH11781-13	JX080309
Mesostigmata	Phytoseiidae	*Phytoseius minutus*	GBCH11782-13	JX080308
Mesostigmata	Phytoseiidae	*Typhlodromips asiaticus*	GBCH11771-13	JX080319
Mesostigmata	Phytoseiidae	*Typhlodromips asiaticus*	GBCH11772-13	JX080318
Mesostigmata	Phytoseiidae	*Typhlodromips asiaticus*	GBCH11773-13	JX080317
Mesostigmata	Phytoseiidae	*Typhlodromips okinawanus*	GBCH11766-13	JX080324
Mesostigmata	Phytoseiidae	*Typhlodromips rademacheri*	GBCH11774-13	JX080316
Mesostigmata	Phytoseiidae	*Typhlodromips rademacheri*	GBCH11775-13	JX080315
Mesostigmata	Phytoseiidae	*Typhlodromips theae*	GBCH11767-13	JX080323
Mesostigmata	Phytoseiidae	*Typhlodromips theae*	GBCH11768-13	JX080322
Mesostigmata	Phytoseiidae	*Typhlodromips theae*	GBCH11769-13	JX080321
Mesostigmata	Phytoseiidae	*Typhlodromips theae*	GBCH11770-13	JX080320
Mesostigmata	Phytoseiidae	*Typhlodromus*	CNGII078-13	KM839839
Mesostigmata	Phytoseiidae	*Typhlodromus*	CNGIK137-13	KM831280
Mesostigmata	Phytoseiidae	*Typhlodromus*	CNGIK139-13	KM824591
Mesostigmata	Phytoseiidae	*Typhlodromus exhilaratus*	GBCH5484-11	JF279183
Mesostigmata	Phytoseiidae	*Typhlodromus exhilaratus*	GBCH5485-11	JF279182
Mesostigmata	Phytoseiidae	*Typhlodromus pyri*	GACAC140-12	FM210180
Mesostigmata	Phytoseiidae	*Typhlodromus pyri*	GBCH5486-11	JF279181
Mesostigmata	Phytoseiidae	*Typhlodromus pyri*	GBCH5487-11	JF279180
Mesostigmata	Phytoseiidae	*Typhlodromus pyri*	GBCH5488-11	JF279179
Mesostigmata	Phytoseiidae	*Typhlodromus pyri*	GBCH5489-11	JF279178
Mesostigmata	Phytoseiidae	*Typhlodromus pyri*	GBCH5490-11	JF279177
Mesostigmata	Phytoseiidae	*Typhlodromus pyri*	GBCH5491-11	JF279176
Mesostigmata	Phytoseiidae	*Typhlodromus pyri*	GBCH5492-11	JF279175
Mesostigmata	Phytoseiidae	*Typhlodromus pyri*	GBCH5493-11	JF279174
Mesostigmata	Phytoseiidae	*Typhlodromus pyri*	GBCH5494-11	JF279173
Mesostigmata	Phytoseiidae	*Typhlodromus pyri*	GBCH5495-11	JF279172
Mesostigmata	Phytoseiidae	*Typhlodromus pyri*	GBCH5496-11	JF279171
Mesostigmata	Phytoseiidae	*Typhlodromus pyri*	GBCH5497-11	JF279170
Mesostigmata	Phytoseiidae	*Typhlodromus pyri*	GBCH5498-11	JF279169
Mesostigmata	Phytoseiidae	*Typhlodromus pyri*	GBCH5499-11	JF279168
Mesostigmata	Phytoseiidae	*Typhlodromus pyri*	GBCH5500-11	JF279167
Mesostigmata	Phytoseiidae	*Typhlodromus pyri*	GBCH5501-11	JF279166
Mesostigmata	Phytoseiidae	*Typhlodromus pyri*	GBCH5502-11	JF279165
Mesostigmata	Phytoseiidae	*Typhlodromus pyri*	GBCH5503-11	JF279164
Mesostigmata	Phytoseiidae	*Typhlodromus pyri*	GBCH5504-11	JF279163
Mesostigmata	Phytoseiidae	*Typhlodromus pyri*	GBCH5505-11	JF279162
Mesostigmata	Phytoseiidae	*Typhlodromus pyri*	GBCH5506-11	JF279161

Mesostigmata	Phytoseiidae	*Typhlodromus pyri*	GBCH5507-11	JF279160
Mesostigmata	Phytoseiidae	*Typhlodromus pyri*	GBCH5508-11	JF279159
Mesostigmata	Phytoseiidae	*Typhlodromus pyri*	GBCH5509-11	JF279158
Mesostigmata	Phytoseiidae	*Typhlodromus pyri*	GBCH5510-11	JF279157
Mesostigmata	Phytoseiidae	*Typhlodromus pyri*	GBCH5511-11	JF279156
Mesostigmata	Phytoseiidae		CHACA988-10	HM405850
Mesostigmata	Phytoseiidae		CHACB1040-10	HM907171
Mesostigmata	Phytoseiidae		CHACB1168-10	HM907247
Mesostigmata	Phytoseiidae		CHACB1194-10	HM907269
Mesostigmata	Phytoseiidae		CHACB147-10	HQ558430
Mesostigmata	Phytoseiidae		CHACB677-10	JX835962
Mesostigmata	Phytoseiidae		CHACB838-10	HQ941509
Mesostigmata	Phytoseiidae		CHACC027-10	HM907428
Mesostigmata	Phytoseiidae		CHACC120-10	JX837899
Mesostigmata	Phytoseiidae		CNBPC226-12	KM834954
Mesostigmata	Phytoseiidae		CNEIE1933-12	KM831976
Mesostigmata	Phytoseiidae		CNEIE1934-12	KM828815
Mesostigmata	Phytoseiidae		CNEIE1937-12	KM836834
Mesostigmata	Phytoseiidae		CNGBE1027-14	KR069519
Mesostigmata	Phytoseiidae		CNGBJ2274-14	KR070597
Mesostigmata	Phytoseiidae		CNGBK1848-14	KR070368
Mesostigmata	Phytoseiidae		CNGIA130-12	KM831748
Mesostigmata	Phytoseiidae		CNGIA134-12	KM835603
Mesostigmata	Phytoseiidae		CNGIA137-12	KM824106
Mesostigmata	Phytoseiidae		CNGIB566-12	KM824490
Mesostigmata	Phytoseiidae		CNGIB569-12	KM837385
Mesostigmata	Phytoseiidae		CNGIB572-12	KM838860
Mesostigmata	Phytoseiidae		CNGIB573-12	KM832019
Mesostigmata	Phytoseiidae		CNGIE399-12	KM826359
Mesostigmata	Phytoseiidae		CNGIM418-13	KM836784
Mesostigmata	Phytoseiidae		CNGLB019-13	KM834020
Mesostigmata	Phytoseiidae		CNGLD033-13	KM827772
Mesostigmata	Phytoseiidae		CNGLE038-13	KM839095
Mesostigmata	Phytoseiidae		CNGLF143-13	KM838074
Mesostigmata	Phytoseiidae		CNGLF2645-13	KM831322
Mesostigmata	Phytoseiidae		CNJAA872-12	KM839318
Mesostigmata	Phytoseiidae		CNJAD2292-12	KM831494
Mesostigmata	Phytoseiidae		CNJAF2018-12	KM827076
Mesostigmata	Phytoseiidae		CNLMR1763-14	KR069685
Mesostigmata	Phytoseiidae		CNLMR1776-14	KR070277
Mesostigmata	Phytoseiidae		CNPAB274-13	KM830580
Mesostigmata	Phytoseiidae		CNPEO1317-14	KR069840
Mesostigmata	Phytoseiidae		CNPPB2354-12	KJ086108
Mesostigmata	Phytoseiidae		CNPPC1933-12	KJ083889
Mesostigmata	Phytoseiidae		CNPPD2658-12	KJ090645
Mesostigmata	Phytoseiidae		CNPPF583-12	KJ163438
Mesostigmata	Phytoseiidae		CNPPI1273-12	KJ444719
Mesostigmata	Phytoseiidae		CNRMA1070-12	KM824728
Mesostigmata	Phytoseiidae		CNRMC1662-12	KM839189
Mesostigmata	Phytoseiidae		CNRMC1687-12	KM828325
Mesostigmata	Phytoseiidae		CNRME4713-12	KM832161
Mesostigmata	Phytoseiidae		CNRME4745-12	KM833259
Mesostigmata	Phytoseiidae		CNRME4763-12	KM825865

Mesostigmata	Phytoseiidae	CNRMF3271-12	KM838329
Mesostigmata	Phytoseiidae	CNWLN304-13	KM827913
Mesostigmata	Phytoseiidae	CNWLN339-13	KM830392
Mesostigmata	Phytoseiidae	CNWLN363-13	KM838381
Mesostigmata	Phytoseiidae	CNWLN365-13	KM833146
Mesostigmata	Phytoseiidae	CNWLN381-13	KM838735
Mesostigmata	Phytoseiidae	CNWLN385-13	KM835327
Mesostigmata	Phytoseiidae	CNWLN387-13	KM824010
Mesostigmata	Phytoseiidae	EAWGA488-13	KR070278
Mesostigmata	Phytoseiidae	JSAUG1866-12	KP979354
Mesostigmata	Phytoseiidae	JSAUG1868-12	KP979278
Mesostigmata	Phytoseiidae	JSOIE067-12	KP979337
Mesostigmata	Phytoseiidae	JSOIE094-12	KR069987
Mesostigmata	Phytoseiidae	JSOIE099-12	KP979347
Mesostigmata	Phytoseiidae	MIONB149-10	KP979155
Mesostigmata	Phytoseiidae	MYMCA090-11	JX835107
Mesostigmata	Phytoseiidae	MYMCA093-11	JX837928
Mesostigmata	Phytoseiidae	MYMCA1398-11	JX833744
Mesostigmata	Phytoseiidae	MYMCA1411-11	JX835214
Mesostigmata	Phytoseiidae	MYMCA1412-11	JX836346
Mesostigmata	Phytoseiidae	MYMCA316-11	JX837810
Mesostigmata	Phytoseiidae	MYMCA391-11	JX835204
Mesostigmata	Phytoseiidae	MYMCA457-11	JX833761
Mesostigmata	Phytoseiidae	MYMCA458-11	JX836662
Mesostigmata	Phytoseiidae	MYMCA518-11	JX836396
Mesostigmata	Phytoseiidae	MYMCA521-11	JX838046
Mesostigmata	Phytoseiidae	MYMCA570-11	JX837883
Mesostigmata	Phytoseiidae	MYMCA631-11	JX835565
Mesostigmata	Phytoseiidae	MYMCA632-11	JX834229
Mesostigmata	Phytoseiidae	MYMCA633-11	JX835810
Mesostigmata	Phytoseiidae	MYMCA634-11	JX835500
Mesostigmata	Phytoseiidae	MYMCA658-11	JX836584
Mesostigmata	Phytoseiidae	MYMCA659-11	JX836681
Mesostigmata	Phytoseiidae	MYMCA743-11	JX836072
Mesostigmata	Phytoseiidae	MYMCA963-11	JX836880
Mesostigmata	Phytoseiidae	MYMCA997-11	JX835797
Mesostigmata	Phytoseiidae	MYMCA998-11	JX838192
Mesostigmata	Phytoseiidae	MYMCB085-11	JX834633
Mesostigmata	Phytoseiidae	MYMCB267-11	JX838098
Mesostigmata	Phytoseiidae	MYMCB661-11	JX838279
Mesostigmata	Phytoseiidae	MYMCB932-11	JX836144
Mesostigmata	Phytoseiidae	MYMCB933-11	JX834231
Mesostigmata	Phytoseiidae	MYMCC286-11	JX834532
Mesostigmata	Phytoseiidae	MYMCC330-11	JX837646
Mesostigmata	Phytoseiidae	MYMCC474-11	JX838071
Mesostigmata	Phytoseiidae	MYMCC497-11	JX835663
Mesostigmata	Phytoseiidae	MYMCC624-11	JX835253
Mesostigmata	Phytoseiidae	MYMCC662-11	JX835047
Mesostigmata	Phytoseiidae	MYMCC664-11	JX838277
Mesostigmata	Phytoseiidae	MYMCC852-11	JX835998
Mesostigmata	Phytoseiidae	MYMCC853-11	JX836620
Mesostigmata	Phytoseiidae	MYMCC926-11	JX838573
Mesostigmata	Phytoseiidae	MYMCD051-11	JX834118

Mesostigmata	Phytoseiidae	MYMCE001-12	JX834623
Mesostigmata	Phytoseiidae	MYMCE002-12	JX838508
Mesostigmata	Phytoseiidae	MYMCE003-12	JX838287
Mesostigmata	Phytoseiidae	MYMCE004-12	JX835405
Mesostigmata	Phytoseiidae	MYMCE006-12	JX834439
Mesostigmata	Phytoseiidae	MYMCE081-12	JX835458
Mesostigmata	Phytoseiidae	MYMCE105-12	JX836588
Mesostigmata	Phytoseiidae	MYMCE106-12	JX836062
Mesostigmata	Phytoseiidae	MYMCE140-12	JX837445
Mesostigmata	Phytoseiidae	MYMCE176-12	JX837446
Mesostigmata	Phytoseiidae	MYMCE177-12	JX834284
Mesostigmata	Phytoseiidae	MYMCE244-12	JX834396
Mesostigmata	Phytoseiidae	MYMCE322-12	JX836575
Mesostigmata	Phytoseiidae	MYMCE393-12	JX836193
Mesostigmata	Phytoseiidae	MYMCE394-12	JX834061
Mesostigmata	Phytoseiidae	MYMCE395-12	JX837379
Mesostigmata	Phytoseiidae	MYMCE396-12	JX838123
Mesostigmata	Phytoseiidae	MYMCE400-12	JX836304
Mesostigmata	Phytoseiidae	MYMCE615-12	JX834009
Mesostigmata	Phytoseiidae	MYMCE616-12	JX837679
Mesostigmata	Phytoseiidae	MYMCE617-12	JX836084
Mesostigmata	Phytoseiidae	MYMCE763-12	JX836286
Mesostigmata	Phytoseiidae	MYMCE840-12	JX838341
Mesostigmata	Phytoseiidae	MYMCF081-12	JX836741
Mesostigmata	Phytoseiidae	MYMCF147-12	JX835524
Mesostigmata	Phytoseiidae	MYMCF192-12	JX834548
Mesostigmata	Phytoseiidae	MYMCF196-12	JX835691
Mesostigmata	Phytoseiidae	MYMCF237-12	JX837091
Mesostigmata	Phytoseiidae	MYMCF410-12	JX835579
Mesostigmata	Phytoseiidae	MYMCF413-12	JX836180
Mesostigmata	Phytoseiidae	MYMCF414-12	JX835723
Mesostigmata	Phytoseiidae	MYMCF438-12	JX837162
Mesostigmata	Phytoseiidae	MYMCF483-12	JX835800
Mesostigmata	Phytoseiidae	MYMCF484-12	JX837219
Mesostigmata	Phytoseiidae	MYMCF485-12	JX836325
Mesostigmata	Phytoseiidae	MYMCF599-12	JX838127
Mesostigmata	Phytoseiidae	MYMCF697-12	JX838336
Mesostigmata	Phytoseiidae	MYMCF859-12	JX835040
Mesostigmata	Phytoseiidae	MYMCF860-12	JX836300
Mesostigmata	Phytoseiidae	MYMCG206-12	JX838668
Mesostigmata	Phytoseiidae	MYMCG208-12	JX835878
Mesostigmata	Phytoseiidae	MYMCG253-12	JX838272
Mesostigmata	Phytoseiidae	MYMCG278-12	JX837904
Mesostigmata	Phytoseiidae	MYMCG305-12	JX836458
Mesostigmata	Phytoseiidae	MYMCG306-12	JX838081
Mesostigmata	Phytoseiidae	MYMCG307-12	JX836879
Mesostigmata	Phytoseiidae	MYMCG344-12	JX837648
Mesostigmata	Phytoseiidae	MYMCG345-12	JX836008
Mesostigmata	Phytoseiidae	MYMCG430-12	JX838073
Mesostigmata	Phytoseiidae	MYMCG431-12	JX836074
Mesostigmata	Phytoseiidae	MYMCG432-12	JX836904
Mesostigmata	Phytoseiidae	MYMCG447-12	JX836656
Mesostigmata	Phytoseiidae	MYMCG510-12	JX835547

Mesostigmata	Phytoseiidae	MYMCG590-12	JX835332
Mesostigmata	Phytoseiidae	MYMCG591-12	JX834608
Mesostigmata	Phytoseiidae	MYMCG616-12	JX838438
Mesostigmata	Phytoseiidae	MYMCG650-12	JX835241
Mesostigmata	Phytoseiidae	MYTMC115-09	KR069715
Mesostigmata	Phytoseiidae	SMTPB1063-13	KP979243
Mesostigmata	Phytoseiidae	SMTPB1064-13	KR069739
Mesostigmata	Phytoseiidae	SMTPB12684-13	KP979122
Mesostigmata	Phytoseiidae	SMTPB12688-13	KP979277
Mesostigmata	Phytoseiidae	SMTPB12837-13	KP979166
Mesostigmata	Phytoseiidae	SMTPB12844-13	KR069447
Mesostigmata	Phytoseiidae	SMTPB14827-13	KR070614
Mesostigmata	Phytoseiidae	SMTPB20520-13	KP979298
Mesostigmata	Phytoseiidae	SMTPB20631-13	KR070230
Mesostigmata	Phytoseiidae	SMTPB22979-13	KP979232
Mesostigmata	Phytoseiidae	SMTPB2349-13	KP979311
Mesostigmata	Phytoseiidae	SMTPB9976-13	KR069941
Mesostigmata	Phytoseiidae	SSBAB123-12	KM839693
Mesostigmata	Phytoseiidae	SSBAD3066-12	KM827877
Mesostigmata	Phytoseiidae	SSBAD3074-12	KM837202
Mesostigmata	Phytoseiidae	SSBAD4047-12	KM829999
Mesostigmata	Phytoseiidae	SSJAE3134-13	KM833860
Mesostigmata	Phytoseiidae	SSPAA7766-13	KM838935
Mesostigmata	Phytoseiidae	SSPAA8154-13	KM833998
Mesostigmata	Phytoseiidae	SSPAB3991-13	KM831980
Mesostigmata	Phytoseiidae	SSPAB4006-13	KM836652
Mesostigmata	Phytoseiidae	SSPAB4026-13	KM829125
Mesostigmata	Phytoseiidae	SSPAC10865-13	KM836837
Mesostigmata	Phytoseiidae	SSPAC6737-13	KM834087
Mesostigmata	Phytoseiidae	SSPAC6762-13	KM830564
Mesostigmata	Phytoseiidae	SSPAC7837-13	KM830500
Mesostigmata	Phytoseiidae	SSPAC7874-13	KM836991
Mesostigmata	Phytoseiidae	SSWEE026-13	KM826667
Mesostigmata	Phytoseiidae	SSWEE122-13	KM831459
Mesostigmata	Phytoseiidae	SSWLB5366-13	KM837593
Mesostigmata	Phytoseiidae	SSWLB5367-13	KM828268
Mesostigmata	Phytoseiidae	SSWLD8209-13	KM840635
Mesostigmata	Phytoseiidae	SSWLD8239-13	KM840495
Mesostigmata	Phytoseiidae	SSWLD8247-13	KM839432
Mesostigmata	Phytoseiidae	SSWLD8287-13	KM827311
Mesostigmata	Phytoseiidae	SSWLD8342-13	KM837063
Mesostigmata	Phytoseiidae	SSWLE3964-13	KM827843
Mesostigmata	Phytoseiidae	SSWLF2217-13	KM836661
Mesostigmata	Phytoseiidae	SSWLF2976-13	KM829202
Mesostigmata	Phytoseiidae	SSWLF3055-13	KM839218
Mesostigmata	Phytoseiidae	SSWLF3059-13	KM835535
Mesostigmata	Phytoseiidae	SSWLF3075-13	KM831588
Mesostigmata	Phytoseiidae	SSWLF3117-13	KM827502
Mesostigmata	Phytoseiidae	SSWLF3150-13	KM836389
Mesostigmata	Phytoseiidae	SSWLF3158-13	KM833687
Mesostigmata	Phytoseiidae	SSWLF3938-13	KM831082
Mesostigmata	Sejidae	CNJAC1110-12	KM827198
Mesostigmata	Sejidae	CNJAC1122-12	KM839198

Mesostigmata	Sejidae		SSEIB4205-13	KM832249
Mesostigmata	Sejidae		SSEIB4208-13	KM832942
Mesostigmata	Sejidae		SSEIB4209-13	KM825826
Mesostigmata	Sejidae		SSEIB4211-13	KM840091
Mesostigmata	Sejidae		SSEIB4214-13	KM828038
Mesostigmata	Sejidae		SSEIB4215-13	KM838182
Mesostigmata	Sejidae		SSEIB4216-13	KM832359
Mesostigmata	Sejidae		SSEIB4217-13	KM832441
Mesostigmata	Sejidae		SSEIB4219-13	KM838595
Mesostigmata	Sejidae		SSEIB4226-13	KM827991
Mesostigmata	Sejidae		SSEIB4227-13	KM839566
Mesostigmata	Sejidae		SSEIB4229-13	KM827363
Mesostigmata	Sejidae		SSEIB4237-13	KM833329
Mesostigmata	Sejidae		SSEIB4246-13	KM839742
Mesostigmata	Trachytidae	*Trachytes*	CHACB040-10	HQ558353
Mesostigmata	Trachytidae	*Trachytes*	CHACB068-10	HQ558376
Mesostigmata	Trachytidae	*Trachytes*	CHACB794-10	JX837441
Mesostigmata	Trachytidae	*Trachytes*	CHACB833-10	JX838469
Mesostigmata	Trachytidae	*Trachytes*	CHACC168-10	JX836492
Mesostigmata	Trachytidae	*Trachytes*	CHACC169-10	JX835369
Mesostigmata	Trachytidae	*Trachytes*	CHACC171-10	JX836554
Mesostigmata	Trachytidae	*Trachytes*	MITMH011-07	KR069458
Mesostigmata	Trachytidae	*Trachytes*	MYMCC661-11	JX837725
Mesostigmata	Trachytidae	*Trachytes*	MYMCE618-12	JX838194
Mesostigmata	Trachytidae	*Trachytes*	MYMCE837-12	JX838111
Mesostigmata	Trachytidae	*Trachytes*	MYMCE885-12	JX834020
Mesostigmata	Trachytidae	*Trachytes*	MYMCF588-12	JX836097
Mesostigmata	Trachytidae	*Trachytes*	MYMCF930-12	JX838207
Mesostigmata	Trachytidae	*Trachytes*	MYMCG065-12	JX838337
Mesostigmata	Trachytidae	*Trachytes*	MYMCG313-12	JX836332
Mesostigmata	Trachytidae	*Trachytes*	MYMCG372-12	JX836450
Mesostigmata	Trachytidae	*Trachytes*	MYTMC148-09	KR069917
Mesostigmata	Trachytidae	*Trachytes*	MYTMC188-09	KR070208
Mesostigmata	Trematuridae	*Nenteria eulaelaptis*	BIOAC205-11	JN992135
Mesostigmata	Trematuridae	*Nenteria moseri*	BIOAC195-11	JN992136
Mesostigmata	Trematuridae	*Trichouropoda idahoensis*	BIOAC211-11	JN992168
Mesostigmata	Trematuridae	*Trichouropoda idahoensis*	BIOAC212-11	JN992169
Mesostigmata	Trematuridae	*Trichouropoda polytricha*	BIOAC216-11	JN992193
Mesostigmata	Trematuridae	*Trichouropoda sp. 11*	BIOAC213-11	JN992197
Mesostigmata	Trematuridae	*Trichouropoda tegucigalpae*	BIOAC203-11	JN992201
Mesostigmata	Trematuridae		CHACA721-09	JX837668
Mesostigmata	Trematuridae		CHACB066-10	HQ558374
Mesostigmata	Trematuridae		CHACB1107-10	JX838160
Mesostigmata	Trematuridae		CNJAF1913-12	KM830660
Mesostigmata	Trematuridae		MYMCB140-11	JX833871
Mesostigmata	Trematuridae		MYMCB771-11	JX838163
Mesostigmata	Trematuridae		MYMCB772-11	JX836454
Mesostigmata	Trematuridae		MYMCB773-11	JX838526
Mesostigmata	Trematuridae		MYMCC044-11	JX835698
Mesostigmata	Trematuridae		MYMCC045-11	JX838410
Mesostigmata	Trematuridae		MYMCC243-11	JX836507
Mesostigmata	Trematuridae		MYMCE596-12	JX836330
Mesostigmata	Trematuridae		MYMCE666-12	JX834265

Mesostigmata	Trematuridae		MYMCE694-12	JX836069
Mesostigmata	Trematuridae		MYMCF179-12	JX834352
Mesostigmata	Trematuridae		MYMCF180-12	JX834695
Mesostigmata	Trematuridae		MYMCF181-12	JX836014
Mesostigmata	Trematuridae		MYMCF182-12	JX836659
Mesostigmata	Trematuridae		MYMCF675-12	JX835293
Mesostigmata	Trematuridae		MYMCG251-12	JX838468
Mesostigmata	Trematuridae		MYMCG366-12	JX836634
Mesostigmata	Trematuridae		MYMCG574-12	JX834521
Mesostigmata	Trematuridae		PHOCT901-11	KR070216
Mesostigmata	Trematuridae		PHOCT908-11	KR070098
Mesostigmata	Trematuridae		PHOCT911-11	KP979282
Mesostigmata	Trematuridae		SSEIC4168-13	KM828859
Mesostigmata	Urodinychidae	*Uroobovella sp. 1WK*	BIOAC005-11	JN992048
Mesostigmata	Urodinychidae	*Uroobovella sp. 1WK*	BIOAC009-11	JN992049
Mesostigmata	Urodinychidae	*Uroobovella sp. 1WK*	BIOAC010-11	JN992050
Mesostigmata	Urodinychidae	*Uroobovella sp. 1WK*	BIOAC012-11	JN992051
Mesostigmata	Urodinychidae	*Uroobovella sp. 1WK*	BIOAC013-11	JN992052
Mesostigmata	Urodinychidae	*Uroobovella sp. 1WK*	BIOAC015-11	JN992053
Mesostigmata	Urodinychidae	*Uroobovella sp. 1WK*	BIOAC025-11	JN992054
Mesostigmata	Urodinychidae	*Uroobovella sp. 1WK*	BIOAC028-11	JN992055
Mesostigmata	Urodinychidae	*Uroobovella sp. 1WK*	BIOAC032-11	JN992056
Mesostigmata	Urodinychidae	*Uroobovella sp. 1WK*	BIOAC037-11	JN992057
Mesostigmata	Urodinychidae	*Uroobovella sp. 1WK*	BIOAC038-11	JN992058
Mesostigmata	Urodinychidae	*Uroobovella sp. 1WK*	BIOAC039-11	JN992059
Mesostigmata	Urodinychidae	*Uroobovella sp. 1WK*	BIOAC040-11	JN992060
Mesostigmata	Urodinychidae	*Uroobovella sp. 1WK*	BIOAC044-11	JN992061
Mesostigmata	Urodinychidae	*Uroobovella sp. 1WK*	BIOAC046-11	JN992062
Mesostigmata	Urodinychidae	*Uroobovella sp. 1WK*	BIOAC053-11	JN992063
Mesostigmata	Urodinychidae	*Uroobovella sp. 1WK*	BIOAC054-11	JN992064
Mesostigmata	Urodinychidae	*Uroobovella sp. 1WK*	BIOAC055-11	JN992065
Mesostigmata	Urodinychidae	*Uroobovella sp. 1WK*	BIOAC060-11	JN992066
Mesostigmata	Urodinychidae	*Uroobovella sp. 1WK*	BIOAC061-11	JN992067
Mesostigmata	Urodinychidae	*Uroobovella sp. 1WK*	BIOAC069-11	JN992068
Mesostigmata	Urodinychidae	*Uroobovella sp. 1WK*	BIOAC078-11	JN992069
Mesostigmata	Urodinychidae	*Uroobovella sp. 1WK*	BIOAC081-11	JN992070
Mesostigmata	Urodinychidae	*Uroobovella sp. 1WK*	BIOAC084-11	JN992071
Mesostigmata	Urodinychidae	*Uroobovella sp. 1WK*	BIOAC085-11	JN992072
Mesostigmata	Urodinychidae	*Uroobovella sp. 1WK*	BIOAC086-11	JN992073
Mesostigmata	Urodinychidae	*Uroobovella sp. 1WK*	BIOAC264-11	JN992074
Mesostigmata	Urodinychidae	*Uroobovella sp. 1WK*	BIOAC266-11	JN992075
Mesostigmata	Urodinychidae	*Uroobovella sp. 2WK*	BIOAC002-11	JN992076
Mesostigmata	Urodinychidae	*Uroobovella sp. 2WK*	BIOAC004-11	JN992077
Mesostigmata	Urodinychidae	*Uroobovella sp. 2WK*	BIOAC007-11	JN992078
Mesostigmata	Urodinychidae	*Uroobovella sp. 2WK*	BIOAC016-11	JN992079
Mesostigmata	Urodinychidae	*Uroobovella sp. 2WK*	BIOAC021-11	JN992080
Mesostigmata	Urodinychidae	*Uroobovella sp. 2WK*	BIOAC024-11	JN992081
Mesostigmata	Urodinychidae	*Uroobovella sp. 2WK*	BIOAC027-11	JN992082
Mesostigmata	Urodinychidae	*Uroobovella sp. 2WK*	BIOAC029-11	JN992083
Mesostigmata	Urodinychidae	*Uroobovella sp. 2WK*	BIOAC030-11	JN992084
Mesostigmata	Urodinychidae	*Uroobovella sp. 2WK*	BIOAC033-11	JN992085
Mesostigmata	Urodinychidae	*Uroobovella sp. 2WK*	BIOAC035-11	JN992086
Mesostigmata	Urodinychidae	*Uroobovella sp. 2WK*	BIOAC036-11	JN992087

Mesostigmata	Urodinychidae	*Uroobovella sp. 2WK*	BIOAC041-11	JN992088
Mesostigmata	Urodinychidae	*Uroobovella sp. 2WK*	BIOAC042-11	JN992089
Mesostigmata	Urodinychidae	*Uroobovella sp. 2WK*	BIOAC043-11	JN992090
Mesostigmata	Urodinychidae	*Uroobovella sp. 2WK*	BIOAC047-11	JN992091
Mesostigmata	Urodinychidae	*Uroobovella sp. 2WK*	BIOAC062-11	JN992092
Mesostigmata	Urodinychidae	*Uroobovella sp. 2WK*	BIOAC077-11	JN992093
Mesostigmata	Urodinychidae	*Uroobovella sp. 2WK*	BIOAC079-11	JN992094
Mesostigmata	Urodinychidae	*Uroobovella sp. 2WK*	BIOAC080-11	JN992095
Mesostigmata	Urodinychidae	*Uroobovella sp. 2WK*	BIOAC263-11	JN992096
Mesostigmata	Urodinychidae	*Uroobovella sp. 2WK*	BIOAC268-11	JN992097
Mesostigmata	Urodinychidae	*Uroobovella sp. 3WK*	BIOAC001-11	JN992098
Mesostigmata	Urodinychidae	*Uroobovella sp. 3WK*	BIOAC006-11	JN992099
Mesostigmata	Urodinychidae	*Uroobovella sp. 3WK*	BIOAC018-11	JN992100
Mesostigmata	Urodinychidae	*Uroobovella sp. 3WK*	BIOAC019-11	JN992101
Mesostigmata	Urodinychidae	*Uroobovella sp. 3WK*	BIOAC265-11	JN992102
Mesostigmata	Urodinychidae	*Uroobovella sp. 4*	BIOAC267-11	JN992103
Mesostigmata	Urodinychidae	*Uroobovella vinicolora*	BIOAC192-11	JN992248
Mesostigmata	Urodinychidae	*Uroobovella vinicolora*	BIOAC242-11	JN992249
Mesostigmata	Urodinychidae	*Uroobovella vinicolora*	BIOAC260-11	JN992250
Mesostigmata	Varroidae	*Varroa destructor*	GBCH0292-06	AJ493124
Mesostigmata	Varroidae	*Varroa destructor*	GBCH0350-06	AJ784872
Mesostigmata	Varroidae	*Varroa destructor*	GBCH11401-13	JX970939
Mesostigmata	Varroidae	*Varroa destructor*	GBCH11402-13	JX970938
Mesostigmata	Varroidae	*Varroa destructor*	GBCH1678-06	NC_004454
Mesostigmata	Varroidae	*Varroa destructor*	GBCH5617-13	GQ379074
Mesostigmata	Varroidae	*Varroa destructor*	GBCH5618-13	GQ379073
Mesostigmata	Varroidae	*Varroa destructor*	GBCH5619-13	GQ379072
Mesostigmata	Varroidae	*Varroa destructor*	GBCH5620-13	GQ379071
Mesostigmata	Varroidae	*Varroa destructor*	GBCH5621-13	GQ379070
Mesostigmata	Varroidae	*Varroa destructor*	GBCH5622-13	GQ379069
Mesostigmata	Varroidae	*Varroa destructor*	GBCH5623-13	GQ379068
Mesostigmata	Varroidae	*Varroa destructor*	GBCH5624-13	GQ379067
Mesostigmata	Varroidae	*Varroa destructor*	GBCH5625-13	GQ379066
Mesostigmata	Varroidae	*Varroa destructor*	GBCH5626-13	GQ379065
Mesostigmata	Varroidae	*Varroa destructor*	GBCH5627-13	GQ379064
Mesostigmata	Varroidae	*Varroa destructor*	GBCH5628-13	GQ379063
Mesostigmata	Varroidae	*Varroa destructor*	GBCH5629-13	GQ379062
Mesostigmata	Varroidae	*Varroa destructor*	GBCH5630-13	GQ379061
Mesostigmata	Varroidae	*Varroa destructor*	GBCH5631-13	GQ379060
Mesostigmata	Varroidae	*Varroa destructor*	GBCH5632-13	GQ379059
Mesostigmata	Varroidae	*Varroa destructor*	GBCH5633-13	GQ379058
Mesostigmata	Varroidae	*Varroa destructor*	GBCH5634-13	GQ379057
Mesostigmata	Varroidae	*Varroa destructor*	GBCH5635-13	GQ379056
Mesostigmata	Veigaiidae		MYMCF612-12	JX837587
Mesostigmata	Veigaiidae		MYMCF718-12	JX838334
Mesostigmata	Veigaiidae		MYMCG096-12	JX833869
Mesostigmata	Zerconidae		CHACA289-08	JX837711
Mesostigmata	Zerconidae		CHACB015-10	HQ558336
Mesostigmata	Zerconidae		CHACB026-10	HQ558345
Mesostigmata	Zerconidae		CHACB065-10	HQ558373
Mesostigmata	Zerconidae		CHACB073-10	HQ558380
Mesostigmata	Zerconidae		CHACB074-10	HQ558381
Mesostigmata	Zerconidae		CHACB075-10	HQ558382

Mesostigmata	Zerconidae	CHACB076-10	HQ558383
Mesostigmata	Zerconidae	CHACB077-10	HQ558384
Mesostigmata	Zerconidae	CHACB1036-10	HM907169
Mesostigmata	Zerconidae	CHACB1037-10	HM907170
Mesostigmata	Zerconidae	CHACB1106-10	HM907203
Mesostigmata	Zerconidae	CHACB1108-10	JX835249
Mesostigmata	Zerconidae	CHACB1109-10	HM907204
Mesostigmata	Zerconidae	CHACB1110-10	HM907205
Mesostigmata	Zerconidae	CHACB1111-10	HM907206
Mesostigmata	Zerconidae	CHACB1112-10	JX836707
Mesostigmata	Zerconidae	CHACB1157-10	KR069406
Mesostigmata	Zerconidae	CHACB163-10	HQ558445
Mesostigmata	Zerconidae	CHACB411-10	HQ558571
Mesostigmata	Zerconidae	CHACB642-10	HQ558722
Mesostigmata	Zerconidae	CHACB904-10	HM907334
Mesostigmata	Zerconidae	CHACB905-10	HM907335
Mesostigmata	Zerconidae	CHACB906-10	HM907336
Mesostigmata	Zerconidae	CHACB908-10	HM907337
Mesostigmata	Zerconidae	CHACB909-10	HM907338
Mesostigmata	Zerconidae	CHACB910-10	HM907339
Mesostigmata	Zerconidae	CHACB911-10	HM907340
Mesostigmata	Zerconidae	CHACB912-10	HM907341
Mesostigmata	Zerconidae	CHACB927-10	JX836256
Mesostigmata	Zerconidae	CHACC127-10	JX834131
Mesostigmata	Zerconidae	CHACC167-10	JX833963
Mesostigmata	Zerconidae	CHACC172-10	JX834415
Mesostigmata	Zerconidae	MIONB067-10	HQ575083
Mesostigmata	Zerconidae	MIONB068-10	HQ575084
Mesostigmata	Zerconidae	MITMH009-07	KR070675
Mesostigmata	Zerconidae	MYMCA1088-11	JX837460
Mesostigmata	Zerconidae	MYMCA1089-11	JX836801
Mesostigmata	Zerconidae	MYMCA1124-11	JX834178
Mesostigmata	Zerconidae	MYMCA1175-11	JX838678
Mesostigmata	Zerconidae	MYMCA1218-11	JX834041
Mesostigmata	Zerconidae	MYMCA1323-11	JX835793
Mesostigmata	Zerconidae	MYMCA198-11	JX837122
Mesostigmata	Zerconidae	MYMCA199-11	JX835292
Mesostigmata	Zerconidae	MYMCA200-11	JX834678
Mesostigmata	Zerconidae	MYMCA201-11	JX834090
Mesostigmata	Zerconidae	MYMCA202-11	JX835880
Mesostigmata	Zerconidae	MYMCA203-11	JX838429
Mesostigmata	Zerconidae	MYMCA204-11	JX835034
Mesostigmata	Zerconidae	MYMCA369-11	JX833656
Mesostigmata	Zerconidae	MYMCA370-11	JX835184
Mesostigmata	Zerconidae	MYMCA371-11	JX836714
Mesostigmata	Zerconidae	MYMCA372-11	JX834852
Mesostigmata	Zerconidae	MYMCA396-11	JX836442
Mesostigmata	Zerconidae	MYMCA397-11	JX836536
Mesostigmata	Zerconidae	MYMCA574-11	JX834792
Mesostigmata	Zerconidae	MYMCA789-11	JX838711
Mesostigmata	Zerconidae	MYMCA804-11	JX835832
Mesostigmata	Zerconidae	MYMCA872-11	JX837838
Mesostigmata	Zerconidae	MYMCA901-11	JX837129

Mesostigmata	Zerconidae	MYMCA980-11	JX835576
Mesostigmata	Zerconidae	MYMCA995-11	JX837866
Mesostigmata	Zerconidae	MYMCA996-11	JX837130
Mesostigmata	Zerconidae	MYMCB105-11	JX838075
Mesostigmata	Zerconidae	MYMCB106-11	JX834594
Mesostigmata	Zerconidae	MYMCB139-11	JX838234
Mesostigmata	Zerconidae	MYMCB269-11	JX837576
Mesostigmata	Zerconidae	MYMCB531-11	JX835826
Mesostigmata	Zerconidae	MYMCB574-11	JX838105
Mesostigmata	Zerconidae	MYMCB752-11	JX835028
Mesostigmata	Zerconidae	MYMCB768-11	JX835001
Mesostigmata	Zerconidae	MYMCB811-11	JX834093
Mesostigmata	Zerconidae	MYMCB913-11	JX834835
Mesostigmata	Zerconidae	MYMCC046-11	JX834779
Mesostigmata	Zerconidae	MYMCC082-11	JX838264
Mesostigmata	Zerconidae	MYMCC265-11	JX836277
Mesostigmata	Zerconidae	MYMCC433-11	JX834557
Mesostigmata	Zerconidae	MYMCC434-11	JX838134
Mesostigmata	Zerconidae	MYMCC496-11	JX836777
Mesostigmata	Zerconidae	MYMCC504-11	JX835742
Mesostigmata	Zerconidae	MYMCC798-11	JX838066
Mesostigmata	Zerconidae	MYMCC894-11	JX838390
Mesostigmata	Zerconidae	MYMCC895-11	JX837323
Mesostigmata	Zerconidae	MYMCC939-11	JX838467
Mesostigmata	Zerconidae	MYMCC940-11	JX834076
Mesostigmata	Zerconidae	MYMCE087-12	JX834680
Mesostigmata	Zerconidae	MYMCE090-12	JX838101
Mesostigmata	Zerconidae	MYMCE162-12	JX834278
Mesostigmata	Zerconidae	MYMCE163-12	JX834073
Mesostigmata	Zerconidae	MYMCE164-12	JX836047
Mesostigmata	Zerconidae	MYMCE293-12	JX838784
Mesostigmata	Zerconidae	MYMCE619-12	JX837536
Mesostigmata	Zerconidae	MYMCE642-12	JX836763
Mesostigmata	Zerconidae	MYMCE697-12	JX833710
Mesostigmata	Zerconidae	MYMCE698-12	JX838273
Mesostigmata	Zerconidae	MYMCE761-12	JX836117
Mesostigmata	Zerconidae	MYMCE762-12	JX837496
Mesostigmata	Zerconidae	MYMCE799-12	JX834249
Mesostigmata	Zerconidae	MYMCE843-12	JX837081
Mesostigmata	Zerconidae	MYMCE941-12	JX836421
Mesostigmata	Zerconidae	MYMCE949-12	JX836596
Mesostigmata	Zerconidae	MYMCE950-12	JX835157
Mesostigmata	Zerconidae	MYMCF044-12	JX838518
Mesostigmata	Zerconidae	MYMCF045-12	JX834052
Mesostigmata	Zerconidae	MYMCF061-12	JX837804
Mesostigmata	Zerconidae	MYMCF062-12	JX834151
Mesostigmata	Zerconidae	MYMCF082-12	JX834165
Mesostigmata	Zerconidae	MYMCF107-12	JX834821
Mesostigmata	Zerconidae	MYMCF108-12	JX836135
Mesostigmata	Zerconidae	MYMCF352-12	JX836998
Mesostigmata	Zerconidae	MYMCF382-12	JX836016
Mesostigmata	Zerconidae	MYMCF560-12	JX833958
Mesostigmata	Zerconidae	MYMCF635-12	JX837083

Mesostigmata	Zerconidae		MYMCF636-12	JX838206
Mesostigmata	Zerconidae		MYMCF736-12	JX838286
Mesostigmata	Zerconidae		MYMCF737-12	JX838010
Mesostigmata	Zerconidae		MYMCF928-12	JX836085
Mesostigmata	Zerconidae		MYMCF929-12	JX835918
Mesostigmata	Zerconidae		MYMCG003-12	JX834766
Mesostigmata	Zerconidae		MYMCG064-12	JX836385
Mesostigmata	Zerconidae		MYMCG234-12	JX837163
Mesostigmata	Zerconidae		MYMCG250-12	JX837972
Mesostigmata	Zerconidae		MYMCG252-12	JX838093
Mesostigmata	Zerconidae		MYMCG373-12	JX834981
Mesostigmata	Zerconidae		MYMCG374-12	JX838785
Mesostigmata	Zerconidae		MYMCG458-12	JX834908
Mesostigmata	Zerconidae		MYMCG500-12	JX837846
Mesostigmata	Zerconidae		MYMCG564-12	JX833664
Mesostigmata	Zerconidae		MYMCG652-12	JX837221
Mesostigmata	Zerconidae		MYTMC065-09	GU680464
Mesostigmata	Zerconidae		MYTMC088-09	GU680457
Mesostigmata	Zerconidae		MYTMC121-09	GU680490
Mesostigmata	Zerconidae		SSJAA1810-13	KM834547
Mesostigmata	Zerconidae		SSJAC1508-13	KM828949
Mesostigmata	Zerconidae		SSWLA5259-13	KR070013
Mesostigmata	Zerconidae		SSWLA5339-13	KM835311
Mesostigmata	Zerconidae		SSWLC4141-13	KM839305
Opilioacarida	Opilioacaridae	*Neocarus*	MXBAC095-11	KP979332
Opiliones	Biantidae	*Metabiantes sp.*	GACAC637-12	JF786450
Opiliones	Ceratolasmatidae	*Acuclavella cf. quattuor SDSU OP*	GBCH12102-13	KF181732
Opiliones	Ceratolasmatidae	*Acuclavella cf. quattuor SDSU OP*	GBCH12103-13	KF181731
Opiliones	Ceratolasmatidae	*Acuclavella cf. quattuor SDSU OP*	GBCH12104-13	KF181730
Opiliones	Ceratolasmatidae	*Acuclavella cosmetoides*	GBCH4487-10	GQ870646
Opiliones	Ceratolasmatidae	*Acuclavella makah*	GBCH12096-13	KF181738
Opiliones	Ceratolasmatidae	*Acuclavella makah*	GBCH12097-13	KF181737
Opiliones	Ceratolasmatidae	*Acuclavella makah*	GBCH12098-13	KF181736
Opiliones	Ceratolasmatidae	*Acuclavella merickeli*	GBCH12099-13	KF181735
Opiliones	Ceratolasmatidae	*Acuclavella merickeli*	GBCH12100-13	KF181734
Opiliones	Ceratolasmatidae	*Acuclavella merickeli*	GBCH12101-13	KF181733
Opiliones	Ceratolasmatidae	*Acuclavella merickeli*	GBCH4489-10	GQ870644
Opiliones	Ceratolasmatidae	*Acuclavella quattuor*	GBCH12094-13	KF181740
Opiliones	Ceratolasmatidae	*Acuclavella quattuor*	GBCH12095-13	KF181739
Opiliones	Ceratolasmatidae	*Acuclavella quattuor*	GBCH12105-13	KF181729
Opiliones	Ceratolasmatidae	*Acuclavella quattuor*	GBCH4488-10	GQ870645
Opiliones	Ceratolasmatidae	*Acuclavella sheari*	GBCH12091-13	KF181743
Opiliones	Ceratolasmatidae	*Acuclavella sheari*	GBCH12092-13	KF181742
Opiliones	Ceratolasmatidae	*Acuclavella sheari*	GBCH12093-13	KF181741
Opiliones	Ceratolasmatidae	*Acuclavella sp. 1 MCH-2009*	GBCH4490-10	GQ870643
Opiliones	Ceratolasmatidae	*Acuclavella sp. 2 MCH-2009*	GBCH4486-10	GQ870647
Opiliones	Ceratolasmatidae	*Acuclavella sp. 3 MCH-2009*	GBCH4485-10	GQ870648
Opiliones	Epedanidae	*Tithaeus sp. DNA104062_1*	GACAC624-12	JF786437
Opiliones	Epedanidae	*Tithaeus sp. DNA104068*	GACAC626-12	JF786439
Opiliones	Gonyleptidae	*Acutisoma longipes*	GACAC628-12	JF786441
Opiliones	Icaleptidae	*Icaleptes sp.*	GACAC644-12	JF786457
Opiliones	Kimulidae	*Kimula goodnightiorum*	GACAC645-12	JF786458
Opiliones	Neogoveidae	*Brasilogovea sp. DNA101665*	GACAC617-12	JF786414

Opiliones	Neogoveidae	*Huitaca sp. DNA101671*	GACAC601-12	JF786398
Opiliones	Neogoveidae	*Huitaca sp. DNA101681*	GACAC600-12	JF786397
Opiliones	Neogoveidae	*Huitaca sp. DNA101683*	GACAC599-12	JF786396
Opiliones	Neogoveidae	*Huitaca sp. MCZDNA101407*	GBCH3699-09	DQ518129
Opiliones	Neogoveidae	*Metagovea sp. DNA101680*	GACAC603-12	JF786400
Opiliones	Neogoveidae	*Metagovea sp. DNA101685*	GACAC606-12	JF786403
Opiliones	Neogoveidae	*Metagovea sp. DNA102151m*	GACAC605-12	JF786402
Opiliones	Neogoveidae	*Metasiro americanus*	GACAC596-12	JF786393
Opiliones	Neogoveidae	*Metasiro americanus*	GBCH3689-09	DQ825645
Opiliones	Neogoveidae	*Neogovea sp. DNA104823*	GACAC608-12	JF786405
Opiliones	Neogoveidae	*Neogovea sp. DNA105825*	GACAC610-12	JF786407
Opiliones	Neogoveidae	*Neogovea sp. DNA105826*	GACAC611-12	JF786408
Opiliones	Neogoveidae	*Neogovea sp. MCZDNA101409*	GBCH1818-07	DQ825646
Opiliones	Neogoveidae	*Neogovea virginie*	GACAC609-12	JF786406
Opiliones	Neogoveidae	*Paragovia gabonica*	GACAC614-12	JF786411
Opiliones	Neogoveidae	*Paragovia sironoides*	GBCH1695-07	DQ518131
Opiliones	Neogoveidae	*Paragovia sironoides*	GBCH1814-07	DQ825650
Opiliones	Neogoveidae	*Paragovia sironoides*	GBCH1815-07	DQ825649
Opiliones	Neogoveidae	*Paragovia sp. DNA104615*	GACAC613-12	JF786410
Opiliones	Neogoveidae	*Paragovia sp. DNA104618*	GACAC615-12	JF786412
Opiliones	Neogoveidae	*Paragovia sp. DNA104619*	GACAC612-12	JF786409
Opiliones	Neogoveidae	*Paragovia sp. DNA105671*	GACAC616-12	JF786413
Opiliones	Neogoveidae	*Paragovia sp. SLB-2006*	GBCH1816-07	DQ825648
Opiliones	Pettalidae	*Aoraki calcarobtusa*	GBCH3170-08	EU673667
Opiliones	Pettalidae	*Chileogovea oedipus*	GBCH3176-08	EU673661
Opiliones	Pettalidae	*Chileogovea sp. MCZDNA101490*	GBCH1829-07	DQ825635
Opiliones	Pettalidae	*Chileogovea sp. SLB-2008*	GBCH3175-08	EU673662
Opiliones	Pettalidae	*Karripurcellia harveyi*	GBCH1719-07	DQ518106
Opiliones	Pettalidae	*Karripurcellia harveyi*	GBCH3708-09	DQ518107
Opiliones	Pettalidae	*Neopurcellia florensis*	GBCH1712-07	DQ518113
Opiliones	Pettalidae	*Neopurcellia florensis*	GBCH2558-08	DQ992325
Opiliones	Pettalidae	*Neopurcellia forsteri*	GBCH3705-09	DQ518110
Opiliones	Pettalidae	*Neopurcellia minutissima*	GBCH1711-07	DQ518114
Opiliones	Pettalidae	*Neopurcellia minutissima*	GBCH2555-08	DQ992328
Opiliones	Pettalidae	*Neopurcellia minutissima*	GBCH2556-08	DQ992327
Opiliones	Pettalidae	*Neopurcellia minutissima*	GBCH2557-08	DQ992326
Opiliones	Pettalidae	*Neopurcellia salmoni*	GBCH1826-07	DQ825638
Opiliones	Pettalidae	*Neopurcellia salmoni*	GBCH2559-08	DQ992323
Opiliones	Pettalidae	*Neopurcellia salmoni*	GBCH3706-09	DQ518109
Opiliones	Pettalidae	*Parapurcellia monticola*	GBCH1727-07	DQ518098
Opiliones	Pettalidae	*Parapurcellia monticola*	GBCH3174-08	EU673663
Opiliones	Pettalidae	*Parapurcellia silvicola*	GBCH0979-06	AY639582
Opiliones	Pettalidae	*Parapurcellia silvicola*	GBCH3173-08	EU673664
Opiliones	Pettalidae	*Pettalus brevicauda*	GBCH1724-07	DQ518101
Opiliones	Pettalidae	*Pettalus sp. 259L*	GBCH1828-07	DQ825636
Opiliones	Pettalidae	*Pettalus sp. 259s*	GBCH1725-07	DQ518100
Opiliones	Pettalidae	*Pettalus sp. 266L*	GBCH1723-07	DQ518102
Opiliones	Pettalidae	*Pettalus sp. 266s*	GBCH1722-07	DQ518103
Opiliones	Pettalidae	*Pettalus sp. 270*	GBCH1721-07	DQ518104
Opiliones	Pettalidae	*Pettalus sp. 274*	GBCH1720-07	DQ518105
Opiliones	Pettalidae	*Pettalus sp. MCZDNA101223*	GBCH3171-08	EU673666
Opiliones	Pettalidae	*Pettalus sp. Sinharaja*	GBCH1827-07	DQ825637
Opiliones	Pettalidae	*Purcellia illustrans*	GBCH1726-07	DQ518099

Opiliones	Pettalidae	*Purcellia illustrans*	GBCH3172-08	EU673665
Opiliones	Pettalidae	*Rakaia antipodiana*	GBCH1710-07	DQ518115
Opiliones	Pettalidae	*Rakaia antipodiana*	GBCH2553-08	DQ992330
Opiliones	Pettalidae	*Rakaia antipodiana*	GBCH2554-08	DQ992329
Opiliones	Pettalidae	*Rakaia arcticosa*	GBCH3704-09	DQ518111
Opiliones	Pettalidae	*Rakaia calcarobtusa*	GBCH1704-07	DQ518121
Opiliones	Pettalidae	*Rakaia calcarobtusa*	GBCH2567-08	DQ992315
Opiliones	Pettalidae	*Rakaia crypta*	GBCH1705-07	DQ518120
Opiliones	Pettalidae	*Rakaia daviesae*	GBCH1713-07	DQ518112
Opiliones	Pettalidae	*Rakaia denticulata*	GBCH2570-08	DQ992312
Opiliones	Pettalidae	*Rakaia denticulata*	GBCH2571-08	DQ992311
Opiliones	Pettalidae	*Rakaia denticulata*	GBCH2572-08	DQ992310
Opiliones	Pettalidae	*Rakaia denticulata*	GBCH2573-08	DQ992309
Opiliones	Pettalidae	*Rakaia denticulata*	GBCH2574-08	DQ992308
Opiliones	Pettalidae	*Rakaia denticulata*	GBCH2575-08	DQ992307
Opiliones	Pettalidae	*Rakaia denticulata*	GBCH2576-08	DQ992306
Opiliones	Pettalidae	*Rakaia denticulata*	GBCH2577-08	DQ992305
Opiliones	Pettalidae	*Rakaia denticulata*	GBCH2578-08	DQ992304
Opiliones	Pettalidae	*Rakaia denticulata*	GBCH2579-08	DQ992303
Opiliones	Pettalidae	*Rakaia denticulata*	GBCH2580-08	DQ992302
Opiliones	Pettalidae	*Rakaia denticulata*	GBCH2581-08	DQ992301
Opiliones	Pettalidae	*Rakaia denticulata*	GBCH2582-08	DQ992300
Opiliones	Pettalidae	*Rakaia denticulata*	GBCH2583-08	DQ992299
Opiliones	Pettalidae	*Rakaia denticulata*	GBCH2584-08	DQ992298
Opiliones	Pettalidae	*Rakaia denticulata*	GBCH2585-08	DQ992297
Opiliones	Pettalidae	*Rakaia denticulata*	GBCH2586-08	DQ992296
Opiliones	Pettalidae	*Rakaia denticulata*	GBCH2587-08	DQ992295
Opiliones	Pettalidae	*Rakaia denticulata*	GBCH2588-08	DQ992294
Opiliones	Pettalidae	*Rakaia denticulata*	GBCH2589-08	DQ992293
Opiliones	Pettalidae	*Rakaia denticulata*	GBCH2590-08	DQ992292
Opiliones	Pettalidae	*Rakaia denticulata*	GBCH2591-08	DQ992291
Opiliones	Pettalidae	*Rakaia denticulata*	GBCH2592-08	DQ992290
Opiliones	Pettalidae	*Rakaia denticulata*	GBCH2593-08	DQ992289
Opiliones	Pettalidae	*Rakaia denticulata*	GBCH2594-08	DQ992288
Opiliones	Pettalidae	*Rakaia denticulata*	GBCH2595-08	DQ992287
Opiliones	Pettalidae	*Rakaia denticulata*	GBCH2596-08	DQ992286
Opiliones	Pettalidae	*Rakaia denticulata*	GBCH2597-08	DQ992285
Opiliones	Pettalidae	*Rakaia denticulata*	GBCH2598-08	DQ992284
Opiliones	Pettalidae	*Rakaia denticulata*	GBCH2599-08	DQ992283
Opiliones	Pettalidae	*Rakaia denticulata*	GBCH2600-08	DQ992282
Opiliones	Pettalidae	*Rakaia denticulata*	GBCH2601-08	DQ992281
Opiliones	Pettalidae	*Rakaia denticulata*	GBCH2602-08	DQ992280
Opiliones	Pettalidae	*Rakaia denticulata*	GBCH2603-08	DQ992279
Opiliones	Pettalidae	*Rakaia denticulata*	GBCH2604-08	DQ992278
Opiliones	Pettalidae	*Rakaia denticulata*	GBCH2605-08	DQ992277
Opiliones	Pettalidae	*Rakaia denticulata*	GBCH2606-08	DQ992276
Opiliones	Pettalidae	*Rakaia denticulata*	GBCH2607-08	DQ992275
Opiliones	Pettalidae	*Rakaia denticulata*	GBCH2608-08	DQ992274
Opiliones	Pettalidae	*Rakaia denticulata*	GBCH2609-08	DQ992273
Opiliones	Pettalidae	*Rakaia denticulata*	GBCH2610-08	DQ992272
Opiliones	Pettalidae	*Rakaia denticulata*	GBCH2611-08	DQ992271
Opiliones	Pettalidae	*Rakaia denticulata*	GBCH2612-08	DQ992270
Opiliones	Pettalidae	*Rakaia denticulata*	GBCH2613-08	DQ992269

Opiliones	Pettalidae	*Rakaia denticulata*	GBCH2614-08	DQ992268
Opiliones	Pettalidae	*Rakaia denticulata*	GBCH2615-08	DQ992267
Opiliones	Pettalidae	*Rakaia denticulata*	GBCH2616-08	DQ992266
Opiliones	Pettalidae	*Rakaia denticulata*	GBCH2617-08	DQ992265
Opiliones	Pettalidae	*Rakaia denticulata*	GBCH2627-08	DQ992255
Opiliones	Pettalidae	*Rakaia denticulata*	GBCH2628-08	DQ992254
Opiliones	Pettalidae	*Rakaia denticulata*	GBCH2629-08	DQ992253
Opiliones	Pettalidae	*Rakaia denticulata*	GBCH2630-08	DQ992252
Opiliones	Pettalidae	*Rakaia denticulata*	GBCH2631-08	DQ992251
Opiliones	Pettalidae	*Rakaia denticulata*	GBCH2632-08	DQ992250
Opiliones	Pettalidae	*Rakaia denticulata*	GBCH2633-08	DQ992249
Opiliones	Pettalidae	*Rakaia denticulata*	GBCH2634-08	DQ992248
Opiliones	Pettalidae	*Rakaia denticulata*	GBCH2635-08	DQ992247
Opiliones	Pettalidae	*Rakaia denticulata*	GBCH2636-08	DQ992246
Opiliones	Pettalidae	*Rakaia denticulata*	GBCH2637-08	DQ992245
Opiliones	Pettalidae	*Rakaia denticulata*	GBCH2638-08	DQ992244
Opiliones	Pettalidae	*Rakaia denticulata*	GBCH2639-08	DQ992243
Opiliones	Pettalidae	*Rakaia denticulata*	GBCH2640-08	DQ992242
Opiliones	Pettalidae	*Rakaia denticulata*	GBCH2641-08	DQ992241
Opiliones	Pettalidae	*Rakaia denticulata*	GBCH2642-08	DQ992240
Opiliones	Pettalidae	*Rakaia denticulata*	GBCH2643-08	DQ992239
Opiliones	Pettalidae	*Rakaia denticulata*	GBCH2644-08	DQ992238
Opiliones	Pettalidae	*Rakaia denticulata*	GBCH2645-08	DQ992237
Opiliones	Pettalidae	*Rakaia denticulata*	GBCH2646-08	DQ992236
Opiliones	Pettalidae	*Rakaia denticulata*	GBCH2647-08	DQ992235
Opiliones	Pettalidae	*Rakaia denticulata*	GBCH2648-08	DQ992234
Opiliones	Pettalidae	*Rakaia denticulata*	GBCH2649-08	DQ992233
Opiliones	Pettalidae	*Rakaia denticulata*	GBCH2650-08	DQ992232
Opiliones	Pettalidae	*Rakaia denticulata*	GBCH2651-08	DQ992231
Opiliones	Pettalidae	*Rakaia denticulata*	GBCH2652-08	DQ992230
Opiliones	Pettalidae	*Rakaia denticulata*	GBCH2653-08	DQ992229
Opiliones	Pettalidae	*Rakaia denticulata*	GBCH2654-08	DQ992228
Opiliones	Pettalidae	*Rakaia denticulata*	GBCH2655-08	DQ992227
Opiliones	Pettalidae	*Rakaia denticulata*	GBCH2656-08	DQ992226
Opiliones	Pettalidae	*Rakaia denticulata*	GBCH2657-08	DQ992225
Opiliones	Pettalidae	*Rakaia denticulata*	GBCH2658-08	DQ992224
Opiliones	Pettalidae	*Rakaia denticulata*	GBCH2659-08	DQ992223
Opiliones	Pettalidae	*Rakaia denticulata*	GBCH2660-08	DQ992222
Opiliones	Pettalidae	*Rakaia denticulata*	GBCH2661-08	DQ992221
Opiliones	Pettalidae	*Rakaia denticulata*	GBCH2662-08	DQ992220
Opiliones	Pettalidae	*Rakaia denticulata*	GBCH2663-08	DQ992219
Opiliones	Pettalidae	*Rakaia denticulata*	GBCH2664-08	DQ992218
Opiliones	Pettalidae	*Rakaia denticulata*	GBCH2665-08	DQ992217
Opiliones	Pettalidae	*Rakaia denticulata*	GBCH2666-08	DQ992216
Opiliones	Pettalidae	*Rakaia denticulata*	GBCH2667-08	DQ992215
Opiliones	Pettalidae	*Rakaia denticulata*	GBCH2668-08	DQ992214
Opiliones	Pettalidae	*Rakaia denticulata*	GBCH2669-08	DQ992213
Opiliones	Pettalidae	*Rakaia denticulata*	GBCH2670-08	DQ992212
Opiliones	Pettalidae	*Rakaia denticulata*	GBCH2671-08	DQ992211
Opiliones	Pettalidae	*Rakaia denticulata*	GBCH2672-08	DQ992210
Opiliones	Pettalidae	*Rakaia denticulata*	GBCH2673-08	DQ992209
Opiliones	Pettalidae	*Rakaia denticulata*	GBCH2674-08	DQ992208
Opiliones	Pettalidae	*Rakaia denticulata*	GBCH2675-08	DQ992207

Opiliones	Pettalidae	*Rakaia denticulata*	GBCH2676-08	DQ992206
Opiliones	Pettalidae	*Rakaia denticulata*	GBCH2677-08	DQ992205
Opiliones	Pettalidae	*Rakaia denticulata*	GBCH2678-08	DQ992204
Opiliones	Pettalidae	*Rakaia denticulata*	GBCH2679-08	DQ992203
Opiliones	Pettalidae	*Rakaia denticulata*	GBCH2680-08	DQ992202
Opiliones	Pettalidae	*Rakaia denticulata*	GBCH2681-08	DQ992201
Opiliones	Pettalidae	*Rakaia denticulata*	GBCH2682-08	DQ992200
Opiliones	Pettalidae	*Rakaia denticulata*	GBCH2683-08	DQ992199
Opiliones	Pettalidae	*Rakaia denticulata*	GBCH2684-08	DQ992198
Opiliones	Pettalidae	*Rakaia denticulata*	GBCH2685-08	DQ992197
Opiliones	Pettalidae	*Rakaia denticulata*	GBCH2686-08	DQ992196
Opiliones	Pettalidae	*Rakaia denticulata*	GBCH2687-08	DQ992195
Opiliones	Pettalidae	*Rakaia denticulata*	GBCH2688-08	DQ992194
Opiliones	Pettalidae	*Rakaia denticulata*	GBCH3702-09	DQ518126
Opiliones	Pettalidae	*Rakaia healyi*	GBCH1703-07	DQ518122
Opiliones	Pettalidae	*Rakaia healyi*	GBCH2561-08	DQ992321
Opiliones	Pettalidae	*Rakaia lindsayi*	GBCH2531-08	DQ992352
Opiliones	Pettalidae	*Rakaia longitarsa*	GBCH2568-08	DQ992314
Opiliones	Pettalidae	*Rakaia longitarsa*	GBCH2569-08	DQ992313
Opiliones	Pettalidae	*Rakaia macra*	GBCH3169-08	EU673668
Opiliones	Pettalidae	*Rakaia magna*	GBCH1701-07	DQ518124
Opiliones	Pettalidae	*Rakaia magna*	GBCH2541-08	DQ992342
Opiliones	Pettalidae	*Rakaia magna*	GBCH2542-08	DQ992341
Opiliones	Pettalidae	*Rakaia magna*	GBCH2543-08	DQ992340
Opiliones	Pettalidae	*Rakaia magna*	GBCH2544-08	DQ992339
Opiliones	Pettalidae	*Rakaia magna*	GBCH2545-08	DQ992338
Opiliones	Pettalidae	*Rakaia magna*	GBCH2546-08	DQ992337
Opiliones	Pettalidae	*Rakaia magna*	GBCH2547-08	DQ992336
Opiliones	Pettalidae	*Rakaia magna*	GBCH2548-08	DQ992335
Opiliones	Pettalidae	*Rakaia magna*	GBCH2549-08	DQ992334
Opiliones	Pettalidae	*Rakaia magna*	GBCH2550-08	DQ992333
Opiliones	Pettalidae	*Rakaia magna*	GBCH2551-08	DQ992332
Opiliones	Pettalidae	*Rakaia media*	GBCH3168-08	EU673669
Opiliones	Pettalidae	*Rakaia media*	GBCH3703-09	DQ518125
Opiliones	Pettalidae	*Rakaia n. sp. Akatarawa*	GBCH2538-08	DQ992345
Opiliones	Pettalidae	*Rakaia n. sp. Akatarawa*	GBCH2539-08	DQ992344
Opiliones	Pettalidae	*Rakaia n. sp. Hineway*	GBCH2534-08	DQ992349
Opiliones	Pettalidae	*Rakaia n. sp. Mt. Stokes*	GBCH2562-08	DQ992320
Opiliones	Pettalidae	*Rakaia n. sp. Mt. Stokes*	GBCH2563-08	DQ992319
Opiliones	Pettalidae	*Rakaia n. sp. Wi Toko*	GBCH2535-08	DQ992348
Opiliones	Pettalidae	*Rakaia n. sp. Wi Toko*	GBCH2536-08	DQ992347
Opiliones	Pettalidae	*Rakaia n. sp. Wi Toko*	GBCH2537-08	DQ992346
Opiliones	Pettalidae	*Rakaia solitaria*	GBCH1706-07	DQ518119
Opiliones	Pettalidae	*Rakaia solitaria*	GBCH2540-08	DQ992343
Opiliones	Pettalidae	*Rakaia sorenseni*	GBCH1709-07	DQ518116
Opiliones	Pettalidae	*Rakaia stewartiensis*	GBCH1708-07	DQ518117
Opiliones	Pettalidae	*Rakaia stewartiensis*	GBCH2532-08	DQ992351
Opiliones	Pettalidae	*Rakaia uniloca*	GBCH3167-08	EU673671
Opiliones	Phalangiidae	*Mitopus glacialis*	GBCH7611-13	JQ746516
Opiliones	Phalangiidae	*Mitopus glacialis*	GBCH7612-13	JQ746515
Opiliones	Phalangiidae	*Oligolophus triangularis*	ARONT605-10	KR069774
Opiliones	Phalangiidae	*Oligolophus triangularis*	CNSLL002-13	KM833344
Opiliones	Phalangiidae	*Oligolophus tridens*	CNEIG035-12	KM838218

Opiliones	Phalangiidae	*Oligolophus tridens*	CNEIH013-13	KM826150
Opiliones	Phalangiidae	*Oligolophus tridens*	CNEII002-13	KM837059
Opiliones	Phalangiidae	*Oligolophus tridens*	SSEIA3002-13	KM825658
Opiliones	Phalangiidae	*Oligolophus tridens*	SSEIA3013-13	KM838453
Opiliones	Phalangiidae	*Oligolophus tridens*	SSEIA3018-13	KM837289
Opiliones	Phalangiidae	*Oligolophus tridens*	SSEIA3021-13	KM829124
Opiliones	Phalangiidae	*Oligolophus tridens*	SSEIA3029-13	KM831000
Opiliones	Phalangiidae	*Oligolophus tridens*	SSEIA3030-13	KM828780
Opiliones	Phalangiidae	*Oligolophus tridens*	SSEIA3031-13	KM840100
Opiliones	Phalangiidae	*Oligolophus tridens*	SSEIA3225-13	KM829619
Opiliones	Phalangiidae	*Oligolophus tridens*	SSEIB8400-13	KM831307
Opiliones	Phalangiidae	*Phalangium opilio*	CNBAE240-12	KM839492
Opiliones	Phalangiidae	*Phalangium opilio*	CNBAE241-12	KM838652
Opiliones	Phalangiidae	*Phalangium opilio*	CNBAK455-13	KM835205
Opiliones	Phalangiidae	*Phalangium opilio*	CNEIC3179-13	KM831212
Opiliones	Phalangiidae	*Phalangium opilio*	CNEIF2377-12	KM828548
Opiliones	Phalangiidae	*Phalangium opilio*	CNGRK549-13	KM831665
Opiliones	Phalangiidae	*Phalangium opilio*	CNJAE763-12	KM837219
Opiliones	Phalangiidae	*Phalangium opilio*	CNJAE765-12	KM836031
Opiliones	Phalangiidae	*Phalangium opilio*	CNJAG1008-12	KM824469
Opiliones	Phalangiidae	*Phalangium opilio*	CNJAI007-12	KM833089
Opiliones	Phalangiidae	*Phalangium opilio*	GBCH2386-08	EU523757
Opiliones	Phalangiidae	*Phalangium opilio*	GBCH3211-08	NC_010766
Opiliones	Phalangiidae	*Phalangium opilio*	PHJUN4055-12	KR069168
Opiliones	Phalangiidae	*Phalangium opilio*	PHMTV446-10	KR070687
Opiliones	Phalangiidae	*Phalangium opilio*	PHMTV447-10	KR069548
Opiliones	Phalangiidae	*Phalangium opilio*	SSBAA5239-12	KM831278
Opiliones	Phalangiidae	*Phalangium opilio*	SSBAA5281-12	KM829631
Opiliones	Phalangiidae	*Phalangium opilio*	SSBAD5928-13	KM832445
Opiliones	Phalangiidae	*Phalangium opilio*	SSBAD5931-13	KM832585
Opiliones	Phalangiidae	*Phalangium opilio*	SSBAD5932-13	KM826036
Opiliones	Phalangiidae	*Phalangium opilio*	SSBAD5948-13	KM830072
Opiliones	Phalangiidae	*Phalangium opilio*	SSBAF7693-13	KM835065
Opiliones	Phalangiidae	*Phalangium opilio*	SSJAB1174-13	KM834733
Opiliones	Phalangiidae	*Phalangium opilio*	SSJAB1972-13	KM837024
Opiliones	Phalangiidae	*Phalangium opilio*	SSJAB1981-13	KM829801
Opiliones	Phalangiidae	*Phalangium opilio*	SSJAB1999-13	KM839229
Opiliones	Phalangiidae	*Phalangium opilio*	SSJAB2004-13	KM832254
Opiliones	Phalangiidae	*Phalangium opilio*	SSJAB2022-13	KM833262
Opiliones	Phalangiidae	*Phalangium opilio*	SSJAB2023-13	KM835031
Opiliones	Phalangiidae	*Phalangium opilio*	SSJAB2033-13	KM835201
Opiliones	Phalangiidae	*Phalangium opilio*	SSJAB2255-13	KM826968
Opiliones	Phalangiidae	*Phalangium opilio*	SSJAB2261-13	KM836271
Opiliones	Phalangiidae	*Phalangium opilio*	SSJAB2263-13	KM826644
Opiliones	Phalangiidae	*Phalangium opilio*	SSJAB3119-13	KM837474
Opiliones	Phalangiidae	*Phalangium opilio*	SSJAB3131-13	KM824303
Opiliones	Phalangiidae	*Phalangium opilio*	SSJAB3371-13	KM835376
Opiliones	Phalangiidae	*Phalangium opilio*	SSJAB3382-13	KM835464
Opiliones	Phalangiidae	*Phalangium opilio*	SSJAB3383-13	KM836703
Opiliones	Phalangiidae	*Phalangium opilio*	SSJAB3397-13	KM838338
Opiliones	Phalangiidae	*Phalangium opilio*	SSJAB3398-13	KM835597
Opiliones	Phalangiidae	*Phalangium opilio*	SSJAB3402-13	KM824640
Opiliones	Phalangiidae	*Phalangium opilio*	SSJAB3403-13	KM834616

Opiliones	Phalangiidae	*Phalangium opilio*	SSPAB5308-13	KM840699
Opiliones	Phalangiidae	*Phalangium opilio*	SSPAB8096-13	KM828647
Opiliones	Phalangiidae	*Phalangium opilio*	TDWGB817-10	HQ979250
Opiliones	Phalangiidae	*Platybunus triangularis*	CNSLA296-12	KM839696
Opiliones	Phalangiidae	*Platybunus triangularis*	CNSLB465-12	KM834322
Opiliones	Phalangiidae	*Platybunus triangularis*	CNSLB466-12	KM824836
Opiliones	Phalangiidae	*Platybunus triangularis*	CNSLC547-12	KM825059
Opiliones	Phalangiidae	*Platybunus triangularis*	CNSLD644-12	KM834213
Opiliones	Phalangiidae	*Rilaena triangularis*	CNSLB460-12	KM827466
Opiliones	Phalangiidae	*Rilaena triangularis*	CNSLB461-12	KM836571
Opiliones	Phalangiidae	*Rilaena triangularis*	CNSLB462-12	KM828089
Opiliones	Phalangiidae	*Rilaena triangularis*	CNSLB463-12	KM824772
Opiliones	Phalangiidae	*Rilaena triangularis*	CNSLB464-12	KM834869
Opiliones	Phalangiidae	*Rilaena triangularis*	PHMTT629-10	KR069805
Opiliones	Phalangiidae	*Rilaena triangularis*	PHMTU068-10	KR069644
Opiliones	Podoctidae	*Lomanius longipalpus longipalpus*	GACAC619-12	JF786432
Opiliones	Samoidae	*Santobius sp. DNA104931*	GACAC620-12	JF786433
Opiliones	Sclerosomatidae	*Hadrobunus nr. maculosus*	CNPPD2562-12	KJ086414
Opiliones	Sclerosomatidae	*Leiobunum*	CNSLC540-12	KM835265
Opiliones	Sclerosomatidae	*Leiobunum*	CNSLC542-12	KM824080
Opiliones	Sclerosomatidae	*Leiobunum*	CNSLD637-12	KM834522
Opiliones	Sclerosomatidae	*Leiobunum*	CNSLM131-13	KM840301
Opiliones	Sclerosomatidae	*Leiobunum*	CNSLM142-13	KM828411
Opiliones	Sclerosomatidae	*Leiobunum*	CNSLN098-13	KM828572
Opiliones	Sclerosomatidae	*Leiobunum*	CNSLO185-13	KM831975
Opiliones	Sclerosomatidae	*Leiobunum*	CNSLO188-13	KM835417
Opiliones	Sclerosomatidae	*Leiobunum*	CNSLO208-13	KM832758
Opiliones	Sclerosomatidae	*Leiobunum aldrichi*	CNBPJ182-13	KM829571
Opiliones	Sclerosomatidae	*Leiobunum exillipes*	SSJAA1389-13	KM838272
Opiliones	Sclerosomatidae	*Leiobunum exillipes*	SSJAA1395-13	KM833834
Opiliones	Sclerosomatidae	*Leiobunum exillipes*	SSJAA1397-13	KM837971
Opiliones	Sclerosomatidae	*Leiobunum exillipes*	SSJAA1398-13	KM837571
Opiliones	Sclerosomatidae	*Leiobunum exillipes*	SSJAA1400-13	KM836525
Opiliones	Sclerosomatidae	*Leiobunum exillipes*	SSJAA1947-13	KM830270
Opiliones	Sclerosomatidae	*Leiobunum exillipes*	SSJAA1948-13	KM826279
Opiliones	Sclerosomatidae	*Leiobunum exillipes*	SSJAA1949-13	KM828463
Opiliones	Sclerosomatidae	*Leiobunum exillipes*	SSJAA1950-13	KM831035
Opiliones	Sclerosomatidae	*Leiobunum exillipes*	SSJAA1951-13	KM829319
Opiliones	Sclerosomatidae	*Leiobunum exillipes*	SSJAA1952-13	KM834648
Opiliones	Sclerosomatidae	*Leiobunum exillipes*	SSJAA1954-13	KM840257
Opiliones	Sclerosomatidae	*Leiobunum exillipes*	SSJAA821-13	KM831993
Opiliones	Sclerosomatidae	*Leiobunum exillipes*	SSJAA830-13	KM839244
Opiliones	Sclerosomatidae	*Leiobunum exillipes*	SSJAA832-13	KM833080
Opiliones	Sclerosomatidae	*Leiobunum exillipes*	SSJAC1233-13	KM836786
Opiliones	Sclerosomatidae	*Leiobunum exillipes*	SSJAC1257-13	KM836386
Opiliones	Sclerosomatidae	*Leiobunum exillipes*	SSJAC1650-13	KM836503
Opiliones	Sclerosomatidae	*Leiobunum vittatum*	CNPPD2560-12	KJ091171
Opiliones	Sclerosomatidae	*Leiobunum vittatum*	CNPPD2561-12	KJ086215
Opiliones	Sclerosomatidae	*Leiobunum vittatum*	CNPPD2563-12	KJ092942
Opiliones	Sclerosomatidae	*Leiobunum vittatum*	CNPPD2564-12	KJ089562
Opiliones	Sclerosomatidae	*Leiobunum vittatum*	CNPPD2565-12	KJ089727
Opiliones	Sclerosomatidae	*Leiobunum vittatum*	CNPPD2566-12	KJ091939
Opiliones	Sclerosomatidae	*Leiobunum vittatum*	CNPPD2567-12	KJ164989

Opiliones	Sclerosomatidae	*Leiobunum vittatum*	CNPPD2568-12	KJ086195
Opiliones	Sclerosomatidae	*Leiobunum vittatum*	CNPPD2569-12	KJ090904
Opiliones	Sclerosomatidae	*Leiobunum vittatum*	CNPPD2570-12	KJ087072
Opiliones	Sclerosomatidae	*Leiobunum vittatum*	CNPPD2571-12	KJ087720
Opiliones	Sclerosomatidae	*Leiobunum vittatum*	CNPPD2573-12	KJ085378
Opiliones	Sclerosomatidae	*Leiobunum vittatum*	CNPPD2574-12	KJ089860
Opiliones	Sclerosomatidae	*Leiobunum vittatum*	CNPPD2575-12	KJ086695
Opiliones	Sclerosomatidae	*Leiobunum vittatum*	CNPPD2576-12	KJ089649
Opiliones	Sclerosomatidae	*Leiobunum vittatum*	CNPPD2577-12	KJ091654
Opiliones	Sclerosomatidae	*Leiobunum vittatum*	CNPPD2578-12	KJ092161
Opiliones	Sclerosomatidae	*Leiobunum vittatum*	CNPPD2579-12	KJ087042
Opiliones	Sclerosomatidae	*Leiobunum vittatum*	CNPPD2580-12	KJ087217
Opiliones	Sclerosomatidae	*Leiobunum vittatum*	CNPPD2581-12	KJ164213
Opiliones	Sclerosomatidae	*Leiobunum vittatum*	CNPPD2582-12	KJ085615
Opiliones	Sclerosomatidae	*Leiobunum vittatum*	CNPPD2583-12	KJ088438
Opiliones	Sclerosomatidae	*Leiobunum vittatum*	CNPPD2584-12	KJ092664
Opiliones	Sclerosomatidae	*Leiobunum vittatum*	CNPPD2585-12	KJ088730
Opiliones	Sclerosomatidae	*Leiobunum vittatum*	CNPPD2586-12	KJ090129
Opiliones	Sclerosomatidae	*Leiobunum vittatum*	CNPPD2587-12	KJ088490
Opiliones	Sclerosomatidae	*Leiobunum vittatum*	CNPPD2588-12	KJ093115
Opiliones	Sclerosomatidae	*Leiobunum vittatum*	CNPPD2589-12	KJ088972
Opiliones	Sclerosomatidae	*Leiobunum vittatum*	CNPPD2590-12	KJ089984
Opiliones	Sclerosomatidae	*Leiobunum vittatum*	CNPPD2591-12	KJ087921
Opiliones	Sclerosomatidae	*Leiobunum vittatum*	CNPPD2592-12	KJ091277
Opiliones	Sclerosomatidae	*Leiobunum vittatum*	CNPPD2593-12	KJ163361
Opiliones	Sclerosomatidae	*Leiobunum vittatum*	CNPPD2594-12	KJ084266
Opiliones	Sclerosomatidae	*Leiobunum vittatum*	CNPPE2309-12	KJ085951
Opiliones	Sclerosomatidae	*Leiobunum vittatum*	CNPPE2310-12	KJ165201
Opiliones	Sclerosomatidae	*Leiobunum vittatum*	CNPPE2311-12	KJ165769
Opiliones	Sclerosomatidae	*Leiobunum vittatum*	CNPPE2312-12	KJ167500
Opiliones	Sclerosomatidae	*Leiobunum vittatum*	CNPPE2313-12	KJ088891
Opiliones	Sclerosomatidae	*Leiobunum vittatum*	CNPPE2315-12	KJ164740
Opiliones	Sclerosomatidae	*Leiobunum vittatum*	CNPPE2318-12	KJ166724
Opiliones	Sclerosomatidae	*Leiobunum vittatum*	GBCH5178-10	EU271671
Opiliones	Sclerosomatidae	*Nelima paessleri*	CNPCC040-13	KM828987
Opiliones	Sclerosomatidae	*Nelima paessleri*	SSBAB2867-12	KM836374
Opiliones	Sclerosomatidae	*Nelima paessleri*	SSBAB2889-12	KM835873
Opiliones	Sclerosomatidae	*Nelima paessleri*	SSBAB2890-12	KM838884
Opiliones	Sclerosomatidae	*Nelima paessleri*	SSBAB3048-13	KM837157
Opiliones	Sclerosomatidae	*Nelima paessleri*	SSBAB486-12	KM829611
Opiliones	Sclerosomatidae	*Nelima paessleri*	SSBAB487-12	KM827766
Opiliones	Sclerosomatidae	*Nelima paessleri*	SSBAB488-12	KM829927
Opiliones	Sclerosomatidae	*Nelima paessleri*	SSBAB502-12	KM829233
Opiliones	Sclerosomatidae	*Nelima paessleri*	SSBAB503-12	KM837433
Opiliones	Sclerosomatidae	*Nelima paessleri*	SSBAB507-12	KM828354
Opiliones	Sclerosomatidae	*Nelima paessleri*	SSBAB525-12	KM831332
Opiliones	Sclerosomatidae	*Nelima paessleri*	SSBAB526-12	KM824807
Opiliones	Sclerosomatidae	*Nelima paessleri*	SSBAD5927-13	KM840534
Opiliones	Sclerosomatidae	*Nelima paessleri*	SSBAE6333-13	KM831992
Opiliones	Sclerosomatidae	*Nelima paessleri*	SSBAE6341-13	KM824387
Opiliones	Sclerosomatidae	*Nelima paessleri*	SSBAE6344-13	KM829501
Opiliones	Sclerosomatidae	*Nelima paessleri*	SSBAE6345-13	KM838078
Opiliones	Sclerosomatidae	*Nelima paessleri*	SSWLC002-13	KM830746

Opiliones	Sclerosomatidae		CNPPF1445-12	KJ167362
Opiliones	Sironidae	*Cyphophthalmus duricorius*	GBCH0953-06	AY639556
Opiliones	Sironidae	*Cyphophthalmus duricorius*	GBCH4324-09	FJ946435
Opiliones	Sironidae	*Cyphophthalmus ere*	GBCH0954-06	AY639557
Opiliones	Sironidae	*Cyphophthalmus ere*	GBCH0955-06	AY639558
Opiliones	Sironidae	*Cyphophthalmus gjorgjevici*	GBCH0956-06	AY639559
Opiliones	Sironidae	*Cyphophthalmus gjorgjevici*	GBCH0957-06	AY639560
Opiliones	Sironidae	*Cyphophthalmus markoi*	GBCH0958-06	AY639561
Opiliones	Sironidae	*Cyphophthalmus martensi*	GBCH0959-06	AY639562
Opiliones	Sironidae	*Cyphophthalmus martensi*	GBCH0960-06	AY639563
Opiliones	Sironidae	*Cyphophthalmus martensi*	GBCH0961-06	AY639564
Opiliones	Sironidae	*Cyphophthalmus minutus*	GBCH0962-06	AY639565
Opiliones	Sironidae	*Cyphophthalmus minutus*	GBCH0963-06	AY639566
Opiliones	Sironidae	*Cyphophthalmus ognjanovici*	GBCH0964-06	AY639567
Opiliones	Sironidae	*Cyphophthalmus rumijae*	GBCH0965-06	AY639568
Opiliones	Sironidae	*Cyphophthalmus rumijae*	GBCH0966-06	AY639569
Opiliones	Sironidae	*Cyphophthalmus rumijae*	GBCH0967-06	AY639570
Opiliones	Sironidae	*Cyphophthalmus sp. 1 Bulgaria*	GBCH1161-06	AY918878
Opiliones	Sironidae	*Cyphophthalmus sp. 2 Bulgaria*	GBCH1162-06	AY918879
Opiliones	Sironidae	*Cyphophthalmus teyrovskyi*	GBCH0968-06	AY639571
Opiliones	Sironidae	*Cyphophthalmus trebinjanum*	GBCH0969-06	AY639572
Opiliones	Sironidae	*Cyphophthalmus zetae*	GBCH0970-06	AY639573
Opiliones	Sironidae	*Cyphophthalmus zetae*	GBCH0971-06	AY639574
Opiliones	Sironidae	*Cyphophthalmus zetae*	GBCH0972-06	AY639575
Opiliones	Sironidae	*Cyphophthalmus zetae*	GBCH0973-06	AY639576
Opiliones	Sironidae	*Cyphophthalmus zetae*	GBCH0974-06	AY639577
Opiliones	Sironidae	*Paramiopsalis ramulosus*	GACAC592-12	JF786389
Opiliones	Sironidae	*Paramiopsalis ramulosus*	GBCH1873-07	DQ513109
Opiliones	Sironidae	*Paramiopsalis ramulosus*	GBCH3693-09	DQ825641
Opiliones	Sironidae	*Parasiro coiffaiti*	GBCH1872-07	DQ513110
Opiliones	Sironidae	*Parasiro coiffaiti*	GBCH3692-09	DQ825642
Opiliones	Sironidae	*Parasiro minor*	GACAC594-12	JF786391
Opiliones	Sironidae	*Siro acaroides*	GBCH3690-09	DQ825644
Opiliones	Sironidae	*Siro boyerae*	GBCH1870-07	DQ513112
Opiliones	Sironidae	*Siro exilis*	GBCH0976-06	AY639579
Opiliones	Stygnommatidae	*Stygnomma sp.*	GACAC639-12	JF786452
Opiliones	Stygnommatidae	*Stygnomma sp.*	GACAC640-12	JF786453
Opiliones	Stygnommatidae	*Stygnomma sp.*	GACAC642-12	JF786455
Opiliones	Stygnommatidae	*Stygnomma teapense*	GACAC641-12	JF786454
Opiliones	Stygnopsidae	*Stygnopsis sp. DNA101416*	GACAC634-12	JF786447
Opiliones	Stygnopsidae	*Stygnopsis sp. DNA104855*	GACAC631-12	JF786444
Opiliones	Stygnopsidae	*Stygnopsis sp. DNA104856*	GACAC632-12	JF786445
Opiliones	Stylocellidae	*Fangensis spelaeus*	GBCH0980-06	AY639583
Opiliones	Stylocellidae	*Stylocellus sp. MCZDNA100609*	GBCH3696-09	DQ825625
Opiliones	Stylocellidae	*Stylocellus sp. MCZDNA101469*	GBCH3695-09	DQ825626
Opiliones	Stylocellidae	*Stylocellus sp. MCZDNA101472*	GBCH3694-09	DQ825627
Opiliones	Stylocellidae	*Stylocellus sp. MCZDNA101483*	GBCH1831-07	DQ825633
Opiliones	Stylocellidae	*Stylocellus sp. MCZDNA101486*	GBCH1833-07	DQ825631
Opiliones	Stylocellidae	*Stylocellus sp. MCZDNA101488*	GBCH1830-07	DQ825634
Opiliones	Stylocellidae	*Stylocellus sp. MCZDNA101494*	GBCH1832-07	DQ825632
Opiliones	Stylocellidae	*Stylocellus sp. MCZDNA101514*	GBCH1836-07	DQ825628
Opiliones	Stylocellidae	*Stylocellus sp. MCZDNA101519*	GBCH1835-07	DQ825629
Opiliones	Stylocellidae	*Stylocellus sp. Pahang 1*	GBCH1728-07	DQ518097

Opiliones	Triaenonychidae	*Cyptobunus ungulatus*	GBCH4393-10	HM056739
Opiliones	Triaenonychidae	*Cyptobunus ungulatus*	GBCH4394-10	HM056738
Opiliones	Triaenonychidae	*Cyptobunus ungulatus*	GBCH4395-10	HM056737
Opiliones	Triaenonychidae	*Cyptobunus ungulatus madhousensis*	GBCH7379-13	JN547483
Opiliones	Triaenonychidae	*Metanonychus setulus*	GBCH4401-10	HM056731
Opiliones	Triaenonychidae	*Paranonychus brunneus*	GBCH4404-10	HM056728
Opiliones	Triaenonychidae	*Zuma acuta*	GBCH4398-10	HM056734
Opiliones	Troglosironidae	*Troglosiro aelleni*	GBCH0981-06	AY639584
Opiliones	Troglosironidae	*Troglosiro brevifossa*	GBCH4284-09	EU887039
Opiliones	Troglosironidae	*Troglosiro cf. juberthiei PS-2008*	GBCH4259-09	EU887072
Opiliones	Troglosironidae	*Troglosiro cf. juberthiei PS-2008*	GBCH4260-09	EU887071
Opiliones	Troglosironidae	*Troglosiro cf. juberthiei PS-2008*	GBCH4261-09	EU887070
Opiliones	Troglosironidae	*Troglosiro cf. juberthiei PS-2008*	GBCH4262-09	EU887069
Opiliones	Troglosironidae	*Troglosiro cf. juberthiei PS-2008*	GBCH4263-09	EU887068
Opiliones	Troglosironidae	*Troglosiro cf. juberthiei PS-2008*	GBCH4264-09	EU887067
Opiliones	Troglosironidae	*Troglosiro cf. juberthiei PS-2008*	GBCH4265-09	EU887066
Opiliones	Troglosironidae	*Troglosiro cf. juberthiei PS-2008*	GBCH4266-09	EU887065
Opiliones	Troglosironidae	*Troglosiro cf. juberthiei PS-2008*	GBCH4267-09	EU887064
Opiliones	Troglosironidae	*Troglosiro cf. juberthiei PS-2008*	GBCH4268-09	EU887063
Opiliones	Troglosironidae	*Troglosiro cf. juberthiei PS-2008*	GBCH4269-09	EU887062
Opiliones	Troglosironidae	*Troglosiro cf. juberthiei PS-2008*	GBCH4271-09	EU887059
Opiliones	Troglosironidae	*Troglosiro cf. juberthiei PS-2008*	GBCH4272-09	EU887058
Opiliones	Troglosironidae	*Troglosiro cf. juberthiei PS-2008*	GBCH4273-09	EU887057
Opiliones	Troglosironidae	*Troglosiro cf. juberthiei PS-2008*	GBCH4274-09	EU887056
Opiliones	Troglosironidae	*Troglosiro cf. juberthiei PS-2008*	GBCH4275-09	EU887055
Opiliones	Troglosironidae	*Troglosiro cf. juberthiei PS-2008*	GBCH4276-09	EU887054
Opiliones	Troglosironidae	*Troglosiro cf. juberthiei PS-2008*	GBCH4277-09	EU887053
Opiliones	Troglosironidae	*Troglosiro cf. juberthiei PS-2008*	GBCH4278-09	EU887045
Opiliones	Troglosironidae	*Troglosiro juberthiei*	GBCH4252-09	EU887052
Opiliones	Troglosironidae	*Troglosiro juberthiei*	GBCH4253-09	EU887051
Opiliones	Troglosironidae	*Troglosiro juberthiei*	GBCH4254-09	EU887050
Opiliones	Troglosironidae	*Troglosiro juberthiei*	GBCH4255-09	EU887049
Opiliones	Troglosironidae	*Troglosiro juberthiei*	GBCH4256-09	EU887048
Opiliones	Troglosironidae	*Troglosiro juberthiei*	GBCH4257-09	EU887047
Opiliones	Troglosironidae	*Troglosiro juberthiei*	GBCH4270-09	EU887060
Opiliones	Troglosironidae	*Troglosiro longifossa*	GBCH1825-07	DQ825639
Opiliones	Troglosironidae	*Troglosiro longifossa*	GBCH3701-09	DQ518127
Opiliones	Troglosironidae	*Troglosiro monteithi*	GBCH4279-09	EU887043
Opiliones	Troglosironidae	*Troglosiro ninqua*	GBCH3700-09	DQ518128
Opiliones	Troglosironidae	*Troglosiro oscitatio*	GBCH4281-09	EU887041
Opiliones	Troglosironidae	*Troglosiro raveni*	GBCH4280-09	EU887042
Opiliones	Troglosironidae	*Troglosiro sheari*	GBCH4285-09	EU887038
Opiliones	Troglosironidae	*Troglosiro sheari*	GBCH4286-09	EU887037
Opiliones	Troglosironidae	*Troglosiro sp. PS-2008*	GBCH4258-09	EU887046
Opiliones	Troglosironidae	*Troglosiro urbanus*	GBCH4282-09	EU887044
Opiliones	Troglosironidae	*Troglosiro urbanus*	GBCH4283-09	EU887040
Opiliones	Troglosironidae	*Troglosiro wilsoni*	GBCH4251-09	EU887061
Opiliones	Trogulidae	*Trogulus nepaeformis*	GBCH7784-13	JN018142
Opiliones	Zalmoxidae	*Zalmoxida sp. DNA102677*	GACAC621-12	JF786434
Palpigradi			GBCH7742-13	JN018214
Palpigradi			GBCH7757-13	JN018169
Pseudoscorpiones	Atemnidae	*Caecatemnus sp. JM-2008*	GBCH3494-09	EU559534
Pseudoscorpiones	Atemnidae	*Cyclatemnus sp. JM-2008*	GBCH3500-09	EU559528

Pseudoscorpiones	Atemnidae	*Miratemnus sp. JM-2008*	GBCH3493-09	EU559535
Pseudoscorpiones	Atemnidae	*Oratemnus curtus*	GBCH3497-09	EU559531
Pseudoscorpiones	Atemnidae	*Stenatemnus sp. JM-2008*	GBCH3499-09	EU559529
Pseudoscorpiones	Atemnidae	*Titanatemnus sp. JM-2008*	GBCH3498-09	EU559530
Pseudoscorpiones	Cheiridiidae	*Apocheiridium reddelli*	GACPS019-13	JN018178
Pseudoscorpiones	Cheiridiidae	*Cheiridium museorum*	GACPS004-13	JN018207
Pseudoscorpiones	Cheiridiidae		GACAC125-12	EU559570
Pseudoscorpiones	Cheliferidae	*Beierochelifer peloponnesiacus*	GACPS022-13	JN018175
Pseudoscorpiones	Cheliferidae	*Nannochelifer sp. JM-2008*	GBCH3496-09	EU559532
Pseudoscorpiones	Cheliferidae	*Philomaoria sp. JM-2008*	GBCH3492-09	EU559536
Pseudoscorpiones	Cheliferidae	*Protochelifer victorianus*	GBCH3495-09	EU559533
Pseudoscorpiones	Cheliferidae	*Rhacochelifer maculatus*	GACPS006-13	JN018205
Pseudoscorpiones	Cheliferidae		CNGIE253-12	KM836901
Pseudoscorpiones	Cheliferidae		CNGIH317-13	KM835770
Pseudoscorpiones	Chernetidae	*Apatochernes sp. JM-2008*	GBCH3501-09	EU559527
Pseudoscorpiones	Chernetidae	*Calymmachernes angulatus*	GBCH3503-09	EU559525
Pseudoscorpiones	Chernetidae	*Conicochernes crassus*	GBCH3502-09	EU559526
Pseudoscorpiones	Chernetidae	*Haplochernes sp. JM-2008*	GBCH3504-09	EU559524
Pseudoscorpiones	Chernetidae	*Incachernes sp. JM-2008*	GBCH3505-09	EU559523
Pseudoscorpiones	Chernetidae	*Lamprochernes savignyi*	GACPS020-13	JN018177
Pseudoscorpiones	Chernetidae	*Marachernes bellus*	GBCH3506-09	EU559522
Pseudoscorpiones	Chernetidae	*Pseudopilanus kuscheli*	GBCH3456-09	EU559574
Pseudoscorpiones	Chernetidae		ASAMT204-12	KR069476
Pseudoscorpiones	Chernetidae		CNEIB165-12	KM836979
Pseudoscorpiones	Chernetidae		CNPAK022-13	KM837464
Pseudoscorpiones	Chernetidae		CNWBB106-13	KM831486
Pseudoscorpiones	Chernetidae		GACPS001-13	EU559573
Pseudoscorpiones	Chernetidae		PAJUN035-12	KR069547
Pseudoscorpiones	Chernetidae		PAJUN036-12	KR069262
Pseudoscorpiones	Chernetidae		SMTPB8974-13	KP979323
Pseudoscorpiones	Chernetidae		SMTPB8975-13	KR070180
Pseudoscorpiones	Chernetidae		SMTPB9463-13	KR070532
Pseudoscorpiones	Chernetidae		SMTPB9464-13	KR070712
Pseudoscorpiones	Chernetidae		SSEIA7678-13	KM836634
Pseudoscorpiones	Chernetidae		SSEIB2132-13	KM826138
Pseudoscorpiones	Chernetidae		SSEIB2133-13	KM824930
Pseudoscorpiones	Chernetidae		SSEIB2134-13	KM824612
Pseudoscorpiones	Chernetidae		SSWLD4310-13	KM840075
Pseudoscorpiones	Chernetidae		TTSOW554-11	KR069803
Pseudoscorpiones	Chernetidae		TTSOW555-11	KR070275
Pseudoscorpiones	Chernetidae		TTSOW556-11	KP979117
Pseudoscorpiones	Chthoniidae	*Afrochthonius godfreyi*	GBCH3521-09	EU559507
Pseudoscorpiones	Chthoniidae	*Apochthonius sp. JM-2008*	GBCH3511-09	EU559517
Pseudoscorpiones	Chthoniidae	*Austrochthonius sp. Australia*	GBCH3515-09	EU559513
Pseudoscorpiones	Chthoniidae	*Austrochthonius sp. JM-2008*	GBCH3514-09	EU559514
Pseudoscorpiones	Chthoniidae	*Austrochthonius sp. JM-2008*	GBCH3516-09	EU559512
Pseudoscorpiones	Chthoniidae	*Chthonius sp. 1 JM-2008*	GBCH3517-09	EU559511
Pseudoscorpiones	Chthoniidae	*Chthonius sp. 2 JM-2008*	GBCH3524-09	EU559504
Pseudoscorpiones	Chthoniidae	*Chthonius tetrachelatus*	GACPS005-13	JN018206
Pseudoscorpiones	Chthoniidae	*Drepanochthonius sp. JM-2008*	GBCH3513-09	EU559515
Pseudoscorpiones	Chthoniidae	*Kleptochthonius sp. JM-2008*	GBCH3510-09	EU559518
Pseudoscorpiones	Chthoniidae	*Lagynochthonius*	GACPS024-13	JN018173
Pseudoscorpiones	Chthoniidae	*Lagynochthonius johni*	GBCH3525-09	EU559503

Pseudoscorpiones	Chthoniidae	*Paraliochthonius sp. JM-2008*	GBCH3523-09	EU559505
Pseudoscorpiones	Chthoniidae	*Pseudochthonius sp. JM-2008*	GBCH3509-09	EU559519
Pseudoscorpiones	Chthoniidae	*Pseudotyrannochthonius sp. Australia*	GBCH3520-09	EU559508
Pseudoscorpiones	Chthoniidae	*Pseudotyrannochthonius sp. JM-2008*	GBCH3519-09	EU559509
Pseudoscorpiones	Chthoniidae	*Sathrochthonius insulanus*	GBCH3507-09	EU559521
Pseudoscorpiones	Chthoniidae	*Selachochthonius sp. JM-2008*	GBCH3518-09	EU559510
Pseudoscorpiones	Chthoniidae	*Tyrannochthonius*	GACPS023-13	JN018174
Pseudoscorpiones	Chthoniidae	*Tyrannochthonius sp. JM-2008*	GBCH3522-09	EU559506
Pseudoscorpiones	Feaellidae	*Feaella anderseni*	GBCH3528-09	EU559500
Pseudoscorpiones	Garypidae	*Anagarypus heatwolei*	GBCH3488-09	EU559540
Pseudoscorpiones	Garypidae	*Synsphyronus apimelus*	GBCH3491-09	EU559537
Pseudoscorpiones	Garypinidae	*Garypinus*	GACPS018-13	JN018179
Pseudoscorpiones	Geogarypidae	*Geogarypus nigrimanus*	GACPS017-13	JN018180
Pseudoscorpiones	Geogarypidae	*Geogarypus sp. JM-2008*	GBCH3468-09	EU559560
Pseudoscorpiones	Gymnobisiidae	*Mirobisium sp. JM-2008*	GBCH3481-09	EU559547
Pseudoscorpiones	Hyidae	*Indohya sp. JM-2008*	GBCH3464-09	EU559564
Pseudoscorpiones	Ideoroncidae	*Pseudalbiorix veracruzensis*	GBCH3461-09	EU559567
Pseudoscorpiones	Larcidae	*Larca lata*	GBCH3465-09	EU559563
Pseudoscorpiones	Lechytiidae	*Lechytia hoffi*	GBCH3512-09	EU559516
Pseudoscorpiones	Neobisiidae	*Bisetocreagris*	GACPS015-13	JN018182
Pseudoscorpiones	Neobisiidae	*Bisetocreagris*	GACPS016-13	JN018181
Pseudoscorpiones	Neobisiidae	*Lissocreagris sp. JM-2008*	GBCH3473-09	EU559555
Pseudoscorpiones	Neobisiidae	*Microbisium*	CNPPB922-12	KJ084070
Pseudoscorpiones	Neobisiidae	*Microbisium*	SSEIA996-13	KM839785
Pseudoscorpiones	Neobisiidae	*Microbisium*	SSEIB8399-13	KM831954
Pseudoscorpiones	Neobisiidae	*Microbisium parvulum*	GBCH3470-09	EU559558
Pseudoscorpiones	Neobisiidae	*Neobisium polonicum*	GBCH3472-09	EU559556
Pseudoscorpiones	Neobisiidae	*Roncus transsilvanicus*	GBCH3471-09	EU559557
Pseudoscorpiones	Olipiidae	*Apolpium parvum*	GBCH3487-09	EU559541
Pseudoscorpiones	Olipiidae	*Beierolpium bornemisszai*	GBCH3483-09	EU559545
Pseudoscorpiones	Olipiidae	*Calocheiridius termitophilus*	GBCH3484-09	EU559544
Pseudoscorpiones	Olipiidae	*Euryolpium sp. JM-2008*	GBCH3482-09	EU559546
Pseudoscorpiones	Olipiidae	*Nanolpium sp. JM-2008*	GBCH3485-09	EU559543
Pseudoscorpiones	Olipiidae	*Pachyolpium sp. JM-2008*	GBCH3486-09	EU559542
Pseudoscorpiones	Olipiidae	*Progarypus sp. JM-2008*	GBCH3490-09	EU559538
Pseudoscorpiones	Olipiidae	*Protogarypinus giganteus*	GBCH3463-09	EU559565
Pseudoscorpiones	Olipiidae	*Pseudogarypinus cooperi*	GBCH3462-09	EU559566
Pseudoscorpiones	Olipiidae	*Xenolpium sp. JM-2008*	GBCH3489-09	EU559539
Pseudoscorpiones	Parahyidae	*Parahya submersa*	GBCH3480-09	EU559548
Pseudoscorpiones	Pseudogarypidae	*Neopseudogarypus scutellatus*	GBCH3526-09	EU559502
Pseudoscorpiones	Pseudogarypidae	*Pseudogarypus bicornis*	GBCH3527-09	EU559501
Pseudoscorpiones	Sternophoridae	*Afrosternophorus sp. JM-2008*	GBCH3460-09	EU559568
Pseudoscorpiones	Sternophoridae	*Garyops depressus*	GBCH3459-09	EU559569
Pseudoscorpiones	Syarinidae	*Chitrella cala*	GBCH3477-09	EU559551
Pseudoscorpiones	Syarinidae	*Ideobisium sp. JM-2008*	GBCH3479-09	EU559549
Pseudoscorpiones	Syarinidae	*Ideoblothrus sp. JM-2008*	GBCH3466-09	EU559562
Pseudoscorpiones	Syarinidae	*Nannobisium sp. JM-2008*	GBCH3467-09	EU559561
Pseudoscorpiones	Syarinidae	*Syarinus sp. JM-2008*	GBCH3478-09	EU559550
Pseudoscorpiones	Tridenchthoniidae	*Anaulacodithella sp. JM-2008*	GBCH3508-09	EU559520
Pseudoscorpiones	Withiidae	*Withius sp. 1 JM-2008*	GBCH3458-09	EU559571
Pseudoscorpiones	Withiidae	*Withius sp. 2 JM-2008*	GBCH3457-09	EU559572
Ricinulei	Ricinoididae	*Pseudocellus pearsei*	GBCH2689-08	NC_009985
Ricinulei	Ricinoididae	*Pseudocellus pearsei*	GBCH2722-08	EU024483

Ricinulei	Ricinoididae	*Ricinoides atewa*	GBCH7756-13	JN018170
Sarcoptiformes	Acaridae	*Acarus farris*	GBCH4463-10	GQ864338
Sarcoptiformes	Acaridae	*Rhizoglyphus robini*	GBCH4446-10	GQ864356
Sarcoptiformes	Acaridae	*Tyroborus sp. AMUEnv048*	GBCH4469-10	GQ864332
Sarcoptiformes	Acaridae		CNPAG349-13	KM828834
Sarcoptiformes	Acaridae		CNPAI056-13	KM827283
Sarcoptiformes	Acaridae		MYMCB545-11	JX833712
Sarcoptiformes	Acaridae		MYMCB546-11	JX836827
Sarcoptiformes	Achipteriidae	*Achipteria*	RBINA5693-13	KP979140
Sarcoptiformes	Achipteriidae	*Achipteria*	SSPAA8262-13	KM825418
Sarcoptiformes	Achipteriidae	*Achipteria coleoptrata*	CHACA198-08	JX833967
Sarcoptiformes	Achipteriidae	*Achipteria coleoptrata*	CHACA199-08	JX836841
Sarcoptiformes	Achipteriidae	*Achipteria coleoptrata*	CHACA200-08	JX837562
Sarcoptiformes	Achipteriidae	*Achipteria coleoptrata*	CHACA207-08	JX834308
Sarcoptiformes	Achipteriidae	*Achipteria coleoptrata*	CHACA211-08	JX837920
Sarcoptiformes	Achipteriidae	*Achipteria coleoptrata*	CHACA212-08	JX836700
Sarcoptiformes	Achipteriidae	*Achipteria coleoptrata*	CHACA545-09	JX837580
Sarcoptiformes	Achipteriidae	*Achipteria coleoptrata*	CHACA574-09	JX835726
Sarcoptiformes	Achipteriidae	*Achipteria coleoptrata*	CHACA575-09	JX833773
Sarcoptiformes	Achipteriidae	*Achipteria coleoptrata*	CHACA576-09	JX835084
Sarcoptiformes	Achipteriidae	*Achipteria coleoptrata*	CHACB229-10	HQ558480
Sarcoptiformes	Achipteriidae	*Achipteria coleoptrata*	CHACB590-10	HQ558690
Sarcoptiformes	Achipteriidae	*Achipteria coleoptrata*	CHACB591-10	HQ558691
Sarcoptiformes	Achipteriidae	*Achipteria coleoptrata*	CHACB592-10	HQ558692
Sarcoptiformes	Achipteriidae	*Achipteria coleoptrata*	CHACB593-10	JX835862
Sarcoptiformes	Achipteriidae	*Achipteria coleoptrata*	CHACB594-10	HQ558693
Sarcoptiformes	Achipteriidae	*Achipteria coleoptrata*	CHACB595-10	HQ558694
Sarcoptiformes	Achipteriidae	*Achipteria coleoptrata*	MITMH004-07	KR069757
Sarcoptiformes	Achipteriidae	*Achipteria coleoptrata*	MYMCE261-12	JX835725
Sarcoptiformes	Achipteriidae	*Anachipteria*	SSBAC3374-12	KM825277
Sarcoptiformes	Achipteriidae	*Anachipteria*	SSPAA7834-13	KM830970
Sarcoptiformes	Achipteriidae	*Anachipteria*	SSPAA7938-13	KM823973
Sarcoptiformes	Achipteriidae	*Anachipteria howardi*	CHACB013-10	HQ558334
Sarcoptiformes	Achipteriidae	*Anachipteria howardi*	CHACB027-10	HQ558346
Sarcoptiformes	Achipteriidae	*Anachipteria howardi*	CHACB046-10	JX835525
Sarcoptiformes	Achipteriidae	*Anachipteria howardi*	CHACB1080-10	HM907193
Sarcoptiformes	Achipteriidae	*Anachipteria howardi*	CHACB1081-10	HM907194
Sarcoptiformes	Achipteriidae	*Anachipteria howardi*	CHACB1116-10	HM907210
Sarcoptiformes	Achipteriidae	*Anachipteria howardi*	CHACB304-10	HM907295
Sarcoptiformes	Achipteriidae	*Anachipteria howardi*	CHACB308-10	HM907299
Sarcoptiformes	Achipteriidae	*Anachipteria howardi*	CHACB589-10	HQ558689
Sarcoptiformes	Achipteriidae	*Anachipteria howardi*	CHACB661-10	HQ558738
Sarcoptiformes	Achipteriidae	*Anachipteria howardi*	CHACC088-10	HM907480
Sarcoptiformes	Achipteriidae	*Anachipteria howardi*	CHACC099-10	JX835677
Sarcoptiformes	Achipteriidae	*Anachipteria howardi*	MITMH012-07	KR070238
Sarcoptiformes	Achipteriidae	*Anachipteria howardi*	MITMH034-07	KR069880
Sarcoptiformes	Achipteriidae	*Anachipteria howardi*	MITMH036-07	KR069971
Sarcoptiformes	Achipteriidae	*Anachipteria howardi*	MITMH061-07	KR069306
Sarcoptiformes	Achipteriidae	*Anachipteria howardi*	MYMCA081-11	JX838689
Sarcoptiformes	Achipteriidae	*Anachipteria howardi*	MYMCA155-11	JX838445
Sarcoptiformes	Achipteriidae	*Anachipteria howardi*	MYMCA156-11	JX838322
Sarcoptiformes	Achipteriidae	*Anachipteria howardi*	MYMCA157-11	JX838173
Sarcoptiformes	Achipteriidae	*Anachipteria howardi*	MYMCA265-11	JX835917

Sarcoptiformes	Achipteriidae	*Anachipteria howardi*	MYMCA266-11	JX838407
Sarcoptiformes	Achipteriidae	*Anachipteria howardi*	MYMCA267-11	JX837618
Sarcoptiformes	Achipteriidae	*Anachipteria howardi*	MYMCB070-11	JX836989
Sarcoptiformes	Achipteriidae	*Anachipteria howardi*	MYMCC006-11	JX834149
Sarcoptiformes	Achipteriidae	*Anachipteria howardi*	MYMCC007-11	JX835260
Sarcoptiformes	Achipteriidae	*Anachipteria howardi*	MYMCC041-11	JX834321
Sarcoptiformes	Achipteriidae	*Anachipteria howardi*	MYMCC300-11	JX834785
Sarcoptiformes	Achipteriidae	*Anachipteria howardi*	MYMCC800-11	JX837554
Sarcoptiformes	Achipteriidae	*Anachipteria howardi*	MYMCE095-12	JX838567
Sarcoptiformes	Achipteriidae	*Anachipteria howardi*	MYMCF693-12	JX835635
Sarcoptiformes	Achipteriidae	*Anachipteria howardi*	MYMCG258-12	JX837274
Sarcoptiformes	Achipteriidae	*Anachipteria howardi*	MYMCG463-12	JX833912
Sarcoptiformes	Achipteriidae	*Protoribates*	SSJAB2153-13	KM833691
Sarcoptiformes	Achipteriidae	*Protoribates*	SSJAB2155-13	KM830330
Sarcoptiformes	Achipteriidae	*Protoribates*	SSPAA6652-13	KM840163
Sarcoptiformes	Achipteriidae	*Protoribates*	SSPAA6655-13	KM824577
Sarcoptiformes	Achipteriidae	*Protoribates*	SSPAA6656-13	KM838075
Sarcoptiformes	Achipteriidae	*Protoribates*	SSPAA6657-13	KM827037
Sarcoptiformes	Achipteriidae	*Protoribates*	SSPAA6662-13	KM835951
Sarcoptiformes	Achipteriidae	*Protoribates*	SSPAA6685-13	KM828466
Sarcoptiformes	Achipteriidae	*Protoribates*	SSPAA6700-13	KM840683
Sarcoptiformes	Achipteriidae	*Protoribates*	SSPAA6704-13	KM830499
Sarcoptiformes	Achipteriidae	*Protoribates*	SSPAA7758-13	KM832194
Sarcoptiformes	Achipteriidae	*Protoribates*	SSPAA7763-13	KM838646
Sarcoptiformes	Achipteriidae	*Protoribates*	SSPAA7770-13	KM825790
Sarcoptiformes	Achipteriidae	*Protoribates*	SSPAA7950-13	KM835394
Sarcoptiformes	Achipteriidae		CNPCM142-13	KM824103
Sarcoptiformes	Achipteriidae		SSBAA2084-12	KM829962
Sarcoptiformes	Achipteriidae		SSBAC4241-12	KM825893
Sarcoptiformes	Achipteriidae		SSJAB1896-13	KM831620
Sarcoptiformes	Achipteriidae		SSPAA7778-13	KM837345
Sarcoptiformes	Alicorhagiidae		CHACB1166-10	HM907245
Sarcoptiformes	Alicorhagiidae		CHACB406-10	HQ558568
Sarcoptiformes	Alicorhagiidae		CHACB407-10	JX835661
Sarcoptiformes	Alicorhagiidae		CHACB693-10	JX837959
Sarcoptiformes	Alicorhagiidae		CHACB741-10	JX837515
Sarcoptiformes	Alicorhagiidae		CHACB742-10	HQ558781
Sarcoptiformes	Alicorhagiidae		CHACB743-10	JX838570
Sarcoptiformes	Alicorhagiidae		CHACB942-10	JX835618
Sarcoptiformes	Alicorhagiidae		CHACB966-10	JX835328
Sarcoptiformes	Alicorhagiidae		MYMCA885-11	JX838240
Sarcoptiformes	Alicorhagiidae		MYMCB435-11	JX836551
Sarcoptiformes	Alicorhagiidae		MYMCB436-11	JX836401
Sarcoptiformes	Alicorhagiidae		MYMCE475-12	JX835745
Sarcoptiformes	Alicorhagiidae		MYMCF657-12	JX834616
Sarcoptiformes	Alicorhagiidae		MYMCF733-12	JX838450
Sarcoptiformes	Alicorhagiidae		MYMCF852-12	JX838512
Sarcoptiformes	Alloptidae	*Alloptes sp.*	GBA14787-14	KF018834
Sarcoptiformes	Alloptidae	*Alloptes sp.*	GBA14788-14	KF018835
Sarcoptiformes	Alloptidae	*Alloptes stercorarii*	GBA14816-14	KF018829
Sarcoptiformes	Alloptidae	*Alloptes stercorarii*	GBA14817-14	KF018830
Sarcoptiformes	Alloptidae	*Alloptes stercorarii*	GBA14818-14	KF018831
Sarcoptiformes	Alloptidae	*Alloptes stercorarii*	GBA14819-14	KF018832

Sarcoptiformes	Alloptidae	*Alloptes stercorarii*	GBA14820-14	KF018833
Sarcoptiformes	Alloptidae	*Alloptes stercorarii*	GBA14821-14	KF018859
Sarcoptiformes	Alloptidae	*Alloptes stercorarii*	GBA14822-14	KF018860
Sarcoptiformes	Alycidae		CHACA021-08	JX835031
Sarcoptiformes	Alycidae		CHACA978-10	HM405842
Sarcoptiformes	Alycidae		CHACA979-10	HM405843
Sarcoptiformes	Alycidae		CHACA980-10	HM405844
Sarcoptiformes	Alycidae		CHACA981-10	HM405845
Sarcoptiformes	Alycidae		CHACA982-10	HM405846
Sarcoptiformes	Alycidae		CHACB093-10	HQ558392
Sarcoptiformes	Alycidae		CHACB1043-10	HM907172
Sarcoptiformes	Alycidae		CHACB1167-10	HM907246
Sarcoptiformes	Alycidae		CHACB133-10	HQ558417
Sarcoptiformes	Alycidae		CHACB134-10	HQ558418
Sarcoptiformes	Alycidae		CHACB474-10	HQ558613
Sarcoptiformes	Alycidae		MYMCA051-11	JX835487
Sarcoptiformes	Alycidae		MYMCA052-11	JX837717
Sarcoptiformes	Alycidae		MYMCA053-11	JX834115
Sarcoptiformes	Alycidae		MYMCA1120-11	JX838340
Sarcoptiformes	Alycidae		MYMCA1430-11	JX837472
Sarcoptiformes	Alycidae		MYMCA524-11	JX833821
Sarcoptiformes	Alycidae		MYMCA666-11	JX835428
Sarcoptiformes	Alycidae		MYMCA951-11	JX836305
Sarcoptiformes	Alycidae		MYMCA952-11	JX834738
Sarcoptiformes	Alycidae		MYMCB432-11	JX834204
Sarcoptiformes	Alycidae		MYMCB433-11	JX835472
Sarcoptiformes	Alycidae		MYMCB902-11	JX835176
Sarcoptiformes	Alycidae		MYMCC580-11	JX836398
Sarcoptiformes	Alycidae		MYMCE080-12	JX834697
Sarcoptiformes	Alycidae		MYMCE543-12	JX836757
Sarcoptiformes	Alycidae		MYMCE856-12	JX837241
Sarcoptiformes	Alycidae		MYMCE928-12	JX833658
Sarcoptiformes	Alycidae		MYMCE929-12	JX836379
Sarcoptiformes	Alycidae		MYMCF085-12	JX834639
Sarcoptiformes	Alycidae		MYMCF086-12	JX838562
Sarcoptiformes	Alycidae		MYMCF624-12	JX837351
Sarcoptiformes	Alycidae		MYMCG294-12	JX836099
Sarcoptiformes	Alycidae		MYMCG295-12	JX834430
Sarcoptiformes	Alycidae		MYMCG296-12	JX838184
Sarcoptiformes	Alycidae		MYMCG410-12	JX834132
Sarcoptiformes	Alycidae		SSEIB8175-13	KM831660
Sarcoptiformes	Alycidae		SSWLC4144-13	KM838688
Sarcoptiformes	Ameronothridae	*Ameronothrus maculatus*	CHACA008-08	JX837164
Sarcoptiformes	Ameronothridae	*Ameronothrus maculatus*	CHACA009-08	JX834891
Sarcoptiformes	Ameronothridae	*Ameronothrus maculatus*	CHACA010-08	JX834181
Sarcoptiformes	Ameronothridae	*Ameronothrus maculatus*	CHACA011-08	JX837117
Sarcoptiformes	Ameronothridae	*Ameronothrus maculatus*	CHACA012-08	JX833735
Sarcoptiformes	Ameronothridae	*Ameronothrus maculatus*	CHACA013-08	JX836001
Sarcoptiformes	Ameronothridae	*Ameronothrus maculatus*	CHACA014-08	JX836990
Sarcoptiformes	Ameronothridae	*Ameronothrus maculatus*	CHACA015-08	JX836296
Sarcoptiformes	Ameronothridae	*Ameronothrus maculatus*	CHACA093-08	JX836689
Sarcoptiformes	Ameronothridae	*Ameronothrus maculatus*	CHACA987-10	HM405849
Sarcoptiformes	Ameronothridae	*Ameronothrus maculatus*	CHACB672-10	HQ558740

Sarcoptiformes	Ameronothridae	*Ameronothrus maculatus*	CHACB674-10	HQ558742
Sarcoptiformes	Ameronothridae	*Ameronothrus maculatus*	CHACB675-10	HQ558743
Sarcoptiformes	Ameronothridae	*Ameronothrus maculatus*	CHACB676-10	JX838639
Sarcoptiformes	Ameronothridae	*Ameronothrus maculatus*	CHACC080-10	HM907473
Sarcoptiformes	Ameronothridae	*Ameronothrus maculatus*	CHACC081-10	HM907474
Sarcoptiformes	Ameronothridae	*Ameronothrus maculatus*	CHACC082-10	HM907475
Sarcoptiformes	Ameronothridae	*Ameronothrus maculatus*	CHACC083-10	HM907476
Sarcoptiformes	Ameronothridae	*Ameronothrus maculatus*	CHACC084-10	HM907477
Sarcoptiformes	Ameronothridae	*Ameronothrus maculatus*	CHACC085-10	HM907478
Sarcoptiformes	Ameronothridae	*Ameronothrus maculatus*	CHACC254-10	HQ941558
Sarcoptiformes	Ameronothridae	*Ameronothrus maculatus*	CHACC255-10	HQ941559
Sarcoptiformes	Ameronothridae	*Ameronothrus maculatus*	CHACC256-10	HQ941560
Sarcoptiformes	Ameronothridae	*Ameronothrus maculatus*	CHACC257-10	HQ941561
Sarcoptiformes	Ameronothridae	*Ameronothrus maculatus*	CHACC258-10	HQ941562
Sarcoptiformes	Ameronothridae	*Ameronothrus maculatus*	CHACC259-10	HQ941563
Sarcoptiformes	Ameronothridae	*Ameronothrus maculatus*	MYMCC125-11	JX835812
Sarcoptiformes	Ameronothridae	*Ameronothrus maculatus*	SSBAD4029-12	KM838799
Sarcoptiformes	Ameronothridae	*Ameronothrus nigrofemoratus*	CHACA088-08	JX835019
Sarcoptiformes	Ameronothridae	*Ameronothrus nigrofemoratus*	CHACA089-08	JX836478
Sarcoptiformes	Ameronothridae	*Ameronothrus nigrofemoratus*	CHACA090-08	JX836451
Sarcoptiformes	Ameronothridae	*Ameronothrus nigrofemoratus*	CHACA091-08	JX837813
Sarcoptiformes	Ameronothridae	*Ameronothrus nigrofemoratus*	CHACA134-08	JX836498
Sarcoptiformes	Ameronothridae	*Ameronothrus nigrofemoratus*	CHACA138-08	JX833692
Sarcoptiformes	Ameronothridae	*Ameronothrus nigrofemoratus*	CHACA214-08	JX837098
Sarcoptiformes	Ameronothridae	*Ameronothrus nigrofemoratus*	CHACA215-08	JX836006
Sarcoptiformes	Ameronothridae	*Ameronothrus nigrofemoratus*	CHACA475-09	JX836609
Sarcoptiformes	Ameronothridae	*Ameronothrus nigrofemoratus*	CHACA522-09	JX834650
Sarcoptiformes	Ameronothridae	*Ameronothrus nigrofemoratus*	CHACA526-09	JX834611
Sarcoptiformes	Ameronothridae	*Ameronothrus nigrofemoratus*	CHACB342-10	HQ558524
Sarcoptiformes	Ameronothridae	*Ameronothrus nigrofemoratus*	CHACB344-10	HQ558525
Sarcoptiformes	Ameronothridae	*Ameronothrus nigrofemoratus*	CHACB345-10	HQ558526
Sarcoptiformes	Analgidae	*Analges corvinus*	GBCH4464-10	GQ864337
Sarcoptiformes	Analgidae	*Analges sturninus*	GBCH4460-10	GQ864342
Sarcoptiformes	Analgidae	*Anhemialges longipes*	GBCH4461-10	GQ864340
Sarcoptiformes	Analgidae	*Mesalgoides*	FMEP314-14	KP193470
Sarcoptiformes	Analgidae	*Mesalgoides*	FMEP333-14	KP193471
Sarcoptiformes	Analgidae	*Mesalgoides megnini*	FMEP279-14	KP193472
Sarcoptiformes	Analgidae	*Mesalgoides megnini*	FMEP280-14	KP193474
Sarcoptiformes	Analgidae	*Mesalgoides megnini*	FMEP281-14	KP193473
Sarcoptiformes	Astegistidae	*Cultroribula*	MYMCA233-11	JX836775
Sarcoptiformes	Astegistidae	*Cultroribula*	MYMCC874-11	JX834473
Sarcoptiformes	Astegistidae	*Cultroribula*	MYMCC875-11	JX837143
Sarcoptiformes	Astegistidae	*Cultroribula*	MYMCF302-12	JX837891
Sarcoptiformes	Astegistidae	*Cultroribula*	MYMCF303-12	JX835755
Sarcoptiformes	Astegistidae	*Cultroribula*	MYMCF806-12	JX838343
Sarcoptiformes	Astegistidae	*Cultroribula*	MYMCF807-12	JX837037
Sarcoptiformes	Avenzoariidae	*Avenzoaria totani*	GBCH4454-10	GQ864348
Sarcoptiformes	Avenzoariidae	*Pomeranzevia ninnii*	GBCH4443-10	GQ864359
Sarcoptiformes	Avenzoariidae	*Promegninia bulweriae*	GBA22646-15	KM401844
Sarcoptiformes	Avenzoariidae	*Promegninia calonectris*	GBA22641-15	KM401839
Sarcoptiformes	Avenzoariidae	*Promegninia calonectris*	GBA22642-15	KM401840
Sarcoptiformes	Avenzoariidae	*Promegninia calonectris*	GBA22643-15	KM401841
Sarcoptiformes	Avenzoariidae	*Promegninia calonectris*	GBA22644-15	KM401842

Sarcoptiformes	Avenzoariidae	*Promegninia calonectris*	GBA22645-15	KM401843
Sarcoptiformes	Avenzoariidae	*Rhinozachvatkinia sp. LS2013*	GBA17826-14	KF111269
Sarcoptiformes	Avenzoariidae	*Rhinozachvatkinia sp. LS2013*	GBA17827-14	KF111270
Sarcoptiformes	Avenzoariidae	*Rhinozachvatkinia sp. LS2013*	GBA17828-14	KF111271
Sarcoptiformes	Avenzoariidae	*Rhinozachvatkinia sp. LS2013*	GBA17829-14	KF111272
Sarcoptiformes	Avenzoariidae	*Rhinozachvatkinia sp. LS2013*	GBA17830-14	KF111273
Sarcoptiformes	Avenzoariidae	*Rhinozachvatkinia sp. LS2013*	GBA17831-14	KF111274
Sarcoptiformes	Avenzoariidae	*Zachvatkinia isolata*	GBA14789-14	KF018823
Sarcoptiformes	Avenzoariidae	*Zachvatkinia isolata*	GBA14790-14	KF018824
Sarcoptiformes	Avenzoariidae	*Zachvatkinia isolata*	GBA14791-14	KF018825
Sarcoptiformes	Avenzoariidae	*Zachvatkinia isolata*	GBA14792-14	KF018826
Sarcoptiformes	Avenzoariidae	*Zachvatkinia isolata*	GBA14793-14	KF018836
Sarcoptiformes	Avenzoariidae	*Zachvatkinia isolata*	GBA14794-14	KF018837
Sarcoptiformes	Avenzoariidae	*Zachvatkinia isolata*	GBA14795-14	KF018838
Sarcoptiformes	Avenzoariidae	*Zachvatkinia isolata*	GBA14796-14	KF018839
Sarcoptiformes	Avenzoariidae	*Zachvatkinia isolata*	GBA14797-14	KF018840
Sarcoptiformes	Avenzoariidae	*Zachvatkinia isolata*	GBA14798-14	KF018841
Sarcoptiformes	Avenzoariidae	*Zachvatkinia isolata*	GBA14799-14	KF018842
Sarcoptiformes	Avenzoariidae	*Zachvatkinia isolata*	GBA14800-14	KF018843
Sarcoptiformes	Avenzoariidae	*Zachvatkinia isolata*	GBA14801-14	KF018844
Sarcoptiformes	Avenzoariidae	*Zachvatkinia isolata*	GBA14802-14	KF018845
Sarcoptiformes	Avenzoariidae	*Zachvatkinia isolata*	GBA14803-14	KF018846
Sarcoptiformes	Avenzoariidae	*Zachvatkinia isolata*	GBA14804-14	KF018847
Sarcoptiformes	Avenzoariidae	*Zachvatkinia isolata*	GBA14805-14	KF018848
Sarcoptiformes	Avenzoariidae	*Zachvatkinia isolata*	GBA14806-14	KF018849
Sarcoptiformes	Avenzoariidae	*Zachvatkinia isolata*	GBA14807-14	KF018850
Sarcoptiformes	Avenzoariidae	*Zachvatkinia isolata*	GBA14808-14	KF018851
Sarcoptiformes	Avenzoariidae	*Zachvatkinia isolata*	GBA14809-14	KF018852
Sarcoptiformes	Avenzoariidae	*Zachvatkinia isolata*	GBA14810-14	KF018853
Sarcoptiformes	Avenzoariidae	*Zachvatkinia isolata*	GBA14811-14	KF018854
Sarcoptiformes	Avenzoariidae	*Zachvatkinia isolata*	GBA14812-14	KF018855
Sarcoptiformes	Avenzoariidae	*Zachvatkinia isolata*	GBA14813-14	KF018856
Sarcoptiformes	Avenzoariidae	*Zachvatkinia isolata*	GBA14814-14	KF018857
Sarcoptiformes	Avenzoariidae	*Zachvatkinia isolata*	GBA14815-14	KF018858
Sarcoptiformes	Avenzoariidae	*Zachvatkinia isolata*	GBA14823-14	KF018827
Sarcoptiformes	Avenzoariidae	*Zachvatkinia isolata*	GBA14824-14	KF018828
Sarcoptiformes	Avenzoariidae	*Zachvatkinia isolata*	GBA14825-14	KF018861
Sarcoptiformes	Avenzoariidae	*Zachvatkinia isolata*	GBA14826-14	KF018862
Sarcoptiformes	Avenzoariidae	*Zachvatkinia isolata*	GBA14827-14	KF018863
Sarcoptiformes	Avenzoariidae	*Zachvatkinia isolata*	GBA14828-14	KF018864
Sarcoptiformes	Avenzoariidae	*Zachvatkinia isolata*	GBA14829-14	KF018865
Sarcoptiformes	Avenzoariidae	*Zachvatkinia isolata*	GBA14830-14	KF018866
Sarcoptiformes	Avenzoariidae	*Zachvatkinia isolata*	GBA14831-14	KF018867
Sarcoptiformes	Avenzoariidae	*Zachvatkinia isolata*	GBA14832-14	KF018868
Sarcoptiformes	Avenzoariidae	*Zachvatkinia isolata*	GBA14833-14	KF018869
Sarcoptiformes	Avenzoariidae	*Zachvatkinia isolata*	GBA14834-14	KF018870
Sarcoptiformes	Avenzoariidae	*Zachvatkinia isolata*	GBA14835-14	KF018871
Sarcoptiformes	Avenzoariidae	*Zachvatkinia isolata*	GBA14836-14	KF018872
Sarcoptiformes	Avenzoariidae	*Zachvatkinia isolata*	GBA14837-14	KF018873
Sarcoptiformes	Avenzoariidae	*Zachvatkinia isolata*	GBA14838-14	KF018874
Sarcoptiformes	Avenzoariidae	*Zachvatkinia isolata*	GBA14839-14	KF018875
Sarcoptiformes	Avenzoariidae	*Zachvatkinia isolata*	GBA14840-14	KF018876
Sarcoptiformes	Avenzoariidae	*Zachvatkinia isolata*	GBA14841-14	KF018877

Sarcoptiformes	Avenzoariidae	*Zachvatkinia isolata*	GBA14842-14	KF018879
Sarcoptiformes	Avenzoariidae	*Zachvatkinia isolata*	GBA14843-14	KF018880
Sarcoptiformes	Avenzoariidae	*Zachvatkinia isolata*	GBA14844-14	KF018881
Sarcoptiformes	Avenzoariidae	*Zachvatkinia isolata*	GBA14845-14	KF018882
Sarcoptiformes	Avenzoariidae	*Zachvatkinia isolata*	GBA14846-14	KF018883
Sarcoptiformes	Avenzoariidae	*Zachvatkinia isolata*	GBA14847-14	KF018884
Sarcoptiformes	Avenzoariidae	*Zachvatkinia isolata*	GBA14848-14	KF018885
Sarcoptiformes	Avenzoariidae	*Zachvatkinia isolata*	GBA14849-14	KF018886
Sarcoptiformes	Avenzoariidae	*Zachvatkinia isolata*	GBA14850-14	KF018887
Sarcoptiformes	Avenzoariidae	*Zachvatkinia isolata*	GBA14851-14	KF018888
Sarcoptiformes	Avenzoariidae	*Zachvatkinia isolata*	GBA14852-14	KF018889
Sarcoptiformes	Avenzoariidae	*Zachvatkinia isolata*	GBA14853-14	KF018890
Sarcoptiformes	Avenzoariidae	*Zachvatkinia isolata*	GBA14854-14	KF018891
Sarcoptiformes	Avenzoariidae	*Zachvatkinia isolata*	GBA14855-14	KF018892
Sarcoptiformes	Avenzoariidae	*Zachvatkinia isolata*	GBA14856-14	KF018893
Sarcoptiformes	Avenzoariidae	*Zachvatkinia isolata*	GBA14857-14	KF018894
Sarcoptiformes	Avenzoariidae	*Zachvatkinia isolata*	GBA14858-14	KF018895
Sarcoptiformes	Avenzoariidae	*Zachvatkinia isolata*	GBA14859-14	KF018896
Sarcoptiformes	Avenzoariidae	*Zachvatkinia isolata*	GBA14860-14	KF018897
Sarcoptiformes	Avenzoariidae	*Zachvatkinia isolata*	GBA14861-14	KF018898
Sarcoptiformes	Avenzoariidae	*Zachvatkinia isolata*	GBA14862-14	KF018899
Sarcoptiformes	Avenzoariidae	*Zachvatkinia isolata*	GBA14863-14	KF018900
Sarcoptiformes	Avenzoariidae	*Zachvatkinia isolata*	GBA14864-14	KF018901
Sarcoptiformes	Avenzoariidae	*Zachvatkinia isolata*	GBA14865-14	KF018902
Sarcoptiformes	Avenzoariidae	*Zachvatkinia isolata*	GBA14866-14	KF018903
Sarcoptiformes	Avenzoariidae	*Zachvatkinia isolata*	GBA14867-14	KF018904
Sarcoptiformes	Avenzoariidae	*Zachvatkinia isolata*	GBA14868-14	KF018905
Sarcoptiformes	Avenzoariidae	*Zachvatkinia isolata*	GBA14869-14	KF018906
Sarcoptiformes	Avenzoariidae	*Zachvatkinia isolata*	GBA14870-14	KF018907
Sarcoptiformes	Avenzoariidae	*Zachvatkinia isolata*	GBA14871-14	KF018908
Sarcoptiformes	Avenzoariidae	*Zachvatkinia isolata*	GBA14872-14	KF018909
Sarcoptiformes	Avenzoariidae	*Zachvatkinia isolata*	GBA14873-14	KF018910
Sarcoptiformes	Avenzoariidae	*Zachvatkinia isolata*	GBA14874-14	KF018911
Sarcoptiformes	Avenzoariidae	*Zachvatkinia stercorarii*	GBA14875-14	KF018820
Sarcoptiformes	Avenzoariidae	*Zachvatkinia stercorarii*	GBA14876-14	KF018821
Sarcoptiformes	Avenzoariidae	*Zachvatkinia stercorarii*	GBA14877-14	KF018822
Sarcoptiformes	Avenzoariidae	*Zachvatkinia stercorarii*	GBA14878-14	KF018878
Sarcoptiformes	Brachychthoniidae		CHACB087-10	JX834282
Sarcoptiformes	Brachychthoniidae		CHACB1048-10	HM907173
Sarcoptiformes	Brachychthoniidae		CHACB1049-10	HM907174
Sarcoptiformes	Brachychthoniidae		CHACB1050-10	HM907175
Sarcoptiformes	Brachychthoniidae		CHACB1051-10	HM907176
Sarcoptiformes	Brachychthoniidae		CHACB1052-10	HM907177
Sarcoptiformes	Brachychthoniidae		CHACB1209-10	HM907278
Sarcoptiformes	Brachychthoniidae		CHACB173-10	HQ558453
Sarcoptiformes	Brachychthoniidae		CHACB408-10	HQ558569
Sarcoptiformes	Brachychthoniidae		CHACB415-10	HQ558573
Sarcoptiformes	Brachychthoniidae		CHACB447-10	HQ558600
Sarcoptiformes	Brachychthoniidae		CHACB448-10	HQ558601
Sarcoptiformes	Brachychthoniidae		CHACB449-10	HQ558602
Sarcoptiformes	Brachychthoniidae		CHACB450-10	HQ558603
Sarcoptiformes	Brachychthoniidae		CHACB451-10	HQ558604
Sarcoptiformes	Brachychthoniidae		CHACB508-10	HQ558636

Sarcoptiformes	Brachychthoniidae	CHACB509-10	HQ558637
Sarcoptiformes	Brachychthoniidae	CHACB586-10	HQ558687
Sarcoptiformes	Brachychthoniidae	CHACB587-10	JX835593
Sarcoptiformes	Brachychthoniidae	CHACB588-10	HQ558688
Sarcoptiformes	Brachychthoniidae	CHACB823-10	HQ941497
Sarcoptiformes	Brachychthoniidae	CHACB883-10	HM907326
Sarcoptiformes	Brachychthoniidae	CHACB884-10	HM907327
Sarcoptiformes	Brachychthoniidae	CHACB946-10	HM907367
Sarcoptiformes	Brachychthoniidae	CHACB947-10	HM907368
Sarcoptiformes	Brachychthoniidae	CHACC066-10	HM907463
Sarcoptiformes	Brachychthoniidae	CHACC142-10	JX837909
Sarcoptiformes	Brachychthoniidae	CHACC143-10	JX837453
Sarcoptiformes	Brachychthoniidae	CHACC212-10	HQ941532
Sarcoptiformes	Brachychthoniidae	MYMCA1073-11	JX834717
Sarcoptiformes	Brachychthoniidae	MYMCA1074-11	JX836290
Sarcoptiformes	Brachychthoniidae	MYMCA1075-11	JX837511
Sarcoptiformes	Brachychthoniidae	MYMCA1096-11	JX836049
Sarcoptiformes	Brachychthoniidae	MYMCA1097-11	JX836378
Sarcoptiformes	Brachychthoniidae	MYMCA1099-11	JX834578
Sarcoptiformes	Brachychthoniidae	MYMCA1110-11	JX838662
Sarcoptiformes	Brachychthoniidae	MYMCA1111-11	JX836328
Sarcoptiformes	Brachychthoniidae	MYMCA1112-11	JX837490
Sarcoptiformes	Brachychthoniidae	MYMCA1151-11	JX838530
Sarcoptiformes	Brachychthoniidae	MYMCA1152-11	JX835584
Sarcoptiformes	Brachychthoniidae	MYMCA1153-11	JX834444
Sarcoptiformes	Brachychthoniidae	MYMCA1155-11	JX834947
Sarcoptiformes	Brachychthoniidae	MYMCA1156-11	JX833641
Sarcoptiformes	Brachychthoniidae	MYMCA1185-11	JX838594
Sarcoptiformes	Brachychthoniidae	MYMCA1186-11	JX836613
Sarcoptiformes	Brachychthoniidae	MYMCA1188-11	JX835620
Sarcoptiformes	Brachychthoniidae	MYMCA1189-11	JX836257
Sarcoptiformes	Brachychthoniidae	MYMCA1459-11	JX835132
Sarcoptiformes	Brachychthoniidae	MYMCA1460-11	JX834856
Sarcoptiformes	Brachychthoniidae	MYMCA232-11	JX833786
Sarcoptiformes	Brachychthoniidae	MYMCA340-11	JX837004
Sarcoptiformes	Brachychthoniidae	MYMCA382-11	JX836446
Sarcoptiformes	Brachychthoniidae	MYMCA421-11	JX838756
Sarcoptiformes	Brachychthoniidae	MYMCA440-11	JX835188
Sarcoptiformes	Brachychthoniidae	MYMCA441-11	JX837002
Sarcoptiformes	Brachychthoniidae	MYMCA442-11	JX833628
Sarcoptiformes	Brachychthoniidae	MYMCA532-11	JX837650
Sarcoptiformes	Brachychthoniidae	MYMCA533-11	JX836534
Sarcoptiformes	Brachychthoniidae	MYMCA535-11	JX836297
Sarcoptiformes	Brachychthoniidae	MYMCA536-11	JX836275
Sarcoptiformes	Brachychthoniidae	MYMCA559-11	JX833933
Sarcoptiformes	Brachychthoniidae	MYMCA580-11	JX837307
Sarcoptiformes	Brachychthoniidae	MYMCA581-11	JX834577
Sarcoptiformes	Brachychthoniidae	MYMCA761-11	JX837540
Sarcoptiformes	Brachychthoniidae	MYMCA762-11	JX836214
Sarcoptiformes	Brachychthoniidae	MYMCA763-11	JX837932
Sarcoptiformes	Brachychthoniidae	MYMCA764-11	JX837327
Sarcoptiformes	Brachychthoniidae	MYMCA781-11	JX836929
Sarcoptiformes	Brachychthoniidae	MYMCA802-11	JX837592

Sarcoptiformes	Brachychthoniidae	MYMCA803-11	JX836611
Sarcoptiformes	Brachychthoniidae	MYMCA822-11	JX833793
Sarcoptiformes	Brachychthoniidae	MYMCA824-11	JX834389
Sarcoptiformes	Brachychthoniidae	MYMCA825-11	JX837113
Sarcoptiformes	Brachychthoniidae	MYMCA831-11	JX837521
Sarcoptiformes	Brachychthoniidae	MYMCA833-11	JX835545
Sarcoptiformes	Brachychthoniidae	MYMCA882-11	JX834596
Sarcoptiformes	Brachychthoniidae	MYMCA883-11	JX834225
Sarcoptiformes	Brachychthoniidae	MYMCA884-11	JX837370
Sarcoptiformes	Brachychthoniidae	MYMCA918-11	JX836044
Sarcoptiformes	Brachychthoniidae	MYMCA919-11	JX838329
Sarcoptiformes	Brachychthoniidae	MYMCA920-11	JX836796
Sarcoptiformes	Brachychthoniidae	MYMCA921-11	JX837653
Sarcoptiformes	Brachychthoniidae	MYMCA945-11	JX838299
Sarcoptiformes	Brachychthoniidae	MYMCA946-11	JX835969
Sarcoptiformes	Brachychthoniidae	MYMCA948-11	JX836255
Sarcoptiformes	Brachychthoniidae	MYMCA949-11	JX835934
Sarcoptiformes	Brachychthoniidae	MYMCA978-11	JX836191
Sarcoptiformes	Brachychthoniidae	MYMCA979-11	JX838074
Sarcoptiformes	Brachychthoniidae	MYMCB052-11	JX834941
Sarcoptiformes	Brachychthoniidae	MYMCB053-11	JX837192
Sarcoptiformes	Brachychthoniidae	MYMCB102-11	JX837593
Sarcoptiformes	Brachychthoniidae	MYMCB136-11	JX837888
Sarcoptiformes	Brachychthoniidae	MYMCB137-11	JX837437
Sarcoptiformes	Brachychthoniidae	MYMCB138-11	JX834095
Sarcoptiformes	Brachychthoniidae	MYMCB172-11	JX838115
Sarcoptiformes	Brachychthoniidae	MYMCB173-11	JX837028
Sarcoptiformes	Brachychthoniidae	MYMCB174-11	JX835436
Sarcoptiformes	Brachychthoniidae	MYMCB175-11	JX833937
Sarcoptiformes	Brachychthoniidae	MYMCB235-11	JX835828
Sarcoptiformes	Brachychthoniidae	MYMCB437-11	JX838090
Sarcoptiformes	Brachychthoniidae	MYMCB438-11	JX836269
Sarcoptiformes	Brachychthoniidae	MYMCB503-11	JX834377
Sarcoptiformes	Brachychthoniidae	MYMCB759-11	JX836150
Sarcoptiformes	Brachychthoniidae	MYMCB792-11	JX836131
Sarcoptiformes	Brachychthoniidae	MYMCB793-11	JX837424
Sarcoptiformes	Brachychthoniidae	MYMCB857-11	JX835908
Sarcoptiformes	Brachychthoniidae	MYMCB858-11	JX838056
Sarcoptiformes	Brachychthoniidae	MYMCB860-11	JX835577
Sarcoptiformes	Brachychthoniidae	MYMCC069-11	JX836059
Sarcoptiformes	Brachychthoniidae	MYMCC070-11	JX836538
Sarcoptiformes	Brachychthoniidae	MYMCC266-11	JX838060
Sarcoptiformes	Brachychthoniidae	MYMCC277-11	JX835970
Sarcoptiformes	Brachychthoniidae	MYMCC278-11	JX837825
Sarcoptiformes	Brachychthoniidae	MYMCC279-11	JX838158
Sarcoptiformes	Brachychthoniidae	MYMCC283-11	JX836418
Sarcoptiformes	Brachychthoniidae	MYMCC373-11	JX836738
Sarcoptiformes	Brachychthoniidae	MYMCC445-11	JX835795
Sarcoptiformes	Brachychthoniidae	MYMCC446-11	JX838586
Sarcoptiformes	Brachychthoniidae	MYMCC605-11	JX837795
Sarcoptiformes	Brachychthoniidae	MYMCC621-11	JX838712
Sarcoptiformes	Brachychthoniidae	MYMCC647-11	JX836322
Sarcoptiformes	Brachychthoniidae	MYMCC844-11	JX834520

Sarcoptiformes	Brachychthoniidae	MYMCC884-11	JX836061
Sarcoptiformes	Brachychthoniidae	MYMCC913-11	JX834761
Sarcoptiformes	Brachychthoniidae	MYMCC935-11	JX835936
Sarcoptiformes	Brachychthoniidae	MYMCC937-11	JX836865
Sarcoptiformes	Brachychthoniidae	MYMCC938-11	JX834934
Sarcoptiformes	Brachychthoniidae	MYMCE074-12	JX836977
Sarcoptiformes	Brachychthoniidae	MYMCE099-12	JX837604
Sarcoptiformes	Brachychthoniidae	MYMCE101-12	JX837426
Sarcoptiformes	Brachychthoniidae	MYMCE227-12	JX837833
Sarcoptiformes	Brachychthoniidae	MYMCE326-12	JX838386
Sarcoptiformes	Brachychthoniidae	MYMCE461-12	JX836111
Sarcoptiformes	Brachychthoniidae	MYMCE602-12	JX835346
Sarcoptiformes	Brachychthoniidae	MYMCE604-12	JX838327
Sarcoptiformes	Brachychthoniidae	MYMCE606-12	JX834208
Sarcoptiformes	Brachychthoniidae	MYMCE660-12	JX834261
Sarcoptiformes	Brachychthoniidae	MYMCE671-12	JX836531
Sarcoptiformes	Brachychthoniidae	MYMCE672-12	JX838779
Sarcoptiformes	Brachychthoniidae	MYMCE673-12	JX835050
Sarcoptiformes	Brachychthoniidae	MYMCE706-12	JX835675
Sarcoptiformes	Brachychthoniidae	MYMCE707-12	JX837673
Sarcoptiformes	Brachychthoniidae	MYMCE764-12	JX834468
Sarcoptiformes	Brachychthoniidae	MYMCE765-12	JX835751
Sarcoptiformes	Brachychthoniidae	MYMCE766-12	JX834607
Sarcoptiformes	Brachychthoniidae	MYMCE767-12	JX838695
Sarcoptiformes	Brachychthoniidae	MYMCE793-12	JX833635
Sarcoptiformes	Brachychthoniidae	MYMCE794-12	JX838269
Sarcoptiformes	Brachychthoniidae	MYMCE859-12	JX835868
Sarcoptiformes	Brachychthoniidae	MYMCE860-12	JX834434
Sarcoptiformes	Brachychthoniidae	MYMCE861-12	JX838348
Sarcoptiformes	Brachychthoniidae	MYMCE880-12	JX838716
Sarcoptiformes	Brachychthoniidae	MYMCE881-12	JX837263
Sarcoptiformes	Brachychthoniidae	MYMCE925-12	JX837811
Sarcoptiformes	Brachychthoniidae	MYMCE926-12	JX837217
Sarcoptiformes	Brachychthoniidae	MYMCE927-12	JX838228
Sarcoptiformes	Brachychthoniidae	MYMCF014-12	JX833757
Sarcoptiformes	Brachychthoniidae	MYMCF087-12	JX835892
Sarcoptiformes	Brachychthoniidae	MYMCF143-12	JX836979
Sarcoptiformes	Brachychthoniidae	MYMCF167-12	JX836262
Sarcoptiformes	Brachychthoniidae	MYMCF208-12	JX834739
Sarcoptiformes	Brachychthoniidae	MYMCF246-12	JX835872
Sarcoptiformes	Brachychthoniidae	MYMCF247-12	JX835054
Sarcoptiformes	Brachychthoniidae	MYMCF248-12	JX835955
Sarcoptiformes	Brachychthoniidae	MYMCF249-12	JX836443
Sarcoptiformes	Brachychthoniidae	MYMCF344-12	JX835849
Sarcoptiformes	Brachychthoniidae	MYMCF364-12	JX835302
Sarcoptiformes	Brachychthoniidae	MYMCF380-12	JX838745
Sarcoptiformes	Brachychthoniidae	MYMCF381-12	JX837315
Sarcoptiformes	Brachychthoniidae	MYMCF397-12	JX833857
Sarcoptiformes	Brachychthoniidae	MYMCF398-12	JX836075
Sarcoptiformes	Brachychthoniidae	MYMCF399-12	JX834323
Sarcoptiformes	Brachychthoniidae	MYMCF430-12	JX837930
Sarcoptiformes	Brachychthoniidae	MYMCF431-12	JX838058
Sarcoptiformes	Brachychthoniidae	MYMCF472-12	JX837089

Sarcoptiformes	Brachychthoniidae	MYMCF512-12	JX837683
Sarcoptiformes	Brachychthoniidae	MYMCF530-12	JX837232
Sarcoptiformes	Brachychthoniidae	MYMCF532-12	JX837444
Sarcoptiformes	Brachychthoniidae	MYMCF533-12	JX837933
Sarcoptiformes	Brachychthoniidae	MYMCF546-12	JX837290
Sarcoptiformes	Brachychthoniidae	MYMCF547-12	JX835802
Sarcoptiformes	Brachychthoniidae	MYMCF551-12	JX835840
Sarcoptiformes	Brachychthoniidae	MYMCF566-12	JX835373
Sarcoptiformes	Brachychthoniidae	MYMCF567-12	JX834134
Sarcoptiformes	Brachychthoniidae	MYMCF569-12	JX837705
Sarcoptiformes	Brachychthoniidae	MYMCF570-12	JX836888
Sarcoptiformes	Brachychthoniidae	MYMCF595-12	JX833683
Sarcoptiformes	Brachychthoniidae	MYMCF627-12	JX838605
Sarcoptiformes	Brachychthoniidae	MYMCF628-12	JX837201
Sarcoptiformes	Brachychthoniidae	MYMCF647-12	JX836776
Sarcoptiformes	Brachychthoniidae	MYMCF648-12	JX833911
Sarcoptiformes	Brachychthoniidae	MYMCF649-12	JX838487
Sarcoptiformes	Brachychthoniidae	MYMCF650-12	JX836861
Sarcoptiformes	Brachychthoniidae	MYMCF668-12	JX836848
Sarcoptiformes	Brachychthoniidae	MYMCF750-12	JX837137
Sarcoptiformes	Brachychthoniidae	MYMCF751-12	JX838542
Sarcoptiformes	Brachychthoniidae	MYMCF770-12	JX836050
Sarcoptiformes	Brachychthoniidae	MYMCF811-12	JX833814
Sarcoptiformes	Brachychthoniidae	MYMCF812-12	JX835530
Sarcoptiformes	Brachychthoniidae	MYMCF813-12	JX838465
Sarcoptiformes	Brachychthoniidae	MYMCF847-12	JX835901
Sarcoptiformes	Brachychthoniidae	MYMCF848-12	JX835226
Sarcoptiformes	Brachychthoniidae	MYMCF897-12	JX837215
Sarcoptiformes	Brachychthoniidae	MYMCF898-12	JX838767
Sarcoptiformes	Brachychthoniidae	MYMCF918-12	JX836477
Sarcoptiformes	Brachychthoniidae	MYMCF919-12	JX838219
Sarcoptiformes	Brachychthoniidae	MYMCF926-12	JX833985
Sarcoptiformes	Brachychthoniidae	MYMCF949-12	JX838447
Sarcoptiformes	Brachychthoniidae	MYMCG016-12	JX833951
Sarcoptiformes	Brachychthoniidae	MYMCG017-12	JX835569
Sarcoptiformes	Brachychthoniidae	MYMCG043-12	JX837835
Sarcoptiformes	Brachychthoniidae	MYMCG045-12	JX835393
Sarcoptiformes	Brachychthoniidae	MYMCG079-12	JX834283
Sarcoptiformes	Brachychthoniidae	MYMCG080-12	JX833756
Sarcoptiformes	Brachychthoniidae	MYMCG081-12	JX835018
Sarcoptiformes	Brachychthoniidae	MYMCG102-12	JX835859
Sarcoptiformes	Brachychthoniidae	MYMCG117-12	JX833914
Sarcoptiformes	Brachychthoniidae	MYMCG187-12	JX837681
Sarcoptiformes	Brachychthoniidae	MYMCG188-12	JX835363
Sarcoptiformes	Brachychthoniidae	MYMCG243-12	JX835374
Sarcoptiformes	Brachychthoniidae	MYMCG244-12	JX836470
Sarcoptiformes	Brachychthoniidae	MYMCG245-12	JX837134
Sarcoptiformes	Brachychthoniidae	MYMCG264-12	JX834253
Sarcoptiformes	Brachychthoniidae	MYMCG266-12	JX838200
Sarcoptiformes	Brachychthoniidae	MYMCG285-12	JX837388
Sarcoptiformes	Brachychthoniidae	MYMCG286-12	JX837344
Sarcoptiformes	Brachychthoniidae	MYMCG287-12	JX838357
Sarcoptiformes	Brachychthoniidae	MYMCG327-12	JX834199

Sarcoptiformes	Brachychthoniidae		MYMCG328-12	JX837780
Sarcoptiformes	Brachychthoniidae		MYMCG329-12	JX834986
Sarcoptiformes	Brachychthoniidae		MYMCG330-12	JX837195
Sarcoptiformes	Brachychthoniidae		MYMCG357-12	JX833845
Sarcoptiformes	Brachychthoniidae		MYMCG384-12	JX837234
Sarcoptiformes	Brachychthoniidae		MYMCG385-12	JX837282
Sarcoptiformes	Brachychthoniidae		MYMCG465-12	JX835819
Sarcoptiformes	Brachychthoniidae		MYMCG468-12	JX838778
Sarcoptiformes	Brachychthoniidae		MYMCG484-12	JX835212
Sarcoptiformes	Brachychthoniidae		MYMCG485-12	JX837807
Sarcoptiformes	Brachychthoniidae		MYMCG518-12	JX835732
Sarcoptiformes	Brachychthoniidae		MYMCG521-12	JX833671
Sarcoptiformes	Brachychthoniidae		MYMCG557-12	JX836790
Sarcoptiformes	Brachychthoniidae		MYMCG612-12	JX836617
Sarcoptiformes	Brachychthoniidae		RBINA5788-13	KP979245
Sarcoptiformes	Brachychthoniidae		SSBAA3697-12	KM827335
Sarcoptiformes	Brachychthoniidae		SSBAA3698-12	KM825887
Sarcoptiformes	Brachychthoniidae		SSBAA3699-12	KM824601
Sarcoptiformes	Brachychthoniidae		SSBAA5053-12	KM833363
Sarcoptiformes	Brachychthoniidae		SSBAB2820-12	KM839289
Sarcoptiformes	Carabodidae	*Carabodes*	SSPAA7999-13	KM836314
Sarcoptiformes	Carabodidae	*Carabodes*	SSPAA8000-13	KM830601
Sarcoptiformes	Carabodidae	*Carabodes labyrinthicus*	CHACB184-10	HQ558456
Sarcoptiformes	Carabodidae	*Carabodes labyrinthicus*	CHACB185-10	HQ558457
Sarcoptiformes	Carabodidae	*Carabodes labyrinthicus*	CHACB921-10	HM907349
Sarcoptiformes	Carabodidae	*Carabodes labyrinthicus*	MYMCA016-11	JX837865
Sarcoptiformes	Carabodidae	*Carabodes labyrinthicus*	MYMCA1128-11	JX834768
Sarcoptiformes	Carabodidae	*Carabodes labyrinthicus*	MYMCA1204-11	JX838701
Sarcoptiformes	Carabodidae	*Carabodes labyrinthicus*	MYMCA1298-11	JX835341
Sarcoptiformes	Carabodidae	*Carabodes labyrinthicus*	MYMCA1325-11	JX835647
Sarcoptiformes	Carabodidae	*Carabodes labyrinthicus*	MYMCA281-11	JX834882
Sarcoptiformes	Carabodidae	*Carabodes labyrinthicus*	MYMCA302-11	JX835081
Sarcoptiformes	Carabodidae	*Carabodes labyrinthicus*	MYMCA303-11	JX835208
Sarcoptiformes	Carabodidae	*Carabodes labyrinthicus*	MYMCA304-11	JX838534
Sarcoptiformes	Carabodidae	*Carabodes labyrinthicus*	MYMCA774-11	JX837330
Sarcoptiformes	Carabodidae	*Carabodes labyrinthicus*	MYMCA791-11	JX834913
Sarcoptiformes	Carabodidae	*Carabodes labyrinthicus*	MYMCA904-11	JX838428
Sarcoptiformes	Carabodidae	*Carabodes labyrinthicus*	MYMCA936-11	JX835995
Sarcoptiformes	Carabodidae	*Carabodes labyrinthicus*	MYMCB063-11	JX835438
Sarcoptiformes	Carabodidae	*Carabodes labyrinthicus*	MYMCB422-11	JX838049
Sarcoptiformes	Carabodidae	*Carabodes labyrinthicus*	MYMCB596-11	JX835598
Sarcoptiformes	Carabodidae	*Carabodes labyrinthicus*	MYMCB645-11	JX834836
Sarcoptiformes	Carabodidae	*Carabodes labyrinthicus*	MYMCB704-11	JX834171
Sarcoptiformes	Carabodidae	*Carabodes labyrinthicus*	MYMCC190-11	JX833997
Sarcoptiformes	Carabodidae	*Carabodes labyrinthicus*	MYMCC460-11	JX837245
Sarcoptiformes	Carabodidae	*Carabodes labyrinthicus*	MYMCC461-11	JX835589
Sarcoptiformes	Carabodidae	*Carabodes labyrinthicus*	MYMCC508-11	JX837180
Sarcoptiformes	Carabodidae	*Carabodes labyrinthicus*	MYMCC564-11	JX834510
Sarcoptiformes	Carabodidae	*Carabodes labyrinthicus*	MYMCC567-11	JX835365
Sarcoptiformes	Carabodidae	*Carabodes labyrinthicus*	MYMCC613-11	JX837451
Sarcoptiformes	Carabodidae	*Carabodes labyrinthicus*	MYMCC614-11	JX834335
Sarcoptiformes	Carabodidae	*Carabodes labyrinthicus*	MYMCC681-11	JX836337
Sarcoptiformes	Carabodidae	*Carabodes labyrinthicus*	MYMCC682-11	JX838356

Sarcoptiformes	Carabodidae	*Carabodes labyrinthicus*	MYMCE550-12	JX833884
Sarcoptiformes	Carabodidae	*Carabodes labyrinthicus*	MYMCE711-12	JX837172
Sarcoptiformes	Carabodidae	*Carabodes labyrinthicus*	MYMCF375-12	JX834659
Sarcoptiformes	Carabodidae	*Carabodes labyrinthicus*	MYMCF743-12	JX834488
Sarcoptiformes	Carabodidae	*Carabodes labyrinthicus*	MYMCF744-12	JX837878
Sarcoptiformes	Carabodidae	*Carabodes labyrinthicus*	MYMCF760-12	JX836122
Sarcoptiformes	Carabodidae	*Carabodes labyrinthicus*	MYMCG159-12	JX834296
Sarcoptiformes	Carabodidae	*Carabodes labyrinthicus*	MYMCG160-12	JX837380
Sarcoptiformes	Carabodidae	*Carabodes labyrinthicus*	MYMCG176-12	JX834507
Sarcoptiformes	Carabodidae	*Carabodes labyrinthicus*	MYTMC143-09	GU680454
Sarcoptiformes	Carpoglyphidae	*Carpoglyphus lactis*	GBCH4457-10	GQ864345
Sarcoptiformes	Cepheidae	*Cepheus*	CHACA176-08	JX835471
Sarcoptiformes	Cepheidae	*Cepheus*	CHACA620-09	JX838182
Sarcoptiformes	Cepheidae	*Cepheus*	CHACB186-10	HQ558458
Sarcoptiformes	Cepheidae	*Cepheus*	CHACB187-10	HQ558459
Sarcoptiformes	Cepheidae	*Cepheus*	CHACB188-10	HQ558460
Sarcoptiformes	Cepheidae	*Cepheus*	CHACB296-10	HM907288
Sarcoptiformes	Cepheidae	*Cepheus*	CHACB981-10	HM907389
Sarcoptiformes	Cepheidae	*Cepheus*	CNPAP518-13	KM833155
Sarcoptiformes	Cepheidae	*Cepheus*	MYMCA1173-11	JX838404
Sarcoptiformes	Cepheidae	*Cepheus*	MYMCA1324-11	JX837100
Sarcoptiformes	Cepheidae	*Cepheus*	MYMCA485-11	JX835767
Sarcoptiformes	Cepheidae	*Cepheus*	MYMCA486-11	JX837546
Sarcoptiformes	Cepheidae	*Cepheus*	MYMCA487-11	JX834926
Sarcoptiformes	Cepheidae	*Cepheus*	MYMCA773-11	JX838118
Sarcoptiformes	Cepheidae	*Cepheus*	MYMCA934-11	JX838157
Sarcoptiformes	Cepheidae	*Cepheus*	MYMCB058-11	JX836264
Sarcoptiformes	Cepheidae	*Cepheus*	MYMCB059-11	JX835811
Sarcoptiformes	Cepheidae	*Cepheus*	MYMCB513-11	JX835456
Sarcoptiformes	Cepheidae	*Cepheus*	MYMCB541-11	JX835344
Sarcoptiformes	Cepheidae	*Cepheus*	MYMCB725-11	JX838369
Sarcoptiformes	Cepheidae	*Cepheus*	MYMCB727-11	JX834418
Sarcoptiformes	Cepheidae	*Cepheus*	MYMCC252-11	JX834561
Sarcoptiformes	Cepheidae	*Cepheus*	MYMCC253-11	JX836027
Sarcoptiformes	Cepheidae	*Cepheus*	MYMCC462-11	JX833688
Sarcoptiformes	Cepheidae	*Cepheus*	MYMCC550-11	JX835605
Sarcoptiformes	Cepheidae	*Cepheus*	MYMCC565-11	JX834595
Sarcoptiformes	Cepheidae	*Cepheus*	MYMCC591-11	JX837853
Sarcoptiformes	Cepheidae	*Cepheus*	MYMCC592-11	JX834969
Sarcoptiformes	Cepheidae	*Cepheus*	MYMCC603-11	JX837109
Sarcoptiformes	Cepheidae	*Cepheus*	MYMCC612-11	JX835517
Sarcoptiformes	Cepheidae	*Cepheus*	MYMCC714-11	JX835813
Sarcoptiformes	Cepheidae	*Cepheus*	MYMCC785-11	JX836581
Sarcoptiformes	Cepheidae	*Cepheus*	MYMCE647-12	JX833769
Sarcoptiformes	Cepheidae	*Cepheus*	MYMCE815-12	JX837000
Sarcoptiformes	Cepheidae	*Cepheus*	MYMCE831-12	JX834207
Sarcoptiformes	Cepheidae	*Cepheus*	MYMCF385-12	JX834921
Sarcoptiformes	Cepheidae	*Cepheus*	MYMCF862-12	JX837265
Sarcoptiformes	Cepheidae	*Cepheus*	MYMCG157-12	JX836655
Sarcoptiformes	Cepheidae	*Cepheus*	MYMCG158-12	JX836302
Sarcoptiformes	Cepheidae	*Cepheus*	SSBAB1148-12	KM832459
Sarcoptiformes	Cepheidae	*Cepheus*	SSEIA039-13	KM824089
Sarcoptiformes	Cepheidae	*Cepheus*	SSJAA2036-13	KM832154

Sarcoptiformes	Cepheidae	*Cepheus*	SSJAA2051-13	KM838531
Sarcoptiformes	Cepheidae	*Cepheus*	SSJAA2053-13	KM836568
Sarcoptiformes	Cepheidae	*Cepheus*	SSPAB7520-13	KM832874
Sarcoptiformes	Cepheidae	*Cepheus*	SSWLA671-13	KM824262
Sarcoptiformes	Cepheidae	*Cepheus*	SSWLC4145-13	KM825818
Sarcoptiformes	Cepheidae	*Cepheus*	SSWLC4155-13	KM825941
Sarcoptiformes	Cepheidae	*Cepheus*	SSWLD8130-13	KM831213
Sarcoptiformes	Cepheidae	*Oribatodes mirabilis*	SSEIB8167-13	KM831084
Sarcoptiformes	Ceratozetidae	*Ceratozetes cuspidatus*	SSJAD3324-13	KM830430
Sarcoptiformes	Ceratozetidae	*Ceratozetes cuspidatus*	SSWLC4157-13	KM840288
Sarcoptiformes	Ceratozetidae	*Ceratozetes gracilis*	CHACA209-08	JX835739
Sarcoptiformes	Ceratozetidae	*Ceratozetes gracilis*	SSPAA8009-13	KM825119
Sarcoptiformes	Ceratozetidae	*Ceratozetes pacificus*	GBCH3204-08	EF989723
Sarcoptiformes	Ceratozetidae	*Ceratozetes thienemanni*	CHACA157-08	JX838553
Sarcoptiformes	Ceratozetidae	*Ceratozetes thienemanni*	CHACA535-09	JX833727
Sarcoptiformes	Ceratozetidae	*Ceratozetes thienemanni*	CHACA630-09	JX833720
Sarcoptiformes	Ceratozetidae	*Ceratozetes thienemanni*	CHACB1091-10	HM907197
Sarcoptiformes	Ceratozetidae	*Ceratozetes thienemanni*	CHACB1092-10	JX838769
Sarcoptiformes	Ceratozetidae	*Ceratozetes thienemanni*	CHACB1093-10	JX838108
Sarcoptiformes	Ceratozetidae	*Ceratozetes thienemanni*	CHACB1126-10	HM907218
Sarcoptiformes	Ceratozetidae	*Ceratozetes thienemanni*	CHACB861-10	HM907312
Sarcoptiformes	Ceratozetidae	*Ceratozetes thienemanni*	CHACB863-10	JX838727
Sarcoptiformes	Ceratozetidae	*Ceratozetes thienemanni*	MYMCA374-11	JX837902
Sarcoptiformes	Ceratozetidae	*Ceratozetes thienemanni*	MYMCB763-11	JX838486
Sarcoptiformes	Ceratozetidae	*Dentizetes*	SSBAB1158-12	KM832305
Sarcoptiformes	Ceratozetidae	*Dentizetes*	SSBAB1193-12	KM832419
Sarcoptiformes	Ceratozetidae	*Dentizetes*	SSBAB1876-12	KM833548
Sarcoptiformes	Ceratozetidae	*Dentizetes*	SSBAB1904-12	KM832957
Sarcoptiformes	Ceratozetidae	*Dentizetes*	SSBAB1908-12	KM834092
Sarcoptiformes	Ceratozetidae	*Dentizetes*	SSBAB1920-12	KM827292
Sarcoptiformes	Ceratozetidae	*Dentizetes*	SSBAB1926-12	KM835712
Sarcoptiformes	Ceratozetidae	*Dentizetes*	SSBAB1931-12	KM839571
Sarcoptiformes	Ceratozetidae	*Dentizetes*	SSBAB1957-12	KM827549
Sarcoptiformes	Ceratozetidae	*Dentizetes*	SSBAB1960-12	KM838789
Sarcoptiformes	Ceratozetidae	*Dentizetes*	SSBAB2826-12	KM824748
Sarcoptiformes	Ceratozetidae	*Dentizetes*	SSBAD6307-13	KM840227
Sarcoptiformes	Ceratozetidae	*Dentizetes*	SSBAD6317-13	KM831619
Sarcoptiformes	Ceratozetidae	*Dentizetes*	SSEIB5791-13	KM831177
Sarcoptiformes	Ceratozetidae	*Dentizetes*	SSJAA1440-13	KM827796
Sarcoptiformes	Ceratozetidae	*Dentizetes*	SSJAA1991-13	KM826284
Sarcoptiformes	Ceratozetidae	*Dentizetes*	SSJAC1519-13	KM831285
Sarcoptiformes	Ceratozetidae	*Dentizetes*	SSJAC1528-13	KM835629
Sarcoptiformes	Ceratozetidae	*Dentizetes*	SSJAC1537-13	KM836731
Sarcoptiformes	Ceratozetidae	*Dentizetes*	SSJAC1546-13	KM824163
Sarcoptiformes	Ceratozetidae	*Dentizetes*	SSJAC1573-13	KM837793
Sarcoptiformes	Ceratozetidae	*Dentizetes*	SSJAC1579-13	KM831483
Sarcoptiformes	Ceratozetidae	*Dentizetes*	SSJAC1596-13	KM826686
Sarcoptiformes	Ceratozetidae	*Dentizetes*	SSJAC1599-13	KM835457
Sarcoptiformes	Ceratozetidae	*Dentizetes*	SSJAC970-13	KM830774
Sarcoptiformes	Ceratozetidae	*Dentizetes*	SSJAF5554-13	KM835233
Sarcoptiformes	Ceratozetidae	*Dentizetes*	SSJAF5584-13	KM836114
Sarcoptiformes	Ceratozetidae	*Dentizetes*	SSJAF7386-13	KM824598
Sarcoptiformes	Ceratozetidae	*Dentizetes*	SSJAF8417-13	KM827172

Sarcoptiformes	Ceratozetidae	*Dentizetes*		SSWLA5271-13	KM833462
Sarcoptiformes	Ceratozetidae	*Dentizetes*		SSWLA677-13	KM835190
Sarcoptiformes	Ceratozetidae	*Dentizetes*		SSWLA679-13	KM828004
Sarcoptiformes	Ceratozetidae	*Dentizetes*		SSWLC1146-13	KM831250
Sarcoptiformes	Ceratozetidae	*Dentizetes*		SSWLC1147-13	KM827372
Sarcoptiformes	Ceratozetidae	*Dentizetes*		SSWLC1148-13	KM836724
Sarcoptiformes	Ceratozetidae	*Dentizetes*		SSWLC1149-13	KM827543
Sarcoptiformes	Ceratozetidae	*Dentizetes*		SSWLC1150-13	KM829958
Sarcoptiformes	Ceratozetidae	*Dentizetes*		SSWLC1153-13	KM824537
Sarcoptiformes	Ceratozetidae	*Dentizetes*		SSWLC1163-13	KM839105
Sarcoptiformes	Ceratozetidae	*Dentizetes*		SSWLC1174-13	KM828123
Sarcoptiformes	Ceratozetidae	*Dentizetes*		SSWLC1175-13	KM830702
Sarcoptiformes	Ceratozetidae	*Dentizetes*		SSWLC1177-13	KM827990
Sarcoptiformes	Ceratozetidae	*Dentizetes*		SSWLC1183-13	KM832066
Sarcoptiformes	Ceratozetidae	*Dentizetes*		SSWLC1189-13	KM827406
Sarcoptiformes	Ceratozetidae	*Dentizetes*		SSWLD8205-13	KM827895
Sarcoptiformes	Ceratozetidae	*Dentizetes*		SSWLD8242-13	KM833685
Sarcoptiformes	Ceratozetidae	*Dentizetes*		SSWLD8277-13	KM826579
Sarcoptiformes	Ceratozetidae	*Dentizetes rudentiger*		SSBAB2093-12	KM829196
Sarcoptiformes	Ceratozetidae	*Dentizetes rudentiger*		SSJAA860-13	KM833726
Sarcoptiformes	Ceratozetidae	*Dentizetes rudentiger*		SSJAA865-13	KM827492
Sarcoptiformes	Ceratozetidae	*Dentizetes rudentiger*		SSWLA039-13	KM831024
Sarcoptiformes	Ceratozetidae	*Diapterobates*		CHACA1066-10	HM907112
Sarcoptiformes	Ceratozetidae	*Diapterobates*		CHACA612-09	JX835987
Sarcoptiformes	Ceratozetidae	*Diapterobates*		CHACA613-09	JX838564
Sarcoptiformes	Ceratozetidae	*Diapterobates*		MYMCA1012-11	JX837178
Sarcoptiformes	Ceratozetidae	*Diapterobates*		MYMCA1042-11	JX835417
Sarcoptiformes	Ceratozetidae	*Diapterobates*		MYMCA113-11	JX834023
Sarcoptiformes	Ceratozetidae	*Diapterobates*		MYMCA114-11	JX835534
Sarcoptiformes	Ceratozetidae	*Diapterobates*		MYMCA1287-11	JX833848
Sarcoptiformes	Ceratozetidae	*Diapterobates*		MYMCA1387-11	JX834242
Sarcoptiformes	Ceratozetidae	*Diapterobates*		MYMCA1440-11	JX835446
Sarcoptiformes	Ceratozetidae	*Diapterobates*		MYMCA1502-11	JX836631
Sarcoptiformes	Ceratozetidae	*Diapterobates*		MYMCA445-11	JX835121
Sarcoptiformes	Ceratozetidae	*Diapterobates*		MYMCA573-11	JX838300
Sarcoptiformes	Ceratozetidae	*Diapterobates*		MYMCA645-11	JX836295
Sarcoptiformes	Ceratozetidae	*Diapterobates*		MYMCA671-11	JX835787
Sarcoptiformes	Ceratozetidae	*Diapterobates*		MYMCA678-11	JX834260
Sarcoptiformes	Ceratozetidae	*Diapterobates*		MYMCA686-11	JX838129
Sarcoptiformes	Ceratozetidae	*Diapterobates*		MYMCA740-11	JX835975
Sarcoptiformes	Ceratozetidae	*Diapterobates*		MYMCB413-11	JX834861
Sarcoptiformes	Ceratozetidae	*Diapterobates*		MYMCB928-11	JX837375
Sarcoptiformes	Ceratozetidae	*Diapterobates*		MYMCC426-11	JX834867
Sarcoptiformes	Ceratozetidae	*Diapterobates*		MYMCD061-11	JX836129
Sarcoptiformes	Ceratozetidae	*Diapterobates*		MYMCD094-11	JX837382
Sarcoptiformes	Ceratozetidae	*Diapterobates*		MYMCD112-11	JX834326
Sarcoptiformes	Ceratozetidae	*Diapterobates*		MYMCD151-11	JX835741
Sarcoptiformes	Ceratozetidae	*Diapterobates*		MYMCD175-11	JX838432
Sarcoptiformes	Ceratozetidae	*Diapterobates*		MYMCE551-12	JX837271
Sarcoptiformes	Ceratozetidae	*Diapterobates*		MYMCE680-12	JX837401
Sarcoptiformes	Ceratozetidae	*Diapterobates*		MYMCE681-12	JX833939
Sarcoptiformes	Ceratozetidae	*Diapterobates*		MYMCF614-12	JX838364
Sarcoptiformes	Ceratozetidae	*Diapterobates*		MYMCG302-12	JX835197

Sarcoptiformes	Ceratozetidae	*Diapterobates*	MYTMC073-09	HM431997
Sarcoptiformes	Ceratozetidae	*Diapterobates*	MYTMC083-09	HM431998
Sarcoptiformes	Ceratozetidae	*Diapterobates*	SSBAB1159-12	KM826878
Sarcoptiformes	Ceratozetidae	*Diapterobates*	SSBAB1932-12	KM831312
Sarcoptiformes	Ceratozetidae	*Diapterobates humeralis*	CHACA1010-10	HM907074
Sarcoptiformes	Ceratozetidae	*Diapterobates humeralis*	CHACA1011-10	HM907075
Sarcoptiformes	Ceratozetidae	*Diapterobates humeralis*	CHACA1012-10	HM907076
Sarcoptiformes	Ceratozetidae	*Diapterobates humeralis*	CHACA1036-10	HM907089
Sarcoptiformes	Ceratozetidae	*Diapterobates humeralis*	CHACA1037-10	HM907090
Sarcoptiformes	Ceratozetidae	*Diapterobates humeralis*	CHACA1038-10	HM907091
Sarcoptiformes	Ceratozetidae	*Diapterobates humeralis*	CHACA1039-10	HM907092
Sarcoptiformes	Ceratozetidae	*Diapterobates humeralis*	CHACA1040-10	HM907093
Sarcoptiformes	Ceratozetidae	*Diapterobates humeralis*	CHACA1059-10	HM907107
Sarcoptiformes	Ceratozetidae	*Diapterobates humeralis*	CHACA1061-10	HM907109
Sarcoptiformes	Ceratozetidae	*Diapterobates humeralis*	CHACA1063-10	HM907110
Sarcoptiformes	Ceratozetidae	*Diapterobates humeralis*	CHACA1067-10	HM907113
Sarcoptiformes	Ceratozetidae	*Diapterobates humeralis*	CHACA1068-10	HM907114
Sarcoptiformes	Ceratozetidae	*Diapterobates humeralis*	CHACA1095-10	HM907133
Sarcoptiformes	Ceratozetidae	*Diapterobates humeralis*	CHACA1096-10	HM907134
Sarcoptiformes	Ceratozetidae	*Diapterobates humeralis*	CHACA1141-10	JX836160
Sarcoptiformes	Ceratozetidae	*Diapterobates humeralis*	CHACA1142-10	JX838087
Sarcoptiformes	Ceratozetidae	*Diapterobates humeralis*	CHACA1143-10	JX834135
Sarcoptiformes	Ceratozetidae	*Diapterobates humeralis*	CHACA1144-10	JX833902
Sarcoptiformes	Ceratozetidae	*Diapterobates humeralis*	CHACA1145-10	JX835029
Sarcoptiformes	Ceratozetidae	*Diapterobates humeralis*	CHACA1149-10	JX835707
Sarcoptiformes	Ceratozetidae	*Diapterobates humeralis*	CHACA1150-10	JX834519
Sarcoptiformes	Ceratozetidae	*Diapterobates humeralis*	CHACA1175-10	JX838262
Sarcoptiformes	Ceratozetidae	*Diapterobates humeralis*	CHACA1176-10	JX833988
Sarcoptiformes	Ceratozetidae	*Diapterobates humeralis*	CHACA1177-10	JX837851
Sarcoptiformes	Ceratozetidae	*Diapterobates humeralis*	CHACA167-08	JX834479
Sarcoptiformes	Ceratozetidae	*Diapterobates humeralis*	CHACA168-08	JX835254
Sarcoptiformes	Ceratozetidae	*Diapterobates humeralis*	CHACA537-09	JX836251
Sarcoptiformes	Ceratozetidae	*Diapterobates humeralis*	CHACA809-09	JX834381
Sarcoptiformes	Ceratozetidae	*Diapterobates humeralis*	CHACA814-09	JX835051
Sarcoptiformes	Ceratozetidae	*Diapterobates humeralis*	CHACB047-10	HQ558358
Sarcoptiformes	Ceratozetidae	*Diapterobates humeralis*	CHACB099-10	HQ558395
Sarcoptiformes	Ceratozetidae	*Diapterobates humeralis*	CHACB100-10	HQ558396
Sarcoptiformes	Ceratozetidae	*Diapterobates humeralis*	CHACB1058-10	HM907182
Sarcoptiformes	Ceratozetidae	*Diapterobates humeralis*	CHACB1059-10	HM907183
Sarcoptiformes	Ceratozetidae	*Diapterobates humeralis*	CHACB1060-10	HM907184
Sarcoptiformes	Ceratozetidae	*Diapterobates humeralis*	CHACB1061-10	HM907185
Sarcoptiformes	Ceratozetidae	*Diapterobates humeralis*	CHACB1077-10	HM907190
Sarcoptiformes	Ceratozetidae	*Diapterobates humeralis*	CHACB1078-10	HM907191
Sarcoptiformes	Ceratozetidae	*Diapterobates humeralis*	CHACB1079-10	HM907192
Sarcoptiformes	Ceratozetidae	*Diapterobates humeralis*	CHACB109-10	HQ558403
Sarcoptiformes	Ceratozetidae	*Diapterobates humeralis*	CHACB1113-10	HM907207
Sarcoptiformes	Ceratozetidae	*Diapterobates humeralis*	CHACB1124-10	HM907216
Sarcoptiformes	Ceratozetidae	*Diapterobates humeralis*	CHACB1133-10	HM907223
Sarcoptiformes	Ceratozetidae	*Diapterobates humeralis*	CHACB1134-10	HM907224
Sarcoptiformes	Ceratozetidae	*Diapterobates humeralis*	CHACB1135-10	HM907225
Sarcoptiformes	Ceratozetidae	*Diapterobates humeralis*	CHACB1143-10	HM907232
Sarcoptiformes	Ceratozetidae	*Diapterobates humeralis*	CHACB1144-10	HM907233
Sarcoptiformes	Ceratozetidae	*Diapterobates humeralis*	CHACB1145-10	HM907234

Sarcoptiformes	Ceratozetidae	*Diapterobates humeralis*	CHACB1146-10	HM907235
Sarcoptiformes	Ceratozetidae	*Diapterobates humeralis*	CHACB1147-10	HM907236
Sarcoptiformes	Ceratozetidae	*Diapterobates humeralis*	CHACB189-10	HQ558461
Sarcoptiformes	Ceratozetidae	*Diapterobates humeralis*	CHACB190-10	HQ558462
Sarcoptiformes	Ceratozetidae	*Diapterobates humeralis*	CHACB192-10	HQ558463
Sarcoptiformes	Ceratozetidae	*Diapterobates humeralis*	CHACB200-10	HQ558470
Sarcoptiformes	Ceratozetidae	*Diapterobates humeralis*	CHACB203-10	HQ558471
Sarcoptiformes	Ceratozetidae	*Diapterobates humeralis*	CHACB252-10	HQ558495
Sarcoptiformes	Ceratozetidae	*Diapterobates humeralis*	CHACB253-10	HQ558496
Sarcoptiformes	Ceratozetidae	*Diapterobates humeralis*	CHACB254-10	HQ558497
Sarcoptiformes	Ceratozetidae	*Diapterobates humeralis*	CHACB255-10	HQ558498
Sarcoptiformes	Ceratozetidae	*Diapterobates humeralis*	CHACB256-10	HQ558499
Sarcoptiformes	Ceratozetidae	*Diapterobates humeralis*	CHACB266-10	HQ558507
Sarcoptiformes	Ceratozetidae	*Diapterobates humeralis*	CHACB285-10	HQ558513
Sarcoptiformes	Ceratozetidae	*Diapterobates humeralis*	CHACB287-10	HM907281
Sarcoptiformes	Ceratozetidae	*Diapterobates humeralis*	CHACB288-10	HM907282
Sarcoptiformes	Ceratozetidae	*Diapterobates humeralis*	CHACB300-10	HM907291
Sarcoptiformes	Ceratozetidae	*Diapterobates humeralis*	CHACB301-10	HM907292
Sarcoptiformes	Ceratozetidae	*Diapterobates humeralis*	CHACB302-10	HM907293
Sarcoptiformes	Ceratozetidae	*Diapterobates humeralis*	CHACB303-10	HM907294
Sarcoptiformes	Ceratozetidae	*Diapterobates humeralis*	CHACB433-10	HQ558589
Sarcoptiformes	Ceratozetidae	*Diapterobates humeralis*	CHACB537-10	HQ558653
Sarcoptiformes	Ceratozetidae	*Diapterobates humeralis*	CHACB538-10	HQ558654
Sarcoptiformes	Ceratozetidae	*Diapterobates humeralis*	CHACB539-10	HQ558655
Sarcoptiformes	Ceratozetidae	*Diapterobates humeralis*	CHACB540-10	HQ558656
Sarcoptiformes	Ceratozetidae	*Diapterobates humeralis*	CHACB564-10	HQ558678
Sarcoptiformes	Ceratozetidae	*Diapterobates humeralis*	CHACB565-10	HQ558679
Sarcoptiformes	Ceratozetidae	*Diapterobates humeralis*	CHACB566-10	HQ558680
Sarcoptiformes	Ceratozetidae	*Diapterobates humeralis*	CHACB651-10	HQ558731
Sarcoptiformes	Ceratozetidae	*Diapterobates humeralis*	CHACB652-10	HQ558732
Sarcoptiformes	Ceratozetidae	*Diapterobates humeralis*	CHACB685-10	HQ558746
Sarcoptiformes	Ceratozetidae	*Diapterobates humeralis*	CHACB975-10	HM907386
Sarcoptiformes	Ceratozetidae	*Diapterobates humeralis*	CHACB984-10	HM907391
Sarcoptiformes	Ceratozetidae	*Diapterobates humeralis*	CHACB985-10	HM907392
Sarcoptiformes	Ceratozetidae	*Diapterobates humeralis*	CHACB986-10	HM907393
Sarcoptiformes	Ceratozetidae	*Diapterobates humeralis*	CHACC086-10	HM907479
Sarcoptiformes	Ceratozetidae	*Diapterobates humeralis*	CHACC185-10	JX834059
Sarcoptiformes	Ceratozetidae	*Diapterobates humeralis*	CHACC186-10	JX833713
Sarcoptiformes	Ceratozetidae	*Diapterobates humeralis*	CHACC193-10	HQ941519
Sarcoptiformes	Ceratozetidae	*Diapterobates humeralis*	CHACC234-10	HQ941542
Sarcoptiformes	Ceratozetidae	*Diapterobates humeralis*	CHACC271-10	HQ941569
Sarcoptiformes	Ceratozetidae	*Diapterobates humeralis*	CHACC272-10	HQ941570
Sarcoptiformes	Ceratozetidae	*Diapterobates humeralis*	CHACC273-10	HQ941571
Sarcoptiformes	Ceratozetidae	*Diapterobates humeralis*	CHACC274-10	HQ941572
Sarcoptiformes	Ceratozetidae	*Diapterobates humeralis*	CHACC275-10	HQ941573
Sarcoptiformes	Ceratozetidae	*Diapterobates humeralis*	CNJAB1034-12	KM832110
Sarcoptiformes	Ceratozetidae	*Diapterobates humeralis*	MYMCA1013-11	JX837621
Sarcoptiformes	Ceratozetidae	*Diapterobates humeralis*	MYMCA1023-11	JX836094
Sarcoptiformes	Ceratozetidae	*Diapterobates humeralis*	MYMCA112-11	JX836432
Sarcoptiformes	Ceratozetidae	*Diapterobates humeralis*	MYMCA115-11	JX838248
Sarcoptiformes	Ceratozetidae	*Diapterobates humeralis*	MYMCA1357-11	JX835581
Sarcoptiformes	Ceratozetidae	*Diapterobates humeralis*	MYMCA1441-11	JX838110
Sarcoptiformes	Ceratozetidae	*Diapterobates humeralis*	MYMCA1464-11	JX836489

Sarcoptiformes	Ceratozetidae	*Diapterobates humeralis*	MYMCA1465-11	JX837625
Sarcoptiformes	Ceratozetidae	*Diapterobates humeralis*	MYMCA1466-11	JX834961
Sarcoptiformes	Ceratozetidae	*Diapterobates humeralis*	MYMCA1503-11	JX838787
Sarcoptiformes	Ceratozetidae	*Diapterobates humeralis*	MYMCA1511-11	JX834873
Sarcoptiformes	Ceratozetidae	*Diapterobates humeralis*	MYMCA1512-11	JX837236
Sarcoptiformes	Ceratozetidae	*Diapterobates humeralis*	MYMCA422-11	JX834292
Sarcoptiformes	Ceratozetidae	*Diapterobates humeralis*	MYMCA424-11	JX834365
Sarcoptiformes	Ceratozetidae	*Diapterobates humeralis*	MYMCA425-11	JX836944
Sarcoptiformes	Ceratozetidae	*Diapterobates humeralis*	MYMCA427-11	JX834213
Sarcoptiformes	Ceratozetidae	*Diapterobates humeralis*	MYMCA483-11	JX837731
Sarcoptiformes	Ceratozetidae	*Diapterobates humeralis*	MYMCA484-11	JX837390
Sarcoptiformes	Ceratozetidae	*Diapterobates humeralis*	MYMCA523-11	JX837197
Sarcoptiformes	Ceratozetidae	*Diapterobates humeralis*	MYMCA546-11	JX836962
Sarcoptiformes	Ceratozetidae	*Diapterobates humeralis*	MYMCA621-11	JX838133
Sarcoptiformes	Ceratozetidae	*Diapterobates humeralis*	MYMCA622-11	JX834032
Sarcoptiformes	Ceratozetidae	*Diapterobates humeralis*	MYMCA623-11	JX834100
Sarcoptiformes	Ceratozetidae	*Diapterobates humeralis*	MYMCA644-11	JX837366
Sarcoptiformes	Ceratozetidae	*Diapterobates humeralis*	MYMCA670-11	JX836571
Sarcoptiformes	Ceratozetidae	*Diapterobates humeralis*	MYMCA677-11	JX837160
Sarcoptiformes	Ceratozetidae	*Diapterobates humeralis*	MYMCA685-11	JX838141
Sarcoptiformes	Ceratozetidae	*Diapterobates humeralis*	MYMCA739-11	JX838035
Sarcoptiformes	Ceratozetidae	*Diapterobates humeralis*	MYMCA801-11	JX837918
Sarcoptiformes	Ceratozetidae	*Diapterobates humeralis*	MYMCA820-11	JX836837
Sarcoptiformes	Ceratozetidae	*Diapterobates humeralis*	MYMCA985-11	JX835891
Sarcoptiformes	Ceratozetidae	*Diapterobates humeralis*	MYMCA986-11	JX837917
Sarcoptiformes	Ceratozetidae	*Diapterobates humeralis*	MYMCB013-11	JX838579
Sarcoptiformes	Ceratozetidae	*Diapterobates humeralis*	MYMCB084-11	JX835902
Sarcoptiformes	Ceratozetidae	*Diapterobates humeralis*	MYMCB150-11	JX834948
Sarcoptiformes	Ceratozetidae	*Diapterobates humeralis*	MYMCB256-11	JX834366
Sarcoptiformes	Ceratozetidae	*Diapterobates humeralis*	MYMCB278-11	JX837066
Sarcoptiformes	Ceratozetidae	*Diapterobates humeralis*	MYMCB384-11	JX836735
Sarcoptiformes	Ceratozetidae	*Diapterobates humeralis*	MYMCB411-11	JX835834
Sarcoptiformes	Ceratozetidae	*Diapterobates humeralis*	MYMCB534-11	JX838241
Sarcoptiformes	Ceratozetidae	*Diapterobates humeralis*	MYMCB575-11	JX834630
Sarcoptiformes	Ceratozetidae	*Diapterobates humeralis*	MYMCB594-11	JX833819
Sarcoptiformes	Ceratozetidae	*Diapterobates humeralis*	MYMCB595-11	JX833952
Sarcoptiformes	Ceratozetidae	*Diapterobates humeralis*	MYMCB700-11	JX835861
Sarcoptiformes	Ceratozetidae	*Diapterobates humeralis*	MYMCB775-11	JX834127
Sarcoptiformes	Ceratozetidae	*Diapterobates humeralis*	MYMCB803-11	JX837582
Sarcoptiformes	Ceratozetidae	*Diapterobates humeralis*	MYMCB844-11	JX838728
Sarcoptiformes	Ceratozetidae	*Diapterobates humeralis*	MYMCB853-11	JX836762
Sarcoptiformes	Ceratozetidae	*Diapterobates humeralis*	MYMCB867-11	JX835140
Sarcoptiformes	Ceratozetidae	*Diapterobates humeralis*	MYMCB877-11	JX834331
Sarcoptiformes	Ceratozetidae	*Diapterobates humeralis*	MYMCB908-11	JX837428
Sarcoptiformes	Ceratozetidae	*Diapterobates humeralis*	MYMCC012-11	JX838281
Sarcoptiformes	Ceratozetidae	*Diapterobates humeralis*	MYMCC040-11	JX834771
Sarcoptiformes	Ceratozetidae	*Diapterobates humeralis*	MYMCC141-11	JX836404
Sarcoptiformes	Ceratozetidae	*Diapterobates humeralis*	MYMCC142-11	JX837578
Sarcoptiformes	Ceratozetidae	*Diapterobates humeralis*	MYMCC309-11	JX835025
Sarcoptiformes	Ceratozetidae	*Diapterobates humeralis*	MYMCC336-11	JX837054
Sarcoptiformes	Ceratozetidae	*Diapterobates humeralis*	MYMCC457-11	JX838302
Sarcoptiformes	Ceratozetidae	*Diapterobates humeralis*	MYMCC506-11	JX835568
Sarcoptiformes	Ceratozetidae	*Diapterobates humeralis*	MYMCC548-11	JX836795

Sarcoptiformes	Ceratozetidae	*Diapterobates humeralis*	MYMCC577-11	JX837913
Sarcoptiformes	Ceratozetidae	*Diapterobates humeralis*	MYMCC578-11	JX834956
Sarcoptiformes	Ceratozetidae	*Diapterobates humeralis*	MYMCC606-11	JX837952
Sarcoptiformes	Ceratozetidae	*Diapterobates humeralis*	MYMCC611-11	JX837031
Sarcoptiformes	Ceratozetidae	*Diapterobates humeralis*	MYMCC672-11	JX834027
Sarcoptiformes	Ceratozetidae	*Diapterobates humeralis*	MYMCC689-11	JX835469
Sarcoptiformes	Ceratozetidae	*Diapterobates humeralis*	MYMCC840-11	JX838256
Sarcoptiformes	Ceratozetidae	*Diapterobates humeralis*	MYMCC901-11	JX837508
Sarcoptiformes	Ceratozetidae	*Diapterobates humeralis*	MYMCD152-11	JX838344
Sarcoptiformes	Ceratozetidae	*Diapterobates humeralis*	MYMCD174-11	JX836926
Sarcoptiformes	Ceratozetidae	*Diapterobates humeralis*	MYMCE200-12	JX834298
Sarcoptiformes	Ceratozetidae	*Diapterobates humeralis*	MYMCE429-12	JX837474
Sarcoptiformes	Ceratozetidae	*Diapterobates humeralis*	MYMCE670-12	JX834126
Sarcoptiformes	Ceratozetidae	*Diapterobates humeralis*	MYMCE677-12	JX834847
Sarcoptiformes	Ceratozetidae	*Diapterobates humeralis*	MYMCE750-12	JX836720
Sarcoptiformes	Ceratozetidae	*Diapterobates humeralis*	MYMCF290-12	JX838575
Sarcoptiformes	Ceratozetidae	*Diapterobates humeralis*	MYMCF365-12	JX838461
Sarcoptiformes	Ceratozetidae	*Diapterobates humeralis*	MYMCF581-12	JX833662
Sarcoptiformes	Ceratozetidae	*Diapterobates humeralis*	MYMCF658-12	JX837042
Sarcoptiformes	Ceratozetidae	*Diapterobates humeralis*	MYMCF837-12	JX835956
Sarcoptiformes	Ceratozetidae	*Diapterobates humeralis*	MYMCG154-12	JX835367
Sarcoptiformes	Ceratozetidae	*Diapterobates humeralis*	MYMCG155-12	JX835752
Sarcoptiformes	Ceratozetidae	*Diapterobates humeralis*	MYMCG565-12	JX837020
Sarcoptiformes	Ceratozetidae	*Diapterobates humeralis*	MYTMC066-09	GU680463
Sarcoptiformes	Ceratozetidae	*Diapterobates humeralis*	MYTMC174-09	GU680425
Sarcoptiformes	Ceratozetidae	*Diapterobates humeralis*	SSBAB1150-12	KM835221
Sarcoptiformes	Ceratozetidae	*Diapterobates humeralis*	SSBAB1151-12	KM832915
Sarcoptiformes	Ceratozetidae	*Diapterobates humeralis*	SSBAB1152-12	KM835726
Sarcoptiformes	Ceratozetidae	*Diapterobates humeralis*	SSBAB1162-12	KM824118
Sarcoptiformes	Ceratozetidae	*Diapterobates humeralis*	SSBAB1163-12	KM826012
Sarcoptiformes	Ceratozetidae	*Diapterobates humeralis*	SSBAB1927-12	KM837724
Sarcoptiformes	Ceratozetidae	*Diapterobates humeralis*	SSBAD3999-12	KM835245
Sarcoptiformes	Ceratozetidae	*Diapterobates humeralis*	SSBAD4705-13	KM833206
Sarcoptiformes	Ceratozetidae	*Diapterobates humeralis*	SSBAD5547-13	KM828818
Sarcoptiformes	Ceratozetidae	*Diapterobates humeralis*	SSBAE3006-13	KM826211
Sarcoptiformes	Ceratozetidae	*Diapterobates humeralis*	SSBAE3622-13	KM833529
Sarcoptiformes	Ceratozetidae	*Diapterobates humeralis*	SSBAE3626-13	KM825791
Sarcoptiformes	Ceratozetidae	*Diapterobates humeralis*	SSEIB4560-13	KM830203
Sarcoptiformes	Ceratozetidae	*Diapterobates humeralis*	SSEIB4570-13	KM833435
Sarcoptiformes	Ceratozetidae	*Diapterobates humeralis*	SSEIB5421-13	KM832094
Sarcoptiformes	Ceratozetidae	*Diapterobates humeralis*	SSPAA6659-13	KM824623
Sarcoptiformes	Ceratozetidae	*Diapterobates humeralis*	SSPAA6661-13	KM833101
Sarcoptiformes	Ceratozetidae	*Diapterobates humeralis*	SSPAA6666-13	KM827462
Sarcoptiformes	Ceratozetidae	*Diapterobates humeralis*	SSPAA6681-13	KM837179
Sarcoptiformes	Ceratozetidae	*Diapterobates humeralis*	SSPAA7798-13	KM832897
Sarcoptiformes	Ceratozetidae	*Diapterobates humeralis*	SSPAA7813-13	KM825711
Sarcoptiformes	Ceratozetidae	*Diapterobates humeralis*	SSPAA7817-13	KM829169
Sarcoptiformes	Ceratozetidae	*Diapterobates notatus*	CHACA162-08	JX836476
Sarcoptiformes	Ceratozetidae	*Diapterobates notatus*	CHACA275-08	JX835326
Sarcoptiformes	Ceratozetidae	*Diapterobates notatus*	CHACA277-08	JX834883
Sarcoptiformes	Ceratozetidae	*Diapterobates notatus*	CHACA278-08	JX836649
Sarcoptiformes	Ceratozetidae	*Diapterobates notatus*	CHACA279-08	JX837449
Sarcoptiformes	Ceratozetidae	*Diapterobates notatus*	CHACA285-08	JX835873

Sarcoptiformes	Ceratozetidae	*Diapterobates notatus*	CHACA286-08	JX838781
Sarcoptiformes	Ceratozetidae	*Diapterobates notatus*	CHACA338-08	JX838041
Sarcoptiformes	Ceratozetidae	*Diapterobates notatus*	CHACA343-08	JX838462
Sarcoptiformes	Ceratozetidae	*Diapterobates notatus*	CHACA367-08	JX835947
Sarcoptiformes	Ceratozetidae	*Diapterobates notatus*	CHACA501-09	JX837448
Sarcoptiformes	Ceratozetidae	*Diapterobates notatus*	CHACA518-09	JX834497
Sarcoptiformes	Ceratozetidae	*Diapterobates notatus*	CHACA611-09	JX833673
Sarcoptiformes	Ceratozetidae	*Diapterobates notatus*	CHACB353-10	HQ558534
Sarcoptiformes	Ceratozetidae	*Diapterobates notatus*	CHACB410-10	HQ558570
Sarcoptiformes	Ceratozetidae	*Diapterobates notatus*	CHACB536-10	HQ558652
Sarcoptiformes	Ceratozetidae	*Diapterobates notatus*	CHACB541-10	HQ558657
Sarcoptiformes	Ceratozetidae	*Diapterobates notatus*	CHACC059-10	HM907456
Sarcoptiformes	Ceratozetidae	*Diapterobates notatus*	MYMCA1336-11	JX836437
Sarcoptiformes	Ceratozetidae	*Diapterobates notatus*	MYMCA1338-11	JX837286
Sarcoptiformes	Ceratozetidae	*Diapterobates notatus*	MYMCA1343-11	JX835757
Sarcoptiformes	Ceratozetidae	*Diapterobates notatus*	MYMCA1344-11	JX838709
Sarcoptiformes	Ceratozetidae	*Diapterobates notatus*	MYMCA136-11	JX838406
Sarcoptiformes	Ceratozetidae	*Diapterobates notatus*	MYMCA137-11	JX837809
Sarcoptiformes	Ceratozetidae	*Diapterobates notatus*	MYMCA138-11	JX833665
Sarcoptiformes	Ceratozetidae	*Diapterobates notatus*	MYMCA139-11	JX834698
Sarcoptiformes	Ceratozetidae	*Diapterobates notatus*	MYMCA140-11	JX835658
Sarcoptiformes	Ceratozetidae	*Diapterobates notatus*	MYMCA161-11	JX835224
Sarcoptiformes	Ceratozetidae	*Diapterobates notatus*	MYMCA162-11	JX835997
Sarcoptiformes	Ceratozetidae	*Diapterobates notatus*	MYMCC101-11	JX833903
Sarcoptiformes	Ceratozetidae	*Diapterobates notatus*	MYMCC102-11	JX838459
Sarcoptiformes	Ceratozetidae	*Diapterobates notatus*	MYMCC119-11	JX838238
Sarcoptiformes	Ceratozetidae	*Diapterobates notatus*	MYMCC299-11	JX833910
Sarcoptiformes	Ceratozetidae	*Diapterobates notatus*	MYMCD115-11	JX835626
Sarcoptiformes	Ceratozetidae	*Diapterobates notatus*	MYMCE049-12	JX834954
Sarcoptiformes	Ceratozetidae	*Diapterobates notatus*	MYMCE152-12	JX838172
Sarcoptiformes	Ceratozetidae	*Diapterobates variabilis*	CHACA045-08	JX834393
Sarcoptiformes	Ceratozetidae	*Diapterobates variabilis*	CHACA1065-10	HM907111
Sarcoptiformes	Ceratozetidae	*Diapterobates variabilis*	CHACA977-10	HM405841
Sarcoptiformes	Ceratozetidae	*Diapterobates variabilis*	CHACB194-10	HQ558464
Sarcoptiformes	Ceratozetidae	*Diapterobates variabilis*	CHACB195-10	HQ558465
Sarcoptiformes	Ceratozetidae	*Diapterobates variabilis*	CHACB196-10	HQ558466
Sarcoptiformes	Ceratozetidae	*Diapterobates variabilis*	CHACB260-10	HQ558503
Sarcoptiformes	Ceratozetidae	*Diapterobates variabilis*	CHACB261-10	HQ558504
Sarcoptiformes	Ceratozetidae	*Diapterobates variabilis*	CHACB262-10	HQ941472
Sarcoptiformes	Ceratozetidae	*Diapterobates variabilis*	CHACB715-10	HQ558765
Sarcoptiformes	Ceratozetidae	*Diapterobates variabilis*	CHACB716-10	HQ558766
Sarcoptiformes	Ceratozetidae	*Diapterobates variabilis*	CHACB717-10	HQ558767
Sarcoptiformes	Ceratozetidae	*Diapterobates variabilis*	CHACB913-10	HM907342
Sarcoptiformes	Ceratozetidae	*Diapterobates variabilis*	CHACC270-10	HQ941568
Sarcoptiformes	Ceratozetidae	*Diapterobates variabilis*	MITMH016-07	KR070198
Sarcoptiformes	Ceratozetidae	*Diapterobates variabilis*	MYMCA041-11	JX838519
Sarcoptiformes	Ceratozetidae	*Diapterobates variabilis*	MYMCA1317-11	JX837569
Sarcoptiformes	Ceratozetidae	*Diapterobates variabilis*	MYMCA1318-11	JX838572
Sarcoptiformes	Ceratozetidae	*Diapterobates variabilis*	MYMCA502-11	JX837291
Sarcoptiformes	Ceratozetidae	*Diapterobates variabilis*	MYMCA503-11	JX838270
Sarcoptiformes	Ceratozetidae	*Diapterobates variabilis*	MYMCA942-11	JX833732
Sarcoptiformes	Ceratozetidae	*Diapterobates variabilis*	MYMCB261-11	JX838069
Sarcoptiformes	Ceratozetidae	*Diapterobates variabilis*	MYMCB515-11	JX834293

Sarcoptiformes	Ceratozetidae	*Diapterobates variabilis*	MYMCB744-11	JX834662
Sarcoptiformes	Ceratozetidae	*Diapterobates variabilis*	MYMCB802-11	JX835903
Sarcoptiformes	Ceratozetidae	*Diapterobates variabilis*	MYMCC015-11	JX837320
Sarcoptiformes	Ceratozetidae	*Diapterobates variabilis*	MYMCC087-11	JX834983
Sarcoptiformes	Ceratozetidae	*Diapterobates variabilis*	MYMCC143-11	JX835536
Sarcoptiformes	Ceratozetidae	*Diapterobates variabilis*	MYMCC144-11	JX837644
Sarcoptiformes	Ceratozetidae	*Diapterobates variabilis*	MYMCC145-11	JX834776
Sarcoptiformes	Ceratozetidae	*Diapterobates variabilis*	MYMCC287-11	JX838258
Sarcoptiformes	Ceratozetidae	*Diapterobates variabilis*	MYMCC410-11	JX836902
Sarcoptiformes	Ceratozetidae	*Diapterobates variabilis*	MYMCC568-11	JX836484
Sarcoptiformes	Ceratozetidae	*Diapterobates variabilis*	MYMCC635-11	JX835927
Sarcoptiformes	Ceratozetidae	*Diapterobates variabilis*	MYMCC638-11	JX834932
Sarcoptiformes	Ceratozetidae	*Diapterobates variabilis*	MYMCC718-11	JX835829
Sarcoptiformes	Ceratozetidae	*Diapterobates variabilis*	MYMCC789-11	JX835560
Sarcoptiformes	Ceratozetidae	*Diapterobates variabilis*	MYMCC903-11	JX836508
Sarcoptiformes	Ceratozetidae	*Diapterobates variabilis*	MYMCD034-11	JX836784
Sarcoptiformes	Ceratozetidae	*Diapterobates variabilis*	MYMCD158-11	JX836846
Sarcoptiformes	Ceratozetidae	*Diapterobates variabilis*	MYMCE430-12	JX838694
Sarcoptiformes	Ceratozetidae	*Diapterobates variabilis*	MYMCE574-12	JX835734
Sarcoptiformes	Ceratozetidae	*Diapterobates variabilis*	MYMCE714-12	JX833878
Sarcoptiformes	Ceratozetidae	*Diapterobates variabilis*	MYMCF092-12	JX836221
Sarcoptiformes	Ceratozetidae	*Diapterobates variabilis*	MYMCF132-12	JX834587
Sarcoptiformes	Ceratozetidae	*Diapterobates variabilis*	MYMCF157-12	JX833717
Sarcoptiformes	Ceratozetidae	*Diapterobates variabilis*	MYMCF304-12	JX834518
Sarcoptiformes	Ceratozetidae	*Diapterobates variabilis*	MYMCF616-12	JX834461
Sarcoptiformes	Ceratozetidae	*Diapterobates variabilis*	MYMCF739-12	JX833705
Sarcoptiformes	Ceratozetidae	*Diapterobates variabilis*	MYMCF740-12	JX834900
Sarcoptiformes	Ceratozetidae	*Diapterobates variabilis*	MYTMC059-09	HM431993
Sarcoptiformes	Ceratozetidae	*Fuscozetes*	MIONB043-10	HM887578
Sarcoptiformes	Ceratozetidae	*Fuscozetes*	MIONB044-10	HM887579
Sarcoptiformes	Ceratozetidae	*Fuscozetes*	MIONB045-10	HM887580
Sarcoptiformes	Ceratozetidae	*Fuscozetes*	MIONB046-10	HM887581
Sarcoptiformes	Ceratozetidae	*Fuscozetes*	MIONB080-10	HQ575093
Sarcoptiformes	Ceratozetidae	*Fuscozetes*	MIONB082-10	HQ575094
Sarcoptiformes	Ceratozetidae	*Fuscozetes fuscipes*	MIONB042-10	HM887577
Sarcoptiformes	Ceratozetidae	*Fuscozetes fuscipes*	MIONB083-10	HQ575095
Sarcoptiformes	Ceratozetidae	*Fuscozetes fuscipes*	SSEIB5800-13	KM828928
Sarcoptiformes	Ceratozetidae	*Fuscozetes fuscipes*	SSEIB8176-13	KM834684
Sarcoptiformes	Ceratozetidae	*Fuscozetes fuscipes*	SSEIB8208-13	KM834777
Sarcoptiformes	Ceratozetidae	*Ghilarovizetes longisetosus*	CHACA151-08	JX835704
Sarcoptiformes	Ceratozetidae	*Ghilarovizetes longisetosus*	CHACA976-10	HM405840
Sarcoptiformes	Ceratozetidae	*Ghilarovizetes longisetosus*	MYMCC121-11	JX837288
Sarcoptiformes	Ceratozetidae	*Ghilarovizetes longisetosus*	MYMCC122-11	JX834086
Sarcoptiformes	Ceratozetidae	*Ghilarovizetes longisetosus*	MYMCC123-11	JX837710
Sarcoptiformes	Ceratozetidae	*Ghilarovizetes longisetosus*	MYMCC392-11	JX836145
Sarcoptiformes	Ceratozetidae	*Ghilarovizetes longisetosus*	MYMCC393-11	JX834477
Sarcoptiformes	Ceratozetidae	*Ghilarovizetes longisetosus*	MYMCC799-11	JX833829
Sarcoptiformes	Ceratozetidae	*Ghilarovizetes longisetosus*	MYMCF423-12	JX836486
Sarcoptiformes	Ceratozetidae	*Ghilarovizetes longisetosus*	MYMCF424-12	JX835855
Sarcoptiformes	Ceratozetidae	*Ghilarovizetes longisetosus*	MYMCF874-12	JX834223
Sarcoptiformes	Ceratozetidae	*Ghilarovizetes longisetosus*	MYMCF893-12	JX836208
Sarcoptiformes	Ceratozetidae	*Jugatala*	CNBAA452-12	KM829382
Sarcoptiformes	Ceratozetidae	*Jugatala*	CNBAA455-12	KM825639

Sarcoptiformes	Ceratozetidae	*Jugatala*	CNBAA456-12	KM835755
Sarcoptiformes	Ceratozetidae	*Jugatala*	CNBAA459-12	KM834925
Sarcoptiformes	Ceratozetidae	*Jugatala*	CNBAA469-12	KM831471
Sarcoptiformes	Ceratozetidae	*Jugatala*	CNBAB376-12	KM830701
Sarcoptiformes	Ceratozetidae	*Jugatala*	CNBAC731-12	KM830815
Sarcoptiformes	Ceratozetidae	*Jugatala*	CNBAC732-12	KM840221
Sarcoptiformes	Ceratozetidae	*Jugatala*	CNBAF207-12	KM837864
Sarcoptiformes	Ceratozetidae	*Jugatala*	CNGLC086-13	KM826561
Sarcoptiformes	Ceratozetidae	*Jugatala*	CNGLD019-13	KM838589
Sarcoptiformes	Ceratozetidae	*Jugatala*	CNGLD022-13	KM826452
Sarcoptiformes	Ceratozetidae	*Jugatala*	CNGLD024-13	KM840791
Sarcoptiformes	Ceratozetidae	*Jugatala*	CNGLD027-13	KM825597
Sarcoptiformes	Ceratozetidae	*Jugatala*	CNGLE035-13	KM825377
Sarcoptiformes	Ceratozetidae	*Jugatala*	CNGLE036-13	KM833993
Sarcoptiformes	Ceratozetidae	*Jugatala*	CNGLE040-13	KM839143
Sarcoptiformes	Ceratozetidae	*Jugatala*	CNGLF132-13	KM840597
Sarcoptiformes	Ceratozetidae	*Jugatala*	CNJAB1008-12	KM840228
Sarcoptiformes	Ceratozetidae	*Jugatala*	SSBAA2053-12	KM835289
Sarcoptiformes	Ceratozetidae	*Jugatala*	SSBAB122-12	KM828569
Sarcoptiformes	Ceratozetidae	*Jugatala*	SSBAB127-12	KM837090
Sarcoptiformes	Ceratozetidae	*Jugatala*	SSBAE3016-13	KM827613
Sarcoptiformes	Ceratozetidae	*Jugatala*	SSBAE939-13	KM836246
Sarcoptiformes	Ceratozetidae	*Jugatala*	SSJAA777-13	KM829517
Sarcoptiformes	Ceratozetidae	*Jugatala*	SSWLA2288-13	KM834569
Sarcoptiformes	Ceratozetidae	*Jugatala*	SSWLC056-13	KM829021
Sarcoptiformes	Ceratozetidae	*Jugatala*	SSWLC057-13	KM833684
Sarcoptiformes	Ceratozetidae	*Jugatala*	SSWLC1152-13	KM829300
Sarcoptiformes	Ceratozetidae	*Jugatala*	SSWLC1184-13	KM830145
Sarcoptiformes	Ceratozetidae	*Jugatala*	SSWLC1186-13	KM832078
Sarcoptiformes	Ceratozetidae	*Lugoribates gracilis*	CHACA1076-10	HM907119
Sarcoptiformes	Ceratozetidae	*Lugoribates gracilis*	CHACA368-08	JX837384
Sarcoptiformes	Ceratozetidae	*Lugoribates gracilis*	CHACA975-10	HM405839
Sarcoptiformes	Ceratozetidae	*Lugoribates gracilis*	CHACB1024-10	HM907157
Sarcoptiformes	Ceratozetidae	*Lugoribates gracilis*	CHACB1025-10	HM907158
Sarcoptiformes	Ceratozetidae	*Lugoribates gracilis*	CHACB124-10	HQ558411
Sarcoptiformes	Ceratozetidae	*Lugoribates gracilis*	CHACC033-10	HM907434
Sarcoptiformes	Ceratozetidae	*Lugoribates gracilis*	CHACC034-10	HM907435
Sarcoptiformes	Ceratozetidae	*Lugoribates gracilis*	CHACC036-10	HM907436
Sarcoptiformes	Ceratozetidae	*Lugoribates gracilis*	CHACC037-10	HM907437
Sarcoptiformes	Ceratozetidae	*Lugoribates gracilis*	CHACC043-10	HM907443
Sarcoptiformes	Ceratozetidae	*Lugoribates gracilis*	CHACC060-10	HM907457
Sarcoptiformes	Ceratozetidae	*Lugoribates gracilis*	CHACC061-10	HM907458
Sarcoptiformes	Ceratozetidae	*Lugoribates gracilis*	CHACC062-10	HM907459
Sarcoptiformes	Ceratozetidae	*Lugoribates gracilis*	CHACC063-10	HM907460
Sarcoptiformes	Ceratozetidae	*Lugoribates gracilis*	CHACC238-10	HQ941546
Sarcoptiformes	Ceratozetidae	*Lugoribates gracilis*	MYMCA269-11	JX835288
Sarcoptiformes	Ceratozetidae	*Lugoribates gracilis*	MYMCA271-11	JX835375
Sarcoptiformes	Ceratozetidae	*Lugoribates gracilis*	MYMCB696-11	JX835634
Sarcoptiformes	Ceratozetidae	*Lugoribates gracilis*	MYMCD178-11	JX837240
Sarcoptiformes	Ceratozetidae	*Lugoribates gracilis*	MYMCE024-12	JX837224
Sarcoptiformes	Ceratozetidae	*Lugoribates gracilis*	MYMCE174-12	JX834586
Sarcoptiformes	Ceratozetidae	*Lugoribates gracilis*	MYMCE499-12	JX838136
Sarcoptiformes	Ceratozetidae	*Lugoribates gracilis*	MYMCF679-12	JX834602

Sarcoptiformes	Ceratozetidae	*Lugoribates gracilis*	MYMCF680-12	JX837660
Sarcoptiformes	Ceratozetidae	*Melanozetes*	CHACA685-09	JX837939
Sarcoptiformes	Ceratozetidae	*Melanozetes*	MYMCA349-11	JX834080
Sarcoptiformes	Ceratozetidae	*Melanozetes*	MYMCE234-12	JX837837
Sarcoptiformes	Ceratozetidae	*Melanozetes*	MYMCE235-12	JX835048
Sarcoptiformes	Ceratozetidae	*Melanozetes*	MYMCE236-12	JX836403
Sarcoptiformes	Ceratozetidae	*Neogymnobates luteus*	CHACA1203-10	JX835014
Sarcoptiformes	Ceratozetidae	*Neogymnobates luteus*	CHACB197-10	HQ558467
Sarcoptiformes	Ceratozetidae	*Neogymnobates luteus*	CHACB198-10	HQ558468
Sarcoptiformes	Ceratozetidae	*Neogymnobates luteus*	CHACB616-10	HQ558703
Sarcoptiformes	Ceratozetidae	*Neogymnobates luteus*	CHACB930-10	HM907357
Sarcoptiformes	Ceratozetidae	*Neogymnobates luteus*	CHACB931-10	HM907358
Sarcoptiformes	Ceratozetidae	*Neogymnobates luteus*	MYMCA1164-11	JX835213
Sarcoptiformes	Ceratozetidae	*Neogymnobates luteus*	MYMCA1183-11	JX834556
Sarcoptiformes	Ceratozetidae	*Neogymnobates luteus*	MYMCA300-11	JX838522
Sarcoptiformes	Ceratozetidae	*Neogymnobates luteus*	MYMCA555-11	JX838786
Sarcoptiformes	Ceratozetidae	*Neogymnobates luteus*	MYMCC147-11	JX837559
Sarcoptiformes	Ceratozetidae	*Neogymnobates luteus*	MYMCC360-11	JX836828
Sarcoptiformes	Ceratozetidae	*Neogymnobates luteus*	MYMCC423-11	JX833745
Sarcoptiformes	Ceratozetidae	*Neogymnobates luteus*	MYMCC552-11	JX833709
Sarcoptiformes	Ceratozetidae	*Neogymnobates luteus*	MYMCC553-11	JX838737
Sarcoptiformes	Ceratozetidae	*Neogymnobates luteus*	MYMCC790-11	JX836729
Sarcoptiformes	Ceratozetidae	*Neogymnobates luteus*	MYMCC932-11	JX833807
Sarcoptiformes	Ceratozetidae	*Neogymnobates luteus*	MYMCD160-11	JX833866
Sarcoptiformes	Ceratozetidae	*Neogymnobates luteus*	MYMCF025-12	JX836553
Sarcoptiformes	Ceratozetidae	*Neogymnobates luteus*	MYMCG545-12	JX834025
Sarcoptiformes	Ceratozetidae	*Neogymnobates luteus*	MYTMC057-09	HQ966230
Sarcoptiformes	Ceratozetidae	*Neogymnobates luteus*	MYTMC119-09	HQ966238
Sarcoptiformes	Ceratozetidae	*Neogymnobates luteus*	SSBAB115-12	KM827679
Sarcoptiformes	Ceratozetidae	*Neogymnobates luteus*	SSBAB1190-12	KM839481
Sarcoptiformes	Ceratozetidae	*Neogymnobates luteus*	SSBAB1874-12	KM837015
Sarcoptiformes	Ceratozetidae	*Neogymnobates luteus*	SSBAB1933-12	KM827109
Sarcoptiformes	Ceratozetidae	*Neogymnobates luteus*	SSJAA2001-13	KM831402
Sarcoptiformes	Ceratozetidae	*Neogymnobates luteus*	SSJAC1561-13	KM834135
Sarcoptiformes	Ceratozetidae	*Neogymnobates luteus*	SSJAC1568-13	KM828391
Sarcoptiformes	Ceratozetidae	*Neogymnobates luteus*	SSPAA7795-13	KM836225
Sarcoptiformes	Ceratozetidae	*Neogymnobates luteus*	SSPAA7800-13	KM830319
Sarcoptiformes	Ceratozetidae	*Neogymnobates luteus*	SSPAA7965-13	KM830076
Sarcoptiformes	Ceratozetidae	*Neogymnobates luteus*	SSPAA7974-13	KM833201
Sarcoptiformes	Ceratozetidae	*Svalbardia paludicola*	CHACA523-09	JX834122
Sarcoptiformes	Ceratozetidae	*Svalbardia paludicola*	CHACB751-10	HQ558785
Sarcoptiformes	Ceratozetidae	*Svalbardia paludicola*	CHACB754-10	HQ558786
Sarcoptiformes	Ceratozetidae	*Trichoribates*	ASAMT047-12	KP979326
Sarcoptiformes	Ceratozetidae	*Trichoribates*	CHACA568-09	JX835279
Sarcoptiformes	Ceratozetidae	*Trichoribates*	CHACB122-10	HQ558409
Sarcoptiformes	Ceratozetidae	*Trichoribates*	CHACB123-10	HQ558410
Sarcoptiformes	Ceratozetidae	*Trichoribates*	CHACB125-10	HQ558412
Sarcoptiformes	Ceratozetidae	*Trichoribates*	CHACB227-10	HQ558479
Sarcoptiformes	Ceratozetidae	*Trichoribates*	CHACB305-10	HM907296
Sarcoptiformes	Ceratozetidae	*Trichoribates*	CHACB306-10	HM907297
Sarcoptiformes	Ceratozetidae	*Trichoribates*	CHACB307-10	HM907298
Sarcoptiformes	Ceratozetidae	*Trichoribates*	CHACB436-10	HQ558592
Sarcoptiformes	Ceratozetidae	*Trichoribates*	CHACB437-10	HQ558593

Sarcoptiformes	Ceratozetidae	*Trichoribates*	CHACB444-10	HQ558598
Sarcoptiformes	Ceratozetidae	*Trichoribates*	CHACB485-10	HQ558620
Sarcoptiformes	Ceratozetidae	*Trichoribates*	CNRMD2664-12	KM831721
Sarcoptiformes	Ceratozetidae	*Trichoribates*	MYMCA001-11	JX835147
Sarcoptiformes	Ceratozetidae	*Trichoribates*	MYMCA002-11	JX834252
Sarcoptiformes	Ceratozetidae	*Trichoribates*	MYMCA003-11	JX838717
Sarcoptiformes	Ceratozetidae	*Trichoribates*	MYMCA005-11	JX834353
Sarcoptiformes	Ceratozetidae	*Trichoribates*	MYMCA1041-11	JX836453
Sarcoptiformes	Ceratozetidae	*Trichoribates*	MYMCA1337-11	JX834770
Sarcoptiformes	Ceratozetidae	*Trichoribates*	MYMCA1389-11	JX835310
Sarcoptiformes	Ceratozetidae	*Trichoribates*	MYMCA1391-11	JX837106
Sarcoptiformes	Ceratozetidae	*Trichoribates*	MYMCA158-11	JX838162
Sarcoptiformes	Ceratozetidae	*Trichoribates*	MYMCA159-11	JX833637
Sarcoptiformes	Ceratozetidae	*Trichoribates*	MYMCA160-11	JX836258
Sarcoptiformes	Ceratozetidae	*Trichoribates*	MYMCA270-11	JX836833
Sarcoptiformes	Ceratozetidae	*Trichoribates*	MYMCA596-11	JX835455
Sarcoptiformes	Ceratozetidae	*Trichoribates*	MYMCA597-11	JX837519
Sarcoptiformes	Ceratozetidae	*Trichoribates*	MYMCA598-11	JX834098
Sarcoptiformes	Ceratozetidae	*Trichoribates*	MYMCA627-11	JX835395
Sarcoptiformes	Ceratozetidae	*Trichoribates*	MYMCA646-11	JX837032
Sarcoptiformes	Ceratozetidae	*Trichoribates*	MYMCA647-11	JX834398
Sarcoptiformes	Ceratozetidae	*Trichoribates*	MYMCA648-11	JX837727
Sarcoptiformes	Ceratozetidae	*Trichoribates*	MYMCA653-11	JX834690
Sarcoptiformes	Ceratozetidae	*Trichoribates*	MYMCA672-11	JX834573
Sarcoptiformes	Ceratozetidae	*Trichoribates*	MYMCA673-11	JX836987
Sarcoptiformes	Ceratozetidae	*Trichoribates*	MYMCA674-11	JX834179
Sarcoptiformes	Ceratozetidae	*Trichoribates*	MYMCA675-11	JX837803
Sarcoptiformes	Ceratozetidae	*Trichoribates*	MYMCA687-11	JX836896
Sarcoptiformes	Ceratozetidae	*Trichoribates*	MYMCA730-11	JX838644
Sarcoptiformes	Ceratozetidae	*Trichoribates*	MYMCB009-11	JX834968
Sarcoptiformes	Ceratozetidae	*Trichoribates*	MYMCB014-11	JX834904
Sarcoptiformes	Ceratozetidae	*Trichoribates*	MYMCB015-11	JX837929
Sarcoptiformes	Ceratozetidae	*Trichoribates*	MYMCB023-11	JX837371
Sarcoptiformes	Ceratozetidae	*Trichoribates*	MYMCB412-11	JX838434
Sarcoptiformes	Ceratozetidae	*Trichoribates*	MYMCB944-11	JX834995
Sarcoptiformes	Ceratozetidae	*Trichoribates*	MYMCB945-11	JX836671
Sarcoptiformes	Ceratozetidae	*Trichoribates*	MYMCB946-11	JX834984
Sarcoptiformes	Ceratozetidae	*Trichoribates*	MYMCC008-11	JX836389
Sarcoptiformes	Ceratozetidae	*Trichoribates*	MYMCC009-11	JX837617
Sarcoptiformes	Ceratozetidae	*Trichoribates*	MYMCC042-11	JX835881
Sarcoptiformes	Ceratozetidae	*Trichoribates*	MYMCC043-11	JX835093
Sarcoptiformes	Ceratozetidae	*Trichoribates*	MYMCC120-11	JX836724
Sarcoptiformes	Ceratozetidae	*Trichoribates*	MYMCC298-11	JX835420
Sarcoptiformes	Ceratozetidae	*Trichoribates*	MYMCC427-11	JX838569
Sarcoptiformes	Ceratozetidae	*Trichoribates*	MYMCC428-11	JX836215
Sarcoptiformes	Ceratozetidae	*Trichoribates*	MYMCC818-11	JX833737
Sarcoptiformes	Ceratozetidae	*Trichoribates*	MYMCC819-11	JX837140
Sarcoptiformes	Ceratozetidae	*Trichoribates*	MYMCD049-11	JX835005
Sarcoptiformes	Ceratozetidae	*Trichoribates*	MYMCD050-11	JX837640
Sarcoptiformes	Ceratozetidae	*Trichoribates*	MYMCD076-11	JX834889
Sarcoptiformes	Ceratozetidae	*Trichoribates*	MYMCD077-11	JX836513
Sarcoptiformes	Ceratozetidae	*Trichoribates*	MYMCD097-11	JX834054
Sarcoptiformes	Ceratozetidae	*Trichoribates*	MYMCD098-11	JX838667

Sarcoptiformes	Ceratozetidae	*Trichoribates*	MYMCD116-11	JX837078
Sarcoptiformes	Ceratozetidae	*Trichoribates*	MYMCD132-11	JX836974
Sarcoptiformes	Ceratozetidae	*Trichoribates*	MYMCD177-11	JX837276
Sarcoptiformes	Ceratozetidae	*Trichoribates*	MYMCD179-11	JX838524
Sarcoptiformes	Ceratozetidae	*Trichoribates*	MYMCE076-12	JX837047
Sarcoptiformes	Ceratozetidae	*Trichoribates*	MYMCE078-12	JX835710
Sarcoptiformes	Ceratozetidae	*Trichoribates*	MYMCE108-12	JX836392
Sarcoptiformes	Ceratozetidae	*Trichoribates*	MYMCE424-12	JX834368
Sarcoptiformes	Ceratozetidae	*Trichoribates*	MYMCE553-12	JX838306
Sarcoptiformes	Ceratozetidae	*Trichoribates*	MYMCG198-12	JX834746
Sarcoptiformes	Ceratozetidae	*Trichoribates*	MYMCG199-12	JX835940
Sarcoptiformes	Ceratozetidae	*Trichoribates*	SMTPB668-13	KR069198
Sarcoptiformes	Ceratozetidae	*Trichoribates*	SMTPB670-13	KR070018
Sarcoptiformes	Ceratozetidae	*Trichoribates*	SMTPB8376-13	KR069171
Sarcoptiformes	Ceratozetidae	*Trichoribates*	SSEIB4547-13	KM836223
Sarcoptiformes	Ceratozetidae	*Trichoribates*	SSEIB4580-13	KM834997
Sarcoptiformes	Ceratozetidae	*Trichoribates*	SSEIB5414-13	KM832283
Sarcoptiformes	Ceratozetidae	*Trichoribates*	SSEIB5418-13	KM838385
Sarcoptiformes	Ceratozetidae	*Trichoribates*	SSEIB5802-13	KM836495
Sarcoptiformes	Ceratozetidae	*Trichoribates*	SSEIB5804-13	KM834078
Sarcoptiformes	Ceratozetidae	*Trichoribates*	SSEIB5809-13	KM827790
Sarcoptiformes	Ceratozetidae	*Trichoribates*	SSPAA6668-13	KM839160
Sarcoptiformes	Ceratozetidae	*Trichoribates*	SSPAA6675-13	KM837143
Sarcoptiformes	Ceratozetidae	*Trichoribates*	SSPAA6701-13	KM838839
Sarcoptiformes	Ceratozetidae	*Trichoribates*	SSPAA7747-13	KM824784
Sarcoptiformes	Ceratozetidae	*Trichoribates*	SSPAA7779-13	KM836272
Sarcoptiformes	Ceratozetidae	*Trichoribates*	SSPAA7963-13	KM833984
Sarcoptiformes	Ceratozetidae	*Trichoribates*	SSPAA7966-13	KM828941
Sarcoptiformes	Ceratozetidae		CHACA525-09	JX834757
Sarcoptiformes	Ceratozetidae		CHACB354-10	HQ558535
Sarcoptiformes	Ceratozetidae		CHACB654-10	JX836653
Sarcoptiformes	Ceratozetidae		CHACB698-10	HQ558755
Sarcoptiformes	Ceratozetidae		CHACB752-10	JX833894
Sarcoptiformes	Ceratozetidae		CHACB753-10	JX834007
Sarcoptiformes	Ceratozetidae		CHACB775-10	HQ941480
Sarcoptiformes	Ceratozetidae		CNBPC217-12	KM833467
Sarcoptiformes	Ceratozetidae		CNBPD588-12	KM836330
Sarcoptiformes	Ceratozetidae		CNBPD607-12	KM833051
Sarcoptiformes	Ceratozetidae		CNBPE658-12	KM838032
Sarcoptiformes	Ceratozetidae		CNBPL294-13	KM834427
Sarcoptiformes	Ceratozetidae		CNGLC081-13	KM828206
Sarcoptiformes	Ceratozetidae		CNGLD017-13	KM824872
Sarcoptiformes	Ceratozetidae		CNPAH426-13	KM826320
Sarcoptiformes	Ceratozetidae		CNPCA041-13	KM827020
Sarcoptiformes	Ceratozetidae		CNPCC060-13	KM830479
Sarcoptiformes	Ceratozetidae		CNPCC063-13	KM830573
Sarcoptiformes	Ceratozetidae		CNPCD081-13	KM830038
Sarcoptiformes	Ceratozetidae		CNPCD083-13	KM833585
Sarcoptiformes	Ceratozetidae		CNPCD084-13	KM833671
Sarcoptiformes	Ceratozetidae		CNPCD094-13	KM828234
Sarcoptiformes	Ceratozetidae		CNPCD101-13	KM838907
Sarcoptiformes	Ceratozetidae		CNPCD124-13	KM824481
Sarcoptiformes	Ceratozetidae		CNPCD134-13	KM840447

Sarcoptiformes	Ceratozetidae	CNPCE097-13	KM835488
Sarcoptiformes	Ceratozetidae	CNPCF104-13	KM830958
Sarcoptiformes	Ceratozetidae	CNPCF113-13	KM837738
Sarcoptiformes	Ceratozetidae	CNPCF126-13	KM834796
Sarcoptiformes	Ceratozetidae	CNPCF130-13	KM824337
Sarcoptiformes	Ceratozetidae	CNPCF135-13	KM837057
Sarcoptiformes	Ceratozetidae	CNPCF138-13	KM838966
Sarcoptiformes	Ceratozetidae	CNRME4771-12	KM832863
Sarcoptiformes	Ceratozetidae	MIAUS002-12	KP979328
Sarcoptiformes	Ceratozetidae	MIAUS004-12	KR069645
Sarcoptiformes	Ceratozetidae	MIAUS009-12	KR070070
Sarcoptiformes	Ceratozetidae	MIAUS010-12	KP979274
Sarcoptiformes	Ceratozetidae	MIAUS011-12	KR070423
Sarcoptiformes	Ceratozetidae	MIAUS013-12	KP979302
Sarcoptiformes	Ceratozetidae	MIAUS014-12	KP979152
Sarcoptiformes	Ceratozetidae	MIONB199-10	KR069420
Sarcoptiformes	Ceratozetidae	MIONB200-10	KP979173
Sarcoptiformes	Ceratozetidae	MYMCB453-11	JX836167
Sarcoptiformes	Ceratozetidae	MYMCB697-11	JX837103
Sarcoptiformes	Ceratozetidae	MYMCC479-11	JX838751
Sarcoptiformes	Ceratozetidae	MYMCC771-11	JX835159
Sarcoptiformes	Ceratozetidae	MYMCD020-11	JX838672
Sarcoptiformes	Ceratozetidae	MYMCE199-12	JX837405
Sarcoptiformes	Ceratozetidae	MYMCE307-12	JX838284
Sarcoptiformes	Ceratozetidae	MYMCF366-12	JX834931
Sarcoptiformes	Ceratozetidae	MYMCF706-12	JX833779
Sarcoptiformes	Ceratozetidae	MYMCF707-12	JX836225
Sarcoptiformes	Ceratozetidae	SSBAA3680-12	KM824866
Sarcoptiformes	Ceratozetidae	SSBAA3689-12	KM834699
Sarcoptiformes	Ceratozetidae	SSBAB1930-12	KM832839
Sarcoptiformes	Ceratozetidae	SSBAD3135-12	KM825326
Sarcoptiformes	Ceratozetidae	SSBAD3137-12	KM825894
Sarcoptiformes	Ceratozetidae	SSBAD3138-12	KM837867
Sarcoptiformes	Ceratozetidae	SSBAD3963-12	KM825395
Sarcoptiformes	Ceratozetidae	SSBAD3970-12	KM836763
Sarcoptiformes	Ceratozetidae	SSBAD3979-12	KM834479
Sarcoptiformes	Ceratozetidae	SSBAD3980-12	KM835165
Sarcoptiformes	Ceratozetidae	SSBAD3982-12	KM840174
Sarcoptiformes	Ceratozetidae	SSBAD3997-12	KM830549
Sarcoptiformes	Ceratozetidae	SSBAD4002-12	KM829797
Sarcoptiformes	Ceratozetidae	SSBAD4701-13	KM825799
Sarcoptiformes	Ceratozetidae	SSBAD4726-13	KM827814
Sarcoptiformes	Ceratozetidae	SSBAD4771-13	KM833607
Sarcoptiformes	Ceratozetidae	SSBAD5572-13	KM827886
Sarcoptiformes	Ceratozetidae	SSBAE3328-13	KM840701
Sarcoptiformes	Ceratozetidae	SSBAE3330-13	KM829271
Sarcoptiformes	Ceratozetidae	SSBAE3627-13	KM828690
Sarcoptiformes	Ceratozetidae	SSJAA2031-13	KM827366
Sarcoptiformes	Ceratozetidae	SSJAB1915-13	KM826521
Sarcoptiformes	Ceratozetidae	SSPAA6653-13	KM826281
Sarcoptiformes	Ceratozetidae	SSPAA6660-13	KM839322
Sarcoptiformes	Ceratozetidae	SSPAA6683-13	KM838875
Sarcoptiformes	Ceratozetidae	SSPAA7805-13	KM824497

Sarcoptiformes	Ceratozetidae		SSPAA7808-13	KM825779
Sarcoptiformes	Ceratozetidae		SSPAA7811-13	KM840641
Sarcoptiformes	Ceratozetidae		SSPAA7812-13	KM825707
Sarcoptiformes	Ceratozetidae		SSWLA032-13	KM824002
Sarcoptiformes	Ceratozetidae		SSWLA037-13	KM830029
Sarcoptiformes	Ceratozetidae		SSWLC061-13	KM826685
Sarcoptiformes	Ceratozetidae		SSWLC064-13	KM834432
Sarcoptiformes	Ceratozetidae		SSWLC066-13	KM826420
Sarcoptiformes	Ceratozetidae		SSWLC1151-13	KM828109
Sarcoptiformes	Ceratozetidae		SSWLC1154-13	KM833549
Sarcoptiformes	Ceratozetidae		SSWLC1155-13	KM825974
Sarcoptiformes	Ceratozetidae		SSWLC1156-13	KM840748
Sarcoptiformes	Ceratozetidae		SSWLC1157-13	KM838459
Sarcoptiformes	Ceratozetidae		SSWLC1160-13	KM832117
Sarcoptiformes	Ceratozetidae		SSWLC1161-13	KM829832
Sarcoptiformes	Ceratozetidae		SSWLC1169-13	KM831935
Sarcoptiformes	Ceratozetidae		SSWLC1181-13	KM826373
Sarcoptiformes	Ceratozetidae		SSWLC1185-13	KM827036
Sarcoptiformes	Ceratozetidae		SSWLC1187-13	KM828019
Sarcoptiformes	Ceratozetidae		SSWLC2254-13	KM828954
Sarcoptiformes	Ceratozetidae		SSWLC2258-13	KM827800
Sarcoptiformes	Chamobatidae	*Chamobates*	CNGMG1628-14	KR070312
Sarcoptiformes	Chamobatidae	*Chamobates*	CNKOG1265-14	KR069768
Sarcoptiformes	Chamobatidae	*Chamobates*	CNKOH1915-14	KR069786
Sarcoptiformes	Chamobatidae	*Chamobates*	CNKOH1921-14	KR069421
Sarcoptiformes	Chamobatidae	*Chamobates*	CNKOR1650-14	KR069178
Sarcoptiformes	Chamobatidae	*Chamobates*	CNKOT249-14	KR070685
Sarcoptiformes	Chamobatidae	*Chamobates*	CNPEQ748-14	KR070262
Sarcoptiformes	Chamobatidae	*Chamobates*	CNPER345-14	KR070387
Sarcoptiformes	Chamobatidae	*Chamobates*	SSEIB4557-13	KM839892
Sarcoptiformes	Chamobatidae	*Chamobates*	SSPAA8268-13	KM837335
Sarcoptiformes	Chamobatidae	*Chamobates*	SSPAA8423-13	KM832485
Sarcoptiformes	Chamobatidae	*Chamobates*	SSPAB7999-13	KM826066
Sarcoptiformes	Chamobatidae	*Chamobates*	SSPAB8706-13	KM840249
Sarcoptiformes	Chamobatidae	*Chamobates*	SSPAC10344-13	KM840560
Sarcoptiformes	Chamobatidae	*Chamobates*	SSPAC10349-13	KM828820
Sarcoptiformes	Chamobatidae	*Chamobates*	SSPAC10870-13	KM830291
Sarcoptiformes	Chamobatidae	*Chamobates*	SSPAC10880-13	KM836112
Sarcoptiformes	Chamobatidae	*Chamobates*	SSPAC10891-13	KM828402
Sarcoptiformes	Chamobatidae	*Chamobates*	SSPAC10909-13	KM831037
Sarcoptiformes	Chamobatidae	*Chamobates*	SSPAC10915-13	KM838548
Sarcoptiformes	Chamobatidae	*Chamobates*	SSPAC10928-13	KM825198
Sarcoptiformes	Chamobatidae	*Chamobates*	SSPAC11064-13	KM832216
Sarcoptiformes	Chamobatidae	*Chamobates*	SSPAC11092-13	KM824992
Sarcoptiformes	Chamobatidae	*Chamobates*	SSPAC12210-13	KM827974
Sarcoptiformes	Chamobatidae	*Chamobates*	SSPAC12255-13	KM827293
Sarcoptiformes	Chamobatidae	*Chamobates*	SSPAC12266-13	KM829321
Sarcoptiformes	Chamobatidae	*Chamobates*	SSPAC12269-13	KM839252
Sarcoptiformes	Chamobatidae	*Chamobates*	SSPAC12270-13	KM834525
Sarcoptiformes	Chamobatidae	*Chamobates*	SSPAC12289-13	KM830546
Sarcoptiformes	Chamobatidae	*Chamobates*	SSPAC12304-13	KM840395
Sarcoptiformes	Chamobatidae	*Chamobates cuspidatus*	SSEIA2102-13	KM827442
Sarcoptiformes	Chamobatidae	*Chamobates cuspidatus*	SSEIB4569-13	KM837928

Sarcoptiformes	Chamobatidae	*Chamobates cuspidatus*	SSEIB4571-13	KM829229
Sarcoptiformes	Chamobatidae	*Chamobates cuspidatus*	SSEIB5412-13	KM825750
Sarcoptiformes	Chamobatidae	*Chamobates cuspidatus*	SSEIB5423-13	KM832081
Sarcoptiformes	Chamobatidae	*Chamobates cuspidatus*	SSEIB5428-13	KM836416
Sarcoptiformes	Chamobatidae	*Chamobates cuspidatus*	SSEIB5792-13	KM827931
Sarcoptiformes	Chamobatidae	*Chamobates cuspidatus*	SSEIB5794-13	KM834993
Sarcoptiformes	Chamobatidae	*Chamobates cuspidatus*	SSEIB5805-13	KM827650
Sarcoptiformes	Chamobatidae	*Chamobates cuspidatus*	SSEIB5807-13	KM833839
Sarcoptiformes	Chamobatidae	*Chamobates*	SSBRC3348-14	KR070041
Sarcoptiformes	Crotoniidae	*Camisia*	SSGBA5100-14	KR069857
Sarcoptiformes	Crotoniidae	*Camisia*	CHACA204-08	JX836165
Sarcoptiformes	Crotoniidae	*Camisia*	CHACA283-08	JX836942
Sarcoptiformes	Crotoniidae	*Camisia*	CNGIB594-12	KM833294
Sarcoptiformes	Crotoniidae	*Camisia*	CNPCD117-13	KM835918
Sarcoptiformes	Crotoniidae	*Camisia*	MYMCB259-11	JX838342
Sarcoptiformes	Crotoniidae	*Camisia*	MYMCB260-11	JX834797
Sarcoptiformes	Crotoniidae	*Camisia*	MYMCB929-11	JX833752
Sarcoptiformes	Crotoniidae	*Camisia*	MYTMC070-09	HM431996
Sarcoptiformes	Crotoniidae	*Camisia*	SSPAA9754-13	KM832943
Sarcoptiformes	Crotoniidae	*Camisia*	SSWLC059-13	KM824322
Sarcoptiformes	Crotoniidae	*Camisia*	SSWLC1165-13	KM836576
Sarcoptiformes	Crotoniidae	*Camisia*	SSWLC1172-13	KM831644
Sarcoptiformes	Crotoniidae	*Camisia*	SSWLC1182-13	KM839494
Sarcoptiformes	Crotoniidae	*Camisia*	SSWLC3129-13	KM840140
Sarcoptiformes	Crotoniidae	*Camisia*	SSWLD8140-13	KM840817
Sarcoptiformes	Crotoniidae	*Camisia biurus*	CHACB979-10	HM907388
Sarcoptiformes	Crotoniidae	*Camisia biurus*	MYMCC083-11	JX833714
Sarcoptiformes	Crotoniidae	*Camisia biurus*	MYMCC084-11	JX837763
Sarcoptiformes	Crotoniidae	*Camisia biurus*	MYMCC899-11	JX837550
Sarcoptiformes	Crotoniidae	*Camisia biurus*	MYMCF662-12	JX838744
Sarcoptiformes	Crotoniidae	*Camisia biurus*	MYMCF663-12	JX836915
Sarcoptiformes	Crotoniidae	*Camisia biverrucata*	CHACA180-08	JX837006
Sarcoptiformes	Crotoniidae	*Camisia biverrucata*	CHACA339-08	JX837953
Sarcoptiformes	Crotoniidae	*Camisia biverrucata*	CHACA810-09	JX834551
Sarcoptiformes	Crotoniidae	*Camisia biverrucata*	CHACB048-10	HQ558359
Sarcoptiformes	Crotoniidae	*Camisia biverrucata*	CHACB521-10	JX834102
Sarcoptiformes	Crotoniidae	*Camisia biverrucata*	CHACB523-10	JX838028
Sarcoptiformes	Crotoniidae	*Camisia biverrucata*	CHACB730-10	JX837715
Sarcoptiformes	Crotoniidae	*Camisia biverrucata*	CHACB731-10	JX837454
Sarcoptiformes	Crotoniidae	*Camisia biverrucata*	CHACB732-10	JX837525
Sarcoptiformes	Crotoniidae	*Camisia biverrucata*	MYMCB845-11	JX835033
Sarcoptiformes	Crotoniidae	*Camisia biverrucata*	MYMCC017-11	JX837819
Sarcoptiformes	Crotoniidae	*Camisia biverrucata*	MYMCC066-11	JX837139
Sarcoptiformes	Crotoniidae	*Camisia biverrucata*	MYMCC092-11	JX836367
Sarcoptiformes	Crotoniidae	*Camisia biverrucata*	MYMCC248-11	JX834528
Sarcoptiformes	Crotoniidae	*Camisia biverrucata*	MYTMC049-09	KR069343
Sarcoptiformes	Crotoniidae	*Camisia biverrucata*	MYTMC055-09	KR069743
Sarcoptiformes	Crotoniidae	*Camisia biverrucata*	MYTMC071-09	KR070021
Sarcoptiformes	Crotoniidae	*Camisia biverrucata*	MYTMC169-09	KR069404
Sarcoptiformes	Crotoniidae	*Camisia biverrucata*	SSBAA3681-12	KM838175
Sarcoptiformes	Crotoniidae	*Camisia biverrucata*	SSBAA3686-12	KM824235
Sarcoptiformes	Crotoniidae	*Camisia biverrucata*	SSBAC2355-12	KM825741
Sarcoptiformes	Crotoniidae	*Camisia biverrucata*	SSJAB1918-13	KM825789

Sarcoptiformes	Crotoniidae	*Camisia biverrucata*	SSJAB4114-13	KM826277
Sarcoptiformes	Crotoniidae	*Camisia biverrucata*	SSWLB3665-13	KM826099
Sarcoptiformes	Crotoniidae	*Camisia biverrucata*	SSWLB3668-13	KM837556
Sarcoptiformes	Crotoniidae	*Camisia biverrucata*	SSWLB5365-13	KM832257
Sarcoptiformes	Crotoniidae	*Camisia biverrucata*	SSWLB6801-13	KM832609
Sarcoptiformes	Crotoniidae	*Camisia horrida*	CHACB1136-10	HM907226
Sarcoptiformes	Crotoniidae	*Camisia horrida*	CHACB1182-10	HM907257
Sarcoptiformes	Crotoniidae	*Camisia horrida*	CHACB1183-10	HM907258
Sarcoptiformes	Crotoniidae	*Camisia horrida*	CHACB1184-10	HM907259
Sarcoptiformes	Crotoniidae	*Camisia horrida*	CHACB1185-10	HM907260
Sarcoptiformes	Crotoniidae	*Camisia horrida*	CHACB1186-10	HM907261
Sarcoptiformes	Crotoniidae	*Camisia horrida*	CHACB1187-10	HM907262
Sarcoptiformes	Crotoniidae	*Camisia horrida*	CHACB1188-10	HM907263
Sarcoptiformes	Crotoniidae	*Camisia horrida*	CHACB1189-10	HM907264
Sarcoptiformes	Crotoniidae	*Camisia horrida*	CHACB1190-10	HM907265
Sarcoptiformes	Crotoniidae	*Camisia horrida*	CHACB1191-10	HM907266
Sarcoptiformes	Crotoniidae	*Camisia horrida*	CHACB1192-10	HM907267
Sarcoptiformes	Crotoniidae	*Camisia horrida*	CHACC174-10	JX833900
Sarcoptiformes	Crotoniidae	*Camisia lapponica*	CHACA006-08	JX834554
Sarcoptiformes	Crotoniidae	*Camisia lapponica*	CHACA144-08	JX835919
Sarcoptiformes	Crotoniidae	*Camisia lapponica*	CHACA165-08	JX834864
Sarcoptiformes	Crotoniidae	*Camisia lapponica*	CHACA546-09	JX833920
Sarcoptiformes	Crotoniidae	*Camisia lapponica*	CHACA547-09	JX835044
Sarcoptiformes	Crotoniidae	*Camisia lapponica*	CHACA578-09	JX836630
Sarcoptiformes	Crotoniidae	*Camisia lapponica*	CHACB470-10	HQ558609
Sarcoptiformes	Crotoniidae	*Camisia lapponica*	CHACB471-10	HQ558610
Sarcoptiformes	Crotoniidae	*Camisia lapponica*	CHACB472-10	HQ558611
Sarcoptiformes	Crotoniidae	*Camisia lapponica*	CHACB899-10	HM907332
Sarcoptiformes	Crotoniidae	*Camisia lapponica*	CHACC018-10	HM907419
Sarcoptiformes	Crotoniidae	*Camisia lapponica*	CHACC019-10	HM907420
Sarcoptiformes	Crotoniidae	*Camisia lapponica*	CHACC020-10	HM907421
Sarcoptiformes	Crotoniidae	*Camisia lapponica*	CHACC123-10	JX837158
Sarcoptiformes	Crotoniidae	*Camisia lapponica*	MHMIT069-07	KR069192
Sarcoptiformes	Crotoniidae	*Camisia lapponica*	MYMCA146-11	JX836641
Sarcoptiformes	Crotoniidae	*Camisia lapponica*	MYMCA1505-11	JX836209
Sarcoptiformes	Crotoniidae	*Camisia lapponica*	MYMCA1506-11	JX836804
Sarcoptiformes	Crotoniidae	*Camisia lapponica*	MYMCA1507-11	JX837575
Sarcoptiformes	Crotoniidae	*Camisia lapponica*	MYMCA1508-11	JX834890
Sarcoptiformes	Crotoniidae	*Camisia lapponica*	MYMCA373-11	JX835619
Sarcoptiformes	Crotoniidae	*Camisia lapponica*	MYMCA601-11	JX837275
Sarcoptiformes	Crotoniidae	*Camisia lapponica*	MYMCA624-11	JX834491
Sarcoptiformes	Crotoniidae	*Camisia lapponica*	MYMCA625-11	JX836243
Sarcoptiformes	Crotoniidae	*Camisia lapponica*	MYMCA626-11	JX836018
Sarcoptiformes	Crotoniidae	*Camisia lapponica*	MYMCA676-11	JX838095
Sarcoptiformes	Crotoniidae	*Camisia lapponica*	MYMCA696-11	JX835384
Sarcoptiformes	Crotoniidae	*Camisia lapponica*	MYMCA717-11	JX833892
Sarcoptiformes	Crotoniidae	*Camisia lapponica*	MYMCA718-11	JX833855
Sarcoptiformes	Crotoniidae	*Camisia lapponica*	MYMCA719-11	JX834124
Sarcoptiformes	Crotoniidae	*Camisia lapponica*	MYMCA720-11	JX838417
Sarcoptiformes	Crotoniidae	*Camisia lapponica*	MYMCA721-11	JX835013
Sarcoptiformes	Crotoniidae	*Camisia lapponica*	MYMCF297-12	JX838250
Sarcoptiformes	Crotoniidae	*Camisia lapponica*	MYMCF820-12	JX835229
Sarcoptiformes	Crotoniidae	*Camisia lapponica*	MYTMC030-09	HM431992

Sarcoptiformes	Crotoniidae	*Heminothrus*	CHACA041-08	JX836164
Sarcoptiformes	Crotoniidae	*Heminothrus*	MYMCA077-11	JX836158
Sarcoptiformes	Crotoniidae	*Heminothrus*	MYMCA079-11	JX835303
Sarcoptiformes	Crotoniidae	*Heminothrus*	MYMCC302-11	JX836599
Sarcoptiformes	Crotoniidae	*Heminothrus*	MYTMC079-09	GU680461
Sarcoptiformes	Crotoniidae	*Heminothrus*	MYTMC094-09	GU680455
Sarcoptiformes	Crotoniidae	*Heminothrus*	MYTMC097-09	GU680496
Sarcoptiformes	Crotoniidae	*Heminothrus longisetosus*	MYMCA1147-11	JX837605
Sarcoptiformes	Crotoniidae	*Heminothrus longisetosus*	MYMCA1148-11	JX835733
Sarcoptiformes	Crotoniidae	*Heminothrus longisetosus*	MYMCA1181-11	JX836948
Sarcoptiformes	Crotoniidae	*Heminothrus longisetosus*	MYMCA481-11	JX835230
Sarcoptiformes	Crotoniidae	*Heminothrus longisetosus*	MYMCA482-11	JX838165
Sarcoptiformes	Crotoniidae	*Heminothrus longisetosus*	MYMCB035-11	JX835641
Sarcoptiformes	Crotoniidae	*Heminothrus longisetosus*	MYMCB490-11	JX838681
Sarcoptiformes	Crotoniidae	*Heminothrus longisetosus*	MYMCB491-11	JX835106
Sarcoptiformes	Crotoniidae	*Heminothrus longisetosus*	MYMCB589-11	JX833707
Sarcoptiformes	Crotoniidae	*Heminothrus longisetosus*	MYMCB590-11	JX838581
Sarcoptiformes	Crotoniidae	*Heminothrus longisetosus*	MYMCB591-11	JX837423
Sarcoptiformes	Crotoniidae	*Heminothrus longisetosus*	MYMCB625-11	JX835845
Sarcoptiformes	Crotoniidae	*Heminothrus longisetosus*	MYMCB663-11	JX834221
Sarcoptiformes	Crotoniidae	*Heminothrus longisetosus*	MYMCB731-11	JX836731
Sarcoptiformes	Crotoniidae	*Heminothrus longisetosus*	MYMCB732-11	JX834772
Sarcoptiformes	Crotoniidae	*Heminothrus longisetosus*	MYMCC183-11	JX836823
Sarcoptiformes	Crotoniidae	*Heminothrus longisetosus*	MYMCC184-11	JX838125
Sarcoptiformes	Crotoniidae	*Heminothrus longisetosus*	MYMCC185-11	JX835143
Sarcoptiformes	Crotoniidae	*Heminothrus longisetosus*	MYMCC214-11	JX838776
Sarcoptiformes	Crotoniidae	*Heminothrus longisetosus*	MYMCC215-11	JX834385
Sarcoptiformes	Crotoniidae	*Heminothrus longisetosus*	MYMCC281-11	JX835950
Sarcoptiformes	Crotoniidae	*Heminothrus longisetosus*	MYMCE627-12	JX836773
Sarcoptiformes	Crotoniidae	*Heminothrus longisetosus*	MYMCE870-12	JX837549
Sarcoptiformes	Crotoniidae	*Heminothrus longisetosus*	MYTMC162-09	GU680432
Sarcoptiformes	Crotoniidae	*Heminothrus longisetosus*	MYTMC170-09	GU680428
Sarcoptiformes	Crotoniidae	*Heminothrus longisetosus*	MYTMC183-09	GU680430
Sarcoptiformes	Crotoniidae	*Heminothrus longisetosus*	MYTMC184-09	GU680431
Sarcoptiformes	Crotoniidae	*Heminothrus thori*	MITMH015-07	KP979191
Sarcoptiformes	Crotoniidae	*Neonothrus humicola*	CHACA051-08	JX838705
Sarcoptiformes	Crotoniidae	*Neonothrus humicola*	CHACA052-08	JX837398
Sarcoptiformes	Crotoniidae	*Neonothrus humicola*	CHACA053-08	JX834833
Sarcoptiformes	Crotoniidae	*Neonothrus humicola*	CHACA054-08	JX838388
Sarcoptiformes	Crotoniidae	*Neonothrus humicola*	CHACA055-08	JX834806
Sarcoptiformes	Crotoniidae	*Neonothrus humicola*	CHACA056-08	JX838109
Sarcoptiformes	Crotoniidae	*Neonothrus humicola*	CHACA059-08	JX835776
Sarcoptiformes	Crotoniidae	*Neonothrus humicola*	CHACA062-08	JX837504
Sarcoptiformes	Crotoniidae	*Neonothrus humicola*	CHACA491-09	JX835915
Sarcoptiformes	Crotoniidae	*Neonothrus humicola*	CHACA502-09	JX834291
Sarcoptiformes	Crotoniidae	*Neonothrus humicola*	CHACA505-09	JX833861
Sarcoptiformes	Crotoniidae	*Neonothrus humicola*	CHACB332-10	HQ558514
Sarcoptiformes	Crotoniidae	*Neonothrus humicola*	CHACB333-10	HQ558515
Sarcoptiformes	Crotoniidae	*Neonothrus humicola*	CHACB334-10	HQ558516
Sarcoptiformes	Crotoniidae	*Neonothrus humicola*	CHACB335-10	HQ558517
Sarcoptiformes	Crotoniidae	*Neonothrus humicola*	CHACB336-10	HQ558518
Sarcoptiformes	Crotoniidae	*Neonothrus humicola*	CHACB337-10	HQ558519
Sarcoptiformes	Crotoniidae	*Neonothrus humicola*	CHACB338-10	HQ558520

Sarcoptiformes	Crotoniidae	*Neonothrus humicola*	CHACB339-10	HQ558521
Sarcoptiformes	Crotoniidae	*Neonothrus humicola*	CHACB340-10	HQ558522
Sarcoptiformes	Crotoniidae	*Neonothrus humicola*	CHACB341-10	HQ558523
Sarcoptiformes	Crotoniidae	*Neonothrus humicola*	CHACB346-10	HQ558527
Sarcoptiformes	Crotoniidae	*Neonothrus humicola*	CHACB347-10	HQ558528
Sarcoptiformes	Crotoniidae	*Neonothrus humicola*	CHACB349-10	HQ558530
Sarcoptiformes	Crotoniidae	*Neonothrus humicola*	CHACB350-10	HQ558531
Sarcoptiformes	Crotoniidae	*Neonothrus humicola*	CHACB351-10	HQ558532
Sarcoptiformes	Crotoniidae	*Neonothrus humicola*	CHACB352-10	HQ558533
Sarcoptiformes	Crotoniidae	*Neonothrus humicola*	CHACB749-10	JX836715
Sarcoptiformes	Crotoniidae	*Neonothrus humicola*	MYMCB446-11	JX835887
Sarcoptiformes	Crotoniidae	*Neonothrus humicola*	MYMCB447-11	JX834280
Sarcoptiformes	Crotoniidae	*Neonothrus humicola*	MYMCC165-11	JX837634
Sarcoptiformes	Crotoniidae	*Neonothrus humicola*	MYMCF867-12	JX835731
Sarcoptiformes	Crotoniidae	*Neonothrus humicola*	MYMCF868-12	JX837824
Sarcoptiformes	Crotoniidae	*Neonothrus humicola*	MYMCG501-12	JX835222
Sarcoptiformes	Crotoniidae	*Platynothrus*	SSWLD3469-13	KM829877
Sarcoptiformes	Crotoniidae	*Platynothrus peltifer*	CHACA1182-10	JX835673
Sarcoptiformes	Crotoniidae	*Platynothrus peltifer*	CHACA248-08	JX836509
Sarcoptiformes	Crotoniidae	*Platynothrus peltifer*	CHACA249-08	JX837701
Sarcoptiformes	Crotoniidae	*Platynothrus peltifer*	CHACA250-08	JX837589
Sarcoptiformes	Crotoniidae	*Platynothrus peltifer*	CHACA251-08	JX835527
Sarcoptiformes	Crotoniidae	*Platynothrus peltifer*	CHACA252-08	JX833990
Sarcoptiformes	Crotoniidae	*Platynothrus peltifer*	CHACA253-08	JX837806
Sarcoptiformes	Crotoniidae	*Platynothrus peltifer*	CHACA254-08	JX835539
Sarcoptiformes	Crotoniidae	*Platynothrus peltifer*	CHACA255-08	JX835770
Sarcoptiformes	Crotoniidae	*Platynothrus peltifer*	CHACA256-08	JX838205
Sarcoptiformes	Crotoniidae	*Platynothrus peltifer*	CHACA257-08	JX834902
Sarcoptiformes	Crotoniidae	*Platynothrus peltifer*	CHACA258-08	JX833645
Sarcoptiformes	Crotoniidae	*Platynothrus peltifer*	CHACA259-08	JX835595
Sarcoptiformes	Crotoniidae	*Platynothrus peltifer*	CHACA260-08	JX833959
Sarcoptiformes	Crotoniidae	*Platynothrus peltifer*	CHACA261-08	JX837862
Sarcoptiformes	Crotoniidae	*Platynothrus peltifer*	CHACA310-08	JX836344
Sarcoptiformes	Crotoniidae	*Platynothrus peltifer*	CHACA319-08	JX834581
Sarcoptiformes	Crotoniidae	*Platynothrus peltifer*	CHACA320-08	JX834937
Sarcoptiformes	Crotoniidae	*Platynothrus peltifer*	CHACA323-08	JX838604
Sarcoptiformes	Crotoniidae	*Platynothrus peltifer*	CHACA324-08	JX836249
Sarcoptiformes	Crotoniidae	*Platynothrus peltifer*	CHACA352-08	JX836917
Sarcoptiformes	Crotoniidae	*Platynothrus peltifer*	CHACA353-08	JX836402
Sarcoptiformes	Crotoniidae	*Platynothrus peltifer*	CHACA354-08	JX835327
Sarcoptiformes	Crotoniidae	*Platynothrus peltifer*	CHACA355-08	JX835244
Sarcoptiformes	Crotoniidae	*Platynothrus peltifer*	CHACA356-08	JX835602
Sarcoptiformes	Crotoniidae	*Platynothrus peltifer*	CHACA357-08	JX838174
Sarcoptiformes	Crotoniidae	*Platynothrus peltifer*	CHACA360-08	JX834600
Sarcoptiformes	Crotoniidae	*Platynothrus peltifer*	CHACA593-09	JX833629
Sarcoptiformes	Crotoniidae	*Platynothrus peltifer*	CHACA594-09	JX837255
Sarcoptiformes	Crotoniidae	*Platynothrus peltifer*	CHACA595-09	JX835268
Sarcoptiformes	Crotoniidae	*Platynothrus peltifer*	CHACA596-09	JX834713
Sarcoptiformes	Crotoniidae	*Platynothrus peltifer*	CHACA597-09	JX837115
Sarcoptiformes	Crotoniidae	*Platynothrus peltifer*	CHACA598-09	JX834034
Sarcoptiformes	Crotoniidae	*Platynothrus peltifer*	CHACA600-09	JX836956
Sarcoptiformes	Crotoniidae	*Platynothrus peltifer*	CHACA601-09	JX836621
Sarcoptiformes	Crotoniidae	*Platynothrus peltifer*	CHACA602-09	JX834855

Sarcoptiformes	Crotoniidae	*Platynothrus peltifer*	CHACA626-09	JX834860
Sarcoptiformes	Crotoniidae	*Platynothrus peltifer*	CHACB348-10	HQ558529
Sarcoptiformes	Crotoniidae	*Platynothrus peltifer*	CHACB516-10	HQ558639
Sarcoptiformes	Crotoniidae	*Platynothrus peltifer*	CHACB517-10	HQ558640
Sarcoptiformes	Crotoniidae	*Platynothrus peltifer*	CHACB518-10	HQ558641
Sarcoptiformes	Crotoniidae	*Platynothrus peltifer*	CHACB519-10	JX835399
Sarcoptiformes	Crotoniidae	*Platynothrus peltifer*	CHACB520-10	HQ558642
Sarcoptiformes	Crotoniidae	*Platynothrus peltifer*	CHACB524-10	HQ558643
Sarcoptiformes	Crotoniidae	*Platynothrus peltifer*	CHACB525-10	HQ558644
Sarcoptiformes	Crotoniidae	*Platynothrus peltifer*	CHACB526-10	HQ558645
Sarcoptiformes	Crotoniidae	*Platynothrus peltifer*	CHACB547-10	HQ558663
Sarcoptiformes	Crotoniidae	*Platynothrus peltifer*	CHACB636-10	HQ558718
Sarcoptiformes	Crotoniidae	*Platynothrus peltifer*	CHACB637-10	HQ558719
Sarcoptiformes	Crotoniidae	*Platynothrus peltifer*	CHACB638-10	HQ558720
Sarcoptiformes	Crotoniidae	*Platynothrus peltifer*	CHACB875-10	HM907320
Sarcoptiformes	Crotoniidae	*Platynothrus peltifer*	CHACB880-10	HM907325
Sarcoptiformes	Crotoniidae	*Platynothrus peltifer*	CHACB949-10	HM907369
Sarcoptiformes	Crotoniidae	*Platynothrus peltifer*	CHACB950-10	HM907370
Sarcoptiformes	Crotoniidae	*Platynothrus peltifer*	CHACB951-10	HM907371
Sarcoptiformes	Crotoniidae	*Platynothrus peltifer*	CHACB952-10	HM907372
Sarcoptiformes	Crotoniidae	*Platynothrus peltifer*	CHACB953-10	HM907373
Sarcoptiformes	Crotoniidae	*Platynothrus peltifer*	CHACB954-10	HM907374
Sarcoptiformes	Crotoniidae	*Platynothrus peltifer*	CHACB961-10	HM907381
Sarcoptiformes	Crotoniidae	*Platynothrus peltifer*	CHACC175-10	JX837165
Sarcoptiformes	Crotoniidae	*Platynothrus peltifer*	GBCH1955-07	DQ381225
Sarcoptiformes	Crotoniidae	*Platynothrus peltifer*	GBCH1956-07	DQ381224
Sarcoptiformes	Crotoniidae	*Platynothrus peltifer*	GBCH1957-07	DQ381223
Sarcoptiformes	Crotoniidae	*Platynothrus peltifer*	GBCH1958-07	DQ381222
Sarcoptiformes	Crotoniidae	*Platynothrus peltifer*	GBCH1959-07	DQ381221
Sarcoptiformes	Crotoniidae	*Platynothrus peltifer*	GBCH1960-07	DQ381220
Sarcoptiformes	Crotoniidae	*Platynothrus peltifer*	GBCH1961-07	DQ381219
Sarcoptiformes	Crotoniidae	*Platynothrus peltifer*	GBCH1962-07	DQ381218
Sarcoptiformes	Crotoniidae	*Platynothrus peltifer*	GBCH1963-07	DQ381217
Sarcoptiformes	Crotoniidae	*Platynothrus peltifer*	GBCH1964-07	DQ381216
Sarcoptiformes	Crotoniidae	*Platynothrus peltifer*	GBCH1965-07	DQ381215
Sarcoptiformes	Crotoniidae	*Platynothrus peltifer*	GBCH1970-07	DQ381210
Sarcoptiformes	Crotoniidae	*Platynothrus peltifer*	GBCH1971-07	DQ381209
Sarcoptiformes	Crotoniidae	*Platynothrus peltifer*	GBCH1972-07	DQ381208
Sarcoptiformes	Crotoniidae	*Platynothrus peltifer*	GBCH1973-07	DQ381207
Sarcoptiformes	Crotoniidae	*Platynothrus peltifer*	GBCH1974-07	DQ381206
Sarcoptiformes	Crotoniidae	*Platynothrus peltifer*	GBCH1975-07	DQ381205
Sarcoptiformes	Crotoniidae	*Platynothrus peltifer*	GBCH1976-07	DQ381204
Sarcoptiformes	Crotoniidae	*Platynothrus peltifer*	GBCH1977-07	DQ381203
Sarcoptiformes	Crotoniidae	*Platynothrus peltifer*	GBCH1978-07	DQ381202
Sarcoptiformes	Crotoniidae	*Platynothrus peltifer*	GBCH1979-07	DQ381201
Sarcoptiformes	Crotoniidae	*Platynothrus peltifer*	GBCH1980-07	DQ381200
Sarcoptiformes	Crotoniidae	*Platynothrus peltifer*	GBCH1981-07	DQ381199
Sarcoptiformes	Crotoniidae	*Platynothrus peltifer*	GBCH1982-07	DQ381198
Sarcoptiformes	Crotoniidae	*Platynothrus peltifer*	GBCH1983-07	DQ381197
Sarcoptiformes	Crotoniidae	*Platynothrus peltifer*	GBCH1988-07	DQ381192
Sarcoptiformes	Crotoniidae	*Platynothrus peltifer*	GBCH1989-07	DQ381191
Sarcoptiformes	Crotoniidae	*Platynothrus peltifer*	GBCH1990-07	DQ381190
Sarcoptiformes	Crotoniidae	*Platynothrus peltifer*	GBCH1991-07	DQ381189

Sarcoptiformes	Crotoniidae	*Platynothrus peltifer*	GBCH1992-07	DQ381188
Sarcoptiformes	Crotoniidae	*Platynothrus peltifer*	GBCH1993-07	DQ381187
Sarcoptiformes	Crotoniidae	*Platynothrus peltifer*	GBCH1994-07	DQ381186
Sarcoptiformes	Crotoniidae	*Platynothrus peltifer*	GBCH1995-07	DQ381185
Sarcoptiformes	Crotoniidae	*Platynothrus peltifer*	GBCH1996-07	DQ381184
Sarcoptiformes	Crotoniidae	*Platynothrus peltifer*	GBCH1997-07	DQ381183
Sarcoptiformes	Crotoniidae	*Platynothrus peltifer*	GBCH2006-07	DQ381174
Sarcoptiformes	Crotoniidae	*Platynothrus peltifer*	GBCH2007-07	DQ381173
Sarcoptiformes	Crotoniidae	*Platynothrus peltifer*	GBCH2008-07	DQ381172
Sarcoptiformes	Crotoniidae	*Platynothrus peltifer*	GBCH2009-07	DQ381171
Sarcoptiformes	Crotoniidae	*Platynothrus peltifer*	GBCH2010-07	DQ381170
Sarcoptiformes	Crotoniidae	*Platynothrus peltifer*	GBCH2011-07	DQ381169
Sarcoptiformes	Crotoniidae	*Platynothrus peltifer*	GBCH2012-07	DQ381168
Sarcoptiformes	Crotoniidae	*Platynothrus peltifer*	GBCH2013-07	DQ381167
Sarcoptiformes	Crotoniidae	*Platynothrus peltifer*	GBCH2014-07	DQ381166
Sarcoptiformes	Crotoniidae	*Platynothrus peltifer*	GBCH2015-07	DQ381165
Sarcoptiformes	Crotoniidae	*Platynothrus peltifer*	GBCH2016-07	DQ381164
Sarcoptiformes	Crotoniidae	*Platynothrus peltifer*	GBCH2017-07	DQ381163
Sarcoptiformes	Crotoniidae	*Platynothrus peltifer*	GBCH2018-07	DQ381162
Sarcoptiformes	Crotoniidae	*Platynothrus peltifer*	GBCH2019-07	DQ381161
Sarcoptiformes	Crotoniidae	*Platynothrus peltifer*	GBCH3772-09	DQ381214
Sarcoptiformes	Crotoniidae	*Platynothrus peltifer*	GBCH3773-09	DQ381213
Sarcoptiformes	Crotoniidae	*Platynothrus peltifer*	GBCH3774-09	DQ381212
Sarcoptiformes	Crotoniidae	*Platynothrus peltifer*	GBCH3775-09	DQ381211
Sarcoptiformes	Crotoniidae	*Platynothrus peltifer*	GBCH3776-09	DQ381196
Sarcoptiformes	Crotoniidae	*Platynothrus peltifer*	GBCH3777-09	DQ381195
Sarcoptiformes	Crotoniidae	*Platynothrus peltifer*	GBCH3778-09	DQ381194
Sarcoptiformes	Crotoniidae	*Platynothrus peltifer*	GBCH3779-09	DQ381193
Sarcoptiformes	Crotoniidae	*Platynothrus peltifer*	GBCH3783-09	DQ381175
Sarcoptiformes	Crotoniidae	*Platynothrus peltifer*	GBCH6510-13	JF263998
Sarcoptiformes	Crotoniidae	*Platynothrus peltifer*	GBCH6511-13	JF263997
Sarcoptiformes	Crotoniidae	*Platynothrus peltifer*	GBCH6512-13	JF263996
Sarcoptiformes	Crotoniidae	*Platynothrus peltifer*	GBCH6513-13	JF263995
Sarcoptiformes	Crotoniidae	*Platynothrus peltifer*	GBCH6514-13	JF263994
Sarcoptiformes	Crotoniidae	*Platynothrus peltifer*	GBCH6515-13	JF263993
Sarcoptiformes	Crotoniidae	*Platynothrus peltifer*	GBCH6516-13	JF263992
Sarcoptiformes	Crotoniidae	*Platynothrus peltifer*	GBCH6517-13	JF263991
Sarcoptiformes	Crotoniidae	*Platynothrus peltifer*	GBCH6518-13	JF263990
Sarcoptiformes	Crotoniidae	*Platynothrus peltifer*	GBCH6519-13	JF263989
Sarcoptiformes	Crotoniidae	*Platynothrus peltifer*	GBCH6520-13	JF263988
Sarcoptiformes	Crotoniidae	*Platynothrus peltifer*	GBCH6521-13	JF263987
Sarcoptiformes	Crotoniidae	*Platynothrus peltifer*	GBCH6522-13	JF263986
Sarcoptiformes	Crotoniidae	*Platynothrus peltifer*	GBCH6523-13	JF263985
Sarcoptiformes	Crotoniidae	*Platynothrus peltifer*	GBCH6524-13	JF263984
Sarcoptiformes	Crotoniidae	*Platynothrus peltifer*	GBCH6525-13	JF263983
Sarcoptiformes	Crotoniidae	*Platynothrus peltifer*	GBCH6526-13	JF263982
Sarcoptiformes	Crotoniidae	*Platynothrus peltifer*	GBCH6527-13	JF263981
Sarcoptiformes	Crotoniidae	*Platynothrus peltifer*	GBCH6528-13	JF263980
Sarcoptiformes	Crotoniidae	*Platynothrus peltifer*	GBCH6529-13	JF263979
Sarcoptiformes	Crotoniidae	*Platynothrus peltifer*	GBCH6530-13	JF263978
Sarcoptiformes	Crotoniidae	*Platynothrus peltifer*	GBCH6531-13	JF263977
Sarcoptiformes	Crotoniidae	*Platynothrus peltifer*	GBCH6532-13	JF263976
Sarcoptiformes	Crotoniidae	*Platynothrus peltifer*	GBCH6533-13	JF263975

Sarcoptiformes	Crotoniidae	*Platynothrus peltifer*	GBCH6534-13	JF263974
Sarcoptiformes	Crotoniidae	*Platynothrus peltifer*	GBCH6535-13	JF263973
Sarcoptiformes	Crotoniidae	*Platynothrus peltifer*	GBCH6536-13	JF263972
Sarcoptiformes	Crotoniidae	*Platynothrus peltifer*	GBCH6537-13	JF263971
Sarcoptiformes	Crotoniidae	*Platynothrus peltifer*	GBCH6538-13	JF263970
Sarcoptiformes	Crotoniidae	*Platynothrus peltifer*	GBCH6539-13	JF263969
Sarcoptiformes	Crotoniidae	*Platynothrus peltifer*	GBCH6540-13	JF263968
Sarcoptiformes	Crotoniidae	*Platynothrus peltifer*	GBCH6541-13	JF263967
Sarcoptiformes	Crotoniidae	*Platynothrus peltifer*	GBCH6542-13	JF263966
Sarcoptiformes	Crotoniidae	*Platynothrus peltifer*	GBCH6543-13	JF263965
Sarcoptiformes	Crotoniidae	*Platynothrus peltifer*	GBCH6544-13	JF263964
Sarcoptiformes	Crotoniidae	*Platynothrus peltifer*	GBCH6545-13	JF263963
Sarcoptiformes	Crotoniidae	*Platynothrus peltifer*	GBCH6546-13	JF263962
Sarcoptiformes	Crotoniidae	*Platynothrus peltifer*	GBCH6547-13	JF263961
Sarcoptiformes	Crotoniidae	*Platynothrus peltifer*	GBCH6548-13	JF263960
Sarcoptiformes	Crotoniidae	*Platynothrus peltifer*	GBCH6549-13	JF263959
Sarcoptiformes	Crotoniidae	*Platynothrus peltifer*	GBCH6550-13	JF263958
Sarcoptiformes	Crotoniidae	*Platynothrus peltifer*	GBCH6551-13	JF263957
Sarcoptiformes	Crotoniidae	*Platynothrus peltifer*	GBCH6552-13	JF263956
Sarcoptiformes	Crotoniidae	*Platynothrus peltifer*	GBCH6553-13	JF263955
Sarcoptiformes	Crotoniidae	*Platynothrus peltifer*	GBCH6554-13	JF263954
Sarcoptiformes	Crotoniidae	*Platynothrus peltifer*	GBCH6555-13	JF263953
Sarcoptiformes	Crotoniidae	*Platynothrus peltifer*	GBCH6556-13	JF263952
Sarcoptiformes	Crotoniidae	*Platynothrus peltifer*	GBCH6557-13	JF263951
Sarcoptiformes	Crotoniidae	*Platynothrus peltifer*	GBCH6558-13	JF263950
Sarcoptiformes	Crotoniidae	*Platynothrus peltifer*	GBCH6559-13	JF263949
Sarcoptiformes	Crotoniidae	*Platynothrus peltifer*	GBCH6560-13	JF263948
Sarcoptiformes	Crotoniidae	*Platynothrus peltifer*	GBCH6561-13	JF263947
Sarcoptiformes	Crotoniidae	*Platynothrus peltifer*	GBCH6562-13	JF263946
Sarcoptiformes	Crotoniidae	*Platynothrus peltifer*	GBCH6563-13	JF263945
Sarcoptiformes	Crotoniidae	*Platynothrus peltifer*	GBCH6564-13	JF263944
Sarcoptiformes	Crotoniidae	*Platynothrus peltifer*	GBCH6565-13	JF263943
Sarcoptiformes	Crotoniidae	*Platynothrus peltifer*	GBCH6566-13	JF263942
Sarcoptiformes	Crotoniidae	*Platynothrus peltifer*	GBCH6567-13	JF263941
Sarcoptiformes	Crotoniidae	*Platynothrus peltifer*	GBCH6568-13	JF263940
Sarcoptiformes	Crotoniidae	*Platynothrus peltifer*	GBCH6569-13	JF263939
Sarcoptiformes	Crotoniidae	*Platynothrus peltifer*	GBCH6570-13	JF263938
Sarcoptiformes	Crotoniidae	*Platynothrus peltifer*	GBCH6571-13	JF263937
Sarcoptiformes	Crotoniidae	*Platynothrus peltifer*	GBCH6573-13	JF263935
Sarcoptiformes	Crotoniidae	*Platynothrus peltifer*	GBCH6574-13	JF263934
Sarcoptiformes	Crotoniidae	*Platynothrus peltifer*	GBCH6575-13	JF263933
Sarcoptiformes	Crotoniidae	*Platynothrus peltifer*	GBCH6576-13	JF263932
Sarcoptiformes	Crotoniidae	*Platynothrus peltifer*	GBCH6577-13	JF263931
Sarcoptiformes	Crotoniidae	*Platynothrus peltifer*	GBCH6578-13	JF263930
Sarcoptiformes	Crotoniidae	*Platynothrus peltifer*	GBCH6579-13	JF263929
Sarcoptiformes	Crotoniidae	*Platynothrus peltifer*	GBCH6580-13	JF263928
Sarcoptiformes	Crotoniidae	*Platynothrus peltifer*	GBCH6581-13	JF263927
Sarcoptiformes	Crotoniidae	*Platynothrus peltifer*	GBCH6582-13	JF263926
Sarcoptiformes	Crotoniidae	*Platynothrus peltifer*	GBCH6583-13	JF263925
Sarcoptiformes	Crotoniidae	*Platynothrus peltifer*	GBCH6584-13	JF263924
Sarcoptiformes	Crotoniidae	*Platynothrus peltifer*	GBCH6590-13	JF263918
Sarcoptiformes	Crotoniidae	*Platynothrus peltifer*	GBCH6591-13	JF263917
Sarcoptiformes	Crotoniidae	*Platynothrus peltifer*	GBCH6592-13	JF263916

Sarcoptiformes	Crotoniidae	*Platynothrus peltifer*	GBCH6593-13	JF263915
Sarcoptiformes	Crotoniidae	*Platynothrus peltifer*	GBCH6594-13	JF263914
Sarcoptiformes	Crotoniidae	*Platynothrus peltifer*	GBCH6595-13	JF263913
Sarcoptiformes	Crotoniidae	*Platynothrus peltifer*	GBCH6596-13	JF263912
Sarcoptiformes	Crotoniidae	*Platynothrus peltifer*	GBCH6597-13	JF263911
Sarcoptiformes	Crotoniidae	*Platynothrus peltifer*	GBCH6598-13	JF263910
Sarcoptiformes	Crotoniidae	*Platynothrus peltifer*	GBCH6599-13	JF263909
Sarcoptiformes	Crotoniidae	*Platynothrus peltifer*	GBCH6600-13	JF263908
Sarcoptiformes	Crotoniidae	*Platynothrus peltifer*	GBCH6601-13	JF263907
Sarcoptiformes	Crotoniidae	*Platynothrus peltifer*	GBCH6602-13	JF263906
Sarcoptiformes	Crotoniidae	*Platynothrus peltifer*	GBCH6603-13	JF263905
Sarcoptiformes	Crotoniidae	*Platynothrus peltifer*	GBCH6604-13	JF263904
Sarcoptiformes	Crotoniidae	*Platynothrus peltifer*	GBCH6605-13	JF263903
Sarcoptiformes	Crotoniidae	*Platynothrus peltifer*	GBCH6606-13	JF263902
Sarcoptiformes	Crotoniidae	*Platynothrus peltifer*	GBCH6607-13	JF263901
Sarcoptiformes	Crotoniidae	*Platynothrus peltifer*	GBCH6608-13	JF263900
Sarcoptiformes	Crotoniidae	*Platynothrus peltifer*	GBCH6609-13	JF263899
Sarcoptiformes	Crotoniidae	*Platynothrus peltifer*	GBCH6610-13	JF263898
Sarcoptiformes	Crotoniidae	*Platynothrus peltifer*	GBCH6611-13	JF263897
Sarcoptiformes	Crotoniidae	*Platynothrus peltifer*	GBCH6612-13	JF263896
Sarcoptiformes	Crotoniidae	*Platynothrus peltifer*	GBCH6613-13	JF263895
Sarcoptiformes	Crotoniidae	*Platynothrus peltifer*	GBCH6614-13	JF263894
Sarcoptiformes	Crotoniidae	*Platynothrus peltifer*	GBCH6615-13	JF263893
Sarcoptiformes	Crotoniidae	*Platynothrus peltifer*	GBCH6616-13	JF263892
Sarcoptiformes	Crotoniidae	*Platynothrus peltifer*	GBCH6617-13	JF263891
Sarcoptiformes	Crotoniidae	*Platynothrus peltifer*	GBCH6618-13	JF263890
Sarcoptiformes	Crotoniidae	*Platynothrus peltifer*	GBCH6619-13	JF263889
Sarcoptiformes	Crotoniidae	*Platynothrus peltifer*	GBCH6620-13	JF263888
Sarcoptiformes	Crotoniidae	*Platynothrus peltifer*	GBCH6621-13	JF263887
Sarcoptiformes	Crotoniidae	*Platynothrus peltifer*	GBCH6622-13	JF263886
Sarcoptiformes	Crotoniidae	*Platynothrus peltifer*	GBCH6623-13	JF263885
Sarcoptiformes	Crotoniidae	*Platynothrus peltifer*	GBCH6624-13	JF263884
Sarcoptiformes	Crotoniidae	*Platynothrus peltifer*	GBCH6625-13	JF263883
Sarcoptiformes	Crotoniidae	*Platynothrus peltifer*	GBCH6626-13	JF263882
Sarcoptiformes	Crotoniidae	*Platynothrus peltifer*	GBCH6627-13	JF263881
Sarcoptiformes	Crotoniidae	*Platynothrus peltifer*	GBCH6628-13	JF263880
Sarcoptiformes	Crotoniidae	*Platynothrus peltifer*	GBCH6629-13	JF263879
Sarcoptiformes	Crotoniidae	*Platynothrus peltifer*	GBCH6630-13	JF263878
Sarcoptiformes	Crotoniidae	*Platynothrus peltifer*	GBCH6631-13	JF263877
Sarcoptiformes	Crotoniidae	*Platynothrus peltifer*	GBCH6632-13	JF263876
Sarcoptiformes	Crotoniidae	*Platynothrus peltifer*	GBCH6633-13	JF263875
Sarcoptiformes	Crotoniidae	*Platynothrus peltifer*	GBCH6634-13	JF263874
Sarcoptiformes	Crotoniidae	*Platynothrus peltifer*	GBCH6635-13	JF263873
Sarcoptiformes	Crotoniidae	*Platynothrus peltifer*	GBCH6636-13	JF263872
Sarcoptiformes	Crotoniidae	*Platynothrus peltifer*	GBCH6637-13	JF263871
Sarcoptiformes	Crotoniidae	*Platynothrus peltifer*	GBCH6638-13	JF263870
Sarcoptiformes	Crotoniidae	*Platynothrus peltifer*	GBCH6639-13	JF263869
Sarcoptiformes	Crotoniidae	*Platynothrus peltifer*	GBCH6640-13	JF263868
Sarcoptiformes	Crotoniidae	*Platynothrus peltifer*	GBCH6641-13	JF263867
Sarcoptiformes	Crotoniidae	*Platynothrus peltifer*	GBCH6642-13	JF263866
Sarcoptiformes	Crotoniidae	*Platynothrus peltifer*	GBCH6643-13	JF263865
Sarcoptiformes	Crotoniidae	*Platynothrus peltifer*	GBCH6644-13	JF263864
Sarcoptiformes	Crotoniidae	*Platynothrus peltifer*	GBCH6645-13	JF263863

Sarcoptiformes	Crotoniidae	*Platynothrus peltifer*	GBCH6646-13	JF263862
Sarcoptiformes	Crotoniidae	*Platynothrus peltifer*	GBCH6647-13	JF263861
Sarcoptiformes	Crotoniidae	*Platynothrus peltifer*	GBCH6648-13	JF263860
Sarcoptiformes	Crotoniidae	*Platynothrus peltifer*	GBCH6649-13	JF263859
Sarcoptiformes	Crotoniidae	*Platynothrus peltifer*	GBCH6650-13	JF263858
Sarcoptiformes	Crotoniidae	*Platynothrus peltifer*	GBCH6651-13	JF263857
Sarcoptiformes	Crotoniidae	*Platynothrus peltifer*	GBCH6652-13	JF263856
Sarcoptiformes	Crotoniidae	*Platynothrus peltifer*	GBCH6653-13	JF263855
Sarcoptiformes	Crotoniidae	*Platynothrus peltifer*	GBCH6654-13	JF263854
Sarcoptiformes	Crotoniidae	*Platynothrus peltifer*	GBCH6655-13	JF263853
Sarcoptiformes	Crotoniidae	*Platynothrus peltifer*	GBCH6656-13	JF263852
Sarcoptiformes	Crotoniidae	*Platynothrus peltifer*	GBCH6657-13	JF263851
Sarcoptiformes	Crotoniidae	*Platynothrus peltifer*	GBCH6658-13	JF263850
Sarcoptiformes	Crotoniidae	*Platynothrus peltifer*	GBCH6659-13	JF263849
Sarcoptiformes	Crotoniidae	*Platynothrus peltifer*	GBCH6660-13	JF263848
Sarcoptiformes	Crotoniidae	*Platynothrus peltifer*	GBCH6661-13	JF263847
Sarcoptiformes	Crotoniidae	*Platynothrus peltifer*	GBCH6662-13	JF263846
Sarcoptiformes	Crotoniidae	*Platynothrus peltifer*	GBCH6663-13	JF263845
Sarcoptiformes	Crotoniidae	*Platynothrus peltifer*	GBCH6664-13	JF263844
Sarcoptiformes	Crotoniidae	*Platynothrus peltifer*	GBCH6665-13	JF263843
Sarcoptiformes	Crotoniidae	*Platynothrus peltifer*	GBCH6666-13	JF263842
Sarcoptiformes	Crotoniidae	*Platynothrus peltifer*	GBCH6667-13	JF263841
Sarcoptiformes	Crotoniidae	*Platynothrus peltifer*	MIONB006-10	HM887559
Sarcoptiformes	Crotoniidae	*Platynothrus peltifer*	MIONB007-10	HM887560
Sarcoptiformes	Crotoniidae	*Platynothrus peltifer*	MIONB008-10	HM887561
Sarcoptiformes	Crotoniidae	*Platynothrus peltifer*	MIONB026-10	HM887569
Sarcoptiformes	Crotoniidae	*Platynothrus peltifer*	MIONB027-10	HM887570
Sarcoptiformes	Crotoniidae	*Platynothrus peltifer*	MYMCA071-11	JX835937
Sarcoptiformes	Crotoniidae	*Platynothrus peltifer*	MYMCB169-11	JX836141
Sarcoptiformes	Crotoniidae	*Platynothrus peltifer*	MYMCB170-11	JX838216
Sarcoptiformes	Crotoniidae	*Platynothrus peltifer*	MYMCB662-11	JX835058
Sarcoptiformes	Crotoniidae	*Platynothrus peltifer*	MYMCD062-11	JX834996
Sarcoptiformes	Crotoniidae	*Platynothrus peltifer*	MYMCD063-11	JX836718
Sarcoptiformes	Crotoniidae	*Platynothrus peltifer*	MYMCD095-11	JX837553
Sarcoptiformes	Crotoniidae	*Platynothrus peltifer*	MYMCD113-11	JX838646
Sarcoptiformes	Crotoniidae	*Platynothrus peltifer*	MYMCD114-11	JX838560
Sarcoptiformes	Crotoniidae	*Platynothrus peltifer*	MYMCE070-12	JX837764
Sarcoptiformes	Crotoniidae	*Platynothrus peltifer*	MYMCE218-12	JX837560
Sarcoptiformes	Crotoniidae	*Platynothrus peltifer*	MYMCF659-12	JX834436
Sarcoptiformes	Crotoniidae	*Platynothrus peltifer*	MYMCF660-12	JX835928
Sarcoptiformes	Crotoniidae	*Platynothrus peltifer*	MYMCG255-12	JX834143
Sarcoptiformes	Crotoniidae	*Platynothrus peltifer*	MYMCG375-12	JX838544
Sarcoptiformes	Crotoniidae	*Platynothrus peltifer*	SSPAA7824-13	KM840472
Sarcoptiformes	Crotoniidae	*Platynothrus peltifer*	SSPAA7828-13	KM826874
Sarcoptiformes	Crotoniidae	*Platynothrus yamasaki*	SSEIA2082-13	KM836024
Sarcoptiformes	Crotoniidae	*Platynothrus yamasaki*	SSEIA2083-13	KM828485
Sarcoptiformes	Crotoniidae	*Platynothrus yamasaki*	SSEIA2090-13	KM836447
Sarcoptiformes	Crotoniidae	*Platynothrus yamasaki*	SSEIA2096-13	KM829699
Sarcoptiformes	Crotoniidae	*Platynothrus yamasaki*	SSEIA2449-13	KM837927
Sarcoptiformes	Crotoniidae	*Platynothrus yamasaki*	SSEIB4552-13	KM832326
Sarcoptiformes	Crotoniidae	*Platynothrus yamasaki*	SSEIB4553-13	KM832456
Sarcoptiformes	Crotoniidae	*Platynothrus yamasaki*	SSEIB4554-13	KM824246
Sarcoptiformes	Crotoniidae	*Platynothrus yamasaki*	SSEIB8163-13	KM825213

Sarcoptiformes	Crotoniidae	*Platynothrus yamasaki*	SSEIB8164-13	KM827614
Sarcoptiformes	Crotoniidae	*Platynothrus yamasaki*	SSEIB8165-13	KM832742
Sarcoptiformes	Crotoniidae	*Platynothrus yamasaki*	SSEIB8166-13	KM827842
Sarcoptiformes	Crotoniidae	*Platynothrus yamasaki*	SSEIB8168-13	KM826566
Sarcoptiformes	Crotoniidae	*Platynothrus yamasaki*	SSEIB8170-13	KM825820
Sarcoptiformes	Crotoniidae	*Platynothrus yamasaki*	SSEIB8173-13	KM824751
Sarcoptiformes	Crotoniidae	*Platynothrus yamasaki*	SSEIB8177-13	KM835709
Sarcoptiformes	Crotoniidae	*Platynothrus yamasaki*	SSEIB8187-13	KM827351
Sarcoptiformes	Crotoniidae	*Platynothrus yamasaki*	SSEIB8194-13	KM835532
Sarcoptiformes	Cymbaeremaeidae		MBIOE2326-13	KR070314
Sarcoptiformes	Cymbaeremaeidae	*Ametroproctus*	SSBAA3690-12	KM824785
Sarcoptiformes	Cymbaeremaeidae	*Ametroproctus*	SSBAD4050-12	KM825482
Sarcoptiformes	Cymbaeremaeidae	*Ametroproctus*	SSJAD2916-13	KM826992
Sarcoptiformes	Cymbaeremaeidae	*Ametroproctus*	SSJAD2921-13	KM824517
Sarcoptiformes	Cymbaeremaeidae	*Ametroproctus*	SSWLC1158-13	KM834886
Sarcoptiformes	Cymbaeremaeidae	*Ametroproctus*	SSWLC1162-13	KM831199
Sarcoptiformes	Cymbaeremaeidae	*Ametroproctus*	SSWLF2965-13	KM824091
Sarcoptiformes	Cymbaeremaeidae	*Ametroproctus*	SSWLF2966-13	KM827334
Sarcoptiformes	Cymbaeremaeidae	*Ametroproctus*	SSWLF2981-13	KM831348
Sarcoptiformes	Cymbaeremaeidae	*Ametroproctus*	SSWLF3001-13	KM838189
Sarcoptiformes	Cymbaeremaeidae	*Ametroproctus*	SSWLF3011-13	KM830834
Sarcoptiformes	Cymbaeremaeidae	*Ametroproctus*	SSWLF3016-13	KM826676
Sarcoptiformes	Cymbaeremaeidae	*Ametroproctus*	SSWLF3034-13	KM827068
Sarcoptiformes	Cymbaeremaeidae	*Ametroproctus*	SSWLF3043-13	KM824793
Sarcoptiformes	Cymbaeremaeidae	*Ametroproctus*	SSWLF3047-13	KM824306
Sarcoptiformes	Cymbaeremaeidae	*Ametroproctus*	SSWLF3091-13	KM835206
Sarcoptiformes	Cymbaeremaeidae	*Ametroproctus*	SSWLF3108-13	KM836577
Sarcoptiformes	Cymbaeremaeidae	*Ametroproctus*	SSWLF3122-13	KM832225
Sarcoptiformes	Cymbaeremaeidae	*Ametroproctus*	SSWLF3151-13	KM824894
Sarcoptiformes	Cymbaeremaeidae	*Ametroproctus*	SSWLF3159-13	KM825969
Sarcoptiformes	Cymbaeremaeidae	*Ametroproctus*	SSWLF3177-13	KM836809
Sarcoptiformes	Cymbaeremaeidae	*Ametroproctus*	SSWLF3919-13	KM829682
Sarcoptiformes	Cymbaeremaeidae	*Cymbaeremaeus cymba*	GBCH5033-10	GU208618
Sarcoptiformes	Cymbaeremaeidae	*Cymbaeremaeus cymba*	GBCH5034-10	GU208617
Sarcoptiformes	Cymbaeremaeidae	*Cymbaeremaeus cymba*	GBCH5035-10	GU208616
Sarcoptiformes	Cymbaeremaeidae	*Scapheremaeus*	MIONB275-10	KP979242
Sarcoptiformes	Cymbaeremaeidae		CNBAC730-12	KM828352
Sarcoptiformes	Cymbaeremaeidae		CNBAD695-12	KM825458
Sarcoptiformes	Cymbaeremaeidae		CNBAF202-12	KM824396
Sarcoptiformes	Cymbaeremaeidae		CNBPA195-12	KM831105
Sarcoptiformes	Cymbaeremaeidae		CNBPJ297-13	KM830043
Sarcoptiformes	Cymbaeremaeidae		CNGIB595-12	KM832618
Sarcoptiformes	Cymbaeremaeidae		CNGID221-12	KM831124
Sarcoptiformes	Cymbaeremaeidae		CNJAF2008-12	KM826722
Sarcoptiformes	Cymbaeremaeidae		CNJAG1834-12	KM830485
Sarcoptiformes	Cymbaeremaeidae		CNJAH787-12	KM825793
Sarcoptiformes	Cymbaeremaeidae		CNPAG376-13	KM829505
Sarcoptiformes	Cymbaeremaeidae		CNPCI012-13	KM831437
Sarcoptiformes	Cymbaeremaeidae		CNROD559-13	KR070646
Sarcoptiformes	Cymbaeremaeidae		CNROO117-13	KR069220
Sarcoptiformes	Cymbaeremaeidae		SSBAD3117-12	KM837194
Sarcoptiformes	Cymbaeremaeidae		SSBAD3141-12	KM829945
Sarcoptiformes	Cymbaeremaeidae		SSBAD3984-12	KM828148

Sarcoptiformes	Cymbaeremaeidae		SSBAD4020-12	KM839842
Sarcoptiformes	Cymbaeremaeidae		SSBAD4042-12	KM836824
Sarcoptiformes	Cymbaeremaeidae		SSJAB1831-13	KM837044
Sarcoptiformes	Cymbaeremaeidae		SSPAB4025-13	KM836439
Sarcoptiformes	Cymbaeremaeidae		SSPAB966-13	KM835062
Sarcoptiformes	Cymbaeremaeidae		SSWLF2971-13	KM824433
Sarcoptiformes	Cymbaeremaeidae		SSWLF3105-13	KM829450
Sarcoptiformes	Cymbaeremaeidae		SSWLF3132-13	KM833295
Sarcoptiformes	Damaeidae	*Epidamaeus*	CHACA1172-10	JX834849
Sarcoptiformes	Damaeidae	*Epidamaeus*	CHACA1173-10	JX836445
Sarcoptiformes	Damaeidae	*Epidamaeus*	CHACB468-10	HQ558608
Sarcoptiformes	Damaeidae	*Epidamaeus*	CHACB542-10	HQ558658
Sarcoptiformes	Damaeidae	*Epidamaeus*	CHACB543-10	HQ558659
Sarcoptiformes	Damaeidae	*Epidamaeus*	CHACB544-10	HQ558660
Sarcoptiformes	Damaeidae	*Epidamaeus*	CHACB545-10	HQ558661
Sarcoptiformes	Damaeidae	*Epidamaeus*	CHACB546-10	HQ558662
Sarcoptiformes	Damaeidae	*Epidamaeus*	CHACC222-10	HQ941536
Sarcoptiformes	Damaeidae	*Epidamaeus*	CHACC223-10	HQ941537
Sarcoptiformes	Damaeidae	*Epidamaeus*	CHACC237-10	HQ941545
Sarcoptiformes	Damaeidae	*Epidamaeus*	SSEIA2091-13	KM839062
Sarcoptiformes	Damaeidae	*Epidamaeus*	SSEIA3050-13	KM824003
Sarcoptiformes	Damaeidae	*Epidamaeus*	SSEIB8174-13	KM828570
Sarcoptiformes	Damaeidae	*Epidamaeus*	SSEIB8200-13	KM827160
Sarcoptiformes	Damaeidae	*Epidamaeus*	SSEIB8209-13	KM840038
Sarcoptiformes	Damaeidae	*Epidamaeus*	SSJAC1525-13	KM837646
Sarcoptiformes	Damaeidae	*Epidamaeus*	SSPAA8014-13	KM830178
Sarcoptiformes	Damaeidae	*Epidamaeus floccosus*	CHACB034-10	HQ558347
Sarcoptiformes	Damaeidae	*Epidamaeus floccosus*	CHACB035-10	HQ558348
Sarcoptiformes	Damaeidae	*Epidamaeus floccosus*	CHACB895-10	HM907331
Sarcoptiformes	Damaeidae	*Epidamaeus floccosus*	MYMCA792-11	JX836947
Sarcoptiformes	Damaeidae	*Epidamaeus floccosus*	MYMCB830-11	JX836650
Sarcoptiformes	Damaeidae	*Epidamaeus floccosus*	MYMCB831-11	JX835410
Sarcoptiformes	Damaeidae	*Epidamaeus floccosus*	MYMCB832-11	JX837152
Sarcoptiformes	Damaeidae	*Epidamaeus floccosus*	MYMCC465-11	JX837901
Sarcoptiformes	Damaeidae	*Epidamaeus floccosus*	MYMCF094-12	JX835586
Sarcoptiformes	Damaeidae	*Epidamaeus floccosus*	MYMCF646-12	JX837400
Sarcoptiformes	Damaeidae	*Epidamaeus floccosus*	MYTMC068-09	HM431995
Sarcoptiformes	Damaeidae	*Epidamaeus floccosus*	SSBAA3632-12	KM826166
Sarcoptiformes	Damaeidae	*Epidamaeus longitarsalis*	CHACA103-08	JX836187
Sarcoptiformes	Damaeidae	*Epidamaeus longitarsalis*	CHACA169-08	JX834451
Sarcoptiformes	Damaeidae	*Epidamaeus longitarsalis*	CHACA170-08	JX836586
Sarcoptiformes	Damaeidae	*Epidamaeus longitarsalis*	CHACA171-08	JX833768
Sarcoptiformes	Damaeidae	*Epidamaeus longitarsalis*	CHACB894-10	HM907330
Sarcoptiformes	Damaeidae	*Epidamaeus longitarsalis*	MYMCB518-11	JX835138
Sarcoptiformes	Damaeidae	*Epidamaeus longitarsalis*	MYMCG359-12	JX836701
Sarcoptiformes	Damaeidae	*Quatrobelba montana*	SSJAD2917-13	KM828070
Sarcoptiformes	Damaeidae		CHACA934-10	HM405811
Sarcoptiformes	Damaeidae		CHACA935-10	HM405812
Sarcoptiformes	Damaeidae		CHACA936-10	HM405813
Sarcoptiformes	Damaeidae		CHACA948-10	KR069415
Sarcoptiformes	Damaeidae		CHACA957-10	HM405828
Sarcoptiformes	Damaeidae		MYMCA1092-11	JX837942
Sarcoptiformes	Damaeidae		MYMCB065-11	JX835721

Sarcoptiformes	Damaeidae		MYMCB747-11	JX835562
Sarcoptiformes	Damaeidae		MYMCB748-11	JX834837
Sarcoptiformes	Damaeidae		MYMCC232-11	JX835838
Sarcoptiformes	Damaeidae		MYMCC257-11	JX837179
Sarcoptiformes	Damaeidae		MYMCC466-11	JX837146
Sarcoptiformes	Damaeidae		MYMCC473-11	JX838304
Sarcoptiformes	Damaeidae		MYMCC619-11	JX834762
Sarcoptiformes	Damaeidae		MYMCC671-11	JX834952
Sarcoptiformes	Damaeidae		SSEIC4928-13	KM830745
Sarcoptiformes	Damaeidae		SSWEE055-13	KM827320
Sarcoptiformes	Damaeidae		SSWEE103-13	KM827985
Sarcoptiformes	Damaeidae		SSWEE125-13	KM834457
Sarcoptiformes	Damaeidae		SSWLA5266-13	KM828865
Sarcoptiformes	Damaeidae		SSWLA5276-13	KM838922
Sarcoptiformes	Damaeidae		SSWLE2956-13	KM838172
Sarcoptiformes	Damaeidae		SSWLF3569-13	KM834969
Sarcoptiformes	Damaeidae		SSWLF3591-13	KM829288
Sarcoptiformes	Eniochthoniidae	*Eniochthonius crosbyi*	MIONB078-10	HQ575092
Sarcoptiformes	Eniochthoniidae		MYMCC715-11	JX835537
Sarcoptiformes	Eniochthoniidae		MYMCD003-11	JX835356
Sarcoptiformes	Eremaeidae	*Eremaeus*	CHACA937-10	HM405814
Sarcoptiformes	Eremaeidae	*Eremaeus*	CHACA938-10	HM405815
Sarcoptiformes	Eremaeidae	*Eremaeus*	CHACA939-10	HM405816
Sarcoptiformes	Eremaeidae	*Eremaeus*	CHACA940-10	HM405817
Sarcoptiformes	Eremaeidae	*Eremaeus*	CHACA941-10	HM405818
Sarcoptiformes	Eremaeidae	*Eremaeus*	CHACA942-10	HM405819
Sarcoptiformes	Eremaeidae	*Eremaeus*	CHACA943-10	HM405820
Sarcoptiformes	Eremaeidae	*Eueremaeus*	CNGLF135-13	KM829137
Sarcoptiformes	Eremaeidae	*Eueremaeus*	SSBAD4700-13	KM838021
Sarcoptiformes	Eremaeidae	*Eueremaeus*	SSJAF2625-13	KM831194
Sarcoptiformes	Eremaeidae	*Eueremaeus*	SSPAB3136-13	KM835219
Sarcoptiformes	Eremaeidae	*Eueremaeus*	SSPAB3974-13	KM829849
Sarcoptiformes	Eremaeidae	*Eueremaeus cf. quadrilamellatus*	CHACA188-08	JX836086
Sarcoptiformes	Eremaeidae	*Eueremaeus cf. quadrilamellatus*	CHACB432-10	HQ558588
Sarcoptiformes	Eremaeidae	*Eueremaeus cf. quadrilamellatus*	MYMCA024-11	JX836312
Sarcoptiformes	Eremaeidae	*Eueremaeus cf. quadrilamellatus*	MYMCA1062-11	JX837998
Sarcoptiformes	Eremaeidae	*Eueremaeus cf. quadrilamellatus*	MYMCA1141-11	JX835615
Sarcoptiformes	Eremaeidae	*Eueremaeus cf. quadrilamellatus*	MYMCA282-11	JX838761
Sarcoptiformes	Eremaeidae	*Eueremaeus cf. quadrilamellatus*	MYMCA314-11	JX837791
Sarcoptiformes	Eremaeidae	*Eueremaeus cf. quadrilamellatus*	MYMCA408-11	JX835402
Sarcoptiformes	Eremaeidae	*Eueremaeus cf. quadrilamellatus*	MYMCA430-11	JX837601
Sarcoptiformes	Eremaeidae	*Eueremaeus cf. quadrilamellatus*	MYMCA494-11	JX835575
Sarcoptiformes	Eremaeidae	*Eueremaeus cf. quadrilamellatus*	MYMCA495-11	JX837354
Sarcoptiformes	Eremaeidae	*Eueremaeus cf. quadrilamellatus*	MYMCA496-11	JX837167
Sarcoptiformes	Eremaeidae	*Eueremaeus cf. quadrilamellatus*	MYMCA541-11	JX836982
Sarcoptiformes	Eremaeidae	*Eueremaeus cf. quadrilamellatus*	MYMCA542-11	JX834627
Sarcoptiformes	Eremaeidae	*Eueremaeus cf. quadrilamellatus*	MYMCB144-11	JX838019
Sarcoptiformes	Eremaeidae	*Eueremaeus cf. quadrilamellatus*	MYMCB145-11	JX837502
Sarcoptiformes	Eremaeidae	*Eueremaeus cf. quadrilamellatus*	MYMCB228-11	JX833999
Sarcoptiformes	Eremaeidae	*Eueremaeus cf. quadrilamellatus*	MYMCB265-11	JX838648
Sarcoptiformes	Eremaeidae	*Eueremaeus cf. quadrilamellatus*	MYMCB493-11	JX838479
Sarcoptiformes	Eremaeidae	*Eueremaeus cf. quadrilamellatus*	MYMCB583-11	JX834460
Sarcoptiformes	Eremaeidae	*Eueremaeus cf. quadrilamellatus*	MYMCB739-11	JX838540

Sarcoptiformes	Eremaeidae	*Eueremaeus cf. quadrilamellatus*	MYMCB849-11	JX833983
Sarcoptiformes	Eremaeidae	*Eueremaeus cf. quadrilamellatus*	MYMCC019-11	JX836090
Sarcoptiformes	Eremaeidae	*Eueremaeus cf. quadrilamellatus*	MYMCC020-11	JX834668
Sarcoptiformes	Eremaeidae	*Eueremaeus cf. quadrilamellatus*	MYMCC440-11	JX835993
Sarcoptiformes	Eremaeidae	*Eueremaeus cf. quadrilamellatus*	MYMCC487-11	JX837480
Sarcoptiformes	Eremaeidae	*Eueremaeus cf. quadrilamellatus*	MYMCC599-11	JX834993
Sarcoptiformes	Eremaeidae	*Eueremaeus cf. quadrilamellatus*	MYMCC617-11	JX837996
Sarcoptiformes	Eremaeidae	*Eueremaeus cf. quadrilamellatus*	MYMCC639-11	JX837450
Sarcoptiformes	Eremaeidae	*Eueremaeus cf. quadrilamellatus*	MYMCC640-11	JX836375
Sarcoptiformes	Eremaeidae	*Eueremaeus cf. quadrilamellatus*	MYMCC686-11	JX834053
Sarcoptiformes	Eremaeidae	*Eueremaeus cf. quadrilamellatus*	MYMCC909-11	JX836017
Sarcoptiformes	Eremaeidae	*Eueremaeus cf. quadrilamellatus*	MYMCC910-11	JX835613
Sarcoptiformes	Eremaeidae	*Eueremaeus cf. quadrilamellatus*	MYMCC931-11	JX834677
Sarcoptiformes	Eremaeidae	*Eueremaeus cf. quadrilamellatus*	MYMCE335-12	JX834832
Sarcoptiformes	Eremaeidae	*Eueremaeus cf. quadrilamellatus*	MYMCE473-12	JX834139
Sarcoptiformes	Eremaeidae	*Eueremaeus cf. quadrilamellatus*	MYMCE588-12	JX836732
Sarcoptiformes	Eremaeidae	*Eueremaeus cf. quadrilamellatus*	MYMCE811-12	JX837608
Sarcoptiformes	Eremaeidae	*Eueremaeus cf. quadrilamellatus*	MYMCE920-12	JX838044
Sarcoptiformes	Eremaeidae	*Eueremaeus cf. quadrilamellatus*	MYMCF197-12	JX838696
Sarcoptiformes	Eremaeidae	*Eueremaeus cf. quadrilamellatus*	MYMCF198-12	JX838613
Sarcoptiformes	Eremaeidae	*Eueremaeus cf. quadrilamellatus*	MYMCF638-12	JX833995
Sarcoptiformes	Eremaeidae	*Eueremaeus cf. quadrilamellatus*	MYMCF763-12	JX834091
Sarcoptiformes	Eremaeidae	*Eueremaeus cf. quadrilamellatus*	MYMCG010-12	JX834661
Sarcoptiformes	Eremaeidae	*Eueremaeus cf. quadrilamellatus*	MYMCG011-12	JX836137
Sarcoptiformes	Eremaeidae	*Eueremaeus cf. quadrilamellatus*	MYMCG377-12	JX834173
Sarcoptiformes	Eremaeidae	*Eueremaeus cf. quadrilamellatus*	MYMCG512-12	JX837041
Sarcoptiformes	Eremaeidae	*Eueremaeus cf. quadrilamellatus*	MYMCG544-12	JX835967
Sarcoptiformes	Eremaeidae	*Eueremaeus cf. quadrilamellatus*	MYTMC011-09	GU680486
Sarcoptiformes	Eremaeidae	*Eueremaeus cf. quadrilamellatus*	MYTMC033-09	GU680477
Sarcoptiformes	Eremaeidae	*Eueremaeus cf. quadrilamellatus*	MYTMC038-09	GU680474
Sarcoptiformes	Eremaeidae	*Eueremaeus cf. quadrilamellatus*	MYTMC039-09	KR069710
Sarcoptiformes	Eremaeidae	*Eueremaeus cf. quadrilamellatus*	MYTMC044-09	GU680475
Sarcoptiformes	Eremaeidae	*Eueremaeus cf. quadrilamellatus*	MYTMC072-09	GU680462
Sarcoptiformes	Eremaeidae	*Eueremaeus cf. quadrilamellatus*	MYTMC105-09	GU680491
Sarcoptiformes	Eremaeidae	*Eueremaeus cf. quadrilamellatus*	MYTMC109-09	GU680492
Sarcoptiformes	Eremaeidae	*Eueremaeus cf. quadrilamellatus*	MYTMC185-09	GU680437
Sarcoptiformes	Eremaeidae	*Eueremaeus cf. quadrilamellatus*	MYTMC186-09	GU680438
Sarcoptiformes	Eremaeidae	*Eueremaeus chiatous*	CNBAF200-12	KP979204
Sarcoptiformes	Eremaeidae	*Eueremaeus chiatous*	CNBAI495-13	KM830060
Sarcoptiformes	Eremaeidae	*Eueremaeus chiatous*	SSEIC1002-13	KM834305
Sarcoptiformes	Eremaeidae	*Eueremaeus chiatous*	SSEIC1299-13	KM826583
Sarcoptiformes	Eremaeidae	*Eueremaeus chiatous*	SSEIC988-13	KR069564
Sarcoptiformes	Eremaeidae	*Eueremaeus chiatous*	SSEIC991-13	KR070438
Sarcoptiformes	Eremaeidae	*Eueremaeus chiatous*	SSPAB3983-13	KM836285
Sarcoptiformes	Eremaeidae	*Eueremaeus chiatous*	SSPAB3989-13	KR070524
Sarcoptiformes	Eremaeidae	*Eueremaeus chiatous*	SSPAB4030-13	KM824005
Sarcoptiformes	Eremaeidae	*Eueremaeus chiatous*	SSPAB7988-13	KM825758
Sarcoptiformes	Eremaeidae	*Eueremaeus chiatous*	SSWLF652-13	KM834511
Sarcoptiformes	Eremaeidae	*Eueremaeus chiatous*	SSWLF658-13	KM828732
Sarcoptiformes	Eremaeidae	*Eueremaeus chiatous*	SSWLF659-13	KM832614
Sarcoptiformes	Eremaeidae	*Eueremaeus chiatous*	SSWLF660-13	KM829532
Sarcoptiformes	Eremaeidae	*Eueremaeus chiatous*	SSWLF666-13	KM834055
Sarcoptiformes	Eremaeidae	*Eueremaeus chiatous*	SSWLF668-13	KM839242

Sarcoptiformes	Eremaeidae	*Eueremaeus chiatous*	SSWLF766-13	KM830275
Sarcoptiformes	Eremaeidae	*Eueremaeus marshalli*	CHACB052-10	HQ558361
Sarcoptiformes	Eremaeidae	*Eueremaeus marshalli*	CHACB673-10	HQ558741
Sarcoptiformes	Eremaeidae	*Eueremaeus marshalli*	CHACB841-10	HQ941511
Sarcoptiformes	Eremaeidae	*Eueremaeus marshalli*	CHACB843-10	HQ941512
Sarcoptiformes	Eremaeidae	*Eueremaeus marshalli*	CHACB845-10	HQ941513
Sarcoptiformes	Eremaeidae	*Eueremaeus marshalli*	CNBPC215-12	KM835333
Sarcoptiformes	Eremaeidae	*Eueremaeus marshalli*	CNBPE660-12	KM833216
Sarcoptiformes	Eremaeidae	*Eueremaeus marshalli*	CNBPK331-13	KM840304
Sarcoptiformes	Eremaeidae	*Eueremaeus marshalli*	CNBPL242-13	KM833573
Sarcoptiformes	Eremaeidae	*Eueremaeus marshalli*	CNBPL245-13	KM830136
Sarcoptiformes	Eremaeidae	*Eueremaeus marshalli*	CNBPN254-13	KM830118
Sarcoptiformes	Eremaeidae	*Eueremaeus marshalli*	MYMCA313-11	JX835490
Sarcoptiformes	Eremaeidae	*Eueremaeus marshalli*	MYMCA431-11	JX837346
Sarcoptiformes	Eremaeidae	*Eueremaeus marshalli*	MYMCB882-11	JX835022
Sarcoptiformes	Eremaeidae	*Eueremaeus marshalli*	MYMCB883-11	JX833700
Sarcoptiformes	Eremaeidae	*Eueremaeus marshalli*	MYMCC507-11	JX838168
Sarcoptiformes	Eremaeidae	*Eueremaeus marshalli*	MYMCC842-11	JX836082
Sarcoptiformes	Eremaeidae	*Eueremaeus marshalli*	MYMCC930-11	JX838352
Sarcoptiformes	Eremaeidae	*Eueremaeus marshalli*	MYMCF238-12	JX838643
Sarcoptiformes	Eremaeidae	*Eueremaeus marshalli*	MYMCF299-12	JX838265
Sarcoptiformes	Eremaeidae	*Eueremaeus marshalli*	MYMCF444-12	JX838710
Sarcoptiformes	Eremaeidae	*Eueremaeus marshalli*	MYMCF445-12	JX838596
Sarcoptiformes	Eremaeidae	*Eueremaeus marshalli*	MYMCF694-12	JX835496
Sarcoptiformes	Eremaeidae	*Eueremaeus marshalli*	MYMCF805-12	JX834224
Sarcoptiformes	Eremaeidae	*Eueremaeus marshalli*	MYMCG422-12	JX836794
Sarcoptiformes	Eremaeidae	*Eueremaeus quadrilamellatus*	CHACB053-10	HQ558362
Sarcoptiformes	Eremaeidae	*Eueremaeus quadrilamellatus*	CHACB842-10	JX836149
Sarcoptiformes	Eremaeidae	*Eueremaeus quadrilamellatus*	MYMCF666-12	JX837845
Sarcoptiformes	Eremaeidae	*Eueremaeus quadrilamellatus*	MYMCG282-12	JX833684
Sarcoptiformes	Eremaeidae	*Eueremaeus tetrosus*	SSBAA3660-12	KM839599
Sarcoptiformes	Eremaeidae	*Eueremaeus tetrosus*	SSBAA3685-12	KM827623
Sarcoptiformes	Eremaeidae	*Eueremaeus tetrosus*	SSBAA3691-12	KM829327
Sarcoptiformes	Eremaeidae	*Eueremaeus tetrosus*	SSBAD4048-12	KM827727
Sarcoptiformes	Eremaeidae	*Eueremaeus tetrosus*	SSBAD4688-13	KM839082
Sarcoptiformes	Eremaeidae		CNGLC082-13	KM832276
Sarcoptiformes	Eremaeidae		CNGLC085-13	KM829296
Sarcoptiformes	Eremaeidae		CNGLD020-13	KM838964
Sarcoptiformes	Eremaeidae		CNGLE042-13	KM829792
Sarcoptiformes	Eremaeidae		MYMCA828-11	JX837285
Sarcoptiformes	Eremaeidae		MYMCB087-11	JX834312
Sarcoptiformes	Eremaeidae		MYMCB088-11	JX837775
Sarcoptiformes	Eremaeidae		MYMCC690-11	JX838474
Sarcoptiformes	Eremaeidae		MYMCC691-11	JX835511
Sarcoptiformes	Eremaeidae		MYMCE192-12	JX835153
Sarcoptiformes	Eremaeidae		MYMCE201-12	JX834967
Sarcoptiformes	Eremaeidae		MYMCF834-12	JX835761
Sarcoptiformes	Eremaeidae		MYMCG448-12	JX834111
Sarcoptiformes	Eremaeidae		MYMCG449-12	JX837968
Sarcoptiformes	Eremaeidae		SSBAA3663-12	KM835787
Sarcoptiformes	Eremaeidae		SSBAA3679-12	KM840040
Sarcoptiformes	Eremaeidae		SSBAB1924-12	KM826830
Sarcoptiformes	Eremaeidae		SSBAB2079-12	KM838742

Sarcoptiformes	Eremaeidae		SSBAD6334-13	KM837104
Sarcoptiformes	Eremaeidae		SSEIA7698-13	KM826325
Sarcoptiformes	Eremaeidae		SSJAA1811-13	KM824283
Sarcoptiformes	Eremaeidae		SSJAA2032-13	KM833610
Sarcoptiformes	Eremaeidae		SSJAA2034-13	KM832787
Sarcoptiformes	Eremaeidae		SSJAA2057-13	KM827815
Sarcoptiformes	Eremaeidae		SSJAC1513-13	KM828650
Sarcoptiformes	Eremaeidae		SSJAC1582-13	KM828671
Sarcoptiformes	Eremaeidae		SSJAC969-13	KM838116
Sarcoptiformes	Eremaeidae		SSPAA6644-13	KM838138
Sarcoptiformes	Eremaeidae		SSPAA8005-13	KM838114
Sarcoptiformes	Eremaeidae		SSWEE036-13	KM833186
Sarcoptiformes	Eremaeidae		SSWLA5281-13	KM826622
Sarcoptiformes	Eremaeidae		SSWLC065-13	KM833443
Sarcoptiformes	Eremaeidae		SSWLC1166-13	KM832856
Sarcoptiformes	Eremaeidae		SSWLC1170-13	KM833883
Sarcoptiformes	Eremaeidae		SSWLD2950-13	KR069798
Sarcoptiformes	Eremaeidae		SSWLD2960-13	KM824810
Sarcoptiformes	Eremaeidae		SSWLD3442-13	KM840072
Sarcoptiformes	Eremaeidae		SSWLD3452-13	KM825057
Sarcoptiformes	Eremaeidae		SSWLD3456-13	KR069694
Sarcoptiformes	Eremaeidae		SSWLD3491-13	KM827099
Sarcoptiformes	Eremaeidae		SSWLD3660-13	KM838758
Sarcoptiformes	Eremaeidae		SSWLD8154-13	KM826715
Sarcoptiformes	Eremaeidae		SSWLD8204-13	KM830064
Sarcoptiformes	Eremaeidae		SSWLD8223-13	KM824856
Sarcoptiformes	Eremaeidae		SSWLD8235-13	KM833550
Sarcoptiformes	Eremaeidae		SSWLD8291-13	KM827121
Sarcoptiformes	Eremaeidae		SSWLD8293-13	KM838311
Sarcoptiformes	Eremaeidae		SSWLD8307-13	KM828213
Sarcoptiformes	Eremaeidae		SSWLD8333-13	KM824212
Sarcoptiformes	Eremaeidae		SSWLD8345-13	KM833981
Sarcoptiformes	Eremaeidae		SSWLF3028-13	KM828657
Sarcoptiformes	Eremaeidae		SSWLF3029-13	KM830286
Sarcoptiformes	Eremaeidae		SSWLF3033-13	KM828508
Sarcoptiformes	Eremaeidae		SSWLF3040-13	KM833069
Sarcoptiformes	Eremaeidae		SSWLF3069-13	KM825334
Sarcoptiformes	Eremaeidae		SSWLF3080-13	KM824195
Sarcoptiformes	Eremaeidae		SSWLF3085-13	KM836603
Sarcoptiformes	Eremaeidae		SSWLF3093-13	KM826252
Sarcoptiformes	Eremaeidae		SSWLF3100-13	KM839003
Sarcoptiformes	Eremaeidae		SSWLF3104-13	KM837001
Sarcoptiformes	Eremaeidae		SSWLF3107-13	KM835609
Sarcoptiformes	Eremaeidae		SSWLF3180-13	KM836684
Sarcoptiformes	Eremaeidae		SSWLF3196-13	KM829213
Sarcoptiformes	Eremobelbidae		MIONB133-10	KR069467
Sarcoptiformes	Eremobelbidae		MIONB134-10	KR069481
Sarcoptiformes	Eremobelbidae		MIONB135-10	KP979314
Sarcoptiformes	Eremobelbidae		MIONB136-10	KR070397
Sarcoptiformes	Eremulidae	*Eremulus*	CHACA951-10	HM405824
Sarcoptiformes	Eremulidae	*Eremulus*	CHACA954-10	HM405826
Sarcoptiformes	Euphthiracaridae		SSROA1923-14	KR070690
Sarcoptiformes	Euphthiracaridae		SSROA1974-14	KR069705

Sarcoptiformes	Euphthiracaridae		SSROA1983-14	KR070500
Sarcoptiformes	Euphthiracaridae	*Rhysotritia ardua*	CHACB901-10	HM907333
Sarcoptiformes	Euphthiracaridae	*Rhysotritia ardua*	MYMCA1313-11	JX835949
Sarcoptiformes	Euphthiracaridae	*Rhysotritia ardua*	MYMCA1314-11	JX835607
Sarcoptiformes	Euphthiracaridae	*Rhysotritia ardua*	MYMCB394-11	JX838135
Sarcoptiformes	Euphthiracaridae	*Rhysotritia ardua*	MYMCB395-11	JX834203
Sarcoptiformes	Euphthiracaridae	*Rhysotritia ardua*	MYMCB418-11	JX838054
Sarcoptiformes	Euphthiracaridae	*Rhysotritia ardua*	MYMCB495-11	JX835110
Sarcoptiformes	Euphthiracaridae	*Rhysotritia ardua*	MYMCB496-11	JX835672
Sarcoptiformes	Euphthiracaridae	*Rhysotritia ardua*	MYMCB576-11	JX835296
Sarcoptiformes	Euphthiracaridae	*Rhysotritia ardua*	MYMCB577-11	JX835528
Sarcoptiformes	Euphthiracaridae	*Rhysotritia ardua*	MYMCB578-11	JX834443
Sarcoptiformes	Euphthiracaridae	*Rhysotritia ardua*	MYMCB736-11	JX835629
Sarcoptiformes	Euphthiracaridae	*Rhysotritia ardua*	MYMCC186-11	JX835898
Sarcoptiformes	Euphthiracaridae	*Rhysotritia ardua*	MYMCC187-11	JX837531
Sarcoptiformes	Euphthiracaridae	*Rhysotritia ardua*	MYMCC226-11	JX834270
Sarcoptiformes	Euphthiracaridae	*Rhysotritia ardua*	MYMCC674-11	JX837457
Sarcoptiformes	Euphthiracaridae	*Rhysotritia ardua*	MYMCC675-11	JX835349
Sarcoptiformes	Euphthiracaridae	*Rhysotritia ardua*	MYMCF933-12	JX837793
Sarcoptiformes	Euphthiracaridae	*Rhysotritia ardua*	MYMCG012-12	JX838307
Sarcoptiformes	Euphthiracaridae	*Rhysotritia ardua*	MYTMC167-09	HQ966245
Sarcoptiformes	Euphthiracaridae	*Rhysotritia ardua*	SSPAA7794-13	KM833502
Sarcoptiformes	Euphthiracaridae		RBINA5628-13	KR070742
Sarcoptiformes	Euphthiracaridae		RBINA5636-13	KR069146
Sarcoptiformes	Euphthiracaridae		RBINA5646-13	KR070713
Sarcoptiformes	Euphthiracaridae		RBINA5668-13	KP979145
Sarcoptiformes	Euphthiracaridae		RBINA5669-13	KP979310
Sarcoptiformes	Euphthiracaridae		RBINA5724-13	KR070095
Sarcoptiformes	Euphthiracaridae		RBINA5761-13	KP979343
Sarcoptiformes	Euzetidae	*Euzetes*	SSROA1921-14	KR070506
Sarcoptiformes	Euzetidae	*Euzetes*	SSROA1922-14	KR069668
Sarcoptiformes	Euzetidae	*Euzetes*	SSROA1934-14	KR069733
Sarcoptiformes	Euzetidae	*Euzetes*	SSROA1941-14	KR069205
Sarcoptiformes	Euzetidae	*Euzetes*	SSROA1944-14	KR069159
Sarcoptiformes	Euzetidae	*Euzetes*	SSROA1949-14	KR069326
Sarcoptiformes	Euzetidae	*Euzetes*	SSROA1955-14	KR070195
Sarcoptiformes	Euzetidae	*Euzetes*	SSROA1959-14	KR069144
Sarcoptiformes	Euzetidae	*Euzetes*	SSROA1965-14	KR070479
Sarcoptiformes	Euzetidae	*Euzetes*	SSROA1972-14	KR069271
Sarcoptiformes	Euzetidae	*Euzetes*	SSROA1976-14	KR070390
Sarcoptiformes	Euzetidae	*Euzetes*	SSROA3046-14	KR069652
Sarcoptiformes	Euzetidae	*Euzetes*	SSROA3049-14	KR070261
Sarcoptiformes	Euzetidae	*Euzetes*	SSROA3053-14	KR070401
Sarcoptiformes	Euzetidae	*Euzetes*	SSROA3057-14	KR069194
Sarcoptiformes	Euzetidae	*Euzetes*	SSROA3062-14	KR070052
Sarcoptiformes	Euzetidae	*Euzetes*	SSROA3074-14	KR069562
Sarcoptiformes	Euzetidae	*Euzetes*	SSROA3076-14	KR070167
Sarcoptiformes	Euzetidae	*Euzetes*	SSROA3078-14	KR069846
Sarcoptiformes	Euzetidae	*Euzetes*	SSROA3085-14	KR070040
Sarcoptiformes	Euzetidae	*Euzetes*	SSROA3617-14	KR070433
Sarcoptiformes	Euzetidae	*Euzetes*	SSROA3625-14	KR070060
Sarcoptiformes	Euzetidae	*Euzetes*	SSROA3636-14	KR070268
Sarcoptiformes	Euzetidae	*Euzetes*	SSROA3659-14	KR069905

Sarcoptiformes	Euzetidae	*Euzetes*	SSROA3663-14	KR069603
Sarcoptiformes	Euzetidae	*Euzetes*	SSROA3674-14	KR069450
Sarcoptiformes	Euzetidae	*Euzetes*	SSROA3699-14	KR070640
Sarcoptiformes	Euzetidae	*Euzetes*	SSROA3714-14	KR069544
Sarcoptiformes	Euzetidae	*Euzetes*	SSROA3720-14	KR070061
Sarcoptiformes	Euzetidae	*Euzetes*	SSROA3722-14	KR069700
Sarcoptiformes	Euzetidae	*Euzetes*	SSROA3724-14	KR070353
Sarcoptiformes	Euzetidae	*Euzetes*	SSROA3731-14	KR070009
Sarcoptiformes	Euzetidae	*Euzetes*	SSROA3733-14	KR070651
Sarcoptiformes	Euzetidae	*Euzetes*	BBLZI149-14	KR069295
Sarcoptiformes	Euzetidae	*Euzetes*	RBINA2936-13	KP979293
Sarcoptiformes	Euzetidae	*Euzetes*	RBINA2939-13	KR069796
Sarcoptiformes	Euzetidae	*Euzetes*	RBINA3954-13	KR070415
Sarcoptiformes	Euzetidae	*Euzetes*	RBINA5568-13	KR070445
Sarcoptiformes	Euzetidae	*Euzetes*	RBINA5656-13	KR070744
Sarcoptiformes	Euzetidae	*Euzetes*	RBINA5755-13	KR069351
Sarcoptiformes	Euzetidae	*Euzetes globulus*	SSROA1925-14	KR070176
Sarcoptiformes	Euzetidae	*Euzetes globulus*	SSROA1926-14	KR070043
Sarcoptiformes	Euzetidae	*Euzetes globulus*	SSROA1927-14	KR069799
Sarcoptiformes	Euzetidae	*Euzetes globulus*	SSROA1928-14	KR069788
Sarcoptiformes	Euzetidae	*Euzetes globulus*	SSROA1929-14	KR070446
Sarcoptiformes	Euzetidae	*Euzetes globulus*	SSROA1930-14	KR069752
Sarcoptiformes	Euzetidae	*Euzetes globulus*	SSROA1931-14	KR070201
Sarcoptiformes	Euzetidae	*Euzetes globulus*	SSROA1932-14	KR070129
Sarcoptiformes	Euzetidae	*Euzetes globulus*	SSROA1933-14	KR070037
Sarcoptiformes	Euzetidae	*Euzetes globulus*	SSROA1935-14	KR069278
Sarcoptiformes	Euzetidae	*Euzetes globulus*	SSROA1936-14	KR070379
Sarcoptiformes	Euzetidae	*Euzetes globulus*	SSROA1937-14	KR069234
Sarcoptiformes	Euzetidae	*Euzetes globulus*	SSROA1938-14	KR070378
Sarcoptiformes	Euzetidae	*Euzetes globulus*	SSROA1939-14	KR069813
Sarcoptiformes	Euzetidae	*Euzetes globulus*	SSROA1940-14	KR070427
Sarcoptiformes	Euzetidae	*Euzetes globulus*	SSROA1942-14	KR069316
Sarcoptiformes	Euzetidae	*Euzetes globulus*	SSROA1943-14	KR070716
Sarcoptiformes	Euzetidae	*Euzetes globulus*	SSROA1945-14	KR070300
Sarcoptiformes	Euzetidae	*Euzetes globulus*	SSROA1946-14	KR070119
Sarcoptiformes	Euzetidae	*Euzetes globulus*	SSROA1947-14	KR069720
Sarcoptiformes	Euzetidae	*Euzetes globulus*	SSROA1948-14	KR070604
Sarcoptiformes	Euzetidae	*Euzetes globulus*	SSROA1950-14	KR069841
Sarcoptiformes	Euzetidae	*Euzetes globulus*	SSROA1951-14	KR070083
Sarcoptiformes	Euzetidae	*Euzetes globulus*	SSROA1952-14	KR070458
Sarcoptiformes	Euzetidae	*Euzetes globulus*	SSROA1953-14	KR069948
Sarcoptiformes	Euzetidae	*Euzetes globulus*	SSROA1954-14	KR069370
Sarcoptiformes	Euzetidae	*Euzetes globulus*	SSROA1956-14	KR070444
Sarcoptiformes	Euzetidae	*Euzetes globulus*	SSROA1957-14	KR069850
Sarcoptiformes	Euzetidae	*Euzetes globulus*	SSROA1958-14	KR070552
Sarcoptiformes	Euzetidae	*Euzetes globulus*	SSROA1960-14	KR069342
Sarcoptiformes	Euzetidae	*Euzetes globulus*	SSROA1961-14	KR070589
Sarcoptiformes	Euzetidae	*Euzetes globulus*	SSROA1962-14	KR069833
Sarcoptiformes	Euzetidae	*Euzetes globulus*	SSROA1963-14	KR070316
Sarcoptiformes	Euzetidae	*Euzetes globulus*	SSROA1964-14	KR069559
Sarcoptiformes	Euzetidae	*Euzetes globulus*	SSROA1966-14	KR070586
Sarcoptiformes	Euzetidae	*Euzetes globulus*	SSROA1967-14	KR070571
Sarcoptiformes	Euzetidae	*Euzetes globulus*	SSROA1968-14	KR069852

Sarcoptiformes	Euzetidae	*Euzetes globulus*	SSROA1969-14	KR069197
Sarcoptiformes	Euzetidae	*Euzetes globulus*	SSROA1970-14	KR069426
Sarcoptiformes	Euzetidae	*Euzetes globulus*	SSROA1971-14	KR070357
Sarcoptiformes	Euzetidae	*Euzetes globulus*	SSROA1973-14	KR070630
Sarcoptiformes	Euzetidae	*Euzetes globulus*	SSROA1975-14	KR070343
Sarcoptiformes	Euzetidae	*Euzetes globulus*	SSROA1977-14	KR069792
Sarcoptiformes	Euzetidae	*Euzetes globulus*	SSROA1978-14	KR070737
Sarcoptiformes	Euzetidae	*Euzetes globulus*	SSROA1979-14	KR069927
Sarcoptiformes	Euzetidae	*Euzetes globulus*	SSROA1980-14	KR070410
Sarcoptiformes	Euzetidae	*Euzetes globulus*	SSROA1981-14	KR070007
Sarcoptiformes	Euzetidae	*Euzetes globulus*	SSROA1982-14	KR070373
Sarcoptiformes	Euzetidae	*Euzetes globulus*	SSROA1984-14	KR069283
Sarcoptiformes	Euzetidae	*Euzetes globulus*	SSROA1985-14	KR069386
Sarcoptiformes	Euzetidae	*Euzetes globulus*	SSROA2431-14	KR069329
Sarcoptiformes	Euzetidae	*Euzetes globulus*	SSROA3040-14	KR070136
Sarcoptiformes	Euzetidae	*Euzetes globulus*	SSROA3041-14	KR069642
Sarcoptiformes	Euzetidae	*Euzetes globulus*	SSROA3042-14	KR070297
Sarcoptiformes	Euzetidae	*Euzetes globulus*	SSROA3044-14	KR069515
Sarcoptiformes	Euzetidae	*Euzetes globulus*	SSROA3045-14	KR070050
Sarcoptiformes	Euzetidae	*Euzetes globulus*	SSROA3047-14	KR070480
Sarcoptiformes	Euzetidae	*Euzetes globulus*	SSROA3048-14	KR069884
Sarcoptiformes	Euzetidae	*Euzetes globulus*	SSROA3050-14	KR070323
Sarcoptiformes	Euzetidae	*Euzetes globulus*	SSROA3051-14	KR070611
Sarcoptiformes	Euzetidae	*Euzetes globulus*	SSROA3052-14	KR069226
Sarcoptiformes	Euzetidae	*Euzetes globulus*	SSROA3054-14	KR069925
Sarcoptiformes	Euzetidae	*Euzetes globulus*	SSROA3055-14	KR070096
Sarcoptiformes	Euzetidae	*Euzetes globulus*	SSROA3056-14	KR069556
Sarcoptiformes	Euzetidae	*Euzetes globulus*	SSROA3058-14	KR070683
Sarcoptiformes	Euzetidae	*Euzetes globulus*	SSROA3061-14	KR070547
Sarcoptiformes	Euzetidae	*Euzetes globulus*	SSROA3063-14	KR069604
Sarcoptiformes	Euzetidae	*Euzetes globulus*	SSROA3064-14	KR070631
Sarcoptiformes	Euzetidae	*Euzetes globulus*	SSROA3065-14	KR069677
Sarcoptiformes	Euzetidae	*Euzetes globulus*	SSROA3066-14	KR069822
Sarcoptiformes	Euzetidae	*Euzetes globulus*	SSROA3068-14	KR069523
Sarcoptiformes	Euzetidae	*Euzetes globulus*	SSROA3072-14	KR069674
Sarcoptiformes	Euzetidae	*Euzetes globulus*	SSROA3075-14	KR069883
Sarcoptiformes	Euzetidae	*Euzetes globulus*	SSROA3077-14	KR069340
Sarcoptiformes	Euzetidae	*Euzetes globulus*	SSROA3083-14	KR070664
Sarcoptiformes	Euzetidae	*Euzetes globulus*	SSROA3084-14	KR069706
Sarcoptiformes	Euzetidae	*Euzetes globulus*	SSROA3097-14	KR069829
Sarcoptiformes	Euzetidae	*Euzetes globulus*	SSROA3098-14	KR070498
Sarcoptiformes	Euzetidae	*Euzetes globulus*	SSROA3099-14	KR069943
Sarcoptiformes	Euzetidae	*Euzetes globulus*	SSROA3103-14	KR069566
Sarcoptiformes	Euzetidae	*Euzetes globulus*	SSROA3615-14	KR069483
Sarcoptiformes	Euzetidae	*Euzetes globulus*	SSROA3621-14	KR069518
Sarcoptiformes	Euzetidae	*Euzetes globulus*	SSROA3623-14	KR069591
Sarcoptiformes	Euzetidae	*Euzetes globulus*	SSROA3626-14	KR070084
Sarcoptiformes	Euzetidae	*Euzetes globulus*	SSROA3628-14	KR069451
Sarcoptiformes	Euzetidae	*Euzetes globulus*	SSROA3631-14	KR070255
Sarcoptiformes	Euzetidae	*Euzetes globulus*	SSROA3632-14	KR069892
Sarcoptiformes	Euzetidae	*Euzetes globulus*	SSROA3633-14	KR070157
Sarcoptiformes	Euzetidae	*Euzetes globulus*	SSROA3635-14	KR069456
Sarcoptiformes	Euzetidae	*Euzetes globulus*	SSROA3642-14	KR069810

Sarcoptiformes	Euzetidae	*Euzetes globulus*	SSROA3644-14	KR069961
Sarcoptiformes	Euzetidae	*Euzetes globulus*	SSROA3654-14	KR070392
Sarcoptiformes	Euzetidae	*Euzetes globulus*	SSROA3655-14	KR070628
Sarcoptiformes	Euzetidae	*Euzetes globulus*	SSROA3656-14	KR069175
Sarcoptiformes	Euzetidae	*Euzetes globulus*	SSROA3657-14	KR069887
Sarcoptiformes	Euzetidae	*Euzetes globulus*	SSROA3658-14	KR070039
Sarcoptiformes	Euzetidae	*Euzetes globulus*	SSROA3660-14	KR069845
Sarcoptiformes	Euzetidae	*Euzetes globulus*	SSROA3661-14	KR069956
Sarcoptiformes	Euzetidae	*Euzetes globulus*	SSROA3662-14	KR070657
Sarcoptiformes	Euzetidae	*Euzetes globulus*	SSROA3664-14	KR069560
Sarcoptiformes	Euzetidae	*Euzetes globulus*	SSROA3696-14	KR069785
Sarcoptiformes	Euzetidae	*Euzetes globulus*	SSROA3697-14	KR070000
Sarcoptiformes	Euzetidae	*Euzetes globulus*	SSROA3698-14	KR070656
Sarcoptiformes	Euzetidae	*Euzetes globulus*	SSROA3700-14	KR070326
Sarcoptiformes	Euzetidae	*Euzetes globulus*	SSROA3702-14	KR070466
Sarcoptiformes	Euzetidae	*Euzetes globulus*	SSROA3703-14	KR070443
Sarcoptiformes	Euzetidae	*Euzetes globulus*	SSROA3704-14	KR070723
Sarcoptiformes	Euzetidae	*Euzetes globulus*	SSROA3705-14	KR069445
Sarcoptiformes	Euzetidae	*Euzetes globulus*	SSROA3706-14	KR069933
Sarcoptiformes	Euzetidae	*Euzetes globulus*	SSROA3707-14	KR069834
Sarcoptiformes	Euzetidae	*Euzetes globulus*	SSROA3708-14	KR069422
Sarcoptiformes	Euzetidae	*Euzetes globulus*	SSROA3709-14	KR069161
Sarcoptiformes	Euzetidae	*Euzetes globulus*	SSROA3710-14	KR069572
Sarcoptiformes	Euzetidae	*Euzetes globulus*	SSROA3711-14	KR070187
Sarcoptiformes	Euzetidae	*Euzetes globulus*	SSROA3712-14	KR069865
Sarcoptiformes	Euzetidae	*Euzetes globulus*	SSROA3713-14	KR070121
Sarcoptiformes	Euzetidae	*Euzetes globulus*	SSROA3715-14	KR070259
Sarcoptiformes	Euzetidae	*Euzetes globulus*	SSROA3716-14	KR070354
Sarcoptiformes	Euzetidae	*Euzetes globulus*	SSROA3717-14	KR070235
Sarcoptiformes	Euzetidae	*Euzetes globulus*	SSROA3718-14	KR070267
Sarcoptiformes	Euzetidae	*Euzetes globulus*	SSROA3719-14	KR070509
Sarcoptiformes	Euzetidae	*Euzetes globulus*	SSROA3721-14	KR070146
Sarcoptiformes	Euzetidae	*Euzetes globulus*	SSROA3723-14	KR069449
Sarcoptiformes	Euzetidae	*Euzetes globulus*	SSROA3725-14	KR070152
Sarcoptiformes	Euzetidae	*Euzetes globulus*	SSROA3726-14	KR069157
Sarcoptiformes	Euzetidae	*Euzetes globulus*	SSROA3727-14	KR070005
Sarcoptiformes	Euzetidae	*Euzetes globulus*	SSROA3728-14	KR069610
Sarcoptiformes	Euzetidae	*Euzetes globulus*	SSROA3729-14	KR070226
Sarcoptiformes	Euzetidae	*Euzetes globulus*	SSROA3730-14	KR069517
Sarcoptiformes	Euzetidae	*Euzetes globulus*	SSROA3732-14	KR069636
Sarcoptiformes	Euzetidae	*Euzetes globulus*	SSROA3734-14	KR070477
Sarcoptiformes	Euzetidae	*Euzetes globulus*	SSROA3735-14	KR070676
Sarcoptiformes	Euzetidae	*Euzetes globulus*	SSROA3736-14	KR069598
Sarcoptiformes	Euzetidae	*Euzetes globulus*	SSROA3737-14	KR069239
Sarcoptiformes	Euzetidae	*Euzetes globulus*	SSROA3738-14	KR070185
Sarcoptiformes	Euzetidae	*Euzetes globulus*	SSROA3740-14	KR069888
Sarcoptiformes	Euzetidae	*Euzetes globulus*	SSROA3741-14	KR070475
Sarcoptiformes	Euzetidae	*Euzetes globulus*	SSROA3771-14	KR070304
Sarcoptiformes	Euzetidae	*Euzetes globulus*	SSROA3781-14	KR070494
Sarcoptiformes	Euzetidae	*Euzetes globulus*	SSROA3782-14	KR070389
Sarcoptiformes	Euzetidae	*Euzetes globulus*	SSROA3783-14	KR070109
Sarcoptiformes	Euzetidae	*Euzetes globulus*	CNROJ1084-13	KR070082
Sarcoptiformes	Euzetidae	*Euzetes globulus*	RBINA1127-13	KR069699

Sarcoptiformes	Euzetidae	*Euzetes globulus*	RBINA1135-13	KR069236
Sarcoptiformes	Euzetidae	*Euzetes globulus*	RBINA1136-13	KR069336
Sarcoptiformes	Euzetidae	*Euzetes globulus*	RBINA1137-13	KR070647
Sarcoptiformes	Euzetidae	*Euzetes globulus*	RBINA1142-13	KR069539
Sarcoptiformes	Euzetidae	*Euzetes globulus*	RBINA1144-13	KR069638
Sarcoptiformes	Euzetidae	*Euzetes globulus*	RBINA1145-13	KR069823
Sarcoptiformes	Euzetidae	*Euzetes globulus*	RBINA1165-13	KR070220
Sarcoptiformes	Euzetidae	*Euzetes globulus*	RBINA1166-13	KR069419
Sarcoptiformes	Euzetidae	*Euzetes globulus*	RBINA1176-13	KR069666
Sarcoptiformes	Euzetidae	*Euzetes globulus*	RBINA2929-13	KR069949
Sarcoptiformes	Euzetidae	*Euzetes globulus*	RBINA2930-13	KR069570
Sarcoptiformes	Euzetidae	*Euzetes globulus*	RBINA2931-13	KR070701
Sarcoptiformes	Euzetidae	*Euzetes globulus*	RBINA2932-13	KR069649
Sarcoptiformes	Euzetidae	*Euzetes globulus*	RBINA2933-13	KR069186
Sarcoptiformes	Euzetidae	*Euzetes globulus*	RBINA2934-13	KR069488
Sarcoptiformes	Euzetidae	*Euzetes globulus*	RBINA2935-13	KR070269
Sarcoptiformes	Euzetidae	*Euzetes globulus*	RBINA2937-13	KR069502
Sarcoptiformes	Euzetidae	*Euzetes globulus*	RBINA2938-13	KR070370
Sarcoptiformes	Euzetidae	*Euzetes globulus*	RBINA2940-13	KR069270
Sarcoptiformes	Euzetidae	*Euzetes globulus*	RBINA2941-13	KR069628
Sarcoptiformes	Euzetidae	*Euzetes globulus*	RBINA2942-13	KR069199
Sarcoptiformes	Euzetidae	*Euzetes globulus*	RBINA2943-13	KR069973
Sarcoptiformes	Euzetidae	*Euzetes globulus*	RBINA3145-13	KR070551
Sarcoptiformes	Euzetidae	*Euzetes globulus*	RBINA5567-13	KR069487
Sarcoptiformes	Euzetidae	*Euzetes globulus*	RBINA5654-13	KR069273
Sarcoptiformes	Euzetidae	*Euzetes globulus*	RBINA5655-13	KR069611
Sarcoptiformes	Euzetidae	*Euzetes globulus*	RBINA5660-13	KR070347
Sarcoptiformes	Euzetidae	*Euzetes globulus*	RBINA5661-13	KR070593
Sarcoptiformes	Euzetidae	*Euzetes globulus*	RBINA5691-13	KP979202
Sarcoptiformes	Euzetidae	*Euzetes globulus*	RBINA5692-13	KR070681
Sarcoptiformes	Euzetidae	*Euzetes globulus*	RBINA5717-13	KR070239
Sarcoptiformes	Euzetidae	*Euzetes globulus*	RBINA5718-13	KR069809
Sarcoptiformes	Euzetidae	*Euzetes globulus*	RBINA5731-13	KR070662
Sarcoptiformes	Euzetidae	*Euzetes globulus*	RBINA5732-13	KR070291
Sarcoptiformes	Euzetidae	*Euzetes globulus*	RBINA5748-13	KR069395
Sarcoptiformes	Euzetidae	*Euzetes globulus*	RBINA5749-13	KR070692
Sarcoptiformes	Euzetidae	*Euzetes globulus*	RBINA5756-13	KR069230
Sarcoptiformes	Euzetidae	*Euzetes globulus*	RBINA5769-13	KR070719
Sarcoptiformes	Euzetidae	*Euzetes globulus*	RBINA5770-13	KR069528
Sarcoptiformes	Euzetidae	*Euzetes globulus*	RBINA5778-13	KR070434
Sarcoptiformes	Euzetidae	*Euzetes globulus*	RBINA5779-13	KR069590
Sarcoptiformes	Gabuciniidae	*Gabucinia delibata*	FMEP278-14	KP193464
Sarcoptiformes	Gabuciniidae	*Hieracolichus nisi*	GBCH4442-10	GQ864360
Sarcoptiformes	Galumnidae		SSROA3673-14	KR069516
Sarcoptiformes	Galumnidae		SSROA3768-14	KR070126
Sarcoptiformes	Galumnidae		SSROA3769-14	KR070710
Sarcoptiformes	Galumnidae	*Galumna*	CHACA1204-10	JX834084
Sarcoptiformes	Galumnidae	*Galumna*	CHACA1207-10	JX835163
Sarcoptiformes	Galumnidae	*Galumna*	CHACB139-10	HQ558423
Sarcoptiformes	Galumnidae	*Galumna*	CHACB140-10	HQ558424
Sarcoptiformes	Galumnidae	*Galumna*	CHACB153-10	HQ558436
Sarcoptiformes	Galumnidae	*Galumna*	CHACB157-10	HQ558440
Sarcoptiformes	Galumnidae	*Galumna*	CHACB158-10	HQ558441

Sarcoptiformes	Galumnidae	*Galumna*	CHACB159-10	HQ558442
Sarcoptiformes	Galumnidae	*Galumna*	CHACB160-10	HQ558443
Sarcoptiformes	Galumnidae	*Galumna*	CHACB161-10	HQ558444
Sarcoptiformes	Galumnidae	*Galumna*	CHACB535-10	HQ558651
Sarcoptiformes	Galumnidae	*Galumna*	CHACB643-10	HQ558723
Sarcoptiformes	Galumnidae	*Galumna*	CHACB644-10	HQ558724
Sarcoptiformes	Galumnidae	*Galumna*	CHACC105-10	JX836869
Sarcoptiformes	Galumnidae	*Galumna*	MYMCA567-11	JX837483
Sarcoptiformes	Galumnidae	*Galumna*	MYMCA568-11	JX835454
Sarcoptiformes	Galumnidae	*Galumna*	MYMCA690-11	JX835503
Sarcoptiformes	Galumnidae	*Galumna*	MYMCA691-11	JX838222
Sarcoptiformes	Galumnidae	*Galumna*	MYMCA692-11	JX837856
Sarcoptiformes	Galumnidae	*Galumna*	MYMCA982-11	JX834432
Sarcoptiformes	Galumnidae	*Galumna*	MYMCC772-11	JX838230
Sarcoptiformes	Galumnidae	*Galumna*	MYMCF838-12	JX835404
Sarcoptiformes	Galumnidae	*Galumna*	SSEIA2087-13	KM829953
Sarcoptiformes	Galumnidae	*Galumna*	SSEIA2088-13	KM828798
Sarcoptiformes	Galumnidae	*Galumna*	SSEIA2093-13	KM840139
Sarcoptiformes	Galumnidae	*Galumna*	SSEIA3028-13	KM831327
Sarcoptiformes	Galumnidae	*Galumna*	SSEIB4542-13	KM827848
Sarcoptiformes	Galumnidae	*Galumna*	SSEIB4546-13	KM839108
Sarcoptiformes	Galumnidae	*Galumna*	SSEIB4551-13	KM840762
Sarcoptiformes	Galumnidae	*Galumna*	SSEIB4555-13	KM832926
Sarcoptiformes	Galumnidae	*Galumna*	SSEIB4577-13	KM835235
Sarcoptiformes	Galumnidae	*Galumna*	SSEIB4579-13	KM832780
Sarcoptiformes	Galumnidae	*Galumna*	SSEIB5405-13	KM834741
Sarcoptiformes	Galumnidae	*Galumna*	SSEIB5419-13	KM829364
Sarcoptiformes	Galumnidae	*Galumna*	SSEIB5797-13	KM830382
Sarcoptiformes	Galumnidae	*Galumna*	SSEIB5801-13	KM840360
Sarcoptiformes	Galumnidae	*Galumna*	SSPAA7997-13	KM831578
Sarcoptiformes	Galumnidae	*Galumna*	SSPAA8001-13	KM835238
Sarcoptiformes	Galumnidae	*Galumna*	SSPAA8019-13	KM834424
Sarcoptiformes	Galumnidae	*Pergalumna*	MIONB055-10	HQ575081
Sarcoptiformes	Galumnidae	*Pergalumna*	MIONB077-10	HQ575091
Sarcoptiformes	Galumnidae	*Pergalumna*	SSEIB5810-13	KM835905
Sarcoptiformes	Galumnidae	*Pergalumna*	SSPAA7806-13	KM831775
Sarcoptiformes	Galumnidae	*Pergalumna*	SSPAA7831-13	KM828796
Sarcoptiformes	Galumnidae	*Pergalumna*	SSPAA7941-13	KM825121
Sarcoptiformes	Galumnidae	*Pergalumna*	SSPAA8012-13	KM827039
Sarcoptiformes	Galumnidae	*Pergalumna nervosa*	GBCH4426-10	GQ864381
Sarcoptiformes	Galumnidae	*Pergalumna sp. AMUENV220*	GBCH4423-10	GQ864384
Sarcoptiformes	Galumnidae	*Pilogalumna*	CHACB259-10	HQ558502
Sarcoptiformes	Galumnidae	*Pilogalumna*	CHACB735-10	HQ558779
Sarcoptiformes	Galumnidae	*Pilogalumna*	CHACB736-10	JX835668
Sarcoptiformes	Galumnidae	*Pilogalumna*	CNRME4710-12	KM829679
Sarcoptiformes	Galumnidae	*Pilogalumna*	CNRMF3211-12	KM834539
Sarcoptiformes	Galumnidae	*Pilogalumna*	MYMCC719-11	JX834251
Sarcoptiformes	Galumnidae	*Pilogalumna*	MYMCE142-12	JX835097
Sarcoptiformes	Galumnidae	*Pilogalumna*	MYMCE547-12	JX834672
Sarcoptiformes	Galumnidae	*Pilogalumna*	MYMCE573-12	JX838149
Sarcoptiformes	Galumnidae	*Pilogalumna*	MYMCE586-12	JX834211
Sarcoptiformes	Galumnidae	*Pilogalumna*	MYMCE872-12	JX836576
Sarcoptiformes	Galumnidae	*Pilogalumna*	MYMCF036-12	JX835039

Sarcoptiformes	Galumnidae	*Pilogalumna*	SSEIB4248-13	KM840076
Sarcoptiformes	Galumnidae	*Pilogalumna*	SSEIB4572-13	KM824605
Sarcoptiformes	Galumnidae	*Pilogalumna*	SSEIB4576-13	KM838965
Sarcoptiformes	Galumnidae	*Pilogalumna*	SSPAA8002-13	KM831318
Sarcoptiformes	Galumnidae		BBLZI152-14	KR069874
Sarcoptiformes	Galumnidae		CNROE179-13	KR070566
Sarcoptiformes	Galumnidae		CNROE187-13	KR070135
Sarcoptiformes	Galumnidae		CNROF646-13	KR069808
Sarcoptiformes	Galumnidae		CNROF647-13	KR069181
Sarcoptiformes	Galumnidae		CNROG270-13	KR069940
Sarcoptiformes	Galumnidae		CNROJ1089-13	KR070634
Sarcoptiformes	Galumnidae		CNROM059-13	KR070616
Sarcoptiformes	Galumnidae		CNROO122-13	KR069722
Sarcoptiformes	Galumnidae		CNROP536-13	KR070207
Sarcoptiformes	Galumnidae		CNROT025-13	KR069770
Sarcoptiformes	Galumnidae		CNROT027-13	KR069879
Sarcoptiformes	Galumnidae		CNROT028-13	KR070327
Sarcoptiformes	Galumnidae		CNROT029-13	KR070066
Sarcoptiformes	Galumnidae		CNROT030-13	KR069587
Sarcoptiformes	Galumnidae		CNROT031-13	KR070128
Sarcoptiformes	Galumnidae		RBINA3937-13	KP979255
Sarcoptiformes	Galumnidae		RBINA3952-13	KR070263
Sarcoptiformes	Galumnidae		RBINA5637-13	KR070324
Sarcoptiformes	Galumnidae		RBINA5638-13	KR069546
Sarcoptiformes	Galumnidae		RBINA5640-13	KR069830
Sarcoptiformes	Galumnidae		RBINA5723-13	KP979318
Sarcoptiformes	Galumnidae		RBINA5725-13	KR069675
Sarcoptiformes	Galumnidae		RBINA5739-13	KP979146
Sarcoptiformes	Galumnidae		RBINA5740-13	KR070161
Sarcoptiformes	Galumnidae		RBINA5772-13	KR069944
Sarcoptiformes	Galumnidae		RBINA5773-13	KP979290
Sarcoptiformes	Galumnidae		RBINA5783-13	KP979351
Sarcoptiformes	Galumnidae		RBINA5784-13	KR069154
Sarcoptiformes	Galumnidae		RBINA5785-13	KR070351
Sarcoptiformes	Galumnidae		SSBAD4724-13	KM830058
Sarcoptiformes	Galumnidae		SSBAD4730-13	KM839721
Sarcoptiformes	Galumnidae		SSBAD4760-13	KM827618
Sarcoptiformes	Galumnidae		SSBAD5559-13	KM834004
Sarcoptiformes	Galumnidae		SSBAD5586-13	KM838464
Sarcoptiformes	Galumnidae		SSBAD5589-13	KM830952
Sarcoptiformes	Galumnidae		SSEIA2092-13	KM829432
Sarcoptiformes	Galumnidae		SSEIA2100-13	KM832608
Sarcoptiformes	Galumnidae		SSEIA3040-13	KM834017
Sarcoptiformes	Galumnidae		SSEIB4541-13	KM832757
Sarcoptiformes	Galumnidae		SSWLA034-13	KM837399
Sarcoptiformes	Galumnidae		SSWLA5256-13	KM840744
Sarcoptiformes	Galumnidae		SSWLA5258-13	KM824953
Sarcoptiformes	Galumnidae		SSWLA5272-13	KM825576
Sarcoptiformes	Galumnidae		SSWLA5273-13	KM833219
Sarcoptiformes	Galumnidae		SSWLA5282-13	KM839954
Sarcoptiformes	Galumnidae		SSWLA5285-13	KM838910
Sarcoptiformes	Galumnidae		SSWLA5286-13	KM839582
Sarcoptiformes	Galumnidae		SSWLA5293-13	KM826749

Sarcoptiformes	Galumnidae		SSWLA5294-13	KM827829
Sarcoptiformes	Galumnidae		SSWLA5298-13	KM835975
Sarcoptiformes	Galumnidae		SSWLA5320-13	KM840846
Sarcoptiformes	Galumnidae		SSWLA5324-13	KM840239
Sarcoptiformes	Galumnidae		SSWLA5326-13	KM833358
Sarcoptiformes	Galumnidae		SSWLA5332-13	KM835194
Sarcoptiformes	Galumnidae		SSWLD8129-13	KM834839
Sarcoptiformes	Galumnidae		SSWLD8133-13	KM824121
Sarcoptiformes	Galumnidae		SSWLD8134-13	KM839844
Sarcoptiformes	Galumnidae		SSWLD8137-13	KM830795
Sarcoptiformes	Galumnidae		SSWLD8139-13	KM832566
Sarcoptiformes	Galumnidae		SSWLD8146-13	KM834015
Sarcoptiformes	Galumnidae		SSWLD8150-13	KM838667
Sarcoptiformes	Galumnidae		SSWLD8151-13	KM824602
Sarcoptiformes	Galumnidae		SSWLD8155-13	KM824285
Sarcoptiformes	Galumnidae		SSWLD8157-13	KM840819
Sarcoptiformes	Galumnidae		SSWLD8190-13	KM837318
Sarcoptiformes	Galumnidae		SSWLD8192-13	KM828360
Sarcoptiformes	Galumnidae		SSWLD8199-13	KM830320
Sarcoptiformes	Galumnidae		SSWLD8206-13	KM840213
Sarcoptiformes	Galumnidae		SSWLD8220-13	KM832988
Sarcoptiformes	Galumnidae		SSWLD8221-13	KM826153
Sarcoptiformes	Galumnidae		SSWLD8222-13	KM826381
Sarcoptiformes	Galumnidae		SSWLD8224-13	KM838551
Sarcoptiformes	Galumnidae		SSWLD8229-13	KM838588
Sarcoptiformes	Galumnidae		SSWLD8231-13	KM830697
Sarcoptiformes	Galumnidae		SSWLD8233-13	KM828729
Sarcoptiformes	Galumnidae		SSWLD8238-13	KM829583
Sarcoptiformes	Galumnidae		SSWLD8244-13	KM833073
Sarcoptiformes	Galumnidae		SSWLD8248-13	KM827897
Sarcoptiformes	Galumnidae		SSWLD8256-13	KM834949
Sarcoptiformes	Galumnidae		SSWLD8265-13	KM831521
Sarcoptiformes	Galumnidae		SSWLD8267-13	KM836810
Sarcoptiformes	Galumnidae		SSWLD8274-13	KM829312
Sarcoptiformes	Galumnidae		SSWLD8282-13	KM837983
Sarcoptiformes	Galumnidae		SSWLD8286-13	KM824946
Sarcoptiformes	Galumnidae		SSWLD8300-13	KM838726
Sarcoptiformes	Galumnidae		SSWLD8305-13	KM837099
Sarcoptiformes	Galumnidae		SSWLD8311-13	KM832657
Sarcoptiformes	Galumnidae		SSWLD8319-13	KM828182
Sarcoptiformes	Galumnidae		SSWLD8334-13	KM833446
Sarcoptiformes	Galumnidae		SSWLD8337-13	KM832007
Sarcoptiformes	Galumnidae		SSWLD8340-13	KM827959
Sarcoptiformes	Galumnidae		SSWLD8341-13	KM824583
Sarcoptiformes	Galumnidae		SSWLD8343-13	KM840711
Sarcoptiformes	Galumnidae		SSWLD8346-13	KM835729
Sarcoptiformes	Galumnidae		SSWLD8350-13	KM824554
Sarcoptiformes	Galumnidae		SSWLD8351-13	KM836250
Sarcoptiformes	Galumnidae		SSWLD8372-13	KM835934
Sarcoptiformes	Galumnidae		SSWLD8376-13	KM830636
Sarcoptiformes	Gustaviidae	*Gustavia*	SSEIA2435-13	KM840290
Sarcoptiformes	Gustaviidae	*Gustavia*	SSEIB8180-13	KM833347
Sarcoptiformes	Gustaviidae	*Gustavia*	SSEIB8204-13	KM838909

Sarcoptiformes	Gustaviidae	*Gustavia microcephala*	SSROA3667-14	KR070670
Sarcoptiformes	Gustaviidae	*Gustavia microcephala*	SSROA3739-14	KR070430
Sarcoptiformes	Gustaviidae	*Gustavia microcephala*	SSROA3774-14	KR070147
Sarcoptiformes	Gustaviidae	*Gustavia microcephala*	RBINA3914-13	KP979329
Sarcoptiformes	Gustaviidae	*Gustavia microcephala*	RBINA5694-13	KR070603
Sarcoptiformes	Gymnodamaeidae	*Gymnodamaeus*	CNGLD021-13	KM827296
Sarcoptiformes	Gymnodamaeidae	*Gymnodamaeus*	CNGLD028-13	KM837488
Sarcoptiformes	Gymnodamaeidae	*Gymnodamaeus*	CNGLD059-13	KM824234
Sarcoptiformes	Gymnodamaeidae	*Gymnodamaeus*	CNRMD2678-12	KM838755
Sarcoptiformes	Gymnodamaeidae	*Gymnodamaeus*	SSBAB1208-12	KM838893
Sarcoptiformes	Gymnodamaeidae	*Gymnodamaeus*	SSBAE3790-13	KM828401
Sarcoptiformes	Gymnodamaeidae	*Gymnodamaeus*	SSJAB1882-13	KM831192
Sarcoptiformes	Gymnodamaeidae	*Gymnodamaeus*	SSJAC988-13	KM839431
Sarcoptiformes	Gymnodamaeidae	*Gymnodamaeus*	SSPAA7961-13	KM836231
Sarcoptiformes	Gymnodamaeidae	*Gymnodamaeus*	SSPAA8145-13	KM832442
Sarcoptiformes	Gymnodamaeidae	*Gymnodamaeus*	SSWLC063-13	KM826102
Sarcoptiformes	Gymnodamaeidae	*Gymnodamaeus*	SSWLE2970-13	KM838958
Sarcoptiformes	Gymnodamaeidae	*Gymnodamaeus ornatus*	SSEIB4559-13	KM840834
Sarcoptiformes	Gymnodamaeidae	*Gymnodamaeus ornatus*	SSJAC1547-13	KM828181
Sarcoptiformes	Gymnodamaeidae	*Gymnodamaeus ornatus*	SSJAC966-13	KM837554
Sarcoptiformes	Gymnodamaeidae	*Gymnodamaeus ornatus*	SSJAC992-13	KM829083
Sarcoptiformes	Gymnodamaeidae	*Gymnodamaeus ornatus*	SSPAA8004-13	KM827830
Sarcoptiformes	Gymnodamaeidae	*Gymnodamaeus ornatus*	SSPAA8006-13	KM838309
Sarcoptiformes	Gymnodamaeidae	*Gymnodamaeus ornatus*	SSPAA8007-13	KM827188
Sarcoptiformes	Gymnodamaeidae	*Gymnodamaeus ornatus*	SSPAA8008-13	KM840276
Sarcoptiformes	Gymnodamaeidae	*Gymnodamaeus ornatus*	SSPAA8010-13	KM825183
Sarcoptiformes	Gymnodamaeidae	*Gymnodamaeus ornatus*	SSPAA8017-13	KM826193
Sarcoptiformes	Haplozetidae	*Peloribates canadensis*	CHACA120-08	JX838145
Sarcoptiformes	Haplozetidae	*Peloribates canadensis*	CHACA712-09	JX837501
Sarcoptiformes	Haplozetidae	*Peloribates canadensis*	CHACA715-09	JX837581
Sarcoptiformes	Haplozetidae	*Peloribates canadensis*	CHACA720-09	KR069299
Sarcoptiformes	Haplozetidae	*Peloribates canadensis*	CHACA722-09	JX837584
Sarcoptiformes	Haplozetidae	*Peloribates canadensis*	CHACA845-09	GU702809
Sarcoptiformes	Haplozetidae	*Peloribates canadensis*	MYMCA1048-11	JX835023
Sarcoptiformes	Haplozetidae	*Peloribates canadensis*	MYMCA1129-11	JX835708
Sarcoptiformes	Haplozetidae	*Peloribates canadensis*	MYMCA192-11	JX836579
Sarcoptiformes	Haplozetidae	*Peloribates canadensis*	MYMCA193-11	JX834975
Sarcoptiformes	Haplozetidae	*Peloribates canadensis*	MYMCA214-11	JX838159
Sarcoptiformes	Haplozetidae	*Peloribates canadensis*	MYMCA297-11	JX838431
Sarcoptiformes	Haplozetidae	*Peloribates canadensis*	MYMCA308-11	JX835172
Sarcoptiformes	Haplozetidae	*Peloribates canadensis*	MYMCA409-11	JX837416
Sarcoptiformes	Haplozetidae	*Peloribates canadensis*	MYMCA410-11	JX838766
Sarcoptiformes	Haplozetidae	*Peloribates canadensis*	MYMCA411-11	JX835783
Sarcoptiformes	Haplozetidae	*Peloribates canadensis*	MYMCA412-11	JX836608
Sarcoptiformes	Haplozetidae	*Peloribates canadensis*	MYMCA413-11	JX834502
Sarcoptiformes	Haplozetidae	*Peloribates canadensis*	MYMCA444-11	JX834362
Sarcoptiformes	Haplozetidae	*Peloribates canadensis*	MYMCA476-11	JX834309
Sarcoptiformes	Haplozetidae	*Peloribates canadensis*	MYMCA798-11	JX833643
Sarcoptiformes	Haplozetidae	*Peloribates canadensis*	MYMCA912-11	JX836200
Sarcoptiformes	Haplozetidae	*Peloribates canadensis*	MYMCB113-11	JX833887
Sarcoptiformes	Haplozetidae	*Peloribates canadensis*	MYMCB387-11	JX838020
Sarcoptiformes	Haplozetidae	*Peloribates canadensis*	MYMCB388-11	JX836818
Sarcoptiformes	Haplozetidae	*Peloribates canadensis*	MYMCB540-11	JX836871

Sarcoptiformes	Haplozetidae	*Peloribates canadensis*	MYMCB597-11	JX837961
Sarcoptiformes	Haplozetidae	*Peloribates canadensis*	MYMCC054-11	JX837950
Sarcoptiformes	Haplozetidae	*Peloribates canadensis*	MYMCC055-11	JX838079
Sarcoptiformes	Haplozetidae	*Peloribates canadensis*	MYMCC088-11	JX834537
Sarcoptiformes	Haplozetidae	*Peloribates canadensis*	MYMCC192-11	JX838632
Sarcoptiformes	Haplozetidae	*Peloribates canadensis*	MYMCC219-11	JX837422
Sarcoptiformes	Haplozetidae	*Peloribates canadensis*	MYMCC220-11	JX838703
Sarcoptiformes	Haplozetidae	*Peloribates canadensis*	MYMCC464-11	JX836357
Sarcoptiformes	Haplozetidae	*Peloribates canadensis*	MYMCC470-11	JX837682
Sarcoptiformes	Haplozetidae	*Peloribates canadensis*	MYMCC491-11	JX838330
Sarcoptiformes	Haplozetidae	*Peloribates canadensis*	MYMCC685-11	JX834516
Sarcoptiformes	Haplozetidae	*Peloribates canadensis*	MYMCC720-11	JX833742
Sarcoptiformes	Haplozetidae	*Peloribates canadensis*	MYMCD001-11	JX834601
Sarcoptiformes	Haplozetidae	*Peloribates canadensis*	MYMCD002-11	JX837479
Sarcoptiformes	Haplozetidae	*Peloribates canadensis*	MYMCE471-12	JX835448
Sarcoptiformes	Haplozetidae	*Peloribates canadensis*	MYMCE653-12	JX836975
Sarcoptiformes	Haplozetidae	*Peloribates canadensis*	MYMCE874-12	JX835951
Sarcoptiformes	Haplozetidae	*Peloribates canadensis*	MYMCE921-12	JX834767
Sarcoptiformes	Haplozetidae	*Peloribates canadensis*	MYMCF038-12	JX838683
Sarcoptiformes	Haplozetidae	*Peloribates canadensis*	MYMCF378-12	JX835146
Sarcoptiformes	Haplozetidae	*Peloribates canadensis*	MYMCF479-12	JX835963
Sarcoptiformes	Haplozetidae	*Peloribates canadensis*	MYMCF639-12	JX838628
Sarcoptiformes	Haplozetidae	*Peloribates canadensis*	MYMCF741-12	JX836903
Sarcoptiformes	Haplozetidae	*Peloribates canadensis*	MYMCG008-12	JX836274
Sarcoptiformes	Haplozetidae	*Peloribates canadensis*	MYMCG009-12	JX834079
Sarcoptiformes	Haplozetidae	*Peloribates canadensis*	MYMCG622-12	JX836723
Sarcoptiformes	Haplozetidae	*Peloribates canadensis*	MYTMC007-09	GU680488
Sarcoptiformes	Haplozetidae	*Peloribates canadensis*	MYTMC134-09	GU680451
Sarcoptiformes	Haplozetidae	*Peloribates canadensis*	MYTMC155-09	GU680449
Sarcoptiformes	Haplozetidae	*Peloribates canadensis*	MYTMC157-09	KR070706
Sarcoptiformes	Haplozetidae	*Peloribates canadensis*	SSPAA7804-13	KM833544
Sarcoptiformes	Haplozetidae	*Peloribates canadensis*	SSPAA7807-13	KM836895
Sarcoptiformes	Haplozetidae	*Peloribates canadensis*	SSPAA7954-13	KM824659
Sarcoptiformes	Haplozetidae	*Peloribates pilosus*	CHACA046-08	JX837882
Sarcoptiformes	Haplozetidae	*Peloribates pilosus*	CHACA1181-10	JX837629
Sarcoptiformes	Haplozetidae	*Peloribates pilosus*	CHACA271-08	JX835559
Sarcoptiformes	Haplozetidae	*Peloribates pilosus*	CHACA272-08	JX836523
Sarcoptiformes	Haplozetidae	*Peloribates pilosus*	CHACA273-08	JX836223
Sarcoptiformes	Haplozetidae	*Peloribates pilosus*	CHACA274-08	JX837270
Sarcoptiformes	Haplozetidae	*Peloribates pilosus*	CHACA340-08	JX836260
Sarcoptiformes	Haplozetidae	*Peloribates pilosus*	CHACA341-08	JX836400
Sarcoptiformes	Haplozetidae	*Peloribates pilosus*	CHACA603-09	JX835958
Sarcoptiformes	Haplozetidae	*Peloribates pilosus*	CHACA625-09	JX838027
Sarcoptiformes	Haplozetidae	*Peloribates pilosus*	CHACA966-10	HM405832
Sarcoptiformes	Haplozetidae	*Peloribates pilosus*	CHACB014-10	HQ558335
Sarcoptiformes	Haplozetidae	*Peloribates pilosus*	CHACB1007-10	HM907143
Sarcoptiformes	Haplozetidae	*Peloribates pilosus*	CHACB1008-10	HM907144
Sarcoptiformes	Haplozetidae	*Peloribates pilosus*	CHACB1010-10	HM907146
Sarcoptiformes	Haplozetidae	*Peloribates pilosus*	CHACB1011-10	HM907147
Sarcoptiformes	Haplozetidae	*Peloribates pilosus*	CHACB1012-10	HM907148
Sarcoptiformes	Haplozetidae	*Peloribates pilosus*	CHACB1013-10	HM907149
Sarcoptiformes	Haplozetidae	*Peloribates pilosus*	CHACB1014-10	HM907150
Sarcoptiformes	Haplozetidae	*Peloribates pilosus*	CHACB148-10	HQ558431

Sarcoptiformes	Haplozetidae	*Peloribates pilosus*	CHACB149-10	HQ558432
Sarcoptiformes	Haplozetidae	*Peloribates pilosus*	CHACB150-10	HQ558433
Sarcoptiformes	Haplozetidae	*Peloribates pilosus*	CHACB263-10	HQ558505
Sarcoptiformes	Haplozetidae	*Peloribates pilosus*	CHACB309-10	HM907300
Sarcoptiformes	Haplozetidae	*Peloribates pilosus*	CHACB418-10	HQ558575
Sarcoptiformes	Haplozetidae	*Peloribates pilosus*	CHACB419-10	HQ558576
Sarcoptiformes	Haplozetidae	*Peloribates pilosus*	CHACB420-10	HQ558577
Sarcoptiformes	Haplozetidae	*Peloribates pilosus*	CHACB421-10	HQ558578
Sarcoptiformes	Haplozetidae	*Peloribates pilosus*	CHACB713-10	HQ558764
Sarcoptiformes	Haplozetidae	*Peloribates pilosus*	CHACC022-10	HM907423
Sarcoptiformes	Haplozetidae	*Peloribates pilosus*	CHACC023-10	HM907424
Sarcoptiformes	Haplozetidae	*Peloribates pilosus*	CHACC024-10	HM907425
Sarcoptiformes	Haplozetidae	*Peloribates pilosus*	CHACC025-10	HM907426
Sarcoptiformes	Haplozetidae	*Peloribates pilosus*	CHACC026-10	HM907427
Sarcoptiformes	Haplozetidae	*Peloribates pilosus*	CHACC044-10	HM907444
Sarcoptiformes	Haplozetidae	*Peloribates pilosus*	CHACC045-10	HM907445
Sarcoptiformes	Haplozetidae	*Peloribates pilosus*	CHACC046-10	HM907446
Sarcoptiformes	Haplozetidae	*Peloribates pilosus*	CHACC047-10	HM907447
Sarcoptiformes	Haplozetidae	*Peloribates pilosus*	CHACC048-10	HM907448
Sarcoptiformes	Haplozetidae	*Peloribates pilosus*	CHACC092-10	HM907481
Sarcoptiformes	Haplozetidae	*Peloribates pilosus*	CHACC135-10	JX835544
Sarcoptiformes	Haplozetidae	*Peloribates pilosus*	CHACC136-10	JX834064
Sarcoptiformes	Haplozetidae	*Peloribates pilosus*	CHACC137-10	JX835758
Sarcoptiformes	Haplozetidae	*Peloribates pilosus*	CHACC138-10	JX834752
Sarcoptiformes	Haplozetidae	*Peloribates pilosus*	CHACC139-10	JX838521
Sarcoptiformes	Haplozetidae	*Peloribates pilosus*	CHACC140-10	JX837258
Sarcoptiformes	Haplozetidae	*Peloribates pilosus*	CHACC141-10	JX836924
Sarcoptiformes	Haplozetidae	*Peloribates pilosus*	CHACC187-10	JX834255
Sarcoptiformes	Haplozetidae	*Peloribates pilosus*	CHACC188-10	JX838361
Sarcoptiformes	Haplozetidae	*Peloribates pilosus*	CHACC189-10	JX836815
Sarcoptiformes	Haplozetidae	*Peloribates pilosus*	CHACC197-10	HQ941523
Sarcoptiformes	Haplozetidae	*Peloribates pilosus*	CHACC198-10	HQ941524
Sarcoptiformes	Haplozetidae	*Peloribates pilosus*	CHACC199-10	HQ941525
Sarcoptiformes	Haplozetidae	*Peloribates pilosus*	CHACC235-10	HQ941543
Sarcoptiformes	Haplozetidae	*Peloribates pilosus*	MYMCA1471-11	JX836177
Sarcoptiformes	Haplozetidae	*Peloribates pilosus*	MYMCA1472-11	JX834508
Sarcoptiformes	Haplozetidae	*Peloribates pilosus*	MYMCA1515-11	JX833975
Sarcoptiformes	Haplozetidae	*Peloribates pilosus*	MYMCA1516-11	JX837520
Sarcoptiformes	Haplozetidae	*Peloribates pilosus*	MYMCA164-11	JX836800
Sarcoptiformes	Haplozetidae	*Peloribates pilosus*	MYMCA166-11	JX836382
Sarcoptiformes	Haplozetidae	*Peloribates pilosus*	MYMCA167-11	JX834386
Sarcoptiformes	Haplozetidae	*Peloribates pilosus*	MYMCA168-11	JX836546
Sarcoptiformes	Haplozetidae	*Peloribates pilosus*	MYMCA591-11	JX837051
Sarcoptiformes	Haplozetidae	*Peloribates pilosus*	MYMCA606-11	JX837758
Sarcoptiformes	Haplozetidae	*Peloribates pilosus*	MYMCA607-11	JX837231
Sarcoptiformes	Haplozetidae	*Peloribates pilosus*	MYMCA608-11	JX834289
Sarcoptiformes	Haplozetidae	*Peloribates pilosus*	MYMCA688-11	JX835382
Sarcoptiformes	Haplozetidae	*Peloribates pilosus*	MYMCA689-11	JX835162
Sarcoptiformes	Haplozetidae	*Peloribates pilosus*	MYMCA729-11	JX833747
Sarcoptiformes	Haplozetidae	*Peloribates pilosus*	MYMCA991-11	JX837313
Sarcoptiformes	Haplozetidae	*Peloribates pilosus*	MYMCA992-11	JX837485
Sarcoptiformes	Haplozetidae	*Peloribates pilosus*	MYMCC305-11	JX837852
Sarcoptiformes	Haplozetidae	*Peloribates pilosus*	MYMCE016-12	JX835418

Sarcoptiformes	Haplozetidae	*Peloribates pilosus*	MYMCE017-12	JX836835
Sarcoptiformes	Haplozetidae	*Peloribates pilosus*	MYMCE048-12	JX835115
Sarcoptiformes	Haplozetidae	*Peloribates pilosus*	MYMCE077-12	JX837733
Sarcoptiformes	Haplozetidae	*Peloribates pilosus*	MYMCE173-12	JX836174
Sarcoptiformes	Haplozetidae	*Peloribates pilosus*	MYMCE426-12	JX835697
Sarcoptiformes	Haplozetidae	*Peloribates pilosus*	MYMCE557-12	JX835191
Sarcoptiformes	Haplozetidae	*Peloribates pilosus*	MYMCF090-12	JX838584
Sarcoptiformes	Haplozetidae	*Peloribates pilosus*	MYMCF240-12	JX835716
Sarcoptiformes	Haplozetidae	*Peloribates pilosus*	MYMCF301-12	JX835744
Sarcoptiformes	Haplozetidae	*Peloribates pilosus*	MYMCF518-12	JX837526
Sarcoptiformes	Haplozetidae	*Peloribates pilosus*	MYMCF519-12	JX837849
Sarcoptiformes	Haplozetidae	*Peloribates pilosus*	MYMCF692-12	JX837687
Sarcoptiformes	Haplozetidae	*Peloribates pilosus*	MYMCG200-12	JX834563
Sarcoptiformes	Haplozetidae	*Peloribates pilosus*	MYMCG224-12	JX835245
Sarcoptiformes	Haplozetidae	*Peloribates pilosus*	MYMCG275-12	JX835666
Sarcoptiformes	Haplozetidae	*Peloribates pilosus*	MYMCG276-12	JX837861
Sarcoptiformes	Haplozetidae	*Peloribates pilosus*	MYMCG277-12	JX837287
Sarcoptiformes	Haplozetidae	*Peloribates pilosus*	MYMCG462-12	JX835079
Sarcoptiformes	Haplozetidae	*Rostrozetes*	MIONB139-10	KP979348
Sarcoptiformes	Haplozetidae	*Rostrozetes*	MIONB201-10	KR069593
Sarcoptiformes	Haplozetidae		CNPAF907-13	KM837599
Sarcoptiformes	Haplozetidae		CNPAH425-13	KM836770
Sarcoptiformes	Haplozetidae		CNRMC1514-12	KM828913
Sarcoptiformes	Haplozetidae		CNRMC1520-12	KM827456
Sarcoptiformes	Haplozetidae		CNRMC1522-12	KM826890
Sarcoptiformes	Haplozetidae		CNRMC1650-12	KM827096
Sarcoptiformes	Haplozetidae		CNRMC1657-12	KM832100
Sarcoptiformes	Haplozetidae		CNRMC1681-12	KM826025
Sarcoptiformes	Haplozetidae		CNRMD2708-12	KM832589
Sarcoptiformes	Haplozetidae		CNRME4756-12	KM840279
Sarcoptiformes	Haplozetidae		MIONB048-10	HM887582
Sarcoptiformes	Haplozetidae		SSEIB5420-13	KM829275
Sarcoptiformes	Hermanniellidae	*Hermanniella robusta*	SSEIB8188-13	KM838734
Sarcoptiformes	Hermanniidae	*Hermannia reticulata*	CHACA099-08	JX835606
Sarcoptiformes	Hermanniidae	*Hermannia reticulata*	CHACA100-08	JX835857
Sarcoptiformes	Hermanniidae	*Hermannia reticulata*	CHACA101-08	JX835521
Sarcoptiformes	Hermanniidae	*Hermannia reticulata*	CHACA124-08	JX837316
Sarcoptiformes	Hermanniidae	*Hermannia reticulata*	CHACA140-08	JX838190
Sarcoptiformes	Hermanniidae	*Hermannia reticulata*	CHACA142-08	JX836914
Sarcoptiformes	Hermanniidae	*Hermannia reticulata*	CHACA145-08	JX836500
Sarcoptiformes	Hermanniidae	*Hermannia reticulata*	CHACA146-08	JX837262
Sarcoptiformes	Hermanniidae	*Hermannia reticulata*	CHACA147-08	JX837848
Sarcoptiformes	Hermanniidae	*Hermannia reticulata*	CHACA160-08	JX834390
Sarcoptiformes	Hermanniidae	*Hermannia reticulata*	CHACA465-09	JX838377
Sarcoptiformes	Hermanniidae	*Hermannia reticulata*	CHACA466-09	JX835884
Sarcoptiformes	Hermanniidae	*Hermannia reticulata*	CHACA467-09	JX833847
Sarcoptiformes	Hermanniidae	*Hermannia reticulata*	CHACB001-10	HQ558324
Sarcoptiformes	Hermanniidae	*Hermannia reticulata*	CHACB004-10	HQ558327
Sarcoptiformes	Hermanniidae	*Hermannia reticulata*	CHACB005-10	HQ558328
Sarcoptiformes	Hermanniidae	*Hermannia reticulata*	CHACB006-10	HQ558329
Sarcoptiformes	Hermanniidae	*Hermannia reticulata*	CHACB007-10	HQ558330
Sarcoptiformes	Hermanniidae	*Hermannia reticulata*	CHACB009-10	HQ558332
Sarcoptiformes	Hermanniidae	*Hermannia reticulata*	CHACB010-10	HQ558333

Sarcoptiformes	Hermanniidae	*Hermannia reticulata*	CHACB098-10	HQ558394
Sarcoptiformes	Hermanniidae	*Hermannia reticulata*	CHACB101-10	HQ558397
Sarcoptiformes	Hermanniidae	*Hermannia reticulata*	CHACB1193-10	HM907268
Sarcoptiformes	Hermanniidae	*Hermannia reticulata*	CHACB548-10	HQ558664
Sarcoptiformes	Hermanniidae	*Hermannia reticulata*	CHACB549-10	HQ558665
Sarcoptiformes	Hermanniidae	*Hermannia reticulata*	CHACB552-10	HQ558668
Sarcoptiformes	Hermanniidae	*Hermannia reticulata*	CHACB553-10	HQ558669
Sarcoptiformes	Hermanniidae	*Hermannia reticulata*	CHACB559-10	JX837978
Sarcoptiformes	Hermanniidae	*Hermannia reticulata*	CHACB560-10	HQ558674
Sarcoptiformes	Hermanniidae	*Hermannia reticulata*	CHACB561-10	HQ558675
Sarcoptiformes	Hermanniidae	*Hermannia reticulata*	CHACB562-10	HQ558676
Sarcoptiformes	Hermanniidae	*Hermannia reticulata*	CHACB563-10	HQ558677
Sarcoptiformes	Hermanniidae	*Hermannia reticulata*	CHACB864-10	HM907313
Sarcoptiformes	Hermanniidae	*Hermannia reticulata*	CHACB865-10	HM907314
Sarcoptiformes	Hermanniidae	*Hermannia reticulata*	CHACB866-10	HM907315
Sarcoptiformes	Hermanniidae	*Hermannia reticulata*	CHACB867-10	HM907316
Sarcoptiformes	Hermanniidae	*Hermannia reticulata*	CHACB868-10	HM907317
Sarcoptiformes	Hermanniidae	*Hermannia reticulata*	CHACB876-10	HM907321
Sarcoptiformes	Hermanniidae	*Hermannia reticulata*	CHACB877-10	HM907322
Sarcoptiformes	Hermanniidae	*Hermannia reticulata*	CHACB878-10	HM907323
Sarcoptiformes	Hermanniidae	*Hermannia reticulata*	CHACB879-10	HM907324
Sarcoptiformes	Hermanniidae	*Hermannia reticulata*	CHACC017-10	HM907418
Sarcoptiformes	Hermanniidae	*Hermannia reticulata*	CHACC028-10	HM907429
Sarcoptiformes	Hermanniidae	*Hermannia reticulata*	CHACC029-10	HM907430
Sarcoptiformes	Hermanniidae	*Hermannia reticulata*	CHACC030-10	HM907431
Sarcoptiformes	Hermanniidae	*Hermannia reticulata*	CHACC031-10	HM907432
Sarcoptiformes	Hermanniidae	*Hermannia reticulata*	CHACC032-10	HM907433
Sarcoptiformes	Hermanniidae	*Hermannia reticulata*	CHACC124-10	JX833864
Sarcoptiformes	Hermanniidae	*Hermannia reticulata*	CHACC125-10	JX838720
Sarcoptiformes	Hermanniidae	*Hermannia reticulata*	CHACC131-10	JX834809
Sarcoptiformes	Hermanniidae	*Hermannia reticulata*	CHACC132-10	JX834970
Sarcoptiformes	Hermanniidae	*Hermannia reticulata*	CHACC133-10	JX835583
Sarcoptiformes	Hermanniidae	*Hermannia reticulata*	CHACC134-10	JX835510
Sarcoptiformes	Hermanniidae	*Hermannia reticulata*	CHACC242-10	HQ941549
Sarcoptiformes	Hermanniidae	*Hermannia reticulata*	CHACC243-10	HQ941550
Sarcoptiformes	Hermanniidae	*Hermannia reticulata*	MYMCA084-11	JX835867
Sarcoptiformes	Hermanniidae	*Hermannia reticulata*	MYMCA085-11	JX837337
Sarcoptiformes	Hermanniidae	*Hermannia reticulata*	MYMCA086-11	JX833651
Sarcoptiformes	Hermanniidae	*Hermannia reticulata*	MYMCA087-11	JX835164
Sarcoptiformes	Hermanniidae	*Hermannia reticulata*	MYMCA145-11	JX833954
Sarcoptiformes	Hermanniidae	*Hermannia reticulata*	MYMCA1467-11	JX836183
Sarcoptiformes	Hermanniidae	*Hermannia reticulata*	MYMCA1468-11	JX838704
Sarcoptiformes	Hermanniidae	*Hermannia reticulata*	MYMCA250-11	JX836211
Sarcoptiformes	Hermanniidae	*Hermannia reticulata*	MYMCA480-11	JX837297
Sarcoptiformes	Hermanniidae	*Hermannia reticulata*	MYMCA493-11	JX835440
Sarcoptiformes	Hermanniidae	*Hermannia reticulata*	MYMCA660-11	JX835114
Sarcoptiformes	Hermanniidae	*Hermannia reticulata*	MYMCB171-11	JX833833
Sarcoptiformes	Hermanniidae	*Hermannia reticulata*	MYMCB277-11	JX836675
Sarcoptiformes	Hermanniidae	*Hermannia reticulata*	MYMCB423-11	JX835008
Sarcoptiformes	Hermanniidae	*Hermannia reticulata*	MYMCB804-11	JX838338
Sarcoptiformes	Hermanniidae	*Hermannia reticulata*	MYMCB805-11	JX833832
Sarcoptiformes	Hermanniidae	*Hermannia reticulata*	MYMCC018-11	JX835444
Sarcoptiformes	Hermanniidae	*Hermannia reticulata*	MYMCC294-11	JX837534

Sarcoptiformes	Hermanniidae	*Hermannia reticulata*	MYMCC358-11	JX836000
Sarcoptiformes	Hermanniidae	*Hermannia reticulata*	MYMCC394-11	JX837336
Sarcoptiformes	Hermanniidae	*Hermannia reticulata*	MYMCD153-11	JX834727
Sarcoptiformes	Hermanniidae	*Hermannia reticulata*	MYMCD176-11	JX834407
Sarcoptiformes	Hermanniidae	*Hermannia reticulata*	MYMCE079-12	JX837321
Sarcoptiformes	Hermanniidae	*Hermannia reticulata*	MYMCE246-12	JX837156
Sarcoptiformes	Hermanniidae	*Hermannia reticulata*	MYMCE252-12	JX837839
Sarcoptiformes	Hermanniidae	*Hermannia reticulata*	MYMCF415-12	JX838513
Sarcoptiformes	Hermanniidae	*Hermannia reticulata*	MYMCF538-12	JX835817
Sarcoptiformes	Hermanniidae	*Hermannia reticulata*	MYMCF661-12	JX834404
Sarcoptiformes	Hermanniidae	*Hermannia reticulata*	MYMCF677-12	JX833882
Sarcoptiformes	Hermanniidae	*Hermannia reticulata*	MYMCF678-12	JX833721
Sarcoptiformes	Hermanniidae	*Hermannia reticulata*	MYMCF803-12	JX836785
Sarcoptiformes	Hermanniidae	*Hermannia reticulata*	MYMCF892-12	JX834012
Sarcoptiformes	Hermanniidae	*Hermannia reticulata*	MYMCG356-12	KR070655
Sarcoptiformes	Hermanniidae	*Hermannia reticulata*	MYMCG584-12	JX837753
Sarcoptiformes	Hermanniidae	*Hermannia reticulata*	MYTMC177-09	GU680442
Sarcoptiformes	Histiostomatidae	*Bonomoia sp. AMUBon02*	GBCH4459-10	GQ864343
Sarcoptiformes	Histiostomatidae	*Histiostoma ovalis*	GBA14668-14	KJ533743
Sarcoptiformes	Histiostomatidae	*Histiostoma ovalis*	GBA14669-14	KJ533744
Sarcoptiformes	Histiostomatidae	*Histiostoma ovalis*	GBA14671-14	KJ533746
Sarcoptiformes	Hydrozetidae	*Hydrozetes*	MYMCE258-12	JX836071
Sarcoptiformes	Hydrozetidae	*Hydrozetes*	MYMCE259-12	JX835835
Sarcoptiformes	Hydrozetidae	*Hydrozetes*	MYMCE260-12	JX835371
Sarcoptiformes	Hydrozetidae	*Hydrozetes*	MYMCE365-12	JX837779
Sarcoptiformes	Hydrozetidae	*Hydrozetes*	MYMCG108-12	JX837497
Sarcoptiformes	Hypochthoniidae	*Hypochthonius rufulus*	MITMH008-07	KR070150
Sarcoptiformes	Hypochthoniidae	*Hypochthonius rufulus*	MYMCG087-12	JX836301
Sarcoptiformes	Knemidokoptidae	*Knemidokoptes jamaicensis*	GBCH11625-13	JQ037816
Sarcoptiformes	Knemidokoptidae	*Knemidokoptes sp.*	GBA17579-14	KJ787640
Sarcoptiformes	Liacaridae	*Dorycranosus*	SSWLB2395-13	KM832484
Sarcoptiformes	Liacaridae	*Dorycranosus acutidens*	CHACA1098-10	JX834657
Sarcoptiformes	Liacaridae	*Dorycranosus acutidens*	CHACA807-09	JX834243
Sarcoptiformes	Liacaridae	*Dorycranosus acutidens*	CHACB183-10	HQ558455
Sarcoptiformes	Liacaridae	*Dorycranosus acutidens*	CHACB299-10	JX837708
Sarcoptiformes	Liacaridae	*Dorycranosus acutidens*	CHACB982-10	JX837873
Sarcoptiformes	Liacaridae	*Dorycranosus acutidens*	CHACB983-10	HM907390
Sarcoptiformes	Liacaridae	*Dorycranosus acutidens*	MYMCA006-11	JX836698
Sarcoptiformes	Liacaridae	*Dorycranosus acutidens*	MYMCB621-11	JX838652
Sarcoptiformes	Liacaridae	*Dorycranosus acutidens*	MYMCB622-11	JX834230
Sarcoptiformes	Liacaridae	*Dorycranosus acutidens*	MYMCC549-11	JX835173
Sarcoptiformes	Liacaridae	*Dorycranosus acutidens*	MYMCC566-11	JX836749
Sarcoptiformes	Liacaridae	*Dorycranosus acutidens*	MYMCC631-11	JX837516
Sarcoptiformes	Liacaridae	*Dorycranosus acutidens*	MYMCC632-11	JX835217
Sarcoptiformes	Liacaridae	*Dorycranosus acutidens*	SSPAB8767-13	KM825796
Sarcoptiformes	Liacaridae	*Dorycranosus acutidens*	SSPAB9002-13	KM837224
Sarcoptiformes	Liacaridae		CHACC249-10	HQ941553
Sarcoptiformes	Liacaridae		CHACC250-10	HQ941554
Sarcoptiformes	Liacaridae		CHACC251-10	HQ941555
Sarcoptiformes	Liacaridae		CHACC252-10	HQ941556
Sarcoptiformes	Liacaridae		CHACC253-10	HQ941557
Sarcoptiformes	Liacaridae		CNSLD654-12	KM830359
Sarcoptiformes	Liacaridae		CNSLH087-12	KM826134

Sarcoptiformes	Liacaridae		MYMCF606-12	JX837703
Sarcoptiformes	Liacaridae		MYMCF608-12	JX833879
Sarcoptiformes	Liacaridae		MYMCF609-12	JX836140
Sarcoptiformes	Listrophoridae	*Leporacarus gibbus*	GBCH4466-10	GQ864335
Sarcoptiformes	Listrophoridae	*Lynxacarus mustelae*	GBCH4465-10	GQ864336
Sarcoptiformes	Listrophoridae	*Schizocarpus sp. AMUChi01*	GBCH4458-10	GQ864344
Sarcoptiformes	Malaconothridae	*Malaconothrus*	CHACB596-10	HQ558695
Sarcoptiformes	Malaconothridae	*Malaconothrus*	CHACB800-10	JX836294
Sarcoptiformes	Malaconothridae	*Malaconothrus*	CHACB801-10	JX834279
Sarcoptiformes	Malaconothridae	*Malaconothrus*	CHACB802-10	JX836043
Sarcoptiformes	Malaconothridae	*Malaconothrus*	CHACB803-10	JX836013
Sarcoptiformes	Malaconothridae	*Malaconothrus*	CHACB804-10	HQ941494
Sarcoptiformes	Malaconothridae	*Malaconothrus*	CHACB805-10	JX837529
Sarcoptiformes	Malaconothridae	*Malaconothrus*	CHACB806-10	HQ941495
Sarcoptiformes	Malaconothridae	*Malaconothrus*	CHACB807-10	JX837905
Sarcoptiformes	Malaconothridae	*Malaconothrus*	CHACB808-10	JX836230
Sarcoptiformes	Malaconothridae	*Malaconothrus*	MYMCC004-11	JX836983
Sarcoptiformes	Malaconothridae	*Malaconothrus*	MYMCC748-11	JX837349
Sarcoptiformes	Malaconothridae	*Malaconothrus*	MYMCC804-11	JX834492
Sarcoptiformes	Malaconothridae	*Malaconothrus*	MYMCD035-11	JX836936
Sarcoptiformes	Malaconothridae	*Malaconothrus*	MYMCE112-12	JX834310
Sarcoptiformes	Malaconothridae	*Malaconothrus*	MYMCE441-12	JX833917
Sarcoptiformes	Malaconothridae	*Malaconothrus*	MYMCF206-12	JX837712
Sarcoptiformes	Malaconothridae	*Malaconothrus*	MYMCF207-12	JX834311
Sarcoptiformes	Malaconothridae	*Malaconothrus mollisetosus*	CHACA201-08	JX834943
Sarcoptiformes	Malaconothridae	*Malaconothrus mollisetosus*	CHACA202-08	JX834028
Sarcoptiformes	Malaconothridae	*Malaconothrus mollisetosus*	CHACA210-08	JX837980
Sarcoptiformes	Malaconothridae	*Malaconothrus mollisetosus*	MYMCF934-12	JX837014
Sarcoptiformes	Malaconothridae	*Malaconothrus mollisetosus*	MYMCF935-12	JX834058
Sarcoptiformes	Malaconothridae		MYMCB443-11	KR069597
Sarcoptiformes	Malaconothridae		MYMCC003-11	JX838251
Sarcoptiformes	Malaconothridae		MYMCC166-11	JX836555
Sarcoptiformes	Malaconothridae		MYMCC802-11	JX834449
Sarcoptiformes	Malaconothridae		MYMCC803-11	JX835614
Sarcoptiformes	Malaconothridae		MYMCE902-12	JX834316
Sarcoptiformes	Malaconothridae		MYMCG503-12	JX834637
Sarcoptiformes	Malaconothridae		MYMCG526-12	JX838365
Sarcoptiformes	Malaconothridae		SSPAA7988-13	KM832716
Sarcoptiformes	Megeremaeidae	*Megeremaeus*	SSBAB1138-12	KM840578
Sarcoptiformes	Megeremaeidae	*Megeremaeus*	SSEIB5806-13	KM830120
Sarcoptiformes	Megeremaeidae	*Megeremaeus*	SSJAA1453-13	KM828081
Sarcoptiformes	Megeremaeidae	*Megeremaeus*	SSJAA1458-13	KM833702
Sarcoptiformes	Megeremaeidae	*Megeremaeus*	SSJAA2177-13	KM839394
Sarcoptiformes	Mochlozetidae	*Podoribates pratensis*	JSAUG1785-12	KR070240
Sarcoptiformes	Mochlozetidae	*Podoribates pratensis*	JSAUG1789-12	KP979190
Sarcoptiformes	Mochlozetidae	*Podoribates pratensis*	JSAUG1793-12	KR069237
Sarcoptiformes	Mochlozetidae	*Podoribates pratensis*	JSAUG1802-12	KR070622
Sarcoptiformes	Mochlozetidae	*Podoribates pratensis*	JSAUG1822-12	KR069320
Sarcoptiformes	Mochlozetidae	*Podoribates pratensis*	JSAUG1831-12	KR070241
Sarcoptiformes	Mochlozetidae	*Podoribates pratensis*	JSAUG1833-12	KR070333
Sarcoptiformes	Mochlozetidae	*Podoribates pratensis*	JSAUG1839-12	KR069582
Sarcoptiformes	Mochlozetidae	*Podoribates pratensis*	JSAUG1856-12	KR069290
Sarcoptiformes	Mochlozetidae	*Podoribates pratensis*	JSAUG1860-12	KR070686

Sarcoptiformes	Mochlozetidae	*Podoribates pratensis*	JSAUG1862-12	KR070081
Sarcoptiformes	Mochlozetidae	*Podoribates pratensis*	JSAUG1865-12	KR070053
Sarcoptiformes	Mochlozetidae	*Podoribates pratensis*	JSJUL2397-12	KR070591
Sarcoptiformes	Mochlozetidae	*Podoribates pratensis*	JSJUL2398-12	KR069641
Sarcoptiformes	Mochlozetidae	*Podoribates pratensis*	JSJUL2399-12	KR069174
Sarcoptiformes	Mochlozetidae	*Podoribates pratensis*	JSJUL2400-12	KR069578
Sarcoptiformes	Mochlozetidae	*Podoribates pratensis*	JSJUL2402-12	KR069760
Sarcoptiformes	Mochlozetidae	*Podoribates pratensis*	JSJUL2527-12	KR070393
Sarcoptiformes	Mochlozetidae	*Podoribates pratensis*	JSJUL2528-12	KR070439
Sarcoptiformes	Mochlozetidae	*Podoribates pratensis*	JSJUL2529-12	KR070125
Sarcoptiformes	Mochlozetidae	*Podoribates pratensis*	JSJUL2531-12	KR070578
Sarcoptiformes	Mochlozetidae	*Podoribates pratensis*	JSJUL2533-12	KR070329
Sarcoptiformes	Mochlozetidae	*Podoribates pratensis*	JSJUL2534-12	KR069814
Sarcoptiformes	Mochlozetidae	*Podoribates pratensis*	JSJUL2535-12	KR070529
Sarcoptiformes	Mochlozetidae	*Podoribates pratensis*	JSJUL2536-12	KR069308
Sarcoptiformes	Mochlozetidae	*Podoribates pratensis*	JSJUL2540-12	KR070732
Sarcoptiformes	Mochlozetidae	*Podoribates pratensis*	JSJUL2543-12	KR069266
Sarcoptiformes	Mochlozetidae	*Podoribates pratensis*	JSJUL2544-12	KR070709
Sarcoptiformes	Mochlozetidae	*Podoribates pratensis*	JSSEP1127-12	KR070624
Sarcoptiformes	Mochlozetidae	*Podoribates pratensis*	JSSEP1129-12	KR069951
Sarcoptiformes	Mochlozetidae	*Podoribates pratensis*	JSSEP1131-12	KR069866
Sarcoptiformes	Mochlozetidae	*Podoribates pratensis*	JSSEP1133-12	KR069991
Sarcoptiformes	Mochlozetidae	*Podoribates pratensis*	JSSEP1134-12	KR070659
Sarcoptiformes	Mochlozetidae	*Podoribates pratensis*	JSSEP1137-12	KR070144
Sarcoptiformes	Mochlozetidae	*Podoribates pratensis*	JSSEP1141-12	KR069524
Sarcoptiformes	Mochlozetidae	*Podoribates pratensis*	JSSEP1142-12	KR070413
Sarcoptiformes	Mochlozetidae	*Podoribates pratensis*	JSSEP1145-12	KR069489
Sarcoptiformes	Mochlozetidae	*Podoribates pratensis*	JSSEP1147-12	KR069690
Sarcoptiformes	Mochlozetidae	*Podoribates pratensis*	JSSEP1148-12	KR069434
Sarcoptiformes	Mochlozetidae	*Podoribates pratensis*	JSSEP1151-12	KR070408
Sarcoptiformes	Mochlozetidae	*Podoribates pratensis*	JSSEP1153-12	KR070617
Sarcoptiformes	Mochlozetidae	*Podoribates pratensis*	JSSEP1154-12	KR069670
Sarcoptiformes	Mochlozetidae	*Podoribates pratensis*	JSSEP1157-12	KR069436
Sarcoptiformes	Mochlozetidae	*Podoribates pratensis*	JSSEP1158-12	KR069844
Sarcoptiformes	Mochlozetidae	*Podoribates pratensis*	JSSEP1160-12	KR069534
Sarcoptiformes	Mochlozetidae		CNPPA4112-12	KJ087519
Sarcoptiformes	Mochlozetidae		CNPPC1940-12	KJ167191
Sarcoptiformes	Mochlozetidae		CNPPC1947-12	KJ089248
Sarcoptiformes	Mochlozetidae		CNPPD2655-12	KJ087608
Sarcoptiformes	Mochlozetidae		CNPPI1272-12	KJ445105
Sarcoptiformes	Mochlozetidae		CNPPI1275-12	KJ209271
Sarcoptiformes	Mochlozetidae		CNPPJ1854-12	KJ209406
Sarcoptiformes	Mochlozetidae		MIONB162-10	KR070386
Sarcoptiformes	Mochlozetidae		MIONB163-10	KP979284
Sarcoptiformes	Mochlozetidae		MIONB165-10	KP979335
Sarcoptiformes	Mochlozetidae		MIONB166-10	KP979300
Sarcoptiformes	Mochlozetidae		NCCE063-11	KP979248
Sarcoptiformes	Mycobatidae	*Mycobates beringianus*	CHACA1073-10	HM907116
Sarcoptiformes	Mycobatidae	*Mycobates beringianus*	CHACA1074-10	HM907117
Sarcoptiformes	Mycobatidae	*Mycobates beringianus*	CHACA1075-10	HM907118
Sarcoptiformes	Mycobatidae	*Mycobates beringianus*	CHACA116-08	JX836745
Sarcoptiformes	Mycobatidae	*Mycobates beringianus*	CHACB054-10	HQ558363
Sarcoptiformes	Mycobatidae	*Mycobates beringianus*	CHACB1125-10	HM907217

Sarcoptiformes	Mycobatidae	*Mycobates beringianus*	CHACB1172-10	HM907248
Sarcoptiformes	Mycobatidae	*Mycobates beringianus*	CHACB1173-10	HM907249
Sarcoptiformes	Mycobatidae	*Mycobates beringianus*	CHACB1174-10	HM907250
Sarcoptiformes	Mycobatidae	*Mycobates beringianus*	CHACB1175-10	HM907251
Sarcoptiformes	Mycobatidae	*Mycobates beringianus*	CHACB1176-10	HM907252
Sarcoptiformes	Mycobatidae	*Mycobates beringianus*	CHACB655-10	HQ558733
Sarcoptiformes	Mycobatidae	*Mycobates beringianus*	CHACB656-10	HQ558734
Sarcoptiformes	Mycobatidae	*Mycobates beringianus*	CHACB657-10	HQ558735
Sarcoptiformes	Mycobatidae	*Mycobates beringianus*	CHACB659-10	HQ558736
Sarcoptiformes	Mycobatidae	*Mycobates beringianus*	CHACB699-10	HQ558756
Sarcoptiformes	Mycobatidae	*Mycobates beringianus*	CHACB700-10	HQ558757
Sarcoptiformes	Mycobatidae	*Mycobates beringianus*	CHACB701-10	HQ558758
Sarcoptiformes	Mycobatidae	*Mycobates beringianus*	CHACB721-10	HQ558771
Sarcoptiformes	Mycobatidae	*Mycobates beringianus*	CHACB722-10	HQ558772
Sarcoptiformes	Mycobatidae	*Mycobates beringianus*	CHACB929-10	HM907356
Sarcoptiformes	Mycobatidae	*Mycobates beringianus*	CHACC006-10	HM907408
Sarcoptiformes	Mycobatidae	*Mycobates beringianus*	CHACC007-10	HM907409
Sarcoptiformes	Mycobatidae	*Mycobates beringianus*	CHACC008-10	HM907410
Sarcoptiformes	Mycobatidae	*Mycobates beringianus*	CHACC009-10	HM907411
Sarcoptiformes	Mycobatidae	*Mycobates beringianus*	MYMCA028-11	JX834163
Sarcoptiformes	Mycobatidae	*Mycobates beringianus*	MYMCA819-11	JX837768
Sarcoptiformes	Mycobatidae	*Mycobates beringianus*	MYMCB246-11	JX835684
Sarcoptiformes	Mycobatidae	*Mycobates beringianus*	MYMCB280-11	JX834938
Sarcoptiformes	Mycobatidae	*Mycobates beringianus*	MYMCB667-11	JX836705
Sarcoptiformes	Mycobatidae	*Mycobates beringianus*	MYMCB668-11	JX835932
Sarcoptiformes	Mycobatidae	*Mycobates beringianus*	MYMCB776-11	JX836997
Sarcoptiformes	Mycobatidae	*Mycobates beringianus*	MYMCB800-11	JX835313
Sarcoptiformes	Mycobatidae	*Mycobates beringianus*	MYMCB815-11	JX835052
Sarcoptiformes	Mycobatidae	*Mycobates beringianus*	MYMCB816-11	JX834831
Sarcoptiformes	Mycobatidae	*Mycobates beringianus*	MYMCB817-11	JX836535
Sarcoptiformes	Mycobatidae	*Mycobates beringianus*	MYMCB820-11	JX837418
Sarcoptiformes	Mycobatidae	*Mycobates beringianus*	MYMCB905-11	JX838453
Sarcoptiformes	Mycobatidae	*Mycobates beringianus*	MYMCB906-11	JX835043
Sarcoptiformes	Mycobatidae	*Mycobates beringianus*	MYMCB907-11	JX835612
Sarcoptiformes	Mycobatidae	*Mycobates beringianus*	MYMCB910-11	JX838301
Sarcoptiformes	Mycobatidae	*Mycobates beringianus*	MYMCB911-11	JX833968
Sarcoptiformes	Mycobatidae	*Mycobates beringianus*	MYMCC328-11	JX836935
Sarcoptiformes	Mycobatidae	*Mycobates beringianus*	MYMCC329-11	JX837254
Sarcoptiformes	Mycobatidae	*Mycobates beringianus*	MYMCC411-11	JX837533
Sarcoptiformes	Mycobatidae	*Mycobates beringianus*	MYMCC412-11	JX836125
Sarcoptiformes	Mycobatidae	*Mycobates beringianus*	MYMCC480-11	JX834101
Sarcoptiformes	Mycobatidae	*Mycobates beringianus*	MYMCC481-11	JX837486
Sarcoptiformes	Mycobatidae	*Mycobates beringianus*	MYMCC742-11	JX838746
Sarcoptiformes	Mycobatidae	*Mycobates beringianus*	MYMCC743-11	JX836817
Sarcoptiformes	Mycobatidae	*Mycobates beringianus*	MYMCC744-11	JX837310
Sarcoptiformes	Mycobatidae	*Mycobates beringianus*	MYMCC859-11	JX837760
Sarcoptiformes	Mycobatidae	*Mycobates beringianus*	MYMCC860-11	JX838527
Sarcoptiformes	Mycobatidae	*Mycobates beringianus*	MYMCC862-11	JX835768
Sarcoptiformes	Mycobatidae	*Mycobates beringianus*	MYMCC864-11	JX838771
Sarcoptiformes	Mycobatidae	*Mycobates beringianus*	MYMCC869-11	JX837828
Sarcoptiformes	Mycobatidae	*Mycobates beringianus*	MYMCC927-11	JX834534
Sarcoptiformes	Mycobatidae	*Mycobates beringianus*	MYMCC928-11	JX834327
Sarcoptiformes	Mycobatidae	*Mycobates beringianus*	MYMCE194-12	JX838268

Sarcoptiformes	Mycobatidae	*Mycobates beringianus*	MYMCE302-12	JX836468
Sarcoptiformes	Mycobatidae	*Mycobates beringianus*	MYMCE332-12	JX836585
Sarcoptiformes	Mycobatidae	*Mycobates beringianus*	MYMCE640-12	JX836414
Sarcoptiformes	Mycobatidae	*Mycobates beringianus*	MYMCF291-12	JX836540
Sarcoptiformes	Mycobatidae	*Mycobates beringianus*	MYMCF434-12	JX834825
Sarcoptiformes	Mycobatidae	*Mycobates beringianus*	MYMCF435-12	JX835501
Sarcoptiformes	Mycobatidae	*Mycobates beringianus*	MYMCF582-12	JX837267
Sarcoptiformes	Mycobatidae	*Mycobates beringianus*	MYMCF583-12	JX837925
Sarcoptiformes	Mycobatidae	*Mycobates beringianus*	MYMCF831-12	JX834810
Sarcoptiformes	Mycobatidae	*Mycobates beringianus*	MYMCF833-12	JX837280
Sarcoptiformes	Mycobatidae	*Mycobates beringianus*	MYMCG423-12	JX838420
Sarcoptiformes	Mycobatidae	*Mycobates beringianus*	MYMCG566-12	JX835359
Sarcoptiformes	Mycobatidae	*Mycobates beringianus*	MYMCG585-12	JX837065
Sarcoptiformes	Mycobatidae	*Mycobates beringianus*	MYTMC035-09	GU680476
Sarcoptiformes	Mycobatidae	*Mycobates conitus*	CHACA018-08	JX835300
Sarcoptiformes	Mycobatidae	*Mycobates conitus*	CHACA019-08	JX838719
Sarcoptiformes	Mycobatidae	*Mycobates conitus*	CHACA022-08	JX838619
Sarcoptiformes	Mycobatidae	*Mycobates conitus*	CHACA094-08	JX835779
Sarcoptiformes	Mycobatidae	*Mycobates conitus*	CHACA592-09	JX836826
Sarcoptiformes	Mycobatidae	*Mycobates conitus*	CHACB924-10	HM907352
Sarcoptiformes	Mycobatidae	*Mycobates conitus*	MYMCA1116-11	JX837223
Sarcoptiformes	Mycobatidae	*Mycobates conitus*	MYMCA776-11	JX836138
Sarcoptiformes	Mycobatidae	*Mycobates conitus*	MYMCA793-11	JX833631
Sarcoptiformes	Mycobatidae	*Mycobates conitus*	MYMCA794-11	JX833949
Sarcoptiformes	Mycobatidae	*Mycobates conitus*	MYMCA800-11	JX834666
Sarcoptiformes	Mycobatidae	*Mycobates conitus*	MYMCB098-11	JX836307
Sarcoptiformes	Mycobatidae	*Mycobates conitus*	MYMCB282-11	JX838612
Sarcoptiformes	Mycobatidae	*Mycobates conitus*	MYMCB283-11	JX834360
Sarcoptiformes	Mycobatidae	*Mycobates conitus*	MYMCB414-11	JX836918
Sarcoptiformes	Mycobatidae	*Mycobates conitus*	MYMCB415-11	JX837373
Sarcoptiformes	Mycobatidae	*Mycobates conitus*	MYMCB643-11	JX837615
Sarcoptiformes	Mycobatidae	*Mycobates conitus*	MYMCB701-11	JX833776
Sarcoptiformes	Mycobatidae	*Mycobates conitus*	MYMCB702-11	JX837088
Sarcoptiformes	Mycobatidae	*Mycobates conitus*	MYMCB705-11	JX837168
Sarcoptiformes	Mycobatidae	*Mycobates conitus*	MYMCB822-11	JX838422
Sarcoptiformes	Mycobatidae	*Mycobates conitus*	MYMCB823-11	JX838048
Sarcoptiformes	Mycobatidae	*Mycobates conitus*	MYMCC146-11	JX833642
Sarcoptiformes	Mycobatidae	*Mycobates conitus*	MYMCC337-11	JX835457
Sarcoptiformes	Mycobatidae	*Mycobates conitus*	MYMCC338-11	JX834874
Sarcoptiformes	Mycobatidae	*Mycobates conitus*	MYMCC475-11	JX834191
Sarcoptiformes	Mycobatidae	*Mycobates conitus*	MYMCC870-11	JX835437
Sarcoptiformes	Mycobatidae	*Mycobates conitus*	MYMCC871-11	JX834290
Sarcoptiformes	Mycobatidae	*Mycobates conitus*	MYMCE314-12	JX838000
Sarcoptiformes	Mycobatidae	*Mycobates conitus*	MYMCE315-12	JX838144
Sarcoptiformes	Mycobatidae	*Mycobates conitus*	MYMCE712-12	JX835012
Sarcoptiformes	Mycobatidae	*Mycobates conitus*	MYMCE784-12	JX836024
Sarcoptiformes	Mycobatidae	*Mycobates conitus*	MYTMC015-09	GU680485
Sarcoptiformes	Mycobatidae	*Mycobates conitus*	MYTMC022-09	GU680483
Sarcoptiformes	Mycobatidae	*Mycobates conitus*	MYTMC023-09	GU680481
Sarcoptiformes	Mycobatidae	*Mycobates conitus*	MYTMC028-09	GU680480
Sarcoptiformes	Mycobatidae	*Mycobates conitus*	MYTMC031-09	GU680479
Sarcoptiformes	Mycobatidae	*Mycobates conitus*	MYTMC048-09	GU680471
Sarcoptiformes	Mycobatidae	*Mycobates conitus*	MYTMC051-09	GU680470

Sarcoptiformes	Mycobatidae	*Mycobates conitus*	MYTMC084-09	GU680459
Sarcoptiformes	Mycobatidae	*Mycobates conitus*	MYTMC090-09	GU680458
Sarcoptiformes	Mycobatidae	*Mycobates conitus*	MYTMC136-09	KR069527
Sarcoptiformes	Mycobatidae	*Mycobates conitus*	MYTMC142-09	GU680436
Sarcoptiformes	Mycobatidae	*Mycobates conitus*	MYTMC175-09	GU680434
Sarcoptiformes	Mycobatidae	*Mycobates conitus*	MYTMC181-09	GU680435
Sarcoptiformes	Mycobatidae	*Mycobates incurvatus*	CHACA037-08	JX834248
Sarcoptiformes	Mycobatidae	*Mycobates incurvatus*	CHACA038-08	JX836241
Sarcoptiformes	Mycobatidae	*Mycobates incurvatus*	CHACA039-08	JX836873
Sarcoptiformes	Mycobatidae	*Mycobates incurvatus*	CHACA1208-10	JX834341
Sarcoptiformes	Mycobatidae	*Mycobates incurvatus*	CHACB788-10	HQ941486
Sarcoptiformes	Mycobatidae	*Mycobates incurvatus*	CHACB789-10	HQ941487
Sarcoptiformes	Mycobatidae	*Mycobates incurvatus*	CHACB790-10	HQ941488
Sarcoptiformes	Mycobatidae	*Mycobates incurvatus*	CHACB791-10	HQ941489
Sarcoptiformes	Mycobatidae	*Mycobates incurvatus*	CHACB792-10	HQ941490
Sarcoptiformes	Mycobatidae	*Mycobates incurvatus*	MYMCA007-11	JX836356
Sarcoptiformes	Mycobatidae	*Mycobates incurvatus*	MYMCA008-11	JX834152
Sarcoptiformes	Mycobatidae	*Mycobates incurvatus*	MYMCA009-11	JX838143
Sarcoptiformes	Mycobatidae	*Mycobates incurvatus*	MYMCA010-11	JX835343
Sarcoptiformes	Mycobatidae	*Mycobates incurvatus*	MYMCA012-11	JX835916
Sarcoptiformes	Mycobatidae	*Mycobates incurvatus*	MYMCA019-11	JX837023
Sarcoptiformes	Mycobatidae	*Mycobates incurvatus*	MYMCA020-11	JX837378
Sarcoptiformes	Mycobatidae	*Mycobates incurvatus*	MYMCA1059-11	JX836965
Sarcoptiformes	Mycobatidae	*Mycobates incurvatus*	MYMCA1060-11	JX837393
Sarcoptiformes	Mycobatidae	*Mycobates incurvatus*	MYMCA1091-11	JX835199
Sarcoptiformes	Mycobatidae	*Mycobates incurvatus*	MYMCA1133-11	JX838031
Sarcoptiformes	Mycobatidae	*Mycobates incurvatus*	MYMCA1134-11	JX835000
Sarcoptiformes	Mycobatidae	*Mycobates incurvatus*	MYMCA1139-11	JX834527
Sarcoptiformes	Mycobatidae	*Mycobates incurvatus*	MYMCA1160-11	JX835251
Sarcoptiformes	Mycobatidae	*Mycobates incurvatus*	MYMCA1161-11	JX836594
Sarcoptiformes	Mycobatidae	*Mycobates incurvatus*	MYMCA307-11	JX836943
Sarcoptiformes	Mycobatidae	*Mycobates incurvatus*	MYMCA426-11	JX838156
Sarcoptiformes	Mycobatidae	*Mycobates incurvatus*	MYMCA490-11	JX833647
Sarcoptiformes	Mycobatidae	*Mycobates incurvatus*	MYMCA491-11	JX836146
Sarcoptiformes	Mycobatidae	*Mycobates incurvatus*	MYMCA492-11	JX838483
Sarcoptiformes	Mycobatidae	*Mycobates incurvatus*	MYMCA537-11	JX836986
Sarcoptiformes	Mycobatidae	*Mycobates incurvatus*	MYMCA538-11	JX837684
Sarcoptiformes	Mycobatidae	*Mycobates incurvatus*	MYMCA539-11	JX833909
Sarcoptiformes	Mycobatidae	*Mycobates incurvatus*	MYMCA547-11	JX837324
Sarcoptiformes	Mycobatidae	*Mycobates incurvatus*	MYMCA548-11	JX834074
Sarcoptiformes	Mycobatidae	*Mycobates incurvatus*	MYMCA913-11	JX837119
Sarcoptiformes	Mycobatidae	*Mycobates incurvatus*	MYMCA941-11	JX834843
Sarcoptiformes	Mycobatidae	*Mycobates incurvatus*	MYMCB026-11	JX837498
Sarcoptiformes	Mycobatidae	*Mycobates incurvatus*	MYMCB027-11	JX836315
Sarcoptiformes	Mycobatidae	*Mycobates incurvatus*	MYMCB043-11	JX838588
Sarcoptiformes	Mycobatidae	*Mycobates incurvatus*	MYMCB112-11	JX838345
Sarcoptiformes	Mycobatidae	*Mycobates incurvatus*	MYMCB514-11	JX838263
Sarcoptiformes	Mycobatidae	*Mycobates incurvatus*	MYMCB535-11	JX834105
Sarcoptiformes	Mycobatidae	*Mycobates incurvatus*	MYMCB536-11	JX833852
Sarcoptiformes	Mycobatidae	*Mycobates incurvatus*	MYMCB537-11	JX835003
Sarcoptiformes	Mycobatidae	*Mycobates incurvatus*	MYMCB846-11	JX835563
Sarcoptiformes	Mycobatidae	*Mycobates incurvatus*	MYMCB847-11	JX835206
Sarcoptiformes	Mycobatidae	*Mycobates incurvatus*	MYMCB909-11	JX835136

Sarcoptiformes	Mycobatidae	*Mycobates incurvatus*	MYMCC013-11	JX835941
Sarcoptiformes	Mycobatidae	*Mycobates incurvatus*	MYMCC014-11	JX837061
Sarcoptiformes	Mycobatidae	*Mycobates incurvatus*	MYMCC052-11	JX838685
Sarcoptiformes	Mycobatidae	*Mycobates incurvatus*	MYMCC085-11	JX834922
Sarcoptiformes	Mycobatidae	*Mycobates incurvatus*	MYMCC188-11	JX835689
Sarcoptiformes	Mycobatidae	*Mycobates incurvatus*	MYMCC189-11	JX834754
Sarcoptiformes	Mycobatidae	*Mycobates incurvatus*	MYMCC191-11	JX837017
Sarcoptiformes	Mycobatidae	*Mycobates incurvatus*	MYMCC216-11	JX835195
Sarcoptiformes	Mycobatidae	*Mycobates incurvatus*	MYMCC218-11	JX835068
Sarcoptiformes	Mycobatidae	*Mycobates incurvatus*	MYMCC359-11	JX836438
Sarcoptiformes	Mycobatidae	*Mycobates incurvatus*	MYMCC485-11	JX834915
Sarcoptiformes	Mycobatidae	*Mycobates incurvatus*	MYMCC486-11	JX836541
Sarcoptiformes	Mycobatidae	*Mycobates incurvatus*	MYMCC593-11	JX835065
Sarcoptiformes	Mycobatidae	*Mycobates incurvatus*	MYMCC594-11	JX834896
Sarcoptiformes	Mycobatidae	*Mycobates incurvatus*	MYMCC633-11	JX838473
Sarcoptiformes	Mycobatidae	*Mycobates incurvatus*	MYMCC634-11	JX837079
Sarcoptiformes	Mycobatidae	*Mycobates incurvatus*	MYMCC683-11	JX834275
Sarcoptiformes	Mycobatidae	*Mycobates incurvatus*	MYMCC902-11	JX836308
Sarcoptiformes	Mycobatidae	*Mycobates incurvatus*	MYMCD155-11	JX834499
Sarcoptiformes	Mycobatidae	*Mycobates incurvatus*	MYMCD156-11	JX833979
Sarcoptiformes	Mycobatidae	*Mycobates incurvatus*	MYMCE472-12	JX837183
Sarcoptiformes	Mycobatidae	*Mycobates incurvatus*	MYMCE659-12	JX836781
Sarcoptiformes	Mycobatidae	*Mycobates incurvatus*	MYMCE710-12	JX837363
Sarcoptiformes	Mycobatidae	*Mycobates incurvatus*	MYMCE752-12	JX833841
Sarcoptiformes	Mycobatidae	*Mycobates incurvatus*	MYMCE814-12	JX835038
Sarcoptiformes	Mycobatidae	*Mycobates incurvatus*	MYMCE832-12	JX833661
Sarcoptiformes	Mycobatidae	*Mycobates incurvatus*	MYMCE923-12	JX836562
Sarcoptiformes	Mycobatidae	*Mycobates incurvatus*	MYMCF064-12	JX838507
Sarcoptiformes	Mycobatidae	*Mycobates incurvatus*	MYMCF159-12	JX837253
Sarcoptiformes	Mycobatidae	*Mycobates incurvatus*	MYMCF761-12	JX834802
Sarcoptiformes	Mycobatidae	*Mycobates incurvatus*	MYMCG033-12	JX836162
Sarcoptiformes	Mycobatidae	*Mycobates incurvatus*	MYMCG097-12	JX836845
Sarcoptiformes	Mycobatidae	*Mycobates incurvatus*	MYMCG222-12	JX835331
Sarcoptiformes	Mycobatidae	*Mycobates incurvatus*	MYMCG223-12	JX835061
Sarcoptiformes	Mycobatidae	*Mycobates incurvatus*	MYMCG281-12	JX837209
Sarcoptiformes	Mycobatidae	*Mycobates incurvatus*	MYMCG435-12	JX838051
Sarcoptiformes	Mycobatidae	*Mycobates incurvatus*	MYMCG476-12	JX836629
Sarcoptiformes	Mycobatidae	*Mycobates incurvatus*	MYMCG478-12	JX835938
Sarcoptiformes	Mycobatidae	*Mycobates incurvatus*	MYMCG479-12	JX834555
Sarcoptiformes	Mycobatidae	*Mycobates incurvatus*	MYTMC054-09	GU680469
Sarcoptiformes	Mycobatidae	*Mycobates incurvatus*	MYTMC112-09	GU680493
Sarcoptiformes	Mycobatidae	*Mycobates incurvatus*	MYTMC114-09	GU680494
Sarcoptiformes	Mycobatidae	*Mycobates incurvatus*	MYTMC138-09	GU680443
Sarcoptiformes	Mycobatidae	*Mycobates incurvatus*	MYTMC150-09	GU680440
Sarcoptiformes	Mycobatidae	*Mycobates incurvatus*	MYTMC161-09	GU680439
Sarcoptiformes	Mycobatidae	*Mycobates incurvatus*	MYTMC165-09	GU680441
Sarcoptiformes	Mycobatidae	*Mycobates perates*	CHACA1120-10	JX835166
Sarcoptiformes	Mycobatidae	*Mycobates perates*	CHACA1121-10	JX836964
Sarcoptiformes	Mycobatidae	*Mycobates perates*	CHACA1122-10	JX836037
Sarcoptiformes	Mycobatidae	*Mycobates perates*	CHACA1123-10	JX836766
Sarcoptiformes	Mycobatidae	*Mycobates perates*	CHACA115-08	JX835102
Sarcoptiformes	Mycobatidae	*Mycobates perates*	CHACA117-08	JX833859
Sarcoptiformes	Mycobatidae	*Mycobates perates*	CHACA118-08	JX837960

Sarcoptiformes	Mycobatidae	*Mycobates perates*	CHACA589-09	JX834287
Sarcoptiformes	Mycobatidae	*Mycobates perates*	CHACB055-10	HQ558364
Sarcoptiformes	Mycobatidae	*Mycobates perates*	CHACB484-10	HQ558619
Sarcoptiformes	Mycobatidae	*Mycobates perates*	CHACB618-10	HQ558705
Sarcoptiformes	Mycobatidae	*Mycobates perates*	CHACB702-10	JX834796
Sarcoptiformes	Mycobatidae	*Mycobates perates*	CHACB793-10	HQ941491
Sarcoptiformes	Mycobatidae	*Mycobates perates*	CHACB870-10	HM907318
Sarcoptiformes	Mycobatidae	*Mycobates perates*	CHACB922-10	HM907350
Sarcoptiformes	Mycobatidae	*Mycobates perates*	CHACB923-10	HM907351
Sarcoptiformes	Mycobatidae	*Mycobates perates*	MYMCA1063-11	JX838412
Sarcoptiformes	Mycobatidae	*Mycobates perates*	MYMCA1131-11	JX835158
Sarcoptiformes	Mycobatidae	*Mycobates perates*	MYMCA268-11	JX834169
Sarcoptiformes	Mycobatidae	*Mycobates perates*	MYMCA550-11	JX836039
Sarcoptiformes	Mycobatidae	*Mycobates perates*	MYMCA551-11	JX838203
Sarcoptiformes	Mycobatidae	*Mycobates perates*	MYMCB205-11	JX834614
Sarcoptiformes	Mycobatidae	*Mycobates perates*	MYMCB279-11	JX835693
Sarcoptiformes	Mycobatidae	*Mycobates perates*	MYMCB539-11	JX836632
Sarcoptiformes	Mycobatidae	*Mycobates perates*	MYMCB706-11	JX835057
Sarcoptiformes	Mycobatidae	*Mycobates perates*	MYMCB777-11	JX836857
Sarcoptiformes	Mycobatidae	*Mycobates perates*	MYMCB801-11	JX837123
Sarcoptiformes	Mycobatidae	*Mycobates perates*	MYMCC307-11	JX838061
Sarcoptiformes	Mycobatidae	*Mycobates perates*	MYMCC308-11	JX833989
Sarcoptiformes	Mycobatidae	*Mycobates perates*	MYMCC738-11	JX834703
Sarcoptiformes	Mycobatidae	*Mycobates perates*	MYMCD066-11	JX834985
Sarcoptiformes	Mycobatidae	*Mycobates perates*	MYMCD119-11	JX837447
Sarcoptiformes	Mycobatidae	*Mycobates perates*	MYMCD120-11	JX834237
Sarcoptiformes	Mycobatidae	*Mycobates perates*	MYMCE431-12	JX834917
Sarcoptiformes	Mycobatidae	*Mycobates perates*	MYMCE833-12	JX837176
Sarcoptiformes	Mycobatidae	*Mycobates perates*	MYMCF541-12	JX837527
Sarcoptiformes	Mycobatidae	*Mycobates perates*	MYMCF542-12	JX835939
Sarcoptiformes	Mycobatidae	*Mycobates perates*	MYMCG161-12	JX835123
Sarcoptiformes	Mycobatidae	*Mycobates perates*	MYMCG162-12	JX833780
Sarcoptiformes	Mycobatidae	*Punctoribates*	MYMCE237-12	JX838100
Sarcoptiformes	Mycobatidae	*Punctoribates*	MYMCE238-12	JX835567
Sarcoptiformes	Mycobatidae	*Punctoribates palustris*	SSEIB4562-13	KM831421
Sarcoptiformes	Mycobatidae	*Punctoribates palustris*	SSEIB4583-13	KM824252
Sarcoptiformes	Mycobatidae	*Punctoribates palustris*	SSPAA6658-13	KM836849
Sarcoptiformes	Mycobatidae	*Punctoribates palustris*	SSPAA6698-13	KM830911
Sarcoptiformes	Mycobatidae		CNPCI013-13	KM824545
Sarcoptiformes	Mycobatidae		CNPCI027-13	KM831793
Sarcoptiformes	Mycobatidae		CNPCI030-13	KM832188
Sarcoptiformes	Mycobatidae		MYMCA346-11	JX838237
Sarcoptiformes	Mycobatidae		MYMCA347-11	JX835475
Sarcoptiformes	Mycobatidae		MYMCB441-11	JX834927
Sarcoptiformes	Mycobatidae		MYMCC167-11	JX835952
Sarcoptiformes	Mycobatidae		MYMCC168-11	JX836151
Sarcoptiformes	Mycobatidae		MYMCC808-11	JX836688
Sarcoptiformes	Mycobatidae		MYMCF936-12	JX837863
Sarcoptiformes	Mycobatidae		MYMCF937-12	JX833805
Sarcoptiformes	Mycobatidae		MYMCG070-12	JX834748
Sarcoptiformes	Mycobatidae		MYMCG071-12	JX835766
Sarcoptiformes	Mycobatidae		MYMCG110-12	JX837831
Sarcoptiformes	Mycobatidae		MYMCG111-12	JX838768

Sarcoptiformes	Mycobatidae		MYMCG112-12	JX833726
Sarcoptiformes	Mycobatidae		MYMCG113-12	JX836046
Sarcoptiformes	Myocoptidae	*Myocoptidae sp. AMUMyo01*	GACAC424-12	GQ864341
Sarcoptiformes	Nanorchestidae	*Nanorchestes*	CHACB1047-10	JX837513
Sarcoptiformes	Nanorchestidae	*Nanorchestes*	CHACB212-10	HQ558474
Sarcoptiformes	Nanorchestidae	*Nanorchestes*	CHACB213-10	JX834648
Sarcoptiformes	Nanorchestidae	*Nanorchestes*	CHACB215-10	HQ558475
Sarcoptiformes	Nanorchestidae	*Nanorchestes*	CHACB329-10	JX833723
Sarcoptiformes	Nanorchestidae	*Nanorchestes*	CHACB453-10	JX838063
Sarcoptiformes	Nanorchestidae	*Nanorchestes*	CHACB454-10	JX833719
Sarcoptiformes	Nanorchestidae	*Nanorchestes*	CHACB625-10	HQ558711
Sarcoptiformes	Nanorchestidae	*Nanorchestes*	CHACB626-10	HQ558712
Sarcoptiformes	Nanorchestidae	*Nanorchestes*	CHACB627-10	HQ558713
Sarcoptiformes	Nanorchestidae	*Nanorchestes*	CHACB628-10	JX836318
Sarcoptiformes	Nanorchestidae	*Nanorchestes*	CHACB665-10	JX837482
Sarcoptiformes	Nanorchestidae	*Nanorchestes*	MYMCA1157-11	JX836660
Sarcoptiformes	Nanorchestidae	*Nanorchestes*	MYMCA1158-11	JX833816
Sarcoptiformes	Nanorchestidae	*Nanorchestes*	MYMCA1159-11	JX837173
Sarcoptiformes	Nanorchestidae	*Nanorchestes*	MYMCA1190-11	JX838188
Sarcoptiformes	Nanorchestidae	*Nanorchestes*	MYMCA1191-11	JX835202
Sarcoptiformes	Nanorchestidae	*Nanorchestes*	MYMCA1192-11	JX834710
Sarcoptiformes	Nanorchestidae	*Nanorchestes*	MYMCA1195-11	JX835248
Sarcoptiformes	Nanorchestidae	*Nanorchestes*	MYMCA1196-11	JX834346
Sarcoptiformes	Nanorchestidae	*Nanorchestes*	MYMCA1447-11	JX836155
Sarcoptiformes	Nanorchestidae	*Nanorchestes*	MYMCA530-11	JX838029
Sarcoptiformes	Nanorchestidae	*Nanorchestes*	MYMCA767-11	JX837376
Sarcoptiformes	Nanorchestidae	*Nanorchestes*	MYMCA977-11	JX838226
Sarcoptiformes	Nanorchestidae	*Nanorchestes*	MYMCB206-11	JX836597
Sarcoptiformes	Nanorchestidae	*Nanorchestes*	MYMCB544-11	JX834702
Sarcoptiformes	Nanorchestidae	*Nanorchestes*	MYMCB641-11	JX837111
Sarcoptiformes	Nanorchestidae	*Nanorchestes*	MYMCB658-11	JX837785
Sarcoptiformes	Nanorchestidae	*Nanorchestes*	MYMCB717-11	JX835323
Sarcoptiformes	Nanorchestidae	*Nanorchestes*	MYMCC160-11	JX837094
Sarcoptiformes	Nanorchestidae	*Nanorchestes*	MYMCC429-11	JX837278
Sarcoptiformes	Nanorchestidae	*Nanorchestes*	MYMCC430-11	JX836591
Sarcoptiformes	Nanorchestidae	*Nanorchestes*	MYMCC523-11	JX838783
Sarcoptiformes	Nanorchestidae	*Nanorchestes*	MYMCC524-11	JX833652
Sarcoptiformes	Nanorchestidae	*Nanorchestes*	MYMCC581-11	JX834830
Sarcoptiformes	Nanorchestidae	*Nanorchestes*	MYMCC589-11	JX837816
Sarcoptiformes	Nanorchestidae	*Nanorchestes*	MYMCC648-11	JX836091
Sarcoptiformes	Nanorchestidae	*Nanorchestes*	MYMCC710-11	JX834433
Sarcoptiformes	Nanorchestidae	*Nanorchestes*	MYMCC711-11	JX837503
Sarcoptiformes	Nanorchestidae	*Nanorchestes*	MYMCC726-11	JX836574
Sarcoptiformes	Nanorchestidae	*Nanorchestes*	MYMCC734-11	JX837159
Sarcoptiformes	Nanorchestidae	*Nanorchestes*	MYMCC754-11	JX834206
Sarcoptiformes	Nanorchestidae	*Nanorchestes*	MYMCC767-11	JX833736
Sarcoptiformes	Nanorchestidae	*Nanorchestes*	MYMCC925-11	JX837663
Sarcoptiformes	Nanorchestidae	*Nanorchestes*	MYMCD146-11	JX835540
Sarcoptiformes	Nanorchestidae	*Nanorchestes*	MYMCD187-11	JX834787
Sarcoptiformes	Nanorchestidae	*Nanorchestes*	MYMCD189-11	JX834579
Sarcoptiformes	Nanorchestidae	*Nanorchestes*	MYMCD190-11	JX835773
Sarcoptiformes	Nanorchestidae	*Nanorchestes*	MYMCE032-12	JX837967
Sarcoptiformes	Nanorchestidae	*Nanorchestes*	MYMCE139-12	JX835796

Sarcoptiformes	Nanorchestidae	*Nanorchestes*		MYMCE172-12	JX834704
Sarcoptiformes	Nanorchestidae	*Nanorchestes*		MYMCE432-12	JX838472
Sarcoptiformes	Nanorchestidae	*Nanorchestes*		MYMCE433-12	JX836186
Sarcoptiformes	Nanorchestidae	*Nanorchestes*		MYMCE484-12	JX837528
Sarcoptiformes	Nanorchestidae	*Nanorchestes*		MYMCE492-12	JX835168
Sarcoptiformes	Nanorchestidae	*Nanorchestes*		MYMCE493-12	JX837036
Sarcoptiformes	Nanorchestidae	*Nanorchestes*		MYMCE626-12	JX836645
Sarcoptiformes	Nanorchestidae	*Nanorchestes*		MYMCE633-12	JX835287
Sarcoptiformes	Nanorchestidae	*Nanorchestes*		MYMCE749-12	JX836331
Sarcoptiformes	Nanorchestidae	*Nanorchestes*		MYMCE768-12	JX836415
Sarcoptiformes	Nanorchestidae	*Nanorchestes*		MYMCE769-12	JX836881
Sarcoptiformes	Nanorchestidae	*Nanorchestes*		MYMCE770-12	JX833901
Sarcoptiformes	Nanorchestidae	*Nanorchestes*		MYMCF009-12	JX834075
Sarcoptiformes	Nanorchestidae	*Nanorchestes*		MYMCF010-12	JX836913
Sarcoptiformes	Nanorchestidae	*Nanorchestes*		MYMCF035-12	JX836598
Sarcoptiformes	Nanorchestidae	*Nanorchestes*		MYMCF145-12	JX838094
Sarcoptiformes	Nanorchestidae	*Nanorchestes*		MYMCF169-12	JX836734
Sarcoptiformes	Nanorchestidae	*Nanorchestes*		MYMCF170-12	JX834640
Sarcoptiformes	Nanorchestidae	*Nanorchestes*		MYMCF171-12	JX835486
Sarcoptiformes	Nanorchestidae	*Nanorchestes*		MYMCF348-12	JX837326
Sarcoptiformes	Nanorchestidae	*Nanorchestes*		MYMCF361-12	JX836963
Sarcoptiformes	Nanorchestidae	*Nanorchestes*		MYMCF471-12	JX833972
Sarcoptiformes	Nanorchestidae	*Nanorchestes*		MYMCF550-12	JX835321
Sarcoptiformes	Nanorchestidae	*Nanorchestes*		MYMCF652-12	JX836548
Sarcoptiformes	Nanorchestidae	*Nanorchestes*		MYMCF682-12	JX837227
Sarcoptiformes	Nanorchestidae	*Nanorchestes*		MYMCF783-12	JX835478
Sarcoptiformes	Nanorchestidae	*Nanorchestes*		MYMCF864-12	JX838603
Sarcoptiformes	Nanorchestidae	*Nanorchestes*		MYMCG020-12	JX834119
Sarcoptiformes	Nanorchestidae	*Nanorchestes*		MYMCG361-12	JX837573
Sarcoptiformes	Nanorchestidae	*Nanorchestes*		MYMCG561-12	JX838397
Sarcoptiformes	Nanorchestidae	*Nanorchestes*		MYMCG624-12	JX836009
Sarcoptiformes	Nanorchestidae	*Nanorchestes*		MYMCG625-12	JX836299
Sarcoptiformes	Nanorchestidae	*Nanorchestes*		MYTMC017-09	HQ966224
Sarcoptiformes	Nanorchestidae	*Nanorchestes*		MYTMC178-09	KR070245
Sarcoptiformes	Neoliodidae			MIONB249-10	KR070203
Sarcoptiformes	Neoliodidae			MIONB250-10	KR070219
Sarcoptiformes	Neoliodidae			MIONB251-10	KP979223
Sarcoptiformes	Neoliodidae			MIONB252-10	KR070217
Sarcoptiformes	Neoliodidae			MIONB253-10	KP979227
Sarcoptiformes	Neoliodidae			MIONB291-10	KP979268
Sarcoptiformes	Nothridae	*Nothrus*		SSGBB1583-14	KR069364
Sarcoptiformes	Nothridae	*Nothrus*		SSGBB1589-14	KR070442
Sarcoptiformes	Nothridae	*Nothrus*		SSGBB1591-14	KR070619
Sarcoptiformes	Nothridae	*Nothrus*		SSGBB1592-14	KR069753
Sarcoptiformes	Nothridae	*Nothrus*		SSGBB1594-14	KR070071
Sarcoptiformes	Nothridae	*Nothrus*		SSGBB1596-14	KR070380
Sarcoptiformes	Nothridae	*Nothrus*		SSGBB1597-14	KR070730
Sarcoptiformes	Nothridae	*Nothrus*		SSGBB1600-14	KR070704
Sarcoptiformes	Nothridae	*Nothrus*		SSGBB1613-14	KR069732
Sarcoptiformes	Nothridae	*Nothrus*		SSGBB1646-14	KR069932
Sarcoptiformes	Nothridae	*Nothrus*		SSGBB1682-14	KR070286
Sarcoptiformes	Nothridae	*Nothrus*		SSGBB1685-14	KR070501
Sarcoptiformes	Nothridae	*Nothrus*		SSGBB1687-14	KR069579

Sarcoptiformes	Nothridae	*Nothrus*	SSGBB1712-14	KR069224
Sarcoptiformes	Nothridae	*Nothrus*	SSGBB1754-14	KR069744
Sarcoptiformes	Nothridae	*Nothrus*	SSGBB1757-14	KR069240
Sarcoptiformes	Nothridae	*Nothrus*	SSGBB1763-14	KR069762
Sarcoptiformes	Nothridae	*Nothrus*	SSGBB1779-14	KR069938
Sarcoptiformes	Nothridae	*Nothrus*	SSGBB1783-14	KR069912
Sarcoptiformes	Nothridae	*Nothrus*	SSGBB1795-14	KR069244
Sarcoptiformes	Nothridae	*Nothrus*	SSGBB1799-14	KR070225
Sarcoptiformes	Nothridae	*Nothrus*	SSGBB1817-14	KR069953
Sarcoptiformes	Nothridae	*Nothrus*	SSGBB1835-14	KR070519
Sarcoptiformes	Nothridae	*Nothrus*	SSGBB1836-14	KR070331
Sarcoptiformes	Nothridae	*Nothrus*	SSGBB1842-14	KR070698
Sarcoptiformes	Nothridae	*Nothrus*	SSGBB1849-14	KR069424
Sarcoptiformes	Nothridae	*Nothrus*	SSGBB1865-14	KR069994
Sarcoptiformes	Nothridae	*Nothrus*	SSGBB1894-14	KR070072
Sarcoptiformes	Nothridae	*Nothrus*	SSGBB1895-14	KR069802
Sarcoptiformes	Nothridae	*Nothrus*	SSGBB1899-14	KR069259
Sarcoptiformes	Nothridae	*Nothrus*	SSGBB1905-14	KR070080
Sarcoptiformes	Nothridae	*Nothrus*	BIOAI182-14	KR070317
Sarcoptiformes	Nothridae	*Nothrus*	BIOAI256-14	KP979253
Sarcoptiformes	Nothridae	*Nothrus*	BIOAI480-14	KR070067
Sarcoptiformes	Nothridae	*Nothrus*	BIOAI486-14	KR069183
Sarcoptiformes	Nothridae	*Nothrus*	BIOAI507-14	KP979305
Sarcoptiformes	Nothridae	*Nothrus*	CHACA1004-10	HM907069
Sarcoptiformes	Nothridae	*Nothrus*	CHACA1005-10	HM907070
Sarcoptiformes	Nothridae	*Nothrus*	CHACA1006-10	HM907071
Sarcoptiformes	Nothridae	*Nothrus*	CHACA1007-10	HM907072
Sarcoptiformes	Nothridae	*Nothrus*	CHACA1008-10	HM907073
Sarcoptiformes	Nothridae	*Nothrus*	CHACA1017-10	HM907077
Sarcoptiformes	Nothridae	*Nothrus*	CHACB008-10	HQ558331
Sarcoptiformes	Nothridae	*Nothrus*	CHACB049-10	HQ558360
Sarcoptiformes	Nothridae	*Nothrus*	CHACB1117-10	HM907211
Sarcoptiformes	Nothridae	*Nothrus*	CHACB179-10	HQ558454
Sarcoptiformes	Nothridae	*Nothrus*	CHACB551-10	HQ558667
Sarcoptiformes	Nothridae	*Nothrus*	CHACB613-10	HQ558701
Sarcoptiformes	Nothridae	*Nothrus*	CHACB705-10	HQ558760
Sarcoptiformes	Nothridae	*Nothrus*	CHACB706-10	HQ558761
Sarcoptiformes	Nothridae	*Nothrus*	CHACB707-10	JX835760
Sarcoptiformes	Nothridae	*Nothrus*	CHACB708-10	HQ558762
Sarcoptiformes	Nothridae	*Nothrus*	CHACB709-10	JX835667
Sarcoptiformes	Nothridae	*Nothrus*	CHACB827-10	HQ941500
Sarcoptiformes	Nothridae	*Nothrus*	CHACB828-10	HQ941501
Sarcoptiformes	Nothridae	*Nothrus*	CHACB829-10	HQ941502
Sarcoptiformes	Nothridae	*Nothrus*	CHACB830-10	HQ941503
Sarcoptiformes	Nothridae	*Nothrus*	CHACB831-10	HQ941504
Sarcoptiformes	Nothridae	*Nothrus*	CHACB832-10	HQ941505
Sarcoptiformes	Nothridae	*Nothrus*	CHACB976-10	HM907387
Sarcoptiformes	Nothridae	*Nothrus*	CHACC121-10	JX837157
Sarcoptiformes	Nothridae	*Nothrus*	CHACC128-10	JX834597
Sarcoptiformes	Nothridae	*Nothrus*	CHACC129-10	JX838514
Sarcoptiformes	Nothridae	*Nothrus*	MIONB276-10	KP979150
Sarcoptiformes	Nothridae	*Nothrus*	MITMH027-07	KP979256
Sarcoptiformes	Nothridae	*Nothrus*	MYMCA083-11	JX838321

Sarcoptiformes	Nothridae	*Nothrus*	MYMCA1065-11	JX834462
Sarcoptiformes	Nothridae	*Nothrus*	MYMCA1423-11	JX835269
Sarcoptiformes	Nothridae	*Nothrus*	MYMCA245-11	JX837767
Sarcoptiformes	Nothridae	*Nothrus*	MYMCA246-11	JX834379
Sarcoptiformes	Nothridae	*Nothrus*	MYMCA247-11	JX836395
Sarcoptiformes	Nothridae	*Nothrus*	MYMCA248-11	JX838261
Sarcoptiformes	Nothridae	*Nothrus*	MYMCA249-11	JX835442
Sarcoptiformes	Nothridae	*Nothrus*	MYMCA428-11	JX834005
Sarcoptiformes	Nothridae	*Nothrus*	MYMCA429-11	JX834125
Sarcoptiformes	Nothridae	*Nothrus*	MYMCA478-11	JX836410
Sarcoptiformes	Nothridae	*Nothrus*	MYMCA479-11	JX834269
Sarcoptiformes	Nothridae	*Nothrus*	MYMCA540-11	JX837147
Sarcoptiformes	Nothridae	*Nothrus*	MYMCB227-11	JX836786
Sarcoptiformes	Nothridae	*Nothrus*	MYMCB264-11	JX834478
Sarcoptiformes	Nothridae	*Nothrus*	MYMCC067-11	JX834910
Sarcoptiformes	Nothridae	*Nothrus*	MYMCC251-11	JX837085
Sarcoptiformes	Nothridae	*Nothrus*	MYMCC291-11	JX835133
Sarcoptiformes	Nothridae	*Nothrus*	MYMCC292-11	JX837762
Sarcoptiformes	Nothridae	*Nothrus*	MYMCC304-11	JX836886
Sarcoptiformes	Nothridae	*Nothrus*	MYMCE245-12	JX834013
Sarcoptiformes	Nothridae	*Nothrus*	MYMCE835-12	JX837177
Sarcoptiformes	Nothridae	*Nothrus*	MYMCF324-12	JX834590
Sarcoptiformes	Nothridae	*Nothrus*	MYMCF433-12	JX837694
Sarcoptiformes	Nothridae	*Nothrus*	MYMCF804-12	JX838236
Sarcoptiformes	Nothridae	*Nothrus*	MYMCG191-12	JX837737
Sarcoptiformes	Nothridae	*Nothrus*	MYTMC014-09	HQ966223
Sarcoptiformes	Nothridae	*Nothrus*	MYTMC026-09	HQ966225
Sarcoptiformes	Nothridae	*Nothrus*	MYTMC027-09	HQ966226
Sarcoptiformes	Nothridae	*Nothrus*	MYTMC058-09	GU680467
Sarcoptiformes	Nothridae	*Nothrus*	MYTMC062-09	GU680465
Sarcoptiformes	Nothridae	*Nothrus*	RBINA5649-13	KP979137
Sarcoptiformes	Nothridae	*Nothrus anauniensis*	RBINA5684-13	KP979344
Sarcoptiformes	Nothridae	*Nothrus borussicus*	SSJAB2151-13	KM832928
Sarcoptiformes	Nothridae	*Nothrus pratensis*	MITMH001-07	KR070485
Sarcoptiformes	Nothridae	*Nothrus pratensis*	MITMH005-07	KR069368
Sarcoptiformes	Nothridae	*Nothrus pratensis*	MYMCB445-11	JX835468
Sarcoptiformes	Nothridae	*Nothrus pratensis*	MYMCB448-11	JX835225
Sarcoptiformes	Nothridae	*Nothrus pratensis*	MYMCC768-11	JX835728
Sarcoptiformes	Nothridae	*Nothrus silvestris*	GBCH3784-09	DQ381160
Sarcoptiformes	Nothridae	*Nothrus silvestris*	GBCH3785-09	DQ381159
Sarcoptiformes	Nothridae	*Nothrus silvestris*	GBCH3786-09	DQ381158
Sarcoptiformes	Nothridae	*Nothrus silvestris*	GBCH3787-09	DQ381157
Sarcoptiformes	Nothridae	*Nothrus silvestris*	GBCH6672-13	JF263836
Sarcoptiformes	Nothridae	*Nothrus silvestris*	GBCH6673-13	JF263835
Sarcoptiformes	Nothridae	*Nothrus silvestris*	GBCH6674-13	JF263834
Sarcoptiformes	Nothridae	*Nothrus silvestris*	GBCH6675-13	JF263833
Sarcoptiformes	Nothridae	*Nothrus silvestris*	GBCH6676-13	JF263832
Sarcoptiformes	Nothridae	*Nothrus silvestris*	GBCH6677-13	JF263831
Sarcoptiformes	Nothridae	*Nothrus silvestris*	GBCH6678-13	JF263830
Sarcoptiformes	Nothridae	*Nothrus silvestris*	GBCH6679-13	JF263829
Sarcoptiformes	Nothridae	*Nothrus silvestris*	GBCH6680-13	JF263828
Sarcoptiformes	Nothridae	*Nothrus silvestris*	GBCH6681-13	JF263827
Sarcoptiformes	Nothridae	*Nothrus silvestris*	GBCH6682-13	JF263826

Sarcoptiformes	Nothridae	*Nothrus silvestris*	GBCH6683-13	JF263825
Sarcoptiformes	Nothridae	*Nothrus silvestris*	GBCH6684-13	JF263824
Sarcoptiformes	Nothridae	*Nothrus silvestris*	GBCH6685-13	JF263823
Sarcoptiformes	Nothridae	*Nothrus silvestris*	GBCH6686-13	JF263822
Sarcoptiformes	Nothridae	*Nothrus silvestris*	GBCH6687-13	JF263821
Sarcoptiformes	Nothridae	*Nothrus silvestris*	GBCH6688-13	JF263820
Sarcoptiformes	Nothridae	*Nothrus silvestris*	GBCH6689-13	JF263819
Sarcoptiformes	Nothridae	*Nothrus silvestris*	GBCH6690-13	JF263818
Sarcoptiformes	Nothridae	*Nothrus silvestris*	GBCH6691-13	JF263817
Sarcoptiformes	Nothridae	*Nothrus silvestris*	GBCH6692-13	JF263816
Sarcoptiformes	Nothridae	*Nothrus silvestris*	GBCH6693-13	JF263815
Sarcoptiformes	Nothridae	*Nothrus silvestris*	GBCH6694-13	JF263814
Sarcoptiformes	Nothridae	*Nothrus silvestris*	GBCH6695-13	JF263813
Sarcoptiformes	Nothridae	*Nothrus silvestris*	GBCH6696-13	JF263812
Sarcoptiformes	Nothridae	*Nothrus silvestris*	GBCH6697-13	JF263811
Sarcoptiformes	Nothridae	*Nothrus silvestris*	GBCH6698-13	JF263810
Sarcoptiformes	Nothridae	*Nothrus silvestris*	GBCH6699-13	JF263809
Sarcoptiformes	Nothridae	*Nothrus silvestris*	GBCH6700-13	JF263808
Sarcoptiformes	Nothridae	*Nothrus silvestris*	GBCH6701-13	JF263807
Sarcoptiformes	Nothridae	*Nothrus silvestris*	GBCH6702-13	JF263806
Sarcoptiformes	Nothridae	*Nothrus silvestris*	GBCH6703-13	JF263805
Sarcoptiformes	Nothridae	*Nothrus silvestris*	GBCH6704-13	JF263804
Sarcoptiformes	Nothridae	*Nothrus silvestris*	GBCH6705-13	JF263803
Sarcoptiformes	Nothridae	*Nothrus silvestris*	GBCH6706-13	JF263802
Sarcoptiformes	Nothridae	*Nothrus silvestris*	GBCH6707-13	JF263801
Sarcoptiformes	Nothridae	*Nothrus silvestris*	GBCH6708-13	JF263800
Sarcoptiformes	Nothridae	*Nothrus silvestris*	GBCH6709-13	JF263799
Sarcoptiformes	Nothridae	*Nothrus silvestris*	GBCH6710-13	JF263798
Sarcoptiformes	Nothridae	*Nothrus silvestris*	GBCH6711-13	JF263797
Sarcoptiformes	Nothridae	*Nothrus silvestris*	GBCH6712-13	JF263796
Sarcoptiformes	Nothridae	*Nothrus silvestris*	GBCH6713-13	JF263795
Sarcoptiformes	Nothridae	*Nothrus silvestris*	GBCH6714-13	JF263794
Sarcoptiformes	Nothridae	*Nothrus silvestris*	GBCH6715-13	JF263793
Sarcoptiformes	Nothridae	*Nothrus silvestris*	GBCH6716-13	JF263792
Sarcoptiformes	Nothridae	*Nothrus silvestris*	GBCH6717-13	JF263791
Sarcoptiformes	Nothridae	*Nothrus silvestris*	GBCH6718-13	JF263790
Sarcoptiformes	Nothridae	*Nothrus silvestris*	GBCH6719-13	JF263789
Sarcoptiformes	Nothridae	*Nothrus silvestris*	GBCH6720-13	JF263788
Sarcoptiformes	Nothridae	*Nothrus silvestris*	GBCH6721-13	JF263787
Sarcoptiformes	Nothridae	*Nothrus silvestris*	GBCH6722-13	JF263786
Sarcoptiformes	Nothridae	*Nothrus silvestris*	GBCH6723-13	JF263785
Sarcoptiformes	Nothridae	*Nothrus silvestris*	GBCH6724-13	JF263784
Sarcoptiformes	Nothridae	*Nothrus silvestris*	GBCH6725-13	JF263783
Sarcoptiformes	Nothridae	*Nothrus silvestris*	GBCH6726-13	JF263782
Sarcoptiformes	Nothridae	*Nothrus silvestris*	GBCH6727-13	JF263781
Sarcoptiformes	Nothridae	*Nothrus silvestris*	GBCH6728-13	JF263780
Sarcoptiformes	Nothridae	*Nothrus silvestris*	GBCH6729-13	JF263779
Sarcoptiformes	Nothridae	*Nothrus silvestris*	GBCH6730-13	JF263778
Sarcoptiformes	Nothridae	*Nothrus silvestris*	GBCH6731-13	JF263777
Sarcoptiformes	Nothridae	*Nothrus silvestris*	GBCH6732-13	JF263776
Sarcoptiformes	Nothridae	*Nothrus silvestris*	GBCH6733-13	JF263775
Sarcoptiformes	Nothridae	*Nothrus silvestris*	GBCH6734-13	JF263774
Sarcoptiformes	Nothridae	*Nothrus silvestris*	GBCH6735-13	JF263773

Sarcoptiformes	Nothridae	*Nothrus silvestris*	GBCH6736-13	JF263772
Sarcoptiformes	Nothridae	*Nothrus silvestris*	GBCH6737-13	JF263771
Sarcoptiformes	Nothridae	*Nothrus silvestris*	GBCH6738-13	JF263770
Sarcoptiformes	Nothridae	*Nothrus silvestris*	GBCH6739-13	JF263769
Sarcoptiformes	Nothridae	*Nothrus silvestris*	GBCH6740-13	JF263768
Sarcoptiformes	Nothridae	*Nothrus silvestris*	GBCH6741-13	JF263767
Sarcoptiformes	Nothridae	*Nothrus silvestris*	GBCH6742-13	JF263766
Sarcoptiformes	Nothridae	*Nothrus silvestris*	GBCH6743-13	JF263765
Sarcoptiformes	Nothridae	*Nothrus silvestris*	GBCH6744-13	JF263764
Sarcoptiformes	Nothridae	*Nothrus silvestris*	GBCH6745-13	JF263763
Sarcoptiformes	Nothridae	*Nothrus silvestris*	GBCH6746-13	JF263762
Sarcoptiformes	Nothridae	*Nothrus silvestris*	GBCH6747-13	JF263761
Sarcoptiformes	Nothridae	*Nothrus silvestris*	GBCH6748-13	JF263760
Sarcoptiformes	Nothridae	*Nothrus silvestris*	GBCH6749-13	JF263759
Sarcoptiformes	Nothridae	*Nothrus silvestris*	GBCH6750-13	JF263758
Sarcoptiformes	Nothridae	*Nothrus silvestris*	GBCH6751-13	JF263757
Sarcoptiformes	Nothridae	*Nothrus silvestris*	GBCH6752-13	JF263756
Sarcoptiformes	Nothridae	*Nothrus silvestris*	GBCH6753-13	JF263755
Sarcoptiformes	Nothridae	*Nothrus silvestris*	GBCH6754-13	JF263754
Sarcoptiformes	Nothridae	*Nothrus silvestris*	GBCH6755-13	JF263753
Sarcoptiformes	Nothridae	*Nothrus silvestris*	GBCH6756-13	JF263752
Sarcoptiformes	Nothridae	*Nothrus silvestris*	GBCH6757-13	JF263751
Sarcoptiformes	Nothridae	*Nothrus silvestris*	GBCH6758-13	JF263750
Sarcoptiformes	Nothridae	*Nothrus silvestris*	GBCH6759-13	JF263749
Sarcoptiformes	Nothridae	*Nothrus silvestris*	GBCH6760-13	JF263748
Sarcoptiformes	Nothridae	*Nothrus silvestris*	GBCH6761-13	JF263747
Sarcoptiformes	Nothridae	*Nothrus silvestris*	GBCH6762-13	JF263746
Sarcoptiformes	Nothridae	*Nothrus silvestris*	GBCH6763-13	JF263745
Sarcoptiformes	Nothridae	*Nothrus silvestris*	GBCH6764-13	JF263744
Sarcoptiformes	Nothridae	*Nothrus silvestris*	GBCH6765-13	JF263743
Sarcoptiformes	Nothridae	*Nothrus silvestris*	GBCH6766-13	JF263742
Sarcoptiformes	Nothridae	*Nothrus silvestris*	GBCH6767-13	JF263741
Sarcoptiformes	Nothridae	*Nothrus silvestris*	GBCH6768-13	JF263740
Sarcoptiformes	Nothridae	*Nothrus silvestris*	GBCH6769-13	JF263739
Sarcoptiformes	Nothridae	*Nothrus silvestris*	GBCH6770-13	JF263738
Sarcoptiformes	Nothridae	*Nothrus silvestris*	GBCH6771-13	JF263737
Sarcoptiformes	Oppiidae		SSPAA9158-13	KR070618
Sarcoptiformes	Oppiidae	*Opiella*	CHACB1129-10	HM907221
Sarcoptiformes	Oppiidae	*Opiella*	CHACB696-10	JX838641
Sarcoptiformes	Oppiidae	*Opiella*	CHACC147-10	JX837651
Sarcoptiformes	Oppiidae	*Opiella*	CHACC148-10	JX837842
Sarcoptiformes	Oppiidae	*Opiella*	CHACC149-10	JX835322
Sarcoptiformes	Oppiidae	*Opiella*	MYMCA376-11	JX835907
Sarcoptiformes	Oppiidae	*Opiella*	MYMCA380-11	JX836218
Sarcoptiformes	Oppiidae	*Opiella*	MYMCA892-11	JX834944
Sarcoptiformes	Oppiidae	*Opiella*	MYMCB229-11	JX836222
Sarcoptiformes	Oppiidae	*Opiella*	MYMCB497-11	JX836557
Sarcoptiformes	Oppiidae	*Opiella*	MYMCB498-11	JX838721
Sarcoptiformes	Oppiidae	*Opiella*	MYMCC805-11	JX836661
Sarcoptiformes	Oppiidae	*Opiella*	MYMCC806-11	JX835318
Sarcoptiformes	Oppiidae	*Opiella*	MYMCC807-11	JX834010
Sarcoptiformes	Oppiidae	*Opiella*	MYMCE328-12	JX836116
Sarcoptiformes	Oppiidae	*Opiella*	MYMCF200-12	JX837207

Sarcoptiformes	Oppiidae	*Opiella*	MYMCF342-12	JX838549
Sarcoptiformes	Oppiidae	*Opiella*	MYMCF502-12	JX836393
Sarcoptiformes	Oppiidae	*Opiella*	MYMCF503-12	JX834625
Sarcoptiformes	Oppiidae	*Opiella*	MYMCF504-12	JX833704
Sarcoptiformes	Oppiidae	*Opiella*	MYMCF544-12	JX838762
Sarcoptiformes	Oppiidae	*Opiella*	MYMCF592-12	JX838119
Sarcoptiformes	Oppiidae	*Opiella*	MYMCF593-12	JX837331
Sarcoptiformes	Oppiidae	*Opiella*	MYMCF729-12	JX836309
Sarcoptiformes	Oppiidae	*Opiella*	MYMCF875-12	JX833783
Sarcoptiformes	Oppiidae	*Opiella*	MYMCF876-12	JX836993
Sarcoptiformes	Oppiidae	*Opiella*	MYMCF916-12	JX835465
Sarcoptiformes	Oppiidae	*Opiella*	MYMCG382-12	JX835433
Sarcoptiformes	Oppiidae	*Opiella*	MYMCG383-12	JX835150
Sarcoptiformes	Oppiidae	*Opiella*	MYTMC075-09	HQ966233
Sarcoptiformes	Oppiidae	*Opiella*	SSBAB2822-12	KM835419
Sarcoptiformes	Oppiidae	*Opiella*	SSBAB2823-12	KM824298
Sarcoptiformes	Oppiidae	*Opiella*	SSBAB2830-12	KM831640
Sarcoptiformes	Oppiidae	*Opiella*	SSBAB2858-12	KM839638
Sarcoptiformes	Oppiidae	*Opiella*	SSBAB2859-12	KM840253
Sarcoptiformes	Oppiidae	*Opiella*	SSBAB2861-12	KM839570
Sarcoptiformes	Oppiidae	*Oppiella clavigera*	CHACB678-10	HQ558744
Sarcoptiformes	Oppiidae		CHACA068-08	JX834925
Sarcoptiformes	Oppiidae		CHACA070-08	JX836771
Sarcoptiformes	Oppiidae		CHACA071-08	JX834901
Sarcoptiformes	Oppiidae		CHACA072-08	JX835910
Sarcoptiformes	Oppiidae		CHACA1001-10	HM405808
Sarcoptiformes	Oppiidae		CHACA1002-10	HM405809
Sarcoptiformes	Oppiidae		CHACA1003-10	HM405810
Sarcoptiformes	Oppiidae		CHACA1125-10	JX835461
Sarcoptiformes	Oppiidae		CHACA1126-10	JX836213
Sarcoptiformes	Oppiidae		CHACA1127-10	JX837092
Sarcoptiformes	Oppiidae		CHACA123-08	JX833922
Sarcoptiformes	Oppiidae		CHACA203-08	JX836447
Sarcoptiformes	Oppiidae		CHACA223-08	JX834804
Sarcoptiformes	Oppiidae		CHACA224-08	JX834929
Sarcoptiformes	Oppiidae		CHACA225-08	JX835370
Sarcoptiformes	Oppiidae		CHACA226-08	JX836862
Sarcoptiformes	Oppiidae		CHACA362-08	JX838130
Sarcoptiformes	Oppiidae		CHACA989-10	HM405851
Sarcoptiformes	Oppiidae		CHACA990-10	JX835355
Sarcoptiformes	Oppiidae		CHACA991-10	HM405852
Sarcoptiformes	Oppiidae		CHACA992-10	HM405853
Sarcoptiformes	Oppiidae		CHACA993-10	HM405854
Sarcoptiformes	Oppiidae		CHACB061-10	HQ558369
Sarcoptiformes	Oppiidae		CHACB062-10	HQ558370
Sarcoptiformes	Oppiidae		CHACB063-10	HQ558371
Sarcoptiformes	Oppiidae		CHACB064-10	HQ558372
Sarcoptiformes	Oppiidae		CHACB1084-10	HM907196
Sarcoptiformes	Oppiidae		CHACB112-10	HQ558405
Sarcoptiformes	Oppiidae		CHACB1128-10	HM907220
Sarcoptiformes	Oppiidae		CHACB1152-10	HM907238
Sarcoptiformes	Oppiidae		CHACB1154-10	HM907239
Sarcoptiformes	Oppiidae		CHACB1155-10	HM907240

Sarcoptiformes	Oppiidae	CHACB1203-10	HM907273
Sarcoptiformes	Oppiidae	CHACB1205-10	HM907275
Sarcoptiformes	Oppiidae	CHACB1207-10	HM907277
Sarcoptiformes	Oppiidae	CHACB374-10	HQ558550
Sarcoptiformes	Oppiidae	CHACB375-10	HQ558551
Sarcoptiformes	Oppiidae	CHACB376-10	HQ558552
Sarcoptiformes	Oppiidae	CHACB608-10	JX837855
Sarcoptiformes	Oppiidae	CHACB609-10	JX836032
Sarcoptiformes	Oppiidae	CHACB610-10	JX836519
Sarcoptiformes	Oppiidae	CHACB611-10	JX834726
Sarcoptiformes	Oppiidae	CHACB612-10	JX836373
Sarcoptiformes	Oppiidae	CHACB620-10	HQ558707
Sarcoptiformes	Oppiidae	CHACB621-10	HQ558708
Sarcoptiformes	Oppiidae	CHACB622-10	HQ558709
Sarcoptiformes	Oppiidae	CHACB623-10	HQ558710
Sarcoptiformes	Oppiidae	CHACB624-10	HQ941473
Sarcoptiformes	Oppiidae	CHACB646-10	HQ558726
Sarcoptiformes	Oppiidae	CHACB647-10	HQ558727
Sarcoptiformes	Oppiidae	CHACB648-10	HQ558728
Sarcoptiformes	Oppiidae	CHACB649-10	HQ558729
Sarcoptiformes	Oppiidae	CHACB650-10	HQ558730
Sarcoptiformes	Oppiidae	CHACB662-10	JX836518
Sarcoptiformes	Oppiidae	CHACB727-10	HQ558776
Sarcoptiformes	Oppiidae	CHACB728-10	HQ558777
Sarcoptiformes	Oppiidae	CHACB811-10	JX834667
Sarcoptiformes	Oppiidae	CHACB813-10	JX837222
Sarcoptiformes	Oppiidae	CHACB814-10	JX838346
Sarcoptiformes	Oppiidae	CHACB933-10	HM907359
Sarcoptiformes	Oppiidae	CHACB934-10	HM907360
Sarcoptiformes	Oppiidae	CHACB935-10	HM907361
Sarcoptiformes	Oppiidae	CHACB936-10	HM907362
Sarcoptiformes	Oppiidae	CHACB960-10	HM907380
Sarcoptiformes	Oppiidae	CHACC202-10	HQ941528
Sarcoptiformes	Oppiidae	CHACC205-10	HQ941530
Sarcoptiformes	Oppiidae	CNPAF837-13	KM824817
Sarcoptiformes	Oppiidae	MIONB065-10	HQ575082
Sarcoptiformes	Oppiidae	MIONB076-10	HQ575090
Sarcoptiformes	Oppiidae	MIONB196-10	KR070016
Sarcoptiformes	Oppiidae	MIONB197-10	KP979127
Sarcoptiformes	Oppiidae	MIONB198-10	KR070237
Sarcoptiformes	Oppiidae	MYMCA1066-11	JX837678
Sarcoptiformes	Oppiidae	MYMCA1067-11	JX836033
Sarcoptiformes	Oppiidae	MYMCA1068-11	JX834190
Sarcoptiformes	Oppiidae	MYMCA1069-11	JX836170
Sarcoptiformes	Oppiidae	MYMCA1135-11	JX837499
Sarcoptiformes	Oppiidae	MYMCA1136-11	JX836435
Sarcoptiformes	Oppiidae	MYMCA1137-11	JX838187
Sarcoptiformes	Oppiidae	MYMCA1207-11	JX834823
Sarcoptiformes	Oppiidae	MYMCA1232-11	JX838610
Sarcoptiformes	Oppiidae	MYMCA1233-11	JX833669
Sarcoptiformes	Oppiidae	MYMCA1234-11	JX835587
Sarcoptiformes	Oppiidae	MYMCA1235-11	JX836397
Sarcoptiformes	Oppiidae	MYMCA182-11	JX835822

Sarcoptiformes	Oppiidae	MYMCA183-11	JX836606
Sarcoptiformes	Oppiidae	MYMCA239-11	JX833680
Sarcoptiformes	Oppiidae	MYMCA240-11	JX838436
Sarcoptiformes	Oppiidae	MYMCA241-11	JX835125
Sarcoptiformes	Oppiidae	MYMCA242-11	JX833928
Sarcoptiformes	Oppiidae	MYMCA243-11	JX837788
Sarcoptiformes	Oppiidae	MYMCA288-11	JX834319
Sarcoptiformes	Oppiidae	MYMCA289-11	JX836666
Sarcoptiformes	Oppiidae	MYMCA290-11	JX835488
Sarcoptiformes	Oppiidae	MYMCA329-11	JX837797
Sarcoptiformes	Oppiidae	MYMCA330-11	JX836728
Sarcoptiformes	Oppiidae	MYMCA331-11	JX834870
Sarcoptiformes	Oppiidae	MYMCA356-11	JX838297
Sarcoptiformes	Oppiidae	MYMCA357-11	JX837907
Sarcoptiformes	Oppiidae	MYMCA358-11	JX838555
Sarcoptiformes	Oppiidae	MYMCA375-11	JX835988
Sarcoptiformes	Oppiidae	MYMCA377-11	JX837523
Sarcoptiformes	Oppiidae	MYMCA378-11	JX833741
Sarcoptiformes	Oppiidae	MYMCA511-11	JX838124
Sarcoptiformes	Oppiidae	MYMCA780-11	JX837425
Sarcoptiformes	Oppiidae	MYMCA859-11	JX833966
Sarcoptiformes	Oppiidae	MYMCA895-11	JX835467
Sarcoptiformes	Oppiidae	MYMCA915-11	JX837535
Sarcoptiformes	Oppiidae	MYMCA916-11	JX837387
Sarcoptiformes	Oppiidae	MYMCA944-11	JX837059
Sarcoptiformes	Oppiidae	MYMCB060-11	JX835815
Sarcoptiformes	Oppiidae	MYMCB119-11	JX837487
Sarcoptiformes	Oppiidae	MYMCB120-11	JX837510
Sarcoptiformes	Oppiidae	MYMCB121-11	JX834196
Sarcoptiformes	Oppiidae	MYMCB248-11	JX835718
Sarcoptiformes	Oppiidae	MYMCB249-11	JX836093
Sarcoptiformes	Oppiidae	MYMCB250-11	JX834155
Sarcoptiformes	Oppiidae	MYMCB284-11	JX835409
Sarcoptiformes	Oppiidae	MYMCB285-11	JX835961
Sarcoptiformes	Oppiidae	MYMCB499-11	JX837210
Sarcoptiformes	Oppiidae	MYMCB500-11	JX837030
Sarcoptiformes	Oppiidae	MYMCB516-11	JX838718
Sarcoptiformes	Oppiidae	MYMCB517-11	JX835935
Sarcoptiformes	Oppiidae	MYMCB709-11	JX838210
Sarcoptiformes	Oppiidae	MYMCB746-11	JX835067
Sarcoptiformes	Oppiidae	MYMCB795-11	JX835030
Sarcoptiformes	Oppiidae	MYMCB825-11	JX837530
Sarcoptiformes	Oppiidae	MYMCB828-11	JX834632
Sarcoptiformes	Oppiidae	MYMCB872-11	JX835670
Sarcoptiformes	Oppiidae	MYMCC059-11	JX838059
Sarcoptiformes	Oppiidae	MYMCC060-11	JX835846
Sarcoptiformes	Oppiidae	MYMCC061-11	JX836471
Sarcoptiformes	Oppiidae	MYMCC095-11	JX837206
Sarcoptiformes	Oppiidae	MYMCC198-11	JX834645
Sarcoptiformes	Oppiidae	MYMCC199-11	JX837633
Sarcoptiformes	Oppiidae	MYMCC230-11	JX838245
Sarcoptiformes	Oppiidae	MYMCC275-11	JX836353
Sarcoptiformes	Oppiidae	MYMCC276-11	JX837187

Sarcoptiformes	Oppiidae	MYMCC361-11	JX836992
Sarcoptiformes	Oppiidae	MYMCC362-11	JX838484
Sarcoptiformes	Oppiidae	MYMCC363-11	JX835791
Sarcoptiformes	Oppiidae	MYMCC443-11	JX838602
Sarcoptiformes	Oppiidae	MYMCC444-11	JX836654
Sarcoptiformes	Oppiidae	MYMCC601-11	JX838558
Sarcoptiformes	Oppiidae	MYMCC602-11	JX835882
Sarcoptiformes	Oppiidae	MYMCC642-11	JX837635
Sarcoptiformes	Oppiidae	MYMCC643-11	JX836853
Sarcoptiformes	Oppiidae	MYMCC877-11	JX838499
Sarcoptiformes	Oppiidae	MYMCC879-11	JX837252
Sarcoptiformes	Oppiidae	MYMCC933-11	JX833670
Sarcoptiformes	Oppiidae	MYMCE020-12	JX834180
Sarcoptiformes	Oppiidae	MYMCE029-12	JX836838
Sarcoptiformes	Oppiidae	MYMCE051-12	JX835913
Sarcoptiformes	Oppiidae	MYMCE052-12	JX834201
Sarcoptiformes	Oppiidae	MYMCE092-12	JX837452
Sarcoptiformes	Oppiidae	MYMCE093-12	JX838324
Sarcoptiformes	Oppiidae	MYMCE094-12	JX838293
Sarcoptiformes	Oppiidae	MYMCE109-12	JX838070
Sarcoptiformes	Oppiidae	MYMCE110-12	JX834358
Sarcoptiformes	Oppiidae	MYMCE165-12	JX835541
Sarcoptiformes	Oppiidae	MYMCE166-12	JX836161
Sarcoptiformes	Oppiidae	MYMCE179-12	JX838666
Sarcoptiformes	Oppiidae	MYMCE210-12	JX838665
Sarcoptiformes	Oppiidae	MYMCE247-12	JX837466
Sarcoptiformes	Oppiidae	MYMCE248-12	JX838252
Sarcoptiformes	Oppiidae	MYMCE249-12	JX838274
Sarcoptiformes	Oppiidae	MYMCE294-12	JX835715
Sarcoptiformes	Oppiidae	MYMCE318-12	JX838500
Sarcoptiformes	Oppiidae	MYMCE463-12	JX836431
Sarcoptiformes	Oppiidae	MYMCE601-12	JX837547
Sarcoptiformes	Oppiidae	MYMCE629-12	JX837670
Sarcoptiformes	Oppiidae	MYMCE630-12	JX834215
Sarcoptiformes	Oppiidae	MYMCE631-12	JX835360
Sarcoptiformes	Oppiidae	MYMCE649-12	JX834511
Sarcoptiformes	Oppiidae	MYMCE650-12	JX836377
Sarcoptiformes	Oppiidae	MYMCE685-12	JX836178
Sarcoptiformes	Oppiidae	MYMCE776-12	JX837438
Sarcoptiformes	Oppiidae	MYMCE777-12	JX835273
Sarcoptiformes	Oppiidae	MYMCE778-12	JX836806
Sarcoptiformes	Oppiidae	MYMCE779-12	JX835347
Sarcoptiformes	Oppiidae	MYMCE805-12	JX838416
Sarcoptiformes	Oppiidae	MYMCE806-12	JX837985
Sarcoptiformes	Oppiidae	MYMCE807-12	JX837188
Sarcoptiformes	Oppiidae	MYMCE825-12	JX834741
Sarcoptiformes	Oppiidae	MYMCE826-12	JX836877
Sarcoptiformes	Oppiidae	MYMCE827-12	JX835429
Sarcoptiformes	Oppiidae	MYMCE876-12	JX837368
Sarcoptiformes	Oppiidae	MYMCE877-12	JX834609
Sarcoptiformes	Oppiidae	MYMCE895-12	JX833823
Sarcoptiformes	Oppiidae	MYMCE896-12	JX833940
Sarcoptiformes	Oppiidae	MYMCE897-12	JX834235

Sarcoptiformes	Oppiidae	MYMCE913-12	JX834241
Sarcoptiformes	Oppiidae	MYMCE914-12	JX835780
Sarcoptiformes	Oppiidae	MYMCE915-12	JX836217
Sarcoptiformes	Oppiidae	MYMCF018-12	JX838433
Sarcoptiformes	Oppiidae	MYMCF020-12	JX833627
Sarcoptiformes	Oppiidae	MYMCF021-12	JX834182
Sarcoptiformes	Oppiidae	MYMCF066-12	JX833797
Sarcoptiformes	Oppiidae	MYMCF067-12	JX835340
Sarcoptiformes	Oppiidae	MYMCF068-12	JX835896
Sarcoptiformes	Oppiidae	MYMCF110-12	JX837799
Sarcoptiformes	Oppiidae	MYMCF111-12	JX837509
Sarcoptiformes	Oppiidae	MYMCF112-12	JX838504
Sarcoptiformes	Oppiidae	MYMCF133-12	JX833877
Sarcoptiformes	Oppiidae	MYMCF165-12	JX837359
Sarcoptiformes	Oppiidae	MYMCF201-12	JX837577
Sarcoptiformes	Oppiidae	MYMCF267-12	JX835145
Sarcoptiformes	Oppiidae	MYMCF268-12	JX835978
Sarcoptiformes	Oppiidae	MYMCF269-12	JX836866
Sarcoptiformes	Oppiidae	MYMCF270-12	JX834525
Sarcoptiformes	Oppiidae	MYMCF328-12	JX835594
Sarcoptiformes	Oppiidae	MYMCF330-12	JX834445
Sarcoptiformes	Oppiidae	MYMCF331-12	JX837557
Sarcoptiformes	Oppiidae	MYMCF343-12	JX835671
Sarcoptiformes	Oppiidae	MYMCF367-12	JX833957
Sarcoptiformes	Oppiidae	MYMCF392-12	JX836932
Sarcoptiformes	Oppiidae	MYMCF393-12	JX838146
Sarcoptiformes	Oppiidae	MYMCF394-12	JX836201
Sarcoptiformes	Oppiidae	MYMCF395-12	JX836279
Sarcoptiformes	Oppiidae	MYMCF591-12	JX834503
Sarcoptiformes	Oppiidae	MYMCF594-12	JX837322
Sarcoptiformes	Oppiidae	MYMCF642-12	JX835617
Sarcoptiformes	Oppiidae	MYMCF644-12	JX835818
Sarcoptiformes	Oppiidae	MYMCF705-12	JX834158
Sarcoptiformes	Oppiidae	MYMCF728-12	JX838441
Sarcoptiformes	Oppiidae	MYMCF730-12	JX837303
Sarcoptiformes	Oppiidae	MYMCF731-12	JX836133
Sarcoptiformes	Oppiidae	MYMCF745-12	JX837099
Sarcoptiformes	Oppiidae	MYMCF746-12	JX838489
Sarcoptiformes	Oppiidae	MYMCF747-12	JX834605
Sarcoptiformes	Oppiidae	MYMCF748-12	JX837858
Sarcoptiformes	Oppiidae	MYMCF766-12	JX835252
Sarcoptiformes	Oppiidae	MYMCF767-12	JX835335
Sarcoptiformes	Oppiidae	MYMCF768-12	JX836405
Sarcoptiformes	Oppiidae	MYMCF808-12	JX837011
Sarcoptiformes	Oppiidae	MYMCF844-12	JX835714
Sarcoptiformes	Oppiidae	MYMCF845-12	JX835258
Sarcoptiformes	Oppiidae	MYMCF846-12	JX834038
Sarcoptiformes	Oppiidae	MYMCF914-12	JX838387
Sarcoptiformes	Oppiidae	MYMCF915-12	JX836577
Sarcoptiformes	Oppiidae	MYMCF940-12	JX838615
Sarcoptiformes	Oppiidae	MYMCF941-12	JX837938
Sarcoptiformes	Oppiidae	MYMCF942-12	JX837628
Sarcoptiformes	Oppiidae	MYMCG039-12	JX834562

Sarcoptiformes	Oppiidae	MYMCG040-12	JX837185
Sarcoptiformes	Oppiidae	MYMCG041-12	JX835743
Sarcoptiformes	Oppiidae	MYMCG075-12	JX836023
Sarcoptiformes	Oppiidae	MYMCG100-12	JX835071
Sarcoptiformes	Oppiidae	MYMCG139-12	JX836687
Sarcoptiformes	Oppiidae	MYMCG141-12	JX835265
Sarcoptiformes	Oppiidae	MYMCG142-12	JX833808
Sarcoptiformes	Oppiidae	MYMCG143-12	JX837798
Sarcoptiformes	Oppiidae	MYMCG168-12	JX836646
Sarcoptiformes	Oppiidae	MYMCG169-12	JX834755
Sarcoptiformes	Oppiidae	MYMCG170-12	JX835558
Sarcoptiformes	Oppiidae	MYMCG228-12	JX834217
Sarcoptiformes	Oppiidae	MYMCG238-12	JX833795
Sarcoptiformes	Oppiidae	MYMCG240-12	JX834419
Sarcoptiformes	Oppiidae	MYMCG241-12	JX838691
Sarcoptiformes	Oppiidae	MYMCG242-12	JX838036
Sarcoptiformes	Oppiidae	MYMCG261-12	JX834582
Sarcoptiformes	Oppiidae	MYMCG262-12	JX836510
Sarcoptiformes	Oppiidae	MYMCG340-12	JX835883
Sarcoptiformes	Oppiidae	MYMCG341-12	JX837600
Sarcoptiformes	Oppiidae	MYMCG342-12	JX837636
Sarcoptiformes	Oppiidae	MYMCG440-12	JX834884
Sarcoptiformes	Oppiidae	MYMCG441-12	JX833899
Sarcoptiformes	Oppiidae	MYMCG442-12	JX834763
Sarcoptiformes	Oppiidae	MYMCG443-12	JX835118
Sarcoptiformes	Oppiidae	MYMCG444-12	JX837840
Sarcoptiformes	Oppiidae	MYMCG481-12	JX834635
Sarcoptiformes	Oppiidae	MYMCG513-12	JX837893
Sarcoptiformes	Oppiidae	MYMCG514-12	JX835295
Sarcoptiformes	Oppiidae	MYMCG515-12	JX838104
Sarcoptiformes	Oppiidae	MYMCG516-12	JX837817
Sarcoptiformes	Oppiidae	MYMCG517-12	JX838212
Sarcoptiformes	Oppiidae	MYMCG552-12	JX836856
Sarcoptiformes	Oppiidae	MYMCG553-12	JX837055
Sarcoptiformes	Oppiidae	MYMCG554-12	JX837602
Sarcoptiformes	Oppiidae	MYMCG555-12	JX833748
Sarcoptiformes	Oppiidae	MYMCG586-12	JX836391
Sarcoptiformes	Oppiidae	MYMCG587-12	JX837774
Sarcoptiformes	Oppiidae	MYMCG588-12	JX836488
Sarcoptiformes	Oppiidae	MYMCG589-12	JX837409
Sarcoptiformes	Oppiidae	MYMCG606-12	JX833687
Sarcoptiformes	Oppiidae	MYMCG663-12	JX838592
Sarcoptiformes	Oppiidae	MYMCG664-12	JX837548
Sarcoptiformes	Oppiidae	MYMCG665-12	JX834338
Sarcoptiformes	Oppiidae	MYTMC127-09	KR069394
Sarcoptiformes	Oppiidae	MYTMC163-09	HQ966244
Sarcoptiformes	Oppiidae	SSEIA2443-13	KM826171
Sarcoptiformes	Oppiidae	SSEIB8196-13	KM834987
Sarcoptiformes	Oppiidae	SSPAA8397-13	KM828351
Sarcoptiformes	Oppiidae	SSPAA9045-13	KM838827
Sarcoptiformes	Oppiidae	SSPAA9105-13	KM832571
Sarcoptiformes	Oppiidae	SSPAA9117-13	KM836563
Sarcoptiformes	Oppiidae	SSPAA9146-13	KM829817

Sarcoptiformes	Oppiidae		SSPAA9160-13	KM825594
Sarcoptiformes	Oppiidae		SSPAB9355-13	KM830287
Sarcoptiformes	Oppiidae		SSPAB9377-13	KM831034
Sarcoptiformes	Oppiidae		SSPAB9408-13	KM825165
Sarcoptiformes	Oppiidae		SSPAB9457-13	KM830855
Sarcoptiformes	Oppiidae		SSPAB9477-13	KM829121
Sarcoptiformes	Oppiidae		SSPAB9533-13	KM830348
Sarcoptiformes	Oribatellidae	*Oribatella*	CHACA1024-10	HM907081
Sarcoptiformes	Oribatellidae	*Oribatella*	CHACA1025-10	HM907082
Sarcoptiformes	Oribatellidae	*Oribatella*	CHACA1119-10	JX837692
Sarcoptiformes	Oribatellidae	*Oribatella*	CHACC194-10	HQ941520
Sarcoptiformes	Oribatellidae	*Oribatella*	CHACC195-10	HQ941521
Sarcoptiformes	Oribatellidae	*Oribatella*	CHACC196-10	HQ941522
Sarcoptiformes	Oribatellidae	*Oribatella*	MYMCA731-11	JX835035
Sarcoptiformes	Oribatellidae	*Oribatella*	MYMCA732-11	JX837068
Sarcoptiformes	Oribatellidae	*Oribatella*	MYMCA733-11	JX838099
Sarcoptiformes	Oribatellidae	*Oribatella*	MYMCB167-11	JX835443
Sarcoptiformes	Oribatellidae	*Oribatella*	MYMCE251-12	JX835207
Sarcoptiformes	Oribatellidae	*Oribatella*	MYMCE295-12	JX836570
Sarcoptiformes	Oribatellidae	*Oribatella*	MYMCE296-12	JX835699
Sarcoptiformes	Oribatellidae	*Oribatella*	MYMCE333-12	JX835556
Sarcoptiformes	Oribatellidae	*Oribatella*	MYMCE425-12	JX834189
Sarcoptiformes	Oribatellidae	*Oribatella*	MYMCF327-12	JX837489
Sarcoptiformes	Oribatellidae	*Oribatella*	MYMCF520-12	JX838323
Sarcoptiformes	Oribatellidae	*Oribatella*	MYMCF521-12	JX836482
Sarcoptiformes	Oribatellidae	*Oribatella*	MYMCF545-12	JX836616
Sarcoptiformes	Oribatellidae	*Oribatella*	MYMCF681-12	JX836416
Sarcoptiformes	Oribatellidae	*Oribatella*	MYMCF894-12	JX835843
Sarcoptiformes	Oribatellidae	*Oribatella*	MYMCF895-12	JX833820
Sarcoptiformes	Oribatellidae	*Oribatella*	MYMCF896-12	JX833888
Sarcoptiformes	Oribatellidae	*Oribatella*	MYMCG121-12	JX837016
Sarcoptiformes	Oribatellidae	*Oribatella*	MYMCG350-12	JX834665
Sarcoptiformes	Oribatellidae	*Oribatella*	MYMCG351-12	JX834481
Sarcoptiformes	Oribatellidae	*Oribatella*	MYMCG353-12	JX836563
Sarcoptiformes	Oribatellidae	*Oribatella*	SSBAC3370-12	KM830010
Sarcoptiformes	Oribatellidae	*Oribatella*	SSJAA2035-13	KM824060
Sarcoptiformes	Oribatellidae		SSEIA3023-13	KM839758
Sarcoptiformes	Oribatellidae		SSJAD2905-13	KM839217
Sarcoptiformes	Oribatellidae		SSJAD3339-13	KM825769
Sarcoptiformes	Oribatellidae		SSPAC12272-13	KM832979
Sarcoptiformes	Oribatellidae		SSPAC12469-13	KM835060
Sarcoptiformes	Oribatellidae		SSPAC12479-13	KM827581
Sarcoptiformes	Oribatulidae	*Liebstadia*	CNPAD566-13	KM826838
Sarcoptiformes	Oribatulidae	*Liebstadia*	CNPAG381-13	KM839683
Sarcoptiformes	Oribatulidae	*Liebstadia*	MYMCE691-12	JX834092
Sarcoptiformes	Oribatulidae	*Liebstadia similis*	CHACA007-08	JX836057
Sarcoptiformes	Oribatulidae	*Liebstadia similis*	CNPAF918-13	KM835891
Sarcoptiformes	Oribatulidae	*Liebstadia similis*	CNPAG379-13	KM833442
Sarcoptiformes	Oribatulidae	*Liebstadia similis*	MYMCC745-11	JX835640
Sarcoptiformes	Oribatulidae	*Liebstadia similis*	MYMCE193-12	JX838656
Sarcoptiformes	Oribatulidae	*Liebstadia similis*	MYMCE946-12	JX836552
Sarcoptiformes	Oribatulidae	*Liebstadia similis*	MYMCF151-12	JX835024
Sarcoptiformes	Oribatulidae	*Liebstadia similis*	MYMCF584-12	JX834997

Sarcoptiformes	Oribatulidae	*Liebstadia similis*	MYMCF709-12	JX835385
Sarcoptiformes	Oribatulidae	*Liebstadia similis*	MYMCG450-12	JX833774
Sarcoptiformes	Oribatulidae	*Lucoppia*	SSPAA7985-13	KM826414
Sarcoptiformes	Oribatulidae	*Lucoppia*	SSPAA8003-13	KM827755
Sarcoptiformes	Oribatulidae	*Oribatula*	CHACA383-09	JX833640
Sarcoptiformes	Oribatulidae	*Oribatula*	CHACA506-09	JX836595
Sarcoptiformes	Oribatulidae	*Oribatula*	CNJAB1012-12	KM837721
Sarcoptiformes	Oribatulidae	*Oribatula*	CNJAB1017-12	KM826520
Sarcoptiformes	Oribatulidae	*Oribatula*	CNJAB1041-12	KM840101
Sarcoptiformes	Oribatulidae	*Oribatula*	CNJAB1046-12	KM824440
Sarcoptiformes	Oribatulidae	*Oribatula*	CNJAC1436-12	KM839412
Sarcoptiformes	Oribatulidae	*Oribatula*	CNJAC1506-12	KM839236
Sarcoptiformes	Oribatulidae	*Oribatula*	CNJAC1593-12	KM829278
Sarcoptiformes	Oribatulidae	*Oribatula*	CNJAD1857-12	KM833722
Sarcoptiformes	Oribatulidae	*Oribatula*	CNJAD2285-12	KM840102
Sarcoptiformes	Oribatulidae	*Oribatula*	CNJAD2305-12	KM825281
Sarcoptiformes	Oribatulidae	*Oribatula*	CNJAD2343-12	KM835738
Sarcoptiformes	Oribatulidae	*Oribatula*	CNJAE1142-12	KM827975
Sarcoptiformes	Oribatulidae	*Oribatula*	CNJAF1965-12	KM830125
Sarcoptiformes	Oribatulidae	*Oribatula*	CNJAF1966-12	KM831117
Sarcoptiformes	Oribatulidae	*Oribatula*	CNJAF1983-12	KM828156
Sarcoptiformes	Oribatulidae	*Oribatula*	CNJAG1829-12	KM826190
Sarcoptiformes	Oribatulidae	*Oribatula*	CNJAI834-12	KM829886
Sarcoptiformes	Oribatulidae	*Oribatula*	CNJAJ693-12	KM824754
Sarcoptiformes	Oribatulidae	*Oribatula*	MYMCA1036-11	JX836064
Sarcoptiformes	Oribatulidae	*Oribatula*	MYMCA1037-11	JX836081
Sarcoptiformes	Oribatulidae	*Oribatula*	MYMCA1044-11	JX838271
Sarcoptiformes	Oribatulidae	*Oribatula*	MYMCA1339-11	JX835345
Sarcoptiformes	Oribatulidae	*Oribatula*	MYMCA779-11	JX835973
Sarcoptiformes	Oribatulidae	*Oribatula*	MYMCE686-12	JX834175
Sarcoptiformes	Oribatulidae	*Oribatula*	MYMCE901-12	JX838166
Sarcoptiformes	Oribatulidae	*Oribatula*	MYMCF266-12	JX834487
Sarcoptiformes	Oribatulidae	*Oribatula*	MYMCF501-12	JX837594
Sarcoptiformes	Oribatulidae	*Oribatula*	MYMCF714-12	JX836759
Sarcoptiformes	Oribatulidae	*Oribatula*	MYMCF715-12	JX836242
Sarcoptiformes	Oribatulidae	*Oribatula*	SMTPB669-13	KP979336
Sarcoptiformes	Oribatulidae	*Oribatula*	SSPAA2222-13	KM825273
Sarcoptiformes	Oribatulidae	*Oribatula*	SSWLD8202-13	KM835050
Sarcoptiformes	Oribatulidae	*Oribatula tibialis*	GBCH1954-07	DQ381226
Sarcoptiformes	Oribatulidae	*Phauloppia boletorum*	CNRMB163-12	KM834644
Sarcoptiformes	Oribatulidae	*Phauloppia boletorum*	SSEIA046-13	KM833112
Sarcoptiformes	Oribatulidae	*Phauloppia boletorum*	SSEIB4204-13	KM831310
Sarcoptiformes	Oribatulidae	*Phauloppia boletorum*	SSEIB4207-13	KM825563
Sarcoptiformes	Oribatulidae	*Phauloppia boletorum*	SSEIB4212-13	KM828590
Sarcoptiformes	Oribatulidae	*Phauloppia boletorum*	SSEIB4222-13	KM840661
Sarcoptiformes	Oribatulidae	*Phauloppia boletorum*	SSEIB4223-13	KM836771
Sarcoptiformes	Oribatulidae	*Phauloppia boletorum*	SSEIB4230-13	KM835174
Sarcoptiformes	Oribatulidae	*Phauloppia boletorum*	SSEIB4231-13	KM824167
Sarcoptiformes	Oribatulidae	*Phauloppia boletorum*	SSEIB4233-13	KM839963
Sarcoptiformes	Oribatulidae	*Phauloppia boletorum*	SSEIB4238-13	KM829628
Sarcoptiformes	Oribatulidae	*Phauloppia boletorum*	SSEIB4244-13	KM825907
Sarcoptiformes	Oribatulidae	*Phauloppia boletorum*	SSEIB4249-13	KM840242
Sarcoptiformes	Oribatulidae	*Phauloppia boletorum*	SSEIB4545-13	KM834998

Sarcoptiformes	Oribatulidae	*Phauloppia boletorum*	SSEIB5408-13	KM831202
Sarcoptiformes	Oribatulidae	*Phauloppia boletorum*	SSEIB5416-13	KM837529
Sarcoptiformes	Oribatulidae	*Phauloppia boletorum*	SSEIB5789-13	KM839673
Sarcoptiformes	Oribatulidae	*Phauloppia boletorum*	SSJAB1018-13	KM829906
Sarcoptiformes	Oribatulidae	*Zygoribatula*	MYMCA1021-11	JX835824
Sarcoptiformes	Oribatulidae	*Zygoribatula*	MYMCA1356-11	JX837970
Sarcoptiformes	Oribatulidae	*Zygoribatula*	MYMCA1396-11	JX838148
Sarcoptiformes	Oribatulidae	*Zygoribatula*	MYMCA1421-11	JX836056
Sarcoptiformes	Oribatulidae	*Zygoribatula*	MYMCA1517-11	JX837107
Sarcoptiformes	Oribatulidae	*Zygoribatula*	MYMCA171-11	JX835678
Sarcoptiformes	Oribatulidae	*Zygoribatula*	MYMCA600-11	JX836829
Sarcoptiformes	Oribatulidae	*Zygoribatula*	MYMCA630-11	JX835491
Sarcoptiformes	Oribatulidae	*Zygoribatula*	MYMCA649-11	JX836467
Sarcoptiformes	Oribatulidae	*Zygoribatula*	MYMCA756-11	JX834989
Sarcoptiformes	Oribatulidae	*Zygoribatula*	MYMCA987-11	JX836517
Sarcoptiformes	Oribatulidae	*Zygoribatula*	MYMCA988-11	JX837340
Sarcoptiformes	Oribatulidae	*Zygoribatula*	MYMCA989-11	JX833896
Sarcoptiformes	Oribatulidae	*Zygoribatula bulanovae*	CHACA1083-10	HM907124
Sarcoptiformes	Oribatulidae	*Zygoribatula bulanovae*	CHACB016-10	HQ558337
Sarcoptiformes	Oribatulidae	*Zygoribatula bulanovae*	CHACB017-10	HQ558338
Sarcoptiformes	Oribatulidae	*Zygoribatula bulanovae*	CHACB489-10	HQ558624
Sarcoptiformes	Oribatulidae	*Zygoribatula bulanovae*	CHACB490-10	HQ558625
Sarcoptiformes	Oribatulidae	*Zygoribatula bulanovae*	CHACB504-10	HQ558634
Sarcoptiformes	Oribatulidae	*Zygoribatula bulanovae*	CHACB726-10	HQ558775
Sarcoptiformes	Oribatulidae	*Zygoribatula bulanovae*	MYMCA628-11	JX835921
Sarcoptiformes	Oribatulidae	*Zygoribatula bulanovae*	MYMCA629-11	JX837026
Sarcoptiformes	Oribatulidae	*Zygoribatula bulanovae*	MYMCF416-12	JX836805
Sarcoptiformes	Oribatulidae	*Zygoribatula bulanovae*	MYMCG303-12	JX837657
Sarcoptiformes	Oribatulidae	*Zygoribatula bulanovae*	MYMCG304-12	JX838616
Sarcoptiformes	Oribatulidae	*Zygoribatula bulanovae*	MYMCG635-12	JX836216
Sarcoptiformes	Oribatulidae		CHACC093-10	HM907482
Sarcoptiformes	Oribatulidae		CHACC103-10	JX834464
Sarcoptiformes	Oribatulidae		CNBPC221-12	KM827986
Sarcoptiformes	Oribatulidae		CNBPK330-13	KM828749
Sarcoptiformes	Oribatulidae		CNBPL238-13	KM833853
Sarcoptiformes	Oribatulidae		CNBPL251-13	KM840771
Sarcoptiformes	Oribatulidae		CNBPL288-13	KM840141
Sarcoptiformes	Oribatulidae		CNBPL289-13	KM830856
Sarcoptiformes	Oribatulidae		CNBPN279-13	KM832237
Sarcoptiformes	Oribatulidae		CNBPN280-13	KM834673
Sarcoptiformes	Oribatulidae		CNBPN283-13	KM835017
Sarcoptiformes	Oribatulidae		CNGIB592-12	KM829472
Sarcoptiformes	Oribatulidae		CNGLA019-13	KM830328
Sarcoptiformes	Oribatulidae		CNGLA020-13	KM831919
Sarcoptiformes	Oribatulidae		CNGLD013-13	KM834198
Sarcoptiformes	Oribatulidae		CNGLD014-13	KM824094
Sarcoptiformes	Oribatulidae		CNPAD549-13	KM832416
Sarcoptiformes	Oribatulidae		CNPCA042-13	KM827625
Sarcoptiformes	Oribatulidae		CNPCA060-13	KM828491
Sarcoptiformes	Oribatulidae		CNPCB077-13	KM832001
Sarcoptiformes	Oribatulidae		CNPCB079-13	KM833134
Sarcoptiformes	Oribatulidae		CNPCB083-13	KM824288
Sarcoptiformes	Oribatulidae		CNPCC059-13	KM839757

Sarcoptiformes	Oribatulidae	CNPCD078-13	KM839654
Sarcoptiformes	Oribatulidae	CNPCD080-13	KM836855
Sarcoptiformes	Oribatulidae	CNPCD082-13	KM832351
Sarcoptiformes	Oribatulidae	CNPCD091-13	KM837457
Sarcoptiformes	Oribatulidae	CNPCD122-13	KM826779
Sarcoptiformes	Oribatulidae	CNPCD123-13	KM840400
Sarcoptiformes	Oribatulidae	CNPCD135-13	KM838547
Sarcoptiformes	Oribatulidae	CNPCD157-13	KM832293
Sarcoptiformes	Oribatulidae	CNPCE096-13	KM833957
Sarcoptiformes	Oribatulidae	CNPCE098-13	KM830956
Sarcoptiformes	Oribatulidae	CNPCE112-13	KM825209
Sarcoptiformes	Oribatulidae	CNPCE132-13	KM834740
Sarcoptiformes	Oribatulidae	CNPCE139-13	KM833408
Sarcoptiformes	Oribatulidae	CNPCE141-13	KM827472
Sarcoptiformes	Oribatulidae	CNPCE143-13	KM835130
Sarcoptiformes	Oribatulidae	CNPCE146-13	KM834611
Sarcoptiformes	Oribatulidae	CNPCE150-13	KM840772
Sarcoptiformes	Oribatulidae	CNPCE154-13	KM840028
Sarcoptiformes	Oribatulidae	CNPCE157-13	KM839129
Sarcoptiformes	Oribatulidae	CNPCF085-13	KM837808
Sarcoptiformes	Oribatulidae	CNPCF087-13	KM828957
Sarcoptiformes	Oribatulidae	CNPCF096-13	KM833296
Sarcoptiformes	Oribatulidae	CNPCF099-13	KM837391
Sarcoptiformes	Oribatulidae	CNPCF102-13	KM825901
Sarcoptiformes	Oribatulidae	CNPCF121-13	KM837838
Sarcoptiformes	Oribatulidae	CNPCF128-13	KM828659
Sarcoptiformes	Oribatulidae	CNPCF131-13	KM831228
Sarcoptiformes	Oribatulidae	CNPCG046-13	KM833409
Sarcoptiformes	Oribatulidae	CNPPG819-12	KJ167788
Sarcoptiformes	Oribatulidae	CNPPH899-12	KJ444301
Sarcoptiformes	Oribatulidae	CNSLB473-12	KM828062
Sarcoptiformes	Oribatulidae	CNSLB475-12	KM831128
Sarcoptiformes	Oribatulidae	CNSLB476-12	KM825100
Sarcoptiformes	Oribatulidae	CNSLB477-12	KM832529
Sarcoptiformes	Oribatulidae	CNSLB479-12	KM825518
Sarcoptiformes	Oribatulidae	CNSLC549-12	KM827841
Sarcoptiformes	Oribatulidae	CNSLC550-12	KM839158
Sarcoptiformes	Oribatulidae	CNSLC557-12	KM830957
Sarcoptiformes	Oribatulidae	CNSLD652-12	KM838669
Sarcoptiformes	Oribatulidae	CNSLD675-12	KM828959
Sarcoptiformes	Oribatulidae	CNSLE506-12	KM826330
Sarcoptiformes	Oribatulidae	CNSLF645-12	KM826299
Sarcoptiformes	Oribatulidae	CNSLF656-12	KM837006
Sarcoptiformes	Oribatulidae	CNSLF658-12	KM833641
Sarcoptiformes	Oribatulidae	CNSLF663-12	KM834626
Sarcoptiformes	Oribatulidae	CNSLH086-12	KM828044
Sarcoptiformes	Oribatulidae	CNSLI064-12	KM838784
Sarcoptiformes	Oribatulidae	CNSLJ026-12	KM832358
Sarcoptiformes	Oribatulidae	CNSLJ027-12	KM828018
Sarcoptiformes	Oribatulidae	CNSLJ427-12	KM840380
Sarcoptiformes	Oribatulidae	SMTPB10636-13	KR069764
Sarcoptiformes	Oribatulidae	SMTPB10813-13	KR069624
Sarcoptiformes	Oribatulidae	SMTPB14243-13	KR070358

Sarcoptiformes	Oribatulidae		SMTPB14244-13	KR069682
Sarcoptiformes	Oribatulidae		SMTPB14245-13	KR069286
Sarcoptiformes	Oribatulidae		SMTPB14246-13	KR069321
Sarcoptiformes	Oribatulidae		SMTPB14247-13	KR069318
Sarcoptiformes	Oribatulidae		SMTPB14249-13	KR069563
Sarcoptiformes	Oribatulidae		SMTPB14250-13	KR070036
Sarcoptiformes	Oribatulidae		SMTPB1473-13	KR069525
Sarcoptiformes	Oribatulidae		SMTPB19761-13	KR069995
Sarcoptiformes	Oribatulidae		SMTPB19762-13	KR069870
Sarcoptiformes	Oribatulidae		SMTPB19763-13	KR069827
Sarcoptiformes	Oribatulidae		SMTPB19764-13	KR069853
Sarcoptiformes	Oribatulidae		SMTPB19765-13	KP979184
Sarcoptiformes	Oribatulidae		SMTPB19767-13	KR069332
Sarcoptiformes	Oribatulidae		SMTPB3362-13	KP979321
Sarcoptiformes	Oribatulidae		SMTPB3368-13	KR069221
Sarcoptiformes	Oribatulidae		SMTPB5824-13	KR069902
Sarcoptiformes	Oribatulidae		SMTPB7090-13	KR069209
Sarcoptiformes	Oribatulidae		SMTPB8377-13	KR069383
Sarcoptiformes	Oribatulidae		SSWLA031-13	KM830510
Sarcoptiformes	Oribatulidae		SSWLA5287-13	KM834836
Sarcoptiformes	Oribatulidae		SSWLA5310-13	KM835994
Sarcoptiformes	Oribatulidae		SSWLA5340-13	KM824068
Sarcoptiformes	Oribatulidae		SSWLA675-13	KM836017
Sarcoptiformes	Oribatulidae		SSWLD146-13	KM835413
Sarcoptiformes	Oribatulidae		SSWLD2914-13	KM824271
Sarcoptiformes	Oribatulidae		SSWLD2967-13	KM825906
Sarcoptiformes	Oribatulidae		SSWLD2972-13	KM834256
Sarcoptiformes	Oribatulidae		SSWLD8131-13	KM825017
Sarcoptiformes	Oribatulidae		SSWLD8148-13	KM835450
Sarcoptiformes	Oribatulidae		SSWLD8191-13	KM836868
Sarcoptiformes	Oribatulidae		SSWLD8200-13	KM836859
Sarcoptiformes	Oribatulidae		SSWLD8213-13	KM829231
Sarcoptiformes	Oribatulidae		SSWLD8237-13	KM833157
Sarcoptiformes	Oribatulidae		SSWLD8255-13	KM827731
Sarcoptiformes	Oribatulidae		SSWLD8263-13	KM832271
Sarcoptiformes	Oribatulidae		SSWLD8264-13	KM832946
Sarcoptiformes	Oribatulidae		SSWLD8266-13	KM828424
Sarcoptiformes	Oribatulidae		SSWLD8279-13	KM827813
Sarcoptiformes	Oribatulidae		SSWLD8304-13	KM827920
Sarcoptiformes	Oribatulidae		SSWLD8325-13	KM824379
Sarcoptiformes	Oribatulidae		SSWLD8327-13	KM824171
Sarcoptiformes	Oribatulidae		SSWLD8348-13	KM831694
Sarcoptiformes	Oribatulidae		SSWLD8365-13	KM838606
Sarcoptiformes	Oribotritiidae	*Protoribotritia*	SSGBB1898-14	KR070695
Sarcoptiformes	Oribotritiidae	*Protoribotritia*	SSJAD3372-13	KM832971
Sarcoptiformes	Oripodidae		CNBPB228-12	KM828056
Sarcoptiformes	Oripodidae		CNBPB238-12	KM829165
Sarcoptiformes	Oripodidae		CNBPB528-12	KM830640
Sarcoptiformes	Oripodidae		CNBPB530-12	KM830419
Sarcoptiformes	Oripodidae		CNBPB539-12	KM836669
Sarcoptiformes	Oripodidae		CNBPB540-12	KM838991
Sarcoptiformes	Oripodidae		CNBPC211-12	KM838720
Sarcoptiformes	Oripodidae		CNBPC219-12	KM833899

Sarcoptiformes	Oripodidae		CNBPC227-12	KM834821
Sarcoptiformes	Oripodidae		CNBPC231-12	KM825158
Sarcoptiformes	Oripodidae		CNBPD587-12	KM828842
Sarcoptiformes	Oripodidae		CNBPD590-12	KM836000
Sarcoptiformes	Oripodidae		CNBPD597-12	KM834390
Sarcoptiformes	Oripodidae		CNBPE669-12	KM832964
Sarcoptiformes	Oripodidae		CNBPJ298-13	KM840005
Sarcoptiformes	Oripodidae		CNBPK322-13	KM830006
Sarcoptiformes	Oripodidae		CNBPK323-13	KM824409
Sarcoptiformes	Oripodidae		CNBPK328-13	KM828606
Sarcoptiformes	Oripodidae		CNBPK329-13	KM833117
Sarcoptiformes	Oripodidae		CNBPK337-13	KM835273
Sarcoptiformes	Oripodidae		CNBPL237-13	KM838762
Sarcoptiformes	Oripodidae		CNBPL240-13	KM838686
Sarcoptiformes	Oripodidae		CNBPL241-13	KM830050
Sarcoptiformes	Oripodidae		CNBPL247-13	KM826312
Sarcoptiformes	Oripodidae		CNBPL268-13	KM837010
Sarcoptiformes	Oripodidae		CNBPL277-13	KM830577
Sarcoptiformes	Oripodidae		CNBPL283-13	KM836766
Sarcoptiformes	Oripodidae		CNBPL285-13	KM838752
Sarcoptiformes	Oripodidae		CNBPL286-13	KM825045
Sarcoptiformes	Oripodidae		CNBPL293-13	KM832641
Sarcoptiformes	Oripodidae		CNPPC1938-12	KJ086999
Sarcoptiformes	Oripodidae		CNSLB469-12	KM825179
Sarcoptiformes	Oripodidae		CNSLB471-12	KM838361
Sarcoptiformes	Oripodidae		JSJUL2541-12	KR069442
Sarcoptiformes	Oripodidae		JSMAY1596-12	KR069893
Sarcoptiformes	Oripodidae		JSSEP1136-12	KR069787
Sarcoptiformes	Oripodidae		JSSEP1159-12	KR069848
Sarcoptiformes	Oripodidae		SMTPB20118-13	KP979251
Sarcoptiformes	Oripodidae		SMTPB9978-13	KP979230
Sarcoptiformes	Parakalummidae	*Neoribates*	SSEIB4540-13	KM832449
Sarcoptiformes	Parakalummidae	*Neoribates*	SSEIB4568-13	KM830978
Sarcoptiformes	Parakalummidae	*Neoribates*	SSPAC12256-13	KM826721
Sarcoptiformes	Parakalummidae	*Neoribates aurantiacus*	CHACA369-08	JX837225
Sarcoptiformes	Parakalummidae	*Neoribates aurantiacus*	MYMCA1150-11	JX834973
Sarcoptiformes	Parakalummidae	*Neoribates aurantiacus*	MYMCB191-11	JX838645
Sarcoptiformes	Parakalummidae	*Neoribates aurantiacus*	MYMCE924-12	JX837801
Sarcoptiformes	Parakalummidae	*Neoribates aurantiacus*	MYMCG618-12	JX834457
Sarcoptiformes	Parakalummidae		CNBRM780-14	KR070111
Sarcoptiformes	Parakalummidae		CNFDB289-14	KR069210
Sarcoptiformes	Parakalummidae		CNFDC384-14	KR069360
Sarcoptiformes	Parakalummidae		CNFDD887-14	KR070402
Sarcoptiformes	Parakalummidae		CNFDE1458-14	KR070110
Sarcoptiformes	Parakalummidae		CNFDK176-14	KR070100
Sarcoptiformes	Parakalummidae		CNFDM514-14	KR070534
Sarcoptiformes	Parakalummidae		CNFDO1003-14	KR069362
Sarcoptiformes	Parakalummidae		CNKOM757-14	KR070381
Sarcoptiformes	Parakalummidae		CNLMC848-14	KR070629
Sarcoptiformes	Parakalummidae		MYMCE597-12	JX834397
Sarcoptiformes	Parakalummidae		MYMCE598-12	JX835664
Sarcoptiformes	Parakalummidae		MYMCE599-12	JX834066
Sarcoptiformes	Parakalummidae		SSEID1707-13	KM829309

Sarcoptiformes	Passalozetidae	*Bipassalozetes*		MYMCB598-11	JX835874
Sarcoptiformes	Peloppiidae	*Ceratoppia*		ARCN013-10	KR069807
Sarcoptiformes	Peloppiidae	*Ceratoppia*		SSBRA2306-14	KR070576
Sarcoptiformes	Peloppiidae	*Ceratoppia*		SSBRA2311-14	KR069974
Sarcoptiformes	Peloppiidae	*Ceratoppia*		SSBRA2320-14	KR069607
Sarcoptiformes	Peloppiidae	*Ceratoppia*		SSBRA274-14	KR069348
Sarcoptiformes	Peloppiidae	*Ceratoppia*		SSBRA2786-14	KR070663
Sarcoptiformes	Peloppiidae	*Ceratoppia*		SSBRA3256-14	KR069664
Sarcoptiformes	Peloppiidae	*Ceratoppia*		CNGID224-12	KM832968
Sarcoptiformes	Peloppiidae	*Ceratoppia*		CNGIE401-12	KM835992
Sarcoptiformes	Peloppiidae	*Ceratoppia*		CNKJF1948-14	KR069711
Sarcoptiformes	Peloppiidae	*Ceratoppia*		CNKJF1951-14	KR070252
Sarcoptiformes	Peloppiidae	*Ceratoppia*		CNKJF1960-14	KR070447
Sarcoptiformes	Peloppiidae	*Ceratoppia*		CNKJM2461-14	KR069463
Sarcoptiformes	Peloppiidae	*Ceratoppia*		CNKJM2475-14	KR070451
Sarcoptiformes	Peloppiidae	*Ceratoppia*		CNKJM2477-14	KR070653
Sarcoptiformes	Peloppiidae	*Ceratoppia*		CNKJM2528-14	KR070344
Sarcoptiformes	Peloppiidae	*Ceratoppia*		CNKJO856-14	KR069691
Sarcoptiformes	Peloppiidae	*Ceratoppia*		CNKJO860-14	KR069369
Sarcoptiformes	Peloppiidae	*Ceratoppia*		CNKJP933-14	KR070431
Sarcoptiformes	Peloppiidae	*Ceratoppia*		CNKJP937-14	KR070407
Sarcoptiformes	Peloppiidae	*Ceratoppia*		CNKJP938-14	KR069304
Sarcoptiformes	Peloppiidae	*Ceratoppia*		CNKJP960-14	KR069843
Sarcoptiformes	Peloppiidae	*Ceratoppia*		CNPCA040-13	KM824734
Sarcoptiformes	Peloppiidae	*Ceratoppia*		CNPCB080-13	KM826105
Sarcoptiformes	Peloppiidae	*Ceratoppia*		CNPCB081-13	KM826076
Sarcoptiformes	Peloppiidae	*Ceratoppia*		CNPCB088-13	KM839733
Sarcoptiformes	Peloppiidae	*Ceratoppia*		CNPCB089-13	KM825112
Sarcoptiformes	Peloppiidae	*Ceratoppia*		CNPCC055-13	KM834468
Sarcoptiformes	Peloppiidae	*Ceratoppia*		CNPCC058-13	KM829931
Sarcoptiformes	Peloppiidae	*Ceratoppia*		CNPCC061-13	KM837574
Sarcoptiformes	Peloppiidae	*Ceratoppia*		CNPCC064-13	KM834073
Sarcoptiformes	Peloppiidae	*Ceratoppia*		CNPCD070-13	KM834614
Sarcoptiformes	Peloppiidae	*Ceratoppia*		CNPCD071-13	KM838662
Sarcoptiformes	Peloppiidae	*Ceratoppia*		CNPCD073-13	KM829145
Sarcoptiformes	Peloppiidae	*Ceratoppia*		CNPCD076-13	KM839449
Sarcoptiformes	Peloppiidae	*Ceratoppia*		CNPCD093-13	KM827579
Sarcoptiformes	Peloppiidae	*Ceratoppia*		CNPCD095-13	KM828728
Sarcoptiformes	Peloppiidae	*Ceratoppia*		CNPCD096-13	KM827294
Sarcoptiformes	Peloppiidae	*Ceratoppia*		CNPCD097-13	KM826423
Sarcoptiformes	Peloppiidae	*Ceratoppia*		CNPCD098-13	KM827508
Sarcoptiformes	Peloppiidae	*Ceratoppia*		CNPCD099-13	KM832291
Sarcoptiformes	Peloppiidae	*Ceratoppia*		CNPCD107-13	KM825829
Sarcoptiformes	Peloppiidae	*Ceratoppia*		CNPCD108-13	KM836956
Sarcoptiformes	Peloppiidae	*Ceratoppia*		CNPCD109-13	KM825725
Sarcoptiformes	Peloppiidae	*Ceratoppia*		CNPCD110-13	KM830571
Sarcoptiformes	Peloppiidae	*Ceratoppia*		CNPCD128-13	KM828455
Sarcoptiformes	Peloppiidae	*Ceratoppia*		CNPCD131-13	KM834538
Sarcoptiformes	Peloppiidae	*Ceratoppia*		CNPCD140-13	KM826519
Sarcoptiformes	Peloppiidae	*Ceratoppia*		CNPCD142-13	KM824480
Sarcoptiformes	Peloppiidae	*Ceratoppia*		CNPCD147-13	KM835715
Sarcoptiformes	Peloppiidae	*Ceratoppia*		CNPCD149-13	KM826832
Sarcoptiformes	Peloppiidae	*Ceratoppia*		CNPCD151-13	KM836479

Sarcoptiformes	Peloppiidae	*Ceratoppia*	CNPCD155-13	KM830482
Sarcoptiformes	Peloppiidae	*Ceratoppia*	CNPCD156-13	KM830279
Sarcoptiformes	Peloppiidae	*Ceratoppia*	CNPCD158-13	KM838119
Sarcoptiformes	Peloppiidae	*Ceratoppia*	CNPCE089-13	KM831462
Sarcoptiformes	Peloppiidae	*Ceratoppia*	CNPCE090-13	KM834647
Sarcoptiformes	Peloppiidae	*Ceratoppia*	CNPCE091-13	KM832021
Sarcoptiformes	Peloppiidae	*Ceratoppia*	CNPCE092-13	KM828521
Sarcoptiformes	Peloppiidae	*Ceratoppia*	CNPCE093-13	KM835470
Sarcoptiformes	Peloppiidae	*Ceratoppia*	CNPCE094-13	KM836687
Sarcoptiformes	Peloppiidae	*Ceratoppia*	CNPCE099-13	KM830232
Sarcoptiformes	Peloppiidae	*Ceratoppia*	CNPCE100-13	KM839344
Sarcoptiformes	Peloppiidae	*Ceratoppia*	CNPCE106-13	KM824558
Sarcoptiformes	Peloppiidae	*Ceratoppia*	CNPCE107-13	KM829247
Sarcoptiformes	Peloppiidae	*Ceratoppia*	CNPCE108-13	KM837260
Sarcoptiformes	Peloppiidae	*Ceratoppia*	CNPCE109-13	KM839791
Sarcoptiformes	Peloppiidae	*Ceratoppia*	CNPCE111-13	KM826027
Sarcoptiformes	Peloppiidae	*Ceratoppia*	CNPCE113-13	KM833293
Sarcoptiformes	Peloppiidae	*Ceratoppia*	CNPCE115-13	KM827479
Sarcoptiformes	Peloppiidae	*Ceratoppia*	CNPCE116-13	KM832211
Sarcoptiformes	Peloppiidae	*Ceratoppia*	CNPCE119-13	KM836567
Sarcoptiformes	Peloppiidae	*Ceratoppia*	CNPCE123-13	KM827918
Sarcoptiformes	Peloppiidae	*Ceratoppia*	CNPCE124-13	KM838025
Sarcoptiformes	Peloppiidae	*Ceratoppia*	CNPCE125-13	KM833840
Sarcoptiformes	Peloppiidae	*Ceratoppia*	CNPCE126-13	KM826765
Sarcoptiformes	Peloppiidae	*Ceratoppia*	CNPCE127-13	KM839050
Sarcoptiformes	Peloppiidae	*Ceratoppia*	CNPCE128-13	KM835524
Sarcoptiformes	Peloppiidae	*Ceratoppia*	CNPCE129-13	KM824222
Sarcoptiformes	Peloppiidae	*Ceratoppia*	CNPCE133-13	KM826906
Sarcoptiformes	Peloppiidae	*Ceratoppia*	CNPCE134-13	KM827747
Sarcoptiformes	Peloppiidae	*Ceratoppia*	CNPCE135-13	KM833844
Sarcoptiformes	Peloppiidae	*Ceratoppia*	CNPCE136-13	KM824929
Sarcoptiformes	Peloppiidae	*Ceratoppia*	CNPCE137-13	KM831269
Sarcoptiformes	Peloppiidae	*Ceratoppia*	CNPCE142-13	KM825812
Sarcoptiformes	Peloppiidae	*Ceratoppia*	CNPCE145-13	KM837250
Sarcoptiformes	Peloppiidae	*Ceratoppia*	CNPCE148-13	KM827542
Sarcoptiformes	Peloppiidae	*Ceratoppia*	CNPCE149-13	KM824219
Sarcoptiformes	Peloppiidae	*Ceratoppia*	CNPCE151-13	KM833106
Sarcoptiformes	Peloppiidae	*Ceratoppia*	CNPCE153-13	KM834314
Sarcoptiformes	Peloppiidae	*Ceratoppia*	CNPCE160-13	KM827737
Sarcoptiformes	Peloppiidae	*Ceratoppia*	CNPCE161-13	KM827680
Sarcoptiformes	Peloppiidae	*Ceratoppia*	CNPCE162-13	KM833252
Sarcoptiformes	Peloppiidae	*Ceratoppia*	CNPCE163-13	KM826258
Sarcoptiformes	Peloppiidae	*Ceratoppia*	CNPCF082-13	KM838779
Sarcoptiformes	Peloppiidae	*Ceratoppia*	CNPCF084-13	KM835063
Sarcoptiformes	Peloppiidae	*Ceratoppia*	CNPCF088-13	KM833657
Sarcoptiformes	Peloppiidae	*Ceratoppia*	CNPCF089-13	KM834480
Sarcoptiformes	Peloppiidae	*Ceratoppia*	CNPCF090-13	KM827739
Sarcoptiformes	Peloppiidae	*Ceratoppia*	CNPCF091-13	KM839495
Sarcoptiformes	Peloppiidae	*Ceratoppia*	CNPCF092-13	KM824708
Sarcoptiformes	Peloppiidae	*Ceratoppia*	CNPCF093-13	KM833595
Sarcoptiformes	Peloppiidae	*Ceratoppia*	CNPCF094-13	KM831896
Sarcoptiformes	Peloppiidae	*Ceratoppia*	CNPCF095-13	KM824405
Sarcoptiformes	Peloppiidae	*Ceratoppia*	CNPCF097-13	KM835064

Sarcoptiformes	Peloppiidae	*Ceratoppia*	CNPCF098-13	KM827597
Sarcoptiformes	Peloppiidae	*Ceratoppia*	CNPCF100-13	KM832505
Sarcoptiformes	Peloppiidae	*Ceratoppia*	CNPCF103-13	KM836214
Sarcoptiformes	Peloppiidae	*Ceratoppia*	CNPCF105-13	KM836985
Sarcoptiformes	Peloppiidae	*Ceratoppia*	CNPCF108-13	KM838455
Sarcoptiformes	Peloppiidae	*Ceratoppia*	CNPCF109-13	KM826580
Sarcoptiformes	Peloppiidae	*Ceratoppia*	CNPCF114-13	KM833678
Sarcoptiformes	Peloppiidae	*Ceratoppia*	CNPCF116-13	KM837178
Sarcoptiformes	Peloppiidae	*Ceratoppia*	CNPCF119-13	KM836088
Sarcoptiformes	Peloppiidae	*Ceratoppia*	CNPCF122-13	KM829520
Sarcoptiformes	Peloppiidae	*Ceratoppia*	CNPCF123-13	KM832990
Sarcoptiformes	Peloppiidae	*Ceratoppia*	CNPCF125-13	KM833622
Sarcoptiformes	Peloppiidae	*Ceratoppia*	CNPCF132-13	KM826912
Sarcoptiformes	Peloppiidae	*Ceratoppia*	CNPCG044-13	KM830373
Sarcoptiformes	Peloppiidae	*Ceratoppia*	CNPCG045-13	KM826292
Sarcoptiformes	Peloppiidae	*Ceratoppia*	CNPCG047-13	KM828534
Sarcoptiformes	Peloppiidae	*Ceratoppia*	CNPCG048-13	KM839894
Sarcoptiformes	Peloppiidae	*Ceratoppia*	CNPCG049-13	KM831717
Sarcoptiformes	Peloppiidae	*Ceratoppia*	CNPCG052-13	KM828640
Sarcoptiformes	Peloppiidae	*Ceratoppia*	CNPCG053-13	KM829040
Sarcoptiformes	Peloppiidae	*Ceratoppia*	MIONB039-10	HM887576
Sarcoptiformes	Peloppiidae	*Ceratoppia*	MIONB053-10	HQ575079
Sarcoptiformes	Peloppiidae	*Ceratoppia*	MIONB054-10	HQ575080
Sarcoptiformes	Peloppiidae	*Ceratoppia*	MIONB070-10	HQ575085
Sarcoptiformes	Peloppiidae	*Ceratoppia*	MIONB071-10	HQ575086
Sarcoptiformes	Peloppiidae	*Ceratoppia*	MIONB072-10	HQ575087
Sarcoptiformes	Peloppiidae	*Ceratoppia*	MIONB073-10	HQ575088
Sarcoptiformes	Peloppiidae	*Ceratoppia*	MIONB074-10	HQ575089
Sarcoptiformes	Peloppiidae	*Ceratoppia*	MYMCA1125-11	JX836699
Sarcoptiformes	Peloppiidae	*Ceratoppia*	MYMCA1145-11	JX838463
Sarcoptiformes	Peloppiidae	*Ceratoppia*	MYMCA790-11	JX834729
Sarcoptiformes	Peloppiidae	*Ceratoppia*	MYMCA799-11	JX834370
Sarcoptiformes	Peloppiidae	*Ceratoppia*	MYMCB848-11	JX838303
Sarcoptiformes	Peloppiidae	*Ceratoppia*	MYMCC488-11	JX834082
Sarcoptiformes	Peloppiidae	*Ceratoppia*	MYMCD159-11	JX838231
Sarcoptiformes	Peloppiidae	*Ceratoppia*	SSEIC4146-13	KM824496
Sarcoptiformes	Peloppiidae	*Ceratoppia*	SSEIC4156-13	KM837769
Sarcoptiformes	Peloppiidae	*Ceratoppia*	SSEIC6022-13	KM838304
Sarcoptiformes	Peloppiidae	*Ceratoppia*	SSJAC1576-13	KM830797
Sarcoptiformes	Peloppiidae	*Ceratoppia*	SSPAB8722-13	KM825114
Sarcoptiformes	Peloppiidae	*Ceratoppia*	SSPAC7406-13	KM835785
Sarcoptiformes	Peloppiidae	*Ceratoppia*	SSPAG392-13	KM824058
Sarcoptiformes	Peloppiidae	*Ceratoppia*	SSWLA029-13	KM839275
Sarcoptiformes	Peloppiidae	*Ceratoppia*	SSWLA030-13	KM830783
Sarcoptiformes	Peloppiidae	*Ceratoppia*	SSWLA033-13	KM824430
Sarcoptiformes	Peloppiidae	*Ceratoppia*	SSWLA040-13	KM833773
Sarcoptiformes	Peloppiidae	*Ceratoppia*	SSWLA041-13	KM838174
Sarcoptiformes	Peloppiidae	*Ceratoppia*	SSWLA1101-13	KM837214
Sarcoptiformes	Peloppiidae	*Ceratoppia*	SSWLA1885-13	KM831253
Sarcoptiformes	Peloppiidae	*Ceratoppia*	SSWLA1888-13	KM840318
Sarcoptiformes	Peloppiidae	*Ceratoppia*	SSWLA1894-13	KM831591
Sarcoptiformes	Peloppiidae	*Ceratoppia*	SSWLA1897-13	KM837503
Sarcoptiformes	Peloppiidae	*Ceratoppia*	SSWLA1900-13	KM840204

Sarcoptiformes	Peloppiidae	*Ceratoppia*	SSWLC1164-13	KM840275
Sarcoptiformes	Peloppiidae	*Ceratoppia*	SSWLC1167-13	KM829839
Sarcoptiformes	Peloppiidae	*Ceratoppia*	SSWLC1168-13	KM831715
Sarcoptiformes	Peloppiidae	*Ceratoppia*	SSWLC1171-13	KM824257
Sarcoptiformes	Peloppiidae	*Ceratoppia*	SSWLC1173-13	KM835187
Sarcoptiformes	Peloppiidae	*Ceratoppia*	SSWLC1179-13	KM840378
Sarcoptiformes	Peloppiidae	*Ceratoppia*	SSWLC1180-13	KM835149
Sarcoptiformes	Peloppiidae	*Ceratoppia*	SSWLC1188-13	KM836512
Sarcoptiformes	Peloppiidae	*Ceratoppia*	SSWLC3115-13	KM831415
Sarcoptiformes	Peloppiidae	*Ceratoppia*	SSWLC3120-13	KM837382
Sarcoptiformes	Peloppiidae	*Ceratoppia*	SSWLC3121-13	KM832407
Sarcoptiformes	Peloppiidae	*Ceratoppia*	SSWLC3125-13	KM831675
Sarcoptiformes	Peloppiidae	*Ceratoppia quadridentata*	CHACA1020-10	HM907079
Sarcoptiformes	Peloppiidae	*Ceratoppia quadridentata*	CHACA1023-10	HM907080
Sarcoptiformes	Peloppiidae	*Ceratoppia quadridentata*	CHACA1060-10	HM907108
Sarcoptiformes	Peloppiidae	*Ceratoppia quadridentata*	CHACA342-08	JX834466
Sarcoptiformes	Peloppiidae	*Ceratoppia quadridentata*	CHACA624-09	JX837889
Sarcoptiformes	Peloppiidae	*Ceratoppia quadridentata*	CHACA714-09	JX834349
Sarcoptiformes	Peloppiidae	*Ceratoppia quadridentata*	CHACA805-09	JX836668
Sarcoptiformes	Peloppiidae	*Ceratoppia quadridentata*	CHACA816-09	JX838339
Sarcoptiformes	Peloppiidae	*Ceratoppia quadridentata*	CHACB257-10	HQ558500
Sarcoptiformes	Peloppiidae	*Ceratoppia quadridentata*	CHACB258-10	HQ558501
Sarcoptiformes	Peloppiidae	*Ceratoppia quadridentata*	CHACB297-10	HM907289
Sarcoptiformes	Peloppiidae	*Ceratoppia quadridentata*	CHACB298-10	HM907290
Sarcoptiformes	Peloppiidae	*Ceratoppia quadridentata*	CHACB645-10	HQ558725
Sarcoptiformes	Peloppiidae	*Ceratoppia quadridentata*	MYMCA022-11	JX835674
Sarcoptiformes	Peloppiidae	*Ceratoppia quadridentata*	MYMCA038-11	JX837155
Sarcoptiformes	Peloppiidae	*Ceratoppia quadridentata*	MYMCA039-11	JX834046
Sarcoptiformes	Peloppiidae	*Ceratoppia quadridentata*	MYMCA040-11	JX838566
Sarcoptiformes	Peloppiidae	*Ceratoppia quadridentata*	MYMCA1046-11	JX835687
Sarcoptiformes	Peloppiidae	*Ceratoppia quadridentata*	MYMCA1061-11	JX838398
Sarcoptiformes	Peloppiidae	*Ceratoppia quadridentata*	MYMCA184-11	JX835283
Sarcoptiformes	Peloppiidae	*Ceratoppia quadridentata*	MYMCA190-11	JX837182
Sarcoptiformes	Peloppiidae	*Ceratoppia quadridentata*	MYMCA191-11	JX836188
Sarcoptiformes	Peloppiidae	*Ceratoppia quadridentata*	MYMCA473-11	JX837916
Sarcoptiformes	Peloppiidae	*Ceratoppia quadridentata*	MYMCA504-11	JX835426
Sarcoptiformes	Peloppiidae	*Ceratoppia quadridentata*	MYMCA827-11	JX837971
Sarcoptiformes	Peloppiidae	*Ceratoppia quadridentata*	MYMCA905-11	JX836812
Sarcoptiformes	Peloppiidae	*Ceratoppia quadridentata*	MYMCA937-11	JX834209
Sarcoptiformes	Peloppiidae	*Ceratoppia quadridentata*	MYMCB481-11	JX836124
Sarcoptiformes	Peloppiidae	*Ceratoppia quadridentata*	MYMCB482-11	JX836115
Sarcoptiformes	Peloppiidae	*Ceratoppia quadridentata*	MYMCB538-11	JX835777
Sarcoptiformes	Peloppiidae	*Ceratoppia quadridentata*	MYMCB623-11	JX833840
Sarcoptiformes	Peloppiidae	*Ceratoppia quadridentata*	MYMCB644-11	JX838698
Sarcoptiformes	Peloppiidae	*Ceratoppia quadridentata*	MYMCB743-11	JX834198
Sarcoptiformes	Peloppiidae	*Ceratoppia quadridentata*	MYMCB879-11	JX837124
Sarcoptiformes	Peloppiidae	*Ceratoppia quadridentata*	MYMCB884-11	JX836106
Sarcoptiformes	Peloppiidae	*Ceratoppia quadridentata*	MYMCB903-11	JX837414
Sarcoptiformes	Peloppiidae	*Ceratoppia quadridentata*	MYMCC217-11	JX835720
Sarcoptiformes	Peloppiidae	*Ceratoppia quadridentata*	MYMCC256-11	JX835990
Sarcoptiformes	Peloppiidae	*Ceratoppia quadridentata*	MYMCC306-11	JX837343
Sarcoptiformes	Peloppiidae	*Ceratoppia quadridentata*	MYMCC310-11	JX835069
Sarcoptiformes	Peloppiidae	*Ceratoppia quadridentata*	MYMCC521-11	JX838138

Sarcoptiformes	Peloppiidae	*Ceratoppia quadridentata*	MYMCC561-11	JX836945
Sarcoptiformes	Peloppiidae	*Ceratoppia quadridentata*	MYMCC595-11	JX836920
Sarcoptiformes	Peloppiidae	*Ceratoppia quadridentata*	MYMCC673-11	JX835120
Sarcoptiformes	Peloppiidae	*Ceratoppia quadridentata*	MYMCC684-11	JX838169
Sarcoptiformes	Peloppiidae	*Ceratoppia quadridentata*	MYMCC713-11	JX837077
Sarcoptiformes	Peloppiidae	*Ceratoppia quadridentata*	MYMCC791-11	JX837404
Sarcoptiformes	Peloppiidae	*Ceratoppia quadridentata*	MYMCC904-11	JX837035
Sarcoptiformes	Peloppiidae	*Ceratoppia quadridentata*	MYMCC929-11	JX835591
Sarcoptiformes	Peloppiidae	*Ceratoppia quadridentata*	MYMCE556-12	JX837090
Sarcoptiformes	Peloppiidae	*Ceratoppia quadridentata*	MYMCE713-12	JX837796
Sarcoptiformes	Peloppiidae	*Ceratoppia quadridentata*	MYMCE867-12	JX836083
Sarcoptiformes	Peloppiidae	*Ceratoppia quadridentata*	MYMCF384-12	JX835604
Sarcoptiformes	Peloppiidae	*Ceratoppia quadridentata*	MYMCF565-12	JX838536
Sarcoptiformes	Peloppiidae	*Ceratoppia quadridentata*	MYMCF637-12	JX833844
Sarcoptiformes	Peloppiidae	*Ceratoppia quadridentata*	MYMCG196-12	JX836342
Sarcoptiformes	Peloppiidae	*Ceratoppia quadridentata*	MYMCG197-12	JX837821
Sarcoptiformes	Peloppiidae	*Ceratoppia quadridentata*	MYMCG477-12	JX834880
Sarcoptiformes	Peloppiidae	*Ceratoppia quadridentata*	MYTMC018-09	GU680484
Sarcoptiformes	Peloppiidae	*Ceratoppia quadridentata*	MYTMC021-09	GU680482
Sarcoptiformes	Peloppiidae	*Ceratoppia quadridentata*	MYTMC034-09	GU680478
Sarcoptiformes	Peloppiidae	*Ceratoppia quadridentata*	MYTMC158-09	GU680453
Sarcoptiformes	Peloppiidae	*Ceratoppia quadridentata*	SSPAA7801-13	KM824649
Sarcoptiformes	Peloppiidae	*Dendrozetes*	CNPCB082-13	KM825535
Sarcoptiformes	Peloppiidae	*Dendrozetes*	CNPCB084-13	KM838064
Sarcoptiformes	Peloppiidae	*Dendrozetes*	CNPCD129-13	KM832737
Sarcoptiformes	Peloppiidae	*Dendrozetes*	CNPCE144-13	KM832952
Sarcoptiformes	Peloppiidae	*Dendrozetes*	CNPCE159-13	KM834961
Sarcoptiformes	Phenopelopidae	*Eupelops*	CHACA290-08	JX837045
Sarcoptiformes	Phenopelopidae	*Eupelops*	CHACA539-09	JX834185
Sarcoptiformes	Phenopelopidae	*Eupelops*	CHACB417-10	HQ558574
Sarcoptiformes	Phenopelopidae	*Eupelops*	CHACB734-10	HQ558778
Sarcoptiformes	Phenopelopidae	*Eupelops*	CHACB925-10	HM907353
Sarcoptiformes	Phenopelopidae	*Eupelops*	MITMH033-07	KR069245
Sarcoptiformes	Phenopelopidae	*Eupelops*	MYMCA141-11	JX836159
Sarcoptiformes	Phenopelopidae	*Eupelops*	MYMCA148-11	JX833798
Sarcoptiformes	Phenopelopidae	*Eupelops*	MYMCA149-11	JX833969
Sarcoptiformes	Phenopelopidae	*Eupelops*	MYMCA150-11	JX834097
Sarcoptiformes	Phenopelopidae	*Eupelops*	MYMCA152-11	JX837411
Sarcoptiformes	Phenopelopidae	*Eupelops*	MYMCB699-11	JX835492
Sarcoptiformes	Phenopelopidae	*Eupelops*	MYMCB733-11	JX835737
Sarcoptiformes	Phenopelopidae	*Eupelops*	MYMCC117-11	JX834467
Sarcoptiformes	Phenopelopidae	*Eupelops*	MYMCC295-11	JX835926
Sarcoptiformes	Phenopelopidae	*Eupelops*	MYMCC296-11	JX837071
Sarcoptiformes	Phenopelopidae	*Eupelops*	MYMCC390-11	JX836280
Sarcoptiformes	Phenopelopidae	*Eupelops*	MYMCC391-11	JX835324
Sarcoptiformes	Phenopelopidae	*Eupelops*	MYMCC747-11	JX838729
Sarcoptiformes	Phenopelopidae	*Eupelops*	MYMCC769-11	JX837469
Sarcoptiformes	Phenopelopidae	*Eupelops*	MYMCD032-11	JX835016
Sarcoptiformes	Phenopelopidae	*Eupelops*	MYMCE143-12	JX833893
Sarcoptiformes	Phenopelopidae	*Eupelops*	MYMCE402-12	JX838022
Sarcoptiformes	Phenopelopidae	*Eupelops*	MYMCE478-12	JX838350
Sarcoptiformes	Phenopelopidae	*Eupelops*	MYMCE548-12	JX834343
Sarcoptiformes	Phenopelopidae	*Eupelops*	MYMCF476-12	JX833695

Sarcoptiformes	Phenopelopidae	*Eupelops*	MYMCG120-12	JX837572
Sarcoptiformes	Phenopelopidae	*Eupelops*	MYMCG380-12	JX835747
Sarcoptiformes	Phenopelopidae	*Eupelops*	RBINA4455-13	KP979346
Sarcoptiformes	Phenopelopidae	*Eupelops septentrionalis*	CHACA152-08	JX838476
Sarcoptiformes	Phenopelopidae	*Eupelops septentrionalis*	CHACA158-08	JX835711
Sarcoptiformes	Phenopelopidae	*Eupelops septentrionalis*	CHACA159-08	JX837877
Sarcoptiformes	Phenopelopidae	*Eupelops septentrionalis*	CHACA161-08	JX834120
Sarcoptiformes	Phenopelopidae	*Eupelops septentrionalis*	CHACB395-10	HQ558560
Sarcoptiformes	Phenopelopidae	*Eupelops septentrionalis*	CHACB396-10	HQ558561
Sarcoptiformes	Phenopelopidae	*Eupelops septentrionalis*	CHACB397-10	HQ558562
Sarcoptiformes	Phenopelopidae	*Eupelops septentrionalis*	CHACB398-10	HQ558563
Sarcoptiformes	Phenopelopidae	*Eupelops septentrionalis*	CHACB399-10	HQ558564
Sarcoptiformes	Phenopelopidae	*Eupelops septentrionalis*	CHACB435-10	HQ558591
Sarcoptiformes	Phenopelopidae	*Eupelops septentrionalis*	MITMH053-07	KR070175
Sarcoptiformes	Phenopelopidae	*Eupelops septentrionalis*	MITMH071-07	KR070510
Sarcoptiformes	Phenopelopidae	*Eupelops septentrionalis*	MYMCB868-11	JX837567
Sarcoptiformes	Phenopelopidae	*Eupelops septentrionalis*	MYMCE059-12	JX834588
Sarcoptiformes	Phenopelopidae	*Eupelops septentrionalis*	MYMCE069-12	JX834202
Sarcoptiformes	Phenopelopidae	*Eupelops septentrionalis*	MYMCE071-12	JX835599
Sarcoptiformes	Phenopelopidae	*Eupelops septentrionalis*	MYMCE107-12	JX838546
Sarcoptiformes	Phenopelopidae	*Eupelops septentrionalis*	MYMCE151-12	JX833666
Sarcoptiformes	Phenopelopidae	*Eupelops septentrionalis*	MYMCE422-12	JX835645
Sarcoptiformes	Phenopelopidae	*Eupelops septentrionalis*	MYMCE423-12	JX838126
Sarcoptiformes	Phenopelopidae	*Eupelops septentrionalis*	MYMCE572-12	JX837690
Sarcoptiformes	Phenopelopidae	*Eupelops septentrionalis*	MYMCG034-12	JX837987
Sarcoptiformes	Phenopelopidae	*Eupelops septentrionalis*	MYMCG192-12	JX838743
Sarcoptiformes	Phenopelopidae	*Eupelops septentrionalis*	MYMCG464-12	JX837073
Sarcoptiformes	Phenopelopidae	*Peloptulus*	SSBAD4014-12	KM832060
Sarcoptiformes	Phenopelopidae	*Propelops alaskensis*	SSEIB5422-13	KM829285
Sarcoptiformes	Phenopelopidae	*Propelops alaskensis*	SSPAC10878-13	KM833055
Sarcoptiformes	Phenopelopidae	*Propelops canadensis*	CHACA1129-10	JX837204
Sarcoptiformes	Phenopelopidae	*Propelops canadensis*	CHACA153-08	JX833634
Sarcoptiformes	Phenopelopidae	*Propelops canadensis*	CHACA154-08	JX836615
Sarcoptiformes	Phenopelopidae	*Propelops canadensis*	CHACA155-08	JX834642
Sarcoptiformes	Phenopelopidae	*Propelops canadensis*	CHACA166-08	JX836807
Sarcoptiformes	Phenopelopidae	*Propelops canadensis*	CHACB438-10	HQ558594
Sarcoptiformes	Phenopelopidae	*Propelops canadensis*	CHACB439-10	HQ558595
Sarcoptiformes	Phenopelopidae	*Propelops canadensis*	CHACB440-10	HQ558596
Sarcoptiformes	Phenopelopidae	*Propelops canadensis*	CHACB441-10	HQ558597
Sarcoptiformes	Phenopelopidae	*Propelops canadensis*	CHACB483-10	HQ558618
Sarcoptiformes	Phenopelopidae	*Propelops canadensis*	CHACB486-10	HQ558621
Sarcoptiformes	Phenopelopidae	*Propelops canadensis*	CHACB487-10	HQ558622
Sarcoptiformes	Phenopelopidae	*Propelops canadensis*	CHACB488-10	HQ558623
Sarcoptiformes	Phenopelopidae	*Propelops canadensis*	CHACB503-10	HQ558633
Sarcoptiformes	Phenopelopidae	*Propelops canadensis*	CHACB617-10	HQ558704
Sarcoptiformes	Phenopelopidae	*Propelops canadensis*	CHACB619-10	HQ558706
Sarcoptiformes	Phenopelopidae	*Propelops canadensis*	CHACB724-10	HQ558774
Sarcoptiformes	Phenopelopidae	*Propelops canadensis*	MITMH069-07	KR069828
Sarcoptiformes	Phenopelopidae	*Propelops canadensis*	MYMCA154-11	JX838242
Sarcoptiformes	Phenopelopidae	*Propelops canadensis*	MYMCA163-11	JX835727
Sarcoptiformes	Phenopelopidae	*Propelops canadensis*	MYMCA255-11	JX833674
Sarcoptiformes	Phenopelopidae	*Propelops canadensis*	MYMCC124-11	JX834725
Sarcoptiformes	Phenopelopidae	*Propelops canadensis*	MYMCC297-11	JX836147

Sarcoptiformes	Phenopelopidae	*Propelops canadensis*	MYMCC596-11	JX835749
Sarcoptiformes	Phenopelopidae	*Propelops canadensis*	MYMCC636-11	JX836021
Sarcoptiformes	Phenopelopidae	*Propelops canadensis*	MYMCD079-11	JX836636
Sarcoptiformes	Phenopelopidae	*Propelops canadensis*	MYMCD080-11	JX837894
Sarcoptiformes	Phenopelopidae	*Propelops canadensis*	MYMCD099-11	JX838475
Sarcoptiformes	Phenopelopidae	*Propelops canadensis*	MYMCD117-11	JX837958
Sarcoptiformes	Phenopelopidae	*Propelops canadensis*	MYMCD118-11	JX838161
Sarcoptiformes	Phenopelopidae	*Propelops canadensis*	MYMCE022-12	JX834734
Sarcoptiformes	Phenopelopidae	*Propelops canadensis*	MYMCE025-12	JX838510
Sarcoptiformes	Phenopelopidae	*Propelops canadensis*	MYMCE153-12	JX835423
Sarcoptiformes	Phenopelopidae	*Propelops canadensis*	MYMCE427-12	JX833904
Sarcoptiformes	Phenopelopidae	*Propelops canadensis*	MYMCE428-12	JX836753
Sarcoptiformes	Phenopelopidae	*Propelops canadensis*	MYMCE497-12	JX835271
Sarcoptiformes	Phenopelopidae	*Propelops canadensis*	MYMCE501-12	JX834340
Sarcoptiformes	Phenopelopidae	*Propelops canadensis*	MYMCE555-12	JX834350
Sarcoptiformes	Phenopelopidae	*Propelops canadensis*	MYMCF615-12	JX835931
Sarcoptiformes	Phenopelopidae	*Propelops canadensis*	MYMCG194-12	JX837458
Sarcoptiformes	Phenopelopidae	*Propelops canadensis*	MYMCG195-12	JX834469
Sarcoptiformes	Phenopelopidae	*Propelops canadensis*	MYMCG235-12	JX836991
Sarcoptiformes	Phenopelopidae	*Unduloribates undulatus*	GBCH5036-10	GU208615
Sarcoptiformes	Phenopelopidae	*Unduloribates undulatus*	GBCH5037-10	GU208614
Sarcoptiformes	Phenopelopidae		CNPCR146-13	KM826176
Sarcoptiformes	Phenopelopidae		SSBAB1925-12	KM827394
Sarcoptiformes	Phenopelopidae		SSBAE3332-13	KM840402
Sarcoptiformes	Phenopelopidae		SSJAB3357-13	KM826764
Sarcoptiformes	Phenopelopidae		SSJAB4115-13	KM837263
Sarcoptiformes	Phenopelopidae		SSJAB4116-13	KM826355
Sarcoptiformes	Phthiracaridae	*Phthiracarus*	MIONB031-10	HM887571
Sarcoptiformes	Phthiracaridae	*Phthiracarus*	SSPAA8013-13	KM827004
Sarcoptiformes	Phthiracaridae	*Phthiracarus boresetosus*	RBINA5733-13	KR070416
Sarcoptiformes	Phthiracaridae	*Phthiracarus boresetosus*	RBINA5734-13	KP979172
Sarcoptiformes	Phthiracaridae	*Phthiracarus boresetosus*	RBINA5735-13	KR069201
Sarcoptiformes	Phthiracaridae	*Phthiracarus boresetosus*	SSEIC4927-13	KM832855
Sarcoptiformes	Phthiracaridae	*Steganacarus magnus*	GACAC1050-12	JN162413
Sarcoptiformes	Phthiracaridae		CHACA1027-10	HM907083
Sarcoptiformes	Phthiracaridae		CHACA1028-10	HM907084
Sarcoptiformes	Phthiracaridae		CHACA1029-10	HM907085
Sarcoptiformes	Phthiracaridae		CHACA1030-10	HM907086
Sarcoptiformes	Phthiracaridae		CHACA999-10	HM405857
Sarcoptiformes	Phthiracaridae		CHACB660-10	HQ558737
Sarcoptiformes	Phthiracaridae		CHACB737-10	JX838758
Sarcoptiformes	Phthiracaridae		MIONB032-10	KR070559
Sarcoptiformes	Phthiracaridae		MIONB034-10	HM887572
Sarcoptiformes	Phthiracaridae		MIONB036-10	HM887573
Sarcoptiformes	Phthiracaridae		MYMCA1070-11	JX836522
Sarcoptiformes	Phthiracaridae		MYMCA1071-11	JX838759
Sarcoptiformes	Phthiracaridae		MYMCA906-11	JX838601
Sarcoptiformes	Phthiracaridae		MYMCB393-11	JX835462
Sarcoptiformes	Phthiracaridae		MYMCB676-11	JX836590
Sarcoptiformes	Phthiracaridae		MYMCB809-11	JX835509
Sarcoptiformes	Phthiracaridae		MYMCB810-11	JX837312
Sarcoptiformes	Phthiracaridae		MYMCC058-11	JX835111
Sarcoptiformes	Phthiracaridae		MYMCC090-11	JX837141

Sarcoptiformes	Phthiracaridae		MYMCC435-11	JX836852
Sarcoptiformes	Phthiracaridae		MYMCC436-11	JX837718
Sarcoptiformes	Phthiracaridae		MYMCC437-11	JX835644
Sarcoptiformes	Phthiracaridae		MYMCC868-11	JX837586
Sarcoptiformes	Phthiracaridae		MYMCC906-11	JX833984
Sarcoptiformes	Phthiracaridae		MYMCC907-11	JX836778
Sarcoptiformes	Phthiracaridae		MYMCC908-11	JX835775
Sarcoptiformes	Phthiracaridae		MYMCE809-12	JX834174
Sarcoptiformes	Phthiracaridae		MYMCE869-12	JX838547
Sarcoptiformes	Phthiracaridae		MYMCF065-12	JX837748
Sarcoptiformes	Phthiracaridae		MYMCF374-12	JX835122
Sarcoptiformes	Phthiracaridae		MYMCF742-12	JX834991
Sarcoptiformes	Phthiracaridae		MYMCF762-12	JX837429
Sarcoptiformes	Phthiracaridae		MYMCG379-12	JX836550
Sarcoptiformes	Phthiracaridae		RBINA5647-13	KR070492
Sarcoptiformes	Phthiracaridae		RBINA5671-13	KP979201
Sarcoptiformes	Phthiracaridae		RBINA5674-13	KP979241
Sarcoptiformes	Phthiracaridae		RBINA5722-13	KR070636
Sarcoptiformes	Phthiracaridae		SSBAB1915-12	KM837460
Sarcoptiformes	Phthiracaridae		SSBAB2085-12	KM829418
Sarcoptiformes	Phthiracaridae		SSBAE3329-13	KM826608
Sarcoptiformes	Phthiracaridae		SSPAA7743-13	KM838083
Sarcoptiformes	Proctophyllodidae	*Dolichodectes*	FMEP100-14	KP193461
Sarcoptiformes	Proctophyllodidae	*Dolichodectes*	FMEP102-14	KP193462
Sarcoptiformes	Proctophyllodidae	*Dolichodectes*	FMEP103-14	KP193463
Sarcoptiformes	Proctophyllodidae	*Dolichodectes*	FMEP105-14	KP193460
Sarcoptiformes	Proctophyllodidae	*Dolichodectes*	FMEP106-14	KP193459
Sarcoptiformes	Proctophyllodidae	*Joubertophyllodes modularis*	FMEP260-14	KP193466
Sarcoptiformes	Proctophyllodidae	*Joubertophyllodes modularis*	FMEP261-14	KP193465
Sarcoptiformes	Proctophyllodidae	*Joubertophyllodes modularis*	FMEP262-14	KP193467
Sarcoptiformes	Proctophyllodidae	*Joubertophyllodes modularis*	FMEP263-14	KP193468
Sarcoptiformes	Proctophyllodidae	*Joubertophyllodes modularis*	FMEP264-14	KP193469
Sarcoptiformes	Proctophyllodidae	*Joubertophyllodes modularis*	GBCH4462-10	GQ864339
Sarcoptiformes	Proctophyllodidae	*Monojoubertia hemiphylla*	FMEP265-14	KP193476
Sarcoptiformes	Proctophyllodidae	*Monojoubertia hemiphylla*	FMEP266-14	KP193477
Sarcoptiformes	Proctophyllodidae	*Monojoubertia hemiphylla*	FMEP267-14	KP193475
Sarcoptiformes	Proctophyllodidae	*Monojoubertia microphylla*	FMEP001-14	KP193483
Sarcoptiformes	Proctophyllodidae	*Monojoubertia microphylla*	FMEP042-14	KP193482
Sarcoptiformes	Proctophyllodidae	*Monojoubertia microphylla*	FMEP043-14	KP193479
Sarcoptiformes	Proctophyllodidae	*Monojoubertia microphylla*	FMEP068-14	KP193478
Sarcoptiformes	Proctophyllodidae	*Monojoubertia microphylla*	FMEP069-14	KP193480
Sarcoptiformes	Proctophyllodidae	*Monojoubertia microphylla*	FMEP099-14	KP193481
Sarcoptiformes	Proctophyllodidae	*Monojoubertia microphylla*	FMEP194-14	KP193484
Sarcoptiformes	Proctophyllodidae	*Monojoubertia microphylla*	FMEP195-14	KP193485
Sarcoptiformes	Proctophyllodidae	*Monojoubertia microphylla*	FMEP196-14	KP193486
Sarcoptiformes	Proctophyllodidae	*Monojoubertia microphylla*	FMEP197-14	KP193487
Sarcoptiformes	Proctophyllodidae	*Monojoubertia microphylla*	GBCH4449-10	GQ864353
Sarcoptiformes	Proctophyllodidae	*Montesauria cilindrica*	FMEP284-14	KP193488
Sarcoptiformes	Proctophyllodidae	*Proctophyllodes*	FMEP003-14	KP193734
Sarcoptiformes	Proctophyllodidae	*Proctophyllodes*	FMEP006-14	KP193693
Sarcoptiformes	Proctophyllodidae	*Proctophyllodes*	FMEP007-14	KP193691
Sarcoptiformes	Proctophyllodidae	*Proctophyllodes*	FMEP009-14	KP193641
Sarcoptiformes	Proctophyllodidae	*Proctophyllodes*	FMEP012-14	KP193489

Sarcoptiformes	Proctophyllodidae	*Proctophyllodes*	FMEP013-14	KP193629
Sarcoptiformes	Proctophyllodidae	*Proctophyllodes*	FMEP026-14	KP193650
Sarcoptiformes	Proctophyllodidae	*Proctophyllodes*	FMEP054-14	KP193742
Sarcoptiformes	Proctophyllodidae	*Proctophyllodes*	FMEP055-14	KP193741
Sarcoptiformes	Proctophyllodidae	*Proctophyllodes*	FMEP056-14	KP193740
Sarcoptiformes	Proctophyllodidae	*Proctophyllodes*	FMEP057-14	KP193739
Sarcoptiformes	Proctophyllodidae	*Proctophyllodes*	FMEP058-14	KP193738
Sarcoptiformes	Proctophyllodidae	*Proctophyllodes*	FMEP059-14	KP193628
Sarcoptiformes	Proctophyllodidae	*Proctophyllodes*	FMEP060-14	KP193737
Sarcoptiformes	Proctophyllodidae	*Proctophyllodes*	FMEP061-14	KP193736
Sarcoptiformes	Proctophyllodidae	*Proctophyllodes*	FMEP062-14	KP193735
Sarcoptiformes	Proctophyllodidae	*Proctophyllodes*	FMEP070-14	KP193627
Sarcoptiformes	Proctophyllodidae	*Proctophyllodes*	FMEP071-14	KP193626
Sarcoptiformes	Proctophyllodidae	*Proctophyllodes*	FMEP081-14	KP193656
Sarcoptiformes	Proctophyllodidae	*Proctophyllodes*	FMEP082-14	KP193635
Sarcoptiformes	Proctophyllodidae	*Proctophyllodes*	FMEP083-14	KP193640
Sarcoptiformes	Proctophyllodidae	*Proctophyllodes*	FMEP084-14	KP193637
Sarcoptiformes	Proctophyllodidae	*Proctophyllodes*	FMEP085-14	KP193638
Sarcoptiformes	Proctophyllodidae	*Proctophyllodes*	FMEP086-14	KP193654
Sarcoptiformes	Proctophyllodidae	*Proctophyllodes*	FMEP087-14	KP193636
Sarcoptiformes	Proctophyllodidae	*Proctophyllodes*	FMEP088-14	KP193642
Sarcoptiformes	Proctophyllodidae	*Proctophyllodes*	FMEP089-14	KP193653
Sarcoptiformes	Proctophyllodidae	*Proctophyllodes*	FMEP096-14	KP193692
Sarcoptiformes	Proctophyllodidae	*Proctophyllodes*	FMEP097-14	KP193655
Sarcoptiformes	Proctophyllodidae	*Proctophyllodes*	FMEP110-14	KP193694
Sarcoptiformes	Proctophyllodidae	*Proctophyllodes*	FMEP111-14	KP193695
Sarcoptiformes	Proctophyllodidae	*Proctophyllodes*	FMEP143-14	KP193631
Sarcoptiformes	Proctophyllodidae	*Proctophyllodes*	FMEP144-14	KP193727
Sarcoptiformes	Proctophyllodidae	*Proctophyllodes*	FMEP145-14	KP193632
Sarcoptiformes	Proctophyllodidae	*Proctophyllodes*	FMEP146-14	KP193633
Sarcoptiformes	Proctophyllodidae	*Proctophyllodes*	FMEP147-14	KP193639
Sarcoptiformes	Proctophyllodidae	*Proctophyllodes*	FMEP148-14	KP193733
Sarcoptiformes	Proctophyllodidae	*Proctophyllodes*	FMEP149-14	KP193732
Sarcoptiformes	Proctophyllodidae	*Proctophyllodes*	FMEP150-14	KP193731
Sarcoptiformes	Proctophyllodidae	*Proctophyllodes*	FMEP151-14	KP193730
Sarcoptiformes	Proctophyllodidae	*Proctophyllodes*	FMEP152-14	KP193729
Sarcoptiformes	Proctophyllodidae	*Proctophyllodes*	FMEP153-14	KP193696
Sarcoptiformes	Proctophyllodidae	*Proctophyllodes*	FMEP154-14	KP193697
Sarcoptiformes	Proctophyllodidae	*Proctophyllodes*	FMEP155-14	KP193689
Sarcoptiformes	Proctophyllodidae	*Proctophyllodes*	FMEP156-14	KP193690
Sarcoptiformes	Proctophyllodidae	*Proctophyllodes*	FMEP157-14	KP193630
Sarcoptiformes	Proctophyllodidae	*Proctophyllodes*	FMEP158-14	KP193646
Sarcoptiformes	Proctophyllodidae	*Proctophyllodes*	FMEP159-14	KP193645
Sarcoptiformes	Proctophyllodidae	*Proctophyllodes*	FMEP160-14	KP193644
Sarcoptiformes	Proctophyllodidae	*Proctophyllodes*	FMEP161-14	KP193643
Sarcoptiformes	Proctophyllodidae	*Proctophyllodes*	FMEP171-14	KP193652
Sarcoptiformes	Proctophyllodidae	*Proctophyllodes*	FMEP181-14	KP193621
Sarcoptiformes	Proctophyllodidae	*Proctophyllodes*	FMEP182-14	KP193625
Sarcoptiformes	Proctophyllodidae	*Proctophyllodes*	FMEP184-14	KP193728
Sarcoptiformes	Proctophyllodidae	*Proctophyllodes*	FMEP292-14	KP193648
Sarcoptiformes	Proctophyllodidae	*Proctophyllodes*	FMEP303-14	KP193647
Sarcoptiformes	Proctophyllodidae	*Proctophyllodes*	FMEP309-14	KP193624
Sarcoptiformes	Proctophyllodidae	*Proctophyllodes*	FMEP324-14	KP193623

Sarcoptiformes	Proctophyllodidae	*Proctophyllodes*	FMEP350-14	KP193622
Sarcoptiformes	Proctophyllodidae	*Proctophyllodes acanthicaulus*	FMEP112-14	KP193492
Sarcoptiformes	Proctophyllodidae	*Proctophyllodes acanthicaulus*	FMEP113-14	KP193493
Sarcoptiformes	Proctophyllodidae	*Proctophyllodes acanthicaulus*	FMEP114-14	KP193494
Sarcoptiformes	Proctophyllodidae	*Proctophyllodes acanthicaulus*	FMEP115-14	KP193495
Sarcoptiformes	Proctophyllodidae	*Proctophyllodes acanthicaulus*	FMEP116-14	KP193496
Sarcoptiformes	Proctophyllodidae	*Proctophyllodes acanthicaulus*	FMEP198-14	KP193491
Sarcoptiformes	Proctophyllodidae	*Proctophyllodes acanthicaulus*	FMEP199-14	KP193490
Sarcoptiformes	Proctophyllodidae	*Proctophyllodes acanthicaulus*	FMEP200-14	KP193499
Sarcoptiformes	Proctophyllodidae	*Proctophyllodes acanthicaulus*	FMEP201-14	KP193498
Sarcoptiformes	Proctophyllodidae	*Proctophyllodes acanthicaulus*	FMEP232-14	KP193497
Sarcoptiformes	Proctophyllodidae	*Proctophyllodes anthi*	FMEP023-14	KP193503
Sarcoptiformes	Proctophyllodidae	*Proctophyllodes anthi*	FMEP257-14	KP193505
Sarcoptiformes	Proctophyllodidae	*Proctophyllodes anthi*	FMEP258-14	KP193504
Sarcoptiformes	Proctophyllodidae	*Proctophyllodes anthi*	FMEP259-14	KP193500
Sarcoptiformes	Proctophyllodidae	*Proctophyllodes anthi*	FMEP276-14	KP193501
Sarcoptiformes	Proctophyllodidae	*Proctophyllodes anthi*	FMEP277-14	KP193502
Sarcoptiformes	Proctophyllodidae	*Proctophyllodes ateri*	FMEP024-14	KP193507
Sarcoptiformes	Proctophyllodidae	*Proctophyllodes ateri*	FMEP268-14	KP193506
Sarcoptiformes	Proctophyllodidae	*Proctophyllodes ateri*	FMEP269-14	KP193509
Sarcoptiformes	Proctophyllodidae	*Proctophyllodes ateri*	FMEP270-14	KP193510
Sarcoptiformes	Proctophyllodidae	*Proctophyllodes ateri*	FMEP282-14	KP193508
Sarcoptiformes	Proctophyllodidae	*Proctophyllodes caulyfer*	FMEP319-14	KP193511
Sarcoptiformes	Proctophyllodidae	*Proctophyllodes cetti*	FMEP166-14	KP193513
Sarcoptiformes	Proctophyllodidae	*Proctophyllodes cetti*	FMEP167-14	KP193512
Sarcoptiformes	Proctophyllodidae	*Proctophyllodes cetti*	FMEP168-14	KP193515
Sarcoptiformes	Proctophyllodidae	*Proctophyllodes cetti*	FMEP169-14	KP193514
Sarcoptiformes	Proctophyllodidae	*Proctophyllodes cetti*	GBCH3312-09	EU258759
Sarcoptiformes	Proctophyllodidae	*Proctophyllodes cetti*	GBCH3313-09	EU258758
Sarcoptiformes	Proctophyllodidae	*Proctophyllodes cetti*	GBCH3314-09	EU258757
Sarcoptiformes	Proctophyllodidae	*Proctophyllodes cetti*	GBCH3315-09	EU258756
Sarcoptiformes	Proctophyllodidae	*Proctophyllodes clavatus*	FMEP133-14	KP193522
Sarcoptiformes	Proctophyllodidae	*Proctophyllodes clavatus*	FMEP134-14	KP193519
Sarcoptiformes	Proctophyllodidae	*Proctophyllodes clavatus*	FMEP135-14	KP193523
Sarcoptiformes	Proctophyllodidae	*Proctophyllodes clavatus*	FMEP136-14	KP193516
Sarcoptiformes	Proctophyllodidae	*Proctophyllodes clavatus*	FMEP202-14	KP193520
Sarcoptiformes	Proctophyllodidae	*Proctophyllodes clavatus*	FMEP203-14	KP193518
Sarcoptiformes	Proctophyllodidae	*Proctophyllodes clavatus*	FMEP204-14	KP193517
Sarcoptiformes	Proctophyllodidae	*Proctophyllodes clavatus*	FMEP233-14	KP193521
Sarcoptiformes	Proctophyllodidae	*Proctophyllodes cotyledon*	FMEP015-14	KP193524
Sarcoptiformes	Proctophyllodidae	*Proctophyllodes cotyledon*	FMEP117-14	KP193528
Sarcoptiformes	Proctophyllodidae	*Proctophyllodes cotyledon*	FMEP137-14	KP193526
Sarcoptiformes	Proctophyllodidae	*Proctophyllodes cotyledon*	FMEP162-14	KP193525
Sarcoptiformes	Proctophyllodidae	*Proctophyllodes cotyledon*	FMEP246-14	KP193527
Sarcoptiformes	Proctophyllodidae	*Proctophyllodes doleophyes*	FMEP008-14	KP193545
Sarcoptiformes	Proctophyllodidae	*Proctophyllodes doleophyes*	FMEP011-14	KP193544
Sarcoptiformes	Proctophyllodidae	*Proctophyllodes doleophyes*	FMEP014-14	KP193529
Sarcoptiformes	Proctophyllodidae	*Proctophyllodes doleophyes*	FMEP122-14	KP193532
Sarcoptiformes	Proctophyllodidae	*Proctophyllodes doleophyes*	FMEP123-14	KP193533
Sarcoptiformes	Proctophyllodidae	*Proctophyllodes doleophyes*	FMEP124-14	KP193535
Sarcoptiformes	Proctophyllodidae	*Proctophyllodes doleophyes*	FMEP125-14	KP193537
Sarcoptiformes	Proctophyllodidae	*Proctophyllodes doleophyes*	FMEP126-14	KP193540
Sarcoptiformes	Proctophyllodidae	*Proctophyllodes doleophyes*	FMEP127-14	KP193541

Sarcoptiformes	Proctophyllodidae	*Proctophyllodes doleophyes*	FMEP128-14	KP193546
Sarcoptiformes	Proctophyllodidae	*Proctophyllodes doleophyes*	FMEP129-14	KP193536
Sarcoptiformes	Proctophyllodidae	*Proctophyllodes doleophyes*	FMEP130-14	KP193534
Sarcoptiformes	Proctophyllodidae	*Proctophyllodes doleophyes*	FMEP131-14	KP193531
Sarcoptiformes	Proctophyllodidae	*Proctophyllodes doleophyes*	FMEP132-14	KP193530
Sarcoptiformes	Proctophyllodidae	*Proctophyllodes doleophyes*	FMEP163-14	KP193547
Sarcoptiformes	Proctophyllodidae	*Proctophyllodes doleophyes*	FMEP179-14	KP193538
Sarcoptiformes	Proctophyllodidae	*Proctophyllodes doleophyes*	FMEP180-14	KP193539
Sarcoptiformes	Proctophyllodidae	*Proctophyllodes doleophyes*	FMEP185-14	KP193542
Sarcoptiformes	Proctophyllodidae	*Proctophyllodes doleophyes*	FMEP186-14	KP193543
Sarcoptiformes	Proctophyllodidae	*Proctophyllodes fuchsi*	FMEP020-14	KP193548
Sarcoptiformes	Proctophyllodidae	*Proctophyllodes glandarinus*	FMEP021-14	KP193549
Sarcoptiformes	Proctophyllodidae	*Proctophyllodes glandarinus*	FMEP271-14	KP193550
Sarcoptiformes	Proctophyllodidae	*Proctophyllodes hipposideros*	FMEP190-14	KP193553
Sarcoptiformes	Proctophyllodidae	*Proctophyllodes hipposideros*	FMEP234-14	KP193555
Sarcoptiformes	Proctophyllodidae	*Proctophyllodes hipposideros*	FMEP235-14	KP193554
Sarcoptiformes	Proctophyllodidae	*Proctophyllodes hipposideros*	FMEP236-14	KP193552
Sarcoptiformes	Proctophyllodidae	*Proctophyllodes hipposideros*	FMEP237-14	KP193551
Sarcoptiformes	Proctophyllodidae	*Proctophyllodes leptocaulus*	FMEP030-14	KP193557
Sarcoptiformes	Proctophyllodidae	*Proctophyllodes leptocaulus*	FMEP320-14	KP193558
Sarcoptiformes	Proctophyllodidae	*Proctophyllodes leptocaulus*	FMEP355-14	KP193556
Sarcoptiformes	Proctophyllodidae	*Proctophyllodes lusciniae*	FMEP002-14	KP193580
Sarcoptiformes	Proctophyllodidae	*Proctophyllodes lusciniae*	FMEP044-14	KP193560
Sarcoptiformes	Proctophyllodidae	*Proctophyllodes lusciniae*	FMEP045-14	KP193561
Sarcoptiformes	Proctophyllodidae	*Proctophyllodes lusciniae*	FMEP046-14	KP193568
Sarcoptiformes	Proctophyllodidae	*Proctophyllodes lusciniae*	FMEP047-14	KP193569
Sarcoptiformes	Proctophyllodidae	*Proctophyllodes lusciniae*	FMEP048-14	KP193570
Sarcoptiformes	Proctophyllodidae	*Proctophyllodes lusciniae*	FMEP049-14	KP193571
Sarcoptiformes	Proctophyllodidae	*Proctophyllodes lusciniae*	FMEP050-14	KP193572
Sarcoptiformes	Proctophyllodidae	*Proctophyllodes lusciniae*	FMEP051-14	KP193573
Sarcoptiformes	Proctophyllodidae	*Proctophyllodes lusciniae*	FMEP052-14	KP193574
Sarcoptiformes	Proctophyllodidae	*Proctophyllodes lusciniae*	FMEP053-14	KP193579
Sarcoptiformes	Proctophyllodidae	*Proctophyllodes lusciniae*	FMEP063-14	KP193578
Sarcoptiformes	Proctophyllodidae	*Proctophyllodes lusciniae*	FMEP064-14	KP193576
Sarcoptiformes	Proctophyllodidae	*Proctophyllodes lusciniae*	FMEP065-14	KP193577
Sarcoptiformes	Proctophyllodidae	*Proctophyllodes lusciniae*	FMEP107-14	KP193567
Sarcoptiformes	Proctophyllodidae	*Proctophyllodes lusciniae*	FMEP108-14	KP193566
Sarcoptiformes	Proctophyllodidae	*Proctophyllodes lusciniae*	FMEP109-14	KP193565
Sarcoptiformes	Proctophyllodidae	*Proctophyllodes lusciniae*	FMEP205-14	KP193562
Sarcoptiformes	Proctophyllodidae	*Proctophyllodes lusciniae*	FMEP206-14	KP193559
Sarcoptiformes	Proctophyllodidae	*Proctophyllodes lusciniae*	FMEP207-14	KP193563
Sarcoptiformes	Proctophyllodidae	*Proctophyllodes lusciniae*	FMEP208-14	KP193575
Sarcoptiformes	Proctophyllodidae	*Proctophyllodes lusciniae*	FMEP242-14	KP193564
Sarcoptiformes	Proctophyllodidae	*Proctophyllodes macedo*	FMEP272-14	KP193581
Sarcoptiformes	Proctophyllodidae	*Proctophyllodes macedo*	FMEP352-14	KP193582
Sarcoptiformes	Proctophyllodidae	*Proctophyllodes macedo*	FMEP353-14	KP193583
Sarcoptiformes	Proctophyllodidae	*Proctophyllodes megaphyllus*	FMEP273-14	KP193584
Sarcoptiformes	Proctophyllodidae	*Proctophyllodes mesocaulus*	FMEP101-14	KP193588
Sarcoptiformes	Proctophyllodidae	*Proctophyllodes mesocaulus*	FMEP104-14	KP193587
Sarcoptiformes	Proctophyllodidae	*Proctophyllodes mesocaulus*	FMEP118-14	KP193594
Sarcoptiformes	Proctophyllodidae	*Proctophyllodes mesocaulus*	FMEP119-14	KP193593
Sarcoptiformes	Proctophyllodidae	*Proctophyllodes mesocaulus*	FMEP120-14	KP193592
Sarcoptiformes	Proctophyllodidae	*Proctophyllodes mesocaulus*	FMEP121-14	KP193591

Sarcoptiformes	Proctophyllodidae	*Proctophyllodes mesocaulus*	FMEP189-14	KP193590
Sarcoptiformes	Proctophyllodidae	*Proctophyllodes mesocaulus*	FMEP191-14	KP193595
Sarcoptiformes	Proctophyllodidae	*Proctophyllodes mesocaulus*	FMEP228-14	KP193589
Sarcoptiformes	Proctophyllodidae	*Proctophyllodes mesocaulus*	FMEP238-14	KP193586
Sarcoptiformes	Proctophyllodidae	*Proctophyllodes mesocaulus*	FMEP239-14	KP193585
Sarcoptiformes	Proctophyllodidae	*Proctophyllodes mesocaulus*	FMEP240-14	KP193597
Sarcoptiformes	Proctophyllodidae	*Proctophyllodes mesocaulus*	FMEP241-14	KP193596
Sarcoptiformes	Proctophyllodidae	*Proctophyllodes miliariae*	FMEP274-14	KP193599
Sarcoptiformes	Proctophyllodidae	*Proctophyllodes miliariae*	FMEP286-14	KP193598
Sarcoptiformes	Proctophyllodidae	*Proctophyllodes motacillae*	FMEP005-14	KP193605
Sarcoptiformes	Proctophyllodidae	*Proctophyllodes motacillae*	FMEP072-14	KP193604
Sarcoptiformes	Proctophyllodidae	*Proctophyllodes motacillae*	FMEP073-14	KP193606
Sarcoptiformes	Proctophyllodidae	*Proctophyllodes motacillae*	FMEP074-14	KP193601
Sarcoptiformes	Proctophyllodidae	*Proctophyllodes motacillae*	FMEP075-14	KP193610
Sarcoptiformes	Proctophyllodidae	*Proctophyllodes motacillae*	FMEP077-14	KP193611
Sarcoptiformes	Proctophyllodidae	*Proctophyllodes motacillae*	FMEP078-14	KP193603
Sarcoptiformes	Proctophyllodidae	*Proctophyllodes motacillae*	FMEP079-14	KP193609
Sarcoptiformes	Proctophyllodidae	*Proctophyllodes motacillae*	FMEP080-14	KP193600
Sarcoptiformes	Proctophyllodidae	*Proctophyllodes motacillae*	FMEP090-14	KP193608
Sarcoptiformes	Proctophyllodidae	*Proctophyllodes motacillae*	FMEP091-14	KP193607
Sarcoptiformes	Proctophyllodidae	*Proctophyllodes motacillae*	FMEP209-14	KP193613
Sarcoptiformes	Proctophyllodidae	*Proctophyllodes motacillae*	FMEP210-14	KP193612
Sarcoptiformes	Proctophyllodidae	*Proctophyllodes motacillae*	FMEP243-14	KP193602
Sarcoptiformes	Proctophyllodidae	*Proctophyllodes musicus*	FMEP018-14	KP193616
Sarcoptiformes	Proctophyllodidae	*Proctophyllodes musicus*	FMEP211-14	KP193618
Sarcoptiformes	Proctophyllodidae	*Proctophyllodes musicus*	FMEP212-14	KP193619
Sarcoptiformes	Proctophyllodidae	*Proctophyllodes musicus*	FMEP213-14	KP193614
Sarcoptiformes	Proctophyllodidae	*Proctophyllodes musicus*	FMEP214-14	KP193615
Sarcoptiformes	Proctophyllodidae	*Proctophyllodes musicus*	FMEP244-14	KP193620
Sarcoptiformes	Proctophyllodidae	*Proctophyllodes musicus*	FMEP245-14	KP193617
Sarcoptiformes	Proctophyllodidae	*Proctophyllodes pinnatus*	FMEP172-14	KP193651
Sarcoptiformes	Proctophyllodidae	*Proctophyllodes pinnatus*	FMEP174-14	KP193649
Sarcoptiformes	Proctophyllodidae	*Proctophyllodes pinnatus*	FMEP297-14	KP193634
Sarcoptiformes	Proctophyllodidae	*Proctophyllodes poublani*	FMEP321-14	KP193657
Sarcoptiformes	Proctophyllodidae	*Proctophyllodes poublani*	FMEP322-14	KP193659
Sarcoptiformes	Proctophyllodidae	*Proctophyllodes poublani*	FMEP354-14	KP193658
Sarcoptiformes	Proctophyllodidae	*Proctophyllodes reguli*	FMEP028-14	KP193661
Sarcoptiformes	Proctophyllodidae	*Proctophyllodes reguli*	FMEP299-14	KP193660
Sarcoptiformes	Proctophyllodidae	*Proctophyllodes remizicola*	FMEP283-14	KP193662
Sarcoptiformes	Proctophyllodidae	*Proctophyllodes remizicola*	FMEP287-14	KP193663
Sarcoptiformes	Proctophyllodidae	*Proctophyllodes rubeculinus*	FMEP004-14	KP193664
Sarcoptiformes	Proctophyllodidae	*Proctophyllodes rubeculinus*	FMEP010-14	KP193672
Sarcoptiformes	Proctophyllodidae	*Proctophyllodes rubeculinus*	FMEP067-14	KP193666
Sarcoptiformes	Proctophyllodidae	*Proctophyllodes rubeculinus*	FMEP098-14	KP193671
Sarcoptiformes	Proctophyllodidae	*Proctophyllodes rubeculinus*	FMEP175-14	KP193670
Sarcoptiformes	Proctophyllodidae	*Proctophyllodes rubeculinus*	FMEP177-14	KP193668
Sarcoptiformes	Proctophyllodidae	*Proctophyllodes rubeculinus*	FMEP215-14	KP193669
Sarcoptiformes	Proctophyllodidae	*Proctophyllodes rubeculinus*	FMEP216-14	KP193667
Sarcoptiformes	Proctophyllodidae	*Proctophyllodes rubeculinus*	FMEP217-14	KP193673
Sarcoptiformes	Proctophyllodidae	*Proctophyllodes rubeculinus*	FMEP218-14	KP193674
Sarcoptiformes	Proctophyllodidae	*Proctophyllodes rubeculinus*	FMEP359-14	KP193665
Sarcoptiformes	Proctophyllodidae	*Proctophyllodes rubeculinus*	FMEP360-14	KP193676
Sarcoptiformes	Proctophyllodidae	*Proctophyllodes rubeculinus*	FMEP361-14	KP193675

Sarcoptiformes	Proctophyllodidae	*Proctophyllodes schoenicli*	FMEP022-14	KP193679
Sarcoptiformes	Proctophyllodidae	*Proctophyllodes schoenicli*	FMEP247-14	KP193684
Sarcoptiformes	Proctophyllodidae	*Proctophyllodes schoenicli*	FMEP248-14	KP193685
Sarcoptiformes	Proctophyllodidae	*Proctophyllodes schoenicli*	FMEP249-14	KP193681
Sarcoptiformes	Proctophyllodidae	*Proctophyllodes schoenicli*	FMEP250-14	KP193682
Sarcoptiformes	Proctophyllodidae	*Proctophyllodes schoenicli*	FMEP251-14	KP193683
Sarcoptiformes	Proctophyllodidae	*Proctophyllodes schoenicli*	FMEP252-14	KP193677
Sarcoptiformes	Proctophyllodidae	*Proctophyllodes schoenicli*	FMEP253-14	KP193678
Sarcoptiformes	Proctophyllodidae	*Proctophyllodes schoenicli*	FMEP275-14	KP193680
Sarcoptiformes	Proctophyllodidae	*Proctophyllodes schwerinensis*	FMEP288-14	KP193687
Sarcoptiformes	Proctophyllodidae	*Proctophyllodes schwerinensis*	FMEP289-14	KP193688
Sarcoptiformes	Proctophyllodidae	*Proctophyllodes schwerinensis*	FMEP300-14	KP193686
Sarcoptiformes	Proctophyllodidae	*Proctophyllodes simillimus*	FMEP029-14	KP193698
Sarcoptiformes	Proctophyllodidae	*Proctophyllodes simillimus*	FMEP290-14	KP193699
Sarcoptiformes	Proctophyllodidae	*Proctophyllodes simillimus*	FMEP291-14	KP193700
Sarcoptiformes	Proctophyllodidae	*Proctophyllodes sp. AMUFM909*	GBCH4470-10	EU371933
Sarcoptiformes	Proctophyllodidae	*Proctophyllodes spini*	FMEP173-14	KP193702
Sarcoptiformes	Proctophyllodidae	*Proctophyllodes spini*	FMEP285-14	KP193703
Sarcoptiformes	Proctophyllodidae	*Proctophyllodes spini*	FMEP298-14	KP193701
Sarcoptiformes	Proctophyllodidae	*Proctophyllodes stylifer*	FMEP019-14	KP193714
Sarcoptiformes	Proctophyllodidae	*Proctophyllodes stylifer*	FMEP039-14	KP193716
Sarcoptiformes	Proctophyllodidae	*Proctophyllodes stylifer*	FMEP183-14	KP193705
Sarcoptiformes	Proctophyllodidae	*Proctophyllodes stylifer*	FMEP219-14	KP193712
Sarcoptiformes	Proctophyllodidae	*Proctophyllodes stylifer*	FMEP220-14	KP193710
Sarcoptiformes	Proctophyllodidae	*Proctophyllodes stylifer*	FMEP221-14	KP193711
Sarcoptiformes	Proctophyllodidae	*Proctophyllodes stylifer*	FMEP222-14	KP193707
Sarcoptiformes	Proctophyllodidae	*Proctophyllodes stylifer*	FMEP223-14	KP193708
Sarcoptiformes	Proctophyllodidae	*Proctophyllodes stylifer*	FMEP230-14	KP193709
Sarcoptiformes	Proctophyllodidae	*Proctophyllodes stylifer*	FMEP231-14	KP193715
Sarcoptiformes	Proctophyllodidae	*Proctophyllodes stylifer*	FMEP356-14	KP193713
Sarcoptiformes	Proctophyllodidae	*Proctophyllodes stylifer*	FMEP357-14	KP193704
Sarcoptiformes	Proctophyllodidae	*Proctophyllodes stylifer*	FMEP358-14	KP193706
Sarcoptiformes	Proctophyllodidae	*Proctophyllodes sylviae*	FMEP016-14	KP193719
Sarcoptiformes	Proctophyllodidae	*Proctophyllodes sylviae*	FMEP093-14	KP193722
Sarcoptiformes	Proctophyllodidae	*Proctophyllodes sylviae*	FMEP094-14	KP193717
Sarcoptiformes	Proctophyllodidae	*Proctophyllodes sylviae*	FMEP095-14	KP193725
Sarcoptiformes	Proctophyllodidae	*Proctophyllodes sylviae*	FMEP165-14	KP193721
Sarcoptiformes	Proctophyllodidae	*Proctophyllodes sylviae*	FMEP187-14	KP193720
Sarcoptiformes	Proctophyllodidae	*Proctophyllodes sylviae*	FMEP224-14	KP193724
Sarcoptiformes	Proctophyllodidae	*Proctophyllodes sylviae*	FMEP225-14	KP193723
Sarcoptiformes	Proctophyllodidae	*Proctophyllodes sylviae*	FMEP318-14	KP193718
Sarcoptiformes	Proctophyllodidae	*Proctophyllodes tenericaulus*	FMEP323-14	KP193726
Sarcoptiformes	Proctophyllodidae	*Proctophyllodes valchukae*	GACAC1193-12	JN936871
Sarcoptiformes	Proctophyllodidae	*Proctophyllodes valchukae*	GACAC1194-12	JN936872
Sarcoptiformes	Proctophyllodidae	*Proctophyllodes valchukae*	GACAC1195-12	JN936873
Sarcoptiformes	Proctophyllodidae	*Proctophyllodes valchukae*	GACAC1196-12	JN936874
Sarcoptiformes	Proctophyllodidae	*Proctophyllodes valchukae*	GBCH11624-13	JN936875
Sarcoptiformes	Proctophyllodidae	*Proctophyllodes vassilevi*	FMEP293-14	KP193743
Sarcoptiformes	Proctophyllodidae	*Proctophyllodes vassilevi*	FMEP294-14	KP193744
Sarcoptiformes	Proctophyllodidae	*Proctophyllodes vegetans*	FMEP301-14	KP193745
Sarcoptiformes	Proctophyllodidae	*Proctophyllodes vitzthumi*	FMEP025-14	KP193746
Sarcoptiformes	Proctophyllodidae	*Proctophyllodes volgini*	FMEP302-14	KP193747
Sarcoptiformes	Proctophyllodidae	*Proctophyllodes weigoldi*	FMEP295-14	KP193749

Sarcoptiformes	Proctophyllodidae	*Proctophyllodes weigoldi*	FMEP304-14	KP193748
Sarcoptiformes	Proctophyllodidae	*Pterodectes rutilus*	FMEP325-14	KP193752
Sarcoptiformes	Proctophyllodidae	*Pterodectes rutilus*	FMEP326-14	KP193753
Sarcoptiformes	Proctophyllodidae	*Pterodectes rutilus*	FMEP327-14	KP193750
Sarcoptiformes	Proctophyllodidae	*Pterodectes rutilus*	FMEP328-14	KP193751
Sarcoptiformes	Psoroptidae	*Chorioptes sp. SW-2010a*	GBCH4531-10	FJ907504
Sarcoptiformes	Psoroptidae	*Psoroptes cuniculi*	GBA16295-14	KJ957822
Sarcoptiformes	Psoroptidae	*Psoroptes cuniculi*	GBCH4532-10	FJ907499
Sarcoptiformes	Pterolichidae	*Grallobia fulicae*	GBCH4468-10	GQ864333
Sarcoptiformes	Pterolichidae	*Xoloptes blaszaki*	GBCH4452-10	GQ864350
Sarcoptiformes	Pteronyssidae	*Pteronyssoides motacillae*	FMEP076-14	KP193754
Sarcoptiformes	Pteronyssidae	*Pteronyssoides parinus*	FMEP229-14	KP193757
Sarcoptiformes	Pteronyssidae	*Pteronyssoides parinus*	FMEP296-14	KP193756
Sarcoptiformes	Pteronyssidae	*Pteronyssoides parinus*	FMEP305-14	KP193755
Sarcoptiformes	Pteronyssidae	*Pteronyssoides piscinotus*	FMEP306-14	KP193759
Sarcoptiformes	Pteronyssidae	*Pteronyssoides piscinotus*	FMEP307-14	KP193758
Sarcoptiformes	Pteronyssidae	*Pteronyssoides striatus*	FMEP329-14	KP193760
Sarcoptiformes	Pteronyssidae	*Pteronyssoides striatus*	GBCH4447-10	GQ864355
Sarcoptiformes	Pteronyssidae	*Scutulanyssus hirundicola*	FMEP027-14	KP193762
Sarcoptiformes	Pteronyssidae	*Scutulanyssus hirundicola*	FMEP308-14	KP193761
Sarcoptiformes	Pteronyssidae	*Scutulanyssus obscurus*	FMEP310-14	KP193763
Sarcoptiformes	Pteronyssidae	*Sturnotrogus*	FMEP330-14	KP193764
Sarcoptiformes	Pteronyssidae	*Sturnotrogus truncatus*	GBCH4444-10	GQ864358
Sarcoptiformes	Pyroglyphidae	*Dermatophagoides farinae*	CYTC3361-12	NC_013184
Sarcoptiformes	Pyroglyphidae	*Dermatophagoides farinae*	GBCH5654-13	GQ465336
Sarcoptiformes	Pyroglyphidae	*Dermatophagoides pteronyssinus*	CYTC4724-12	GQ469891
Sarcoptiformes	Pyroglyphidae	*Dermatophagoides pteronyssinus*	GBCH3917-09	NC_012218
Sarcoptiformes	Pyroglyphidae	*Dermatophagoides pteronyssinus*	GBCH3949-09	EU884425
Sarcoptiformes	Pyroglyphidae	*Euroglyphus sp. AMUEUR1506*	GBCH4456-10	GQ864346
Sarcoptiformes	Quadroppiidae	*Quadroppia*	CHACA1084-10	HM907125
Sarcoptiformes	Quadroppiidae	*Quadroppia*	CHACA1085-10	HM907126
Sarcoptiformes	Quadroppiidae	*Quadroppia*	CHACB171-10	HQ558452
Sarcoptiformes	Quadroppiidae	*Quadroppia*	MYMCA1262-11	JX835662
Sarcoptiformes	Quadroppiidae	*Quadroppia*	MYMCA1434-11	JX837021
Sarcoptiformes	Quadroppiidae	*Quadroppia*	MYMCA1435-11	JX835886
Sarcoptiformes	Quadroppiidae	*Quadroppia*	MYMCA1519-11	JX834047
Sarcoptiformes	Quadroppiidae	*Quadroppia*	MYMCE377-12	JX834979
Sarcoptiformes	Quadroppiidae	*Quadroppia*	MYMCE462-12	JX835010
Sarcoptiformes	Quadroppiidae	*Quadroppia*	MYMCE466-12	JX836487
Sarcoptiformes	Quadroppiidae	*Quadroppia*	MYMCE474-12	JX837062
Sarcoptiformes	Quadroppiidae	*Quadroppia*	MYMCE648-12	JX837338
Sarcoptiformes	Quadroppiidae	*Quadroppia*	MYMCE655-12	JX837906
Sarcoptiformes	Quadroppiidae	*Quadroppia*	MYMCE656-12	JX834687
Sarcoptiformes	Quadroppiidae	*Quadroppia*	MYMCE657-12	JX837194
Sarcoptiformes	Quadroppiidae	*Quadroppia*	MYMCF243-12	JX834192
Sarcoptiformes	Quadroppiidae	*Quadroppia*	MYMCF329-12	JX834696
Sarcoptiformes	Quadroppiidae	*Quadroppia*	MYMCF505-12	JX836380
Sarcoptiformes	Quadroppiidae	*Quadroppia*	MYMCF525-12	JX837019
Sarcoptiformes	Quadroppiidae	*Quadroppia*	MYMCF526-12	JX835876
Sarcoptiformes	Quadroppiidae	*Quadroppia*	MYMCF527-12	JX837058
Sarcoptiformes	Quadroppiidae	*Quadroppia*	MYMCF528-12	JX834037
Sarcoptiformes	Quadroppiidae	*Quadroppia*	MYMCG466-12	JX837374
Sarcoptiformes	Quadroppiidae	*Quadroppia*	MYMCG482-12	JX833977

Sarcoptiformes	Sarcoptidae	*Sarcoptes scabiei*	GBA16271-14	KJ748527
Sarcoptiformes	Sarcoptidae	*Sarcoptes scabiei*	GBA16272-14	KJ748528
Sarcoptiformes	Sarcoptidae	*Sarcoptes scabiei*	GBA16277-14	KJ499544
Sarcoptiformes	Scheloribatidae	*Scheloribates*	CHACA1114-10	JX836556
Sarcoptiformes	Scheloribatidae	*Scheloribates*	CHACA1202-10	JX834088
Sarcoptiformes	Scheloribatidae	*Scheloribates*	CHACB044-10	HQ558356
Sarcoptiformes	Scheloribatidae	*Scheloribates*	CHACB045-10	HQ558357
Sarcoptiformes	Scheloribatidae	*Scheloribates*	CHACB060-10	HQ558368
Sarcoptiformes	Scheloribatidae	*Scheloribates*	CHACB1179-10	HM907254
Sarcoptiformes	Scheloribatidae	*Scheloribates*	CHACB1180-10	HM907255
Sarcoptiformes	Scheloribatidae	*Scheloribates*	CHACB1181-10	HM907256
Sarcoptiformes	Scheloribatidae	*Scheloribates*	CHACB151-10	HQ558434
Sarcoptiformes	Scheloribatidae	*Scheloribates*	CHACB859-10	HM907311
Sarcoptiformes	Scheloribatidae	*Scheloribates*	CHACC005-10	HM907407
Sarcoptiformes	Scheloribatidae	*Scheloribates*	CHACC200-10	HQ941526
Sarcoptiformes	Scheloribatidae	*Scheloribates*	CNPCD077-13	KM840143
Sarcoptiformes	Scheloribatidae	*Scheloribates*	MYMCA1019-11	JX837135
Sarcoptiformes	Scheloribatidae	*Scheloribates*	MYMCA1020-11	JX835864
Sarcoptiformes	Scheloribatidae	*Scheloribates*	MYMCA1130-11	JX834446
Sarcoptiformes	Scheloribatidae	*Scheloribates*	MYMCA1162-11	JX838320
Sarcoptiformes	Scheloribatidae	*Scheloribates*	MYMCA1163-11	JX834919
Sarcoptiformes	Scheloribatidae	*Scheloribates*	MYMCA1393-11	JX835189
Sarcoptiformes	Scheloribatidae	*Scheloribates*	MYMCA1442-11	JX834893
Sarcoptiformes	Scheloribatidae	*Scheloribates*	MYMCA1443-11	JX835170
Sarcoptiformes	Scheloribatidae	*Scheloribates*	MYMCA1444-11	JX837935
Sarcoptiformes	Scheloribatidae	*Scheloribates*	MYMCA294-11	JX837923
Sarcoptiformes	Scheloribatidae	*Scheloribates*	MYMCA295-11	JX838608
Sarcoptiformes	Scheloribatidae	*Scheloribates*	MYMCA296-11	JX836321
Sarcoptiformes	Scheloribatidae	*Scheloribates*	MYMCA406-11	JX836547
Sarcoptiformes	Scheloribatidae	*Scheloribates*	MYMCA507-11	JX837868
Sarcoptiformes	Scheloribatidae	*Scheloribates*	MYMCA592-11	JX833974
Sarcoptiformes	Scheloribatidae	*Scheloribates*	MYMCA651-11	JX836065
Sarcoptiformes	Scheloribatidae	*Scheloribates*	MYMCA694-11	JX838739
Sarcoptiformes	Scheloribatidae	*Scheloribates*	MYMCA728-11	JX836816
Sarcoptiformes	Scheloribatidae	*Scheloribates*	MYMCA911-11	JX838291
Sarcoptiformes	Scheloribatidae	*Scheloribates*	MYMCA923-11	JX833789
Sarcoptiformes	Scheloribatidae	*Scheloribates*	MYMCB114-11	JX836102
Sarcoptiformes	Scheloribatidae	*Scheloribates*	MYMCB115-11	JX837614
Sarcoptiformes	Scheloribatidae	*Scheloribates*	MYMCB234-11	JX836406
Sarcoptiformes	Scheloribatidae	*Scheloribates*	MYMCB244-11	JX837756
Sarcoptiformes	Scheloribatidae	*Scheloribates*	MYMCB245-11	JX838359
Sarcoptiformes	Scheloribatidae	*Scheloribates*	MYMCB247-11	JX833691
Sarcoptiformes	Scheloribatidae	*Scheloribates*	MYMCB262-11	JX837745
Sarcoptiformes	Scheloribatidae	*Scheloribates*	MYMCB798-11	JX837986
Sarcoptiformes	Scheloribatidae	*Scheloribates*	MYMCB856-11	JX835578
Sarcoptiformes	Scheloribatidae	*Scheloribates*	MYMCC056-11	JX836459
Sarcoptiformes	Scheloribatidae	*Scheloribates*	MYMCC057-11	JX838753
Sarcoptiformes	Scheloribatidae	*Scheloribates*	MYMCC628-11	JX837507
Sarcoptiformes	Scheloribatidae	*Scheloribates*	MYMCC637-11	JX835870
Sarcoptiformes	Scheloribatidae	*Scheloribates*	MYMCC873-11	JX836460
Sarcoptiformes	Scheloribatidae	*Scheloribates*	MYMCE783-12	JX834060
Sarcoptiformes	Scheloribatidae	*Scheloribates*	MYMCF024-12	JX834239
Sarcoptiformes	Scheloribatidae	*Scheloribates*	MYMCF300-12	JX833626

Sarcoptiformes	Scheloribatidae	*Scheloribates*	MYMCF305-12	JX836939
Sarcoptiformes	Scheloribatidae	*Scheloribates*	MYMCF306-12	JX834456
Sarcoptiformes	Scheloribatidae	*Scheloribates*	MYMCF446-12	JX834744
Sarcoptiformes	Scheloribatidae	*Scheloribates*	MYMCF447-12	JX835925
Sarcoptiformes	Scheloribatidae	*Scheloribates*	MYMCF448-12	JX835981
Sarcoptiformes	Scheloribatidae	*Scheloribates*	MYMCF667-12	JX833996
Sarcoptiformes	Scheloribatidae	*Scheloribates*	MYMCF764-12	JX835210
Sarcoptiformes	Scheloribatidae	*Scheloribates*	MYMCF840-12	JX835282
Sarcoptiformes	Scheloribatidae	*Scheloribates*	MYMCG137-12	JX836533
Sarcoptiformes	Scheloribatidae	*Scheloribates*	MYMCG273-12	JX836674
Sarcoptiformes	Scheloribatidae	*Scheloribates*	MYMCG480-12	JX836839
Sarcoptiformes	Scheloribatidae	*Scheloribates*	SSPAA8015-13	KM827987
Sarcoptiformes	Scheloribatidae	*Scheloribates pallidulus*	CHACA1112-10	JX834560
Sarcoptiformes	Scheloribatidae	*Scheloribates pallidulus*	CHACA1113-10	JX835879
Sarcoptiformes	Scheloribatidae	*Scheloribates pallidulus*	CHACA1115-10	JX838587
Sarcoptiformes	Scheloribatidae	*Scheloribates pallidulus*	CHACA1116-10	JX836867
Sarcoptiformes	Scheloribatidae	*Scheloribates pallidulus*	CHACB042-10	HQ558355
Sarcoptiformes	Scheloribatidae	*Scheloribates pallidulus*	CHACB1127-10	HM907219
Sarcoptiformes	Scheloribatidae	*Scheloribates pallidulus*	CHACB1177-10	KR069227
Sarcoptiformes	Scheloribatidae	*Scheloribates pallidulus*	CHACB1178-10	HM907253
Sarcoptiformes	Scheloribatidae	*Scheloribates pallidulus*	CHACB697-10	HQ558754
Sarcoptiformes	Scheloribatidae	*Scheloribates pallidulus*	CHACC003-10	HM907405
Sarcoptiformes	Scheloribatidae	*Scheloribates pallidulus*	CHACC004-10	HM907406
Sarcoptiformes	Scheloribatidae	*Scheloribates pallidulus*	CHACC201-10	HQ941527
Sarcoptiformes	Scheloribatidae	*Scheloribates pallidulus*	MYMCA1018-11	JX838515
Sarcoptiformes	Scheloribatidae	*Scheloribates pallidulus*	MYMCA1022-11	JX838259
Sarcoptiformes	Scheloribatidae	*Scheloribates pallidulus*	MYMCA1278-11	JX834014
Sarcoptiformes	Scheloribatidae	*Scheloribates pallidulus*	MYMCA1279-11	JX836909
Sarcoptiformes	Scheloribatidae	*Scheloribates pallidulus*	MYMCA1282-11	JX833646
Sarcoptiformes	Scheloribatidae	*Scheloribates pallidulus*	MYMCA1395-11	JX838552
Sarcoptiformes	Scheloribatidae	*Scheloribates pallidulus*	MYMCA1439-11	JX833874
Sarcoptiformes	Scheloribatidae	*Scheloribates pallidulus*	MYMCA1494-11	JX836587
Sarcoptiformes	Scheloribatidae	*Scheloribates pallidulus*	MYMCA1495-11	JX837982
Sarcoptiformes	Scheloribatidae	*Scheloribates pallidulus*	MYMCA1496-11	JX836428
Sarcoptiformes	Scheloribatidae	*Scheloribates pallidulus*	MYMCA256-11	JX836870
Sarcoptiformes	Scheloribatidae	*Scheloribates pallidulus*	MYMCA257-11	JX834388
Sarcoptiformes	Scheloribatidae	*Scheloribates pallidulus*	MYMCA508-11	JX835830
Sarcoptiformes	Scheloribatidae	*Scheloribates pallidulus*	MYMCA509-11	JX837626
Sarcoptiformes	Scheloribatidae	*Scheloribates pallidulus*	MYMCA652-11	JX833625
Sarcoptiformes	Scheloribatidae	*Scheloribates pallidulus*	MYMCA983-11	JX837500
Sarcoptiformes	Scheloribatidae	*Scheloribates pallidulus*	MYMCB010-11	JX837171
Sarcoptiformes	Scheloribatidae	*Scheloribates pallidulus*	MYMCB581-11	JX833863
Sarcoptiformes	Scheloribatidae	*Scheloribates pallidulus*	MYMCB710-11	JX835695
Sarcoptiformes	Scheloribatidae	*Scheloribates pallidulus*	MYMCB711-11	JX837696
Sarcoptiformes	Scheloribatidae	*Scheloribates pallidulus*	MYMCB745-11	JX834077
Sarcoptiformes	Scheloribatidae	*Scheloribates pallidulus*	MYMCB780-11	JX836028
Sarcoptiformes	Scheloribatidae	*Scheloribates pallidulus*	MYMCB781-11	JX836686
Sarcoptiformes	Scheloribatidae	*Scheloribates pallidulus*	MYMCB799-11	JX836899
Sarcoptiformes	Scheloribatidae	*Scheloribates pallidulus*	MYMCC089-11	JX836683
Sarcoptiformes	Scheloribatidae	*Scheloribates pallidulus*	MYMCC193-11	JX833649
Sarcoptiformes	Scheloribatidae	*Scheloribates pallidulus*	MYMCC439-11	JX834078
Sarcoptiformes	Scheloribatidae	*Scheloribates pallidulus*	MYMCC463-11	JX837015
Sarcoptiformes	Scheloribatidae	*Scheloribates pallidulus*	MYMCC597-11	JX833701

Sarcoptiformes	Scheloribatidae	*Scheloribates pallidulus*	MYMCC598-11	JX836622
Sarcoptiformes	Scheloribatidae	*Scheloribates pallidulus*	MYMCC736-11	JX838638
Sarcoptiformes	Scheloribatidae	*Scheloribates pallidulus*	MYMCC820-11	JX835526
Sarcoptiformes	Scheloribatidae	*Scheloribates pallidulus*	MYMCE347-12	JX838520
Sarcoptiformes	Scheloribatidae	*Scheloribates pallidulus*	MYMCE349-12	JX833849
Sarcoptiformes	Scheloribatidae	*Scheloribates pallidulus*	MYMCE353-12	JX835799
Sarcoptiformes	Scheloribatidae	*Scheloribates pallidulus*	MYMCE813-12	JX836515
Sarcoptiformes	Scheloribatidae	*Scheloribates pallidulus*	MYMCE834-12	JX835765
Sarcoptiformes	Scheloribatidae	*Scheloribates pallidulus*	MYMCF113-12	JX834109
Sarcoptiformes	Scheloribatidae	*Scheloribates pallidulus*	MYMCF164-12	JX838624
Sarcoptiformes	Scheloribatidae	*Scheloribates pallidulus*	MYMCF839-12	JX837908
Sarcoptiformes	Scheloribatidae	*Scheloribates pallidulus*	MYMCG136-12	JX836270
Sarcoptiformes	Scheloribatidae	*Scheloribates pallidulus*	MYMCG138-12	JX835435
Sarcoptiformes	Scheloribatidae	*Scheloribates pallidulus*	MYMCG283-12	JX835338
Sarcoptiformes	Scheloribatidae	*Scheloribates pallidulus*	MYMCG543-12	JX836543
Sarcoptiformes	Scheloribatidae	*Scheloribates pallidulus*	MYMCG593-12	JX836281
Sarcoptiformes	Scheloribatidae	*Scheloribates pallidulus*	MYTMC137-09	GU680452
Sarcoptiformes	Scheloribatidae	*Scheloribates pallidulus*	MYTMC160-09	GU680444
Sarcoptiformes	Scheloribatidae	*Scheloribates pallidulus*	SSPAA7934-13	KM839103
Sarcoptiformes	Scheloribatidae	*Scheloribates pallidulus*	SSPAA7973-13	KM838115
Sarcoptiformes	Scheloribatidae	*Scheloribates pallidulus*	SSPAA7975-13	KM836085
Sarcoptiformes	Scheloribatidae	*Scheloribates pallidulus*	SSPAA8016-13	KM837804
Sarcoptiformes	Scheloribatidae	*Scheloribates pallidulus*	SSPAA8018-13	KM828743
Sarcoptiformes	Scheloribatidae	*Scheloribates pallidulus*	SSPAA8020-13	KM827170
Sarcoptiformes	Scheloribatidae	*Scheloribates pallidulus*	SSPAA8022-13	KM824436
Sarcoptiformes	Scheloribatidae	*Scheloribates pallidulus*	SSPAA8023-13	KM839997
Sarcoptiformes	Scheloribatidae		CNBPB541-12	KM835987
Sarcoptiformes	Scheloribatidae		CNBPB542-12	KM824150
Sarcoptiformes	Scheloribatidae		CNBPB549-12	KM830143
Sarcoptiformes	Scheloribatidae		CNBPD603-12	KM831577
Sarcoptiformes	Scheloribatidae		CNBPF134-12	KM833709
Sarcoptiformes	Scheloribatidae		CNBPK327-13	KM839840
Sarcoptiformes	Scheloribatidae		CNBPK335-13	KM840470
Sarcoptiformes	Scheloribatidae		CNBPL250-13	KM834842
Sarcoptiformes	Scheloribatidae		CNBPL269-13	KM834435
Sarcoptiformes	Scheloribatidae		CNBPL276-13	KM833042
Sarcoptiformes	Scheloribatidae		CNPPD2653-12	KR070266
Sarcoptiformes	Scheloribatidae		CNPPE1441-12	KJ091539
Sarcoptiformes	Scheloribatidae		CNPPE1442-12	KJ083951
Sarcoptiformes	Scheloribatidae		CNPPE1452-12	KJ166742
Sarcoptiformes	Scheloribatidae		CNPPE2113-12	KJ164704
Sarcoptiformes	Scheloribatidae		CNPPE2114-12	KJ163993
Sarcoptiformes	Scheloribatidae		CNPPE2116-12	KJ165636
Sarcoptiformes	Scheloribatidae		CNPPE2117-12	KJ163534
Sarcoptiformes	Scheloribatidae		CNPPE2119-12	KR069148
Sarcoptiformes	Scheloribatidae		CNPPE2120-12	KJ088010
Sarcoptiformes	Scheloribatidae		CNPPE2121-12	KJ164691
Sarcoptiformes	Scheloribatidae		CNPPE2123-12	KJ166831
Sarcoptiformes	Scheloribatidae		CNPPE2129-12	KJ166128
Sarcoptiformes	Scheloribatidae		CNPPE2130-12	KJ165377
Sarcoptiformes	Scheloribatidae		CNPPE2133-12	KJ085321
Sarcoptiformes	Scheloribatidae		CNPPE2140-12	KJ091436
Sarcoptiformes	Scheloribatidae		CNPPE2364-12	KR070383

Sarcoptiformes	Scheloribatidae	CNPPE2365-12	KR070536
Sarcoptiformes	Scheloribatidae	CNPPE2366-12	KR069430
Sarcoptiformes	Scheloribatidae	CNPPE2367-12	KR070455
Sarcoptiformes	Scheloribatidae	CNPPF1039-12	KJ166193
Sarcoptiformes	Scheloribatidae	CNPPF1053-12	KJ166315
Sarcoptiformes	Scheloribatidae	CNPPF1058-12	KJ166660
Sarcoptiformes	Scheloribatidae	CNPPF1300-12	KJ167834
Sarcoptiformes	Scheloribatidae	CNPPF582-12	KR070328
Sarcoptiformes	Scheloribatidae	CNPPF584-12	KJ164171
Sarcoptiformes	Scheloribatidae	CNPPF585-12	KJ163847
Sarcoptiformes	Scheloribatidae	CNPPF589-12	KJ163209
Sarcoptiformes	Scheloribatidae	CNPPF593-12	KJ167678
Sarcoptiformes	Scheloribatidae	CNPPF595-12	KJ163120
Sarcoptiformes	Scheloribatidae	CNPPG497-12	KJ166272
Sarcoptiformes	Scheloribatidae	CNPPH1078-12	KJ444290
Sarcoptiformes	Scheloribatidae	CNPPH1079-12	KR070258
Sarcoptiformes	Scheloribatidae	CNPPH901-12	KR069491
Sarcoptiformes	Scheloribatidae	CNPPI1278-12	KJ208173
Sarcoptiformes	Scheloribatidae	CNPPI1280-12	KR070371
Sarcoptiformes	Scheloribatidae	CNPPI1845-12	KJ445551
Sarcoptiformes	Scheloribatidae	CNPPJ1840-12	KJ207725
Sarcoptiformes	Scheloribatidae	CNPPJ1864-12	KJ207944
Sarcoptiformes	Scheloribatidae	CNRMC1519-12	KM834217
Sarcoptiformes	Scheloribatidae	CNRMC1524-12	KM839815
Sarcoptiformes	Scheloribatidae	CNRMC1531-12	KM828379
Sarcoptiformes	Scheloribatidae	CNRMC1646-12	KM831076
Sarcoptiformes	Scheloribatidae	CNRMC1647-12	KM835480
Sarcoptiformes	Scheloribatidae	CNRMC1656-12	KM835300
Sarcoptiformes	Scheloribatidae	CNRMC1659-12	KM825399
Sarcoptiformes	Scheloribatidae	CNRMC1660-12	KM831455
Sarcoptiformes	Scheloribatidae	CNRMC1674-12	KM831496
Sarcoptiformes	Scheloribatidae	CNRMC1679-12	KM824211
Sarcoptiformes	Scheloribatidae	CNRMC1680-12	KM837722
Sarcoptiformes	Scheloribatidae	CNRMC1688-12	KM828143
Sarcoptiformes	Scheloribatidae	CNRMD2670-12	KM827881
Sarcoptiformes	Scheloribatidae	CNRMD2672-12	KM838502
Sarcoptiformes	Scheloribatidae	CNRMD2673-12	KM840679
Sarcoptiformes	Scheloribatidae	CNRMD2677-12	KM832179
Sarcoptiformes	Scheloribatidae	CNRMD2680-12	KM826903
Sarcoptiformes	Scheloribatidae	CNRMD2688-12	KM824317
Sarcoptiformes	Scheloribatidae	CNRMD2689-12	KM836235
Sarcoptiformes	Scheloribatidae	CNRMD2698-12	KM831209
Sarcoptiformes	Scheloribatidae	CNRMD2706-12	KM833537
Sarcoptiformes	Scheloribatidae	CNRMD2711-12	KM830309
Sarcoptiformes	Scheloribatidae	CNRME4704-12	KM837629
Sarcoptiformes	Scheloribatidae	CNRME4709-12	KM837962
Sarcoptiformes	Scheloribatidae	CNRME4714-12	KM830689
Sarcoptiformes	Scheloribatidae	CNRME4730-12	KM832715
Sarcoptiformes	Scheloribatidae	CNRME4739-12	KM833009
Sarcoptiformes	Scheloribatidae	CNRME4757-12	KM836599
Sarcoptiformes	Scheloribatidae	CNRME4766-12	KM833198
Sarcoptiformes	Scheloribatidae	CNRMF3184-12	KM826854
Sarcoptiformes	Scheloribatidae	CNRMF3212-12	KM824240

Sarcoptiformes	Scheloribatidae		CNRMF3242-12	KM839364
Sarcoptiformes	Scheloribatidae		CNSLD680-12	KM834753
Sarcoptiformes	Scheloribatidae		JSAUG1786-12	KP979333
Sarcoptiformes	Scheloribatidae		JSAUG1790-12	KR069745
Sarcoptiformes	Scheloribatidae		JSAUG1796-12	KR069381
Sarcoptiformes	Scheloribatidae		JSAUG1797-12	KR069655
Sarcoptiformes	Scheloribatidae		JSAUG1800-12	KR069693
Sarcoptiformes	Scheloribatidae		JSAUG1804-12	KR070242
Sarcoptiformes	Scheloribatidae		JSAUG1809-12	KR069969
Sarcoptiformes	Scheloribatidae		JSAUG1810-12	KR070727
Sarcoptiformes	Scheloribatidae		JSAUG1812-12	KR069748
Sarcoptiformes	Scheloribatidae		JSAUG1820-12	KR069357
Sarcoptiformes	Scheloribatidae		JSAUG1824-12	KR070493
Sarcoptiformes	Scheloribatidae		JSAUG1828-12	KR069811
Sarcoptiformes	Scheloribatidae		JSAUG1834-12	KR069646
Sarcoptiformes	Scheloribatidae		JSAUG1836-12	KR069514
Sarcoptiformes	Scheloribatidae		JSAUG1840-12	KR069625
Sarcoptiformes	Scheloribatidae		JSAUG1843-12	KR070101
Sarcoptiformes	Scheloribatidae		JSAUG1846-12	KR069217
Sarcoptiformes	Scheloribatidae		JSAUG1854-12	KR070227
Sarcoptiformes	Scheloribatidae		JSJUL2407-12	KR069150
Sarcoptiformes	Scheloribatidae		JSJUL2538-12	KR070064
Sarcoptiformes	Scheloribatidae		JSJUL2539-12	KR070620
Sarcoptiformes	Scheloribatidae		JSJUL2545-12	KR069897
Sarcoptiformes	Scheloribatidae		JSJUN2340-12	KR069692
Sarcoptiformes	Scheloribatidae		JSJUN2346-12	KR069939
Sarcoptiformes	Scheloribatidae		JSMAY1542-12	KR070092
Sarcoptiformes	Scheloribatidae		JSMAY1564-12	KR070055
Sarcoptiformes	Scheloribatidae		JSMAY1575-12	KR069651
Sarcoptiformes	Scheloribatidae		JSMAY1597-12	KR069896
Sarcoptiformes	Scheloribatidae		JSMAY1600-12	KR070720
Sarcoptiformes	Scheloribatidae		JSSEP1130-12	KR069696
Sarcoptiformes	Scheloribatidae		JSSEP1132-12	KR070270
Sarcoptiformes	Scheloribatidae		JSSEP1146-12	KR070472
Sarcoptiformes	Scheloribatidae		JSSEP1156-12	KR070332
Sarcoptiformes	Scheloribatidae		MIONB138-10	KP979175
Sarcoptiformes	Scheloribatidae		MIONB294-10	KP979214
Sarcoptiformes	Scheloribatidae		MIONB295-10	KP979158
Sarcoptiformes	Scheloribatidae		MIONB296-10	KP979131
Sarcoptiformes	Scheloribatidae		MIONB297-10	KR069957
Sarcoptiformes	Scheloribatidae		MIONB298-10	KR070250
Sarcoptiformes	Scheloribatidae		MYMCA283-11	JX838131
Sarcoptiformes	Scheloribatidae		MYMCA285-11	JX838713
Sarcoptiformes	Scheloribatidae		SSBAA3642-12	KM826536
Sarcoptiformes	Scheloribatidae		SSBAA3643-12	KM837841
Sarcoptiformes	Scheloribatidae		SSBAA3659-12	KM839513
Sarcoptiformes	Scheloribatidae		SSBAA3661-12	KM831311
Sarcoptiformes	Scheloribatidae		SSBAA3662-12	KM824434
Sarcoptiformes	Scheloribatidae		SSBAA3664-12	KM831317
Sarcoptiformes	Scheloribatidae		SSBAA3671-12	KM838833
Sarcoptiformes	Scheloribatidae		SSBAA3694-12	KM838813
Sarcoptiformes	Scheloribatidae		SSBAB1951-12	KM839914
Sarcoptiformes	Scheloribatidae		SSBAD3975-12	KM830436

Sarcoptiformes	Scheloribatidae	SSBAD4019-12	KM833492
Sarcoptiformes	Scheloribatidae	SSBAD4043-12	KM829918
Sarcoptiformes	Scheloribatidae	SSBAD4731-13	KM831947
Sarcoptiformes	Scheloribatidae	SSBAD4732-13	KM829052
Sarcoptiformes	Scheloribatidae	SSBAD4735-13	KM832300
Sarcoptiformes	Scheloribatidae	SSBAD4742-13	KM827647
Sarcoptiformes	Scheloribatidae	SSBAD4743-13	KM830127
Sarcoptiformes	Scheloribatidae	SSBAD4762-13	KM825285
Sarcoptiformes	Scheloribatidae	SSBAD4766-13	KM832767
Sarcoptiformes	Scheloribatidae	SSBAD5517-13	KM828808
Sarcoptiformes	Scheloribatidae	SSBAD5519-13	KM828992
Sarcoptiformes	Scheloribatidae	SSBAD5520-13	KM834683
Sarcoptiformes	Scheloribatidae	SSBAD5522-13	KM839073
Sarcoptiformes	Scheloribatidae	SSBAD5528-13	KM829975
Sarcoptiformes	Scheloribatidae	SSBAD5529-13	KM829703
Sarcoptiformes	Scheloribatidae	SSBAD5532-13	KM832064
Sarcoptiformes	Scheloribatidae	SSBAD5534-13	KM826619
Sarcoptiformes	Scheloribatidae	SSBAD5542-13	KM832830
Sarcoptiformes	Scheloribatidae	SSBAD5555-13	KM831682
Sarcoptiformes	Scheloribatidae	SSBAD5573-13	KM834140
Sarcoptiformes	Scheloribatidae	SSBAD5574-13	KM840543
Sarcoptiformes	Scheloribatidae	SSBAD5578-13	KM833411
Sarcoptiformes	Scheloribatidae	SSBAD5580-13	KM825076
Sarcoptiformes	Scheloribatidae	SSBAD5582-13	KM833660
Sarcoptiformes	Scheloribatidae	SSBAD5584-13	KM840841
Sarcoptiformes	Scheloribatidae	SSBAD5585-13	KM832394
Sarcoptiformes	Scheloribatidae	SSBAD5593-13	KM839817
Sarcoptiformes	Scheloribatidae	SSBAD5594-13	KM832391
Sarcoptiformes	Scheloribatidae	SSBAD5600-13	KM833382
Sarcoptiformes	Scheloribatidae	SSBAD5602-13	KM837230
Sarcoptiformes	Scheloribatidae	SSBAD5604-13	KM833290
Sarcoptiformes	Scheloribatidae	SSBAD6299-13	KM828438
Sarcoptiformes	Scheloribatidae	SSBAD6303-13	KM837264
Sarcoptiformes	Scheloribatidae	SSBAD6306-13	KM824562
Sarcoptiformes	Scheloribatidae	SSBAD6314-13	KM831272
Sarcoptiformes	Scheloribatidae	SSBAD6325-13	KM824709
Sarcoptiformes	Scheloribatidae	SSBAD6326-13	KM825307
Sarcoptiformes	Scheloribatidae	SSBAD6327-13	KM831953
Sarcoptiformes	Scheloribatidae	SSBAD6329-13	KM837848
Sarcoptiformes	Scheloribatidae	SSBAD6330-13	KM832840
Sarcoptiformes	Scheloribatidae	SSBAD6331-13	KM834369
Sarcoptiformes	Scheloribatidae	SSBAD6336-13	KM827665
Sarcoptiformes	Scheloribatidae	SSBAD6339-13	KM835137
Sarcoptiformes	Scheloribatidae	SSBAD6347-13	KM829245
Sarcoptiformes	Scheloribatidae	SSBAD6352-13	KM831985
Sarcoptiformes	Scheloribatidae	SSBAD6356-13	KM839314
Sarcoptiformes	Scheloribatidae	SSEIA049-13	KM838741
Sarcoptiformes	Scheloribatidae	SSEIA050-13	KM838328
Sarcoptiformes	Scheloribatidae	SSEIA2104-13	KM827516
Sarcoptiformes	Scheloribatidae	SSEIA7679-13	KM830379
Sarcoptiformes	Scheloribatidae	SSEIB4235-13	KM833233
Sarcoptiformes	Scheloribatidae	SSEIB4563-13	KM836145
Sarcoptiformes	Scheloribatidae	SSEIB4566-13	KM838305

Sarcoptiformes	Scheloribatidae		SSEIB4581-13	KM836451
Sarcoptiformes	Scheloribatidae		SSEIB4582-13	KM836126
Sarcoptiformes	Scheloribatidae		SSEIB5415-13	KM837455
Sarcoptiformes	Scheloribatidae		SSEIB5417-13	KM825509
Sarcoptiformes	Scheloribatidae		SSEIB8197-13	KM833456
Sarcoptiformes	Scheloribatidae		SSPAA2230-13	KM825989
Sarcoptiformes	Scheloribatidae		SSPAA2234-13	KM836490
Sarcoptiformes	Scheloribatidae		SSPAA6630-13	KM840198
Sarcoptiformes	Scheloribatidae		SSPAA6663-13	KM827318
Sarcoptiformes	Scheloribatidae		SSPAA6678-13	KM834403
Sarcoptiformes	Scheloribatidae		SSPAA6690-13	KM840630
Sarcoptiformes	Scheloribatidae		SSPAA6703-13	KM832922
Sarcoptiformes	Scheloribatidae		SSPAA7744-13	KM840800
Sarcoptiformes	Scheloribatidae		SSPAA7759-13	KM833956
Sarcoptiformes	Scheloribatidae		SSPAA7776-13	KM835181
Sarcoptiformes	Scheloribatidae		SSPAA7792-13	KM827249
Sarcoptiformes	Scheloribatidae		SSPAA7818-13	KM824755
Sarcoptiformes	Scheloribatidae		SSPAA7836-13	KM839851
Sarcoptiformes	Scheloribatidae		SSPAA7943-13	KM825905
Sarcoptiformes	Scheloribatidae		SSPAA7947-13	KM838135
Sarcoptiformes	Scheloribatidae		SSPAA7949-13	KM831557
Sarcoptiformes	Scheloribatidae		SSPAA7955-13	KM825441
Sarcoptiformes	Scheloribatidae		SSWLC3116-13	KM831120
Sarcoptiformes	Scheloribatidae		SSWLC3117-13	KM827698
Sarcoptiformes	Scutoverticidae	*Exochocepheus hungaricus*	GBCH5038-10	GU208613
Sarcoptiformes	Scutoverticidae	*Exochocepheus hungaricus*	GBCH5039-10	GU208612
Sarcoptiformes	Scutoverticidae	*Exochocepheus hungaricus*	GBCH5040-10	GU208611
Sarcoptiformes	Scutoverticidae	*Lamellovertex caelatus*	GBCH5045-10	GU208606
Sarcoptiformes	Scutoverticidae	*Lamellovertex caelatus*	GBCH5046-10	GU208605
Sarcoptiformes	Scutoverticidae	*Provertex kuehnelti*	GBCH5041-10	GU208610
Sarcoptiformes	Scutoverticidae	*Provertex kuehnelti*	GBCH5042-10	GU208609
Sarcoptiformes	Scutoverticidae	*Provertex kuehnelti*	GBCH5043-10	GU208608
Sarcoptiformes	Scutoverticidae	*Provertex kuehnelti*	GBCH5044-10	GU208607
Sarcoptiformes	Scutoverticidae	*Scutovertex arenocolus*	GBCH5072-10	GU208579
Sarcoptiformes	Scutoverticidae	*Scutovertex arenocolus*	GBCH5073-10	GU208578
Sarcoptiformes	Scutoverticidae	*Scutovertex minutus*	GACAC436-12	GQ890361
Sarcoptiformes	Scutoverticidae	*Scutovertex minutus*	GACAC437-12	GQ890362
Sarcoptiformes	Scutoverticidae	*Scutovertex minutus*	GACAC438-12	GQ890363
Sarcoptiformes	Scutoverticidae	*Scutovertex minutus*	GACAC439-12	GQ890364
Sarcoptiformes	Scutoverticidae	*Scutovertex minutus*	GACAC440-12	GQ890365
Sarcoptiformes	Scutoverticidae	*Scutovertex minutus*	GACAC441-12	GQ890366
Sarcoptiformes	Scutoverticidae	*Scutovertex minutus*	GACAC442-12	GQ890367
Sarcoptiformes	Scutoverticidae	*Scutovertex minutus*	GACAC443-12	GQ890368
Sarcoptiformes	Scutoverticidae	*Scutovertex minutus*	GACAC444-12	GQ890369
Sarcoptiformes	Scutoverticidae	*Scutovertex minutus*	GACAC445-12	GQ890370
Sarcoptiformes	Scutoverticidae	*Scutovertex minutus*	GACAC446-12	GQ890371
Sarcoptiformes	Scutoverticidae	*Scutovertex minutus*	GACAC447-12	GQ890372
Sarcoptiformes	Scutoverticidae	*Scutovertex minutus*	GACAC448-12	GQ890373
Sarcoptiformes	Scutoverticidae	*Scutovertex minutus*	GACAC449-12	GQ890374
Sarcoptiformes	Scutoverticidae	*Scutovertex minutus*	GACAC450-12	GQ890375
Sarcoptiformes	Scutoverticidae	*Scutovertex minutus*	GACAC451-12	GQ890376
Sarcoptiformes	Scutoverticidae	*Scutovertex minutus*	GACAC452-12	GQ890377
Sarcoptiformes	Scutoverticidae	*Scutovertex minutus*	GACAC453-12	GQ890378

Sarcoptiformes	Scutoverticidae	*Scutovertex minutus*	GACAC454-12	GQ890379
Sarcoptiformes	Scutoverticidae	*Scutovertex minutus*	GACAC455-12	GQ890380
Sarcoptiformes	Scutoverticidae	*Scutovertex minutus*	GACAC456-12	GQ890381
Sarcoptiformes	Scutoverticidae	*Scutovertex minutus*	GACAC457-12	GQ890382
Sarcoptiformes	Scutoverticidae	*Scutovertex minutus*	GACAC458-12	GQ890383
Sarcoptiformes	Scutoverticidae	*Scutovertex minutus*	GACAC459-12	GQ890384
Sarcoptiformes	Scutoverticidae	*Scutovertex minutus*	GACAC460-12	GQ890385
Sarcoptiformes	Scutoverticidae	*Scutovertex minutus*	GACAC461-12	GQ890386
Sarcoptiformes	Scutoverticidae	*Scutovertex minutus*	GACAC462-12	GQ890387
Sarcoptiformes	Scutoverticidae	*Scutovertex minutus*	GACAC463-12	GQ890388
Sarcoptiformes	Scutoverticidae	*Scutovertex minutus*	GACAC464-12	GQ890389
Sarcoptiformes	Scutoverticidae	*Scutovertex minutus*	GACAC465-12	GQ890390
Sarcoptiformes	Scutoverticidae	*Scutovertex minutus*	GACAC466-12	GQ890391
Sarcoptiformes	Scutoverticidae	*Scutovertex minutus*	GACAC467-12	GQ890392
Sarcoptiformes	Scutoverticidae	*Scutovertex minutus*	GACAC468-12	GQ890393
Sarcoptiformes	Scutoverticidae	*Scutovertex minutus*	GACAC469-12	GQ890394
Sarcoptiformes	Scutoverticidae	*Scutovertex minutus*	GACAC470-12	GQ890395
Sarcoptiformes	Scutoverticidae	*Scutovertex pannonicus*	GACAC519-12	GQ890444
Sarcoptiformes	Scutoverticidae	*Scutovertex pannonicus*	GACAC520-12	GQ890445
Sarcoptiformes	Scutoverticidae	*Scutovertex pictus*	GBCH5064-10	GU208587
Sarcoptiformes	Scutoverticidae	*Scutovertex pictus*	GBCH5065-10	GU208586
Sarcoptiformes	Scutoverticidae	*Scutovertex pileatus*	GBCH5062-10	GU208589
Sarcoptiformes	Scutoverticidae	*Scutovertex pileatus*	GBCH5063-10	GU208588
Sarcoptiformes	Scutoverticidae	*Scutovertex sculptus*	GACAC471-12	GQ890396
Sarcoptiformes	Scutoverticidae	*Scutovertex sculptus*	GACAC472-12	GQ890397
Sarcoptiformes	Scutoverticidae	*Scutovertex sculptus*	GACAC473-12	GQ890398
Sarcoptiformes	Scutoverticidae	*Scutovertex sculptus*	GACAC474-12	GQ890399
Sarcoptiformes	Scutoverticidae	*Scutovertex sculptus*	GACAC475-12	GQ890400
Sarcoptiformes	Scutoverticidae	*Scutovertex sculptus*	GACAC476-12	GQ890401
Sarcoptiformes	Scutoverticidae	*Scutovertex sculptus*	GACAC477-12	GQ890402
Sarcoptiformes	Scutoverticidae	*Scutovertex sculptus*	GACAC478-12	GQ890403
Sarcoptiformes	Scutoverticidae	*Scutovertex sculptus*	GACAC479-12	GQ890404
Sarcoptiformes	Scutoverticidae	*Scutovertex sculptus*	GACAC480-12	GQ890405
Sarcoptiformes	Scutoverticidae	*Scutovertex sculptus*	GACAC481-12	GQ890406
Sarcoptiformes	Scutoverticidae	*Scutovertex sculptus*	GACAC482-12	GQ890407
Sarcoptiformes	Scutoverticidae	*Scutovertex sculptus*	GACAC483-12	GQ890408
Sarcoptiformes	Scutoverticidae	*Scutovertex sculptus*	GACAC484-12	GQ890409
Sarcoptiformes	Scutoverticidae	*Scutovertex sculptus*	GACAC485-12	GQ890410
Sarcoptiformes	Scutoverticidae	*Scutovertex sculptus*	GACAC486-12	GQ890411
Sarcoptiformes	Scutoverticidae	*Scutovertex sculptus*	GACAC487-12	GQ890412
Sarcoptiformes	Scutoverticidae	*Scutovertex sculptus*	GACAC488-12	GQ890413
Sarcoptiformes	Scutoverticidae	*Scutovertex sculptus*	GACAC489-12	GQ890414
Sarcoptiformes	Scutoverticidae	*Scutovertex sculptus*	GACAC490-12	GQ890415
Sarcoptiformes	Scutoverticidae	*Scutovertex sculptus*	GACAC491-12	GQ890416
Sarcoptiformes	Scutoverticidae	*Scutovertex sculptus*	GACAC492-12	GQ890417
Sarcoptiformes	Scutoverticidae	*Scutovertex sculptus*	GACAC493-12	GQ890418
Sarcoptiformes	Scutoverticidae	*Scutovertex sculptus*	GACAC494-12	GQ890419
Sarcoptiformes	Scutoverticidae	*Scutovertex sculptus*	GACAC495-12	GQ890420
Sarcoptiformes	Scutoverticidae	*Scutovertex sculptus*	GACAC496-12	GQ890421
Sarcoptiformes	Scutoverticidae	*Scutovertex sculptus*	GACAC497-12	GQ890422
Sarcoptiformes	Scutoverticidae	*Scutovertex sculptus*	GACAC498-12	GQ890423
Sarcoptiformes	Scutoverticidae	*Scutovertex sculptus*	GACAC499-12	GQ890424
Sarcoptiformes	Scutoverticidae	*Scutovertex sculptus*	GACAC500-12	GQ890425

Sarcoptiformes	Scutoverticidae	*Scutovertex sculptus*	GACAC501-12	GQ890426
Sarcoptiformes	Scutoverticidae	*Scutovertex sculptus*	GACAC502-12	GQ890427
Sarcoptiformes	Scutoverticidae	*Scutovertex sculptus*	GACAC503-12	GQ890428
Sarcoptiformes	Scutoverticidae	*Scutovertex sculptus*	GACAC504-12	GQ890429
Sarcoptiformes	Scutoverticidae	*Scutovertex sculptus*	GACAC505-12	GQ890430
Sarcoptiformes	Scutoverticidae	*Scutovertex sculptus*	GACAC506-12	GQ890431
Sarcoptiformes	Scutoverticidae	*Scutovertex sculptus*	GACAC507-12	GQ890432
Sarcoptiformes	Scutoverticidae	*Scutovertex sculptus*	GACAC508-12	GQ890433
Sarcoptiformes	Scutoverticidae	*Scutovertex sculptus*	GACAC509-12	GQ890434
Sarcoptiformes	Scutoverticidae	*Scutovertex sculptus*	GACAC510-12	GQ890435
Sarcoptiformes	Scutoverticidae	*Scutovertex sculptus*	GACAC511-12	GQ890436
Sarcoptiformes	Scutoverticidae	*Scutovertex sculptus*	GACAC512-12	GQ890437
Sarcoptiformes	Scutoverticidae	*Scutovertex sculptus*	GACAC513-12	GQ890438
Sarcoptiformes	Scutoverticidae	*Scutovertex sculptus*	GACAC514-12	GQ890439
Sarcoptiformes	Scutoverticidae	*Scutovertex sculptus*	GACAC515-12	GQ890440
Sarcoptiformes	Scutoverticidae	*Scutovertex sculptus*	GACAC516-12	GQ890441
Sarcoptiformes	Scutoverticidae	*Scutovertex sculptus*	GACAC517-12	GQ890442
Sarcoptiformes	Scutoverticidae	*Scutovertex sculptus*	GACAC518-12	GQ890443
Sarcoptiformes	Scutoverticidae	*Scutovertex sculptus*	GBCH5047-10	GU208604
Sarcoptiformes	Scutoverticidae	*Scutovertex sculptus*	GBCH5048-10	GU208603
Sarcoptiformes	Scutoverticidae	*Scutovertex sculptus*	GBCH5049-10	GU208602
Sarcoptiformes	Scutoverticidae	*Scutovertex sculptus*	GBCH5050-10	GU208601
Sarcoptiformes	Scutoverticidae	*Scutovertex sculptus*	GBCH5051-10	GU208600
Sarcoptiformes	Scutoverticidae	*Scutovertex sculptus*	GBCH5052-10	GU208599
Sarcoptiformes	Scutoverticidae	*Scutovertex sculptus*	GBCH5053-10	GU208598
Sarcoptiformes	Scutoverticidae	*Scutovertex sculptus*	GBCH5054-10	GU208597
Sarcoptiformes	Scutoverticidae	*Scutovertex sculptus*	GBCH5055-10	GU208596
Sarcoptiformes	Scutoverticidae	*Scutovertex sculptus*	GBCH5056-10	GU208595
Sarcoptiformes	Scutoverticidae	*Scutovertex sculptus*	GBCH5057-10	GU208594
Sarcoptiformes	Scutoverticidae	*Scutovertex sp. Hungary*	GBCH5060-10	GU208591
Sarcoptiformes	Scutoverticidae	*Scutovertex sp. Hungary*	GBCH5061-10	GU208590
Sarcoptiformes	Scutoverticidae	*Scutovertex sp. ianus*	GBCH5066-10	GU208585
Sarcoptiformes	Scutoverticidae	*Scutovertex sp. ianus*	GBCH5067-10	GU208584
Sarcoptiformes	Scutoverticidae	*Scutovertex sp. ianus*	GBCH5068-10	GU208583
Sarcoptiformes	Scutoverticidae	*Scutovertex sp. ianus*	GBCH5069-10	GU208582
Sarcoptiformes	Scutoverticidae	*Scutovertex sp. ianus*	GBCH5070-10	GU208581
Sarcoptiformes	Scutoverticidae	*Scutovertex sp. ianus*	GBCH5071-10	GU208580
Sarcoptiformes	Scutoverticidae	*Scutovertex sp. Wangen*	GBCH5058-10	GU208593
Sarcoptiformes	Scutoverticidae	*Scutovertex sp. Wangen*	GBCH5059-10	GU208592
Sarcoptiformes	Scutoverticidae		SSBAA3625-12	KM828547
Sarcoptiformes	Scutoverticidae		SSBAA3639-12	KM828405
Sarcoptiformes	Scutoverticidae		SSWLC062-13	KM833395
Sarcoptiformes	Suctobelbidae	*Suctobelbella*	CHACA955-10	HM405827
Sarcoptiformes	Suctobelbidae	*Suctobelbella*	CHACB020-10	HQ558340
Sarcoptiformes	Suctobelbidae	*Suctobelbella*	CHACB1009-10	HM907145
Sarcoptiformes	Suctobelbidae	*Suctobelbella*	CHACB1156-10	HM907241
Sarcoptiformes	Suctobelbidae	*Suctobelbella*	CHACB1204-10	HM907274
Sarcoptiformes	Suctobelbidae	*Suctobelbella*	CHACB1206-10	HM907276
Sarcoptiformes	Suctobelbidae	*Suctobelbella*	CHACB937-10	HM907363
Sarcoptiformes	Suctobelbidae	*Suctobelbella*	MYMCA054-11	JX834355
Sarcoptiformes	Suctobelbidae	*Suctobelbella*	MYMCA056-11	JX836368
Sarcoptiformes	Suctobelbidae	*Suctobelbella*	MYMCA287-11	JX835522
Sarcoptiformes	Suctobelbidae	*Suctobelbella*	MYMCA437-11	JX838733

Sarcoptiformes	Suctobelbidae	*Suctobelbella*	MYMCA439-11	JX837063
Sarcoptiformes	Suctobelbidae	*Suctobelbella*	MYMCA860-11	JX837391
Sarcoptiformes	Suctobelbidae	*Suctobelbella*	MYMCB231-11	JX838030
Sarcoptiformes	Suctobelbidae	*Suctobelbella*	MYMCB855-11	JX833755
Sarcoptiformes	Suctobelbidae	*Suctobelbella*	MYMCC494-11	JX836142
Sarcoptiformes	Suctobelbidae	*Suctobelbella*	MYMCF578-12	JX838113
Sarcoptiformes	Suctobelbidae	*Suctobelbella*	MYMCF580-12	JX835425
Sarcoptiformes	Suctobelbidae	*Suctobelbella*	MYMCF643-12	JX834247
Sarcoptiformes	Suctobelbidae	*Suctobelbella*	MYMCF917-12	JX836205
Sarcoptiformes	Suctobelbidae	*Suctobelbella*	MYMCG639-12	JX835378
Sarcoptiformes	Suctobelbidae	*Suctobelbella sarekensis*	CHACA122-08	JX836501
Sarcoptiformes	Suctobelbidae	*Suctobelbella sarekensis*	CHACC204-10	HQ941529
Sarcoptiformes	Suctobelbidae	*Suctobelbella sarekensis*	CHACC206-10	HQ941531
Sarcoptiformes	Suctobelbidae	*Suctobelbella sarekensis*	MYMCF131-12	JX838253
Sarcoptiformes	Suctobelbidae		MYMCA055-11	JX836748
Sarcoptiformes	Suctobelbidae		MYMCA436-11	JX838164
Sarcoptiformes	Suctobelbidae		MYMCA438-11	JX836824
Sarcoptiformes	Suctobelbidae		MYMCB054-11	JX838395
Sarcoptiformes	Suctobelbidae		MYMCB055-11	JX838452
Sarcoptiformes	Suctobelbidae		MYMCB230-11	JX835999
Sarcoptiformes	Suctobelbidae		MYMCB439-11	JX838589
Sarcoptiformes	Suctobelbidae		MYMCB587-11	JX835508
Sarcoptiformes	Suctobelbidae		MYMCB796-11	JX835152
Sarcoptiformes	Suctobelbidae		MYMCB797-11	JX837131
Sarcoptiformes	Suctobelbidae		MYMCC171-11	JX836265
Sarcoptiformes	Suctobelbidae		MYMCC172-11	JX837341
Sarcoptiformes	Suctobelbidae		MYMCC687-11	JX834655
Sarcoptiformes	Suctobelbidae		MYMCE808-12	JX836485
Sarcoptiformes	Suctobelbidae		MYMCF199-12	JX835759
Sarcoptiformes	Suctobelbidae		MYMCG076-12	JX837769
Sarcoptiformes	Suctobelbidae		MYMCG339-12	JX838590
Sarcoptiformes	Syringobiidae	*Syringobia longipenis*	GBCH4453-10	GQ864349
Sarcoptiformes	Tectocepheidae	*Tectocepheus*	CHACA1077-10	HM907120
Sarcoptiformes	Tectocepheidae	*Tectocepheus*	CHACA1078-10	HM907121
Sarcoptiformes	Tectocepheidae	*Tectocepheus*	CHACA1079-10	HM907122
Sarcoptiformes	Tectocepheidae	*Tectocepheus*	CHACA1080-10	HM907123
Sarcoptiformes	Tectocepheidae	*Tectocepheus*	CHACA508-09	JX837238
Sarcoptiformes	Tectocepheidae	*Tectocepheus*	CHACA533-09	JX837007
Sarcoptiformes	Tectocepheidae	*Tectocepheus*	CHACB152-10	HQ558435
Sarcoptiformes	Tectocepheidae	*Tectocepheus*	CHACB166-10	HQ558448
Sarcoptiformes	Tectocepheidae	*Tectocepheus*	CHACB377-10	HQ558553
Sarcoptiformes	Tectocepheidae	*Tectocepheus*	CHACB378-10	JX837277
Sarcoptiformes	Tectocepheidae	*Tectocepheus*	CHACB379-10	JX837311
Sarcoptiformes	Tectocepheidae	*Tectocepheus*	CHACB393-10	JX838294
Sarcoptiformes	Tectocepheidae	*Tectocepheus*	CHACB394-10	HQ558559
Sarcoptiformes	Tectocepheidae	*Tectocepheus*	CHACB492-10	HQ558627
Sarcoptiformes	Tectocepheidae	*Tectocepheus*	CHACB505-10	HQ558635
Sarcoptiformes	Tectocepheidae	*Tectocepheus*	CHACB534-10	JX835479
Sarcoptiformes	Tectocepheidae	*Tectocepheus*	CHACB575-10	HQ558681
Sarcoptiformes	Tectocepheidae	*Tectocepheus*	CHACB576-10	HQ558682
Sarcoptiformes	Tectocepheidae	*Tectocepheus*	CHACB597-10	JX836110
Sarcoptiformes	Tectocepheidae	*Tectocepheus*	CHACB600-10	JX834183
Sarcoptiformes	Tectocepheidae	*Tectocepheus*	CHACB663-10	JX836704

Sarcoptiformes	Tectocepheidae	*Tectocepheus*		CHACB664-10	JX835075
Sarcoptiformes	Tectocepheidae	*Tectocepheus*		CHACB710-10	HQ558763
Sarcoptiformes	Tectocepheidae	*Tectocepheus*		CHACB714-10	JX836327
Sarcoptiformes	Tectocepheidae	*Tectocepheus*		CHACB809-10	JX835032
Sarcoptiformes	Tectocepheidae	*Tectocepheus*		CHACB858-10	HM907310
Sarcoptiformes	Tectocepheidae	*Tectocepheus*		CHACC038-10	HM907438
Sarcoptiformes	Tectocepheidae	*Tectocepheus*		CHACC039-10	HM907439
Sarcoptiformes	Tectocepheidae	*Tectocepheus*		CHACC040-10	HM907440
Sarcoptiformes	Tectocepheidae	*Tectocepheus*		CHACC041-10	HM907441
Sarcoptiformes	Tectocepheidae	*Tectocepheus*		CHACC042-10	HM907442
Sarcoptiformes	Tectocepheidae	*Tectocepheus*		CHACC094-10	HM907483
Sarcoptiformes	Tectocepheidae	*Tectocepheus*		CHACC095-10	HM907484
Sarcoptiformes	Tectocepheidae	*Tectocepheus*		CHACC096-10	JX834552
Sarcoptiformes	Tectocepheidae	*Tectocepheus*		CHACC097-10	JX833948
Sarcoptiformes	Tectocepheidae	*Tectocepheus*		CHACC098-10	JX833930
Sarcoptiformes	Tectocepheidae	*Tectocepheus*		CHACC100-10	JX834782
Sarcoptiformes	Tectocepheidae	*Tectocepheus*		CHACC102-10	JX837365
Sarcoptiformes	Tectocepheidae	*Tectocepheus*		CNJAB1025-12	KM829408
Sarcoptiformes	Tectocepheidae	*Tectocepheus*		MIONB140-10	KP979224
Sarcoptiformes	Tectocepheidae	*Tectocepheus*		MYMCA1072-11	JX837722
Sarcoptiformes	Tectocepheidae	*Tectocepheus*		MYMCA1138-11	JX835427
Sarcoptiformes	Tectocepheidae	*Tectocepheus*		MYMCA169-11	JX836364
Sarcoptiformes	Tectocepheidae	*Tectocepheus*		MYMCA234-11	JX837352
Sarcoptiformes	Tectocepheidae	*Tectocepheus*		MYMCA235-11	JX835452
Sarcoptiformes	Tectocepheidae	*Tectocepheus*		MYMCA236-11	JX836369
Sarcoptiformes	Tectocepheidae	*Tectocepheus*		MYMCA237-11	JX834373
Sarcoptiformes	Tectocepheidae	*Tectocepheus*		MYMCA432-11	JX835314
Sarcoptiformes	Tectocepheidae	*Tectocepheus*		MYMCA433-11	JX837491
Sarcoptiformes	Tectocepheidae	*Tectocepheus*		MYMCA510-11	JX833932
Sarcoptiformes	Tectocepheidae	*Tectocepheus*		MYMCA552-11	JX835232
Sarcoptiformes	Tectocepheidae	*Tectocepheus*		MYMCA553-11	JX834541
Sarcoptiformes	Tectocepheidae	*Tectocepheus*		MYMCA554-11	JX837654
Sarcoptiformes	Tectocepheidae	*Tectocepheus*		MYMCA734-11	JX837308
Sarcoptiformes	Tectocepheidae	*Tectocepheus*		MYMCA862-11	JX835077
Sarcoptiformes	Tectocepheidae	*Tectocepheus*		MYMCA863-11	JX834356
Sarcoptiformes	Tectocepheidae	*Tectocepheus*		MYMCA864-11	JX838218
Sarcoptiformes	Tectocepheidae	*Tectocepheus*		MYMCA909-11	JX835570
Sarcoptiformes	Tectocepheidae	*Tectocepheus*		MYMCA910-11	JX838409
Sarcoptiformes	Tectocepheidae	*Tectocepheus*		MYMCB096-11	JX838096
Sarcoptiformes	Tectocepheidae	*Tectocepheus*		MYMCB116-11	JX837890
Sarcoptiformes	Tectocepheidae	*Tectocepheus*		MYMCB117-11	JX834267
Sarcoptiformes	Tectocepheidae	*Tectocepheus*		MYMCB118-11	JX835447
Sarcoptiformes	Tectocepheidae	*Tectocepheus*		MYMCB147-11	JX837790
Sarcoptiformes	Tectocepheidae	*Tectocepheus*		MYMCB148-11	JX835333
Sarcoptiformes	Tectocepheidae	*Tectocepheus*		MYMCB232-11	JX833862
Sarcoptiformes	Tectocepheidae	*Tectocepheus*		MYMCB233-11	JX836153
Sarcoptiformes	Tectocepheidae	*Tectocepheus*		MYMCB501-11	JX837783
Sarcoptiformes	Tectocepheidae	*Tectocepheus*		MYMCB586-11	JX837524
Sarcoptiformes	Tectocepheidae	*Tectocepheus*		MYMCB620-11	JX835858
Sarcoptiformes	Tectocepheidae	*Tectocepheus*		MYMCB712-11	JX837898
Sarcoptiformes	Tectocepheidae	*Tectocepheus*		MYMCB713-11	JX834604
Sarcoptiformes	Tectocepheidae	*Tectocepheus*		MYMCB807-11	JX836905
Sarcoptiformes	Tectocepheidae	*Tectocepheus*		MYMCB808-11	JX838657

Sarcoptiformes	Tectocepheidae	*Tectocepheus*	MYMCB826-11	JX837709
Sarcoptiformes	Tectocepheidae	*Tectocepheus*	MYMCB829-11	JX838582
Sarcoptiformes	Tectocepheidae	*Tectocepheus*	MYMCB850-11	JX837772
Sarcoptiformes	Tectocepheidae	*Tectocepheus*	MYMCB873-11	JX836199
Sarcoptiformes	Tectocepheidae	*Tectocepheus*	MYMCB881-11	JX833766
Sarcoptiformes	Tectocepheidae	*Tectocepheus*	MYMCC062-11	JX834218
Sarcoptiformes	Tectocepheidae	*Tectocepheus*	MYMCC063-11	JX834617
Sarcoptiformes	Tectocepheidae	*Tectocepheus*	MYMCC064-11	JX834964
Sarcoptiformes	Tectocepheidae	*Tectocepheus*	MYMCC065-11	JX834394
Sarcoptiformes	Tectocepheidae	*Tectocepheus*	MYMCC194-11	JX838770
Sarcoptiformes	Tectocepheidae	*Tectocepheus*	MYMCC196-11	JX838045
Sarcoptiformes	Tectocepheidae	*Tectocepheus*	MYMCC197-11	JX836168
Sarcoptiformes	Tectocepheidae	*Tectocepheus*	MYMCC224-11	JX834137
Sarcoptiformes	Tectocepheidae	*Tectocepheus*	MYMCC262-11	JX835623
Sarcoptiformes	Tectocepheidae	*Tectocepheus*	MYMCC280-11	JX836433
Sarcoptiformes	Tectocepheidae	*Tectocepheus*	MYMCC492-11	JX837305
Sarcoptiformes	Tectocepheidae	*Tectocepheus*	MYMCC641-11	JX835305
Sarcoptiformes	Tectocepheidae	*Tectocepheus*	MYMCC679-11	JX835557
Sarcoptiformes	Tectocepheidae	*Tectocepheus*	MYMCC809-11	JX837702
Sarcoptiformes	Tectocepheidae	*Tectocepheus*	MYMCC810-11	JX836184
Sarcoptiformes	Tectocepheidae	*Tectocepheus*	MYMCC811-11	JX835416
Sarcoptiformes	Tectocepheidae	*Tectocepheus*	MYMCC911-11	JX834764
Sarcoptiformes	Tectocepheidae	*Tectocepheus*	MYMCC934-11	JX836192
Sarcoptiformes	Tectocepheidae	*Tectocepheus*	MYMCE026-12	JX835609
Sarcoptiformes	Tectocepheidae	*Tectocepheus*	MYMCE027-12	JX836625
Sarcoptiformes	Tectocepheidae	*Tectocepheus*	MYMCE028-12	JX833702
Sarcoptiformes	Tectocepheidae	*Tectocepheus*	MYMCE053-12	JX837872
Sarcoptiformes	Tectocepheidae	*Tectocepheus*	MYMCE054-12	JX834081
Sarcoptiformes	Tectocepheidae	*Tectocepheus*	MYMCE055-12	JX835379
Sarcoptiformes	Tectocepheidae	*Tectocepheus*	MYMCE060-12	JX838193
Sarcoptiformes	Tectocepheidae	*Tectocepheus*	MYMCE211-12	JX837539
Sarcoptiformes	Tectocepheidae	*Tectocepheus*	MYMCE220-12	JX834575
Sarcoptiformes	Tectocepheidae	*Tectocepheus*	MYMCE221-12	JX834963
Sarcoptiformes	Tectocepheidae	*Tectocepheus*	MYMCE222-12	JX837397
Sarcoptiformes	Tectocepheidae	*Tectocepheus*	MYMCE223-12	JX835633
Sarcoptiformes	Tectocepheidae	*Tectocepheus*	MYMCE224-12	JX835284
Sarcoptiformes	Tectocepheidae	*Tectocepheus*	MYMCE282-12	JX837097
Sarcoptiformes	Tectocepheidae	*Tectocepheus*	MYMCE283-12	JX835516
Sarcoptiformes	Tectocepheidae	*Tectocepheus*	MYMCE284-12	JX833873
Sarcoptiformes	Tectocepheidae	*Tectocepheus*	MYMCE285-12	JX835850
Sarcoptiformes	Tectocepheidae	*Tectocepheus*	MYMCE319-12	JX834543
Sarcoptiformes	Tectocepheidae	*Tectocepheus*	MYMCE320-12	JX838347
Sarcoptiformes	Tectocepheidae	*Tectocepheus*	MYMCE321-12	JX834222
Sarcoptiformes	Tectocepheidae	*Tectocepheus*	MYMCE329-12	JX834892
Sarcoptiformes	Tectocepheidae	*Tectocepheus*	MYMCE337-12	JX835236
Sarcoptiformes	Tectocepheidae	*Tectocepheus*	MYMCE375-12	JX834676
Sarcoptiformes	Tectocepheidae	*Tectocepheus*	MYMCE376-12	JX837707
Sarcoptiformes	Tectocepheidae	*Tectocepheus*	MYMCE388-12	JX838742
Sarcoptiformes	Tectocepheidae	*Tectocepheus*	MYMCE389-12	JX838724
Sarcoptiformes	Tectocepheidae	*Tectocepheus*	MYMCE390-12	JX833934
Sarcoptiformes	Tectocepheidae	*Tectocepheus*	MYMCE467-12	JX837467
Sarcoptiformes	Tectocepheidae	*Tectocepheus*	MYMCE468-12	JX834788
Sarcoptiformes	Tectocepheidae	*Tectocepheus*	MYMCE469-12	JX833696

Sarcoptiformes	Tectocepheidae	*Tectocepheus*	MYMCE500-12	JX836984
Sarcoptiformes	Tectocepheidae	*Tectocepheus*	MYMCE558-12	JX838557
Sarcoptiformes	Tectocepheidae	*Tectocepheus*	MYMCE589-12	JX833919
Sarcoptiformes	Tectocepheidae	*Tectocepheus*	MYMCE635-12	JX833630
Sarcoptiformes	Tectocepheidae	*Tectocepheus*	MYMCE636-12	JX836384
Sarcoptiformes	Tectocepheidae	*Tectocepheus*	MYMCE637-12	JX834950
Sarcoptiformes	Tectocepheidae	*Tectocepheus*	MYMCE638-12	JX835624
Sarcoptiformes	Tectocepheidae	*Tectocepheus*	MYMCE658-12	JX834638
Sarcoptiformes	Tectocepheidae	*Tectocepheus*	MYMCE682-12	JX833794
Sarcoptiformes	Tectocepheidae	*Tectocepheus*	MYMCE683-12	JX834774
Sarcoptiformes	Tectocepheidae	*Tectocepheus*	MYMCE684-12	JX834911
Sarcoptiformes	Tectocepheidae	*Tectocepheus*	MYMCE708-12	JX837543
Sarcoptiformes	Tectocepheidae	*Tectocepheus*	MYMCE709-12	JX835738
Sarcoptiformes	Tectocepheidae	*Tectocepheus*	MYMCE780-12	JX834800
Sarcoptiformes	Tectocepheidae	*Tectocepheus*	MYMCE781-12	JX833825
Sarcoptiformes	Tectocepheidae	*Tectocepheus*	MYMCE782-12	JX833697
Sarcoptiformes	Tectocepheidae	*Tectocepheus*	MYMCE801-12	JX835167
Sarcoptiformes	Tectocepheidae	*Tectocepheus*	MYMCE802-12	JX836041
Sarcoptiformes	Tectocepheidae	*Tectocepheus*	MYMCE803-12	JX835330
Sarcoptiformes	Tectocepheidae	*Tectocepheus*	MYMCE828-12	JX837518
Sarcoptiformes	Tectocepheidae	*Tectocepheus*	MYMCE829-12	JX835705
Sarcoptiformes	Tectocepheidae	*Tectocepheus*	MYMCE863-12	JX837734
Sarcoptiformes	Tectocepheidae	*Tectocepheus*	MYMCE903-12	JX837743
Sarcoptiformes	Tectocepheidae	*Tectocepheus*	MYMCE904-12	JX838092
Sarcoptiformes	Tectocepheidae	*Tectocepheus*	MYMCE905-12	JX838571
Sarcoptiformes	Tectocepheidae	*Tectocepheus*	MYMCE906-12	JX837786
Sarcoptiformes	Tectocepheidae	*Tectocepheus*	MYMCE916-12	JX837506
Sarcoptiformes	Tectocepheidae	*Tectocepheus*	MYMCE917-12	JX835391
Sarcoptiformes	Tectocepheidae	*Tectocepheus*	MYMCE918-12	JX834544
Sarcoptiformes	Tectocepheidae	*Tectocepheus*	MYMCE919-12	JX836604
Sarcoptiformes	Tectocepheidae	*Tectocepheus*	MYMCF022-12	JX836139
Sarcoptiformes	Tectocepheidae	*Tectocepheus*	MYMCF023-12	JX834851
Sarcoptiformes	Tectocepheidae	*Tectocepheus*	MYMCF042-12	JX833699
Sarcoptiformes	Tectocepheidae	*Tectocepheus*	MYMCF069-12	JX834535
Sarcoptiformes	Tectocepheidae	*Tectocepheus*	MYMCF070-12	JX835027
Sarcoptiformes	Tectocepheidae	*Tectocepheus*	MYMCF134-12	JX834707
Sarcoptiformes	Tectocepheidae	*Tectocepheus*	MYMCF135-12	JX837476
Sarcoptiformes	Tectocepheidae	*Tectocepheus*	MYMCF166-12	JX836236
Sarcoptiformes	Tectocepheidae	*Tectocepheus*	MYMCF185-12	JX835267
Sarcoptiformes	Tectocepheidae	*Tectocepheus*	MYMCF186-12	JX837433
Sarcoptiformes	Tectocepheidae	*Tectocepheus*	MYMCF187-12	JX834827
Sarcoptiformes	Tectocepheidae	*Tectocepheus*	MYMCF189-12	JX837914
Sarcoptiformes	Tectocepheidae	*Tectocepheus*	MYMCF190-12	JX834951
Sarcoptiformes	Tectocepheidae	*Tectocepheus*	MYMCF203-12	JX836521
Sarcoptiformes	Tectocepheidae	*Tectocepheus*	MYMCF205-12	JX837945
Sarcoptiformes	Tectocepheidae	*Tectocepheus*	MYMCF241-12	JX836248
Sarcoptiformes	Tectocepheidae	*Tectocepheus*	MYMCF242-12	JX835638
Sarcoptiformes	Tectocepheidae	*Tectocepheus*	MYMCF334-12	JX836096
Sarcoptiformes	Tectocepheidae	*Tectocepheus*	MYMCF388-12	JX838649
Sarcoptiformes	Tectocepheidae	*Tectocepheus*	MYMCF389-12	JX836802
Sarcoptiformes	Tectocepheidae	*Tectocepheus*	MYMCF390-12	JX838531
Sarcoptiformes	Tectocepheidae	*Tectocepheus*	MYMCF391-12	JX837228
Sarcoptiformes	Tectocepheidae	*Tectocepheus*	MYMCF425-12	JX836088

Sarcoptiformes	Tectocepheidae	*Tectocepheus*		MYMCF426-12	JX838088
Sarcoptiformes	Tectocepheidae	*Tectocepheus*		MYMCF451-12	JX835553
Sarcoptiformes	Tectocepheidae	*Tectocepheus*		MYMCF507-12	JX834070
Sarcoptiformes	Tectocepheidae	*Tectocepheus*		MYMCF508-12	JX834569
Sarcoptiformes	Tectocepheidae	*Tectocepheus*		MYMCF509-12	JX837220
Sarcoptiformes	Tectocepheidae	*Tectocepheus*		MYMCF522-12	JX835801
Sarcoptiformes	Tectocepheidae	*Tectocepheus*		MYMCF523-12	JX838315
Sarcoptiformes	Tectocepheidae	*Tectocepheus*		MYMCF524-12	JX836638
Sarcoptiformes	Tectocepheidae	*Tectocepheus*		MYMCF590-12	JX837983
Sarcoptiformes	Tectocepheidae	*Tectocepheus*		MYMCF640-12	JX834599
Sarcoptiformes	Tectocepheidae	*Tectocepheus*		MYMCF641-12	JX835502
Sarcoptiformes	Tectocepheidae	*Tectocepheus*		MYMCF725-12	JX837826
Sarcoptiformes	Tectocepheidae	*Tectocepheus*		MYMCF726-12	JX835814
Sarcoptiformes	Tectocepheidae	*Tectocepheus*		MYMCF749-12	JX837105
Sarcoptiformes	Tectocepheidae	*Tectocepheus*		MYMCF769-12	JX838736
Sarcoptiformes	Tectocepheidae	*Tectocepheus*		MYMCF809-12	JX837973
Sarcoptiformes	Tectocepheidae	*Tectocepheus*		MYMCF841-12	JX836233
Sarcoptiformes	Tectocepheidae	*Tectocepheus*		MYMCF842-12	JX836559
Sarcoptiformes	Tectocepheidae	*Tectocepheus*		MYMCF871-12	JX835464
Sarcoptiformes	Tectocepheidae	*Tectocepheus*		MYMCG013-12	JX835325
Sarcoptiformes	Tectocepheidae	*Tectocepheus*		MYMCG014-12	JX836565
Sarcoptiformes	Tectocepheidae	*Tectocepheus*		MYMCG015-12	JX834853
Sarcoptiformes	Tectocepheidae	*Tectocepheus*		MYMCG036-12	JX835255
Sarcoptiformes	Tectocepheidae	*Tectocepheus*		MYMCG038-12	JX836123
Sarcoptiformes	Tectocepheidae	*Tectocepheus*		MYMCG074-12	JX834723
Sarcoptiformes	Tectocepheidae	*Tectocepheus*		MYMCG144-12	JX836228
Sarcoptiformes	Tectocepheidae	*Tectocepheus*		MYMCG164-12	JX834515
Sarcoptiformes	Tectocepheidae	*Tectocepheus*		MYMCG165-12	JX833889
Sarcoptiformes	Tectocepheidae	*Tectocepheus*		MYMCG166-12	JX835781
Sarcoptiformes	Tectocepheidae	*Tectocepheus*		MYMCG167-12	JX838013
Sarcoptiformes	Tectocepheidae	*Tectocepheus*		MYMCG227-12	JX834123
Sarcoptiformes	Tectocepheidae	*Tectocepheus*		MYMCG236-12	JX837417
Sarcoptiformes	Tectocepheidae	*Tectocepheus*		MYMCG237-12	JX834474
Sarcoptiformes	Tectocepheidae	*Tectocepheus*		MYMCG259-12	JX837632
Sarcoptiformes	Tectocepheidae	*Tectocepheus*		MYMCG260-12	JX836058
Sarcoptiformes	Tectocepheidae	*Tectocepheus*		MYMCG333-12	JX836495
Sarcoptiformes	Tectocepheidae	*Tectocepheus*		MYMCG336-12	JX834769
Sarcoptiformes	Tectocepheidae	*Tectocepheus*		MYMCG337-12	JX836350
Sarcoptiformes	Tectocepheidae	*Tectocepheus*		MYMCG355-12	JX833946
Sarcoptiformes	Tectocepheidae	*Tectocepheus*		MYMCG483-12	JX838034
Sarcoptiformes	Tectocepheidae	*Tectocepheus*		MYMCG546-12	JX837261
Sarcoptiformes	Tectocepheidae	*Tectocepheus*		MYMCG548-12	JX834942
Sarcoptiformes	Tectocepheidae	*Tectocepheus*		MYMCG549-12	JX836919
Sarcoptiformes	Tectocepheidae	*Tectocepheus*		MYMCG550-12	JX835042
Sarcoptiformes	Tectocepheidae	*Tectocepheus*		MYMCG551-12	JX838699
Sarcoptiformes	Tectocepheidae	*Tectocepheus*		MYMCG607-12	JX836951
Sarcoptiformes	Tectocepheidae	*Tectocepheus*		MYMCG608-12	JX837235
Sarcoptiformes	Tectocepheidae	*Tectocepheus*		MYMCG609-12	JX835424
Sarcoptiformes	Tectocepheidae	*Tectocepheus*		MYMCG610-12	JX835415
Sarcoptiformes	Tectocepheidae	*Tectocepheus*		MYMCG611-12	JX836426
Sarcoptiformes	Tectocepheidae	*Tectocepheus*		MYMCG638-12	JX834531
Sarcoptiformes	Tectocepheidae	*Tectocepheus*		MYTMC016-09	JX836618
Sarcoptiformes	Tectocepheidae	*Tectocepheus*		MYTMC047-09	HQ966228

Sarcoptiformes	Tectocepheidae	*Tectocepheus*	MYTMC146-09	JX833826
Sarcoptiformes	Tectocepheidae	*Tectocepheus*	MYTMC154-09	HQ966242
Sarcoptiformes	Tectocepheidae	*Tectocepheus*	MYTMC176-09	HQ966246
Sarcoptiformes	Tectocepheidae	*Tectocepheus*	SSBAA3693-12	KM831801
Sarcoptiformes	Tectocepheidae	*Tectocepheus*	SSPAA7964-13	KM836514
Sarcoptiformes	Tectocepheidae	*Tectocepheus velatus*	MYMCA238-11	JX836610
Sarcoptiformes	Tectocepheidae	*Tectocepheus velatus*	MYMCA512-11	JX837789
Sarcoptiformes	Tectocepheidae	*Tectocepheus velatus*	MYMCB584-11	JX838247
Sarcoptiformes	Tectocepheidae	*Tectocepheus velatus*	MYMCB585-11	JX835041
Sarcoptiformes	Tectocepheidae	*Tectocepheus velatus*	MYMCB599-11	JX836755
Sarcoptiformes	Tectocepheidae	*Tectocepheus velatus*	MYMCC195-11	JX833843
Sarcoptiformes	Tectocepheidae	*Tectocepheus velatus*	MYMCC680-11	JX837096
Sarcoptiformes	Tectocepheidae	*Tectocepheus velatus*	MYMCC712-11	JX836542
Sarcoptiformes	Tectocepheidae	*Tectocepheus velatus*	MYMCE600-12	JX835754
Sarcoptiformes	Tectocepheidae	*Tectocepheus velatus*	MYMCE628-12	JX833851
Sarcoptiformes	Tectocepheidae	*Tectocepheus velatus*	MYMCF039-12	JX835400
Sarcoptiformes	Tectocepheidae	*Tectocepheus velatus*	MYMCF041-12	JX833987
Sarcoptiformes	Tectocepheidae	*Tectocepheus velatus*	MYTMC187-09	HQ966247
Sarcoptiformes	Tectocepheidae	*Tectocepheus velatus velatus*	CHACA069-08	JX833981
Sarcoptiformes	Tectocepheidae	*Tectocepheus velatus velatus*	MYMCF204-12	JX834613
Sarcoptiformes	Tectocepheidae	*Tectocepheus velatus velatus*	MYMCF271-12	JX835959
Sarcoptiformes	Tectocepheidae	*Tectocepheus velatus velatus*	MYMCF332-12	JX837641
Sarcoptiformes	Tectocepheidae	*Tectocepheus velatus velatus*	MYMCF333-12	JX835948
Sarcoptiformes	Tectocepheidae	*Tectocepheus velatus velatus*	MYMCF428-12	JX837082
Sarcoptiformes	Tectocepheidae	*Tectocepheus velatus velatus*	MYMCF843-12	JX836457
Sarcoptiformes	Tectocepheidae	*Tectocepheus velatus velatus*	MYMCG334-12	JX837721
Sarcoptiformes	Tectocepheidae	*Tectocepheus velatus velatus*	MYMCG335-12	JX838392
Sarcoptiformes	Tegoribatidae	*Scutozetes lanceolatus*	CHACA1205-10	JX837413
Sarcoptiformes	Tegoribatidae	*Scutozetes lanceolatus*	CHACA1206-10	JX836109
Sarcoptiformes	Tegoribatidae	*Scutozetes lanceolatus*	CHACA534-09	JX836726
Sarcoptiformes	Tegoribatidae	*Scutozetes lanceolatus*	CHACA618-09	JX837410
Sarcoptiformes	Tegoribatidae	*Scutozetes lanceolatus*	CHACB1062-10	HM907186
Sarcoptiformes	Tegoribatidae	*Scutozetes lanceolatus*	CHACB847-10	HQ941514
Sarcoptiformes	Tegoribatidae	*Scutozetes lanceolatus*	CHACB987-10	HM907394
Sarcoptiformes	Tegoribatidae	*Scutozetes lanceolatus*	CHACB988-10	HM907395
Sarcoptiformes	Tegoribatidae	*Scutozetes lanceolatus*	CHACB989-10	HM907396
Sarcoptiformes	Tegoribatidae	*Scutozetes lanceolatus*	MYMCA032-11	JX834409
Sarcoptiformes	Tegoribatidae	*Scutozetes lanceolatus*	MYMCA034-11	JX835098
Sarcoptiformes	Tegoribatidae	*Scutozetes lanceolatus*	MYMCA069-11	JX834912
Sarcoptiformes	Tegoribatidae	*Scutozetes lanceolatus*	MYMCA1132-11	JX837823
Sarcoptiformes	Tegoribatidae	*Scutozetes lanceolatus*	MYMCA401-11	JX834236
Sarcoptiformes	Tegoribatidae	*Scutozetes lanceolatus*	MYMCA549-11	JX837771
Sarcoptiformes	Tegoribatidae	*Scutozetes lanceolatus*	MYMCA795-11	JX836076
Sarcoptiformes	Tegoribatidae	*Scutozetes lanceolatus*	MYMCB062-11	JX834958
Sarcoptiformes	Tegoribatidae	*Scutozetes lanceolatus*	MYMCB204-11	JX834117
Sarcoptiformes	Tegoribatidae	*Scutozetes lanceolatus*	MYMCB243-11	JX838180
Sarcoptiformes	Tegoribatidae	*Scutozetes lanceolatus*	MYMCB385-11	JX835837
Sarcoptiformes	Tegoribatidae	*Scutozetes lanceolatus*	MYMCB386-11	JX837551
Sarcoptiformes	Tegoribatidae	*Scutozetes lanceolatus*	MYMCB416-11	JX833638
Sarcoptiformes	Tegoribatidae	*Scutozetes lanceolatus*	MYMCB484-11	JX834875
Sarcoptiformes	Tegoribatidae	*Scutozetes lanceolatus*	MYMCB579-11	JX835630
Sarcoptiformes	Tegoribatidae	*Scutozetes lanceolatus*	MYMCB580-11	JX834476
Sarcoptiformes	Tegoribatidae	*Scutozetes lanceolatus*	MYMCB665-11	JX834447

Sarcoptiformes	Tegoribatidae	*Scutozetes lanceolatus*	MYMCC053-11	JX838585
Sarcoptiformes	Tegoribatidae	*Scutozetes lanceolatus*	MYMCC086-11	JX837362
Sarcoptiformes	Tegoribatidae	*Scutozetes lanceolatus*	MYMCC458-11	JX836107
Sarcoptiformes	Tegoribatidae	*Scutozetes lanceolatus*	MYMCC459-11	JX837622
Sarcoptiformes	Tegoribatidae	*Scutozetes lanceolatus*	MYMCC551-11	JX835621
Sarcoptiformes	Tegoribatidae	*Scutozetes lanceolatus*	MYMCC562-11	JX835281
Sarcoptiformes	Tegoribatidae	*Scutozetes lanceolatus*	MYMCC563-11	JX835376
Sarcoptiformes	Tegoribatidae	*Scutozetes lanceolatus*	MYMCC579-11	JX836284
Sarcoptiformes	Tegoribatidae	*Scutozetes lanceolatus*	MYMCC615-11	JX834257
Sarcoptiformes	Tegoribatidae	*Scutozetes lanceolatus*	MYMCC616-11	JX836444
Sarcoptiformes	Tegoribatidae	*Scutozetes lanceolatus*	MYMCC787-11	JX837333
Sarcoptiformes	Tegoribatidae	*Scutozetes lanceolatus*	MYMCC788-11	JX838068
Sarcoptiformes	Tegoribatidae	*Scutozetes lanceolatus*	MYMCC792-11	JX837302
Sarcoptiformes	Tegoribatidae	*Scutozetes lanceolatus*	MYMCC872-11	JX834897
Sarcoptiformes	Tegoribatidae	*Scutozetes lanceolatus*	MYMCC876-11	JX834589
Sarcoptiformes	Tegoribatidae	*Scutozetes lanceolatus*	MYMCC905-11	JX838107
Sarcoptiformes	Tegoribatidae	*Scutozetes lanceolatus*	MYMCE549-12	JX835237
Sarcoptiformes	Tegoribatidae	*Scutozetes lanceolatus*	MYMCE587-12	JX835026
Sarcoptiformes	Tegoribatidae	*Scutozetes lanceolatus*	MYMCE654-12	JX837309
Sarcoptiformes	Tegoribatidae	*Scutozetes lanceolatus*	MYMCE868-12	JX834087
Sarcoptiformes	Tegoribatidae	*Scutozetes lanceolatus*	MYMCE873-12	JX836449
Sarcoptiformes	Tegoribatidae	*Scutozetes lanceolatus*	MYMCE899-12	JX834743
Sarcoptiformes	Tegoribatidae	*Scutozetes lanceolatus*	MYMCF109-12	JX833965
Sarcoptiformes	Tegoribatidae	*Scutozetes lanceolatus*	MYMCF477-12	JX834429
Sarcoptiformes	Tegoribatidae	*Scutozetes lanceolatus*	MYMCF478-12	JX838723
Sarcoptiformes	Tegoribatidae	*Scutozetes lanceolatus*	MYMCF780-12	JX838749
Sarcoptiformes	Tegoribatidae	*Scutozetes lanceolatus*	MYMCG004-12	JX838221
Sarcoptiformes	Tegoribatidae	*Scutozetes lanceolatus*	MYMCG225-12	JX834939
Sarcoptiformes	Tegoribatidae	*Scutozetes lanceolatus*	MYMCG620-12	JX838663
Sarcoptiformes	Tegoribatidae	*Scutozetes lanceolatus*	MYMCG621-12	JX836271
Sarcoptiformes	Tegoribatidae	*Scutozetes lanceolatus*	MYTMC117-09	HQ966236
Sarcoptiformes	Tegoribatidae	*Scutozetes lanceolatus*	MYTMC129-09	HQ966239
Sarcoptiformes	Tegoribatidae	*Tegoribates americanus*	CHACA217-08	JX836605
Sarcoptiformes	Tegoribatidae	*Tegoribates americanus*	CHACA218-08	JX834509
Sarcoptiformes	Tegoribatidae	*Tegoribates americanus*	CHACA219-08	KR069365
Sarcoptiformes	Tegoribatidae	*Tegoribates americanus*	CHACA335-08	JX834749
Sarcoptiformes	Tegoribatidae	*Tegoribates americanus*	CHACA344-08	JX836282
Sarcoptiformes	Tegoribatidae	*Tegoribates americanus*	CHACA345-08	JX837001
Sarcoptiformes	Tegoribatidae	*Tegoribates americanus*	CHACA346-08	JX836851
Sarcoptiformes	Tegoribatidae	*Tegoribates americanus*	CHACB711-10	JX837568
Sarcoptiformes	Tegoribatidae	*Tegoribates americanus*	CHACC089-10	JX836747
Sarcoptiformes	Tegoribatidae	*Tegoribates americanus*	CHACC090-10	JX838457
Sarcoptiformes	Tegoribatidae	*Tegoribates americanus*	CHACC091-10	JX836911
Sarcoptiformes	Tegoribatidae	*Tegoribates americanus*	CHACC227-10	HQ941541
Sarcoptiformes	Tegoribatidae	*Tegoribates americanus*	MITMH031-07	KR069206
Sarcoptiformes	Tegoribatidae	*Tegoribates americanus*	MYMCA1016-11	JX838471
Sarcoptiformes	Tegoribatidae	*Tegoribates americanus*	MYMCA1358-11	JX835590
Sarcoptiformes	Tegoribatidae	*Tegoribates americanus*	MYMCB666-11	JX838631
Sarcoptiformes	Tegoribatidae	*Tegoribates americanus*	MYMCB762-11	JX835596
Sarcoptiformes	Tegoribatidae	*Tegoribates americanus*	MYMCC801-11	JX837871
Sarcoptiformes	Tegoribatidae	*Tegoribates americanus*	MYMCE023-12	JX837392
Sarcoptiformes	Tegoribatidae	*Tegoribates americanus*	MYMCE091-12	JX837205
Sarcoptiformes	Tegoribatidae	*Tegoribates americanus*	MYMCE374-12	JX838415

Sarcoptiformes	Tegoribatidae	*Tegoribates americanus*	MYMCF265-12	JX835656
Sarcoptiformes	Tegoribatidae	*Tegoribates americanus*	MYMCF325-12	JX834576
Sarcoptiformes	Tegoribatidae	*Tegoribates americanus*	MYMCF500-12	JX838102
Sarcoptiformes	Tegoribatidae	*Tegoribates americanus*	MYMCG604-12	JX838052
Sarcoptiformes	Tegoribatidae	*Tegoribates americanus*	MYMCG605-12	JX838734
Sarcoptiformes	Tegoribatidae		SSBAD4756-13	KM828641
Sarcoptiformes	Tegoribatidae		SSBAD5571-13	KM825343
Sarcoptiformes	Tegoribatidae		SSBAD6348-13	KM830259
Sarcoptiformes	Tenuialidae		SSBAD5546-13	KM826429
Sarcoptiformes	Terpnacaridae		MYMCA640-11	JX838217
Sarcoptiformes	Terpnacaridae		MYMCA642-11	JX833758
Sarcoptiformes	Terpnacaridae		MYMCB122-11	JX836206
Sarcoptiformes	Terpnacaridae		MYMCB123-11	JX837013
Sarcoptiformes	Terpnacaridae		MYMCB151-11	JX836171
Sarcoptiformes	Terpnacaridae		MYMCB161-11	JX835397
Sarcoptiformes	Terpnacaridae		MYMCB162-11	JX835836
Sarcoptiformes	Terpnacaridae		MYMCC161-11	JX836564
Sarcoptiformes	Terpnacaridae		MYMCC511-11	JX833715
Sarcoptiformes	Terpnacaridae		MYMCC669-11	JX836831
Sarcoptiformes	Terpnacaridae		MYMCE114-12	JX836607
Sarcoptiformes	Terpnacaridae		MYMCE115-12	JX838541
Sarcoptiformes	Terpnacaridae		MYMCE116-12	JX834526
Sarcoptiformes	Terpnacaridae		MYMCE171-12	JX834425
Sarcoptiformes	Terpnacaridae		MYMCE330-12	JX834244
Sarcoptiformes	Terpnacaridae		MYMCF034-12	JX835992
Sarcoptiformes	Terpnacaridae		MYMCF154-12	JX834403
Sarcoptiformes	Terpnacaridae		MYMCF335-12	JX835507
Sarcoptiformes	Terpnacaridae		MYMCF336-12	JX836073
Sarcoptiformes	Terpnacaridae		MYMCF359-12	JX838082
Sarcoptiformes	Terpnacaridae		MYMCF865-12	JX836036
Sarcoptiformes	Terpnacaridae		MYMCG021-12	JX838170
Sarcoptiformes	Terpnacaridae		MYMCG534-12	JX836015
Sarcoptiformes	Thyrisomidae	*Banksinoma*	MYMCE875-12	JX838578
Sarcoptiformes	Thyrisomidae	*Banksinoma*	MYMCG636-12	JX835179
Sarcoptiformes	Thyrisomidae	*Banksinoma*	MYMCG637-12	JX836288
Sarcoptiformes	Thyrisomidae	*Banksinoma spinifera*	MITMH063-07	KR069858
Sarcoptiformes	Thyrisomidae	*Banksinoma spinifera*	MYMCC169-11	JX836658
Sarcoptiformes	Thyrisomidae	*Banksinoma spinifera*	MYMCC170-11	JX833950
Sarcoptiformes	Thyrisomidae	*Banksinoma spinifera*	MYMCC174-11	JX838378
Sarcoptiformes	Thyrisomidae	*Banksinoma spinifera*	MYMCC175-11	JX838707
Sarcoptiformes	Thyrisomidae	*Banksinoma spinifera*	MYMCF873-12	JX835186
Sarcoptiformes	Trhypochthoniidae	*Mucronothus*	CHACB289-10	HM907283
Sarcoptiformes	Trhypochthoniidae	*Mucronothus*	MYMCB674-11	JX834803
Sarcoptiformes	Trhypochthoniidae	*Mucronothus*	MYMCG502-12	JX834263
Sarcoptiformes	Trhypochthoniidae	*Trhypochthonius*	MYMCG068-12	JX836394
Sarcoptiformes	Trhypochthoniidae	*Trhypochthonius*	MYMCG069-12	JX838680
Sarcoptiformes	Trhypochthoniidae	*Trhypochthonius*	SSBAA3652-12	KM835621
Sarcoptiformes	Trhypochthoniidae	*Trhypochthonius*	SSBAD5516-13	KM826695
Sarcoptiformes	Trhypochthoniidae	*Trhypochthonius*	SSBAD5536-13	KM830470
Sarcoptiformes	Trhypochthoniidae	*Trhypochthonius*	SSBAD5558-13	KM835941
Sarcoptiformes	Trhypochthoniidae	*Trhypochthonius*	SSBAD6313-13	KM828141
Sarcoptiformes	Trhypochthoniidae	*Trhypochthonius*	SSBAD6354-13	KM837197
Sarcoptiformes	Trhypochthoniidae	*Trhypochthonius cladonicolus*	CHACA709-09	JX838084

Sarcoptiformes	Trhypochthoniidae	*Trhypochthonius cladonicolus*	CHACA710-09	JX838333
Sarcoptiformes	Trhypochthoniidae	*Trhypochthonius cladonicolus*	CHACA716-09	KR070645
Sarcoptiformes	Trhypochthoniidae	*Trhypochthonius cladonicolus*	CHACA717-09	JX836772
Sarcoptiformes	Trhypochthoniidae	*Trhypochthonius cladonicolus*	CHACA719-09	JX835381
Sarcoptiformes	Trhypochthoniidae	*Trhypochthonius cladonicolus*	CHACA812-09	JX835657
Sarcoptiformes	Trhypochthoniidae	*Trhypochthonius cladonicolus*	CHACB550-10	HQ558666
Sarcoptiformes	Trhypochthoniidae	*Trhypochthonius cladonicolus*	CHACB686-10	HQ558747
Sarcoptiformes	Trhypochthoniidae	*Trhypochthonius cladonicolus*	CHACB687-10	HQ558748
Sarcoptiformes	Trhypochthoniidae	*Trhypochthonius cladonicolus*	CHACB688-10	HQ558749
Sarcoptiformes	Trhypochthoniidae	*Trhypochthonius cladonicolus*	CHACB689-10	JX837984
Sarcoptiformes	Trhypochthoniidae	*Trhypochthonius cladonicolus*	CHACB690-10	HQ558750
Sarcoptiformes	Trhypochthoniidae	*Trhypochthonius cladonicolus*	MYMCA042-11	JX837948
Sarcoptiformes	Trhypochthoniidae	*Trhypochthonius cladonicolus*	MYMCA043-11	JX833771
Sarcoptiformes	Trhypochthoniidae	*Trhypochthonius cladonicolus*	MYMCA045-11	JX838312
Sarcoptiformes	Trhypochthoniidae	*Trhypochthonius cladonicolus*	MYMCA047-11	JX835317
Sarcoptiformes	Trhypochthoniidae	*Trhypochthonius cladonicolus*	MYMCA057-11	JX837981
Sarcoptiformes	Trhypochthoniidae	*Trhypochthonius cladonicolus*	MYMCA059-11	JX837456
Sarcoptiformes	Trhypochthoniidae	*Trhypochthonius cladonicolus*	MYMCA060-11	JX836226
Sarcoptiformes	Trhypochthoniidae	*Trhypochthonius cladonicolus*	MYMCA062-11	JX838183
Sarcoptiformes	Trhypochthoniidae	*Trhypochthonius cladonicolus*	MYMCA063-11	JX838325
Sarcoptiformes	Trhypochthoniidae	*Trhypochthonius cladonicolus*	MYMCA065-11	JX836692
Sarcoptiformes	Trhypochthoniidae	*Trhypochthonius cladonicolus*	MYMCA066-11	JX835538
Sarcoptiformes	Trhypochthoniidae	*Trhypochthonius cladonicolus*	MYMCA067-11	JX834019
Sarcoptiformes	Trhypochthoniidae	*Trhypochthonius cladonicolus*	MYMCA1300-11	JX834501
Sarcoptiformes	Trhypochthoniidae	*Trhypochthonius cladonicolus*	MYMCA210-11	JX833891
Sarcoptiformes	Trhypochthoniidae	*Trhypochthonius cladonicolus*	MYMCA211-11	JX833760
Sarcoptiformes	Trhypochthoniidae	*Trhypochthonius cladonicolus*	MYMCA212-11	JX837208
Sarcoptiformes	Trhypochthoniidae	*Trhypochthonius cladonicolus*	MYMCA852-11	JX835175
Sarcoptiformes	Trhypochthoniidae	*Trhypochthonius cladonicolus*	MYMCA939-11	JX835105
Sarcoptiformes	Trhypochthoniidae	*Trhypochthonius cladonicolus*	MYMCB066-11	JX837542
Sarcoptiformes	Trhypochthoniidae	*Trhypochthonius cladonicolus*	MYMCB097-11	JX835390
Sarcoptiformes	Trhypochthoniidae	*Trhypochthonius cladonicolus*	MYMCB224-11	JX838595
Sarcoptiformes	Trhypochthoniidae	*Trhypochthonius cladonicolus*	MYMCB420-11	JX837385
Sarcoptiformes	Trhypochthoniidae	*Trhypochthonius cladonicolus*	MYMCB421-11	JX837512
Sarcoptiformes	Trhypochthoniidae	*Trhypochthonius cladonicolus*	MYMCB834-11	JX837570
Sarcoptiformes	Trhypochthoniidae	*Trhypochthonius cladonicolus*	MYMCB950-11	JX838437
Sarcoptiformes	Trhypochthoniidae	*Trhypochthonius cladonicolus*	MYMCC339-11	JX833802
Sarcoptiformes	Trhypochthoniidae	*Trhypochthonius cladonicolus*	MYMCC469-11	JX837212
Sarcoptiformes	Trhypochthoniidae	*Trhypochthonius cladonicolus*	MYMCC509-11	JX834008
Sarcoptiformes	Trhypochthoniidae	*Trhypochthonius cladonicolus*	MYMCC560-11	JX837782
Sarcoptiformes	Trhypochthoniidae	*Trhypochthonius cladonicolus*	MYMCC618-11	JX837459
Sarcoptiformes	Trhypochthoniidae	*Trhypochthonius cladonicolus*	MYMCC676-11	JX834485
Sarcoptiformes	Trhypochthoniidae	*Trhypochthonius cladonicolus*	MYMCC863-11	JX835816
Sarcoptiformes	Trhypochthoniidae	*Trhypochthonius cladonicolus*	MYMCC865-11	JX834315
Sarcoptiformes	Trhypochthoniidae	*Trhypochthonius cladonicolus*	MYMCE717-12	JX838700
Sarcoptiformes	Trhypochthoniidae	*Trhypochthonius cladonicolus*	MYMCE865-12	JX833788
Sarcoptiformes	Trhypochthoniidae	*Trhypochthonius cladonicolus*	MYMCE866-12	JX838789
Sarcoptiformes	Trhypochthoniidae	*Trhypochthonius cladonicolus*	MYMCF239-12	JX838627
Sarcoptiformes	Trhypochthoniidae	*Trhypochthonius cladonicolus*	MYMCF539-12	JX835972
Sarcoptiformes	Trhypochthoniidae	*Trhypochthonius cladonicolus*	MYMCF540-12	JX834317
Sarcoptiformes	Trhypochthoniidae	*Trhypochthonius cladonicolus*	MYMCG408-12	JX835219
Sarcoptiformes	Trhypochthoniidae	*Trhypochthonius cladonicolus*	MYTMC012-09	KR069910
Sarcoptiformes	Trhypochthoniidae	*Trhypochthonius cladonicolus*	MYTMC045-09	GU680472

Sarcoptiformes	Trhypochthoniidae	*Trhypochthonius cladonicolus*	MYTMC046-09	GU680473
Sarcoptiformes	Trhypochthoniidae	*Trhypochthonius cladonicolus*	MYTMC113-09	GU680497
Sarcoptiformes	Trhypochthoniidae	*Trhypochthonius cladonicolus*	MYTMC122-09	GU680495
Sarcoptiformes	Trhypochthoniidae	*Trhypochthonius cladonicolus*	MYTMC123-09	GU680450
Sarcoptiformes	Trhypochthoniidae	*Trhypochthonius cladonicolus*	MYTMC126-09	GU680446
Sarcoptiformes	Trhypochthoniidae	*Trhypochthonius cladonicolus*	MYTMC128-09	GU680447
Sarcoptiformes	Trhypochthoniidae	*Trhypochthonius cladonicolus*	MYTMC131-09	KR070437
Sarcoptiformes	Trhypochthoniidae	*Trhypochthonius cladonicolus*	MYTMC144-09	GU680448
Sarcoptiformes	Trhypochthoniidae	*Trhypochthonius cladonicolus*	MYTMC145-09	GU680445
Sarcoptiformes	Trhypochthoniidae	*Trhypochthonius cladonicolus*	MYTMC147-09	GU680429
Sarcoptiformes	Trhypochthoniidae	*Trhypochthonius cladonicolus*	MYTMC173-09	GU680426
Sarcoptiformes	Trhypochthoniidae	*Trhypochthonius tectorum*	CHACA1111-10	JX836950
Sarcoptiformes	Trhypochthoniidae	*Trhypochthonius tectorum*	CHACA173-08	JX838007
Sarcoptiformes	Trhypochthoniidae	*Trhypochthonius tectorum*	CHACA174-08	JX835686
Sarcoptiformes	Trhypochthoniidae	*Trhypochthonius tectorum*	CHACA175-08	JX836349
Sarcoptiformes	Trhypochthoniidae	*Trhypochthonius tectorum*	CHACA177-08	JX836676
Sarcoptiformes	Trhypochthoniidae	*Trhypochthonius tectorum*	CHACA178-08	JX837946
Sarcoptiformes	Trhypochthoniidae	*Trhypochthonius tectorum*	CHACA187-08	JX834568
Sarcoptiformes	Trhypochthoniidae	*Trhypochthonius tectorum*	CHACA189-08	JX836840
Sarcoptiformes	Trhypochthoniidae	*Trhypochthonius tectorum*	CHACA192-08	JX835848
Sarcoptiformes	Trhypochthoniidae	*Trhypochthonius tectorum*	CHACA195-08	JX834918
Sarcoptiformes	Trhypochthoniidae	*Trhypochthonius tectorum*	CHACA196-08	JX834307
Sarcoptiformes	Trhypochthoniidae	*Trhypochthonius tectorum*	CHACA197-08	JX836181
Sarcoptiformes	Trhypochthoniidae	*Trhypochthonius tectorum*	CHACA280-08	JX836842
Sarcoptiformes	Trhypochthoniidae	*Trhypochthonius tectorum*	CHACA281-08	JX834675
Sarcoptiformes	Trhypochthoniidae	*Trhypochthonius tectorum*	CHACA291-08	JX838086
Sarcoptiformes	Trhypochthoniidae	*Trhypochthonius tectorum*	CHACA292-08	JX836371
Sarcoptiformes	Trhypochthoniidae	*Trhypochthonius tectorum*	CHACA293-08	JX836901
Sarcoptiformes	Trhypochthoniidae	*Trhypochthonius tectorum*	CHACA294-08	JX837836
Sarcoptiformes	Trhypochthoniidae	*Trhypochthonius tectorum*	CHACA295-08	JX838213
Sarcoptiformes	Trhypochthoniidae	*Trhypochthonius tectorum*	CHACA296-08	JX838176
Sarcoptiformes	Trhypochthoniidae	*Trhypochthonius tectorum*	CHACA297-08	JX836657
Sarcoptiformes	Trhypochthoniidae	*Trhypochthonius tectorum*	CHACA298-08	JX836855
Sarcoptiformes	Trhypochthoniidae	*Trhypochthonius tectorum*	CHACA299-08	JX836409
Sarcoptiformes	Trhypochthoniidae	*Trhypochthonius tectorum*	CHACA309-08	JX833685
Sarcoptiformes	Trhypochthoniidae	*Trhypochthonius tectorum*	CHACA311-08	JX835827
Sarcoptiformes	Trhypochthoniidae	*Trhypochthonius tectorum*	CHACA312-08	JX835866
Sarcoptiformes	Trhypochthoniidae	*Trhypochthonius tectorum*	CHACA313-08	JX837070
Sarcoptiformes	Trhypochthoniidae	*Trhypochthonius tectorum*	CHACA358-08	JX837377
Sarcoptiformes	Trhypochthoniidae	*Trhypochthonius tectorum*	CHACA363-08	JX835368
Sarcoptiformes	Trhypochthoniidae	*Trhypochthonius tectorum*	CHACA580-09	JX834274
Sarcoptiformes	Trhypochthoniidae	*Trhypochthonius tectorum*	CHACA581-09	JX838623
Sarcoptiformes	Trhypochthoniidae	*Trhypochthonius tectorum*	CHACA582-09	JX836166
Sarcoptiformes	Trhypochthoniidae	*Trhypochthonius tectorum*	CHACA587-09	JX835869
Sarcoptiformes	Trhypochthoniidae	*Trhypochthonius tectorum*	CHACA811-09	JX835945
Sarcoptiformes	Trhypochthoniidae	*Trhypochthonius tectorum*	CHACB094-10	HQ558393
Sarcoptiformes	Trhypochthoniidae	*Trhypochthonius tectorum*	CHACB102-10	HQ558398
Sarcoptiformes	Trhypochthoniidae	*Trhypochthonius tectorum*	CHACB1026-10	HM907159
Sarcoptiformes	Trhypochthoniidae	*Trhypochthonius tectorum*	CHACB1027-10	HM907160
Sarcoptiformes	Trhypochthoniidae	*Trhypochthonius tectorum*	CHACB1028-10	HM907161
Sarcoptiformes	Trhypochthoniidae	*Trhypochthonius tectorum*	CHACB1029-10	HM907162
Sarcoptiformes	Trhypochthoniidae	*Trhypochthonius tectorum*	CHACB1030-10	HM907163
Sarcoptiformes	Trhypochthoniidae	*Trhypochthonius tectorum*	CHACB103-10	HQ558399

Sarcoptiformes	Trhypochthoniidae	*Trhypochthonius tectorum*	CHACB1031-10	HM907164
Sarcoptiformes	Trhypochthoniidae	*Trhypochthonius tectorum*	CHACB1032-10	HM907165
Sarcoptiformes	Trhypochthoniidae	*Trhypochthonius tectorum*	CHACB1033-10	HM907166
Sarcoptiformes	Trhypochthoniidae	*Trhypochthonius tectorum*	CHACB1034-10	HM907167
Sarcoptiformes	Trhypochthoniidae	*Trhypochthonius tectorum*	CHACB1035-10	HM907168
Sarcoptiformes	Trhypochthoniidae	*Trhypochthonius tectorum*	CHACB104-10	JX834344
Sarcoptiformes	Trhypochthoniidae	*Trhypochthonius tectorum*	CHACB105-10	HQ558400
Sarcoptiformes	Trhypochthoniidae	*Trhypochthonius tectorum*	CHACB106-10	HQ558401
Sarcoptiformes	Trhypochthoniidae	*Trhypochthonius tectorum*	CHACB108-10	HQ558402
Sarcoptiformes	Trhypochthoniidae	*Trhypochthonius tectorum*	CHACB1114-10	HM907208
Sarcoptiformes	Trhypochthoniidae	*Trhypochthonius tectorum*	CHACB1115-10	HM907209
Sarcoptiformes	Trhypochthoniidae	*Trhypochthonius tectorum*	CHACB154-10	HQ558437
Sarcoptiformes	Trhypochthoniidae	*Trhypochthonius tectorum*	CHACB155-10	HQ558438
Sarcoptiformes	Trhypochthoniidae	*Trhypochthonius tectorum*	CHACB156-10	HQ558439
Sarcoptiformes	Trhypochthoniidae	*Trhypochthonius tectorum*	CHACB286-10	HM907280
Sarcoptiformes	Trhypochthoniidae	*Trhypochthonius tectorum*	CHACB445-10	HQ558599
Sarcoptiformes	Trhypochthoniidae	*Trhypochthonius tectorum*	CHACB914-10	HM907343
Sarcoptiformes	Trhypochthoniidae	*Trhypochthonius tectorum*	CHACB915-10	HM907344
Sarcoptiformes	Trhypochthoniidae	*Trhypochthonius tectorum*	CHACB916-10	HM907345
Sarcoptiformes	Trhypochthoniidae	*Trhypochthonius tectorum*	CHACB917-10	HM907346
Sarcoptiformes	Trhypochthoniidae	*Trhypochthonius tectorum*	CHACB918-10	HM907347
Sarcoptiformes	Trhypochthoniidae	*Trhypochthonius tectorum*	CHACB919-10	HM907348
Sarcoptiformes	Trhypochthoniidae	*Trhypochthonius tectorum*	CHACC240-10	HQ941547
Sarcoptiformes	Trhypochthoniidae	*Trhypochthonius tectorum*	CHACC241-10	HQ941548
Sarcoptiformes	Trhypochthoniidae	*Trhypochthonius tectorum*	MITMH024-07	KR070200
Sarcoptiformes	Trhypochthoniidae	*Trhypochthonius tectorum*	MYMCA072-11	JX837242
Sarcoptiformes	Trhypochthoniidae	*Trhypochthonius tectorum*	MYMCA074-11	JX837765
Sarcoptiformes	Trhypochthoniidae	*Trhypochthonius tectorum*	MYMCA075-11	JX835011
Sarcoptiformes	Trhypochthoniidae	*Trhypochthonius tectorum*	MYMCA076-11	JX834949
Sarcoptiformes	Trhypochthoniidae	*Trhypochthonius tectorum*	MYMCA1014-11	JX835625
Sarcoptiformes	Trhypochthoniidae	*Trhypochthonius tectorum*	MYMCA1015-11	JX837018
Sarcoptiformes	Trhypochthoniidae	*Trhypochthonius tectorum*	MYMCA1126-11	JX838629
Sarcoptiformes	Trhypochthoniidae	*Trhypochthonius tectorum*	MYMCA125-11	JX838255
Sarcoptiformes	Trhypochthoniidae	*Trhypochthonius tectorum*	MYMCA126-11	JX834184
Sarcoptiformes	Trhypochthoniidae	*Trhypochthonius tectorum*	MYMCA127-11	JX834816
Sarcoptiformes	Trhypochthoniidae	*Trhypochthonius tectorum*	MYMCA128-11	JX835753
Sarcoptiformes	Trhypochthoniidae	*Trhypochthonius tectorum*	MYMCA129-11	JX836972
Sarcoptiformes	Trhypochthoniidae	*Trhypochthonius tectorum*	MYMCA131-11	JX833835
Sarcoptiformes	Trhypochthoniidae	*Trhypochthonius tectorum*	MYMCA133-11	JX838062
Sarcoptiformes	Trhypochthoniidae	*Trhypochthonius tectorum*	MYMCA135-11	JX837233
Sarcoptiformes	Trhypochthoniidae	*Trhypochthonius tectorum*	MYMCA1352-11	JX834258
Sarcoptiformes	Trhypochthoniidae	*Trhypochthonius tectorum*	MYMCA1461-11	JX837118
Sarcoptiformes	Trhypochthoniidae	*Trhypochthonius tectorum*	MYMCA1463-11	JX836376
Sarcoptiformes	Trhypochthoniidae	*Trhypochthonius tectorum*	MYMCA1509-11	JX838687
Sarcoptiformes	Trhypochthoniidae	*Trhypochthonius tectorum*	MYMCA251-11	JX834960
Sarcoptiformes	Trhypochthoniidae	*Trhypochthonius tectorum*	MYMCA252-11	JX836719
Sarcoptiformes	Trhypochthoniidae	*Trhypochthonius tectorum*	MYMCA274-11	JX838326
Sarcoptiformes	Trhypochthoniidae	*Trhypochthonius tectorum*	MYMCA405-11	JX836893
Sarcoptiformes	Trhypochthoniidae	*Trhypochthonius tectorum*	MYMCA543-11	JX833827
Sarcoptiformes	Trhypochthoniidae	*Trhypochthonius tectorum*	MYMCA604-11	JX835403
Sarcoptiformes	Trhypochthoniidae	*Trhypochthonius tectorum*	MYMCA605-11	JX837565
Sarcoptiformes	Trhypochthoniidae	*Trhypochthonius tectorum*	MYMCA679-11	JX837033
Sarcoptiformes	Trhypochthoniidae	*Trhypochthonius tectorum*	MYMCA741-11	JX837609

Sarcoptiformes	Trhypochthoniidae	*Trhypochthonius tectorum*	MYMCB057-11	JX834647
Sarcoptiformes	Trhypochthoniidae	*Trhypochthonius tectorum*	MYMCB143-11	JX837175
Sarcoptiformes	Trhypochthoniidae	*Trhypochthonius tectorum*	MYMCB146-11	JX835996
Sarcoptiformes	Trhypochthoniidae	*Trhypochthonius tectorum*	MYMCB542-11	JX834711
Sarcoptiformes	Trhypochthoniidae	*Trhypochthonius tectorum*	MYMCB602-11	JX834652
Sarcoptiformes	Trhypochthoniidae	*Trhypochthonius tectorum*	MYMCB603-11	JX838017
Sarcoptiformes	Trhypochthoniidae	*Trhypochthonius tectorum*	MYMCB624-11	JX834885
Sarcoptiformes	Trhypochthoniidae	*Trhypochthonius tectorum*	MYMCB742-11	JX837328
Sarcoptiformes	Trhypochthoniidae	*Trhypochthonius tectorum*	MYMCB806-11	JX837199
Sarcoptiformes	Trhypochthoniidae	*Trhypochthonius tectorum*	MYMCB854-11	JX834016
Sarcoptiformes	Trhypochthoniidae	*Trhypochthonius tectorum*	MYMCB870-11	JX836441
Sarcoptiformes	Trhypochthoniidae	*Trhypochthonius tectorum*	MYMCB878-11	JX836673
Sarcoptiformes	Trhypochthoniidae	*Trhypochthonius tectorum*	MYMCB930-11	JX835099
Sarcoptiformes	Trhypochthoniidae	*Trhypochthonius tectorum*	MYMCC001-11	JX836089
Sarcoptiformes	Trhypochthoniidae	*Trhypochthonius tectorum*	MYMCC091-11	JX838355
Sarcoptiformes	Trhypochthoniidae	*Trhypochthonius tectorum*	MYMCC148-11	JX835649
Sarcoptiformes	Trhypochthoniidae	*Trhypochthonius tectorum*	MYMCC231-11	JX837815
Sarcoptiformes	Trhypochthoniidae	*Trhypochthonius tectorum*	MYMCC293-11	JX838067
Sarcoptiformes	Trhypochthoniidae	*Trhypochthonius tectorum*	MYMCC327-11	JX837685
Sarcoptiformes	Trhypochthoniidae	*Trhypochthonius tectorum*	MYMCC364-11	JX836060
Sarcoptiformes	Trhypochthoniidae	*Trhypochthonius tectorum*	MYMCC607-11	JX838655
Sarcoptiformes	Trhypochthoniidae	*Trhypochthonius tectorum*	MYMCC721-11	JX834719
Sarcoptiformes	Trhypochthoniidae	*Trhypochthonius tectorum*	MYMCC737-11	JX833667
Sarcoptiformes	Trhypochthoniidae	*Trhypochthonius tectorum*	MYMCC841-11	JX835412
Sarcoptiformes	Trhypochthoniidae	*Trhypochthonius tectorum*	MYMCC900-11	JX838050
Sarcoptiformes	Trhypochthoniidae	*Trhypochthonius tectorum*	MYMCC914-11	JX834228
Sarcoptiformes	Trhypochthoniidae	*Trhypochthonius tectorum*	MYMCD004-11	JX838446
Sarcoptiformes	Trhypochthoniidae	*Trhypochthonius tectorum*	MYMCD064-11	JX834314
Sarcoptiformes	Trhypochthoniidae	*Trhypochthonius tectorum*	MYMCD065-11	JX837279
Sarcoptiformes	Trhypochthoniidae	*Trhypochthonius tectorum*	MYMCD161-11	JX836370
Sarcoptiformes	Trhypochthoniidae	*Trhypochthonius tectorum*	MYMCE371-12	JX834606
Sarcoptiformes	Trhypochthoniidae	*Trhypochthonius tectorum*	MYMCE372-12	JX837216
Sarcoptiformes	Trhypochthoniidae	*Trhypochthonius tectorum*	MYMCE812-12	JX837677
Sarcoptiformes	Trhypochthoniidae	*Trhypochthonius tectorum*	MYMCF224-12	JX836717
Sarcoptiformes	Trhypochthoniidae	*Trhypochthonius tectorum*	MYMCF386-12	JX836317
Sarcoptiformes	Trhypochthoniidae	*Trhypochthonius tectorum*	MYMCF387-12	JX834281
Sarcoptiformes	Trhypochthoniidae	*Trhypochthonius tectorum*	MYMCF617-12	JX834333
Sarcoptiformes	Trhypochthoniidae	*Trhypochthonius tectorum*	MYMCF691-12	JX837439
Sarcoptiformes	Trhypochthoniidae	*Trhypochthonius tectorum*	MYMCF765-12	JX835128
Sarcoptiformes	Trhypochthoniidae	*Trhypochthonius tectorum*	MYMCF821-12	JX834790
Sarcoptiformes	Trhypochthoniidae	*Trhypochthonius tectorum*	MYMCG376-12	JX834000
Sarcoptiformes	Trhypochthoniidae	*Trhypochthonius tectorum*	MYMCG460-12	JX837116
Sarcoptiformes	Trhypochthoniidae	*Trhypochthonius tectorum*	MYMCG461-12	JX835174
Sarcoptiformes	Trhypochthoniidae	*Trhypochthonius tectorum*	MYMCG602-12	JX834784
Sarcoptiformes	Trhypochthoniidae	*Trhypochthonius tectorum*	MYMCG603-12	JX834505
Sarcoptiformes	Trhypochthoniidae	*Trhypochthonius tectorum*	MYTMC166-09	GU680427
Sarcoptiformes	Trhypochthoniidae	*Trhypochthonius tectorum*	SSEIB4561-13	KM830228
Sarcoptiformes	Trhypochthoniidae	*Trhypochthonius tectorum*	SSEIB4586-13	KM839025
Sarcoptiformes	Trhypochthoniidae	*Trhypochthonius tectorum*	SSPAA7837-13	KM830597
Sarcoptiformes	Trhypochthoniidae		MYMCE056-12	JX835665
Sarcoptiformes	Trhypochthoniidae		MYMCE057-12	JX838559
Sarcoptiformes	Trhypochthoniidae		MYMCF904-12	JX836582
Sarcoptiformes	Trouessartiidae	*Trouessartia appendiculata*	FMEP315-14	KP193765

Sarcoptiformes	Trouessartiidae	*Trouessartia bifurcata*	FMEP316-14	KP193766
Sarcoptiformes	Trouessartiidae	*Trouessartia bifurcata*	FMEP317-14	KP193767
Sarcoptiformes	Trouessartiidae	*Trouessartia inexpectata*	FMEP164-14	KP193768
Sarcoptiformes	Trouessartiidae	*Trouessartia jedliczkai*	FMEP033-14	KP193786
Sarcoptiformes	Trouessartiidae	*Trouessartia jedliczkai*	FMEP034-14	KP193785
Sarcoptiformes	Trouessartiidae	*Trouessartia jedliczkai*	FMEP170-14	KP193769
Sarcoptiformes	Trouessartiidae	*Trouessartia jedliczkai*	FMEP192-14	KP193772
Sarcoptiformes	Trouessartiidae	*Trouessartia jedliczkai*	FMEP193-14	KP193773
Sarcoptiformes	Trouessartiidae	*Trouessartia jedliczkai*	FMEP254-14	KP193770
Sarcoptiformes	Trouessartiidae	*Trouessartia jedliczkai*	FMEP255-14	KP193771
Sarcoptiformes	Trouessartiidae	*Trouessartia jedliczkai*	FMEP256-14	KP193774
Sarcoptiformes	Trouessartiidae	*Trouessartia jedliczkai*	FMEP339-14	KP193787
Sarcoptiformes	Trouessartiidae	*Trouessartia jedliczkai*	FMEP340-14	KP193784
Sarcoptiformes	Trouessartiidae	*Trouessartia ripariae*	FMEP036-14	KP193794
Sarcoptiformes	Trouessartiidae	*Trouessartia ripariae*	FMEP344-14	KP193793
Sarcoptiformes	Trouessartiidae	*Trouessartia ripariae*	FMEP345-14	KP193792
Sarcoptiformes	Trouessartiidae	*Trouessartia rosterii*	FMEP037-14	KP193796
Sarcoptiformes	Trouessartiidae	*Trouessartia rosterii*	FMEP038-14	KP193798
Sarcoptiformes	Trouessartiidae	*Trouessartia rosterii*	FMEP346-14	KP193795
Sarcoptiformes	Trouessartiidae	*Trouessartia rosterii*	FMEP347-14	KP193797
Sarcoptiformes	Trouessartiidae	*Trouessartia rubecula*	FMEP040-14	KP193808
Sarcoptiformes	Trouessartiidae	*Trouessartia rubecula*	FMEP041-14	KP193806
Sarcoptiformes	Trouessartiidae	*Trouessartia rubecula*	FMEP066-14	KP193807
Sarcoptiformes	Trouessartiidae	*Trouessartia rubecula*	FMEP092-14	KP193801
Sarcoptiformes	Trouessartiidae	*Trouessartia rubecula*	FMEP138-14	KP193809
Sarcoptiformes	Trouessartiidae	*Trouessartia rubecula*	FMEP139-14	KP193800
Sarcoptiformes	Trouessartiidae	*Trouessartia rubecula*	FMEP140-14	KP193803
Sarcoptiformes	Trouessartiidae	*Trouessartia rubecula*	FMEP176-14	KP193805
Sarcoptiformes	Trouessartiidae	*Trouessartia rubecula*	FMEP178-14	KP193804
Sarcoptiformes	Trouessartiidae	*Trouessartia rubecula*	FMEP226-14	KP193802
Sarcoptiformes	Trouessartiidae	*Trouessartia rubecula*	FMEP227-14	KP193799
Sarcoptiformes	Trouessartiidae	*Trouessartia simillima*	FMEP348-14	KP193810
Sarcoptiformes	Trouessartiidae	*Trouessartia simillima*	FMEP349-14	KP193811
Sarcoptiformes	Trouessartiidae	*Trouessartia simillima*	FMEP351-14	KP193812
Sarcoptiformes	Trouessartiidae	*Trouessartia sp. AMUFM627*	GBCH4467-10	GQ864334
Sarcoptiformes	Trouessartiidae	*Trouessartia swidwiensis*	FMEP188-14	KP193813
Sarcoptiformes	Trouessartiidae	*Trouessartia tenuipilata*	FMEP311-14	KP193816
Sarcoptiformes	Trouessartiidae	*Trouessartia tenuipilata*	FMEP312-14	KP193815
Sarcoptiformes	Trouessartiidae	*Trouessartia tenuipilata*	FMEP313-14	KP193814
Sarcoptiformes	Trouessartiidae	*Trouessartia trouessarti*	FMEP017-14	KP193819
Sarcoptiformes	Trouessartiidae	*Trouessartia trouessarti*	FMEP141-14	KP193817
Sarcoptiformes	Trouessartiidae	*Trouessartia trouessarti*	FMEP142-14	KP193818
Sarcoptiformes	Xolalgidae	*Glaucalges attenuatus*	GBCH3318-09	EU271958
Sarcoptiformes	Xolalgidae	*Glaucalges attenuatus*	GBCH3319-09	EU271957
Sarcoptiformes	Xolalgidae	*Glaucalges sp. n. MD-2007*	GBCH3320-09	EU271956
Sarcoptiformes	Xolalgidae	*Glaucalges sp. n. MD-2007*	GBCH3321-09	EU271955
Sarcoptiformes	Xolalgidae	*Ingrassia sp. AMUFM204*	GBCH4455-10	GQ864347
Sarcoptiformes	Xolalgidae	*Ingrassia sp. n. MD-2007*	GBCH3316-09	EU271954
Schizomida	Hubbardiidae	*Stenochrus portoricensis*	GBCH11738-13	JX280415
Schizomida	Hubbardiidae	*Stenochrus portoricensis*	GBCH11739-13	JX280414
Schizomida	Hubbardiidae	*Stenochrus portoricensis*	GBCH11740-13	JX280413
Schizomida			GBCH7781-13	JN018145
Schizomida			GBCH7782-13	JN018144

Schizomida			GBCH7783-13	JN018143
Scorpiones	Buthidae	*Androctonus hoggarensis*	GBCH7776-13	JN018150
Scorpiones	Buthidae	*Androctonus mauritanicus*	GBCH6935-13	JF820097
Scorpiones	Buthidae	*Buthus*	GBCH11869-13	JQ775966
Scorpiones	Buthidae	*Buthus*	GBCH11870-13	JQ775965
Scorpiones	Buthidae	*Buthus*	GBCH11871-13	JQ775964
Scorpiones	Buthidae	*Buthus*	GBCH11925-13	JQ775910
Scorpiones	Buthidae	*Buthus*	GBCH11927-13	JQ775908
Scorpiones	Buthidae	*Buthus*	GBCH11928-13	JQ775907
Scorpiones	Buthidae	*Buthus*	GBCH11929-13	JQ775906
Scorpiones	Buthidae	*Buthus*	GBCH6936-13	JF820096
Scorpiones	Buthidae	*Buthus ibericus*	GBCH4342-10	GQ168542
Scorpiones	Buthidae	*Buthus ibericus*	GBCH4344-10	GQ168540
Scorpiones	Buthidae	*Buthus ibericus*	GBCH4345-10	GQ168539
Scorpiones	Buthidae	*Buthus ibericus*	GBCH4346-10	GQ168538
Scorpiones	Buthidae	*Buthus ibericus*	GBCH4347-10	GQ168537
Scorpiones	Buthidae	*Buthus ibericus*	GBCH4348-10	GQ168536
Scorpiones	Buthidae	*Buthus ibericus*	GBCH4351-10	GQ168533
Scorpiones	Buthidae	*Buthus ibericus*	GBCH4352-10	GQ168532
Scorpiones	Buthidae	*Buthus ibericus*	GBCH4353-10	GQ168531
Scorpiones	Buthidae	*Buthus ibericus*	GBCH4354-10	GQ168530
Scorpiones	Buthidae	*Buthus ibericus*	GBCH4355-10	GQ168529
Scorpiones	Buthidae	*Buthus ibericus*	GBCH4356-10	GQ168528
Scorpiones	Buthidae	*Buthus ibericus*	GBCH4357-10	GQ168527
Scorpiones	Buthidae	*Buthus ibericus*	GBCH4364-10	GQ168520
Scorpiones	Buthidae	*Buthus ibericus*	GBCH4365-10	GQ168519
Scorpiones	Buthidae	*Buthus occitanus*	GBCH0329-06	AJ506905
Scorpiones	Buthidae	*Buthus occitanus*	GBCH0330-06	AJ506906
Scorpiones	Buthidae	*Buthus occitanus*	GBCH0332-06	AJ506908
Scorpiones	Buthidae	*Buthus occitanus*	GBCH0333-06	AJ506909
Scorpiones	Buthidae	*Buthus occitanus*	GBCH0334-06	AJ506910
Scorpiones	Buthidae	*Buthus occitanus*	GBCH0349-06	AJ517296
Scorpiones	Buthidae	*Buthus occitanus*	GBCH2387-08	EU523755
Scorpiones	Buthidae	*Buthus occitanus*	GBCH3212-08	NC_010765
Scorpiones	Buthidae	*Buthus occitanus*	GBCH4360-10	GQ168524
Scorpiones	Buthidae	*Buthus sp. Sc095*	GBCH4363-10	GQ168521
Scorpiones	Buthidae	*Buthus sp. Sc096*	GBCH4362-10	GQ168522
Scorpiones	Buthidae	*Buthus sp. Sc098*	GBCH4361-10	GQ168523
Scorpiones	Buthidae	*Buthus sp. Sc1*	GBCH3421-09	FJ198055
Scorpiones	Buthidae	*Buthus sp. Sc104*	GBCH4358-10	GQ168526
Scorpiones	Buthidae	*Buthus sp. Sc114*	GBCH4350-10	GQ168534
Scorpiones	Buthidae	*Buthus sp. Sc161*	GBCH4343-10	GQ168541
Scorpiones	Buthidae	*Buthus sp. Sc4*	GBCH3420-09	FJ198056
Scorpiones	Buthidae	*Centruroides exilicauda*	GBCH1946-07	AY995833
Scorpiones	Buthidae	*Centruroides flavopictus*	GBCH1953-07	AY995825
Scorpiones	Buthidae	*Centruroides fulvipes*	GBCH1945-07	AY995834
Scorpiones	Buthidae	*Centruroides gracilis*	GBCH1949-07	AY995830
Scorpiones	Buthidae	*Centruroides infamatus*	GBCH1952-07	AY995826
Scorpiones	Buthidae	*Centruroides limpidus*	GBCH1089-06	AY803353
Scorpiones	Buthidae	*Centruroides nigrescens*	GBCH1951-07	AY995828
Scorpiones	Buthidae	*Centruroides nigrimanus*	GBCH3771-09	AY995838
Scorpiones	Buthidae	*Centruroides nigrovariatus*	GBCH1944-07	AY995837
Scorpiones	Buthidae	*Centruroides noxius*	GBCH1950-07	AY995829

Scorpiones	Buthidae	*Centruroides sculpturatus*	GBCH1948-07	AY995831
Scorpiones	Buthidae	*Centruroides sculpturatus*	GBCH2511-08	EU381065
Scorpiones	Buthidae	*Centruroides thorellii*	GBCH1947-07	AY995832
Scorpiones	Buthidae	*Centruroides vittatus*	GBCH2461-08	EU404118
Scorpiones	Buthidae	*Centruroides vittatus*	GBCH2462-08	EU404117
Scorpiones	Buthidae	*Centruroides vittatus*	GBCH2463-08	EU404116
Scorpiones	Buthidae	*Centruroides vittatus*	GBCH2464-08	EU404115
Scorpiones	Buthidae	*Centruroides vittatus*	GBCH2465-08	EU404114
Scorpiones	Buthidae	*Centruroides vittatus*	GBCH2466-08	EU381110
Scorpiones	Buthidae	*Centruroides vittatus*	GBCH2467-08	EU381109
Scorpiones	Buthidae	*Centruroides vittatus*	GBCH2468-08	EU381108
Scorpiones	Buthidae	*Centruroides vittatus*	GBCH2469-08	EU381107
Scorpiones	Buthidae	*Centruroides vittatus*	GBCH2470-08	EU381106
Scorpiones	Buthidae	*Centruroides vittatus*	GBCH2471-08	EU381105
Scorpiones	Buthidae	*Centruroides vittatus*	GBCH2472-08	EU381104
Scorpiones	Buthidae	*Centruroides vittatus*	GBCH2473-08	EU381103
Scorpiones	Buthidae	*Centruroides vittatus*	GBCH2474-08	EU381102
Scorpiones	Buthidae	*Centruroides vittatus*	GBCH2475-08	EU381101
Scorpiones	Buthidae	*Centruroides vittatus*	GBCH2476-08	EU381100
Scorpiones	Buthidae	*Centruroides vittatus*	GBCH2477-08	EU381099
Scorpiones	Buthidae	*Centruroides vittatus*	GBCH2478-08	EU381098
Scorpiones	Buthidae	*Centruroides vittatus*	GBCH2479-08	EU381097
Scorpiones	Buthidae	*Centruroides vittatus*	GBCH2480-08	EU381096
Scorpiones	Buthidae	*Centruroides vittatus*	GBCH2481-08	EU381095
Scorpiones	Buthidae	*Centruroides vittatus*	GBCH2482-08	EU381094
Scorpiones	Buthidae	*Centruroides vittatus*	GBCH2483-08	EU381093
Scorpiones	Buthidae	*Centruroides vittatus*	GBCH2484-08	EU381092
Scorpiones	Buthidae	*Centruroides vittatus*	GBCH2485-08	EU381091
Scorpiones	Buthidae	*Centruroides vittatus*	GBCH2486-08	EU381090
Scorpiones	Buthidae	*Centruroides vittatus*	GBCH2487-08	EU381089
Scorpiones	Buthidae	*Centruroides vittatus*	GBCH2488-08	EU381088
Scorpiones	Buthidae	*Centruroides vittatus*	GBCH2489-08	EU381087
Scorpiones	Buthidae	*Centruroides vittatus*	GBCH2490-08	EU381086
Scorpiones	Buthidae	*Centruroides vittatus*	GBCH2491-08	EU381085
Scorpiones	Buthidae	*Centruroides vittatus*	GBCH2492-08	EU381084
Scorpiones	Buthidae	*Centruroides vittatus*	GBCH2493-08	EU381083
Scorpiones	Buthidae	*Centruroides vittatus*	GBCH2494-08	EU381082
Scorpiones	Buthidae	*Centruroides vittatus*	GBCH2495-08	EU381081
Scorpiones	Buthidae	*Centruroides vittatus*	GBCH2496-08	EU381080
Scorpiones	Buthidae	*Centruroides vittatus*	GBCH2497-08	EU381079
Scorpiones	Buthidae	*Centruroides vittatus*	GBCH2498-08	EU381078
Scorpiones	Buthidae	*Centruroides vittatus*	GBCH2499-08	EU381077
Scorpiones	Buthidae	*Centruroides vittatus*	GBCH2500-08	EU381076
Scorpiones	Buthidae	*Centruroides vittatus*	GBCH2501-08	EU381075
Scorpiones	Buthidae	*Centruroides vittatus*	GBCH2502-08	EU381074
Scorpiones	Buthidae	*Centruroides vittatus*	GBCH2503-08	EU381073
Scorpiones	Buthidae	*Centruroides vittatus*	GBCH2504-08	EU381072
Scorpiones	Buthidae	*Centruroides vittatus*	GBCH2505-08	EU381071
Scorpiones	Buthidae	*Centruroides vittatus*	GBCH2506-08	EU381070
Scorpiones	Buthidae	*Centruroides vittatus*	GBCH2507-08	EU381069
Scorpiones	Buthidae	*Centruroides vittatus*	GBCH2508-08	EU381068
Scorpiones	Buthidae	*Centruroides vittatus*	GBCH2509-08	EU381067
Scorpiones	Buthidae	*Centruroides vittatus*	GBCH2510-08	EU381066

Scorpiones	Buthidae	*Centruroides vittatus*	GBCH2512-08	EU381064
Scorpiones	Buthidae	*Centruroides vittatus*	GBCH2513-08	EU381063
Scorpiones	Buthidae	*Centruroides vittatus*	GBCH2514-08	EU381062
Scorpiones	Buthidae	*Centruroides vittatus*	GBCH2515-08	EU381061
Scorpiones	Buthidae	*Centruroides vittatus*	GBCH2516-08	EU381060
Scorpiones	Buthidae	*Centruroides vittatus*	GBCH2517-08	EU381059
Scorpiones	Buthidae	*Centruroides vittatus*	GBCH2518-08	EU381058
Scorpiones	Buthidae	*Centruroides vittatus*	GBCH2519-08	EU381057
Scorpiones	Buthidae	*Centruroides vittatus*	GBCH2520-08	EU381056
Scorpiones	Buthidae	*Centruroides vittatus*	GBCH2521-08	EU381055
Scorpiones	Buthidae	*Centruroides vittatus*	GBCH2522-08	EU381054
Scorpiones	Buthidae	*Centruroides vittatus*	GBCH2523-08	EU381053
Scorpiones	Buthidae	*Centruroides vittatus*	GBCH2524-08	EU381052
Scorpiones	Buthidae	*Centruroides vittatus*	GBCH2525-08	EU381051
Scorpiones	Buthidae	*Centruroides vittatus*	GBCH2526-08	EU381050
Scorpiones	Buthidae	*Centruroides vittatus*	GBCH2527-08	EU381049
Scorpiones	Buthidae	*Centruroides vittatus*	GBCH2528-08	EU381048
Scorpiones	Buthidae	*Centruroides vittatus*	GBCH2529-08	EU381047
Scorpiones	Buthidae	*Centruroides vittatus*	GBCH2530-08	EU381046
Scorpiones	Buthidae	*Centruroides vittatus*	RBCH027-04	DQ127507
Scorpiones	Buthidae	*Grosphus flavopiceus*	GBCH7625-13	JQ514254
Scorpiones	Buthidae	*Grosphus flavopiceus*	GBCH7774-13	JN018152
Scorpiones	Buthidae	*Hottentotta franzwerneri*	GBCH6937-13	JF820095
Scorpiones	Buthidae	*Hottentotta franzwerneri*	GBCH6938-13	JF820094
Scorpiones	Buthidae	*Hottentotta gentili*	GBCH6939-13	JF820093
Scorpiones	Buthidae	*Hottentotta gentili*	GBCH6940-13	JF820092
Scorpiones	Buthidae	*Hottentotta gentili*	GBCH6941-13	JF820091
Scorpiones	Buthidae	*Hottentotta gentili*	GBCH6942-13	JF820090
Scorpiones	Buthidae	*Hottentotta gentili*	GBCH6943-13	JF820089
Scorpiones	Buthidae	*Hottentotta gentili*	GBCH6944-13	JF820088
Scorpiones	Buthidae	*Hottentotta gentili*	GBCH6945-13	JF820087
Scorpiones	Buthidae	*Hottentotta gentili*	GBCH6948-13	JF820084
Scorpiones	Buthidae	*Hottentotta gentili*	GBCH6949-13	JF820083
Scorpiones	Buthidae	*Hottentotta gentili*	GBCH6950-13	JF820082
Scorpiones	Buthidae	*Hottentotta gentili*	GBCH6951-13	JF820081
Scorpiones	Buthidae	*Hottentotta gentili*	GBCH6952-13	JF820080
Scorpiones	Buthidae	*Hottentotta gentili*	GBCH6953-13	JF820079
Scorpiones	Buthidae	*Hottentotta gentili*	GBCH6955-13	JF820077
Scorpiones	Buthidae	*Hottentotta gentili*	GBCH6956-13	JF820076
Scorpiones	Buthidae	*Hottentotta gentili*	GBCH6957-13	JF820075
Scorpiones	Buthidae	*Hottentotta gentili*	GBCH7633-13	JQ514245
Scorpiones	Buthidae	*Lychas mucronatus*	GBCH7773-13	JN018153
Scorpiones	Buthidae	*Mesobuthus gibbosus*	GBCH1688-06	NC_006515
Scorpiones	Buthidae	*Mesobuthus gibbosus*	GBCH2211-07	DQ310851
Scorpiones	Buthidae	*Mesobuthus gibbosus*	GBCH2212-07	DQ310850
Scorpiones	Buthidae	*Mesobuthus gibbosus*	GBCH3826-09	DQ310854
Scorpiones	Buthidae	*Mesobuthus martensii*	GBCH3166-08	NC_009738
Scorpiones	Buthidae	*Mesobuthus martensii*	GBCH3698-09	DQ340065
Scorpiones	Buthidae	*Mesobuthus martensii*	GBCH6915-13	JF700146
Scorpiones	Buthidae	*Mesobuthus martensii*	GBCH6916-13	JF700145
Scorpiones	Buthidae	*Orthochirus innesi*	GBCH7634-13	JQ514244
Scorpiones	Buthidae	*Tityus magnimanus*	GBCH4092-09	FJ525427
Scorpiones	Buthidae	*Tityus magnimanus*	GBCH4093-09	FJ525426

Scorpiones	Buthidae	*Tityus magnimanus*	GBCH4094-09	FJ525425
Scorpiones	Buthidae	*Tityus magnimanus*	GBCH4095-09	FJ525424
Scorpiones	Buthidae	*Tityus serrulatus*	GBCH7771-13	JN018155
Scorpiones	Buthidae	*Zabius fuscus*	GBCH4098-09	FJ525421
Scorpiones	Chactidae	*Belisarius xambeui*	GBCH7770-13	JN018156
Scorpiones	Chactidae	*Chactas*	GBCH7624-13	JQ514255
Scorpiones	Chactidae	*Uroctonus mordax*	GBCH2388-08	EU523756
Scorpiones	Chactidae	*Uroctonus mordax*	GBCH3206-08	NC_010782
Scorpiones	Euscorpiidae	*Euscorpius flavicaudis*	AMSCO002-10	HM418268
Scorpiones	Euscorpiidae	*Euscorpius flavicaudis*	GBCH1036-06	AY731175
Scorpiones	Euscorpiidae	*Euscorpius flavicaudis*	GBCH7743-13	JN018212
Scorpiones	Hemiscorpiidae	*Liocheles*	RBCH026-04	DQ127506
Scorpiones	Scorpionidae	*Heterometrus fulvipes*	GBCH0449-06	AY156572
Scorpiones	Scorpionidae	*Heterometrus laoticus*	GBCH0450-06	AY156573
Scorpiones	Scorpionidae	*Heterometrus spinifer*	GBCH0451-06	AY156574
Scorpiones	Scorpionidae	*Heterometrus swammerdami*	GBCH0452-06	AY156575
Scorpiones	Scorpionidae	*Nebo hierichonticus*	GBCH0448-06	AY156571
Scorpiones	Scorpionidae	*Opistophthalmus boehmi*	GBCH0453-06	AY156576
Scorpiones	Scorpionidae	*Opistophthalmus boehmi*	GBCH7630-13	JQ514248
Scorpiones	Scorpionidae	*Opistophthalmus capensis*	GBCH0454-06	AY156577
Scorpiones	Scorpionidae	*Opistophthalmus carinatus*	GBCH0455-06	AY156578
Scorpiones	Scorpionidae	*Opistophthalmus holmi*	GBCH0456-06	AY156579
Scorpiones	Scorpionidae	*Pandinus cavimanus*	GBCH0457-06	AY156580
Scorpiones	Scorpionidae	*Pandinus dictator*	GBCH0458-06	AY156581
Scorpiones	Scorpionidae	*Pandinus imperator*	GBCH0459-06	AY156582
Scorpiones	Scorpionidae	*Pandinus imperator*	GBCH7627-13	JQ514251
Scorpiones	Scorpionidae	*Pandinus viatoris*	GBCH0460-06	AY156583
Scorpiones	Scorpionidae	*Scorpio fuliginosus*	GBCH7620-13	JQ514259
Scorpiones	Scorpionidae	*Scorpio maurus*	GBCH0461-06	AY156584
Scorpiones	Scorpionidae	*Scorpio maurus*	GBCH0462-06	AY156585
Scorpiones	Scorpionidae	*Scorpio maurus*	GBCH3410-09	FJ198066
Scorpiones	Scorpionidae	*Scorpio maurus*	GBCH3411-09	FJ198065
Scorpiones	Scorpionidae	*Scorpio maurus*	GBCH3412-09	FJ198064
Scorpiones	Scorpionidae	*Scorpio maurus*	GBCH3413-09	FJ198063
Scorpiones	Scorpionidae	*Scorpio maurus*	GBCH3414-09	FJ198062
Scorpiones	Scorpionidae	*Scorpio maurus*	GBCH3415-09	FJ198061
Scorpiones	Scorpionidae	*Scorpio maurus*	GBCH3416-09	FJ198060
Scorpiones	Scorpionidae	*Scorpio maurus*	GBCH3417-09	FJ198059
Scorpiones	Scorpionidae	*Scorpio maurus*	GBCH3418-09	FJ198058
Scorpiones	Scorpionidae	*Scorpio maurus*	GBCH3419-09	FJ198057
Scorpiones	Vaejovidae	*Paruroctonus boreus*	GBCH11290-13	JX909544
Scorpiones	Vaejovidae	*Vaejovis*	GBCH11229-13	JX909605
Scorpiones	Vaejovidae	*Vaejovis*	GBCH11230-13	JX909604
Scorpiones	Vaejovidae	*Vaejovis bandido*	GBCH11248-13	JX909586
Scorpiones	Vaejovidae	*Vaejovis cashi*	GBCH11245-13	JX909589
Scorpiones	Vaejovidae	*Vaejovis cashi*	GBCH11246-13	JX909588
Scorpiones	Vaejovidae	*Vaejovis cashi*	GBCH11247-13	JX909587
Scorpiones	Vaejovidae	*Vaejovis crumpi*	GBCH11243-13	JX909591
Scorpiones	Vaejovidae	*Vaejovis crumpi*	GBCH11244-13	JX909590
Scorpiones	Vaejovidae	*Vaejovis deboerae*	GBCH11242-13	JX909592
Scorpiones	Vaejovidae	*Vaejovis electrum*	GBCH11239-13	JX909595
Scorpiones	Vaejovidae	*Vaejovis electrum*	GBCH11240-13	JX909594
Scorpiones	Vaejovidae	*Vaejovis electrum*	GBCH11241-13	JX909593

Scorpiones	Vaejovidae	*Vaejovis feti*	GBCH11238-13	JX909596
Scorpiones	Vaejovidae	*Vaejovis franckei*	GBCH11237-13	JX909597
Scorpiones	Vaejovidae	*Vaejovis granulatus*	GBCH11236-13	JX909598
Scorpiones	Vaejovidae	*Vaejovis jonesi*	GBCH11235-13	JX909599
Scorpiones	Vaejovidae	*Vaejovis lapidicola*	GBCH11234-13	JX909600
Scorpiones	Vaejovidae	*Vaejovis mexicanus*	GBCH11225-13	JX909609
Scorpiones	Vaejovidae	*Vaejovis mexicanus*	GBCH11226-13	JX909608
Scorpiones	Vaejovidae	*Vaejovis mexicanus*	GBCH11227-13	JX909607
Scorpiones	Vaejovidae	*Vaejovis montanus*	GBCH11231-13	JX909603
Scorpiones	Vaejovidae	*Vaejovis montanus*	GBCH11232-13	JX909602
Scorpiones	Vaejovidae	*Vaejovis montanus*	GBCH11233-13	JX909601
Scorpiones	Vaejovidae	*Vaejovis paysonensis*	GBCH11228-13	JX909606
Scorpiones	Vaejovidae	*Vaejovis tenuipalpus*	GBCH11224-13	JX909610
Scorpiones	Vaejovidae	*Vaejovis tenuipalpus*	GBCH11271-13	JX909563
Scorpiones	Vaejovidae	*Vaejovis vorhiesi*	GBCH11218-13	JX909616
Scorpiones	Vaejovidae	*Vaejovis vorhiesi*	GBCH11219-13	JX909615
Scorpiones	Vaejovidae	*Vaejovis vorhiesi*	GBCH11220-13	JX909614
Scorpiones	Vaejovidae	*Vaejovis vorhiesi*	GBCH11221-13	JX909613
Scorpiones	Vaejovidae	*Vaejovis vorhiesi*	GBCH11222-13	JX909612
Scorpiones	Vaejovidae	*Vaejovis vorhiesi*	GBCH11223-13	JX909611
Scorpiones	Vaejovidae	*Vaejovis vorhiesi*	GBCH11249-13	JX909585
Scorpiones	Vaejovidae	*Vaejovis vorhiesi*	GBCH11250-13	JX909584
Scorpiones	Vaejovidae	*Vaejovis vorhiesi*	GBCH11251-13	JX909583
Scorpiones	Vaejovidae	*Vaejovis vorhiesi*	GBCH11252-13	JX909582
Scorpiones	Vaejovidae	*Vaejovis vorhiesi*	GBCH11253-13	JX909581
Scorpiones	Vaejovidae	*Vaejovis vorhiesi*	GBCH11254-13	JX909580
Scorpiones	Vaejovidae	*Vaejovis vorhiesi*	GBCH11255-13	JX909579
Scorpiones	Vaejovidae	*Vaejovis vorhiesi*	GBCH11256-13	JX909578
Scorpiones	Vaejovidae	*Vaejovis vorhiesi*	GBCH11257-13	JX909577
Scorpiones	Vaejovidae	*Vaejovis vorhiesi*	GBCH11258-13	JX909576
Scorpiones	Vaejovidae	*Vaejovis vorhiesi*	GBCH11259-13	JX909575
Scorpiones	Vaejovidae	*Vaejovis vorhiesi*	GBCH11260-13	JX909574
Scorpiones	Vaejovidae	*Vaejovis vorhiesi*	GBCH11261-13	JX909573
Scorpiones	Vaejovidae	*Vaejovis vorhiesi*	GBCH11262-13	JX909572
Scorpiones	Vaejovidae	*Vaejovis vorhiesi*	GBCH11263-13	JX909571
Scorpiones	Vaejovidae	*Vaejovis vorhiesi*	GBCH11264-13	JX909570
Scorpiones	Vaejovidae	*Vaejovis vorhiesi*	GBCH11265-13	JX909569
Scorpiones	Vaejovidae	*Vaejovis vorhiesi*	GBCH11266-13	JX909568
Scorpiones	Vaejovidae	*Vaejovis vorhiesi*	GBCH11267-13	JX909567
Scorpiones	Vaejovidae	*Vaejovis vorhiesi*	GBCH11268-13	JX909566
Scorpiones	Vaejovidae	*Vaejovis vorhiesi*	GBCH11269-13	JX909565
Scorpiones	Vaejovidae	*Vaejovis vorhiesi*	GBCH11270-13	JX909564
Scorpiones	Vaejovidae	*Vaejovis vorhiesi*	GBCH11272-13	JX909562
Scorpiones	Vaejovidae	*Vaejovis vorhiesi*	GBCH11273-13	JX909561
Scorpiones	Vaejovidae	*Vaejovis vorhiesi*	GBCH11274-13	JX909560
Scorpiones	Vaejovidae	*Vaejovis vorhiesi*	GBCH11275-13	JX909559
Scorpiones	Vaejovidae	*Vaejovis vorhiesi*	GBCH11276-13	JX909558
Scorpiones	Vaejovidae	*Vaejovis vorhiesi*	GBCH11277-13	JX909557
Scorpiones	Vaejovidae	*Vaejovis vorhiesi*	GBCH11278-13	JX909556
Scorpiones	Vaejovidae	*Vaejovis vorhiesi*	GBCH11279-13	JX909555
Scorpiones	Vaejovidae	*Vaejovis vorhiesi*	GBCH11280-13	JX909554
Scorpiones	Vaejovidae	*Vaejovis vorhiesi*	GBCH11282-13	JX909552
Scorpiones	Vaejovidae	*Vaejovis vorhiesi*	GBCH11283-13	JX909551

Scorpiones	Vaejovidae	*Vaejovis vorhiesi*	GBCH11284-13	JX909550
Scorpiones	Vaejovidae	*Vaejovis vorhiesi*	GBCH11285-13	JX909549
Scorpiones	Vaejovidae	*Vaejovis vorhiesi*	GBCH11286-13	JX909548
Scorpiones	Vaejovidae	*Vaejovis vorhiesi*	GBCH11287-13	JX909547
Scorpiones	Vaejovidae	*Vaejovis vorhiesi*	GBCH11288-13	JX909546
Scorpiones	Vaejovidae	*Vaejovis vorhiesi*	GBCH11289-13	JX909545
Solifugae	Ammotrechidae	*Nothopuga sp. 1 LP-2008*	GBCH2403-08	NC_009984
Solifugae	Ammotrechidae	*Nothopuga sp. 1 LP-2008*	GBCH2404-08	EU024482
Solifugae	Eremobatidae	*Eremobates cf. palpisetulosus SEM-2008*	GBCH2391-08	EU520642
Solifugae	Eremobatidae	*Eremobates cf. palpisetulosus SEM-2008*	GBCH3209-08	NC_010779
Solifugae	Galeodidae		GBCH7760-13	JN018166
Solifugae	Rhagodidae		GBCH7758-13	JN018168
Solifugae	Rhagodidae		GBCH7759-13	JN018167
Trombidiformes	Acalyptonotidae	*Paenecalyptonotus*	MYMCB485-11	JX837029
Trombidiformes	Acalyptonotidae	*Paenecalyptonotus*	MYMCB670-11	JX837830
Trombidiformes	Anystidae		ARCN010-10	KM825187
Trombidiformes	Anystidae		ARCN012-10	HQ924258
Trombidiformes	Anystidae		ARCN023-10	HQ924267
Trombidiformes	Anystidae		ARCN028-10	HQ924271
Trombidiformes	Anystidae		ARCN029-10	HQ924272
Trombidiformes	Anystidae		ARCN030-10	HQ924273
Trombidiformes	Anystidae		ARCN031-10	HQ924274
Trombidiformes	Anystidae		ARCN032-10	HQ924275
Trombidiformes	Anystidae		ARCN033-10	HQ924276
Trombidiformes	Anystidae		ARCN044-10	HQ924280
Trombidiformes	Anystidae		ARCN093-10	HQ924313
Trombidiformes	Anystidae		ARCN095-10	HQ924314
Trombidiformes	Anystidae		ARCN144-10	HQ924351
Trombidiformes	Anystidae		ARCN165-10	HQ924367
Trombidiformes	Anystidae		ASAMT005-12	KP979209
Trombidiformes	Anystidae		ASAMT007-12	KP979234
Trombidiformes	Anystidae		ASAMT009-12	KP979153
Trombidiformes	Anystidae		ASAMT029-12	KP979296
Trombidiformes	Anystidae		ASAMT031-12	KR070541
Trombidiformes	Anystidae		CHACA1048-10	HM907100
Trombidiformes	Anystidae		CHACA1099-10	JX833883
Trombidiformes	Anystidae		CHACA1101-10	JX837579
Trombidiformes	Anystidae		CHACA1102-10	JX838147
Trombidiformes	Anystidae		CHACA1103-10	JX837256
Trombidiformes	Anystidae		CHACA332-08	JX836134
Trombidiformes	Anystidae		CHACA552-09	JX836355
Trombidiformes	Anystidae		CHACA553-09	JX835531
Trombidiformes	Anystidae		CHACA559-09	JX836511
Trombidiformes	Anystidae		CHACB1132-10	HM907222
Trombidiformes	Anystidae		CHACB115-10	HQ558406
Trombidiformes	Anystidae		CHACB1160-10	HM907244
Trombidiformes	Anystidae		CHACB271-10	HQ558509
Trombidiformes	Anystidae		CHACB317-10	HM907303
Trombidiformes	Anystidae		CHACB318-10	HM907304
Trombidiformes	Anystidae		CHACB319-10	HM907305
Trombidiformes	Anystidae		CHACB321-10	HM907306
Trombidiformes	Anystidae		CHACB384-10	HQ558557
Trombidiformes	Anystidae		CHACB385-10	HQ558558

Trombidiformes	Anystidae	CHACB443-10	JX834136
Trombidiformes	Anystidae	CHACB556-10	HQ558672
Trombidiformes	Anystidae	CHACB604-10	HQ558699
Trombidiformes	Anystidae	CHACB605-10	HQ558700
Trombidiformes	Anystidae	CHACB606-10	JX836849
Trombidiformes	Anystidae	CHACC021-10	HM907422
Trombidiformes	Anystidae	CNBAD670-12	KM825621
Trombidiformes	Anystidae	CNBAD679-12	KM833418
Trombidiformes	Anystidae	CNBAD682-12	KM839069
Trombidiformes	Anystidae	CNBAE288-12	KM827347
Trombidiformes	Anystidae	CNBAE289-12	KM832611
Trombidiformes	Anystidae	CNBAE291-12	KM830158
Trombidiformes	Anystidae	CNBAF206-12	KM830440
Trombidiformes	Anystidae	CNBAG055-12	KM838234
Trombidiformes	Anystidae	CNBPA196-12	KM827282
Trombidiformes	Anystidae	CNBPA203-12	KM827017
Trombidiformes	Anystidae	CNBPC213-12	KM839554
Trombidiformes	Anystidae	CNBPC216-12	KM825106
Trombidiformes	Anystidae	CNBPD582-12	KM826433
Trombidiformes	Anystidae	CNBPD589-12	KM839703
Trombidiformes	Anystidae	CNBPD592-12	KM838889
Trombidiformes	Anystidae	CNBPD594-12	KM837634
Trombidiformes	Anystidae	CNBPD598-12	KM836715
Trombidiformes	Anystidae	CNBPD605-12	KM824453
Trombidiformes	Anystidae	CNBPF131-12	KM831338
Trombidiformes	Anystidae	CNBPF132-12	KM824740
Trombidiformes	Anystidae	CNBPG402-12	KM830811
Trombidiformes	Anystidae	CNBPH405-13	KM825899
Trombidiformes	Anystidae	CNBPI447-12	KM836003
Trombidiformes	Anystidae	CNBPI448-12	KM833278
Trombidiformes	Anystidae	CNBPK336-13	KM829055
Trombidiformes	Anystidae	CNBPL271-13	KM835302
Trombidiformes	Anystidae	CNBPL272-13	KM829928
Trombidiformes	Anystidae	CNBPL273-13	KM834150
Trombidiformes	Anystidae	CNBPL300-13	KM838664
Trombidiformes	Anystidae	CNBPL302-13	KM831315
Trombidiformes	Anystidae	CNBPL303-13	KM832778
Trombidiformes	Anystidae	CNBPN237-13	KM838925
Trombidiformes	Anystidae	CNBPN239-13	KM838143
Trombidiformes	Anystidae	CNBPN241-13	KM827453
Trombidiformes	Anystidae	CNBPN245-13	KM830371
Trombidiformes	Anystidae	CNBPN263-13	KM825006
Trombidiformes	Anystidae	CNBPN269-13	KM830184
Trombidiformes	Anystidae	CNBPN274-13	KM836805
Trombidiformes	Anystidae	CNBPN276-13	KM824645
Trombidiformes	Anystidae	CNBPN278-13	KM824147
Trombidiformes	Anystidae	CNBPN289-13	KM838270
Trombidiformes	Anystidae	CNBPN294-13	KM830816
Trombidiformes	Anystidae	CNBPN295-13	KM836144
Trombidiformes	Anystidae	CNBPN296-13	KM833861
Trombidiformes	Anystidae	CNBPN308-13	KM840060
Trombidiformes	Anystidae	CNBPP099-13	KM838546
Trombidiformes	Anystidae	CNBPQ355-13	KM828681

Trombidiformes	Anystidae	CNBPQ359-13	KM831200
Trombidiformes	Anystidae	CNBPQ362-13	KM835068
Trombidiformes	Anystidae	CNBPR100-13	KM829038
Trombidiformes	Anystidae	CNBPR101-13	KM825581
Trombidiformes	Anystidae	CNBPR102-13	KM829199
Trombidiformes	Anystidae	CNBPR103-13	KM840529
Trombidiformes	Anystidae	CNBPR104-13	KM831594
Trombidiformes	Anystidae	CNEIA2620-12	KM834774
Trombidiformes	Anystidae	CNEIF2380-12	KM834829
Trombidiformes	Anystidae	CNEIF2385-12	KM830842
Trombidiformes	Anystidae	CNGIB575-12	KM836780
Trombidiformes	Anystidae	CNGIB597-12	KM828866
Trombidiformes	Anystidae	CNGLD030-13	KM830458
Trombidiformes	Anystidae	CNGLD031-13	KM825406
Trombidiformes	Anystidae	CNGLE043-13	KM824938
Trombidiformes	Anystidae	CNJAA870-12	KM835461
Trombidiformes	Anystidae	CNJAA873-12	KM838894
Trombidiformes	Anystidae	CNJAA874-12	KM826346
Trombidiformes	Anystidae	CNJAA876-12	KM839881
Trombidiformes	Anystidae	CNJAA877-12	KM833497
Trombidiformes	Anystidae	CNJAA878-12	KM837620
Trombidiformes	Anystidae	CNJAA880-12	KM830283
Trombidiformes	Anystidae	CNJAA881-12	KM838053
Trombidiformes	Anystidae	CNJAA882-12	KM830698
Trombidiformes	Anystidae	CNJAA884-12	KM835122
Trombidiformes	Anystidae	CNJAA885-12	KM838153
Trombidiformes	Anystidae	CNJAA889-12	KM824629
Trombidiformes	Anystidae	CNJAA890-12	KM837564
Trombidiformes	Anystidae	CNJAA892-12	KM824199
Trombidiformes	Anystidae	CNJAA893-12	KM827951
Trombidiformes	Anystidae	CNJAA895-12	KM839462
Trombidiformes	Anystidae	CNJAA896-12	KM830801
Trombidiformes	Anystidae	CNJAA897-12	KM835033
Trombidiformes	Anystidae	CNJAA898-12	KM826083
Trombidiformes	Anystidae	CNJAA899-12	KM829439
Trombidiformes	Anystidae	CNJAA901-12	KM827847
Trombidiformes	Anystidae	CNJAA902-12	KM825032
Trombidiformes	Anystidae	CNJAA904-12	KM838344
Trombidiformes	Anystidae	CNJAA905-12	KM835491
Trombidiformes	Anystidae	CNJAA907-12	KM824158
Trombidiformes	Anystidae	CNJAA908-12	KM829045
Trombidiformes	Anystidae	CNJAA909-12	KM838278
Trombidiformes	Anystidae	CNJAA911-12	KM826601
Trombidiformes	Anystidae	CNJAB1000-12	KM828487
Trombidiformes	Anystidae	CNJAB1001-12	KM835506
Trombidiformes	Anystidae	CNJAB1002-12	KM833704
Trombidiformes	Anystidae	CNJAB1004-12	KM833737
Trombidiformes	Anystidae	CNJAB1009-12	KM833196
Trombidiformes	Anystidae	CNJAB1011-12	KM830484
Trombidiformes	Anystidae	CNJAB1018-12	KM827180
Trombidiformes	Anystidae	CNJAB1023-12	KM829856
Trombidiformes	Anystidae	CNJAB1033-12	KM832031
Trombidiformes	Anystidae	CNJAB1035-12	KM826634

Trombidiformes	Anystidae	CNJAB996-12	KM837012
Trombidiformes	Anystidae	CNJAB997-12	KM833950
Trombidiformes	Anystidae	CNJAC1099-12	KM834737
Trombidiformes	Anystidae	CNJAC1109-12	KM837840
Trombidiformes	Anystidae	CNJAC1111-12	KM831325
Trombidiformes	Anystidae	CNJAC1123-12	KM830338
Trombidiformes	Anystidae	CNJAC1434-12	KM836836
Trombidiformes	Anystidae	CNJAC1435-12	KM828698
Trombidiformes	Anystidae	CNJAC1490-12	KM840844
Trombidiformes	Anystidae	CNJAC1495-12	KM834533
Trombidiformes	Anystidae	CNJAC1497-12	KM837763
Trombidiformes	Anystidae	CNJAC1498-12	KM828263
Trombidiformes	Anystidae	CNJAC1500-12	KM838349
Trombidiformes	Anystidae	CNJAC1509-12	KM830149
Trombidiformes	Anystidae	CNJAC1510-12	KM835961
Trombidiformes	Anystidae	CNJAC1519-12	KM838672
Trombidiformes	Anystidae	CNJAC1520-12	KM832397
Trombidiformes	Anystidae	CNJAC1521-12	KM839369
Trombidiformes	Anystidae	CNJAC1544-12	KM829260
Trombidiformes	Anystidae	CNJAC1571-12	KM827686
Trombidiformes	Anystidae	CNJAC1575-12	KM836363
Trombidiformes	Anystidae	CNJAC1581-12	KM825387
Trombidiformes	Anystidae	CNJAC1608-12	KM836561
Trombidiformes	Anystidae	CNJAD1795-12	KM825188
Trombidiformes	Anystidae	CNJAD1797-12	KM832286
Trombidiformes	Anystidae	CNJAD1798-12	KM840361
Trombidiformes	Anystidae	CNJAD1800-12	KM829318
Trombidiformes	Anystidae	CNJAD1801-12	KM835955
Trombidiformes	Anystidae	CNJAD1802-12	KM825764
Trombidiformes	Anystidae	CNJAD1803-12	KM831772
Trombidiformes	Anystidae	CNJAD1804-12	KM838391
Trombidiformes	Anystidae	CNJAD1805-12	KM826701
Trombidiformes	Anystidae	CNJAD1806-12	KM825646
Trombidiformes	Anystidae	CNJAD1807-12	KM836313
Trombidiformes	Anystidae	CNJAD1808-12	KM828215
Trombidiformes	Anystidae	CNJAD1809-12	KM829630
Trombidiformes	Anystidae	CNJAD1810-12	KM836449
Trombidiformes	Anystidae	CNJAD1811-12	KM839918
Trombidiformes	Anystidae	CNJAD1812-12	KM829451
Trombidiformes	Anystidae	CNJAD1813-12	KM838212
Trombidiformes	Anystidae	CNJAD1814-12	KM839500
Trombidiformes	Anystidae	CNJAD1816-12	KM837814
Trombidiformes	Anystidae	CNJAD1818-12	KM824593
Trombidiformes	Anystidae	CNJAD1819-12	KM839904
Trombidiformes	Anystidae	CNJAD1824-12	KM825957
Trombidiformes	Anystidae	CNJAD1826-12	KM832766
Trombidiformes	Anystidae	CNJAD1827-12	KM829551
Trombidiformes	Anystidae	CNJAD1829-12	KM834850
Trombidiformes	Anystidae	CNJAD1834-12	KM832803
Trombidiformes	Anystidae	CNJAD1835-12	KM839456
Trombidiformes	Anystidae	CNJAD1836-12	KM830567
Trombidiformes	Anystidae	CNJAD1838-12	KM840251
Trombidiformes	Anystidae	CNJAD1844-12	KM835733

Trombidiformes	Anystidae	CNJAD1845-12	KM839831
Trombidiformes	Anystidae	CNJAD1847-12	KM840203
Trombidiformes	Anystidae	CNJAD1848-12	KM834752
Trombidiformes	Anystidae	CNJAD1849-12	KM834102
Trombidiformes	Anystidae	CNJAD1851-12	KM834896
Trombidiformes	Anystidae	CNJAD1852-12	KM837003
Trombidiformes	Anystidae	CNJAD1854-12	KM827430
Trombidiformes	Anystidae	CNJAD1856-12	KM840123
Trombidiformes	Anystidae	CNJAD1858-12	KM834423
Trombidiformes	Anystidae	CNJAD2258-12	KM824668
Trombidiformes	Anystidae	CNJAD2274-12	KM825178
Trombidiformes	Anystidae	CNJAD2275-12	KM830417
Trombidiformes	Anystidae	CNJAD2276-12	KM835101
Trombidiformes	Anystidae	CNJAD2277-12	KM831454
Trombidiformes	Anystidae	CNJAD2283-12	KM826262
Trombidiformes	Anystidae	CNJAD2293-12	KM836742
Trombidiformes	Anystidae	CNJAD2296-12	KM834498
Trombidiformes	Anystidae	CNJAD2299-12	KM827809
Trombidiformes	Anystidae	CNJAD2300-12	KM825574
Trombidiformes	Anystidae	CNJAD2303-12	KM839983
Trombidiformes	Anystidae	CNJAD2304-12	KM834477
Trombidiformes	Anystidae	CNJAD2306-12	KM830095
Trombidiformes	Anystidae	CNJAD2307-12	KM827135
Trombidiformes	Anystidae	CNJAD2308-12	KM832872
Trombidiformes	Anystidae	CNJAD2310-12	KM831561
Trombidiformes	Anystidae	CNJAD2320-12	KM830926
Trombidiformes	Anystidae	CNJAD2322-12	KM835254
Trombidiformes	Anystidae	CNJAD2324-12	KM837891
Trombidiformes	Anystidae	CNJAD2326-12	KM837150
Trombidiformes	Anystidae	CNJAD2327-12	KM829544
Trombidiformes	Anystidae	CNJAD2330-12	KM824264
Trombidiformes	Anystidae	CNJAD2331-12	KM824804
Trombidiformes	Anystidae	CNJAD2334-12	KM825782
Trombidiformes	Anystidae	CNJAD2335-12	KM830180
Trombidiformes	Anystidae	CNJAD2344-12	KM828460
Trombidiformes	Anystidae	CNJAD2345-12	KM834030
Trombidiformes	Anystidae	CNJAD2348-12	KM832020
Trombidiformes	Anystidae	CNJAD2351-12	KM833695
Trombidiformes	Anystidae	CNJAD2353-12	KM835353
Trombidiformes	Anystidae	CNJAD2354-12	KM829652
Trombidiformes	Anystidae	CNJAD2355-12	KM835349
Trombidiformes	Anystidae	CNJAD2356-12	KM830703
Trombidiformes	Anystidae	CNJAD2357-12	KM830216
Trombidiformes	Anystidae	CNJAD2358-12	KM831218
Trombidiformes	Anystidae	CNJAD2360-12	KM833667
Trombidiformes	Anystidae	CNJAD2361-12	KM838316
Trombidiformes	Anystidae	CNJAD2362-12	KM836611
Trombidiformes	Anystidae	CNJAD2363-12	KM825362
Trombidiformes	Anystidae	CNJAD2364-12	KM828100
Trombidiformes	Anystidae	CNJAD2365-12	KM840217
Trombidiformes	Anystidae	CNJAE1119-12	KM828257
Trombidiformes	Anystidae	CNJAE1128-12	KM838366
Trombidiformes	Anystidae	CNJAE1129-12	KM833070

Trombidiformes	Anystidae	CNJAE1134-12	KM837249
Trombidiformes	Anystidae	CNJAE1135-12	KM839281
Trombidiformes	Anystidae	CNJAE1136-12	KM832473
Trombidiformes	Anystidae	CNJAE1140-12	KM837284
Trombidiformes	Anystidae	CNJAE1141-12	KM840616
Trombidiformes	Anystidae	CNJAE1146-12	KM826763
Trombidiformes	Anystidae	CNJAE1147-12	KM827903
Trombidiformes	Anystidae	CNJAE1151-12	KM827080
Trombidiformes	Anystidae	CNJAE1152-12	KM824691
Trombidiformes	Anystidae	CNJAF1906-12	KM828824
Trombidiformes	Anystidae	CNJAF1916-12	KM828378
Trombidiformes	Anystidae	CNJAF1924-12	KM827321
Trombidiformes	Anystidae	CNJAF1937-12	KM826692
Trombidiformes	Anystidae	CNJAF1999-12	KM829224
Trombidiformes	Anystidae	CNJAF2022-12	KM836954
Trombidiformes	Anystidae	CNJAG1828-12	KM827269
Trombidiformes	Anystidae	CNJAG1842-12	KM838517
Trombidiformes	Anystidae	CNJAH788-12	KM830445
Trombidiformes	Anystidae	CNJAI861-12	KM825427
Trombidiformes	Anystidae	CNJAJ697-12	KM825685
Trombidiformes	Anystidae	CNPAA457-13	KM837527
Trombidiformes	Anystidae	CNPAD557-13	KM839809
Trombidiformes	Anystidae	CNPAD559-13	KM829759
Trombidiformes	Anystidae	CNPAD561-13	KM830525
Trombidiformes	Anystidae	CNPAD563-13	KM832786
Trombidiformes	Anystidae	CNPAD838-13	KM824819
Trombidiformes	Anystidae	CNPAD859-13	KM839515
Trombidiformes	Anystidae	CNPAD860-13	KM837718
Trombidiformes	Anystidae	CNPAD861-13	KM833184
Trombidiformes	Anystidae	CNPAD862-13	KM825917
Trombidiformes	Anystidae	CNPAD863-13	KM836493
Trombidiformes	Anystidae	CNPAD864-13	KM830081
Trombidiformes	Anystidae	CNPAD865-13	KM830197
Trombidiformes	Anystidae	CNPAD867-13	KM829141
Trombidiformes	Anystidae	CNPAD868-13	KM834632
Trombidiformes	Anystidae	CNPAD869-13	KM825629
Trombidiformes	Anystidae	CNPAE463-13	KM830162
Trombidiformes	Anystidae	CNPAE464-13	KM828300
Trombidiformes	Anystidae	CNPAF924-13	KM829990
Trombidiformes	Anystidae	CNPAI467-13	KM826907
Trombidiformes	Anystidae	CNRMC1518-12	KM826398
Trombidiformes	Anystidae	CNRME4721-12	KM833716
Trombidiformes	Anystidae	CNRME4735-12	KM830836
Trombidiformes	Anystidae	CNRME4746-12	KM828095
Trombidiformes	Anystidae	CNRME4748-12	KM829009
Trombidiformes	Anystidae	CNRME4751-12	KM837594
Trombidiformes	Anystidae	CNRME4759-12	KM829248
Trombidiformes	Anystidae	CNRME4778-12	KM838094
Trombidiformes	Anystidae	CNRME4783-12	KM830034
Trombidiformes	Anystidae	CNRME4791-12	KM826581
Trombidiformes	Anystidae	CNRME4795-12	KM838374
Trombidiformes	Anystidae	CNRMF3185-12	KM824600
Trombidiformes	Anystidae	CNRMF3190-12	KM831028

Trombidiformes	Anystidae	CNRMF3193-12	KM826344
Trombidiformes	Anystidae	CNRMF3195-12	KM830624
Trombidiformes	Anystidae	CNRMF3197-12	KM835653
Trombidiformes	Anystidae	CNRMF3202-12	KM825534
Trombidiformes	Anystidae	CNRMF3204-12	KM825622
Trombidiformes	Anystidae	CNRMF3208-12	KM825475
Trombidiformes	Anystidae	CNRMF3210-12	KM824971
Trombidiformes	Anystidae	CNRMF3220-12	KM834285
Trombidiformes	Anystidae	CNRMF3223-12	KM835845
Trombidiformes	Anystidae	CNRMF3227-12	KM837326
Trombidiformes	Anystidae	CNRMF3229-12	KM834265
Trombidiformes	Anystidae	CNRMF3257-12	KM833966
Trombidiformes	Anystidae	CNRMF3268-12	KM825520
Trombidiformes	Anystidae	CNRMG690-12	KM826123
Trombidiformes	Anystidae	CNSLB474-12	KM827768
Trombidiformes	Anystidae	CNSLB481-12	KM828605
Trombidiformes	Anystidae	CNSLC548-12	KM831169
Trombidiformes	Anystidae	CNSLC551-12	KM836360
Trombidiformes	Anystidae	CNSLC552-12	KM832203
Trombidiformes	Anystidae	CNSLC555-12	KM831119
Trombidiformes	Anystidae	CNSLC562-12	KM834881
Trombidiformes	Anystidae	CNSLD669-12	KM831513
Trombidiformes	Anystidae	CNSLD673-12	KM831392
Trombidiformes	Anystidae	CNSLD683-12	KM834169
Trombidiformes	Anystidae	CNSLE491-12	KM828195
Trombidiformes	Anystidae	CNSLE494-12	KM839523
Trombidiformes	Anystidae	CNSLE497-12	KM840154
Trombidiformes	Anystidae	CNSLE498-12	KM838955
Trombidiformes	Anystidae	CNSLE499-12	KM826404
Trombidiformes	Anystidae	CNSLE500-12	KM832282
Trombidiformes	Anystidae	CNSLE503-12	KM826283
Trombidiformes	Anystidae	CNSLE504-12	KM833029
Trombidiformes	Anystidae	CNSLE505-12	KM836167
Trombidiformes	Anystidae	CNSLE509-12	KM831345
Trombidiformes	Anystidae	CNSLF657-12	KM829964
Trombidiformes	Anystidae	CNSLH070-12	KM838562
Trombidiformes	Anystidae	CNSLH071-12	KM838709
Trombidiformes	Anystidae	CNSLH073-12	KM831495
Trombidiformes	Anystidae	CNSLH074-12	KM829222
Trombidiformes	Anystidae	CNSLH075-12	KM830737
Trombidiformes	Anystidae	CNSLH076-12	KM833623
Trombidiformes	Anystidae	CNSLH079-12	KM838975
Trombidiformes	Anystidae	CNSLI045-12	KM838339
Trombidiformes	Anystidae	CNSLI046-12	KM827261
Trombidiformes	Anystidae	CNSLI047-12	KM832209
Trombidiformes	Anystidae	CNSLI052-12	KM834274
Trombidiformes	Anystidae	CNSLI055-12	KM838458
Trombidiformes	Anystidae	CNSLI061-12	KM838110
Trombidiformes	Anystidae	CNSLI065-12	KM833215
Trombidiformes	Anystidae	CNSLI067-12	KM831485
Trombidiformes	Anystidae	CNSLJ024-12	KM829465
Trombidiformes	Anystidae	CNSLJ415-12	KM826717
Trombidiformes	Anystidae	CNSLJ416-12	KM824323

Trombidiformes	Anystidae	CNSLJ418-12	KM834006
Trombidiformes	Anystidae	CNSLJ419-12	KM832838
Trombidiformes	Anystidae	CNSLJ420-12	KM836839
Trombidiformes	Anystidae	CNSLJ421-12	KM832343
Trombidiformes	Anystidae	CNSLJ422-12	KM837096
Trombidiformes	Anystidae	CNSLJ423-12	KM832648
Trombidiformes	Anystidae	CNSLJ424-12	KM839112
Trombidiformes	Anystidae	CNSLJ425-12	KM838242
Trombidiformes	Anystidae	CNSLJ426-12	KM826058
Trombidiformes	Anystidae	CNSLJ429-12	KM836032
Trombidiformes	Anystidae	CNSLJ431-12	KM833903
Trombidiformes	Anystidae	CNSLJ432-12	KM826957
Trombidiformes	Anystidae	CNSLK068-12	KM829807
Trombidiformes	Anystidae	CNSLK071-12	KM829642
Trombidiformes	Anystidae	CNSLK072-12	KM836016
Trombidiformes	Anystidae	CNSLK073-12	KM840394
Trombidiformes	Anystidae	CNSLK074-12	KM828902
Trombidiformes	Anystidae	CNWBD028-13	KM825501
Trombidiformes	Anystidae	CNWLD1158-13	KM831874
Trombidiformes	Anystidae	CNWLF160-12	KM834361
Trombidiformes	Anystidae	CNWLG815-12	KM837417
Trombidiformes	Anystidae	CNWLG816-12	KM836320
Trombidiformes	Anystidae	CNWLG819-12	KM837308
Trombidiformes	Anystidae	CNWLG820-12	KM829180
Trombidiformes	Anystidae	CNWLG821-12	KM831728
Trombidiformes	Anystidae	CNWLG830-12	KM835872
Trombidiformes	Anystidae	CNWLG836-12	KM839316
Trombidiformes	Anystidae	CNWLG842-12	KM830628
Trombidiformes	Anystidae	CNWLG843-12	KM828626
Trombidiformes	Anystidae	CNWLG845-12	KM825674
Trombidiformes	Anystidae	CNWLG847-12	KM828516
Trombidiformes	Anystidae	CNWLG848-12	KM839030
Trombidiformes	Anystidae	CNWLG849-12	KM824969
Trombidiformes	Anystidae	CNWLG850-12	KM833630
Trombidiformes	Anystidae	CNWLG853-12	KM833963
Trombidiformes	Anystidae	CNWLG854-12	KM838729
Trombidiformes	Anystidae	CNWLG859-12	KM829524
Trombidiformes	Anystidae	CNWLG861-12	KM836797
Trombidiformes	Anystidae	CNWLH195-12	KM840758
Trombidiformes	Anystidae	CNWLH200-12	KM828406
Trombidiformes	Anystidae	CNWLH202-12	KM833663
Trombidiformes	Anystidae	CNWLH203-12	KM828045
Trombidiformes	Anystidae	CNWLH204-12	KM834321
Trombidiformes	Anystidae	CNWLH205-12	KM839216
Trombidiformes	Anystidae	CNWLH206-12	KM837226
Trombidiformes	Anystidae	CNWLH207-12	KM840235
Trombidiformes	Anystidae	CNWLH208-12	KM833240
Trombidiformes	Anystidae	CNWLH209-12	KM829941
Trombidiformes	Anystidae	CNWLH210-12	KM828393
Trombidiformes	Anystidae	CNWLH213-12	KM835217
Trombidiformes	Anystidae	CNWLH214-12	KM826501
Trombidiformes	Anystidae	CNWLH215-12	KM827794
Trombidiformes	Anystidae	CNWLH216-12	KM826038

Trombidiformes	Anystidae	CNWLH217-12	KM828090
Trombidiformes	Anystidae	CNWLH218-12	KM828415
Trombidiformes	Anystidae	CNWLH219-12	KM836748
Trombidiformes	Anystidae	CNWLH220-12	KM832392
Trombidiformes	Anystidae	CNWLH221-12	KM830520
Trombidiformes	Anystidae	CNWLH223-12	KM825722
Trombidiformes	Anystidae	CNWLH224-12	KM834971
Trombidiformes	Anystidae	CNWLH225-12	KM828937
Trombidiformes	Anystidae	CNWLH226-12	KM831230
Trombidiformes	Anystidae	CNWLH228-12	KM832853
Trombidiformes	Anystidae	CNWLH313-12	KM839708
Trombidiformes	Anystidae	CNWLH314-12	KM835445
Trombidiformes	Anystidae	CNWLH316-12	KM837162
Trombidiformes	Anystidae	CNWLH317-12	KM836118
Trombidiformes	Anystidae	CNWLH318-12	KM832817
Trombidiformes	Anystidae	CNWLH321-12	KM825819
Trombidiformes	Anystidae	CNWLH322-12	KM830316
Trombidiformes	Anystidae	CNWLH323-12	KM826416
Trombidiformes	Anystidae	CNWLH324-12	KM828702
Trombidiformes	Anystidae	CNWLH326-12	KM824444
Trombidiformes	Anystidae	CNWLH327-12	KM832372
Trombidiformes	Anystidae	CNWLH328-12	KM826240
Trombidiformes	Anystidae	CNWLH330-12	KM827095
Trombidiformes	Anystidae	CNWLH331-12	KM832655
Trombidiformes	Anystidae	CNWLH333-12	KM831394
Trombidiformes	Anystidae	CNWLH334-12	KM837900
Trombidiformes	Anystidae	CNWLH335-12	KM830562
Trombidiformes	Anystidae	CNWLH336-12	KM829341
Trombidiformes	Anystidae	CNWLH337-12	KM835132
Trombidiformes	Anystidae	CNWLH339-12	KM839457
Trombidiformes	Anystidae	CNWLH340-12	KM824814
Trombidiformes	Anystidae	CNWLH341-12	KM838655
Trombidiformes	Anystidae	CNWLI093-12	KM835301
Trombidiformes	Anystidae	CNWLI094-12	KM832187
Trombidiformes	Anystidae	CNWLI095-12	KM825560
Trombidiformes	Anystidae	CNWLI096-12	KM837163
Trombidiformes	Anystidae	CNWLI099-12	KM840144
Trombidiformes	Anystidae	CNWLI100-12	KM824187
Trombidiformes	Anystidae	CNWLI101-12	KM839442
Trombidiformes	Anystidae	CNWLI102-12	KM838757
Trombidiformes	Anystidae	CNWLI103-12	KM836273
Trombidiformes	Anystidae	CNWLI104-12	KM838685
Trombidiformes	Anystidae	CNWLI105-12	KM834600
Trombidiformes	Anystidae	CNWLI106-12	KM840265
Trombidiformes	Anystidae	CNWLI107-12	KM837852
Trombidiformes	Anystidae	CNWLI108-12	KM830077
Trombidiformes	Anystidae	CNWLI109-12	KM825577
Trombidiformes	Anystidae	CNWLJ025-12	KM839054
Trombidiformes	Anystidae	ERSCH054-07	KR069349
Trombidiformes	Anystidae	ERSCH056-07	KR070310
Trombidiformes	Anystidae	ERSCH059-07	KR070527
Trombidiformes	Anystidae	ERSCH060-07	KR070422
Trombidiformes	Anystidae	JSMAY1602-12	KP979352

Trombidiformes	Anystidae	JSOIE053-12	KR069279
Trombidiformes	Anystidae	JSOIE054-12	KP979289
Trombidiformes	Anystidae	JSOIE057-12	KR069967
Trombidiformes	Anystidae	JSOIE058-12	KR070546
Trombidiformes	Anystidae	JSOIE062-12	KR070535
Trombidiformes	Anystidae	JSOIE066-12	KR070197
Trombidiformes	Anystidae	JSOIE071-12	KR069520
Trombidiformes	Anystidae	JSOIE072-12	KR069355
Trombidiformes	Anystidae	JSOIE080-12	KR070516
Trombidiformes	Anystidae	JSOIE081-12	KR069665
Trombidiformes	Anystidae	JSOIE088-12	KR069200
Trombidiformes	Anystidae	JSOIE090-12	KR069725
Trombidiformes	Anystidae	JSOIE095-12	KR069881
Trombidiformes	Anystidae	JSOIE096-12	KR069162
Trombidiformes	Anystidae	JSOIE101-12	KR069185
Trombidiformes	Anystidae	JSOIE103-12	KR070495
Trombidiformes	Anystidae	JSOIE105-12	KR070679
Trombidiformes	Anystidae	JSOIE106-12	KR069166
Trombidiformes	Anystidae	JSOIE107-12	KR070633
Trombidiformes	Anystidae	JSOIE108-12	KR070741
Trombidiformes	Anystidae	JSOIE119-12	KR070743
Trombidiformes	Anystidae	JSOIE126-12	KR069863
Trombidiformes	Anystidae	MHMIT005-07	KR069498
Trombidiformes	Anystidae	MHMIT060-07	KR069367
Trombidiformes	Anystidae	MHMIT062-07	KR070045
Trombidiformes	Anystidae	MHMIT063-07	KR070273
Trombidiformes	Anystidae	MHMIT064-07	KR069673
Trombidiformes	Anystidae	MHMIT065-07	KR069716
Trombidiformes	Anystidae	MHMIT066-07	KR069775
Trombidiformes	Anystidae	MHMIT067-07	KR069980
Trombidiformes	Anystidae	MIAUS018-12	KP979128
Trombidiformes	Anystidae	MIAUS032-12	KP979338
Trombidiformes	Anystidae	MIAUS034-12	KR069503
Trombidiformes	Anystidae	MIAUS048-12	KR070464
Trombidiformes	Anystidae	MIONB145-10	KR070627
Trombidiformes	Anystidae	MIONB146-10	KP979182
Trombidiformes	Anystidae	MIONB150-10	KP979280
Trombidiformes	Anystidae	MIONB186-10	KP979142
Trombidiformes	Anystidae	MIONB187-10	KR069274
Trombidiformes	Anystidae	MIONB188-10	KR070612
Trombidiformes	Anystidae	MIONB189-10	KP979124
Trombidiformes	Anystidae	MIONB202-10	KR069609
Trombidiformes	Anystidae	MIONB238-10	KR070649
Trombidiformes	Anystidae	MIONB239-10	KR070440
Trombidiformes	Anystidae	MIONB240-10	KR069410
Trombidiformes	Anystidae	MIONB241-10	KP979147
Trombidiformes	Anystidae	MIONB242-10	KP979239
Trombidiformes	Anystidae	MIONB299-10	KR070069
Trombidiformes	Anystidae	MIONB300-10	KP979259
Trombidiformes	Anystidae	MIONB301-10	KP979196
Trombidiformes	Anystidae	MIONB302-10	KR070638
Trombidiformes	Anystidae	MIONB311-10	KR069195
Trombidiformes	Anystidae	MIONB312-10	KP979130

Trombidiformes	Anystidae	MIONB322-10	KR070233
Trombidiformes	Anystidae	MIONB325-10	KP979195
Trombidiformes	Anystidae	MIONB329-10	KP979157
Trombidiformes	Anystidae	MIONB330-10	KR069256
Trombidiformes	Anystidae	MIONB331-10	KP979170
Trombidiformes	Anystidae	MIONB332-10	KP979286
Trombidiformes	Anystidae	MIONB333-10	KR069637
Trombidiformes	Anystidae	MIONB335-10	KP979216
Trombidiformes	Anystidae	MIONB342-10	KR069384
Trombidiformes	Anystidae	MIONB343-10	KP979164
Trombidiformes	Anystidae	MIONB344-10	KP979356
Trombidiformes	Anystidae	MIONB345-10	KR070272
Trombidiformes	Anystidae	MIONB403-10	KP979235
Trombidiformes	Anystidae	MIONB416-10	KP979266
Trombidiformes	Anystidae	MIONB498-11	KM838961
Trombidiformes	Anystidae	MIONB510-11	KM825262
Trombidiformes	Anystidae	MYMCA837-11	JX837820
Trombidiformes	Anystidae	MYMCB019-11	JX834112
Trombidiformes	Anystidae	MYMCB892-11	JX835790
Trombidiformes	Anystidae	MYMCC132-11	JX834110
Trombidiformes	Anystidae	MYMCC205-11	JX836026
Trombidiformes	Anystidae	MYMCC396-11	JX835112
Trombidiformes	Anystidae	MYMCC397-11	JX838637
Trombidiformes	Anystidae	MYMCC660-11	JX834450
Trombidiformes	Anystidae	MYMCC854-11	JX835871
Trombidiformes	Anystidae	MYMCC896-11	JX834886
Trombidiformes	Anystidae	MYMCC948-11	JX836055
Trombidiformes	Anystidae	MYMCD100-11	JX838005
Trombidiformes	Anystidae	MYMCD101-11	JX838444
Trombidiformes	Anystidae	MYMCD170-11	JX838391
Trombidiformes	Anystidae	MYMCE144-12	JX834031
Trombidiformes	Anystidae	MYMCF463-12	JX834015
Trombidiformes	Anystidae	MYMCF585-12	JX834689
Trombidiformes	Anystidae	MYMCG297-12	JX835320
Trombidiformes	Anystidae	PHAUG1668-11	KP979271
Trombidiformes	Anystidae	PHAUG1678-11	KR070658
Trombidiformes	Anystidae	PHAUG1683-11	KR069855
Trombidiformes	Anystidae	PHJUN3806-11	KR070065
Trombidiformes	Anystidae	PHJUN3811-11	KR069453
Trombidiformes	Anystidae	PHSEP1995-11	KR070699
Trombidiformes	Anystidae	SSBAA2017-12	KM832572
Trombidiformes	Anystidae	SSBAA2018-12	KM827289
Trombidiformes	Anystidae	SSBAA2025-12	KM833985
Trombidiformes	Anystidae	SSBAA2026-12	KM828020
Trombidiformes	Anystidae	SSBAA2035-12	KM833682
Trombidiformes	Anystidae	SSBAA2038-12	KM824047
Trombidiformes	Anystidae	SSBAA2043-12	KM840171
Trombidiformes	Anystidae	SSBAA2048-12	KM836713
Trombidiformes	Anystidae	SSBAA2052-12	KM837757
Trombidiformes	Anystidae	SSBAA2054-12	KM829540
Trombidiformes	Anystidae	SSBAA2057-12	KM834440
Trombidiformes	Anystidae	SSBAA2060-12	KM833763
Trombidiformes	Anystidae	SSBAA2061-12	KM840052

Trombidiformes	Anystidae	SSBAA2087-12	KM840801
Trombidiformes	Anystidae	SSBAB093-12	KM830676
Trombidiformes	Anystidae	SSBAB094-12	KM836594
Trombidiformes	Anystidae	SSBAB095-12	KM830517
Trombidiformes	Anystidae	SSBAB096-12	KM830568
Trombidiformes	Anystidae	SSBAB097-12	KM831131
Trombidiformes	Anystidae	SSBAB101-12	KM837561
Trombidiformes	Anystidae	SSBAB102-12	KM838418
Trombidiformes	Anystidae	SSBAB103-12	KM829525
Trombidiformes	Anystidae	SSBAB105-12	KM831412
Trombidiformes	Anystidae	SSBAB106-12	KM830352
Trombidiformes	Anystidae	SSBAB109-12	KM834508
Trombidiformes	Anystidae	SSBAB112-12	KM831902
Trombidiformes	Anystidae	SSBAB1131-12	KM827636
Trombidiformes	Anystidae	SSBAB113-12	KM837420
Trombidiformes	Anystidae	SSBAB1133-12	KM834676
Trombidiformes	Anystidae	SSBAB1134-12	KM824606
Trombidiformes	Anystidae	SSBAB114-12	KM834825
Trombidiformes	Anystidae	SSBAB116-12	KM827226
Trombidiformes	Anystidae	SSBAB119-12	KM833526
Trombidiformes	Anystidae	SSBAB120-12	KM826932
Trombidiformes	Anystidae	SSBAB124-12	KM836184
Trombidiformes	Anystidae	SSBAB126-12	KM833033
Trombidiformes	Anystidae	SSBAB128-12	KM839335
Trombidiformes	Anystidae	SSBAB130-12	KM833824
Trombidiformes	Anystidae	SSBAB132-12	KM829693
Trombidiformes	Anystidae	SSBAB133-12	KM830979
Trombidiformes	Anystidae	SSBAB134-12	KM831827
Trombidiformes	Anystidae	SSBAB135-12	KM839294
Trombidiformes	Anystidae	SSBAB136-12	KM829073
Trombidiformes	Anystidae	SSBAB137-12	KM833911
Trombidiformes	Anystidae	SSBAB139-12	KM832133
Trombidiformes	Anystidae	SSBAB140-12	KM827150
Trombidiformes	Anystidae	SSBAB141-12	KM836357
Trombidiformes	Anystidae	SSBAB142-12	KM825079
Trombidiformes	Anystidae	SSBAB143-12	KM838538
Trombidiformes	Anystidae	SSBAB144-12	KM832823
Trombidiformes	Anystidae	SSBAB145-12	KM831245
Trombidiformes	Anystidae	SSBAB146-12	KM840443
Trombidiformes	Anystidae	SSBAB147-12	KM837714
Trombidiformes	Anystidae	SSBAB148-12	KM836841
Trombidiformes	Anystidae	SSBAB150-12	KM835244
Trombidiformes	Anystidae	SSBAB151-12	KM835757
Trombidiformes	Anystidae	SSBAB152-12	KM838638
Trombidiformes	Anystidae	SSBAB153-12	KM825775
Trombidiformes	Anystidae	SSBAB154-12	KM828434
Trombidiformes	Anystidae	SSBAB157-12	KM832681
Trombidiformes	Anystidae	SSBAB158-12	KM829448
Trombidiformes	Anystidae	SSBAB159-12	KM832296
Trombidiformes	Anystidae	SSBAB160-12	KM838284
Trombidiformes	Anystidae	SSBAB161-12	KM830013
Trombidiformes	Anystidae	SSBAC2356-12	KM830753
Trombidiformes	Anystidae	SSBAC2357-12	KM839271

Trombidiformes	Anystidae	SSBAC2359-12	KM837434
Trombidiformes	Anystidae	SSBAC2372-12	KM840108
Trombidiformes	Anystidae	SSBAC2373-12	KM840684
Trombidiformes	Anystidae	SSBAC2374-12	KM827743
Trombidiformes	Anystidae	SSBAC2378-12	KM833974
Trombidiformes	Anystidae	SSBAC2379-12	KM824769
Trombidiformes	Anystidae	SSBAC2385-12	KM828057
Trombidiformes	Anystidae	SSBAC2387-12	KM825995
Trombidiformes	Anystidae	SSBAC2389-12	KM830935
Trombidiformes	Anystidae	SSBAC2390-12	KM835498
Trombidiformes	Anystidae	SSBAC3301-12	KM832486
Trombidiformes	Anystidae	SSBAC3306-12	KM833970
Trombidiformes	Anystidae	SSBAC3309-12	KM827244
Trombidiformes	Anystidae	SSBAC3310-12	KM827491
Trombidiformes	Anystidae	SSBAC3311-12	KM838451
Trombidiformes	Anystidae	SSBAC3312-12	KM829604
Trombidiformes	Anystidae	SSBAC3313-12	KM827040
Trombidiformes	Anystidae	SSBAC3314-12	KM832039
Trombidiformes	Anystidae	SSBAC3316-12	KM826814
Trombidiformes	Anystidae	SSBAC3317-12	KM838280
Trombidiformes	Anystidae	SSBAC3327-12	KM830553
Trombidiformes	Anystidae	SSBAD3067-12	KM831726
Trombidiformes	Anystidae	SSBAD3068-12	KM825628
Trombidiformes	Anystidae	SSBAD3073-12	KM831651
Trombidiformes	Anystidae	SSBAD3097-12	KM830922
Trombidiformes	Anystidae	SSBAD3098-12	KM834341
Trombidiformes	Anystidae	SSBAD3108-12	KM838307
Trombidiformes	Anystidae	SSBAD3109-12	KM824697
Trombidiformes	Anystidae	SSBAD3119-12	KM827431
Trombidiformes	Anystidae	SSBAD3123-12	KM829025
Trombidiformes	Anystidae	SSBAD3124-12	KM832663
Trombidiformes	Anystidae	SSBAD3127-12	KM825720
Trombidiformes	Anystidae	SSBAD3130-12	KM830385
Trombidiformes	Anystidae	SSBAD3131-12	KM829925
Trombidiformes	Anystidae	SSBAD3132-12	KM824888
Trombidiformes	Anystidae	SSBAD3134-12	KM829609
Trombidiformes	Anystidae	SSBAD3140-12	KM833463
Trombidiformes	Anystidae	SSBAD3142-12	KM829664
Trombidiformes	Anystidae	SSBAD3143-12	KM836889
Trombidiformes	Anystidae	SSBAD3958-12	KM837884
Trombidiformes	Anystidae	SSBAD3962-12	KM834572
Trombidiformes	Anystidae	SSBAD3965-12	KM824699
Trombidiformes	Anystidae	SSBAD3967-12	KM828381
Trombidiformes	Anystidae	SSBAD3972-12	KM829936
Trombidiformes	Anystidae	SSBAD3973-12	KM824330
Trombidiformes	Anystidae	SSBAD3986-12	KM828543
Trombidiformes	Anystidae	SSBAD3989-12	KM840150
Trombidiformes	Anystidae	SSBAD3993-12	KM830897
Trombidiformes	Anystidae	SSBAD3994-12	KM831903
Trombidiformes	Anystidae	SSBAD4006-12	KM838057
Trombidiformes	Anystidae	SSBAD4017-12	KM832729
Trombidiformes	Anystidae	SSBAD4018-12	KM829198
Trombidiformes	Anystidae	SSBAD4025-12	KM833372

Trombidiformes	Anystidae	SSBAD4026-12	KM834407
Trombidiformes	Anystidae	SSBAD4027-12	KM835995
Trombidiformes	Anystidae	SSBAD4031-12	KM824305
Trombidiformes	Anystidae	SSBAD4692-13	KM832205
Trombidiformes	Anystidae	SSBAD4720-13	KM830936
Trombidiformes	Anystidae	SSBAD4738-13	KM831204
Trombidiformes	Anystidae	SSBAD4764-13	KM836828
Trombidiformes	Anystidae	SSBAD4776-13	KM835027
Trombidiformes	Anystidae	SSBAD5537-13	KM838087
Trombidiformes	Anystidae	SSBAD6322-13	KM828222
Trombidiformes	Anystidae	SSBAD6353-13	KM825270
Trombidiformes	Anystidae	SSBAE942-13	KM840245
Trombidiformes	Anystidae	SSBAE957-13	KM832033
Trombidiformes	Anystidae	SSBAF5229-13	KM828973
Trombidiformes	Anystidae	SSBAF5757-13	KM831223
Trombidiformes	Anystidae	SSEIB7634-13	KM825526
Trombidiformes	Anystidae	SSJAA1101-13	KM836556
Trombidiformes	Anystidae	SSJAA858-13	KM825391
Trombidiformes	Anystidae	SSJAB1004-13	KM837469
Trombidiformes	Anystidae	SSJAB1005-13	KM831647
Trombidiformes	Anystidae	SSJAB1012-13	KM830357
Trombidiformes	Anystidae	SSJAB1017-13	KM835140
Trombidiformes	Anystidae	SSJAB1021-13	KM834443
Trombidiformes	Anystidae	SSJAB1833-13	KM830899
Trombidiformes	Anystidae	SSJAB2180-13	KM840728
Trombidiformes	Anystidae	SSJAB3342-13	KM830649
Trombidiformes	Anystidae	SSJAB3364-13	KM839053
Trombidiformes	Anystidae	SSJAC1509-13	KM834109
Trombidiformes	Anystidae	SSJAC1514-13	KM831252
Trombidiformes	Anystidae	SSJAC1523-13	KM831254
Trombidiformes	Anystidae	SSJAC179-13	KM834067
Trombidiformes	Anystidae	SSJAC180-13	KM833519
Trombidiformes	Anystidae	SSJAC181-13	KM840588
Trombidiformes	Anystidae	SSJAC182-13	KM840128
Trombidiformes	Anystidae	SSJAC183-13	KM831572
Trombidiformes	Anystidae	SSJAC184-13	KM830488
Trombidiformes	Anystidae	SSJAC190-13	KM837286
Trombidiformes	Anystidae	SSJAC191-13	KM840074
Trombidiformes	Anystidae	SSJAC192-13	KM836666
Trombidiformes	Anystidae	SSJAC193-13	KM838505
Trombidiformes	Anystidae	SSJAC194-13	KM838371
Trombidiformes	Anystidae	SSJAC195-13	KM833385
Trombidiformes	Anystidae	SSJAC196-13	KM825715
Trombidiformes	Anystidae	SSJAC980-13	KM835028
Trombidiformes	Anystidae	SSJAC995-13	KM830056
Trombidiformes	Anystidae	SSJAE4327-13	KM832016
Trombidiformes	Anystidae	SSJAE4331-13	KM829549
Trombidiformes	Anystidae	SSJAE4352-13	KM828456
Trombidiformes	Anystidae	SSJAE4359-13	KM829032
Trombidiformes	Anystidae	SSJAE5087-13	KM824336
Trombidiformes	Anystidae	SSJAE5122-13	KM830643
Trombidiformes	Anystidae	SSJAE5646-13	KM830767
Trombidiformes	Anystidae	SSJAE5666-13	KM831809

Trombidiformes	Anystidae		SSPAA2219-13	KM839999
Trombidiformes	Anystidae		SSPAA2226-13	KM830159
Trombidiformes	Anystidae		SSPAA2229-13	KM838480
Trombidiformes	Anystidae		SSPAA2237-13	KM828279
Trombidiformes	Anystidae		SSPAA2243-13	KM824018
Trombidiformes	Anystidae		SSPAA2248-13	KM827676
Trombidiformes	Anystidae		SSPAA2289-13	KM828674
Trombidiformes	Anystidae		SSPAA6621-13	KM824007
Trombidiformes	Anystidae		SSPAA6641-13	KM830907
Trombidiformes	Anystidae		SSPAA6670-13	KM837383
Trombidiformes	Anystidae		SSPAA6673-13	KM835684
Trombidiformes	Anystidae		SSPAA6674-13	KM827924
Trombidiformes	Anystidae		SSPAA6676-13	KM828971
Trombidiformes	Anystidae		SSPAA6679-13	KM838131
Trombidiformes	Anystidae		SSPAA6688-13	KM826646
Trombidiformes	Anystidae		SSPAA6696-13	KM833333
Trombidiformes	Anystidae		SSPAA6697-13	KM826951
Trombidiformes	Anystidae		SSPAA6709-13	KM830412
Trombidiformes	Anystidae		SSPAA7760-13	KM840789
Trombidiformes	Anystidae		SSPAA7780-13	KM838694
Trombidiformes	Anystidae		SSPAA7789-13	KM840082
Trombidiformes	Anystidae		SSPAA7833-13	KM831731
Trombidiformes	Anystidae		SSPAA7940-13	KM827027
Trombidiformes	Anystidae		SSPAA7960-13	KM840084
Trombidiformes	Anystidae		SSPAB3198-13	KM827642
Trombidiformes	Anystidae		SSWEE119-13	KM825855
Trombidiformes	Anystidae		SSWLA683-13	KM825192
Trombidiformes	Anystidae		SSWLB1520-13	KM839541
Trombidiformes	Anystidae		SSWLB2457-13	KM840568
Trombidiformes	Anystidae		SSWLB2491-13	KM824683
Trombidiformes	Anystidae		SSWLB3428-13	KM837631
Trombidiformes	Anystidae		SSWLB3464-13	KM835976
Trombidiformes	Anystidae		SSWLB3539-13	KM824124
Trombidiformes	Anystidae		SSWLB6800-13	KM826884
Trombidiformes	Anystidae		SSWLE2957-13	KM835908
Trombidiformes	Anystidae		SSWLE3963-13	KM835930
Trombidiformes	Anystidae		SSWLE455-13	KM839126
Trombidiformes	Anystidae		SSWLE473-13	KM825542
Trombidiformes	Arrenuridae	*Arrenurus*	CNNHA1945-14	KR070361
Trombidiformes	Arrenuridae	*Arrenurus*	ARCN066-10	HQ924289
Trombidiformes	Arrenuridae	*Arrenurus*	ARCN070-10	HQ924293
Trombidiformes	Arrenuridae	*Arrenurus*	ARCN071-10	HQ924294
Trombidiformes	Arrenuridae	*Arrenurus*	ARCN072-10	HQ924295
Trombidiformes	Arrenuridae	*Arrenurus*	ARCN073-10	HQ924296
Trombidiformes	Arrenuridae	*Arrenurus*	ARCN074-10	HQ924297
Trombidiformes	Arrenuridae	*Arrenurus*	ARCN088-10	HQ924308
Trombidiformes	Arrenuridae	*Arrenurus*	ARCN089-10	HQ924309
Trombidiformes	Arrenuridae	*Arrenurus*	ARCN091-10	HQ924311
Trombidiformes	Arrenuridae	*Arrenurus*	ARCN092-10	HQ924312
Trombidiformes	Arrenuridae	*Arrenurus*	CNPPB2356-12	KJ088280
Trombidiformes	Arrenuridae	*Arrenurus*	CNPPB2357-12	KJ164435
Trombidiformes	Arrenuridae	*Arrenurus*	CNPPB2360-12	KJ089982
Trombidiformes	Arrenuridae	*Arrenurus*	CNPPE1440-12	KJ083628

Trombidiformes	Arrenuridae	*Arrenurus*	CNPPE2126-12	KJ089118
Trombidiformes	Arrenuridae	*Arrenurus*	CNPPE2128-12	KJ167600
Trombidiformes	Arrenuridae	*Arrenurus*	JSOIE074-12	KR069474
Trombidiformes	Arrenuridae	*Arrenurus*	MIONB453-11	KM825054
Trombidiformes	Arrenuridae	*Arrenurus*	SSBAB1129-12	KM838262
Trombidiformes	Arrenuridae	*Arrenurus*	SSBAF4469-13	KM828892
Trombidiformes	Arrenuridae	*Arrenurus*	SSBAF4471-13	KM835514
Trombidiformes	Arrenuridae	*Arrenurus*	SSBAF4482-13	KM824201
Trombidiformes	Arrenuridae	*Arrenurus*	SSBAF4487-13	KM835403
Trombidiformes	Arrenuridae	*Arrenurus*	SSBAF4491-13	KM832883
Trombidiformes	Arrenuridae	*Arrenurus*	SSJAB3341-13	KM840459
Trombidiformes	Arrenuridae	*Arrenurus*	SSJAB3345-13	KM834658
Trombidiformes	Arrenuridae	*Arrenurus*	SSJAB3348-13	KM836788
Trombidiformes	Arrenuridae	*Arrenurus*	SSJAB3350-13	KM833781
Trombidiformes	Arrenuridae	*Arrenurus*	SSJAB3353-13	KM827589
Trombidiformes	Arrenuridae	*Arrenurus*	SSJAB3355-13	KM830409
Trombidiformes	Arrenuridae	*Arrenurus*	SSJAB3356-13	KM829270
Trombidiformes	Arrenuridae	*Arrenurus*	SSJAB3358-13	KM828061
Trombidiformes	Arrenuridae	*Arrenurus*	SSJAB3359-13	KM837528
Trombidiformes	Arrenuridae	*Arrenurus*	SSJAB3361-13	KM824017
Trombidiformes	Arrenuridae	*Arrenurus*	SSJAB3366-13	KM830586
Trombidiformes	Arrenuridae	*Arrenurus*	SSJAE12106-13	KM840544
Trombidiformes	Arrenuridae	*Arrenurus*	SSPAA2284-13	KM833746
Trombidiformes	Arrenuridae	*Arrenurus*	SSPAA6616-13	KM824875
Trombidiformes	Arrenuridae	*Arrenurus*	SSPAA6618-13	KM838879
Trombidiformes	Arrenuridae	*Arrenurus*	SSPAA6620-13	KM839979
Trombidiformes	Arrenuridae	*Arrenurus*	SSPAA6622-13	KM832553
Trombidiformes	Arrenuridae	*Arrenurus*	SSPAA6623-13	KM829339
Trombidiformes	Arrenuridae	*Arrenurus*	SSPAA6626-13	KM825250
Trombidiformes	Arrenuridae	*Arrenurus*	SSPAA6629-13	KM837220
Trombidiformes	Arrenuridae	*Arrenurus*	SSPAA6633-13	KM833583
Trombidiformes	Arrenuridae	*Arrenurus*	SSPAA6634-13	KM837585
Trombidiformes	Arrenuridae	*Arrenurus*	SSPAA6635-13	KM832779
Trombidiformes	Arrenuridae	*Arrenurus*	SSPAA6636-13	KM832103
Trombidiformes	Arrenuridae	*Arrenurus*	SSPAA6637-13	KM836482
Trombidiformes	Arrenuridae	*Arrenurus*	SSPAA6638-13	KM839558
Trombidiformes	Arrenuridae	*Arrenurus*	SSPAA6639-13	KM834864
Trombidiformes	Arrenuridae	*Arrenurus*	SSPAA6694-13	KM833001
Trombidiformes	Arrenuridae	*Arrenurus longicaudatus*	ARCN015-10	HQ924260
Trombidiformes	Arrenuridae	*Arrenurus planus*	ARCN090-10	HQ924310
Trombidiformes	Arrenuridae	*Arrenurus wardi*	SSPAA2239-13	KM827870
Trombidiformes	Arrenuridae	*Arrenurus wardi*	SSPAA2246-13	KM833758
Trombidiformes	Arrenuridae	*Arrenurus wardi*	SSPAA2247-13	KM831006
Trombidiformes	Arrenuridae	*Arrenurus wardi*	SSPAA2249-13	KM839514
Trombidiformes	Arrenuridae	*Arrenurus wardi*	SSPAA2256-13	KM831767
Trombidiformes	Arrenuridae	*Arrenurus wardi*	SSPAA2261-13	KM827641
Trombidiformes	Arrenuridae	*Arrenurus wardi*	SSPAA2262-13	KM832367
Trombidiformes	Arrenuridae	*Arrenurus wardi*	SSPAA2263-13	KM838659
Trombidiformes	Arrenuridae	*Arrenurus wardi*	SSPAA2264-13	KM826410
Trombidiformes	Arrenuridae	*Arrenurus wardi*	SSPAA2265-13	KM830004
Trombidiformes	Arrenuridae	*Arrenurus wardi*	SSPAA2268-13	KM833124
Trombidiformes	Arrenuridae	*Arrenurus wardi*	SSPAA2269-13	KM825703
Trombidiformes	Arrenuridae	*Arrenurus wardi*	SSPAA2274-13	KM838807

Trombidiformes	Arrenuridae	*Arrenurus wardi*	SSPAA2277-13	KM827240
Trombidiformes	Arrenuridae	*Arrenurus wardi*	SSPAA2281-13	KM827656
Trombidiformes	Arrenuridae	*Arrenurus wardi*	SSPAA2288-13	KM828115
Trombidiformes	Arrenuridae	*Arrenurus wardi*	SSPAA2294-13	KM828158
Trombidiformes	Arrenuridae	*Arrenurus wardi*	SSPAA2296-13	KM836945
Trombidiformes	Arrenuridae	*Arrenurus wardi*	SSPAA2301-13	KM835151
Trombidiformes	Arrenuridae	*Arrenurus wardi*	SSPAA2302-13	KM824466
Trombidiformes	Arrenuridae	*Arrenurus wardi*	SSPAA6631-13	KM828567
Trombidiformes	Arrenuridae	*Arrenurus wardi*	SSPAA6643-13	KM833176
Trombidiformes	Arrenuridae	*Arrenurus wardi*	SSPAA6649-13	KM838103
Trombidiformes	Arrenuridae		BIOAI151-14	KR069680
Trombidiformes	Arrenuridae		SSBAD3084-12	KM831888
Trombidiformes	Bdellidae	*Biscuris*	CHACB1148-10	KR069687
Trombidiformes	Bdellidae	*Biscuris*	MIONB183-10	KP979287
Trombidiformes	Bdellidae	*Biscuris*	MYMCA466-11	JX835361
Trombidiformes	Bdellidae	*Biscuris*	MYMCB404-11	JX833945
Trombidiformes	Bdellidae	*Biscuris*	MYMCB524-11	JX833923
Trombidiformes	Bdellidae	*Biscuris*	MYMCB560-11	JX836568
Trombidiformes	Bdellidae	*Biscuris*	MYMCF281-12	JX835785
Trombidiformes	Bdellidae	*Cyta*	CHACA026-08	JX835483
Trombidiformes	Bdellidae	*Cyta*	CHACA029-08	JX837561
Trombidiformes	Bdellidae	*Cyta*	CHACA104-08	JX836423
Trombidiformes	Bdellidae	*Cyta*	CHACA1047-10	HM907099
Trombidiformes	Bdellidae	*Cyta*	CHACA1072-10	HM907115
Trombidiformes	Bdellidae	*Cyta*	CHACA1183-10	JX838055
Trombidiformes	Bdellidae	*Cyta*	CHACA1184-10	JX833739
Trombidiformes	Bdellidae	*Cyta*	CHACA129-08	JX834538
Trombidiformes	Bdellidae	*Cyta*	CHACA130-08	JX838525
Trombidiformes	Bdellidae	*Cyta*	CHACA135-08	JX833927
Trombidiformes	Bdellidae	*Cyta*	CHACA136-08	JX834144
Trombidiformes	Bdellidae	*Cyta*	CHACA137-08	JX835020
Trombidiformes	Bdellidae	*Cyta*	CHACA148-08	JX833885
Trombidiformes	Bdellidae	*Cyta*	CHACA149-08	JX836949
Trombidiformes	Bdellidae	*Cyta*	CHACA371-08	JX838598
Trombidiformes	Bdellidae	*Cyta*	CHACA542-09	KP979133
Trombidiformes	Bdellidae	*Cyta*	CHACA567-09	JX834571
Trombidiformes	Bdellidae	*Cyta*	CHACA569-09	JX838349
Trombidiformes	Bdellidae	*Cyta*	CHACA570-09	JX837552
Trombidiformes	Bdellidae	*Cyta*	CHACA571-09	JX833872
Trombidiformes	Bdellidae	*Cyta*	CHACA572-09	JX834828
Trombidiformes	Bdellidae	*Cyta*	CHACA573-09	JX835965
Trombidiformes	Bdellidae	*Cyta*	CHACA815-09	JX835762
Trombidiformes	Bdellidae	*Cyta*	CHACA970-10	HM405835
Trombidiformes	Bdellidae	*Cyta*	CHACB018-10	HQ558339
Trombidiformes	Bdellidae	*Cyta*	CHACB092-10	HQ558391
Trombidiformes	Bdellidae	*Cyta*	CHACB1022-10	HM907155
Trombidiformes	Bdellidae	*Cyta*	CHACB135-10	HQ558419
Trombidiformes	Bdellidae	*Cyta*	CHACB136-10	HQ558420
Trombidiformes	Bdellidae	*Cyta*	CHACB137-10	HQ558421
Trombidiformes	Bdellidae	*Cyta*	CHACB242-10	HQ558487
Trombidiformes	Bdellidae	*Cyta*	CHACB243-10	HQ558488
Trombidiformes	Bdellidae	*Cyta*	CHACB270-10	HQ558508
Trombidiformes	Bdellidae	*Cyta*	CHACB383-10	HQ558556

Trombidiformes	Bdellidae	*Cyta*	CHACB422-10	HQ558579
Trombidiformes	Bdellidae	*Cyta*	CHACB423-10	HQ558580
Trombidiformes	Bdellidae	*Cyta*	CHACB424-10	HQ558581
Trombidiformes	Bdellidae	*Cyta*	CHACB425-10	HQ558582
Trombidiformes	Bdellidae	*Cyta*	CHACB494-10	HQ558629
Trombidiformes	Bdellidae	*Cyta*	CHACB495-10	HQ558630
Trombidiformes	Bdellidae	*Cyta*	CHACB496-10	HQ558631
Trombidiformes	Bdellidae	*Cyta*	CHACB497-10	HQ558632
Trombidiformes	Bdellidae	*Cyta*	CHACB555-10	HQ558671
Trombidiformes	Bdellidae	*Cyta*	CHACB615-10	HQ558702
Trombidiformes	Bdellidae	*Cyta*	CHACB703-10	JX835994
Trombidiformes	Bdellidae	*Cyta*	CHACB824-10	HQ941498
Trombidiformes	Bdellidae	*Cyta*	CHACB825-10	HQ941499
Trombidiformes	Bdellidae	*Cyta*	CHACB882-10	HQ941515
Trombidiformes	Bdellidae	*Cyta*	CHACB903-10	JX836711
Trombidiformes	Bdellidae	*Cyta*	CHACB971-10	JX834872
Trombidiformes	Bdellidae	*Cyta*	CHACB993-10	HM907398
Trombidiformes	Bdellidae	*Cyta*	CHACC049-10	JX836479
Trombidiformes	Bdellidae	*Cyta*	CHACC050-10	HM907449
Trombidiformes	Bdellidae	*Cyta*	CHACC052-10	HM907451
Trombidiformes	Bdellidae	*Cyta*	CHACC055-10	HM907453
Trombidiformes	Bdellidae	*Cyta*	CHACC056-10	HM907454
Trombidiformes	Bdellidae	*Cyta*	CHACC057-10	HM907455
Trombidiformes	Bdellidae	*Cyta*	CHACC058-10	JX836063
Trombidiformes	Bdellidae	*Cyta*	CHACC106-10	JX834336
Trombidiformes	Bdellidae	*Cyta*	CHACC191-10	HQ941517
Trombidiformes	Bdellidae	*Cyta*	CHACC192-10	HQ941518
Trombidiformes	Bdellidae	*Cyta*	CHACC247-10	HQ941552
Trombidiformes	Bdellidae	*Cyta*	CHACC260-10	HQ941564
Trombidiformes	Bdellidae	*Cyta*	CHACC262-10	HQ941565
Trombidiformes	Bdellidae	*Cyta*	CHACC263-10	HQ941566
Trombidiformes	Bdellidae	*Cyta*	CHACC264-10	HQ941567
Trombidiformes	Bdellidae	*Cyta*	CHACC265-10	KR070674
Trombidiformes	Bdellidae	*Cyta*	CHACC282-10	HQ941576
Trombidiformes	Bdellidae	*Cyta*	CHACC283-10	HQ941577
Trombidiformes	Bdellidae	*Cyta*	CHACC284-10	HQ941578
Trombidiformes	Bdellidae	*Cyta*	CHACC285-10	HQ941579
Trombidiformes	Bdellidae	*Cyta*	CHACC286-10	HQ558787
Trombidiformes	Bdellidae	*Cyta*	CHACC287-10	HQ558788
Trombidiformes	Bdellidae	*Cyta*	CHACC288-10	HQ558789
Trombidiformes	Bdellidae	*Cyta*	CHACC289-10	HQ558790
Trombidiformes	Bdellidae	*Cyta*	CHACC290-10	HQ558791
Trombidiformes	Bdellidae	*Cyta*	CNBAD673-12	KM840719
Trombidiformes	Bdellidae	*Cyta*	CNBAD686-12	KM838029
Trombidiformes	Bdellidae	*Cyta*	CNBAD690-12	KM834631
Trombidiformes	Bdellidae	*Cyta*	CNBAE231-12	KM824165
Trombidiformes	Bdellidae	*Cyta*	CNGIB579-12	KM833873
Trombidiformes	Bdellidae	*Cyta*	CNGIB586-12	KM835677
Trombidiformes	Bdellidae	*Cyta*	CNJAC1492-12	KM833417
Trombidiformes	Bdellidae	*Cyta*	CNPPA4084-12	KJ088714
Trombidiformes	Bdellidae	*Cyta*	MHMIT001-07	KR070212
Trombidiformes	Bdellidae	*Cyta*	MHMIT002-07	KR070179
Trombidiformes	Bdellidae	*Cyta*	MHMIT003-07	KR069350

Trombidiformes	Bdellidae	*Cyta*		MHMIT004-07	KR069428
Trombidiformes	Bdellidae	*Cyta*		MHMIT006-07	KR070141
Trombidiformes	Bdellidae	*Cyta*		MHMIT007-07	KR069413
Trombidiformes	Bdellidae	*Cyta*		MHMIT008-07	KR070355
Trombidiformes	Bdellidae	*Cyta*		MHMIT009-07	KR069868
Trombidiformes	Bdellidae	*Cyta*		MHMIT010-07	KR070406
Trombidiformes	Bdellidae	*Cyta*		MHMIT011-07	KR070274
Trombidiformes	Bdellidae	*Cyta*		MHMIT012-07	KR070520
Trombidiformes	Bdellidae	*Cyta*		MHMIT013-07	KR069492
Trombidiformes	Bdellidae	*Cyta*		MHMIT014-07	KR069417
Trombidiformes	Bdellidae	*Cyta*		MHMIT015-07	KR069738
Trombidiformes	Bdellidae	*Cyta*		MHMIT016-07	KR069427
Trombidiformes	Bdellidae	*Cyta*		MHMIT017-07	KR070234
Trombidiformes	Bdellidae	*Cyta*		MHMIT018-07	KR070715
Trombidiformes	Bdellidae	*Cyta*		MHMIT019-07	KR069789
Trombidiformes	Bdellidae	*Cyta*		MHMIT020-07	KR069695
Trombidiformes	Bdellidae	*Cyta*		MHMIT021-07	KR070491
Trombidiformes	Bdellidae	*Cyta*		MHMIT022-07	KR069285
Trombidiformes	Bdellidae	*Cyta*		MHMIT023-07	KR069214
Trombidiformes	Bdellidae	*Cyta*		MHMIT024-07	KR070211
Trombidiformes	Bdellidae	*Cyta*		MHMIT026-07	KR070652
Trombidiformes	Bdellidae	*Cyta*		MHMIT028-07	KR069542
Trombidiformes	Bdellidae	*Cyta*		MHMIT029-07	KR070609
Trombidiformes	Bdellidae	*Cyta*		MHMIT030-07	KR069552
Trombidiformes	Bdellidae	*Cyta*		MHMIT031-07	KR069333
Trombidiformes	Bdellidae	*Cyta*		MHMIT032-07	KR070322
Trombidiformes	Bdellidae	*Cyta*		MHMIT034-07	KR069632
Trombidiformes	Bdellidae	*Cyta*		MHMIT035-07	KR070186
Trombidiformes	Bdellidae	*Cyta*		MHMIT036-07	KR070162
Trombidiformes	Bdellidae	*Cyta*		MHMIT037-07	KR070450
Trombidiformes	Bdellidae	*Cyta*		MHMIT058-07	KR070159
Trombidiformes	Bdellidae	*Cyta*		MHMIT059-07	KR070058
Trombidiformes	Bdellidae	*Cyta*		MIONB023-10	HM887567
Trombidiformes	Bdellidae	*Cyta*		MIONB518-11	KM826091
Trombidiformes	Bdellidae	*Cyta*		MIONB533-11	KP979206
Trombidiformes	Bdellidae	*Cyta*		MYMCA030-11	JX833886
Trombidiformes	Bdellidae	*Cyta*		MYMCA031-11	JX838706
Trombidiformes	Bdellidae	*Cyta*		MYMCA050-11	JX834684
Trombidiformes	Bdellidae	*Cyta*		MYMCA064-11	JX837884
Trombidiformes	Bdellidae	*Cyta*		MYMCA096-11	JX838288
Trombidiformes	Bdellidae	*Cyta*		MYMCA097-11	JX834294
Trombidiformes	Bdellidae	*Cyta*		MYMCA098-11	JX835087
Trombidiformes	Bdellidae	*Cyta*		MYMCA099-11	JX838693
Trombidiformes	Bdellidae	*Cyta*		MYMCA1000-11	JX837408
Trombidiformes	Bdellidae	*Cyta*		MYMCA1001-11	JX833765
Trombidiformes	Bdellidae	*Cyta*		MYMCA1002-11	JX835724
Trombidiformes	Bdellidae	*Cyta*		MYMCA1033-11	JX833730
Trombidiformes	Bdellidae	*Cyta*		MYMCA105-11	JX838494
Trombidiformes	Bdellidae	*Cyta*		MYMCA107-11	JX836293
Trombidiformes	Bdellidae	*Cyta*		MYMCA108-11	JX836092
Trombidiformes	Bdellidae	*Cyta*		MYMCA1087-11	JX836185
Trombidiformes	Bdellidae	*Cyta*		MYMCA109-11	JX835180
Trombidiformes	Bdellidae	*Cyta*		MYMCA1121-11	JX838760

Trombidiformes	Bdellidae	*Cyta*	MYMCA116-11	JX838763
Trombidiformes	Bdellidae	*Cyta*	MYMCA117-11	JX836098
Trombidiformes	Bdellidae	*Cyta*	MYMCA118-11	JX837915
Trombidiformes	Bdellidae	*Cyta*	MYMCA121-11	JX838574
Trombidiformes	Bdellidae	*Cyta*	MYMCA122-11	JX835453
Trombidiformes	Bdellidae	*Cyta*	MYMCA123-11	JX834219
Trombidiformes	Bdellidae	*Cyta*	MYMCA1236-11	JX834869
Trombidiformes	Bdellidae	*Cyta*	MYMCA1237-11	JX838278
Trombidiformes	Bdellidae	*Cyta*	MYMCA1320-11	JX836894
Trombidiformes	Bdellidae	*Cyta*	MYMCA1332-11	JX836245
Trombidiformes	Bdellidae	*Cyta*	MYMCA1333-11	JX838419
Trombidiformes	Bdellidae	*Cyta*	MYMCA1364-11	JX836825
Trombidiformes	Bdellidae	*Cyta*	MYMCA1365-11	JX835943
Trombidiformes	Bdellidae	*Cyta*	MYMCA1404-11	JX837993
Trombidiformes	Bdellidae	*Cyta*	MYMCA1452-11	JX837191
Trombidiformes	Bdellidae	*Cyta*	MYMCA1453-11	JX837024
Trombidiformes	Bdellidae	*Cyta*	MYMCA1475-11	JX833815
Trombidiformes	Bdellidae	*Cyta*	MYMCA1476-11	JX836246
Trombidiformes	Bdellidae	*Cyta*	MYMCA1477-11	JX834421
Trombidiformes	Bdellidae	*Cyta*	MYMCA1478-11	JX834854
Trombidiformes	Bdellidae	*Cyta*	MYMCA1479-11	JX836751
Trombidiformes	Bdellidae	*Cyta*	MYMCA253-11	JX838012
Trombidiformes	Bdellidae	*Cyta*	MYMCA291-11	JX834866
Trombidiformes	Bdellidae	*Cyta*	MYMCA334-11	JX837436
Trombidiformes	Bdellidae	*Cyta*	MYMCA560-11	JX834982
Trombidiformes	Bdellidae	*Cyta*	MYMCA586-11	JX838554
Trombidiformes	Bdellidae	*Cyta*	MYMCA587-11	JX834923
Trombidiformes	Bdellidae	*Cyta*	MYMCA609-11	JX836737
Trombidiformes	Bdellidae	*Cyta*	MYMCA610-11	JX835982
Trombidiformes	Bdellidae	*Cyta*	MYMCA638-11	JX834067
Trombidiformes	Bdellidae	*Cyta*	MYMCA639-11	JX835985
Trombidiformes	Bdellidae	*Cyta*	MYMCA661-11	JX837903
Trombidiformes	Bdellidae	*Cyta*	MYMCA664-11	JX837881
Trombidiformes	Bdellidae	*Cyta*	MYMCA665-11	JX836420
Trombidiformes	Bdellidae	*Cyta*	MYMCA684-11	JX837964
Trombidiformes	Bdellidae	*Cyta*	MYMCA698-11	JX834700
Trombidiformes	Bdellidae	*Cyta*	MYMCA699-11	JX838018
Trombidiformes	Bdellidae	*Cyta*	MYMCA700-11	JX835432
Trombidiformes	Bdellidae	*Cyta*	MYMCA701-11	JX836725
Trombidiformes	Bdellidae	*Cyta*	MYMCA702-11	JX837643
Trombidiformes	Bdellidae	*Cyta*	MYMCA736-11	JX836566
Trombidiformes	Bdellidae	*Cyta*	MYMCA745-11	JX838757
Trombidiformes	Bdellidae	*Cyta*	MYMCA746-11	JX838775
Trombidiformes	Bdellidae	*Cyta*	MYMCA753-11	JX834133
Trombidiformes	Bdellidae	*Cyta*	MYMCA754-11	JX834320
Trombidiformes	Bdellidae	*Cyta*	MYMCA838-11	JX834972
Trombidiformes	Bdellidae	*Cyta*	MYMCA953-11	JX833858
Trombidiformes	Bdellidae	*Cyta*	MYMCA954-11	JX837541
Trombidiformes	Bdellidae	*Cyta*	MYMCA955-11	JX835476
Trombidiformes	Bdellidae	*Cyta*	MYMCA956-11	JX835036
Trombidiformes	Bdellidae	*Cyta*	MYMCA957-11	JX833964
Trombidiformes	Bdellidae	*Cyta*	MYMCA958-11	JX835319
Trombidiformes	Bdellidae	*Cyta*	MYMCA959-11	JX834721

Trombidiformes	Bdellidae	*Cyta*	MYMCA960-11	JX834898
Trombidiformes	Bdellidae	*Cyta*	MYMCA961-11	JX835477
Trombidiformes	Bdellidae	*Cyta*	MYMCB042-11	JX836048
Trombidiformes	Bdellidae	*Cyta*	MYMCB047-11	JX836635
Trombidiformes	Bdellidae	*Cyta*	MYMCB076-11	JX836953
Trombidiformes	Bdellidae	*Cyta*	MYMCB110-11	JX836961
Trombidiformes	Bdellidae	*Cyta*	MYMCB128-11	JX833982
Trombidiformes	Bdellidae	*Cyta*	MYMCB208-11	JX834276
Trombidiformes	Bdellidae	*Cyta*	MYMCB270-11	JX836118
Trombidiformes	Bdellidae	*Cyta*	MYMCB405-11	JX835072
Trombidiformes	Bdellidae	*Cyta*	MYMCB425-11	JX836427
Trombidiformes	Bdellidae	*Cyta*	MYMCB461-11	JX837462
Trombidiformes	Bdellidae	*Cyta*	MYMCB608-11	JX837257
Trombidiformes	Bdellidae	*Cyta*	MYMCB691-11	JX835636
Trombidiformes	Bdellidae	*Cyta*	MYMCB785-11	JX835441
Trombidiformes	Bdellidae	*Cyta*	MYMCB786-11	JX836219
Trombidiformes	Bdellidae	*Cyta*	MYMCB837-11	JX837283
Trombidiformes	Bdellidae	*Cyta*	MYMCB839-11	JX834669
Trombidiformes	Bdellidae	*Cyta*	MYMCB869-11	JX833675
Trombidiformes	Bdellidae	*Cyta*	MYMCB885-11	JX835354
Trombidiformes	Bdellidae	*Cyta*	MYMCB886-11	JX834494
Trombidiformes	Bdellidae	*Cyta*	MYMCB922-11	JX833860
Trombidiformes	Bdellidae	*Cyta*	MYMCB934-11	JX834646
Trombidiformes	Bdellidae	*Cyta*	MYMCB936-11	JX833853
Trombidiformes	Bdellidae	*Cyta*	MYMCC023-11	JX836273
Trombidiformes	Bdellidae	*Cyta*	MYMCC024-11	JX835466
Trombidiformes	Bdellidae	*Cyta*	MYMCC032-11	JX838491
Trombidiformes	Bdellidae	*Cyta*	MYMCC128-11	JX836007
Trombidiformes	Bdellidae	*Cyta*	MYMCC156-11	JX838533
Trombidiformes	Bdellidae	*Cyta*	MYMCC202-11	JX836756
Trombidiformes	Bdellidae	*Cyta*	MYMCC264-11	JX835171
Trombidiformes	Bdellidae	*Cyta*	MYMCC324-11	JX836030
Trombidiformes	Bdellidae	*Cyta*	MYMCC325-11	JX836764
Trombidiformes	Bdellidae	*Cyta*	MYMCC332-11	JX838503
Trombidiformes	Bdellidae	*Cyta*	MYMCC424-11	JX835129
Trombidiformes	Bdellidae	*Cyta*	MYMCC500-11	JX834612
Trombidiformes	Bdellidae	*Cyta*	MYMCC512-11	JX837829
Trombidiformes	Bdellidae	*Cyta*	MYMCC583-11	JX835046
Trombidiformes	Bdellidae	*Cyta*	MYMCC626-11	JX835304
Trombidiformes	Bdellidae	*Cyta*	MYMCC656-11	JX834337
Trombidiformes	Bdellidae	*Cyta*	MYMCC657-11	JX836876
Trombidiformes	Bdellidae	*Cyta*	MYMCC701-11	JX836602
Trombidiformes	Bdellidae	*Cyta*	MYMCC702-11	JX836383
Trombidiformes	Bdellidae	*Cyta*	MYMCC728-11	JX837138
Trombidiformes	Bdellidae	*Cyta*	MYMCC765-11	JX834103
Trombidiformes	Bdellidae	*Cyta*	MYMCC776-11	JX836600
Trombidiformes	Bdellidae	*Cyta*	MYMCC777-11	JX837432
Trombidiformes	Bdellidae	*Cyta*	MYMCC782-11	JX836112
Trombidiformes	Bdellidae	*Cyta*	MYMCC783-11	JX837613
Trombidiformes	Bdellidae	*Cyta*	MYMCC816-11	JX836967
Trombidiformes	Bdellidae	*Cyta*	MYMCC817-11	JX834765
Trombidiformes	Bdellidae	*Cyta*	MYMCC828-11	JX833898
Trombidiformes	Bdellidae	*Cyta*	MYMCC829-11	JX833711

Trombidiformes	Bdellidae	*Cyta*	MYMCC850-11	JX834709
Trombidiformes	Bdellidae	*Cyta*	MYMCC851-11	JX837213
Trombidiformes	Bdellidae	*Cyta*	MYMCC897-11	JX838621
Trombidiformes	Bdellidae	*Cyta*	MYMCC898-11	JX838537
Trombidiformes	Bdellidae	*Cyta*	MYMCC917-11	JX835709
Trombidiformes	Bdellidae	*Cyta*	MYMCC944-11	JX838670
Trombidiformes	Bdellidae	*Cyta*	MYMCC946-11	JX838654
Trombidiformes	Bdellidae	*Cyta*	MYMCD018-11	JX836684
Trombidiformes	Bdellidae	*Cyta*	MYMCD019-11	JX834116
Trombidiformes	Bdellidae	*Cyta*	MYMCD022-11	JX835823
Trombidiformes	Bdellidae	*Cyta*	MYMCD023-11	JX834378
Trombidiformes	Bdellidae	*Cyta*	MYMCD048-11	JX837284
Trombidiformes	Bdellidae	*Cyta*	MYMCD081-11	JX835238
Trombidiformes	Bdellidae	*Cyta*	MYMCD082-11	JX836525
Trombidiformes	Bdellidae	*Cyta*	MYMCD121-11	JX833782
Trombidiformes	Bdellidae	*Cyta*	MYMCD133-11	JX838023
Trombidiformes	Bdellidae	*Cyta*	MYMCD134-11	JX833762
Trombidiformes	Bdellidae	*Cyta*	MYMCD169-11	JX836229
Trombidiformes	Bdellidae	*Cyta*	MYMCE007-12	JX835820
Trombidiformes	Bdellidae	*Cyta*	MYMCE043-12	JX835974
Trombidiformes	Bdellidae	*Cyta*	MYMCE044-12	JX836937
Trombidiformes	Bdellidae	*Cyta*	MYMCE072-12	JX835357
Trombidiformes	Bdellidae	*Cyta*	MYMCE254-12	JX835946
Trombidiformes	Bdellidae	*Cyta*	MYMCE256-12	JX835182
Trombidiformes	Bdellidae	*Cyta*	MYMCE298-12	JX838408
Trombidiformes	Bdellidae	*Cyta*	MYMCE324-12	JX837353
Trombidiformes	Bdellidae	*Cyta*	MYMCE343-12	JX838418
Trombidiformes	Bdellidae	*Cyta*	MYMCE350-12	JX835309
Trombidiformes	Bdellidae	*Cyta*	MYMCE355-12	JX834157
Trombidiformes	Bdellidae	*Cyta*	MYMCE417-12	JX834068
Trombidiformes	Bdellidae	*Cyta*	MYMCE450-12	JX835312
Trombidiformes	Bdellidae	*Cyta*	MYMCE452-12	JX836029
Trombidiformes	Bdellidae	*Cyta*	MYMCE453-12	JX836895
Trombidiformes	Bdellidae	*Cyta*	MYMCE454-12	JX836179
Trombidiformes	Bdellidae	*Cyta*	MYMCE490-12	JX835786
Trombidiformes	Bdellidae	*Cyta*	MYMCE530-12	JX837892
Trombidiformes	Bdellidae	*Cyta*	MYMCE621-12	JX836819
Trombidiformes	Bdellidae	*Cyta*	MYMCE622-12	JX837598
Trombidiformes	Bdellidae	*Cyta*	MYMCE645-12	JX836788
Trombidiformes	Bdellidae	*Cyta*	MYMCE675-12	JX837247
Trombidiformes	Bdellidae	*Cyta*	MYMCE701-12	JX838731
Trombidiformes	Bdellidae	*Cyta*	MYMCE756-12	JX836603
Trombidiformes	Bdellidae	*Cyta*	MYMCE757-12	JX838032
Trombidiformes	Bdellidae	*Cyta*	MYMCE785-12	JX835646
Trombidiformes	Bdellidae	*Cyta*	MYMCE849-12	JX834414
Trombidiformes	Bdellidae	*Cyta*	MYMCF001-12	JX838532
Trombidiformes	Bdellidae	*Cyta*	MYMCF002-12	JX837148
Trombidiformes	Bdellidae	*Cyta*	MYMCF256-12	JX838208
Trombidiformes	Bdellidae	*Cyta*	MYMCF383-12	JX835278
Trombidiformes	Bdellidae	*Cyta*	MYMCF408-12	JX834399
Trombidiformes	Bdellidae	*Cyta*	MYMCF418-12	JX836970
Trombidiformes	Bdellidae	*Cyta*	MYMCF419-12	JX835414
Trombidiformes	Bdellidae	*Cyta*	MYMCF436-12	JX834033

Trombidiformes	Bdellidae	*Cyta*	MYMCF437-12	JX837464
Trombidiformes	Bdellidae	*Cyta*	MYMCF464-12	JX834879
Trombidiformes	Bdellidae	*Cyta*	MYMCF625-12	JX835592
Trombidiformes	Bdellidae	*Cyta*	MYMCF654-12	JX834732
Trombidiformes	Bdellidae	*Cyta*	MYMCF711-12	JX835351
Trombidiformes	Bdellidae	*Cyta*	MYMCF755-12	JX835929
Trombidiformes	Bdellidae	*Cyta*	MYMCF800-12	JX838754
Trombidiformes	Bdellidae	*Cyta*	MYMCF802-12	JX835980
Trombidiformes	Bdellidae	*Cyta*	MYMCF815-12	JX834629
Trombidiformes	Bdellidae	*Cyta*	MYMCF816-12	JX837647
Trombidiformes	Bdellidae	*Cyta*	MYMCF822-12	JX835247
Trombidiformes	Bdellidae	*Cyta*	MYMCG130-12	JX833639
Trombidiformes	Bdellidae	*Cyta*	MYMCG132-12	JX837300
Trombidiformes	Bdellidae	*Cyta*	MYMCG133-12	JX836399
Trombidiformes	Bdellidae	*Cyta*	MYMCG149-12	JX837834
Trombidiformes	Bdellidae	*Cyta*	MYMCG182-12	JX837269
Trombidiformes	Bdellidae	*Cyta*	MYMCG183-12	JX835841
Trombidiformes	Bdellidae	*Cyta*	MYMCG184-12	JX838198
Trombidiformes	Bdellidae	*Cyta*	MYMCG209-12	JX838396
Trombidiformes	Bdellidae	*Cyta*	MYMCG210-12	JX838780
Trombidiformes	Bdellidae	*Cyta*	MYMCG272-12	JX837050
Trombidiformes	Bdellidae	*Cyta*	MYMCG290-12	JX833800
Trombidiformes	Bdellidae	*Cyta*	MYMCG362-12	JX836434
Trombidiformes	Bdellidae	*Cyta*	MYMCG405-12	JX836883
Trombidiformes	Bdellidae	*Cyta*	MYMCG415-12	JX834226
Trombidiformes	Bdellidae	*Cyta*	MYMCG452-12	JX835497
Trombidiformes	Bdellidae	*Cyta*	MYMCG474-12	JX836933
Trombidiformes	Bdellidae	*Cyta*	MYMCG496-12	JX834405
Trombidiformes	Bdellidae	*Cyta*	MYMCG568-12	JX834422
Trombidiformes	Bdellidae	*Cyta*	MYMCG632-12	JX836996
Trombidiformes	Bdellidae	*Cyta*	MYTMC032-09	KR070703
Trombidiformes	Bdellidae	*Cyta*	MYTMC041-09	KR070573
Trombidiformes	Bdellidae	*Cyta*	MYTMC141-09	KR069779
Trombidiformes	Bdellidae	*Cyta*	RBINA4468-13	KP979237
Trombidiformes	Bdellidae	*Cyta*	SSBAA2073-12	KM827177
Trombidiformes	Bdellidae	*Cyta*	SSBAA2076-12	KM832195
Trombidiformes	Bdellidae	*Cyta*	SSBAA3615-12	KM826578
Trombidiformes	Bdellidae	*Cyta*	SSBAA3665-12	KM832972
Trombidiformes	Bdellidae	*Cyta*	SSBAA3668-12	KM831134
Trombidiformes	Bdellidae	*Cyta*	SSBAE3766-13	KM836665
Trombidiformes	Bdellidae	*Cyta*	SSEIB8192-13	KM834202
Trombidiformes	Bdellidae	*Cyta*	SSJAC1520-13	KM830024
Trombidiformes	Bdellidae	*Cyta*	SSJAC1533-13	KM826005
Trombidiformes	Bdellidae	*Cyta*	SSJAC1549-13	KM830962
Trombidiformes	Bdellidae	*Cyta*	SSJAC1551-13	KM824339
Trombidiformes	Bdellidae	*Cyta*	SSPAA6702-13	KM825470
Trombidiformes	Bdellidae	*Cyta*	SSPAA7761-13	KM834297
Trombidiformes	Bdellidae	*Cyta*	SSPAA7786-13	KM830105
Trombidiformes	Bdellidae	*Cyta*	SSPAA7802-13	KM831566
Trombidiformes	Bdellidae	*Cyta*	SSPAA7935-13	KM826185
Trombidiformes	Bdellidae	*Cyta*	SSPAC12453-13	KM840607
Trombidiformes	Bdellidae	*Cyta*	SSWLC1178-13	KM830035
Trombidiformes	Bdellidae	*Cyta*	SSWLC3126-13	KM837701

Trombidiformes	Bdellidae		ARCN011-10	HQ924257
Trombidiformes	Bdellidae		CHACB111-10	HQ558404
Trombidiformes	Bdellidae		CNEIF2386-12	KM839510
Trombidiformes	Bdellidae		CNEIF2388-12	KM828423
Trombidiformes	Bdellidae		CNEIG1591-13	KM831050
Trombidiformes	Bdellidae		CNEIH060-13	KM829108
Trombidiformes	Bdellidae		CNEIH062-13	KM830000
Trombidiformes	Bdellidae		CNKJI426-14	KR069494
Trombidiformes	Bdellidae		CNKJI429-14	KR069231
Trombidiformes	Bdellidae		CNKJI496-14	KR069163
Trombidiformes	Bdellidae		CNKJI511-14	KR069423
Trombidiformes	Bdellidae		CNKJI517-14	KR069431
Trombidiformes	Bdellidae		CNKJI522-14	KR069755
Trombidiformes	Bdellidae		CNKJP942-14	KR070426
Trombidiformes	Bdellidae		CNKJQ1080-14	KR070204
Trombidiformes	Bdellidae		CNKJQ1177-14	KR069184
Trombidiformes	Bdellidae		CNKJR448-14	KR070684
Trombidiformes	Bdellidae		CNKJR468-14	KR069371
Trombidiformes	Bdellidae		CNKOI598-14	KR069924
Trombidiformes	Bdellidae		CNKOS1427-14	KR069616
Trombidiformes	Bdellidae		CNKOT246-14	KR070010
Trombidiformes	Bdellidae		CNLMS1066-14	KR070399
Trombidiformes	Bdellidae		CNLMS1101-14	KR069737
Trombidiformes	Bdellidae		CNLMS1131-14	KR069954
Trombidiformes	Bdellidae		CNPAE465-13	KM826748
Trombidiformes	Bdellidae		CNPPB2355-12	KJ090776
Trombidiformes	Bdellidae		CNRMD2666-12	KM826530
Trombidiformes	Bdellidae		CNRMF3264-12	KM825592
Trombidiformes	Bdellidae		CNTNI1667-14	KR069470
Trombidiformes	Bdellidae		MIONB022-10	HM887566
Trombidiformes	Bdellidae		MIONB124-10	KR070661
Trombidiformes	Bdellidae		MIONB182-10	KR070471
Trombidiformes	Bdellidae		MIONB184-10	KR069986
Trombidiformes	Bdellidae		MIONB206-10	KP979132
Trombidiformes	Bdellidae		MIONB224-10	KP979185
Trombidiformes	Bdellidae		MIONB256-10	KP979350
Trombidiformes	Bdellidae		MIONB278-10	KR070570
Trombidiformes	Bdellidae		MIONB279-10	KP979294
Trombidiformes	Bdellidae		MIONB280-10	KP979307
Trombidiformes	Bdellidae		MIONB286-10	KP979178
Trombidiformes	Bdellidae		RBINA3933-13	KP979283
Trombidiformes	Bdellidae		RBINA5704-13	KP979151
Trombidiformes	Bdellidae		SSBAD4049-12	KR070635
Trombidiformes	Bdellidae		SSPAA6689-13	KM836316
Trombidiformes	Bdellidae		SSPAA7799-13	KM840733
Trombidiformes	Bdellidae		SSPAB8742-13	KM840153
Trombidiformes	Bdellidae		SSWLC3119-13	KM832720
Trombidiformes	Bdellidae		SSWLC3124-13	KM830847
Trombidiformes	Bdellidae		SSWLD8326-13	KM834671
Trombidiformes	Calyptostomatidae	*Calyptostoma*	MHMIT057-07	KP979177
Trombidiformes	Calyptostomatidae	*Calyptostoma*	MYMCE120-12	JX834838
Trombidiformes	Calyptostomatidae	*Calyptostoma*	SSEIA7693-13	KM826585
Trombidiformes	Calyptostomatidae	*Calyptostoma*	SSEIB4241-13	KM824224

Trombidiformes	Cheyletidae	*Cheyletus malaccensis*	GACAC524-12	GU064914
Trombidiformes	Cheyletidae	*Cheyletus malaccensis*	GBCH10722-13	KC507934
Trombidiformes	Cheyletidae	*Cheyletus malaccensis*	GBCH10723-13	KC507933
Trombidiformes	Cheyletidae	*Cheyletus malaccensis*	GBCH10724-13	KC507932
Trombidiformes	Cheyletidae	*Cheyletus malaccensis*	GBCH10725-13	KC507931
Trombidiformes	Cheyletidae	*Cheyletus malaccensis*	GBCH10726-13	KC507930
Trombidiformes	Cheyletidae	*Cheyletus malaccensis*	GBCH10727-13	KC507929
Trombidiformes	Cheyletidae	*Cheyletus malaccensis*	GBCH10728-13	KC507928
Trombidiformes	Cheyletidae	*Cheyletus malaccensis*	GBCH10729-13	KC507927
Trombidiformes	Cheyletidae	*Cheyletus malaccensis*	GBCH10730-13	KC507926
Trombidiformes	Cheyletidae	*Cheyletus malaccensis*	GBCH10731-13	KC507925
Trombidiformes	Cheyletidae	*Cheyletus malaccensis*	GBCH10732-13	KC507924
Trombidiformes	Cheyletidae	*Cheyletus malaccensis*	GBCH10733-13	KC507923
Trombidiformes	Cheyletidae	*Cheyletus malaccensis*	GBCH10734-13	KC507922
Trombidiformes	Cheyletidae	*Cheyletus malaccensis*	GBCH10735-13	KC507921
Trombidiformes	Cheyletidae	*Cheyletus malaccensis*	GBCH10736-13	KC507920
Trombidiformes	Cheyletidae	*Cheyletus malaccensis*	GBCH10737-13	KC507919
Trombidiformes	Cheyletidae	*Cheyletus malaccensis*	GBCH10738-13	KC507918
Trombidiformes	Cheyletidae	*Cheyletus malaccensis*	GBCH10739-13	KC507917
Trombidiformes	Cheyletidae	*Cheyletus malaccensis*	GBCH10740-13	KC507916
Trombidiformes	Cheyletidae	*Cheyletus malaccensis*	GBCH10741-13	KC507915
Trombidiformes	Cheyletidae	*Cheyletus malaccensis*	GBCH10742-13	KC507914
Trombidiformes	Cheyletidae	*Cheyletus malaccensis*	GBCH10743-13	KC507913
Trombidiformes	Cheyletidae	*Cheyletus malaccensis*	GBCH10744-13	KC507912
Trombidiformes	Cheyletidae	*Cheyletus malaccensis*	GBCH10745-13	KC507911
Trombidiformes	Cheyletidae	*Cheyletus malaccensis*	GBCH10746-13	KC507910
Trombidiformes	Cheyletidae	*Cheyletus malaccensis*	GBCH10747-13	KC507909
Trombidiformes	Cheyletidae	*Cheyletus malaccensis*	GBCH10748-13	KC507908
Trombidiformes	Cheyletidae	*Cheyletus malaccensis*	GBCH10749-13	KC507907
Trombidiformes	Cheyletidae	*Cheyletus malaccensis*	GBCH10750-13	KC507906
Trombidiformes	Cheyletidae	*Cheyletus malaccensis*	GBCH10751-13	KC507905
Trombidiformes	Cheyletidae	*Cheyletus malaccensis*	GBCH10752-13	KC507904
Trombidiformes	Cheyletidae	*Cheyletus malaccensis*	GBCH10753-13	KC507903
Trombidiformes	Cheyletidae	*Cheyletus malaccensis*	GBCH10754-13	KC507902
Trombidiformes	Cheyletidae	*Cheyletus malaccensis*	GBCH10755-13	KC507901
Trombidiformes	Cheyletidae	*Cheyletus malaccensis*	GBCH10756-13	KC507900
Trombidiformes	Cheyletidae	*Cheyletus malaccensis*	GBCH10757-13	KC507899
Trombidiformes	Cheyletidae	*Cheyletus malaccensis*	GBCH10758-13	KC507898
Trombidiformes	Cheyletidae	*Cheyletus malaccensis*	GBCH10759-13	KC507897
Trombidiformes	Cheyletidae	*Cheyletus malaccensis*	GBCH10760-13	KC507896
Trombidiformes	Cheyletidae	*Cheyletus malaccensis*	GBCH11580-13	JX436160
Trombidiformes	Cheyletidae	*Cheyletus malaccensis*	GBCH5762-13	HM854747
Trombidiformes	Cheyletidae		MYMCB152-11	JX837010
Trombidiformes	Cheyletidae		MYMCB157-11	JX835196
Trombidiformes	Cunaxidae		CHACA105-08	JX837389
Trombidiformes	Cunaxidae		CHACB1023-10	HM907156
Trombidiformes	Cunaxidae		CHACB1053-10	HM907178
Trombidiformes	Cunaxidae		CHACB1150-10	HM907237
Trombidiformes	Cunaxidae		CHACB138-10	HQ558422
Trombidiformes	Cunaxidae		CHACB356-10	HQ558536
Trombidiformes	Cunaxidae		CHACB455-10	HQ558605
Trombidiformes	Cunaxidae		CHACC051-10	HM907450
Trombidiformes	Cunaxidae		CHACC053-10	JX835459

Trombidiformes	Cunaxidae	CHACC054-10	HM907452
Trombidiformes	Cunaxidae	CNPAF888-13	KM824477
Trombidiformes	Cunaxidae	CNPPH1064-12	KJ166477
Trombidiformes	Cunaxidae	CNPPH1076-12	KJ443951
Trombidiformes	Cunaxidae	MIONB272-10	KP979186
Trombidiformes	Cunaxidae	MYMCA309-11	JX835794
Trombidiformes	Cunaxidae	MYMCA310-11	JX838642
Trombidiformes	Cunaxidae	MYMCA561-11	JX837203
Trombidiformes	Cunaxidae	MYMCA562-11	JX836227
Trombidiformes	Cunaxidae	MYMCA924-11	JX837963
Trombidiformes	Cunaxidae	MYMCB529-11	JX836506
Trombidiformes	Cunaxidae	MYMCB558-11	JX838370
Trombidiformes	Cunaxidae	MYMCB721-11	JX836798
Trombidiformes	Cunaxidae	MYMCB838-11	JX837304
Trombidiformes	Cunaxidae	MYMCB941-11	JX835515
Trombidiformes	Cunaxidae	MYMCB942-11	JX836095
Trombidiformes	Cunaxidae	MYMCC203-11	JX833867
Trombidiformes	Cunaxidae	MYMCC331-11	JX834714
Trombidiformes	Cunaxidae	MYMCC344-11	JX835141
Trombidiformes	Cunaxidae	MYMCC454-11	JX837339
Trombidiformes	Cunaxidae	MYMCC455-11	JX833733
Trombidiformes	Cunaxidae	MYMCC472-11	JX834857
Trombidiformes	Cunaxidae	MYMCC537-11	JX834653
Trombidiformes	Cunaxidae	MYMCC658-11	JX835493
Trombidiformes	Cunaxidae	MYMCC659-11	JX838658
Trombidiformes	Cunaxidae	MYMCC666-11	JX835264
Trombidiformes	Cunaxidae	MYMCC693-11	JX837074
Trombidiformes	Cunaxidae	MYMCC723-11	JX838722
Trombidiformes	Cunaxidae	MYMCC740-11	JX835200
Trombidiformes	Cunaxidae	MYMCD126-11	JX834994
Trombidiformes	Cunaxidae	MYMCD167-11	JX834489
Trombidiformes	Cunaxidae	MYMCE062-12	JX836978
Trombidiformes	Cunaxidae	MYMCE196-12	JX833908
Trombidiformes	Cunaxidae	MYMCE197-12	JX834848
Trombidiformes	Cunaxidae	MYMCE305-12	JX836010
Trombidiformes	Cunaxidae	MYMCE755-12	JX833734
Trombidiformes	Cunaxidae	MYMCE820-12	JX836891
Trombidiformes	Cunaxidae	MYMCF149-12	JX833790
Trombidiformes	Cunaxidae	MYMCF176-12	JX837556
Trombidiformes	Cunaxidae	MYMCF177-12	JX834306
Trombidiformes	Cunaxidae	MYMCF289-12	JX834674
Trombidiformes	Cunaxidae	MYMCF828-12	JX835177
Trombidiformes	Cunaxidae	MYMCF829-12	JX838675
Trombidiformes	Cunaxidae	MYMCF830-12	JX834747
Trombidiformes	Cunaxidae	MYMCF946-12	JX834458
Trombidiformes	Cunaxidae	MYMCG029-12	JX836652
Trombidiformes	Cunaxidae	MYMCG569-12	JX836390
Trombidiformes	Cunaxidae	SMTPB1856-13	KP979126
Trombidiformes	Cunaxidae	SMTPB1859-13	KR070400
Trombidiformes	Cunaxidae	SMTPB9981-13	KR069169
Trombidiformes	Cunaxidae	SSPAA2215-13	KM826575
Trombidiformes	Cunaxidae	SSWLF3572-13	KM837062
Trombidiformes	Ereynetidae	MYMCA742-11	JX837133

Trombidiformes	Ereynetidae		MYMCB788-11	JX834186
Trombidiformes	Ereynetidae		MYMCE290-12	JX836430
Trombidiformes	Ereynetidae		MYMCE379-12	JX835460
Trombidiformes	Ereynetidae		MYMCE411-12	JX834113
Trombidiformes	Ereynetidae		MYMCE414-12	JX835784
Trombidiformes	Ereynetidae		MYMCG293-12	JX837345
Trombidiformes	Ereynetidae		MYMCG640-12	JX838664
Trombidiformes	Ereynetidae		MYMCG641-12	JX838458
Trombidiformes	Ereynetidae		MYMCG645-12	JX836461
Trombidiformes	Ereynetidae		SSBAB104-12	KM831555
Trombidiformes	Eriophyidae	*Aceria eximia*	GBCH3757-09	EF409415
Trombidiformes	Eriophyidae	*Aceria tosichella*	GBCH12047-13	JF920090
Trombidiformes	Eriophyidae	*Aceria tosichella*	GBCH12056-13	JF920081
Trombidiformes	Eriophyidae	*Aceria tulipae*	GBCH12041-13	JF920096
Trombidiformes	Eriophyidae	*Aceria tulipae*	GBCH12042-13	JF920095
Trombidiformes	Eriophyidae	*Aceria tulipae*	GBCH1881-07	EF409416
Trombidiformes	Eriophyidae		EAWGA063-13	KR069325
Trombidiformes	Eriophyidae		EAWGA074-13	KR069272
Trombidiformes	Eriophyidae		EAWGA075-13	KR069545
Trombidiformes	Eriophyidae		EAWGA076-13	KR070094
Trombidiformes	Eriophyidae		EAWGA078-13	KR069618
Trombidiformes	Eriophyidae		EAWGA079-13	KR070280
Trombidiformes	Eriophyidae		EAWGA080-13	KR070124
Trombidiformes	Eriophyidae		EAWGA081-13	KR069683
Trombidiformes	Eriophyidae		EAWGA082-13	KR070194
Trombidiformes	Eriophyidae		EAWGA083-13	KR069189
Trombidiformes	Eriophyidae		EAWGA084-13	KR069344
Trombidiformes	Eriophyidae		MYMCE502-12	JX833708
Trombidiformes	Eriophyidae		MYMCE504-12	JX836814
Trombidiformes	Eriophyidae		MYMCE505-12	JX833876
Trombidiformes	Eriophyidae		MYMCE519-12	JX836644
Trombidiformes	Eriophyidae		MYMCE731-12	JX835648
Trombidiformes	Eriophyidae		MYMCE732-12	JX836212
Trombidiformes	Eriophyidae		MYMCE733-12	JX834574
Trombidiformes	Eriophyidae		MYMCE743-12	JX834049
Trombidiformes	Eriophyidae		SSJAF7350-13	KM840579
Trombidiformes	Eriophyidae		SSJAF7371-13	KM830432
Trombidiformes	Erythraeidae		MBIOF1628-13	KR069291
Trombidiformes	Erythraeidae		MBIOK588-14	KR069999
Trombidiformes	Erythraeidae		MBIOL533-14	KR070600
Trombidiformes	Erythraeidae		MBIOL551-14	KR069921
Trombidiformes	Erythraeidae	*Abrolophus*	CHACB635-10	JX838006
Trombidiformes	Erythraeidae	*Abrolophus*	CHACB671-10	JX837046
Trombidiformes	Erythraeidae	*Abrolophus*	CHACB928-10	HM907355
Trombidiformes	Erythraeidae	*Abrolophus*	CNRMA1044-12	KM825660
Trombidiformes	Erythraeidae	*Abrolophus*	CNRMC1512-12	KM836657
Trombidiformes	Erythraeidae	*Abrolophus*	CNRMD2665-12	KM833143
Trombidiformes	Erythraeidae	*Abrolophus*	JSJUN2329-12	KP979303
Trombidiformes	Erythraeidae	*Abrolophus*	MBIOD1150-13	KR069875
Trombidiformes	Erythraeidae	*Abrolophus*	MBIOD1189-13	KR069759
Trombidiformes	Erythraeidae	*Abrolophus*	MIAUS045-12	KR069212
Trombidiformes	Erythraeidae	*Abrolophus*	MIAUS057-12	KP979334
Trombidiformes	Erythraeidae	*Abrolophus*	MYMCA029-11	JX837190

Trombidiformes	Erythraeidae	*Abrolophus*	MYMCA1399-11	JX837186
Trombidiformes	Erythraeidae	*Abrolophus*	MYMCA293-11	JX836619
Trombidiformes	Erythraeidae	*Abrolophus*	MYMCA522-11	JX835825
Trombidiformes	Erythraeidae	*Abrolophus*	MYMCA594-11	JX833824
Trombidiformes	Erythraeidae	*Abrolophus*	MYMCA811-11	JX837076
Trombidiformes	Erythraeidae	*Abrolophus*	MYMCA929-11	JX837281
Trombidiformes	Erythraeidae	*Abrolophus*	MYMCA966-11	JX835181
Trombidiformes	Erythraeidae	*Abrolophus*	MYMCC022-11	JX838576
Trombidiformes	Erythraeidae	*Abrolophus*	MYMCC207-11	JX838636
Trombidiformes	Erythraeidae	*Abrolophus*	MYMCC425-11	JX838223
Trombidiformes	Erythraeidae	*Abrolophus*	MYMCC476-11	JX833624
Trombidiformes	Erythraeidae	*Abrolophus*	MYMCC915-11	JX835789
Trombidiformes	Erythraeidae	*Abrolophus*	MYMCC947-11	JX837729
Trombidiformes	Erythraeidae	*Abrolophus*	MYMCE084-12	JX838382
Trombidiformes	Erythraeidae	*Abrolophus*	MYMCE255-12	JX837555
Trombidiformes	Erythraeidae	*Abrolophus*	MYMCE327-12	JX837463
Trombidiformes	Erythraeidae	*Abrolophus*	MYMCE459-12	JX834493
Trombidiformes	Erythraeidae	*Abrolophus*	MYMCE643-12	JX834641
Trombidiformes	Erythraeidae	*Abrolophus*	MYMCE644-12	JX838351
Trombidiformes	Erythraeidae	*Abrolophus*	MYMCE760-12	JX838367
Trombidiformes	Erythraeidae	*Abrolophus*	MYMCF121-12	JX836640
Trombidiformes	Erythraeidae	*Abrolophus*	MYMCG647-12	JX836872
Trombidiformes	Erythraeidae	*Abrolophus*	RBINA4085-13	KP979281
Trombidiformes	Erythraeidae	*Abrolophus*	SMTPD2236-13	KR070672
Trombidiformes	Erythraeidae	*Abrolophus*	SSJAD3357-13	KM824154
Trombidiformes	Erythraeidae	*Abrolophus*	SSPAA6627-13	KM834548
Trombidiformes	Erythraeidae	*Abrolophus*	SSPAA6642-13	KM825366
Trombidiformes	Erythraeidae	*Abrolophus*	SSWLE2844-13	KM839199
Trombidiformes	Erythraeidae	*Abrolophus*	SSWLE2846-13	KM831739
Trombidiformes	Erythraeidae	*Abrolophus*	SSWLE463-13	KM839694
Trombidiformes	Erythraeidae	*Abrolophus*	SSWLE474-13	KM832147
Trombidiformes	Erythraeidae	*Abrolophus*	SSWLE479-13	KM830840
Trombidiformes	Erythraeidae	*Balaustium*	CNPAC504-13	KM837049
Trombidiformes	Erythraeidae	*Balaustium*	CNPAD870-13	KM837068
Trombidiformes	Erythraeidae	*Balaustium*	CNPAF923-13	KM828084
Trombidiformes	Erythraeidae	*Balaustium*	CNRMC1523-12	KM834353
Trombidiformes	Erythraeidae	*Balaustium*	CNRMF3206-12	KM830230
Trombidiformes	Erythraeidae	*Balaustium*	SSPAA6672-13	KM825549
Trombidiformes	Erythraeidae	*Erythraeus*	MYMCC532-11	JX836834
Trombidiformes	Erythraeidae	*Erythraeus*	MYMCC533-11	JX837954
Trombidiformes	Erythraeidae	*Leptus*	ARCN123-10	HQ924339
Trombidiformes	Erythraeidae	*Leptus*	CHACA1035-10	HM907088
Trombidiformes	Erythraeidae	*Leptus*	CHACA1049-10	HM907101
Trombidiformes	Erythraeidae	*Leptus*	CHACA1050-10	HM907102
Trombidiformes	Erythraeidae	*Leptus*	CHACA1051-10	HM907103
Trombidiformes	Erythraeidae	*Leptus*	CHACA1052-10	HM907104
Trombidiformes	Erythraeidae	*Leptus*	CHACA1053-10	HM907105
Trombidiformes	Erythraeidae	*Leptus*	CHACA1151-10	JX834325
Trombidiformes	Erythraeidae	*Leptus*	CHACA1152-10	JX835860
Trombidiformes	Erythraeidae	*Leptus*	CHACA1154-10	JX836289
Trombidiformes	Erythraeidae	*Leptus*	CHACA617-09	JX835439
Trombidiformes	Erythraeidae	*Leptus*	CHACB579-10	HQ558683
Trombidiformes	Erythraeidae	*Leptus*	CNBAB377-12	KM827356

Trombidiformes	Erythraeidae	*Leptus*		CNBAC733-12	KM829402
Trombidiformes	Erythraeidae	*Leptus*		CNBAC735-12	KM837201
Trombidiformes	Erythraeidae	*Leptus*		CNEIA2624-12	KM837517
Trombidiformes	Erythraeidae	*Leptus*		CNEIA2632-12	KM829930
Trombidiformes	Erythraeidae	*Leptus*		CNEIA2635-12	KM838044
Trombidiformes	Erythraeidae	*Leptus*		CNEIA2638-12	KM830807
Trombidiformes	Erythraeidae	*Leptus*		CNEIA2643-12	KM837039
Trombidiformes	Erythraeidae	*Leptus*		CNEIE1939-12	KM828751
Trombidiformes	Erythraeidae	*Leptus*		CNEIE1940-12	KM826397
Trombidiformes	Erythraeidae	*Leptus*		CNEIE1942-12	KM835474
Trombidiformes	Erythraeidae	*Leptus*		CNEIF2378-12	KM830391
Trombidiformes	Erythraeidae	*Leptus*		CNEIF2379-12	KM832782
Trombidiformes	Erythraeidae	*Leptus*		CNEIF2382-12	KM834022
Trombidiformes	Erythraeidae	*Leptus*		CNEIF2383-12	KM831810
Trombidiformes	Erythraeidae	*Leptus*		CNEIF2387-12	KM837317
Trombidiformes	Erythraeidae	*Leptus*		CNEIF2390-12	KM832726
Trombidiformes	Erythraeidae	*Leptus*		CNEIF2397-12	KM840606
Trombidiformes	Erythraeidae	*Leptus*		CNFDH631-14	KR069218
Trombidiformes	Erythraeidae	*Leptus*		CNFDH640-14	KR069356
Trombidiformes	Erythraeidae	*Leptus*		CNFDH654-14	KR070644
Trombidiformes	Erythraeidae	*Leptus*		CNFDH655-14	KR069736
Trombidiformes	Erythraeidae	*Leptus*		CNFDH693-14	KR069155
Trombidiformes	Erythraeidae	*Leptus*		CNFDH721-14	KR069243
Trombidiformes	Erythraeidae	*Leptus*		CNFDH725-14	KR069330
Trombidiformes	Erythraeidae	*Leptus*		CNFDH746-14	KR070260
Trombidiformes	Erythraeidae	*Leptus*		CNFDH765-14	KR069851
Trombidiformes	Erythraeidae	*Leptus*		CNFDH796-14	KR070468
Trombidiformes	Erythraeidae	*Leptus*		CNFDH798-14	KR070577
Trombidiformes	Erythraeidae	*Leptus*		CNFDH816-14	KR070251
Trombidiformes	Erythraeidae	*Leptus*		CNFDH827-14	KR070284
Trombidiformes	Erythraeidae	*Leptus*		CNGBG1732-14	KR069653
Trombidiformes	Erythraeidae	*Leptus*		CNGBG1737-14	KR069965
Trombidiformes	Erythraeidae	*Leptus*		CNGBG1747-14	KR069164
Trombidiformes	Erythraeidae	*Leptus*		CNGBG1781-14	KR070448
Trombidiformes	Erythraeidae	*Leptus*		CNGBG1856-14	KR070584
Trombidiformes	Erythraeidae	*Leptus*		CNGBG1885-14	KR070718
Trombidiformes	Erythraeidae	*Leptus*		CNGBG1927-14	KR069228
Trombidiformes	Erythraeidae	*Leptus*		CNGBH697-14	KR070465
Trombidiformes	Erythraeidae	*Leptus*		CNGBK090-14	KR070134
Trombidiformes	Erythraeidae	*Leptus*		CNGBL914-14	KR069719
Trombidiformes	Erythraeidae	*Leptus*		CNGBN2969-14	KR069772
Trombidiformes	Erythraeidae	*Leptus*		CNGBN3021-14	KR069465
Trombidiformes	Erythraeidae	*Leptus*		CNGBN3047-14	KR070338
Trombidiformes	Erythraeidae	*Leptus*		CNGBO1002-14	KR069586
Trombidiformes	Erythraeidae	*Leptus*		CNGBO991-14	KR070364
Trombidiformes	Erythraeidae	*Leptus*		CNGBO999-14	KR069826
Trombidiformes	Erythraeidae	*Leptus*		CNJAD1837-12	KM836643
Trombidiformes	Erythraeidae	*Leptus*		CNJAD1850-12	KM837987
Trombidiformes	Erythraeidae	*Leptus*		CNJAE1130-12	KM837715
Trombidiformes	Erythraeidae	*Leptus*		CNJAE1148-12	KM838348
Trombidiformes	Erythraeidae	*Leptus*		CNJAF1996-12	KM830704
Trombidiformes	Erythraeidae	*Leptus*		CNJAF2002-12	KM824965
Trombidiformes	Erythraeidae	*Leptus*		CNKJE2231-14	KR070073

Trombidiformes	Erythraeidae	*Leptus*	CNKJE2237-14	KR070436
Trombidiformes	Erythraeidae	*Leptus*	CNKJH908-14	KR069454
Trombidiformes	Erythraeidae	*Leptus*	CNKJH921-14	KR070214
Trombidiformes	Erythraeidae	*Leptus*	CNKJL1182-14	KR070575
Trombidiformes	Erythraeidae	*Leptus*	CNKJM2493-14	KR069284
Trombidiformes	Erythraeidae	*Leptus*	CNKJN843-14	KR070556
Trombidiformes	Erythraeidae	*Leptus*	CNKJP1038-14	KR070086
Trombidiformes	Erythraeidae	*Leptus*	CNKJP955-14	KR069238
Trombidiformes	Erythraeidae	*Leptus*	CNKOE1924-14	KR070632
Trombidiformes	Erythraeidae	*Leptus*	CNLMF1928-14	KR069345
Trombidiformes	Erythraeidae	*Leptus*	CNLMF1953-14	KR070549
Trombidiformes	Erythraeidae	*Leptus*	CNLMN1850-14	KR069712
Trombidiformes	Erythraeidae	*Leptus*	CNLMO2427-14	KR069935
Trombidiformes	Erythraeidae	*Leptus*	CNLMR1743-14	KR069346
Trombidiformes	Erythraeidae	*Leptus*	CNPAC532-13	KM826851
Trombidiformes	Erythraeidae	*Leptus*	CNPAC538-13	KM839770
Trombidiformes	Erythraeidae	*Leptus*	CNPAC539-13	KM836485
Trombidiformes	Erythraeidae	*Leptus*	CNPAC552-13	KM833431
Trombidiformes	Erythraeidae	*Leptus*	CNPAD552-13	KM824944
Trombidiformes	Erythraeidae	*Leptus*	CNPAE453-13	KM831008
Trombidiformes	Erythraeidae	*Leptus*	CNPAE454-13	KM840799
Trombidiformes	Erythraeidae	*Leptus*	CNPAE462-13	KM824292
Trombidiformes	Erythraeidae	*Leptus*	CNPAF860-13	KM836148
Trombidiformes	Erythraeidae	*Leptus*	CNPAF867-13	KM830276
Trombidiformes	Erythraeidae	*Leptus*	CNPAF873-13	KM836339
Trombidiformes	Erythraeidae	*Leptus*	CNPAF880-13	KM827660
Trombidiformes	Erythraeidae	*Leptus*	CNPAF881-13	KM835998
Trombidiformes	Erythraeidae	*Leptus*	CNPAF883-13	KM840157
Trombidiformes	Erythraeidae	*Leptus*	CNPAF884-13	KM825555
Trombidiformes	Erythraeidae	*Leptus*	CNPAF885-13	KM839743
Trombidiformes	Erythraeidae	*Leptus*	CNPAF886-13	KM833732
Trombidiformes	Erythraeidae	*Leptus*	CNPAF887-13	KM836040
Trombidiformes	Erythraeidae	*Leptus*	CNPAF889-13	KM824813
Trombidiformes	Erythraeidae	*Leptus*	CNPAF890-13	KM824025
Trombidiformes	Erythraeidae	*Leptus*	CNPAF892-13	KM836299
Trombidiformes	Erythraeidae	*Leptus*	CNPAF895-13	KM835946
Trombidiformes	Erythraeidae	*Leptus*	CNPAF896-13	KM826120
Trombidiformes	Erythraeidae	*Leptus*	CNPAF897-13	KM832489
Trombidiformes	Erythraeidae	*Leptus*	CNPAF898-13	KM838585
Trombidiformes	Erythraeidae	*Leptus*	CNPAF901-13	KM835411
Trombidiformes	Erythraeidae	*Leptus*	CNPAF904-13	KM827700
Trombidiformes	Erythraeidae	*Leptus*	CNPAF905-13	KM838739
Trombidiformes	Erythraeidae	*Leptus*	CNPAF906-13	KM824185
Trombidiformes	Erythraeidae	*Leptus*	CNPAF910-13	KM828066
Trombidiformes	Erythraeidae	*Leptus*	CNPAF911-13	KM835093
Trombidiformes	Erythraeidae	*Leptus*	CNPAF913-13	KM831420
Trombidiformes	Erythraeidae	*Leptus*	CNPAF922-13	KM826199
Trombidiformes	Erythraeidae	*Leptus*	CNPEE1880-14	KR069165
Trombidiformes	Erythraeidae	*Leptus*	CNPEE1881-14	KR070133
Trombidiformes	Erythraeidae	*Leptus*	CNPKG1166-14	KR069647
Trombidiformes	Erythraeidae	*Leptus*	CNPKG1312-14	KR069504
Trombidiformes	Erythraeidae	*Leptus*	CNPKG1336-14	KR070567
Trombidiformes	Erythraeidae	*Leptus*	CNPKH604-14	KR070223

Trombidiformes	Erythraeidae	*Leptus*	CNPKH658-14	KR069411
Trombidiformes	Erythraeidae	*Leptus*	CNPKI1396-14	KR069307
Trombidiformes	Erythraeidae	*Leptus*	CNPKI1405-14	KR069620
Trombidiformes	Erythraeidae	*Leptus*	CNPKI1411-14	KR070503
Trombidiformes	Erythraeidae	*Leptus*	CNPKI1455-14	KR070301
Trombidiformes	Erythraeidae	*Leptus*	CNPPC1937-12	KJ092146
Trombidiformes	Erythraeidae	*Leptus*	CNPPH1067-12	KJ444206
Trombidiformes	Erythraeidae	*Leptus*	CNRMC1509-12	KM826847
Trombidiformes	Erythraeidae	*Leptus*	CNRMC1511-12	KM840317
Trombidiformes	Erythraeidae	*Leptus*	CNRMC1513-12	KM825879
Trombidiformes	Erythraeidae	*Leptus*	CNRMC1515-12	KM830792
Trombidiformes	Erythraeidae	*Leptus*	CNRMC1525-12	KM838346
Trombidiformes	Erythraeidae	*Leptus*	CNRMC1526-12	KM832488
Trombidiformes	Erythraeidae	*Leptus*	CNRMC1532-12	KM836293
Trombidiformes	Erythraeidae	*Leptus*	CNRMC1533-12	KM825155
Trombidiformes	Erythraeidae	*Leptus*	CNRMC1643-12	KM835600
Trombidiformes	Erythraeidae	*Leptus*	CNRMC1644-12	KM839584
Trombidiformes	Erythraeidae	*Leptus*	CNRMC1648-12	KM840500
Trombidiformes	Erythraeidae	*Leptus*	CNRMC1652-12	KM840717
Trombidiformes	Erythraeidae	*Leptus*	CNRMC1653-12	KM829938
Trombidiformes	Erythraeidae	*Leptus*	CNRMC1661-12	KM836038
Trombidiformes	Erythraeidae	*Leptus*	CNRMC1663-12	KM836870
Trombidiformes	Erythraeidae	*Leptus*	CNRMC1673-12	KM827336
Trombidiformes	Erythraeidae	*Leptus*	CNRMC1677-12	KM838536
Trombidiformes	Erythraeidae	*Leptus*	CNRMC1684-12	KM838605
Trombidiformes	Erythraeidae	*Leptus*	CNRMD2681-12	KM834540
Trombidiformes	Erythraeidae	*Leptus*	CNRMD2696-12	KM840049
Trombidiformes	Erythraeidae	*Leptus*	CNRMD2697-12	KM839573
Trombidiformes	Erythraeidae	*Leptus*	CNRMD2701-12	KM828867
Trombidiformes	Erythraeidae	*Leptus*	CNRME4719-12	KM837612
Trombidiformes	Erythraeidae	*Leptus*	CNRME4743-12	KM836916
Trombidiformes	Erythraeidae	*Leptus*	CNRME4744-12	KM832603
Trombidiformes	Erythraeidae	*Leptus*	CNRME4752-12	KM830192
Trombidiformes	Erythraeidae	*Leptus*	CNRME4758-12	KM829305
Trombidiformes	Erythraeidae	*Leptus*	CNRME4760-12	KM837573
Trombidiformes	Erythraeidae	*Leptus*	CNRME4768-12	KM836973
Trombidiformes	Erythraeidae	*Leptus*	CNRME4781-12	KM835393
Trombidiformes	Erythraeidae	*Leptus*	CNRME4784-12	KM831331
Trombidiformes	Erythraeidae	*Leptus*	CNRME4787-12	KM829989
Trombidiformes	Erythraeidae	*Leptus*	CNRME4788-12	KM840761
Trombidiformes	Erythraeidae	*Leptus*	CNRMF3183-12	KM830026
Trombidiformes	Erythraeidae	*Leptus*	CNRMF3186-12	KM829130
Trombidiformes	Erythraeidae	*Leptus*	CNRMF3188-12	KM824713
Trombidiformes	Erythraeidae	*Leptus*	CNRMF3189-12	KM827883
Trombidiformes	Erythraeidae	*Leptus*	CNRMF3192-12	KM833816
Trombidiformes	Erythraeidae	*Leptus*	CNRMF3194-12	KM833199
Trombidiformes	Erythraeidae	*Leptus*	CNRMF3199-12	KM834415
Trombidiformes	Erythraeidae	*Leptus*	CNRMF3201-12	KM833389
Trombidiformes	Erythraeidae	*Leptus*	CNRMF3205-12	KM833939
Trombidiformes	Erythraeidae	*Leptus*	CNRMF3218-12	KM825185
Trombidiformes	Erythraeidae	*Leptus*	CNRMF3219-12	KM834200
Trombidiformes	Erythraeidae	*Leptus*	CNRMF3226-12	KM834418
Trombidiformes	Erythraeidae	*Leptus*	CNRMF3230-12	KM832789

Trombidiformes	Erythraeidae	*Leptus*	CNRMF3231-12	KM832406
Trombidiformes	Erythraeidae	*Leptus*	CNRMF3232-12	KM835096
Trombidiformes	Erythraeidae	*Leptus*	CNRMF3233-12	KM836955
Trombidiformes	Erythraeidae	*Leptus*	CNRMF3236-12	KM832369
Trombidiformes	Erythraeidae	*Leptus*	CNRMF3238-12	KM824127
Trombidiformes	Erythraeidae	*Leptus*	CNRMF3248-12	KM828785
Trombidiformes	Erythraeidae	*Leptus*	CNRMF3252-12	KM838618
Trombidiformes	Erythraeidae	*Leptus*	CNRMF3253-12	KM836893
Trombidiformes	Erythraeidae	*Leptus*	CNRMF3255-12	KM826679
Trombidiformes	Erythraeidae	*Leptus*	CNRMF3258-12	KM826200
Trombidiformes	Erythraeidae	*Leptus*	CNRMF3259-12	KM831143
Trombidiformes	Erythraeidae	*Leptus*	CNRMF3261-12	KM838233
Trombidiformes	Erythraeidae	*Leptus*	CNRMF3262-12	KM826699
Trombidiformes	Erythraeidae	*Leptus*	CNRMF3265-12	KM829563
Trombidiformes	Erythraeidae	*Leptus*	CNRMF3267-12	KM839938
Trombidiformes	Erythraeidae	*Leptus*	CNRMF3269-12	KM828267
Trombidiformes	Erythraeidae	*Leptus*	CNSLB478-12	KM824695
Trombidiformes	Erythraeidae	*Leptus*	CNSLB480-12	KM826443
Trombidiformes	Erythraeidae	*Leptus*	CNSLC558-12	KM828036
Trombidiformes	Erythraeidae	*Leptus*	CNSLD645-12	KM836414
Trombidiformes	Erythraeidae	*Leptus*	CNSLD646-12	KM834732
Trombidiformes	Erythraeidae	*Leptus*	CNSLD647-12	KM827637
Trombidiformes	Erythraeidae	*Leptus*	CNSLD655-12	KM840454
Trombidiformes	Erythraeidae	*Leptus*	CNSLI054-12	KM835577
Trombidiformes	Erythraeidae	*Leptus*	CNSLJ413-12	KM826069
Trombidiformes	Erythraeidae	*Leptus*	CNSLJ434-12	KM834433
Trombidiformes	Erythraeidae	*Leptus*	CNWBA490-13	KM826909
Trombidiformes	Erythraeidae	*Leptus*	CNWLD1148-13	KM824332
Trombidiformes	Erythraeidae	*Leptus*	CNWLE2567-13	KM837523
Trombidiformes	Erythraeidae	*Leptus*	CNWLE2586-13	KM829879
Trombidiformes	Erythraeidae	*Leptus*	CNWLE2594-13	KM826636
Trombidiformes	Erythraeidae	*Leptus*	CNWLF135-12	KM825448
Trombidiformes	Erythraeidae	*Leptus*	CNWLF139-12	KM828513
Trombidiformes	Erythraeidae	*Leptus*	CNWLF148-12	KM838690
Trombidiformes	Erythraeidae	*Leptus*	JSJUL2524-12	KR069901
Trombidiformes	Erythraeidae	*Leptus*	JSMAY1559-12	KP979270
Trombidiformes	Erythraeidae	*Leptus*	JSMAY1562-12	KR069821
Trombidiformes	Erythraeidae	*Leptus*	MIAUS031-12	KP979304
Trombidiformes	Erythraeidae	*Leptus*	MIAUS037-12	KP979279
Trombidiformes	Erythraeidae	*Leptus*	MIAUS046-12	KP979210
Trombidiformes	Erythraeidae	*Leptus*	MIAUS047-12	KR069934
Trombidiformes	Erythraeidae	*Leptus*	MIAUS058-12	KR069495
Trombidiformes	Erythraeidae	*Leptus*	MIONB411-10	KP979264
Trombidiformes	Erythraeidae	*Leptus*	MIONB412-10	KP979213
Trombidiformes	Erythraeidae	*Leptus*	MIONB516-11	KM832504
Trombidiformes	Erythraeidae	*Leptus*	MIONB519-11	KM833340
Trombidiformes	Erythraeidae	*Leptus*	MYMCA027-11	JX836425
Trombidiformes	Erythraeidae	*Leptus*	MYMCA836-11	JX838244
Trombidiformes	Erythraeidae	*Leptus*	MYMCC365-11	JX838454
Trombidiformes	Erythraeidae	*Leptus*	MYMCC395-11	JX836702
Trombidiformes	Erythraeidae	*Leptus*	MYMCC949-11	JX837407
Trombidiformes	Erythraeidae	*Leptus*	MYMCC950-11	JX835062
Trombidiformes	Erythraeidae	*Leptus*	MYMCE121-12	JX835688

Trombidiformes	Erythraeidae	*Leptus*	MYMCE212-12	KP979295
Trombidiformes	Erythraeidae	*Leptus*	MYMCE214-12	KR069180
Trombidiformes	Erythraeidae	*Leptus*	MYMCF287-12	JX835161
Trombidiformes	Erythraeidae	*Leptus*	MYMCG567-12	JX834987
Trombidiformes	Erythraeidae	*Leptus*	NCCE022-11	KP979123
Trombidiformes	Erythraeidae	*Leptus*	NCCE027-11	KP979246
Trombidiformes	Erythraeidae	*Leptus*	SSBAB1191-12	KM833707
Trombidiformes	Erythraeidae	*Leptus*	SSBAB2058-12	KM834048
Trombidiformes	Erythraeidae	*Leptus*	SSBAC3352-12	KM829398
Trombidiformes	Erythraeidae	*Leptus*	SSBAE3009-13	KM830428
Trombidiformes	Erythraeidae	*Leptus*	SSBAE3011-13	KM827159
Trombidiformes	Erythraeidae	*Leptus*	SSBAE3012-13	KM840029
Trombidiformes	Erythraeidae	*Leptus*	SSBAE3015-13	KM824509
Trombidiformes	Erythraeidae	*Leptus*	SSBAE3620-13	KM827153
Trombidiformes	Erythraeidae	*Leptus*	SSBAE3621-13	KM828039
Trombidiformes	Erythraeidae	*Leptus*	SSBAE3623-13	KM833192
Trombidiformes	Erythraeidae	*Leptus*	SSBAE3795-13	KM829808
Trombidiformes	Erythraeidae	*Leptus*	SSBAE934-13	KM832119
Trombidiformes	Erythraeidae	*Leptus*	SSBAE935-13	KM826883
Trombidiformes	Erythraeidae	*Leptus*	SSBAE937-13	KM827639
Trombidiformes	Erythraeidae	*Leptus*	SSBAE940-13	KM831750
Trombidiformes	Erythraeidae	*Leptus*	SSBAE941-13	KM827329
Trombidiformes	Erythraeidae	*Leptus*	SSBAE944-13	KM834080
Trombidiformes	Erythraeidae	*Leptus*	SSBAE945-13	KM828827
Trombidiformes	Erythraeidae	*Leptus*	SSBAE949-13	KM839220
Trombidiformes	Erythraeidae	*Leptus*	SSBAE950-13	KM832141
Trombidiformes	Erythraeidae	*Leptus*	SSBAE951-13	KM829151
Trombidiformes	Erythraeidae	*Leptus*	SSBAE952-13	KM836716
Trombidiformes	Erythraeidae	*Leptus*	SSBAE955-13	KM832690
Trombidiformes	Erythraeidae	*Leptus*	SSBAE958-13	KM839295
Trombidiformes	Erythraeidae	*Leptus*	SSEIA3008-13	KM826799
Trombidiformes	Erythraeidae	*Leptus*	SSEIA3020-13	KM835530
Trombidiformes	Erythraeidae	*Leptus*	SSEIA5513-13	KM828431
Trombidiformes	Erythraeidae	*Leptus*	SSEIA7684-13	KM825751
Trombidiformes	Erythraeidae	*Leptus*	SSEIB7649-13	KM827213
Trombidiformes	Erythraeidae	*Leptus*	SSEIB7677-13	KM839592
Trombidiformes	Erythraeidae	*Leptus*	SSJAB1890-13	KM830224
Trombidiformes	Erythraeidae	*Leptus*	SSJAB3343-13	KM829578
Trombidiformes	Erythraeidae	*Leptus*	SSJAC1535-13	KM831456
Trombidiformes	Erythraeidae	*Leptus*	SSJAC983-13	KM839270
Trombidiformes	Erythraeidae	*Leptus*	SSJAF2459-13	KM832462
Trombidiformes	Erythraeidae	*Leptus*	SSJAF5573-13	KM826128
Trombidiformes	Erythraeidae	*Leptus*	SSJAF5587-13	KM829119
Trombidiformes	Erythraeidae	*Leptus*	SSJAF5622-13	KM836819
Trombidiformes	Erythraeidae	*Leptus*	SSJAF7400-13	KM828998
Trombidiformes	Erythraeidae	*Leptus*	SSJAF8220-13	KR069968
Trombidiformes	Erythraeidae	*Leptus*	SSJAF8379-13	KM824912
Trombidiformes	Erythraeidae	*Leptus*	SSPAA2213-13	KM831283
Trombidiformes	Erythraeidae	*Leptus*	SSPAA2216-13	KM827626
Trombidiformes	Erythraeidae	*Leptus*	SSPAA2218-13	KM836922
Trombidiformes	Erythraeidae	*Leptus*	SSPAA2227-13	KM830176
Trombidiformes	Erythraeidae	*Leptus*	SSPAA2228-13	KM830809
Trombidiformes	Erythraeidae	*Leptus*	SSPAA2240-13	KM834593

Trombidiformes	Erythraeidae	*Leptus*	SSPAA2244-13	KM834363
Trombidiformes	Erythraeidae	*Leptus*	SSPAA2275-13	KM824395
Trombidiformes	Erythraeidae	*Leptus*	SSPAA2282-13	KM825211
Trombidiformes	Erythraeidae	*Leptus*	SSPAA2291-13	KM831784
Trombidiformes	Erythraeidae	*Leptus*	SSPAA2295-13	KM839231
Trombidiformes	Erythraeidae	*Leptus*	SSPAA6665-13	KM834450
Trombidiformes	Erythraeidae	*Leptus*	SSPAA6667-13	KM838313
Trombidiformes	Erythraeidae	*Leptus*	SSPAA6669-13	KM838801
Trombidiformes	Erythraeidae	*Leptus*	SSPAA6671-13	KM836020
Trombidiformes	Erythraeidae	*Leptus*	SSPAA6677-13	KM831079
Trombidiformes	Erythraeidae	*Leptus*	SSPAA6686-13	KM824072
Trombidiformes	Erythraeidae	*Leptus*	SSPAA6687-13	KM825020
Trombidiformes	Erythraeidae	*Leptus*	SSPAA6692-13	KM828207
Trombidiformes	Erythraeidae	*Leptus*	SSPAA6693-13	KM831164
Trombidiformes	Erythraeidae	*Leptus*	SSPAA6710-13	KM833926
Trombidiformes	Erythraeidae	*Leptus*	SSPAA7748-13	KM828072
Trombidiformes	Erythraeidae	*Leptus*	SSPAA7749-13	KM838365
Trombidiformes	Erythraeidae	*Leptus*	SSPAA7750-13	KM835887
Trombidiformes	Erythraeidae	*Leptus*	SSPAA7751-13	KM836012
Trombidiformes	Erythraeidae	*Leptus*	SSPAA7752-13	KM833699
Trombidiformes	Erythraeidae	*Leptus*	SSPAA7755-13	KM827973
Trombidiformes	Erythraeidae	*Leptus*	SSPAA7756-13	KM827397
Trombidiformes	Erythraeidae	*Leptus*	SSPAA7757-13	KM831738
Trombidiformes	Erythraeidae	*Leptus*	SSPAA7762-13	KM825436
Trombidiformes	Erythraeidae	*Leptus*	SSPAA7764-13	KM834584
Trombidiformes	Erythraeidae	*Leptus*	SSPAA7769-13	KM836596
Trombidiformes	Erythraeidae	*Leptus*	SSPAA7772-13	KM823978
Trombidiformes	Erythraeidae	*Leptus*	SSPAA7773-13	KM828618
Trombidiformes	Erythraeidae	*Leptus*	SSPAA7775-13	KM831231
Trombidiformes	Erythraeidae	*Leptus*	SSPAA7777-13	KM825323
Trombidiformes	Erythraeidae	*Leptus*	SSPAA7781-13	KM832820
Trombidiformes	Erythraeidae	*Leptus*	SSPAA7782-13	KM837343
Trombidiformes	Erythraeidae	*Leptus*	SSPAA7787-13	KM836065
Trombidiformes	Erythraeidae	*Leptus*	SSPAA7814-13	KM826060
Trombidiformes	Erythraeidae	*Leptus*	SSPAA7832-13	KM831511
Trombidiformes	Erythraeidae	*Leptus*	SSPAA7933-13	KM832794
Trombidiformes	Erythraeidae	*Leptus*	SSPAA7936-13	KM829779
Trombidiformes	Erythraeidae	*Leptus*	SSPAA7942-13	KM831836
Trombidiformes	Erythraeidae	*Leptus*	SSPAA7951-13	KM834347
Trombidiformes	Erythraeidae	*Leptus*	SSPAA7953-13	KM833169
Trombidiformes	Erythraeidae	*Leptus*	SSPAA7957-13	KM834475
Trombidiformes	Erythraeidae	*Leptus*	SSPAA7971-13	KM834697
Trombidiformes	Erythraeidae	*Leptus*	SSPAA7978-13	KM828246
Trombidiformes	Erythraeidae	*Leptus*	SSPAA7979-13	KM833930
Trombidiformes	Erythraeidae	*Leptus*	SSPAA7986-13	KM839988
Trombidiformes	Erythraeidae	*Leptus*	SSPAA7987-13	KM828298
Trombidiformes	Erythraeidae	*Leptus*	SSPAB3163-13	KM836957
Trombidiformes	Erythraeidae	*Leptus*	SSPAB3165-13	KM836815
Trombidiformes	Erythraeidae	*Leptus*	SSPAB3178-13	KM835686
Trombidiformes	Erythraeidae	*Leptus*	SSPAB384-13	KM832548
Trombidiformes	Erythraeidae	*Leptus*	SSPAB7533-13	KM835936
Trombidiformes	Erythraeidae	*Leptus*	SSPAB8697-13	KM835697
Trombidiformes	Erythraeidae	*Leptus*	SSPAB8729-13	KM825084

Trombidiformes	Erythraeidae	*Leptus*		SSPAB953-13	KM835191
Trombidiformes	Erythraeidae	*Leptus*		SSPAB954-13	KM837752
Trombidiformes	Erythraeidae	*Leptus*		SSPAB971-13	KM824982
Trombidiformes	Erythraeidae	*Leptus*		SSPAC2354-13	KM838888
Trombidiformes	Erythraeidae	*Leptus*		SSPAC7400-13	KM831655
Trombidiformes	Erythraeidae	*Leptus*		SSPAC7825-13	KM829557
Trombidiformes	Erythraeidae	*Leptus*		SSPAC7826-13	KM826282
Trombidiformes	Erythraeidae	*Leptus*		SSPAC7851-13	KM826161
Trombidiformes	Erythraeidae	*Leptus*		SSPAC7891-13	KM825485
Trombidiformes	Erythraeidae	*Leptus*		SSWLA038-13	KM835379
Trombidiformes	Erythraeidae	*Leptus*		SSWLC3128-13	KM834965
Trombidiformes	Erythraeidae	*Leptus*		SSWLE2865-13	KM839125
Trombidiformes	Erythraeidae	*Leptus*		SSWLE2873-13	KM835982
Trombidiformes	Erythraeidae	*Leptus*		SSWLE2883-13	KM827389
Trombidiformes	Erythraeidae	*Leptus*		SSWLE3149-13	KM830582
Trombidiformes	Erythraeidae	*Leptus*		SSWLE3154-13	KM832788
Trombidiformes	Erythraeidae	*Leptus*		SSWLE3189-13	KM835274
Trombidiformes	Erythraeidae	*Leptus*		SSWLE3193-13	KM834705
Trombidiformes	Erythraeidae	*Leptus*		SSWLE482-13	KM833674
Trombidiformes	Erythraeidae			ARCN022-10	HQ924266
Trombidiformes	Erythraeidae			ARCN024-10	HQ924268
Trombidiformes	Erythraeidae			ARCN025-10	HQ924269
Trombidiformes	Erythraeidae			ARCN027-10	HQ924270
Trombidiformes	Erythraeidae			ARCN034-10	HQ924277
Trombidiformes	Erythraeidae			ARCN035-10	HQ924278
Trombidiformes	Erythraeidae			ARCN047-10	HQ924281
Trombidiformes	Erythraeidae			ARCN048-10	KM827995
Trombidiformes	Erythraeidae			ARCN049-10	HQ924282
Trombidiformes	Erythraeidae			ARCN050-10	HQ924283
Trombidiformes	Erythraeidae			ARCN051-10	HQ924284
Trombidiformes	Erythraeidae			ARCN052-10	HQ924285
Trombidiformes	Erythraeidae			ARCN053-10	HQ924286
Trombidiformes	Erythraeidae			ARCN064-10	HQ924287
Trombidiformes	Erythraeidae			ARCN075-10	HQ924298
Trombidiformes	Erythraeidae			ARCN076-10	HQ924299
Trombidiformes	Erythraeidae			ARCN078-10	HQ924300
Trombidiformes	Erythraeidae			ARCN079-10	HQ924301
Trombidiformes	Erythraeidae			ARCN080-10	HQ924302
Trombidiformes	Erythraeidae			ARCN081-10	HQ924303
Trombidiformes	Erythraeidae			ARCN082-10	KM827561
Trombidiformes	Erythraeidae			ARCN083-10	HQ924304
Trombidiformes	Erythraeidae			ARCN097-10	HQ924315
Trombidiformes	Erythraeidae			ARCN098-10	HQ924316
Trombidiformes	Erythraeidae			ARCN099-10	HQ924317
Trombidiformes	Erythraeidae			ARCN100-10	HQ924318
Trombidiformes	Erythraeidae			ARCN101-10	HQ924319
Trombidiformes	Erythraeidae			ARCN102-10	HQ924320
Trombidiformes	Erythraeidae			ARCN103-10	HQ924321
Trombidiformes	Erythraeidae			ARCN104-10	HQ924322
Trombidiformes	Erythraeidae			ARCN105-10	HQ924323
Trombidiformes	Erythraeidae			ARCN106-10	HQ924324
Trombidiformes	Erythraeidae			ARCN107-10	HQ924325
Trombidiformes	Erythraeidae			ARCN108-10	HQ924326

Trombidiformes	Erythraeidae	ARCN109-10	HQ924327
Trombidiformes	Erythraeidae	ARCN110-10	HQ924328
Trombidiformes	Erythraeidae	ARCN111-10	HQ924329
Trombidiformes	Erythraeidae	ARCN112-10	KM827617
Trombidiformes	Erythraeidae	ARCN113-10	HQ924330
Trombidiformes	Erythraeidae	ARCN114-10	HQ924331
Trombidiformes	Erythraeidae	ARCN115-10	HQ924332
Trombidiformes	Erythraeidae	ARCN116-10	HQ924333
Trombidiformes	Erythraeidae	ARCN117-10	HQ924334
Trombidiformes	Erythraeidae	ARCN119-10	HQ924336
Trombidiformes	Erythraeidae	ARCN120-10	HQ924337
Trombidiformes	Erythraeidae	ARCN122-10	HQ924338
Trombidiformes	Erythraeidae	ARCN124-10	HQ924340
Trombidiformes	Erythraeidae	ARCN125-10	HQ924341
Trombidiformes	Erythraeidae	ARCN126-10	KM837234
Trombidiformes	Erythraeidae	ARCN127-10	HQ924342
Trombidiformes	Erythraeidae	ARCN128-10	HQ924343
Trombidiformes	Erythraeidae	ARCN129-10	HQ924344
Trombidiformes	Erythraeidae	ARCN130-10	HQ924345
Trombidiformes	Erythraeidae	ARCN131-10	HQ924346
Trombidiformes	Erythraeidae	ARCN145-10	HQ924352
Trombidiformes	Erythraeidae	ARCN146-10	HQ924353
Trombidiformes	Erythraeidae	ARCN148-10	HQ924354
Trombidiformes	Erythraeidae	ARCN149-10	HQ924355
Trombidiformes	Erythraeidae	ARCN150-10	HQ924356
Trombidiformes	Erythraeidae	ARCN151-10	HQ924357
Trombidiformes	Erythraeidae	ARCN152-10	HQ924358
Trombidiformes	Erythraeidae	ARCN153-10	HQ924359
Trombidiformes	Erythraeidae	ARCN154-10	HQ924360
Trombidiformes	Erythraeidae	ARCN155-10	HQ924361
Trombidiformes	Erythraeidae	ARCN156-10	KM824515
Trombidiformes	Erythraeidae	ARCN166-10	HQ924368
Trombidiformes	Erythraeidae	ARCN167-10	HQ924369
Trombidiformes	Erythraeidae	ARCN168-10	HQ924370
Trombidiformes	Erythraeidae	ARCN177-10	KM829521
Trombidiformes	Erythraeidae	ASAMT049-12	KP979192
Trombidiformes	Erythraeidae	ASAMT050-12	KR069952
Trombidiformes	Erythraeidae	CHACA1130-10	JX835002
Trombidiformes	Erythraeidae	CHACA1131-10	JX836372
Trombidiformes	Erythraeidae	CHACA1132-10	JX837639
Trombidiformes	Erythraeidae	CHACA1133-10	JX834751
Trombidiformes	Erythraeidae	CHACA1134-10	JX837599
Trombidiformes	Erythraeidae	CHACA1195-10	JX838373
Trombidiformes	Erythraeidae	CHACA550-09	JX834839
Trombidiformes	Erythraeidae	CHACA551-09	JX836637
Trombidiformes	Erythraeidae	CHACA555-09	JX833818
Trombidiformes	Erythraeidae	CHACA556-09	JX836334
Trombidiformes	Erythraeidae	CHACA557-09	JX836958
Trombidiformes	Erythraeidae	CHACA561-09	KR069405
Trombidiformes	Erythraeidae	CHACA563-09	JX836051
Trombidiformes	Erythraeidae	CHACA566-09	JX835519
Trombidiformes	Erythraeidae	CHACA614-09	JX834834
Trombidiformes	Erythraeidae	CHACB230-10	HQ941471

Trombidiformes	Erythraeidae	CHACB231-10	HQ558481
Trombidiformes	Erythraeidae	CHACB232-10	HQ558482
Trombidiformes	Erythraeidae	CHACB233-10	HQ558483
Trombidiformes	Erythraeidae	CHACB234-10	HQ558484
Trombidiformes	Erythraeidae	CHACB235-10	HQ558485
Trombidiformes	Erythraeidae	CHACB236-10	HQ558486
Trombidiformes	Erythraeidae	CHACB969-10	HM907383
Trombidiformes	Erythraeidae	CHACB991-10	HM907397
Trombidiformes	Erythraeidae	CHACB995-10	HM907400
Trombidiformes	Erythraeidae	CNBAD677-12	KM828663
Trombidiformes	Erythraeidae	CNBAK368-13	KM832065
Trombidiformes	Erythraeidae	CNBAK386-13	KM828601
Trombidiformes	Erythraeidae	CNBPF124-12	KM836887
Trombidiformes	Erythraeidae	CNEIA2568-12	KM837489
Trombidiformes	Erythraeidae	CNEIA2573-12	KM824315
Trombidiformes	Erythraeidae	CNEIA2576-12	KM837717
Trombidiformes	Erythraeidae	CNEIA2584-12	KM833380
Trombidiformes	Erythraeidae	CNEIA2587-12	KM825159
Trombidiformes	Erythraeidae	CNEIA2588-12	KM835259
Trombidiformes	Erythraeidae	CNEIA2594-12	KM834024
Trombidiformes	Erythraeidae	CNEIA2598-12	KM835034
Trombidiformes	Erythraeidae	CNEIA2610-12	KM828849
Trombidiformes	Erythraeidae	CNEIA2628-12	KM829028
Trombidiformes	Erythraeidae	CNEIF2381-12	KM830747
Trombidiformes	Erythraeidae	CNEIH067-13	KM837588
Trombidiformes	Erythraeidae	CNJAB1019-12	KM829269
Trombidiformes	Erythraeidae	CNJAB998-12	KM839749
Trombidiformes	Erythraeidae	CNJAC1108-12	KM838930
Trombidiformes	Erythraeidae	CNJAC1440-12	KM828889
Trombidiformes	Erythraeidae	CNJAC1576-12	KM835325
Trombidiformes	Erythraeidae	CNPAD871-13	KM824157
Trombidiformes	Erythraeidae	CNPAF987-13	KM832772
Trombidiformes	Erythraeidae	CNPAF988-13	KM838868
Trombidiformes	Erythraeidae	CNPAF989-13	KM836469
Trombidiformes	Erythraeidae	CNPAF990-13	KM839262
Trombidiformes	Erythraeidae	CNPAK428-13	KM835268
Trombidiformes	Erythraeidae	CNPCF124-13	KM831681
Trombidiformes	Erythraeidae	CNPCF129-13	KM830399
Trombidiformes	Erythraeidae	CNPKJ3116-14	KR069615
Trombidiformes	Erythraeidae	CNPPB2362-12	KJ089243
Trombidiformes	Erythraeidae	CNPPC1935-12	KJ090430
Trombidiformes	Erythraeidae	CNPPC1939-12	KJ090671
Trombidiformes	Erythraeidae	CNPPC1960-12	KJ085076
Trombidiformes	Erythraeidae	CNPPD2644-12	KJ089103
Trombidiformes	Erythraeidae	CNPPD2646-12	KJ084235
Trombidiformes	Erythraeidae	CNPPD2647-12	KJ087420
Trombidiformes	Erythraeidae	CNPPD2648-12	KJ093073
Trombidiformes	Erythraeidae	CNPPD2649-12	KJ088351
Trombidiformes	Erythraeidae	CNPPD2650-12	KJ091254
Trombidiformes	Erythraeidae	CNPPD2665-12	KJ088741
Trombidiformes	Erythraeidae	CNPPE1432-12	KJ166931
Trombidiformes	Erythraeidae	CNPPE2136-12	KJ085509
Trombidiformes	Erythraeidae	CNPPF1030-12	KJ164468

Trombidiformes	Erythraeidae	CNPPF587-12	KJ163649
Trombidiformes	Erythraeidae	CNPPH1073-12	KJ165007
Trombidiformes	Erythraeidae	CNPPI1251-12	KJ445377
Trombidiformes	Erythraeidae	CNPPJ1839-12	KJ207998
Trombidiformes	Erythraeidae	CNRMD2695-12	KM837776
Trombidiformes	Erythraeidae	CNRME4725-12	KM839331
Trombidiformes	Erythraeidae	CNRME4738-12	KM832365
Trombidiformes	Erythraeidae	CNRME4742-12	KM830672
Trombidiformes	Erythraeidae	CNRME4777-12	KM837894
Trombidiformes	Erythraeidae	CNRMF3180-12	KM825193
Trombidiformes	Erythraeidae	CNRMF3198-12	KM835534
Trombidiformes	Erythraeidae	CNRMF3203-12	KM835523
Trombidiformes	Erythraeidae	CNRMF3221-12	KM838157
Trombidiformes	Erythraeidae	CNRMF3241-12	KM828058
Trombidiformes	Erythraeidae	CNRMF3246-12	KM834768
Trombidiformes	Erythraeidae	CNRMF3254-12	KM828814
Trombidiformes	Erythraeidae	CNRMF3266-12	KM836879
Trombidiformes	Erythraeidae	CNSLC553-12	KM835341
Trombidiformes	Erythraeidae	CNSLC556-12	KM839401
Trombidiformes	Erythraeidae	CNSLC559-12	KM828037
Trombidiformes	Erythraeidae	CNSLC561-12	KM834038
Trombidiformes	Erythraeidae	CNSLD648-12	KM825290
Trombidiformes	Erythraeidae	CNSLD649-12	KM824299
Trombidiformes	Erythraeidae	CNSLD650-12	KM828150
Trombidiformes	Erythraeidae	CNSLD651-12	KM825005
Trombidiformes	Erythraeidae	CNSLD653-12	KM840511
Trombidiformes	Erythraeidae	CNSLD668-12	KM831306
Trombidiformes	Erythraeidae	CNSLD670-12	KM827638
Trombidiformes	Erythraeidae	CNSLD672-12	KM828528
Trombidiformes	Erythraeidae	CNSLD674-12	KM838321
Trombidiformes	Erythraeidae	CNSLD677-12	KM824607
Trombidiformes	Erythraeidae	CNSLD678-12	KM829576
Trombidiformes	Erythraeidae	CNSLD679-12	KM838408
Trombidiformes	Erythraeidae	CNSLD684-12	KM830091
Trombidiformes	Erythraeidae	CNSLE492-12	KM832909
Trombidiformes	Erythraeidae	CNSLE493-12	KM830675
Trombidiformes	Erythraeidae	CNSLE501-12	KM825451
Trombidiformes	Erythraeidae	CNSLE502-12	KM833769
Trombidiformes	Erythraeidae	CNSLE508-12	KM830405
Trombidiformes	Erythraeidae	CNSLF646-12	KM837813
Trombidiformes	Erythraeidae	CNSLG116-12	KM840546
Trombidiformes	Erythraeidae	CNSLG117-12	KM827301
Trombidiformes	Erythraeidae	CNSLG118-12	KM826358
Trombidiformes	Erythraeidae	CNSLG119-12	KM834604
Trombidiformes	Erythraeidae	CNSLG120-12	KM838165
Trombidiformes	Erythraeidae	CNSLG121-12	KM833490
Trombidiformes	Erythraeidae	CNSLG122-12	KM826239
Trombidiformes	Erythraeidae	CNSLG124-12	KM826234
Trombidiformes	Erythraeidae	CNSLG125-12	KM828050
Trombidiformes	Erythraeidae	CNSLH085-12	KM837795
Trombidiformes	Erythraeidae	CNSLH088-12	KM835790
Trombidiformes	Erythraeidae	CNSLI050-12	KM836719
Trombidiformes	Erythraeidae	CNSLI051-12	KM828899

Trombidiformes	Erythraeidae	CNSLI057-12	KM831926
Trombidiformes	Erythraeidae	CNSLI059-12	KM840745
Trombidiformes	Erythraeidae	CNSLI063-12	KM830888
Trombidiformes	Erythraeidae	CNSLJ029-12	KM828012
Trombidiformes	Erythraeidae	CNSLJ433-12	KM834639
Trombidiformes	Erythraeidae	CNSLJ435-12	KM825904
Trombidiformes	Erythraeidae	CNSLJ438-12	KM827166
Trombidiformes	Erythraeidae	CNWBA488-13	KM836820
Trombidiformes	Erythraeidae	CNWBA491-13	KM828868
Trombidiformes	Erythraeidae	CNWBA492-13	KM835455
Trombidiformes	Erythraeidae	CNWBB108-13	KM830028
Trombidiformes	Erythraeidae	CNWBB109-13	KM830732
Trombidiformes	Erythraeidae	CNWBB110-13	KM834621
Trombidiformes	Erythraeidae	CNWBB111-13	KM839797
Trombidiformes	Erythraeidae	CNWBB112-13	KM836404
Trombidiformes	Erythraeidae	CNWBB113-13	KM835039
Trombidiformes	Erythraeidae	CNWBB114-13	KM829861
Trombidiformes	Erythraeidae	CNWBB115-13	KM825027
Trombidiformes	Erythraeidae	CNWBB116-13	KM827968
Trombidiformes	Erythraeidae	CNWLB765-13	KM830325
Trombidiformes	Erythraeidae	CNWLD1152-13	KM836660
Trombidiformes	Erythraeidae	CNWLD1157-13	KM832750
Trombidiformes	Erythraeidae	CNWLE2568-13	KM829917
Trombidiformes	Erythraeidae	CNWLE2569-13	KM839489
Trombidiformes	Erythraeidae	CNWLE2570-13	KM824086
Trombidiformes	Erythraeidae	CNWLE2572-13	KM828015
Trombidiformes	Erythraeidae	CNWLE2573-13	KM828995
Trombidiformes	Erythraeidae	CNWLE2575-13	KM829338
Trombidiformes	Erythraeidae	CNWLE2580-13	KM838227
Trombidiformes	Erythraeidae	CNWLE2582-13	KM828132
Trombidiformes	Erythraeidae	CNWLF127-12	KM831229
Trombidiformes	Erythraeidae	CNWLF130-12	KM830258
Trombidiformes	Erythraeidae	CNWLF133-12	KM830110
Trombidiformes	Erythraeidae	CNWLF137-12	KM831597
Trombidiformes	Erythraeidae	CNWLF141-12	KM825396
Trombidiformes	Erythraeidae	CNWLF143-12	KM825267
Trombidiformes	Erythraeidae	CNWLF144-12	KM833475
Trombidiformes	Erythraeidae	CNWLF145-12	KM825015
Trombidiformes	Erythraeidae	CNWLF147-12	KM830707
Trombidiformes	Erythraeidae	CNWLF150-12	KM831173
Trombidiformes	Erythraeidae	CNWLF151-12	KM829601
Trombidiformes	Erythraeidae	CNWLF155-12	KM829692
Trombidiformes	Erythraeidae	CNWLF157-12	KM829995
Trombidiformes	Erythraeidae	CNWLF161-12	KM838086
Trombidiformes	Erythraeidae	CNWLF162-12	KM828370
Trombidiformes	Erythraeidae	CNWLF167-12	KM835881
Trombidiformes	Erythraeidae	CNWLF169-12	KM838200
Trombidiformes	Erythraeidae	CNWLF174-12	KM835954
Trombidiformes	Erythraeidae	CNWLF175-12	KM832308
Trombidiformes	Erythraeidae	CNWLF177-12	KM836436
Trombidiformes	Erythraeidae	CNWLF180-12	KM839005
Trombidiformes	Erythraeidae	CNWLG807-12	KM833975
Trombidiformes	Erythraeidae	CNWLG808-12	KM836437

Trombidiformes	Erythraeidae	CNWLG809-12	KM836989
Trombidiformes	Erythraeidae	CNWLG811-12	KM833890
Trombidiformes	Erythraeidae	CNWLG813-12	KM828727
Trombidiformes	Erythraeidae	CNWLG817-12	KM824024
Trombidiformes	Erythraeidae	CNWLG818-12	KM830757
Trombidiformes	Erythraeidae	CNWLG823-12	KM838469
Trombidiformes	Erythraeidae	CNWLG824-12	KM826354
Trombidiformes	Erythraeidae	CNWLG826-12	KM838035
Trombidiformes	Erythraeidae	CNWLG827-12	KM840403
Trombidiformes	Erythraeidae	CNWLG828-12	KM832619
Trombidiformes	Erythraeidae	CNWLG829-12	KM834802
Trombidiformes	Erythraeidae	CNWLG832-12	KM827147
Trombidiformes	Erythraeidae	CNWLG833-12	KM827161
Trombidiformes	Erythraeidae	CNWLG834-12	KM826151
Trombidiformes	Erythraeidae	CNWLG835-12	KM825341
Trombidiformes	Erythraeidae	CNWLG839-12	KM828620
Trombidiformes	Erythraeidae	CNWLG840-12	KM834141
Trombidiformes	Erythraeidae	CNWLG841-12	KM837976
Trombidiformes	Erythraeidae	CNWLG844-12	KM838511
Trombidiformes	Erythraeidae	CNWLG846-12	KM840044
Trombidiformes	Erythraeidae	CNWLG851-12	KM828860
Trombidiformes	Erythraeidae	CNWLG856-12	KM836795
Trombidiformes	Erythraeidae	CNWLG857-12	KM838896
Trombidiformes	Erythraeidae	CNWLG858-12	KM836754
Trombidiformes	Erythraeidae	CNWLG860-12	KM826461
Trombidiformes	Erythraeidae	CNWLH194-12	KM827517
Trombidiformes	Erythraeidae	CNWLH197-12	KM838804
Trombidiformes	Erythraeidae	CNWLH198-12	KM830085
Trombidiformes	Erythraeidae	CNWLH199-12	KM833500
Trombidiformes	Erythraeidae	CNWLH201-12	KM828940
Trombidiformes	Erythraeidae	CNWLH211-12	KM830632
Trombidiformes	Erythraeidae	CNWLH222-12	KM840133
Trombidiformes	Erythraeidae	CNWLH315-12	KM824880
Trombidiformes	Erythraeidae	CNWLH319-12	KM830678
Trombidiformes	Erythraeidae	CNWLH320-12	KM835408
Trombidiformes	Erythraeidae	CNWLH329-12	KM827463
Trombidiformes	Erythraeidae	CNWLH332-12	KM831705
Trombidiformes	Erythraeidae	CNWLH338-12	KM834400
Trombidiformes	Erythraeidae	JSAUG1781-12	KR069585
Trombidiformes	Erythraeidae	JSAUG1783-12	KR070131
Trombidiformes	Erythraeidae	JSAUG1787-12	KR069812
Trombidiformes	Erythraeidae	JSAUG1788-12	KR069455
Trombidiformes	Erythraeidae	JSAUG1791-12	KR069366
Trombidiformes	Erythraeidae	JSAUG1792-12	KR070607
Trombidiformes	Erythraeidae	JSAUG1795-12	KR070425
Trombidiformes	Erythraeidae	JSAUG1803-12	KR069471
Trombidiformes	Erythraeidae	JSAUG1806-12	KR069782
Trombidiformes	Erythraeidae	JSAUG1808-12	KR069614
Trombidiformes	Erythraeidae	JSAUG1813-12	KR070334
Trombidiformes	Erythraeidae	JSAUG1814-12	KR069425
Trombidiformes	Erythraeidae	JSAUG1818-12	KR070623
Trombidiformes	Erythraeidae	JSAUG1819-12	KR069531
Trombidiformes	Erythraeidae	JSAUG1825-12	KR069225

Trombidiformes	Erythraeidae	JSAUG1826-12	KR070553
Trombidiformes	Erythraeidae	JSAUG1827-12	KP979231
Trombidiformes	Erythraeidae	JSAUG1830-12	KR070108
Trombidiformes	Erythraeidae	JSAUG1832-12	KR069401
Trombidiformes	Erythraeidae	JSAUG1835-12	KR069907
Trombidiformes	Erythraeidae	JSAUG1838-12	KR069328
Trombidiformes	Erythraeidae	JSAUG1841-12	KR069289
Trombidiformes	Erythraeidae	JSAUG1845-12	KR069740
Trombidiformes	Erythraeidae	JSAUG1847-12	KR069688
Trombidiformes	Erythraeidae	JSAUG1849-12	KR069864
Trombidiformes	Erythraeidae	JSAUG1851-12	KR069876
Trombidiformes	Erythraeidae	JSAUG1859-12	KR070694
Trombidiformes	Erythraeidae	JSAUG1864-12	KR069916
Trombidiformes	Erythraeidae	JSJUL2396-12	KR069443
Trombidiformes	Erythraeidae	JSJUL2403-12	KR070022
Trombidiformes	Erythraeidae	JSJUL2406-12	KR070459
Trombidiformes	Erythraeidae	JSJUL2525-12	KR070160
Trombidiformes	Erythraeidae	JSJUL2542-12	KR069281
Trombidiformes	Erythraeidae	JSJUL2546-12	KR069403
Trombidiformes	Erythraeidae	JSJUL2547-12	KR070454
Trombidiformes	Erythraeidae	JSJUL2549-12	KR070034
Trombidiformes	Erythraeidae	JSJUL2550-12	KR069250
Trombidiformes	Erythraeidae	JSJUN2322-12	KR070544
Trombidiformes	Erythraeidae	JSJUN2326-12	KR069661
Trombidiformes	Erythraeidae	JSJUN2334-12	KR070421
Trombidiformes	Erythraeidae	JSJUN2337-12	KR070613
Trombidiformes	Erythraeidae	JSJUN2338-12	KR070155
Trombidiformes	Erythraeidae	JSOIE076-12	KR070097
Trombidiformes	Erythraeidae	JSOIE077-12	KR069627
Trombidiformes	Erythraeidae	JSOIE079-12	KR069567
Trombidiformes	Erythraeidae	JSOIE098-12	KR069261
Trombidiformes	Erythraeidae	JSSEP1126-12	KR070289
Trombidiformes	Erythraeidae	JSSEP1138-12	KR069347
Trombidiformes	Erythraeidae	JSSEP1152-12	KR070248
Trombidiformes	Erythraeidae	MIAUS042-12	KR069464
Trombidiformes	Erythraeidae	MIAUS043-12	KR069966
Trombidiformes	Erythraeidae	MIAUS044-12	KR070572
Trombidiformes	Erythraeidae	MIAUS051-12	KR070476
Trombidiformes	Erythraeidae	MIAUS052-12	KR070085
Trombidiformes	Erythraeidae	MIAUS059-12	KR069650
Trombidiformes	Erythraeidae	MIAUS061-12	KR069253
Trombidiformes	Erythraeidae	MIAUS062-12	KR069634
Trombidiformes	Erythraeidae	MIAUS063-12	KR069173
Trombidiformes	Erythraeidae	MIAUS064-12	KR070601
Trombidiformes	Erythraeidae	MIAUS065-12	KR069324
Trombidiformes	Erythraeidae	MIAUS066-12	KR069890
Trombidiformes	Erythraeidae	MIAUS067-12	KP979262
Trombidiformes	Erythraeidae	MIAUS068-12	KR069361
Trombidiformes	Erythraeidae	MIAUS069-12	KR069795
Trombidiformes	Erythraeidae	MIAUS070-12	KR070057
Trombidiformes	Erythraeidae	MIAUS071-12	KR069529
Trombidiformes	Erythraeidae	MIAUS072-12	KR070533
Trombidiformes	Erythraeidae	MIAUS073-12	KR069229

Trombidiformes	Erythraeidae	MIONB097-10	KP979327
Trombidiformes	Erythraeidae	MIONB098-10	KR070030
Trombidiformes	Erythraeidae	MIONB109-10	KP979141
Trombidiformes	Erythraeidae	MIONB116-10	KR069860
Trombidiformes	Erythraeidae	MIONB147-10	KP979225
Trombidiformes	Erythraeidae	MIONB174-10	KR069608
Trombidiformes	Erythraeidae	MIONB175-10	KR069533
Trombidiformes	Erythraeidae	MIONB176-10	KP979313
Trombidiformes	Erythraeidae	MIONB177-10	KR070320
Trombidiformes	Erythraeidae	MIONB178-10	KR069621
Trombidiformes	Erythraeidae	MIONB179-10	KR069658
Trombidiformes	Erythraeidae	MIONB204-10	KR069496
Trombidiformes	Erythraeidae	MIONB205-10	KP979249
Trombidiformes	Erythraeidae	MIONB208-10	KP979174
Trombidiformes	Erythraeidae	MIONB209-10	KR070734
Trombidiformes	Erythraeidae	MIONB210-10	KP979199
Trombidiformes	Erythraeidae	MIONB216-10	KR069418
Trombidiformes	Erythraeidae	MIONB219-10	KR069208
Trombidiformes	Erythraeidae	MIONB221-10	KR069886
Trombidiformes	Erythraeidae	MIONB223-10	KR069761
Trombidiformes	Erythraeidae	MIONB313-10	KP979193
Trombidiformes	Erythraeidae	MIONB314-10	KP979292
Trombidiformes	Erythraeidae	MIONB315-10	KR069557
Trombidiformes	Erythraeidae	MIONB316-10	KP979252
Trombidiformes	Erythraeidae	MIONB317-10	KR069363
Trombidiformes	Erythraeidae	MIONB318-10	KR070170
Trombidiformes	Erythraeidae	MIONB319-10	KR069780
Trombidiformes	Erythraeidae	MIONB334-10	KP979301
Trombidiformes	Erythraeidae	MIONB414-10	KR069751
Trombidiformes	Erythraeidae	MIONB429-10	KR069530
Trombidiformes	Erythraeidae	MIONB436-10	KR069847
Trombidiformes	Erythraeidae	MIONB448-10	KM837890
Trombidiformes	Erythraeidae	MIONB449-10	KM824042
Trombidiformes	Erythraeidae	MIONB450-10	KM826793
Trombidiformes	Erythraeidae	MIONB465-11	KM837360
Trombidiformes	Erythraeidae	MIONB467-11	KM830022
Trombidiformes	Erythraeidae	MIONB468-11	KM840492
Trombidiformes	Erythraeidae	MIONB469-11	KM834662
Trombidiformes	Erythraeidae	MIONB470-11	KM838354
Trombidiformes	Erythraeidae	MIONB471-11	KM838069
Trombidiformes	Erythraeidae	MIONB472-11	KM833210
Trombidiformes	Erythraeidae	MIONB509-11	KM833103
Trombidiformes	Erythraeidae	MIONB513-11	KM832316
Trombidiformes	Erythraeidae	MIONB514-11	KM827280
Trombidiformes	Erythraeidae	MIONB515-11	KM831049
Trombidiformes	Erythraeidae	MIONB517-11	KM838515
Trombidiformes	Erythraeidae	MIONB532-11	KP979345
Trombidiformes	Erythraeidae	MYMCA088-11	JX838401
Trombidiformes	Erythraeidae	MYMCA1348-11	JX836361
Trombidiformes	Erythraeidae	MYMCB219-11	JX837585
Trombidiformes	Erythraeidae	MYMCB220-11	JX837332
Trombidiformes	Erythraeidae	MYMCB221-11	JX837716
Trombidiformes	Erythraeidae	MYMCB466-11	JX837292

Trombidiformes	Erythraeidae	MYMCB467-11	JX834482
Trombidiformes	Erythraeidae	MYMCB646-11	JX836647
Trombidiformes	Erythraeidae	MYMCB680-11	JX835178
Trombidiformes	Erythraeidae	MYMCC421-11	JX834006
Trombidiformes	Erythraeidae	MYMCC534-11	JX838618
Trombidiformes	Erythraeidae	MYMCC554-11	JX836126
Trombidiformes	Erythraeidae	MYMCC590-11	JX835130
Trombidiformes	Erythraeidae	MYMCC694-11	JX837669
Trombidiformes	Erythraeidae	MYMCC695-11	JX837885
Trombidiformes	Erythraeidae	MYMCC696-11	JX838477
Trombidiformes	Erythraeidae	MYMCC722-11	JX838318
Trombidiformes	Erythraeidae	MYMCC756-11	JX836512
Trombidiformes	Erythraeidae	MYMCC757-11	JX835096
Trombidiformes	Erythraeidae	MYMCC773-11	JX836844
Trombidiformes	Erythraeidae	MYMCD149-11	JX835289
Trombidiformes	Erythraeidae	MYMCE146-12	JX834051
Trombidiformes	Erythraeidae	MYMCE147-12	JX835627
Trombidiformes	Erythraeidae	MYMCE485-12	JX838673
Trombidiformes	Erythraeidae	MYMCE486-12	JX835431
Trombidiformes	Erythraeidae	MYMCE528-12	JX834819
Trombidiformes	Erythraeidae	MYMCF118-12	JX834465
Trombidiformes	Erythraeidae	MYMCF634-12	JX837075
Trombidiformes	Erythraeidae	MYTMC001-09	HQ966220
Trombidiformes	Erythraeidae	PHAUG1660-11	KR070188
Trombidiformes	Erythraeidae	PHAUG1661-11	KR070473
Trombidiformes	Erythraeidae	PHJUN3813-11	KR070256
Trombidiformes	Erythraeidae	PHJUN3844-11	KR069606
Trombidiformes	Erythraeidae	SMTPF6017-14	KR069287
Trombidiformes	Erythraeidae	SSBAA1998-12	KM838023
Trombidiformes	Erythraeidae	SSBAA2004-12	KM824677
Trombidiformes	Erythraeidae	SSBAA2011-12	KM834567
Trombidiformes	Erythraeidae	SSBAA2012-12	KM835161
Trombidiformes	Erythraeidae	SSBAA2040-12	KM829894
Trombidiformes	Erythraeidae	SSBAA2050-12	KM826295
Trombidiformes	Erythraeidae	SSBAA2082-12	KM833432
Trombidiformes	Erythraeidae	SSBAB1130-12	KM835929
Trombidiformes	Erythraeidae	SSBAB1132-12	KM830875
Trombidiformes	Erythraeidae	SSBAB1135-12	KM825203
Trombidiformes	Erythraeidae	SSBAB1139-12	KM830709
Trombidiformes	Erythraeidae	SSBAB1140-12	KM830791
Trombidiformes	Erythraeidae	SSBAB1142-12	KM834780
Trombidiformes	Erythraeidae	SSBAB1143-12	KM831373
Trombidiformes	Erythraeidae	SSBAB1144-12	KM835711
Trombidiformes	Erythraeidae	SSBAB1156-12	KM835145
Trombidiformes	Erythraeidae	SSBAB1178-12	KM835775
Trombidiformes	Erythraeidae	SSBAB156-12	KM829400
Trombidiformes	Erythraeidae	SSBAC2360-12	KM834526
Trombidiformes	Erythraeidae	SSBAC2361-12	KM834049
Trombidiformes	Erythraeidae	SSBAC2362-12	KM827541
Trombidiformes	Erythraeidae	SSBAC2363-12	KM838689
Trombidiformes	Erythraeidae	SSBAC2364-12	KM837618
Trombidiformes	Erythraeidae	SSBAC2365-12	KM837944
Trombidiformes	Erythraeidae	SSBAC2368-12	KM840146

Trombidiformes	Erythraeidae	SSBAC2369-12	KM840718
Trombidiformes	Erythraeidae	SSBAC2370-12	KM824916
Trombidiformes	Erythraeidae	SSBAC2371-12	KM834550
Trombidiformes	Erythraeidae	SSBAC3320-12	KM828168
Trombidiformes	Erythraeidae	SSBAD3090-12	KM831015
Trombidiformes	Erythraeidae	SSBAD3139-12	KM838423
Trombidiformes	Erythraeidae	SSBAD3957-12	KM835668
Trombidiformes	Erythraeidae	SSBAD3964-12	KM839976
Trombidiformes	Erythraeidae	SSBAD3966-12	KM839555
Trombidiformes	Erythraeidae	SSBAD3987-12	KM827674
Trombidiformes	Erythraeidae	SSBAD3995-12	KM839731
Trombidiformes	Erythraeidae	SSBAD3996-12	KM834232
Trombidiformes	Erythraeidae	SSBAD4004-12	KM839155
Trombidiformes	Erythraeidae	SSBAD4706-13	KM839181
Trombidiformes	Erythraeidae	SSBAD4716-13	KM825469
Trombidiformes	Erythraeidae	SSBAD4737-13	KM825532
Trombidiformes	Erythraeidae	SSBAD4750-13	KM826584
Trombidiformes	Erythraeidae	SSBAD5544-13	KM829072
Trombidiformes	Erythraeidae	SSBAE3005-13	KM826115
Trombidiformes	Erythraeidae	SSBAE3764-13	KM832368
Trombidiformes	Erythraeidae	SSBAE3808-13	KM829384
Trombidiformes	Erythraeidae	SSBAE3809-13	KM834278
Trombidiformes	Erythraeidae	SSEIA044-13	KM829548
Trombidiformes	Erythraeidae	SSEIA2086-13	KM836641
Trombidiformes	Erythraeidae	SSEIA2097-13	KM828161
Trombidiformes	Erythraeidae	SSEIA2099-13	KM836442
Trombidiformes	Erythraeidae	SSEIA3015-13	KM838997
Trombidiformes	Erythraeidae	SSEIA3467-13	KM829624
Trombidiformes	Erythraeidae	SSEIA4482-13	KM835741
Trombidiformes	Erythraeidae	SSEIA5512-13	KM828470
Trombidiformes	Erythraeidae	SSEIA7680-13	KM828624
Trombidiformes	Erythraeidae	SSEIA7681-13	KM824295
Trombidiformes	Erythraeidae	SSEIA7682-13	KM825572
Trombidiformes	Erythraeidae	SSEIA7685-13	KM832917
Trombidiformes	Erythraeidae	SSEIA7688-13	KM837271
Trombidiformes	Erythraeidae	SSEIB11403-13	KM825770
Trombidiformes	Erythraeidae	SSEIB4220-13	KM837285
Trombidiformes	Erythraeidae	SSEIB4224-13	KM837168
Trombidiformes	Erythraeidae	SSEIB4225-13	KM825002
Trombidiformes	Erythraeidae	SSEIB4232-13	KM834553
Trombidiformes	Erythraeidae	SSEIB4243-13	KM824022
Trombidiformes	Erythraeidae	SSEIB4584-13	KM826810
Trombidiformes	Erythraeidae	SSEIC1305-13	KM826988
Trombidiformes	Erythraeidae	SSEIC982-13	KM827496
Trombidiformes	Erythraeidae	SSJAB1826-13	KM828167
Trombidiformes	Erythraeidae	SSJAB1827-13	KM834832
Trombidiformes	Erythraeidae	SSJAB1828-13	KM831656
Trombidiformes	Erythraeidae	SSJAB1829-13	KM830927
Trombidiformes	Erythraeidae	SSJAB1830-13	KM831383
Trombidiformes	Erythraeidae	SSJAB1834-13	KM831355
Trombidiformes	Erythraeidae	SSJAB1837-13	KM826739
Trombidiformes	Erythraeidae	SSJAB1840-13	KM827649
Trombidiformes	Erythraeidae	SSJAB1842-13	KM829087

Trombidiformes	Erythraeidae	SSJAB1843-13	KM832620
Trombidiformes	Erythraeidae	SSJAB1866-13	KM830195
Trombidiformes	Erythraeidae	SSJAB1867-13	KM831802
Trombidiformes	Erythraeidae	SSJAB1868-13	KM827043
Trombidiformes	Erythraeidae	SSJAB1869-13	KM840580
Trombidiformes	Erythraeidae	SSJAB1871-13	KM831010
Trombidiformes	Erythraeidae	SSJAB2194-13	KM828478
Trombidiformes	Erythraeidae	SSJAB2410-13	KM835745
Trombidiformes	Erythraeidae	SSJAB2411-13	KM826097
Trombidiformes	Erythraeidae	SSJAB2412-13	KM835456
Trombidiformes	Erythraeidae	SSJAB2413-13	KM824478
Trombidiformes	Erythraeidae	SSJAB2414-13	KM828428
Trombidiformes	Erythraeidae	SSJAB2985-13	KM833809
Trombidiformes	Erythraeidae	SSJAB2986-13	KM831632
Trombidiformes	Erythraeidae	SSJAB2987-13	KM837964
Trombidiformes	Erythraeidae	SSJAB2989-13	KM838653
Trombidiformes	Erythraeidae	SSJAB2992-13	KM824703
Trombidiformes	Erythraeidae	SSJAB2994-13	KM827559
Trombidiformes	Erythraeidae	SSJAB2995-13	KM832809
Trombidiformes	Erythraeidae	SSJAB2996-13	KM824849
Trombidiformes	Erythraeidae	SSJAB2997-13	KM824268
Trombidiformes	Erythraeidae	SSJAB2998-13	KM833147
Trombidiformes	Erythraeidae	SSJAB2999-13	KM825013
Trombidiformes	Erythraeidae	SSJAB3003-13	KM833942
Trombidiformes	Erythraeidae	SSJAB3004-13	KM839182
Trombidiformes	Erythraeidae	SSJAB3006-13	KM825590
Trombidiformes	Erythraeidae	SSJAB3007-13	KM840558
Trombidiformes	Erythraeidae	SSJAB3008-13	KM836899
Trombidiformes	Erythraeidae	SSJAB3009-13	KM837606
Trombidiformes	Erythraeidae	SSJAB3010-13	KM839487
Trombidiformes	Erythraeidae	SSJAB3013-13	KM828643
Trombidiformes	Erythraeidae	SSJAB3014-13	KM830186
Trombidiformes	Erythraeidae	SSJAB3017-13	KM836487
Trombidiformes	Erythraeidae	SSJAB3018-13	KM824279
Trombidiformes	Erythraeidae	SSJAB3021-13	KM834399
Trombidiformes	Erythraeidae	SSJAB3024-13	KM833642
Trombidiformes	Erythraeidae	SSJAB3025-13	KM827380
Trombidiformes	Erythraeidae	SSJAB3026-13	KM832537
Trombidiformes	Erythraeidae	SSJAB3027-13	KM837999
Trombidiformes	Erythraeidae	SSJAB3028-13	KM824958
Trombidiformes	Erythraeidae	SSJAB3029-13	KM834956
Trombidiformes	Erythraeidae	SSJAB3031-13	KM838712
Trombidiformes	Erythraeidae	SSJAB3032-13	KM827260
Trombidiformes	Erythraeidae	SSJAB3034-13	KM829406
Trombidiformes	Erythraeidae	SSJAB3035-13	KM839439
Trombidiformes	Erythraeidae	SSJAB3038-13	KM836604
Trombidiformes	Erythraeidae	SSJAB3039-13	KM835260
Trombidiformes	Erythraeidae	SSJAB3040-13	KM829895
Trombidiformes	Erythraeidae	SSJAB3041-13	KM835483
Trombidiformes	Erythraeidae	SSJAB3042-13	KM839385
Trombidiformes	Erythraeidae	SSJAB3043-13	KM824756
Trombidiformes	Erythraeidae	SSJAB3044-13	KM833833
Trombidiformes	Erythraeidae	SSJAB3045-13	KM830566

Trombidiformes	Erythraeidae		SSJAB3046-13	KM836930
Trombidiformes	Erythraeidae		SSJAB3049-13	KM835730
Trombidiformes	Erythraeidae		SSJAB3050-13	KM829354
Trombidiformes	Erythraeidae		SSJAB3051-13	KM826273
Trombidiformes	Erythraeidae		SSJAB3052-13	KM833335
Trombidiformes	Erythraeidae		SSJAB3053-13	KM833489
Trombidiformes	Erythraeidae		SSJAB3054-13	KM839093
Trombidiformes	Erythraeidae		SSJAB3055-13	KM826374
Trombidiformes	Erythraeidae		SSJAB3056-13	KM838724
Trombidiformes	Erythraeidae		SSJAB3058-13	KM828994
Trombidiformes	Erythraeidae		SSJAB3060-13	KM835428
Trombidiformes	Erythraeidae		SSJAB3061-13	KM837792
Trombidiformes	Erythraeidae		SSJAB3063-13	KM837443
Trombidiformes	Erythraeidae		SSJAB3066-13	KM830609
Trombidiformes	Erythraeidae		SSJAB3068-13	KM827239
Trombidiformes	Erythraeidae		SSJAB3069-13	KM836513
Trombidiformes	Erythraeidae		SSJAB3071-13	KM830895
Trombidiformes	Erythraeidae		SSJAB3072-13	KM840364
Trombidiformes	Erythraeidae		SSJAB3073-13	KM828064
Trombidiformes	Erythraeidae		SSJAB3074-13	KM826450
Trombidiformes	Erythraeidae		SSJAB3075-13	KM838853
Trombidiformes	Erythraeidae		SSJAB3076-13	KM840168
Trombidiformes	Erythraeidae		SSJAB3077-13	KM834942
Trombidiformes	Erythraeidae		SSJAB3078-13	KM839114
Trombidiformes	Erythraeidae		SSJAB3079-13	KM827157
Trombidiformes	Erythraeidae		SSJAB3363-13	KM838540
Trombidiformes	Erythraeidae		SSJAF3112-13	KM827550
Trombidiformes	Erythraeidae		SSJAF3169-13	KM838851
Trombidiformes	Erythraeidae		SSJAF3170-13	KM825164
Trombidiformes	Erythraeidae		SSJAF3522-13	KM834367
Trombidiformes	Erythraeidae		SSJAF5560-13	KM826204
Trombidiformes	Erythraeidae		SSPAA6650-13	KM831352
Trombidiformes	Erythraeidae		SSPAA6651-13	KM829117
Trombidiformes	Erythraeidae		SSWLA036-13	KM833228
Trombidiformes	Erythraeidae		SSWLB3546-13	KM832398
Trombidiformes	Erythraeidae		SSWLB5364-13	KM836290
Trombidiformes	Erythraeidae		SSWLF2941-13	KM834496
Trombidiformes	Erythraeidae		SSWLF4869-13	KM834500
Trombidiformes	Erythraeidae		SSWLF4871-13	KM839298
Trombidiformes	Erythraeidae		SSWLF677-13	KM826626
Trombidiformes	Erythraeidae		SSWLF678-13	KM838452
Trombidiformes	Erythraeidae		SSWLF682-13	KM838695
Trombidiformes	Erythraeidae		SSWLF691-13	KM824192
Trombidiformes	Erythraeidae		SSWLF715-13	KM831150
Trombidiformes	Eupodidae		SSBRA3406-14	KR070725
Trombidiformes	Eupodidae	*Eupodes*	CHACA1156-10	JX834442
Trombidiformes	Eupodidae	*Eupodes*	CHACA1157-10	JX838214
Trombidiformes	Eupodidae	*Eupodes*	CHACA1220-10	JX836310
Trombidiformes	Eupodidae	*Eupodes*	CHACA185-08	JX837800
Trombidiformes	Eupodidae	*Eupodes*	CHACB024-10	HQ558344
Trombidiformes	Eupodidae	*Eupodes*	CHACB1016-10	HM907152
Trombidiformes	Eupodidae	*Eupodes*	CHACB1017-10	HM907153
Trombidiformes	Eupodidae	*Eupodes*	CHACB1072-10	HM907189

Trombidiformes	Eupodidae	*Eupodes*	CHACB1073-10	JX838679
Trombidiformes	Eupodidae	*Eupodes*	CHACB117-10	HQ558407
Trombidiformes	Eupodidae	*Eupodes*	CHACB118-10	HQ558408
Trombidiformes	Eupodidae	*Eupodes*	CHACB170-10	HQ558451
Trombidiformes	Eupodidae	*Eupodes*	CHACB220-10	HQ558478
Trombidiformes	Eupodidae	*Eupodes*	CHACB275-10	HQ558510
Trombidiformes	Eupodidae	*Eupodes*	CHACB276-10	HQ558511
Trombidiformes	Eupodidae	*Eupodes*	CHACB323-10	HM907307
Trombidiformes	Eupodidae	*Eupodes*	CHACB403-10	HQ558565
Trombidiformes	Eupodidae	*Eupodes*	CHACB404-10	HQ558566
Trombidiformes	Eupodidae	*Eupodes*	CHACB405-10	HQ558567
Trombidiformes	Eupodidae	*Eupodes*	CHACB718-10	HQ558768
Trombidiformes	Eupodidae	*Eupodes*	CHACB719-10	HQ558769
Trombidiformes	Eupodidae	*Eupodes*	CHACB720-10	HQ558770
Trombidiformes	Eupodidae	*Eupodes*	CHACB855-10	HM907308
Trombidiformes	Eupodidae	*Eupodes*	CHACC064-10	HM907461
Trombidiformes	Eupodidae	*Eupodes*	CHACC065-10	HM907462
Trombidiformes	Eupodidae	*Eupodes*	CHACC217-10	HQ941535
Trombidiformes	Eupodidae	*Eupodes*	JSAUG1853-12	KR070418
Trombidiformes	Eupodidae	*Eupodes*	MIONB260-10	KP979325
Trombidiformes	Eupodidae	*Eupodes*	MYMCA1003-11	JX833929
Trombidiformes	Eupodidae	*Eupodes*	MYMCA1004-11	JX835380
Trombidiformes	Eupodidae	*Eupodes*	MYMCA1005-11	JX838539
Trombidiformes	Eupodidae	*Eupodes*	MYMCA1027-11	JX835401
Trombidiformes	Eupodidae	*Eupodes*	MYMCA1030-11	JX833880
Trombidiformes	Eupodidae	*Eupodes*	MYMCA1031-11	JX838091
Trombidiformes	Eupodidae	*Eupodes*	MYMCA1256-11	JX835301
Trombidiformes	Eupodidae	*Eupodes*	MYMCA1275-11	JX835804
Trombidiformes	Eupodidae	*Eupodes*	MYMCA1290-11	JX835712
Trombidiformes	Eupodidae	*Eupodes*	MYMCA1291-11	JX836231
Trombidiformes	Eupodidae	*Eupodes*	MYMCA1292-11	JX837995
Trombidiformes	Eupodidae	*Eupodes*	MYMCA1369-11	JX838535
Trombidiformes	Eupodidae	*Eupodes*	MYMCA1371-11	JX835498
Trombidiformes	Eupodidae	*Eupodes*	MYMCA1372-11	JX833777
Trombidiformes	Eupodidae	*Eupodes*	MYMCA1383-11	JX835216
Trombidiformes	Eupodidae	*Eupodes*	MYMCA1385-11	JX835535
Trombidiformes	Eupodidae	*Eupodes*	MYMCA1454-11	JX838611
Trombidiformes	Eupodidae	*Eupodes*	MYMCA1455-11	JX837741
Trombidiformes	Eupodidae	*Eupodes*	MYMCA1456-11	JX834328
Trombidiformes	Eupodidae	*Eupodes*	MYMCA1457-11	JX834210
Trombidiformes	Eupodidae	*Eupodes*	MYMCA1458-11	JX834455
Trombidiformes	Eupodidae	*Eupodes*	MYMCA1484-11	JX834162
Trombidiformes	Eupodidae	*Eupodes*	MYMCA1487-11	JX834164
Trombidiformes	Eupodidae	*Eupodes*	MYMCA583-11	JX835774
Trombidiformes	Eupodidae	*Eupodes*	MYMCA584-11	JX835692
Trombidiformes	Eupodidae	*Eupodes*	MYMCA585-11	JX834176
Trombidiformes	Eupodidae	*Eupodes*	MYMCA613-11	JX837226
Trombidiformes	Eupodidae	*Eupodes*	MYMCA643-11	JX836388
Trombidiformes	Eupodidae	*Eupodes*	MYMCA667-11	JX835923
Trombidiformes	Eupodidae	*Eupodes*	MYMCA709-11	JX834546
Trombidiformes	Eupodidae	*Eupodes*	MYMCA710-11	JX834345
Trombidiformes	Eupodidae	*Eupodes*	MYMCA735-11	JX835642
Trombidiformes	Eupodidae	*Eupodes*	MYMCA970-11	JX835055

Trombidiformes	Eupodidae	*Eupodes*	MYMCA971-11	JX833836
Trombidiformes	Eupodidae	*Eupodes*	MYMCA972-11	JX834936
Trombidiformes	Eupodidae	*Eupodes*	MYMCB008-11	JX836172
Trombidiformes	Eupodidae	*Eupodes*	MYMCB473-11	JX836493
Trombidiformes	Eupodidae	*Eupodes*	MYMCB616-11	JX835685
Trombidiformes	Eupodidae	*Eupodes*	MYMCB686-11	JX834148
Trombidiformes	Eupodidae	*Eupodes*	MYMCB688-11	JX836797
Trombidiformes	Eupodidae	*Eupodes*	MYMCB888-11	JX837655
Trombidiformes	Eupodidae	*Eupodes*	MYMCB896-11	JX834533
Trombidiformes	Eupodidae	*Eupodes*	MYMCC110-11	JX836220
Trombidiformes	Eupodidae	*Eupodes*	MYMCC111-11	JX838243
Trombidiformes	Eupodidae	*Eupodes*	MYMCC133-11	JX834205
Trombidiformes	Eupodidae	*Eupodes*	MYMCC134-11	JX837666
Trombidiformes	Eupodidae	*Eupodes*	MYMCC136-11	JX837619
Trombidiformes	Eupodidae	*Eupodes*	MYMCC138-11	JX835983
Trombidiformes	Eupodidae	*Eupodes*	MYMCC162-11	JX837746
Trombidiformes	Eupodidae	*Eupodes*	MYMCC320-11	JX836448
Trombidiformes	Eupodidae	*Eupodes*	MYMCC323-11	JX837912
Trombidiformes	Eupodidae	*Eupodes*	MYMCC349-11	JX838053
Trombidiformes	Eupodidae	*Eupodes*	MYMCC351-11	JX836897
Trombidiformes	Eupodidae	*Eupodes*	MYMCC352-11	JX838042
Trombidiformes	Eupodidae	*Eupodes*	MYMCC354-11	JX834295
Trombidiformes	Eupodidae	*Eupodes*	MYMCC404-11	JX834692
Trombidiformes	Eupodidae	*Eupodes*	MYMCC415-11	JX837876
Trombidiformes	Eupodidae	*Eupodes*	MYMCC541-11	JX835198
Trombidiformes	Eupodidae	*Eupodes*	MYMCC584-11	JX834406
Trombidiformes	Eupodidae	*Eupodes*	MYMCC753-11	JX836253
Trombidiformes	Eupodidae	*Eupodes*	MYMCC760-11	JX837056
Trombidiformes	Eupodidae	*Eupodes*	MYMCC761-11	JX836079
Trombidiformes	Eupodidae	*Eupodes*	MYMCC762-11	JX835286
Trombidiformes	Eupodidae	*Eupodes*	MYMCC763-11	JX835119
Trombidiformes	Eupodidae	*Eupodes*	MYMCC764-11	JX833810
Trombidiformes	Eupodidae	*Eupodes*	MYMCC781-11	JX836887
Trombidiformes	Eupodidae	*Eupodes*	MYMCC827-11	JX835555
Trombidiformes	Eupodidae	*Eupodes*	MYMCC833-11	JX835221
Trombidiformes	Eupodidae	*Eupodes*	MYMCC835-11	JX833728
Trombidiformes	Eupodidae	*Eupodes*	MYMCC889-11	JX836022
Trombidiformes	Eupodidae	*Eupodes*	MYMCC945-11	JX835504
Trombidiformes	Eupodidae	*Eupodes*	MYMCD040-11	JX834585
Trombidiformes	Eupodidae	*Eupodes*	MYMCD041-11	JX835398
Trombidiformes	Eupodidae	*Eupodes*	MYMCD046-11	JX838199
Trombidiformes	Eupodidae	*Eupodes*	MYMCD068-11	JX835650
Trombidiformes	Eupodidae	*Eupodes*	MYMCD071-11	JX836413
Trombidiformes	Eupodidae	*Eupodes*	MYMCD086-11	JX836204
Trombidiformes	Eupodidae	*Eupodes*	MYMCD090-11	JX838360
Trombidiformes	Eupodidae	*Eupodes*	MYMCD104-11	JX837517
Trombidiformes	Eupodidae	*Eupodes*	MYMCD107-11	JX835805
Trombidiformes	Eupodidae	*Eupodes*	MYMCD124-11	JX837166
Trombidiformes	Eupodidae	*Eupodes*	MYMCD139-11	JX834978
Trombidiformes	Eupodidae	*Eupodes*	MYMCD181-11	JX834909
Trombidiformes	Eupodidae	*Eupodes*	MYMCD182-11	JX838195
Trombidiformes	Eupodidae	*Eupodes*	MYMCE008-12	JX834786
Trombidiformes	Eupodidae	*Eupodes*	MYMCE040-12	JX834688

Trombidiformes	Eupodidae	*Eupodes*	MYMCE041-12	JX838561
Trombidiformes	Eupodidae	*Eupodes*	MYMCE046-12	JX837895
Trombidiformes	Eupodidae	*Eupodes*	MYMCE064-12	JX836148
Trombidiformes	Eupodidae	*Eupodes*	MYMCE073-12	JX835702
Trombidiformes	Eupodidae	*Eupodes*	MYMCE096-12	JX838715
Trombidiformes	Eupodidae	*Eupodes*	MYMCE187-12	JX836561
Trombidiformes	Eupodidae	*Eupodes*	MYMCE230-12	JX835243
Trombidiformes	Eupodidae	*Eupodes*	MYMCE239-12	JX834412
Trombidiformes	Eupodidae	*Eupodes*	MYMCE299-12	JX834297
Trombidiformes	Eupodidae	*Eupodes*	MYMCE300-12	JX834299
Trombidiformes	Eupodidae	*Eupodes*	MYMCE323-12	JX837145
Trombidiformes	Eupodidae	*Eupodes*	MYMCE345-12	JX838548
Trombidiformes	Eupodidae	*Eupodes*	MYMCE382-12	JX833694
Trombidiformes	Eupodidae	*Eupodes*	MYMCE399-12	JX838747
Trombidiformes	Eupodidae	*Eupodes*	MYMCE408-12	JX838332
Trombidiformes	Eupodidae	*Eupodes*	MYMCE409-12	JX833725
Trombidiformes	Eupodidae	*Eupodes*	MYMCE410-12	JX837777
Trombidiformes	Eupodidae	*Eupodes*	MYMCE412-12	JX834018
Trombidiformes	Eupodidae	*Eupodes*	MYMCE413-12	JX838423
Trombidiformes	Eupodidae	*Eupodes*	MYMCE415-12	JX833839
Trombidiformes	Eupodidae	*Eupodes*	MYMCE444-12	JX836195
Trombidiformes	Eupodidae	*Eupodes*	MYMCE445-12	JX836921
Trombidiformes	Eupodidae	*Eupodes*	MYMCE446-12	JX835979
Trombidiformes	Eupodidae	*Eupodes*	MYMCE447-12	JX833729
Trombidiformes	Eupodidae	*Eupodes*	MYMCE449-12	JX837072
Trombidiformes	Eupodidae	*Eupodes*	MYMCE460-12	JX834453
Trombidiformes	Eupodidae	*Eupodes*	MYMCE480-12	JX837040
Trombidiformes	Eupodidae	*Eupodes*	MYMCE481-12	JX834846
Trombidiformes	Eupodidae	*Eupodes*	MYMCE482-12	JX836885
Trombidiformes	Eupodidae	*Eupodes*	MYMCE538-12	JX835652
Trombidiformes	Eupodidae	*Eupodes*	MYMCE580-12	JX836386
Trombidiformes	Eupodidae	*Eupodes*	MYMCE716-12	JX834545
Trombidiformes	Eupodidae	*Eupodes*	MYMCE789-12	JX835906
Trombidiformes	Eupodidae	*Eupodes*	MYMCF217-12	JX838443
Trombidiformes	Eupodidae	*Eupodes*	MYMCF252-12	JX838556
Trombidiformes	Eupodidae	*Eupodes*	MYMCF253-12	JX837652
Trombidiformes	Eupodidae	*Eupodes*	MYMCF497-12	JX836760
Trombidiformes	Eupodidae	*Eupodes*	MYMCF536-12	JX836136
Trombidiformes	Eupodidae	*Eupodes*	MYMCF572-12	JX838283
Trombidiformes	Eupodidae	*Eupodes*	MYMCF601-12	JX833750
Trombidiformes	Eupodidae	*Eupodes*	MYMCF619-12	JX834266
Trombidiformes	Eupodidae	*Eupodes*	MYMCF620-12	JX836298
Trombidiformes	Eupodidae	*Eupodes*	MYMCF621-12	JX838635
Trombidiformes	Eupodidae	*Eupodes*	MYMCF622-12	JX834240
Trombidiformes	Eupodidae	*Eupodes*	MYMCF623-12	JX835277
Trombidiformes	Eupodidae	*Eupodes*	MYMCF685-12	JX835719
Trombidiformes	Eupodidae	*Eupodes*	MYMCF686-12	JX837361
Trombidiformes	Eupodidae	*Eupodes*	MYMCF712-12	JX837151
Trombidiformes	Eupodidae	*Eupodes*	MYMCF752-12	JX833837
Trombidiformes	Eupodidae	*Eupodes*	MYMCF814-12	JX835352
Trombidiformes	Eupodidae	*Eupodes*	MYMCF817-12	JX836995
Trombidiformes	Eupodidae	*Eupodes*	MYMCF905-12	JX833828
Trombidiformes	Eupodidae	*Eupodes*	MYMCF906-12	JX837110

Trombidiformes	Eupodidae	*Eupodes*	MYMCF907-12	JX837348
Trombidiformes	Eupodidae	*Eupodes*	MYMCF909-12	JX837080
Trombidiformes	Eupodidae	*Eupodes*	MYMCF910-12	JX838466
Trombidiformes	Eupodidae	*Eupodes*	MYMCG126-12	JX836335
Trombidiformes	Eupodidae	*Eupodes*	MYMCG127-12	JX836892
Trombidiformes	Eupodidae	*Eupodes*	MYMCG128-12	JX836119
Trombidiformes	Eupodidae	*Eupodes*	MYMCG148-12	JX837386
Trombidiformes	Eupodidae	*Eupodes*	MYMCG178-12	JX837052
Trombidiformes	Eupodidae	*Eupodes*	MYMCG179-12	JX836456
Trombidiformes	Eupodidae	*Eupodes*	MYMCG181-12	JX836113
Trombidiformes	Eupodidae	*Eupodes*	MYMCG189-12	JX833653
Trombidiformes	Eupodidae	*Eupodes*	MYMCG216-12	JX835680
Trombidiformes	Eupodidae	*Eupodes*	MYMCG279-12	JX833751
Trombidiformes	Eupodidae	*Eupodes*	MYMCG299-12	JX838202
Trombidiformes	Eupodidae	*Eupodes*	MYMCG469-12	JX838490
Trombidiformes	Eupodidae	*Eupodes*	MYMCG472-12	JX835049
Trombidiformes	Eupodidae	*Eupodes*	MYMCG488-12	JX836078
Trombidiformes	Eupodidae	*Eupodes*	MYMCG493-12	JX837755
Trombidiformes	Eupodidae	*Eupodes*	MYMCG497-12	JX835890
Trombidiformes	Eupodidae	*Eupodes*	MYMCG642-12	JX837249
Trombidiformes	Eupodidae	*Eupodes*	MYMCG644-12	JX834730
Trombidiformes	Eupodidae	*Eupodes*	MYTMC005-09	GU680487
Trombidiformes	Eupodidae	*Eupodes*	MYTMC010-09	HQ966221
Trombidiformes	Eupodidae	*Eupodes*	MYTMC082-09	GU680460
Trombidiformes	Eupodidae	*Eupodes*	RBINA3915-13	KP979299
Trombidiformes	Eupodidae	*Eupodes*	SSBAD3960-12	KM828764
Trombidiformes	Eupodidae	*Eupodes*	SSBAD4021-12	KM839001
Trombidiformes	Eupodidae	*Eupodes*	SSBAD4039-12	KM838774
Trombidiformes	Eupodidae	*Eupodes*	SSBAD4734-13	KM825748
Trombidiformes	Eupodidae	*Eupodes*	SSBAD4751-13	KM828918
Trombidiformes	Eupodidae	*Eupodes*	SSEIB7657-13	KM833450
Trombidiformes	Eupodidae	*Eupodes*	SSEIB7663-13	KM829409
Trombidiformes	Eupodidae	*Eupodes*	SSEIB7665-13	KM831196
Trombidiformes	Eupodidae	*Eupodes*	SSEIB7678-13	KM840250
Trombidiformes	Eupodidae	*Eupodes*	SSEIB8206-13	KM831302
Trombidiformes	Eupodidae	*Eupodes*	SSJAB2167-13	KM836372
Trombidiformes	Eupodidae	*Linopodes*	CHACA540-09	JX834232
Trombidiformes	Eupodidae	*Linopodes*	CHACB603-10	HQ558698
Trombidiformes	Eupodidae	*Linopodes*	CNEIF2393-12	KM826758
Trombidiformes	Eupodidae	*Linopodes*	CNEIF2394-12	KM828220
Trombidiformes	Eupodidae	*Linopodes*	CNEIF2398-12	KM827312
Trombidiformes	Eupodidae	*Linopodes*	CNEIG1587-13	KM827862
Trombidiformes	Eupodidae	*Linopodes*	CNEIG1590-13	KM828535
Trombidiformes	Eupodidae	*Linopodes*	CNJAH772-12	KM829836
Trombidiformes	Eupodidae	*Linopodes*	CNRMD2692-12	KM836559
Trombidiformes	Eupodidae	*Linopodes*	CNRMD2704-12	KM829205
Trombidiformes	Eupodidae	*Linopodes*	CNRME4712-12	KM827792
Trombidiformes	Eupodidae	*Linopodes*	CNRME4727-12	KM831663
Trombidiformes	Eupodidae	*Linopodes*	CNRME4729-12	KM838327
Trombidiformes	Eupodidae	*Linopodes*	CNRME4731-12	KM835967
Trombidiformes	Eupodidae	*Linopodes*	CNRME4737-12	KM829732
Trombidiformes	Eupodidae	*Linopodes*	CNRME4776-12	KM825324
Trombidiformes	Eupodidae	*Linopodes*	CNRME4796-12	KM829208

Trombidiformes	Eupodidae	*Linopodes*	MYMCB022-11	JX837638
Trombidiformes	Eupodidae	*Linopodes*	MYMCB184-11	JX838186
Trombidiformes	Eupodidae	*Linopodes*	MYMCB186-11	JX834354
Trombidiformes	Eupodidae	*Linopodes*	MYMCB893-11	JX838280
Trombidiformes	Eupodidae	*Linopodes*	MYMCB894-11	JX838026
Trombidiformes	Eupodidae	*Linopodes*	MYMCC106-11	JX835842
Trombidiformes	Eupodidae	*Linopodes*	MYMCC347-11	JX834106
Trombidiformes	Eupodidae	*Linopodes*	MYMCC348-11	JX835571
Trombidiformes	Eupodidae	*Linopodes*	MYMCC378-11	JX836132
Trombidiformes	Eupodidae	*Linopodes*	MYMCC703-11	JX834916
Trombidiformes	Eupodidae	*Linopodes*	MYMCC758-11	JX837766
Trombidiformes	Eupodidae	*Linopodes*	MYMCC839-11	JX834402
Trombidiformes	Eupodidae	*Linopodes*	MYMCD036-11	JX837193
Trombidiformes	Eupodidae	*Linopodes*	MYMCD047-11	JX833961
Trombidiformes	Eupodidae	*Linopodes*	MYMCF102-12	JX837493
Trombidiformes	Eupodidae	*Linopodes*	MYMCG633-12	JX836338
Trombidiformes	Eupodidae	*Linopodes*	SSEIB7651-13	KM826341
Trombidiformes	Eupodidae	*Linopodes*	SSJAC1510-13	KM829419
Trombidiformes	Eupodidae	*Linopodes*	SSJAC1517-13	KM824131
Trombidiformes	Eupodidae	*Linopodes*	SSJAC1524-13	KM826413
Trombidiformes	Eupodidae	*Linopodes*	SSJAC1588-13	KM836587
Trombidiformes	Eupodidae	*Linopodes*	SSJAC963-13	KM825508
Trombidiformes	Eupodidae	*Linopodes*	SSJAC985-13	KM828864
Trombidiformes	Eupodidae	*Linopodes*	SSJAC987-13	KM825124
Trombidiformes	Eupodidae	*Linopodes*	SSJAC990-13	KM828765
Trombidiformes	Eupodidae	*Linopodes*	SSPAA7793-13	KM830268
Trombidiformes	Eupodidae	*Linopodes*	SSPAA7820-13	KM824587
Trombidiformes	Eupodidae	*Linopodes*	SSPAA7822-13	KM831343
Trombidiformes	Eupodidae	*Linopodes*	SSPAA7823-13	KM831208
Trombidiformes	Eupodidae	*Linopodes*	SSPAA7825-13	KM830777
Trombidiformes	Eupodidae	*Linopodes*	SSPAA7826-13	KM837120
Trombidiformes	Eupodidae	*Linopodes*	SSPAA7829-13	KM840307
Trombidiformes	Eupodidae	*Linopodes*	SSPAA7835-13	KM832578
Trombidiformes	Eupodidae	*Linopodes*	SSPAA7939-13	KM839899
Trombidiformes	Eupodidae	*Linopodes*	SSPAA7956-13	KM839684
Trombidiformes	Eupodidae	*Linopodes*	SSPAA7958-13	KM837811
Trombidiformes	Eupodidae	*Linopodes*	SSPAA7983-13	KM840835
Trombidiformes	Eupodidae	*Linopodes*	SSPAB8702-13	KM830418
Trombidiformes	Eupodidae	*Linopodes*	SSPAB8711-13	KM839087
Trombidiformes	Eupodidae	*Linopodes*	SSPAB8714-13	KM829376
Trombidiformes	Eupodidae	*Linopodes*	SSPAB8715-13	KM833002
Trombidiformes	Eupodidae	*Linopodes*	SSPAB980-13	KM831734
Trombidiformes	Eupodidae	*Linopodes*	SSPAC11103-13	KM825439
Trombidiformes	Eupodidae		BBLZI147-14	KR070512
Trombidiformes	Eupodidae		CHACA1055-10	HM907106
Trombidiformes	Eupodidae		CHACA1218-10	JX834940
Trombidiformes	Eupodidae		CHACA543-09	JX836053
Trombidiformes	Eupodidae		CHACA818-09	JX838385
Trombidiformes	Eupodidae		CHACA965-10	HM405831
Trombidiformes	Eupodidae		CHACB023-10	HQ558343
Trombidiformes	Eupodidae		CHACB1020-10	HM907154
Trombidiformes	Eupodidae		CHACB1021-10	JX837044
Trombidiformes	Eupodidae		CHACB1071-10	HM907188

Trombidiformes	Eupodidae	CHACB1120-10	HM907213
Trombidiformes	Eupodidae	CHACB1121-10	HM907214
Trombidiformes	Eupodidae	CHACB1122-10	HM907215
Trombidiformes	Eupodidae	CHACB119-10	JX835342
Trombidiformes	Eupodidae	CHACB1210-10	HM907279
Trombidiformes	Eupodidae	CHACB312-10	JX837590
Trombidiformes	Eupodidae	CHACB313-10	HM907301
Trombidiformes	Eupodidae	CHACB460-10	HQ558606
Trombidiformes	Eupodidae	CHACB462-10	HQ558607
Trombidiformes	Eupodidae	CHACB512-10	HQ558638
Trombidiformes	Eupodidae	CHACB630-10	HQ558714
Trombidiformes	Eupodidae	CHACB631-10	HQ558715
Trombidiformes	Eupodidae	CHACB633-10	JX837043
Trombidiformes	Eupodidae	CHACB634-10	HQ558717
Trombidiformes	Eupodidae	CHACB669-10	HQ558739
Trombidiformes	Eupodidae	CHACB694-10	HQ558752
Trombidiformes	Eupodidae	CHACB744-10	HQ558782
Trombidiformes	Eupodidae	CHACB745-10	HQ558783
Trombidiformes	Eupodidae	CHACB747-10	HQ558784
Trombidiformes	Eupodidae	CHACB856-10	HM907309
Trombidiformes	Eupodidae	CHACB940-10	HM907364
Trombidiformes	Eupodidae	CHACB941-10	HM907365
Trombidiformes	Eupodidae	CHACB996-10	HM907401
Trombidiformes	Eupodidae	CHACB997-10	HM907402
Trombidiformes	Eupodidae	CHACB998-10	HM907403
Trombidiformes	Eupodidae	CHACB999-10	HM907404
Trombidiformes	Eupodidae	CHACC002-10	HQ941516
Trombidiformes	Eupodidae	CHACC104-10	JX833976
Trombidiformes	Eupodidae	CHACC214-10	HQ941533
Trombidiformes	Eupodidae	CHACC215-10	HQ941534
Trombidiformes	Eupodidae	CHACC216-10	KR069776
Trombidiformes	Eupodidae	CHACC236-10	HQ941544
Trombidiformes	Eupodidae	CHACC246-10	HQ941551
Trombidiformes	Eupodidae	CHACC248-10	JX838692
Trombidiformes	Eupodidae	CNBAB372-12	KM833654
Trombidiformes	Eupodidae	CNBAI500-13	KM834491
Trombidiformes	Eupodidae	CNBPD599-12	KM835765
Trombidiformes	Eupodidae	CNBPF125-12	KM825561
Trombidiformes	Eupodidae	CNBPF133-12	KM835643
Trombidiformes	Eupodidae	CNGIA132-12	KM833980
Trombidiformes	Eupodidae	CNGIA136-12	KM834194
Trombidiformes	Eupodidae	CNGIB570-12	KM826684
Trombidiformes	Eupodidae	CNGIB580-12	KM834890
Trombidiformes	Eupodidae	CNGIC296-12	KM833665
Trombidiformes	Eupodidae	CNGID227-12	KM827259
Trombidiformes	Eupodidae	CNGID228-12	KM824530
Trombidiformes	Eupodidae	CNGID230-12	KM835373
Trombidiformes	Eupodidae	CNGIE403-12	KM828812
Trombidiformes	Eupodidae	CNJAC1120-12	KM838829
Trombidiformes	Eupodidae	CNJAC1438-12	KM840802
Trombidiformes	Eupodidae	CNJAF1986-12	KM831758
Trombidiformes	Eupodidae	CNJAG1825-12	KM832415
Trombidiformes	Eupodidae	CNJAH782-12	KM825611

Trombidiformes	Eupodidae	CNJAI856-12	KM837422
Trombidiformes	Eupodidae	CNJAI863-12	KM840496
Trombidiformes	Eupodidae	CNJAJ718-12	KM827702
Trombidiformes	Eupodidae	CNJAJ720-12	KM829212
Trombidiformes	Eupodidae	CNKJE2228-14	KR069292
Trombidiformes	Eupodidae	CNKJE2229-14	KR069825
Trombidiformes	Eupodidae	CNKJE2261-14	KR069859
Trombidiformes	Eupodidae	CNKJH896-14	KR069390
Trombidiformes	Eupodidae	CNKJH897-14	KR069601
Trombidiformes	Eupodidae	CNKJH905-14	KR069835
Trombidiformes	Eupodidae	CNKJH913-14	KR069663
Trombidiformes	Eupodidae	CNKJH928-14	KR069255
Trombidiformes	Eupodidae	CNKJI405-14	KR070026
Trombidiformes	Eupodidae	CNKJI475-14	KR069819
Trombidiformes	Eupodidae	CNKJI484-14	KR070376
Trombidiformes	Eupodidae	CNKJI519-14	KR069302
Trombidiformes	Eupodidae	CNKJI551-14	KR069596
Trombidiformes	Eupodidae	CNKJI567-14	KR069216
Trombidiformes	Eupodidae	CNKJI580-14	KR069612
Trombidiformes	Eupodidae	CNKJM2446-14	KR069849
Trombidiformes	Eupodidae	CNKJM2500-14	KR069179
Trombidiformes	Eupodidae	CNKJM2553-14	KR070105
Trombidiformes	Eupodidae	CNKJP926-14	KR069435
Trombidiformes	Eupodidae	CNKJP936-14	KR070059
Trombidiformes	Eupodidae	CNKJQ1046-14	KR070453
Trombidiformes	Eupodidae	CNKJQ1048-14	KR069831
Trombidiformes	Eupodidae	CNKJQ1071-14	KR070193
Trombidiformes	Eupodidae	CNKJQ1073-14	KR069906
Trombidiformes	Eupodidae	CNKJQ1078-14	KR069176
Trombidiformes	Eupodidae	CNKJQ1093-14	KR070429
Trombidiformes	Eupodidae	CNKJQ1184-14	KR069276
Trombidiformes	Eupodidae	CNKJQ1192-14	KR070608
Trombidiformes	Eupodidae	CNKJQ933-14	KR069771
Trombidiformes	Eupodidae	CNKJQ945-14	KR070056
Trombidiformes	Eupodidae	CNKJQ953-14	KR069942
Trombidiformes	Eupodidae	CNKJQ962-14	KR070626
Trombidiformes	Eupodidae	CNKJQ977-14	KR070033
Trombidiformes	Eupodidae	CNKJQ980-14	KR069594
Trombidiformes	Eupodidae	CNKJR408-14	KR069466
Trombidiformes	Eupodidae	CNKJR429-14	KR069219
Trombidiformes	Eupodidae	CNKJR432-14	KR070142
Trombidiformes	Eupodidae	CNKJR443-14	KR070602
Trombidiformes	Eupodidae	CNKJR469-14	KR069507
Trombidiformes	Eupodidae	CNKJR475-14	KR070531
Trombidiformes	Eupodidae	CNKJR480-14	KR069480
Trombidiformes	Eupodidae	CNKJR488-14	KR069588
Trombidiformes	Eupodidae	CNKJR506-14	KR070282
Trombidiformes	Eupodidae	CNLMS1085-14	KR069540
Trombidiformes	Eupodidae	CNPAC519-13	KM826226
Trombidiformes	Eupodidae	CNPAD556-13	KM834703
Trombidiformes	Eupodidae	CNPAD560-13	KM838224
Trombidiformes	Eupodidae	CNPAE449-13	KM836074
Trombidiformes	Eupodidae	CNPAE458-13	KM831593

Trombidiformes	Eupodidae	CNPAF862-13	KM825939
Trombidiformes	Eupodidae	CNPAF865-13	KM827276
Trombidiformes	Eupodidae	CNPAF879-13	KM827963
Trombidiformes	Eupodidae	CNPAF882-13	KM838716
Trombidiformes	Eupodidae	CNPAF891-13	KM832352
Trombidiformes	Eupodidae	CNPAF893-13	KM839534
Trombidiformes	Eupodidae	CNPAF894-13	KM833569
Trombidiformes	Eupodidae	CNPAF908-13	KM825037
Trombidiformes	Eupodidae	CNPAF909-13	KM833319
Trombidiformes	Eupodidae	CNPAF912-13	KM824029
Trombidiformes	Eupodidae	CNPAH423-13	KM835406
Trombidiformes	Eupodidae	CNPAI465-13	KM834163
Trombidiformes	Eupodidae	CNPKI1415-14	KR069595
Trombidiformes	Eupodidae	CNPKI1449-14	KR070341
Trombidiformes	Eupodidae	CNPKI1451-14	KR069170
Trombidiformes	Eupodidae	CNPKI1458-14	KR069669
Trombidiformes	Eupodidae	CNPKI1521-14	KR070483
Trombidiformes	Eupodidae	CNPKI1546-14	KR069756
Trombidiformes	Eupodidae	CNPKJ2912-14	KR070189
Trombidiformes	Eupodidae	CNPKJ2950-14	KR070283
Trombidiformes	Eupodidae	CNPKJ2963-14	KR069945
Trombidiformes	Eupodidae	CNPKJ2973-14	KR069408
Trombidiformes	Eupodidae	CNPKJ2982-14	KR069153
Trombidiformes	Eupodidae	CNPKJ2989-14	KR069836
Trombidiformes	Eupodidae	CNPKJ3018-14	KR069553
Trombidiformes	Eupodidae	CNPKJ3019-14	KR069640
Trombidiformes	Eupodidae	CNPKJ3020-14	KR069989
Trombidiformes	Eupodidae	CNPKJ3035-14	KR070667
Trombidiformes	Eupodidae	CNPKJ3040-14	KR070424
Trombidiformes	Eupodidae	CNPKJ3041-14	KR069871
Trombidiformes	Eupodidae	CNPKJ3049-14	KR069301
Trombidiformes	Eupodidae	CNPKJ3050-14	KR070023
Trombidiformes	Eupodidae	CNPKJ3053-14	KR069922
Trombidiformes	Eupodidae	CNPKJ3065-14	KR070363
Trombidiformes	Eupodidae	CNPKJ3075-14	KR070154
Trombidiformes	Eupodidae	CNPKJ3079-14	KR069311
Trombidiformes	Eupodidae	CNPKJ3080-14	KR069904
Trombidiformes	Eupodidae	CNPKJ3093-14	KR070172
Trombidiformes	Eupodidae	CNPKJ3096-14	KR069213
Trombidiformes	Eupodidae	CNPKJ3101-14	KR069265
Trombidiformes	Eupodidae	CNPKJ3102-14	KR069643
Trombidiformes	Eupodidae	CNPKJ3120-14	KR069506
Trombidiformes	Eupodidae	CNPKJ3124-14	KR070340
Trombidiformes	Eupodidae	CNPKJ3132-14	KR069532
Trombidiformes	Eupodidae	CNPKJ3150-14	KR069617
Trombidiformes	Eupodidae	CNPKJ3168-14	KR069824
Trombidiformes	Eupodidae	CNPKJ3171-14	KR070456
Trombidiformes	Eupodidae	CNPKJ3178-14	KR070192
Trombidiformes	Eupodidae	CNPKJ3185-14	KR069359
Trombidiformes	Eupodidae	CNPKJ3193-14	KR070103
Trombidiformes	Eupodidae	CNPKJ3221-14	KR070173
Trombidiformes	Eupodidae	CNPKJ3270-14	KR069501
Trombidiformes	Eupodidae	CNPKJ3279-14	KR069898

Trombidiformes	Eupodidae	CNPKJ3296-14	KR069382
Trombidiformes	Eupodidae	CNPKJ3301-14	KR070728
Trombidiformes	Eupodidae	CNPKJ3306-14	KR070298
Trombidiformes	Eupodidae	CNPKJ3324-14	KR070311
Trombidiformes	Eupodidae	CNPKJ3325-14	KR070163
Trombidiformes	Eupodidae	CNPKK077-14	KR070228
Trombidiformes	Eupodidae	CNPKK1496-14	KR070102
Trombidiformes	Eupodidae	CNPKK1502-14	KR069473
Trombidiformes	Eupodidae	CNPKK1504-14	KR069667
Trombidiformes	Eupodidae	CNPKK1519-14	KR070564
Trombidiformes	Eupodidae	CNPKK1526-14	KR069509
Trombidiformes	Eupodidae	CNPKK1593-14	KR069574
Trombidiformes	Eupodidae	CNPKK1653-14	KR069754
Trombidiformes	Eupodidae	CNPKK1665-14	KR069920
Trombidiformes	Eupodidae	CNPKK1683-14	KR070565
Trombidiformes	Eupodidae	CNPKK1688-14	KR070697
Trombidiformes	Eupodidae	CNPKK1721-14	KR070028
Trombidiformes	Eupodidae	CNPKK1744-14	KR069626
Trombidiformes	Eupodidae	CNPKK1766-14	KR070568
Trombidiformes	Eupodidae	CNPKK1825-14	KR070721
Trombidiformes	Eupodidae	CNPKK1837-14	KR069763
Trombidiformes	Eupodidae	CNPKK1852-14	KR070032
Trombidiformes	Eupodidae	CNPKK1872-14	KR070178
Trombidiformes	Eupodidae	CNPKK1874-14	KR070305
Trombidiformes	Eupodidae	CNPKK1899-14	KR069742
Trombidiformes	Eupodidae	CNPKK1915-14	KR069446
Trombidiformes	Eupodidae	CNPKK1920-14	KR070391
Trombidiformes	Eupodidae	CNPKK1924-14	KR069746
Trombidiformes	Eupodidae	CNPKK1928-14	KR070306
Trombidiformes	Eupodidae	CNPKK1936-14	KR069930
Trombidiformes	Eupodidae	CNPKK1942-14	KR069790
Trombidiformes	Eupodidae	CNPKK1951-14	KR070319
Trombidiformes	Eupodidae	CNPKK1984-14	KR069397
Trombidiformes	Eupodidae	CNPKK1987-14	KR070315
Trombidiformes	Eupodidae	CNPKK1988-14	KR070688
Trombidiformes	Eupodidae	CNPKK1996-14	KR069376
Trombidiformes	Eupodidae	CNPKM1006-14	KR069899
Trombidiformes	Eupodidae	CNPKM1007-14	KR069378
Trombidiformes	Eupodidae	CNPKM1011-14	KR069335
Trombidiformes	Eupodidae	CNPKM1013-14	KR069837
Trombidiformes	Eupodidae	CNPKM917-14	KR069432
Trombidiformes	Eupodidae	CNPKO1243-14	KR070637
Trombidiformes	Eupodidae	CNPKO1245-14	KR069878
Trombidiformes	Eupodidae	CNPKO1251-14	KR069251
Trombidiformes	Eupodidae	CNPKO1254-14	KR069758
Trombidiformes	Eupodidae	CNPKO1257-14	KR069203
Trombidiformes	Eupodidae	CNPKO1267-14	KR070561
Trombidiformes	Eupodidae	CNPKO1269-14	KR070574
Trombidiformes	Eupodidae	CNPKO1271-14	KR069414
Trombidiformes	Eupodidae	CNPKO1290-14	KR069497
Trombidiformes	Eupodidae	CNPKO1308-14	KR070411
Trombidiformes	Eupodidae	CNPKO1314-14	KR070736
Trombidiformes	Eupodidae	CNPKO1322-14	KR070693

Trombidiformes	Eupodidae	CNPKO3957-14	KR069657
Trombidiformes	Eupodidae	CNPKO3959-14	KR070303
Trombidiformes	Eupodidae	CNPKO3980-14	KR070428
Trombidiformes	Eupodidae	CNPKO4001-14	KR070089
Trombidiformes	Eupodidae	CNPKO4003-14	KR069460
Trombidiformes	Eupodidae	CNPKO4005-14	KR070247
Trombidiformes	Eupodidae	CNPKO4006-14	KR070395
Trombidiformes	Eupodidae	CNPKO4007-14	KR070115
Trombidiformes	Eupodidae	CNPKO4019-14	KR070515
Trombidiformes	Eupodidae	CNPKO4020-14	KR070164
Trombidiformes	Eupodidae	CNPPA4109-12	KJ086010
Trombidiformes	Eupodidae	CNPPA4140-12	KJ084695
Trombidiformes	Eupodidae	CNPPC1928-12	KJ083605
Trombidiformes	Eupodidae	CNPPC1996-12	KJ085520
Trombidiformes	Eupodidae	CNSLJ025-12	KM832588
Trombidiformes	Eupodidae	CNSLK070-12	KM830356
Trombidiformes	Eupodidae	CNSLU272-13	KM840248
Trombidiformes	Eupodidae	GACAC433-12	GQ864397
Trombidiformes	Eupodidae	GACAC435-12	GQ864399
Trombidiformes	Eupodidae	MIONB125-10	KR070156
Trombidiformes	Eupodidae	MIONB126-10	KR070051
Trombidiformes	Eupodidae	MIONB127-10	KR070474
Trombidiformes	Eupodidae	MIONB128-10	KR069955
Trombidiformes	Eupodidae	MIONB129-10	KP979212
Trombidiformes	Eupodidae	MIONB173-10	KP979331
Trombidiformes	Eupodidae	MIONB192-10	KR069439
Trombidiformes	Eupodidae	MIONB193-10	KR069337
Trombidiformes	Eupodidae	MIONB194-10	KR069392
Trombidiformes	Eupodidae	MIONB229-10	KR069469
Trombidiformes	Eupodidae	MIONB230-10	KR070369
Trombidiformes	Eupodidae	MIONB231-10	KP979181
Trombidiformes	Eupodidae	MIONB232-10	KR070107
Trombidiformes	Eupodidae	MIONB261-10	KP979138
Trombidiformes	Eupodidae	MIONB273-10	KP979260
Trombidiformes	Eupodidae	MIONB274-10	KP979315
Trombidiformes	Eupodidae	MIONB281-10	KP979163
Trombidiformes	Eupodidae	MIONB282-10	KR070669
Trombidiformes	Eupodidae	MIONB283-10	KR069437
Trombidiformes	Eupodidae	MIONB284-10	KR070689
Trombidiformes	Eupodidae	MIONB290-10	KP979221
Trombidiformes	Eupodidae	MIONB308-10	KP979198
Trombidiformes	Eupodidae	MIONB310-10	KP979250
Trombidiformes	Eupodidae	MYMCA1054-11	JX833854
Trombidiformes	Eupodidae	MYMCA1081-11	JX838440
Trombidiformes	Eupodidae	MYMCA1082-11	JX835109
Trombidiformes	Eupodidae	MYMCA1083-11	JX834083
Trombidiformes	Eupodidae	MYMCA1102-11	JX834694
Trombidiformes	Eupodidae	MYMCA1103-11	JX837991
Trombidiformes	Eupodidae	MYMCA1104-11	JX838478
Trombidiformes	Eupodidae	MYMCA1117-11	JX838394
Trombidiformes	Eupodidae	MYMCA1118-11	JX838492
Trombidiformes	Eupodidae	MYMCA1119-11	JX835924
Trombidiformes	Eupodidae	MYMCA1167-11	JX838725

Trombidiformes	Eupodidae	MYMCA1169-11	JX834558
Trombidiformes	Eupodidae	MYMCA1170-11	JX836549
Trombidiformes	Eupodidae	MYMCA292-11	JX836670
Trombidiformes	Eupodidae	MYMCA359-11	JX834384
Trombidiformes	Eupodidae	MYMCA744-11	JX838015
Trombidiformes	Eupodidae	MYMCA758-11	JX838033
Trombidiformes	Eupodidae	MYMCA759-11	JX836971
Trombidiformes	Eupodidae	MYMCA769-11	JX834159
Trombidiformes	Eupodidae	MYMCA771-11	JX836792
Trombidiformes	Eupodidae	MYMCA782-11	JX837606
Trombidiformes	Eupodidae	MYMCA783-11	JX833677
Trombidiformes	Eupodidae	MYMCA785-11	JX835272
Trombidiformes	Eupodidae	MYMCA834-11	JX837244
Trombidiformes	Eupodidae	MYMCA845-11	JX837805
Trombidiformes	Eupodidae	MYMCA846-11	JX836821
Trombidiformes	Eupodidae	MYMCA848-11	JX834271
Trombidiformes	Eupodidae	MYMCA854-11	JX837676
Trombidiformes	Eupodidae	MYMCA877-11	JX837357
Trombidiformes	Eupodidae	MYMCB006-11	JX833962
Trombidiformes	Eupodidae	MYMCB177-11	JX837299
Trombidiformes	Eupodidae	MYMCB180-11	JX835495
Trombidiformes	Eupodidae	MYMCB185-11	JX837335
Trombidiformes	Eupodidae	MYMCB196-11	JX837478
Trombidiformes	Eupodidae	MYMCB197-11	JX835989
Trombidiformes	Eupodidae	MYMCB198-11	JX834824
Trombidiformes	Eupodidae	MYMCB199-11	JX837759
Trombidiformes	Eupodidae	MYMCB200-11	JX834233
Trombidiformes	Eupodidae	MYMCB201-11	JX834234
Trombidiformes	Eupodidae	MYMCB213-11	JX833792
Trombidiformes	Eupodidae	MYMCB214-11	JX836481
Trombidiformes	Eupodidae	MYMCB215-11	JX836503
Trombidiformes	Eupodidae	MYMCB216-11	JX835156
Trombidiformes	Eupodidae	MYMCB240-11	JX836343
Trombidiformes	Eupodidae	MYMCB241-11	JX834069
Trombidiformes	Eupodidae	MYMCB242-11	JX836411
Trombidiformes	Eupodidae	MYMCB251-11	JX835772
Trombidiformes	Eupodidae	MYMCB252-11	JX833809
Trombidiformes	Eupodidae	MYMCB457-11	JX837295
Trombidiformes	Eupodidae	MYMCB458-11	JX836336
Trombidiformes	Eupodidae	MYMCB472-11	JX834933
Trombidiformes	Eupodidae	MYMCB474-11	JX837794
Trombidiformes	Eupodidae	MYMCB486-11	JX837093
Trombidiformes	Eupodidae	MYMCB487-11	JX836247
Trombidiformes	Eupodidae	MYMCB530-11	JX838456
Trombidiformes	Eupodidae	MYMCB548-11	JX836156
Trombidiformes	Eupodidae	MYMCB553-11	JX836813
Trombidiformes	Eupodidae	MYMCB615-11	JX837108
Trombidiformes	Eupodidae	MYMCB631-11	JX838376
Trombidiformes	Eupodidae	MYMCB632-11	JX838008
Trombidiformes	Eupodidae	MYMCB633-11	JX836680
Trombidiformes	Eupodidae	MYMCB634-11	JX835297
Trombidiformes	Eupodidae	MYMCB635-11	JX836045
Trombidiformes	Eupodidae	MYMCB638-11	JX838004

Trombidiformes	Eupodidae	MYMCB640-11	JX838506
Trombidiformes	Eupodidae	MYMCB649-11	JX833759
Trombidiformes	Eupodidae	MYMCB651-11	JX835004
Trombidiformes	Eupodidae	MYMCB654-11	JX834036
Trombidiformes	Eupodidae	MYMCB660-11	JX836744
Trombidiformes	Eupodidae	MYMCB687-11	JX838617
Trombidiformes	Eupodidae	MYMCB755-11	JX837724
Trombidiformes	Eupodidae	MYMCB756-11	JX836472
Trombidiformes	Eupodidae	MYMCB757-11	JX834631
Trombidiformes	Eupodidae	MYMCB758-11	JX834716
Trombidiformes	Eupodidae	MYMCB789-11	JX833921
Trombidiformes	Eupodidae	MYMCB818-11	JX836875
Trombidiformes	Eupodidae	MYMCB895-11	JX835655
Trombidiformes	Eupodidae	MYMCB897-11	JX835489
Trombidiformes	Eupodidae	MYMCB898-11	JX835966
Trombidiformes	Eupodidae	MYMCB915-11	JX836360
Trombidiformes	Eupodidae	MYMCB916-11	JX834030
Trombidiformes	Eupodidae	MYMCB917-11	JX838782
Trombidiformes	Eupodidae	MYMCB918-11	JX834463
Trombidiformes	Eupodidae	MYMCB919-11	JX834686
Trombidiformes	Eupodidae	MYMCB920-11	JX834334
Trombidiformes	Eupodidae	MYMCC076-11	JX836580
Trombidiformes	Eupodidae	MYMCC077-11	JX838311
Trombidiformes	Eupodidae	MYMCC079-11	JX836999
Trombidiformes	Eupodidae	MYMCC109-11	JX836067
Trombidiformes	Eupodidae	MYMCC157-11	JX836736
Trombidiformes	Eupodidae	MYMCC158-11	JX834753
Trombidiformes	Eupodidae	MYMCC240-11	JX836694
Trombidiformes	Eupodidae	MYMCC368-11	JX836319
Trombidiformes	Eupodidae	MYMCC381-11	JX834268
Trombidiformes	Eupodidae	MYMCC382-11	JX836739
Trombidiformes	Eupodidae	MYMCC403-11	JX835073
Trombidiformes	Eupodidae	MYMCC414-11	JX835193
Trombidiformes	Eupodidae	MYMCC416-11	JX836464
Trombidiformes	Eupodidae	MYMCC456-11	JX838599
Trombidiformes	Eupodidae	MYMCC477-11	JX835139
Trombidiformes	Eupodidae	MYMCC527-11	JX834783
Trombidiformes	Eupodidae	MYMCC528-11	JX837038
Trombidiformes	Eupodidae	MYMCC529-11	JX833938
Trombidiformes	Eupodidae	MYMCC530-11	JX836768
Trombidiformes	Eupodidae	MYMCC540-11	JX834894
Trombidiformes	Eupodidae	MYMCC542-11	JX838196
Trombidiformes	Eupodidae	MYMCC558-11	JX837747
Trombidiformes	Eupodidae	MYMCC572-11	JX837642
Trombidiformes	Eupodidae	MYMCC573-11	JX836347
Trombidiformes	Eupodidae	MYMCC574-11	JX833803
Trombidiformes	Eupodidae	MYMCC576-11	JX835905
Trombidiformes	Eupodidae	MYMCC585-11	JX834490
Trombidiformes	Eupodidae	MYMCC587-11	JX837514
Trombidiformes	Eupodidae	MYMCC653-11	JX833681
Trombidiformes	Eupodidae	MYMCC698-11	JX835080
Trombidiformes	Eupodidae	MYMCC700-11	JX836316
Trombidiformes	Eupodidae	MYMCC707-11	JX835215

Trombidiformes	Eupodidae	MYMCC708-11	JX838047
Trombidiformes	Eupodidae	MYMCC709-11	JX833746
Trombidiformes	Eupodidae	MYMCC725-11	JX837318
Trombidiformes	Eupodidae	MYMCC729-11	JX834167
Trombidiformes	Eupodidae	MYMCC730-11	JX834570
Trombidiformes	Eupodidae	MYMCC741-11	JX836787
Trombidiformes	Eupodidae	MYMCC784-11	JX835144
Trombidiformes	Eupodidae	MYMCC834-11	JX837664
Trombidiformes	Eupodidae	MYMCC855-11	JX837264
Trombidiformes	Eupodidae	MYMCC923-11	JX837665
Trombidiformes	Eupodidae	MYMCC942-11	JX834736
Trombidiformes	Eupodidae	MYMCD008-11	JX834220
Trombidiformes	Eupodidae	MYMCD009-11	JX835977
Trombidiformes	Eupodidae	MYMCD010-11	JX835274
Trombidiformes	Eupodidae	MYMCD011-11	JX837048
Trombidiformes	Eupodidae	MYMCD012-11	JX835608
Trombidiformes	Eupodidae	MYMCD013-11	JX835066
Trombidiformes	Eupodidae	MYMCD015-11	JX836868
Trombidiformes	Eupodidae	MYMCD028-11	JX834017
Trombidiformes	Eupodidae	MYMCD029-11	JX837360
Trombidiformes	Eupodidae	MYMCD039-11	JX835246
Trombidiformes	Eupodidae	MYMCD042-11	JX836311
Trombidiformes	Eupodidae	MYMCD044-11	JX834391
Trombidiformes	Eupodidae	MYMCD045-11	JX834142
Trombidiformes	Eupodidae	MYMCD067-11	JX834250
Trombidiformes	Eupodidae	MYMCD069-11	JX837144
Trombidiformes	Eupodidae	MYMCD085-11	JX834357
Trombidiformes	Eupodidae	MYMCD087-11	JX836524
Trombidiformes	Eupodidae	MYMCD102-11	JX836232
Trombidiformes	Eupodidae	MYMCD103-11	JX837571
Trombidiformes	Eupodidae	MYMCD105-11	JX833636
Trombidiformes	Eupodidae	MYMCD108-11	JX834807
Trombidiformes	Eupodidae	MYMCD122-11	JX836898
Trombidiformes	Eupodidae	MYMCD123-11	JX833801
Trombidiformes	Eupodidae	MYMCD162-11	JX836969
Trombidiformes	Eupodidae	MYMCD163-11	JX836633
Trombidiformes	Eupodidae	MYMCD164-11	JX834953
Trombidiformes	Eupodidae	MYMCD180-11	JX835474
Trombidiformes	Eupodidae	MYMCE133-12	JX833654
Trombidiformes	Eupodidae	MYMCE135-12	JX838430
Trombidiformes	Eupodidae	MYMCE149-12	JX834065
Trombidiformes	Eupodidae	MYMCE170-12	JX837008
Trombidiformes	Eupodidae	MYMCE288-12	JX834440
Trombidiformes	Eupodidae	MYMCE407-12	JX837431
Trombidiformes	Eupodidae	MYMCE438-12	JX835336
Trombidiformes	Eupodidae	MYMCE440-12	JX834001
Trombidiformes	Eupodidae	MYMCE561-12	JX837402
Trombidiformes	Eupodidae	MYMCE563-12	JX836758
Trombidiformes	Eupodidae	MYMCE566-12	JX837532
Trombidiformes	Eupodidae	MYMCE567-12	JX834920
Trombidiformes	Eupodidae	MYMCE578-12	JX836934
Trombidiformes	Eupodidae	MYMCE595-12	JX838497
Trombidiformes	Eupodidae	MYMCE612-12	JX837367

Trombidiformes	Eupodidae	MYMCE632-12	JX836730
Trombidiformes	Eupodidae	MYMCE715-12	JX837394
Trombidiformes	Eupodidae	MYMCE758-12	JX835392
Trombidiformes	Eupodidae	MYMCE788-12	JX837266
Trombidiformes	Eupodidae	MYMCE790-12	JX834799
Trombidiformes	Eupodidae	MYMCE791-12	JX837896
Trombidiformes	Eupodidae	MYMCE822-12	JX837396
Trombidiformes	Eupodidae	MYMCE910-12	JX834161
Trombidiformes	Eupodidae	MYMCE911-12	JX837273
Trombidiformes	Eupodidae	MYMCE936-12	JX836847
Trombidiformes	Eupodidae	MYMCE937-12	JX836890
Trombidiformes	Eupodidae	MYMCF031-12	JX835017
Trombidiformes	Eupodidae	MYMCF096-12	JX835103
Trombidiformes	Eupodidae	MYMCF100-12	JX838620
Trombidiformes	Eupodidae	MYMCF103-12	JX835387
Trombidiformes	Eupodidae	MYMCF155-12	JX837294
Trombidiformes	Eupodidae	MYMCF172-12	JX834878
Trombidiformes	Eupodidae	MYMCF218-12	JX836025
Trombidiformes	Eupodidae	MYMCF229-12	JX837949
Trombidiformes	Eupodidae	MYMCF230-12	JX837218
Trombidiformes	Eupodidae	MYMCF275-12	JX838765
Trombidiformes	Eupodidae	MYMCF465-12	JX835660
Trombidiformes	Eupodidae	MYMCF468-12	JX838120
Trombidiformes	Eupodidae	MYMCF488-12	JX834114
Trombidiformes	Eupodidae	MYMCF489-12	JX837084
Trombidiformes	Eupodidae	MYMCF493-12	JX835053
Trombidiformes	Eupodidae	MYMCF494-12	JX836722
Trombidiformes	Eupodidae	MYMCF571-12	JX836938
Trombidiformes	Eupodidae	MYMCF573-12	JX837153
Trombidiformes	Eupodidae	MYMCF586-12	JX837910
Trombidiformes	Eupodidae	MYMCF596-12	JX838583
Trombidiformes	Eupodidae	MYMCF598-12	JX836859
Trombidiformes	Eupodidae	MYMCF603-12	JX835101
Trombidiformes	Eupodidae	MYMCF631-12	JX838358
Trombidiformes	Eupodidae	MYMCF632-12	JX835285
Trombidiformes	Eupodidae	MYMCF673-12	JX837900
Trombidiformes	Eupodidae	MYMCF753-12	JX836822
Trombidiformes	Eupodidae	MYMCF797-12	JX835294
Trombidiformes	Eupodidae	MYMCF924-12	JX838481
Trombidiformes	Eupodidae	MYMCG093-12	JX838260
Trombidiformes	Eupodidae	MYMCG107-12	JX836754
Trombidiformes	Eupodidae	MYMCG152-12	JX838375
Trombidiformes	Eupodidae	MYMCG177-12	JX837298
Trombidiformes	Eupodidae	MYMCG180-12	JX834435
Trombidiformes	Eupodidae	MYMCG190-12	JX834348
Trombidiformes	Eupodidae	MYMCG271-12	JX837149
Trombidiformes	Eupodidae	MYMCG280-12	JX836836
Trombidiformes	Eupodidae	MYMCG298-12	JX836927
Trombidiformes	Eupodidae	MYMCG300-12	JX836592
Trombidiformes	Eupodidae	MYMCG301-12	JX838565
Trombidiformes	Eupodidae	MYMCG387-12	JX834042
Trombidiformes	Eupodidae	MYMCG398-12	JX837956
Trombidiformes	Eupodidae	MYMCG400-12	JX837832

Trombidiformes	Eupodidae	MYMCG401-12	JX835552
Trombidiformes	Eupodidae	MYMCG402-12	JX835396
Trombidiformes	Eupodidae	MYMCG404-12	JX833787
Trombidiformes	Eupodidae	MYMCG406-12	JX834154
Trombidiformes	Eupodidae	MYMCG570-12	JX836782
Trombidiformes	Eupodidae	MYMCG583-12	JX836114
Trombidiformes	Eupodidae	MYMCG628-12	JX837259
Trombidiformes	Eupodidae	MYMCG648-12	JX837112
Trombidiformes	Eupodidae	MYTMC013-09	HQ966222
Trombidiformes	Eupodidae	MYTMC042-09	KR069631
Trombidiformes	Eupodidae	MYTMC092-09	HQ966234
Trombidiformes	Eupodidae	MYTMC104-09	HQ966235
Trombidiformes	Eupodidae	MYTMC153-09	HQ966241
Trombidiformes	Eupodidae	NCCE009-11	KP979339
Trombidiformes	Eupodidae	RBINA1191-13	KP979188
Trombidiformes	Eupodidae	RBINA1193-13	KR070202
Trombidiformes	Eupodidae	RBINA1195-13	KP979341
Trombidiformes	Eupodidae	RBINA5644-13	KR069380
Trombidiformes	Eupodidae	RBINA5645-13	KR070106
Trombidiformes	Eupodidae	RBINA5650-13	KR069565
Trombidiformes	Eupodidae	RBINA5651-13	KR069482
Trombidiformes	Eupodidae	RBINA5652-13	KR070469
Trombidiformes	Eupodidae	RBINA5659-13	KR070264
Trombidiformes	Eupodidae	RBINA5688-13	KR069152
Trombidiformes	Eupodidae	RBINA5697-13	KR069338
Trombidiformes	Eupodidae	RBINA5698-13	KR069979
Trombidiformes	Eupodidae	RBINA5699-13	KP979134
Trombidiformes	Eupodidae	RBINA5707-13	KR070177
Trombidiformes	Eupodidae	RBINA5710-13	KP979135
Trombidiformes	Eupodidae	RBINA5741-13	KR069747
Trombidiformes	Eupodidae	RBINA5743-13	KR070560
Trombidiformes	Eupodidae	RBINA5747-13	KR070079
Trombidiformes	Eupodidae	RBINA5777-13	KR070027
Trombidiformes	Eupodidae	RBINA5789-13	KP979139
Trombidiformes	Eupodidae	RBINA5790-13	KP979207
Trombidiformes	Eupodidae	SSBAA3673-12	KM833521
Trombidiformes	Eupodidae	SSBAB108-12	KM830965
Trombidiformes	Eupodidae	SSBAB110-12	KM839164
Trombidiformes	Eupodidae	SSBAB1136-12	KM831512
Trombidiformes	Eupodidae	SSBAB117-12	KM835788
Trombidiformes	Eupodidae	SSBAB129-12	KM839347
Trombidiformes	Eupodidae	SSBAB131-12	KM834720
Trombidiformes	Eupodidae	SSBAB1950-12	KM835074
Trombidiformes	Eupodidae	SSBAB1952-12	KM832073
Trombidiformes	Eupodidae	SSBAD3069-12	KM835924
Trombidiformes	Eupodidae	SSBAD3991-12	KM830446
Trombidiformes	Eupodidae	SSBAD4005-12	KM840697
Trombidiformes	Eupodidae	SSBAD4011-12	KM839841
Trombidiformes	Eupodidae	SSBAD4016-12	KM838569
Trombidiformes	Eupodidae	SSBAD4033-12	KM836253
Trombidiformes	Eupodidae	SSBAD4035-12	KM839060
Trombidiformes	Eupodidae	SSBAD4036-12	KM825991
Trombidiformes	Eupodidae	SSBAD4717-13	KM840590

Trombidiformes	Eupodidae	SSBAD4725-13	KM832719
Trombidiformes	Eupodidae	SSBAD4727-13	KM839436
Trombidiformes	Eupodidae	SSBAD4728-13	KM828226
Trombidiformes	Eupodidae	SSBAD4749-13	KM839652
Trombidiformes	Eupodidae	SSBAD4777-13	KM824463
Trombidiformes	Eupodidae	SSBAD5515-13	KM827162
Trombidiformes	Eupodidae	SSBAD5530-13	KM824957
Trombidiformes	Eupodidae	SSBAD5539-13	KM824854
Trombidiformes	Eupodidae	SSBAD5556-13	KM831581
Trombidiformes	Eupodidae	SSBAD5564-13	KM840693
Trombidiformes	Eupodidae	SSBAD5583-13	KM840124
Trombidiformes	Eupodidae	SSBAD6315-13	KM825302
Trombidiformes	Eupodidae	SSBAD6340-13	KM824370
Trombidiformes	Eupodidae	SSBAE3770-13	KM828227
Trombidiformes	Eupodidae	SSBAE3811-13	KM835540
Trombidiformes	Eupodidae	SSEIA3007-13	KM839957
Trombidiformes	Eupodidae	SSEIA3012-13	KM824743
Trombidiformes	Eupodidae	SSEIA3019-13	KM829268
Trombidiformes	Eupodidae	SSEIA3024-13	KM838870
Trombidiformes	Eupodidae	SSEIB7648-13	KM828446
Trombidiformes	Eupodidae	SSEIB7652-13	KM834679
Trombidiformes	Eupodidae	SSEIB7658-13	KM826870
Trombidiformes	Eupodidae	SSEIB7662-13	KM828972
Trombidiformes	Eupodidae	SSEIB7667-13	KM840577
Trombidiformes	Eupodidae	SSEIB7668-13	KM835805
Trombidiformes	Eupodidae	SSEIB7670-13	KM829502
Trombidiformes	Eupodidae	SSEIB7674-13	KM824229
Trombidiformes	Eupodidae	SSEID052-13	KM826781
Trombidiformes	Eupodidae	SSEID054-13	KM825697
Trombidiformes	Eupodidae	SSEID063-13	KM827378
Trombidiformes	Eupodidae	SSJAB1844-13	KM832280
Trombidiformes	Eupodidae	SSJAB1846-13	KM824066
Trombidiformes	Eupodidae	SSJAB1851-13	KM833185
Trombidiformes	Eupodidae	SSJAB1855-13	KM836384
Trombidiformes	Eupodidae	SSJAB1858-13	KM840537
Trombidiformes	Eupodidae	SSJAB1860-13	KM838442
Trombidiformes	Eupodidae	SSJAB1862-13	KM836509
Trombidiformes	Eupodidae	SSJAB1864-13	KM835318
Trombidiformes	Eupodidae	SSJAB1873-13	KM833770
Trombidiformes	Eupodidae	SSJAB1874-13	KM838194
Trombidiformes	Eupodidae	SSJAB1876-13	KM839681
Trombidiformes	Eupodidae	SSJAB1878-13	KM826872
Trombidiformes	Eupodidae	SSJAB1886-13	KM830885
Trombidiformes	Eupodidae	SSJAB1888-13	KM829905
Trombidiformes	Eupodidae	SSJAB1891-13	KM838873
Trombidiformes	Eupodidae	SSJAB1892-13	KM836943
Trombidiformes	Eupodidae	SSJAB1893-13	KM837764
Trombidiformes	Eupodidae	SSJAB1897-13	KM833927
Trombidiformes	Eupodidae	SSJAB1899-13	KM831085
Trombidiformes	Eupodidae	SSJAB1901-13	KM827722
Trombidiformes	Eupodidae	SSJAB1902-13	KM829428
Trombidiformes	Eupodidae	SSJAB1911-13	KM836667
Trombidiformes	Eupodidae	SSJAB2117-13	KM832496

Trombidiformes	Eupodidae	SSJAB2120-13	KM824778
Trombidiformes	Eupodidae	SSJAB2121-13	KM833130
Trombidiformes	Eupodidae	SSJAB2122-13	KM833929
Trombidiformes	Eupodidae	SSJAB2123-13	KM836177
Trombidiformes	Eupodidae	SSJAB2127-13	KM824168
Trombidiformes	Eupodidae	SSJAB2128-13	KM825223
Trombidiformes	Eupodidae	SSJAB2138-13	KM831556
Trombidiformes	Eupodidae	SSJAB2140-13	KM839264
Trombidiformes	Eupodidae	SSJAB2148-13	KM831022
Trombidiformes	Eupodidae	SSJAB2152-13	KM825224
Trombidiformes	Eupodidae	SSJAB2154-13	KM837305
Trombidiformes	Eupodidae	SSJAB2159-13	KM826043
Trombidiformes	Eupodidae	SSJAB2168-13	KM827432
Trombidiformes	Eupodidae	SSJAB2169-13	KM837095
Trombidiformes	Eupodidae	SSJAB2171-13	KM835436
Trombidiformes	Eupodidae	SSJAB2172-13	KM824961
Trombidiformes	Eupodidae	SSJAB2183-13	KM827707
Trombidiformes	Eupodidae	SSJAB2187-13	KM830323
Trombidiformes	Eupodidae	SSJAB2191-13	KM839168
Trombidiformes	Eupodidae	SSJAB2193-13	KM832273
Trombidiformes	Eupodidae	SSJAB2196-13	KM838832
Trombidiformes	Eupodidae	SSJAB2197-13	KM833972
Trombidiformes	Eupodidae	SSJAB2198-13	KM835247
Trombidiformes	Eupodidae	SSJAB2206-13	KM831239
Trombidiformes	Eupodidae	SSJAC1515-13	KM832702
Trombidiformes	Eupodidae	SSJAC1516-13	KM837649
Trombidiformes	Eupodidae	SSJAC1521-13	KM839210
Trombidiformes	Eupodidae	SSJAC1526-13	KM835740
Trombidiformes	Eupodidae	SSJAC1530-13	KM834542
Trombidiformes	Eupodidae	SSJAC1532-13	KM824660
Trombidiformes	Eupodidae	SSJAC1534-13	KM836281
Trombidiformes	Eupodidae	SSJAC1541-13	KM840682
Trombidiformes	Eupodidae	SSJAC1544-13	KM834051
Trombidiformes	Eupodidae	SSJAC1545-13	KM834885
Trombidiformes	Eupodidae	SSJAC1552-13	KM839838
Trombidiformes	Eupodidae	SSJAC1554-13	KM828439
Trombidiformes	Eupodidae	SSJAC1556-13	KM836168
Trombidiformes	Eupodidae	SSJAC1557-13	KM838730
Trombidiformes	Eupodidae	SSJAC1559-13	KM826655
Trombidiformes	Eupodidae	SSJAC1562-13	KM828265
Trombidiformes	Eupodidae	SSJAC1564-13	KM838620
Trombidiformes	Eupodidae	SSJAC1565-13	KM826431
Trombidiformes	Eupodidae	SSJAC1567-13	KM830879
Trombidiformes	Eupodidae	SSJAC1569-13	KM834153
Trombidiformes	Eupodidae	SSJAC1574-13	KM832219
Trombidiformes	Eupodidae	SSJAC1575-13	KM830599
Trombidiformes	Eupodidae	SSJAC1577-13	KM832711
Trombidiformes	Eupodidae	SSJAC1580-13	KM838375
Trombidiformes	Eupodidae	SSJAC1583-13	KM837754
Trombidiformes	Eupodidae	SSJAC1586-13	KM830078
Trombidiformes	Eupodidae	SSJAC1587-13	KM832180
Trombidiformes	Eupodidae	SSJAC1590-13	KM839396
Trombidiformes	Eupodidae	SSJAC1593-13	KM828781

Trombidiformes	Eupodidae	SSJAC1594-13	KM826544
Trombidiformes	Eupodidae	SSJAC1595-13	KM832692
Trombidiformes	Eupodidae	SSJAC1597-13	KM839660
Trombidiformes	Eupodidae	SSJAC1598-13	KM824178
Trombidiformes	Eupodidae	SSJAC1600-13	KM839519
Trombidiformes	Eupodidae	SSJAC1602-13	KM830464
Trombidiformes	Eupodidae	SSJAC974-13	KM836226
Trombidiformes	Eupodidae	SSJAC978-13	KM830784
Trombidiformes	Eupodidae	SSJAC993-13	KM830827
Trombidiformes	Eupodidae	SSJAD3369-13	KM829574
Trombidiformes	Eupodidae	SSJAF5572-13	KM830071
Trombidiformes	Eupodidae	SSJAF7320-13	KM836205
Trombidiformes	Eupodidae	SSJAF8356-13	KM838573
Trombidiformes	Eupodidae	SSJAF8457-13	KM826093
Trombidiformes	Eupodidae	SSJAF8637-13	KM837526
Trombidiformes	Eupodidae	SSJAF8638-13	KM824948
Trombidiformes	Eupodidae	SSPAA2224-13	KM839981
Trombidiformes	Eupodidae	SSPAA2231-13	KM826073
Trombidiformes	Eupodidae	SSPAA7990-13	KM829527
Trombidiformes	Eupodidae	SSPAB3971-13	KM824603
Trombidiformes	Eupodidae	SSPAB4002-13	KM828945
Trombidiformes	Eupodidae	SSPAB983-13	KM831971
Trombidiformes	Eupodidae	SSPAB988-13	KM825090
Trombidiformes	Eupodidae	SSPAC10887-13	KM837794
Trombidiformes	Eupodidae	SSPAC10910-13	KM837129
Trombidiformes	Eupodidae	SSPAC10917-13	KM835131
Trombidiformes	Eupodidae	SSPAC11066-13	KM827250
Trombidiformes	Eupodidae	SSPAC11101-13	KM828932
Trombidiformes	Eupodidae	SSPAC11102-13	KM829834
Trombidiformes	Eupodidae	SSPAC11107-13	KM839706
Trombidiformes	Eupodidae	SSPAC7880-13	KM825204
Trombidiformes	Eupodidae	SSPAC7892-13	KM830404
Trombidiformes	Eupodidae	SSWLA5261-13	KM828303
Trombidiformes	Eupodidae	SSWLA5262-13	KM840169
Trombidiformes	Eupodidae	SSWLA5270-13	KM828047
Trombidiformes	Eupodidae	SSWLA5279-13	KM834026
Trombidiformes	Eupodidae	SSWLA5280-13	KM830612
Trombidiformes	Eupodidae	SSWLA5291-13	KM826798
Trombidiformes	Eupodidae	SSWLA5299-13	KM827256
Trombidiformes	Eupodidae	SSWLA5300-13	KM827197
Trombidiformes	Eupodidae	SSWLA5303-13	KM840408
Trombidiformes	Eupodidae	SSWLA5313-13	KM827854
Trombidiformes	Eupodidae	SSWLA5314-13	KM839639
Trombidiformes	Eupodidae	SSWLA5315-13	KM832756
Trombidiformes	Eupodidae	SSWLA5316-13	KM836367
Trombidiformes	Eupodidae	SSWLA5318-13	KM829082
Trombidiformes	Eupodidae	SSWLA5319-13	KM836467
Trombidiformes	Eupodidae	SSWLA5321-13	KM828331
Trombidiformes	Eupodidae	SSWLA5322-13	KM829647
Trombidiformes	Eupodidae	SSWLA5325-13	KM838390
Trombidiformes	Eupodidae	SSWLA5327-13	KM825301
Trombidiformes	Eupodidae	SSWLA5329-13	KM835728
Trombidiformes	Eupodidae	SSWLA5333-13	KM825467

Trombidiformes	Eupodidae		SSWLA5341-13	KM840379
Trombidiformes	Eupodidae		SSWLA5344-13	KM832914
Trombidiformes	Eupodidae		SSWLB2496-13	KM827865
Trombidiformes	Eupodidae		SSWLC3118-13	KM824864
Trombidiformes	Eupodidae		SSWLC4151-13	KM838323
Trombidiformes	Eupodidae		SSWLD2912-13	KM833466
Trombidiformes	Eupodidae		SSWLD2942-13	KM827112
Trombidiformes	Eupodidae		SSWLD2944-13	KM834337
Trombidiformes	Eupodidae		SSWLD3519-13	KM834276
Trombidiformes	Eupodidae		SSWLE2866-13	KM825666
Trombidiformes	Eupodidae		SSWLE2874-13	KM830023
Trombidiformes	Eupodidae		SSWLE3181-13	KM833938
Trombidiformes	Eupodidae		SSWLE3182-13	KM836995
Trombidiformes	Eupodidae		SSWLE470-13	KM832768
Trombidiformes	Eupodidae		SSWLE480-13	KM837617
Trombidiformes	Eupodidae		SSWLF3640-13	KM840798
Trombidiformes	Eupodidae		SSWLF3651-13	KM825952
Trombidiformes	Eupodidae		SSWLF3661-13	KM824114
Trombidiformes	Eupodidae		SSWLF3898-13	KM837539
Trombidiformes	Eupodidae		SSWLF3901-13	KM834189
Trombidiformes	Eupodidae		SSWLF3904-13	KM827788
Trombidiformes	Eupodidae		SSWLF3912-13	KR069400
Trombidiformes	Eupodidae		SSWLF3921-13	KM839995
Trombidiformes	Eupodidae		SSWLF3934-13	KM836291
Trombidiformes	Eylaidae	*Eylais*	ARCN043-10	HQ924279
Trombidiformes	Eylaidae	*Eylais*	ARSO204-08	KM828553
Trombidiformes	Eylaidae	*Eylais*	MIONB522-11	KM838710
Trombidiformes	Eylaidae	*Eylais*	MIONB528-11	KM826061
Trombidiformes	Eylaidae	*Eylais*	MIONB529-11	KM838933
Trombidiformes	Eylaidae	*Eylais*	MIONB531-11	KM836347
Trombidiformes	Eylaidae	*Eylais*	MYMCA101-11	JX833906
Trombidiformes	Eylaidae	*Eylais*	MYMCA102-11	JX836526
Trombidiformes	Eylaidae	*Eylais sp. Ey2*	GACAC043-12	AB530317
Trombidiformes	Hydrachnidae		MIONB521-11	KM828091
Trombidiformes	Hydrachnidae		MIONB523-11	KM829829
Trombidiformes	Hydrachnidae		MIONB524-11	KM824435
Trombidiformes	Hydrachnidae		MIONB525-11	KM839004
Trombidiformes	Hydrachnidae		MIONB526-11	KM830307
Trombidiformes	Hydrachnidae		MIONB530-11	KM838199
Trombidiformes	Hydryphantidae	*Hydryphantes*	MYMCB689-11	JX838402
Trombidiformes	Hydryphantidae	*Hydryphantes*	MYMCE357-12	JX835377
Trombidiformes	Hydryphantidae	*Hydryphantes*	MYMCE358-12	JX836743
Trombidiformes	Hydryphantidae	*Hydryphantes*	SSJAE5668-13	KM834895
Trombidiformes	Hydryphantidae	*Hydryphantes sp.*	GBA17686-14	KF000207
Trombidiformes	Hydryphantidae	*Todothyas*	MYMCB451-11	JX837770
Trombidiformes	Hydryphantidae	*Todothyas*	MYMCB452-11	JX833812
Trombidiformes	Hydryphantidae	*Todothyas*	MYMCE125-12	JX837306
Trombidiformes	Hydryphantidae		ARCN137-10	HQ924349
Trombidiformes	Hydryphantidae		PHAUG1675-11	KP979119
Trombidiformes	Hydryphantidae		PHAUG1679-11	KR070484
Trombidiformes	Hydryphantidae		PHAUG1681-11	KR069160
Trombidiformes	Hydryphantidae		SSEIB4245-13	KM824216
Trombidiformes	Hydryphantidae		SSJAE5650-13	KM835334

Trombidiformes	Hygrobatidae	*Atractides*	CFWIA186-10	HQ938548
Trombidiformes	Hygrobatidae	*Atractides*	CFWIA187-10	HQ938549
Trombidiformes	Hygrobatidae	*Atractides*	CFWIA188-10	HQ938550
Trombidiformes	Hygrobatidae	*Atractides*	CFWIA189-10	HQ938551
Trombidiformes	Hygrobatidae	*Atractides*	CFWIA618-10	HQ938805
Trombidiformes	Hygrobatidae	*Atractides*	CFWIA619-10	KR070405
Trombidiformes	Hygrobatidae	*Atractides*	CFWIA620-10	HQ938806
Trombidiformes	Hygrobatidae	*Atractides*	CFWIA621-10	KR070595
Trombidiformes	Hygrobatidae	*Atractides*	CFWIA622-10	KR070068
Trombidiformes	Hygrobatidae	*Atractides*	CFWIB561-10	HQ939593
Trombidiformes	Hygrobatidae	*Atractides*	CFWIB562-10	HQ939594
Trombidiformes	Hygrobatidae	*Atractides*	CFWIB563-10	HQ939595
Trombidiformes	Hygrobatidae	*Atractides*	CFWIB564-10	KR069781
Trombidiformes	Hygrobatidae	*Atractides*	CFWIB565-10	HQ939596
Trombidiformes	Hygrobatidae	*Atractides latisetus*	GBCH1840-07	EF633505
Trombidiformes	Hygrobatidae	*Atractides sp. At3*	GACAC044-12	AB530318
Trombidiformes	Hygrobatidae	*Hygrobates*	MYMCA100-11	JX834050
Trombidiformes	Hygrobatidae	*Hygrobates fluviatilis*	GACAC001-12	AB530275
Trombidiformes	Hygrobatidae	*Hygrobates fluviatilis*	GACAC002-12	AB530276
Trombidiformes	Hygrobatidae	*Hygrobates fluviatilis*	GACAC003-12	AB530277
Trombidiformes	Hygrobatidae	*Hygrobates fluviatilis*	GACAC004-12	AB530278
Trombidiformes	Hygrobatidae	*Hygrobates fluviatilis*	GACAC005-12	AB530279
Trombidiformes	Hygrobatidae	*Hygrobates fluviatilis*	GACAC006-12	AB530280
Trombidiformes	Hygrobatidae	*Hygrobates fluviatilis*	GACAC007-12	AB530281
Trombidiformes	Hygrobatidae	*Hygrobates fluviatilis*	GACAC008-12	AB530282
Trombidiformes	Hygrobatidae	*Hygrobates fluviatilis*	GACAC009-12	AB530283
Trombidiformes	Hygrobatidae	*Hygrobates fluviatilis*	GACAC010-12	AB530284
Trombidiformes	Hygrobatidae	*Hygrobates fluviatilis*	GACAC011-12	AB530285
Trombidiformes	Hygrobatidae	*Hygrobates fluviatilis*	GACAC012-12	AB530286
Trombidiformes	Hygrobatidae	*Hygrobates fluviatilis*	GACAC013-12	AB530287
Trombidiformes	Hygrobatidae	*Hygrobates fluviatilis*	GACAC014-12	AB530288
Trombidiformes	Hygrobatidae	*Hygrobates fluviatilis*	GACAC015-12	AB530289
Trombidiformes	Hygrobatidae	*Hygrobates fluviatilis*	GACAC016-12	AB530290
Trombidiformes	Hygrobatidae	*Hygrobates fluviatilis*	GACAC017-12	AB530291
Trombidiformes	Hygrobatidae	*Hygrobates fluviatilis*	GACAC018-12	AB530292
Trombidiformes	Hygrobatidae	*Hygrobates fluviatilis*	GACAC019-12	AB530293
Trombidiformes	Hygrobatidae	*Hygrobates fluviatilis*	GACAC020-12	AB530294
Trombidiformes	Hygrobatidae	*Hygrobates fluviatilis*	GACAC021-12	AB530295
Trombidiformes	Hygrobatidae	*Hygrobates fluviatilis*	GACAC022-12	AB530296
Trombidiformes	Hygrobatidae	*Hygrobates fluviatilis*	GACAC023-12	AB530297
Trombidiformes	Hygrobatidae	*Hygrobates fluviatilis*	GACAC024-12	AB530298
Trombidiformes	Hygrobatidae	*Hygrobates fluviatilis*	GACAC025-12	AB530299
Trombidiformes	Hygrobatidae	*Hygrobates fluviatilis*	GACAC026-12	AB530300
Trombidiformes	Hygrobatidae	*Hygrobates fluviatilis*	GACAC027-12	AB530301
Trombidiformes	Hygrobatidae	*Hygrobates fluviatilis*	GACAC028-12	AB530302
Trombidiformes	Hygrobatidae	*Hygrobates fluviatilis*	GACAC029-12	AB530303
Trombidiformes	Hygrobatidae	*Hygrobates fluviatilis*	GACAC030-12	AB530304
Trombidiformes	Hygrobatidae	*Hygrobates fluviatilis*	GACAC031-12	AB530305
Trombidiformes	Hygrobatidae	*Hygrobates fluviatilis*	GACAC032-12	AB530306
Trombidiformes	Hygrobatidae	*Hygrobates fluviatilis*	GACAC033-12	AB530307
Trombidiformes	Hygrobatidae	*Hygrobates fluviatilis*	GACAC034-12	AB530308
Trombidiformes	Hygrobatidae	*Hygrobates fluviatilis*	GACAC035-12	AB530309
Trombidiformes	Hygrobatidae	*Hygrobates fluviatilis*	GACAC036-12	AB530310

Trombidiformes	Hygrobatidae	*Hygrobates fluviatilis*	GACAC037-12	AB530311
Trombidiformes	Hygrobatidae	*Hygrobates fluviatilis*	GACAC038-12	AB530312
Trombidiformes	Hygrobatidae	*Hygrobates fluviatilis*	GACAC039-12	AB530313
Trombidiformes	Hygrobatidae	*Hygrobates fluviatilis*	GBCH5184-10	FJ668595
Trombidiformes	Hygrobatidae	*Hygrobates fluviatilis*	GBCH5185-10	FJ668594
Trombidiformes	Hygrobatidae	*Hygrobates fluviatilis*	GBCH5186-10	FJ668593
Trombidiformes	Hygrobatidae	*Hygrobates fluviatilis*	GBCH5187-10	FJ668592
Trombidiformes	Hygrobatidae	*Hygrobates fluviatilis*	GBCH5188-10	FJ668591
Trombidiformes	Hygrobatidae	*Hygrobates fluviatilis*	GBCH5189-10	FJ668590
Trombidiformes	Hygrobatidae	*Hygrobates fluviatilis*	GBCH5190-10	FJ668589
Trombidiformes	Hygrobatidae	*Hygrobates fluviatilis*	GBCH5191-10	FJ668588
Trombidiformes	Hygrobatidae	*Hygrobates fluviatilis*	GBCH5192-10	FJ668587
Trombidiformes	Hygrobatidae	*Hygrobates fluviatilis*	GBCH5193-10	FJ668586
Trombidiformes	Lebertiidae	*Lebertia*	BBLZI110-14	KR070377
Trombidiformes	Lebertiidae	*Lebertia*	CFWIA164-10	HQ938528
Trombidiformes	Lebertiidae	*Lebertia*	CFWIA165-10	HQ938529
Trombidiformes	Lebertiidae	*Lebertia*	CFWIA166-10	HQ938530
Trombidiformes	Lebertiidae	*Lebertia*	CFWIA167-10	KR070660
Trombidiformes	Lebertiidae	*Lebertia*	CFWIA642-10	HQ938821
Trombidiformes	Lebertiidae	*Lebertia*	CFWIA643-10	HQ938822
Trombidiformes	Lebertiidae	*Lebertia*	JSMAY1598-12	KP979219
Trombidiformes	Lebertiidae	*Lebertia*	PMWMC005-07	KR070606
Trombidiformes	Lebertiidae	*Lebertia*	RBINA5582-13	KP979189
Trombidiformes	Lebertiidae	*Lebertia*	SSBAF2267-13	KM825233
Trombidiformes	Lebertiidae	*Lebertia*	SSBAF2291-13	KM840164
Trombidiformes	Lebertiidae	*Lebertia*	SSBAF2293-13	KM838614
Trombidiformes	Lebertiidae	*Lebertia*	SSPAA6640-13	KM827742
Trombidiformes	Lebertiidae	*Lebertia sp.*	MBIOD1186-13	KR069484
Trombidiformes	Lebertiidae	*Lebertia sp.*	MBIOD1193-13	KR070650
Trombidiformes	Lebertiidae	*Lebertia sp.*	MBIOD1197-13	KR070457
Trombidiformes	Lebertiidae	*Lebertia sp.*	MBIOD1198-13	KR069269
Trombidiformes	Lebertiidae	*Lebertia sp.*	MBIOD1200-13	KR069537
Trombidiformes	Lebertiidae	*Lebertia sp.*	MBIOD1203-13	KR069773
Trombidiformes	Lebertiidae	*Lebertia*	CNNHB2928-14	KR070530
Trombidiformes	Lebertiidae	*Lebertia*	CNNHB2933-14	KR069992
Trombidiformes	Limnesiidae	*Limnesia*	BBLZI091-14	KR070049
Trombidiformes	Limnesiidae	*Limnesia*	CNPAD554-13	KM837294
Trombidiformes	Limnesiidae	*Limnesia*	CNPPF1000-12	KJ163747
Trombidiformes	Limnesiidae	*Limnesia*	CNPPF578-12	KJ166832
Trombidiformes	Limnesiidae	*Limnesia*	CNPPG818-12	KJ165513
Trombidiformes	Limnesiidae	*Limnesia*	CNPPI1215-12	KJ445096
Trombidiformes	Limnesiidae	*Limnesia*	CNPPI1260-12	KJ207601
Trombidiformes	Limnesiidae	*Limnesia*	CNPPI1295-12	KJ445356
Trombidiformes	Limnesiidae	*Limnesia*	CNPPJ1872-12	KJ208985
Trombidiformes	Limnesiidae	*Limnesia*	SSBAF2277-13	KM824097
Trombidiformes	Limnesiidae	*Limnesia*	SSJAE1649-13	KM829058
Trombidiformes	Limnesiidae	*Limnesia*	SSPAA2255-13	KM829634
Trombidiformes	Limnesiidae	*Limnesia*	SSPAA5627-13	KM833097
Trombidiformes	Limnesiidae	*Limnesia sp0936BC*	GBA17793-14	KF000122
Trombidiformes	Limnesiidae	*Limnesia sp0936BC*	GBA17794-14	KF000160
Trombidiformes	Limnesiidae	*Limnesia sp0936BC*	GBA17795-14	KF000208
Trombidiformes	Limnesiidae	*Limnesia sp0936BC*	GBA17796-14	KF000209
Trombidiformes	Limnesiidae	*Limnesia sp0936BC*	GBA17797-14	KF000236

Trombidiformes	Limnesiidae	*Limnesia sp0936BC*	GBA17798-14	KF000237
Trombidiformes	Limnesiidae	*Limnesia sp0936BC*	GBA17799-14	KF000238
Trombidiformes	Limnesiidae	*Limnesia sp0936BC*	GBA17800-14	KF000239
Trombidiformes	Limnesiidae	*Limnesia sp0936BC*	GBA17801-14	KF000268
Trombidiformes	Limnesiidae	*Limnesia sp0936BC*	GBA17802-14	KF000269
Trombidiformes	Limnesiidae	*Limnesia sp0936BC*	GBA17803-14	KF000270
Trombidiformes	Limnesiidae	*Limnesia sp0936BC*	GBA17804-14	KF000271
Trombidiformes	Limnesiidae	*Limnesia sp0936BC*	GBA17805-14	KF000272
Trombidiformes	Limnesiidae	*Limnesia sp0936BC*	GBA17806-14	KF000320
Trombidiformes	Limnesiidae	*Limnesia sp0936BC*	GBA17807-14	KF000337
Trombidiformes	Limnesiidae	*Limnesia sp0936BC*	SAMIT327-10	HM382928
Trombidiformes	Limnesiidae		CNBPL275-13	KM829643
Trombidiformes	Limnesiidae		CNBPL290-13	KM830052
Trombidiformes	Limnesiidae		CNBPL292-13	KM825003
Trombidiformes	Limnesiidae		CNBPL295-13	KM832800
Trombidiformes	Limnesiidae		SSPAA2235-13	KM836690
Trombidiformes	Limnesiidae		SSPAA2236-13	KM824023
Trombidiformes	Limnesiidae		SSPAA2253-13	KM824242
Trombidiformes	Limnesiidae		SSPAA5638-13	KM829177
Trombidiformes	Limnesiidae		SSPAC13790-13	KM833430
Trombidiformes	Limnesiidae		SSPAC13792-13	KM830905
Trombidiformes	Limnesiidae		SSPAC7877-13	KM836129
Trombidiformes	Microdispidae		SSWEE109-13	KM832354
Trombidiformes	Microdispidae		SSWEE139-13	KM832381
Trombidiformes	Microdispidae		SSWEE162-13	KM832846
Trombidiformes	Microdispidae		SSWEE165-13	KM833100
Trombidiformes	Microtrombidiidae		CHACA048-08	JX836314
Trombidiformes	Microtrombidiidae		CNPAD841-13	KM830088
Trombidiformes	Microtrombidiidae		CNPPD2664-12	KJ090683
Trombidiformes	Microtrombidiidae		CNPPE2139-12	KJ165845
Trombidiformes	Microtrombidiidae		CNPPH1074-12	KJ637633
Trombidiformes	Microtrombidiidae		CNPPH1081-12	KJ637514
Trombidiformes	Microtrombidiidae		CNPPJ1862-12	KJ209219
Trombidiformes	Microtrombidiidae		MYMCA317-11	JX835192
Trombidiformes	Microtrombidiidae		MYMCB629-11	JX836793
Trombidiformes	Microtrombidiidae		MYMCB914-11	JX837943
Trombidiformes	Microtrombidiidae		MYMCE123-12	JX835070
Trombidiformes	Microtrombidiidae		MYMCE124-12	JX833738
Trombidiformes	Microtrombidiidae		MYMCE534-12	JX837087
Trombidiformes	Microtrombidiidae		MYMCG031-12	JX837924
Trombidiformes	Microtrombidiidae		PHAUG1674-11	KR069249
Trombidiformes	Microtrombidiidae		SSPAA2278-13	KM828290
Trombidiformes	Microtrombidiidae		SSPAA2283-13	KM836491
Trombidiformes	Microtrombidiidae		SSPAA2292-13	KM835177
Trombidiformes	Mideopsidae	*Mideopsis*	SSPAA2266-13	KM826475
Trombidiformes	Mideopsidae	*Mideopsis*	SSPAC13791-13	KM824355
Trombidiformes	Mideopsidae	*Mideopsis*	SSPAC13800-13	KM835045
Trombidiformes	Mideopsidae	*Mideopsis*	SSWLE3970-13	KM827481
Trombidiformes	Mideopsidae	*Mideopsis*	SSWLE3971-13	KM840547
Trombidiformes	Penthaleidae	*Penthaleus*	CHACA266-08	JX838308
Trombidiformes	Penthaleidae	*Penthaleus*	CHACA269-08	JX836182
Trombidiformes	Penthaleidae	*Penthaleus*	MYMCB890-11	JX834200
Trombidiformes	Penthaleidae	*Penthaleus*	MYMCB891-11	JX836957

Trombidiformes	Penthaleidae	*Penthaleus*	MYMCB921-11	JX836593
Trombidiformes	Penthaleidae	*Penthaleus*	MYMCC830-11	JX835942
Trombidiformes	Penthaleidae	*Penthaleus*	MYMCC831-11	JX835135
Trombidiformes	Penthaleidae	*Penthaleus cf. major MD-2010*	GBCH4418-10	GQ864389
Trombidiformes	Penthaleidae		CHACA1018-10	HM907078
Trombidiformes	Penthaleidae		CHACA1041-10	HM907094
Trombidiformes	Penthaleidae		CHACA1042-10	HM907095
Trombidiformes	Penthaleidae		CHACA1043-10	HM907096
Trombidiformes	Penthaleidae		CHACA1044-10	HM907097
Trombidiformes	Penthaleidae		CHACA1045-10	HM907098
Trombidiformes	Penthaleidae		CHACA1090-10	HM907130
Trombidiformes	Penthaleidae		CHACA1091-10	HM907131
Trombidiformes	Penthaleidae		CHACA1092-10	HM907132
Trombidiformes	Penthaleidae		CHACA376-08	JX834363
Trombidiformes	Penthaleidae		CHACB1000-10	HM907138
Trombidiformes	Penthaleidae		CHACB1001-10	HM907139
Trombidiformes	Penthaleidae		CHACB1002-10	HM907140
Trombidiformes	Penthaleidae		CHACB1003-10	HM907141
Trombidiformes	Penthaleidae		CHACB1004-10	HM907142
Trombidiformes	Penthaleidae		CHACB205-10	HQ558472
Trombidiformes	Penthaleidae		CHACB206-10	HQ558473
Trombidiformes	Penthaleidae		CHACB244-10	HQ558489
Trombidiformes	Penthaleidae		CHACB245-10	HQ558490
Trombidiformes	Penthaleidae		CHACB246-10	HQ558491
Trombidiformes	Penthaleidae		CHACB247-10	HQ558492
Trombidiformes	Penthaleidae		CHACB248-10	HQ558493
Trombidiformes	Penthaleidae		CHACB249-10	HQ558494
Trombidiformes	Penthaleidae		CHACB291-10	HM907284
Trombidiformes	Penthaleidae		CHACB292-10	HM907285
Trombidiformes	Penthaleidae		CHACB293-10	HM907286
Trombidiformes	Penthaleidae		CHACB294-10	JX834795
Trombidiformes	Penthaleidae		CHACB295-10	HM907287
Trombidiformes	Penthaleidae		CHACB381-10	HQ558554
Trombidiformes	Penthaleidae		CHACB382-10	HQ558555
Trombidiformes	Penthaleidae		CHACB972-10	HM907384
Trombidiformes	Penthaleidae		CHACB973-10	HM907385
Trombidiformes	Penthaleidae		CHACC176-10	JX835856
Trombidiformes	Penthaleidae		CHACC177-10	JX833830
Trombidiformes	Penthaleidae		CHACC178-10	JX836483
Trombidiformes	Penthaleidae		CHACC179-10	JX837887
Trombidiformes	Penthaleidae		MYMCA1258-11	JX837607
Trombidiformes	Penthaleidae		MYMCB468-11	JX834862
Trombidiformes	Penthaleidae		MYMCB469-11	JX835520
Trombidiformes	Penthaleidae		MYMCB889-11	JX837750
Trombidiformes	Penthaleidae		MYMCB925-11	JX835585
Trombidiformes	Penthaleidae		MYMCC105-11	JX834486
Trombidiformes	Penthaleidae		MYMCC376-11	JX834313
Trombidiformes	Penthaleidae		MYMCC535-11	JX838057
Trombidiformes	Penthaleidae		MYMCC536-11	JX838682
Trombidiformes	Penthaleidae		MYMCC727-11	JX837481
Trombidiformes	Penthaleidae		MYMCC778-11	JX836473
Trombidiformes	Penthaleidae		MYMCE754-12	JX838738
Trombidiformes	Penthalodidae		CHACA529-09	JX837415

Trombidiformes	Penthalodidae		CHACA609-09	JX834706
Trombidiformes	Penthalodidae		CHACB668-10	JX837420
Trombidiformes	Penthalodidae		MIONB255-10	KR069188
Trombidiformes	Penthalodidae		MIONB263-10	KR070722
Trombidiformes	Penthalodidae		MIONB264-10	KR070502
Trombidiformes	Penthalodidae		MIONB265-10	KR070046
Trombidiformes	Penthalodidae		MIONB266-10	KP979171
Trombidiformes	Penthalodidae		MIONB267-10	KR069791
Trombidiformes	Penthalodidae		MIONB268-10	KR070537
Trombidiformes	Penthalodidae		MYMCA335-11	JX837874
Trombidiformes	Penthalodidae		MYMCA336-11	JX834811
Trombidiformes	Penthalodidae		MYMCA338-11	JX836189
Trombidiformes	Penthalodidae		MYMCB001-11	JX838764
Trombidiformes	Penthalodidae		MYMCB003-11	JX834195
Trombidiformes	Penthalodidae		MYMCB004-11	JX836981
Trombidiformes	Penthalodidae		MYMCB007-11	JX835833
Trombidiformes	Penthalodidae		MYMCB462-11	JX834380
Trombidiformes	Penthalodidae		MYMCB690-11	JX835654
Trombidiformes	Penthalodidae		MYMCC113-11	JX834818
Trombidiformes	Penthalodidae		MYMCC129-11	JX838597
Trombidiformes	Penthalodidae		MYMCC130-11	JX837069
Trombidiformes	Penthalodidae		MYMCC131-11	JX834347
Trombidiformes	Penthalodidae		MYMCC135-11	JX834789
Trombidiformes	Penthalodidae		MYMCC399-11	JX836910
Trombidiformes	Penthalodidae		MYMCC400-11	JX835984
Trombidiformes	Penthalodidae		MYMCC401-11	JX837025
Trombidiformes	Penthalodidae		MYMCC837-11	JX835203
Trombidiformes	Penthalodidae		MYMCD027-11	JX834387
Trombidiformes	Penthalodidae		MYMCD030-11	JX838460
Trombidiformes	Penthalodidae		MYMCD043-11	JX836002
Trombidiformes	Penthalodidae		MYMCD083-11	JX838038
Trombidiformes	Penthalodidae		MYMCD084-11	JX835888
Trombidiformes	Penthalodidae		MYMCD088-11	JX834374
Trombidiformes	Penthalodidae		MYMCD125-11	JX838399
Trombidiformes	Penthalodidae		MYMCE489-12	JX836537
Trombidiformes	Penthalodidae		MYMCE565-12	JX837522
Trombidiformes	Penthalodidae		MYMCF216-12	JX836642
Trombidiformes	Penthalodidae		MYMCF552-12	JX834318
Trombidiformes	Penthalodidae		MYMCF553-12	JX838414
Trombidiformes	Penthalodidae		MYMCF554-12	JX833781
Trombidiformes	Penthalodidae		MYMCF687-12	JX838246
Trombidiformes	Penthalodidae		MYMCF688-12	JX836811
Trombidiformes	Penthalodidae		MYMCF689-12	JX837150
Trombidiformes	Penthalodidae		MYMCF900-12	JX837656
Trombidiformes	Penthalodidae		MYMCF901-12	JX838295
Trombidiformes	Penthalodidae		MYMCG318-12	JX835481
Trombidiformes	Penthalodidae		MYMCG319-12	JX834504
Trombidiformes	Penthalodidae		MYMCG320-12	JX834428
Trombidiformes	Penthalodidae		SSPAA7790-13	KM826101
Trombidiformes	Pionidae	*Forelia sp0961A*	CNPAC309-13	KM831116
Trombidiformes	Pionidae	*Forelia sp0961A*	CNPAC310-13	KM835204
Trombidiformes	Pionidae	*Forelia sp0961A*	CNPAC311-13	KM826800
Trombidiformes	Pionidae	*Piona*	ARCN017-10	HQ924261

Trombidiformes	Pionidae	*Piona*	ARCN018-10	HQ924262
Trombidiformes	Pionidae	*Piona*	ARCN019-10	HQ924263
Trombidiformes	Pionidae	*Piona*	ARCN020-10	HQ924264
Trombidiformes	Pionidae	*Piona*	ARCN021-10	HQ924265
Trombidiformes	Pionidae	*Piona*	ARCN065-10	HQ924288
Trombidiformes	Pionidae	*Piona*	ARCN067-10	HQ924290
Trombidiformes	Pionidae	*Piona*	ARCN069-10	HQ924292
Trombidiformes	Pionidae	*Piona*	CNGIB598-12	KM825988
Trombidiformes	Pionidae	*Piona*	CNPAB275-13	KM836392
Trombidiformes	Pionidae	*Piona*	CNPAC559-13	KM827696
Trombidiformes	Pionidae	*Piona*	CNPAF868-13	KM839625
Trombidiformes	Pionidae	*Piona*	MYMCA110-11	JX835088
Trombidiformes	Pionidae	*Piona*	MYMCE363-12	JX836721
Trombidiformes	Pionidae	*Piona*	SSBAF2268-13	KM834304
Trombidiformes	Pionidae	*Piona*	SSJAE3131-13	KM824678
Trombidiformes	Pionidae		ARCN068-10	HQ924291
Trombidiformes	Pionidae		CNJAF1921-12	KM826914
Trombidiformes	Pionidae		CNJAH761-12	KM829893
Trombidiformes	Pionidae		CNJAH777-12	KM826568
Trombidiformes	Pionidae		CNPPC1929-12	KJ090583
Trombidiformes	Pionidae		CNPPC1931-12	KJ083325
Trombidiformes	Pionidae		CNPPC1934-12	KJ084272
Trombidiformes	Pionidae		CNPPC1936-12	KJ089959
Trombidiformes	Pionidae		SSJAE3140-13	KM824353
Trombidiformes	Pionidae		SSJAE3146-13	KM836300
Trombidiformes	Pionidae		SSPAA2307-13	KM838383
Trombidiformes	Pionidae		SSPAB4021-13	KM835270
Trombidiformes	Pionidae		SSPAB957-13	KM830961
Trombidiformes	Protziidae	*Protzia*	CFWIA644-10	HQ938823
Trombidiformes	Protziidae	*Protzia*	CFWIB546-10	HQ939579
Trombidiformes	Protziidae	*Protzia*	CFWIB547-10	HQ939580
Trombidiformes	Protziidae	*Protzia*	CFWIB548-10	HQ939581
Trombidiformes	Pyemotidae	*Pyemotes*	MYMCA1211-11	JX837857
Trombidiformes	Pyemotidae	*Pyemotes*	MYMCE607-12	JX835637
Trombidiformes	Pyemotidae	*Pyemotes*	MYMCG578-12	JX833796
Trombidiformes	Pyemotidae	*Pyemotes*	MYMCG579-12	JX835094
Trombidiformes	Pyemotidae	*Pyemotes*	MYMCG580-12	JX835291
Trombidiformes	Pyemotidae	*Pyemotes*	MYMCG581-12	JX833686
Trombidiformes	Pyemotidae	*Pyemotes*	MYMCG582-12	JX835095
Trombidiformes	Rhagidiidae		SSKJB4557-14	KR069187
Trombidiformes	Rhagidiidae		CHACA1160-10	JX836696
Trombidiformes	Rhagidiidae		CHACA1209-10	JX835242
Trombidiformes	Rhagidiidae		CHACA1211-10	JX838080
Trombidiformes	Rhagidiidae		CHACA1213-10	JX837880
Trombidiformes	Rhagidiidae		CHACA1216-10	JX838393
Trombidiformes	Rhagidiidae		CHACA1217-10	JX836080
Trombidiformes	Rhagidiidae		CHACA182-08	JX835546
Trombidiformes	Rhagidiidae		CHACA183-08	JX834955
Trombidiformes	Rhagidiidae		CHACA326-08	JX834156
Trombidiformes	Rhagidiidae		CHACA327-08	JX837726
Trombidiformes	Rhagidiidae		CHACA328-08	JX834619
Trombidiformes	Rhagidiidae		CHACA329-08	JX837473
Trombidiformes	Rhagidiidae		CHACA330-08	JX833994

Trombidiformes	Rhagidiidae	CHACA373-08	JX837468
Trombidiformes	Rhagidiidae	CHACA454-09	JX836087
Trombidiformes	Rhagidiidae	CHACA969-10	HQ941470
Trombidiformes	Rhagidiidae	CHACA983-10	HM405847
Trombidiformes	Rhagidiidae	CHACA985-10	HM405848
Trombidiformes	Rhagidiidae	CHACB021-10	HQ558341
Trombidiformes	Rhagidiidae	CHACB022-10	HQ558342
Trombidiformes	Rhagidiidae	CHACB056-10	HQ558365
Trombidiformes	Rhagidiidae	CHACB057-10	HQ558366
Trombidiformes	Rhagidiidae	CHACB078-10	HQ558385
Trombidiformes	Rhagidiidae	CHACB1054-10	HM907179
Trombidiformes	Rhagidiidae	CHACB1055-10	HM907180
Trombidiformes	Rhagidiidae	CHACB1070-10	HM907187
Trombidiformes	Rhagidiidae	CHACB169-10	HQ558450
Trombidiformes	Rhagidiidae	CHACB456-10	JX834737
Trombidiformes	Rhagidiidae	CHACB464-10	JX835231
Trombidiformes	Rhagidiidae	CHACB601-10	HQ558696
Trombidiformes	Rhagidiidae	CHACB602-10	HQ558697
Trombidiformes	Rhagidiidae	CHACB632-10	HQ558716
Trombidiformes	Rhagidiidae	CHACB691-10	HQ558751
Trombidiformes	Rhagidiidae	CHACB962-10	HM907382
Trombidiformes	Rhagidiidae	CHACB994-10	HM907399
Trombidiformes	Rhagidiidae	CHACC144-10	JX834140
Trombidiformes	Rhagidiidae	CHACC146-10	JX836980
Trombidiformes	Rhagidiidae	CHACC218-10	JX836761
Trombidiformes	Rhagidiidae	CHACC219-10	KR069869
Trombidiformes	Rhagidiidae	CNJAF1922-12	KM831617
Trombidiformes	Rhagidiidae	MIONB154-10	KP979176
Trombidiformes	Rhagidiidae	MIONB155-10	KR069765
Trombidiformes	Rhagidiidae	MIONB167-10	KR069660
Trombidiformes	Rhagidiidae	MIONB168-10	KP979160
Trombidiformes	Rhagidiidae	MIONB169-10	KR070169
Trombidiformes	Rhagidiidae	MIONB170-10	KR070232
Trombidiformes	Rhagidiidae	MIONB171-10	KP979306
Trombidiformes	Rhagidiidae	MIONB190-10	KP979263
Trombidiformes	Rhagidiidae	MIONB227-10	KP979116
Trombidiformes	Rhagidiidae	MIONB228-10	KP979320
Trombidiformes	Rhagidiidae	MIONB257-10	KP979208
Trombidiformes	Rhagidiidae	MIONB258-10	KR070696
Trombidiformes	Rhagidiidae	MIONB259-10	KP979309
Trombidiformes	Rhagidiidae	MYMCA1006-11	JX834495
Trombidiformes	Rhagidiidae	MYMCA1166-11	JX835513
Trombidiformes	Rhagidiidae	MYMCA1201-11	JX835639
Trombidiformes	Rhagidiidae	MYMCA1244-11	JX835792
Trombidiformes	Rhagidiidae	MYMCA219-11	JX836320
Trombidiformes	Rhagidiidae	MYMCA220-11	JX833850
Trombidiformes	Rhagidiidae	MYMCA221-11	JX833907
Trombidiformes	Rhagidiidae	MYMCA385-11	JX837293
Trombidiformes	Rhagidiidae	MYMCA727-11	JX833784
Trombidiformes	Rhagidiidae	MYMCA839-11	JX835788
Trombidiformes	Rhagidiidae	MYMCA840-11	JX837867
Trombidiformes	Rhagidiidae	MYMCA841-11	JX834277
Trombidiformes	Rhagidiidae	MYMCA875-11	JX836988

Trombidiformes	Rhagidiidae	MYMCA876-11	JX838425
Trombidiformes	Rhagidiidae	MYMCA889-11	JX835611
Trombidiformes	Rhagidiidae	MYMCA984-11	JX838267
Trombidiformes	Rhagidiidae	MYMCB131-11	JX837680
Trombidiformes	Rhagidiidae	MYMCB178-11	JX837342
Trombidiformes	Rhagidiidae	MYMCB194-11	JX834881
Trombidiformes	Rhagidiidae	MYMCB195-11	JX833754
Trombidiformes	Rhagidiidae	MYMCB210-11	JX837875
Trombidiformes	Rhagidiidae	MYMCB211-11	JX833926
Trombidiformes	Rhagidiidae	MYMCB212-11	JX834197
Trombidiformes	Rhagidiidae	MYMCB217-11	JX838249
Trombidiformes	Rhagidiidae	MYMCB218-11	JX838714
Trombidiformes	Rhagidiidae	MYMCB434-11	JX838439
Trombidiformes	Rhagidiidae	MYMCB456-11	JX834564
Trombidiformes	Rhagidiidae	MYMCB460-11	JX838122
Trombidiformes	Rhagidiidae	MYMCB463-11	JX838427
Trombidiformes	Rhagidiidae	MYMCB470-11	JX834990
Trombidiformes	Rhagidiidae	MYMCB471-11	JX837691
Trombidiformes	Rhagidiidae	MYMCB475-11	JX837057
Trombidiformes	Rhagidiidae	MYMCB489-11	JX838014
Trombidiformes	Rhagidiidae	MYMCB508-11	JX835422
Trombidiformes	Rhagidiidae	MYMCB555-11	JX834775
Trombidiformes	Rhagidiidae	MYMCB567-11	JX837976
Trombidiformes	Rhagidiidae	MYMCB637-11	JX838659
Trombidiformes	Rhagidiidae	MYMCB647-11	JX836203
Trombidiformes	Rhagidiidae	MYMCB648-11	JX834817
Trombidiformes	Rhagidiidae	MYMCB652-11	JX836832
Trombidiformes	Rhagidiidae	MYMCB678-11	JX837850
Trombidiformes	Rhagidiidae	MYMCB679-11	JX834452
Trombidiformes	Rhagidiidae	MYMCB682-11	JX835566
Trombidiformes	Rhagidiidae	MYMCB684-11	JX837034
Trombidiformes	Rhagidiidae	MYMCB685-11	JX834029
Trombidiformes	Rhagidiidae	MYMCB753-11	JX837616
Trombidiformes	Rhagidiidae	MYMCB790-11	JX833657
Trombidiformes	Rhagidiidae	MYMCB840-11	JX837301
Trombidiformes	Rhagidiidae	MYMCB841-11	JX837198
Trombidiformes	Rhagidiidae	MYMCB924-11	JX837003
Trombidiformes	Rhagidiidae	MYMCB926-11	JX835854
Trombidiformes	Rhagidiidae	MYMCC033-11	JX836207
Trombidiformes	Rhagidiidae	MYMCC107-11	JX837965
Trombidiformes	Rhagidiidae	MYMCC112-11	JX834056
Trombidiformes	Rhagidiidae	MYMCC153-11	JX837754
Trombidiformes	Rhagidiidae	MYMCC154-11	JX834584
Trombidiformes	Rhagidiidae	MYMCC155-11	JX836572
Trombidiformes	Rhagidiidae	MYMCC241-11	JX837596
Trombidiformes	Rhagidiidae	MYMCC284-11	JX836469
Trombidiformes	Rhagidiidae	MYMCC326-11	JX838626
Trombidiformes	Rhagidiidae	MYMCC346-11	JX838225
Trombidiformes	Rhagidiidae	MYMCC353-11	JX835148
Trombidiformes	Rhagidiidae	MYMCC380-11	JX836266
Trombidiformes	Rhagidiidae	MYMCC383-11	JX835089
Trombidiformes	Rhagidiidae	MYMCC384-11	JX835631
Trombidiformes	Rhagidiidae	MYMCC405-11	JX837627

Trombidiformes	Rhagidiidae	MYMCC418-11	JX838671
Trombidiformes	Rhagidiidae	MYMCC419-11	JX838313
Trombidiformes	Rhagidiidae	MYMCC420-11	JX836163
Trombidiformes	Rhagidiidae	MYMCC499-11	JX834624
Trombidiformes	Rhagidiidae	MYMCC531-11	JX836035
Trombidiformes	Rhagidiidae	MYMCC545-11	JX838502
Trombidiformes	Rhagidiidae	MYMCC555-11	JX836529
Trombidiformes	Rhagidiidae	MYMCC556-11	JX838106
Trombidiformes	Rhagidiidae	MYMCC557-11	JX833703
Trombidiformes	Rhagidiidae	MYMCC586-11	JX835549
Trombidiformes	Rhagidiidae	MYMCC697-11	JX836173
Trombidiformes	Rhagidiidae	MYMCC699-11	JX836339
Trombidiformes	Rhagidiidae	MYMCC750-11	JX836417
Trombidiformes	Rhagidiidae	MYMCC752-11	JX835506
Trombidiformes	Rhagidiidae	MYMCC779-11	JX836465
Trombidiformes	Rhagidiidae	MYMCC780-11	JX833785
Trombidiformes	Rhagidiidae	MYMCD014-11	JX835308
Trombidiformes	Rhagidiidae	MYMCD024-11	JX834395
Trombidiformes	Rhagidiidae	MYMCD025-11	JX834411
Trombidiformes	Rhagidiidae	MYMCD026-11	JX836973
Trombidiformes	Rhagidiidae	MYMCD031-11	JX836583
Trombidiformes	Rhagidiidae	MYMCD037-11	JX838292
Trombidiformes	Rhagidiidae	MYMCD054-11	JX838551
Trombidiformes	Rhagidiidae	MYMCD089-11	JX836697
Trombidiformes	Rhagidiidae	MYMCD165-11	JX837974
Trombidiformes	Rhagidiidae	MYMCD171-11	JX835651
Trombidiformes	Rhagidiidae	MYMCE130-12	JX834863
Trombidiformes	Rhagidiidae	MYMCE131-12	JX838121
Trombidiformes	Rhagidiidae	MYMCE132-12	JX834773
Trombidiformes	Rhagidiidae	MYMCE136-12	JX834129
Trombidiformes	Rhagidiidae	MYMCE186-12	JX834022
Trombidiformes	Rhagidiidae	MYMCE287-12	JX834644
Trombidiformes	Rhagidiidae	MYMCE416-12	JX838752
Trombidiformes	Rhagidiidae	MYMCE418-12	JX836031
Trombidiformes	Rhagidiidae	MYMCE434-12	JX833813
Trombidiformes	Rhagidiidae	MYMCE435-12	JX834107
Trombidiformes	Rhagidiidae	MYMCE436-12	JX838708
Trombidiformes	Rhagidiidae	MYMCE437-12	JX834153
Trombidiformes	Rhagidiidae	MYMCE535-12	JX836908
Trombidiformes	Rhagidiidae	MYMCE537-12	JX833905
Trombidiformes	Rhagidiidae	MYMCE539-12	JX837886
Trombidiformes	Rhagidiidae	MYMCE541-12	JX838314
Trombidiformes	Rhagidiidae	MYMCE564-12	JX838150
Trombidiformes	Rhagidiidae	MYMCE582-12	JX837854
Trombidiformes	Rhagidiidae	MYMCE583-12	JX836190
Trombidiformes	Rhagidiidae	MYMCE634-12	JX834724
Trombidiformes	Rhagidiidae	MYMCE850-12	JX834905
Trombidiformes	Rhagidiidae	MYMCF008-12	JX835920
Trombidiformes	Rhagidiidae	MYMCF221-12	JX837544
Trombidiformes	Rhagidiidae	MYMCF222-12	JX837101
Trombidiformes	Rhagidiidae	MYMCF223-12	JX835350
Trombidiformes	Rhagidiidae	MYMCF233-12	JX835971
Trombidiformes	Rhagidiidae	MYMCF473-12	JX835616

Trombidiformes	Rhagidiidae	MYMCF474-12	JX838282
Trombidiformes	Rhagidiidae	MYMCF480-12	JX836623
Trombidiformes	Rhagidiidae	MYMCF498-12	JX833868
Trombidiformes	Rhagidiidae	MYMCF618-12	JX834522
Trombidiformes	Rhagidiidae	MYMCF700-12	JX837329
Trombidiformes	Rhagidiidae	MYMCF795-12	JX837698
Trombidiformes	Rhagidiidae	MYMCF796-12	JX837864
Trombidiformes	Rhagidiidae	MYMCF798-12	JX837751
Trombidiformes	Rhagidiidae	MYMCF799-12	JX835449
Trombidiformes	Rhagidiidae	MYMCF851-12	JX836685
Trombidiformes	Rhagidiidae	MYMCF908-12	JX834108
Trombidiformes	Rhagidiidae	MYMCF948-12	JX835831
Trombidiformes	Rhagidiidae	MYMCG027-12	JX838383
Trombidiformes	Rhagidiidae	MYMCG028-12	JX835353
Trombidiformes	Rhagidiidae	MYMCG088-12	JX836774
Trombidiformes	Rhagidiidae	MYMCG089-12	JX838451
Trombidiformes	Rhagidiidae	MYMCG090-12	JX838528
Trombidiformes	Rhagidiidae	MYMCG106-12	JX838688
Trombidiformes	Rhagidiidae	MYMCG123-12	JX836682
Trombidiformes	Rhagidiidae	MYMCG124-12	JX836291
Trombidiformes	Rhagidiidae	MYMCG125-12	JX838201
Trombidiformes	Rhagidiidae	MYMCG150-12	JX834959
Trombidiformes	Rhagidiidae	MYMCG213-12	JX837202
Trombidiformes	Rhagidiidae	MYMCG214-12	JX833659
Trombidiformes	Rhagidiidae	MYMCG471-12	JX838296
Trombidiformes	Rhagidiidae	MYMCG562-12	JX837009
Trombidiformes	Rhagidiidae	MYMCG614-12	JX835551
Trombidiformes	Rhagidiidae	MYMCG629-12	JX838697
Trombidiformes	Rhagidiidae	MYMCG630-12	JX834976
Trombidiformes	Rhagidiidae	MYMCG634-12	JX836780
Trombidiformes	Rhagidiidae	MYTMC064-09	HQ966231
Trombidiformes	Rhagidiidae	RBINA3916-13	KP979161
Trombidiformes	Rhagidiidae	SSBAB1145-12	KM825749
Trombidiformes	Rhagidiidae	SSBAB1164-12	KM824893
Trombidiformes	Rhagidiidae	SSBAB1166-12	KM827064
Trombidiformes	Rhagidiidae	SSBAB1179-12	KM831434
Trombidiformes	Rhagidiidae	SSBAB1180-12	KM831300
Trombidiformes	Rhagidiidae	SSBAB1182-12	KM830150
Trombidiformes	Rhagidiidae	SSBAB1194-12	KM831606
Trombidiformes	Rhagidiidae	SSBAB1204-12	KM832348
Trombidiformes	Rhagidiidae	SSBAB1205-12	KM827452
Trombidiformes	Rhagidiidae	SSBAB1207-12	KM832130
Trombidiformes	Rhagidiidae	SSBAB1215-12	KM824861
Trombidiformes	Rhagidiidae	SSBAB1216-12	KM834373
Trombidiformes	Rhagidiidae	SSBAB1218-12	KM831984
Trombidiformes	Rhagidiidae	SSBAB1219-12	KM829623
Trombidiformes	Rhagidiidae	SSBAB1220-12	KM838680
Trombidiformes	Rhagidiidae	SSBAB1222-12	KM831069
Trombidiformes	Rhagidiidae	SSBAB1223-12	KM829815
Trombidiformes	Rhagidiidae	SSBAB1872-12	KM837346
Trombidiformes	Rhagidiidae	SSBAB1929-12	KM833288
Trombidiformes	Rhagidiidae	SSBAB2072-12	KM827418
Trombidiformes	Rhagidiidae	SSBAB2084-12	KM833326

Trombidiformes	Rhagidiidae	SSBAB2094-12	KM833593
Trombidiformes	Rhagidiidae	SSBAC3328-12	KM838746
Trombidiformes	Rhagidiidae	SSBAD3095-12	KM827190
Trombidiformes	Rhagidiidae	SSBAD4739-13	KM834358
Trombidiformes	Rhagidiidae	SSBAD4745-13	KM826498
Trombidiformes	Rhagidiidae	SSBAD4746-13	KM824073
Trombidiformes	Rhagidiidae	SSBAD4757-13	KM830046
Trombidiformes	Rhagidiidae	SSBAE3624-13	KM828735
Trombidiformes	Rhagidiidae	SSBAE3760-13	KM824791
Trombidiformes	Rhagidiidae	SSBAE3762-13	KM840564
Trombidiformes	Rhagidiidae	SSBAE3763-13	KM835362
Trombidiformes	Rhagidiidae	SSBAE3765-13	KM835548
Trombidiformes	Rhagidiidae	SSBAE3767-13	KM826761
Trombidiformes	Rhagidiidae	SSBAE3769-13	KM829803
Trombidiformes	Rhagidiidae	SSBAE3771-13	KM827441
Trombidiformes	Rhagidiidae	SSBAE3773-13	KM839463
Trombidiformes	Rhagidiidae	SSBAE3774-13	KM824273
Trombidiformes	Rhagidiidae	SSBAE3776-13	KM837196
Trombidiformes	Rhagidiidae	SSBAE3777-13	KM834490
Trombidiformes	Rhagidiidae	SSBAE3778-13	KM839975
Trombidiformes	Rhagidiidae	SSBAE3780-13	KM823984
Trombidiformes	Rhagidiidae	SSBAE3783-13	KM833132
Trombidiformes	Rhagidiidae	SSBAE3787-13	KM826213
Trombidiformes	Rhagidiidae	SSBAE3807-13	KM835823
Trombidiformes	Rhagidiidae	SSBAE3812-13	KM831264
Trombidiformes	Rhagidiidae	SSEIA3014-13	KM824510
Trombidiformes	Rhagidiidae	SSEIA3016-13	KM834606
Trombidiformes	Rhagidiidae	SSEIA3025-13	KM831499
Trombidiformes	Rhagidiidae	SSEIA3026-13	KM832382
Trombidiformes	Rhagidiidae	SSEIA3033-13	KM830610
Trombidiformes	Rhagidiidae	SSEIA3038-13	KM829136
Trombidiformes	Rhagidiidae	SSEIA3042-13	KM827836
Trombidiformes	Rhagidiidae	SSEIA3045-13	KM825890
Trombidiformes	Rhagidiidae	SSEIA3053-13	KM824038
Trombidiformes	Rhagidiidae	SSEIB7629-13	KM825311
Trombidiformes	Rhagidiidae	SSEIB7633-13	KM833792
Trombidiformes	Rhagidiidae	SSEIB7635-13	KM827416
Trombidiformes	Rhagidiidae	SSEIB7637-13	KM835306
Trombidiformes	Rhagidiidae	SSEIB7638-13	KM833476
Trombidiformes	Rhagidiidae	SSEIB7642-13	KM827666
Trombidiformes	Rhagidiidae	SSEIB7643-13	KM837802
Trombidiformes	Rhagidiidae	SSEIB7676-13	KM837275
Trombidiformes	Rhagidiidae	SSJAA1989-13	KM836163
Trombidiformes	Rhagidiidae	SSJAB1850-13	KM839779
Trombidiformes	Rhagidiidae	SSJAB1883-13	KM829244
Trombidiformes	Rhagidiidae	SSJAB2135-13	KM829939
Trombidiformes	Rhagidiidae	SSJAB2137-13	KM830583
Trombidiformes	Rhagidiidae	SSJAC1538-13	KM826953
Trombidiformes	Rhagidiidae	SSJAC1543-13	KM837509
Trombidiformes	Rhagidiidae	SSJAC1550-13	KM831482
Trombidiformes	Rhagidiidae	SSJAC1563-13	KM825846
Trombidiformes	Rhagidiidae	SSJAC1571-13	KM838046
Trombidiformes	Rhagidiidae	SSJAC1578-13	KM830686

Trombidiformes	Rhagidiidae	SSJAC1589-13	KM825379
Trombidiformes	Rhagidiidae	SSJAC972-13	KM825523
Trombidiformes	Rhagidiidae	SSJAC975-13	KM838000
Trombidiformes	Rhagidiidae	SSJAC989-13	KM827628
Trombidiformes	Rhagidiidae	SSJAF7398-13	KM833841
Trombidiformes	Rhagidiidae	SSJAF7424-13	KM831906
Trombidiformes	Rhagidiidae	SSJAF8408-13	KM837384
Trombidiformes	Rhagidiidae	SSJAF8642-13	KM827411
Trombidiformes	Rhagidiidae	SSJAF8647-13	KM836884
Trombidiformes	Rhagidiidae	SSPAA7816-13	KM827285
Trombidiformes	Rhagidiidae	SSPAC10912-13	KM824805
Trombidiformes	Rhagidiidae	SSWLA1883-13	KM840159
Trombidiformes	Rhagidiidae	SSWLA5302-13	KM834033
Trombidiformes	Rhagidiidae	SSWLA5308-13	KM833553
Trombidiformes	Rhagidiidae	SSWLA5336-13	KM826486
Trombidiformes	Rhagidiidae	SSWLC4128-13	KM825792
Trombidiformes	Rhagidiidae	SSWLC4129-13	KM826887
Trombidiformes	Rhagidiidae	SSWLC4133-13	KM836212
Trombidiformes	Rhagidiidae	SSWLC4142-13	KM837118
Trombidiformes	Rhagidiidae	SSWLC4148-13	KM834700
Trombidiformes	Rhagidiidae	SSWLC4150-13	KM824690
Trombidiformes	Rhagidiidae	SSWLC4152-13	KM831858
Trombidiformes	Rhagidiidae	SSWLC4153-13	KM825949
Trombidiformes	Rhagidiidae	SSWLC4160-13	KM837652
Trombidiformes	Rhagidiidae	SSWLC4161-13	KR070419
Trombidiformes	Rhagidiidae	SSWLD2920-13	KM826074
Trombidiformes	Rhagidiidae	SSWLD3510-13	KP979220
Trombidiformes	Scutacaridae	CHACB080-10	HQ558386
Trombidiformes	Scutacaridae	CHACB081-10	HQ558387
Trombidiformes	Scutacaridae	CHACB083-10	HQ558388
Trombidiformes	Scutacaridae	CHACB1118-10	HM907212
Trombidiformes	Scutacaridae	CHACB370-10	HQ558548
Trombidiformes	Scutacaridae	CHACB774-10	HQ941479
Trombidiformes	Scutacaridae	CNJAC1524-12	KM834045
Trombidiformes	Scutacaridae	CNJAC1606-12	KM836566
Trombidiformes	Scutacaridae	CNJAD2278-12	KM831506
Trombidiformes	Scutacaridae	CNJAE1131-12	KM834357
Trombidiformes	Scutacaridae	CNJAE1144-12	KM832973
Trombidiformes	Scutacaridae	CNJAE1149-12	KM827367
Trombidiformes	Scutacaridae	CNJAF1912-12	KM836201
Trombidiformes	Scutacaridae	CNJAF1914-12	KM831284
Trombidiformes	Scutacaridae	CNJAF1917-12	KM832754
Trombidiformes	Scutacaridae	CNJAF1931-12	KM826454
Trombidiformes	Scutacaridae	CNJAF1932-12	KM840750
Trombidiformes	Scutacaridae	CNJAF1940-12	KM834426
Trombidiformes	Scutacaridae	CNJAF1945-12	KM840686
Trombidiformes	Scutacaridae	CNJAF1959-12	KM825432
Trombidiformes	Scutacaridae	CNJAF1963-12	KM829612
Trombidiformes	Scutacaridae	CNJAF1967-12	KM837100
Trombidiformes	Scutacaridae	CNJAF1973-12	KM832984
Trombidiformes	Scutacaridae	CNJAF1977-12	KM829830
Trombidiformes	Scutacaridae	CNJAF1982-12	KM831330
Trombidiformes	Scutacaridae	CNJAF1984-12	KM839675

Trombidiformes	Scutacaridae		CNJAF1997-12	KM839516
Trombidiformes	Scutacaridae		CNJAF2003-12	KM840659
Trombidiformes	Scutacaridae		CNJAF2004-12	KM839920
Trombidiformes	Scutacaridae		CNJAF2009-12	KM840399
Trombidiformes	Scutacaridae		CNJAF2011-12	KM836057
Trombidiformes	Scutacaridae		CNJAF2023-12	KM835602
Trombidiformes	Scutacaridae		CNJAG1803-12	KM826487
Trombidiformes	Scutacaridae		CNJAG1816-12	KM832960
Trombidiformes	Scutacaridae		CNJAG1827-12	KM837040
Trombidiformes	Scutacaridae		CNJAH773-12	KM836874
Trombidiformes	Scutacaridae		CNJAH774-12	KM827811
Trombidiformes	Scutacaridae		CNJAI835-12	KM840438
Trombidiformes	Scutacaridae		CNJAI838-12	KM837552
Trombidiformes	Scutacaridae		CNJAI841-12	KM827673
Trombidiformes	Scutacaridae		CNJAI843-12	KM840634
Trombidiformes	Scutacaridae		CNJAI845-12	KM831432
Trombidiformes	Scutacaridae		CNJAI846-12	KM834876
Trombidiformes	Scutacaridae		CNJAI848-12	KM825672
Trombidiformes	Scutacaridae		CNJAI851-12	KM830966
Trombidiformes	Scutacaridae		CNJAI852-12	KM840797
Trombidiformes	Scutacaridae		CNJAI857-12	KM825773
Trombidiformes	Scutacaridae		CNJAI859-12	KM834571
Trombidiformes	Scutacaridae		CNJAI860-12	KM827444
Trombidiformes	Scutacaridae		CNJAJ695-12	KM825612
Trombidiformes	Scutacaridae		CNJAJ698-12	KM830486
Trombidiformes	Scutacaridae		CNJAJ699-12	KM840062
Trombidiformes	Scutacaridae		CNJAJ702-12	KM830523
Trombidiformes	Scutacaridae		CNJAJ708-12	KM828289
Trombidiformes	Scutacaridae		CNJAJ710-12	KM828284
Trombidiformes	Scutacaridae		CNPAE418-13	KM824269
Trombidiformes	Scutacaridae		CNPAE419-13	KM824149
Trombidiformes	Scutacaridae		CNPAE420-13	KM827518
Trombidiformes	Scutacaridae		CNPAE422-13	KM824425
Trombidiformes	Scutacaridae		CNPAE423-13	KM828726
Trombidiformes	Scutacaridae		CNPAE424-13	KM838045
Trombidiformes	Scutacaridae		CNPAF587-13	KM831922
Trombidiformes	Scutacaridae		CNPAF609-13	KM825655
Trombidiformes	Scutacaridae		CNPAF610-13	KM836180
Trombidiformes	Scutacaridae		CNPAF626-13	KM837195
Trombidiformes	Scutacaridae		CNPAF830-13	KM824166
Trombidiformes	Scutacaridae		CNPAF835-13	KM837783
Trombidiformes	Scutacaridae		CNPAG368-13	KM836465
Trombidiformes	Scutacaridae		CNPAG369-13	KM831855
Trombidiformes	Scutacaridae		CNPAG370-13	KM834275
Trombidiformes	Scutacaridae		CNPAG371-13	KM830079
Trombidiformes	Scutacaridae		CNPAH396-13	KM828816
Trombidiformes	Scutacaridae		CNPAH399-13	KM827851
Trombidiformes	Scutacaridae		CNPAH401-13	KM827648
Trombidiformes	Scutacaridae		CNPAH402-13	KM836862
Trombidiformes	Scutacaridae		CNPAH403-13	KM839423
Trombidiformes	Scutacaridae		CNPAH405-13	KM831336
Trombidiformes	Scutacaridae		CNPAH406-13	KM834838
Trombidiformes	Scutacaridae		CNPAH407-13	KM833615

Trombidiformes	Scutacaridae	CNPAH408-13	KM838106
Trombidiformes	Scutacaridae	CNPAH410-13	KM832770
Trombidiformes	Scutacaridae	CNPAH412-13	KM839007
Trombidiformes	Scutacaridae	CNPAH414-13	KM831730
Trombidiformes	Scutacaridae	CNPAI060-13	KM839475
Trombidiformes	Scutacaridae	CNPAI061-13	KM836775
Trombidiformes	Scutacaridae	CNPAI062-13	KM839517
Trombidiformes	Scutacaridae	CNPAI063-13	KM832696
Trombidiformes	Scutacaridae	CNPAI065-13	KM837208
Trombidiformes	Scutacaridae	CNPAI067-13	KM829979
Trombidiformes	Scutacaridae	CNPAI069-13	KM831880
Trombidiformes	Scutacaridae	CNPAI462-13	KM828623
Trombidiformes	Scutacaridae	CNPAI463-13	KM839768
Trombidiformes	Scutacaridae	CNPPF1050-12	KJ165455
Trombidiformes	Scutacaridae	CNPPI1277-12	KJ208582
Trombidiformes	Scutacaridae	MYMCA1380-11	JX834731
Trombidiformes	Scutacaridae	MYMCA1381-11	JX837545
Trombidiformes	Scutacaridae	MYMCA1382-11	JX836959
Trombidiformes	Scutacaridae	MYMCE771-12	JX835100
Trombidiformes	Scutacaridae	MYMCE795-12	JX836677
Trombidiformes	Scutacaridae	MYMCE796-12	JX835372
Trombidiformes	Scutacaridae	MYMCE930-12	JX836455
Trombidiformes	Scutacaridae	MYMCE931-12	JX833924
Trombidiformes	Scutacaridae	MYMCE932-12	JX836952
Trombidiformes	Scutacaridae	MYMCF071-12	JX835154
Trombidiformes	Scutacaridae	MYMCF072-12	JX838788
Trombidiformes	Scutacaridae	MYMCF073-12	JX838640
Trombidiformes	Scutacaridae	MYMCF360-12	JX835893
Trombidiformes	Scutacaridae	MYMCG649-12	JX836783
Trombidiformes	Scutacaridae	SSPAA2303-13	KM826607
Trombidiformes	Scutacaridae	SSPAA6647-13	KM829848
Trombidiformes	Siteroptidae	CHACB1158-10	HM907242
Trombidiformes	Siteroptidae	CHACB1159-10	HM907243
Trombidiformes	Siteroptidae	CHACB167-10	HQ558449
Trombidiformes	Siteroptidae	CHACB373-10	HQ558549
Trombidiformes	Siteroptidae	CNJAF2021-12	KM834307
Trombidiformes	Siteroptidae	CNJAG1808-12	KM840137
Trombidiformes	Siteroptidae	CNPAC333-13	KM827520
Trombidiformes	Siteroptidae	CNPAD834-13	KM839115
Trombidiformes	Siteroptidae	CNPAF590-13	KM840328
Trombidiformes	Siteroptidae	CNPAF851-13	KM824431
Trombidiformes	Siteroptidae	CNPAG385-13	KM835083
Trombidiformes	Siteroptidae	CNPAH404-13	KM824522
Trombidiformes	Siteroptidae	CNRMF3237-12	KM833269
Trombidiformes	Siteroptidae	MYMCA1034-11	JX838317
Trombidiformes	Siteroptidae	MYMCA1168-11	JX834238
Trombidiformes	Siteroptidae	MYMCA1266-11	JX838117
Trombidiformes	Siteroptidae	MYMCA1438-11	JX837406
Trombidiformes	Siteroptidae	MYMCA868-11	JX838774
Trombidiformes	Siteroptidae	MYMCB861-11	JX834733
Trombidiformes	Siteroptidae	MYMCB862-11	JX834342
Trombidiformes	Siteroptidae	MYMCC010-11	JX836463
Trombidiformes	Siteroptidae	MYMCC075-11	JX836925

Trombidiformes	Siteroptidae		MYMCC115-11	JX836362
Trombidiformes	Siteroptidae		MYMCC127-11	JX835082
Trombidiformes	Siteroptidae		MYMCC151-11	JX834636
Trombidiformes	Siteroptidae		MYMCC285-11	JX836742
Trombidiformes	Siteroptidae		MYMCC645-11	JX836966
Trombidiformes	Siteroptidae		MYMCE168-12	JX838516
Trombidiformes	Siteroptidae		MYMCE169-12	JX834895
Trombidiformes	Siteroptidae		MYMCE651-12	JX836651
Trombidiformes	Siteroptidae		MYMCE652-12	JX838413
Trombidiformes	Siteroptidae		MYMCE679-12	JX835518
Trombidiformes	Siteroptidae		MYMCE700-12	JX835514
Trombidiformes	Siteroptidae		MYMCE751-12	JX834643
Trombidiformes	Siteroptidae		MYMCE855-12	JX836752
Trombidiformes	Siteroptidae		MYMCF232-12	JX837251
Trombidiformes	Siteroptidae		MYMCF234-12	JX836900
Trombidiformes	Siteroptidae		MYMCF250-12	JX835968
Trombidiformes	Siteroptidae		MYMCF251-12	JX837381
Trombidiformes	Siteroptidae		MYMCF272-12	JX836387
Trombidiformes	Siteroptidae		MYMCF273-12	JX837645
Trombidiformes	Siteroptidae		MYMCF315-12	JX837442
Trombidiformes	Siteroptidae		MYMCF355-12	JX836263
Trombidiformes	Siteroptidae		MYMCF356-12	JX833672
Trombidiformes	Siteroptidae		MYMCG145-12	JX833953
Trombidiformes	Siteroptidae		MYMCG267-12	JX834663
Trombidiformes	Siteroptidae		MYMCG268-12	JX833875
Trombidiformes	Siteroptidae		MYMCG393-12	JX837667
Trombidiformes	Siteroptidae		MYMCG394-12	JX834376
Trombidiformes	Siteroptidae		MYMCG395-12	JX836889
Trombidiformes	Siteroptidae		MYMCG396-12	JX838178
Trombidiformes	Siteroptidae		RBINA5682-13	KP979226
Trombidiformes	Siteroptidae		SSBAA2065-12	KM824811
Trombidiformes	Siteroptidae		SSWEE021-13	KM828988
Trombidiformes	Siteroptidae		SSWEE029-13	KM825973
Trombidiformes	Siteroptidae		SSWEE040-13	KM828921
Trombidiformes	Siteroptidae		SSWEE046-13	KM839804
Trombidiformes	Sperchontidae	*Sperchon*	CFWIA168-10	HQ938531
Trombidiformes	Sperchontidae	*Sperchon*	CFWIA169-10	HQ938532
Trombidiformes	Sperchontidae	*Sperchon*	CFWIA170-10	HQ938533
Trombidiformes	Sperchontidae	*Sperchon*	CFWIA171-10	HQ938534
Trombidiformes	Sperchontidae	*Sperchon*	CFWIA172-10	HQ938535
Trombidiformes	Sperchontidae	*Sperchon*	CFWIA173-10	HQ938536
Trombidiformes	Sperchontidae	*Sperchon*	CFWIA174-10	KR070048
Trombidiformes	Sperchontidae	*Sperchon*	CFWIA175-10	HQ938537
Trombidiformes	Sperchontidae	*Sperchon*	CFWIA176-10	HQ938538
Trombidiformes	Sperchontidae	*Sperchon*	CFWIA177-10	HQ938539
Trombidiformes	Sperchontidae	*Sperchon*	CFWIA178-10	HQ938540
Trombidiformes	Sperchontidae	*Sperchon*	CFWIA179-10	HQ938541
Trombidiformes	Sperchontidae	*Sperchon*	CFWIA180-10	HQ938542
Trombidiformes	Sperchontidae	*Sperchon*	CFWIA181-10	HQ938543
Trombidiformes	Sperchontidae	*Sperchon*	CFWIA182-10	HQ938544
Trombidiformes	Sperchontidae	*Sperchon*	CFWIA183-10	HQ938545
Trombidiformes	Sperchontidae	*Sperchon*	CFWIA184-10	HQ938546
Trombidiformes	Sperchontidae	*Sperchon*	CFWIA185-10	HQ938547

Trombidiformes	Sperchontidae	*Sperchon*	CFWIA609-10	HQ938797
Trombidiformes	Sperchontidae	*Sperchon*	CFWIA610-10	KR069931
Trombidiformes	Sperchontidae	*Sperchon*	CFWIA611-10	HQ938798
Trombidiformes	Sperchontidae	*Sperchon*	CFWIA612-10	HQ938799
Trombidiformes	Sperchontidae	*Sperchon*	CFWIA613-10	HQ938800
Trombidiformes	Sperchontidae	*Sperchon*	CFWIA614-10	HQ938801
Trombidiformes	Sperchontidae	*Sperchon*	CFWIA615-10	HQ938802
Trombidiformes	Sperchontidae	*Sperchon*	CFWIA616-10	HQ938803
Trombidiformes	Sperchontidae	*Sperchon*	CFWIA617-10	HQ938804
Trombidiformes	Sperchontidae	*Sperchon*	CFWIB566-10	HQ939597
Trombidiformes	Sperchontidae	*Sperchon*	CFWIB567-10	HQ939598
Trombidiformes	Sperchontidae	*Sperchon*	CFWIB568-10	HQ939599
Trombidiformes	Sperchontidae	*Sperchon*	CFWIB569-10	HQ939600
Trombidiformes	Sperchontidae	*Sperchon*	CFWIB570-10	HQ939601
Trombidiformes	Sperchontidae	*Sperchon*	CFWIB571-10	HQ939602
Trombidiformes	Sperchontidae	*Sperchon*	CFWIB572-10	HQ939603
Trombidiformes	Sperchontidae	*Sperchon*	CFWIB573-10	HQ939604
Trombidiformes	Sperchontidae	*Sperchon*	CFWIB574-10	HQ939605
Trombidiformes	Sperchontidae	*Sperchon sp. SP1*	GACAC040-12	AB530314
Trombidiformes	Stigmaeidae	*Eustigmaeus*	MYMCA306-11	JX837620
Trombidiformes	Stigmaeidae	*Eustigmaeus*	MYMCE847-12	JX835275
Trombidiformes	Stigmaeidae	*Eustigmaeus*	MYMCE848-12	JX836664
Trombidiformes	Stigmaeidae		CHACA040-08	JX834705
Trombidiformes	Stigmaeidae		CHACA503-09	JX834417
Trombidiformes	Stigmaeidae		CHACA507-09	JX834413
Trombidiformes	Stigmaeidae		CHACA521-09	KR069448
Trombidiformes	Stigmaeidae		CHACA530-09	JX837427
Trombidiformes	Stigmaeidae		CHACB059-10	HQ558367
Trombidiformes	Stigmaeidae		CHACB1044-10	JX837484
Trombidiformes	Stigmaeidae		CHACB1045-10	JX835364
Trombidiformes	Stigmaeidae		CHACB1046-10	JX838389
Trombidiformes	Stigmaeidae		CHACB1074-10	JX834147
Trombidiformes	Stigmaeidae		CHACB1149-10	KR070345
Trombidiformes	Stigmaeidae		CHACB1170-10	KR069793
Trombidiformes	Stigmaeidae		CHACB1201-10	KR070158
Trombidiformes	Stigmaeidae		CHACB216-10	HQ558476
Trombidiformes	Stigmaeidae		CHACB315-10	HM907302
Trombidiformes	Stigmaeidae		CHACB362-10	HQ558542
Trombidiformes	Stigmaeidae		CHACB582-10	JX838085
Trombidiformes	Stigmaeidae		CHACB583-10	HQ558684
Trombidiformes	Stigmaeidae		CHACB584-10	HQ558685
Trombidiformes	Stigmaeidae		CHACB585-10	HQ558686
Trombidiformes	Stigmaeidae		CHACB666-10	JX834254
Trombidiformes	Stigmaeidae		CHACB695-10	HQ558753
Trombidiformes	Stigmaeidae		CHACB760-10	HQ941474
Trombidiformes	Stigmaeidae		CHACB762-10	HQ941475
Trombidiformes	Stigmaeidae		CHACB763-10	HQ941476
Trombidiformes	Stigmaeidae		CHACB767-10	HQ941477
Trombidiformes	Stigmaeidae		CHACB769-10	HQ941478
Trombidiformes	Stigmaeidae		CHACB819-10	HQ941496
Trombidiformes	Stigmaeidae		CHACB820-10	JX838424
Trombidiformes	Stigmaeidae		CHACB840-10	HQ941510
Trombidiformes	Stigmaeidae		CHACB945-10	HM907366

Trombidiformes	Stigmaeidae	CHACC261-10	JX836292
Trombidiformes	Stigmaeidae	CNPAE452-13	KM834074
Trombidiformes	Stigmaeidae	CNPPE1451-12	KJ166386
Trombidiformes	Stigmaeidae	MIONB089-10	KR069820
Trombidiformes	Stigmaeidae	MIONB090-10	KR070452
Trombidiformes	Stigmaeidae	MIONB091-10	KR070733
Trombidiformes	Stigmaeidae	MYMCA1028-11	JX834288
Trombidiformes	Stigmaeidae	MYMCA1106-11	JX835169
Trombidiformes	Stigmaeidae	MYMCA1200-11	JX838185
Trombidiformes	Stigmaeidae	MYMCA1431-11	JX835700
Trombidiformes	Stigmaeidae	MYMCA1432-11	JX834517
Trombidiformes	Stigmaeidae	MYMCA1433-11	JX838523
Trombidiformes	Stigmaeidae	MYMCA173-11	JX838064
Trombidiformes	Stigmaeidae	MYMCA614-11	JX835126
Trombidiformes	Stigmaeidae	MYMCA813-11	JX836545
Trombidiformes	Stigmaeidae	MYMCA826-11	JX835407
Trombidiformes	Stigmaeidae	MYMCA874-11	JX837947
Trombidiformes	Stigmaeidae	MYMCB111-11	JX836345
Trombidiformes	Stigmaeidae	MYMCB130-11	JX836012
Trombidiformes	Stigmaeidae	MYMCB179-11	JX837844
Trombidiformes	Stigmaeidae	MYMCB774-11	JX835009
Trombidiformes	Stigmaeidae	MYMCC322-11	JX835895
Trombidiformes	Stigmaeidae	MYMCC333-11	JX836884
Trombidiformes	Stigmaeidae	MYMCC367-11	JX834924
Trombidiformes	Stigmaeidae	MYMCC812-11	JX838235
Trombidiformes	Stigmaeidae	MYMCC813-11	JX834166
Trombidiformes	Stigmaeidae	MYMCC814-11	JX838653
Trombidiformes	Stigmaeidae	MYMCC845-11	JX834146
Trombidiformes	Stigmaeidae	MYMCC918-11	JX838684
Trombidiformes	Stigmaeidae	MYMCD017-11	JX835485
Trombidiformes	Stigmaeidae	MYMCE082-12	JX833986
Trombidiformes	Stigmaeidae	MYMCE083-12	JX837693
Trombidiformes	Stigmaeidae	MYMCE289-12	JX838290
Trombidiformes	Stigmaeidae	MYMCE306-12	JX838181
Trombidiformes	Stigmaeidae	MYMCE356-12	JX834194
Trombidiformes	Stigmaeidae	MYMCE364-12	JX834339
Trombidiformes	Stigmaeidae	MYMCE516-12	JX836601
Trombidiformes	Stigmaeidae	MYMCE517-12	JX837538
Trombidiformes	Stigmaeidae	MYMCE687-12	JX834062
Trombidiformes	Stigmaeidae	MYMCE688-12	JX838154
Trombidiformes	Stigmaeidae	MYMCE690-12	JX837440
Trombidiformes	Stigmaeidae	MYMCE692-12	JX834506
Trombidiformes	Stigmaeidae	MYMCE748-12	JX835220
Trombidiformes	Stigmaeidae	MYMCE908-12	JX837787
Trombidiformes	Stigmaeidae	MYMCE909-12	JX837812
Trombidiformes	Stigmaeidae	MYMCF005-12	JX833690
Trombidiformes	Stigmaeidae	MYMCF150-12	JX836791
Trombidiformes	Stigmaeidae	MYMCF178-12	JX838690
Trombidiformes	Stigmaeidae	MYMCF316-12	JX835746
Trombidiformes	Stigmaeidae	MYMCF345-12	JX836268
Trombidiformes	Stigmaeidae	MYMCF346-12	JX836627
Trombidiformes	Stigmaeidae	MYMCF347-12	JX833856
Trombidiformes	Stigmaeidae	MYMCF555-12	JX835628

Trombidiformes	Stigmaeidae		MYMCF690-12	JX834300
Trombidiformes	Stigmaeidae		MYMCF778-12	JX835228
Trombidiformes	Stigmaeidae		MYMCF826-12	JX834512
Trombidiformes	Stigmaeidae		MYMCF886-12	JX835550
Trombidiformes	Stigmaeidae		MYMCF887-12	JX835964
Trombidiformes	Stigmaeidae		MYMCF889-12	JX835865
Trombidiformes	Stigmaeidae		MYMCF943-12	JX835383
Trombidiformes	Stigmaeidae		MYMCF944-12	JX834484
Trombidiformes	Stigmaeidae		MYMCF945-12	JX838177
Trombidiformes	Stigmaeidae		MYMCG308-12	JX836341
Trombidiformes	Stigmaeidae		MYMCG321-12	JX834626
Trombidiformes	Stigmaeidae		MYMCG322-12	JX836407
Trombidiformes	Stigmaeidae		MYMCG323-12	JX834530
Trombidiformes	Stigmaeidae		MYMCG427-12	JX837603
Trombidiformes	Stigmaeidae		MYMCG494-12	JX836040
Trombidiformes	Stigmaeidae		MYMCG505-12	JX835108
Trombidiformes	Stigmaeidae		MYMCG527-12	JX833947
Trombidiformes	Stigmaeidae		MYMCG528-12	JX837154
Trombidiformes	Stigmaeidae		MYMCG529-12	JX834868
Trombidiformes	Stigmaeidae		MYMCG613-12	JX835512
Trombidiformes	Stigmaeidae		MYTMC037-09	HQ966227
Trombidiformes	Stigmaeidae		SSJAB1016-13	KM834366
Trombidiformes	Tarsonemidae	*Acarapis externus*	GBCH7501-13	AB634839
Trombidiformes	Tarsonemidae	*Acarapis woodi*	GBCH7502-13	AB634838
Trombidiformes	Tarsonemidae	*Acarapis woodi*	GBCH7503-13	AB634837
Trombidiformes	Tarsonemidae		MYMCA1011-11	JX837461
Trombidiformes	Tarsonemidae		MYMCA618-11	JX834085
Trombidiformes	Tarsonemidae		MYMCA619-11	JX834483
Trombidiformes	Tarsonemidae		MYMCA809-11	JX837566
Trombidiformes	Tarsonemidae		MYMCC646-11	JX834523
Trombidiformes	Tarsonemidae		MYMCC924-11	JX834598
Trombidiformes	Tarsonemidae		MYMCE066-12	JX835933
Trombidiformes	Tarsonemidae		MYMCE113-12	JX836968
Trombidiformes	Tarsonemidae		MYMCE773-12	JX837761
Trombidiformes	Tarsonemidae		MYMCE774-12	JX836278
Trombidiformes	Tarsonemidae		MYMCE892-12	JX835807
Trombidiformes	Tarsonemidae		MYMCE942-12	JX834193
Trombidiformes	Tarsonemidae		MYMCF053-12	JX837383
Trombidiformes	Tarsonemidae		MYMCF054-12	JX838112
Trombidiformes	Tarsonemidae		MYMCF213-12	JX837477
Trombidiformes	Tarsonemidae		MYMCF402-12	JX836928
Trombidiformes	Tarsonemidae		MYMCF456-12	JX833718
Trombidiformes	Tarsonemidae		MYMCF537-12	JX837623
Trombidiformes	Tarsonemidae		MYMCF577-12	JX836323
Trombidiformes	Tarsonemidae		MYMCF824-12	JX834369
Trombidiformes	Tarsonemidae		MYMCG082-12	JX834603
Trombidiformes	Tarsonemidae		MYMCG084-12	JX835190
Trombidiformes	Tarsonemidae		MYMCG094-12	JX834907
Trombidiformes	Tarsonemidae		MYMCG171-12	JX836130
Trombidiformes	Tarsonemidae		MYMCG175-12	JX834566
Trombidiformes	Tarsonemidae		MYMCG186-12	JX835218
Trombidiformes	Tarsonemidae		MYMCG246-12	JX836238
Trombidiformes	Tarsonemidae		MYMCG247-12	JX836569

Trombidiformes	Tarsonemidae		MYMCG269-12	JX838003
Trombidiformes	Tarsonemidae		MYMCG390-12	JX836052
Trombidiformes	Tarsonemidae		MYMCG391-12	JX835090
Trombidiformes	Tarsonemidae		MYMCG392-12	JX838155
Trombidiformes	Tarsonemidae		MYMCG397-12	JX836157
Trombidiformes	Tarsonemidae		MYMCG662-12	JX833834
Trombidiformes	Tenuipalpidae	*Dolichotetranychus sp. APGD-2008*	GBCH3946-09	EU682418
Trombidiformes	Tenuipalpidae	*Raoiella*	GBCH7714-13	JF928432
Trombidiformes	Tenuipalpidae	*Raoiella*	GBCH7716-13	JF928430
Trombidiformes	Tenuipalpidae	*Raoiella*	GBCH7717-13	JF928429
Trombidiformes	Tenuipalpidae	*Raoiella*	GBCH7718-13	JF928428
Trombidiformes	Tenuipalpidae	*Raoiella*	GBCH7719-13	JF928427
Trombidiformes	Tenuipalpidae	*Raoiella*	GBCH7720-13	JF928426
Trombidiformes	Tenuipalpidae	*Raoiella*	GBCH7721-13	JF928425
Trombidiformes	Tenuipalpidae	*Raoiella*	GBCH7722-13	JF928424
Trombidiformes	Tenuipalpidae	*Raoiella*	GBCH7723-13	JF928423
Trombidiformes	Tenuipalpidae	*Raoiella*	GBCH7724-13	JF928422
Trombidiformes	Tenuipalpidae	*Raoiella*	GBCH7725-13	JF928421
Trombidiformes	Tenuipalpidae	*Raoiella indica*	GBCH3921-09	EU682445
Trombidiformes	Tenuipalpidae	*Raoiella indica*	GBCH3922-09	EU682444
Trombidiformes	Tenuipalpidae	*Raoiella indica*	GBCH3923-09	EU682443
Trombidiformes	Tenuipalpidae	*Raoiella indica*	GBCH3924-09	EU682442
Trombidiformes	Tenuipalpidae	*Raoiella indica*	GBCH3925-09	EU682441
Trombidiformes	Tenuipalpidae	*Raoiella indica*	GBCH3926-09	EU682440
Trombidiformes	Tenuipalpidae	*Raoiella indica*	GBCH3927-09	EU682439
Trombidiformes	Tenuipalpidae	*Raoiella indica*	GBCH3928-09	EU682438
Trombidiformes	Tenuipalpidae	*Raoiella indica*	GBCH3929-09	EU682437
Trombidiformes	Tenuipalpidae	*Raoiella indica*	GBCH3930-09	EU682436
Trombidiformes	Tenuipalpidae	*Raoiella indica*	GBCH3931-09	EU682434
Trombidiformes	Tenuipalpidae	*Raoiella indica*	GBCH3932-09	EU682433
Trombidiformes	Tenuipalpidae	*Raoiella indica*	GBCH3933-09	EU682432
Trombidiformes	Tenuipalpidae	*Raoiella indica*	GBCH3934-09	EU682431
Trombidiformes	Tenuipalpidae	*Raoiella indica*	GBCH3935-09	EU682430
Trombidiformes	Tenuipalpidae	*Raoiella indica*	GBCH3936-09	EU682428
Trombidiformes	Tenuipalpidae	*Raoiella indica*	GBCH3937-09	EU682427
Trombidiformes	Tenuipalpidae	*Raoiella indica*	GBCH3939-09	EU682425
Trombidiformes	Tenuipalpidae	*Raoiella indica*	GBCH3940-09	EU682424
Trombidiformes	Tenuipalpidae	*Raoiella sp. APGD-2008*	GBCH3941-09	EU682423
Trombidiformes	Tenuipalpidae	*Raoiella sp. APGD-2008*	GBCH3942-09	EU682422
Trombidiformes	Tenuipalpidae	*Raoiella sp. APGD-2008*	GBCH3943-09	EU682421
Trombidiformes	Tenuipalpidae	*Raoiella sp. APGD-2008*	GBCH3944-09	EU682420
Trombidiformes	Tenuipalpidae	*Raoiella sp. APGD-2008*	GBCH3945-09	EU682419
Trombidiformes	Tetranychidae	*Eutetranychus shii*	GBCH7958-13	AB531841
Trombidiformes	Tetranychidae	*Oligonychus perseae*	GBCH8390-13	KF011473
Trombidiformes	Tetranychidae	*Oligonychus perseae*	GBCH8391-13	KF011472
Trombidiformes	Tetranychidae	*Oligonychus perseae*	GBCH8393-13	KF011470
Trombidiformes	Tetranychidae	*Oligonychus perseae*	GBCH8395-13	KF011468
Trombidiformes	Tetranychidae	*Oligonychus punicae*	GBCH8396-13	KF011467
Trombidiformes	Tetranychidae	*Oligonychus punicae*	GBCH8397-13	KF011466
Trombidiformes	Tetranychidae	*Oligonychus punicae*	GBCH8398-13	KF011465
Trombidiformes	Tetranychidae	*Oligonychus punicae*	GBCH8399-13	KF011464
Trombidiformes	Tetranychidae	*Oligonychus punicae*	GBCH8401-13	KF011461
Trombidiformes	Tetranychidae	*Oligonychus punicae*	GBCH8402-13	KF011460

Trombidiformes	Tetranychidae	*Oligonychus punicae*	GBCH8404-13	KF011458
Trombidiformes	Tetranychidae	*Oligonychus punicae*	GBCH8405-13	KF011457
Trombidiformes	Tetranychidae	*Oligonychus punicae*	GBCH8406-13	KF011456
Trombidiformes	Tetranychidae	*Oligonychus punicae*	GBCH8407-13	KF011455
Trombidiformes	Tetranychidae	*Oligonychus punicae*	GBCH8409-13	KF011453
Trombidiformes	Tetranychidae	*Oligonychus punicae*	GBCH8410-13	KF011452
Trombidiformes	Tetranychidae	*Panonychus ulmi*	GBCH3918-09	NC_012571
Trombidiformes	Tetranychidae	*Stigmaeopsis celarius*	GBCH7976-13	AB531823
Trombidiformes	Tetranychidae	*Stigmaeopsis celarius*	GBCH8380-13	AB429469
Trombidiformes	Tetranychidae	*Stigmaeopsis longus*	GBCH7964-13	AB531835
Trombidiformes	Tetranychidae	*Stigmaeopsis longus*	GBCH7965-13	AB531834
Trombidiformes	Tetranychidae	*Stigmaeopsis longus*	GBCH7966-13	AB531833
Trombidiformes	Tetranychidae	*Stigmaeopsis longus*	GBCH7967-13	AB531832
Trombidiformes	Tetranychidae	*Stigmaeopsis longus*	GBCH7968-13	AB531831
Trombidiformes	Tetranychidae	*Stigmaeopsis longus*	GBCH7969-13	AB531830
Trombidiformes	Tetranychidae	*Stigmaeopsis longus*	GBCH7970-13	AB531829
Trombidiformes	Tetranychidae	*Stigmaeopsis longus*	GBCH7971-13	AB531828
Trombidiformes	Tetranychidae	*Stigmaeopsis longus*	GBCH7972-13	AB531827
Trombidiformes	Tetranychidae	*Stigmaeopsis longus*	GBCH7973-13	AB531826
Trombidiformes	Tetranychidae	*Stigmaeopsis longus*	GBCH7974-13	AB531825
Trombidiformes	Tetranychidae	*Stigmaeopsis longus*	GBCH8375-13	AB429485
Trombidiformes	Tetranychidae	*Stigmaeopsis longus*	GBCH8382-13	AB429467
Trombidiformes	Tetranychidae	*Stigmaeopsis longus*	GBCH8384-13	AB429465
Trombidiformes	Tetranychidae	*Stigmaeopsis miscanthi*	GBCH8387-13	AB429422
Trombidiformes	Tetranychidae	*Stigmaeopsis miscanthi*	GBCH8388-13	AB429416
Trombidiformes	Tetranychidae	*Stigmaeopsis sp. Kikuchi_1*	GBCH8385-13	AB429450
Trombidiformes	Tetranychidae	*Stigmaeopsis sp. Kikuchi_2*	GBCH8386-13	AB429449
Trombidiformes	Tetranychidae	*Tetranychus cinnabarinus*	CYTC3553-12	NC_014399
Trombidiformes	Tetranychidae	*Tetranychus cinnabarinus*	CYTC4960-12	HM753535
Trombidiformes	Tetranychidae	*Tetranychus kanzawai*	GBCH0357-06	AY044642
Trombidiformes	Tetranychidae	*Tetranychus kanzawai*	GBCH0358-06	AY044643
Trombidiformes	Tetranychidae	*Tetranychus kanzawai*	GBCH0359-06	AY044644
Trombidiformes	Tetranychidae	*Tetranychus kanzawai*	GBCH0360-06	AY044645
Trombidiformes	Tetranychidae	*Tetranychus kanzawai*	GBCH0361-06	AY044646
Trombidiformes	Tetranychidae	*Tetranychus neocaledonicus*	GBCH11792-13	JX075251
Trombidiformes	Tetranychidae	*Tetranychus truncatus*	GBCH11794-13	JX075249
Trombidiformes	Tetranychidae	*Tetranychus turkestani*	GBCH0242-06	AJ316600
Trombidiformes	Tetranychidae	*Tetranychus turkestani*	GBCH0243-06	AJ316601
Trombidiformes	Tetranychidae	*Tetranychus turkestani*	GBCH0250-06	AJ414583
Trombidiformes	Tetranychidae	*Tetranychus urticae*	CYTC4552-12	EU345430
Trombidiformes	Tetranychidae	*Tetranychus urticae*	GBCH11793-13	JX075250
Trombidiformes	Tetranychidae	*Tetranychus urticae*	GBCH2385-08	NC_010526
Trombidiformes	Tetranychidae		CHACB128-10	HQ558415
Trombidiformes	Tetranychidae		CHACB131-10	HQ558416
Trombidiformes	Tetranychidae		CHACC011-10	HM907412
Trombidiformes	Tetranychidae		CNBPC232-12	KM831509
Trombidiformes	Tetranychidae		CNBPD583-12	KM827353
Trombidiformes	Tetranychidae		CNBPD585-12	KM829869
Trombidiformes	Tetranychidae		CNBPD586-12	KM833035
Trombidiformes	Tetranychidae		CNBPF129-12	KM829135
Trombidiformes	Tetranychidae		CNBPJ299-13	KM824572
Trombidiformes	Tetranychidae		CNBPJ300-13	KM827831
Trombidiformes	Tetranychidae		CNBPJ302-13	KM830661

Trombidiformes	Tetranychidae	CNBPJ304-13	KM831839
Trombidiformes	Tetranychidae	CNBPJ305-13	KM839518
Trombidiformes	Tetranychidae	CNBPJ306-13	KM831176
Trombidiformes	Tetranychidae	CNBPQ357-13	KM835821
Trombidiformes	Tetranychidae	CNBPQ360-13	KM834155
Trombidiformes	Tetranychidae	CNBPQ363-13	KM833697
Trombidiformes	Tetranychidae	CNBPQ385-13	KM832908
Trombidiformes	Tetranychidae	CNBPQ387-13	KM828224
Trombidiformes	Tetranychidae	CNBPQ389-13	KM833574
Trombidiformes	Tetranychidae	CNGIA133-12	KM836675
Trombidiformes	Tetranychidae	CNGIB576-12	KM831806
Trombidiformes	Tetranychidae	CNGIB577-12	KM825636
Trombidiformes	Tetranychidae	CNGIB582-12	KM831358
Trombidiformes	Tetranychidae	CNGIB587-12	KM827486
Trombidiformes	Tetranychidae	CNGIB588-12	KM830627
Trombidiformes	Tetranychidae	CNGIB589-12	KM829049
Trombidiformes	Tetranychidae	CNGIB590-12	KM835986
Trombidiformes	Tetranychidae	CNGIC299-12	KM826082
Trombidiformes	Tetranychidae	CNGIC300-12	KM826178
Trombidiformes	Tetranychidae	CNGLF2453-13	KM826428
Trombidiformes	Tetranychidae	CNGLF2658-13	KM829116
Trombidiformes	Tetranychidae	CNGMC2048-13	KR069512
Trombidiformes	Tetranychidae	CNJAC1485-12	KM834828
Trombidiformes	Tetranychidae	CNJAE1138-12	KM831891
Trombidiformes	Tetranychidae	CNPAF876-13	KM839618
Trombidiformes	Tetranychidae	CNPAG375-13	KM824684
Trombidiformes	Tetranychidae	CNPAG382-13	KM833817
Trombidiformes	Tetranychidae	CNPPE2125-12	KJ166555
Trombidiformes	Tetranychidae	CNPPE2131-12	KJ092874
Trombidiformes	Tetranychidae	CNPPF1046-12	KJ166102
Trombidiformes	Tetranychidae	CNPPJ1865-12	KJ207735
Trombidiformes	Tetranychidae	CNRMA1045-12	KM837623
Trombidiformes	Tetranychidae	CNRMA1047-12	KM833745
Trombidiformes	Tetranychidae	CNRMA1048-12	KM828451
Trombidiformes	Tetranychidae	CNRMA1049-12	KM830048
Trombidiformes	Tetranychidae	CNRMA1050-12	KM828896
Trombidiformes	Tetranychidae	CNRMA1051-12	KM828348
Trombidiformes	Tetranychidae	CNRMA1054-12	KM835669
Trombidiformes	Tetranychidae	CNRMA1056-12	KM827299
Trombidiformes	Tetranychidae	CNRMA1057-12	KM824070
Trombidiformes	Tetranychidae	CNRMA1058-12	KM832547
Trombidiformes	Tetranychidae	CNRMA1059-12	KM827713
Trombidiformes	Tetranychidae	CNRMA1060-12	KM835295
Trombidiformes	Tetranychidae	CNRMA1063-12	KM829308
Trombidiformes	Tetranychidae	CNRMA1064-12	KM827047
Trombidiformes	Tetranychidae	CNRMA1066-12	KM837967
Trombidiformes	Tetranychidae	CNRMA1067-12	KM833307
Trombidiformes	Tetranychidae	CNRMA1068-12	KM831442
Trombidiformes	Tetranychidae	CNRMA1071-12	KM828007
Trombidiformes	Tetranychidae	CNRMA1072-12	KM832676
Trombidiformes	Tetranychidae	CNRMA1073-12	KM832577
Trombidiformes	Tetranychidae	CNRMA1074-12	KM833427
Trombidiformes	Tetranychidae	CNRMA1077-12	KM840193

Trombidiformes	Tetranychidae	CNRMA1078-12	KM824679
Trombidiformes	Tetranychidae	CNRMA1079-12	KM834470
Trombidiformes	Tetranychidae	CNRMA1080-12	KM836415
Trombidiformes	Tetranychidae	CNRMA1081-12	KM826276
Trombidiformes	Tetranychidae	CNRMA1082-12	KM835208
Trombidiformes	Tetranychidae	CNRMA1083-12	KM834300
Trombidiformes	Tetranychidae	CNRMA1084-12	KM836726
Trombidiformes	Tetranychidae	CNRMA1085-12	KM839753
Trombidiformes	Tetranychidae	CNRMA1087-12	KM827876
Trombidiformes	Tetranychidae	CNRMA1088-12	KM839883
Trombidiformes	Tetranychidae	CNRMA1089-12	KM824874
Trombidiformes	Tetranychidae	CNRMA1090-12	KM838276
Trombidiformes	Tetranychidae	CNRMA1091-12	KM834790
Trombidiformes	Tetranychidae	CNRMA1092-12	KM834315
Trombidiformes	Tetranychidae	CNRMA1093-12	KM838582
Trombidiformes	Tetranychidae	CNRMA1094-12	KM829615
Trombidiformes	Tetranychidae	CNRMA1095-12	KM835558
Trombidiformes	Tetranychidae	CNRMA1096-12	KM824538
Trombidiformes	Tetranychidae	CNRMA1097-12	KM826784
Trombidiformes	Tetranychidae	CNRMA1098-12	KM839328
Trombidiformes	Tetranychidae	CNRMA1099-12	KM825435
Trombidiformes	Tetranychidae	CNRMA1100-12	KM828465
Trombidiformes	Tetranychidae	CNRMA1101-12	KM825981
Trombidiformes	Tetranychidae	CNRMA1103-12	KM826342
Trombidiformes	Tetranychidae	CNRMC1665-12	KM826643
Trombidiformes	Tetranychidae	CNRMC1682-12	KM837897
Trombidiformes	Tetranychidae	CNRME4794-12	KM825316
Trombidiformes	Tetranychidae	CNRMF3243-12	KM840392
Trombidiformes	Tetranychidae	CNSLN205-13	KM828286
Trombidiformes	Tetranychidae	CNSLP438-13	KM837846
Trombidiformes	Tetranychidae	MIONB099-10	KP979148
Trombidiformes	Tetranychidae	MIONB110-10	KP979272
Trombidiformes	Tetranychidae	MIONB111-10	KR069145
Trombidiformes	Tetranychidae	MIONB112-10	KR070590
Trombidiformes	Tetranychidae	MIONB113-10	KR070717
Trombidiformes	Tetranychidae	MIONB114-10	KR069584
Trombidiformes	Tetranychidae	MIONB144-10	KR070641
Trombidiformes	Tetranychidae	MIONB160-10	KR070463
Trombidiformes	Tetranychidae	MIONB161-10	KR070205
Trombidiformes	Tetranychidae	MIONB321-10	KR069648
Trombidiformes	Tetranychidae	MIONB338-10	KP979319
Trombidiformes	Tetranychidae	MIONB339-10	KR070042
Trombidiformes	Tetranychidae	MYMCA964-11	JX837997
Trombidiformes	Tetranychidae	MYMCB627-11	JX834670
Trombidiformes	Tetranychidae	MYMCB628-11	JX838609
Trombidiformes	Tetranychidae	MYMCB636-11	JX834351
Trombidiformes	Tetranychidae	MYMCD141-11	JX834735
Trombidiformes	Tetranychidae	MYMCD142-11	JX834514
Trombidiformes	Tetranychidae	PHAUG1656-11	KR070563
Trombidiformes	Tetranychidae	PHAUG1658-11	KR070218
Trombidiformes	Tetranychidae	PHAUG1662-11	KR070004
Trombidiformes	Tetranychidae	PHAUG1663-11	KR069568
Trombidiformes	Tetranychidae	PHAUG1666-11	KR070554

Trombidiformes	Tetranychidae		PHAUG1670-11	KR069908
Trombidiformes	Tetranychidae		PHAUG1671-11	KR070594
Trombidiformes	Tetranychidae		PHAUG1676-11	KR069817
Trombidiformes	Tetranychidae		PHAUG1677-11	KP979254
Trombidiformes	Tetranychidae		PHAUG1680-11	KR069462
Trombidiformes	Tetranychidae		PHAUG1682-11	KR069856
Trombidiformes	Tetranychidae		PHAUG1686-11	KR070139
Trombidiformes	Tetranychidae		SMTPB14235-13	KR069323
Trombidiformes	Tetranychidae		SMTPB14236-13	KR070352
Trombidiformes	Tetranychidae		SMTPB14237-13	KP979312
Trombidiformes	Tetranychidae		SMTPB14240-13	KR069412
Trombidiformes	Tetranychidae		SSBAA3636-12	KM828883
Trombidiformes	Tetranychidae		SSBAD4009-12	KM835825
Trombidiformes	Tetranychidae		SSPAA2250-13	KM826727
Trombidiformes	Tetranychidae		SSPAC6755-13	KM837806
Trombidiformes	Tetranychidae		SSPAC7886-13	KM827506
Trombidiformes	Tetranychidae		SSWLC2257-13	KM833166
Trombidiformes	Thyasidae	*Thyopsis*	MYMCA103-11	JX836733
Trombidiformes	Thyasidae	*Zschokkea*	CHACA049-08	JX833831
Trombidiformes	Thyasidae	*Zschokkea*	MYMCA048-11	JX833918
Trombidiformes	Thyasidae	*Zschokkea*	MYMCA049-11	JX833913
Trombidiformes	Thyasidae	*Zschokkea*	MYMCB450-11	JX834026
Trombidiformes	Torrenticolidae	*Torrenticola*	CFWIA190-10	HQ938552
Trombidiformes	Torrenticolidae	*Torrenticola*	CFWIA191-10	KR069794
Trombidiformes	Torrenticolidae	*Torrenticola*	CFWIA192-10	HQ938553
Trombidiformes	Torrenticolidae	*Torrenticola*	CFWIA193-10	KR069854
Trombidiformes	Torrenticolidae	*Torrenticola*	CFWIA194-10	KR069639
Trombidiformes	Torrenticolidae	*Torrenticola*	CFWIA195-10	HQ938554
Trombidiformes	Torrenticolidae	*Torrenticola*	CFWIA196-10	HQ938555
Trombidiformes	Torrenticolidae	*Torrenticola*	CFWIA197-10	HQ938556
Trombidiformes	Torrenticolidae	*Torrenticola*	CFWIA623-10	HQ938807
Trombidiformes	Torrenticolidae	*Torrenticola*	CFWIA624-10	KR070558
Trombidiformes	Torrenticolidae	*Torrenticola*	CFWIA625-10	HQ938808
Trombidiformes	Torrenticolidae	*Torrenticola*	CFWIA626-10	HQ938809
Trombidiformes	Torrenticolidae	*Torrenticola*	CFWIA627-10	HQ938810
Trombidiformes	Torrenticolidae	*Torrenticola*	CFWIA628-10	HQ938811
Trombidiformes	Torrenticolidae	*Torrenticola*	CFWIA629-10	KR069862
Trombidiformes	Torrenticolidae	*Torrenticola*	CFWIA631-10	KR070350
Trombidiformes	Torrenticolidae	*Torrenticola*	CFWIA632-10	HQ938812
Trombidiformes	Torrenticolidae	*Torrenticola*	CFWIA633-10	HQ938813
Trombidiformes	Torrenticolidae	*Torrenticola*	CFWIA634-10	HQ938814
Trombidiformes	Torrenticolidae	*Torrenticola*	CFWIA635-10	HQ938815
Trombidiformes	Torrenticolidae	*Torrenticola*	CFWIA636-10	HQ938816
Trombidiformes	Torrenticolidae	*Torrenticola*	CFWIA637-10	HQ938817
Trombidiformes	Torrenticolidae	*Torrenticola*	CFWIA638-10	HQ938818
Trombidiformes	Torrenticolidae	*Torrenticola*	CFWIA639-10	HQ938819
Trombidiformes	Torrenticolidae	*Torrenticola*	CFWIA640-10	HQ938820
Trombidiformes	Torrenticolidae	*Torrenticola*	CFWIB549-10	HQ939582
Trombidiformes	Torrenticolidae	*Torrenticola*	CFWIB550-10	HQ939583
Trombidiformes	Torrenticolidae	*Torrenticola*	CFWIB551-10	HQ939584
Trombidiformes	Torrenticolidae	*Torrenticola*	CFWIB552-10	HQ939585
Trombidiformes	Torrenticolidae	*Torrenticola*	CFWIB553-10	HQ939586
Trombidiformes	Torrenticolidae	*Torrenticola*	CFWIB554-10	HQ939587

Trombidiformes	Torrenticolidae	*Torrenticola*	CFWIB557-10	HQ939589
Trombidiformes	Torrenticolidae	*Torrenticola*	CFWIB558-10	HQ939590
Trombidiformes	Torrenticolidae	*Torrenticola*	CFWIB559-10	HQ939591
Trombidiformes	Torrenticolidae	*Torrenticola*	CFWIB560-10	HQ939592
Trombidiformes	Torrenticolidae	*Torrenticola amplexa*	GBCH7806-13	JN018109
Trombidiformes	Torrenticolidae	*Torrenticola lukai*	GBCH12086-13	JX629054
Trombidiformes	Torrenticolidae	*Torrenticola lukai*	GBCH12087-13	JX629053
Trombidiformes	Torrenticolidae	*Torrenticola lundbladi*	GBCH12088-13	JX629052
Trombidiformes	Torrenticolidae	*Torrenticola lundbladi*	GBCH12089-13	JX629051
Trombidiformes	Torrenticolidae	*Torrenticola lundbladi*	GBCH12090-13	JX629050
Trombidiformes	Torrenticolidae	*Torrenticola sp. To*	GACAC042-12	AB530316
Trombidiformes	Torrenticolidae		CFWIA641-10	KR070011
Trombidiformes	Torrenticolidae		CFWIB555-10	KR069511
Trombidiformes	Torrenticolidae		CFWIB556-10	HQ939588
Trombidiformes	Trombiculidae	*Ascoschoengastia sp. TATW-1*	GBCH2296-08	NC_010596
Trombidiformes	Trombiculidae	*Ascoschoengastia sp. TATW-1*	GBCH2374-08	AB300501
Trombidiformes	Trombiculidae	*Leptotrombidium akamushi*	GBCH0045-06	AB194045
Trombidiformes	Trombiculidae	*Leptotrombidium akamushi*	GBCH1692-06	NC_007601
Trombidiformes	Trombiculidae	*Leptotrombidium chiangraiensis*	GBCH2383-08	AB300488
Trombidiformes	Trombiculidae	*Leptotrombidium chiangraiensis*	GBCH7520-13	HQ324988
Trombidiformes	Trombiculidae	*Leptotrombidium chiangraiensis*	GBCH7521-13	HQ324987
Trombidiformes	Trombiculidae	*Leptotrombidium chiangraiensis*	GBCH7522-13	HQ324986
Trombidiformes	Trombiculidae	*Leptotrombidium chiangraiensis*	GBCH7523-13	HQ324985
Trombidiformes	Trombiculidae	*Leptotrombidium chiangraiensis*	GBCH7524-13	HQ324984
Trombidiformes	Trombiculidae	*Leptotrombidium chiangraiensis*	GBCH7525-13	HQ324983
Trombidiformes	Trombiculidae	*Leptotrombidium chiangraiensis*	GBCH7526-13	HQ324982
Trombidiformes	Trombiculidae	*Leptotrombidium chiangraiensis*	GBCH7527-13	HQ324973
Trombidiformes	Trombiculidae	*Leptotrombidium chiangraiensis*	GBCH7528-13	HQ324972
Trombidiformes	Trombiculidae	*Leptotrombidium chiangraiensis*	GBCH7529-13	HQ324971
Trombidiformes	Trombiculidae	*Leptotrombidium chiangraiensis*	GBCH7530-13	HQ324970
Trombidiformes	Trombiculidae	*Leptotrombidium chiangraiensis*	GBCH7531-13	HQ324969
Trombidiformes	Trombiculidae	*Leptotrombidium chiangraiensis*	GBCH7532-13	HQ324968
Trombidiformes	Trombiculidae	*Leptotrombidium chiangraiensis*	GBCH7533-13	HQ324967
Trombidiformes	Trombiculidae	*Leptotrombidium chiangraiensis*	GBCH7534-13	HQ324966
Trombidiformes	Trombiculidae	*Leptotrombidium chiangraiensis*	GBCH7535-13	HQ324965
Trombidiformes	Trombiculidae	*Leptotrombidium chiangraiensis*	GBCH7536-13	HQ324964
Trombidiformes	Trombiculidae	*Leptotrombidium chiangraiensis*	GBCH7537-13	HQ324963
Trombidiformes	Trombiculidae	*Leptotrombidium chiangraiensis*	GBCH7538-13	HQ324962
Trombidiformes	Trombiculidae	*Leptotrombidium chiangraiensis*	GBCH7539-13	HQ324961
Trombidiformes	Trombiculidae	*Leptotrombidium chiangraiensis*	GBCH7540-13	HQ324960
Trombidiformes	Trombiculidae	*Leptotrombidium chiangraiensis*	GBCH7541-13	HQ324959
Trombidiformes	Trombiculidae	*Leptotrombidium chiangraiensis*	GBCH7542-13	HQ324958
Trombidiformes	Trombiculidae	*Leptotrombidium chiangraiensis*	GBCH7543-13	HQ324957
Trombidiformes	Trombiculidae	*Leptotrombidium chiangraiensis*	GBCH7544-13	HQ324956
Trombidiformes	Trombiculidae	*Leptotrombidium chiangraiensis*	GBCH7545-13	HQ324955
Trombidiformes	Trombiculidae	*Leptotrombidium chiangraiensis*	GBCH7546-13	HQ324954
Trombidiformes	Trombiculidae	*Leptotrombidium chiangraiensis*	GBCH7547-13	HQ324953
Trombidiformes	Trombiculidae	*Leptotrombidium chiangraiensis*	GBCH7548-13	HQ324952
Trombidiformes	Trombiculidae	*Leptotrombidium chiangraiensis*	GBCH7549-13	HQ324951
Trombidiformes	Trombiculidae	*Leptotrombidium chiangraiensis*	GBCH7550-13	HQ324950
Trombidiformes	Trombiculidae	*Leptotrombidium deliense*	GBCH0044-06	AB194044
Trombidiformes	Trombiculidae	*Leptotrombidium deliense*	GBCH1691-06	NC_007600
Trombidiformes	Trombiculidae	*Leptotrombidium deliense*	GBCH7512-13	HQ324981

Trombidiformes	Trombiculidae	*Leptotrombidium deliense*	GBCH7513-13	HQ324980
Trombidiformes	Trombiculidae	*Leptotrombidium deliense*	GBCH7514-13	HQ324979
Trombidiformes	Trombiculidae	*Leptotrombidium deliense*	GBCH7515-13	HQ324978
Trombidiformes	Trombiculidae	*Leptotrombidium deliense*	GBCH7516-13	HQ324977
Trombidiformes	Trombiculidae	*Leptotrombidium deliense*	GBCH7517-13	HQ324976
Trombidiformes	Trombiculidae	*Leptotrombidium deliense*	GBCH7518-13	HQ324975
Trombidiformes	Trombiculidae	*Leptotrombidium deliense*	GBCH7519-13	HQ324974
Trombidiformes	Trombiculidae	*Leptotrombidium fuji*	GBCH2378-08	AB300496
Trombidiformes	Trombiculidae	*Leptotrombidium imphalum*	GBCH2381-08	AB300490
Trombidiformes	Trombiculidae	*Leptotrombidium imphalum*	GBCH7551-13	HQ324949
Trombidiformes	Trombiculidae	*Leptotrombidium imphalum*	GBCH7552-13	HQ324948
Trombidiformes	Trombiculidae	*Leptotrombidium imphalum*	GBCH7553-13	HQ324947
Trombidiformes	Trombiculidae	*Leptotrombidium imphalum*	GBCH7554-13	HQ324946
Trombidiformes	Trombiculidae	*Leptotrombidium imphalum*	GBCH7555-13	HQ324945
Trombidiformes	Trombiculidae	*Leptotrombidium imphalum*	GBCH7556-13	HQ324944
Trombidiformes	Trombiculidae	*Leptotrombidium imphalum*	GBCH7557-13	HQ324943
Trombidiformes	Trombiculidae	*Leptotrombidium imphalum*	GBCH7558-13	HQ324942
Trombidiformes	Trombiculidae	*Leptotrombidium imphalum*	GBCH7559-13	HQ324941
Trombidiformes	Trombiculidae	*Leptotrombidium imphalum*	GBCH7560-13	HQ324940
Trombidiformes	Trombiculidae	*Leptotrombidium imphalum*	GBCH7561-13	HQ324939
Trombidiformes	Trombiculidae	*Leptotrombidium imphalum*	GBCH7562-13	HQ324938
Trombidiformes	Trombiculidae	*Leptotrombidium imphalum*	GBCH7563-13	HQ324937
Trombidiformes	Trombiculidae	*Leptotrombidium imphalum*	GBCH7564-13	HQ324936
Trombidiformes	Trombiculidae	*Leptotrombidium imphalum*	GBCH7565-13	HQ324935
Trombidiformes	Trombiculidae	*Leptotrombidium imphalum*	GBCH7566-13	HQ324934
Trombidiformes	Trombiculidae	*Leptotrombidium imphalum*	GBCH7567-13	HQ324933
Trombidiformes	Trombiculidae	*Leptotrombidium intermedium*	GBCH2380-08	AB300492
Trombidiformes	Trombiculidae	*Leptotrombidium pallidum*	GBCH0043-06	AB180098
Trombidiformes	Trombiculidae	*Leptotrombidium pallidum*	GBCH1690-06	NC_007177
Trombidiformes	Trombiculidae	*Leptotrombidium palpale*	GBCH2376-08	AB300499
Trombidiformes	Trombiculidae	*Leptotrombidium scutellare*	GBCH2377-08	AB300498
Trombidiformes	Trombiculidae	*Leptotrombidium sp. TLMW-1*	GBCH2379-08	AB300494
Trombidiformes	Trombiculidae	*Neotrombicula*	SSJAE11558-13	KM840032
Trombidiformes	Trombiculidae	*Neotrombicula microti*	MYMCE126-12	JX836578
Trombidiformes	Trombiculidae	*Neotrombicula microti*	MYMCE127-12	JX835076
Trombidiformes	Trombiculidae	*Neotrombicula microti*	MYMCE529-12	JX834814
Trombidiformes	Trombiculidae	*Walchia hayashii*	GBCH2295-08	NC_010595
Trombidiformes	Trombiculidae	*Walchia hayashii*	GBCH2375-08	AB300500
Trombidiformes	Trombiculidae		MYMCF885-12	JX834572
Trombidiformes	Trombiculidae		MYMCG116-12	JX834448
Trombidiformes	Trombiculidae		SSBAC3351-12	KM825953
Trombidiformes	Trombidiidae		ARCN008-10	KM834920
Trombidiformes	Trombidiidae		CHACA1086-10	HM907127
Trombidiformes	Trombidiidae		CHACA1124-10	JX836254
Trombidiformes	Trombidiidae		CHACA1136-10	JX834840
Trombidiformes	Trombidiidae		CHACA1137-10	JX838025
Trombidiformes	Trombidiidae		CHACA1138-10	JX836497
Trombidiformes	Trombidiidae		CHACA1139-10	JX835548
Trombidiformes	Trombidiidae		CHACA1140-10	JX834887
Trombidiformes	Trombidiidae		CHACA636-09	JX837184
Trombidiformes	Trombidiidae		CHACA639-09	JX835853
Trombidiformes	Trombidiidae		CNBAD668-12	KM840640
Trombidiformes	Trombidiidae		CNBAF196-12	KM830761

Trombidiformes	Trombidiidae	CNBAF197-12	KM829217
Trombidiformes	Trombidiidae	CNBAF198-12	KM837130
Trombidiformes	Trombidiidae	CNBPC228-12	KM839117
Trombidiformes	Trombidiidae	CNBPC229-12	KM833668
Trombidiformes	Trombidiidae	CNBPC230-12	KM832931
Trombidiformes	Trombidiidae	CNBPL274-13	KM839667
Trombidiformes	Trombidiidae	CNBPL297-13	KM826179
Trombidiformes	Trombidiidae	CNBPL298-13	KM830475
Trombidiformes	Trombidiidae	CNBPL299-13	KM834136
Trombidiformes	Trombidiidae	CNGID225-12	KM839579
Trombidiformes	Trombidiidae	CNGIE402-12	KM833376
Trombidiformes	Trombidiidae	CNPAE450-13	KM838077
Trombidiformes	Trombidiidae	CNPAF858-13	KM828256
Trombidiformes	Trombidiidae	JSSEP1135-12	KP979229
Trombidiformes	Trombidiidae	MIONB460-11	KM838636
Trombidiformes	Trombidiidae	MIONB461-11	KM831161
Trombidiformes	Trombidiidae	MIONB463-11	KM835234
Trombidiformes	Trombidiidae	MIONB483-11	KM833193
Trombidiformes	Trombidiidae	MIONB484-11	KM832896
Trombidiformes	Trombidiidae	MIONB485-11	KM840839
Trombidiformes	Trombidiidae	MIONB487-11	KM824988
Trombidiformes	Trombidiidae	MIONB488-11	KM827624
Trombidiformes	Trombidiidae	MIONB491-11	KM835758
Trombidiformes	Trombidiidae	MIONB492-11	KM824676
Trombidiformes	Trombidiidae	MIONB493-11	KM834801
Trombidiformes	Trombidiidae	MIONB494-11	KM840331
Trombidiformes	Trombidiidae	MIONB497-11	KM835870
Trombidiformes	Trombidiidae	MIONB500-11	KM835705
Trombidiformes	Trombidiidae	MIONB501-11	KM825800
Trombidiformes	Trombidiidae	MIONB502-11	KM832417
Trombidiformes	Trombidiidae	MIONB503-11	KM837395
Trombidiformes	Trombidiidae	MIONB504-11	KM828700
Trombidiformes	Trombidiidae	MYMCA025-11	JX838651
Trombidiformes	Trombidiidae	MYMCA318-11	JX837732
Trombidiformes	Trombidiidae	MYMCA319-11	JX836505
Trombidiformes	Trombidiidae	MYMCE215-12	KR069499
Trombidiformes	Trombidiidae	MYMCE216-12	KP979149
Trombidiformes	Trombidiidae	MYMCE217-12	KR069619
Trombidiformes	Trombidiidae	MYMCF788-12	JX838732
Trombidiformes	Trombidiidae	SSBAC3302-12	KM836027
Trombidiformes	Trombidiidae	SSBAC3307-12	KM838481
Trombidiformes	Trombidiidae	SSBAC3308-12	KM838905
Trombidiformes	Trombidiidae	SSBAC3321-12	KM834841
Trombidiformes	Trombidiidae	SSBAC3326-12	KM824318
Trombidiformes	Trombidiidae	SSBAC3332-12	KM839471
Trombidiformes	Trombidiidae	SSBAC3347-12	KM833096
Trombidiformes	Trombidiidae	SSBAC3349-12	KM831737
Trombidiformes	Trombidiidae	SSEIA3005-13	KM830735
Trombidiformes	Trombidiidae	SSEIB4575-13	KM832070
Trombidiformes	Trombidiidae	SSEIB7646-13	KM836108
Trombidiformes	Trombidiidae	SSPAA2260-13	KM840172
Trombidiformes	Trombidiidae	SSPAA7827-13	KM830346
Trombidiformes	Trombidiidae	SSWLA1886-13	KM833226

Trombidiformes	Tydeidae	*Tydeus*	CHACA128-08	JX838600
Trombidiformes	Tydeidae	*Tydeus*	MYMCE507-12	JX838175
Trombidiformes	Tydeidae	*Tydeus*	MYMCE508-12	JX836520
Trombidiformes	Tydeidae	*Tydeus*	MYMCE509-12	JX837704
Trombidiformes	Tydeidae	*Tydeus*	MYMCE522-12	JX838384
Trombidiformes	Tydeidae	*Tydeus*	MYMCE523-12	JX835909
Trombidiformes	Tydeidae	*Tydeus*	MYMCE525-12	JX837720
Trombidiformes	Tydeidae	*Tydeus*	MYMCE526-12	JX833941
Trombidiformes	Tydeidae	*Tydeus*	MYMCE527-12	JX838372
Trombidiformes	Tydeidae	*Tydeus*	MYMCE689-12	JX836703
Trombidiformes	Tydeidae	*Tydeus*	MYMCE718-12	JX835406
Trombidiformes	Tydeidae	*Tydeus*	MYMCE722-12	JX834805
Trombidiformes	Tydeidae	*Tydeus*	MYMCE724-12	JX834656
Trombidiformes	Tydeidae	*Tydeus*	MYMCE725-12	JX834441
Trombidiformes	Tydeidae	*Tydeus*	MYMCE726-12	JX836340
Trombidiformes	Tydeidae	*Tydeus*	MYMCE727-12	JX835722
Trombidiformes	Tydeidae	*Tydeus*	MYMCE728-12	JX837869
Trombidiformes	Tydeidae	*Tydeus*	MYMCE730-12	JX835227
Trombidiformes	Tydeidae	*Tydeus*	MYMCE737-12	JX835986
Trombidiformes	Tydeidae	*Tydeus*	MYMCE738-12	JX834820
Trombidiformes	Tydeidae	*Tydeus*	MYMCE739-12	JX836276
Trombidiformes	Tydeidae	*Tydeus*	MYMCE740-12	JX837674
Trombidiformes	Tydeidae	*Tydeus*	MYMCE745-12	JX837142
Trombidiformes	Tydeidae		CHACB002-10	HQ558325
Trombidiformes	Tydeidae		CHACB003-10	HQ558326
Trombidiformes	Tydeidae		CHACB036-10	HQ558349
Trombidiformes	Tydeidae		CHACB037-10	HQ558350
Trombidiformes	Tydeidae		CHACB1015-10	HM907151
Trombidiformes	Tydeidae		CHACB1169-10	KR069569
Trombidiformes	Tydeidae		CHACB265-10	HQ558506
Trombidiformes	Tydeidae		CHACB430-10	HQ558586
Trombidiformes	Tydeidae		CHACB431-10	HQ558587
Trombidiformes	Tydeidae		CHACB667-10	JX835808
Trombidiformes	Tydeidae		CHACB680-10	HQ558745
Trombidiformes	Tydeidae		CHACB893-10	HM907329
Trombidiformes	Tydeidae		CHACC012-10	HM907413
Trombidiformes	Tydeidae		CHACC013-10	HM907414
Trombidiformes	Tydeidae		CHACC014-10	HM907415
Trombidiformes	Tydeidae		CHACC015-10	HM907416
Trombidiformes	Tydeidae		CHACC016-10	HM907417
Trombidiformes	Tydeidae		CNPPI876-12	KJ445378
Trombidiformes	Tydeidae		CNPPI881-12	KJ444716
Trombidiformes	Tydeidae		CNPPI898-12	KJ444554
Trombidiformes	Tydeidae		CNPPJ1857-12	KJ208341
Trombidiformes	Tydeidae		MIONB101-10	KP979115
Trombidiformes	Tydeidae		MYMCA082-11	JX837749
Trombidiformes	Tydeidae		MYMCA094-11	JX833740
Trombidiformes	Tydeidae		MYMCA1007-11	JX836906
Trombidiformes	Tydeidae		MYMCA1008-11	JX837919
Trombidiformes	Tydeidae		MYMCA1009-11	JX835015
Trombidiformes	Tydeidae		MYMCA1010-11	JX837649
Trombidiformes	Tydeidae		MYMCA1029-11	JX835885
Trombidiformes	Tydeidae		MYMCA1051-11	JX838137

Trombidiformes	Tydeidae	MYMCA1076-11	JX837067
Trombidiformes	Tydeidae	MYMCA1077-11	JX837060
Trombidiformes	Tydeidae	MYMCA1078-11	JX838580
Trombidiformes	Tydeidae	MYMCA1079-11	JX833722
Trombidiformes	Tydeidae	MYMCA1080-11	JX837818
Trombidiformes	Tydeidae	MYMCA1107-11	JX834899
Trombidiformes	Tydeidae	MYMCA1108-11	JX833870
Trombidiformes	Tydeidae	MYMCA1109-11	JX833955
Trombidiformes	Tydeidae	MYMCA1231-11	JX838421
Trombidiformes	Tydeidae	MYMCA1271-11	JX835386
Trombidiformes	Tydeidae	MYMCA1293-11	JX835851
Trombidiformes	Tydeidae	MYMCA1335-11	JX838309
Trombidiformes	Tydeidae	MYMCA1373-11	JX838316
Trombidiformes	Tydeidae	MYMCA1376-11	JX837471
Trombidiformes	Tydeidae	MYMCA1400-11	JX837630
Trombidiformes	Tydeidae	MYMCA1401-11	JX838741
Trombidiformes	Tydeidae	MYMCA176-11	JX836765
Trombidiformes	Tydeidae	MYMCA258-11	JX836669
Trombidiformes	Tydeidae	MYMCA399-11	JX835610
Trombidiformes	Tydeidae	MYMCA400-11	JX838353
Trombidiformes	Tydeidae	MYMCA418-11	JX837412
Trombidiformes	Tydeidae	MYMCA528-11	JX836946
Trombidiformes	Tydeidae	MYMCA529-11	JX834304
Trombidiformes	Tydeidae	MYMCA558-11	JX834437
Trombidiformes	Tydeidae	MYMCA760-11	JX837243
Trombidiformes	Tydeidae	MYMCA806-11	JX835484
Trombidiformes	Tydeidae	MYMCA931-11	JX838614
Trombidiformes	Tydeidae	MYMCA968-11	JX837053
Trombidiformes	Tydeidae	MYMCB124-11	JX838171
Trombidiformes	Tydeidae	MYMCB125-11	JX834903
Trombidiformes	Tydeidae	MYMCB126-11	JX836152
Trombidiformes	Tydeidae	MYMCB133-11	JX834876
Trombidiformes	Tydeidae	MYMCB156-11	JX836663
Trombidiformes	Tydeidae	MYMCB202-11	JX834245
Trombidiformes	Tydeidae	MYMCB203-11	JX833811
Trombidiformes	Tydeidae	MYMCB273-11	JX838593
Trombidiformes	Tydeidae	MYMCB476-11	JX837999
Trombidiformes	Tydeidae	MYMCB609-11	JX835413
Trombidiformes	Tydeidae	MYMCB617-11	JX836105
Trombidiformes	Tydeidae	MYMCB655-11	JX835329
Trombidiformes	Tydeidae	MYMCB720-11	JX837843
Trombidiformes	Tydeidae	MYMCB791-11	JX834829
Trombidiformes	Tydeidae	MYMCB939-11	JX836648
Trombidiformes	Tydeidae	MYMCC026-11	JX838227
Trombidiformes	Tydeidae	MYMCC073-11	JX834072
Trombidiformes	Tydeidae	MYMCC078-11	JX834778
Trombidiformes	Tydeidae	MYMCC100-11	JX834322
Trombidiformes	Tydeidae	MYMCC114-11	JX834691
Trombidiformes	Tydeidae	MYMCC177-11	JX838331
Trombidiformes	Tydeidae	MYMCC289-11	JX838482
Trombidiformes	Tydeidae	MYMCC345-11	JX834424
Trombidiformes	Tydeidae	MYMCC356-11	JX834660
Trombidiformes	Tydeidae	MYMCC369-11	JX835473

Trombidiformes	Tydeidae	MYMCC370-11	JX837792
Trombidiformes	Tydeidae	MYMCC371-11	JX837612
Trombidiformes	Tydeidae	MYMCC372-11	JX835713
Trombidiformes	Tydeidae	MYMCC447-11	JX834048
Trombidiformes	Tydeidae	MYMCC448-11	JX834039
Trombidiformes	Tydeidae	MYMCC449-11	JX833679
Trombidiformes	Tydeidae	MYMCC450-11	JX833693
Trombidiformes	Tydeidae	MYMCC451-11	JX836799
Trombidiformes	Tydeidae	MYMCC452-11	JX836539
Trombidiformes	Tydeidae	MYMCC518-11	JX835298
Trombidiformes	Tydeidae	MYMCC649-11	JX838495
Trombidiformes	Tydeidae	MYMCC651-11	JX833960
Trombidiformes	Tydeidae	MYMCC652-11	JX836412
Trombidiformes	Tydeidae	MYMCC849-11	JX836108
Trombidiformes	Tydeidae	MYMCC892-11	JX833698
Trombidiformes	Tydeidae	MYMCC919-11	JX837537
Trombidiformes	Tydeidae	MYMCC920-11	JX834798
Trombidiformes	Tydeidae	MYMCC922-11	JX837988
Trombidiformes	Tydeidae	MYMCD016-11	JX835976
Trombidiformes	Tydeidae	MYMCD056-11	JX838001
Trombidiformes	Tydeidae	MYMCD057-11	JX838411
Trombidiformes	Tydeidae	MYMCD135-11	JX838488
Trombidiformes	Tydeidae	MYMCD136-11	JX835127
Trombidiformes	Tydeidae	MYMCD137-11	JX834992
Trombidiformes	Tydeidae	MYMCE010-12	JX837859
Trombidiformes	Tydeidae	MYMCE012-12	JX835160
Trombidiformes	Tydeidae	MYMCE013-12	JX834615
Trombidiformes	Tydeidae	MYMCE031-12	JX836713
Trombidiformes	Tydeidae	MYMCE065-12	JX835239
Trombidiformes	Tydeidae	MYMCE067-12	JX837591
Trombidiformes	Tydeidae	MYMCE086-12	JX834822
Trombidiformes	Tydeidae	MYMCE119-12	JX836994
Trombidiformes	Tydeidae	MYMCE189-12	JX835839
Trombidiformes	Tydeidae	MYMCE190-12	JX834427
Trombidiformes	Tydeidae	MYMCE231-12	JX836439
Trombidiformes	Tydeidae	MYMCE291-12	JX833992
Trombidiformes	Tydeidae	MYMCE380-12	JX838072
Trombidiformes	Tydeidae	MYMCE384-12	JX833660
Trombidiformes	Tydeidae	MYMCE401-12	JX834658
Trombidiformes	Tydeidae	MYMCE458-12	JX834170
Trombidiformes	Tydeidae	MYMCE510-12	JX836466
Trombidiformes	Tydeidae	MYMCE542-12	JX834685
Trombidiformes	Tydeidae	MYMCE576-12	JX834728
Trombidiformes	Tydeidae	MYMCE704-12	JX838368
Trombidiformes	Tydeidae	MYMCE753-12	JX834094
Trombidiformes	Tydeidae	MYMCE792-12	JX838381
Trombidiformes	Tydeidae	MYMCE823-12	JX835211
Trombidiformes	Tydeidae	MYMCE824-12	JX835290
Trombidiformes	Tydeidae	MYMCE939-12	JX833991
Trombidiformes	Tydeidae	MYMCE940-12	JX835083
Trombidiformes	Tydeidae	MYMCF055-12	JX833935
Trombidiformes	Tydeidae	MYMCF056-12	JX838371
Trombidiformes	Tydeidae	MYMCF057-12	JX836077

Trombidiformes	Tydeidae	MYMCF058-12	JX833799
Trombidiformes	Tydeidae	MYMCF059-12	JX834367
Trombidiformes	Tydeidae	MYMCF097-12	JX838076
Trombidiformes	Tydeidae	MYMCF099-12	JX836532
Trombidiformes	Tydeidae	MYMCF101-12	JX835600
Trombidiformes	Tydeidae	MYMCF173-12	JX834842
Trombidiformes	Tydeidae	MYMCF174-12	JX833689
Trombidiformes	Tydeidae	MYMCF175-12	JX835852
Trombidiformes	Tydeidae	MYMCF276-12	JX833804
Trombidiformes	Tydeidae	MYMCF312-12	JX837897
Trombidiformes	Tydeidae	MYMCF403-12	JX834475
Trombidiformes	Tydeidae	MYMCF405-12	JX837132
Trombidiformes	Tydeidae	MYMCF457-12	JX835389
Trombidiformes	Tydeidae	MYMCF458-12	JX837879
Trombidiformes	Tydeidae	MYMCF491-12	JX834371
Trombidiformes	Tydeidae	MYMCF511-12	JX836351
Trombidiformes	Tydeidae	MYMCF574-12	JX834974
Trombidiformes	Tydeidae	MYMCF629-12	JX834935
Trombidiformes	Tydeidae	MYMCF653-12	JX838002
Trombidiformes	Tydeidae	MYMCF669-12	JX834699
Trombidiformes	Tydeidae	MYMCF670-12	JX834858
Trombidiformes	Tydeidae	MYMCF671-12	JX833650
Trombidiformes	Tydeidae	MYMCF702-12	JX834742
Trombidiformes	Tydeidae	MYMCF775-12	JX838011
Trombidiformes	Tydeidae	MYMCF776-12	JX837296
Trombidiformes	Tydeidae	MYMCF777-12	JX836359
Trombidiformes	Tydeidae	MYMCG023-12	JX838435
Trombidiformes	Tydeidae	MYMCG030-12	JX836103
Trombidiformes	Tydeidae	MYMCG146-12	JX835430
Trombidiformes	Tydeidae	MYMCG147-12	JX837169
Trombidiformes	Tydeidae	MYMCG172-12	JX835499
Trombidiformes	Tydeidae	MYMCG174-12	JX837814
Trombidiformes	Tydeidae	MYMCG230-12	JX835844
Trombidiformes	Tydeidae	MYMCG309-12	JX835316
Trombidiformes	Tydeidae	MYMCG411-12	JX836475
Trombidiformes	Tydeidae	MYMCG412-12	JX834011
Trombidiformes	Tydeidae	MYMCG413-12	JX834480
Trombidiformes	Tydeidae	MYMCG414-12	JX837822
Trombidiformes	Tydeidae	MYMCG490-12	JX835582
Trombidiformes	Tydeidae	MYMCG491-12	JX834256
Trombidiformes	Tydeidae	MYMCG492-12	JX833817
Trombidiformes	Tydeidae	MYMCG658-12	JX837237
Trombidiformes	Tydeidae	MYTMC067-09	KR070588
Trombidiformes	Tydeidae	MYTMC069-09	HQ966232
Trombidiformes	Tydeidae	MYTMC106-09	JX834715
Trombidiformes	Tydeidae	MYTMC149-09	HQ966240
Trombidiformes	Tydeidae	MYTMC159-09	HQ966243
Trombidiformes	Tydeidae	SSBAD3079-12	KM831533
Trombidiformes	Tydeidae	SSBAD4013-12	KM837778
Trombidiformes	Tydeidae	SSBAD4038-12	KM839607
Trombidiformes	Tydeidae	SSBAD4040-12	KM832695
Trombidiformes	Tydeidae	SSJAC1584-13	KM830456
Trombidiformes	Tydeidae	SSJAC1592-13	KM838931

Trombidiformes	Unionicolidae	*Neumania sp0988B*	CNSLM152-13	KM839290
Trombidiformes	Unionicolidae	*Unionicola*	CNPAC505-13	KM825900
Trombidiformes	Unionicolidae	*Unionicola*	CNPAC506-13	KM828756
Trombidiformes	Unionicolidae	*Unionicola*	CNPAC509-13	KM836781
Trombidiformes	Unionicolidae	*Unionicola*	CNPAC543-13	KM833059
Trombidiformes	Unionicolidae	*Unionicola*	CNPAD835-13	KM826896
Trombidiformes	Unionicolidae	*Unionicola*	CNPPB2359-12	KJ089239
Trombidiformes	Unionicolidae	*Unionicola*	CNPPC1930-12	KJ084240
Trombidiformes	Unionicolidae	*Unionicola*	CNPPI868-12	KJ207628
Trombidiformes	Unionicolidae	*Unionicola*	CNPPJ1856-12	KJ208020
Trombidiformes	Unionicolidae	*Unionicola*	CNSLO1213-13	KP979236
Trombidiformes	Unionicolidae	*Unionicola*	SSPAA2245-13	KM832431
Trombidiformes	Unionicolidae	*Unionicola*	SSPAA2251-13	KM827915
Trombidiformes	Unionicolidae	*Unionicola*	SSPAA2254-13	KM827049
Trombidiformes	Unionicolidae	*Unionicola*	SSPAA2267-13	KM837946
Trombidiformes	Unionicolidae	*Unionicola*	SSPAA2271-13	KM828481
Trombidiformes	Unionicolidae	*Unionicola*	SSPAA2293-13	KM840319
Trombidiformes	Unionicolidae	*Unionicola*	SSPAA2299-13	KM834261
Trombidiformes	Unionicolidae	*Unionicola*	SSPAA2304-13	KM829649
Trombidiformes	Unionicolidae	*Unionicola crassipes*	GBCH3249-09	FJ524382
Trombidiformes	Unionicolidae	*Unionicola foili*	GBCH3177-08	NC_011036
Trombidiformes	Unionicolidae	*Unionicola foili*	GBCH3178-08	EU856396
Trombidiformes	Unionicolidae		CNPPB2361-12	KJ089749
Trombidiformes	Unionicolidae		CNROC084-13	KR069172
Uropygi	Thelyphonidae	*Mastigoproctus giganteus*	GBCH2384-08	NC_010430
Uropygi	Thelyphonidae	*Mastigoproctus giganteus*	GBCH2402-08	EU520643
Uropygi	Thelyphonidae		GBCH7778-13	JN018148
Uropygi	Thelyphonidae		GBCH7779-13	JN018147
