# Supplementary material for: Patterns of Protein Evolution in Cytochrome c Oxidase 1 (COI) from the Class Arachnida
Source: PLoS One. 2015 Aug 26;10(8):e0135053. doi: 10.1371/journal.pone.0135053 (PMC4550450; doi:10.1371/journal.pone.0135053)
Supplement: S2 Table — (PDF) [file pone.0135053.s005.pdf]

S2 Table. List of sequences from each order used for family level analyses and the outgroup (n=4178), with associated taxonomic assignments, Process IDs, GenBank Accession numbers, from the dataset MYBCRED (dx.doi.org/10.5883/DS-MYBCRED) on BOLD, the Barcode of Life Datasystems.

Family	Genus/Speceies	Process ID	Accession
**Amblypygi**			
Charinidae		GACAM014-13	JN018110
Charontidae	*Stygophrynus*	GACAM013-13	JN018111
Charontidae	*Stygophrynus*	GACAM012-13	JN018112
Charontidae	*Stygophrynus*	GACAM011-13	JN018113
Phrynichidae	*Damon diadema*	GBCH3455-09	NC_011293
Phrynichidae	*Phrynichus orientalis*	GACAM001-13	JN018195
Phrynidae	*Heterophrynus longicornis*	GACAM005-13	JN018119
Phrynidae	*Phrynus sp. 1 SEM-2008*	GBCH3208-08	NC_010775
**Araneae**			
Actinopodidae	*Missulena granulosa*	GBCH8414-13	KC708094
Actinopodidae	*Missulena pruinosa*	GBCH8412-13	KC708096
Actinopodidae	*Missulena*	GBCH8434-13	KC708074
Actinopodidae	*Missulena*	GBCH8433-13	KC708075
Actinopodidae	*Missulena*	GBCH8432-13	KC708076
Actinopodidae	*Missulena*	GBCH8431-13	KC708077
Actinopodidae	*Missulena*	GBCH8430-13	KC708078
Actinopodidae	*Missulena*	GBCH8428-13	KC708080
Actinopodidae	*Missulena*	GBCH8427-13	KC708081
Actinopodidae	*Missulena*	GBCH8425-13	KC708083
Actinopodidae	*Missulena*	GBCH8424-13	KC708084
Actinopodidae	*Missulena*	GBCH8422-13	KC708086
Actinopodidae	*Missulena*	GBCH8421-13	KC708087
Actinopodidae	*Missulena*	GBCH8420-13	KC708088
Actinopodidae	*Missulena*	GBCH8419-13	KC708089
Actinopodidae	*Missulena*	GBCH8416-13	KC708092
Actinopodidae	*Missulena*	GBCH8415-13	KC708093
Actinopodidae	*Missulena*	GBCH8413-13	KC708095
Agelenidae	*Agelena koreana*	GBCH11069-13	JN817201
Agelenidae	*Agelena labyrinthica*	GBCH11071-13	JN817199
Agelenidae	*Agelenopsis aleenae*	GBCH1049-06	AY770790
Agelenidae	*Agelenopsis aperta*	GBCH1044-06	AY770785
Agelenidae	*Agelenopsis aperta*	GBCH4047-09	FJ607552
Agelenidae	*Agelenopsis emertoni*	ARONT059-09	GU682803
Agelenidae	*Agelenopsis longistyla*	GBCH1073-06	AY770814
Agelenidae	*Agelenopsis longistyla*	GBCH1087-06	AY770828
Agelenidae	*Agelenopsis n. sp. NAA-2005*	GBCH1076-06	AY770817
Agelenidae	*Agelenopsis naevia*	GBCH1060-06	AY770801

Agelenidae	*Agelenopsis oklahoma*	GBCH1063-06	AY770804
Agelenidae	*Agelenopsis utahana*	RBCH014-04	DQ127510
Agelenidae	*Allagelena difficilis*	GBCH11070-13	JN817200
Agelenidae	*Barronopsis sp. NAA-2005*	GBCH1069-06	AY770810
Agelenidae	*Barronopsis texana*	GBCH1074-06	AY770815
Agelenidae	*Coras juvenilis*	ARONT517-10	HQ924407
Agelenidae	*Coras lamellosus*	ERSPI247-09	HM376097
Agelenidae	*Coras montanus*	ARONT425-09	GU682929
Agelenidae	*Draconarius coreanus*	GBCH11061-13	JN817209
Agelenidae	*Draconarius kayasanensis*	GBCH11060-13	JN817210
Agelenidae	*Eratigena agrestis*	ARONT348-09	GU682856
Agelenidae	*Eratigena atrica*	SPRMA491-10	JF887027
Agelenidae	*Iwogumoa songminjae*	GBCH11059-13	JN817211
Agelenidae	*Novalena intermedia*	GBCH3530-09	EU979472
Agelenidae	*Novalena sp. NAA-2005*	GBCH1057-06	AY770798
Agelenidae	*Pireneitega spinivulva*	GBCH11057-13	JN817213
Agelenidae	*Tegecoelotes secundus*	GBCH11058-13	JN817212
Agelenidae	*Tegenaria domestica*	ARONT322-09	GU682888
Agelenidae	*Wadotes calcaratus*	ARONT363-09	GU682892
Agelenidae	*Wadotes hybridus*	RBINA4171-13	KP654006
Agelenidae	*Wadotes*	TDWGB828-10	HQ979253
Amaurobiidae	*Amaurobius borealis*	ARONT544-10	HQ924431
Amaurobiidae	*Amaurobius ferox*	ARONT332-09	GU682864
Amaurobiidae	*Amaurobius sp. 1RB*	SPRMA722-10	JF887147
Amaurobiidae	*Callobius bennetti*	ARONT357-09	GU682866
Amaurobiidae	*Callobius enus*	SPRMA861-12	KP652721
Amaurobiidae	*Callobius koreanus*	GBCH11062-13	JN817208
Amaurobiidae	*Callobius nomeus*	SPRMA528-10	JF887043
Amaurobiidae	*Callobius pictus*	SPRMA960-12	KP655754
Amaurobiidae	*Callobius severus*	SPRMA525-10	JF887040
Amaurobiidae	*Callobius sp. TAB-2009*	GBCH4040-09	FJ607559
Amaurobiidae	*Cybaeopsis euopla*	SPICH998-09	GU684503
Amaurobiidae	*Cybaeopsis wabritaska*	SPRMA216-10	HQ580829
Amaurobiidae	*Zanomys aquilonia*	SPRMA987-12	KP646830
Anapidae	*Acrobleps*	GBCH8372-13	GU456874
Anapidae	*Anapisona kethleyi*	GBCH8371-13	GU456875
Anapidae	*Conculus*	GBCH8373-13	GU456873
Antrodiaetidae	*Antrodiaetus pacificus*	SPRMA004-10	HQ977116
Anyphaenidae	*Anyphaena aperta*	SPRMA587-10	JF887077
Anyphaenidae	*Anyphaena celer*	ARONT409-09	HM434065
Anyphaenidae	*Anyphaena pacifica*	SPRMA708-10	JF887139
Anyphaenidae	*Anyphaena pectorosa*	ARONT684-10	HQ924543
Anyphaenidae	*Anyphaena*	TDWGB902-10	HQ979325
Anyphaenidae	*Hibana gracilis*	PHSEP1958-11	KP648612

Anyphaenidae	*Wulfila saltabundus*	ARONT408-09	GU682895
Araneidae	*Acanthepeira stellata*	ERSPI299-09	GU682531
Araneidae	*Aculepeira carbonarioides*	GBADC022-10	HQ956685
Araneidae	*Aculepeira packardi*	SSBAF7746-13	KM832933
Araneidae	*Araneus acusisetus*	GBCH11126-13	JN817144
Araneidae	*Araneus angulatus*	GBCH11132-13	JN817138
Araneidae	*Araneus corticarius*	SPIRU1088-11	KF367836
Araneidae	*Araneus diadematus*	GBCH4046-09	FJ607553
Araneidae	*Araneus ejusmodi*	GBCH11127-13	JN817143
Araneidae	*Araneus gemmoides*	SPRMA015-10	HQ977124
Araneidae	*Araneus groenlandicola*	SWSWE077-09	GU681023
Araneidae	*Araneus ishisawai*	GBCH11130-13	JN817140
Araneidae	*Araneus iviei*	SSPAB5306-13	KM832263
Araneidae	*Araneus marmoreus*	GBCH3915-09	EU003278
Araneidae	*Araneus marmoreus*	ARONT312-09	GU682584
Araneidae	*Araneus nordmanni*	SPICH1163-09	GU684587
Araneidae	*Araneus pratensis*	TDWGB737-10	HQ979182
Araneidae	*Araneus quadratus*	GACAC390-12	FR775772
Araneidae	*Araneus saevus*	ARONT021-09	GU682760
Araneidae	*Araneus sp. 1GAB*	SSBAE6064-13	KM839354
Araneidae	*Araneus sp. 2GAB*	ARONT313-09	HM434063
Araneidae	*Araneus stella*	GBCH11128-13	JN817142
Araneidae	*Araneus thaddeus*	ARONT153-09	HM434053
Araneidae	*Araneus trifolium*	ARONT309-09	GU682571
Araneidae	*Araneus variegatus*	GBCH11131-13	JN817139
Araneidae	*Araniella displicata*	ARONT116-09	GU682652
Araneidae	*Araniella displicata*	SSBAC2918-12	KM834835
Araneidae	*Araniella displicata*	SSWLC4118-13	KM836477
Araneidae	*Araniella yaginumai*	GBCH11107-13	JN817163
Araneidae	*Argiope amoena*	GBCH11113-13	JN817157
Araneidae	*Argiope argentata*	GBCH4045-09	FJ607554
Araneidae	*Argiope aurantia*	GBCH3232-09	FJ525332
Araneidae	*Argiope bruennichi*	GBCH9087-13	KC195255
Araneidae	*Argiope dang*	GBCH11500-13	AB753452
Araneidae	*Argiope minuta*	GBCH11111-13	JN817159
Araneidae	*Argiope submaronica*	GBCH3914-09	EU003279
Araneidae	*Argiope trifasciata*	GBCH3248-09	FJ525316
Araneidae	*Arkys cornutus*	GBCH4043-09	FJ607556
Araneidae	*Backobourkia brouni*	GBCH5079-10	FJ873122
Araneidae	*Backobourkia collina*	GBCH5078-10	FJ873123
Araneidae	*Backobourkia heroine*	GBCH5075-10	FJ873126
Araneidae	*Chorizopes nipponicus*	GBCH11105-13	JN817165
Araneidae	*Cyclosa alba*	GBCH4112-09	AB453394
Araneidae	*Cyclosa argenteoalba*	GBCH11098-13	JN817172

Araneidae	*Cyclosa conica*	ERSPI322-09	GU682513
Araneidae	*Cyclosa japonica*	GBCH11099-13	JN817171
Araneidae	*Cyclosa laticauda*	GBCH11101-13	JN817169
Araneidae	*Cyclosa mulmeinensis*	GBCH4117-09	AB453766
Araneidae	*Cyclosa mulmeinensis*	GBCH4120-09	AB453769
Araneidae	*Cyclosa octotuberculata*	GBCH11103-13	JN817167
Araneidae	*Cyrtophora moluccensis*	GBCH4039-09	FJ607560
Araneidae	*Eriophora astridae*	GBCH11094-13	JN817176
Araneidae	*Eriophora pustulosa*	GBCH5074-10	FJ873127
Araneidae	*Eriophora sachalinensis*	GBCH11095-13	JN817175
Araneidae	*Eustala anastera*	ERSPI320-09	GU682512
Araneidae	*Eustala cepina*	ERSPI318-09	HM376104
Araneidae	*Eustala emertoni*	PHMTU094-10	KP648805
Araneidae	*Eustala sp. 1GAB*	TDWGB839-10	HQ979264
Araneidae	*Eustala sp. TAB-2009*	GBCH3244-09	FJ525320
Araneidae	*Gasteracantha cancriformis*	GBCH3243-09	FJ525321
Araneidae	*Gasteracantha kuhli*	GBCH11106-13	JN817164
Araneidae	*Gasteracantha sp.*	RBCH176-04	DQ127354
Araneidae	*Gea heptagon*	ARONT414-09	GU682951
Araneidae	*Hypsosinga pygmaea*	ARONT503-10	HQ924397
Araneidae	*Hypsosinga pygmaea*	ARONT505-10	HQ924399
Araneidae	*Hypsosinga pygmaea*	GBADC048-10	HQ956709
Araneidae	*Larinioides cornutus*	GBCH3242-09	FJ525322
Araneidae	*Larinioides cornutus*	GBCH11125-13	JN817145
Araneidae	*Larinioides patagiatus*	ARONT369-09	GU682749
Araneidae	*Larinioides sclopetarius*	RBCH204-04	DQ127334
Araneidae	*Larinioides sclopetarius*	GBCH11124-13	JN817146
Araneidae	*Mangora gibberosa*	ARONT811-10	HQ924656
Araneidae	*Mangora maculata*	GBCH3900-09	EU003293
Araneidae	*Mangora placida*	ARONT268-09	GU682728
Araneidae	*Mangora placida*	ARONT276-09	HM434062
Araneidae	*Mangora placida*	ARONT716-10	HQ924572
Araneidae	*Mangora placida*	RBINA1845-13	KP647542
Araneidae	*Mecynogea lemniscata*	GBCH3899-09	EU003294
Araneidae	*Metepeira palustris*	SSJAB3596-13	KM836852
Araneidae	*Metepeira sp. 1GAB*	SSJAB3595-13	KM835110
Araneidae	*Metepeira sp. TAB-2009*	GBCH3239-09	FJ525325
Araneidae	*Neoscona arabesca*	ARONT339-09	GU682737
Araneidae	*Neoscona crucifera*	GBCH3237-09	FJ525327
Araneidae	*Neoscona multiplicans*	GBCH11120-13	JN817150
Araneidae	*Neoscona nautica*	GBCH11123-13	JN817147
Araneidae	*Neoscona pseudonautica*	GBCH11122-13	JN817148
Araneidae	*Neoscona punctigera*	GBCH11119-13	JN817151
Araneidae	*Neoscona scylloides*	GBCH11117-13	JN817153

Araneidae	*Neoscona subpullata*	GBCH11114-13	JN817156
Araneidae	*Neoscona theisi*	GBCH11115-13	JN817155
Araneidae	*Neoscona vigilans*	GBCH7175-13	HQ441945
Araneidae	*Paraplectana sakaguchii*	GBCH7256-13	AB627002
Araneidae	*Paraplectana tsushimensis*	GBCH7253-13	AB627005
Araneidae	*Parazygiella dispar*	SPRMA201-10	HQ580815
Araneidae	*Yaginumia sia*	GBCH11096-13	JN817174
Araneidae	*Zygiella atrica*	GBCH4010-09	FJ607589
Araneidae	*Zygiella nearctica*	SPICH1126-09	GU684584
Araneidae	*Zygiella x-notata*	SPRMA687-10	JF887124
Archaeidae	*Afrarchaea woodae*	GBCH11408-13	JX240249
Archaeidae	*Afrarchaea*	GBCH11409-13	JX240248
Archaeidae	*Austrarchaea nodosa*	GBCH11407-13	JX240250
Archaeidae	*Austrarchaea*	GBCH11406-13	JX240251
Archaeidae	*Eriauchenius bourgini*	GBCH11410-13	JX240247
Archaeidae	*Eriauchenius griswoldi*	GBCH2828-08	DQ914574
Archaeidae	*Eriauchenius griswoldi*	GBCH2827-08	DQ914575
Archaeidae	*Eriauchenius jeanneli*	GBCH11413-13	JX240244
Archaeidae	*Eriauchenius lavatenda*	GBCH11414-13	JX240243
Archaeidae	*Eriauchenius legendrei*	GBCH11412-13	JX240245
Archaeidae	*Eriauchenius voronakely*	GBCH2838-08	DQ914564
Archaeidae	*Eriauchenius voronakely*	GBCH2837-08	DQ914565
Archaeidae	*Eriauchenius workmani*	GBCH11411-13	JX240246
Archaeidae	*Zephyrarchaea mainae*	GBCH11405-13	JX240252
Caponiidae	*Caponia*	GBCH7209-13	JN689215
Clubionidae	*Clubiona abboti*	ARONT169-09	GU682612
Clubionidae	*Clubiona blesti*	GACAC1052-12	JN377952
Clubionidae	*Clubiona blesti*	GACAC1054-12	JN377954
Clubionidae	*Clubiona blesti*	GACAC1058-12	JN377958
Clubionidae	*Clubiona bryantae*	ARONT417-09	GU682907
Clubionidae	*Clubiona cada*	GACAC1060-12	JN377960
Clubionidae	*Clubiona cambridgei*	GACAC1067-12	JN377967
Clubionidae	*Clubiona canadensis*	SPRMA831-12	KP652419
Clubionidae	*Clubiona clima*	GACAC1066-12	JN377966
Clubionidae	*Clubiona consensa*	GACAC1070-12	JN377970
Clubionidae	*Clubiona convoluta*	GACAC1072-12	JN377972
Clubionidae	*Clubiona coreana*	GBCH11050-13	JN817220
Clubionidae	*Clubiona elaphines*	GBCH11790-13	JQ347510
Clubionidae	*Clubiona furcata*	GBADC025-10	HQ956688
Clubionidae	*Clubiona huttoni*	GACAC1064-12	JN377964
Clubionidae	*Clubiona japonicola*	GBCH11049-13	JN817221
Clubionidae	*Clubiona johnsoni*	ARONT698-10	HQ924556
Clubionidae	*Clubiona kastoni*	CNRMB231-12	KM837727
Clubionidae	*Clubiona kulczynskii*	CNJAI008-12	KM827784

Clubionidae	*Clubiona maritima*	SMTPB14447-13	KP653578
Clubionidae	*Clubiona moesta*	PHSEP1964-11	KP655215
Clubionidae	*Clubiona mutata*	SSWLB5776-13	KM837651
Clubionidae	*Clubiona norvegica*	KKCHE962-09	GU683661
Clubionidae	*Clubiona obesa*	ARONT393-09	GU682902
Clubionidae	*Clubiona opeongo*	SSBAC2910-12	KM831032
Clubionidae	*Clubiona pallidula*	ARONT372-09	GU682869
Clubionidae	*Clubiona peculiaris*	GBCH11788-13	JQ347512
Clubionidae	*Clubiona pikei*	GMNCG466-12	KJ085672
Clubionidae	*Clubiona praematura*	KKCHE977-09	GU683674
Clubionidae	*Clubiona pygmaea*	CNSLG294-12	KM839234
Clubionidae	*Clubiona riparia*	ARONT120-09	GU682610
Clubionidae	*Clubiona riparia*	ARONT119-09	HM434051
Clubionidae	*Clubiona saltitans*	SPRMA583-10	JF887073
Clubionidae	*Clubiona trivialis*	GBADC017-10	HQ956680
Clubionidae	*Elaver excepta*	TDWGB733-10	HQ979178
Clubionidae		CNBAN622-13	KP979205
Clubionidae		CNBAN019-13	KP979165
Clubionidae		CNBAN020-13	KP979267
Corinnidae	*Castianeira sp. 1GAB*	SPRMA703-10	JF887134
Corinnidae	*Castianeira variata*	CNPCB045-12	KM839949
Corinnidae	*Castianeira walsinghami*	SPRMA140-10	HQ580769
Ctenidae	*Anahita fauna*	GBCH11054-13	JN817216
Ctenizidae	*Conothele*	GBCH8418-13	KC708090
Ctenizidae	*Conothele*	GBCH8417-13	KC708091
Cybaeidae	*Allocybaeina littlewalteri*	SPRMA720-10	JF887145
Cybaeidae	*Argyroneta aquatica*	GBCH11068-13	JN817202
Cybaeidae	*Cybaeota nana*	GBCH5431-10	FJ263787
Cybaeidae	*Cybaeota shastae*	SPRMA507-10	JF887036
Cybaeidae	*Cybaeus angustiarum*	GBCH5428-10	FJ263790
Cybaeidae	*Cybaeus chauliodous*	GBCH5419-10	FJ263799
Cybaeidae	*Cybaeus gidneyi*	GBCH5418-10	FJ263800
Cybaeidae	*Cybaeus giganteus*	GBCH5430-10	FJ263788
Cybaeidae	*Cybaeus hesper*	GBCH5422-10	FJ263796
Cybaeidae	*Cybaeus morosus*	GBCH5426-10	FJ263792
Cybaeidae	*Cybaeus morosus*	SPRMA939-12	KP646799
Cybaeidae	*Cybaeus mosanensis*	GBCH11067-13	JN817203
Cybaeidae	*Cybaeus patritus*	GBCH5429-10	FJ263789
Cybaeidae	*Cybaeus penedentatus*	GBCH5421-10	FJ263797
Cybaeidae	*Cybaeus reticulatus*	GBCH5425-10	FJ263793
Cybaeidae	*Cybaeus reticulatus*	SPRMA682-10	JF887120
Cybaeidae	*Cybaeus sanbruno*	GBCH5423-10	FJ263795
Cybaeidae	*Cybaeus signifer*	GBCH5424-10	FJ263794
Cybaeidae	*Cybaeus sinuosus*	SPRMA306-10	HQ977174

Cybaeidae	*Cybaeus somesbar*	GBCH5420-10	FJ263798
Cybaeidae	*Cybaeus sp. 1GAB*	SPRMA719-10	JF887144
Cybaeidae	*Cybaeus sp. 2GAB*	SPRMA727-10	JF887150
Cybaeidae	*Cybaeus thermydrinos*	SPRMA724-10	KP979257
Cybaeidae	*Dolichocybaeus*	GBCH11066-13	JN817204
Desidae	*Badumna insignis*	CNBAN015-13	KP979154
Desidae	*Badumna longinqua*	GBCH4041-09	FJ607558
Desidae	*Paratheuma shirahamaensis*	GBCH11065-13	JN817205
Dictynidae	*Arctella lapponica*	KKCHE1044-09	GU683728
Dictynidae	*Argenna obesa*	SMTPB21299-13	KP646247
Dictynidae	*Brommella monticola*	SPRMA981-12	KP656238
Dictynidae	*Cicurina arcuata*	ERSPI154-09	KP653066
Dictynidae	*Cicurina brevis*	ARONT455-09	GU682906
Dictynidae	*Cicurina intermedia*	SPRMA153-10	HQ580778
Dictynidae	*Cicurina itasca*	ERSPI144-09	KP653448
Dictynidae	*Cicurina japonica*	GBCH11063-13	JN817207
Dictynidae	*Cicurina pallida*	ERSPI249-09	GU682563
Dictynidae	*Cicurina placida*	ARONT424-09	GU682908
Dictynidae	*Cicurina robusta*	ERSPI235-09	KP652201
Dictynidae	*Cicurina sp. 1GAB*	ARONT699-10	HQ924557
Dictynidae	*Cicurina tersa*	SPRMA973-12	KP649008
Dictynidae	*Cicurina toreriaria*	SPRMA760-10	JF887174
Dictynidae	*Dictyna bellans*	ARONT482-10	HQ924381
Dictynidae	*Dictyna brevitarsa*	GBADC009-10	HQ956673
Dictynidae	*Dictyna brevitarsa*	PHMTU053-10	JN307930
Dictynidae	*Dictyna foliacea*	ARONT034-09	GU682808
Dictynidae	*Dictyna major*	SPICH1048-09	GU684716
Dictynidae	*Dictyna major*	SPRMA906-12	KP649790
Dictynidae	*Dictyna personata*	SPRMA895-12	KP656097
Dictynidae	*Dictyna sp. 1GAB*	SSWLB473-13	KM833356
Dictynidae	*Dictyna volucripes*	ERSPI390-09	GU682457
Dictynidae	*Dictyna volucripes*	ARONT638-10	HQ924503
Dictynidae	*Emblyna annulipes*	ARONT251-09	GU682683
Dictynidae	*Emblyna annulipes*	ARONT252-09	GU682686
Dictynidae	*Emblyna borealis*	SSWLB470-13	KM836124
Dictynidae	*Emblyna borealis*	SPRMA875-12	KP655409
Dictynidae	*Emblyna hentzi*	SMTPB3691-13	KP655025
Dictynidae	*Emblyna manitoba*	ARONT506-10	HQ924400
Dictynidae	*Emblyna maxima*	CNRMD2866-13	KM829494
Dictynidae	*Emblyna maxima*	SSPAB9957-13	KM834908
Dictynidae	*Emblyna peragrata*	SPICH1043-09	GU684713
Dictynidae	*Emblyna peragrata*	SSBAA5288-12	KM834837
Dictynidae	*Emblyna peragrata*	SPRMA866-12	KP649576
Dictynidae	*Emblyna phylax*	CNJAB108-12	KM839035

Dictynidae	*Emblyna phylax*	JSARA046-11	KP656852
Dictynidae	*Emblyna sp. 1GAB*	SSJAB4170-13	KM837244
Dictynidae	*Emblyna sublata*	ARONT679-10	HQ924538
Dictynidae	*Emblyna sublata*	CNPPC2006-12	KJ085208
Dictynidae	*Hackmania prominula*	SPICH097-09	HM432624
Dictynidae	*Hackmania saphes*	SSBAA5276-12	KM830608
Diguetidae	*Diguetia sp. BB-2004*	GBCH0805-06	AY560795
Dipluridae	*Euagrus chisoseus*	GBCH4035-09	FJ607564
Dipluridae	*Phyxioschema suthepium*	GBCH10811-13	NC_020322
Dysderidae	*Cryptoparachtes*	GBCH7245-13	JN689136
Dysderidae	*Dysdera alegranzaensis*	GBCH3184-08	EU139631
Dysderidae	*Dysdera crocata*	ARONT178-09	HM434054
Dysderidae	*Dysdera crocata*	GBCH7752-13	JN018196
Dysderidae	*Dysdera crocata*	GBCH12418-13	JX017359
Dysderidae	*Dysdera erythrina*	GBCH4976-10	GQ285630
Dysderidae	*Dysdera erythrina*	GBCH4964-10	GQ285642
Dysderidae	*Dysdera erythrina*	GBCH4963-10	GQ285643
Dysderidae	*Dysdera erythrina lantosquensis*	GBCH4975-10	GQ285631
Dysderidae	*Dysdera gibbifera*	GBCH3675-09	EU068034
Dysderidae	*Dysdera hernandezi*	GBCH3672-09	EU068037
Dysderidae	*Dysdera levipes*	GBCH3668-09	EU068041
Dysderidae	*Dysdera lusitanica*	GBCH4977-10	GQ285629
Dysderidae	*Dysdera madai*	GBCH3666-09	EU068043
Dysderidae	*Dysdera nesiotes*	GBCH3192-08	EU139621
Dysderidae	*Dysdera ratonensis*	GBCH3657-09	EU068053
Dysderidae	*Dysdera sanborondon*	GBCH3187-08	EU139626
Dysderidae	*Dysdera shardana*	GBCH4968-10	GQ285638
Dysderidae	*Dysdera shardana*	GBCH4967-10	GQ285639
Dysderidae	*Dysdera spinidorsa*	GBCH3185-08	EU139628
Dysderidae	*Dysdera valentina*	GBCH4974-10	GQ285632
Dysderidae	*Holissus unciger*	GBCH7238-13	JN689149
Dysderidae	*Parachtes andreinii*	GBCH7212-13	JN689175
Dysderidae	*Parachtes deminutus*	GBCH7214-13	JN689173
Dysderidae	*Parachtes deminutus*	GBCH7213-13	JN689174
Dysderidae	*Parachtes ignavus*	GBCH7236-13	JN689151
Dysderidae	*Parachtes limbarae*	GBCH7234-13	JN689153
Dysderidae	*Parachtes limbarae*	GBCH7215-13	JN689172
Dysderidae	*Parachtes siculus*	GBCH7216-13	JN689171
Dysderidae	*Parachtes teruelis*	GBCH7222-13	JN689165
Dysderidae	*Parachtes teruelis*	GBCH7217-13	JN689170
Dysderidae	*Parachtes vernae*	GBCH7211-13	JN689176
Dysderidae	*Parachtes*	GBCH7226-13	JN689161
Dysderidae	*Parachtes*	GBCH7210-13	JN689177
Eresidae	*Stegodyphus*	GBCH11423-13	JX240234

Eutichuridae	*Cheiracanthium inclusum*	SPRMA686-10	JF887123
Eutichuridae	*Cheiracanthium japonicum*	GBCH11052-13	JN817218
Eutichuridae	*Cheiracanthium mildei*	ARONT139-09	GU682647
Eutichuridae	*Cheiracanthium uncinatum*	GBCH11051-13	JN817219
Eutichuridae	*Strotarchus piscatorius*	CNSLH310-12	KM824524
Filistatidae	*Kukulcania hibernalis*	GBCH11424-13	JX240233
Gnaphosidae	*Callilepis pluto*	CNPPE2326-12	KJ167562
Gnaphosidae	*Cesonia bilineata*	SPRMA601-10	JF887082
Gnaphosidae	*Drassodes lapidosus*	GBCH0808-06	AY560798
Gnaphosidae	*Drassodes mirus*	SPISH001-09	GU683947
Gnaphosidae	*Drassodes neglectus*	SPICH1162-09	GU684591
Gnaphosidae	*Drassodes serratidens*	GBCH11045-13	JN817225
Gnaphosidae	*Drassyllus biglobus*	GBMIN12198-13	JN817226
Gnaphosidae	*Drassyllus depressus*	ARONT743-10	HQ924596
Gnaphosidae	*Drassyllus dromeus*	SPRMA605-10	JF887083
Gnaphosidae	*Drassyllus insularis*	SPRMA938-12	KP649465
Gnaphosidae	*Drassyllus niger*	ERSPI263-09	HM376100
Gnaphosidae	*Drassyllus*	TDWGB933-10	HQ979353
Gnaphosidae	*Gnaphosa borea*	JDTGS009-09	GU679903
Gnaphosidae	*Gnaphosa brumalis*	SPICH213-09	GU683954
Gnaphosidae	*Gnaphosa kompirensis*	GBCH11044-13	JN817227
Gnaphosidae	*Gnaphosa microps*	KKCHE755-07	KF368100
Gnaphosidae	*Gnaphosa muscorum*	SPICH047-09	GU683906
Gnaphosidae	*Gnaphosa orites*	SPIRU1298-11	KF368103
Gnaphosidae	*Gnaphosa parvula*	ARONT410-09	GU682914
Gnaphosidae	*Gnaphosa snohomish*	SPRMA615-10	JF887090
Gnaphosidae	*Gnaphosa*	TDWGB940-10	HQ979360
Gnaphosidae	*Haplodrassus bicornis*	SPRMA614-10	JF887089
Gnaphosidae	*Haplodrassus eunis*	SSBAA5243-12	KM824773
Gnaphosidae	*Haplodrassus hiemalis*	SPISH036-09	GU683958
Gnaphosidae	*Haplodrassus signifer*	ERSPI254-09	GU682568
Gnaphosidae	*Haplodrassus signifer*	SPICH145-09	GU683772
Gnaphosidae	*Herpyllus ecclesiasticus*	ARONT345-09	GU682872
Gnaphosidae	*Micaria aenea*	SPICH157-09	GU683781
Gnaphosidae	*Micaria alpina*	SPICH990-09	GU684496
Gnaphosidae	*Micaria constricta*	SPRMA194-10	HQ580808
Gnaphosidae	*Micaria dives*	GBCH11043-13	JN817228
Gnaphosidae	*Micaria elizabethae*	ERSPI341-09	HM376105
Gnaphosidae	*Micaria foxi*	SPRMA826-12	KP650704
Gnaphosidae	*Micaria pulicaria*	SPICH215-09	GU683944
Gnaphosidae	*Micaria pulicaria*	SSWLD2595-13	KM827168
Gnaphosidae	*Nodocion mateonus*	CNGRF421-12	KM836659
Gnaphosidae	*Orodrassus canadensis*	CNJAG1007-12	KM828187
Gnaphosidae	*Orodrassus coloradensis*	SPRMA124-10	HQ580758

Gnaphosidae	*Scotophaeus blackwalli*	SPRMA742-10	JF887159
Gnaphosidae	*Sergiolus columbianus*	SPRMA628-10	JF887094
Gnaphosidae	*Sergiolus montanus*	SSJAB3538-13	KM831862
Gnaphosidae	*Sergiolus montanus*	CNWLF011-12	KM834495
Gnaphosidae	*Sergiolus montanus*	SPRMA809-12	KP656031
Gnaphosidae	*Sosticus insularis*	SPRMA629-10	JF887095
Gnaphosidae	*Trachyzelotes jaxartensis*	GBCH11041-13	JN817230
Gnaphosidae	*Zelotes fratris*	ARONT802-10	HQ924647
Gnaphosidae	*Zelotes puritanus*	SPRMA638-10	JF887098
Gnaphosidae	*Zelotes sula*	KKCHE082-06	KF369090
Gnaphosidae	*Zelotes tuobus*	SPRMA160-10	HQ580783
Hahniidae	*Antistea brunnea*	SSPAA6495-13	KM839029
Hahniidae	*Calymmaria nana*	SPRMA276-10	HQ580876
Hahniidae	*Cryphoeca exlineae*	SPRMA184-10	HQ580801
Hahniidae	*Cryphoeca exlineae*	SSWLC3110-13	KM838516
Hahniidae	*Cryphoeca montana*	ARONT546-10	HQ924433
Hahniidae	*Dirksia cinctipes*	SPRMA235-10	HQ580842
Hahniidae	*Ethobuella tuonops*	SPRMA484-10	JF887021
Hahniidae	*Hahnia cinerea*	SPISH024-09	GU683968
Hahniidae	*Hahnia cinerea*	SSJAB2025-13	KM824967
Hahniidae	*Hahnia glacialis*	SSJAD2547-13	KM829983
Hahniidae	*Hahnia sp. 1GAB*	TDWGB777-10	HQ979220
Hahniidae	*Neoantistea agilis*	SPRMA145-10	HQ580773
Hahniidae	*Neoantistea gosiuta*	SPRMA976-12	KP654212
Hahniidae	*Neoantistea magna*	ERSPI372-09	GU682475
Hahniidae	*Neoantistea magna*	SPRMA204-10	HQ580818
Hahniidae	*Neoantistea quelpartensis*	GBCH11064-13	JN817206
Huttoniidae	*Huttonia*	GBCH11420-13	JX240237
Hypochilidae	*Hypochilus thorelli*	GBCH2390-08	EU523753
Leptonetidae	*Leptoneta coreana*	GBCH11202-13	JN817068
Leptonetidae	*Leptoneta hwanseonensis*	GBCH11201-13	JN817069
Linyphiidae	*Acanthoneta aggressa*	SSBAE5755-13	KM826711
Linyphiidae	*Agyneta allosubtilis*	SPICH004-09	GU683853
Linyphiidae	*Agyneta amersaxatilis*	SPICH1095-09	HM416913
Linyphiidae	*Agyneta bucklei*	SPRMA1039-12	KP656292
Linyphiidae	*Agyneta danielbelangeri*	SPRMA1144-12	KP653192
Linyphiidae	*Agyneta decorata*	SPRMA1051-12	KP652844
Linyphiidae	*Agyneta fabra*	ARONT689-10	HQ924548
Linyphiidae	*Agyneta fillmorana*	CNJAE769-12	KM827074
Linyphiidae	*Agyneta fillmorana cf.*	SPRMA916-12	KP655699
Linyphiidae	*Agyneta flibuscrocus*	SPRMA1143-12	KP654090
Linyphiidae	*Agyneta jacksoni*	SPICH745-09	GU684372
Linyphiidae	*Agyneta lophophor*	SPRMA915-12	KP648928
Linyphiidae	*Agyneta micaria*	RBINA1878-13	KP654499

Linyphiidae	*Agyneta nigripes*	SSBAA5273-12	KM827873
Linyphiidae	*Agyneta olivacea*	SPICH002-09	GU683856
Linyphiidae	*Agyneta ordinaria*	SPRMA1167-12	KP656369
Linyphiidae	*Agyneta perspicua*	SPRMA1025-12	KP655426
Linyphiidae	*Agyneta protrudens*	SPRMA1220-12	KP648712
Linyphiidae	*Agyneta serrata*	ARONT808-10	HQ924653
Linyphiidae	*Agyneta simplex*	SSBAD5941-13	KM826339
Linyphiidae	*Agyneta simplex*	SSJAB2027-13	KM833337
Linyphiidae	*Agyneta simplex*	SPRMA887-12	KP649979
Linyphiidae	*Agyneta sp. 2GAB*	SSBAB2775-12	KM828403
Linyphiidae	*Agyneta sp. 3RB*	SPRMA886-12	KP650295
Linyphiidae	*Agyneta sp. 4GAB*	SPRMA1040-12	KP646169
Linyphiidae	*Agyneta unimaculata*	ERSPI328-09	GU682508
Linyphiidae	*Agyneta watertoni*	SPRMA1056-12	KP649392
Linyphiidae	*Allomengea dentisetis*	KKCHE971-09	GU683668
Linyphiidae	*Allomengea scopigera*	KKCHE896-09	KF367812
Linyphiidae	*Anguliphantes nasus*	GBCH11155-13	JN817115
Linyphiidae	*Arcuphantes pennatus*	GBCH11153-13	JN817117
Linyphiidae	*Arcuphantes sylvaticus*	SPRMA1006-12	KP649166
Linyphiidae	*Arcuphantes sylvaticus*	SPRMA846-12	KP651989
Linyphiidae	*Asiceratinops kolymensis*	SPIRU1004-11	KF368808
Linyphiidae	*Baryphyma trifrons*	SPIRU1007-11	KF367910
Linyphiidae	*Baryphyma trifrons affine*	SPRMA1048-12	KF408094
Linyphiidae	*Bathyphantes alascensis*	SPRMA833-12	KP651297
Linyphiidae	*Bathyphantes alboventris*	ARONT625-10	HQ924490
Linyphiidae	*Bathyphantes brevipes*	SPRMA073-10	HQ977153
Linyphiidae	*Bathyphantes brevis*	ARONT606-10	HQ924474
Linyphiidae	*Bathyphantes canadensis*	BBCAN388-09	GU683434
Linyphiidae	*Bathyphantes eumenis*	SPICH872-09	HM416915
Linyphiidae	*Bathyphantes eumenis*	SSPAA6492-13	KM835809
Linyphiidae	*Bathyphantes gracilis*	GBCH5128-10	FJ838650
Linyphiidae	*Bathyphantes gracilis*	CNPAR001-13	KM836935
Linyphiidae	*Bathyphantes keeni*	SPRMA081-10	HQ977154
Linyphiidae	*Bathyphantes keeni*	SPRMA082-10	HQ977155
Linyphiidae	*Bathyphantes orica*	SPRMA263-10	HQ580866
Linyphiidae	*Bathyphantes pallidus*	SPISH038-09	GU683961
Linyphiidae	*Bathyphantes pallidus*	SSPAB8297-13	KM823986
Linyphiidae	*Bathyphantes pallidus*	SSEIA3216-13	KM837259
Linyphiidae	*Bathyphantes pallidus*	SSPAB8326-13	KM838204
Linyphiidae	*Bathyphantes reprobus*	SPICH1033-09	GU684721
Linyphiidae	*Bolyphantes alticeps*	GBCH0411-06	AY078691
Linyphiidae	*Carorita limnaea*	SSPAA7508-13	KM831479
Linyphiidae	*Carorita limnaea*	SSPAA7561-13	KM838590
Linyphiidae	*Centromerita bicolor*	GBCH5720-13	FJ899800

Linyphiidae	*Centromerus longibulbus*	SSBAC4398-13	KM832388
Linyphiidae	*Centromerus sp. 2GAB*	SPRMA1094-12	KP650321
Linyphiidae	*Centromerus sylvaticus*	ERSPI165-09	KP649505
Linyphiidae	*Ceraticelus atriceps*	JSARA026-11	KP651166
Linyphiidae	*Ceraticelus crassiceps*	SPICH227-09	GU683972
Linyphiidae	*Ceraticelus fissiceps*	TDWGB952-10	HQ979372
Linyphiidae	*Ceraticelus laetabilis*	SSEIB8404-13	KM824172
Linyphiidae	*Ceraticelus rowensis*	SSBAF6185-13	KM830878
Linyphiidae	*Ceraticelus similis*	ARONT432-09	GU682939
Linyphiidae	*Ceratinella alaskae*	SPRMA879-12	KP645550
Linyphiidae	*Ceratinella brunnea*	KKCHE594-07	KF367928
Linyphiidae	*Ceratinella brunnea*	SMTPB1484-13	KP651858
Linyphiidae	*Ceratinella ornatula*	SPICH001-09	GU683858
Linyphiidae	*Ceratinella ornatula alaskana*	SPRMA991-12	KP652912
Linyphiidae	*Ceratinops annulipes*	SSPAA6502-13	KM827304
Linyphiidae	*Ceratinops crenatus*	SMTPB1066-13	KP654059
Linyphiidae	*Ceratinops inflatus*	SPRMA978-12	KP657259
Linyphiidae	*Ceratinops latus*	CNPPB2377-12	KM839041
Linyphiidae	*Ceratinopsis labradorensis*	SSBAF6158-13	KM825109
Linyphiidae	*Ceratinopsis nigriceps*	CNBPH357-12	KM828696
Linyphiidae	*Ceratinopsis setoensis*	GBCH11149-13	JN817121
Linyphiidae	*Ceratinopsis*	TDWGB775-10	HQ979218
Linyphiidae	*Ceratinopsis*	TDWGB923-10	HQ979346
Linyphiidae	*Cnephalocotes obscurus*	SPICH405-09	GU684235
Linyphiidae	*Collinsia ksenia*	SPRMA341-10	HQ580900
Linyphiidae	*Collinsia plumosa*	ARONT514-10	HQ924405
Linyphiidae	*Diplocentria bidentata*	ARONT554-10	HQ924441
Linyphiidae	*Diplocentria perplexa*	SPRMA1135-12	KP656885
Linyphiidae	*Diplocentria rectangulata*	SPRMA971-12	KP656567
Linyphiidae	*Diplocephalus cristatus*	ARONT564-10	HQ924450
Linyphiidae	*Diplocephalus subrostratus*	CNRMC1581-12	KM830092
Linyphiidae	*Diplostyla concolor*	ARONT567-10	HQ924453
Linyphiidae	*Dismodicus decemoculatus*	SPICH051-09	GU683835
Linyphiidae	*Doenitzius pruvus*	GBCH11154-13	JN817116
Linyphiidae	*Drapetisca alteranda*	TDWGB913-10	HQ979336
Linyphiidae	*Entelecara acuminata*	CNGIC289-12	KM840446
Linyphiidae	*Entelecara sp. 1GAB*	SPICH869-09	GU684440
Linyphiidae	*Entelecara sp. 1GAB*	CNWLE2557-13	KM832298
Linyphiidae	*Eridantes erigonoides*	ERSPI356-09	HM376106
Linyphiidae	*Eridantes utibilis*	CNPAA470-13	KM833280
Linyphiidae	*Erigone aletris*	SPRMA1029-12	KP651229
Linyphiidae	*Erigone arctica*	SPRMA1092-12	KP647890
Linyphiidae	*Erigone arctica*	SPRMA1126-12	KP657305
Linyphiidae	*Erigone arctophylacis*	SPICH848-09	GU684448

Linyphiidae	*Erigone atra*	ARONT654-10	HQ924519
Linyphiidae	*Erigone atra*	SMTPB6317-13	KP647623
Linyphiidae	*Erigone autumnalis*	ERSPI342-09	GU682502
Linyphiidae	*Erigone blaesa*	ARONT639-10	HQ924504
Linyphiidae	*Erigone cristatopalpus*	SPRMA1105-12	KP657238
Linyphiidae	*Erigone koshiensis*	GBCH11150-13	JN817120
Linyphiidae	*Erigone tirolensis*	KKCHE1005-09	GU683692
Linyphiidae	*Erigone tirolensis*	SPIRU1034-11	KF368029
Linyphiidae	*Erigonoploides sp. 1GAB*	SPRMA1060-12	KP650724
Linyphiidae	*Eskovina clava*	GBCH11148-13	JN817122
Linyphiidae	*Estrandia grandaeva*	GBADC011-10	HQ956675
Linyphiidae	*Eulaira obscura*	SSWLD3219-13	KM828852
Linyphiidae	*Floricomus rostratus*	SPIRU1266-11	KF368088
Linyphiidae	*Floronia bucculenta*	GBCH5124-10	FJ838654
Linyphiidae	*Frederickus coylei*	SPRMA974-12	KP652067
Linyphiidae	*Frederickus wilburi*	SPRMA980-12	KP652350
Linyphiidae	*Frontinella communis*	ARONT707-10	HQ924564
Linyphiidae	*Glyphesis scopulifer*	SMTPB17495-13	KP651921
Linyphiidae	*Gnathonarium taczanowskii*	SPRMA1164-12	KP654106
Linyphiidae	*Gnathonarium*	GBCH11147-13	JN817123
Linyphiidae	*Gonatium crassipalpum*	SPIRU1074-11	KF368109
Linyphiidae	*Gonatium rubellum*	GBCH5122-10	FJ838656
Linyphiidae	*Grammonota angusta*	PHMTU100-10	JN307954
Linyphiidae	*Grammonota angusta*	SPIRU1087-11	KF368113
Linyphiidae	*Grammonota angusta*	CNBPG393-12	KM835917
Linyphiidae	*Grammonota angusta*	CNBPG395-12	KM837668
Linyphiidae	*Grammonota angusta*	SMTPB12363-13	KP646955
Linyphiidae	*Grammonota gentilis*	ARONT374-09	GU682938
Linyphiidae	*Grammonota gigas*	ARONT806-10	HQ924651
Linyphiidae	*Grammonota kincaidi*	CNGIC294-12	KM837707
Linyphiidae	*Grammonota maritima*	KKCHE509-07	KF368130
Linyphiidae	*Grammonota ornata*	ARONT489-10	HQ924384
Linyphiidae	*Grammonota ornata*	ARONT491-10	HQ924386
Linyphiidae	*Grammonota ornata*	ARONT498-10	HQ924393
Linyphiidae	*Helophora insignis*	SSEIA3469-13	KM834413
Linyphiidae	*Helophora reducta*	SPRMA387-10	JF886993
Linyphiidae	*Hilaira canaliculata*	SPICH022-09	HM432622
Linyphiidae	*Hilaira herniosa*	SSBAB512-12	KM825946
Linyphiidae	*Horcotes quadricristatus*	SPIRU1347-11	KF368151
Linyphiidae	*Hybauchenidium cymbadentatum*	SSPAB8136-13	KM825650
Linyphiidae	*Hybauchenidium gibbosum*	KKCHE1002-09	GU683694
Linyphiidae	*Hylyphantes graminicola*	GBCH11151-13	JN817119
Linyphiidae	*Hypomma marxi*	SPICH1070-09	GU684696
Linyphiidae	*Hypomma subarcticum*	SPRMA098-10	HQ580739

Linyphiidae	*Idionella rugosa*	RBINA236-13	KP652658
Linyphiidae	*Improphantes complicatus*	SPRMA1218-12	KP645962
Linyphiidae	*Improphantes sp. 1GAB*	SPRMA898-12	KP648260
Linyphiidae	*Improphantes sp. 1RB*	SPRMA889-12	KP652828
Linyphiidae	*Incestophantes lamprus*	SPRMA793-12	KP657335
Linyphiidae	*Incestophantes mercedes*	SPRMA880-12	KP649775
Linyphiidae	*Incestophantes washingtoni*	KKCHE459-07	KF368176
Linyphiidae	*Incestophantes washingtoni*	SSBAB2888-12	KM824615
Linyphiidae	*Incestophantes washingtoni*	SSBAB2754-12	KM837313
Linyphiidae	*Islandiana falsifica*	SPICH661-09	GU684536
Linyphiidae	*Islandiana flaveola*	SPRMA1123-12	KP657121
Linyphiidae	*Islandiana holmi*	SPICH003-09	GU683875
Linyphiidae	*Kaestneria pullata*	SPICH1084-09	GU684646
Linyphiidae	*Kaestneria pullata*	SSEIB8433-13	KM828518
Linyphiidae	*Kaestneria pullata*	JSARA020-11	KP650093
Linyphiidae	*Kaestneria rufula*	SPICH885-09	GU684458
Linyphiidae	*Lepthyphantes alpinus*	SPICH1021-09	GU684730
Linyphiidae	*Lepthyphantes furcillifer*	SPRMA903-12	KP646800
Linyphiidae	*Lepthyphantes furcillifer*	SPRMA801-12	KP652815
Linyphiidae	*Lepthyphantes intricatus*	SSPAB8244-13	KM840065
Linyphiidae	*Lepthyphantes intricatus*	SPRMA953-12	KP649800
Linyphiidae	*Lepthyphantes leprosus*	SPRMA1083-12	KP648738
Linyphiidae	*Lepthyphantes minutus*	GBCH0409-06	AY078689
Linyphiidae	*Lepthyphantes turbatrix*	SPRMA818-12	KP656674
Linyphiidae	*Linyphantes aeronauticus*	SPRMA1111-12	KP650251
Linyphiidae	*Linyphantes nehalem*	CNGIJ927-13	KM827495
Linyphiidae	*Linyphantes nehalem*	SPRMA1130-12	KP646860
Linyphiidae	*Linyphantes orcinus*	CNPCE075-12	KM829017
Linyphiidae	*Linyphantes pacificus*	SPRMA237-10	HQ580844
Linyphiidae	*Linyphantes pualla*	SPRMA1187-12	KP648901
Linyphiidae	*Linyphantes pualla*	SPRMA1186-12	KP649670
Linyphiidae	*Linyphantes pualla*	SPRMA907-12	KP650109
Linyphiidae	*Linyphantes pualla*	SPRMA806-12	KP653111
Linyphiidae	*Linyphantes victoria*	SPRMA1037-12	KP651004
Linyphiidae	*Linyphia triangularis*	GACAC389-12	FR775771
Linyphiidae	*Lophomma depressum*	SSEIB11406-13	KM833851
Linyphiidae	*Lophomma depressum*	CNPAA469-13	KM834458
Linyphiidae	*Macrargus multesimus*	ARONT428-09	HM434066
Linyphiidae	*Maso sundevalli*	CNEIB1684-12	KM825357
Linyphiidae	*Mecynargus paetulus*	SPICH025-09	GU683878
Linyphiidae	*Mecynargus sp. 2GAB*	SPRMA185-10	HQ580802
Linyphiidae	*Megalepthyphantes nebulosus*	ARONT598-10	HQ924472
Linyphiidae	*Mermessus maculatus*	TDWGB767-10	HQ979210
Linyphiidae	*Mermessus tridentatus*	ARONT817-10	HQ924662

Linyphiidae	*Mermessus tridentatus*	RBINA152-13	KP647140
Linyphiidae	*Mermessus trilobatus*	SPRMA1189-12	KP648686
Linyphiidae	*Mermessus undulatus*	SSBAC4408-13	KM827116
Linyphiidae	*Metopobactrus prominulus*	CNJAC1505-12	KM827412
Linyphiidae	*Microlinyphia dana*	SPRMA1076-12	KP649881
Linyphiidae	*Microlinyphia mandibulata*	ARONT380-09	GU682937
Linyphiidae	*Microlinyphia pusilla*	SPIRU1035-11	KF368296
Linyphiidae	*Microlinyphia pusilla*	CNBAG053-12	KM832934
Linyphiidae	*Microlinyphia pusilla*	CNBAG036-12	KM840638
Linyphiidae	*Microneta viaria*	GBCH11427-13	JQ801599
Linyphiidae	*Microneta viaria*	SPRMA1064-12	KP654855
Linyphiidae	*Mughiphantes sp. 1GAB*	SPICH930-09	GU684464
Linyphiidae	*Mughiphantes sp. 1GAB*	SPRMA1087-12	KP647023
Linyphiidae	*Mythoplastoides erectus*	SPRMA840-12	KP654913
Linyphiidae	*Neriene clathrata*	GBCH5714-13	FJ899809
Linyphiidae	*Neriene clathrata*	ARONT134-09	GU682598
Linyphiidae	*Neriene clathrata*	ARONT589-10	HQ924464
Linyphiidae	*Neriene digna*	SPRMA411-10	JF887002
Linyphiidae	*Neriene emphana*	GBCH11164-13	JN817106
Linyphiidae	*Neriene japonica*	GBCH11156-13	JN817114
Linyphiidae	*Neriene limbatinella*	GBCH11161-13	JN817109
Linyphiidae	*Neriene montana*	ARONT014-09	GU682833
Linyphiidae	*Neriene nigripectoris*	GBCH11157-13	JN817113
Linyphiidae	*Neriene oidedicata*	GBCH11158-13	JN817112
Linyphiidae	*Neriene radiata*	ERSPI312-09	GU682518
Linyphiidae	*Neriene sp. TAB-2009*	GBCH4023-09	FJ607576
Linyphiidae	*Neriene variabilis*	ERSPI265-09	GU682555
Linyphiidae	*Notholepthyphantes australis*	GBCH5117-10	FJ838662
Linyphiidae	*Oedothorax alascensis*	SPRMA133-10	HQ580762
Linyphiidae	*Oedothorax alascensis*	SPRMA325-10	HQ580892
Linyphiidae	*Oedothorax alascensis*	SPRMA1137-12	KP647294
Linyphiidae	*Oedothorax alascensis*	SPRMA1190-12	KP651122
Linyphiidae	*Oedothorax apicatus*	GBCH5115-10	FJ838664
Linyphiidae	*Oedothorax fuscus*	GBCH5712-13	FJ899811
Linyphiidae	*Oedothorax retusus*	GBCH5711-13	FJ899812
Linyphiidae	*Oedothorax trilobatus*	SPICH1103-09	GU684608
Linyphiidae	*Oreoneta banffkluane*	SPRMA1229-12	KP653604
Linyphiidae	*Oreoneta leviceps*	SPRMA1145-12	KP646880
Linyphiidae	*Oreonetides rectangulatus*	SSPAB8101-13	KM836160
Linyphiidae	*Oreonetides sp. 1GAB*	SPRMA890-12	KP653342
Linyphiidae	*Oreonetides vaginatus*	SPICH080-09	GU683850
Linyphiidae	*Pelecopsis mengei*	SPRMA1067-12	KP651028
Linyphiidae	*Pelecopsis sculpta*	SPRMA1023-12	KP652430
Linyphiidae	*Phlattothrata flagellata*	CNRMC1690-12	KM836826

Linyphiidae	*Phlattothrata parva*	SPIRU1272-11	KF368661
Linyphiidae	*Pityohyphantes alticeps*	SPRMA1141-12	KP651218
Linyphiidae	*Pityohyphantes costatus*	ARONT118-09	GU682604
Linyphiidae	*Pityohyphantes cristatus*	ARONT126-09	HM434052
Linyphiidae	*Pityohyphantes cristatus*	CNBAI523-13	KM833736
Linyphiidae	*Pityohyphantes limitaneus*	ERSCH087-07	KF368698
Linyphiidae	*Pityohyphantes subarcticus*	ARONT125-09	GU682600
Linyphiidae	*Pityohyphantes subarcticus*	SSJAA2175-13	KM830138
Linyphiidae	*Pityohyphantes subarcticus*	SSBAB543-12	KM831309
Linyphiidae	*Pocadicnemis americana*	SPICH180-09	GU684007
Linyphiidae	*Pocadicnemis pumila*	SPRMA244-10	HQ580850
Linyphiidae	*Pocadicnemis pumila*	SPRMA421-10	JN310301
Linyphiidae	*Pocadicnemis sp. 2GAB*	ARONT746-10	HQ924599
Linyphiidae	*Poeciloneta calcaratus*	ARONT529-10	HQ924417
Linyphiidae	*Poeciloneta lyrica*	SPRMA957-12	KP655947
Linyphiidae	*Poeciloneta lyrica*	SPRMA910-12	KP656691
Linyphiidae	*Poeciloneta variegata*	SSEIA7675-13	KM838470
Linyphiidae	*Poeciloneta variegata*	SPRMA856-12	KP651834
Linyphiidae	*Porrhomma convexum*	SPIEU154-10	HQ975061
Linyphiidae	*Porrhomma convexum*	SPIEU158-10	HQ975064
Linyphiidae	*Porrhomma convexum*	SSJAA1084-13	KM828801
Linyphiidae	*Porrhomma terrestre*	SPRMA1101-12	KP656791
Linyphiidae	*Praestigia kulczynskii*	SPICH241-09	HM432628
Linyphiidae	*Satilatlas marxi*	SPICH686-09	GU684545
Linyphiidae	*Satilatlas monticola*	SPICH083-09	GU683842
Linyphiidae	*Sciastes dubius*	KKCHE534-07	KF368772
Linyphiidae	*Sciastes hastatus*	SPICH524-09	HM432631
Linyphiidae	*Sciastes mentasta*	ARONT559-10	HQ924446
Linyphiidae	*Sciastes truncatus*	SPRMA1155-12	KP646480
Linyphiidae	*Scirites finitimus*	SPRMA1119-12	KP651148
Linyphiidae	*Scirites sp. 1GAB*	SSPAA7581-13	KM824160
Linyphiidae	*Scironis sima*	SPRMA1139-12	KP654741
Linyphiidae	*Scironis sima*	SPRMA790-12	KP655938
Linyphiidae	*Scironis tarsalis*	ARONT703-10	HQ924561
Linyphiidae	*Scironis tarsalis*	ARONT751-10	HQ924603
Linyphiidae	*Scotinotylus alpinus*	SPICH828-09	GU684442
Linyphiidae	*Scotinotylus bicavatus*	SPRMA766-12	KP648474
Linyphiidae	*Scotinotylus columbia*	SPRMA1103-12	KP651553
Linyphiidae	*Scotinotylus columbia*	SPRMA821-12	KP656392
Linyphiidae	*Scotinotylus evansi*	SPICH098-09	HM432625
Linyphiidae	*Scotinotylus kenus cf.*	SPRMA926-12	KP647756
Linyphiidae	*Scotinotylus majesticus*	SPRMA853-12	KP652090
Linyphiidae	*Scotinotylus pallidus*	SSPAA7537-13	KM836188
Linyphiidae	*Scotinotylus patellatus*	SPRMA761-12	KP656103

Linyphiidae	*Scotinotylus sacer*	SPICH033-09	GU683849
Linyphiidae	*Scotinotylus sanctus*	SPRMA186-10	HQ580803
Linyphiidae	*Scotinotylus sp. 2GAB*	SPRMA429-10	JF887010
Linyphiidae	*Scotinotylus sp. 4GAB*	SSBAC4381-13	KM839663
Linyphiidae	*Scyletria inflata*	SSPAB8308-13	KM831368
Linyphiidae	*Semljicola lapponicus*	KKCHE781-07	KF368781
Linyphiidae	*Semljicola obtusus*	KKCHE950-09	HM377209
Linyphiidae	*Semljicola sp. 1GAB*	SPRMA1047-12	KP652202
Linyphiidae	*Silometopoides pingrensis*	SSBAC4377-13	KM834014
Linyphiidae	*Sisicus apertus*	SSBAB2720-12	KM826850
Linyphiidae	*Sisis rotundus*	SPIRU1348-11	KF368782
Linyphiidae	*Soucron arenarium*	SSWLB5807-13	KM832319
Linyphiidae	*Souessa spinifera*	JSARA098-11	KP652316
Linyphiidae	*Soulgas corticarius*	CNSLQ082-13	KM827773
Linyphiidae	*Spirembolus mundus*	CNEII006-13	KM831852
Linyphiidae	*Spirembolus prominens*	SPRMA245-10	HQ580851
Linyphiidae	*Styloctetor stativus*	SSEIA2106-13	KM830218
Linyphiidae	*Symmigma minimum*	SSWLC3530-13	KM838219
Linyphiidae	*Symmigma minimum*	SSWLC1138-13	KM839372
Linyphiidae	*Tachygyna ursina*	SPRMA224-10	HQ580835
Linyphiidae	*Tachygyna vancouverana*	SPRMA992-12	KP647734
Linyphiidae	*Tachygyna vancouverana*	SPRMA995-12	KP653095
Linyphiidae	*Tapinocyba affinis cf.*	CNWLB645-12	KM835871
Linyphiidae	*Tapinocyba bicarinata*	SPICH031-09	GU683883
Linyphiidae	*Tapinocyba cameroni*	SSEIC4641-13	KM824383
Linyphiidae	*Tapinocyba dietrichi*	SPRMA985-12	KP653802
Linyphiidae	*Tapinocyba hortensis*	ERSPI357-09	GU682485
Linyphiidae	*Tapinocyba minuta*	SPICH189-09	HM432626
Linyphiidae	*Tapinocyba minuta cf.*	SSWLB2490-13	KM826807
Linyphiidae	*Tapinocyba simplex*	SSPAB8861-13	KM832017
Linyphiidae	*Tapinocyba sp. 3GAB*	CNSLC534-12	KM829127
Linyphiidae	*Tennesseellum formica*	SPRMA1042-12	KP651887
Linyphiidae	*Tenuiphantes tenuis*	GBCH12475-13	KC244266
Linyphiidae	*Tenuiphantes zebra*	ARONT560-10	HQ924447
Linyphiidae	*Tenuiphantes zelatus*	SPRMA966-12	KP645772
Linyphiidae	*Tenuiphantes zibus*	SPRMA240-10	HQ580846
Linyphiidae	*Thyreosthenius parasiticus*	SMTPB10640-13	KP646429
Linyphiidae	*Tiso aestivus*	KKCHE1057-09	GU683735
Linyphiidae	*Tiso vagans*	SPRMA1120-12	KP655845
Linyphiidae	*Tmeticus affinis*	SPICH1091-09	GU684611
Linyphiidae	*Tmeticus ornatus*	SPIRU1179-11	KF368924
Linyphiidae	*Tunagyna debilis*	SPICH1108-09	GU684637
Linyphiidae	*Typhochrestus pygmaeus*	SPRMA1022-12	KP645520
Linyphiidae	*Vermontia thoracica*	SSPAA7584-13	KM826743

Linyphiidae	*Wabasso cacuminatus*	SPIRU1335-11	KF368932
Linyphiidae	*Wabasso quaestio*	SPICH423-09	GU684195
Linyphiidae	*Walckenaeria atrotibialis*	ARONT807-10	HQ924652
Linyphiidae	*Walckenaeria castanea*	ARONT547-10	HQ924434
Linyphiidae	*Walckenaeria clavicornis*	SPICH085-09	GU683868
Linyphiidae	*Walckenaeria columbia*	SPRMA838-12	KP646704
Linyphiidae	*Walckenaeria communis*	SPRMA1050-12	KP649982
Linyphiidae	*Walckenaeria cornuella*	SPRMA791-12	KP650463
Linyphiidae	*Walckenaeria directa*	CNEIA2649-12	KM835704
Linyphiidae	*Walckenaeria directa*	SPRMA1138-12	KP647237
Linyphiidae	*Walckenaeria exigua*	SPRMA1121-12	KP652446
Linyphiidae	*Walckenaeria fallax*	ARONT520-10	HQ924409
Linyphiidae	*Walckenaeria kochi*	SPICH071-09	GU683871
Linyphiidae	*Walckenaeria lepida*	SPIRU975-11	KF368943
Linyphiidae	*Walckenaeria obtusa*	GBCH11425-13	JQ801601
Linyphiidae	*Walckenaeria palustris*	SSWLB3474-13	KM826678
Linyphiidae	*Walckenaeria sp. 3GAB*	SSJAC2356-13	KM837865
Linyphiidae	*Walckenaeria spiralis*	SPICH038-09	HM432623
Linyphiidae	*Walckenaeria tibialis*	ERSPI244-09	HM376095
Linyphiidae	*Walckenaeria tricornis*	SPICH007-09	GU683865
Linyphiidae	*Walckenaeria tumida*	SMTPB8135-13	KP654180
Linyphiidae	*Walckenaerianus aimakensis*	SPICH1089-09	GU684629
Linyphiidae	*Wubana drassoides*	SPRMA272-10	HQ580873
Linyphiidae	*Wubana pacifica*	SPRMA965-12	KP652893
Linyphiidae	*Zornella armata*	SPICH199-09	HM432627
Linyphiidae	*Zornella cryptodon*	SPRMA1085-12	KP646234
Linyphiidae	*Zygottus corvallis*	SPRMA1091-12	KP656136
Liocranidae	*Agroeca ornata*	SPICH122-09	GU683786
Liocranidae	*Agroeca ornata*	ARONT800-10	HQ924645
Liocranidae	*Agroeca pratensis*	ERSPI215-09	KP652411
Liphistiidae	*Liphistius erawan*	GBCH11967-13	JQ407803
Lycosidae	*Alopecosa aculeata*	KKCHE053-06	KF367815
Lycosidae	*Alopecosa aculeata*	SSJAB1150-13	KM834031
Lycosidae	*Alopecosa kochi*	SPRMA541-10	JF887050
Lycosidae	*Alopecosa kochi*	SPRMA967-12	KM254048
Lycosidae	*Alopecosa koponeni*	KKCHE1008-09	GU683697
Lycosidae	*Alopecosa licenti*	GBCH11093-13	JN817177
Lycosidae	*Arctosa alpigena*	SSJAA824-13	KM831132
Lycosidae	*Arctosa insignita*	SSJAA1958-13	KM829701
Lycosidae	*Arctosa perita*	SPRMA543-10	JF887052
Lycosidae	*Arctosa raptor*	JDTGS007-09	GU679905
Lycosidae	*Arctosa rubicunda*	SSJAB1159-13	KM833801
Lycosidae	*Geolycosa escambiensis*	GBCH3038-08	DQ151676
Lycosidae	*Geolycosa escambiensis*	GBCH3031-08	DQ151683

Lycosidae	*Geolycosa fatifera*	GBCH3029-08	DQ151685
Lycosidae	*Geolycosa fatifera*	GBCH3028-08	DQ151686
Lycosidae	*Geolycosa micanopy*	GBCH3012-08	DQ151702
Lycosidae	*Geolycosa ornatipes*	GBCH2979-08	DQ151735
Lycosidae	*Geolycosa patellonigra*	GBCH2974-08	DQ151740
Lycosidae	*Geolycosa patellonigra*	GBCH2972-08	DQ151742
Lycosidae	*Geolycosa patellonigra*	GBCH2968-08	DQ151746
Lycosidae	*Geolycosa patellonigra*	GBCH2964-08	DQ151750
Lycosidae	*Geolycosa patellonigra*	GBCH2959-08	DQ151755
Lycosidae	*Geolycosa pikei*	GBCH2950-08	DQ151764
Lycosidae	*Geolycosa pikei*	GBCH2949-08	DQ151765
Lycosidae	*Geolycosa rafaelana*	GBCH2947-08	DQ151767
Lycosidae	*Geolycosa rafaelana*	GBCH2945-08	DQ151769
Lycosidae	*Geolycosa riograndae*	GBCH2943-08	DQ151771
Lycosidae	*Geolycosa turricola*	GBCH2940-08	DQ151774
Lycosidae	*Geolycosa turricola*	GBCH2936-08	DQ151778
Lycosidae	*Geolycosa vultuosa*	GBCH2896-08	DQ151818
Lycosidae	*Geolycosa wrighti*	GBCH2933-08	DQ151781
Lycosidae	*Geolycosa xera*	GBCH2927-08	DQ151787
Lycosidae	*Geolycosa xera*	GBCH2898-08	DQ151816
Lycosidae	*Gladicosa gulosa*	SPRMA542-10	JF887051
Lycosidae	*Hogna carolinensis*	GBCH2890-08	DQ151824
Lycosidae	*Hogna carolinensis*	GBCH2888-08	DQ151826
Lycosidae	*Hogna espanola*	GBCH5763-13	HM171122
Lycosidae	*Hogna frondicola*	SPRMA142-10	KP649116
Lycosidae	*Hogna galapagoensis*	GBCH5765-13	HM171120
Lycosidae	*Hogna junco*	GBCH5769-13	HM171116
Lycosidae	*Lycosa godeffroyi*	GBCH2895-08	DQ151819
Lycosidae	*Pardosa altamontis*	SPRMA963-12	KP653003
Lycosidae	*Pardosa amentata*	GBCH5703-13	FJ899820
Lycosidae	*Pardosa astrigera*	GBCH11088-13	JN817182
Lycosidae	*Pardosa brevivulva*	GBCH11084-13	JN817186
Lycosidae	*Pardosa californica*	GBCNC112-09	KP979217
Lycosidae	*Pardosa distincta*	SPRMA883-12	KP655138
Lycosidae	*Pardosa dorsuncata*	SPRMA553-10	JF887059
Lycosidae	*Pardosa fuscula*	KKCHE032-06	KF368417
Lycosidae	*Pardosa giebeli*	GBCH7617-13	JQ746510
Lycosidae	*Pardosa glacialis*	SPUCH001-12	KF368428
Lycosidae	*Pardosa hedini*	GBCH11085-13	JN817185
Lycosidae	*Pardosa hyperborea*	JDTGS003-09	GU679909
Lycosidae	*Pardosa hyperborea*	SSBAA5246-12	KM834335
Lycosidae	*Pardosa lapidicina*	ARONT612-10	HQ924477
Lycosidae	*Pardosa lapponica*	SPRMA376-10	HQ580923
Lycosidae	*Pardosa lowriei*	SPRMA558-10	JF887063

Lycosidae	*Pardosa metlakatla*	SPRMA562-10	JF887066
Lycosidae	*Pardosa milvina*	ERSPI395-09	GU682447
Lycosidae	*Pardosa moesta*	ERSPI252-09	GU682566
Lycosidae	*Pardosa moesta*	SPRMA561-10	JF887065
Lycosidae	*Pardosa mulaiki*	SSEIA5762-13	KM840327
Lycosidae	*Pardosa nigra*	GBCH7615-13	JQ746512
Lycosidae	*Pardosa ontariensis*	SPRMA547-10	JF887054
Lycosidae	*Pardosa palustris*	GBCH5702-13	FJ899821
Lycosidae	*Pardosa prativaga*	GBCH5701-13	FJ899822
Lycosidae	*Pardosa saturatior*	GBCH7616-13	JQ746511
Lycosidae	*Pardosa saxatilis*	ARONT664-10	HQ924524
Lycosidae	*Pardosa sp. 1GAB*	SPRMA369-10	HQ580918
Lycosidae	*Pardosa tesquorum*	SPRMA958-12	KP652395
Lycosidae	*Pardosa uintana*	JDTGS011-09	GU679901
Lycosidae	*Pardosa vancouveri*	SPRMA564-10	JF887067
Lycosidae	*Pardosa wyuta*	SPRMA788-12	KP650600
Lycosidae	*Pardosa xerampelina*	ERSPI064-08	KP649716
Lycosidae	*Pirata bryantae*	SPISH002-09	HM432633
Lycosidae	*Pirata montanus*	ERSPI350-09	GU682490
Lycosidae	*Pirata piraticus*	ARONT590-10	HQ924465
Lycosidae	*Pirata praedo*	ARONT427-09	GU682896
Lycosidae	*Pirata procurvus*	GBCH11081-13	JN817189
Lycosidae	*Pirata sedentarius*	ARONT112-09	GU682627
Lycosidae	*Pirata sp. 1GAB*	ARONT446-09	HM434070
Lycosidae	*Pirata subpiraticus*	GBCH11083-13	JN817187
Lycosidae	*Piratula cantralli*	ARONT513-10	HQ924404
Lycosidae	*Piratula insularis*	SSBAC2882-12	KM835125
Lycosidae	*Piratula minuta*	ERSPI340-09	GU682501
Lycosidae	*Rabidosa punctulata*	GBCH2897-08	DQ151817
Lycosidae	*Rabidosa rabida*	GBCH5180-10	EU271654
Lycosidae	*Schizocosa ocreata*	GBCH2751-08	EF112495
Lycosidae	*Schizocosa saltatrix*	ARONT461-10	HM880614
Lycosidae	*Schizocosa stridulans*	GBCH2748-08	EF112519
Lycosidae	*Sosippus placidus*	GBCH2891-08	DQ151823
Lycosidae	*Tigrosa helluo*	RBCH119-04	DQ127407
Lycosidae	*Trebacosa marxi*	ARONT795-10	HQ924641
Lycosidae	*Trochosa ruricola*	ARONT319-09	GU682880
Lycosidae	*Trochosa terricola*	ERSPI348-09	GU682499
Lycosidae	*Varacosa avara*	ERSPI065-08	KP654643
Lycosidae	*Venator spenceri*	GBCH7637-13	JQ240194
Lycosidae	*Venatrix pseudospeciosa*	GBCH7636-13	JQ240195
Lycosidae	*Lycosidae sp. lSAWT68*	GBCH2894-08	DQ151820
Lycosidae	*Lycosidae sp. LSAWT89*	GBCH2893-08	DQ151821
Lycosidae		CNBAN018-13	KP979291

Mecicobothriidae	*Hexura picea*	SPRMA286-10	HQ977161
Mecysmaucheniidae	*Aotearoa magna*	GBCH11419-13	JX240238
Mecysmaucheniidae	*Chilarchaea quellon*	GBCH11416-13	JX240241
Mecysmaucheniidae	*Mecysmauchenius segmentatus*	GBCH11417-13	JX240240
Mecysmaucheniidae	*Mesarchaea bellavista*	GBCH11418-13	JX240239
Mecysmaucheniidae	*Zearchaea*	GBCH11415-13	JX240242
Migidae	*Moggridgea australis*	GBCH5437-11	JF749924
Migidae	*Moggridgea intermedia*	GBCH5433-11	JF749928
Migidae	*Moggridgea mordax*	GBCH5432-11	JF749929
Migidae	*Moggridgea peringueyi*	GBCH5434-11	JF749927
Migidae	*Moggridgea rupicoloides*	GBCH5436-11	JF749925
Migidae	*Moggridgea terrestris*	GBCH5435-11	JF749926
Migidae	*Moggridgea tingle*	GBCH5482-11	JF749879
Migidae	*Moggridgea tingle*	GBCH5470-11	JF749891
Migidae	*Moggridgea tingle*	GBCH5461-11	JF749900
Migidae	*Moggridgea tingle*	GBCH5459-11	JF749902
Migidae	*Moggridgea tingle*	GBCH5458-11	JF749903
Migidae	*Moggridgea tingle*	GBCH5457-11	JF749904
Migidae	*Moggridgea tingle*	GBCH5456-11	JF749905
Migidae	*Moggridgea tingle*	GBCH5455-11	JF749906
Migidae	*Moggridgea tingle*	GBCH5454-11	JF749907
Migidae	*Moggridgea tingle*	GBCH5453-11	JF749908
Migidae	*Moggridgea tingle*	GBCH5452-11	JF749909
Migidae	*Moggridgea tingle*	GBCH5449-11	JF749912
Migidae	*Moggridgea tingle*	GBCH5447-11	JF749914
Migidae	*Moggridgea tingle*	GBCH5446-11	JF749915
Migidae	*Moggridgea tingle*	GBCH5445-11	JF749916
Migidae	*Moggridgea tingle*	GBCH5444-11	JF749917
Migidae	*Moggridgea tingle*	GBCH5443-11	JF749918
Migidae	*Moggridgea tingle*	GBCH5441-11	JF749920
Migidae	*Moggridgea tingle*	GBCH5440-11	JF749921
Migidae	*Moggridgea tingle*	GBCH5439-11	JF749922
Migidae	*Moggridgea tingle*	GBCH5438-11	JF749923
Mimetidae	*Ero japonica*	GBCH11193-13	JN817077
Mimetidae	*Mimetus haynesi*	JSARA005-11	KP657148
Mimetidae	*Mimetus hesperus*	SPRMA732-10	JF887154
Mimetidae	*Mimetus notius*	ARONT272-09	GU682725
Miturgidae	*Zora hespera*	SPRMA814-12	KP648913
Mysmenidae	*Maymena ambita*	GBCH8370-13	GU456876
Mysmenidae	*Microdipoena nyungwe*	GBCH8368-13	GU456878
Mysmenidae	*Mysmena*	GBCH8361-13	GU456885
Mysmenidae	*Mysmena*	GBCH8360-13	GU456886
Mysmenidae	*Mysmena*	GBCH8359-13	GU456887
Mysmenidae	*Mysmena*	GBCH8348-13	GU456899

Mysmenidae	*Mysmena*	GBCH8347-13	GU456900
Mysmenidae	*Mysmena*	GBCH8346-13	GU456901
Mysmenidae	*Mysmena*	GBCH8345-13	GU456902
Mysmenidae	*Mysmena*	GBCH8344-13	GU456903
Mysmenidae	*Trogloneta*	GBCH8356-13	GU456891
Mysmenidae		GBCH8364-13	GU456882
Mysmenidae		GBCH8353-13	GU456894
Mysmenidae		GBCH8349-13	GU456898
Nemesiidae	*Aname*	GBCH11866-13	JQ772125
Nemesiidae	*Aname*	GBCH11864-13	JQ772127
Nemesiidae	*Aname*	GBCH11862-13	JQ772129
Nemesiidae	*Aname*	GBCH11860-13	JQ772131
Nemesiidae	*Aname*	GBCH11856-13	JQ772135
Nemesiidae	*Aname*	GBCH11855-13	JQ772136
Nemesiidae	*Aname*	GBCH11853-13	JQ772138
Nemesiidae	*Aname*	GBCH11849-13	JQ772142
Nemesiidae	*Aname*	GBCH11848-13	JQ772143
Nemesiidae	*Aname*	GBCH11841-13	JQ772150
Nemesiidae	*Aname*	GBCH11840-13	JQ772151
Nemesiidae	*Pseudoteyl*	GBCH11867-13	JQ772124
Nemesiidae	*Teyl luculentus*	GBCH11868-13	JQ772123
Nephilidae	*Clitaetra sp. FAPDNA029*	GBCH3912-09	EU003281
Nephilidae	*Herennia multipuncta*	GBCH7179-13	HQ441941
Nephilidae	*Herennia sp. FAPDNA031*	GBCH3905-09	EU003288
Nephilidae	*Nephila antipodiana*	GBCH7196-13	HQ441924
Nephilidae	*Nephila clavata*	GBCH7191-13	HQ441929
Nephilidae	*Nephila clavipes*	GBCH3236-09	FJ525328
Nephilidae	*Nephila clavipes*	GBCH7190-13	HQ441930
Nephilidae	*Nephila fenestrata*	GBCH7186-13	HQ441934
Nephilidae	*Nephila pilipes*	GBCH0373-06	AY052597
Nephilidae	*Nephila pilipes*	GBCH1601-06	DQ779262
Nephilidae	*Nephila pilipes*	GBCH7183-13	HQ441937
Nephilidae	*Nephila plumipes*	GBCH7182-13	HQ441938
Nephilidae	*Nephilengys malabarensis*	GBCH4024-09	FJ607575
Nesticidae	*Nesticella quelpartensis*	GBCH11185-13	JN817085
Nesticidae	*Nesticus barri*	GBCH5009-10	GQ421658
Nesticidae	*Nesticus barri*	GBCH5002-10	GQ421665
Nesticidae	*Nesticus barri*	GBCH4997-10	GQ421670
Nesticidae	*Nesticus barri*	GBCH4995-10	GQ421672
Nesticidae	*Nesticus barri*	GBCH4993-10	GQ421674
Nesticidae	*Nesticus barri*	GBCH4989-10	GQ421678
Nesticidae	*Nesticus barri*	GBCH4983-10	GQ421684
Nesticidae	*Nesticus cellulanus*	ARONT010-09	GU682834
Nesticidae	*Nesticus coreanus*	GBCH11187-13	JN817083

Palpimanidae	*Palpimanus*	GBCH11422-13	JX240235
Periegopidae	*Periegops*	GBCH10173-13	JX174290
Philodromidae	*Apollophanes margareta*	CNGLF1917-13	KM829654
Philodromidae	*Ebo bucklei*	SSWLB017-13	KM830711
Philodromidae	*Ebo evansae*	SPRMA359-10	HQ580910
Philodromidae	*Ebo evansae*	SPRMA942-12	KP651289
Philodromidae	*Ebo iviei*	ARONT492-10	HQ924387
Philodromidae	*Philodromus alascensis*	JDTGS052-09	GU679872
Philodromidae	*Philodromus alascensis*	KKCHE1071-09	GU683746
Philodromidae	*Philodromus aureolus*	GACAC535-12	HE575186
Philodromidae	*Philodromus cespitum*	ARONT400-09	GU682901
Philodromidae	*Philodromus cespitum*	GACAC534-12	HE575185
Philodromidae	*Philodromus cespitum*	GBCH11036-13	JN817235
Philodromidae	*Philodromus dispar*	SPRMA747-10	JF887163
Philodromidae	*Philodromus histrio*	KKCHE1036-09	GU683720
Philodromidae	*Philodromus mineri*	ERSPI291-09	HM376102
Philodromidae	*Philodromus mysticus*	CNWBA002-13	KM840389
Philodromidae	*Philodromus oneida*	CNGLF1528-13	KM836103
Philodromidae	*Philodromus peninsulanus*	ARONT754-10	HQ924606
Philodromidae	*Philodromus rufus*	GBCH11034-13	JN817237
Philodromidae	*Philodromus rufus vibrans*	SPRMA199-10	HQ580813
Philodromidae	*Philodromus rufus vibrans*	SPRMA849-12	KP647263
Philodromidae	*Philodromus spectabilis*	SPRMA945-12	KP649908
Philodromidae	*Philodromus subaureolus*	GBCH11035-13	JN817236
Philodromidae	*Philodromus vulgaris*	ERSPI352-09	GU682491
Philodromidae	*Philodromus vulgaris*	ARONT617-10	HQ924482
Philodromidae	*Thanatus bungei*	SPRMA372-10	HQ580919
Philodromidae	*Thanatus formicinus*	ERSPI242-09	KP647096
Philodromidae	*Thanatus rubicellus*	JDTGS022-09	GU679896
Philodromidae	*Thanatus striatus*	SMTPB18885-13	KP646743
Philodromidae	*Tibellus asiaticus*	SSWLB3951-13	KM833791
Philodromidae	*Tibellus maritimus*	GBADC046-10	HQ956707
Philodromidae	*Tibellus oblongus*	GBCH11032-13	JN817239
Pholcidae	*Anansus*	GBCH11538-13	JX023572
Pholcidae	*Artema atlanta*	GBCH0782-06	AY560771
Pholcidae	*Buitinga cf. nigrescens*	GBCH11537-13	JX023573
Pholcidae	*Buitinga*	GBCH11558-13	JX023552
Pholcidae	*Carapoia genitalis*	GBCH11528-13	JX023582
Pholcidae	*Carapoia*	GBCH11527-13	JX023583
Pholcidae	*Carapoia*	GBCH11526-13	JX023584
Pholcidae	*Carapoia*	GBCH11525-13	JX023585
Pholcidae	*Carapoia*	GBCH11523-13	JX023587
Pholcidae	*Ciboneya antraia*	GBCH0804-06	AY560794
Pholcidae	*Crossopriza lyoni*	GBCH11559-13	JX023551

Pholcidae	*Holocnemus hispanicus*	GBCH11510-13	JX023600
Pholcidae	*Holocnemus pluchei*	GBCH0787-06	AY560776
Pholcidae	*Holocnemus pluchei*	GBCH0788-06	AY560777
Pholcidae	*Khorata khammouan*	GBCH11514-13	JX023596
Pholcidae	*Leptopholcus dschang*	GBCH11548-13	JX023562
Pholcidae	*Leptopholcus gracilis*	GBCH11556-13	JX023554
Pholcidae	*Leptopholcus guineensis*	GBCH11562-13	JX023548
Pholcidae	*Leptopholcus tipula*	GBCH11565-13	JX023545
Pholcidae	*Leptopholcus tipula*	GBCH11508-13	JX023602
Pholcidae	*Leptopholcus*	GBCH11552-13	JX023558
Pholcidae	*Mesabolivar aurantiacus*	GBCH0790-06	AY560779
Pholcidae	*Mesabolivar brasiliensis*	GBCH0791-06	AY560780
Pholcidae	*Mesabolivar cyaneotaeniatus*	GBCH0792-06	AY560781
Pholcidae	*Mesabolivar*	GBCH11520-13	JX023590
Pholcidae	*Mesabolivar*	GBCH11518-13	JX023592
Pholcidae	*Metagonia cf. beni*	GBCH11534-13	JX023576
Pholcidae	*Metagonia cf. bifida*	GBCH11533-13	JX023577
Pholcidae	*Metagonia cf. petropolis*	GBCH11532-13	JX023578
Pholcidae	*Metagonia cf. petropolis*	GBCH11505-13	JX023605
Pholcidae	*Metagonia furcata*	GBCH11519-13	JX023591
Pholcidae	*Metagonia sp. BB-2004b*	GBCH0795-06	AY560784
Pholcidae	*Micropholcus fauroti*	GBCH11536-13	JX023574
Pholcidae	*Modisimus coco*	GBCH11513-13	JX023597
Pholcidae	*Nita elsaff*	GBCH11509-13	JX023601
Pholcidae	*Nyikoa limbe*	GBCH11566-13	JX023544
Pholcidae	*Nyikoa limbe*	GBCH11546-13	JX023564
Pholcidae	*Paramicromerys*	GBCH11516-13	JX023594
Pholcidae	*Pehrforsskalia conopyga*	GBCH11551-13	JX023559
Pholcidae	*Pholcophora americana*	SPRMA735-10	JF887156
Pholcidae	*Pholcophora americana*	SPRMA780-12	KP647977
Pholcidae	*Pholcus atrigularis*	GBCH11504-13	JX023606
Pholcidae	*Pholcus attuleh*	GBCH11547-13	JX023563
Pholcidae	*Pholcus baka*	GBCH11535-13	JX023575
Pholcidae	*Pholcus bourgini*	GBCH11564-13	JX023546
Pholcidae	*Pholcus cf. jaegeri*	GBCH11507-13	JX023603
Pholcidae	*Pholcus crypticolens*	GBCH11544-13	JX023566
Pholcidae	*Pholcus dade*	GBCH11515-13	JX023595
Pholcidae	*Pholcus doucki*	GBCH11563-13	JX023547
Pholcidae	*Pholcus fagei*	GBCH11557-13	JX023553
Pholcidae	*Pholcus fengcheng*	GBCH11502-13	JX023608
Pholcidae	*Pholcus gosuensis*	GBCH11198-13	JN817072
Pholcidae	*Pholcus jiuwei*	GBCH11501-13	JX023609
Pholcidae	*Pholcus kindia*	GBCH11530-13	JX023580
Pholcidae	*Pholcus kribi*	GBCH11549-13	JX023561

Pholcidae	*Pholcus lerutht*	GBCH11554-13	JX023556
Pholcidae	*Pholcus manueli*	ARONT200-09	GU682732
Pholcidae	*Pholcus montanus*	GBCH11194-13	JN817076
Pholcidae	*Pholcus ornatus*	GBCH11511-13	JX023599
Pholcidae	*Pholcus phalangioides*	GBCH11966-13	JQ407804
Pholcidae	*Pholcus sokkrisanensis*	GBCH11196-13	JN817074
Pholcidae	*Pholcus*	GBCH11531-13	JX023579
Pholcidae	*Pholcus*	GBCH11512-13	JX023598
Pholcidae	*Physocyclus globosus*	GBCH0798-06	AY560788
Pholcidae	*Psilochorus hesperus*	SPRMA009-10	HQ977120
Pholcidae	*Psilochorus itaguyrussu*	GBCH11517-13	JX023593
Pholcidae	*Psilochorus simoni*	GBCH0799-06	AY560789
Pholcidae	*Psilochorus*	GBCH11524-13	JX023586
Pholcidae	*Quamtana filmeri*	GBCH11503-13	JX023607
Pholcidae	*Quamtana vidal*	GBCH0802-06	AY560792
Pholcidae	*Quamtana*	GBCH11543-13	JX023567
Pholcidae	*Quamtana*	GBCH11529-13	JX023581
Pholcidae	*Smeringopina guineensis*	GBCH11560-13	JX023550
Pholcidae	*Smeringopina*	GBCH11545-13	JX023565
Pholcidae	*Smeringopus*	GBCH11555-13	JX023555
Pholcidae	*Spermophora cf. berlandi*	GBCH11542-13	JX023568
Pholcidae	*Spermophora minotaura*	GBCH11541-13	JX023569
Pholcidae	*Spermophora minotaura*	GBCH11506-13	JX023604
Pholcidae	*Spermophora senoculata*	GBCH11195-13	JN817075
Pholcidae	*Spermophora*	GBCH11540-13	JX023570
Pholcidae	*Spermophora*	GBCH11539-13	JX023571
Pholcidae	*Trichocyclus sp. BB-2004*	GBCH0783-06	AY560772
Pholcidae	*Tupigea iguassuensis*	GBCH11522-13	JX023588
Pholcidae	*Tupigea nadleri*	GBCH11521-13	JX023589
Phrurolithidae	*Phrurotimpus alarius*	ARONT750-10	HQ924602
Phrurolithidae	*Phrurotimpus borealis*	ARONT438-09	GU682918
Phrurolithidae	*Phrurotimpus certus*	SPRMA961-12	KP649354
Phrurolithidae	*Scotinella britcheri*	ERSPI283-09	GU682538
Phrurolithidae	*Scotinella madisonia*	SMTPB15674-13	KP650459
Phrurolithidae	*Scotinella minnetonka*	ARONT745-10	HQ924598
Phrurolithidae	*Scotinella pugnata*	ERSPI259-09	HM376098
Phyxelididae	*Ambohima andrefana*	GACAC559-12	JF411095
Phyxelididae	*Ambohima antisinanana*	GACAC565-12	JF411101
Phyxelididae	*Ambohima avaratra*	GACAC574-12	JF411110
Phyxelididae	*Ambohima maizina*	GACAC554-12	JF411090
Phyxelididae	*Ambohima ranohira*	GACAC562-12	JF411098
Phyxelididae	*Ambohima sublima*	GACAC566-12	JF411102
Phyxelididae	*Ambohima sublima*	GACAC568-12	JF411104
Phyxelididae	*Ambohima sublima*	GACAC569-12	JF411105

Phyxelididae	*Ambohima sublima*	GACAC571-12	JF411107
Phyxelididae	*Ambohima sublima*	GACAC572-12	JF411108
Phyxelididae	*Ambohima sublima*	GACAC573-12	JF411109
Phyxelididae	*Ambohima vato*	GACAC563-12	JF411099
Phyxelididae	*Ambohima vato*	GACAC564-12	JF411100
Phyxelididae	*Ambohima zandry*	GACAC553-12	JF411089
Phyxelididae	*Ambohima zoky*	GACAC555-12	JF411091
Phyxelididae	*Malaika longipes*	GACAC550-12	JF411086
Phyxelididae	*Manampoka atsimo*	GACAC583-12	JF411119
Phyxelididae	*Phyxelida tanganensis*	GACAC551-12	JF411087
Phyxelididae	*Rahavavy fanivelona*	GACAC576-12	JF411112
Phyxelididae	*Rahavavy ida*	GACAC578-12	JF411114
Phyxelididae	*Rahavavy malagasyana*	GACAC580-12	JF411116
Phyxelididae	*Rahavavy malagasyana*	GACAC582-12	JF411118
Phyxelididae	*Themacrys irrorata*	GACAC575-12	JF411111
Phyxelididae	*Xevioso colobata*	GACAC549-12	JF411085
Phyxelididae	*Xevioso kulufa*	GACAC548-12	JF411084
Pimoidae	*Pimoa altioculata*	CNPCO029-13	KM826392
Pimoidae	*Pimoa altioculata*	SPRMA867-12	KP654481
Pimoidae	*Pimoa haden*	SPRMA827-12	KP650096
Pimoidae	*Pimoa sp. TAB-2009*	GBCH4015-09	FJ607584
Pisauridae	*Dolomedes aquaticus*	GBCH5799-13	GQ337356
Pisauridae	*Dolomedes horishanus*	GBCH3538-09	AB374063
Pisauridae	*Dolomedes japonicus*	GBCH11078-13	JN817192
Pisauridae	*Dolomedes raptor*	GBCH11077-13	JN817193
Pisauridae	*Dolomedes scriptus*	ARONT340-09	GU682879
Pisauridae	*Dolomedes sulfureus*	GBCH11079-13	JN817191
Pisauridae	*Dolomedes tenebrosus*	GBCH4037-09	FJ607562
Pisauridae	*Dolomedes triton*	ARONT497-10	HQ924392
Pisauridae	*Pisaura ancora*	GBCH11076-13	JN817194
Pisauridae	*Pisaura lama*	GBCH11075-13	JN817195
Pisauridae	*Pisaurina brevipes*	RBINA3403-13	KP657364
Pisauridae		GBCH10172-13	JX137131
Psechridae	*Fecenia cylindrata*	GBCH10093-13	JX137210
Psechridae	*Fecenia cylindrata*	GBCH10091-13	JX137212
Psechridae	*Fecenia cylindrata*	GBCH10089-13	JX137214
Psechridae	*Fecenia cylindrata*	GBCH10085-13	JX137218
Psechridae	*Fecenia ochracea*	GBCH10147-13	JX137156
Psechridae	*Fecenia ochracea*	GBCH10134-13	JX137169
Psechridae	*Fecenia travancoria*	GBCH10165-13	JX137138
Psechridae	*Fecenia*	GBCH10151-13	JX137152
Psechridae	*Fecenia*	GBCH10149-13	JX137154
Psechridae	*Fecenia*	GBCH10133-13	JX137170
Psechridae	*Fecenia*	GBCH10096-13	JX137207

Psechridae	*Psechrus ancoralis*	GBCH10154-13	JX137149
Psechridae	*Psechrus ancoralis*	GBCH10123-13	JX137180
Psechridae	*Psechrus ancoralis*	GBCH10116-13	JX137187
Psechridae	*Psechrus ancoralis*	GBCH10114-13	JX137189
Psechridae	*Psechrus ancoralis*	GBCH10113-13	JX137190
Psechridae	*Psechrus ancoralis*	GBCH10112-13	JX137191
Psechridae	*Psechrus ancoralis*	GBCH10107-13	JX137196
Psechridae	*Psechrus ancoralis*	GBCH10098-13	JX137205
Psechridae	*Psechrus antraeus*	GBCH10082-13	JX137221
Psechridae	*Psechrus ghecuanus*	GBCH10138-13	JX137165
Psechridae	*Psechrus himalayanus*	GBCH10135-13	JX137168
Psechridae	*Psechrus khammouan*	GBCH10125-13	JX137178
Psechridae	*Psechrus rani*	GBCH10140-13	JX137163
Psechridae	*Psechrus senoculatus*	GBCH10155-13	JX137148
Psechridae	*Psechrus sinensis*	GBCH10157-13	JX137146
Psechridae	*Psechrus singaporensis*	GBCH10141-13	JX137162
Psechridae	*Psechrus*	GBCH10153-13	JX137150
Psechridae	*Psechrus*	GBCH10136-13	JX137167
Psechridae	*Psechrus*	GBCH10124-13	JX137179
Psechridae	*Psechrus*	GBCH10121-13	JX137182
Psechridae		GBCH10171-13	JX137132
Psechridae		GBCH10169-13	JX137134
Psechridae		GBCH10168-13	JX137135
Psechridae		GBCH10164-13	JX137139
Psechridae		GBCH10162-13	JX137141
Psechridae		GBCH10160-13	JX137143
Psechridae		GBCH10152-13	JX137151
Psechridae		GBCH10144-13	JX137159
Psechridae		GBCH10132-13	JX137171
Psechridae		GBCH10130-13	JX137173
Psechridae		GBCH10129-13	JX137174
Psechridae		GBCH10105-13	JX137198
Psechridae		GBCH10087-13	JX137216
Salticidae	*Admestina tibialis*	GMNCG467-12	KJ091626
Salticidae	*Asianellus festivus*	GBCH11017-13	JN817254
Salticidae	*Carrhotus xanthogramma*	GBCH11016-13	JN817255
Salticidae	*Chalcoscirtus alpicola*	SPIRU1282-11	KF367929
Salticidae	*Dendryphantes czekanowskii*	SPRMA374-10	HQ580921
Salticidae	*Dendryphantes nigromaculatus*	SPRMA859-12	KP649328
Salticidae	*Dendryphantes nigromaculatus*	SPRMA860-12	KP652587
Salticidae	*Dendryphantes sp. 1GAB*	RBINA3400-13	KP652400
Salticidae	*Eris militaris*	ARONT350-09	GU682851
Salticidae	*Euophrys monadnock*	CNGRD1300-12	KM824291
Salticidae	*Euophrys monadnock*	SPRMA776-12	KP647526

Salticidae	*Evarcha albaria*	GBCH11015-13	JN817256
Salticidae	*Evarcha hoyi*	ARONT351-09	GU682853
Salticidae	*Evarcha hoyi*	ARONT188-09	HM434057
Salticidae	*Evarcha hoyi*	SPRMA175-10	HQ580795
Salticidae	*Evarcha hoyi*	SPRMA281-10	HQ580880
Salticidae	*Evarcha hoyi*	SPRMA658-10	JF887112
Salticidae	*Evarcha hoyi*	SSEIB11399-13	KM836492
Salticidae	*Evarcha proszynskii*	SSJAB2259-13	KM824759
Salticidae	*Habronattus americanus*	SPRMA273-10	HQ580874
Salticidae	*Habronattus decorus*	ERSPI313-09	GU682519
Salticidae	*Habronattus oregonensis*	GBCH0812-06	AY571145
Salticidae	*Habronattus viridipes*	RBCH143-04	DQ127385
Salticidae	*Hakka himeshimensis*	GBCH10993-13	JN817278
Salticidae	*Heliophanus flavipes*	GACAC388-12	FR775770
Salticidae	*Heliophanus ussuricus*	GBCH10994-13	JN817277
Salticidae	*Hentzia mitrata*	TDWGB950-10	HQ979370
Salticidae	*Hentzia mitrata*	CNSLP1064-13	KM831623
Salticidae	*Maevia inclemens*	ARONT151-09	GU682638
Salticidae	*Marpissa formosa*	ARONT765-10	HQ924616
Salticidae	*Marpissa milleri*	GBCH11008-13	JN817263
Salticidae	*Mendoza canestrinii*	GBCH11007-13	JN817264
Salticidae	*Menemerus fulvus*	GBCH11004-13	JN817267
Salticidae	*Metaphidippus sp. 1GAB*	SPRMA183-10	HQ580800
Salticidae	*Myrmarachne japonica*	GBCH10988-13	JN817283
Salticidae	*Naphrys pulex*	ARONT068-09	GU682819
Salticidae	*Neon nelli*	SPRMA665-10	JF887113
Salticidae	*Paraphidippus aurantius*	GBCH4018-09	FJ607581
Salticidae	*Pelegrina aeneola*	SSWLD7249-13	KM839358
Salticidae	*Pelegrina aeneola*	SPRMA977-12	KP651664
Salticidae	*Pelegrina flaviceps*	SSPAA6455-13	KM834379
Salticidae	*Pelegrina flavipes*	ARONT210-09	GU682659
Salticidae	*Pelegrina galathea*	ARONT722-10	HQ924577
Salticidae	*Pelegrina insignis*	ERSPI304-09	GU682524
Salticidae	*Pelegrina montana*	CNRMC1574-12	KM836993
Salticidae	*Pelegrina proterva*	TDWGB924-10	HQ979347
Salticidae	*Pelegrina proterva*	RBINA2802-13	KP649076
Salticidae	*Pellenes ignifrons*	SPRMA782-12	KP645944
Salticidae	*Pellenes lapponicus*	SPISH009-09	GU683925
Salticidae	*Phanias albeolus*	SPRMA315-10	HQ977181
Salticidae	*Phidippus audax*	ARONT300-09	GU682852
Salticidae	*Phidippus borealis*	CNRMD2873-13	KM825265
Salticidae	*Phidippus clarus*	ARONT704-10	HQ924562
Salticidae	*Phidippus johnsoni*	SPRMA670-10	JF887115
Salticidae	*Phidippus princeps*	ARONT383-09	HM434064

Salticidae	*Phidippus purpuratus*	ARONT022-09	GU682794
Salticidae	*Phintella abnormis*	GBCH10987-13	JN817284
Salticidae	*Phintella arenicolor*	GBCH10986-13	JN817285
Salticidae	*Platycryptus californicus*	SPRMA671-10	JF887116
Salticidae	*Platycryptus undatus*	ARONT039-09	GU682796
Salticidae	*Plexippoides annulipedis*	GBCH11012-13	JN817259
Salticidae	*Plexippus paykulli*	GBCH11010-13	JN817261
Salticidae	*Pseudeuophrys iwatensis*	GBCH11000-13	JN817271
Salticidae	*Pseudicius vulpes*	GBCH10992-13	JN817279
Salticidae	*Rhene atrata*	GBCH11003-13	JN817268
Salticidae	*Salticus scenicus*	ARONT009-09	GU682804
Salticidae	*Salticus scenicus*	ARONT007-09	GU682812
Salticidae	*Sibianor pullus*	GBCH11002-13	JN817269
Salticidae	*Siler cupreus*	GBCH11001-13	JN817270
Salticidae	*Sitticus albolineatus*	GBCH10999-13	JN817272
Salticidae	*Sitticus ammophilus*	ARONT179-09	HM434055
Salticidae	*Sitticus concolor*	CNPPE2332-12	KJ166443
Salticidae	*Sitticus finschi*	SPIRU1078-11	KF368783
Salticidae	*Sitticus floricola palustris*	ERSPI394-09	GU682461
Salticidae	*Sitticus floricola palustris*	SPIRU1012-11	KF368788
Salticidae	*Sitticus penicillatus*	GBCH10996-13	JN817275
Salticidae	*Sitticus ranieri*	KKCHE945-09	GU683651
Salticidae	*Sitticus striatus*	SPIRU1159-11	KF368804
Salticidae	*Synageles occidentalis*	CNWBE379-13	KM839132
Salticidae	*Synagelides agoriformis*	GBCH10989-13	JN817282
Salticidae	*Synemosyna formica*	SPRMA675-10	JF887118
Salticidae	*Talavera minuta*	ARONT803-10	HQ924648
Salticidae	*Telamonia vlijmi*	GBCH10990-13	JN817281
Salticidae	*Terralonus sp. 1GAB*	CNPPF1456-12	KJ166721
Salticidae	*Trite planiceps*	GBCH7202-13	JF836858
Salticidae	*Trite planiceps*	GBCH7197-13	JF836863
Salticidae	*Tutelina harti*	JSARA019-11	KP652795
Salticidae	*Tutelina similis*	ARONT787-10	HQ924634
Salticidae	*Tutelina sp. 1GAB*	RBINA5809-13	KP647738
Scytodidae	*Scytodes thoracica*	ARONT183-09	HM434056
Segestriidae	*Segestria bavarica*	GBCH0811-06	AY560802
Segestriidae	*Segestria pacifica*	CNGID206-12	KM827799
Sicariidae	*Loxosceles sp. RPD-2010*	GACAC391-12	GQ279111
Sicariidae	*Loxosceles sp. RPD-2010*	GACAC402-12	GQ279221
Sicariidae	*Loxosceles sp. RPD-2010*	GACAC417-12	GQ279236
Sparassidae	*Damastes sp. SB-2009*	GBCH5137-10	GQ855822
Sparassidae	*Heteropoda aemulans*	GBCH5134-10	GQ855825
Sparassidae	*Heteropoda dagmarae*	GBCH5146-10	GQ855813
Sparassidae	*Heteropoda maxima*	GBCH5147-10	GQ855812

Sparassidae	*Heteropoda simplex*	GBCH5143-10	GQ855816
Sparassidae	*Heteropoda simplex*	GBCH5133-10	GQ855826
Sparassidae	*Heteropoda simplex*	GBCH5131-10	GQ855828
Sparassidae	*Heteropoda steineri*	GBCH5132-10	GQ855827
Sparassidae	*Heteropoda tetrica*	GBCH5148-10	GQ855811
Sparassidae	*Heteropoda venatoria*	GBCH5142-10	GQ855817
Sparassidae	*Pseudopoda namkhan*	GBCH5138-10	GQ855821
Sparassidae	*Sinopoda koreana*	GBCH11039-13	JN817232
Sparassidae	*Sinopoda sp. SB-2009*	GBCH5151-10	GQ855808
Sparassidae	*Sinopoda stellatops*	GBCH11038-13	JN817233
Stenochilidae	*Colopea*	GBCH11421-13	JX240236
Stiphidiidae	*Stiphidion facetum*	GBCH10137-13	JX137166
Symphytognathidae		GBCH8339-13	GU456909
Symphytognathidae		GBCH8338-13	GU456910
Symphytognathidae		GBCH8337-13	GU456911
Symphytognathidae		GBCH8336-13	GU456912
Symphytognathidae		GBCH8335-13	GU456913
Telemidae	*Usofila pacifica*	SSWLC3102-13	KM828192
Tetragnathidae	*Azilia guatemalensis*	GBCH3913-09	EU003280
Tetragnathidae	*Cyrtognatha espanola*	GBCH3910-09	EU003283
Tetragnathidae	*Dolichognatha sp. FAPDNA061*	GBCH3908-09	EU003285
Tetragnathidae	*Glenognatha foxi*	ARONT263-09	HM434061
Tetragnathidae	*Leucauge argyra*	GBCH3903-09	EU003290
Tetragnathidae	*Leucauge blanda*	GBCH11141-13	JN817129
Tetragnathidae	*Leucauge venusta*	GBCH3241-09	FJ525323
Tetragnathidae	*Leucauge venusta*	ARONT081-09	GU682832
Tetragnathidae	*Leucauge*	GBCH7174-13	HQ441946
Tetragnathidae	*Leucauge*	GBCH11140-13	JN817130
Tetragnathidae	*Menosira ornata*	GBCH11138-13	JN817132
Tetragnathidae	*Meta manchurica*	GBCH11137-13	JN817133
Tetragnathidae	*Meta menardi*	GBCH3898-09	EU003295
Tetragnathidae	*Meta ovalis*	GBCH4028-09	FJ607571
Tetragnathidae	*Metabus ebanoverde*	GBCH3897-09	EU003296
Tetragnathidae	*Metellina curtisi*	SPRMA234-10	HQ580841
Tetragnathidae	*Metellina segmentata*	GBCH4027-09	FJ607572
Tetragnathidae	*Metleucauge yunohamensis*	GBCH11136-13	JN817134
Tetragnathidae	*Opadometa sp. FAPDNA047*	GBCH3890-09	EU003304
Tetragnathidae	*Orsinome sp. FAPDNA052*	GBCH3889-09	EU003305
Tetragnathidae	*Pachygnatha autumnalis*	ERSPI246-09	HM376096
Tetragnathidae	*Pachygnatha clercki*	GBCH5709-13	FJ899814
Tetragnathidae	*Pachygnatha clercki*	GBCH5708-13	FJ899815
Tetragnathidae	*Pachygnatha clercki*	SPICH1078-09	GU684581
Tetragnathidae	*Pachygnatha clercki*	GBCH11135-13	JN817135
Tetragnathidae	*Pachygnatha clercki*	SSPAB8079-13	KM831461

Tetragnathidae	*Pachygnatha degeeri*	GBCH3888-09	EU003306
Tetragnathidae	*Pachygnatha degeeri*	GBCH5706-13	FJ899817
Tetragnathidae	*Pachygnatha dorothea*	ARONT569-10	HQ924455
Tetragnathidae	*Pachygnatha tristriata*	ARONT521-10	HQ924410
Tetragnathidae	*Pachygnatha xanthostoma*	RBINA2246-13	KP653681
Tetragnathidae	*Tetragnatha anuenue*	GBCH0762-06	AY530512
Tetragnathidae	*Tetragnatha brevignatha*	GBCH0731-06	AY530481
Tetragnathidae	*Tetragnatha brevignatha*	GBCH0744-06	AY530494
Tetragnathidae	*Tetragnatha brevignatha*	GBCH0751-06	AY530501
Tetragnathidae	*Tetragnatha caudata*	TDWGB921-10	HQ979344
Tetragnathidae	*Tetragnatha caudata*	SSPAB9958-13	KM825786
Tetragnathidae	*Tetragnatha caudata*	RBINA116-13	KP654977
Tetragnathidae	*Tetragnatha caudata*	RBINA238-13	KP657466
Tetragnathidae	*Tetragnatha dearmata*	SSEIC4895-13	KM825870
Tetragnathidae	*Tetragnatha elongata*	ARONT073-09	GU682831
Tetragnathidae	*Tetragnatha extensa*	ERSCH034-07	KF368815
Tetragnathidae	*Tetragnatha extensa*	ARSO001-08	KM827368
Tetragnathidae	*Tetragnatha extensa*	SSBAF7744-13	KM834537
Tetragnathidae	*Tetragnatha laboriosa*	ERSPI398-09	GU682453
Tetragnathidae	*Tetragnatha laboriosa*	ARSO009-08	KM824128
Tetragnathidae	*Tetragnatha montana*	GBCH5697-13	FJ899831
Tetragnathidae	*Tetragnatha moua*	GBCH3585-09	EU796906
Tetragnathidae	*Tetragnatha nigrita*	GBCH11146-13	JN817124
Tetragnathidae	*Tetragnatha pallescens*	RBCH185-04	DQ127347
Tetragnathidae	*Tetragnatha rava*	GBCH3589-09	EU796902
Tetragnathidae	*Tetragnatha straminea*	ARONT819-10	HQ924664
Tetragnathidae	*Tetragnatha straminea*	SSPAB9129-13	KM836544
Tetragnathidae	*Tetragnatha straminea*	CNSLO002-13	KM840669
Tetragnathidae	*Tetragnatha versicolor*	GBCH3247-09	FJ525317
Tetragnathidae	*Tetragnatha versicolor*	ARONT604-10	HQ924473
Tetragnathidae	*Tetragnatha versicolor*	SPRMA032-10	HQ977134
Tetragnathidae	*Tetragnatha versicolor*	KKCHE190-06	KF368892
Tetragnathidae	*Tetragnatha versicolor*	CNPAH370-13	KM824717
Tetragnathidae	*Tetragnatha versicolor*	SSPAB120-13	KM827548
Tetragnathidae	*Tetragnatha versicolor*	SSPAA5568-13	KM828384
Tetragnathidae	*Tetragnatha versicolor*	SSPAB5307-13	KM833818
Tetragnathidae	*Tetragnatha versicolor*	SSPAB9133-13	KM834894
Tetragnathidae	*Tetragnatha versicolor*	SSPAB4591-13	KM838299
Tetragnathidae	*Tetragnatha versicolor*	RBINA252-13	KP647886
Tetragnathidae	*Tetragnatha versicolor*	PHMTU099-10	KP654884
Tetragnathidae	*Tetragnatha versicolor*	ERSCH029-07	KP656014
Tetragnathidae	*Tetragnatha viridis*	ARONT714-10	HQ924570
Tetragnathidae	*Tetragnatha*	GBCH5699-13	FJ899828
Tetragnathidae	*Tylorida striata*	GBCH3885-09	EU003309

Tetragnathidae	*Metinae sp. FAPDNA067*	GBCH4335-09	EU003299
Theraphosidae	*Aphonopelma sp. 1 SEM-2008*	GBCH2389-08	EU523754
Theraphosidae	*Coremiocnemis cunicularia*	GBCH7750-13	JN018198
Theraphosidae	*Cyriopagopus schioedtei*	GBCH7800-13	JN018126
Theraphosidae	*Cyriopagopus schioedtei*	GBCH7749-13	JN018199
Theraphosidae	*Haplopelma schmidti*	GBCH7799-13	JN018127
Theridiidae	*Anelosimus crassipes*	GBCH11174-13	JN817096
Theridiidae	*Ariamnes cylindrogaster*	GBCH11165-13	JN817105
Theridiidae	*Asagena americana*	SPRMA055-10	HQ977144
Theridiidae	*Canalidion montanum*	SSBAB2896-12	KM838796
Theridiidae	*Chikunia albipes*	GBCH11172-13	JN817098
Theridiidae	*Chrysso octomaculata*	GBCH11171-13	JN817099
Theridiidae	*Crustulina sticta*	SPRMA882-12	KP653582
Theridiidae	*Cryptachaea blattea*	GBCH4238-09	EU935469
Theridiidae	*Cryptachaea blattea*	GBCH11639-13	JN859114
Theridiidae	*Cryptachaea canionis*	SPRMA038-10	HQ977136
Theridiidae	*Cryptachaea veruculata*	GBCH11650-13	JN859103
Theridiidae	*Cryptachaea veruculata*	GBCH11648-13	JN859105
Theridiidae	*Dipoena nigra*	ARONT390-09	GU682916
Theridiidae	*Dipoena sp. 1GAB*	SSPAA6458-13	KM831562
Theridiidae	*Dipoena washougalia*	SPRMA975-12	KP651070
Theridiidae	*Enoplognatha intrepida*	SPRMA920-12	KP653158
Theridiidae	*Enoplognatha ovata*	ARONT062-09	GU682761
Theridiidae	*Enoplognatha thoracica*	SPRMA040-10	HQ977138
Theridiidae	*Episinus nubilus*	GBCH11169-13	JN817101
Theridiidae	*Euryopis argentea*	CNWLL215-13	KM826661
Theridiidae	*Euryopis funebris*	ARONT711-10	HQ924567
Theridiidae	*Euryopis pepini*	SSWLB2434-13	KM839178
Theridiidae	*Euryopis scriptipes*	SSJAB3385-13	KM839048
Theridiidae	*Lasaeola prona*	SPRMA919-12	KP648424
Theridiidae	*Latrodectus geometricus*	GBCH4032-09	FJ607567
Theridiidae	*Latrodectus hasselti*	GBCH2694-08	EF121033
Theridiidae	*Latrodectus hesperus*	RBCH214-04	DQ127315
Theridiidae	*Latrodectus hesperus*	SPRMA047-10	HQ977141
Theridiidae	*Latrodectus hesperus*	GBCH11212-13	KC414080
Theridiidae	*Latrodectus katipo*	GBCH3290-09	EU305454
Theridiidae	*Latrodectus mactans*	RBCH207-04	DQ127321
Theridiidae	*Latrodectus pallidus*	GBCH11210-13	KC414082
Theridiidae	*Latrodectus thoracicus*	GBCH8329-13	GU112101
Theridiidae	*Latrodectus tredecimguttatus*	GBCH11208-13	KC414084
Theridiidae	*Latrodectus*	GBCH11217-13	KC414075
Theridiidae	*Neospintharus trigonum*	ARONT712-10	HQ924568
Theridiidae	*Neottiura bimaculata*	ERSPI295-09	HM376103
Theridiidae	*Neottiura bimaculata*	SPRMA755-10	JF887169

Theridiidae	*Nesticodes rufipes*	GBCH11629-13	JN859124
Theridiidae	*Ohlertidion ohlerti*	GBADC002-10	HQ956666
Theridiidae	*Parasteatoda kompirensis*	GBCH11179-13	JN817091
Theridiidae	*Parasteatoda tabulata*	ERSPI378-09	GU682469
Theridiidae	*Parasteatoda tepidariorum*	GBCH11628-13	JN859125
Theridiidae	*Phoroncidia americana*	GBCH4016-09	FJ607583
Theridiidae	*Phycosoma mustelinum*	GBCH11173-13	JN817097
Theridiidae	*Phylloneta impressa*	SPIRU977-11	KF368663
Theridiidae	*Platnickina mneon*	GBCH11626-13	JN859127
Theridiidae	*Platnickina tincta*	ARONT108-09	HM434050
Theridiidae	*Robertus borealis*	SSBAC2895-12	KM825401
Theridiidae	*Robertus crosbyi*	SPIRU1039-11	KF368756
Theridiidae	*Robertus fuscus*	SPICH626-09	GU684519
Theridiidae	*Robertus lyrifer*	KKCHE1087-09	HM377208
Theridiidae	*Robertus vigerens*	SPRMA126-10	HQ580760
Theridiidae	*Rugathodes aurantius*	SSEIC4894-13	KM836123
Theridiidae	*Rugathodes sexpunctatus*	SPRMA329-10	HQ580894
Theridiidae	*Steatoda albomaculata*	SPRMA952-12	KP654227
Theridiidae	*Steatoda bipunctata*	ERSPI376-09	GU682467
Theridiidae	*Steatoda borealis*	ARONT065-09	GU682827
Theridiidae	*Steatoda borealis*	SPRMA308-10	HQ977176
Theridiidae	*Steatoda capensis*	GBCH11206-13	KC414086
Theridiidae	*Steatoda cingulata*	GBCH11167-13	JN817103
Theridiidae	*Steatoda grossa*	SPRMA279-10	HQ580878
Theridiidae	*Steatoda grossa*	SPRMA056-10	HQ977145
Theridiidae	*Steatoda grossa*	SPRMA067-10	HQ977151
Theridiidae	*Steatoda grossa*	SPRMA068-10	HQ977152
Theridiidae	*Steatoda hespera*	SPRMA152-10	HQ580777
Theridiidae	*Steatoda triangulosa*	ERSPI337-09	GU682505
Theridiidae	*Steatoda triangulosa*	GBCH11166-13	JN817104
Theridiidae	*Stemmops nipponicus*	GBCH11168-13	JN817102
Theridiidae	*Takayus latifolius*	GBCH11178-13	JN817092
Theridiidae	*Takayus quadrimaculatus*	GBCH11175-13	JN817095
Theridiidae	*Takayus takayensis*	GBCH11177-13	JN817093
Theridiidae	*Theonoe stridula*	SSBAC4373-13	KM830622
Theridiidae	*Theridion albidum*	CNPPH1198-12	KJ443993
Theridiidae	*Theridion californicum*	GACAC785-12	JF979389
Theridiidae	*Theridion differens*	ARONT405-09	GU682912
Theridiidae	*Theridion frondeum*	CNEIC3170-12	KM824750
Theridiidae	*Theridion gigantipes*	GBCH11655-13	JN859098
Theridiidae	*Theridion glaucescens*	CNPPE2335-12	KJ085199
Theridiidae	*Theridion grallator*	GBCH11990-13	JN863369
Theridiidae	*Theridion murarium*	ERSPI374-09	GU682477
Theridiidae	*Theridion mystaceum*	SPRMA756-10	JF887170

Theridiidae	*Theridion petraeum*	SPRMA918-12	KP648658
Theridiidae	*Theridion pictum*	GBADC026-10	HQ956689
Theridiidae	*Theridion sp. 2GAB*	SPRMA063-10	HQ977148
Theridiidae	*Theridion varians*	ARONT231-09	GU682663
Theridiidae	*Theridula emertoni*	CNPPI1857-12	KJ444474
Theridiidae	*Thymoites camano*	SPRMA768-12	KP646651
Theridiidae	*Thymoites oleatus*	SPICH338-09	GU684347
Theridiidae	*Thymoites unimaculatus*	ARONT415-09	GU682915
Theridiidae	*Wamba crispulus*	PHJUN4070-12	KP651256
Theridiidae	*Yunohamella lyrica*	ARONT260-09	HM434060
Theridiidae		GBCH7797-13	JN018129
Theridiosomatidae	*Theridiosoma gemmosum*	ARONT452-09	GU682945
Theridiosomatidae		GBCH8334-13	GU456914
Thomisidae	*Bassaniana utahensis*	ARONT329-09	GU682878
Thomisidae	*Coriarachne brunneipes*	SPIRU1095-11	KF367975
Thomisidae	*Diaea subdola*	GBCH11029-13	JN817242
Thomisidae	*Ebelingia kumadai*	GBCH11030-13	JN817241
Thomisidae	*Ebrechtella tricuspidata*	GBCH11031-13	JN817240
Thomisidae	*Lysiteles coronatus*	GBCH11026-13	JN817245
Thomisidae	*Mecaphesa asperata*	ARONT155-09	GU682624
Thomisidae	*Misumena vatia*	JSARA011-11	KP650268
Thomisidae	*Misumessus oblongus*	TDWGB785-10	HQ979227
Thomisidae	*Ozyptila arctica*	JDTGS010-09	GU679904
Thomisidae	*Ozyptila conspurcata*	SPRMA970-12	KP648683
Thomisidae	*Ozyptila gertschi*	ERSPI359-09	GU682487
Thomisidae	*Ozyptila pacifica*	SPRMA644-10	JF887103
Thomisidae	*Ozyptila praticola*	ARONT138-09	GU682617
Thomisidae	*Ozyptila sincera canadensis*	SPRMA771-12	KP650846
Thomisidae	*Synema globosum*	GBCH11025-13	JN817246
Thomisidae	*Tmarus angulatus*	ARONT185-09	GU682620
Thomisidae	*Tmarus angulatus*	JSARA083-11	KP651599
Thomisidae	*Tmarus piger*	GBCH11023-13	JN817248
Thomisidae	*Tmarus stellio*	GBCH11022-13	JN817249
Thomisidae	*Xysticus benefactor*	SPRMA173-10	HQ580793
Thomisidae	*Xysticus bicuspis*	ERSPI290-09	GU682533
Thomisidae	*Xysticus britcheri*	JDTGS014-09	GU679900
Thomisidae	*Xysticus californicus*	SPRMA648-10	JF887105
Thomisidae	*Xysticus canadensis*	CNPAC645-13	KM833331
Thomisidae	*Xysticus chippewa*	SSBAA2020-12	KM837148
Thomisidae	*Xysticus cristatus*	SPRMA647-10	JF887104
Thomisidae	*Xysticus cunctator*	SPRMA862-12	KP647331
Thomisidae	*Xysticus deichmanni*	KKCHE1078-09	GU683755
Thomisidae	*Xysticus discursans*	ARONT511-10	HQ924403
Thomisidae	*Xysticus durus*	KKCHE1079-09	GU683594

Thomisidae	*Xysticus elegans*	ARONT382-09	GU682927
Thomisidae	*Xysticus ellipticus*	SPICH116-09	GU683801
Thomisidae	*Xysticus ellipticus*	SSWLE1825-13	KM832669
Thomisidae	*Xysticus emertoni*	ARONT314-09	GU682874
Thomisidae	*Xysticus ephippiatus*	GBCH11021-13	JN817250
Thomisidae	*Xysticus ferox*	ARONT467-10	HM880619
Thomisidae	*Xysticus ferox*	SSJAB1364-13	KM832710
Thomisidae	*Xysticus funestus*	ARONT334-09	GU682875
Thomisidae	*Xysticus gulosus*	SPRMA649-10	JN310305
Thomisidae	*Xysticus insulicola*	GBCH11020-13	JN817251
Thomisidae	*Xysticus labradorensis*	KKCHE1075-09	GU683754
Thomisidae	*Xysticus locuples*	SPRMA947-12	KP647328
Thomisidae	*Xysticus luctans*	ERSPI257-09	GU682560
Thomisidae	*Xysticus luctuosus*	SPICH118-09	GU683803
Thomisidae	*Xysticus luctuosus*	ARSO275-08	KM840130
Thomisidae	*Xysticus montanensis*	SPRMA652-10	JF887107
Thomisidae	*Xysticus montanensis*	SPRMA650-10	KP645629
Thomisidae	*Xysticus nigromaculatus*	TWSC036-07	KF369066
Thomisidae	*Xysticus obscurus*	SPICH1009-09	GU684734
Thomisidae	*Xysticus pellax*	ARONT170-09	GU682618
Thomisidae	*Xysticus pellax*	CNPPE2323-12	KJ167178
Thomisidae	*Xysticus pellax*	CNPPJ1366-12	KJ207934
Thomisidae	*Xysticus pretiosus*	SPRMA835-12	KP656759
Thomisidae	*Xysticus punctatus*	ERSPI297-09	GU682529
Thomisidae	*Xysticus triangulosus*	KKCHE1086-09	GU683600
Thomisidae	*Xysticus triangulosus*	SPICH1007-09	GU684731
Thomisidae	*Xysticus triguttatus*	SPICH128-09	GU683802
Thomisidae	*Xysticus winnipegensis*	ARONT509-10	HQ924402
Trachelidae	*Meriola californica*	CNGIF086-12	KM835023
Trachelidae	*Trachelas japonicus*	GBCH11046-13	JN817224
Trachelidae	*Trachelas tranquillus*	JSARA090-11	KP646050
Uloboridae	*Hyptiotes gertschi*	GBCH4033-09	FJ607566
Uloboridae	*Octonoba sinensis*	GBCH11190-13	JN817080
Uloboridae	*Octonoba yesoensis*	GBCH11189-13	JN817081
Uloboridae	*Uloborus diversus*	GBCH3235-09	FJ525329
Uloboridae	*Uloborus glomosus*	ARONT719-10	HQ924574
Zoropsidae	*Takeoa nishimurai*	GBCH11055-13	JN817215
**Holothyrida**			
Allothyridae	*Allothyrus sp. LamingtonNP-QMS95173*	CYTC5876-15	KC769586
**Ixodida**			
Argasidae	*Argas africolumbae*	GBCH11499-13	JQ665720
Argasidae	*Carios capensis*	GBCH0011-06	AB075953
Argasidae	*Ornithodoros moubata*	GBCH1676-06	NC_004357
Argasidae	*Ornithodoros porcinus*	GBCH0032-06	AB105451

Ixodidae	*Amblyomma americanum*	GBCH1239-06	DQ168131
Ixodidae	*Amblyomma cajennense*	GBCH10808-13	NC_020333
Ixodidae	*Amblyomma maculatum*	MIONB499-11	KM839245
Ixodidae	*Amblyomma sphenodonti*	GBCH1453-06	DQ507239
Ixodidae	*Amblyomma triguttatum*	GBCH0033-06	AB113317
Ixodidae	*Amblyomma variegatum*	GBCH5183-10	GU062743
Ixodidae	*Bothriocroton hydrosauri*	GBCH4088-09	FJ584425
Ixodidae	*Dermacentor marginatus*	GBCH0078-06	AF132828
Ixodidae	*Dermacentor nitens*	GBCH0351-06	AY008679
Ixodidae	*Dermacentor reticulatus*	GBCH0079-06	AF132829
Ixodidae	*Dermacentor rhinocerinus*	GBCH0080-06	AF132830
Ixodidae	*Dermacentor variabilis*	GBCH0081-06	AF132831
Ixodidae	*Haemaphysalis flava*	GBCH0012-06	AB075954
Ixodidae	*Haemaphysalis formosensis*	GBCH11572-13	JX573135
Ixodidae	*Haemaphysalis humerosa*	GBCH0069-06	AF132819
Ixodidae	*Haemaphysalis humerosa*	GBCH11569-13	JX573138
Ixodidae	*Haemaphysalis longicornis*	GBCH0070-06	AF132820
Ixodidae	*Haemaphysalis parva*	GBCH10806-13	NC_020335
Ixodidae	*Haemaphysalis*	GBCH11570-13	JX573137
Ixodidae	*Hyalomma aegyptium*	GBCH0071-06	AF132821
Ixodidae	*Hyalomma detritum*	GBCH4210-09	EU827695
Ixodidae	*Hyalomma dromedarii*	GBCH0259-06	AJ437069
Ixodidae	*Hyalomma dromedarii*	GBCH0270-06	AJ437080
Ixodidae	*Hyalomma lusitanicum*	GBCH4192-09	EU827713
Ixodidae	*Hyalomma marginatum*	GBCH0073-06	AF132823
Ixodidae	*Hyalomma marginatum*	GBCH4212-09	EU827693
Ixodidae	*Hyalomma truncatum*	GBCH0074-06	AF132824
Ixodidae	*Hyalomma truncatum*	GBCH0275-06	AJ437085
Ixodidae	*Ixodes acutitarsus*	GBCH0031-06	AB105166
Ixodidae	*Ixodes arboricola*	GBCH11602-13	JX394195
Ixodidae	*Ixodes asanumai*	GBCH0057-06	AB231674
Ixodidae	*Ixodes cornuatus*	GBCH6981-13	HM545823
Ixodidae	*Ixodes granulatus*	GBCH0056-06	AB231673
Ixodidae	*Ixodes hexagonus*	CYTC4101-12	AF081828
Ixodidae	*Ixodes holocyclus*	GBCH0013-06	AB075955
Ixodidae	*Ixodes lividus*	GACAC525-12	GU124743
Ixodidae	*Ixodes monospinosus*	GBCH0055-06	AB231672
Ixodidae	*Ixodes ovatus*	GBCH0053-06	AB231670
Ixodidae	*Ixodes pavlovskyi*	GBCH11665-13	JX288763
Ixodidae	*Ixodes persulcatus*	GBCH11664-13	JX288764
Ixodidae	*Ixodes philipi*	GBCH0049-06	AB231666
Ixodidae	*Ixodes redikorzevi*	GBCH11597-13	JX394200
Ixodidae	*Ixodes ricinus*	GBCH11333-13	NC_018369
Ixodidae	*Ixodes scapularis*	ETBM003-11	JQ350479

Ixodidae	*Ixodes turdus*	GBCH0051-06	AB231668
Ixodidae	*Ixodes uriae*	GBCH0030-06	AB087746
Ixodidae	*Ixodes vespertilionis*	GBCH0050-06	AB231667
Ixodidae	*Nosomma monstrosum*	GBCH0082-06	AF132832
Ixodidae	*Rhipicephalus annulatus*	GBCH0075-06	AF132825
Ixodidae	*Rhipicephalus appendiculatus*	GBCH0083-06	AF132833
Ixodidae	*Rhipicephalus compositus*	GBCH0084-06	AF132834
Ixodidae	*Rhipicephalus decoloratus*	GBCH0076-06	AF132826
Ixodidae	*Rhipicephalus evertsi*	GBCH0085-06	AF132835
Ixodidae	*Rhipicephalus evertsi*	GBCH0086-06	AF132836
Ixodidae	*Rhipicephalus geigyi*	GBCH0352-06	AY008680
Ixodidae	*Rhipicephalus maculatus*	GBCH0353-06	AY008681
Ixodidae	*Rhipicephalus microplus*	GBCH0077-06	AF132827
Ixodidae	*Rhipicephalus pravus*	GBCH0087-06	AF132837
Ixodidae	*Rhipicephalus pulchellus*	GBCH0354-06	AY008682
Ixodidae	*Rhipicephalus pumilio*	GBCH0356-06	AY008684
Ixodidae	*Rhipicephalus punctatus*	GBCH0088-06	AF132838
Ixodidae	*Rhipicephalus sanguineus*	CYTC4102-12	AF081829
Ixodidae	*Rhipicephalus sanguineus*	GBCH0089-06	AF132839
Ixodidae	*Rhipicephalus sanguineus*	GBCH11623-13	JX416325
Ixodidae	*Rhipicephalus simus*	GBCH0090-06	AF132840
Ixodidae	*Rhipicephalus turanicus*	GBCH0091-06	AF132841
Ixodidae	*Rhipicephalus turanicus*	GBCH12084-13	KF251020
Nuttalliellidae		GBCH11403-13	NC_019663
**Mesostigmata**			
Ameroseiidae		CHACA968-10	HM405834
Ameroseiidae		SSPAC12226-13	KM824656
Ameroseiidae		CNPAF902-13	KM830755
Ameroseiidae		SSWLE2881-13	KM833090
Ameroseiidae		SSPAC2158-13	KM839086
Ameroseiidae		JSAUG1857-12	KP979322
Ameroseiidae		PHAUG1672-11	KP979162
Arctacaridae	*Arctacarus*	CHACB477-10	HQ558615
Arctacaridae		SSJAA2021-13	KM827181
Ascidae	*Antennoseius*	CHACA967-10	HM405833
Ascidae	*Antennoseius*	CHACB412-10	HQ558572
Ascidae	*Antennoseius*	CHACA302-08	JX837595
Ascidae	*Antennoseius*	MYMCA452-11	JX838669
Ascidae	*Arctoseius*	CHACC161-10	JX837921
Ascidae	*Arctoseius*	MIONB410-10	KP979120
Ascidae		CHACB926-10	HM907354
Ascidae		CHACB071-10	HQ558379
Ascidae		CHACB357-10	HQ558537
Ascidae		CHACB533-10	HQ558650

Ascidae		CHACB787-10	HQ941485
Ascidae		CHACA498-09	JX833842
Ascidae		MYMCB874-11	JX834096
Ascidae		MYMCE699-12	JX834216
Ascidae		MYMCG522-12	JX834553
Ascidae		MYMCA1025-11	JX834701
Ascidae		CHACA438-09	JX835137
Ascidae		MYMCE278-12	JX835480
Ascidae		CHACA234-08	JX835532
Ascidae		CHACA163-08	JX835847
Ascidae		MYMCA873-11	JX836365
Ascidae		MYMCG367-12	JX836530
Ascidae		MYMCF861-12	JX836769
Ascidae		MYMCA194-11	JX836930
Ascidae		MYMCF441-12	JX837289
Ascidae		SSBAF2274-13	KM827087
Ascidae		CNBAA463-12	KM827471
Ascidae		SSBAD3976-12	KM827604
Ascidae		SSPAB4020-13	KM830099
Ascidae		CNRMC1516-12	KM831744
Ascidae		SSPAA8234-13	KM832051
Ascidae		CNPAC507-13	KM832420
Ascidae		SSPAA9197-13	KM832808
Ascidae		SSBAD3070-12	KM833994
Ascidae		CNEIA2612-12	KM834069
Ascidae		CNJAH781-12	KM836338
Ascidae		CNJAB1038-12	KM836920
Ascidae		CNJAG1840-12	KM837421
Ascidae		SSPAA7996-13	KM837816
Ascidae		SSPAA2270-13	KM838026
Ascidae		SSPAC2153-13	KM838722
Ascidae		CNEIH065-13	KM839713
Ascidae		SSPAB3993-13	KM839824
Ascidae		SMTPB671-13	KP979129
Ascidae		JSJUN2325-12	KP979197
Ascidae		SMTPB7907-13	KP979183
Ascidae		SMTPB14723-13	KP979118
Ascidae		SMTPB5054-13	KP979247
Ascidae		CHACC117-10	KP979114
Ascidae		SMTPB5188-13	KP979121
Ascidae		JSOIE075-12	KP979240
Blattisociidae	*Cheiroseius*	MYMCB949-11	JX834177
Blattisociidae	*Cheiroseius*	MYMCC796-11	JX835411
Blattisociidae	*Cheiroseius*	MYMCF323-12	JX835904

Blattisociidae	*Cheiroseius*	CHACA485-09	JX836234
Blattisociidae	*Cheiroseius*	MYMCA326-11	JX836462
Blattisociidae	*Cheiroseius*	CHACA213-08	JX837403
Blattisociidae	*Cheiroseius*	MYMCC793-11	JX838448
Blattisociidae	*Cheiroseius*	SSJAE1613-13	KM827232
Blattisociidae	*Cheiroseius*	CNBAI477-13	KM832411
Blattisociidae	*Cheiroseius*	SSPAB8731-13	KM834489
Blattisociidae	*Cheiroseius*	MIONB225-10	KP979143
Blattisociidae	*Cheiroseius*	JSOIE085-12	KP979194
Blattisociidae	*Cheiroseius*	MIONB158-10	KP979144
Blattisociidae	*Cheiroseius*	JSOIE123-12	KP979200
Blattisociidae	*Cheiroseius*	MIAUS024-12	KP979324
Blattisociidae		MYMCG651-12	JX833775
Blattisociidae		MYMCG067-12	JX835187
Blattisociidae		MYMCE068-12	JX836250
Blattisociidae		MYMCA1177-11	JX838726
Blattisociidae		CNPPI1837-12	KJ445002
Blattisociidae		CNSLJ430-12	KM824328
Blattisociidae		CNEIG1588-13	KM827237
Blattisociidae		CNPAC546-13	KM828205
Blattisociidae		CNEIG1589-13	KM831960
Blattisociidae		SSBAC2380-12	KM836029
Blattisociidae		MIONB009-10	KP979273
Dermanyssidae	*Dermanyssus longipes*	MIONB534-12	KP979215
Digamasellidae		CHACB1142-10	HM907231
Digamasellidae		CHACB359-10	HQ558539
Digamasellidae		MYMCC413-11	JX835572
Digamasellidae		CHACB990-10	JX836283
Digamasellidae		MYMCA345-11	JX837870
Digamasellidae		CNPPF1036-12	KJ167283
Digamasellidae		CNJAC1513-12	KM824108
Digamasellidae		CNEIA2575-12	KM824117
Digamasellidae		SSBAD3086-12	KM825070
Digamasellidae		CNSLC560-12	KM825073
Digamasellidae		CNGLF134-13	KM825605
Digamasellidae		SSPAB3130-13	KM826271
Digamasellidae		CNJAA875-12	KM826750
Digamasellidae		CNGLC073-13	KM826931
Digamasellidae		SSWLA2270-13	KM826973
Digamasellidae		CNGLA015-13	KM827176
Digamasellidae		CNPAL453-13	KM829149
Digamasellidae		SSJAC198-13	KM829496
Digamasellidae		SSPAC2155-13	KM829665
Digamasellidae		SSPAC7890-13	KM830989

Digamasellidae		CNBPC214-12	KM831342
Digamasellidae		CNPAC362-13	KM831729
Digamasellidae		CNPAC553-13	KM831811
Digamasellidae		CNGLF2452-13	KM833394
Digamasellidae		SSWLD3516-13	KM833448
Digamasellidae		SSWLD4483-13	KM833494
Digamasellidae		CNJAC1572-12	KM834272
Digamasellidae		CNJAC1543-12	KM835154
Digamasellidae		SSJAF3157-13	KM835907
Digamasellidae		SSJAC202-13	KM836033
Digamasellidae		CNGLF2449-13	KM836229
Digamasellidae		SSPAC2190-13	KM836261
Digamasellidae		CNGLF2730-13	KM836501
Digamasellidae		CNGLC076-13	KM836678
Digamasellidae		CNPAE440-13	KM837113
Digamasellidae		SSPAA9725-13	KM837919
Digamasellidae		CNPAC541-13	KM838250
Digamasellidae		CNPAC508-13	KM838785
Digamasellidae		CNJAC1488-12	KM840449
Digamasellidae		CNPAC365-13	KM840642
Digamasellidae		CNGLF2728-13	KM840692
Digamasellidae		NCCE072-11	KP979228
Digamasellidae		PHAPR1305-11	KP979342
Digamasellidae		PHAPR1304-11	KP979218
Digamasellidae		PHJUN3841-11	KP979125
Digamasellidae		SMTPB14724-13	KP979308
Digamasellidae		SMTPB14682-13	KP979233
Dinychidae		CHACA044-08	JX834361
Dinychidae		MYMCE584-12	JX834565
Dinychidae		CHACA243-08	JX836003
Dinychidae		MYMCF589-12	JX838647
Dinychidae		CNPAL906-13	KM827245
Dinychidae		CNPAC515-13	KM827355
Dinychidae		SSEIA7691-13	KM829074
Dinychidae		SSEIA7687-13	KM830015
Dinychidae		CNPAC545-13	KM830122
Laelapidae	*Cosmolaelaps*	CHACA998-10	HM405856
Laelapidae	*Haemogamasus ambulans*	MYMCC547-11	JX835676
Laelapidae	*Hypoaspis aculeifer*	GACAC136-12	FM210170
Laelapidae	*Hypoaspis*	CHACA972-10	HM405837
Laelapidae	*Hypoaspis*	CHACA583-09	JX835348
Laelapidae	*Hypoaspis*	MYMCA323-11	JX837421
Laelapidae	*Hypoaspis*	MIONB289-10	KP979169
Laelapidae	*Ololaelaps*	MYMCF260-12	JX833993

Laelapidae	*Ololaelaps*	MYMCE570-12	JX836779
Laelapidae	*Ololaelaps*	MYMCB078-11	JX837120
Laelapidae	*Ololaelaps*	MYMCE552-12	JX837773
Laelapidae	*Ololaelaps*	MYMCC103-11	JX838224
Laelapidae	*Ololaelaps*	MYMCC179-11	JX838511
Laelapidae	*Ololaelaps*	SSPAA7962-13	KM834536
Laelapidae		CHACA960-10	HM405829
Laelapidae		CHACA971-10	HM405836
Laelapidae		CHACB955-10	HM907375
Laelapidae		MYMCE175-12	JX835078
Laelapidae		MYMCF294-12	JX835261
Laelapidae		MYMCC269-11	JX837125
Laelapidae		MYMCE878-12	JX838442
Laelapidae		MYMCE451-12	JX838735
Laelapidae		SSPAA7819-13	KM826237
Laelapidae		SSEIB7673-13	KM832468
Laelapidae		SSWLA2262-13	KM833110
Laelapidae		CNRME4767-12	KM833768
Laelapidae		SSPAA6684-13	KM837690
Laelapidae		CNGLD016-13	KM837895
Laelapidae		CNWLE2589-13	KM838753
Laelapidae		SMTPB5830-13	KP979317
Macrochelidae	*Macrocheles*	CNPPA4153-12	KJ086359
Macrochelidae	*Macrocheles*	MIONB478-11	KM828634
Macrochelidae	*Macrocheles*	MIONB107-10	KP979340
Macrochelidae	*Macrocheles*	MIONB108-10	KP979203
Macrochelidae	*Macrocheles*	MIONB122-10	KP979285
Macrochelidae	*Macrocheles*	MIONB123-10	KP979187
Macrochelidae	*Macrocheles*	MIONB336-10	KP979156
Macrochelidae	*Macrocheles*	MIONB419-10	KP979180
Macrochelidae	*Macrocheles*	JSOIE070-12	KP979269
Macrochelidae		MYMCE204-12	JX834758
Macrochelidae		SSEIB4228-13	KM834316
Macrochelidae		SSEIB5795-13	KM836965
Macrochelidae		SSWLA1882-13	KM839835
Macrochelidae		ASAMT013-12	KP979316
Melicharidae	*Proctolaelaps*	MYMCC893-11	JX834791
Melicharidae	*Proctolaelaps*	MYMCE311-12	JX835529
Melicharidae	*Proctolaelaps*	CNPPA4087-12	KJ083789
Melicharidae	*Proctolaelaps*	CNPPA4082-12	KJ092971
Melicharidae	*Proctolaelaps*	CNPPA4129-12	KJ165242
Melicharidae	*Proctolaelaps*	CNEIG1585-13	KM824226
Melicharidae	*Proctolaelaps*	SSJAE3151-13	KM824732
Melicharidae	*Proctolaelaps*	SSPAB7978-13	KM825580

Melicharidae	*Proctolaelaps*	SSBAD4744-13	KM826037
Melicharidae	*Proctolaelaps*	CNPAD550-13	KM826495
Melicharidae	*Proctolaelaps*	CNEIG1584-13	KM827861
Melicharidae	*Proctolaelaps*	CNJAF1991-12	KM828629
Melicharidae	*Proctolaelaps*	SSPAC7842-13	KM830032
Melicharidae	*Proctolaelaps*	CNPCE166-13	KM830734
Melicharidae	*Proctolaelaps*	SSBAE3916-13	KM834744
Melicharidae	*Proctolaelaps*	CNSLF662-12	KM834804
Melicharidae	*Proctolaelaps*	CNRME4793-12	KM837505
Melicharidae	*Proctolaelaps*	SSWLD4499-13	KM837847
Melicharidae	*Proctolaelaps*	SSWLF3583-13	KM838195
Melicharidae	*Proctolaelaps*	CNRMC1658-12	KM839466
Microgyniidae		CHACB067-10	HQ558375
Microgyniidae		CNGLE039-13	KM830249
Microgyniidae		SSBAE929-13	KM835462
Microgyniidae		CNGLF129-13	KM839854
Ologamasidae	*Gamasellus*	CHACA804-09	JX834214
Ologamasidae	*Stylochyrus rarior*	GBCH5106-10	GQ927176
Ologamasidae		CHACB041-10	HQ558354
Ologamasidae		CHACB479-10	HQ558617
Ologamasidae		CHACA096-08	JX834808
Ologamasidae		MYMCC318-11	JX837399
Ologamasidae		SSWLC4139-13	KM824733
Ologamasidae		SSWEE018-13	KM826049
Ologamasidae		SSWLA676-13	KM836814
Ologamasidae		SSWLC1159-13	KM838616
Ologamasidae		CHACA548-09	KP979349
Ologamasidae		MIONB019-10	KP979136
Oplitidae		MIONB328-10	KP979276
Pachylaelapidae	*Pachylaelaps*	CHACB528-10	HQ558646
Pachylaelapidae		CHACA517-09	JX837563
Pachylaelapidae		RBINA5685-13	KP979258
Parasitidae		GBCH7735-13	FJ577976
Parasitidae		MIONB014-10	HM887564
Parasitidae		MIONB038-10	HM887575
Parasitidae		CHACB1141-10	HM907230
Parasitidae		ARCN159-10	HQ924364
Parasitidae		ARCN163-10	HQ924365
Parasitidae		CHACA412-09	JX833970
Parasitidae		CHACA092-08	JX834035
Parasitidae		MYMCE263-12	JX834329
Parasitidae		MYMCB222-11	JX834740
Parasitidae		MYMCB426-11	JX837039
Parasitidae		CHACA1148-10	JX838501

Parasitidae		SSWLA5269-13	KM824418
Parasitidae		CNWBE709-13	KM825696
Parasitidae		CNJAC1441-12	KM825910
Parasitidae		SSJAF5635-13	KM826485
Parasitidae		CNRMC1510-12	KM826777
Parasitidae		CNEIA2553-12	KM827019
Parasitidae		SSBAD4772-13	KM827965
Parasitidae		SSPAA7830-13	KM828118
Parasitidae		SSBAD4719-13	KM828580
Parasitidae		CNWBE664-13	KM829042
Parasitidae		CNRMF4054-12	KM829707
Parasitidae		SSBAF4461-13	KM830260
Parasitidae		SSBAF4454-13	KM830830
Parasitidae		CNSLJ030-12	KM831122
Parasitidae		CNSLF653-12	KM832387
Parasitidae		SSJAF2633-13	KM832439
Parasitidae		CNWLE2578-13	KM832826
Parasitidae		CNRMD2700-12	KM833845
Parasitidae		CNRMD2702-12	KM833933
Parasitidae		CNEIA2609-12	KM834257
Parasitidae		CNWLE2566-13	KM834931
Parasitidae		SSPAB7516-13	KM835018
Parasitidae		SSPAB8000-13	KM835256
Parasitidae		CNEIH064-13	KM835965
Parasitidae		MIONB455-11	KM837107
Parasitidae		SSBAB2864-12	KM839154
Parasitidae		SSBAF4474-13	KM839440
Parasitidae		CNRME4707-12	KM839526
Parasitidae		SSBAD3063-12	KM839548
Parasitidae		CNWLF136-12	KM840126
Parasitidae		CNPAM237-13	KM840306
Parasitidae		SSBAD4721-13	KM840382
Parasitidae		SSEIB7660-13	KM840479
Parasitidae		CNPAJ043-13	KM840714
Parasitidae		TDWGB354-10	KP979244
Parasitidae		RBINA5623-13	KP979167
Parasitidae		JSOIE100-12	KP979353
Parasitidae		PHMTT635-10	KP979168
Parasitidae		MIONB212-10	KP979222
Parasitidae		MIONB420-10	KP979275
Parasitidae		MIONB431-10	KP979330
Parasitidae		MIONB213-10	KP979297
Paratennulidae		MIONB348-10	KP979211
Phytoseiidae	*Amblyseius degenerans*	GACAC133-12	FM210119

Phytoseiidae	*Amblyseius eharai*	GBCH11752-13	JX080338
Phytoseiidae	*Amblyseius eharai*	GBCH11746-13	JX080344
Phytoseiidae	*Amblyseius herbicolus*	GBCH11764-13	JX080326
Phytoseiidae	*Amblyseius largoensis*	GBCH11742-13	JX080348
Phytoseiidae	*Metaseiulus occidentalis*	GBCH3756-09	EF221760
Phytoseiidae	*Phytoseiulus persimilis*	GBCH4472-10	NC_014049
Phytoseiidae	*Typhlodromus exhilaratus*	GBCH5484-11	JF279183
Phytoseiidae	*Typhlodromus pyri*	GBCH5500-11	JF279167
Phytoseiidae	*Typhlodromus pyri*	GBCH5497-11	JF279170
Phytoseiidae	*Typhlodromus pyri*	GBCH5493-11	JF279174
Phytoseiidae	*Typhlodromus pyri*	GBCH5491-11	JF279176
Phytoseiidae	*Typhlodromus pyri*	GBCH5488-11	JF279179
Phytoseiidae	*Typhlodromus*	CNGII078-13	KM839839
Phytoseiidae		CHACA988-10	HM405850
Phytoseiidae		CHACB1168-10	HM907247
Phytoseiidae		CHACB1194-10	HM907269
Phytoseiidae		CHACC027-10	HM907428
Phytoseiidae		CHACB147-10	HQ558430
Phytoseiidae		GBCH7582-13	JQ864550
Phytoseiidae		GBCH11784-13	JX080306
Phytoseiidae		GBCH11782-13	JX080308
Phytoseiidae		GBCH11781-13	JX080309
Phytoseiidae		GBCH11776-13	JX080314
Phytoseiidae		GBCH11775-13	JX080315
Phytoseiidae		GBCH11774-13	JX080316
Phytoseiidae		GBCH11772-13	JX080318
Phytoseiidae		GBCH11769-13	JX080321
Phytoseiidae		GBCH11766-13	JX080324
Phytoseiidae		MYMCE177-12	JX834284
Phytoseiidae		MYMCE001-12	JX834623
Phytoseiidae		MYMCB085-11	JX834633
Phytoseiidae		MYMCC662-11	JX835047
Phytoseiidae		MYMCA090-11	JX835107
Phytoseiidae		MYMCA391-11	JX835204
Phytoseiidae		MYMCE004-12	JX835405
Phytoseiidae		MYMCG510-12	JX835547
Phytoseiidae		MYMCA631-11	JX835565
Phytoseiidae		MYMCE322-12	JX836575
Phytoseiidae		MYMCF081-12	JX836741
Phytoseiidae		MYMCF484-12	JX837219
Phytoseiidae		MYMCA316-11	JX837810
Phytoseiidae		MYMCB267-11	JX838098
Phytoseiidae		MYMCA998-11	JX838192
Phytoseiidae		MYMCC664-11	JX838277

Phytoseiidae	MYMCC926-11	JX838573
Phytoseiidae	MYMCG206-12	JX838668
Phytoseiidae	CNPPB2354-12	KJ086108
Phytoseiidae	CNPPD2658-12	KJ090645
Phytoseiidae	CNPPF583-12	KJ163438
Phytoseiidae	CNPPI1273-12	KJ444719
Phytoseiidae	CNRMA1070-12	KM824728
Phytoseiidae	CNGIE399-12	KM826359
Phytoseiidae	SSWEE026-13	KM826667
Phytoseiidae	SSWLD8287-13	KM827311
Phytoseiidae	SSWLE3964-13	KM827843
Phytoseiidae	SSBAD3066-12	KM827877
Phytoseiidae	SSWLB5367-13	KM828268
Phytoseiidae	SSPAB4026-13	KM829125
Phytoseiidae	SSBAD4047-12	KM829999
Phytoseiidae	SSPAC6762-13	KM830564
Phytoseiidae	CNPAB274-13	KM830580
Phytoseiidae	SSPAB3991-13	KM831980
Phytoseiidae	CNRME4745-12	KM833259
Phytoseiidae	SSJAE3134-13	KM833860
Phytoseiidae	SSPAA8154-13	KM833998
Phytoseiidae	CNGLB019-13	KM834020
Phytoseiidae	CNBPC226-12	KM834954
Phytoseiidae	CNGIA134-12	KM835603
Phytoseiidae	SSPAB4006-13	KM836652
Phytoseiidae	SSWLF2217-13	KM836661
Phytoseiidae	CNEIE1937-12	KM836834
Phytoseiidae	SSPAC7874-13	KM836991
Phytoseiidae	SSBAD3074-12	KM837202
Phytoseiidae	SSWLB5366-13	KM837593
Phytoseiidae	CNGLF143-13	KM838074
Phytoseiidae	CNRMF3271-12	KM838329
Phytoseiidae	CNRMC1662-12	KM839189
Phytoseiidae	SSWLF3055-13	KM839218
Phytoseiidae	CNJAA872-12	KM839318
Phytoseiidae	SSWLD8247-13	KM839432
Phytoseiidae	CNPAJ177-13	KM840542
Phytoseiidae	MIONB149-10	KP979155
Phytoseiidae	JSAUG1866-12	KP979354
Phytoseiidae	JSAUG1868-12	KP979278
Phytoseiidae	JSOIE067-12	KP979337
Phytoseiidae	JSOIE099-12	KP979347
Phytoseiidae	SMTPB22979-13	KP979232
Phytoseiidae	SMTPB2349-13	KP979311

Phytoseiidae		SMTPB1063-13	KP979243
Phytoseiidae		SMTPB20520-13	KP979298
Phytoseiidae		SMTPB12837-13	KP979166
Phytoseiidae		SMTPB12688-13	KP979277
Phytoseiidae		SMTPB12684-13	KP979122
Sejidae		SSEIB4209-13	KM825826
Sejidae		CNJAC1110-12	KM827198
Trachytidae	*Trachytes*	MYMCC661-11	JX837725
Trematuridae	*Nenteria eulaelaptis*	BIOAC205-11	JN992135
Trematuridae	*Nenteria moseri*	BIOAC195-11	JN992136
Trematuridae	*Trichouropoda idahoensis*	BIOAC212-11	JN992169
Trematuridae	*Trichouropoda polytricha*	BIOAC216-11	JN992193
Trematuridae	*Trichouropoda tegucigalpae*	BIOAC203-11	JN992201
Trematuridae		CHACB066-10	HQ558374
Trematuridae		MYMCE666-12	JX834265
Trematuridae		MYMCC243-11	JX836507
Trematuridae		MYMCB773-11	JX838526
Trematuridae		SSEIC4168-13	KM828859
Trematuridae		CNJAF1913-12	KM830660
Trematuridae		PHOCT911-11	KP979282
Urodinychidae	*Uroobovella sp. 1WK*	BIOAC032-11	JN992056
Urodinychidae	*Uroobovella sp. 2WK*	BIOAC024-11	JN992081
Urodinychidae	*Uroobovella sp. 3WK*	BIOAC006-11	JN992099
Urodinychidae	*Uroobovella sp. 4*	BIOAC267-11	JN992103
Urodinychidae	*Uroobovella vinicolora*	BIOAC260-11	JN992250
Varroidae	*Varroa destructor*	GBCH5621-13	GQ379070
Veigaiidae		MYMCG096-12	JX833869
Veigaiidae		MYMCF612-12	JX837587
Veigaiidae		MYMCF718-12	JX838334
Zerconidae		CHACB1036-10	HM907169
Zerconidae		CHACB1110-10	HM907205
Zerconidae		CHACB906-10	HM907336
Zerconidae		CHACB077-10	HQ558384
Zerconidae		CHACB163-10	HQ558445
Zerconidae		MIONB068-10	HQ575084
Zerconidae		CHACC167-10	JX833963
Zerconidae		MYMCG373-12	JX834981
Zerconidae		CHACB927-10	JX836256
Zerconidae		MYMCA198-11	JX837122
Zerconidae		MYMCE090-12	JX838101
Zerconidae		SSJAC1508-13	KM828949
Zerconidae		SSJAA1810-13	KM834547
Zerconidae		SSWLC4141-13	KM839305
**Opilioacarida**			

Opilioacaridae	*Neocarus*	MXBAC095-11	KP979332
**Opiliones**			
Biantidae	*Metabiantes sp.*	GACAC637-12	JF786450
Ceratolasmatidae	*Acuclavella*	GBCH12098-13	KF181736
Ceratolasmatidae	*Acuclavella*	GBCH12096-13	KF181738
Ceratolasmatidae	*Acuclavella cf. quattuor SDSU OP*	GBCH12104-13	KF181730
Ceratolasmatidae	*Acuclavella cf. quattuor SDSU OP*	GBCH12102-13	KF181732
Ceratolasmatidae	*Acuclavella cosmetoides*	GBCH4487-10	GQ870646
Ceratolasmatidae	*Acuclavella merickeli*	GBCH12100-13	KF181734
Ceratolasmatidae	*Acuclavella sheari*	GBCH12091-13	KF181743
Ceratolasmatidae	*Acuclavella sp. 3 MCH-2009*	GBCH4485-10	GQ870648
Epedanidae	*Tithaeus sp. DNA104062_1*	GACAC624-12	JF786437
Epedanidae	*Tithaeus sp. DNA104068*	GACAC626-12	JF786439
Gonyleptidae	*Acutisoma longipes*	GACAC628-12	JF786441
Icaleptidae	*Icaleptes sp.*	GACAC644-12	JF786457
Kimulidae	*Kimula goodnightiorum*	GACAC645-12	JF786458
Neogoveidae	*Brasilogovea sp. DNA101665*	GACAC617-12	JF786414
Neogoveidae	*Huitaca sp. DNA101671*	GACAC601-12	JF786398
Neogoveidae	*Huitaca sp. DNA101681*	GACAC600-12	JF786397
Neogoveidae	*Huitaca sp. DNA101683*	GACAC599-12	JF786396
Neogoveidae	*Huitaca sp. MCZDNA101407*	GBCH3699-09	DQ518129
Neogoveidae	*Metagovea sp. DNA101680*	GACAC603-12	JF786400
Neogoveidae	*Metagovea sp. DNA101685*	GACAC606-12	JF786403
Neogoveidae	*Metagovea sp. DNA102151m*	GACAC605-12	JF786402
Neogoveidae	*Metasiro americanus*	GBCH3689-09	DQ825645
Neogoveidae	*Metasiro americanus*	GACAC596-12	JF786393
Neogoveidae	*Neogovea sp. DNA104823*	GACAC608-12	JF786405
Neogoveidae	*Neogovea sp. DNA105825*	GACAC610-12	JF786407
Neogoveidae	*Neogovea sp. DNA105826*	GACAC611-12	JF786408
Neogoveidae	*Neogovea sp. MCZDNA101409*	GBCH1818-07	DQ825646
Neogoveidae	*Neogovea virginie*	GACAC609-12	JF786406
Neogoveidae	*Paragovia gabonica*	GACAC614-12	JF786411
Neogoveidae	*Paragovia sironoides*	GBCH1695-07	DQ518131
Neogoveidae	*Paragovia sironoides*	GBCH1815-07	DQ825649
Neogoveidae	*Paragovia sironoides*	GBCH1814-07	DQ825650
Neogoveidae	*Paragovia sp. DNA104615*	GACAC613-12	JF786410
Neogoveidae	*Paragovia sp. DNA104618*	GACAC615-12	JF786412
Neogoveidae	*Paragovia sp. DNA104619*	GACAC612-12	JF786409
Neogoveidae	*Paragovia sp. DNA105671*	GACAC616-12	JF786413
Neogoveidae	*Paragovia sp. SLB-2006*	GBCH1816-07	DQ825648
Pettalidae	*Aoraki calcarobtusa*	GBCH3170-08	EU673667
Pettalidae	*Chileogovea oedipus*	GBCH3176-08	EU673661
Pettalidae	*Chileogovea sp. MCZDNA101490*	GBCH1829-07	DQ825635
Pettalidae	*Karripurcellia harveyi*	GBCH3708-09	DQ518107

Pettalidae	*Neopurcellia florensis*	GBCH2558-08	DQ992325
Pettalidae	*Neopurcellia forsteri*	GBCH3705-09	DQ518110
Pettalidae	*Neopurcellia minutissima*	GBCH2557-08	DQ992326
Pettalidae	*Neopurcellia minutissima*	GBCH2555-08	DQ992328
Pettalidae	*Neopurcellia salmoni*	GBCH3706-09	DQ518109
Pettalidae	*Neopurcellia salmoni*	GBCH2559-08	DQ992323
Pettalidae	*Parapurcellia monticola*	GBCH1727-07	DQ518098
Pettalidae	*Parapurcellia silvicola*	GBCH0979-06	AY639582
Pettalidae	*Pettalus brevicauda*	GBCH1724-07	DQ518101
Pettalidae	*Pettalus sp. 259L*	GBCH1828-07	DQ825636
Pettalidae	*Pettalus sp. 259s*	GBCH1725-07	DQ518100
Pettalidae	*Pettalus sp. 266L*	GBCH1723-07	DQ518102
Pettalidae	*Pettalus sp. 266s*	GBCH1722-07	DQ518103
Pettalidae	*Pettalus sp. 270*	GBCH1721-07	DQ518104
Pettalidae	*Pettalus sp. 274*	GBCH1720-07	DQ518105
Pettalidae	*Pettalus sp. Sinharaja*	GBCH1827-07	DQ825637
Pettalidae	*Purcellia illustrans*	GBCH1726-07	DQ518099
Pettalidae	*Rakaia antipodiana*	GBCH2554-08	DQ992329
Pettalidae	*Rakaia arcticosa*	GBCH3704-09	DQ518111
Pettalidae	*Rakaia calcarobtusa*	GBCH2567-08	DQ992315
Pettalidae	*Rakaia crypta*	GBCH1705-07	DQ518120
Pettalidae	*Rakaia daviesae*	GBCH1713-07	DQ518112
Pettalidae	*Rakaia denticulata*	GBCH2681-08	DQ992201
Pettalidae	*Rakaia denticulata*	GBCH2676-08	DQ992206
Pettalidae	*Rakaia denticulata*	GBCH2649-08	DQ992233
Pettalidae	*Rakaia denticulata*	GBCH2647-08	DQ992235
Pettalidae	*Rakaia denticulata*	GBCH2646-08	DQ992236
Pettalidae	*Rakaia denticulata*	GBCH2640-08	DQ992242
Pettalidae	*Rakaia denticulata*	GBCH2636-08	DQ992246
Pettalidae	*Rakaia denticulata*	GBCH2610-08	DQ992272
Pettalidae	*Rakaia denticulata*	GBCH2609-08	DQ992273
Pettalidae	*Rakaia denticulata*	GBCH2608-08	DQ992274
Pettalidae	*Rakaia denticulata*	GBCH2607-08	DQ992275
Pettalidae	*Rakaia denticulata*	GBCH2606-08	DQ992276
Pettalidae	*Rakaia denticulata*	GBCH2604-08	DQ992278
Pettalidae	*Rakaia denticulata*	GBCH2601-08	DQ992281
Pettalidae	*Rakaia denticulata*	GBCH2600-08	DQ992282
Pettalidae	*Rakaia denticulata*	GBCH2596-08	DQ992286
Pettalidae	*Rakaia denticulata*	GBCH2592-08	DQ992290
Pettalidae	*Rakaia denticulata*	GBCH2589-08	DQ992293
Pettalidae	*Rakaia denticulata*	GBCH2587-08	DQ992295
Pettalidae	*Rakaia denticulata*	GBCH2584-08	DQ992298
Pettalidae	*Rakaia denticulata*	GBCH2581-08	DQ992301
Pettalidae	*Rakaia denticulata*	GBCH2574-08	DQ992308

Pettalidae	*Rakaia denticulata*	GBCH2573-08	DQ992309
Pettalidae	*Rakaia denticulata*	GBCH2571-08	DQ992311
Pettalidae	*Rakaia denticulata*	GBCH2570-08	DQ992312
Pettalidae	*Rakaia healyi*	GBCH2561-08	DQ992321
Pettalidae	*Rakaia lindsayi*	GBCH2531-08	DQ992352
Pettalidae	*Rakaia longitarsa*	GBCH2569-08	DQ992313
Pettalidae	*Rakaia macra*	GBCH3169-08	EU673668
Pettalidae	*Rakaia magna*	GBCH2542-08	DQ992341
Pettalidae	*Rakaia media*	GBCH3703-09	DQ518125
Pettalidae	*Rakaia media*	GBCH3168-08	EU673669
Pettalidae	*Rakaia n. sp. Akatarawa*	GBCH2539-08	DQ992344
Pettalidae	*Rakaia n. sp. Hineway*	GBCH2534-08	DQ992349
Pettalidae	*Rakaia n. sp. Mt. Stokes*	GBCH2562-08	DQ992320
Pettalidae	*Rakaia n. sp. Wi Toko*	GBCH2537-08	DQ992346
Pettalidae	*Rakaia n. sp. Wi Toko*	GBCH2535-08	DQ992348
Pettalidae	*Rakaia solitaria*	GBCH2540-08	DQ992343
Pettalidae	*Rakaia sorenseni*	GBCH1709-07	DQ518116
Pettalidae	*Rakaia stewartiensis*	GBCH2532-08	DQ992351
Pettalidae	*Rakaia uniloca*	GBCH3167-08	EU673671
Phalangiidae	*Mitopus glacialis*	GBCH7611-13	JQ746516
Phalangiidae	*Oligolophus tridens*	SSEIA3021-13	KM829124
Phalangiidae	*Phalangium opilio*	GBCH2386-08	EU523757
Phalangiidae	*Rilaena triangularis*	CNSLB461-12	KM836571
Podoctidae	*Lomanius longipalpus longipalpus*	GACAC619-12	JF786432
Samoidae	*Santobius sp. DNA104931*	GACAC620-12	JF786433
Sclerosomatidae	*Leiobunum*	CNSLN098-13	KM828572
Sclerosomatidae	*Leiobunum exillipes*	SSJAA1950-13	KM831035
Sclerosomatidae	*Leiobunum vittatum*	GBCH5178-10	EU271671
Sclerosomatidae	*Leiobunum vittatum*	CNPPD2569-12	KJ090904
Sclerosomatidae	*Nelima paessleri*	CNPCC040-13	KM828987
Sclerosomatidae	*Nelima paessleri*	SSBAB2890-12	KM838884
Sclerosomatidae		CNPPF1445-12	KJ167362
Sironidae	*Cyphophthalmus duricorius*	GBCH0953-06	AY639556
Sironidae	*Cyphophthalmus ere*	GBCH0954-06	AY639557
Sironidae	*Cyphophthalmus gjorgjevici*	GBCH0957-06	AY639560
Sironidae	*Cyphophthalmus markoi*	GBCH0958-06	AY639561
Sironidae	*Cyphophthalmus martensi*	GBCH0960-06	AY639563
Sironidae	*Cyphophthalmus minutus*	GBCH0963-06	AY639566
Sironidae	*Cyphophthalmus ognjanovici*	GBCH0964-06	AY639567
Sironidae	*Cyphophthalmus rumijae*	GBCH0967-06	AY639570
Sironidae	*Cyphophthalmus sp. 1 Bulgaria*	GBCH1161-06	AY918878
Sironidae	*Cyphophthalmus sp. 2 Bulgaria*	GBCH1162-06	AY918879
Sironidae	*Cyphophthalmus teyrovskyi*	GBCH0968-06	AY639571
Sironidae	*Cyphophthalmus trebinjanum*	GBCH0969-06	AY639572

Sironidae	*Cyphophthalmus zetae*	GBCH0971-06	AY639574
Sironidae	*Cyphophthalmus zetae*	GBCH0972-06	AY639575
Sironidae	*Paramiopsalis ramulosus*	GBCH1873-07	DQ513109
Sironidae	*Paramiopsalis ramulosus*	GACAC592-12	JF786389
Sironidae	*Parasiro coiffaiti*	GBCH1872-07	DQ513110
Sironidae	*Parasiro minor*	GACAC594-12	JF786391
Sironidae	*Siro boyerae*	GBCH1870-07	DQ513112
Sironidae	*Siro exilis*	GBCH0976-06	AY639579
Stygnommatidae	*Stygnomma sp.*	GACAC639-12	JF786452
Stygnommatidae	*Stygnomma sp.*	GACAC640-12	JF786453
Stygnommatidae	*Stygnomma sp.*	GACAC642-12	JF786455
Stygnommatidae	*Stygnomma teapense*	GACAC641-12	JF786454
Stygnopsidae	*Stygnopsis sp. DNA101416*	GACAC634-12	JF786447
Stygnopsidae	*Stygnopsis sp. DNA104855*	GACAC631-12	JF786444
Stygnopsidae	*Stygnopsis sp. DNA104856*	GACAC632-12	JF786445
Stylocellidae	*Fangensis spelaeus*	GBCH0980-06	AY639583
Stylocellidae	*Stylocellus sp. MCZDNA100609*	GBCH3696-09	DQ825625
Stylocellidae	*Stylocellus sp. MCZDNA101472*	GBCH3694-09	DQ825627
Stylocellidae	*Stylocellus sp. MCZDNA101483*	GBCH1831-07	DQ825633
Stylocellidae	*Stylocellus sp. MCZDNA101486*	GBCH1833-07	DQ825631
Stylocellidae	*Stylocellus sp. MCZDNA101488*	GBCH1830-07	DQ825634
Stylocellidae	*Stylocellus sp. MCZDNA101494*	GBCH1832-07	DQ825632
Stylocellidae	*Stylocellus sp. MCZDNA101514*	GBCH1836-07	DQ825628
Stylocellidae	*Stylocellus sp. MCZDNA101519*	GBCH1835-07	DQ825629
Stylocellidae	*Stylocellus sp. Pahang 1*	GBCH1728-07	DQ518097
Triaenonychidae	*Cyptobunus ungulatus madhousensis*	GBCH7379-13	JN547483
Triaenonychidae	*Metanonychus setulus*	GBCH4401-10	HM056731
Triaenonychidae	*Paranonychus brunneus*	GBCH4404-10	HM056728
Triaenonychidae	*Zuma acuta*	GBCH4398-10	HM056734
Troglosironidae	*Troglosiro aelleni*	GBCH0981-06	AY639584
Troglosironidae	*Troglosiro brevifossa*	GBCH4284-09	EU887039
Troglosironidae	*Troglosiro cf. juberthiei PS-2008*	GBCH4278-09	EU887045
Troglosironidae	*Troglosiro cf. juberthiei PS-2008*	GBCH4272-09	EU887058
Troglosironidae	*Troglosiro juberthiei*	GBCH4270-09	EU887060
Troglosironidae	*Troglosiro longifossa*	GBCH3701-09	DQ518127
Troglosironidae	*Troglosiro monteithi*	GBCH4279-09	EU887043
Troglosironidae	*Troglosiro ninqua*	GBCH3700-09	DQ518128
Troglosironidae	*Troglosiro oscitatio*	GBCH4281-09	EU887041
Troglosironidae	*Troglosiro raveni*	GBCH4280-09	EU887042
Troglosironidae	*Troglosiro sheari*	GBCH4286-09	EU887037
Troglosironidae	*Troglosiro sp. PS-2008*	GBCH4258-09	EU887046
Troglosironidae	*Troglosiro urbanus*	GBCH4282-09	EU887044
Troglosironidae	*Troglosiro wilsoni*	GBCH4251-09	EU887061
Trogulidae	*Trogulus nepaeformis*	GBCH7784-13	JN018142

Zalmoxidae	*Zalmoxida sp. DNA102677*	GACAC621-12	JF786434
**Palpigradi**			
Unknown family		GBCH7757-13	JN018169
**Pseudoscorpiones**			
Atemnidae	*Caecatemnus sp. JM-2008*	GBCH3494-09	EU559534
Atemnidae	*Cyclatemnus sp. JM-2008*	GBCH3500-09	EU559528
Atemnidae	*Miratemnus sp. JM-2008*	GBCH3493-09	EU559535
Atemnidae	*Oratemnus curtus*	GBCH3497-09	EU559531
Atemnidae	*Stenatemnus sp. JM-2008*	GBCH3499-09	EU559529
Atemnidae	*Titanatemnus sp. JM-2008*	GBCH3498-09	EU559530
Cheiridiidae		GACAC125-12	EU559570
Cheiridiidae		GACPS019-13	JN018178
Cheiridiidae		GACPS004-13	JN018207
Cheliferidae	*Nannochelifer sp. JM-2008*	GBCH3496-09	EU559532
Cheliferidae	*Philomaoria sp. JM-2008*	GBCH3492-09	EU559536
Cheliferidae	*Protochelifer victorianus*	GBCH3495-09	EU559533
Cheliferidae		GACPS022-13	JN018175
Cheliferidae		GACPS006-13	JN018205
Cheliferidae		CNGIE253-12	KM836901
Chernetidae	*Apatochernes sp. JM-2008*	GBCH3501-09	EU559527
Chernetidae	*Calymmachernes angulatus*	GBCH3503-09	EU559525
Chernetidae	*Conicochernes crassus*	GBCH3502-09	EU559526
Chernetidae	*Haplochernes sp. JM-2008*	GBCH3504-09	EU559524
Chernetidae	*Incachernes sp. JM-2008*	GBCH3505-09	EU559523
Chernetidae	*Marachernes bellus*	GBCH3506-09	EU559522
Chernetidae	*Pseudopilanus kuscheli*	GBCH3456-09	EU559574
Chernetidae		GACPS001-13	EU559573
Chernetidae		GACPS020-13	JN018177
Chernetidae		CNWBB106-13	KM831486
Chernetidae		SSEIA7678-13	KM836634
Chernetidae		SSWLD4310-13	KM840075
Chernetidae		TTSOW556-11	KP979117
Chernetidae		SMTPB8974-13	KP979323
Chthoniidae	*Afrochthonius godfreyi*	GBCH3521-09	EU559507
Chthoniidae	*Apochthonius sp. JM-2008*	GBCH3511-09	EU559517
Chthoniidae	*Austrochthonius sp. Australia*	GBCH3515-09	EU559513
Chthoniidae	*Austrochthonius sp. JM-2008*	GBCH3516-09	EU559512
Chthoniidae	*Austrochthonius sp. JM-2008*	GBCH3514-09	EU559514
Chthoniidae	*Chthonius sp. 1 JM-2008*	GBCH3517-09	EU559511
Chthoniidae	*Chthonius sp. 2 JM-2008*	GBCH3524-09	EU559504
Chthoniidae	*Chthonius tetrachelatus*	GACPS005-13	JN018206
Chthoniidae	*Drepanochthonius sp. JM-2008*	GBCH3513-09	EU559515
Chthoniidae	*Kleptochthonius sp. JM-2008*	GBCH3510-09	EU559518
Chthoniidae	*Lagynochthonius johni*	GBCH3525-09	EU559503

Chthoniidae	*Lagynochthonius*	GACPS024-13	JN018173
Chthoniidae	*Paraliochthonius sp. JM-2008*	GBCH3523-09	EU559505
Chthoniidae	*Pseudochthonius sp. JM-2008*	GBCH3509-09	EU559519
Chthoniidae	*Pseudotyrannochthonius sp. Australia*	GBCH3520-09	EU559508
Chthoniidae	*Pseudotyrannochthonius sp. JM-2008*	GBCH3519-09	EU559509
Chthoniidae	*Sathrochthonius insulanus*	GBCH3507-09	EU559521
Chthoniidae	*Selachochthonius sp. JM-2008*	GBCH3518-09	EU559510
Chthoniidae	*Tyrannochthonius sp. JM-2008*	GBCH3522-09	EU559506
Chthoniidae	*Tyrannochthonius*	GACPS023-13	JN018174
Feaellidae	*Feaella anderseni*	GBCH3528-09	EU559500
Garypidae	*Anagarypus heatwolei*	GBCH3488-09	EU559540
Garypidae	*Synsphyronus apimelus*	GBCH3491-09	EU559537
Geogarypidae	*Geogarypus nigrimanus*	GACPS017-13	JN018180
Geogarypidae	*Geogarypus sp. JM-2008*	GBCH3468-09	EU559560
Gymnobisiidae	*Mirobisium sp. JM-2008*	GBCH3481-09	EU559547
Hyidae	*Indohya sp. JM-2008*	GBCH3464-09	EU559564
Ideoroncidae	*Pseudalbiorix veracruzensis*	GBCH3461-09	EU559567
Larcidae	*Larca lata*	GBCH3465-09	EU559563
Lechytiidae	*Lechytia hoffi*	GBCH3512-09	EU559516
Neobisiidae	*Bisetocreagris*	GACPS016-13	JN018181
Neobisiidae	*Bisetocreagris*	GACPS015-13	JN018182
Neobisiidae	*Lissocreagris sp. JM-2008*	GBCH3473-09	EU559555
Neobisiidae	*Microbisium parvulum*	GBCH3470-09	EU559558
Neobisiidae	*Microbisium*	CNPPB922-12	KJ084070
Neobisiidae	*Microbisium*	SSEIA996-13	KM839785
Neobisiidae	*Neobisium polonicum*	GBCH3472-09	EU559556
Neobisiidae	*Roncus transsilvanicus*	GBCH3471-09	EU559557
Olipiidae	*Apolpium parvum*	GBCH3487-09	EU559541
Olipiidae	*Beierolpium bornemisszai*	GBCH3483-09	EU559545
Olipiidae	*Calocheiridius termitophilus*	GBCH3484-09	EU559544
Olipiidae	*Euryolpium sp. JM-2008*	GBCH3482-09	EU559546
Olipiidae	*Nanolpium sp. JM-2008*	GBCH3485-09	EU559543
Olipiidae	*Pachyolpium sp. JM-2008*	GBCH3486-09	EU559542
Olipiidae	*Progarypus sp. JM-2008*	GBCH3490-09	EU559538
Olipiidae	*Protogarypinus giganteus*	GBCH3463-09	EU559565
Olipiidae	*Pseudogarypinus cooperi*	GBCH3462-09	EU559566
Olipiidae	*Xenolpium sp. JM-2008*	GBCH3489-09	EU559539
Olipiidae		GACPS018-13	JN018179
Parahyidae	*Parahya submersa*	GBCH3480-09	EU559548
Pseudogarypidae	*Neopseudogarypus scutellatus*	GBCH3526-09	EU559502
Pseudogarypidae	*Pseudogarypus bicornis*	GBCH3527-09	EU559501
Sternophoridae	*Afrosternophorus sp. JM-2008*	GBCH3460-09	EU559568
Sternophoridae	*Garyops depressus*	GBCH3459-09	EU559569
Syarinidae	*Chitrella cala*	GBCH3477-09	EU559551

Syarinidae	*Ideobisium sp. JM-2008*	GBCH3479-09	EU559549
Syarinidae	*Ideoblothrus sp. JM-2008*	GBCH3466-09	EU559562
Syarinidae	*Nannobisium sp. JM-2008*	GBCH3467-09	EU559561
Syarinidae	*Syarinus sp. JM-2008*	GBCH3478-09	EU559550
Tridenchthoniidae	*Anaulacodithella sp. JM-2008*	GBCH3508-09	EU559520
Withiidae	*Withius sp. 1 JM-2008*	GBCH3458-09	EU559571
**Ricinulei**			
Ricinoididae	*Pseudocellus pearsei*	GBCH2722-08	EU024483
Ricinoididae		GBCH7756-13	JN018170
**Sarcoptiformes**			
Acaridae	*Acarus farris*	GBCH4463-10	GQ864338
Acaridae	Rhizoglyphus robini	GBCH4446-10	GQ864356
Acaridae	Tyroborus sp. AMUEnv048	GBCH4469-10	GQ864332
Acaridae		MYMCB545-11	JX833712
Achipteriidae	*Achipteria*	RBINA5693-13	KP979140
Achipteriidae	*Achipteria*	SSPAA8262-13	KM825418
Achipteriidae	*Achipteria coleoptrata*	CHACA545-09	JX837580
Achipteriidae	*Anachipteria*	SSBAC3374-12	KM825277
Achipteriidae	*Anachipteria howardi*	CHACB027-10	HQ558346
Achipteriidae	*Protoribates*	SSPAA6656-13	KM838075
Achipteriidae		SSBAA2084-12	KM829962
Achipteriidae		SSJAD2912-13	KM837596
Achipteriidae		SSPAA7778-13	KM837345
Alicorhagiidae		CHACB1166-10	HM907245
Alicorhagiidae		CHACB406-10	HQ558568
Alicorhagiidae		CHACB741-10	JX837515
Alicorhagiidae		CHACB942-10	JX835618
Alicorhagiidae		MYMCB435-11	JX836551
Alloptidae	Alloptes sp.	GBA14787-14	KF018834
Alloptidae	Alloptes stercorarii	GBA14816-14	KF018829
Alycidae		CHACA981-10	HM405845
Alycidae		CHACB1167-10	HM907246
Alycidae		MYMCA524-11	JX833821
Alycidae		MYMCG294-12	JX836099
Alycidae		SSWLC4144-13	KM838688
Ameronothridae	*Ameronothrus maculatus*	CHACA093-08	JX836689
Ameronothridae	*Ameronothrus maculatus*	SSBAF5815-13	KM832266
Ameronothridae	*Ameronothrus nigrofemoratus*	CHACA134-08	JX836498
Analgidae	Analges corvinus	GBCH4464-10	GQ864337
Analgidae	Analges sturninus	GBCH4460-10	GQ864342
Analgidae	Anhemialges longipes	GBCH4461-10	GQ864340
Analgidae	Mesalgoides	FMEP314-14	KP193470
Analgidae	Mesalgoides megnini	FMEP279-14	KP193472
Astegistidae	*Cultroribula*	MYMCA233-11	JX836775

Avenzoariidae	Avenzoaria totani	GBCH4454-10	GQ864348
Avenzoariidae	Pomeranzevia ninnii	GBCH4443-10	GQ864359
Avenzoariidae	Promegninia bulweriae	GBA22646-15	KM401844
Avenzoariidae	Promegninia calonectris	GBA22641-15	KM401839
Avenzoariidae	Rhinozachvatkinia sp. LS2013	GBA17826-14	KF111269
Avenzoariidae	Zachvatkinia isolata	GBA14789-14	KF018823
Avenzoariidae	Zachvatkinia stercorarii	GBA14875-14	KF018820
Brachychthoniidae		CHACB1048-10	HM907173
Brachychthoniidae		CHACB1049-10	HM907174
Brachychthoniidae		CHACB1050-10	HM907175
Brachychthoniidae		CHACB1209-10	HM907278
Brachychthoniidae		CHACB408-10	HQ558569
Brachychthoniidae		CHACB449-10	HQ558602
Brachychthoniidae		CHACB508-10	HQ558636
Brachychthoniidae		CHACB509-10	HQ558637
Brachychthoniidae		CHACB588-10	HQ558688
Brachychthoniidae		CHACB883-10	HM907326
Brachychthoniidae		CHACB884-10	HM907327
Brachychthoniidae		CHACB946-10	HM907367
Brachychthoniidae		CHACB947-10	HM907368
Brachychthoniidae		CHACC066-10	HM907463
Brachychthoniidae		MYMCA1188-11	JX835620
Brachychthoniidae		MYMCA232-11	JX833786
Brachychthoniidae		MYMCA340-11	JX837004
Brachychthoniidae		MYMCA382-11	JX836446
Brachychthoniidae		MYMCA421-11	JX838756
Brachychthoniidae		MYMCA533-11	JX836534
Brachychthoniidae		MYMCA559-11	JX833933
Brachychthoniidae		MYMCA581-11	JX834577
Brachychthoniidae		MYMCA833-11	JX835545
Brachychthoniidae		MYMCA883-11	JX834225
Brachychthoniidae		MYMCA884-11	JX837370
Brachychthoniidae		MYMCA919-11	JX838329
Brachychthoniidae		MYMCA921-11	JX837653
Brachychthoniidae		MYMCA978-11	JX836191
Brachychthoniidae		MYMCB102-11	JX837593
Brachychthoniidae		MYMCB175-11	JX833937
Brachychthoniidae		MYMCB235-11	JX835828
Brachychthoniidae		MYMCB438-11	JX836269
Brachychthoniidae		MYMCB857-11	JX835908
Brachychthoniidae		MYMCC069-11	JX836059
Brachychthoniidae		MYMCC070-11	JX836538
Brachychthoniidae		MYMCC278-11	JX837825
Brachychthoniidae		MYMCC446-11	JX838586

Brachychthoniidae		MYMCC605-11	JX837795
Brachychthoniidae		MYMCC621-11	JX838712
Brachychthoniidae		MYMCC844-11	JX834520
Brachychthoniidae		MYMCE227-12	JX837833
Brachychthoniidae		MYMCE606-12	JX834208
Brachychthoniidae		MYMCE706-12	JX835675
Brachychthoniidae		MYMCE859-12	JX835868
Brachychthoniidae		MYMCF208-12	JX834739
Brachychthoniidae		MYMCF380-12	JX838745
Brachychthoniidae		MYMCF398-12	JX836075
Brachychthoniidae		MYMCF431-12	JX838058
Brachychthoniidae		MYMCF512-12	JX837683
Brachychthoniidae		MYMCF530-12	JX837232
Brachychthoniidae		MYMCF547-12	JX835802
Brachychthoniidae		MYMCF551-12	JX835840
Brachychthoniidae		MYMCF569-12	JX837705
Brachychthoniidae		MYMCF570-12	JX836888
Brachychthoniidae		MYMCF595-12	JX833683
Brachychthoniidae		MYMCF628-12	JX837201
Brachychthoniidae		MYMCF647-12	JX836776
Brachychthoniidae		MYMCF926-12	JX833985
Brachychthoniidae		MYMCF949-12	JX838447
Brachychthoniidae		MYMCG329-12	JX834986
Brachychthoniidae		MYMCG465-12	JX835819
Brachychthoniidae		MYMCG521-12	JX833671
Brachychthoniidae		MYMCG557-12	JX836790
Brachychthoniidae		RBINA5788-13	KP979245
Brachychthoniidae		SSBAA3699-12	KM824601
Brachychthoniidae		SSBAB2820-12	KM839289
Carabodidae	*Carabodes*	SSPAA9183-13	KM839008
Carabodidae	*Carabodes labyrinthicus*	MYTMC143-09	GU680454
Carpoglyphidae	Carpoglyphus lactis	GBCH4457-10	GQ864345
Cepheidae	*Cepheus*	CHACA176-08	JX835471
Cepheidae	*Cepheus*	CNPAP518-13	KM833155
Cepheidae	*Cepheus*	SSEIA039-13	KM824089
Cepheidae	*Cepheus*	SSPAB7994-13	KM830040
Cepheidae	*Cepheus*	SSWLA671-13	KM824262
Cepheidae	*Cepheus*	SSWLC4145-13	KM825818
Cepheidae	*Cepheus*	SSWLC4155-13	KM825941
Cepheidae	*Oribatodes mirabilis*	RBINA5675-13	KP979179
Ceratozetidae	*Ceratozetes cuspidatus*	SSJAD3324-13	KM830430
Ceratozetidae	*Ceratozetes cuspidatus*	SSWLC4157-13	KM840288
Ceratozetidae	*Ceratozetes gracilis*	CHACA209-08	JX835739
Ceratozetidae	*Ceratozetes gracilis*	SSPAA8314-13	KM827061

Ceratozetidae	*Ceratozetes pacificus*	GBCH3204-08	EF989723
Ceratozetidae	*Ceratozetes thienemanni*	CHACA535-09	JX833727
Ceratozetidae	*Dentizetes*	SSBAB1904-12	KM832957
Ceratozetidae	*Dentizetes*	SSBAB1920-12	KM827292
Ceratozetidae	*Dentizetes*	SSBAB1926-12	KM835712
Ceratozetidae	*Dentizetes*	SSBAB1957-12	KM827549
Ceratozetidae	*Dentizetes*	SSBAB2826-12	KM824748
Ceratozetidae	*Dentizetes*	SSBAD6307-13	KM840227
Ceratozetidae	*Dentizetes*	SSEIB5791-13	KM831177
Ceratozetidae	*Dentizetes*	SSJAA1440-13	KM827796
Ceratozetidae	*Dentizetes*	SSJAA1991-13	KM826284
Ceratozetidae	*Dentizetes*	SSJAC970-13	KM830774
Ceratozetidae	*Dentizetes*	SSJAF5554-13	KM835233
Ceratozetidae	*Dentizetes*	SSJAF7386-13	KM824598
Ceratozetidae	*Dentizetes*	SSWLA5271-13	KM833462
Ceratozetidae	*Dentizetes*	SSWLA679-13	KM828004
Ceratozetidae	*Dentizetes*	SSWLC1149-13	KM827543
Ceratozetidae	*Dentizetes*	SSWLC1163-13	KM839105
Ceratozetidae	*Dentizetes*	SSWLC1175-13	KM830702
Ceratozetidae	*Dentizetes*	SSWLD8277-13	KM826579
Ceratozetidae	*Dentizetes rudentiger*	SSBAB2093-12	KM829196
Ceratozetidae	*Dentizetes rudentiger*	SSJAA865-13	KM827492
Ceratozetidae	*Diapterobates*	CHACA1066-10	HM907112
Ceratozetidae	*Diapterobates*	MYMCA1042-11	JX835417
Ceratozetidae	*Diapterobates*	MYMCA114-11	JX835534
Ceratozetidae	*Diapterobates*	MYMCA1287-11	JX833848
Ceratozetidae	*Diapterobates*	MYMCE681-12	JX833939
Ceratozetidae	*Diapterobates*	MYMCF614-12	JX838364
Ceratozetidae	*Diapterobates*	MYTMC083-09	HM431998
Ceratozetidae	*Diapterobates*	SSBAB1159-12	KM826878
Ceratozetidae	*Diapterobates*	SSBAB1932-12	KM831312
Ceratozetidae	*Diapterobates humeralis*	CHACA1011-10	HM907075
Ceratozetidae	*Diapterobates humeralis*	CHACA1096-10	HM907134
Ceratozetidae	*Diapterobates humeralis*	CHACA1175-10	JX838262
Ceratozetidae	*Diapterobates humeralis*	CHACA168-08	JX835254
Ceratozetidae	*Diapterobates humeralis*	CHACB1058-10	HM907182
Ceratozetidae	*Diapterobates humeralis*	CHACB189-10	HQ558461
Ceratozetidae	*Diapterobates humeralis*	CHACB266-10	HQ558507
Ceratozetidae	*Diapterobates humeralis*	CHACB564-10	HQ558678
Ceratozetidae	*Diapterobates humeralis*	CHACB565-10	HQ558679
Ceratozetidae	*Diapterobates humeralis*	CHACC086-10	HM907479
Ceratozetidae	*Diapterobates humeralis*	MYMCB877-11	JX834331
Ceratozetidae	*Diapterobates humeralis*	MYMCC040-11	JX834771
Ceratozetidae	*Diapterobates notatus*	CHACA501-09	JX837448

Ceratozetidae	*Diapterobates notatus*	CHACA611-09	JX833673
Ceratozetidae	*Diapterobates variabilis*	CHACA977-10	HM405841
Ceratozetidae	*Fuscozetes*	MIONB043-10	HM887578
Ceratozetidae	*Fuscozetes*	MIONB082-10	HQ575094
Ceratozetidae	*Fuscozetes fuscipes*	MIONB083-10	HQ575095
Ceratozetidae	*Fuscozetes fuscipes*	SSEIB8361-13	KM833473
Ceratozetidae	*Ghilarovizetes longisetosus*	CHACA151-08	JX835704
Ceratozetidae	*Jugatala*	CNBAA455-12	KM825639
Ceratozetidae	*Jugatala*	CNBAA459-12	KM834925
Ceratozetidae	*Jugatala*	CNBAA469-12	KM831471
Ceratozetidae	*Jugatala*	CNBAB376-12	KM830701
Ceratozetidae	*Jugatala*	CNGLD019-13	KM838589
Ceratozetidae	*Jugatala*	CNGLD024-13	KM840791
Ceratozetidae	*Jugatala*	CNGLD027-13	KM825597
Ceratozetidae	*Jugatala*	SSBAB127-12	KM837090
Ceratozetidae	*Jugatala*	SSWLC1152-13	KM829300
Ceratozetidae	*Lugoribates gracilis*	CHACA368-08	JX837384
Ceratozetidae	*Melanozetes*	CHACA685-09	JX837939
Ceratozetidae	*Melanozetes*	MYMCE235-12	JX835048
Ceratozetidae	*Neogymnobates luteus*	CHACA1203-10	JX835014
Ceratozetidae	*Svalbardia paludicola*	CHACA523-09	JX834122
Ceratozetidae	*Svalbardia paludicola*	CHACB751-10	HQ558785
Ceratozetidae	*Trichoribates*	ASAMT047-12	KP979326
Ceratozetidae	*Trichoribates*	CHACA568-09	JX835279
Ceratozetidae		CHACA525-09	JX834757
Ceratozetidae		CNBAK398-13	KM837748
Ceratozetidae		CNBPC217-12	KM833467
Ceratozetidae		CNBPE658-12	KM838032
Ceratozetidae		CNPAH426-13	KM826320
Ceratozetidae		CNPCA041-13	KM827020
Ceratozetidae		CNPCC060-13	KM830479
Ceratozetidae		CNPCC063-13	KM830573
Ceratozetidae		CNPCD081-13	KM830038
Ceratozetidae		CNPCD083-13	KM833585
Ceratozetidae		CNPCD101-13	KM838907
Ceratozetidae		CNPCD124-13	KM824481
Ceratozetidae		CNPCD134-13	KM840447
Ceratozetidae		CNPCF113-13	KM837738
Ceratozetidae		CNPCF130-13	KM824337
Ceratozetidae		CNPCF138-13	KM838966
Ceratozetidae		CNRME4771-12	KM832863
Ceratozetidae		MIAUS002-12	KP979328
Ceratozetidae		MIAUS010-12	KP979274
Ceratozetidae		MIAUS013-12	KP979302

Ceratozetidae		MIAUS014-12	KP979152
Ceratozetidae		MIONB200-10	KP979173
Ceratozetidae		MYMCB697-11	JX837103
Ceratozetidae		MYMCC479-11	JX838751
Ceratozetidae		SSBAA3680-12	KM824866
Ceratozetidae		SSBAA3689-12	KM834699
Ceratozetidae		SSBAD3137-12	KM825894
Ceratozetidae		SSBAD5572-13	KM827886
Ceratozetidae		SSPAA6653-13	KM826281
Ceratozetidae		SSWLA037-13	KM830029
Ceratozetidae		SSWLC064-13	KM834432
Ceratozetidae		SSWLC1156-13	KM840748
Chamobatidae	*Chamobates*	SSEIB4557-13	KM839892
Chamobatidae	*Chamobates*	SSPAA8423-13	KM832485
Chamobatidae	*Chamobates cuspidatus*	SSEIB4571-13	KM829229
Crotoniidae	*Camisia*	CHACA204-08	JX836165
Crotoniidae	*Camisia*	CHACA283-08	JX836942
Crotoniidae	*Camisia*	CNGIB594-12	KM833294
Crotoniidae	*Camisia*	MYMCB929-11	JX833752
Crotoniidae	*Camisia*	MYTMC070-09	HM431996
Crotoniidae	*Camisia*	SSPAA9754-13	KM832943
Crotoniidae	*Camisia*	SSWLC1165-13	KM836576
Crotoniidae	*Camisia*	SSWLC3632-13	KM840769
Crotoniidae	*Camisia*	SSWLD8140-13	KM840817
Crotoniidae	*Camisia biurus*	CHACB979-10	HM907388
Crotoniidae	*Camisia biverrucata*	CHACA339-08	JX837953
Crotoniidae	*Camisia biverrucata*	MYMCC066-11	JX837139
Crotoniidae	*Camisia biverrucata*	SSBAA3681-12	KM838175
Crotoniidae	*Camisia biverrucata*	SSWLB5365-13	KM832257
Crotoniidae	*Camisia horrida*	CHACB1189-10	HM907264
Crotoniidae	*Camisia lapponica*	CHACA165-08	JX834864
Crotoniidae	*Camisia lapponica*	CHACA546-09	JX833920
Crotoniidae	*Camisia lapponica*	MYTMC030-09	HM431992
Crotoniidae	*Heminothrus*	MYMCA079-11	JX835303
Crotoniidae	*Heminothrus*	MYTMC094-09	GU680455
Crotoniidae	*Heminothrus longisetosus*	MYTMC170-09	GU680428
Crotoniidae	*Heminothrus thori*	MITMH015-07	KP979191
Crotoniidae	*Neonothrus humicola*	CHACA056-08	JX838109
Crotoniidae	*Platynothrus*	SSWLD3469-13	KM829877
Crotoniidae	*Platynothrus peltifer*	CHACA256-08	JX838205
Crotoniidae	*Platynothrus peltifer*	GBCH1955-07	DQ381225
Crotoniidae	*Platynothrus peltifer*	GBCH1970-07	DQ381210
Crotoniidae	*Platynothrus peltifer*	GBCH1971-07	DQ381209
Crotoniidae	*Platynothrus peltifer*	GBCH1974-07	DQ381206

Crotoniidae	*Platynothrus peltifer*	GBCH1995-07	DQ381185
Crotoniidae	*Platynothrus peltifer*	GBCH1997-07	DQ381183
Crotoniidae	*Platynothrus peltifer*	GBCH2016-07	DQ381164
Crotoniidae	*Platynothrus peltifer*	MIONB006-10	HM887559
Crotoniidae	*Platynothrus peltifer*	MYMCB170-11	JX838216
Crotoniidae	*Platynothrus yamasaki*	SSEIA2090-13	KM836447
Cymbaeremaeidae	*Ametroproctus*	SSBAA3690-12	KM824785
Cymbaeremaeidae	*Ametroproctus*	SSJAD2916-13	KM826992
Cymbaeremaeidae	*Ametroproctus*	SSWLC1158-13	KM834886
Cymbaeremaeidae	*Ametroproctus*	SSWLF3159-13	KM825969
Cymbaeremaeidae	*Cymbaeremaeus cymba*	GBCH5033-10	GU208618
Cymbaeremaeidae	*Cymbaeremaeus cymba*	GBCH5035-10	GU208616
Cymbaeremaeidae	*Scapheremaeus*	MIONB275-10	KP979242
Cymbaeremaeidae		CNBAF202-12	KM824396
Cymbaeremaeidae		CNBPA195-12	KM831105
Cymbaeremaeidae		CNBPJ297-13	KM830043
Cymbaeremaeidae		CNGIB595-12	KM832618
Cymbaeremaeidae		CNPAG376-13	KM829505
Cymbaeremaeidae		CNPCI012-13	KM831437
Cymbaeremaeidae		SSPAB4025-13	KM836439
Cymbaeremaeidae		SSPAB966-13	KM835062
Damaeidae	*Epidamaeus*	CHACA1173-10	JX836445
Damaeidae	*Epidamaeus*	SSEIA3050-13	KM824003
Damaeidae	*Epidamaeus*	SSJAC1525-13	KM837646
Damaeidae	*Epidamaeus floccosus*	CHACB034-10	HQ558347
Damaeidae	*Epidamaeus floccosus*	SSBAA3632-12	KM826166
Damaeidae	*Epidamaeus longitarsalis*	CHACA171-08	JX833768
Damaeidae	*Quatrobelba montana*	SSJAD2917-13	KM828070
Damaeidae		CHACA936-10	HM405813
Damaeidae		CHACA957-10	HM405828
Damaeidae		MYMCB747-11	JX835562
Damaeidae		MYMCC473-11	JX838304
Damaeidae		SSEIC4928-13	KM830745
Damaeidae		SSWEE055-13	KM827320
Damaeidae		SSWLA5276-13	KM838922
Damaeidae		SSWLE2956-13	KM838172
Damaeidae		SSWLF3569-13	KM834969
Eniochthoniidae	*Eniochthonius crosbyi*	MIONB078-10	HQ575092
Eniochthoniidae		MYMCC715-11	JX835537
Eremaeidae	*Eremaeus*	CHACA937-10	HM405814
Eremaeidae	*Eremaeus*	CHACA943-10	HM405820
Eremaeidae	*Eueremaeus*	CNGLF135-13	KM829137
Eremaeidae	*Eueremaeus*	SSBAD4700-13	KM838021
Eremaeidae	*Eueremaeus*	SSPAB3136-13	KM835219

Eremaeidae	*Eueremaeus*	SSPAB3974-13	KM829849
Eremaeidae	*Eueremaeus cf. quadrilamellatus*	CHACA188-08	JX836086
Eremaeidae	*Eueremaeus chiatous*	CNBAF200-12	KP979204
Eremaeidae	*Eueremaeus marshalli*	CHACB673-10	HQ558741
Eremaeidae	*Eueremaeus quadrilamellatus*	MYMCF666-12	JX837845
Eremaeidae	*Eueremaeus tetrosus*	SSBAA3685-12	KM827623
Eremaeidae		CNGLC085-13	KM829296
Eremaeidae		MYMCB087-11	JX834312
Eremaeidae		SSBAA3663-12	KM835787
Eremaeidae		SSBAA3679-12	KM840040
Eremaeidae		SSBAB2079-12	KM838742
Eremaeidae		SSEIA7698-13	KM826325
Eremaeidae		SSJAA2032-13	KM833610
Eremaeidae		SSPAA9717-13	KM826478
Eremaeidae		SSWEE036-13	KM833186
Eremaeidae		SSWLC3648-13	KM829633
Eremaeidae		SSWLD3660-13	KM838758
Eremaeidae		SSWLD8204-13	KM830064
Eremaeidae		SSWLD8235-13	KM833550
Eremaeidae		SSWLD8307-13	KM828213
Eremaeidae		SSWLF3028-13	KM828657
Eremaeidae		SSWLF3040-13	KM833069
Eremaeidae		SSWLF3100-13	KM839003
Eremobelbidae		MIONB135-10	KP979314
Eremulidae	*Eremulus*	CHACA954-10	HM405826
Euphthiracaridae	*Rhysotritia ardua*	CHACB901-10	HM907333
Euphthiracaridae		RBINA5668-13	KP979145
Euphthiracaridae		RBINA5669-13	KP979310
Euphthiracaridae		RBINA5761-13	KP979343
Euzetidae	*Euzetes*	RBINA2936-13	KP979293
Euzetidae	*Euzetes globulus*	RBINA5691-13	KP979202
Gabuciniidae	Gabucinia delibata	FMEP278-14	KP193464
Gabuciniidae	Hieracolichus nisi	GBCH4442-10	GQ864360
Galumnidae	*Galumna*	CHACA1207-10	JX835163
Galumnidae	*Pergalumna*	CNSLR103-13	KP979288
Galumnidae	*Pergalumna*	MIONB055-10	HQ575081
Galumnidae	*Pergalumna*	SSPAA9091-13	KM831020
Galumnidae	*Pilogalumna*	CHACB735-10	HQ558779
Galumnidae	*Pilogalumna*	MYMCE872-12	JX836576
Galumnidae		RBINA3937-13	KP979255
Galumnidae		RBINA5723-13	KP979318
Galumnidae		RBINA5739-13	KP979146
Galumnidae		RBINA5773-13	KP979290
Galumnidae		RBINA5783-13	KP979351

Galumnidae		SSBAD4760-13	KM827618
Galumnidae		SSBAD5559-13	KM834004
Galumnidae		SSEIA2092-13	KM829432
Galumnidae		SSEIA3040-13	KM834017
Galumnidae		SSEIB4541-13	KM832757
Galumnidae		SSWLA034-13	KM837399
Galumnidae		SSWLD8157-13	KM840819
Galumnidae		SSWLD8319-13	KM828182
Galumnidae		SSWLD8346-13	KM835729
Gustaviidae	*Gustavia*	SSEIB8271-13	KM840784
Gustaviidae	*Gustavia microcephala*	RBINA3914-13	KP979329
Gymnodamaeidae	*Gymnodamaeus*	CNGLD028-13	KM837488
Gymnodamaeidae	*Gymnodamaeus*	CNRMD2678-12	KM838755
Gymnodamaeidae	*Gymnodamaeus*	SSBAB1208-12	KM838893
Gymnodamaeidae	*Gymnodamaeus*	SSJAB1882-13	KM831192
Gymnodamaeidae	*Gymnodamaeus*	SSJAC988-13	KM839431
Gymnodamaeidae	*Gymnodamaeus*	SSPAA7961-13	KM836231
Gymnodamaeidae	*Gymnodamaeus*	SSPAA8145-13	KM832442
Gymnodamaeidae	*Gymnodamaeus*	SSWLE2970-13	KM838958
Gymnodamaeidae	*Gymnodamaeus ornatus*	SSEIB4559-13	KM840834
Haplozetidae	*Peloribates canadensis*	CHACA845-09	GU702809
Haplozetidae	*Peloribates pilosus*	CHACA272-08	JX836523
Haplozetidae	*Peloribates pilosus*	CHACA340-08	JX836260
Haplozetidae	*Peloribates pilosus*	MYMCA729-11	JX833747
Haplozetidae	*Rostrozetes*	MIONB139-10	KP979348
Haplozetidae		CNPAF907-13	KM837599
Haplozetidae		MIONB048-10	HM887582
Hermanniellidae	*Hermanniella robusta*	SSEIB8188-13	KM838734
Hermanniidae	*Hermannia reticulata*	CHACA100-08	JX835857
Hermanniidae	*Hermannia reticulata*	CHACA147-08	JX837848
Histiostomatidae	Bonomoia sp. AMUBon02	GBCH4459-10	GQ864343
Histiostomatidae	Histiostoma ovalis	GBA14668-14	KJ533743
Hydrozetidae	*Hydrozetes*	MYMCE365-12	JX837779
Hydrozetidae	*Hydrozetes*	MYMCG108-12	JX837497
Hypochthoniidae	*Hypochthonius rufulus*	MYMCG087-12	JX836301
Knemidokoptidae	Knemidokoptes jamaicensis	GBCH11625-13	JQ037816
Knemidokoptidae	Knemidokoptes sp.	GBA17579-14	KJ787640
Liacaridae	*Dorycranosus*	SSWLB2395-13	KM832484
Liacaridae	*Dorycranosus acutidens*	CHACB983-10	HM907390
Liacaridae	*Dorycranosus acutidens*	SSPAB9002-13	KM837224
Liacaridae		CHACC249-10	HQ941553
Liacaridae		CNSLD654-12	KM830359
Listrophoridae	Leporacarus gibbus	GBCH4466-10	GQ864335
Listrophoridae	Lynxacarus mustelae	GBCH4465-10	GQ864336

Listrophoridae	Schizocarpus sp. AMUChi01	GBCH4458-10	GQ864344
Malaconothridae	*Malaconothrus*	CHACB596-10	HQ558695
Malaconothridae	*Malaconothrus*	CHACB804-10	HQ941494
Malaconothridae	*Malaconothrus*	MYMCF207-12	JX834311
Malaconothridae	*Malaconothrus mollisetosus*	CHACA202-08	JX834028
Malaconothridae		MYMCC003-11	JX838251
Malaconothridae		MYMCC166-11	JX836555
Malaconothridae		MYMCG503-12	JX834637
Malaconothridae		SSPAA7988-13	KM832716
Megeremaeidae	*Megeremaeus*	SSJAA2177-13	KM839394
Mochlozetidae	*Podoribates pratensis*	JSAUG1789-12	KP979190
Mochlozetidae		CNPPA4112-12	KJ087519
Mochlozetidae		CNPPC1947-12	KJ089248
Mochlozetidae		MIONB163-10	KP979284
Mochlozetidae		MIONB165-10	KP979335
Mochlozetidae		MIONB166-10	KP979300
Mochlozetidae		NCCE063-11	KP979248
Mycobatidae	*Mycobates beringianus*	CHACA1074-10	HM907117
Mycobatidae	*Mycobates conitus*	CHACA094-08	JX835779
Mycobatidae	*Mycobates incurvatus*	CHACA1208-10	JX834341
Mycobatidae	*Mycobates perates*	CHACA115-08	JX835102
Mycobatidae	*Punctoribates*	MYMCE237-12	JX838100
Mycobatidae	*Punctoribates palustris*	SSPAA6658-13	KM836849
Mycobatidae		CNPCI027-13	KM831793
Mycobatidae		MYMCA347-11	JX835475
Mycobatidae		MYMCC167-11	JX835952
Mycobatidae		MYMCC168-11	JX836151
Mycobatidae		MYMCG071-12	JX835766
Myocoptidae	Myocoptidae sp. AMUMyo01	GACAC424-12	GQ864341
Nanorchestidae	*Nanorchestes*	CHACB215-10	HQ558475
Nanorchestidae	*Nanorchestes*	CHACB329-10	JX833723
Nanorchestidae	*Nanorchestes*	CHACB627-10	HQ558713
Nanorchestidae	*Nanorchestes*	MYMCA1196-11	JX834346
Nanorchestidae	*Nanorchestes*	MYMCA1447-11	JX836155
Nanorchestidae	*Nanorchestes*	MYMCB206-11	JX836597
Nanorchestidae	*Nanorchestes*	MYMCE633-12	JX835287
Nanorchestidae	*Nanorchestes*	MYMCF035-12	JX836598
Nanorchestidae	*Nanorchestes*	MYMCF864-12	JX838603
Neoliodidae		MIONB251-10	KP979223
Neoliodidae		MIONB253-10	KP979227
Neoliodidae		MIONB291-10	KP979268
Nothridae	*Nothrus*	BIOAI256-14	KP979253
Nothridae	*Nothrus*	BIOAI507-14	KP979305
Nothridae	*Nothrus*	CHACA1005-10	HM907070

Nothridae	*Nothrus*	CHACA1006-10	HM907071
Nothridae	*Nothrus*	CHACB708-10	HQ558762
Nothridae	*Nothrus*	MIONB276-10	KP979150
Nothridae	*Nothrus*	MITMH027-07	KP979256
Nothridae	*Nothrus*	MYMCF433-12	JX837694
Nothridae	*Nothrus*	RBINA5649-13	KP979137
Nothridae	*Nothrus anauniensis*	RBINA5684-13	KP979344
Nothridae	*Nothrus borussicus*	SSJAB2151-13	KM832928
Nothridae	*Nothrus pratensis*	MYMCC768-11	JX835728
Nothridae	*Nothrus silvestris*	GBCH3787-09	DQ381157
Oppiidae	*Opiella*	CHACB1129-10	HM907221
Oppiidae	*Oppiella clavigera*	CHACB678-10	HQ558744
Oppiidae		CHACA1001-10	HM405808
Oppiidae		CHACA1002-10	HM405809
Oppiidae		CHACA1003-10	HM405810
Oppiidae		CHACA1126-10	JX836213
Oppiidae		CHACA123-08	JX833922
Oppiidae		CHACA203-08	JX836447
Oppiidae		CHACA223-08	JX834804
Oppiidae		CHACA226-08	JX836862
Oppiidae		CHACA362-08	JX838130
Oppiidae		CHACB1152-10	HM907238
Oppiidae		CHACB1203-10	HM907273
Oppiidae		CHACB374-10	HQ558550
Oppiidae		CHACB621-10	HQ558708
Oppiidae		CHACB647-10	HQ558727
Oppiidae		CHACB727-10	HQ558776
Oppiidae		CHACB934-10	HM907360
Oppiidae		CNPAF837-13	KM824817
Oppiidae		MIONB065-10	HQ575082
Oppiidae		MIONB076-10	HQ575090
Oppiidae		MIONB197-10	KP979127
Oppiidae		MYMCA1234-11	JX835587
Oppiidae		MYMCA182-11	JX835822
Oppiidae		MYMCA240-11	JX838436
Oppiidae		MYMCA356-11	JX838297
Oppiidae		MYMCB060-11	JX835815
Oppiidae		MYMCB828-11	JX834632
Oppiidae		MYMCC643-11	JX836853
Oppiidae		MYMCE052-12	JX834201
Oppiidae		MYMCE807-12	JX837188
Oppiidae		MYMCE896-12	JX833940
Oppiidae		MYMCE897-12	JX834235
Oppiidae		MYMCF591-12	JX834503

Oppiidae		MYMCF940-12	JX838615
Oppiidae		MYMCG075-12	JX836023
Oppiidae		MYMCG342-12	JX837636
Oppiidae		SSPAA8496-13	KM832763
Oppiidae		SSPAA9146-13	KM829817
Oribatellidae	*Oribatella*	CHACA1119-10	JX837692
Oribatellidae		SSEIA3023-13	KM839758
Oribatellidae		SSJAD2905-13	KM839217
Oribatellidae		SSJAD3339-13	KM825769
Oribatellidae		SSPAC12479-13	KM827581
Oribatulidae	*Liebstadia*	MYMCE691-12	JX834092
Oribatulidae	*Liebstadia similis*	MYMCE946-12	JX836552
Oribatulidae	*Lucoppia*	SSPAA8163-13	KM831553
Oribatulidae	*Oribatula*	CHACA383-09	JX833640
Oribatulidae	*Oribatula*	CNJAD1857-12	KM833722
Oribatulidae	*Oribatula*	CNJAE1142-12	KM827975
Oribatulidae	*Oribatula*	SMTPB669-13	KP979336
Oribatulidae	*Oribatula*	SSWLD8202-13	KM835050
Oribatulidae	*Oribatula tibialis*	GBCH1954-07	DQ381226
Oribatulidae	*Phauloppia boletorum*	CNRMB163-12	KM834644
Oribatulidae	*Zygoribatula*	MYMCA171-11	JX835678
Oribatulidae	*Zygoribatula*	MYMCA630-11	JX835491
Oribatulidae	*Zygoribatula bulanovae*	CHACA1083-10	HM907124
Oribatulidae	*Zygoribatula bulanovae*	CHACB017-10	HQ558338
Oribatulidae	*Zygoribatula bulanovae*	CHACB489-10	HQ558624
Oribatulidae		CHACC093-10	HM907482
Oribatulidae		CHACC103-10	JX834464
Oribatulidae		CNBAI485-13	KM834577
Oribatulidae		CNBPC221-12	KM827986
Oribatulidae		CNBPK330-13	KM828749
Oribatulidae		CNBPL289-13	KM830856
Oribatulidae		CNBPN280-13	KM834673
Oribatulidae		CNGIB592-12	KM829472
Oribatulidae		CNGLA019-13	KM830328
Oribatulidae		CNGLA020-13	KM831919
Oribatulidae		CNPCA060-13	KM828491
Oribatulidae		CNPCF096-13	KM833296
Oribatulidae		CNSLB473-12	KM828062
Oribatulidae		CNSLC557-12	KM830957
Oribatulidae		NCCE012-11	KP979261
Oribatulidae		SMTPB19765-13	KP979184
Oribatulidae		SMTPB3362-13	KP979321
Oribatulidae		SSWLA031-13	KM830510
Oribatulidae		SSWLA675-13	KM836017

Oribatulidae		SSWLD2914-13	KM824271
Oribatulidae		SSWLD8131-13	KM825017
Oribatulidae		SSWLD8304-13	KM827920
Oribatulidae		SSWLD8325-13	KM824379
Oribatulidae		SSWLD8327-13	KM824171
Oribatulidae		SSWLD8365-13	KM838606
Oribotritiidae	*Protoribotritia*	SSJAD3372-13	KM832971
Oripodidae		CNBPB228-12	KM828056
Oripodidae		CNBPB539-12	KM836669
Oripodidae		CNBPB540-12	KM838991
Oripodidae		CNSLB469-12	KM825179
Oripodidae		SMTPB20118-13	KP979251
Oripodidae		SMTPB9978-13	KP979230
Parakalummidae	*Neoribates*	SSEIB4568-13	KM830978
Parakalummidae	*Neoribates*	SSPAC12256-13	KM826721
Parakalummidae	*Neoribates aurantiacus*	CHACA369-08	JX837225
Parakalummidae		MYMCE597-12	JX834397
Parakalummidae		SSEID1707-13	KM829309
Passalozetidae	*Bipassalozetes*	MYMCB598-11	JX835874
Peloppiidae	*Ceratoppia*	CNGID224-12	KM832968
Peloppiidae	*Ceratoppia*	CNGIE401-12	KM835992
Peloppiidae	*Ceratoppia*	CNPCB088-13	KM839733
Peloppiidae	*Ceratoppia*	CNPCE128-13	KM835524
Peloppiidae	*Ceratoppia*	CNPCF093-13	KM833595
Peloppiidae	*Ceratoppia*	CNPCF119-13	KM836088
Peloppiidae	*Ceratoppia*	CNPCF125-13	KM833622
Peloppiidae	*Ceratoppia*	CNPCG047-13	KM828534
Peloppiidae	*Ceratoppia*	MIONB039-10	HM887576
Peloppiidae	*Ceratoppia*	MIONB053-10	HQ575079
Peloppiidae	*Ceratoppia*	MIONB070-10	HQ575085
Peloppiidae	*Ceratoppia*	MYMCB848-11	JX838303
Peloppiidae	*Ceratoppia*	SSPAB8722-13	KM825114
Peloppiidae	*Ceratoppia*	SSWLA033-13	KM824430
Peloppiidae	*Ceratoppia*	SSWLA1888-13	KM840318
Peloppiidae	*Ceratoppia quadridentata*	CHACA1020-10	HM907079
Peloppiidae	*Dendrozetes*	CNPCD129-13	KM832737
Peloppiidae	*Dendrozetes*	CNPCI023-13	KM834716
Phenopelopidae	*Eupelops*	CHACA539-09	JX834185
Phenopelopidae	*Eupelops*	CHACB734-10	HQ558778
Phenopelopidae	*Eupelops*	CHACB925-10	HM907353
Phenopelopidae	*Eupelops*	RBINA4455-13	KP979346
Phenopelopidae	*Eupelops septentrionalis*	CHACA161-08	JX834120
Phenopelopidae	*Peloptulus*	SSBAD4014-12	KM832060
Phenopelopidae	*Propelops alaskensis*	SSPAA9036-13	KM838539

Phenopelopidae	*Propelops alaskensis*	SSPAC10878-13	KM833055
Phenopelopidae	*Propelops canadensis*	CHACA153-08	JX833634
Phenopelopidae	*Unduloribates undulatus*	GBCH5037-10	GU208614
Phenopelopidae		SSBAB1925-12	KM827394
Phenopelopidae		SSJAB3357-13	KM826764
Phthiracaridae	*Phthiracarus*	MIONB031-10	HM887571
Phthiracaridae	*Phthiracarus*	SSJAD2976-13	KM824555
Phthiracaridae	*Phthiracarus boresetosus*	RBINA5734-13	KP979172
Phthiracaridae		CHACA1030-10	HM907086
Phthiracaridae		MIONB034-10	HM887572
Phthiracaridae		MIONB036-10	HM887573
Phthiracaridae		RBINA5671-13	KP979201
Phthiracaridae		RBINA5674-13	KP979241
Phthiracaridae		SSPAA7743-13	KM838083
Proctophyllodidae	Dolichodectes	FMEP100-14	KP193461
Proctophyllodidae	Joubertophyllodes modularis	FMEP260-14	KP193466
Proctophyllodidae	Monojoubertia hemiphylla	FMEP265-14	KP193476
Proctophyllodidae	Monojoubertia microphylla	FMEP001-14	KP193483
Proctophyllodidae	Montesauria cilindrica	FMEP284-14	KP193488
Proctophyllodidae	Proctophyllodes	FMEP003-14	KP193734
Proctophyllodidae	Proctophyllodes	FMEP006-14	KP193693
Proctophyllodidae	Proctophyllodes	FMEP012-14	KP193489
Proctophyllodidae	Proctophyllodes acanthicaulus	FMEP112-14	KP193492
Proctophyllodidae	Proctophyllodes anthi	FMEP023-14	KP193503
Proctophyllodidae	Proctophyllodes ateri	FMEP024-14	KP193507
Proctophyllodidae	Proctophyllodes caulyfer	FMEP319-14	KP193511
Proctophyllodidae	*Proctophyllodes cetti*	GBCH3314-09	EU258757
Proctophyllodidae	Proctophyllodes clavatus	FMEP133-14	KP193522
Proctophyllodidae	Proctophyllodes clavatus	FMEP203-14	KP193518
Proctophyllodidae	Proctophyllodes cotyledon	FMEP015-14	KP193524
Proctophyllodidae	Proctophyllodes doleophyes	FMEP008-14	KP193545
Proctophyllodidae	Proctophyllodes fuchsi	FMEP020-14	KP193548
Proctophyllodidae	Proctophyllodes glandarinus	FMEP021-14	KP193549
Proctophyllodidae	Proctophyllodes hipposideros	FMEP190-14	KP193553
Proctophyllodidae	Proctophyllodes leptocaulus	FMEP030-14	KP193557
Proctophyllodidae	Proctophyllodes lusciniae	FMEP002-14	KP193580
Proctophyllodidae	Proctophyllodes macedo	FMEP272-14	KP193581
Proctophyllodidae	Proctophyllodes megaphyllus	FMEP273-14	KP193584
Proctophyllodidae	Proctophyllodes mesocaulus	FMEP101-14	KP193588
Proctophyllodidae	Proctophyllodes miliariae	FMEP274-14	KP193599
Proctophyllodidae	Proctophyllodes motacillae	FMEP005-14	KP193605
Proctophyllodidae	Proctophyllodes musicus	FMEP018-14	KP193616
Proctophyllodidae	Proctophyllodes musicus	FMEP211-14	KP193618
Proctophyllodidae	Proctophyllodes pinnatus	FMEP172-14	KP193651

Proctophyllodidae	Proctophyllodes poublani	FMEP321-14	KP193657
Proctophyllodidae	Proctophyllodes reguli	FMEP028-14	KP193661
Proctophyllodidae	Proctophyllodes remizicola	FMEP283-14	KP193662
Proctophyllodidae	Proctophyllodes rubeculinus	FMEP004-14	KP193664
Proctophyllodidae	Proctophyllodes schoenicli	FMEP022-14	KP193679
Proctophyllodidae	Proctophyllodes schwerinensis	FMEP288-14	KP193687
Proctophyllodidae	Proctophyllodes simillimus	FMEP029-14	KP193698
Proctophyllodidae	Proctophyllodes spini	FMEP173-14	KP193702
Proctophyllodidae	Proctophyllodes stylifer	FMEP019-14	KP193714
Proctophyllodidae	Proctophyllodes stylifer	FMEP039-14	KP193716
Proctophyllodidae	Proctophyllodes stylifer	FMEP183-14	KP193705
Proctophyllodidae	Proctophyllodes stylifer	FMEP219-14	KP193712
Proctophyllodidae	Proctophyllodes stylifer	FMEP231-14	KP193715
Proctophyllodidae	Proctophyllodes sylviae	FMEP016-14	KP193719
Proctophyllodidae	Proctophyllodes tenericaulus	FMEP323-14	KP193726
Proctophyllodidae	Proctophyllodes valchukae	GACAC1193-12	JN936871
Proctophyllodidae	Proctophyllodes vassilevi	FMEP293-14	KP193743
Proctophyllodidae	Proctophyllodes vegetans	FMEP301-14	KP193745
Proctophyllodidae	Proctophyllodes vitzthumi	FMEP025-14	KP193746
Proctophyllodidae	Proctophyllodes volgini	FMEP302-14	KP193747
Proctophyllodidae	Proctophyllodes weigoldi	FMEP295-14	KP193749
Proctophyllodidae	Pterodectes rutilus	FMEP325-14	KP193752
Psoroptidae	*Chorioptes sp. SW-2010a*	GBCH4531-10	FJ907504
Psoroptidae	*Psoroptes cuniculi*	GBCH4532-10	FJ907499
Pterolichidae	Grallobia fulicae	GBCH4468-10	GQ864333
Pterolichidae	*Xoloptes blaszaki*	GBCH4452-10	GQ864350
Pteronyssidae	Pteronyssoides motacillae	FMEP076-14	KP193754
Pteronyssidae	Pteronyssoides parinus	FMEP229-14	KP193757
Pteronyssidae	Pteronyssoides piscinotus	FMEP306-14	KP193759
Pteronyssidae	Pteronyssoides striatus	FMEP329-14	KP193760
Pteronyssidae	Scutulanyssus hirundicola	FMEP027-14	KP193762
Pteronyssidae	Scutulanyssus obscurus	FMEP310-14	KP193763
Pteronyssidae	*Sturnotrogus truncatus*	GBCH4444-10	GQ864358
Pyroglyphidae	*Dermatophagoides farinae*	GBCH5654-13	GQ465336
Pyroglyphidae	*Dermatophagoides pteronyssinus*	GBCH3949-09	EU884425
Pyroglyphidae	Euroglyphus sp. AMUEUR1506	GBCH4456-10	GQ864346
Quadroppiidae	*Quadroppia*	CHACA1085-10	HM907126
Quadroppiidae	*Quadroppia*	MYMCA1519-11	JX834047
Quadroppiidae	*Quadroppia*	MYMCE377-12	JX834979
Quadroppiidae	*Quadroppia*	MYMCF528-12	JX834037
Sarcoptidae	Sarcoptes scabiei	GBA16271-14	KJ748527
Scheloribatidae	*Scheloribates*	CHACA1114-10	JX836556
Scheloribatidae	*Scheloribates*	CHACC005-10	HM907407
Scheloribatidae	*Scheloribates*	CHACC200-10	HQ941526

Scheloribatidae	*Scheloribates*	CNPCD077-13	KM840143
Scheloribatidae	*Scheloribates*	MYMCA1019-11	JX837135
Scheloribatidae	*Scheloribates*	MYMCA507-11	JX837868
Scheloribatidae	*Scheloribates*	MYMCF447-12	JX835925
Scheloribatidae	*Scheloribates*	MYMCF448-12	JX835981
Scheloribatidae	*Scheloribates pallidulus*	CHACA1112-10	JX834560
Scheloribatidae	*Scheloribates pallidulus*	CHACA1113-10	JX835879
Scheloribatidae	*Scheloribates pallidulus*	CHACA1115-10	JX838587
Scheloribatidae	*Scheloribates pallidulus*	CHACB042-10	HQ558355
Scheloribatidae	*Scheloribates pallidulus*	MYMCA983-11	JX837500
Scheloribatidae	*Scheloribates pallidulus*	MYMCG136-12	JX836270
Scheloribatidae	*Scheloribates pallidulus*	SSPAA7975-13	KM836085
Scheloribatidae		CNBPB542-12	KM824150
Scheloribatidae		CNBPF134-12	KM833709
Scheloribatidae		CNBPL250-13	KM834842
Scheloribatidae		CNBPL269-13	KM834435
Scheloribatidae		CNPPH1078-12	KJ444290
Scheloribatidae		JSAUG1786-12	KP979333
Scheloribatidae		MIONB138-10	KP979175
Scheloribatidae		MIONB294-10	KP979214
Scheloribatidae		MIONB295-10	KP979158
Scheloribatidae		MIONB296-10	KP979131
Scheloribatidae		MYMCA283-11	JX838131
Scheloribatidae		SSBAB1951-12	KM839914
Scheloribatidae		SSBAD3975-12	KM830436
Scheloribatidae		SSBAD5519-13	KM828992
Scheloribatidae		SSEIB4235-13	KM833233
Scheloribatidae		SSEIB8197-13	KM833456
Scheloribatidae		SSPAA6690-13	KM840630
Scheloribatidae		SSPAA7955-13	KM825441
Scheloribatidae		SSWLC3117-13	KM827698
Scutoverticidae	*Exochocepheus hungaricus*	GBCH5038-10	GU208613
Scutoverticidae	*Lamellovertex caelatus*	GBCH5045-10	GU208606
Scutoverticidae	*Provertex kuehnelti*	GBCH5041-10	GU208610
Scutoverticidae	*Provertex kuehnelti*	GBCH5042-10	GU208609
Scutoverticidae	*Provertex kuehnelti*	GBCH5043-10	GU208608
Scutoverticidae	*Provertex kuehnelti*	GBCH5044-10	GU208607
Scutoverticidae	*Scutovertex arenocolus*	GBCH5072-10	GU208579
Scutoverticidae	*Scutovertex minutus*	GACAC447-12	GQ890372
Scutoverticidae	*Scutovertex minutus*	GACAC452-12	GQ890377
Scutoverticidae	*Scutovertex minutus*	GACAC465-12	GQ890390
Scutoverticidae	*Scutovertex pannonicus*	GACAC520-12	GQ890445
Scutoverticidae	*Scutovertex pictus*	GBCH5064-10	GU208587
Scutoverticidae	*Scutovertex pictus*	GBCH5065-10	GU208586

Scutoverticidae	*Scutovertex pileatus*	GBCH5063-10	GU208588
Scutoverticidae	*Scutovertex sculptus*	GACAC471-12	GQ890396
Scutoverticidae	*Scutovertex sculptus*	GACAC473-12	GQ890398
Scutoverticidae	*Scutovertex sculptus*	GACAC488-12	GQ890413
Scutoverticidae	*Scutovertex sculptus*	GACAC490-12	GQ890415
Scutoverticidae	*Scutovertex sculptus*	GACAC493-12	GQ890418
Scutoverticidae	*Scutovertex sculptus*	GACAC496-12	GQ890421
Scutoverticidae	*Scutovertex sculptus*	GACAC498-12	GQ890423
Scutoverticidae	*Scutovertex sculptus*	GACAC501-12	GQ890426
Scutoverticidae	*Scutovertex sculptus*	GACAC504-12	GQ890429
Scutoverticidae	*Scutovertex sculptus*	GACAC505-12	GQ890430
Scutoverticidae	*Scutovertex sculptus*	GACAC514-12	GQ890439
Scutoverticidae	*Scutovertex sculptus*	GACAC515-12	GQ890440
Scutoverticidae	*Scutovertex sculptus*	GACAC517-12	GQ890442
Scutoverticidae	*Scutovertex sculptus*	GBCH5048-10	GU208603
Scutoverticidae	*Scutovertex sculptus*	GBCH5050-10	GU208601
Scutoverticidae	*Scutovertex sculptus*	GBCH5051-10	GU208600
Scutoverticidae	*Scutovertex sculptus*	GBCH5056-10	GU208595
Scutoverticidae	*Scutovertex sculptus*	GBCH5057-10	GU208594
Scutoverticidae	*Scutovertex sp. Hungary*	GBCH5060-10	GU208591
Scutoverticidae	*Scutovertex sp. ianus*	GBCH5066-10	GU208585
Scutoverticidae	*Scutovertex sp. ianus*	GBCH5068-10	GU208583
Scutoverticidae	*Scutovertex sp. ianus*	GBCH5070-10	GU208581
Scutoverticidae	*Scutovertex sp. Wangen*	GBCH5059-10	GU208592
Scutoverticidae		SSBAA3639-12	KM828405
Scutoverticidae		SSWLC062-13	KM833395
Suctobelbidae	*Suctobelbella*	CHACA955-10	HM405827
Suctobelbidae	*Suctobelbella*	CHACB020-10	HQ558340
Suctobelbidae	*Suctobelbella*	CHACB937-10	HM907363
Suctobelbidae	*Suctobelbella*	MYMCA437-11	JX838733
Suctobelbidae	*Suctobelbella*	MYMCB231-11	JX838030
Suctobelbidae	*Suctobelbella*	MYMCF917-12	JX836205
Suctobelbidae	*Suctobelbella sarekensis*	CHACA122-08	JX836501
Suctobelbidae		MYMCA055-11	JX836748
Suctobelbidae		MYMCA438-11	JX836824
Suctobelbidae		MYMCB054-11	JX838395
Suctobelbidae		MYMCB055-11	JX838452
Suctobelbidae		MYMCF199-12	JX835759
Syringobiidae	Syringobia longipenis	GBCH4453-10	GQ864349
Tectocepheidae	*Tectocepheus*	CHACA1078-10	HM907121
Tectocepheidae	*Tectocepheus*	CHACA1080-10	HM907123
Tectocepheidae	*Tectocepheus*	CHACA508-09	JX837238
Tectocepheidae	*Tectocepheus*	CHACB152-10	HQ558435
Tectocepheidae	*Tectocepheus*	CHACB379-10	JX837311

Tectocepheidae	*Tectocepheus*	CHACB534-10	JX835479
Tectocepheidae	*Tectocepheus*	CHACB576-10	HQ558682
Tectocepheidae	*Tectocepheus*	CHACB858-10	HM907310
Tectocepheidae	*Tectocepheus*	CHACC042-10	HM907442
Tectocepheidae	*Tectocepheus*	CHACC094-10	HM907483
Tectocepheidae	*Tectocepheus*	CHACC097-10	JX833948
Tectocepheidae	*Tectocepheus*	CNJAB1025-12	KM829408
Tectocepheidae	*Tectocepheus*	MIONB140-10	KP979224
Tectocepheidae	*Tectocepheus*	MYMCA169-11	JX836364
Tectocepheidae	*Tectocepheus*	MYMCA236-11	JX836369
Tectocepheidae	*Tectocepheus*	MYMCA510-11	JX833932
Tectocepheidae	*Tectocepheus*	MYMCA554-11	JX837654
Tectocepheidae	*Tectocepheus*	MYMCA862-11	JX835077
Tectocepheidae	*Tectocepheus*	MYMCA863-11	JX834356
Tectocepheidae	*Tectocepheus*	MYMCA910-11	JX838409
Tectocepheidae	*Tectocepheus*	MYMCB117-11	JX834267
Tectocepheidae	*Tectocepheus*	MYMCB148-11	JX835333
Tectocepheidae	*Tectocepheus*	MYMCB808-11	JX838657
Tectocepheidae	*Tectocepheus*	MYMCB873-11	JX836199
Tectocepheidae	*Tectocepheus*	MYMCB881-11	JX833766
Tectocepheidae	*Tectocepheus*	MYMCC197-11	JX836168
Tectocepheidae	*Tectocepheus*	MYMCC641-11	JX835305
Tectocepheidae	*Tectocepheus*	MYMCE221-12	JX834963
Tectocepheidae	*Tectocepheus*	MYMCE222-12	JX837397
Tectocepheidae	*Tectocepheus*	MYMCE321-12	JX834222
Tectocepheidae	*Tectocepheus*	MYMCE376-12	JX837707
Tectocepheidae	*Tectocepheus*	MYMCE389-12	JX838724
Tectocepheidae	*Tectocepheus*	MYMCE467-12	JX837467
Tectocepheidae	*Tectocepheus*	MYMCE635-12	JX833630
Tectocepheidae	*Tectocepheus*	MYMCE782-12	JX833697
Tectocepheidae	*Tectocepheus*	MYMCE905-12	JX838571
Tectocepheidae	*Tectocepheus*	MYMCE906-12	JX837786
Tectocepheidae	*Tectocepheus*	MYMCE916-12	JX837506
Tectocepheidae	*Tectocepheus*	MYMCF134-12	JX834707
Tectocepheidae	*Tectocepheus*	MYMCF203-12	JX836521
Tectocepheidae	*Tectocepheus*	MYMCF522-12	JX835801
Tectocepheidae	*Tectocepheus*	MYMCF590-12	JX837983
Tectocepheidae	*Tectocepheus*	MYMCF726-12	JX835814
Tectocepheidae	*Tectocepheus*	MYMCG237-12	JX834474
Tectocepheidae	*Tectocepheus*	MYTMC154-09	HQ966242
Tectocepheidae	*Tectocepheus*	MYTMC176-09	HQ966246
Tectocepheidae	*Tectocepheus*	SSBAA3693-12	KM831801
Tectocepheidae	*Tectocepheus*	SSPAA7964-13	KM836514
Tectocepheidae	*Tectocepheus velatus*	MYMCC712-11	JX836542

Tectocepheidae	*Tectocepheus velatus*	MYTMC187-09	HQ966247
Tectocepheidae	*Tectocepheus velatus velatus*	MYMCG335-12	JX838392
Tegoribatidae	*Scutozetes lanceolatus*	CHACA618-09	JX837410
Tegoribatidae	*Tegoribates americanus*	CHACA218-08	JX834509
Tegoribatidae		SSBAD5571-13	KM825343
Tenuialidae		SSBAD5546-13	KM826429
Terpnacaridae		MYMCA640-11	JX838217
Terpnacaridae		MYMCC511-11	JX833715
Terpnacaridae		MYMCE116-12	JX834526
Thyrisomidae	*Banksinoma*	MYMCE875-12	JX838578
Thyrisomidae	*Banksinoma*	MYMCG636-12	JX835179
Thyrisomidae	*Banksinoma spinifera*	MYMCC169-11	JX836658
Trhypochthoniidae	*Mucronothus*	MYMCB674-11	JX834803
Trhypochthoniidae	*Trhypochthonius*	MYMCG068-12	JX836394
Trhypochthoniidae	*Trhypochthonius*	SSBAD5536-13	KM830470
Trhypochthoniidae	*Trhypochthonius*	SSBAD5558-13	KM835941
Trhypochthoniidae	*Trhypochthonius cladonicolus*	CHACA719-09	JX835381
Trhypochthoniidae	*Trhypochthonius tectorum*	CHACA177-08	JX836676
Trhypochthoniidae	*Trhypochthonius tectorum*	CHACA581-09	JX838623
Trhypochthoniidae	*Trhypochthonius tectorum*	SSEIB4561-13	KM830228
Trhypochthoniidae	*Trhypochthonius tectorum*	SSEIB4586-13	KM839025
Trhypochthoniidae		MYMCE056-12	JX835665
Trouessartiidae	Trouessartia appendiculata	FMEP315-14	KP193765
Trouessartiidae	Trouessartia bifurcata	FMEP316-14	KP193766
Trouessartiidae	Trouessartia bifurcata	FMEP317-14	KP193767
Trouessartiidae	Trouessartia inexpectata	FMEP164-14	KP193768
Trouessartiidae	Trouessartia jedliczkai	FMEP033-14	KP193786
Trouessartiidae	Trouessartia ripariae	FMEP036-14	KP193794
Trouessartiidae	Trouessartia rosterii	FMEP037-14	KP193796
Trouessartiidae	Trouessartia rubecula	FMEP040-14	KP193808
Trouessartiidae	Trouessartia simillima	FMEP348-14	KP193810
Trouessartiidae	Trouessartia sp. AMUFM627	GBCH4467-10	GQ864334
Trouessartiidae	Trouessartia swidwiensis	FMEP188-14	KP193813
Trouessartiidae	Trouessartia tenuipilata	FMEP311-14	KP193816
Trouessartiidae	Trouessartia trouessarti	FMEP017-14	KP193819
Xolalgidae	*Glaucalges attenuatus*	GBCH3318-09	EU271958
Xolalgidae	*Glaucalges sp. n. MD-2007*	GBCH3320-09	EU271956
Xolalgidae	*Ingrassia sp. n. MD-2007*	GBCH3316-09	EU271954
**Schizomida**			
Unknown family		GBCH7783-13	JN018143
Unknown family		GBCH7782-13	JN018144
Unknown family		GBCH7781-13	JN018145
Unknown family		GBCH11739-13	JX280414
**Scorpiones**			

Buthidae	*Androctonus hoggarensis*	GBCH7776-13	JN018150
Buthidae	*Androctonus mauritanicus*	GBCH6935-13	JF820097
Buthidae	*Buthus ibericus*	GBCH4364-10	GQ168520
Buthidae	*Buthus ibericus*	GBCH4357-10	GQ168527
Buthidae	*Buthus ibericus*	GBCH4354-10	GQ168530
Buthidae	*Buthus ibericus*	GBCH4347-10	GQ168537
Buthidae	*Buthus ibericus*	GBCH4346-10	GQ168538
Buthidae	*Buthus ibericus*	GBCH4342-10	GQ168542
Buthidae	*Buthus occitanus*	GBCH2387-08	EU523755
Buthidae	*Buthus occitanus*	GBCH4360-10	GQ168524
Buthidae	*Buthus sp. Sc095*	GBCH4363-10	GQ168521
Buthidae	*Buthus sp. Sc096*	GBCH4362-10	GQ168522
Buthidae	*Buthus sp. Sc1*	GBCH3421-09	FJ198055
Buthidae	*Buthus sp. Sc4*	GBCH3420-09	FJ198056
Buthidae	*Buthus*	GBCH11870-13	JQ775965
Buthidae	*Centruroides*	GBCH1946-07	AY995833
Buthidae	*Centruroides*	GBCH1953-07	AY995825
Buthidae	*Centruroides*	GBCH1945-07	AY995834
Buthidae	*Centruroides*	GBCH1949-07	AY995830
Buthidae	*Centruroides*	GBCH1952-07	AY995826
Buthidae	*Centruroides*	GBCH1089-06	AY803353
Buthidae	*Centruroides*	GBCH3771-09	AY995838
Buthidae	*Centruroides*	GBCH1944-07	AY995837
Buthidae	*Centruroides*	GBCH1950-07	AY995829
Buthidae	*Centruroides*	GBCH1948-07	AY995831
Buthidae	*Centruroides*	GBCH2511-08	EU381065
Buthidae	*Centruroides*	GBCH1947-07	AY995832
Buthidae	*Centruroides*	GBCH2527-08	EU381049
Buthidae	*Centruroides*	GBCH2520-08	EU381056
Buthidae	*Centruroides*	GBCH2512-08	EU381064
Buthidae	*Centruroides*	GBCH2495-08	EU381081
Buthidae	*Centruroides*	GBCH2494-08	EU381082
Buthidae	*Centruroides*	GBCH2492-08	EU381084
Buthidae	*Centruroides*	GBCH2484-08	EU381092
Buthidae	*Centruroides*	GBCH2469-08	EU381107
Buthidae	*Mesobuthus*	GBCH1688-06	NC_006515
Buthidae	*Mesobuthus*	GBCH3698-09	DQ340065
Buthidae	*Tityus*	GBCH4095-09	FJ525424
Buthidae	*Tityus*	GBCH7771-13	JN018155
Buthidae	*Zabius*	GBCH4098-09	FJ525421
Buthidae		GBCH6956-13	JF820076
Buthidae		GBCH6948-13	JF820084
Buthidae		GBCH6944-13	JF820088
Buthidae		GBCH6938-13	JF820094

Buthidae		GBCH7774-13	JN018152
Buthidae		GBCH7773-13	JN018153
Buthidae		GBCH7634-13	JQ514244
Chactidae	*Chactas*	GBCH7624-13	JQ514255
Chactidae	*Uroctonus*	GBCH3206-08	NC_010782
Chactidae		GBCH7770-13	JN018156
Euscorpiidae	*Euscorpius*	GBCH1036-06	AY731175
Euscorpiidae	*Euscorpius*	GBCH7743-13	JN018212
Hemiscorpiidae	*Liocheles*	RBCH026-04	DQ127506
Scorpionidae	*Heterometrus*	GBCH0449-06	AY156572
Scorpionidae	*Heterometrus*	GBCH0450-06	AY156573
Scorpionidae	*Heterometrus*	GBCH0451-06	AY156574
Scorpionidae	*Heterometrus*	GBCH0452-06	AY156575
Scorpionidae	*Nebo*	GBCH0448-06	AY156571
Scorpionidae	*Opistophthalmus*	GBCH0453-06	AY156576
Scorpionidae	*Opistophthalmus*	GBCH7630-13	JQ514248
Scorpionidae	*Opistophthalmus*	GBCH0454-06	AY156577
Scorpionidae	*Opistophthalmus*	GBCH0455-06	AY156578
Scorpionidae	*Opistophthalmus*	GBCH0456-06	AY156579
Scorpionidae	*Pandinus*	GBCH0457-06	AY156580
Scorpionidae	*Pandinus*	GBCH0458-06	AY156581
Scorpionidae	*Pandinus*	GBCH0459-06	AY156582
Scorpionidae	*Pandinus*	GBCH7627-13	JQ514251
Scorpionidae	*Pandinus*	GBCH0460-06	AY156583
Scorpionidae	*Scorpio*	GBCH0461-06	AY156584
Scorpionidae	*Scorpio*	GBCH0462-06	AY156585
Scorpionidae	*Scorpio*	GBCH3418-09	FJ198058
Scorpionidae	*Scorpio*	GBCH3417-09	FJ198059
Scorpionidae	*Scorpio*	GBCH3416-09	FJ198060
Scorpionidae	*Scorpio*	GBCH3413-09	FJ198063
Scorpionidae	*Scorpio*	GBCH3412-09	FJ198064
Vaejovidae	*Paruroctonus*	GBCH11290-13	JX909544
Vaejovidae	*Vaejovis*	GBCH11248-13	JX909586
Vaejovidae	*Vaejovis*	GBCH11244-13	JX909590
Vaejovidae	*Vaejovis*	GBCH11242-13	JX909592
Vaejovidae	*Vaejovis*	GBCH11241-13	JX909593
Vaejovidae	*Vaejovis*	GBCH11238-13	JX909596
Vaejovidae	*Vaejovis*	GBCH11237-13	JX909597
Vaejovidae	*Vaejovis*	GBCH11236-13	JX909598
Vaejovidae	*Vaejovis*	GBCH11234-13	JX909600
Vaejovidae	*Vaejovis*	GBCH11227-13	JX909607
Vaejovidae	*Vaejovis*	GBCH11226-13	JX909608
Vaejovidae	*Vaejovis*	GBCH11225-13	JX909609
Vaejovidae	*Vaejovis*	GBCH11228-13	JX909606

Vaejovidae	*Vaejovis*	GBCH11224-13	JX909610
Vaejovidae	*Vaejovis*	GBCH11289-13	JX909545
Vaejovidae	*Vaejovis*	GBCH11288-13	JX909546
Vaejovidae	*Vaejovis*	GBCH11287-13	JX909547
Vaejovidae	*Vaejovis*	GBCH11286-13	JX909548
Vaejovidae	*Vaejovis*	GBCH11284-13	JX909550
Vaejovidae	*Vaejovis*	GBCH11282-13	JX909552
Vaejovidae	*Vaejovis*	GBCH11280-13	JX909554
Vaejovidae	*Vaejovis*	GBCH11279-13	JX909555
Vaejovidae	*Vaejovis*	GBCH11278-13	JX909556
Vaejovidae	*Vaejovis*	GBCH11277-13	JX909557
Vaejovidae	*Vaejovis*	GBCH11276-13	JX909558
Vaejovidae	*Vaejovis*	GBCH11274-13	JX909560
Vaejovidae	*Vaejovis*	GBCH11273-13	JX909561
Vaejovidae	*Vaejovis*	GBCH11270-13	JX909564
Vaejovidae	*Vaejovis*	GBCH11269-13	JX909565
Vaejovidae	*Vaejovis*	GBCH11268-13	JX909566
Vaejovidae	*Vaejovis*	GBCH11265-13	JX909569
Vaejovidae	*Vaejovis*	GBCH11264-13	JX909570
Vaejovidae	*Vaejovis*	GBCH11263-13	JX909571
Vaejovidae	*Vaejovis*	GBCH11262-13	JX909572
Vaejovidae	*Vaejovis*	GBCH11260-13	JX909574
Vaejovidae	*Vaejovis*	GBCH11258-13	JX909576
Vaejovidae	*Vaejovis*	GBCH11257-13	JX909577
Vaejovidae	*Vaejovis*	GBCH11256-13	JX909578
Vaejovidae	*Vaejovis*	GBCH11253-13	JX909581
Vaejovidae	*Vaejovis*	GBCH11251-13	JX909583
Vaejovidae	*Vaejovis*	GBCH11249-13	JX909585
Vaejovidae	*Vaejovis*	GBCH11223-13	JX909611
Vaejovidae	*Vaejovis*	GBCH11221-13	JX909613
Vaejovidae	*Vaejovis*	GBCH11220-13	JX909614
Vaejovidae	*Vaejovis*	GBCH11219-13	JX909615
Vaejovidae	*Vaejovis*	GBCH11247-13	JX909587
Vaejovidae	*Vaejovis*	GBCH11245-13	JX909589
Vaejovidae	*Vaejovis*	GBCH11233-13	JX909601
Vaejovidae	*Vaejovis*	GBCH11232-13	JX909602
Vaejovidae	*Vaejovis*	GBCH11231-13	JX909603
Vaejovidae	*Vaejovis*	GBCH11230-13	JX909604
Vaejovidae	*Vaejovis*	GBCH11229-13	JX909605
**Solifugae**			
Ammotrechidae	*Nothopuga*	GBCH2404-08	EU024482
Eremobatidae	*Eremobates*	GBCH2391-08	EU520642
Galeodidae		GBCH7760-13	JN018166
Rhagodidae		GBCH7759-13	JN018167

Rhagodidae		GBCH7758-13	JN018168
**Trombidiformes**			
Acalyptonotidae	*Paenecalyptonotus*	MYMCB670-11	JX837830
Anystidae		CHACA1048-10	HM907100
Anystidae		CHACB1132-10	HM907222
Anystidae		ARCN012-10	HQ924258
Anystidae		ARCN023-10	HQ924267
Anystidae		ARCN031-10	HQ924274
Anystidae		ARCN044-10	HQ924280
Anystidae		ARCN095-10	HQ924314
Anystidae		ARCN165-10	HQ924367
Anystidae		CHACB443-10	JX834136
Anystidae		CHACA552-09	JX836355
Anystidae		CHACA1101-10	JX837579
Anystidae		SSBAD3132-12	KM824888
Anystidae		CNPAL850-13	KM824907
Anystidae		SSWLE473-13	KM825542
Anystidae		SSBAA2018-12	KM827289
Anystidae		SSBAD3986-12	KM828543
Anystidae		CNSLT174-13	KM828644
Anystidae		SSBAF5229-13	KM828973
Anystidae		CNJAD1827-12	KM829551
Anystidae		SSBAD3142-12	KM829664
Anystidae		CNBAE291-12	KM830158
Anystidae		CNEIF2385-12	KM830842
Anystidae		SSBAF5819-13	KM831334
Anystidae		CNSLC552-12	KM832203
Anystidae		SSBAA2017-12	KM832572
Anystidae		CNSLJ418-12	KM834006
Anystidae		SSWLE2957-13	KM835908
Anystidae		CNBPD598-12	KM836715
Anystidae		SSBAD3143-12	KM836889
Anystidae		SSBAC2359-12	KM837434
Anystidae		CNJAD2324-12	KM837891
Anystidae		SSBAD4006-12	KM838057
Anystidae		CNJAA876-12	KM839881
Anystidae		SSBAA2087-12	KM840801
Anystidae		ASAMT007-12	KP979234
Anystidae		MIONB189-10	KP979124
Anystidae		MIONB146-10	KP979182
Anystidae		MIONB150-10	KP979280
Anystidae		MIONB403-10	KP979235
Anystidae		MIONB186-10	KP979142
Anystidae		MIONB301-10	KP979196

Anystidae		MIONB241-10	KP979147
Anystidae		MIONB242-10	KP979239
Anystidae		MIONB300-10	KP979259
Anystidae		MIONB312-10	KP979130
Anystidae		MIONB325-10	KP979195
Anystidae		MIONB329-10	KP979157
Anystidae		MIONB332-10	KP979286
Anystidae		MIONB331-10	KP979170
Anystidae		MIONB335-10	KP979216
Anystidae		MIONB343-10	KP979164
Anystidae		MIONB344-10	KP979356
Anystidae		JSMAY1602-12	KP979352
Anystidae		MIAUS032-12	KP979338
Anystidae		ASAMT005-12	KP979209
Anystidae		MIAUS018-12	KP979128
Anystidae		PHAUG1668-11	KP979271
Anystidae		ASAMT009-12	KP979153
Anystidae		JSOIE054-12	KP979289
Anystidae		ASAMT029-12	KP979296
Anystidae		MIONB416-10	KP979266
Arrenuridae	*Arrenurus*	ARCN015-10	HQ924260
Arrenuridae	*Arrenurus*	ARCN090-10	HQ924310
Arrenuridae	*Arrenurus*	SSPAA2281-13	KM827656
Arrenuridae	*Arrenurus*	ARCN070-10	HQ924293
Arrenuridae	*Arrenurus*	ARCN088-10	HQ924308
Arrenuridae	*Arrenurus*	ARCN092-10	HQ924312
Arrenuridae	*Arrenurus*	CNPPB2356-12	KJ088280
Arrenuridae	*Arrenurus*	MIONB453-11	KM825054
Arrenuridae	*Arrenurus*	SSBAF4469-13	KM828892
Arrenuridae	*Arrenurus*	SSJAB3355-13	KM830409
Arrenuridae	*Arrenurus*	SSPAA2284-13	KM833746
Arrenuridae	*Arrenurus*	SSJAB3350-13	KM833781
Arrenuridae	*Arrenurus*	SSBAB1129-12	KM838262
Arrenuridae	*Arrenurus*	SSPAA6618-13	KM838879
Arrenuridae	*Arrenurus*	CNSLP1089-13	KP979159
Arrenuridae		SSBAD3084-12	KM831888
Bdellidae	*Biscuris*	MYMCB524-11	JX833923
Bdellidae	*Biscuris*	MYMCA466-11	JX835361
Bdellidae	*Biscuris*	MIONB183-10	KP979287
Bdellidae	*Cyta*	CHACA970-10	HM405835
Bdellidae	*Cyta*	MIONB023-10	HM887567
Bdellidae	*Cyta*	CHACA1047-10	HM907099
Bdellidae	*Cyta*	CHACA1072-10	HM907115
Bdellidae	*Cyta*	CHACB092-10	HQ558391

Bdellidae	*Cyta*	CHACB270-10	HQ558508
Bdellidae	*Cyta*	CHACB383-10	HQ558556
Bdellidae	*Cyta*	CHACC263-10	HQ941566
Bdellidae	*Cyta*	CHACC264-10	HQ941567
Bdellidae	*Cyta*	CHACA1184-10	JX833739
Bdellidae	*Cyta*	CHACA571-09	JX833872
Bdellidae	*Cyta*	MYMCB128-11	JX833982
Bdellidae	*Cyta*	CHACA129-08	JX834538
Bdellidae	*Cyta*	MYMCA560-11	JX834982
Bdellidae	*Cyta*	MYMCE450-12	JX835312
Bdellidae	*Cyta*	MYMCF711-12	JX835351
Bdellidae	*Cyta*	CHACA815-09	JX835762
Bdellidae	*Cyta*	MYMCE490-12	JX835786
Bdellidae	*Cyta*	MYMCA117-11	JX836098
Bdellidae	*Cyta*	MYMCC782-11	JX836112
Bdellidae	*Cyta*	MYMCB270-11	JX836118
Bdellidae	*Cyta*	MYMCA1332-11	JX836245
Bdellidae	*Cyta*	MYMCE675-12	JX837247
Bdellidae	*Cyta*	MYMCC777-11	JX837432
Bdellidae	*Cyta*	MYMCA334-11	JX837436
Bdellidae	*Cyta*	MYMCA1404-11	JX837993
Bdellidae	*Cyta*	CHACA1183-10	JX838055
Bdellidae	*Cyta*	MYMCA1333-11	JX838419
Bdellidae	*Cyta*	MYMCA745-11	JX838757
Bdellidae	*Cyta*	CNPPA4084-12	KJ088714
Bdellidae	*Cyta*	SSJAC1533-13	KM826005
Bdellidae	*Cyta*	SSPAA7935-13	KM826185
Bdellidae	*Cyta*	SSPAA7802-13	KM831566
Bdellidae	*Cyta*	SSBAA2076-12	KM832195
Bdellidae	*Cyta*	SSBAA3665-12	KM832972
Bdellidae	*Cyta*	CNJAC1492-12	KM833417
Bdellidae	*Cyta*	CNGIB579-12	KM833873
Bdellidae	*Cyta*	SSEIB8192-13	KM834202
Bdellidae	*Cyta*	CNBAD690-12	KM834631
Bdellidae	*Cyta*	CNBAC179-12	KM834974
Bdellidae	*Cyta*	CNGIB586-12	KM835677
Bdellidae	*Cyta*	CNBAD686-12	KM838029
Bdellidae	*Cyta*	SSPAC12453-13	KM840607
Bdellidae	*Cyta*	CNBAD673-12	KM840719
Bdellidae	*Cyta*	CHACA542-09	KP979133
Bdellidae	*Cyta*	RBINA4468-13	KP979237
Bdellidae	*Cyta*	MIONB533-11	KP979206
Bdellidae		CHACB111-10	HQ558404
Bdellidae		CNPPB2355-12	KJ090776

Bdellidae		CNRMD2666-12	KM826530
Bdellidae		SSWLC3124-13	KM830847
Bdellidae		SSWLD8326-13	KM834671
Bdellidae		SSPAA6689-13	KM836316
Bdellidae		SSPAB8742-13	KM840153
Bdellidae		MIONB286-10	KP979178
Bdellidae		MIONB280-10	KP979307
Bdellidae		MIONB206-10	KP979132
Bdellidae		MIONB224-10	KP979185
Bdellidae		MIONB256-10	KP979350
Bdellidae		MIONB279-10	KP979294
Bdellidae		RBINA3933-13	KP979283
Bdellidae		RBINA5704-13	KP979151
Calyptostomatidae	*Calyptostoma*	MYMCE120-12	JX834838
Calyptostomatidae	*Calyptostoma*	MHMIT057-07	KP979177
Cheyletidae	*Cheyletus*	GBCH10760-13	KC507896
Cheyletidae	*Cheyletus*	GBCH10755-13	KC507901
Cheyletidae	*Cheyletus*	GBCH10754-13	KC507902
Cheyletidae		MYMCB157-11	JX835196
Cunaxidae		CHACB1053-10	HM907178
Cunaxidae		CHACB1150-10	HM907237
Cunaxidae		CHACB455-10	HQ558605
Cunaxidae		MYMCC455-11	JX833733
Cunaxidae		MYMCE755-12	JX833734
Cunaxidae		MYMCC331-11	JX834714
Cunaxidae		MYMCD126-11	JX834994
Cunaxidae		MYMCC344-11	JX835141
Cunaxidae		MYMCA309-11	JX835794
Cunaxidae		MYMCG569-12	JX836390
Cunaxidae		MYMCG029-12	JX836652
Cunaxidae		MYMCB721-11	JX836798
Cunaxidae		MYMCA561-11	JX837203
Cunaxidae		CHACA105-08	JX837389
Cunaxidae		MYMCA924-11	JX837963
Cunaxidae		MYMCC723-11	JX838722
Cunaxidae		CNPPH1076-12	KJ443951
Cunaxidae		CNPAF888-13	KM824477
Cunaxidae		SSPAA2215-13	KM826575
Cunaxidae		SSWLF3572-13	KM837062
Cunaxidae		MIONB272-10	KP979186
Cunaxidae		SMTPB1856-13	KP979126
Ereynetidae		MYMCE411-12	JX834113
Ereynetidae		MYMCG645-12	JX836461
Ereynetidae		MYMCA742-11	JX837133

Ereynetidae		MYMCG293-12	JX837345
Ereynetidae		SSBAB104-12	KM831555
Eriophyidae	*Aceria*	GBCH3757-09	EF409415
Eriophyidae	*Aceria*	GBCH1881-07	EF409416
Eriophyidae		MYMCE505-12	JX833876
Eriophyidae		MYMCE743-12	JX834049
Eriophyidae		MYMCE733-12	JX834574
Eriophyidae		MYMCE732-12	JX836212
Eriophyidae		MYMCE519-12	JX836644
Eriophyidae		MYMCE504-12	JX836814
Eriophyidae		SSJAF7371-13	KM830432
Erythraeidae	*Abrolophus*	CHACB928-10	HM907355
Erythraeidae	*Abrolophus*	MYMCC476-11	JX833624
Erythraeidae	*Abrolophus*	MYMCA293-11	JX836619
Erythraeidae	*Abrolophus*	MYMCC425-11	JX838223
Erythraeidae	*Abrolophus*	MYMCC022-11	JX838576
Erythraeidae	*Abrolophus*	SSJAD3357-13	KM824154
Erythraeidae	*Abrolophus*	CNRMA1044-12	KM825660
Erythraeidae	*Abrolophus*	SSWLE474-13	KM832147
Erythraeidae	*Abrolophus*	JSJUN2329-12	KP979303
Erythraeidae	*Abrolophus*	MIAUS057-12	KP979334
Erythraeidae	*Abrolophus*	RBINA4085-13	KP979281
Erythraeidae	*Balaustium*	CNPAC504-13	KM837049
Erythraeidae	*Erythraeus*	MYMCC533-11	JX837954
Erythraeidae	*Leptus*	CHACA1049-10	HM907101
Erythraeidae	*Leptus*	ARCN123-10	HQ924339
Erythraeidae	*Leptus*	CHACA1151-10	JX834325
Erythraeidae	*Leptus*	MYMCA027-11	JX836425
Erythraeidae	*Leptus*	CNPPC1937-12	KJ092146
Erythraeidae	*Leptus*	CNPPH1067-12	KJ444206
Erythraeidae	*Leptus*	SSEIC1283-13	KM824035
Erythraeidae	*Leptus*	SSBAE3015-13	KM824509
Erythraeidae	*Leptus*	SSJAF8379-13	KM824912
Erythraeidae	*Leptus*	SSPAA6687-13	KM825020
Erythraeidae	*Leptus*	SSPAA7762-13	KM825436
Erythraeidae	*Leptus*	SSPAC7891-13	KM825485
Erythraeidae	*Leptus*	SSEIA7684-13	KM825751
Erythraeidae	*Leptus*	SSPAC7826-13	KM826282
Erythraeidae	*Leptus*	CNEIE1940-12	KM826397
Erythraeidae	*Leptus*	CNRMC1509-12	KM826847
Erythraeidae	*Leptus*	SSPAA2216-13	KM827626
Erythraeidae	*Leptus*	CNPAF880-13	KM827660
Erythraeidae	*Leptus*	SSJAF7400-13	KM828998
Erythraeidae	*Leptus*	SSJAF5587-13	KM829119

Erythraeidae	*Leptus*		SSBAC3352-12	KM829398
Erythraeidae	*Leptus*		CNRME4787-12	KM829989
Erythraeidae	*Leptus*		CNRME4752-12	KM830192
Erythraeidae	*Leptus*		CNRMC1515-12	KM830792
Erythraeidae	*Leptus*		SSPAA7775-13	KM831231
Erythraeidae	*Leptus*		SSPAC7400-13	KM831655
Erythraeidae	*Leptus*		CNPAF897-13	KM832489
Erythraeidae	*Leptus*		CNSLQ334-13	KM832503
Erythraeidae	*Leptus*		MIONB516-11	KM832504
Erythraeidae	*Leptus*		SSPAA7953-13	KM833169
Erythraeidae	*Leptus*		SSWLE482-13	KM833674
Erythraeidae	*Leptus*		SSPAA7951-13	KM834347
Erythraeidae	*Leptus*		CNSLI054-12	KM835577
Erythraeidae	*Leptus*		CNPAF873-13	KM836339
Erythraeidae	*Leptus*		CNPAC539-13	KM836485
Erythraeidae	*Leptus*		SSPAB3165-13	KM836815
Erythraeidae	*Leptus*		CNRMF3253-12	KM836893
Erythraeidae	*Leptus*		CNJAD1850-12	KM837987
Erythraeidae	*Leptus*		SSPAC2354-13	KM838888
Erythraeidae	*Leptus*		CNRMC1511-12	KM840317
Erythraeidae	*Leptus*		MYMCE212-12	KP979295
Erythraeidae	*Leptus*		MIONB412-10	KP979213
Erythraeidae	*Leptus*		NCCE027-11	KP979246
Erythraeidae	*Leptus*		JSMAY1559-12	KP979270
Erythraeidae	*Leptus*		MIAUS046-12	KP979210
Erythraeidae	*Leptus*		MIAUS037-12	KP979279
Erythraeidae	*Leptus*		MIAUS031-12	KP979304
Erythraeidae	*Leptus*		NCCE022-11	KP979123
Erythraeidae	*Leptus*		SSPAA6691-13	KP979238
Erythraeidae	*Leptus*		MIONB411-10	KP979264
Erythraeidae			ARCN022-10	HQ924266
Erythraeidae			ARCN034-10	HQ924277
Erythraeidae			ARCN047-10	HQ924281
Erythraeidae			ARCN050-10	HQ924283
Erythraeidae			ARCN078-10	HQ924300
Erythraeidae			ARCN083-10	HQ924304
Erythraeidae			ARCN097-10	HQ924315
Erythraeidae			ARCN107-10	HQ924325
Erythraeidae			ARCN110-10	HQ924328
Erythraeidae			ARCN111-10	HQ924329
Erythraeidae			ARCN117-10	HQ924334
Erythraeidae			ARCN122-10	HQ924338
Erythraeidae			ARCN130-10	HQ924345
Erythraeidae			ARCN155-10	HQ924361

Erythraeidae	MYMCC421-11	JX834006
Erythraeidae	MYMCB680-11	JX835178
Erythraeidae	CHACA1132-10	JX837639
Erythraeidae	CNPPD2644-12	KJ089103
Erythraeidae	CNPPB2362-12	KJ089243
Erythraeidae	CNPPC1935-12	KJ090430
Erythraeidae	CNPPC1939-12	KJ090671
Erythraeidae	SSEIB4243-13	KM824022
Erythraeidae	ARCN156-10	KM824515
Erythraeidae	SSBAE3005-13	KM826115
Erythraeidae	SSJAF5560-13	KM826204
Erythraeidae	CNPCD125-13	KM827100
Erythraeidae	SSEIC982-13	KM827496
Erythraeidae	CNWLE2572-13	KM828015
Erythraeidae	CNSLD672-12	KM828528
Erythraeidae	CNBAD677-12	KM828663
Erythraeidae	SSBAB156-12	KM829400
Erythraeidae	CNEIF2381-12	KM830747
Erythraeidae	SSJAB1871-13	KM831010
Erythraeidae	SSWLF715-13	KM831150
Erythraeidae	CNPCF124-13	KM831681
Erythraeidae	SSEIA7685-13	KM832917
Erythraeidae	MIONB472-11	KM833210
Erythraeidae	SSWLF2941-13	KM834496
Erythraeidae	CNRMF3246-12	KM834768
Erythraeidae	SSWLE453-13	KM834809
Erythraeidae	CNRMF3203-12	KM835523
Erythraeidae	SSJAB3049-13	KM835730
Erythraeidae	SSWLB5364-13	KM836290
Erythraeidae	CNBPF124-12	KM836887
Erythraeidae	MIONB465-11	KM837360
Erythraeidae	SSJAF3169-13	KM838851
Erythraeidae	CNPAF988-13	KM838868
Erythraeidae	SSBAC2369-12	KM840718
Erythraeidae	MIONB097-10	KP979327
Erythraeidae	MIONB109-10	KP979141
Erythraeidae	MIONB208-10	KP979174
Erythraeidae	MIONB147-10	KP979225
Erythraeidae	ASAMT049-12	KP979192
Erythraeidae	MIONB176-10	KP979313
Erythraeidae	MIONB205-10	KP979249
Erythraeidae	MIONB313-10	KP979193
Erythraeidae	MIONB316-10	KP979252
Erythraeidae	MIONB334-10	KP979301

Erythraeidae		MIONB532-11	KP979345
Erythraeidae		JSAUG1827-12	KP979231
Erythraeidae		MIAUS067-12	KP979262
Erythraeidae		MIONB314-10	KP979292
Erythraeidae		MIONB210-10	KP979199
Eupodidae	*Eupodes*	CHACB170-10	HQ558451
Eupodidae	*Eupodes*	MYTMC010-09	HQ966221
Eupodidae	*Eupodes*	MYMCE239-12	JX834412
Eupodidae	*Eupodes*	MYMCA970-11	JX835055
Eupodidae	*Eupodes*	MYMCC110-11	JX836220
Eupodidae	*Eupodes*	MYMCF712-12	JX837151
Eupodidae	*Eupodes*	MYMCG299-12	JX838202
Eupodidae	*Eupodes*	CHACA1157-10	JX838214
Eupodidae	*Eupodes*	MIONB260-10	KP979325
Eupodidae	*Eupodes*	RBINA3915-13	KP979299
Eupodidae	*Linopodes*	CHACB603-10	HQ558698
Eupodidae	*Linopodes*	MYMCD047-11	JX833961
Eupodidae	*Linopodes*	CHACA540-09	JX834232
Eupodidae	*Linopodes*	MYMCC839-11	JX834402
Eupodidae	*Linopodes*	MYMCC703-11	JX834916
Eupodidae	*Linopodes*	MYMCD036-11	JX837193
Eupodidae	*Linopodes*	MYMCB022-11	JX837638
Eupodidae	*Linopodes*	MYMCB184-11	JX838186
Eupodidae	*Linopodes*	MYMCB893-11	JX838280
Eupodidae	*Linopodes*	SSPAC11103-13	KM825439
Eupodidae	*Linopodes*	CNRME4712-12	KM827792
Eupodidae	*Linopodes*	CNEIF2394-12	KM828220
Eupodidae	*Linopodes*	SSJAC990-13	KM828765
Eupodidae	*Linopodes*	CNRME4796-12	KM829208
Eupodidae	*Linopodes*	SSPAA7823-13	KM831208
Eupodidae	*Linopodes*	SSPAB980-13	KM831734
Eupodidae	*Linopodes*	CNRME4731-12	KM835967
Eupodidae	*Linopodes*	SSPAA7826-13	KM837120
Eupodidae	*Linopodes*	SSPAA7956-13	KM839684
Eupodidae		CHACA965-10	HM405831
Eupodidae		CHACA1055-10	HM907106
Eupodidae		CHACB1020-10	HM907154
Eupodidae		CHACB1071-10	HM907188
Eupodidae		CHACB1121-10	HM907214
Eupodidae		CHACB1122-10	HM907215
Eupodidae		CHACB1210-10	HM907279
Eupodidae		CHACB856-10	HM907309
Eupodidae		CHACB940-10	HM907364
Eupodidae		CHACB941-10	HM907365

Eupodidae	CHACB998-10	HM907403
Eupodidae	CHACB999-10	HM907404
Eupodidae	CHACB460-10	HQ558606
Eupodidae	CHACB512-10	HQ558638
Eupodidae	CHACB634-10	HQ558717
Eupodidae	CHACB747-10	HQ558784
Eupodidae	CHACC236-10	HQ941544
Eupodidae	CHACC246-10	HQ941551
Eupodidae	MYTMC013-09	HQ966222
Eupodidae	MYTMC092-09	HQ966234
Eupodidae	MYMCA783-11	JX833677
Eupodidae	MYMCB789-11	JX833921
Eupodidae	MYMCB654-11	JX834036
Eupodidae	MYMCC729-11	JX834167
Eupodidae	MYMCD085-11	JX834357
Eupodidae	MYMCA359-11	JX834384
Eupodidae	MYMCD044-11	JX834391
Eupodidae	MYMCE288-12	JX834440
Eupodidae	MYMCD108-11	JX834807
Eupodidae	MYMCB198-11	JX834824
Eupodidae	MYMCE567-12	JX834920
Eupodidae	CHACA1218-10	JX834940
Eupodidae	MYMCF031-12	JX835017
Eupodidae	MYMCD010-11	JX835274
Eupodidae	MYMCB458-11	JX836336
Eupodidae	MYMCA1170-11	JX836549
Eupodidae	MYMCE632-12	JX836730
Eupodidae	MYMCB660-11	JX836744
Eupodidae	MYMCC530-11	JX836768
Eupodidae	MYMCA759-11	JX836971
Eupodidae	MYMCB486-11	JX837093
Eupodidae	MYMCD069-11	JX837144
Eupodidae	MYMCE911-12	JX837273
Eupodidae	MYMCB457-11	JX837295
Eupodidae	MYMCB177-11	JX837299
Eupodidae	MYMCC725-11	JX837318
Eupodidae	MYMCA854-11	JX837676
Eupodidae	MYMCG398-12	JX837956
Eupodidae	MYMCB632-11	JX838008
Eupodidae	MYMCA744-11	JX838015
Eupodidae	MYMCC542-11	JX838196
Eupodidae	MYMCB631-11	JX838376
Eupodidae	MYMCE595-12	JX838497
Eupodidae	MYMCA1167-11	JX838725

Eupodidae	CNPPA4109-12	KJ086010
Eupodidae	SSJAC1598-13	KM824178
Eupodidae	SSPAB3971-13	KM824603
Eupodidae	SSEIA3012-13	KM824743
Eupodidae	SSBAD5539-13	KM824854
Eupodidae	SSJAF8638-13	KM824948
Eupodidae	SSPAB988-13	KM825090
Eupodidae	SSPAC7880-13	KM825204
Eupodidae	CNPAF862-13	KM825939
Eupodidae	SSWLF3651-13	KM825952
Eupodidae	SSJAB2159-13	KM826043
Eupodidae	SSEIB8354-13	KM826500
Eupodidae	SSWLA5299-13	KM827256
Eupodidae	SSEID063-13	KM827378
Eupodidae	SSPAB3117-13	KM827522
Eupodidae	SSBAD4728-13	KM828226
Eupodidae	CNPAP073-13	KM828527
Eupodidae	SSPAC11101-13	KM828932
Eupodidae	SSPAB4002-13	KM828945
Eupodidae	SSPAA7990-13	KM829527
Eupodidae	SSJAD3369-13	KM829574
Eupodidae	SSPAC11102-13	KM829834
Eupodidae	SSWLE2874-13	KM830023
Eupodidae	SSJAF5572-13	KM830071
Eupodidae	SSJAC1586-13	KM830078
Eupodidae	CNSLK070-12	KM830356
Eupodidae	SSBAB108-12	KM830965
Eupodidae	SSBAD5556-13	KM831581
Eupodidae	CNPAE458-13	KM831593
Eupodidae	SSBAB1952-12	KM832073
Eupodidae	SSJAB2117-13	KM832496
Eupodidae	CNSLJ025-12	KM832588
Eupodidae	SSJAC1595-13	KM832692
Eupodidae	SSJAC1515-13	KM832702
Eupodidae	SSBAD4725-13	KM832719
Eupodidae	SSWLE470-13	KM832768
Eupodidae	CNPAF909-13	KM833319
Eupodidae	CNPAF894-13	KM833569
Eupodidae	CNBAB372-12	KM833654
Eupodidae	SSJAB2122-13	KM833929
Eupodidae	SSJAC1544-13	KM834051
Eupodidae	CNBAI516-13	KM834064
Eupodidae	CNGIA136-12	KM834194
Eupodidae	SSWLD2944-13	KM834337

Eupodidae	SSWLE3153-13	KM834419
Eupodidae	CNBAI500-13	KM834491
Eupodidae	SSBAB131-12	KM834720
Eupodidae	SSJAC1545-13	KM834885
Eupodidae	SSBAB1950-12	KM835074
Eupodidae	SSPAC10917-13	KM835131
Eupodidae	SSBAE3811-13	KM835540
Eupodidae	CNBPD599-12	KM835765
Eupodidae	CNPAE449-13	KM836074
Eupodidae	SSJAF7320-13	KM836205
Eupodidae	SSJAC974-13	KM836226
Eupodidae	SSJAB1911-13	KM836667
Eupodidae	SSWLE481-13	KM836897
Eupodidae	SSWLE3182-13	KM836995
Eupodidae	SSWLE480-13	KM837617
Eupodidae	SSPAC10887-13	KM837794
Eupodidae	SSWLC4151-13	KM838323
Eupodidae	SSJAC1580-13	KM838375
Eupodidae	SSJAF8356-13	KM838573
Eupodidae	CNPAF882-13	KM838716
Eupodidae	SSEIA3024-13	KM838870
Eupodidae	SSBAB110-12	KM839164
Eupodidae	SSBAB129-12	KM839347
Eupodidae	SSJAC1590-13	KM839396
Eupodidae	CNPAL901-13	KM839498
Eupodidae	SSBAD4749-13	KM839652
Eupodidae	SSPAC11107-13	KM839706
Eupodidae	SSWLA5341-13	KM840379
Eupodidae	SSWLA5303-13	KM840408
Eupodidae	CNJAI863-12	KM840496
Eupodidae	SSJAC1541-13	KM840682
Eupodidae	MIONB129-10	KP979212
Eupodidae	MIONB281-10	KP979163
Eupodidae	MIONB173-10	KP979331
Eupodidae	MIONB231-10	KP979181
Eupodidae	MIONB261-10	KP979138
Eupodidae	MIONB273-10	KP979260
Eupodidae	MIONB274-10	KP979315
Eupodidae	MIONB290-10	KP979221
Eupodidae	MIONB308-10	KP979198
Eupodidae	MIONB310-10	KP979250
Eupodidae	NCCE009-11	KP979339
Eupodidae	RBINA5726-13	KP979265
Eupodidae	RBINA5681-13	KP979355

Eupodidae		RBINA5789-13	KP979139
Eupodidae		RBINA5699-13	KP979134
Eupodidae		RBINA5790-13	KP979207
Eupodidae		RBINA5710-13	KP979135
Eupodidae		RBINA1191-13	KP979188
Eupodidae		RBINA1195-13	KP979341
Eylaidae	*Eylais*	GACAC043-12	AB530317
Eylaidae	*Eylais*	ARCN043-10	HQ924279
Eylaidae	*Eylais*	MYMCA102-11	JX836526
Eylaidae	*Eylais*	ARSO204-08	KM828553
Eylaidae	*Eylais*	MIONB531-11	KM836347
Eylaidae	*Eylais*	MIONB529-11	KM838933
Hydrachnidae		MIONB523-11	KM829829
Hydrachnidae		MIONB530-11	KM838199
Hydryphantidae	*Hydryphantes*	MYMCE358-12	JX836743
Hydryphantidae	*Hydryphantes*	MYMCB689-11	JX838402
Hydryphantidae	*Hydryphantes*	SSJAE5668-13	KM834895
Hydryphantidae	*Todothyas*	MYMCB451-11	JX837770
Hydryphantidae		ARCN137-10	HQ924349
Hydryphantidae		SSEIB4245-13	KM824216
Hydryphantidae		SSJAE5650-13	KM835334
Hydryphantidae		PHAUG1675-11	KP979119
Hygrobatidae	*Atractides*	GBCH1840-07	EF633505
Hygrobatidae	*Atractides*	GACAC044-12	AB530318
Hygrobatidae	*Atractides*	CFWIA186-10	HQ938548
Hygrobatidae	*Atractides*	CFWIA618-10	HQ938805
Hygrobatidae	*Atractides*	CFWIA620-10	HQ938806
Hygrobatidae	*Atractides*	CFWIB563-10	HQ939595
Hygrobatidae	*Hygrobates*	GACAC003-12	AB530277
Hygrobatidae	*Hygrobates*	GACAC022-12	AB530296
Hygrobatidae	*Hygrobates*	GACAC028-12	AB530302
Hygrobatidae	*Hygrobates*	MYMCA100-11	JX834050
Lebertiidae	*Lebertia*	CFWIA165-10	HQ938529
Lebertiidae	*Lebertia*	CFWIA642-10	HQ938821
Lebertiidae	*Lebertia*	SSBAF2267-13	KM825233
Lebertiidae	*Lebertia*	SSPAA6640-13	KM827742
Lebertiidae	*Lebertia*	SSBAF2291-13	KM840164
Lebertiidae	*Lebertia*	JSMAY1598-12	KP979219
Lebertiidae	*Lebertia*	RBINA5582-13	KP979189
Limnesiidae	*Limnesia*	CNPPJ1872-12	KJ208985
Limnesiidae	*Limnesia*	SSBAF2277-13	KM824097
Limnesiidae	*Limnesia*	SSJAE1649-13	KM829058
Limnesiidae	*Limnesia*	SSPAA2255-13	KM829634
Limnesiidae	*Limnesia*	SSPAA5627-13	KM833097

Limnesiidae	*Limnesia*	CNPAD554-13	KM837294
Limnesiidae		SSPAA2236-13	KM824023
Limnesiidae		CNBPL292-13	KM825003
Limnesiidae		SSPAA5638-13	KM829177
Limnesiidae		SSPAC13790-13	KM833430
Microtrombidiidae		MYMCE124-12	JX833738
Microtrombidiidae		MYMCE123-12	JX835070
Microtrombidiidae		MYMCA317-11	JX835192
Microtrombidiidae		CHACA048-08	JX836314
Microtrombidiidae		MYMCB629-11	JX836793
Microtrombidiidae		MYMCE534-12	JX837087
Microtrombidiidae		MYMCG031-12	JX837924
Microtrombidiidae		MYMCB914-11	JX837943
Microtrombidiidae		CNPPD2664-12	KJ090683
Microtrombidiidae		CNPPE2139-12	KJ165845
Microtrombidiidae		CNPPH1074-12	KJ637633
Microtrombidiidae		CNPAD841-13	KM830088
Microtrombidiidae		SSPAA2292-13	KM835177
Microtrombidiidae		SSBAF4466-13	KM837038
Mideopsidae	*Mideopsis*	SSPAC13791-13	KM824355
Mideopsidae	*Mideopsis*	SSPAA2266-13	KM826475
Mideopsidae	*Mideopsis*	SSWLE3970-13	KM827481
Penthaleidae	*Penthaleus*	MYMCB890-11	JX834200
Penthaleidae	*Penthaleus*	MYMCB891-11	JX836957
Penthaleidae	*Penthaleus*	CHACA266-08	JX838308
Penthaleidae		CHACA1090-10	HM907130
Penthaleidae		CHACB1000-10	HM907138
Penthaleidae		CHACB244-10	HQ558489
Penthaleidae		MYMCC727-11	JX837481
Penthalodidae		MYMCB462-11	JX834380
Penthalodidae		MYMCB690-11	JX835654
Penthalodidae		MYMCB007-11	JX835833
Penthalodidae		MYMCD043-11	JX836002
Penthalodidae		MYMCA338-11	JX836189
Penthalodidae		MYMCF216-12	JX836642
Penthalodidae		MYMCB004-11	JX836981
Penthalodidae		CHACA529-09	JX837415
Penthalodidae		MYMCE565-12	JX837522
Penthalodidae		MYMCA335-11	JX837874
Penthalodidae		MYMCC129-11	JX838597
Penthalodidae		MIONB266-10	KP979171
Pionidae	*Forelia*	CNPAC311-13	KM826800
Pionidae	*Piona*	ARCN069-10	HQ924292
Pionidae	*Piona*	MYMCA110-11	JX835088

Pionidae	*Piona*	MYMCE363-12	JX836721
Pionidae	*Piona*	CNGIB598-12	KM825988
Pionidae	*Piona*	CNPAC559-13	KM827696
Pionidae	*Piona*	SSBAF2268-13	KM834304
Pionidae	*Piona*	CNPAB275-13	KM836392
Pionidae	*Piona*	CNPAF868-13	KM839625
Pionidae		ARCN068-10	HQ924291
Pionidae		CNPPC1931-12	KJ083325
Pionidae		SSJAE3140-13	KM824353
Pionidae		CNJAF1921-12	KM826914
Pionidae		SSPAB957-13	KM830961
Pionidae		SSPAB4021-13	KM835270
Pionidae		SSPAA2307-13	KM838383
Protziidae	*Protzia*	CFWIA644-10	HQ938823
Pyemotidae	*Pyemotes*	MYMCE607-12	JX835637
Pyemotidae	*Pyemotes*	MYMCA1211-11	JX837857
Rhagidiidae		CHACA983-10	HM405847
Rhagidiidae		CHACA985-10	HM405848
Rhagidiidae		CHACB1054-10	HM907179
Rhagidiidae		CHACB056-10	HQ558365
Rhagidiidae		CHACB169-10	HQ558450
Rhagidiidae		MYMCB790-11	JX833657
Rhagidiidae		MYMCC557-11	JX833703
Rhagidiidae		MYMCB195-11	JX833754
Rhagidiidae		MYMCA220-11	JX833850
Rhagidiidae		MYMCA221-11	JX833907
Rhagidiidae		MYMCB685-11	JX834029
Rhagidiidae		MYMCC112-11	JX834056
Rhagidiidae		MYMCB648-11	JX834817
Rhagidiidae		MYMCB470-11	JX834990
Rhagidiidae		CHACB464-10	JX835231
Rhagidiidae		CHACA1209-10	JX835242
Rhagidiidae		MYMCB508-11	JX835422
Rhagidiidae		MYMCA889-11	JX835611
Rhagidiidae		MYMCA1201-11	JX835639
Rhagidiidae		MYMCA1244-11	JX835792
Rhagidiidae		MYMCF008-12	JX835920
Rhagidiidae		CHACA1217-10	JX836080
Rhagidiidae		CHACA454-09	JX836087
Rhagidiidae		MYMCC697-11	JX836173
Rhagidiidae		MYMCA219-11	JX836320
Rhagidiidae		MYMCC750-11	JX836417
Rhagidiidae		MYMCC284-11	JX836469
Rhagidiidae		MYMCD031-11	JX836583

Rhagidiidae	MYMCA875-11	JX836988
Rhagidiidae	MYMCG562-12	JX837009
Rhagidiidae	MYMCB684-11	JX837034
Rhagidiidae	MYMCB475-11	JX837057
Rhagidiidae	MYMCA385-11	JX837293
Rhagidiidae	MYMCF700-12	JX837329
Rhagidiidae	CHACA373-08	JX837468
Rhagidiidae	MYMCC405-11	JX837627
Rhagidiidae	CHACA327-08	JX837726
Rhagidiidae	CHACA1211-10	JX838080
Rhagidiidae	MYMCB217-11	JX838249
Rhagidiidae	MYMCA984-11	JX838267
Rhagidiidae	CHACA1216-10	JX838393
Rhagidiidae	MYMCA876-11	JX838425
Rhagidiidae	MYMCB463-11	JX838427
Rhagidiidae	MYMCB434-11	JX838439
Rhagidiidae	MYMCG089-12	JX838451
Rhagidiidae	MYMCD054-11	JX838551
Rhagidiidae	SSEIA3053-13	KM824038
Rhagidiidae	SSPAC10912-13	KM824805
Rhagidiidae	SSWLC4129-13	KM826887
Rhagidiidae	SSJAF8642-13	KM827411
Rhagidiidae	SSEIA3042-13	KM827836
Rhagidiidae	SSJAB1883-13	KM829244
Rhagidiidae	SSBAE4272-13	KM830374
Rhagidiidae	SSBAB1194-12	KM831606
Rhagidiidae	SSEIB8331-13	KM831822
Rhagidiidae	SSWLC4152-13	KM831858
Rhagidiidae	SSJAF7424-13	KM831906
Rhagidiidae	SSWLA5302-13	KM834033
Rhagidiidae	SSBAB1216-12	KM834373
Rhagidiidae	SSWLC4148-13	KM834700
Rhagidiidae	SSBAB1872-12	KM837346
Rhagidiidae	SSJAC975-13	KM838000
Rhagidiidae	MIONB154-10	KP979176
Rhagidiidae	MIONB168-10	KP979160
Rhagidiidae	MIONB171-10	KP979306
Rhagidiidae	MIONB190-10	KP979263
Rhagidiidae	MIONB227-10	KP979116
Rhagidiidae	MIONB228-10	KP979320
Rhagidiidae	MIONB257-10	KP979208
Rhagidiidae	MIONB259-10	KP979309
Rhagidiidae	SSWLD3510-13	KP979220
Rhagidiidae	RBINA3916-13	KP979161

Scutacaridae	CHACB1118-10	HM907212
Scutacaridae	CHACB081-10	HQ558387
Scutacaridae	CHACB083-10	HQ558388
Scutacaridae	CHACB370-10	HQ558548
Scutacaridae	CHACB774-10	HQ941479
Scutacaridae	MYMCE931-12	JX833924
Scutacaridae	MYMCF360-12	JX835893
Scutacaridae	MYMCE795-12	JX836677
Scutacaridae	MYMCG649-12	JX836783
Scutacaridae	MYMCE932-12	JX836952
Scutacaridae	MYMCA1382-11	JX836959
Scutacaridae	CNPPF1050-12	KJ165455
Scutacaridae	CNPPI1277-12	KJ208582
Scutacaridae	CNPAE418-13	KM824269
Scutacaridae	SSPAA2303-13	KM826607
Scutacaridae	CNJAE1149-12	KM827367
Scutacaridae	CNPAI462-13	KM828623
Scutacaridae	SSPAA6647-13	KM829848
Scutacaridae	CNJAD2278-12	KM831506
Scutacaridae	CNJAC1524-12	KM834045
Scutacaridae	CNPAE424-13	KM838045
Scutacaridae	CNPAH412-13	KM839007
Scutacaridae	CNJAJ699-12	KM840062
Scutacaridae	CNJAI852-12	KM840797
Siteroptidae	CHACB1159-10	HM907243
Siteroptidae	CHACB373-10	HQ558549
Siteroptidae	MYMCG145-12	JX833953
Siteroptidae	MYMCB862-11	JX834342
Siteroptidae	MYMCC151-11	JX834636
Siteroptidae	MYMCE751-12	JX834643
Siteroptidae	MYMCC127-11	JX835082
Siteroptidae	MYMCE679-12	JX835518
Siteroptidae	MYMCC115-11	JX836362
Siteroptidae	MYMCC010-11	JX836463
Siteroptidae	MYMCE651-12	JX836651
Siteroptidae	MYMCC285-11	JX836742
Siteroptidae	MYMCC075-11	JX836925
Siteroptidae	MYMCF232-12	JX837251
Siteroptidae	MYMCF251-12	JX837381
Siteroptidae	MYMCA1438-11	JX837406
Siteroptidae	MYMCF273-12	JX837645
Siteroptidae	MYMCA1266-11	JX838117
Siteroptidae	MYMCG396-12	JX838178
Siteroptidae	MYMCA1034-11	JX838317

Siteroptidae		MYMCE652-12	JX838413
Siteroptidae		MYMCE168-12	JX838516
Siteroptidae		CNPAH404-13	KM824522
Siteroptidae		SSBAA2065-12	KM824811
Siteroptidae		CNPAC333-13	KM827520
Siteroptidae		CNRMF3237-12	KM833269
Siteroptidae		CNJAF2021-12	KM834307
Siteroptidae		CNPAD834-13	KM839115
Siteroptidae		SSWEE046-13	KM839804
Siteroptidae		RBINA5682-13	KP979226
Sperchontidae	*Sperchon*	GACAC040-12	AB530314
Sperchontidae	*Sperchon*	CFWIA169-10	HQ938532
Sperchontidae	*Sperchon*	CFWIA171-10	HQ938534
Sperchontidae	*Sperchon*	CFWIA173-10	HQ938536
Sperchontidae	*Sperchon*	CFWIA179-10	HQ938541
Stigmaeidae	*Eustigmaeus*	MYMCA306-11	JX837620
Stigmaeidae		CHACB315-10	HM907302
Stigmaeidae		CHACB059-10	HQ558367
Stigmaeidae		CHACB216-10	HQ558476
Stigmaeidae		CHACB585-10	HQ558686
Stigmaeidae		CHACB760-10	HQ941474
Stigmaeidae		CHACB840-10	HQ941510
Stigmaeidae		MYTMC037-09	HQ966227
Stigmaeidae		MYMCC845-11	JX834146
Stigmaeidae		CHACB1074-10	JX834147
Stigmaeidae		CHACB666-10	JX834254
Stigmaeidae		CHACA507-09	JX834413
Stigmaeidae		MYMCE692-12	JX834506
Stigmaeidae		MYMCF826-12	JX834512
Stigmaeidae		MYMCA614-11	JX835126
Stigmaeidae		MYMCF943-12	JX835383
Stigmaeidae		MYMCA826-11	JX835407
Stigmaeidae		MYMCD017-11	JX835485
Stigmaeidae		CHACC261-10	JX836292
Stigmaeidae		MYMCA813-11	JX836545
Stigmaeidae		MYMCF150-12	JX836791
Stigmaeidae		MYMCC333-11	JX836884
Stigmaeidae		MYMCG528-12	JX837154
Stigmaeidae		CHACA530-09	JX837427
Stigmaeidae		MYMCE517-12	JX837538
Stigmaeidae		MYMCA173-11	JX838064
Stigmaeidae		MYMCC812-11	JX838235
Stigmaeidae		MYMCE289-12	JX838290
Stigmaeidae		MYMCA1433-11	JX838523

Stigmaeidae		CNPPE1451-12	KJ166386
Stigmaeidae		CNPAE452-13	KM834074
Tarsonemidae	*Acarapis*	GBCH7501-13	AB634839
Tarsonemidae	*Acarapis*	GBCH7502-13	AB634838
Tarsonemidae		MYMCE942-12	JX834193
Tarsonemidae		MYMCA619-11	JX834483
Tarsonemidae		MYMCC646-11	JX834523
Tarsonemidae		MYMCG094-12	JX834907
Tarsonemidae		MYMCG186-12	JX835218
Tarsonemidae		MYMCE892-12	JX835807
Tarsonemidae		MYMCE066-12	JX835933
Tarsonemidae		MYMCG390-12	JX836052
Tarsonemidae		MYMCG246-12	JX836238
Tarsonemidae		MYMCF577-12	JX836323
Tarsonemidae		MYMCE113-12	JX836968
Tarsonemidae		MYMCA1011-11	JX837461
Tarsonemidae		MYMCA809-11	JX837566
Tarsonemidae		MYMCE773-12	JX837761
Tarsonemidae		MYMCF054-12	JX838112
Tenuipalpidae	*Dolichotetranychus*	GBCH3946-09	EU682418
Tenuipalpidae	*Raoiella*	GBCH3931-09	EU682434
Tenuipalpidae	*Raoiella*	GBCH3929-09	EU682437
Tenuipalpidae	*Raoiella*	GBCH7725-13	JF928421
Tenuipalpidae	*Raoiella*	GBCH7724-13	JF928422
Tenuipalpidae	*Raoiella*	GBCH7722-13	JF928424
Tenuipalpidae	*Raoiella*	GBCH7721-13	JF928425
Tenuipalpidae	*Raoiella*	GBCH7720-13	JF928426
Tenuipalpidae	*Raoiella*	GBCH7719-13	JF928427
Tenuipalpidae	*Raoiella*	GBCH7718-13	JF928428
Tenuipalpidae	*Raoiella*	GBCH7717-13	JF928429
Tenuipalpidae	*Raoiella*	GBCH7716-13	JF928430
Tenuipalpidae	*Raoiella*	GBCH7714-13	JF928432
Tetranychidae	*Oligonychus*	GBCH8393-13	KF011470
Tetranychidae	*Oligonychus*	GBCH8405-13	KF011457
Tetranychidae	*Oligonychus*	GBCH8402-13	KF011460
Tetranychidae	*Panonychus*	GBCH3918-09	NC_012571
Tetranychidae	*Stigmaeopsis*	GBCH7976-13	AB531823
Tetranychidae	*Stigmaeopsis*	GBCH7964-13	AB531835
Tetranychidae	*Stigmaeopsis*	GBCH8388-13	AB429416
Tetranychidae	*Stigmaeopsis*	GBCH8387-13	AB429422
Tetranychidae	*Tetranychus*	CYTC4960-12	HM753535
Tetranychidae	*Tetranychus*	GBCH11792-13	JX075251
Tetranychidae	*Tetranychus*	GBCH11794-13	JX075249
Tetranychidae	*Tetranychus*	CYTC4552-12	EU345430

Tetranychidae	*Tetranychus*	GBCH11793-13	JX075250
Tetranychidae		GBCH7958-13	AB531841
Tetranychidae		CHACC011-10	HM907412
Tetranychidae		CHACB128-10	HQ558415
Tetranychidae		MYMCB636-11	JX834351
Tetranychidae		MYMCB627-11	JX834670
Tetranychidae		MYMCA964-11	JX837997
Tetranychidae		CNPPE2131-12	KJ092874
Tetranychidae		CNPPE2125-12	KJ166555
Tetranychidae		CNPPJ1865-12	KJ207735
Tetranychidae		CNPAG375-13	KM824684
Tetranychidae		CNGIB577-12	KM825636
Tetranychidae		CNRMC1665-12	KM826643
Tetranychidae		SSPAC7886-13	KM827506
Tetranychidae		SSBAA3636-12	KM828883
Tetranychidae		CNRMA1050-12	KM828896
Tetranychidae		CNGLF2658-13	KM829116
Tetranychidae		CNGIB582-12	KM831358
Tetranychidae		CNJAE1138-12	KM831891
Tetranychidae		CNPAG382-13	KM833817
Tetranychidae		CNJAC1485-12	KM834828
Tetranychidae		SSPAC6755-13	KM837806
Tetranychidae		MIONB099-10	KP979148
Tetranychidae		MIONB110-10	KP979272
Tetranychidae		MIONB338-10	KP979319
Tetranychidae		PHAUG1677-11	KP979254
Tetranychidae		SMTPB14237-13	KP979312
Thyasidae	*Thyopsis*	MYMCA103-11	JX836733
Thyasidae	*Zschokkea*	CHACA049-08	JX833831
Thyasidae	*Zschokkea*	MYMCA049-11	JX833913
Thyasidae	*Zschokkea*	MYMCB450-11	JX834026
Torrenticolidae	*Torrenticola*	GBCH7806-13	JN018109
Torrenticolidae	*Torrenticola*	GBCH12087-13	JX629053
Torrenticolidae	*Torrenticola*	GBCH12089-13	JX629051
Torrenticolidae	*Torrenticola*	GACAC042-12	AB530316
Torrenticolidae	*Torrenticola*	CFWIA190-10	HQ938552
Torrenticolidae	*Torrenticola*	CFWIA639-10	HQ938819
Torrenticolidae	*Torrenticola*	CFWIB552-10	HQ939585
Trombiculidae	*Ascoschoengastia*	GBCH2296-08	NC_010596
Trombiculidae	*Leptotrombidium*	GBCH0045-06	AB194045
Trombiculidae	*Leptotrombidium*	GBCH7543-13	HQ324957
Trombiculidae	*Leptotrombidium*	GBCH7530-13	HQ324970
Trombiculidae	*Leptotrombidium*	GBCH7516-13	HQ324977
Trombiculidae	*Leptotrombidium*	GBCH7513-13	HQ324980

Trombiculidae	*Leptotrombidium*	GBCH1691-06	NC_007600
Trombiculidae	*Leptotrombidium*	GBCH2378-08	AB300496
Trombiculidae	*Leptotrombidium*	GBCH7565-13	HQ324935
Trombiculidae	*Leptotrombidium*	GBCH7564-13	HQ324936
Trombiculidae	*Leptotrombidium*	GBCH2380-08	AB300492
Trombiculidae	*Leptotrombidium*	GBCH1690-06	NC_007177
Trombiculidae	*Leptotrombidium*	GBCH2376-08	AB300499
Trombiculidae	*Leptotrombidium*	GBCH2377-08	AB300498
Trombiculidae	*Leptotrombidium*	GBCH2379-08	AB300494
Trombiculidae	*Neotrombicula*	MYMCE127-12	JX835076
Trombiculidae	*Neotrombicula*	MYMCE126-12	JX836578
Trombiculidae	*Neotrombicula*	SSJAE11558-13	KM840032
Trombiculidae	*Walchia*	GBCH2295-08	NC_010595
Trombiculidae		MYMCG116-12	JX834448
Trombiculidae		MYMCF885-12	JX834572
Trombiculidae		SSBAC3351-12	KM825953
Trombidiidae		CHACA1086-10	HM907127
Trombidiidae		CHACA1124-10	JX836254
Trombidiidae		MYMCA318-11	JX837732
Trombidiidae		CHACA1137-10	JX838025
Trombidiidae		CNPAF858-13	KM828256
Trombidiidae		CNGIE402-12	KM833376
Trombidiidae		SSEIB7646-13	KM836108
Trombidiidae		CNBPC228-12	KM839117
Trombidiidae		CNGID225-12	KM839579
Trombidiidae		MIONB485-11	KM840839
Trombidiidae		JSSEP1135-12	KP979229
Trombidiidae		MYMCE216-12	KP979149
Tydeidae	*Tydeus*	MYMCE526-12	JX833941
Tydeidae	*Tydeus*	MYMCE740-12	JX837674
Tydeidae		CHACB893-10	HM907329
Tydeidae		CHACC012-10	HM907413
Tydeidae		CHACC015-10	HM907416
Tydeidae		CHACC016-10	HM907417
Tydeidae		CHACB002-10	HQ558325
Tydeidae		CHACB430-10	HQ558586
Tydeidae		CHACB680-10	HQ558745
Tydeidae		MYTMC069-09	HQ966232
Tydeidae		MYTMC159-09	HQ966243
Tydeidae		MYMCE384-12	JX833660
Tydeidae		MYMCA094-11	JX833740
Tydeidae		MYMCC448-11	JX834039
Tydeidae		MYMCC073-11	JX834072
Tydeidae		MYMCE753-12	JX834094

Tydeidae	MYMCC345-11	JX834424
Tydeidae	MYMCA558-11	JX834437
Tydeidae	MYMCE401-12	JX834658
Tydeidae	MYMCC356-11	JX834660
Tydeidae	MYMCE576-12	JX834728
Tydeidae	MYMCF702-12	JX834742
Tydeidae	MYMCB133-11	JX834876
Tydeidae	MYMCF574-12	JX834974
Tydeidae	MYMCA1009-11	JX835015
Tydeidae	MYMCD136-11	JX835127
Tydeidae	MYMCE823-12	JX835211
Tydeidae	MYMCE824-12	JX835290
Tydeidae	MYMCB655-11	JX835329
Tydeidae	MYMCF457-12	JX835389
Tydeidae	MYMCB609-11	JX835413
Tydeidae	CHACB667-10	JX835808
Tydeidae	MYMCA1293-11	JX835851
Tydeidae	MYMCF175-12	JX835852
Tydeidae	MYMCD016-11	JX835976
Tydeidae	MYMCB617-11	JX836105
Tydeidae	MYMCF511-12	JX836351
Tydeidae	MYMCF777-12	JX836359
Tydeidae	MYMCE231-12	JX836439
Tydeidae	MYMCB939-11	JX836648
Tydeidae	MYMCB156-11	JX836663
Tydeidae	MYMCE031-12	JX836713
Tydeidae	MYMCA176-11	JX836765
Tydeidae	MYMCG658-12	JX837237
Tydeidae	MYMCA760-11	JX837243
Tydeidae	MYMCF776-12	JX837296
Tydeidae	MYMCA082-11	JX837749
Tydeidae	MYMCB476-11	JX837999
Tydeidae	MYMCD056-11	JX838001
Tydeidae	MYMCA1335-11	JX838309
Tydeidae	MYMCE792-12	JX838381
Tydeidae	MYMCA1231-11	JX838421
Tydeidae	MYMCC289-11	JX838482
Tydeidae	MYMCC649-11	JX838495
Tydeidae	MYMCB273-11	JX838593
Tydeidae	MYMCA931-11	JX838614
Tydeidae	CNPPI898-12	KJ444554
Tydeidae	SSJAC1584-13	KM830456
Tydeidae	SSBAD3079-12	KM831533
Tydeidae	SSBAD4013-12	KM837778

Tydeidae		SSJAC1592-13	KM838931
Tydeidae		MIONB101-10	KP979115
Unionicolidae	*Neumania*	CNSLM152-13	KM839290
Unionicolidae	*Unionicola*	GBCH3249-09	FJ524382
Unionicolidae	*Unionicola*	GBCH3178-08	EU856396
Unionicolidae	*Unionicola*	CNPPC1930-12	KJ084240
Unionicolidae	*Unionicola*	CNPPB2359-12	KJ089239
Unionicolidae	*Unionicola*	CNPPI868-12	KJ207628
Unionicolidae	*Unionicola*	CNPAD835-13	KM826896
Unionicolidae	*Unionicola*	SSPAA2251-13	KM827915
Unionicolidae	*Unionicola*	CNPAC506-13	KM828756
Unionicolidae	*Unionicola*	CNPAC543-13	KM833059
Unionicolidae	*Unionicola*	SSPAA2293-13	KM840319
Unionicolidae	*Unionicola*	CNSLO1213-13	KP979236
Unionicolidae		CNPPB2361-12	KJ089749
**Uropygi**			
Thelyphonidae	*Mastigoproctus*	GBCH2384-08	NC_010430
Thelyphonidae		GBCH7779-13	JN018147
Thelyphonidae		GBCH7778-13	JN018148
**Xiphosurida**			
Limulidae	*Limulus polyphemus*	GBA10398-13	HQ588747
