# Supplementary material for: Patterns of Protein Evolution in Cytochrome c Oxidase 1 (COI) from the Class Arachnida
Source: PLoS One. 2015 Aug 26;10(8):e0135053. doi: 10.1371/journal.pone.0135053 (PMC4550450; doi:10.1371/journal.pone.0135053)
Supplement: S3 Table — Values are reported for the divergences among families in each order (Family Distance) and among the BINs in each family (BIN Distance). All Distances > 0.25 are shown in bold, while families highlighted in green are those which included at least one species with an indel. (PDF) [file pone.0135053.s006.pdf]

S3 Table. Mean Dayhoff distance (+/- SE) based on amino acid divergences in the barcode region of COI for ten orders of arachnids with data for more than one family. Values are reported for the divergences among families in each order (Family Distance) and among the BINs in each family (BIN Distance). All Distances > 0.25 are shown in bold, while families highlighted in green are those which included at least one species with an indel.

Order	Suborder	Family	Family Distance	BIN Distance	BIN n
Solifugae			0.071 (SE = 0.001)	0.023 (SE = NA)	
		Rhagodidae	0.069	0.023	2
		Galeodidae	0.070	NA	1
		Ammotrechidae	0.072	NA	1
		Eremobatidae	0.074	NA	1
Amblypygi			0.084 (SE = 0.003)	0.051 (SE = 0.007)	
	Euamblypygi	Phrynichidae	0.076	0.038	2
	Euamblypygi	Charontidae	0.081	0.053	3
	Euamblypygi	Phrynidae	0.086	0.062	2
	Euamblypygi	Charinidae	0.092	NA	1
Scorpiones			0.099 (SE = 0.010)	0.036 (SE = 0.012)	
		Euscorpiidae	0.080	0.000	2
		Vaejovidae	0.078	0.015	52
		Chactidae	0.086	0.062	3
		Scorpionidae	0.094	0.051	22
		Hemiscorpiidae	0.114	NA	1
		Buthidae	0.144	0.053	47
Araneae			0.158 (SE = 0.003)	0.064 (SE = 0.005)	
	Araneomorphae	Corinnidae	0.123	0.007	3
	Araneomorphae	Desidae	0.124	0.059	3
	Araneomorphae	Nephilidae	0.125	0.039	13
	Araneomorphae	Nesticidae	0.132	0.048	10
	Araneomorphae	Stiphidiidae	0.130	NA	1
	Araneomorphae	Dictynidae	0.132	0.062	42
	Araneomorphae	Pimoidae	0.135	0.024	4
	Araneomorphae	Ctenidae	0.136	NA	1
	Araneomorphae	Gnaphosidae	0.135	0.068	49
	Araneomorphae	Sparassidae	0.136	0.057	14
	Araneomorphae	Araneidae	0.136	0.035	97
	Araneomorphae	Liocranidae	0.135	0.012	3
	Araneomorphae	Mimetidae	0.137	0.053	4
	Araneomorphae	Mysmenidae	0.136	0.057	14
	Araneomorphae	Agelenidae	0.140	0.079	31
	Araneomorphae	Hahniidae	0.138	0.086	16
	Araneomorphae	Linyphiidae	0.139	0.050	348
	Araneomorphae	Mecysmaucheniidae	0.138	0.064	5
	Araneomorphae	Thomisidae	0.139	0.032	59
	Araneomorphae	Trachelidae	0.140	0.087	3

Araneomorphae	Cybaeidae	0.139	0.063	24
Araneomorphae	Miturgidae	0.137	NA	1
Araneomorphae	Theridiidae	0.140	0.080	93
Araneomorphae	Anyphaenidae	0.142	0.047	7
Araneomorphae	Pisauridae	0.144	0.032	12
Araneomorphae	Theridiosomatidae	0.144	0.103	2
Araneomorphae	Uloboridae	0.147	0.066	5
Araneomorphae	Lycosidae	0.146	0.045	101
Araneomorphae	Salticidae	0.144	0.057	89
Araneomorphae	Stenochilidae	0.145	NA	1
Araneomorphae	Archaeidae	0.148	0.079	14
Araneomorphae	Eutichuridae	0.148	0.078	5
Araneomorphae	Eresidae	0.149	NA	1
Araneomorphae	Philodromidae	0.150	0.061	31
Araneomorphae	Tetragnathidae	0.150	0.064	71
Araneomorphae	Phrurolithidae	0.148	0.023	7
Araneomorphae	Psechridae	0.151	0.082	44
Araneomorphae	Zoropsidae	0.162	NA	1
Araneomorphae	Segestriidae	0.158	0.058	2
Araneomorphae	Clubionidae	0.156	0.039	38
Araneomorphae	Dysderidae	0.160	0.062	34
Mygalomorphae	Nemesiidae	0.158	0.048	13
Araneomorphae	Amaurobiidae	0.159	0.090	13
Araneomorphae	Periegopidae	0.160	NA	1
Araneomorphae	Phyxelididae	0.161	0.088	25
Araneomorphae	Caponiidae	0.164	NA	1
Mygalomorphae	Dipluridae	0.166	0.112	2
Araneomorphae	Diguetidae	0.169	NA	1
Araneomorphae	Filistatidae	0.172	NA	1
Araneomorphae	Palpimanidae	0.175	NA	1
Araneomorphae	Hypochilidae	0.176	NA	1
Mygalomorphae	Ctenizidae	0.182	0.058	2
Araneomorphae	Huttoniidae	0.182	NA	1
Mygalomorphae	Actinopodidae	0.187	0.047	18
Araneomorphae	Anapidae	0.194	0.181	3
Mygalomorphae	Antrodiaetidae	0.193	NA	1
Araneomorphae	Pholcidae	0.191	0.123	84
Araneomorphae	Symphytognathidae	0.187	0.155	5
Mygalomorphae	Migidae	0.193	0.083	27
Araneomorphae	Leptonetidae	0.196	0.059	2
Araneomorphae	Scytodidae	0.198	NA	1
Mygalomorphae	Theraphosidae	0.198	0.082	5
Mygalomorphae	Mecicobothriidae	0.197	NA	1
Araneomorphae	Telemidae	0.205	NA	1

	Araneomorphae	Sicariidae	0.227	0.006	3
	Mesothelae	Liphistiidae	0.246	NA	1
Ixodida			0.213 (SE = 0.008)	0.097 (SE = 0.017)	
	Ixodoidea	Argasidae	0.206	0.114	4
	Ixodoidea	Ixodidae	0.203	0.080	67
	Ixodoidea	Nuttalliellidae	0.229	NA	1
Sarcoptiformes			0.250 (SE = 0.010)	0.046 (SE = 0.005)	
	Oribatida	Cepheidae	0.173	0.008	8
	Oribatida	Chamobatidae	0.176	0.000	3
	Oribatida	Eremaeidae	0.176	0.020	28
	Oribatida	Mochlozetidae	0.176	0.014	7
	Oribatida	Tenuialidae	0.177	NA	1
	Oribatida	Tegoribatidae	0.178	0.032	3
	Oribatida	Phenopelopidae	0.178	0.021	12
	Oribatida	Ceratozetidae	0.178	0.026	104
	Oribatida	Mycobatidae	0.179	0.037	11
	Oribatida	Achipteriidae	0.179	0.037	9
	Oribatida	Peloppiidae	0.179	0.020	18
	Oribatida	Megeremaeidae	0.180	NA	1
	Oribatida	Hydrozetidae	0.180	0.023	2
	Oribatida	Scutoverticidae	0.180	0.028	39
	Oribatida	Cymbaeremaeidae	0.181	0.044	15
	Oribatida	Oppiidae	0.181	0.024	39
	Oribatida	Eremulidae	0.183	NA	1
	Oribatida	Haplozetidae	0.183	0.045	7
	Oribatida	Oribatulidae	0.184	0.024	40
	Oribatida	Malaconothridae	0.184	0.006	8
	Oribatida	Eremobelbidae	0.186	NA	1
	Oribatida	Astegistidae	0.186	NA	1
	Oribatida	Oribatellidae	0.188	0.065	5
	Oribatida	Trhypochthoniidae	0.189	0.011	10
	Oribatida	Hermanniidae	0.190	0.004	2
	Oribatida	Ameronothridae	0.192	0.034	3
	Oribatida	Gymnodamaeidae	0.193	0.013	9
	Oribatida	Gustaviidae	0.194	0.064	2
	Oribatida	Suctobelbidae	0.194	0.021	12
	Oribatida	Neoliodidae	0.194	0.028	3
	Oribatida	Galumnidae	0.195	0.089	20
	Oribatida	Quadroppiidae	0.198	0.010	4
	Oribatida	Crotoniidae	0.200	0.048	35
	Oribatida	Hermanniellidae	0.200	NA	1
	Oribatida	Euzetidae	0.202	0.005	2
	Oribatida	Parakalummidae	0.203	0.058	5
	Oribatida	Liacaridae	0.204	0.101	5

Oribatida	Nothridae	0.206	0.012	13
Oribatida	Scheloribatidae	0.208	0.071	34
Oribatida	Damaeidae	0.212	0.067	16
Oribatida	Tectocepheidae	0.212	0.076	51
Oribatida	Thyrisomidae	0.227	0.141	3
Oribatida	Oripodidae	0.232	0.038	6
Oribatida	Passalozetidae	0.239	NA	1
Endeostigmata	Alicorhagiidae	0.240	0.004	5
Oribatida	Oribotritiidae	0.241	NA	1
Oribatida	Eniochthoniidae	0.242	0.004	2
Oribatida	Brachychthoniidae	**0.254**	0.051	66
Oribatida	Euphthiracaridae	**0.273**	0.149	4
Oribatida	Carabodidae	**0.278**	0.140	2
Oribatida	Hypochthoniidae	**0.282**	NA	1
Endeostigmata	Alycidae	**0.310**	0.119	5
Astigmata	Alloptidae	**0.316**	0.025	2
Endeostigmata	Nanorchestidae	**0.321**	0.133	9
Astigmata	Acaridae	**0.322**	0.034	4
Astigmata	Pterolichidae	**0.322**	0.016	1
Astigmata	Proctophyllodidae	**0.324**	0.018	52
Astigmata	Pyroglyphidae	**0.326**	0.022	7
Astigmata	Avenzoariidae	**0.327**	0.072	7
Astigmata	Xolalgidae	**0.331**	0.040	3
Astigmata	Trouessartiidae	**0.332**	0.027	13
Astigmata	Psoroptidae	**0.332**	0.039	2
Astigmata	Carpoglyphidae	**0.334**	NA	1
Astigmata	Gabuciniidae	**0.335**	0.016	2
Astigmata	Analgidae	**0.337**	0.037	5
Astigmata	Pteronyssidae	**0.338**	0.052	7
Astigmata	Histiostomatidae	**0.341**	0.087	2
Astigmata	Listrophoridae	**0.348**	0.093	3
Oribatida	Phthiracaridae	**0.350**	0.174	9
Astigmata	Syringobiidae	**0.377**	NA	1
Astigmata	Myocoptidae	**0.378**	NA	1
Astigmata	Knemidokoptidae	**0.385**	0.044	2
Endeostigmata	Terpnacaridae	**0.406**	0.061	3
Astigmata	Sarcoptidae	**0.460**	NA	1
Mesostigmata		0.229 (SE = 0.009)	0.120 (SE = 0.017)	
Monogynaspida	Paratennulidae	0.179	NA	1
Monogynaspida	Parasitidae	0.182	0.059	54
Monogynaspida	Arctacaridae	0.187	0.080	2
Monogynaspida	Microgyniidae	0.188	0.057	4
Monogynaspida	Oplitidae	0.188	NA	1
Sejida	Sejidae	0.188	0.036	2

Monogynaspida	Urodinychidae	0.195	0.035	5
Monogynaspida	Trematuridae	0.199	0.035	12
Monogynaspida	Ologamasidae	0.199	0.073	12
Monogynaspida	Pachylaelapidae	0.204	0.178	3
Monogynaspida	Dinychidae	0.215	0.084	9
Monogynaspida	Trachytidae	0.218	NA	1
Monogynaspida	Macrochelidae	0.223	0.119	14
Monogynaspida	Digamasellidae	0.231	0.198	47
Monogynaspida	Melicharidae	0.231	0.106	20
Monogynaspida	Veigaiidae	0.236	0.093	3
Monogynaspida	Varroidae	0.247	NA	1
Monogynaspida	Ascidae	0.246	0.157	51
Monogynaspida	Zerconidae	**0.255**	0.183	14
Monogynaspida	Laelapidae	**0.289**	**0.273**	30
Monogynaspida	Phytoseiidae	**0.285**	0.234	94
Monogynaspida	Blattisociidae	**0.283**	0.201	26
Monogynaspida	Ameroseiidae	**0.315**	0.078	7
Monogynaspida	Dermanyssidae	**0.316**	NA	1
Pseudoscorpiones		0.331 (SE = 0.004)	0.190 (SE = 0.017)	
Iocheirata	Hyidae	**0.304**	NA	1
Iocheirata	Geogarypidae	**0.307**	0.083	2
Iocheirata	Larcidae	**0.305**	NA	1
Iocheirata	Cheliferidae	**0.311**	0.141	6
Iocheirata	Olipiidae	**0.311**	0.214	11
Iocheirata	Ideoroncidae	**0.311**	NA	1
Iocheirata	Syarinidae	**0.318**	**0.263**	5
Iocheirata	Gymnobisiidae	**0.323**	NA	1
Iocheirata	Neobisiidae	**0.322**	0.172	8
Iocheirata	Atemnidae	**0.327**	0.157	6
Iocheirata	Garypidae	**0.328**	0.164	2
Iocheirata	Parahyidae	**0.346**	NA	1
Iocheirata	Chernetidae	**0.340**	0.172	14
Epiocheirata	Chthoniidae	**0.338**	0.194	20
Epiocheirata	Feaellidae	**0.349**	NA	1
Epiocheirata	Tridenchthoniidae	**0.340**	NA	1
Iocheirata	Sternophoridae	**0.341**	0.171	2
Iocheirata	Withiidae	**0.338**	NA	1
Epiocheirata	Lechytiidae	**0.363**	NA	1
Epiocheirata	Pseudogarypidae	**0.365**	**0.251**	2
Iocheirata	Cheiridiidae	**0.370**	**0.298**	3
Trombidiformes		0.341 (SE = 0.013)	0.111 (SE = 0.012)	
Prostigmata	Protziidae	0.238	NA	1
Prostigmata	Hydryphantidae	**0.251**	0.025	8
Prostigmata	Sperchontidae	**0.250**	0.035	5

	Prostigmata	Lebertiidae	**0.255**	0.017	7
	Prostigmata	Torrenticolidae	**0.252**	0.020	7
	Prostigmata	Thyasidae	**0.256**	0.055	4
	Prostigmata	Mideopsidae	**0.264**	0.034	3
	Prostigmata	Pionidae	**0.263**	0.046	16
	Prostigmata	Acalyptonotidae	**0.273**	NA	1
	Prostigmata	Arrenuridae	**0.275**	0.042	16
	Prostigmata	Unionicolidae	**0.279**	0.059	13
	Prostigmata	Eylaidae	**0.298**	0.016	6
	Prostigmata	Anystidae	**0.291**	0.085	61
	Prostigmata	Rhagidiidae	**0.290**	0.042	72
	Prostigmata	Limnesiidae	**0.296**	0.075	10
	Prostigmata	Bdellidae	**0.304**	0.125	65
	Prostigmata	Hydrachnidae	**0.312**	0.042	2
	Prostigmata	Ereynetidae	**0.323**	0.108	5
	Prostigmata	Eupodidae	**0.337**	0.120	177
	Prostigmata	Trombidiidae	**0.335**	0.098	12
	Prostigmata	Penthaleidae	**0.342**	0.097	7
	Prostigmata	Penthalodidae	**0.342**	0.104	12
	Prostigmata	Tydeidae	**0.344**	0.192	62
	Prostigmata	Trombiculidae	**0.345**	0.180	22
	Prostigmata	Siteroptidae	**0.351**	0.096	30
	Prostigmata	Scutacaridae	**0.350**	0.098	24
	Prostigmata	Erythraeidae	**0.374**	0.200	126
	Prostigmata	Hygrobatidae	**0.388**	**0.292**	10
	Prostigmata	Calyptostomatidae	**0.390**	0.086	2
	Prostigmata	Cunaxidae	**0.408**	0.181	22
	Prostigmata	Stigmaeidae	**0.399**	**0.313**	31
	Prostigmata	Pyemotidae	**0.397**	0.167	2
	Prostigmata	Microtrombidiidae	**0.417**	0.181	14
	Prostigmata	Tarsonemidae	**0.449**	0.171	17
	Prostigmata	Cheyletidae	**0.459**	0.117	4
	Prostigmata	Eriophyidae	**0.479**	0.128	9
	Prostigmata	Tetranychidae	**0.522**	0.168	39
	Prostigmata	Tenuipalpidae	**0.574**	0.176	13
Opiliones			0.354 (SE = 0.010)	0.108 (SE = 0.029)	
	Dyspnoi	Ceratolasmatidae	**0.308**	0.013	8
	Euponoi	Phalangiidae	**0.308**	0.027	4
	Laniatores	Gonyleptidae	**0.316**	NA	1
	Laniatores	Stygnopsidae	**0.316**	0.155	3
	Laniatores	Stygnommatidae	**0.321**	0.037	4
	Laniatores	Epedanidae	**0.322**	0.004	2
	Cyphophthalmi	Troglosironidae	**0.324**	0.025	14
	Dyspnoi	Trogulidae	**0.329**	NA	1

Laniatores	Kimulidae	**0.331**	NA	1
Laniatores	Biantidae	**0.331**	NA	1
Euponoi	Sclerosomatidae	**0.333**	0.051	7
Cyphophthalmi	Stylocellidae	**0.345**	0.105	10
Laniatores	Zalmoxidae	**0.357**	NA	1
Cyphophthalmi	Pettalidae	**0.359**	0.120	67
Laniatores	Samoidae	**0.380**	NA	1
Laniatores	Icaleptidae	**0.390**	NA	1
Cyphophthalmi	Neogoveidae	**0.404**	**0.266**	24
Laniatores	Triaenonychidae	**0.420**	0.195	4
Laniatores	Podoctidae	**0.421**	NA	1
Cyphophthalmi	Sironidae	**0.492**	**0.300**	20
