# Supplementary material for: Patterns of Protein Evolution in Cytochrome c Oxidase 1 (COI) from the Class Arachnida
Source: PLoS One. 2015 Aug 26;10(8):e0135053. doi: 10.1371/journal.pone.0135053 (PMC4550450; doi:10.1371/journal.pone.0135053)
Supplement: S4 Table — (PDF) [file pone.0135053.s007.pdf]

S4 Table. Mean generation time estimated for 12 of the 16 arachnid orders.

Order	Generation Time (Months)
Amblypygi	18[a]
Araneae	12[b]
Holothyrida	NA
Ixodida	24[c]
Mesostigmata	2[d]
Opilioacarida	NA
Opiliones	8[e]
Palpigradi	NA
Pseudoscorpiones	12[f]
Ricinulei	30[g]
Sarcoptiformes	12[d]
Schizomida	NA
Scorpiones	24[h]
Solifugae	12[i]
Trombidiformes	2[d]
Uropygi	24[j]

[a] [30]

[b] Gergin Blagoev personal communication

[c] [31]

[d] [3]

[e] [32]

[f] [33]

[g] [8]

[h] [34]

[i] [35]

[j] [36]
